# Association of general health and lifestyle factors with the salivary microbiota – Lessons learned from the ADDITION-PRO cohort

**DOI:** 10.3389/fcimb.2022.1055117

**Published:** 2022-11-16

**Authors:** Casper Sahl Poulsen, Nikoline Nygaard, Florentin Constancias, Evelina Stankevic, Timo Kern, Daniel R. Witte, Dorte Vistisen, Niels Grarup, Oluf Borbye Pedersen, Daniel Belstrøm, Torben Hansen

**Affiliations:** ^1^ The Novo Nordisk Foundation Center for Basic Metabolic Research, Faculty of Health and Medical Sciences, Copenhagen University, Copenhagen, Denmark; ^2^ Institute of Odontology, Section of Oral Microbiology, Faculty of Health and Medical Sciences, Copenhagen University, Copenhagen, Denmark; ^3^ Swiss Federal Institute of Technology in Zürich, Department of Health Sciences and Technology, Zürich, Switzerland; ^4^ Department of Public Health, Aarhus University, Aarhus, Denmark; ^5^ Steno Diabetes Center Aarhus, Aarhus, Denmark; ^6^ Steno Diabetes Center, Copenhagen, Denmark; ^7^ Department of Public Health, University of Copenhagen, Copenhagen, Denmark; ^8^ Center for Clinical Metabolic Research, Herlev-Gentofte Hospital, Gentofte, Denmark

**Keywords:** saliva, type 2 diabetes (T2D), smoking, microbiota, biomarker

## Abstract

**Introduction:**

Previous research indicates that the salivary microbiota may be a biomarker of oral as well as systemic disease. However, clarifying the potential bias from general health status and lifestyle-associated factors is a prerequisite of using the salivary microbiota for screening.

**Materials & Methods:**

ADDDITION-PRO is a nationwide Danish cohort, nested within the Danish arm of the Anglo-Danish-Dutch Study of Intensive treatment in People with Screen-Detected Diabetes in Primary Care. Saliva samples from n=746 individuals from the ADDITION-PRO cohort were characterized using 16s rRNA sequencing. Alpha- and beta diversity as well as relative abundance of genera was examined in relation to general health and lifestyle-associated variables. Permutational multivariate analysis of variance (PERMANOVA) was performed on individual variables and all variables together. Classification models were created using sparse partial-least squares discriminant analysis (sPLSDA) for variables that showed statistically significant differences based on PERMANOVA analysis (p < 0.05).

**Results:**

Glycemic status, hemoglobin-A_1c_ (HbA_1c_) level, sex, smoking and weekly alcohol intake were found to be significantly associated with salivary microbial composition (individual variables PERMANOVA, p < 0.05). Collectively, these variables were associated with approximately 5.8% of the observed differences in the composition of the salivary microbiota. Smoking status was associated with 3.3% of observed difference, and smoking could be detected with good accuracy based on salivary microbial composition (AUC 0.95, correct classification rate 79.6%).

**Conclusions:**

Glycemic status, HbA_1c_ level, sex, smoking and weekly alcohol intake were significantly associated with the composition of the salivary microbiota. Despite smoking only being associated with 3.3% of the difference in overall salivary microbial composition, it was possible to create a model for detection of smoking status with a high correct classification rate. However, the lack of information on the oral health status of participants serves as a limitation in the present study. Further studies in other cohorts are needed to validate the external validity of these findings.

## Introduction

Saliva is the biological fluid of the oral cavity, which is critical to maintenance of oral health, as well as basic functions, such as speech, taste and swallowing (Pedersen et al., 2018; Lynge Pedersen and Belstrøm, 2019). In addition, saliva harbors a wealth of biological substances including DNA, RNA and proteins of both microbial and human origin, which is why saliva is referred to as the mirror of the body (Greabu et al., 2009; Tiwari, 2011). Therefore, due to the easy and non-invasive sampling, saliva is an attractive medium for biomarker analysis in both dentistry and general medicine (Yoshizawa et al., 2013).

Saliva is sterile when entering the oral cavity (Schrøder et al., 2017), and consequently the salivary microbiota is a conglomerate of bacteria shed from oral surfaces. Prime donor sites are the tongue, the throat and the palatine tonsils (Segata et al., 2012). There are also minor contributions from oral sites such as supragingival and subgingival plaques, which have direct relevance to the major oral diseases, periodontitis and dental caries (Segata et al., 2012). Several studies have shown that salivary levels of specific pathogens associated with periodontitis, such as *Porphyromonas gingivalis, Tannerella forsythia, Treponema denticola* and *Prevotella intermedia* correlate with that of subgingival plaque found in the periodontitis lesion (He et al., 2012; Haririan et al., 2014; Belstrøm et al., 2017; Nickles et al., 2017; Belstrøm et al., 2018). In addition, it has been documented that salivary abundance of specific pathogens, including *P. gingivalis*, associates with periodontal status (Morozumi et al., 2016; Kageyama et al., 2017; Damgaard et al., 2019). Consequently, salivary abundance of specific bacterial species has been suggested as a diagnostic biomarker of periodontitis, with the potential to be used in the dental office. However, a prerequisite of using the salivary microbiota for screening of oral diseases is to clarify the potential bias from general health status and life-style associated factors, which inevitably are different among patients.

Type 2 diabetes (T2D) and cardiovascular diseases are some of the most prevalent general medical disorders, and tobacco smoking is a common habit (World Health Organization, 2021a; World Health Organization, 2021b; World Health Organization, 2022). Cross-sectional data suggests that the salivary microbiota in patients with T2D is less diverse and harbors significantly higher levels of periodontal pathogens (Gogeneni et al., 2015; Ogawa et al., 2017; Saeb et al., 2019). Likewise, smoking has been described to have an impact on the salivary microbiota (Belstrøm et al., 2014; Wirth et al., 2020; Belstrøm et al., 2021). The salivary microbiota have even been found to predict the risk of cardiovascular disease with some success (Murugesan et al., 2021). In general, current evidence suggests that the salivary microbiota may not only be a diagnostic biomarker of oral diseases, but potentially also of general medical disorders. Whether these associations are causal or due to an indirect confounded association is currently not clear, and available data originates primarily from studies with small sample sizes in selected populations. Studies with a large sample size and substantial information on general medical health and lifestyle-associated factors are therefore needed, as they will allow for a more detailed assessment of associations and provide better options to account for confounding.

The ADDITION-PRO cohort is a prospective cohort nested within the Danish arm (ADDITION-DK) of the Anglo-Danish-Dutch Study of Intensive Treatment In People with Screen-Detected Diabetes in Primary Care study (ADDITION-Europe) (Johansen et al., 2012). ADDITION-PRO consists of 2082 Danes from across the country, at varying risk of developing T2D measured by their fasting blood glucose and glucose tolerance. Participants underwent a comprehensive health assessment at inclusion, resulting in an extensive dataset with measurements of anthropometry, biochemistry, physical fitness, cardiovascular risk factors and questionnaire data. In addition, blood, urine and saliva samples were collected from the participants. Given the considerable size of the population and its comprehensive characterization the ADDITION-PRO cohort makes for a highly relevant source of information in determining the association of general health status with lifestyle-associated factors on the composition of the salivary microbiota.

Therefore, the purpose of the present study was to evaluate the association of general medical disorders and lifestyle-associated factors with the composition of the salivary microbiota. To do this, we used 16S rRNA sequencing to characterize the salivary microbiota in N = 786 individuals from the ADDITION-PRO cohort, from which we also had access to comprehensive information on general medical health and lifestyle-associated factors. We tested the hypothesis that general medical health and lifestyle are significantly associated with the composition of the salivary microbiota, and that general health status and lifestyle can be predicted based on the salivary microbiota.

## Methods

### The ADDITION-PRO cohort

The ADDITION-PRO cohort has been described in detail elsewhere (Johansen et al., 2012). In brief, it is a cohort nested within the Danish arm (ADDITION-DK) of the ADDITION-Europe study, consisting of individuals at differing risks of T2D. Individuals were recruited for ADDITION-PRO between 2009 and 2011 during follow-up health examinations of a subset of the population from ADDITION-DK. Eligible for the follow-up health examination were those who were still alive, and still lived in the regions of the four participating research centers (Steno Diabetes Center, Aarhus University Hospital, Holstebro Hospital, and Hospital of South West Jutland). Lastly, eligibility required not having withdrawn consent to study participation.

Of the 16,136 participants in the follow-up health examination, all individuals with impaired glucose regulation at the time of screening, along with those diagnosed with diabetes during the follow-up period (n=1483), along with a 19% random sample of individuals from the low and high-risk groups (n=2705) were invited to participate in ADDITION-PRO. In total, 50% (n=2082) of invitees agreed to participate (Johansen et al., 2012). Saliva samples were collected only at the Copenhagen site, resulting in 786 saliva samples to be analyzed in the present study.

The ADDITION-PRO cohort was approved by the scientific ethics committee in the Central Denmark Region (H-20000183). Participants gave their written informed consent to participate in the study and for linkage of their data with national registers for the purposes of the ADDITION-PRO study. In addition, the present study was performed in compliance with the Helsinki Declaration.

### Data collection

The health assessment performed is described in detail elsewhere (Johansen et al., 2012). In brief, information on sex, age, and lifestyle-associated factors (smoking, alcohol consumption and physical activity) were collected *via* a general health questionnaire. Clinical measurements of height, weight, and waist circumference were performed, and BMI was calculated based on height and weight measurements. Blood pressure (systolic and diastolic) and heart rate was calculated as the average of three measures after a 10-minute rest (Omron M6 comfort, Omron Healthcare, Milton Keynes, UK).

### Biochemical analyses

The biochemical analyses have been described in detail elsewhere (Johansen et al., 2012). Briefly, biochemical measures were analyzed at the Clinical Chemistry Department at the Steno Diabetes Center in Gentofte, Denmark. HbA_1c_ was measured by HPLC (TOSOH G7, Tokyo, Japan). Total cholesterol, HDL-cholesterol, LDL-cholesterol, triglycerides, were measured using the Hitachi 912 system (Roche Diagnostics, Mannheim, Germany).

### Classification of variables of interest

Classification of the different variables of interest was done based on either internationally acknowledged classifications from the World Health Organization on BMI, HbA_1c_ and physical activity (The Global Diabetes Community, 2019; World Health Organization, 2020; World Health Organization, 2021a), the American Heart Association on blood pressure (American Heart Association, 2022), or recommendations from the Danish Health Authority for weekly alcohol intake (The Danish Health Authority, 2022).

Upon further inspection, 40 individuals were missing significant amounts of data from biochemical analyses, questionnaires etc., and were therefore excluded, resulting in a final dataset of 746 individuals.

### Sample collection

Venous blood samples were drawn after an overnight fast of more than 8 hours. Individuals without known diabetes diagnosis received a standard 75g oral glucose tolerance test with blood samples drawn at 0, 30 and 120 minutes. Further details on the storage and processing of blood samples can be found elsewhere (Johansen et al., 2012).

Paraffin-stimulated saliva samples were collected following a standardized protocol, as previously described (Johansen et al., 2012). Participants were instructed not to consume any food or beverages in the 8 hours prior to sample collection, and not to brush teeth on the morning of collection. All samples were stored at -80^○^C until further analysis (Johansen et al., 2012).

### DNA extraction and 16S rRNA sequencing

Core probes (200 mg) from the frozen samples were taken prior to bacterial DNA isolation. A CryoXtract CXT353 instrument operating below -120°C was used to preserve the sample integrity. Bacterial DNA was isolated, using the NucleoSpinSoil kit (Macherey-Nagel, Germany), following the manufacturer’s instruction. Bacterial cells were lysed using SL1 + Enhancer buffer SX. DNA yield and purity were assessed using a Qubit 2.0 flourometer, and a NanoDrop 2000 spectrometer (Thermo Fisher Scientific Inc., MA, USA). Genomic DNA in each sample was normalized to 30ng prior PCR amplification of the V4 hypervariable region using the 515F/806R primer set. The PCR products were purified with the AmpureXP 17 magnetic bead-based clean-up and size selection kit. The final library was quantified by determining the average molecule length using the 2100 bioanalyzer instrument (Agilent, DNA 1000 Reagents), and by library using real-time quantitative PCR (EvaGreenTM). All samples were processed consecutively at the same location and by the same equipment. Owing to the sample number, sequencing was carried out in two runs using paired-end 250 (PE251+8+8+251) sequencing chemistry on an Illumina HiSeq 2500 platform.

### Data processing

Raw sequencing data were processed with the dada2 (v.1.19.1) (Holmes et al., 2017) algorithm as implemented in metabaRpipe (Constancias and Mahé, 2022). Reads were first truncated after 170 for forward reads, and after 160 for reverse reads. Reads with expected error rates higher than 3 and 4 for forward and reverse reads were subsequently removed. After filtering, error rate learning was performed on all the reads (run_dada2_pipe (nbases = 10 11)), and ASV inference was run using pseudo-pooling strategy. Forward and reverse reads were then merged with a minimum overlap of 40 nucleotides (run_dada2_pipe (minover = 40)). As error rate profile was run dependent, the previous steps were performed independently on samples included in each of the 2 HiSeq runs. ASV tables were then merged and chimeric sequences were identified and removed. A final clustering step was performed to detect potential HiSeq run specific artefacts (run_dada2_pipe(collapseNoMis = TRUE)) and no ASV were clustered confirming the high sensitivity of the approach. Taxonomy was assigned to ASVs using dada2 against eHOMD database (V15.22) (eHOMD, 2020),dada2::assignTaxonomy() and dada2::assignSpecies() (Constancias, 2022) as implemented in the funci phyloseq_dada2_tax(), and a phylogenetic of ASV sequences was build using add_phylogeny_to_phyloseq().

### Statistical analysis

All data analysis was performed using R statistical software (v4.2.1) (R Core Team, 2020). The script was created as an R notebook and made into a pdf file using Rmarkdown, where all function calls are available for the whole analysis (**Supplementary file 1** and https://github.com/csapou/Additionpro_Poulsenetal2022). Sample variables were compared using chi-square tests and the -log10 transformed p-values with an upper limit of 3 were visualized using pheatmap 1.0.12 (Kolde, 2019) to investigate association patterns between the variables. Data were aggregated to genus level in the presented results. Study variables were not available for all individuals, therefore based on the variable of interest, filtering was done to remove these individuals separately for each analysis. Hence, a complete case analyses approach was used.

Alpha diversity including richness and Shannon diversity was performed using the diversity function in the package vegan 2.6.2 (Oksanen et al., 2022) and compared both using parametric tests (ANOVA and t-test if binary) and non-parametrically tests (Kruskal-Wallis and Mann-Whitney if binary). The beta diversity metric used were Bray-Curtis dissimilarities calculated from Hellinger transformed total sum scaled data and used to perform PERMANOVA and principal coordinate analysis (PCoA). The NA individuals were still visualized in the PCoAs

**Figure 1:**
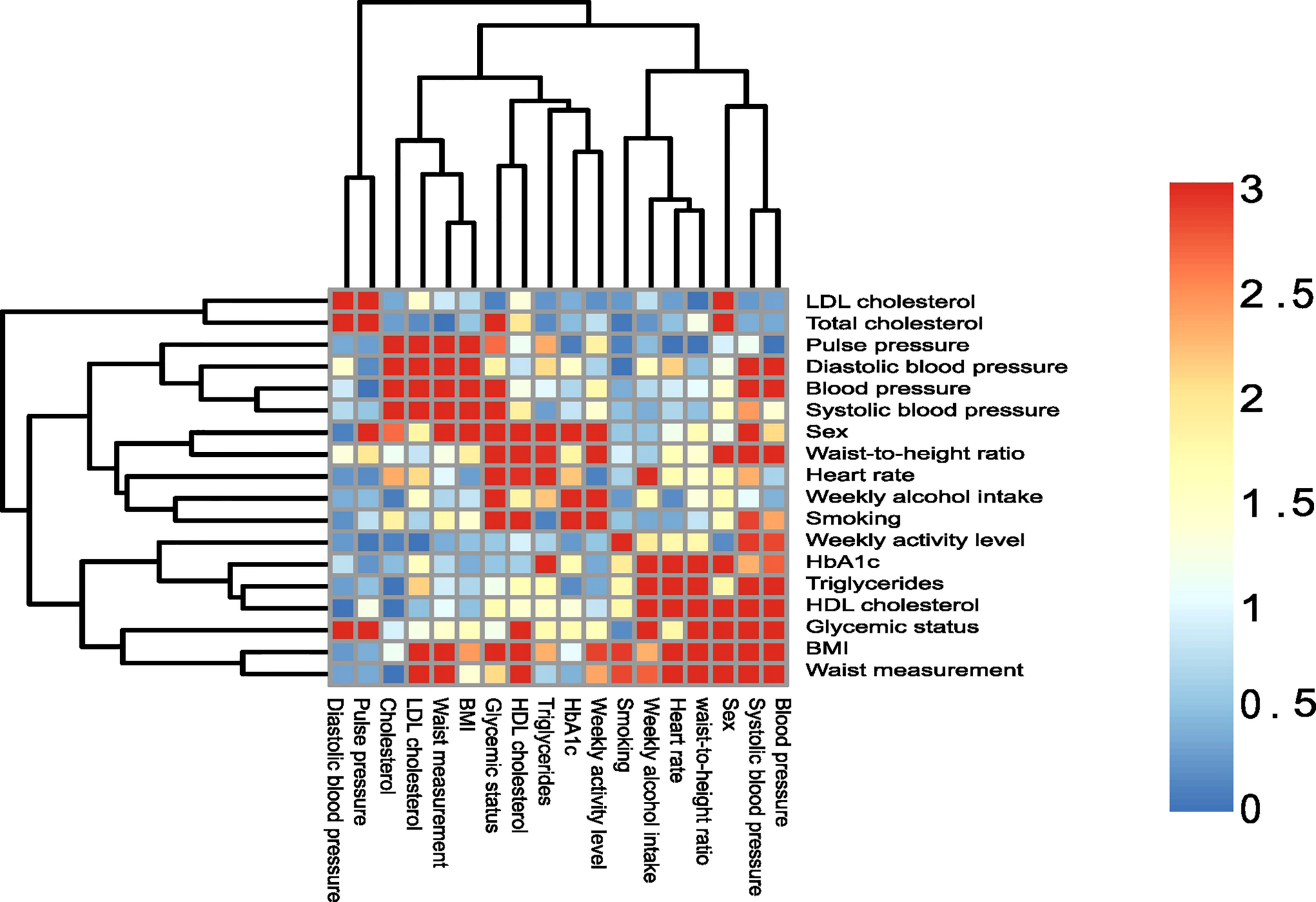
Association of sample variables. Heatmap of sample variables compared by chi-squared tests. The p-values are visualized on a log scale (-log10(pvalue)). A value of 1.30 represent a 0.05 significance cut-off, and values above 1.30 are considered statistically significant.

In order to choose a set of variables of interest, PERMANOVAs were run with individual variables as outcome, and significant variables (smoking, alcohol consumption, glycemic status, sex and HbA_1c_ level) are presented in the present paper. In addition, we chose to present a variable that did not have a statistically significant association in the PERMANOVA in the present paper, using the variable physical activity, as it had the least associations to the other variables (**Figure 1**). PERMANOVA was also run with all variables (consequently on fewer individuals since complete variable availability is a prerequisite), and with just the 5 selected variables found to be statistically significant in the PERMANOVA run, with the addition of the variable physical activity.

Performance of using different types of differential abundance tests was assessed with DAtest 2.8.0. Data were subset to only include taxa present in more than 20% of samples. Non-parametrical tests (Kruskal-Wallis and Mann-Whitney if binary) and DESeq2 (likelihood ratio test (LRT) and Wald test if binary) using relative log values are enclosed in the present study to be used as a reference (**Supplementary files 2-3**).

Stacked bar charts, horizontal relative abundance violin plots of individual genera, and horizontal bar charts were generated with rabuplot 0.0.1.04 (Stockholm, 2022). The p-values presented in the horizontal relative abundance violin plots were obtained from the non-parametric tests. Both the most abundant genera and significant differential abundant genera were presented. A p-value of less than 0.05 were considered statistically significant. All adjusted p-values presented are corrected by the Benjamini-Hochberg method.

To assess the classification potential of the salivary microbiota to differentiate the variable groups the mixOmics 6.20.0 (Rohart et al., 2017) implementation of Sparse partial least square discriminant analysis (sPLS-DA) with Hellinger transformed total sum scaled data were run on the selected variables from the PERMANOVA, but also important features for discrimination are made available (**Supplementary file 4**). Discrimination was measured by area under the curve (AUC) and correct classification rate.

## Results

### Characterization of the study population

**Table 1** details the study population. Study participants were on average 68 years of age, and 55% were males. The majority of the study population (56%) was classified as normoglycemic. The majority of study participants (66%) were either overweight or obese, with the average blood pressure as well as the average HbA_1c_ measure being within the normal range. About half of participants (47%) classified themselves as ex-smokers, while less than 20% reported being currents smokers. More than half (61%) reported spending more than one hour on moderate to vigorous activity daily and drinking on average 11 units of alcohol per week. To test whether the variables of interest correlated with each other, a heatmap of variable association was performed (**Figure 1**). As can be seen, many of the variables presented were correlated with each other.

**Table 1:** Population characteristics, N (%) and mean (SD).

	Total
**N**	746 (100%)
**Age**	68.0 (63.6;72.5)
**Sex**	
*Female*	339 (45%)
*Male*	407 (55%)
**BMI**	26.9 (4.2)
*Underweight (<18.5)*	3 (0.4%)
*Normal weight (18.5 - 24.9)*	251 (34%)
*Overweight (25 - 29.9)*	337 (45%)
*Obese (>30)*	155 (21%)
**Blood pressure**	
*Normal (SBP <120 and DBP <80)*	157 (21%)
*Mildly elevated (SBP 120-129 and DBP < 80)*	93 (12%)
*Moderately elevated (SBP 130-139 or DBP 80-89)*	356 (48%)
*Severely elevated (SBP ≥140 or DBP ≥90)*	138 (19%)
*Unclassified*	2 (≈1%)
**Glycemic status**	
*Low (Normoglycemic)*	421 (56%)
*High (IFG and/or IGT or screening detected DM)*	301 (40)
*Known diabetes mellitus*	21 (3%)
*Unclassified*	3 (≈1%)
**HbA1c %**	5.71 (0.43)
*Normal (<6)*	600 (80%)
*Elevated (6-6.4)*	119 (16%)
*High (>6.5)*	26 (3.5%)
*Missing*	3 (≈1%)
**Smoking status**	
*Nonsmoker*	261 (35%)
*Former smoker*	351 (47%)
*Smoker*	129 (17%)
*Missing*	5 (≈1%)
**Units alcohol per week**	11 (10)
*Abstinence (0)*	79 (11%)
*Moderate (1-10)*	302 (40%)
*High (>10)*	262 (35%)
*Missing*	103 (14%)
**Moderate to vigorous activity, hours per day**	1.43 (0.79;2.41)
*Low (<0.5)*	100 (13%)
*Medium (0.5-1)*	149 (20%)
*High (>1)*	453 (61%)
*Missing*	44 (6%)

### Sequencing metadata

Saliva samples were obtained from 786 individuals and sequenced in two runs (R1, n = 666 and R2, n = 120) resulting in a total of 33,448,846 reads (mean=42,556, sd=22,408). After removal of chimeras, a total of 20,669,468 reads (mean=26,972, sd=14,336) were left, resulting in 5,748 unique amplicon sequence variants (ASVs). Differences between sequencing runs were assessed by PCoA and PERMANOVA. Three samples were included on both runs and differed marginally (Bray-Curtis dissimilarity: 0.010, 0.011 and 0.014) as also visualized in the PCoA (**Supplementary Figure 1**). From the PERMANOVA, no difference was observed between sequencing runs (p=0.45). Thus, the two datasets were merged for analysis.

**Figure 2:**
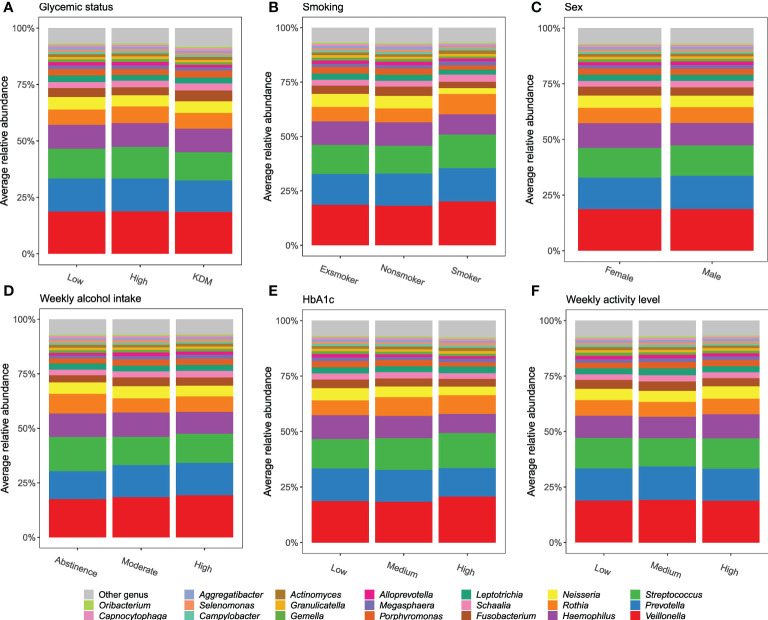
Predominant genera. Stacked bar charts of the 20 most abundant genera expressed as mean relative abundance. **(A–F)**: glycemic status, smoking, sex, weekly alcohol intake, HbA_1c_, and weekly activity level.

The core salivary microbiota consisted of 26 genera present in >95% of all samples, which accounted for 94.2% (range [45.4%; 99.8%]) of reads from each participant. The dominant genera were *Veillonella*, *Streptococcus*, *Haemophilus* and *Prevotella*, which represented 56.7% of the microbiota. In general, the composition of the predominant genera was not influenced by the variables tested (**Figure 2**, **Supplementary Figure 2**).

### Alpha and beta diversity

**Figure 3:**
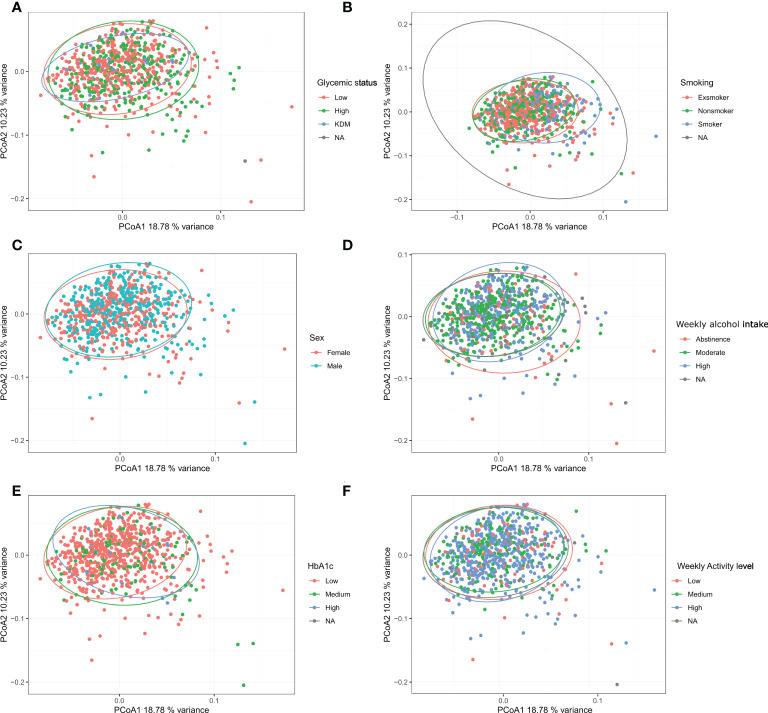
Beta diversity: Bray-Curtis dissimilarity calculated from Hellinger transformed total sum scaled data was used as beta-diversity measure and visualized with principal coordinate analysis (PCoA). **(A–F)**: glycemic status, smoking, sex, weekly alcohol intake, HbA_1c_, and weekly activity level.

Examining the alpha diversity between groups within the different variables in ADDITION-PRO, we found statistically significant differences in Richness for glycemic status, HbA_1c_, LDL cholesterol, total cholesterol and weekly alcohol intake, and in Shannon index for smoking, weekly alcohol intake and LDL cholesterol (**Table 2**, see **Supplementary Figure 3.1**, **3.2** for violin plots with ANOVA). To assess beta diversity, PCoA plots were used to visualize the data in a two-dimensional space representing most variation in the data, and plots were generated and colored according to each of the variables. No clear separation was observed, based on any of the variables included (**Figure 3** and **Supplementary Figure 4**). Using PERMANOVA on each variable individually, the variables glycemic status, HbA_1c_, sex, smoking and weekly alcohol intake were found to have a significant association with salivary microbial composition (PERMANOVA unadjusted p < 0.05). In a PERMANOVA run with all variables included, only smoking and weekly alcohol intake remained statistically significant (**Table 2**) and accounted for R2 = 0.030 (3%) and R2 = 0.006 (0.6%) of the observed sample variance in salivary microbial composition, respectively (**Table 2**). Running the model with the variables in a continuous instead of categorical form yielded similar results, both in terms of variables included and the magnitude of their contribution to microbial composition, though with the addition of BMI and average waist measurement, and the exclusion of Glycemic status and HbA_1c_, as significant factors (**Supplementary file 5**).

**Table 2:** Alpha diversity by Shannon index and Richness.

	Alpha diversity		PERMANOVA Individual variables		PERMANOVA all variables		PERMANOVA select variables		sPLS-DA	
Variable	Richness	Shannon	R2 (%)	P (>F)	R2 (%)	P (>F)	R2 (%)	P (>F)	AUC	CCR (%)
Smoking	0.3498	2.185x10^-0.5^	3.254	0.001	3.020	0.001	2.937	0.001	0.95	79.6
Alcohol intake	0.02736	0.04786	1.066	0.001	0.605	0.01	0.649	0.01	0.66 0.61 0.64	51.6
Glycemic status category	0.006883	0.08179	0.435	0.043	0.339	0.37	0.482	0.08	0.69	55.8
Sex	0.09062	0.995	0.334	0.01	0.270	0.08	0.290	0.04	0.73	51.3
HbA1c	0.0138	0.2477	0.698	0.002	0.408	0.15	0.440	0.12	0.66	83.9
Activity level	0.2949	0.1229	0.423	0.07	0.366	0.25	0.405	0.17	-	-
BMI	0.1654	0.8998	0.506	0.12	0.593	0.18	-	**-**	**-**	**-**
Blood pressure	0.148	0.2339	0.413	0.39	0.487	0.41	-	-	-	-
Systolic blood pressure	0.7762	0.2679	0.474	0.21	0.604	0.12	-	-	-	-
Diastolic blood pressure	0.5455	0.2382	0.563	0.08	0.464	0.48	-	-	-	-
Heart rate	0.3565	0.5538	0.377	0.10	0.301	0.50	-	-	-	-
Pulse pressure	0.7721	0.8423	0.091	0.77	0.258	0.08	-	-	-	-
Triglycerides	0.6569	0.07657	0.231	0.08	0.236	0.13	-	-	-	-
HDL cholesterol	0.7306	0.2483	0.203	0.12	0.085	0.91	-	-	-	-
LDL cholesterol	0.02109	0.03238	0.195	0.12	0.212	0,21	-	-	-	-
Total cholesterol	0.02435	0.2011	0.139	0.38	0.119	0,69	-	-	-	-
Waist	0.8994	0.6909	0.246	0.54	0.260	0,69	-	-	-	-
Waist to height ratio	0.2941	0.3842	0.189	0.15	0.188	0.27	-	-	-	-

PERMANOVA results for all variables individual and together. AUC and correct classification rate of predictive models using sPLSDA.

### Differential abundance

More than unique 40 genera were found to appear in significantly different abundances (p < 0.05), when examining differential abundance in groups within the variables found significant in the PERMANOVA runs (sex, glycemic status, HbA_1c_, weekly alcohol intake and smoking) (**Figure 4**, **Supplementary Figure 5**). Although significant, only minor differences in relative abundance were observed. Smoking status and sex were found to be associated with a substantially higher number of differentially abundant bacterial genera, as compared to the other variables. Genus *Streptococcus* was most frequently found to have a statistically significant differential abundance, and was associated with four out of the five variables of interest, namely smoking, weekly alcohol intake, HbA_1c_ levels and glycemic status. Overall, *Streptococcus* was associated with high-risk groups, with the exception of variable alcohol intake, where the genus appeared in the highest abundance in those who abstained from alcohol. Current smoking was significantly associated with lower abundance of *Neisseria*, and higher abundance of *Rothia* species compared to former-smokers and those who had never smoked.

**Figure 4.1:**
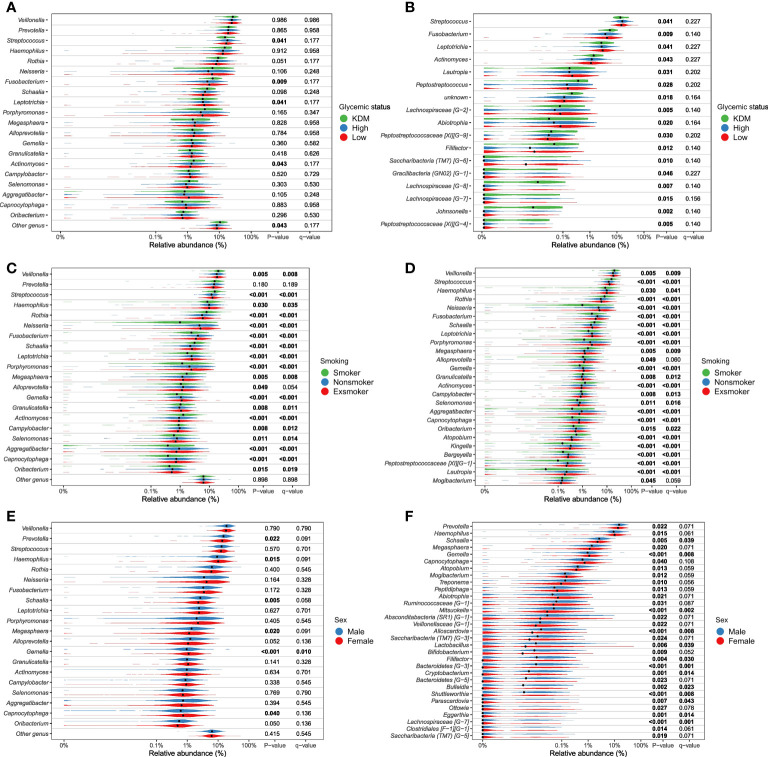
Genera significantly associated with glycemic status, smoking and sex. Relative abundance of most abundant genera (panels **A**, **C**, **E**) and differential abundant genera (panels **B**, **D**, **F**). Comparison of groups were performed with non-parametric tests (Kruskal-Wallis). Unadjusted p-values as well as Benjamini-Hochberg corrected q-values are included in the plot. **(A, B)**, glycemic status, **(C, D)**, smoking status, **(E, F)**, sex. All tests are included in **Supplementary File 2** as well as a parametric approach (DESeq as implemented in DAtest) in **Supplementary file 3**).

**Figure 4.2:**
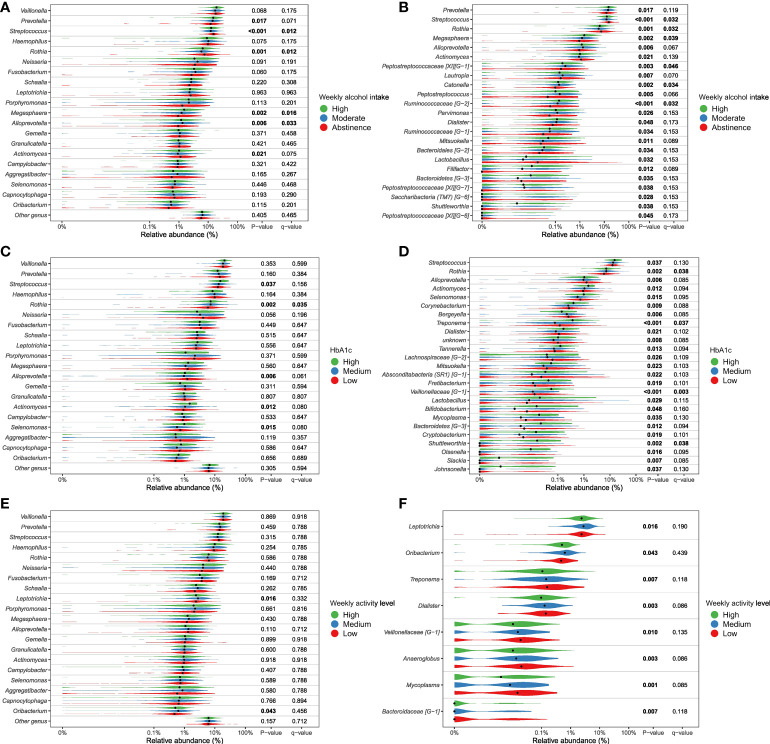
Genera significantly associated with alcohol intake, Hb1Ac and physical activity. Relative abundance of most abundant genera (panels **A**, **C**, **E**) and differential abundant genera (panels **B**, **D**, **F**). Comparison of groups were performed with non-parametric tests (Kruskal-Wallis). Unadjusted p-values as well as Benjamini-Hochberg corrected q-values are included in the plot. **(A, B)** weekly alcohol intake, **(C, D)** Hb1Ac, **(E, F)** weekly activity level. All tests are included in **Supplementary File 2** as well as a parametric approach (DESeq as implemented in DAtest) in **Supplementary File 3**).

### Variable classification potential of the salivary microbiota

**Figure 5:**
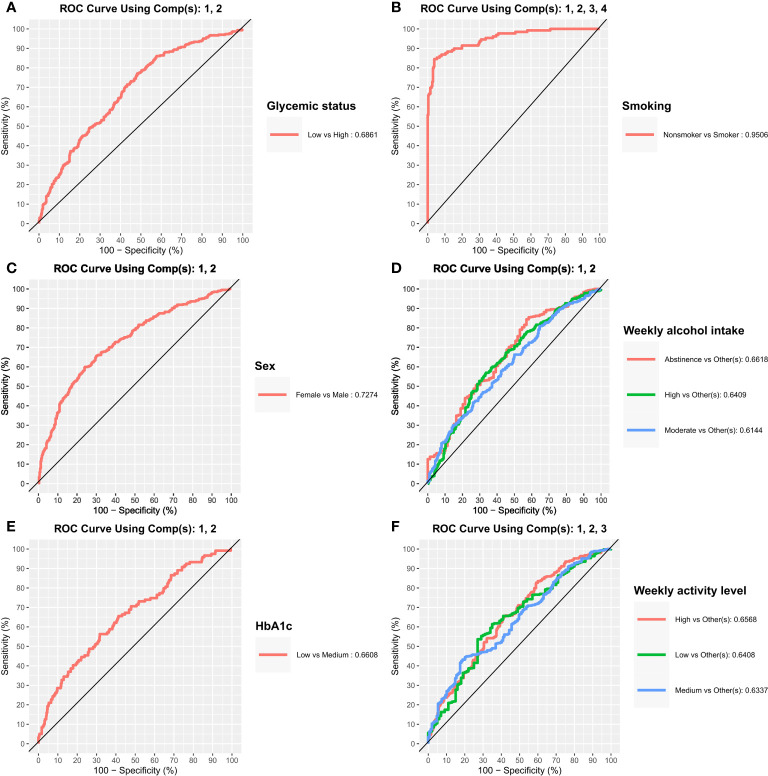
Classification potential of the salivary microbiota. Receiver Operating Characteristics (ROC) curves from sparse partial least squares discriminant analysis where used to assess classification of the different groups. Area under the curve (AUC) were used to quantify the performance of the classifier. **(A–F)** glycemic status, smoking, sex, weekly alcohol intake, HbA_1c_, and weekly activity level.

sPLSDA, was used both to investigate classification potential of the salivary microbiota and to investigate important features, and see if they correspond to the findings from the differential abundance analysis. sPLSDA was performed using the 5 variables found significant in the PERMANOVA (**Figure 5** and **Table 2**). The classifier performed well in differentiating between smokers and non-smokers with an AUC=0.95. A relatively high correct classification rate of 79.6% was obtained, when validating the classifier with a test set. The model performed almost equally with guessing, obtaining correct classification rates of approx. 50-60%, with regards to sex, glycemic status and weekly alcohol intake. It was not possible to construct a model of HbA_1c_ due to unbalanced group sizes and classifying almost all samples as a specific group.

## Discussion

The purpose of the present study was to evaluate the association of general medical disorders and lifestyle-associated factors with the composition of the salivary microbiota. We tested the hypothesis that the composition of the salivary microbiota associates with both general health status and lifestyle.

The main finding was that the variables (sex, glycemic status, HbA_1c_ levels, smoking and weekly alcohol intake), which in the present study were identified to have significant associations with the composition of the salivary microbiota, altogether accounted for only 5.8% of the observed differences in salivary microbial composition in the PERMANOVA run of individual variables (**Table 2**). In addition, none of the tested variables had a clear association with the variation of the data, as evaluated by clustering of samples in the PCoA plot (**Figure 3**). Thus, our data suggests that general health status and lifestyle-associated factors are not the main determinants of the composition of the salivary microbiota.

As expected, smoking was associated with characteristics of the salivary microbiota. Specifically, higher abundance of specific genera, such as *Veillonella*, *Streptococcus* and *Rothia*, and significantly lower abundance of *Neisseria*, *Haemophilus*, *Pophyromonas* and *Actinomyces* in smokers compared to ex-smokers and never-smokers, was observed (**Figure 4.1C, D**). In general, these findings are in line with previous studies, which have also identified taxonomic differences in the salivary microbiota in smokers versus non-smokers in different populations, with varying sample size (Wu et al., 2016; Rodríguez-Rabassa et al., 2018; Suzuki et al., 2022; Wang et al., 2022). Thus, our data confirms that smoking to some degree associates with characteristics of the salivary microbiota. However, the PERMANOVA analysis revealed that smoking status accounted for as little as 3.3% of the observed differences between individuals in the PERMANOVA run of individual variables, and 2.9% in the PERMANOVA run of select variables together (**Table 2**). Data from the present study suggest only a minor association of smoking and the composition of the salivary microbiota, which is different from the subgingival microbiota, which has been shown to strongly associate with smoking status. Nevertheless, it is noteworthy that it was still possible to build a good classifier based on the salivary microbiota to differentiate between current smokers and current non-smokers in the present study. Indeed, we are not the first to do so, as in 2022, another study used data on the salivary microbiota from 175 current smokers and 1070 ex- and non-smokers to create a model that achieved an AUC of 0.81 (Díez López et al., 2022), which is comparable with our findings (**Table 2**). Importantly, when considering that smoking, which accounted for only 3.2% of differences in our dataset, can deliver such a robust model, this reinforces the concept of using the salivary microbiota for identification of oral diseases, as periodontitis has been shown to have a much higher impact on the composition of the salivary microbiota, than smoking (Belstrøm et al., 2021).

Second to smoking, weekly alcohol intake was the variable with the greatest association with salivary microbial composition. Specifically, in the individual PERMANOVA analysis, weekly alcohol intake accounted for 1% of the observed difference in the composition of the salivary microbiota, which decreased to 0.6%, when including all variables in the same model (**Table 2**). The considerable decrease in contribution of weekly alcohol intake to salivary microbial composition between the two PERMANOVA runs was most likely the consequence of the high degree of correlation between smoking status and weekly alcohol intake, as seen in **Figure 1**. As such, confounding by smoking likely yields an artificially high estimate of the effect of weekly alcohol intake in unadjusted analyses. Likewise, it is likely that there are many other confounding pathways, such as socio-economic status and diet, which could be linked with both a higher alcohol intake and differences in the salivary microbiota. It should be noted that our measure of alcohol intake was self-reported and is therefore potentially subject to information bias (social desirability and recall bias). Nevertheless, we found genus *Prevotella* to significantly associate with alcohol intake, as evaluated by almost 2% higher relative abundance in individuals with high intake versus low intake of alcohol (**Figure 4.2A, B**). This result is in line with findings from previous reports (Fan et al., 2018; Liao et al., 2022). On the other hand, our finding of a lower abundance of *Streptococcus* in individuals with high alcohol intake is in conflict with previous studies, which finds *Streptococcus* to be higher in individuals with high alcohol intake (Fan et al., 2018; Barb et al., 2022). Taken together, our data suggest that alcohol intake may only cause relatively minor perturbations to the salivary microbiota. However, it should be noted that the majority of the study subjects (40%) display moderate drinking habits, with only a minority of the 35% in the category of heavy drinkers drinking more than 20 units of alcohol per week. As such, it is not unlikely that a high alcohol intake may associate with salivary microbial composition, but that our data does not show it, as our population is skewed towards drinking less.

Glycemic status and HbA_1c_ were both found to have a significant association with salivary microbial composition, albeit a very small one (**Table 2**). Quite a few studies, most with a much smaller sample size than the present study, have previously examined the association of T2D with the oral microbiota. In general, previous data have been conflicting, where some studies find higher bacterial diversity (Casarin et al., 2013; Ganesan et al., 2017; Chen et al., 2020), and others lower in individuals with T2D (Ogawa et al., 2017; Longo et al., 2018; Saeb et al., 2019; Matsha et al., 2020; Yang et al., 2020; Sabharwal et al., 2021). In addition, several studies have reported no significant difference of the oral microbiota in people with diabetes and healthy controls (Kampoo et al., 2014; Janem et al., 2017; Sun et al., 2020; Almeida-Santos et al., 2021; Liu et al., 2021). It is therefore noteworthy that data from the present study clearly shows that diabetes, as evaluated by glycemic status and HbA_1c_ level, only account for 0.4% and 0.3% of the observed sample differences, respectively (**Table 2**), and that these variables had a minimal influence on relative abundance of predominant genera (**Figure 2**) and variation of the data (**Figure 3**). Moreover, when running the PERMANOVA with all variables, no significant association between glycemic status and HbA_1c_, was observed (**Table 2**). We therefore speculate that the minimal effect of glycemic status and HbA_1c_ on salivary microbial composition observed in the individual PERMANOVA runs, were merely the consequence that glycemic status and HbA_1c_ were highly correlated with smoking status (**Figure 1**). Thus, data shows a minimal direct association of glycemic status and HbA_1c_ level with the composition on the salivary microbiota, suggesting that the salivary microbiota is probably not a good biomarker candidate for classification of diabetes. This finding is in contrast to a recent study in 133 individuals with and without periodontitis and/or diabetes, using oral microbial composition to create models, which classified groups with AUCs between 96.3% and 100% (Sun et al., 2020). One possible explanation to this discrepancy may be that our model was only constructed to classify glycemic status groups or HbA_1c_ levels, as compared to the other study, where the model distinguished individuals with different glycemic states combined with different periodontal status. Indeed, periodontitis is associated with significant changes in oral microbial composition (Sun et al., 2020; Ko et al., 2020), which may have contributed a stronger predictive ability, than we achieved in the present study using diabetic parameters alone. Taken together, there is a need for well-constructed studies with even larger sample sizes in order to determine, whether glycemic status is independently associated with the salivary microbiota, or merely the consequence of the association of periodontitis and T2D.

In addition to the aforementioned variables, sex showed a statistically significant association with salivary microbial composition, as evaluated by PERMANOVA (p < 0.05). However, sex accounted for just 0.3% of the observed differences in salivary microbial composition in this study sample (**Table 2**), and had minimal influence on relative abundance of predominant genera (**Figure 2**) and clustering of data (**Figure 3**). Moreover, by use of PERMANOVA we did not find any significant associations of anthropometric factors such as BMI, waist circumference, waist- to-hip ratio, blood pressure and dyslipidemia with the composition of the salivary microbiota. Previous studies have reported age (LaMonte et al., 2019; Chaudhari et al., 2020; Liu et al., 2020; Nearing et al., 2020), sex (Fischer et al., 2008; Nearing et al., 2020; Minty et al., 2021; Zhao et al., 2021) and anthropometric factors (Takeshita et al., 2016; Si et al., 2017; Tam et al., 2018; Fei et al., 2019; Yang et al., 2019; Nearing et al., 2020; LaMonte et al., 2022) to associate with characteristics of the oral microbiota. These findings are in line with differential abundance data from the present study, which identifies relative abundance of a number of bacterial genera to associate with each of the variables (**Figures 4.1**, **4.2**; **Supplementary Figure 5**). However, the small magnitude of the differences observed, questions the clinical relevance of these findings.

The main limitation of the present study is that the oral health status was not recorded, as part of data collection in ADDITION-PRO. Indeed, periodontitis associates with both T2D and cardiovascular disease (Holmstrup et al., 2017), through systemic low-grade inflammation (Hajishengallis and Chavakis, 2021), and smoking is a risk factor of periodontitis (Rivera-Hidalgo, 2000). Moreover, based on epidemiological data from the US (Eke et al., 2020) we should expect at least 40% of the participants to have some degree of periodontitis, since the mean age in the ADDITION-PRO cohort is 68 years (**Table 1**). Likewise, epidemiological data from China suggest that as much as 60% of the cohort could have treatment requiring dental caries (Liu et al., 2013). Importantly, periodontitis and dental caries has been shown to associate with characteristics of the salivary microbiota (Belstrøm, 2020). In the same vein data on the salivary flow rate of participants were not collected, despite it being an important risk factor for oral disease (Dodds et al., 2015; Vallabhan et al., 2020). Thus, it is almost certain that the potentially heterogeneous oral health status of the ADDITION-PRO cohort may have confounded the observed associations. Another limitation was the selection process of ADDITION-PRO, which involved progressive diabetes screening steps, resulting in a population of largely middle-aged and predominantly sedentary individuals, which are not representative of the background population, and therefore hampers the external validity of the data presented. The main strength of the present study is the relatively large sample size, in combination with detailed information on a large number of highly relevant variables. Accordingly, this provided a hitherto unprecedented opportunity to characterize the association between multiple dependent variables and the composition of the salivary microbiota in one single cohort. In the **Supplementary files 2-3**, we offer an overview of all genera found to be significant for all variables examined, for comparison of data in future studies of salivary microbial composition.

In conclusion, data from the present study shows a modest association of general health and lifestyle-associated parameters with the composition of the salivary microbiota. However, it should be emphasized that our data on explained variance estimates are highly sample specific and not estimates of population explained variance. Consequently, data from the present study should be validated in independent cohorts, preferably with the inclusion of data on the oral health status of study participants, which was not available in the present study. Despite smoking accounting for only 3% of the difference observed, it was possible to create a model for classification of smoking status, with a correct classification rate of 79.6%. Given the fact that presence of manifest periodontitis and dental caries have much stronger associations with the composition of the salivary microbiota than smoking, further studies are warranted to determine, whether the salivary microbiota can be used as a diagnostic biomarker of periodontitis and dental caries at preclinical stages.

## Data availability statement

The data for this study have been deposited in the European Nucleotide Archive (ENA) at EMBL-EBI under accession number PRJEB57196 (https://www.ebi.ac.uk/ena/browser/view/PRJEB57196).

## Ethics statement

The studies involving human participants were reviewed and approved by The scientific ethics committee in the Central Denmark Region (case ID: H-20000183). The patients/participants provided their written informed consent to participate in this study.

## Author contributions

Data collection and curation: DW, OP, and TH. Data pre-processing, statistics, and analysis: CS, TK, FC, and NN. Conceptualisation: TK, DB, NN, CS, and TH. Writing: NN, DB, and CS. Editing: NN, DB, CS, ES, DW, OP, TH, and FC. All authors contributed to the article and approved the submitted version.

## Conflict of interest

The authors declare that the research was conducted in the absence of any commercial or financial relationships that could be construed as a potential conflict of interest.

## Publisher’s note

All claims expressed in this article are solely those of the authors and do not necessarily represent those of their affiliated organizations, or those of the publisher, the editors and the reviewers. Any product that may be evaluated in this article, or claim that may be made by its manufacturer, is not guaranteed or endorsed by the publisher.

## Supplementary material

Supplementary Figure 1 Comparison of sequencing batches. Bray-Curtis dissimilarity calculated from Hellinger transformed total sum scaled data was used as beta-diversity measure and visualized with principal coordinate analysis (PCoA).

Supplementary Figure 2 Predominant genera. Stacked bar charts of the 20 most abundant genera aggregated for variables that was not significant by PERMANOVA analysis. The variables include BMI, waist measurement, waist-to-height ratio, blood pressure, systolic blood pressure, diastolic blood pressure, heart rate, pulse pressure, triglyceride levels, HDL cholesterol, LDL cholesterol and total cholesterol.

Supplementary Figure 3.1 Alpha diversity. Comparison of alpha diversity between selected samples variable groups by richness (panels **A, C, E, G, I** and **K**) and Shannon diversity index (panels **B, D, F, H, J** and **L**), with ANOVA. Selected variables include weekly alcohol intake, HbA1c, and weekly activity level depicted in panel **(A–L)** respectively.

Supplementary Figure 3.2 Alpha diversity. Comparison of alpha diversity between additional samples variable groups by richness (panels **A, C, E, G, I, K, M, O, Q, S, U** and **W**) and Shannon diversity index (panels **B, D, F, H, J, L, N, P, R, T, V** and **X**), with ANOVA. The variables include BMI, waist measurement, waist-to-height ratio, blood pressure, systolic blood pressure, diastolic blood pressure, heart rate, pulse pressure, triglyceride levels, HDL cholesterol, LDL cholesterol and total cholesterol depicted in panel **(A–X)**.

Supplementary Figure 4 Beta diversity. Comparison of beta diversity exploring differences between additional sample variables. Bray-Curtis dissimilarity calculated from Hellinger transformed total sum scaled data was used as beta-diversity measure and visualized with principal coordinate analysis (PCoA). The variables include BMI, waist measurement, waist-to-height ratio, blood pressure, systolic blood pressure, diastolic blood pressure, heart rate, pulse pressure, triglyceride levels, HDL cholesterol, LDL cholesterol and total cholesterol depicted in panel **(A–X)**, respectively.

Supplementary Figure 5 Relative abundance plots. Relative abundance of most abundant genera (panels **A, C, E, G, I, K, M, O, Q, S, U** and **W**) and differential abundant genera (panels **B, D, F, H, J, L, N, P, R, T, V** and **X**). Variables include BMI, waist measurement, waist-to-height ratio, blood pressure, systolic blood pressure, diastolic blood pressure, heart rate, pulse pressure, triglyceride levels, HDL cholesterol, LDL cholesterol and total cholesterol depicted in panel (**A–X**), respectively. Comparison of groups were performed with non-parametric tests (Kruskal-Wallis). Unadjusted as well as Benjamini-Hochberg corrected q-values are included in the plot. All tests are included in supplementary file 2 as well as a parametric approach (DESeq as implemented in DAtest) in supplementary file 3).
