# Supplementary material for: Association of general health and lifestyle factors with the salivary microbiota – Lessons learned from the ADDITION-PRO cohort
Source: Front Cell Infect Microbiol. 2022 Nov 16;12:1055117. doi: 10.3389/fcimb.2022.1055117 (PMC9709502; doi:10.3389/fcimb.2022.1055117)
Supplement: Supplementary Figure 1 — Comparison of sequencing batches. Bray-Curtis dissimilarity calculated from Hellinger transformed total sum scaled data was used as beta-diversity measure and visualized with principal coordinate analysis (PCoA). [file DataSheet_1.zip › Supplementary_file_1_AdditionPRO_metabaRpipe_220809.pdf]

Updated metabaRpipe AdditionPRO exploration microbiome

Casper Sahl Poulsen

22042022

Contents

Introduction 2
Samples 2
Sample selection 3
Other datasets 3
Packages 3
Reading in new data 4
Comparing with previous data 5
Pre-processing 6
Subset Pheno 6
Modify microbiome 10
Functional prediction Picrust2 12
Remove unused datasets and objects 12
Compare sequencing runs 13
Remove re samples 21
Define phenotypes according to categorical categories 21
Analyses 27
Phenotype overview 27
Model sample variables 41
Contingency tables 41
Correlation network of sample variables 65
Stacked bar charts and bubble plots 68
Alpha diversity 72
Microbiome 75
PCoA, rabuplot and PERMANOVA 118
Microbiome in loop 121
PERMANOVA 229

MaAsLin: Multivariate Association with Linear Models . . . 230
Filtration for both DAtest and sPLSDA also core microbiome . . . 233
DAtest . . . 233
sPLS-DA mixOmics . . . 483
Smoking . . . 483
Smoking clr . . . 502
sex_cat . . . 519
Risk . . . 534
Alcprweek_cat . . . 549
BMI_cat . . . 569
act_cat . . . 569
hba1c_cat . . . 584
Additional . . . 598
Session information . . . 598
This document was processed on: . . . 600

Introduction

This is an updated script including the data that was run with the metabaRpipe pipeline. https://github.com/fconstancias/metabaRpipe. See Rstudio server for detailed running of the pipeline. Summary below:

```
#Custom script from Oto to cp and rename cp_and_rename.sh also solved issue with wrong naming of files
#Had four files (10282, 10329, 10585, 10817) in both batches renamed to .....re in batch 120, but 10282
#Not able to submit with sbatch submitted with a screen (TMUX)


Rscript ${MY_DIR}metabaRpipe/Rscripts/dada2_metabarcoding_pipeline.Rscript \
-i metabaRpipe/test-data/ \
--preset V4-Addition-PRO \
--rm_primers FALSE \
--db /home/lqr378/metabaRpipe/test4_cloneagain_ownsmall/metabaRpipe/databases/eHOMD_RefSeq_dada2_V15.22
--db_species /home/lqr378/metabaRpipe/test4_cloneagain_ownsmall/metabaRpipe/databases/eHOMD_RefSeq_dada
--metadata metabaRpipe/metadata.xlsx \
--save_out test_pipe_Rscript.RDS \
-f ${MY_DIR}metabaRpipe/Rscripts/functions.R > mylogs.txt 2>&1
```

This script is initially only intended to investigate microbiome, see other script for preliminary analyses of the metabolomics.

Samples

Ref: https://www.ncbi.nlm.nih.gov/pmc/articles/PMC3583712/ "Protocol for ADDITION-PRO: a longitudinal cohort study of the cardiovascular experience of individuals at high risk for diabetes recruited from Danish primary care"

Sample selection

How were samples selected for omics analysis??? Maybe all were included where saliva was available (Saliva n=786).

Other datasets

Metabolomics: Metabolomics saliva. Extracted with 4-vol methanol and analysed by reverse phase LC-MS in both negative and positive mode. Metabolites are identified by their m/z, fragmentation pattern and intensities normalized to QC samples. Two samples were excluded due to lack of material.
In file obtained data from 775 samples on 224 metabolites.
GWAS: Genotyped with Illumina Infinium HumanCoreExome Beadchip(n=1657) Phenotypic data: Diabetes risk, anthropometry, body composition, biochemistry, physical activity and cardiovascular risk factors including aortic stiffness and central blood pressure.

Packages

```r
##install.packages("Rmisc")
#library(Rmisc)

library(plyr)

#install.packages(c("dplyr"))
library(dplyr)

#install.packages("readxl")
library("readxl")

#install.packages("VennDiagram")
library(VennDiagram)

library(gridExtra)

library(stringr)

library(ggplot2)

library(vegan)

#install.packages("plotly")
library(plotly)

library(igraph)

library(knitr)

#install.packages("BiocManager")
#BiocManager::install("SIAMCAT")
#library("SIAMCAT")

#install.packages("BiodiversityR")
```

```r
library(BiodiversityR)

#install.packages("reshape2")
library(reshape2)

#BiocManager::install("DESeq2")
library("DESeq2")

#devtools::install_bitbucket("biobakery/maaslin2@default", ref="tip")
#BiocManager::install("Maaslin2")
library("Maaslin2")

library("phyloseq")

#install.packages("remotes")
#remotes::install_github("jstokholm/rabuplot")
library("rabuplot")

#install.packages("ggmosaic")
library("ggmosaic")

#install.packages("ggraph")
library("ggraph")

library("igraph")

library("pheatmap")

library("RColorBrewer")

library("cowplot")

library("forcats")

#BiocManager::install('mixOmics')
library("mixOmics")

library("ggpubr")

#remotes::install_github("jonathanth/copiome@main", force=TRUE)
library("copiome")
```

Reading in new data

```r
readRDS("dada2/phyloseq.RDS") -> ps

ps
```

```
## phyloseq-class experiment-level object
## otu_table()   OTU Table:         [ 5748 taxa and 789 samples ]
## sample_data() Sample Data:       [ 789 samples by 18 sample variables ]
```

```
## tax_table()   Taxonomy Table:    [ 5748 taxa by 7 taxonomic ranks ]
## refseq()      DNAStringSet:      [ 5748 reference sequences ]
```

```
#ps %>% refseq()

#ps %>%  tax_table()
feature <- ps %>%  tax_table() %>% data.frame()


#ps %>%  otu_table()
microbio <- ps %>%  otu_table() %>% data.frame() %>% t() %>% data.frame() #Adds X

#ps %>% sample_data()
metaq <- ps %>% sample_data()


#readRDS("dada2/test_pipe_Rscript.RDS") -> out #Large file
#ls(out)
#Contains "filtering_denoising", "merging", "physeq", "qplot", "taxo"
```

Comparing with previous data

```
load("dada2/DenoisedData.RData") #From Timo
Microbio <- data.frame(seqtab.nochim)
#Total reads
sum(ReadSummary$NoReads)
```

```
## [1] 33448846
```

```
mean(ReadSummary$NoReads)
```

```
## [1] 42555.78
```

```
sd(ReadSummary$NoReads)
```

```
## [1] 22408.29
```

```
median(ReadSummary$NoReads)
```

```
## [1] 39927
```

```
range(ReadSummary$NoReads)
```

```
## [1]   1234 317076
```

```
sum(Microbio)
```

```
## [1] 20669468
```

```r
#Microbio: 786 obs. of 3319 variables old
#microbio: 789 obs. of 5748 variables new but below renames to Microbio a little confusing
mean(colSums(Microbio))
```

```
## [1] 6227.619
```

```r
sd(colSums(Microbio))
```

```
## [1] 61226.71
```

```r
median(colSums(Microbio))
```

```
## [1] 68
```

```r
range(colSums(Microbio))
```

```
## [1]       2 2002742
```

```r
## Phenotypes
#Shared
length(intersect(paste("X", rownames(Microbio), sep=""), rownames(microbio)))
```

```
## [1] 786
```

```r
setdiff(paste("X", rownames(Microbio), sep=""), rownames(microbio))
```

```
## character(0)
```

```r
setdiff(rownames(microbio), paste("X", rownames(Microbio), sep="")) #Only the 3 re
```

```
## [1] "X10329re" "X10585re" "X10817re"
```

```r
#load("../../../Data/TimoMail210227/AdditionPro_data_objects/HOMD_Taxonomy.RData")
#FeatureHOMD <- data.frame(taxa)
#Feature<-FeatureHOMD
```

Pre-processing

Subset Pheno

```r
##Overwrite the previous files to make naming compatible with the rest of the script copied from
  #AdditionPRO_MetaboExpl_200710.Rmd. Is confusing since above the old ones are named the same
Feature <- feature
Microbio <- microbio
```

```r
##Phenotypes
#From Anna mail
Pheno <- read.delim(file="dada2/AdditionPro_05122014_final_primaryID.txt",
                       check.names=FALSE,
                       stringsAsFactors = FALSE,
                       strip.white=TRUE,
                       dec=".")

#PhenoExp <- read_excel("../../../Data/Variable name for Physical activity_AddPro.xlsx")
  #169 variables explained

Pheno$IDX<-paste("X", Pheno$pro_id, sep="")

#Add "X10329re" "X10585re" "X10817re" samples as a copy of not re with rbind
PhenoRE<-filter(Pheno, IDX=="X10329" | IDX=="X10585" | IDX=="X10817")
PhenoRE$IDX <- paste(PhenoRE$IDX, "re", sep="")
Pheno <- rbind(PhenoRE, Pheno)


length(intersect(Pheno$IDX, rownames(Microbio))) #746 (749 with re)
```

```
## [1] 749
```

```r
#setdiff(Pheno$IDX, rownames(Microbio)) #samples in pheno not in microbio
setdiff(rownames(Microbio), Pheno$IDX) #Samples in microbio not in pheno
```

```
##  [1] "X10003" "X10004" "X10005" "X10401" "X10417" "X10425" "X10445" "X10463"
##  [9] "X10471" "X10502" "X10006" "X10308" "X10406" "X10415" "X10416" "X10418"
## [17] "X10424" "X10431" "X10433" "X10434" "X10435" "X10436" "X10443" "X10444"
## [25] "X10446" "X10447" "X10448" "X10450" "X10451" "X10456" "X10457" "X10460"
## [33] "X10464" "X10467" "X10468" "X10472" "X10501" "X10531" "X10653" "X10774"
```

```r
#missingsamples<-setdiff(rownames(Microbio), Pheno$IDX) #Samples in microbio not in pheno
#write.table(missingsamples, file="../ADDPRO_missingpheno_220426.txt",
#             quote = F, row.names = F, sep="\t")
length(setdiff(rownames(Microbio), Pheno$IDX)) #43 Samples not in Pheno (40 after include re)
```

```
## [1] 40
```

```r
#Consider to remove 0 and 6 gly_stat and pool group 2-5.
table(Pheno$gly_stat, useNA="always")
```

```
##
##    0    1    2    3    4    5    6 <NA>
##   15  901  334  133  162  183  334    0
```

```r
##Remove group 0 and 6
#Pheno<-subset(Pheno, gly_stat %in% c(1:5))

#Make cat variable gly_stat
```

```r
Pheno$Glycaemia_Status<-ifelse(Pheno$gly_stat==0,
                                   "Unclass",
                               ifelse(Pheno$gly_stat==1,
                                   "Gr1ngt",
                                   ifelse(Pheno$gly_stat==2,
                                      "Gr2ilFG",
                                      ifelse(Pheno$gly_stat==3,
                                             "Gr3ilGT",
                                             ifelse(Pheno$gly_stat==4,
                                             "Gr4IFG_IGT",
                                             ifelse(Pheno$gly_stat==5,
                                             "Gr5SDM",
                                             ifelse(Pheno$gly_stat==6,
                                             "KDM",

                 "UA")))))))
table(Pheno$Glycaemia_Status, useNA="always")
```

```
##
##     Gr1ngt    Gr2ilFG    Gr3ilGT Gr4IFG_IGT     Gr5SDM        KDM    Unclass
##        901        334        133        162        183        334         15
##       <NA>
##          0
```

```r
#Make classification diabetes risk based on gly_stat
Pheno$Risk<-as.character(ifelse(Pheno$gly_stat == 0, "Unclass",
                              ifelse(Pheno$gly_stat == 1, "Low",
                                     ifelse(Pheno$gly_stat == 2 | Pheno$gly_stat == 3 | Pheno$gly_stat =
                                            ifelse(Pheno$gly_stat == 6, "KDM",
                              "UA")))))
table(Pheno$Risk, useNA="always")
```

```
##
##    High      KDM      Low Unclass     <NA>
##     812      334      901      15        0
```

```r
#Create vector names of shared samples
length(intersect(Pheno$IDX, rownames(Microbio))) #Shared samples 722 (725 with re), after keep gly_stat
```

```
## [1] 749
```

```r
setdiff(rownames(Microbio), Pheno$IDX) #Samples in microbio not in pheno
```

```
##  [1] "X10003" "X10004" "X10005" "X10401" "X10417" "X10425" "X10445" "X10463"
##  [9] "X10471" "X10502" "X10006" "X10308" "X10406" "X10415" "X10416" "X10418"
## [17] "X10424" "X10431" "X10433" "X10434" "X10435" "X10436" "X10443" "X10444"
## [25] "X10446" "X10447" "X10448" "X10450" "X10451" "X10456" "X10457" "X10460"
## [33] "X10464" "X10467" "X10468" "X10472" "X10501" "X10531" "X10653" "X10774"
```

```r
length(setdiff(rownames(Microbio), Pheno$IDX)) #67 Samples not in Pheno (64 with re), after keep gly_st
```

```
## [1] 40
```

```r
y<-intersect(Pheno$IDX, rownames(Microbio))

#Transpose
Microbio<-data.frame(t(Microbio))
MicrobioSub<-dplyr::select(Microbio, one_of(y))

PhenoSub<-subset(Pheno, IDX %in% y)

#Sammenligner glycemia status
table(PhenoSub$gly_stat)
```

```
##
##   0   1   2   3   4   5   6
##   3 424 112  58  63  68  21
```

```r
table(PhenoSub$gly_stat)/nrow(PhenoSub)
```

```
##
##          0          1          2          3          4          5          6
## 0.00400534 0.56608812 0.14953271 0.07743658 0.08411215 0.09078772 0.02803738
```

```r
table(Pheno$gly_stat)
```

```
##
##   0   1   2   3   4   5   6
##  15 901 334 133 162 183 334
```

```r
table(Pheno$gly_stat)/nrow(Pheno)
```

```
##
##           0           1           2           3           4           5
## 0.007274491 0.436954413 0.161978661 0.064500485 0.078564500 0.088748788
##           6
## 0.161978661
```

```r
#By group risk
aggregate(PhenoSub[, c(5, 9:11)], list(PhenoSub$Risk), mean)
```

```
##   Group.1       sex   height   weight      bmi
## 1    High 0.5980066 171.3186 81.87309 27.80931
## 2     KDM 0.5238095 170.3190 82.51905 28.44234
## 3     Low 0.5094340 170.7208 76.28608 26.08956
## 4 Unclass 0.3333333 171.0333 85.63333 29.41812
```

```r
aggregate(PhenoSub[, c(5, 9:11)], list(PhenoSub$Risk), sd)
```

```
##   Group.1       sex   height    weight      bmi
## 1    High 0.4911171 8.872489 14.947759 4.185932
## 2     KDM 0.5117663 8.160798 13.716837 4.384448
## 3     Low 0.5005016 9.499058 14.294270 3.969799
## 4 Unclass 0.5773503 7.569897  1.871719 3.092529
```

Modify microbiome

```r
#Merge feature and count table
TaxEdit <- merge(Feature, MicrobioSub, by="row.names")

#Aggregate the rows that have exactly the same entries and sum counts
Tax2<-TaxEdit %>% group_by(Kingdom, Phylum, Class, Order, Family, Genus) %>%
  summarise_if(is.numeric, funs(sum)) #193 genera previous 164 genera for further analyses

## Select how to handle NA
######### Remove all rows containing na values at the specified taxonomic level #########

##Warning, discuss if this is the right way
#before<-colSums(Tax2[,7:ncol(Tax2)])
#Tax2 <- Tax2[!is.na(Tax2$Genus), ]
#after<-colSums(Tax2[,7:ncol(Tax2)])
#before-after
##How much data is lost by doing this
#(before-after)/before

####################### Put all NA into an unknown category ############################

#Tax2$Genus <- as.character(Tax2$Genus)
#Tax2$Genus <- ifelse(is.na(Tax2$Genus), "Unknown", Tax2$Genus)

#if (sum(table(Tax2$Genus)>1)!=1) {
#  stop("Need unique entries for all genera except unknown")
#}

#res <- Tax2 %>%
#  mutate(Genus = ifelse(Genus == "Unknown",
#                            paste("Class(", as.character(Class), ")", sep=""), Genus))

#res <- Tax2 %>%
#  filter(Genus=="Unknown") %>%
#  mutate(Genus = as.character(Class))

#res <- df %>%
#  filter(category=="Shirts") %>%
#  mutate(category=subcategory)

##Aggregate the unknwown
#Tax2<-Tax2 %>% group_by(Kingdom, Phylum, Class, Order, Class, Genus) %>%
```

```r
#  summarise_if(is.numeric, funs(sum))

#### Create classification for NA to higher taxonomic assignment in all tax columns #####

###Does not work
##for (i in c(2,3,4,5,6)) {
##  Tax2[,i] <- if(is.na(Tax2[,i])
##                 {replace(paste("(", colnames(Tax2[,c(i-1)]),
##                      as.character(Tax2[,c(i-1)]), ")", sep="")))
##  #Tax2[,i] <- if(is.na(Tax2[,i]), )
##  }
##}

############ Create classification for NA to higher taxonomic assignment ################

##Create classification for NA to higher taxonomic assignment
##Put Class NA into an unknown category
#Tax2$Species <- as.character(Tax2$Species)
#Tax2$Species <- ifelse(is.na(Tax2$Species), "Unknown", Tax2$Species)

Tax2$Genus <- as.character(Tax2$Genus)
Tax2$Genus <- ifelse(is.na(Tax2$Genus), "unknown", Tax2$Genus)
Tax2$Genus <- str_replace(Tax2$Genus, "-", ".")

##Have two entries named acidifaciens
#Tax2$Family[Tax2$Family == "Family_XI"  & Tax2$Order == "Clostridiales"]<-
#  "Family_XI(Order(Clostridiales))"
#Tax2$Family[Tax2$Family == "Family_XI"  & Tax2$Order == "Bacillales"]<-
#  "Family_XI(Order(Bacillales))"

##Many entries are the same. Provide full species name
#Tax2$Species <- as.character(paste(Tax2$Genus, Tax2$Species, sep = "_"))


#Check if there are multiple entries of the same
if (sum(table(Tax2$Genus)>1)!=1) {
  stop("Need unique entries for all species except unknown")
}

#Assigning new names depending on higher taxonomic rank
Tax2 <- Tax2 %>%
  mutate(Genus = ifelse(Genus == "unknown",
                        paste("Family.", as.character(Family), ".", sep=""), Genus))
Tax2 <- Tax2 %>%
  mutate(Genus = ifelse(Genus == "Family.unknown.",
                        paste("Order.", as.character(Order), ".", sep=""), Genus))
Tax2 <- Tax2 %>%
  mutate(Genus = ifelse(Genus == "Order.unknown.",
                        paste("Class.", as.character(Class), ".", sep=""), Genus))
Tax2 <- Tax2 %>%
  mutate(Genus = ifelse(Genus == "Class.unknown.",
                        paste("Phylum.", as.character(Phylum), ".", sep=""), Genus))
Tax2 <- Tax2 %>%
```

```r
  mutate(Genus = ifelse(Genus == "Phylum.unknown.",
                        paste("Kingdom.", as.character(Kingdom), ".", sep=""), Genus))

#Assigning names to the phylum column
##Put Phylum NA into an unknown category
Tax2$Phylum <- as.character(Tax2$Phylum)
Tax2$Phylum <- ifelse(is.na(Tax2$Phylum), "unknown", Tax2$Phylum)
Tax2 <- Tax2 %>% ungroup() %>%
  mutate(Phylum = ifelse(Phylum == "unknown",
                         paste("Kingdom.", as.character(Kingdom), ".", sep=""), Phylum))

#Made unknown phyla into Kingdom.Bacteria. and Kingdom.unknown.
table(Tax2$Phylum)
```

```
##
## Absconditabacteria_(SR1)           Actinobacteria            Bacteroidetes
##                        1                       29                       21
##                 Chlorobi              Chloroflexi            Cyanobacteria
##                        1                        1                        1
##               Firmicutes             Fusobacteria   Gracilibacteria_(GN02)
##                       77                        5                        2
##        Kingdom.Bacteria.         Kingdom.unknown.           Proteobacteria
##                        1                        1                       41
##   Saccharibacteria_(TM7)             Spirochaetes            Synergistetes
##                        8                        1                        3
```

```r
##########################################################################################
#Make feature table
FeatureMic<-Tax2[,1:6]


##Include for all selections on how to handle NA
#Add Class as row names and keep numeric columns
#rownames(Tax2) <- Tax2$Class
Microbio<-data.frame(Tax2[,7:ncol(Tax2)], row.names = Tax2$Genus,
                     check.names=FALSE) #Change when having other taxonomy tables

# Rename phenotypes
Phe<-PhenoSub
```

Functional prediction Picrust2

See AdditionPRO_FUNCpicrust_210823 for generation of data, have to update according to new data

```r
#FeaturePic2<-read.csv(file="../../Data/OtoMail210827/pathways/metacyc_categories.csv")
#Pic2<-read.table(file="../../Data/path_abun_unstrat.tsv", header=TRUE)
```

Remove unused datasets and objects

```
rm(list=setdiff(ls(), c("FeaturePic2", "Pic2", "Phe", "Microbio", "FeatureMic")))
```

Compare sequencing runs

```
rm(list=setdiff(ls(), c("FeaturePic2", "Pic2", "Phe", "Microbio", "FeatureMic")))

#Read in metadata from dada2
metabatch <-
  read_excel("./dada2/metadata.xlsx")

metabatch$IDX <- paste("X", metabatch$sample_name, sep="")

metac <- merge(Phe, metabatch, by="IDX")

table(metac$seq_batch)
```

```
##
## B120 B666
##  113  636
```

```
i <- c("All")





#Hellinger transformation
Microbio2 <- data.frame(t(decostand(t(Microbio), method="hellinger")))
#Maks TSS
Microbio2<-sweep(Microbio2, 2, colSums(Microbio2), FUN="/")
#Dissimilarity
distmatrix <- vegdist(t(Microbio2), method="bray")
#Multi dimensional scaling with capscale
PCoAcsObject<-capscale(distmatrix~1)


##Add eig to plot axes. with cmdscale there are negative values not with capscale
eig <- PCoAcsObject$CA$eig
# Calculate the variation explained by PCoA1, 2, 3 and 4
# and use it to generate axis labels
eig_1_2 <- eig[1:4] / sum(eig) * 100 #Vector with variance explained
#  by the first 4 axes
eig_1 <- paste("PCoA1", round(eig_1_2[1], digits = 2), "% variance")
eig_2 <- paste("PCoA2", round(eig_1_2[2], digits = 2), "% variance")
eig_3 <- paste("PCoA3", round(eig_1_2[3], digits = 2), "% variance")
eig_4 <- paste("PCoA4", round(eig_1_2[4], digits = 2), "% variance")

##Pull out coordinates for plotting from the ca object
#Structuring to add to metac
PCoACA<-PCoAcsObject$CA #The ca object contains the actual ordination results:
#u ((Weighted) orthonormal site scores),
```

```r
#v ((Weighted) orthonormal species scores) all na in mine (unconstrained),
#Xbar (The standardized data matrix after previous stages of analysis),
#and imaginary.u.eig ???.
#Info http://cc.oulu.fi/~jarioksa/softhelp/vegan/html/cca.object.html
PCoA<-as.data.frame(PCoACA$u)
#Change colnames. Now add dis and trans info to names

colnames(PCoA) <- paste("MDS", 1:length(PCoA), "BrayHel", sep="")
#Add row names to df
PCoA$IDX <- row.names(PCoA)
#Merge according to Sample
metac<-merge(metac, PCoA, by="IDX")


metac$gly_stat<-as.factor(metac$gly_stat)
metac$p_gq_smoke<-as.factor(metac$p_gq_smoke)

# #PCoA MDS1 and MDS2 pdf
# pdf(paste("ADDPRO_Microbio_glystat_PCoA", i, ".pdf", sep=""), width=9, height=6)
# print(ggplot(metac) +
#   geom_point(aes(x=MDS1BrayHel, y=MDS2BrayHel, color = gly_stat,
#                  group = gly_stat), size=3) +
#   stat_ellipse(aes(MDS1BrayHel, y=MDS2BrayHel, color = gly_stat,
#                    group = gly_stat)) +
#   scale_color_manual(values=c("1"="#0000FF","2"="#FF0000", "3"="#228B22",
#                               "4"="#FFD700", "5"="#800080")) +
#   ggtitle(paste("PCoA", i, sep=" ")) +
#   labs(colour="gly_stat", x = eig_1, y = eig_2) +
#   theme_bw() +
#   theme(panel.grid.major = element_blank(), panel.grid.minor = element_blank(),
#         axis.title=element_text(size=12), legend.position="bottom"))
# dev.off()
#
# #PCoA MDS1 and MDS2 pdf
# pdf(paste("ADDPRO_Microbio_smoke_PCoA", i, ".pdf", sep=""), width=9, height=6)
# print(ggplot(metac[!is.na(metac$p_gq_smoke),]) + #Removed the samples wNAs smoke
#   geom_point(aes(x=MDS1BrayHel, y=MDS2BrayHel, color = p_gq_smoke,
#                  group = p_gq_smoke), size=3) +
#   stat_ellipse(aes(MDS1BrayHel, y=MDS2BrayHel, color = p_gq_smoke,
#                    group = p_gq_smoke)) +
#   scale_color_manual(values=c("1"="#0000FF","2"="#FF0000", "3"="#228B22")) +
#   ggtitle(paste("PCoA", i, sep=" ")) +
#   labs(colour="p_gq_smoke", x = eig_1, y = eig_2) +
#   theme_bw() +
#   theme(panel.grid.major = element_blank(), panel.grid.minor = element_blank(),
#         axis.title=element_text(size=12), legend.position="bottom"))
# dev.off()
#
# #coloring<-"p_gq_smoke" #Remember NAs are not plottet
# coloring<-"gly_stat"
#
# #PCoA MDS1 and MDS2
# print(ggplot(metac) +
```

```r
#   geom_point(aes_string(x="MDS1BrayHel", y="MDS2BrayHel", color = coloring,
#                  group = coloring), size=3) +
#   stat_ellipse(aes_string("MDS1BrayHel", y="MDS2BrayHel", color = coloring,
#                  group = coloring)) +
#   scale_color_manual(values=c("1"="#0000FF","2"="#FF0000", "3"="#228B22",
#                            "4"="#FFD700", "5"="#800080")) +
#   ggtitle(paste("PCoA", i, sep=" ")) +
#   labs(colour="gly_stat", x = eig_1, y = eig_2) +
#   theme_bw() +
#   theme(panel.grid.major = element_blank(), panel.grid.minor = element_blank(),
#         axis.title=element_text(size=12), legend.position="bottom"))
#
# #PCoA MDS1 and MDS3
# print(ggplot(metac) +
#   geom_point(aes_string(x="MDS1BrayHel", y="MDS3BrayHel", color = coloring,
#                  group = coloring), size=3) +
#   stat_ellipse(aes_string("MDS1BrayHel", y="MDS3BrayHel", color = coloring,
#                  group = coloring)) +
#   scale_color_manual(values=c("1"="#0000FF","2"="#FF0000", "3"="#228B22",
#                            "4"="#FFD700", "5"="#800080")) +
#   ggtitle(paste("PCoA", i, sep=" ")) +
#   labs(colour="gly_stat", x = eig_1, y = eig_3) +
#   theme_bw() +
#   theme(panel.grid.major = element_blank(), panel.grid.minor = element_blank(),
#         axis.title=element_text(size=12), legend.position="bottom"))
#
# #PCoA MDS2 and MDS3
# print(ggplot(metac) +
#   geom_point(aes_string(x="MDS2BrayHel", y="MDS3BrayHel", color = coloring,
#                  group = coloring), size=3) +
#   stat_ellipse(aes_string("MDS2BrayHel", y="MDS3BrayHel", color = coloring,
#                  group = coloring)) +
#   scale_color_manual(values=c("1"="#0000FF","2"="#FF0000", "3"="#228B22",
#                            "4"="#FFD700", "5"="#800080")) +
#   ggtitle(paste("PCoA", i, sep=" ")) +
#   labs(colour="gly_stat", x = eig_2, y = eig_3) +
#   theme_bw() +
#   theme(panel.grid.major = element_blank(), panel.grid.minor = element_blank(),
#         axis.title=element_text(size=12), legend.position="bottom"))



#coloring<-"p_gq_smoke" #Remember NAs are not plottet
#coloring<-"gly_stat"
coloring<-"seq_batch"

# PC1 loadings
#Line specifier
metac$lines <-
  ifelse(metac$IDX=="X10329" | metac$IDX=="X10329re", "re10329",
         ifelse(metac$IDX=="X10585" | metac$IDX=="X10585re", "re10585",
                ifelse(metac$IDX=="X10817" | metac$IDX=="X10817re", "re10817",
                       NA)))
```

```r
#PC1 line coordinates
metac$MDS1lines <-
  ifelse(metac$IDX=="X10329" | metac$IDX=="X10585" | metac$IDX=="X10817" |
           metac$IDX=="X10329re" | metac$IDX=="X10585re" | metac$IDX=="X10817re",
         metac$MDS1BrayHel, NA)

#PC2 line coordinates
metac$MDS2lines <-
  ifelse(metac$IDX=="X10329" | metac$IDX=="X10585" | metac$IDX=="X10817" |
           metac$IDX=="X10329re" | metac$IDX=="X10585re" | metac$IDX=="X10817re",
         metac$MDS2BrayHel, NA)

##
#PCoA MDS1 and MDS2
print(ggplot(metac) +
  geom_point(aes_string(x="MDS1BrayHel", y="MDS2BrayHel", color = coloring,
                 group = coloring), size=2) +
  geom_line(aes_string(x="MDS1lines", y="MDS2lines", group="lines", na.rm=TRUE)) +
  stat_ellipse(aes_string("MDS1BrayHel", y="MDS2BrayHel", color = coloring,
                   group = coloring)) +
  scale_color_manual(values=c("B120"="#0000FF","B666"="#FF0000")) +
  ggtitle(paste("PCoA", i, sep=" ")) +
  labs(colour=coloring, x = eig_1, y = eig_2) +
  theme_bw() +
  theme(panel.grid.major = element_blank(), panel.grid.minor = element_blank(),
        axis.title=element_text(size=12), legend.position="bottom"))
```

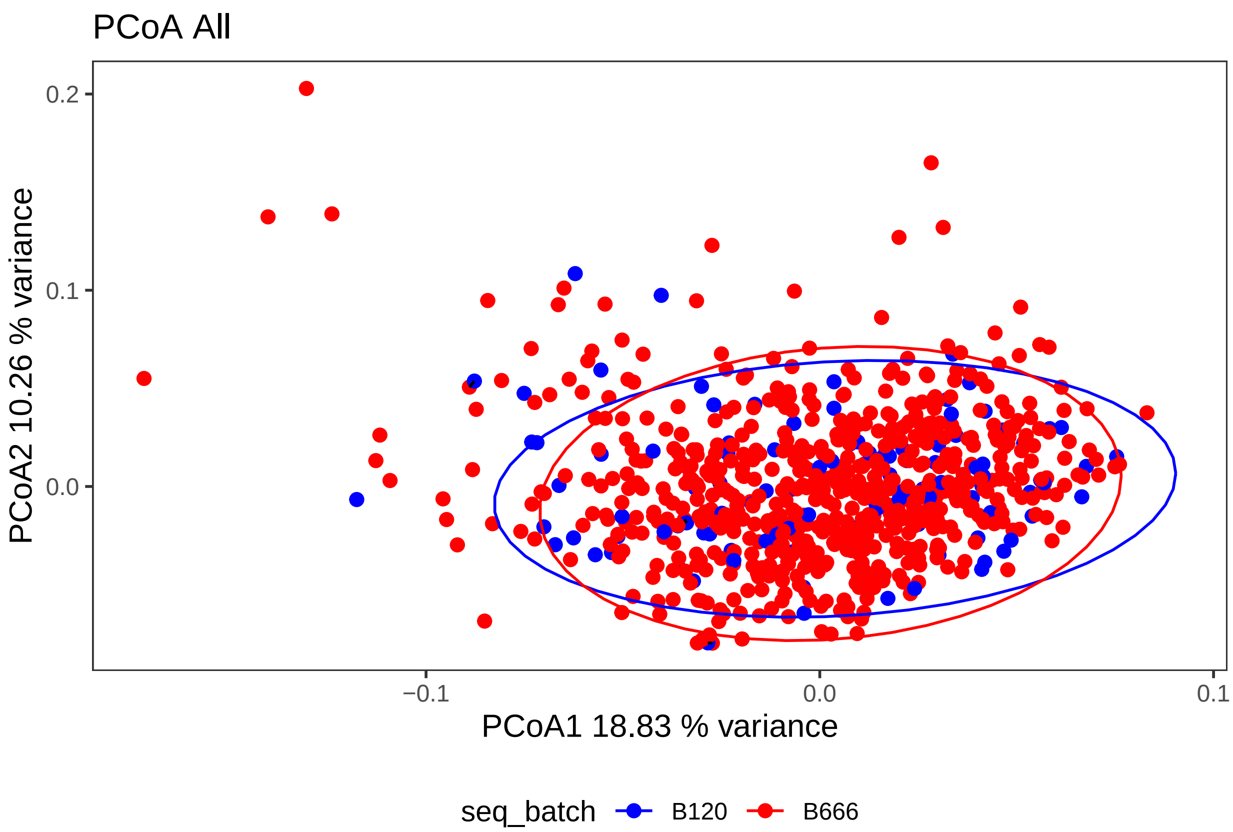


```
#Changed size
print(ggplot(metac) +
  geom_point(aes_string(x="MDS1BrayHel", y="MDS2BrayHel", color = coloring,
               group = coloring), size=1) +
  geom_line(aes_string(x="MDS1lines", y="MDS2lines", group="lines", na.rm=TRUE), size=2) +
  stat_ellipse(aes_string("MDS1BrayHel", y="MDS2BrayHel", color = coloring,
                group = coloring)) +
  scale_color_manual(values=c("B120"="#0000FF","B666"="#FF0000")) +
  ggtitle(paste("PCoA", i, sep=" ")) +
  labs(colour=coloring, x = eig_1, y = eig_2) +
  theme_bw() +
  theme(panel.grid.major = element_blank(), panel.grid.minor = element_blank(),
        axis.title=element_text(size=12), legend.position="bottom"))
```

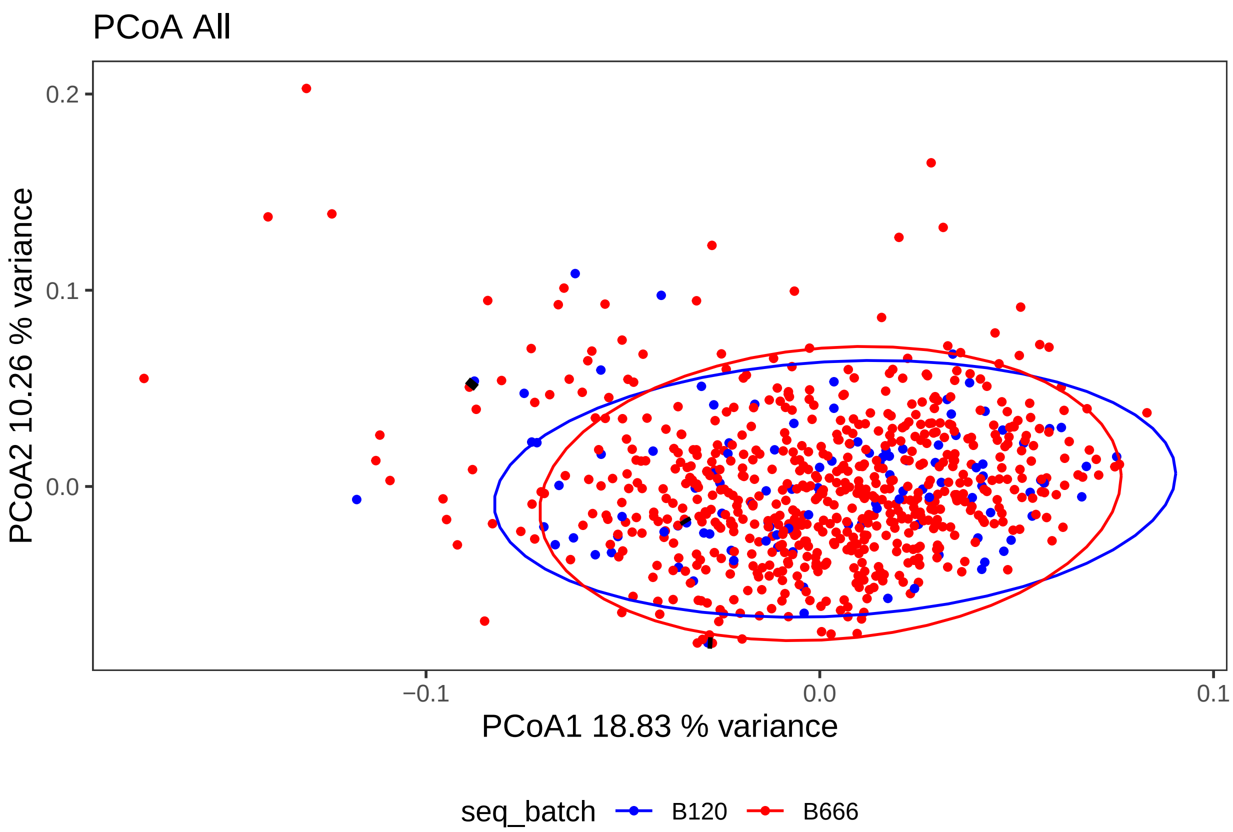


```
#PCoA MDS1 and MDS3
print(ggplot(metac) +
  geom_point(aes_string(x="MDS1BrayHel", y="MDS3BrayHel", color = coloring,
                group = coloring), size=2) +
  geom_line(aes_string(x="MDS1lines", y="MDS2lines", group="lines", na.rm=TRUE), size=2) +
  stat_ellipse(aes_string("MDS1BrayHel", y="MDS3BrayHel", color = coloring,
                 group = coloring)) +
  scale_color_manual(values=c("B120"="#0000FF","B666"="#FF0000")) +
  ggtitle(paste("PCoA", i, sep=" ")) +
  labs(colour=coloring, x = eig_1, y = eig_2) +
  theme_bw() +
  theme(panel.grid.major = element_blank(), panel.grid.minor = element_blank(),
        axis.title=element_text(size=12), legend.position="bottom"))
```

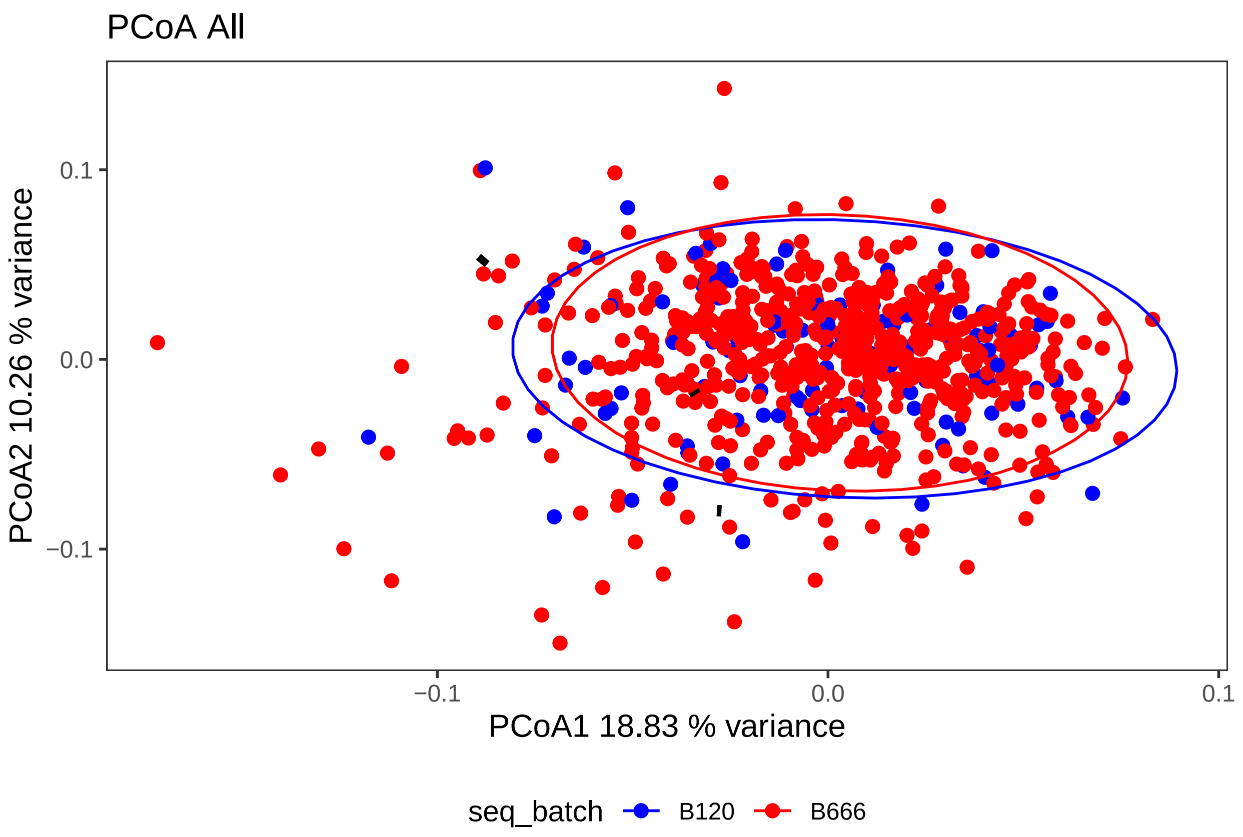


```
#PCoA MDS2 and MDS3
print(ggplot(metac) +
  geom_point(aes_string(x="MDS2BrayHel", y="MDS3BrayHel", color = coloring,
               group = coloring), size=2) +
  geom_line(aes_string(x="MDS1lines", y="MDS2lines", group="lines", na.rm=TRUE), size=2) +
  stat_ellipse(aes_string("MDS2BrayHel", y="MDS3BrayHel", color = coloring,
                group = coloring)) +
  scale_color_manual(values=c("B120"="#0000FF","B666"="#FF0000")) +
  ggtitle(paste("PCoA", i, sep=" ")) +
  labs(colour=coloring, x = eig_1, y = eig_2) +
  theme_bw() +
  theme(panel.grid.major = element_blank(), panel.grid.minor = element_blank(),
        axis.title=element_text(size=12), legend.position="bottom"))
```

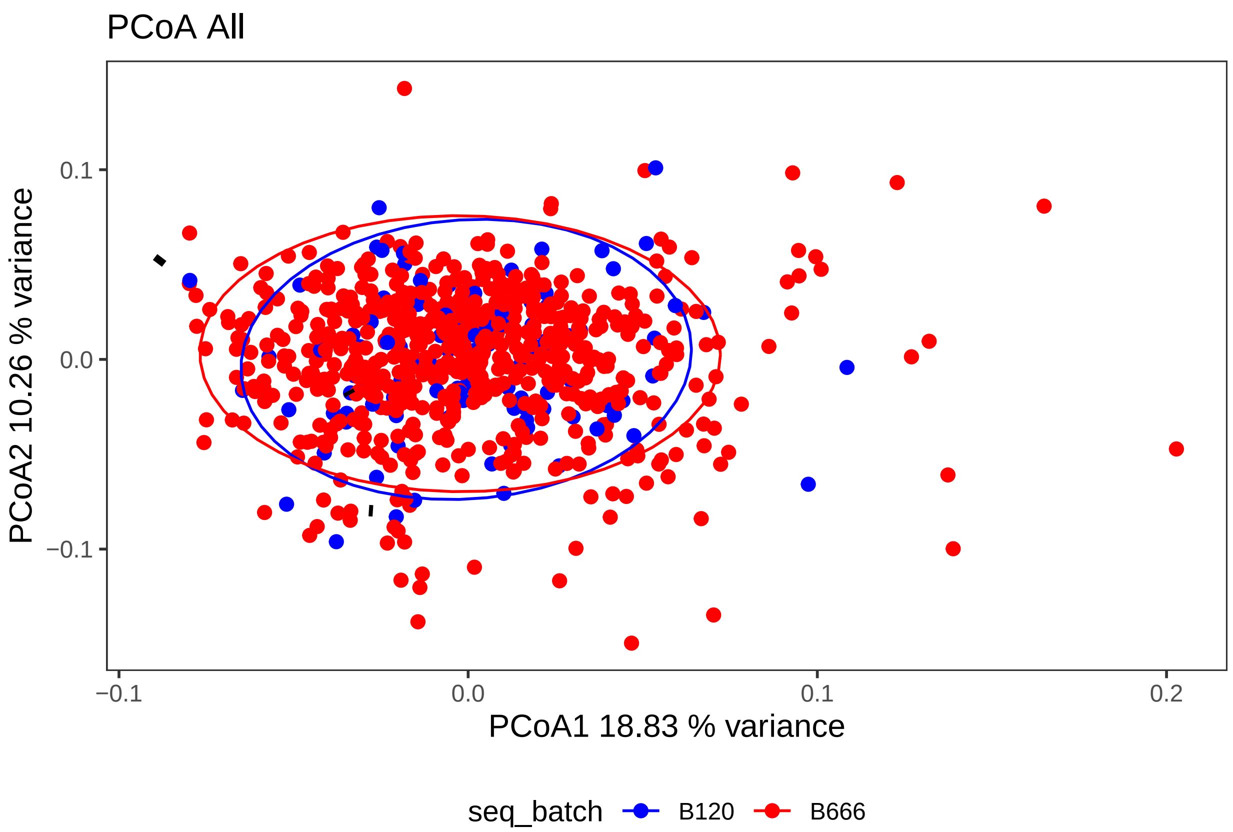


```r
##PERMANOVA
#adonis can handle both continous and factor predictors
set.seed(1)
adonisObject<-adonis2(distmatrix ~ seq_batch, metac, by="terms",
                      perm=999) #, perm=999 can increase to get exact p-values
adonisObject #If significant then difference between groups
```

```
## Permutation test for adonis under reduced model
## Terms added sequentially (first to last)
## Permutation: free
## Number of permutations: 999
##
## adonis2(formula = distmatrix ~ seq_batch, data = metac, permutations = 999, by = "terms")
##            Df SumOfSqs      R2      F Pr(>F)
## seq_batch   1   0.0345 0.00127 0.9512  0.451
## Residual  747  27.0633 0.99873
## Total     748  27.0977 1.00000
```

```r
#dist samples on both runs
subset_dist2 <- function (d, keep) {return(as.dist(as.matrix(d)[keep, keep]))}
both<-subset_dist2(distmatrix, c("X10329", "X10585", "X10817", "X10329re", "X10585re", "X10817re"))
both
```

```
##                X10329     X10585     X10817   X10329re   X10585re
## X10585   0.23991160
```

```
## X10817   0.30355291 0.39563704
## X10329re 0.01043064 0.23645810 0.30441364
## X10585re 0.23880465 0.01067202 0.39212978 0.23375822
## X10817re 0.30311778 0.39491527 0.01435947 0.30397851 0.39140800
```

```
#PCoA MDS1 and MDS2
pdf(paste("ADDPRO_Microbio_seqbatch_PCoA", i, ".pdf", sep=""), width=9, height=6)
print(ggplot(metac) +
  geom_point(aes_string(x="MDS1BrayHel", y="MDS2BrayHel", color = coloring,
               group = coloring), size=1) +
  geom_line(aes_string(x="MDS1lines", y="MDS2lines", group="lines", na.rm=TRUE), size=2) +
  stat_ellipse(aes_string("MDS1BrayHel", y="MDS2BrayHel", color = coloring,
                group = coloring)) +
  scale_color_manual(values=c("B120"="#0000FF","B666"="#FF0000")) +
  ggtitle(paste("PCoA sequencing batch - PERMANOVA pvalue =", adonisObject$`Pr(>F)`, sep=" ")) +
  labs(colour=coloring, x = eig_1, y = eig_2) +
  theme_bw() +
  theme(panel.grid.major = element_blank(), panel.grid.minor = element_blank(),
      axis.title=element_text(size=12), legend.position="bottom")) +
  geom_text(x=-0.034, y=-0.021, label="BC=0.010", size=2.5) + #X10329
  geom_text(x=-0.028, y=-0.084, label="BC=0.011", size=2.5) + #X10585
  geom_text(x=-0.089, y=0.056, label="BC=0.014", size=2.5) #X10817
dev.off()
```

```
## pdf
##   2
```

```
#test<-metac[,c("IDX", "MDS1lines")] #X10817=-0.089, X10329=-0.034, X10585=-0.028
```

Remove re samples

```
rm(list=setdiff(ls(), c("FeaturePic2", "Pic2", "Phe", "Microbio", "FeatureMic")))

Microbio <- dplyr::select(Microbio, -one_of(c("X10329re", "X10585re", "X10817re")))
Phe<-subset(Phe, IDX %in% colnames(Microbio))
```

Define phenotypes according to categorical categories

The UA category is just to check if a nonsensical value is in the table U+2265

```
Phe$sex_cat<-factor(ifelse(Phe$sex==1, "Male",
                ifelse(Phe$sex==0, "Female", "NA")))
table(Phe$sex_cat, useNA="always")
```

```
##
## Female   Male   <NA>
##    339    407      0
```

```
##Don't have encoded p_gv_sex 0=women, 1=man, but in Phe have "sex", make sanity check.
#boxplot(height ~ sex_cat, data = Phe, frame = FALSE)


#Overweight
##BMI - bmi: < 18.5, 18.5-24.9, 25-29.9, =>30
Phe$BMI_cat<-factor(ifelse(Phe$bmi < 18.5, "Underweight",
                           ifelse(18.5 <= Phe$bmi & Phe$bmi < 25, "Healthyweight",
                                  ifelse(25 <= Phe$bmi & Phe$bmi < 30,  "Overweight",
                                         ifelse(Phe$bmi >= 30, "Obese", "UA")))))
Phe$BMI_cat<-factor(Phe$BMI_cat, levels=c("Underweight", "Healthyweight", "Overweight", "Obese", "UA"))
table(Phe$BMI_cat, useNA="always")
```

```
##
##   Underweight Healthyweight    Overweight         Obese            UA
##             3           251           337           155             0
##          <NA>
##             0
```

```
##Fat percentage - Fat_pc: male =<25% og >25%,  female =<33% og >33%
Phe$Fat_cat<-as.character(ifelse(Phe$fat_pc <= 25 & Phe$sex == 1 | Phe$fat_pc <= 33 & Phe$sex == 0 , "Lo
                          ifelse(Phe$fat_pc > 25 & Phe$sex == 1 | Phe$fat_pc > 33 & Phe$sex == 0 ,
                          "UA")))
table(Phe$Fat_cat, useNA="always")
```

```
##
## High  Low <NA>
##  485  254    7
```

```
##Waist measurement - Waist_av: females <80cm, 80-88cm, >88cm, for males <94cm, 94-102cm, >102cm
Phe$Waist_cat<-as.character(ifelse(Phe$waist_av < 80 & Phe$sex == 0 | Phe$waist_av < 94 & Phe$sex == 1
                                   ifelse(Phe$waist_av <= 88 & Phe$waist_av >= 80 & Phe$sex == 0 | Phe$w
                                          ifelse(Phe$waist_av > 88 & Phe$sex == 0 | Phe$waist_av > 102 &
                                                 "UA"))))
Phe$Waist_cat<-factor(Phe$Waist_cat, levels=c("Low", "Medium", "High"))
table(Phe$Waist_cat, useNA="always")
```

```
##
##    Low Medium   High   <NA>
##    197    220    329      0
```

```
##Waist to hip ratio - waist_av/hip_av: females <0.85cm og =>0.85cm, males <0.90cm og =>0.90cm
Phe$whratio<-Phe$waist_av/Phe$hip_av
#Phe$whratio
Phe$whratio_cat<-as.character(ifelse(Phe$whratio <= 0.85 & Phe$sex == 0 | Phe$whratio <= 0.90 & Phe$sex
                              ifelse(Phe$whratio > 0.85 & Phe$sex == 0 | Phe$whratio > 0.90 & Phe$sex ==
                              "UA")))
table(Phe$whratio_cat, useNA="always")
```

```
##
## High  Low <NA>
##  592  154    0
```

```r
#Alcohol intake (should it be the same for males and females)
##Weekly alcohol intake - Sum (p_gq_beer_week+ p_gq_wine_week+ p_gq_liquour_week)
  #Divided into 0 units/week, 1-10 units/week, >10 units/week
Phe$Alcprweek <- Phe$p_gq_beer_week+Phe$p_gq_wine_week+Phe$p_gq_liquour_week
sum(is.na(Phe$Alcprweek))
```

```
## [1] 103
```

```r
#Phe$Alcprweek
Phe$Alcprweek_cat <- as.character(ifelse(Phe$Alcprweek == 0, "Abstinence",
                              ifelse(0 < Phe$Alcprweek & Phe$Alcprweek <= 10, "Moderate",
                                     ifelse(Phe$Alcprweek > 10, "High", "UA"))))
Phe$Alcprweek_cat<-factor(Phe$Alcprweek_cat, levels=c("Abstinence", "Moderate", "High"))
table(Phe$Alcprweek_cat, useNA="always")
```

```
##
## Abstinence   Moderate       High       <NA>
##         79        302        262        103
```

```r
#sex_cat
##Smoking - p_gq_smoke: grouping as is 1, 2, 3
#Some chunks uses as.factor(p_gq_smoke)
table(Phe$p_gq_smoke, useNA="always")
```

```
##
##    1    2    3 <NA>
##  129  351  261    5
```

```r
Phe$Smoking<-as.character(ifelse(Phe$p_gq_smoke==1, "Smoker",
                              ifelse(Phe$p_gq_smoke==2, "Exsmoker",
                                     ifelse(Phe$p_gq_smoke==3,
                                            "Nonsmoker", "UA"))))
table(Phe$Smoking, useNA="always")
```

```
##
##  Exsmoker Nonsmoker    Smoker      <NA>
##       351       261       129         5
```

```r
#Cardiovascular
##Blodtryk - sbp_av/dbp_av:
#Lav = sbp <120 og dbp <80
#Normal = sbp 120-139,9 og dpb 80-89,9
#Mild = sbp =>140-159,9 og dbp =>90-99,9
#Moderat-alvorlig = sbp =>160 og dbp =>100
sum(is.na(Phe$sbp_av))
```

```
## [1] 2
```

```r
sum(is.na(Phe$dbp_av))
```

```
## [1] 2
```

```r
# Phe$bp_cat<-as.character(ifelse(Phe$sbp_av < 120  & Phe$dbp_av < 80, "Low",
#                                 ifelse(120 <= Phe$sbp_av & Phe$sbp_av < 140 & 80 <= Phe$dbp_av & Phe$db
#                                        ifelse(140 <= Phe$sbp_av & Phe$sbp_av < 160 & 90 <= Phe$dbp_av &
#                                               ifelse(Phe$sbp_av >= 160 & Phe$dbp_av >= 100, "Moderate_s
##New american heart association, also consider have 4 higher than 180
#Normal = sbp <120 og dbp <80
#Mild_elevated = sbp 120-129 og dbp <80
#Moderate_elevated = sbp 130-139 og dbp 80-89
#Severely_elevated = sbp =>140 og dbp =>90
Phe$bp_cat<-as.character(ifelse(Phe$sbp_av < 120  & Phe$dbp_av < 80, "Normal",
                                ifelse(120 <= Phe$sbp_av & Phe$sbp_av < 130 & Phe$dbp_av < 80, "Mild_eleva
                                       ifelse(130 <= Phe$sbp_av & Phe$sbp_av < 140 | 80 <= Phe$dbp_av & Pl
                                              ifelse(Phe$sbp_av >= 140 | Phe$dbp_av >= 90, "Severely_eleva
Phe$bp_cat<-factor(Phe$bp_cat, levels=c("Normal", "Mild_elevated", "Moderate_elevated", "Severely_elevat
table(Phe$bp_cat, useNA="always")
```

```
##
##            Normal     Mild_elevated Moderate_elevated Severely_elevated
##               157                93               356               138
##              <NA>
##                 2
```

```r
#sbp alone
Phe$sbp_cat<-as.character(ifelse(Phe$sbp_av < 120, "Low",
                                ifelse(120 <= Phe$sbp_av & Phe$sbp_av < 140, "Normal",
                                       ifelse(140 <= Phe$sbp_av & Phe$sbp_av < 160,  "Mild_elevated",
                                              ifelse(Phe$sbp_av >= 160, "Moderate_severe", "UA")))))
Phe$sbp_cat<-factor(Phe$sbp_cat, levels=c("Low", "Normal", "Mild_elevated", "Moderate_severe"))
table(Phe$sbp_cat, useNA="always")
```

```
##
##             Low          Normal   Mild_elevated Moderate_severe            <NA>
##             175             347             179              43               2
```

```r
#dbp alone
Phe$dbp_cat<-as.character(ifelse(Phe$dbp_av < 80, "Low",
                                ifelse(80 <= Phe$dbp_av & Phe$dbp_av < 90, "Normal",
                                       ifelse(90 <= Phe$dbp_av & Phe$dbp_av < 100,  "Mild_elevated",
                                              ifelse(Phe$dbp_av >= 100, "Moderate_severe", "UA")))))
Phe$dbp_cat<-factor(Phe$dbp_cat, levels=c("Low", "Normal", "Mild_elevated", "Moderate_severe"))
table(Phe$dbp_cat, useNA="always")
```

```
##
##             Low          Normal   Mild_elevated Moderate_severe            <NA>
##             342             267             103              32               2
```

```
table(Phe$sbp_cat, Phe$dbp_cat, useNA="always")
```

```
##
##                   Low Normal Mild_elevated Moderate_severe <NA>
##   Low             157     18             0               0    0
##   Normal          154    160            32               1    0
##   Mild_elevated    29     80            56              14    0
##   Moderate_severe   2      9            15              17    0
##   <NA>              0      0             0               0    2
```

```
##Puls/heart rate - hr_av: females <70, 70-90, >90 and males <60, 60-80, >80
Phe$hr_cat<-as.character(ifelse(Phe$hr_av < 70 & Phe$sex == 0 | Phe$hr_av < 60 & Phe$sex == 1 , "Low",
                                   ifelse(Phe$hr_av <= 90 & Phe$hr_av >= 70 & Phe$sex == 0 | Phe$hr_av 
                                          ifelse(Phe$hr_av > 90 & Phe$sex == 0 | Phe$hr_av > 80 & Phe$s
                                                 "UA"))))
Phe$hr_cat<-factor(Phe$hr_cat, levels=c("Low", "Medium", "High"))
table(Phe$hr_cat, useNA="always")
```

```
##
##    Low Medium   High   <NA>
##    345    344     55      2
```

```
##Pulspres - pp_av:  =<40 og <40
Phe$pp_cat<-as.character(ifelse(Phe$pp_av <= 40, "Low",
                              ifelse(Phe$pp_av > 40, "High",
                              "UA")))
table(Phe$pp_cat, useNA="always")
```

```
##
## High  Low <NA>
##  605  139    2
```

```
##Triglycerider - p_lab_trig: <2 og =>2
Phe$trig_cat<-as.character(ifelse(Phe$p_lab_trig < 2, "Low",
                              ifelse(Phe$p_lab_trig >= 2, "High",
                              "UA")))
table(Phe$trig_cat, useNA="always")
```

```
##
## High  Low <NA>
##   72  674    0
```

```
##HDL cholesterol - p_lab_hdlc: =<1 og >1
Phe$hdlc_cat<-as.character(ifelse(Phe$p_lab_hdlc <= 1, "Low",
                              ifelse(Phe$p_lab_hdlc > 1, "High",
                              "UA")))
table(Phe$hdlc_cat, useNA="always")
```

```
##
## High  Low <NA>
##  697   49    0
```

```r
##LDL cholesterol - p_lab_ldl: <3 og =>3
Phe$ldlc_cat<-as.character(ifelse(Phe$p_lab_ldl < 3, "Low",
                              ifelse(Phe$p_lab_ldl >= 3, "High",
                              "UA")))
table(Phe$ldlc_cat, useNA="always")
```

```
##
## High  Low <NA>
##  469  275    2
```

```r
##Total cholesterol - p_lab_chol: <5 og =>5
Phe$chol_cat<-as.character(ifelse(Phe$p_lab_chol < 5, "Low",
                              ifelse(Phe$p_lab_chol >= 5, "High",
                              "UA")))
table(Phe$chol_cat, useNA="always")
```

```
##
## High  Low <NA>
##  495  251    0
```

```r
#table(Phe$trig_cat, Phe$hdlc_cat, Phe$ldlc_cat, Phe$chol_cat)
##Fysisk aktivitet - sum(p_rpaq_MODERATE + p_rpaq_VIGOR): <0,5, 0,5-1 >1 #Do we want to weight it?
Phe$act<-Phe$p_rpaq_MODERATE + Phe$p_rpaq_VIGOR
Phe$act_cat<-as.character(ifelse(Phe$act < 0.5, "Low",
                              ifelse(0.5 <= Phe$act & Phe$act <= 1, "Medium",
                                     ifelse(Phe$act > 1, "High", "UA"))))
Phe$act_cat<-factor(Phe$act_cat, levels=c("Low", "Medium", "High"))
table(Phe$act_cat, useNA="always")
```

```
##
##    Low Medium   High   <NA>
##    100    149    453     44
```

```r
#Type 2 diabetes
## Glycemia status - gly_stat: grouping as is
#Some chunks uses as.factor(gly_stat)
#See Pre-processing - Subset Pheno
table(Phe$Glycaemia_Status, useNA="always")
```

```
##
##     Gr1ngt    Gr2ilFG    Gr3ilGT Gr4IFG_IGT     Gr5SDM        KDM    Unclass
##        421        112         58         63         68         21          3
##       <NA>
##          0
```

```r
Phe$Risk<-factor(Phe$Risk, levels=c("Low", "High", "KDM", "unclass"))
table(Phe$Risk, useNA="always")
```

```
##
##     Low    High     KDM unclass    <NA>
##     421     301      21       0       3
```

```r
## HbA1c: <6, 6-6,4,=>6,5
Phe$hba1c_cat<-as.character(ifelse(Phe$p_lab_hba1c < 6, "Low",
                              ifelse(6 <= Phe$p_lab_hba1c & Phe$p_lab_hba1c < 6.5, "Medium",
                                     ifelse(Phe$p_lab_hba1c >= 6.5, "High", "UA"))))
Phe$hba1c_cat<-factor(Phe$hba1c_cat, levels=c("Low", "Medium", "High"))
table(Phe$hba1c_cat, useNA="always")
```

```
##
##    Low Medium   High   <NA>
##    600    119     26      1
```

```r
kable(data.frame(sex=sum(is.na(Phe$sex_cat)),
                 BMI=sum(is.na(Phe$BMI_cat)),
                 Fat=sum(is.na(Phe$Fat_cat)),
                 Waist=sum(is.na(Phe$Waist_cat)),
                 whratio=sum(is.na(Phe$whratio_cat)),
                 Alc=sum(is.na(Phe$Alcprweek)),
                 Smoke=sum(is.na(Phe$Smoking)),
                 bp=sum(is.na(Phe$bp_cat)),
                 sbp=sum(is.na(Phe$sbp_cat)),
                 dbp=sum(is.na(Phe$dbp_cat)),
                 heartrate=sum(is.na(Phe$hr_cat)),
                 pulsepres=sum(is.na(Phe$pp_cat)),
                 trig=sum(is.na(Phe$trig_cat)),
                 hdlc=sum(is.na(Phe$hdlc_cat)),
                 ldlc=sum(is.na(Phe$ldlc_cat)),
                 chol=sum(is.na(Phe$chol_cat)),
                 act=sum(is.na(Phe$act_cat)),
                 Gly=sum(is.na(Phe$Glycaemia_Status)),
                 Risk=sum(is.na(Phe$Risk)),
                 hba1c=sum(is.na(Phe$hba1c_cat))))
```

sex	BMI	Fat	Waist	whratio	Alc	Smoke	bp	sbp	dbp	heartrate	pulsepres	trig	hdlc	ldlc	chol	act	Gly	Risk	hba1c
0	0	7	0	0	103	5	2	2	2	2	2	0	0	2	0	44	0	3	1

Analyses

Phenotype overview

```r
rm(list=setdiff(ls(), c("FeaturePic2", "Pic2", "Phe", "Microbio", "FeatureMic")))

#Scatterplots samlet
pairs(Phe[,c(103,107:109)]) #Be aware of NA
```

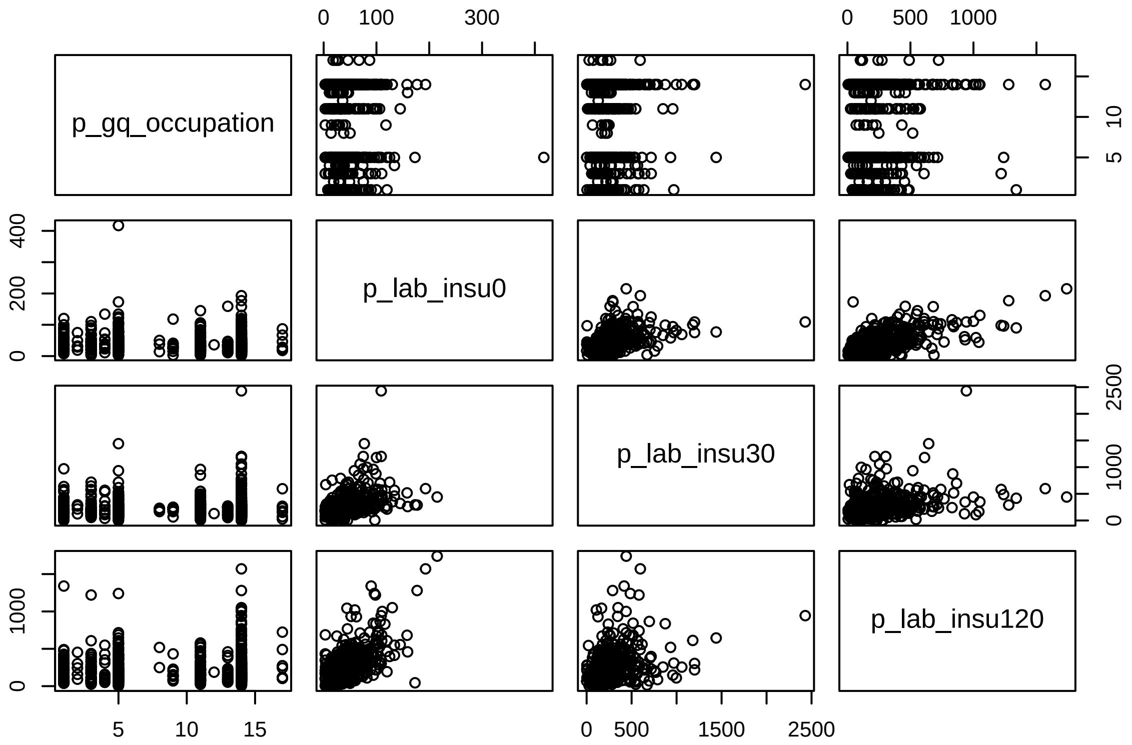


```r
Phe$gly_stat<-as.factor(Phe$gly_stat)
pairs(Phe[,107:109],
      pch = 21,
      bg=c("1"="#0000FF","2"="#FF0000", "3"="#228B22", "4"="#FFD700", "5"="#800080")
      [unclass(Phe$gly_stat)])
```

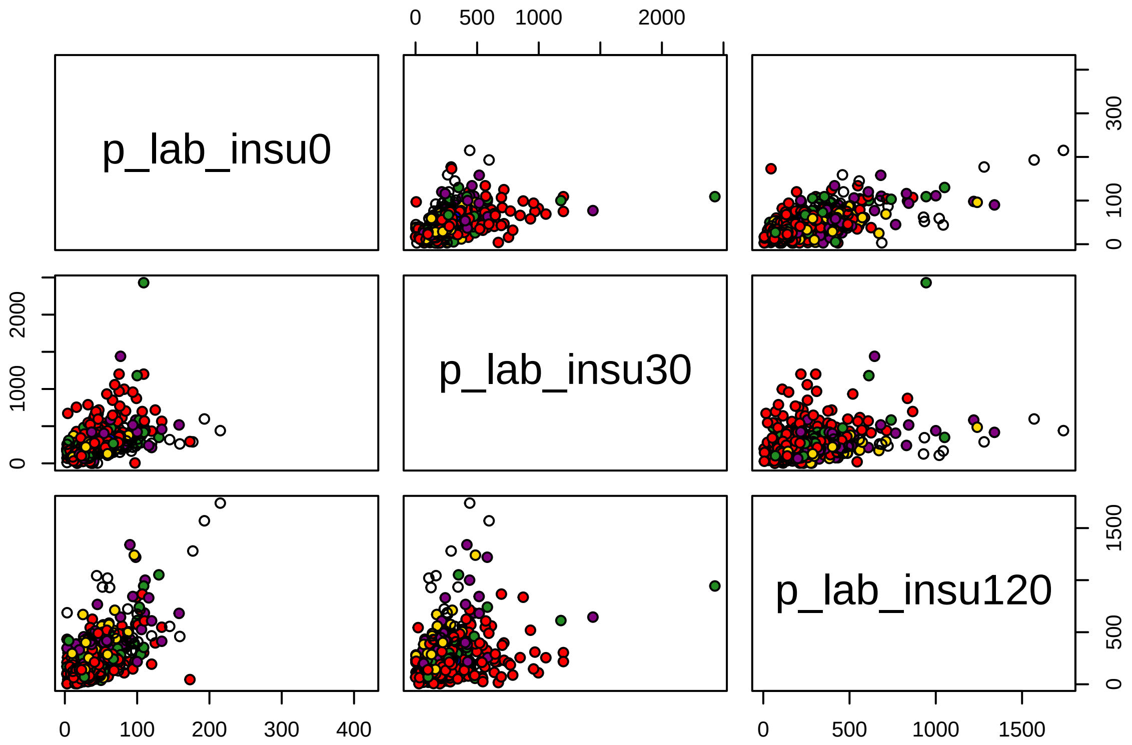


```
#Scatterplots enkeltvis
for (i in c("p_lab_insu0", "p_lab_insu30", "p_lab_insu120")) {
  for (j in c("p_lab_insu0", "p_lab_insu30", "p_lab_insu120")) {
    print(ggplot(Phe, aes_string(x=i, y=j, color="gly_stat")) +
            geom_point() +
            scale_color_manual(values=c("1"="#0000FF","2"="#FF0000", "3"="#228B22",
                                        "4"="#FFD700", "5"="#800080"))+
            theme_bw())
  }
}
```

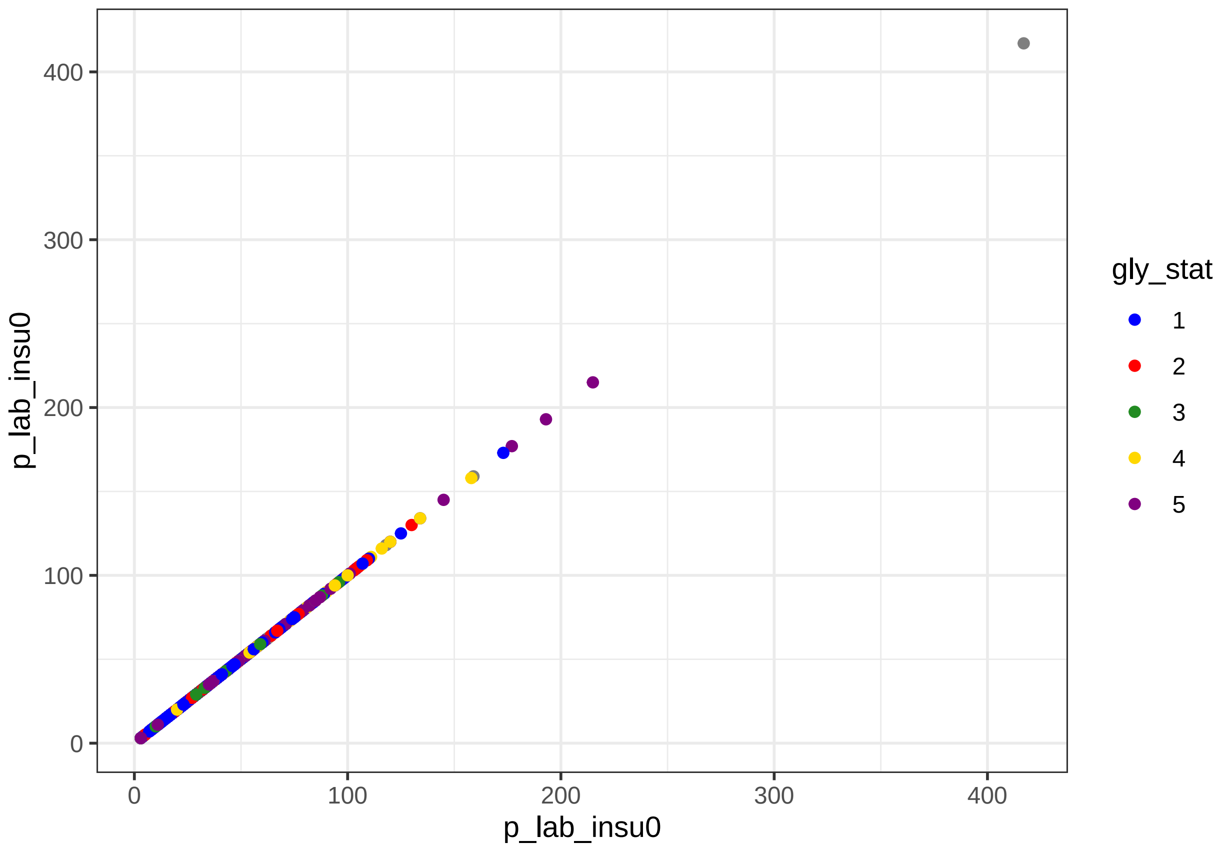

gly_stat
1
2
3
4
5
p_lab_insu0
p_lab_insu0
0
100
200
300
400

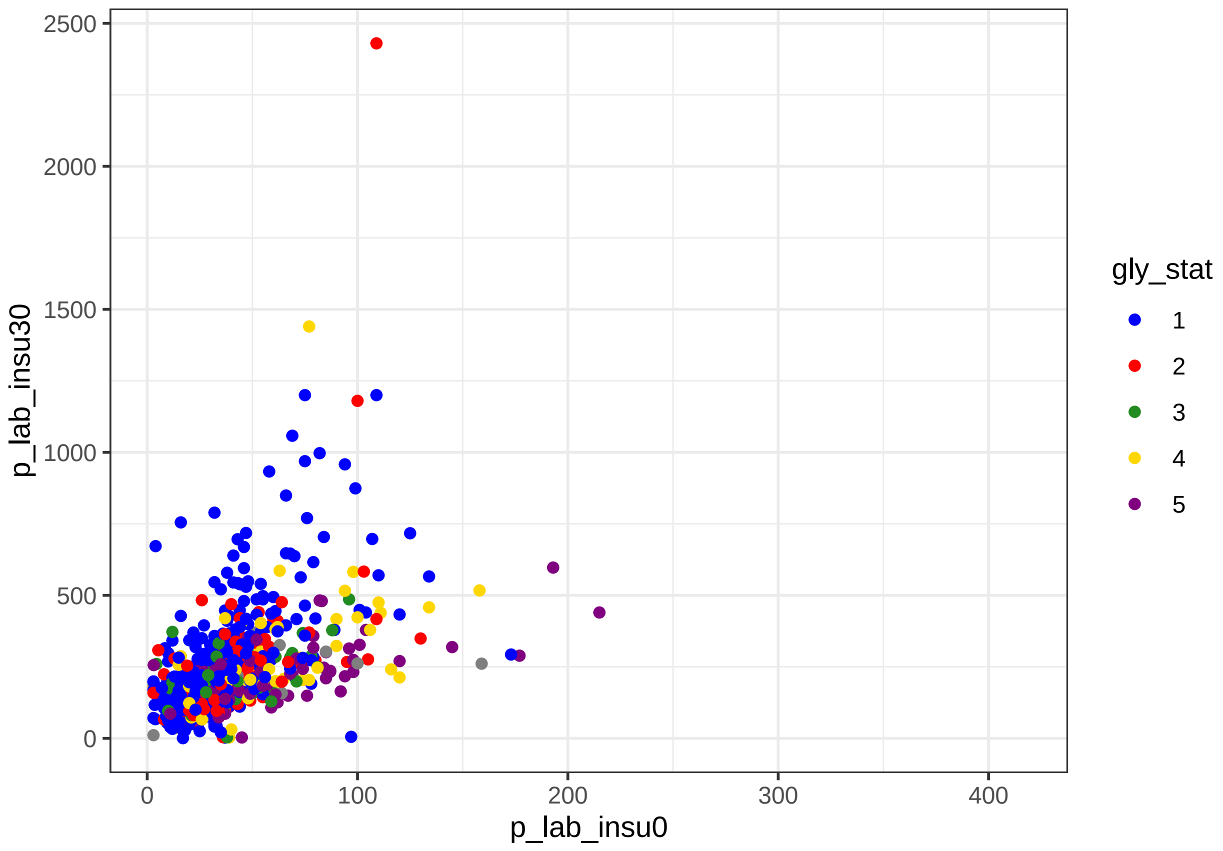

2500
2000
1500
1000
500
0
p_lab_insu30
0
100
200
300
400
p_lab_insu0
gly_stat
1
2
3
4
5

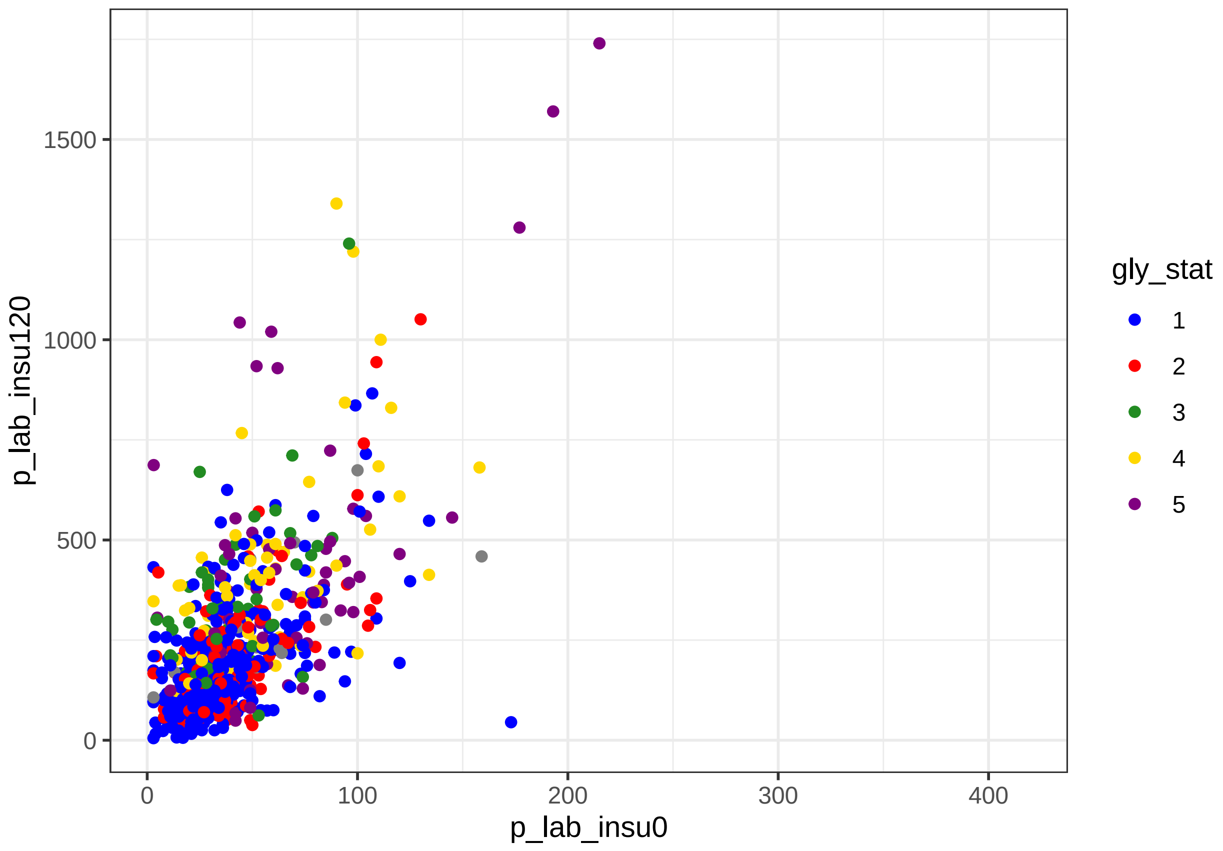

p_lab_insu120
p_lab_insu0
1500
1000
500
0
0
100
200
300
400
gly_stat
1
2
3
4
5

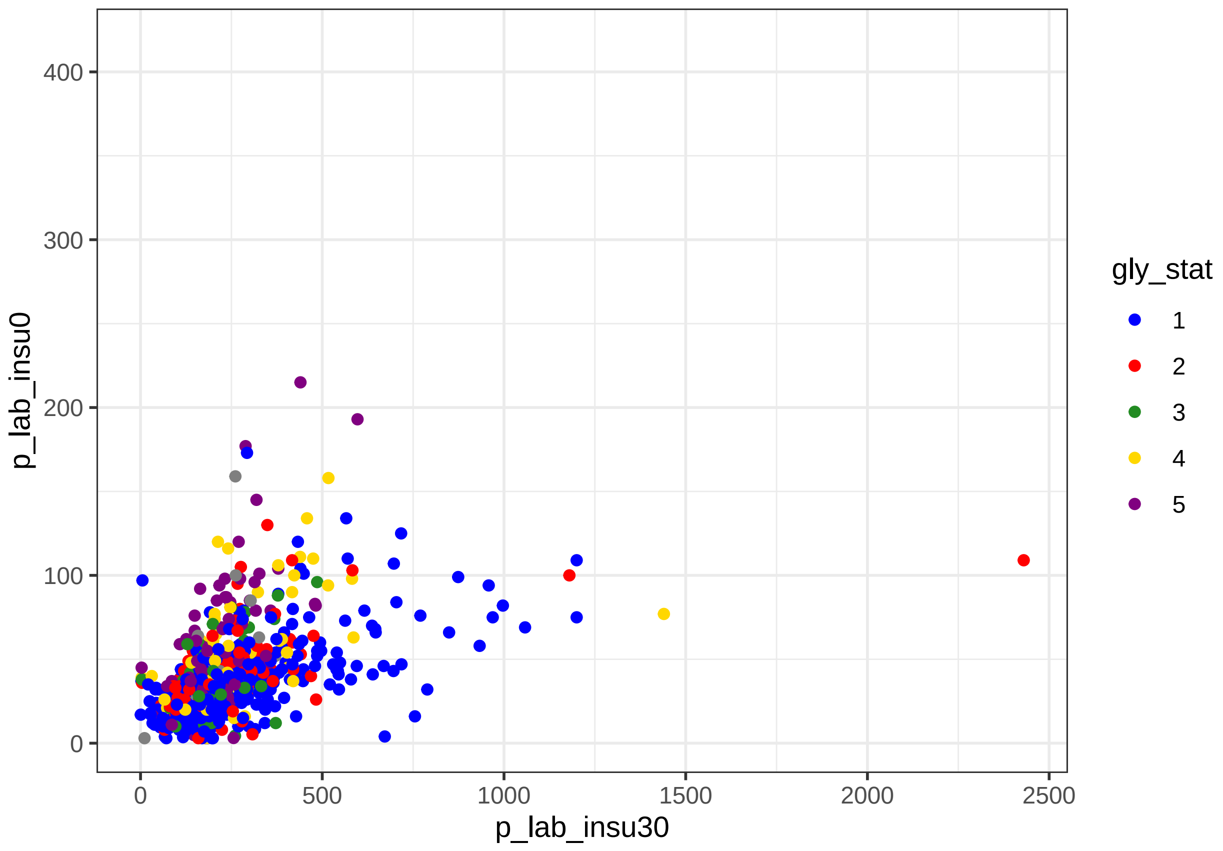

400
300
200
100
0
p_lab_insu0
0
500
1000
1500
2000
2500
p_lab_insu30
gly_stat
1
2
3
4
5

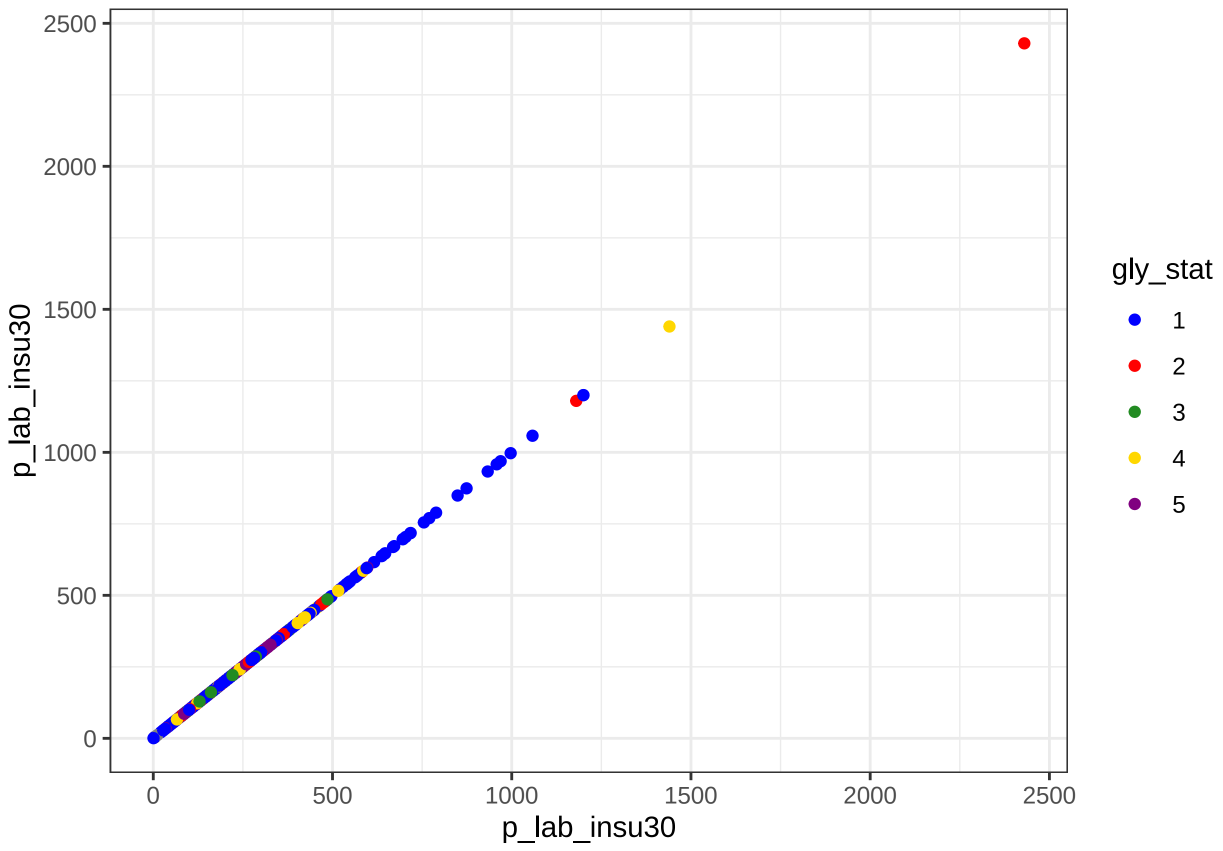

2500
2000
1500
1000
500
0
p_lab_insu30
0
500
1000
1500
2000
2500
p_lab_insu30
gly_stat
1
2
3
4
5

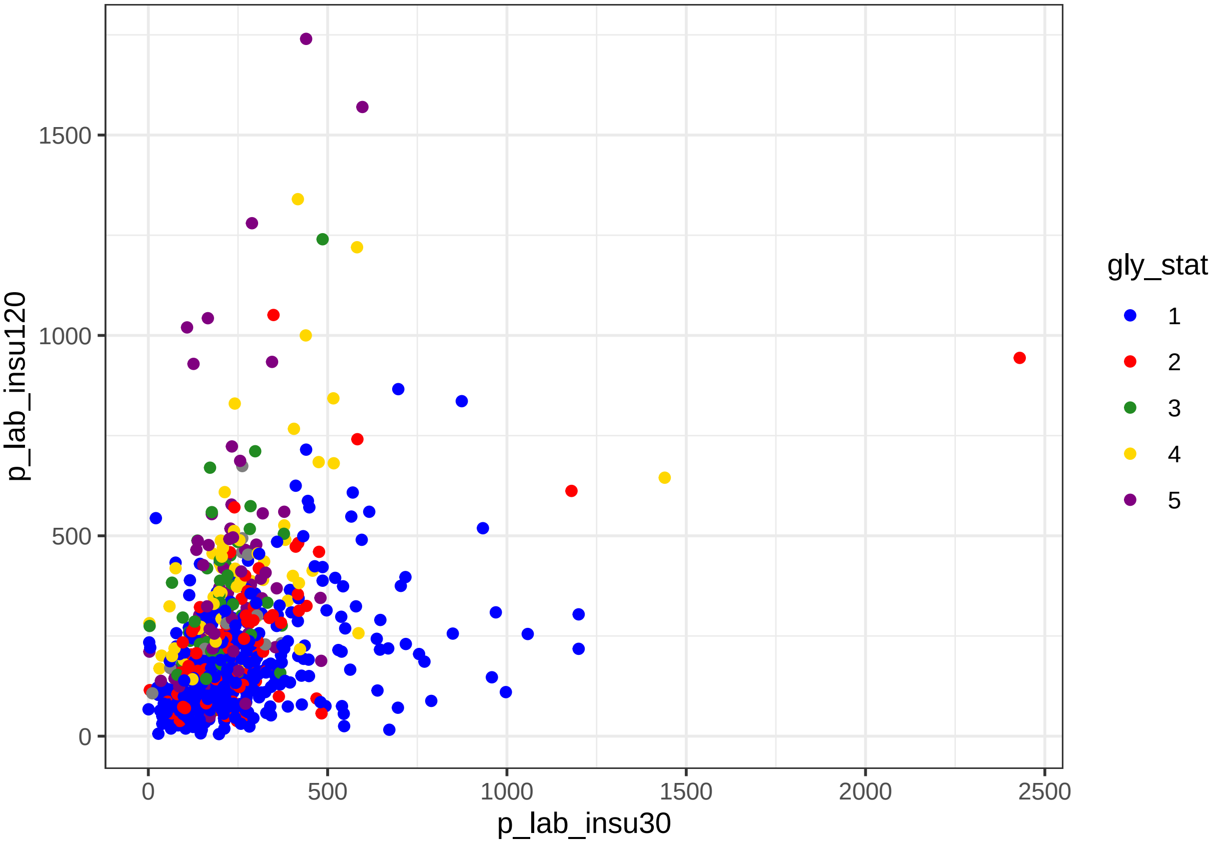

1500
1000
500
0
0
500
1000
1500
2000
2500
p_lab_insu30
p_lab_insu120
gly_stat
1
2
3
4
5

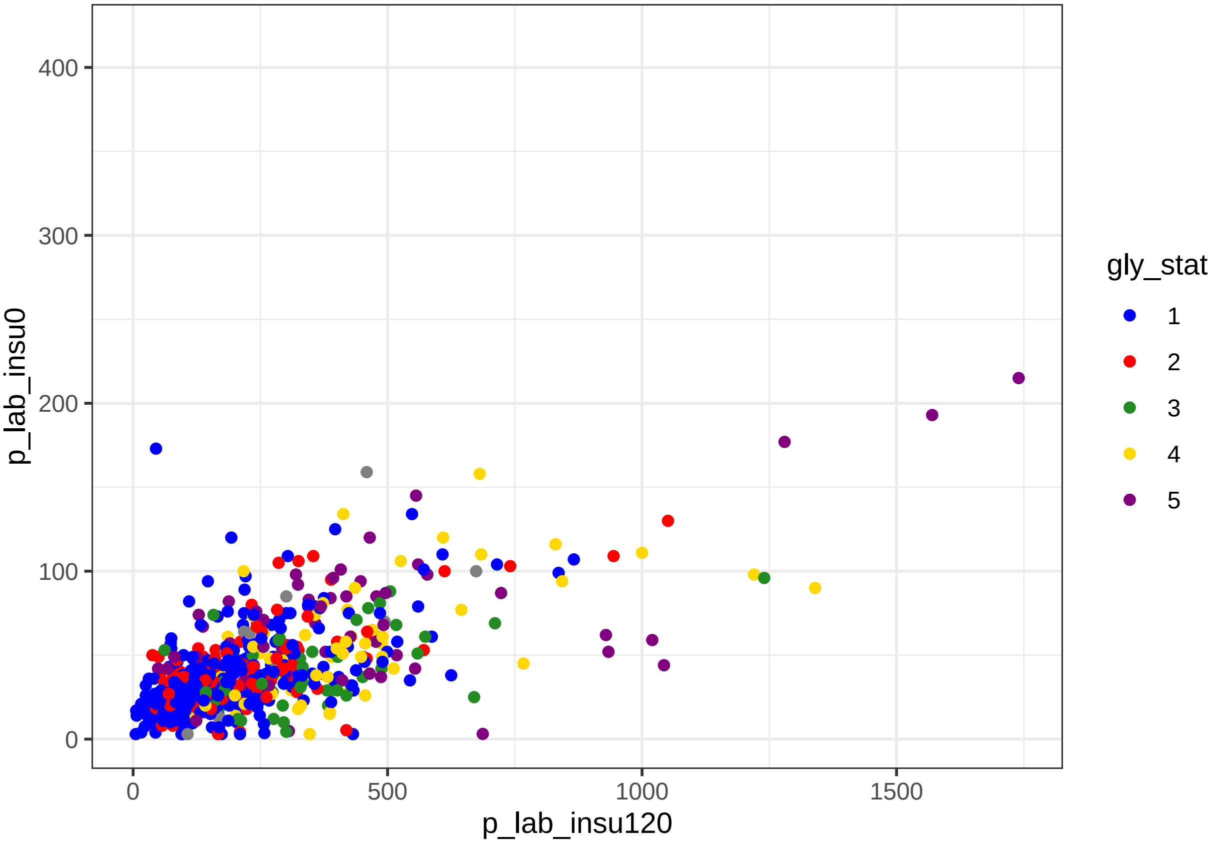

400
300
200
100
0
p_lab_insu0
0
500
1000
1500
p_lab_insu120
gly_stat
1
2
3
4
5

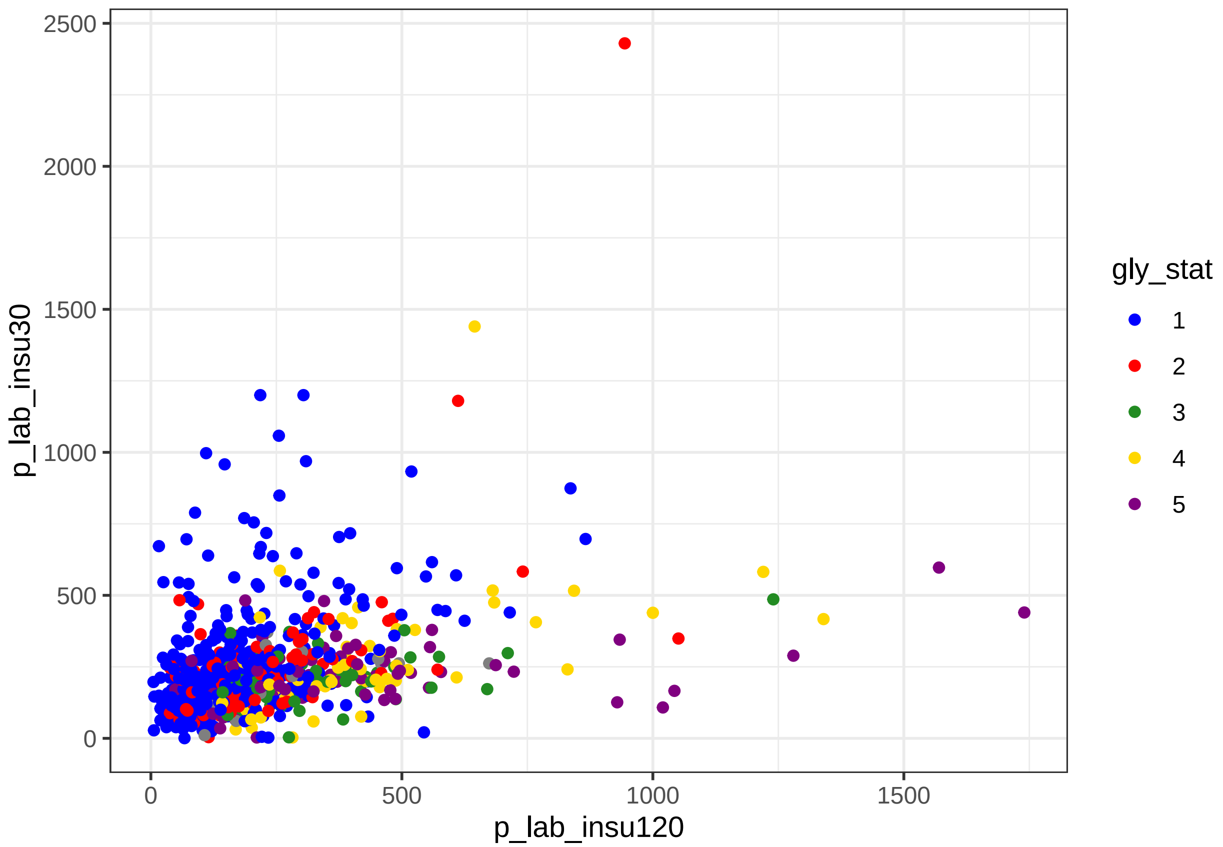
2500
2000
1500
1000
500
0
p_lab_insu30
0
500
1000
1500
p_lab_insu120
gly_stat
1
2
3
4
5

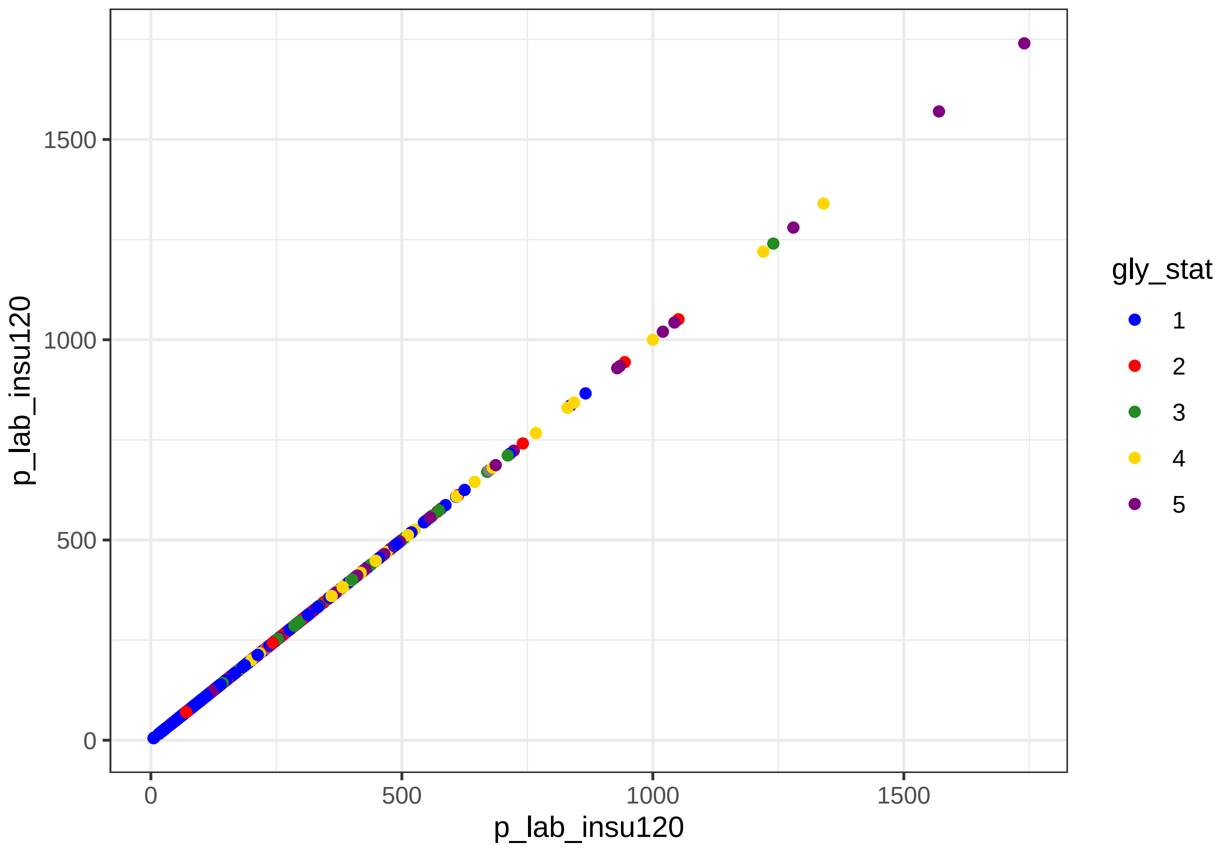


```
#Boxplots
for (i in c("p_lab_insu0", "p_lab_insu30", "p_lab_insu120")) {
  print(ggplot(Phe, aes_string(x="gly_stat", y=i, group="gly_stat")) +
    geom_boxplot() +
    theme_bw())
}
```

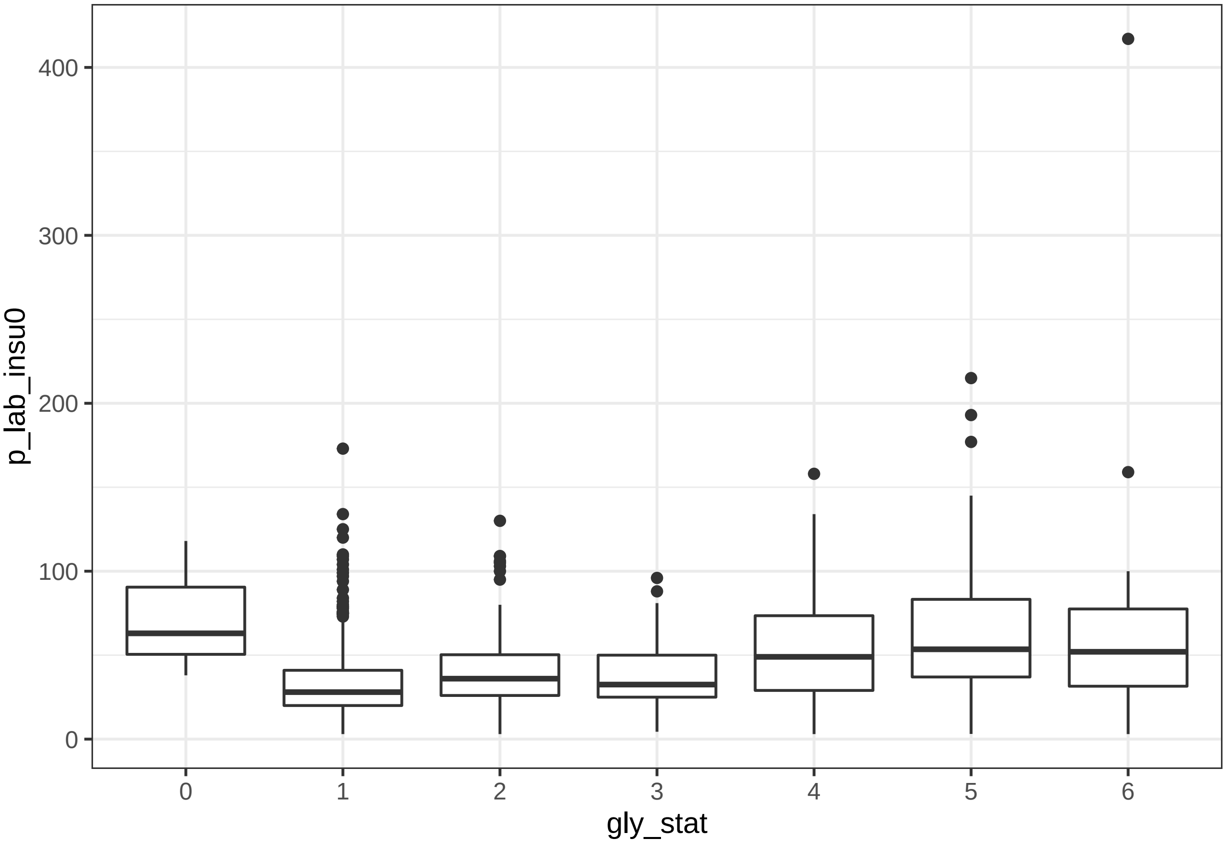
400
300
200
100
0
p_lab_insu0
0
1
2
3
4
5
6
gly_stat

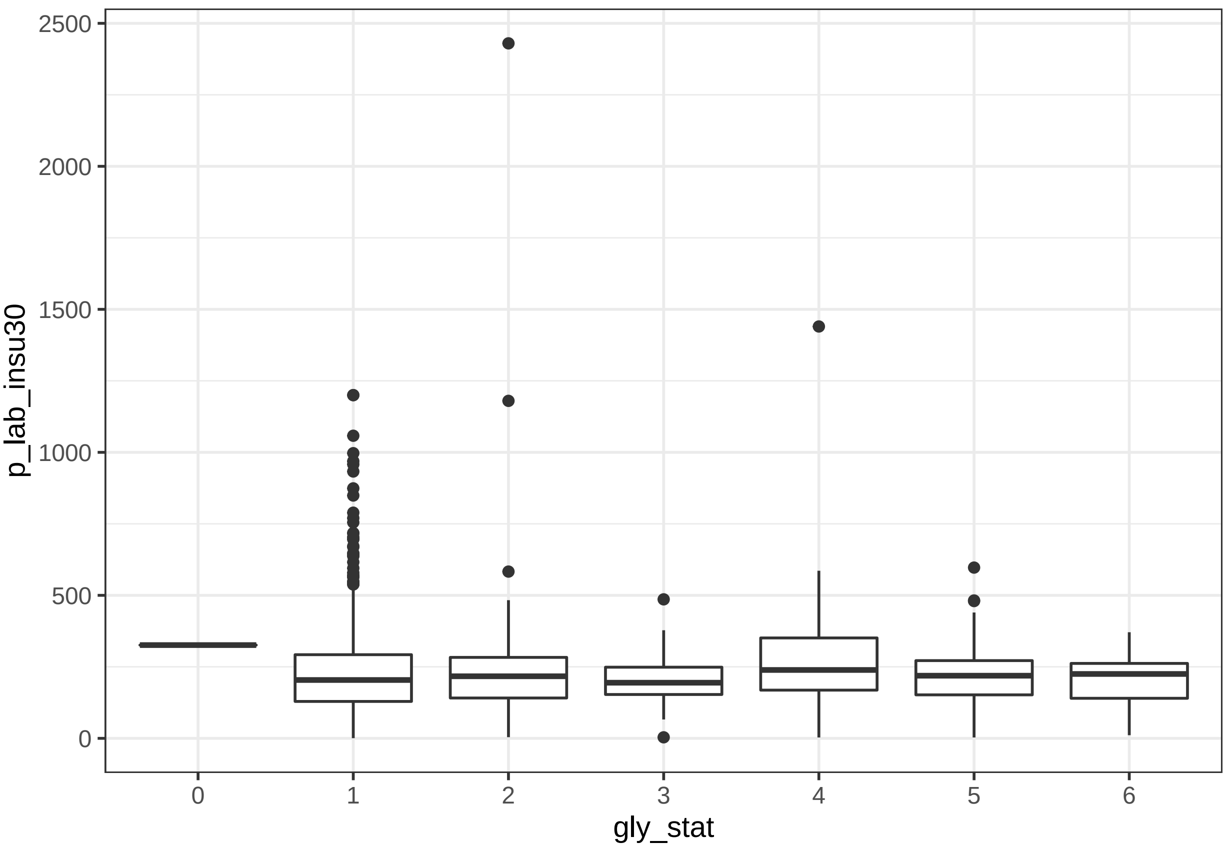

2500
2000
1500
1000
500
0
p_lab_insu30
0
1
2
3
4
5
6
gly_stat

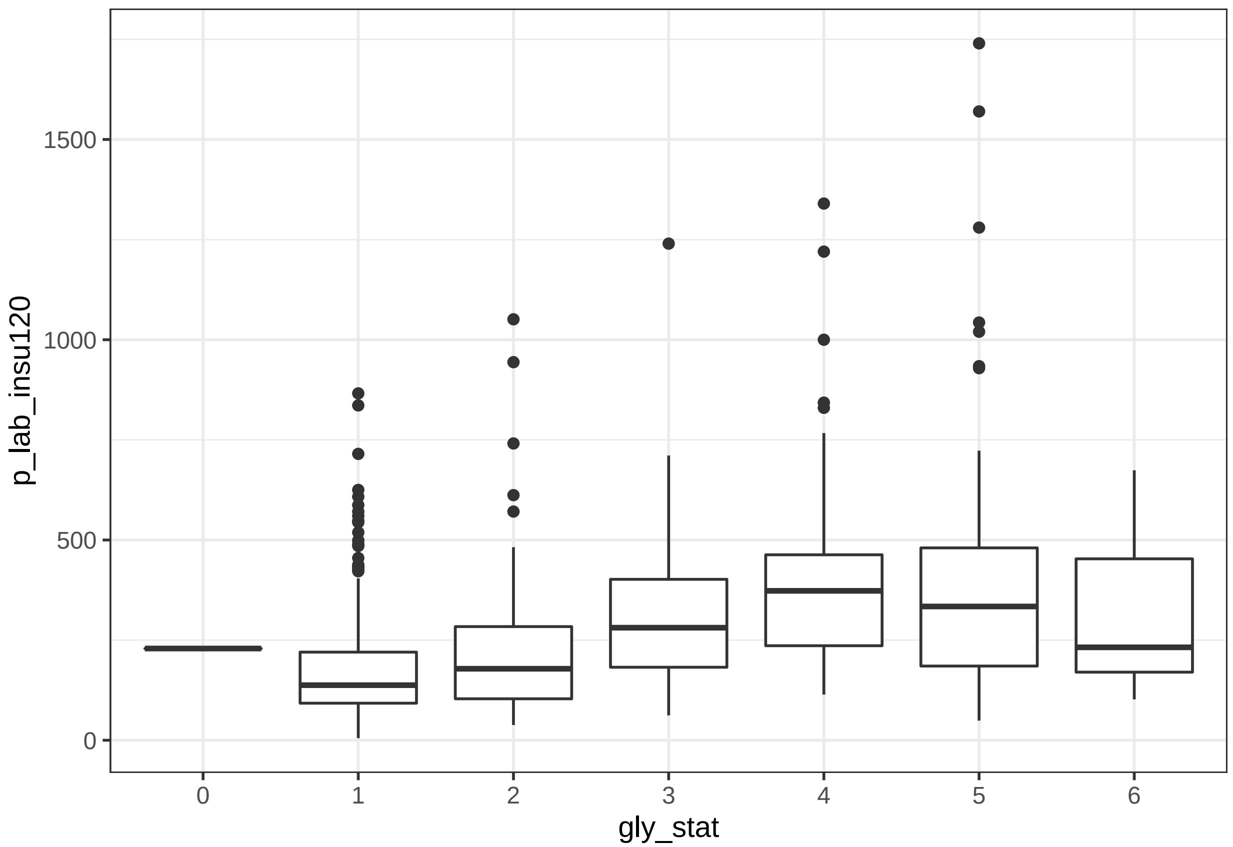


```
# pdf("ADDPRO_Phe_hist.pdf", width=12, height=6)
# hist(Phe$p_lab_trig)
# dev.off()
```

Model sample variables

How to handle different clasess?
How to handle NA? Can remove all negative to have direction is the positive. Thinking more about microbiome here
Sample data should be nondirectional. Using p-values

```
head(Phe)
```

Contingency tables

```
##     #FAM_ID  IND_ID FAT_ID MOT_ID sex     age2 log10_bmi p_gv_dob.x height
## 17  5150493 5150493      0      0   0 5880.087  1.446251  8/28/1933  167.4
## 61  5153433 5153433      0      0   1 4827.254  1.383020   1/5/1941  198.0
## 110 5153256 5153256      0      0   1 4229.895  1.420326  5/12/1944  178.0
## 113 5154542 5154542      0      0   0 4342.097  1.471367   5/2/1943  165.0
## 114 5154240 5154240      0      0   1 2797.042  1.470801   7/6/1956  188.0
```

```
## 115 3000082 3000082      0      0   1 5535.930  1.393746 10/14/1934  171.0
##     weight      bmi impedance fat_pc hours_fasting fasting waist_av hip_av
## 17    78.3 27.94157       450   39.2   10:40:00 AM       1   101.75 107.00
## 61    94.7 24.15570       548   24.5   11:32:00 AM       1    98.00 101.00
## 110   83.4 26.32243       423   22.3    1:13:00 PM       1    90.25 100.00
## 113   80.6 29.60514       524   34.9   10:56:00 AM       1   102.00 109.00
## 114  104.5 29.56655       506   31.7    2:07:00 PM       1   108.25 109.75
## 115   72.4 24.75976       498   24.3    3:12:00 PM       1    93.75  94.75
##       sbp_av    dbp_av    pp_av    hr_av crf_pw_sbp crf_pw_dbp centre
## 17  137.0000  83.00000 54.00000 59.66667        134         75      1
## 61  124.6667  73.66667 51.00000 59.33333        116         81      1
## 110 140.3333  89.00000 51.33333 63.00000        151         81      1
## 113 167.6667 102.33333 65.33333 64.33333        162         85      1
## 114 138.3333  91.33333 47.00000 51.00000        148         80      1
## 115 163.3333  91.66667 71.66667 80.00000        169         92      1
##     pwv_c_mean pwv_hr_mean crf_pw_mbp crf_pw_map operator mean_hr mean_c_sp
## 17   10.665617       56.25       98.6   94.66667    ltttp    55.5     125.0
## 61    7.567796       54.75       95.0   92.66667     lttp    56.0     108.5
## 110   9.789030       59.50      109.0  104.33333     binl    56.0     137.0
## 113   8.727473       66.25      115.8  110.66667     bihe    66.5     149.0
## 114   9.186072       47.50      107.2  102.66667     binl    50.5     132.5
## 115   9.450980       87.50      122.8  117.66667     bihe    88.0     139.0
##     mean_c_dp mean_c_pp p_gq_high_chol p_gq_high_chol_year p_gq_high_chol_code
## 17       75.5      49.5              1                2003                <NA>
## 61       82.0      26.5              1                2006                <NA>
## 110      82.0      55.0              1                2008                  Z1
## 113      87.0      62.0              0                  NA                <NA>
## 114      80.5      52.0              1                2008                <NA>
## 115      93.5      45.5              1                2006                  Z9
##     p_gq_high_bp p_gq_high_bp_year p_gq_high_bp_code p_gq_angina
## 17             1              1982              <NA>           0
## 61             0                NA              <NA>           0
## 110            1              2008              <NA>           0
## 113            1              2000              <NA>           0
## 114            0                NA              <NA>           0
## 115            1              2006                Z9           0
##     p_gq_angina_year p_gq_angina_code p_gq_mi p_gq_mi_year p_gq_mi_code
## 17                NA             <NA>       0           NA         <NA>
## 61                NA             <NA>       0           NA         <NA>
## 110               NA             <NA>       0           NA         <NA>
## 113               NA             <NA>       0           NA         <NA>
## 114               NA             <NA>       0           NA         <NA>
## 115               NA             <NA>       0           NA         <NA>
##     p_gq_stroke p_gq_stroke_year p_gq_stroke_code p_gq_palp p_gq_palp_year
## 17            0               NA             <NA>         0             NA
## 61            0               NA             <NA>         0             NA
## 110           0               NA             <NA>         0             NA
## 113           0               NA             <NA>         0             NA
## 114           0               NA             <NA>         0             NA
## 115           0               NA             <NA>         0             NA
##     p_gq_palp_code p_gq_card_sur p_gq_smoke p_gq_dia_met p_gq_dia_ins
## 17            <NA>             0          1            1            0
## 61            <NA>             0          1            0            0
## 110           <NA>             0          2            0            0
```

```
## 113          <NA>            0          2           0           0
## 114          <NA>            0          3           0           0
## 115          <NA>            0          2           0           0
##     p_gq_dia_sul p_gq_dia_tzd p_gq_dia_oth p_gq_dia_any p_gq_dia_num
## 17             0            0            0            1            1
## 61             0            0            0            0            0
## 110            0            0            0            0            0
## 113            0            0            0            0            0
## 114            0            0            0            0            0
## 115            0            0            0            0            0
##     p_gq_hyp_ace_arb p_gq_hyp_bet p_gq_hyp_cal p_gq_hyp_diu p_gq_hyp_oth
## 17                 0            0            1            1            0
## 61                 0            0            0            0            0
## 110                0            0            0            0            0
## 113                1            0            0            1            0
## 114                0            0            0            0            0
## 115                1            0            0            0            0
##     p_gq_hyp_any p_gq_hyp_num p_gq_lip_any p_gq_lip_sta p_gq_asa target_pop
## 17             1            2            1            1        0          1
## 61             0            0            0            0        0          1
## 110            0            0            1            1        0          1
## 113            1            2            0            0        0          1
## 114            0            0            0            0        0          1
## 115            1            1            1            1        0          1
##     p_lab_glp1_0 p_lab_glp1_30 p_lab_glp1_120 p_lab_glucagon_0
## 17            NA            NA             NA               NA
## 61            NA            NA             NA               NA
## 110            2            16             18               13
## 113            4            16             16                6
## 114            5            11             13               19
## 115           14            17             23               15
##     p_lab_glucagon_30 p_lab_glucagon_120 p_lab_gip_0 p_lab_gip_30 p_lab_gip_120
## 17                 NA                 NA          NA           NA            NA
## 61                 NA                 NA          NA           NA            NA
## 110                13                  9           4           28            18
## 113                 5                  4           5           53            65
## 114                16                 10           8           50            49
## 115                10                  6          19           62           103
##     auc_glp1_120 auc_gip_120 auc_glucagon_120 iauc_glp1_120 iauc_gip_120
## 17            NA          NA               NA            NA           NA
## 61            NA          NA               NA            NA           NA
## 110         1800        2550             1380          1560         2070
## 113         1740        6180              570          1260         5580
## 114         1320        5325             1695           720         4365
## 115         2265        8640             1095           585         6360
##     iauc_glucagon_120 auc_glp1_30 auc_gip_30 auc_glucagon_30 iauc_glp1_30
## 17                 NA          NA         NA              NA           NA
## 61                 NA          NA         NA              NA           NA
## 110              -180         270        480             390          210
## 113              -150         300        870             165          180
## 114              -585         240        870             525           90
## 115              -705         465       1215             375           45
##     iauc_gip_30 iauc_glucagon_30 diff_glp1_pct_30 diff_gip_pct_30
## 17           NA               NA               NA              NA
```

```
## 61           NA              NA              NA             NA
## 110         360               0       700.00000       600.0000
## 113         720             -15       300.00000       960.0000
## 114         630             -45       120.00000       525.0000
## 115         645             -75        21.42857       226.3158
##     diff_glucagon_pct_30 fup_date  age_fup inc_dm gly_stat p_gq_beer_week
## 17                    NA    18386 76.68172      1        6              0
## 61                    NA    18442 69.47844     NA        1              0
## 110              0.00000    18043 65.03765      1        6              1
## 113            -16.66667    17980 65.89459     NA        1              0
## 114            -15.78947    18043 52.88706     NA        4             10
## 115            -33.33333    17966 74.40383     NA        3              3
##     p_gq_wine_week p_gq_liquour_week p_gq_occupation p_gq_occupation_code
## 17               6                 1              14                   NA
## 61              20                 4              14                   NA
## 110              8                 0               5                   NA
## 113              0                 0              14                   NA
## 114             10                 2               4                   NA
## 115              1                 3              14                   NA
##     provedato p_lab_hba1c p_lab_insu0 p_lab_insu30 p_lab_insu120 p_lab_pglu0
## 17   5/4/2010         5.6          NA           NA            NA          NA
## 61  6/29/2010         5.3          20          343           167     5.35473
## 110 5/26/2009         7.6          36          140           102     7.50000
## 113 3/24/2009         5.4          41          207           178     5.70000
## 114 5/26/2009         5.7          29          153           311     6.60000
## 115 3/10/2009         5.5          37          229           451     6.00000
##     p_lab_pglu30 p_lab_pglu120 p_lab_bas p_lab_alat p_lab_creae p_lab_hdlc
## 17            NA            NA  41.36570   20.26667    63.32877   1.141017
## 61      8.406612      5.761648  42.32126   26.41358    82.05351   1.620846
## 110    12.300000      8.800000  70.00000   49.00000    71.00000   1.790000
## 113    10.000000      6.000000  77.00000   28.00000    62.00000   1.240000
## 114     9.800000      9.800000  54.00000   44.00000    74.00000   1.300000
## 115    10.100000      8.200000  76.00000   27.00000    73.00000   1.420000
##     p_lab_trig p_lab_chol p_lab_ldl p_lab_ualbcre p_ah_RMR p_ah_vo2max
## 17   1.6788599    4.40015  2.496015     54.000000 51.24982          NA
## 61   0.9254788    6.25469  4.213172      6.690594 58.16218    30.35638
## 110  1.3600000    4.40000  2.000000     10.000000 57.78144    35.68975
## 113  1.4000000    6.70000  4.800000     12.000000 50.12889    25.16387
## 114  2.0100000    6.90000  4.700000      3.000000 57.47608    41.71450
## 115  1.8400000    4.50000  2.200000     22.000000 59.99961    25.04898
##     p_ah_Pwear p_ah_SHR   p_ah_ACC p_ah_HRaS p_ah_PAEE_Branch4
## 17    94.07108       NA 0.06814791        NA                NA
## 61   187.70932       53 0.12762378  13.18944          38.01788
## 110  167.13553       52 0.17660184  16.66899          59.49018
## 113  139.73453       58 0.05942320  14.63449          22.67938
## 114  166.80020       51 0.08696414  10.89079          28.27822
## 115  137.39621       65 0.08448558  11.35122          16.37271
##     p_ah_PAEE_Branch7 p_ah_PAEE p_ah_sl_min p_ah_sb_min p_ah_light_min
## 17                 NA        NA          NA          NA             NA
## 61           36.19898  38.01788   1053.6475    32.95907       243.9554
## 110          53.00706  59.49018    956.1035    36.40088       275.4707
## 113          26.02414  22.67938   1047.0216    54.61235       304.5953
## 114          21.72524  28.27822   1094.3317    61.11291       228.3755
## 115          18.83500  16.37271   1182.9388    97.83360       147.2727
```

```
##     p_ah_mod_min p_ah_vig_min p_ah_tot_min p_ah_sl_hrs p_ah_sb_hrs
## 17            NA           NA           NA          NA          NA
## 61     108.84705    0.5910301     1440.000    17.56079   0.5493178
## 110    143.68873   27.8336906     1439.497    15.93506   0.6066813
## 113     32.34118    1.4295959     1440.000    17.45036   0.9102058
## 114     51.85959    4.3203735     1440.000    18.23886   1.0185485
## 115     11.95493    0.0000000     1440.000    19.71565   1.6305599
##     p_ah_light_hrs p_ah_mod_hrs p_ah_vig_hrs p_ah_tot_hrs p_ah_mvpa_hrs
## 17              NA           NA          NA           NA            NA
## 61        4.065923    1.8141174  0.009850502     24.00000     1.8239679
## 110       4.591178    2.3948121  0.463894844     23.99162     2.8587070
## 113       5.076588    0.5390196  0.023826599     24.00000     0.5628462
## 114       3.806258    0.8643265  0.072006226     24.00000     0.9363327
## 115       2.454545    0.1992488  0.000000000     24.00000     0.1992488
##     p_ah_sedent_hrs p_ah_sl_min_std p_ah_sb_min_std p_ah_light_min_std
## 17               NA              NA              NA                 NA
## 61         18.11011       1068.8121        26.76244           265.8308
## 110        16.54174        973.4598        24.00109           304.8315
## 113        18.36057       1070.9866       112.67647           246.2043
## 114        19.25741       1117.5615        53.18155           238.8804
## 115        21.34621       1211.3816        95.62097           130.2818
##     p_ah_mod_min_std p_ah_vig_min_std p_ah_tot_min_std p_ah_sl_hrs_std
## 17                NA               NA               NA              NA
## 61         78.594732         0.000000             1440        17.81353
## 110       121.144781        16.562834             1440        16.22433
## 113        10.132656         0.000000             1440        17.84978
## 114        28.933611         1.442986             1440        18.62602
## 115         2.715597         0.000000             1440        20.18969
##     p_ah_sb_hrs_std p_ah_light_hrs_std p_ah_mod_hrs_std p_ah_vig_hrs_std
## 17               NA                 NA               NA               NA
## 61        0.4460406           4.430513       1.30991221       0.00000000
## 110       0.4000182           5.080524       2.01907968       0.27604723
## 113       1.8779411           4.103405       0.16887760       0.00000000
## 114       0.8863592           3.981339       0.48222685       0.02404976
## 115       1.5936828           2.171363       0.04525995       0.00000000
##     p_ah_tot_hrs_std p_ah_mvpa_hrs_std p_ah_sedent_hrs_std p_rpaq_SCORERECREA
## 17                NA                NA                  NA           1.556548
## 61                24        1.30991221            18.25957           4.857143
## 110               24        2.29512691            16.62435                 NA
## 113               24        0.16887760            19.72772           4.553571
## 114               24        0.50627661            19.51238                 NA
## 115               24        0.04525995            21.78338           6.589286
##     p_rpaq_SCORE1 p_rpaq_SCORE2 p_rpaq_LIGHT p_rpaq_MODERATE p_rpaq_VIGOR
## 17       26.31557      33.51557    0.1071429        3.779762   0.00000000
## 61       23.44464      30.64464    0.1071429        1.232143   0.00000000
## 110            NA            NA           NA              NA           NA
## 113      22.58811      29.78811    0.0000000        1.428571   0.02222222
## 114            NA            NA           NA              NA           NA
## 115      21.20652      28.40652    0.0000000        1.196429   0.25833333
##     p_rpaq_SEDENT p_rpaq_rmrrpaq p_rpaq_PAEEHOME p_rpaq_PAEEJOB
## 17       20.11310        5688.92       1908.4019       2620.596
## 61       22.66071        7931.46       2658.8509          0.000
## 110            NA             NA              NA             NA
## 113      22.54921        5728.56       2718.5739          0.000
```

```
## 114            NA            NA            NA            NA
## 115      22.54524       6255.32       718.2633         0.000
##     p_rpaq_PAEETRANSPORT p_rpaq_PAEERECREA p_rpaq_PAEE p_rpaq_PAEE_kj_kg_day
## 17              51.41814           410.922    4991.338              63.74633
## 61               0.00000          1518.254    4177.105              44.10882
## 110                   NA                NA          NA                    NA
## 113              0.00000          1223.611    3942.185              48.91049
## 114                   NA                NA          NA                    NA
## 115              0.00000          1656.730    2374.994              32.80378
##       vat   sat   id pro_id    IDX Glycaemia_Status Risk sex_cat       BMI_cat
## 17  7.730 2.575  136  10543 X10543              KDM  KDM  Female    Overweight
## 61  6.225 2.445  540  10628 X10628           Gr1ngt  Low    Male Healthyweight
## 110 5.725 1.430 1169  10157 X10157              KDM  KDM    Male    Overweight
## 113 7.010 2.745 1215  10024 X10024           Gr1ngt  Low  Female    Overweight
## 114 9.850 2.705 1228  10165 X10165       Gr4IFG_IGT High    Male    Overweight
## 115 6.360 1.470 1231  10010 X10010          Gr3ilGT High    Male Healthyweight
##     Fat_cat Waist_cat   whratio whratio_cat Alcprweek Alcprweek_cat   Smoking
## 17     High      High 0.9509346        High         7      Moderate    Smoker
## 61      Low    Medium 0.9702970        High        24          High    Smoker
## 110     Low       Low 0.9025000        High         9      Moderate  Exsmoker
## 113    High      High 0.9357798        High         0    Abstinence  Exsmoker
## 114    High      High 0.9863326        High        22          High Nonsmoker
## 115     Low       Low 0.9894459        High         7      Moderate  Exsmoker
##               bp_cat         sbp_cat         dbp_cat hr_cat pp_cat trig_cat
## 17  Moderate_elevated          Normal          Normal    Low   High      Low
## 61      Mild_elevated          Normal             Low    Low   High      Low
## 110 Moderate_elevated   Mild_elevated          Normal Medium   High      Low
## 113 Severely_elevated Moderate_severe Moderate_severe    Low   High      Low
## 114 Moderate_elevated          Normal   Mild_elevated    Low   High     High
## 115 Severely_elevated Moderate_severe   Mild_elevated Medium   High      Low
##     hdlc_cat ldlc_cat chol_cat      act act_cat hba1c_cat
## 17      High      Low      Low 3.779762    High       Low
## 61      High     High     High 1.232143    High       Low
## 110     High      Low      Low       NA    <NA>      High
## 113     High     High     High 1.450794    High       Low
## 114     High     High     High       NA    <NA>       Low
## 115     High      Low      Low 1.454762    High       Low
```

```
summary(Phe)
```

```
##     #FAM_ID            IND_ID            FAT_ID      MOT_ID       sex
##  Min.   :3000082   Min.   :3000082   Min.   :0   Min.   :0   Min.   :0.0000
##  1st Qu.:3027105   1st Qu.:3027105   1st Qu.:0   1st Qu.:0   1st Qu.:0.0000
##  Median :5169456   Median :5169456   Median :0   Median :0   Median :1.0000
##  Mean   :4440921   Mean   :4440921   Mean   :0   Mean   :0   Mean   :0.5456
##  3rd Qu.:5185706   3rd Qu.:5185706   3rd Qu.:0   3rd Qu.:0   3rd Qu.:1.0000
##  Max.   :5250044   Max.   :5250044   Max.   :0   Max.   :0   Max.   :1.0000
##
##       age2        log10_bmi      p_gv_dob.x           height
##  Min.   :2381   Min.   :1.205   Length:746         Min.   :146.0
##  1st Qu.:4049   1st Qu.:1.381   Class :character   1st Qu.:164.1
##  Median :4622   Median :1.423   Mode  :character   Median :171.2
##  Mean   :4624   Mean   :1.424                      Mean   :171.0
##  3rd Qu.:5253   3rd Qu.:1.468                      3rd Qu.:177.5
```

```
##  Max.   :6304   Max.   :1.644                     Max.   :198.0  
##                                                                  
##      weight           bmi          impedance         fat_pc     
##  Min.   : 43.60   Min.   :16.01   Min.   :317.0   Min.   : 7.30  
##  1st Qu.: 68.30   1st Qu.:24.04   1st Qu.:452.8   1st Qu.:25.30  
##  Median : 78.60   Median :26.48   Median :498.0   Median :30.80  
##  Mean   : 78.78   Mean   :26.87   Mean   :503.1   Mean   :31.54  
##  3rd Qu.: 87.97   3rd Qu.:29.35   3rd Qu.:551.0   3rd Qu.:37.85  
##  Max.   :129.70   Max.   :44.02   Max.   :844.0   Max.   :52.90  
##                                   NA's   :10      NA's   :7      
##  hours_fasting         fasting         waist_av          hip_av     
##  Length:746         Min.   :0.000   Min.   : 62.70   Min.   : 66.1  
##  Class :character   1st Qu.:1.000   1st Qu.: 86.55   1st Qu.: 96.0  
##  Mode  :character   Median :1.000   Median : 94.50   Median :101.0  
##                     Mean   :0.996   Mean   : 94.97   Mean   :101.6  
##                     3rd Qu.:1.000   3rd Qu.:103.00   3rd Qu.:106.5  
##                     Max.   :1.000   Max.   :140.00   Max.   :152.0  
##                     NA's   :3                                       
##      sbp_av           dbp_av           pp_av           hr_av       
##  Min.   : 83.33   Min.   : 53.00   Min.   :23.67   Min.   : 40.33  
##  1st Qu.:120.33   1st Qu.: 74.67   1st Qu.:42.33   1st Qu.: 58.92  
##  Median :131.67   Median : 80.67   Median :50.17   Median : 66.00  
##  Mean   :132.13   Mean   : 81.29   Mean   :50.84   Mean   : 66.93  
##  3rd Qu.:142.00   3rd Qu.: 87.42   3rd Qu.:57.67   3rd Qu.: 74.08  
##  Max.   :193.67   Max.   :113.00   Max.   :95.67   Max.   :105.00  
##  NA's   :2        NA's   :2        NA's   :2       NA's   :2       
##    crf_pw_sbp      crf_pw_dbp         centre    pwv_c_mean      pwv_hr_mean    
##  Min.   : 85.0   Min.   : 51.00   Min.   :1   Min.   : 4.183   Min.   : 40.00  
##  1st Qu.:122.0   1st Qu.: 70.00   1st Qu.:1   1st Qu.: 6.851   1st Qu.: 56.00  
##  Median :132.0   Median : 76.00   Median :1   Median : 7.974   Median : 62.00  
##  Mean   :133.2   Mean   : 76.46   Mean   :1   Mean   : 8.244   Mean   : 63.20  
##  3rd Qu.:144.0   3rd Qu.: 82.00   3rd Qu.:1   3rd Qu.: 9.348   3rd Qu.: 69.25  
##  Max.   :208.0   Max.   :111.00   Max.   :1   Max.   :16.985   Max.   :135.25  
##  NA's   :11      NA's   :11                   NA's   :48       NA's   :48      
##    crf_pw_mbp       crf_pw_map       operator            mean_hr     
##  Min.   : 67.00   Min.   : 65.00   Length:746         Min.   :38.00  
##  1st Qu.: 91.60   1st Qu.: 88.17   Class :character   1st Qu.:57.00  
##  Median : 98.20   Median : 94.33   Mode  :character   Median :63.00  
##  Mean   : 99.16   Mean   : 95.37                      Mean   :63.87  
##  3rd Qu.:106.40   3rd Qu.:102.67                      3rd Qu.:70.00  
##  Max.   :146.20   Max.   :139.33                      Max.   :99.00  
##  NA's   :11       NA's   :11                          NA's   :37     
##    mean_c_sp       mean_c_dp        mean_c_pp     p_gq_high_chol  
##  Min.   : 77.0   Min.   : 52.00   Min.   :18.33   Min.   :0.0000  
##  1st Qu.:112.5   1st Qu.: 71.00   1st Qu.:37.00   1st Qu.:0.0000  
##  Median :122.0   Median : 77.00   Median :44.00   Median :0.0000  
##  Mean   :123.2   Mean   : 77.66   Mean   :45.55   Mean   :0.4019  
##  3rd Qu.:132.5   3rd Qu.: 83.50   3rd Qu.:53.00   3rd Qu.:1.0000  
##  Max.   :198.0   Max.   :112.50   Max.   :92.00   Max.   :1.0000  
##  NA's   :37      NA's   :37       NA's   :37      NA's   :7       
##  p_gq_high_chol_year p_gq_high_chol_code  p_gq_high_bp    p_gq_high_bp_year
##  Min.   :1970        Length:746          Min.   :0.0000   Min.   :1960     
##  1st Qu.:2002        Class :character    1st Qu.:0.0000   1st Qu.:1995     
##  Median :2006        Mode  :character    Median :0.0000   Median :2002     
```

```
##  Mean   :2004                           Mean   :0.4818   Mean   :1999
##  3rd Qu.:2008                           3rd Qu.:1.0000   3rd Qu.:2007
##  Max.   :2010                           Max.   :1.0000   Max.   :2010
##  NA's   :507                            NA's   :5        NA's   :469
##  p_gq_high_bp_code   p_gq_angina      p_gq_angina_year p_gq_angina_code
##  Length:746         Min.   :0.00000   Min.   :1987     Length:746
##  Class :character   1st Qu.:0.00000   1st Qu.:1999     Class :character
##  Mode  :character   Median :0.00000   Median :2002     Mode  :character
##                     Mean   :0.07027   Mean   :2002
##                     3rd Qu.:0.00000   3rd Qu.:2007
##                     Max.   :1.00000   Max.   :2010
##                     NA's   :6         NA's   :710
##     p_gq_mi         p_gq_mi_year  p_gq_mi_code        p_gq_stroke
##  Min.   :0.00000   Min.   :1991   Length:746         Min.   :0.00000
##  1st Qu.:0.00000   1st Qu.:1995   Class :character   1st Qu.:0.00000
##  Median :0.00000   Median :2000   Mode  :character   Median :0.00000
##  Mean   :0.03374   Mean   :2001                      Mean   :0.04858
##  3rd Qu.:0.00000   3rd Qu.:2007                      3rd Qu.:0.00000
##  Max.   :1.00000   Max.   :2009                      Max.   :1.00000
##  NA's   :5         NA's   :722                       NA's   :5
##  p_gq_stroke_year p_gq_stroke_code     p_gq_palp      p_gq_palp_year
##  Min.   :1964     Length:746         Min.   :0.0000   Min.   :1958
##  1st Qu.:1995     Class :character   1st Qu.:0.0000   1st Qu.:1995
##  Median :2001     Mode  :character   Median :0.0000   Median :2003
##  Mean   :1998                        Mean   :0.1438   Mean   :1999
##  3rd Qu.:2006                        3rd Qu.:0.0000   3rd Qu.:2007
##  Max.   :2009                        Max.   :1.0000   Max.   :2010
##  NA's   :715                         NA's   :9        NA's   :670
##  p_gq_palp_code     p_gq_card_sur       p_gq_smoke     p_gq_dia_met
##  Length:746         Min.   :0.00000   Min.   :1.000   Min.   :0.0000
##  Class :character   1st Qu.:0.00000   1st Qu.:2.000   1st Qu.:0.0000
##  Mode  :character   Median :0.00000   Median :2.000   Median :0.0000
##                     Mean   :0.04595   Mean   :2.178   Mean   :0.0108
##                     3rd Qu.:0.00000   3rd Qu.:3.000   3rd Qu.:0.0000
##                     Max.   :1.00000   Max.   :3.000   Max.   :1.0000
##                     NA's   :6         NA's   :5       NA's   :5
##   p_gq_dia_ins  p_gq_dia_sul      p_gq_dia_tzd  p_gq_dia_oth  p_gq_dia_any
##  Min.   :0     Min.   :0.00000   Min.   :0     Min.   :0     Min.   :0.0000
##  1st Qu.:0     1st Qu.:0.00000   1st Qu.:0     1st Qu.:0     1st Qu.:0.0000
##  Median :0     Median :0.00000   Median :0     Median :0     Median :0.0000
##  Mean   :0     Mean   :0.00135   Mean   :0     Mean   :0     Mean   :0.0108
##  3rd Qu.:0     3rd Qu.:0.00000   3rd Qu.:0     3rd Qu.:0     3rd Qu.:0.0000
##  Max.   :0     Max.   :1.00000   Max.   :0     Max.   :0     Max.   :1.0000
##  NA's   :5     NA's   :5         NA's   :5     NA's   :5     NA's   :5
##   p_gq_dia_num     p_gq_hyp_ace_arb  p_gq_hyp_bet    p_gq_hyp_cal
##  Min.   :0.00000   Min.   :0.0000   Min.   :0.000   Min.   :0.0000
##  1st Qu.:0.00000   1st Qu.:0.0000   1st Qu.:0.000   1st Qu.:0.0000
##  Median :0.00000   Median :0.0000   Median :0.000   Median :0.0000
##  Mean   :0.01215   Mean   :0.2901   Mean   :0.108   Mean   :0.1457
##  3rd Qu.:0.00000   3rd Qu.:1.0000   3rd Qu.:0.000   3rd Qu.:0.0000
##  Max.   :2.00000   Max.   :1.0000   Max.   :1.000   Max.   :1.0000
##  NA's   :5         NA's   :5        NA's   :5       NA's   :5
##   p_gq_hyp_diu     p_gq_hyp_oth      p_gq_hyp_any     p_gq_hyp_num
##  Min.   :0.0000   Min.   :0.00000   Min.   :0.0000   Min.   :0.0000
```

```
##  1st Qu.:0.0000   1st Qu.:0.00000   1st Qu.:0.0000   1st Qu.:0.0000  
##  Median :0.0000   Median :0.00000   Median :0.0000   Median :0.0000  
##  Mean   :0.2402   Mean   :0.01485   Mean   :0.4426   Mean   :0.8178  
##  3rd Qu.:0.0000   3rd Qu.:0.00000   3rd Qu.:1.0000   3rd Qu.:2.0000  
##  Max.   :1.0000   Max.   :1.00000   Max.   :1.0000   Max.   :6.0000  
##  NA's   :5        NA's   :5         NA's   :5        NA's   :5       
##   p_gq_lip_any     p_gq_lip_sta      p_gq_asa        target_pop  p_lab_glp1_0
##  Min.   :0.0000   Min.   :0.000   Min.   :0.0000   Min.   :1    Min.   : 1.0  
##  1st Qu.:0.0000   1st Qu.:0.000   1st Qu.:0.0000   1st Qu.:1    1st Qu.: 8.0  
##  Median :0.0000   Median :0.000   Median :0.0000   Median :1    Median :12.0  
##  Mean   :0.2578   Mean   :0.251   Mean   :0.1633   Mean   :1    Mean   :11.9  
##  3rd Qu.:1.0000   3rd Qu.:1.000   3rd Qu.:0.0000   3rd Qu.:1    3rd Qu.:16.0  
##  Max.   :1.0000   Max.   :1.000   Max.   :1.0000   Max.   :1    Max.   :31.0  
##  NA's   :5        NA's   :5       NA's   :5                     NA's   :119   
##  p_lab_glp1_30    p_lab_glp1_120   p_lab_glucagon_0 p_lab_glucagon_30
##  Min.   :  1.00   Min.   :  1.00   Min.   :  0.50   Min.   :  0.000  
##  1st Qu.: 18.00   1st Qu.: 13.50   1st Qu.:  6.00   1st Qu.:  6.000  
##  Median : 27.00   Median : 19.00   Median :  9.00   Median :  8.000  
##  Mean   : 32.72   Mean   : 21.56   Mean   : 10.35   Mean   :  9.396  
##  3rd Qu.: 38.00   3rd Qu.: 26.00   3rd Qu.: 13.00   3rd Qu.: 11.000  
##  Max.   :201.00   Max.   :201.00   Max.   :189.00   Max.   :185.000  
##  NA's   :128      NA's   :127      NA's   :119      NA's   :127      
##  p_lab_glucagon_120  p_lab_gip_0       p_lab_gip_30   p_lab_gip_120   
##  Min.   : 0.000     Min.   :  0.500   Min.   :  7.0   Min.   :  9.00  
##  1st Qu.: 4.000     1st Qu.:  6.000   1st Qu.: 36.0   1st Qu.: 32.00  
##  Median : 5.000     Median :  8.000   Median : 47.0   Median : 44.00  
##  Mean   : 5.868     Mean   :  9.186   Mean   : 51.6   Mean   : 46.93  
##  3rd Qu.: 7.000     3rd Qu.: 11.000   3rd Qu.: 63.5   3rd Qu.: 58.00  
##  Max.   :94.000     Max.   :113.000   Max.   :187.0   Max.   :162.00  
##  NA's   :126        NA's   :125       NA's   :135     NA's   :133     
##   auc_glp1_120    auc_gip_120    auc_glucagon_120  iauc_glp1_120  
##  Min.   :  165   Min.   :  900   Min.   :  135.0   Min.   : -690  
##  1st Qu.: 1980   1st Qu.: 3881   1st Qu.:  615.0   1st Qu.:  720  
##  Median : 2685   Median : 4965   Median :  840.0   Median : 1222  
##  Mean   : 3106   Mean   : 5340   Mean   :  983.4   Mean   : 1677  
##  3rd Qu.: 3664   3rd Qu.: 6390   3rd Qu.: 1200.0   3rd Qu.: 2040  
##  Max.   :21555   Max.   :17130   Max.   :18165.0   Max.   :17955  
##  NA's   :130     NA's   :138     NA's   :129       NA's   :130    
##   iauc_gip_120   iauc_glucagon_120  auc_glp1_30       auc_gip_30    
##  Min.   :-1920   Min.   :-4515.0   Min.   :  30.0   Min.   : 135.0  
##  1st Qu.: 2891   1st Qu.: -420.0   1st Qu.: 435.0   1st Qu.: 645.0  
##  Median : 3968   Median : -210.0   Median : 600.0   Median : 855.0  
##  Mean   : 4242   Mean   : -256.5   Mean   : 669.5   Mean   : 911.7  
##  3rd Qu.: 5265   3rd Qu.:  -30.0   3rd Qu.: 795.0   3rd Qu.:1095.0  
##  Max.   :15570   Max.   : 1380.0   Max.   :3465.0   Max.   :3450.0  
##  NA's   :138     NA's   :129       NA's   :128      NA's   :135     
##  auc_glucagon_30   iauc_glp1_30     iauc_gip_30     iauc_glucagon_30 
##  Min.   :  30.0   Min.   : -75.0   Min.   : -15.0   Min.   :-240.00  
##  1st Qu.: 180.0   1st Qu.: 120.0   1st Qu.: 420.0   1st Qu.: -45.00  
##  Median : 255.0   Median : 210.0   Median : 585.0   Median : -15.00  
##  Mean   : 295.9   Mean   : 312.1   Mean   : 636.3   Mean   : -14.03  
##  3rd Qu.: 360.0   3rd Qu.: 375.0   3rd Qu.: 795.0   3rd Qu.:  15.00  
##  Max.   :5610.0   Max.   :2925.0   Max.   :2610.0   Max.   : 270.00  
##  NA's   :127      NA's   :128      NA's   :135      NA's   :127      
```

```
##  diff_glp1_pct_30  diff_gip_pct_30  diff_glucagon_pct_30    fup_date
##  Min.   : -23.81   Min.   : -12.5   Min.   :-100.000     Min.   :17966
##  1st Qu.:  67.85   1st Qu.: 333.3   1st Qu.: -25.000     1st Qu.:18099
##  Median : 132.29   Median : 490.0   Median :  -9.091     Median :18372
##  Mean   : 323.19   Mean   : 600.0   Mean   :  10.508     Mean   :18327
##  3rd Qu.: 282.95   3rd Qu.: 700.0   3rd Qu.:  11.111     3rd Qu.:18519
##  Max.   :8800.00   Max.   :6700.0   Max.   :1700.000     Max.   :18646
##  NA's   :128       NA's   :135      NA's   :127
##     age_fup          inc_dm    gly_stat p_gq_beer_week   p_gq_wine_week
##  Min.   :48.79   Min.   :1     0:  3    Min.   : 0.000   Min.   : 0.00
##  1st Qu.:63.63   1st Qu.:1     1:421    1st Qu.: 0.000   1st Qu.: 2.00
##  Median :67.98   Median :1     2:112    Median : 1.000   Median : 6.00
##  Mean   :67.74   Mean   :1     3: 58    Mean   : 2.653   Mean   : 7.37
##  3rd Qu.:72.47   3rd Qu.:1     4: 63    3rd Qu.: 3.000   3rd Qu.:10.00
##  Max.   :79.40   Max.   :1     5: 68    Max.   :30.000   Max.   :42.00
##                  NA's   :725   6: 21    NA's   :68       NA's   :19
##  p_gq_liquour_week p_gq_occupation  p_gq_occupation_code  provedato
##  Min.   :-9.000    Min.   : 1.000   Min.   :-7.00        Length:746
##  1st Qu.: 0.000    1st Qu.: 5.000   1st Qu.:23.00        Class :character
##  Median : 0.000    Median :14.000   Median :27.00        Mode  :character
##  Mean   : 1.281    Mean   : 9.969   Mean   :22.89
##  3rd Qu.: 2.000    3rd Qu.:14.000   3rd Qu.:28.00
##  Max.   :15.000    Max.   :17.000   Max.   :60.00
##  NA's   :78        NA's   :5        NA's   :737
##   p_lab_hba1c     p_lab_insu0      p_lab_insu30    p_lab_insu120
##  Min.   :3.700   Min.   :  3.00   Min.   :   0.6   Min.   :   5.2
##  1st Qu.:5.500   1st Qu.: 22.75   1st Qu.: 139.0   1st Qu.: 111.0
##  Median :5.700   Median : 33.00   Median : 206.0   Median : 180.0
##  Mean   :5.707   Mean   : 40.81   Mean   : 243.6   Mean   : 234.8
##  3rd Qu.:5.900   3rd Qu.: 49.25   3rd Qu.: 286.0   3rd Qu.: 299.5
##  Max.   :9.500   Max.   :417.00   Max.   :2430.0   Max.   :1740.0
##  NA's   :1       NA's   :6        NA's   :13       NA's   :11
##   p_lab_pglu0      p_lab_pglu30    p_lab_pglu120      p_lab_bas
##  Min.   : 2.913   Min.   : 4.032   Min.   : 2.303   Min.   : 13.65
##  1st Qu.: 5.500   1st Qu.: 8.100   1st Qu.: 5.355   1st Qu.: 53.00
##  Median : 5.863   Median : 9.100   Median : 6.400   Median : 62.39
##  Mean   : 5.960   Mean   : 9.137   Mean   : 6.864   Mean   : 64.65
##  3rd Qu.: 6.270   3rd Qu.:10.136   3rd Qu.: 7.800   3rd Qu.: 73.64
##  Max.   :12.400   Max.   :15.800   Max.   :20.100   Max.   :170.37
##  NA's   :6        NA's   :12       NA's   :11
##    p_lab_alat       p_lab_creae       p_lab_hdlc      p_lab_trig
##  Min.   :  6.948   Min.   : 44.60   Min.   :0.620   Min.   :0.3300
##  1st Qu.: 18.000   1st Qu.: 66.00   1st Qu.:1.260   1st Qu.:0.7825
##  Median : 23.340   Median : 75.00   Median :1.510   Median :1.0600
##  Mean   : 26.326   Mean   : 76.78   Mean   :1.583   Mean   :1.2177
##  3rd Qu.: 31.536   3rd Qu.: 85.00   3rd Qu.:1.858   3rd Qu.:1.4610
##  Max.   :207.000   Max.   :252.55   Max.   :3.510   Max.   :6.6300
##
##    p_lab_chol      p_lab_ldl     p_lab_ualbcre        p_ah_RMR
##  Min.   :2.649   Min.   :1.200   Min.   :   1.144   Min.   :41.91
##  1st Qu.:4.709   1st Qu.:2.600   1st Qu.:   5.245   1st Qu.:53.48
##  Median :5.400   Median :3.300   Median :   8.374   Median :56.75
##  Mean   :5.433   Mean   :3.300   Mean   :  20.154   Mean   :56.44
##  3rd Qu.:6.152   3rd Qu.:3.900   3rd Qu.:  15.151   3rd Qu.:59.36
```

```
##  Max.   :8.315   Max.   :6.561   Max.   :1214.620   Max.   :74.26
##                  NA's   :2       NA's   :4          NA's   :147
##   p_ah_vo2max      p_ah_Pwear        p_ah_SHR        p_ah_ACC
##  Min.   :18.10   Min.   : 24.85   Min.   :37.00   Min.   :0.00528
##  1st Qu.:26.28   1st Qu.:141.72   1st Qu.:52.00   1st Qu.:0.04711
##  Median :28.57   Median :164.56   Median :57.00   Median :0.06431
##  Mean   :29.56   Mean   :151.44   Mean   :57.51   Mean   :0.07101
##  3rd Qu.:32.62   3rd Qu.:171.01   3rd Qu.:62.00   3rd Qu.:0.08911
##  Max.   :49.10   Max.   :272.87   Max.   :85.00   Max.   :0.24084
##  NA's   :434     NA's   :147      NA's   :210     NA's   :153
##    p_ah_HRaS      p_ah_PAEE_Branch4 p_ah_PAEE_Branch7   p_ah_PAEE
##  Min.   : 4.649   Min.   : 7.208    Min.   :  5.238   Min.   :  5.238
##  1st Qu.:11.388   1st Qu.:21.245    1st Qu.: 19.575   1st Qu.: 19.356
##  Median :13.528   Median :28.397    Median : 27.073   Median : 26.776
##  Mean   :14.091   Mean   :31.000    Mean   : 28.998   Mean   : 29.169
##  3rd Qu.:16.658   3rd Qu.:40.401    3rd Qu.: 35.373   3rd Qu.: 37.109
##  Max.   :32.757   Max.   :78.975    Max.   :101.850   Max.   :101.850
##  NA's   :210      NA's   :427       NA's   :213       NA's   :213
##   p_ah_sl_min      p_ah_sb_min      p_ah_light_min   p_ah_mod_min
##  Min.   : 720.7   Min.   :  8.358   Min.   : 22.9   Min.   :  0.00
##  1st Qu.: 955.8   1st Qu.: 46.387   1st Qu.:201.5   1st Qu.: 20.72
##  Median :1044.2   Median : 66.658   Median :266.9   Median : 42.96
##  Mean   :1038.8   Mean   : 72.031   Mean   :272.7   Mean   : 54.40
##  3rd Qu.:1124.3   3rd Qu.: 90.081   3rd Qu.:329.1   3rd Qu.: 73.66
##  Max.   :1366.3   Max.   :230.174   Max.   :603.0   Max.   :323.17
##  NA's   :211      NA's   :211       NA's   :211     NA's   :211
##   p_ah_vig_min      p_ah_tot_min   p_ah_sl_hrs     p_ah_sb_hrs
##  Min.   :  0.000   Min.   :1424   Min.   :12.01   Min.   :0.1393
##  1st Qu.:  0.000   1st Qu.:1440   1st Qu.:15.93   1st Qu.:0.7731
##  Median :  0.000   Median :1440   Median :17.40   Median :1.1110
##  Mean   :  1.963   Mean   :1440   Mean   :17.31   Mean   :1.2005
##  3rd Qu.:  1.218   3rd Qu.:1440   3rd Qu.:18.74   3rd Qu.:1.5013
##  Max.   :102.078   Max.   :1440   Max.   :22.77   Max.   :3.8362
##  NA's   :211       NA's   :211    NA's   :211     NA's   :211
##  p_ah_light_hrs     p_ah_mod_hrs     p_ah_vig_hrs      p_ah_tot_hrs
##  Min.   : 0.3817   Min.   :0.0000   Min.   :0.00000   Min.   :23.73
##  1st Qu.: 3.3576   1st Qu.:0.3454   1st Qu.:0.00000   1st Qu.:24.00
##  Median : 4.4475   Median :0.7160   Median :0.00000   Median :24.00
##  Mean   : 4.5458   Mean   :0.9067   Mean   :0.03272   Mean   :24.00
##  3rd Qu.: 5.4849   3rd Qu.:1.2277   3rd Qu.:0.02029   3rd Qu.:24.00
##  Max.   :10.0507   Max.   :5.3862   Max.   :1.70130   Max.   :24.00
##  NA's   :211       NA's   :211      NA's   :211       NA's   :211
##  p_ah_mvpa_hrs    p_ah_sedent_hrs p_ah_sl_min_std  p_ah_sb_min_std
##  Min.   :0.0000   Min.   :12.51   Min.   : 726.8   Min.   :  5.16
##  1st Qu.:0.3476   1st Qu.:17.13   1st Qu.: 983.8   1st Qu.: 43.71
##  Median :0.7408   Median :18.69   Median :1076.5   Median : 67.64
##  Mean   :0.9394   Mean   :18.51   Mean   :1068.6   Mean   : 74.27
##  3rd Qu.:1.2726   3rd Qu.:20.15   3rd Qu.:1157.5   3rd Qu.: 97.75
##  Max.   :5.3941   Max.   :23.59   Max.   :1392.8   Max.   :276.80
##  NA's   :211      NA's   :211     NA's   :211      NA's   :211
##  p_ah_light_min_std p_ah_mod_min_std  p_ah_vig_min_std  p_ah_tot_min_std
##  Min.   : 13.93     Min.   :  0.000   Min.   : 0.0000   Min.   :1432
##  1st Qu.:183.93     1st Qu.:  7.913   1st Qu.: 0.0000   1st Qu.:1440
##  Median :260.32     Median : 19.404   Median : 0.0000   Median :1440
```

```
##  Mean   :266.18     Mean   : 29.896   Mean   : 1.0721   Mean   :1440
##  3rd Qu.:336.11     3rd Qu.: 38.911   3rd Qu.: 0.3398   3rd Qu.:1440
##  Max.   :626.82     Max.   :195.958   Max.   :81.4936   Max.   :1440
##  NA's   :211        NA's   :211       NA's   :211       NA's   :211
##  p_ah_sl_hrs_std p_ah_sb_hrs_std  p_ah_light_hrs_std p_ah_mod_hrs_std
##  Min.   :12.11   Min.   :0.0860   Min.   : 0.2322    Min.   :0.0000
##  1st Qu.:16.40   1st Qu.:0.7286   1st Qu.: 3.0655    1st Qu.:0.1319
##  Median :17.94   Median :1.1273   Median : 4.3387    Median :0.3234
##  Mean   :17.81   Mean   :1.2378   Mean   : 4.4364    Mean   :0.4983
##  3rd Qu.:19.29   3rd Qu.:1.6291   3rd Qu.: 5.6018    3rd Qu.:0.6485
##  Max.   :23.21   Max.   :4.6133   Max.   :10.4471    Max.   :3.2660
##  NA's   :211     NA's   :211      NA's   :211        NA's   :211
##  p_ah_vig_hrs_std  p_ah_tot_hrs_std p_ah_mvpa_hrs_std p_ah_sedent_hrs_std
##  Min.   :0.00000   Min.   :23.87    Min.   :0.0000    Min.   :12.83
##  1st Qu.:0.00000   1st Qu.:24.00    1st Qu.:0.1336    1st Qu.:17.56
##  Median :0.00000   Median :24.00    Median :0.3302    Median :19.27
##  Mean   :0.01787   Mean   :24.00    Mean   :0.5161    Mean   :19.05
##  3rd Qu.:0.00566   3rd Qu.:24.00    3rd Qu.:0.6755    3rd Qu.:20.66
##  Max.   :1.35823   Max.   :24.00    Max.   :4.1220    Max.   :23.75
##  NA's   :211       NA's   :211      NA's   :211       NA's   :211
##  p_rpaq_SCORERECREA p_rpaq_SCORE1   p_rpaq_SCORE2    p_rpaq_LIGHT
##  Min.   : 0.000     Min.   :16.00   Min.   :23.20   Min.   :0.0000
##  1st Qu.: 2.917     1st Qu.:20.90   1st Qu.:28.10   1st Qu.:0.0000
##  Median : 5.250     Median :23.57   Median :30.77   Median :0.0000
##  Mean   : 6.993     Mean   :24.47   Mean   :31.67   Mean   :0.5116
##  3rd Qu.: 9.328     3rd Qu.:26.65   3rd Qu.:33.85   3rd Qu.:0.1786
##  Max.   :51.929     Max.   :58.28   Max.   :65.48   Max.   :4.4286
##  NA's   :44         NA's   :44      NA's   :44      NA's   :44
##  p_rpaq_MODERATE  p_rpaq_VIGOR     p_rpaq_SEDENT   p_rpaq_rmrrpaq
##  Min.   :0.000   Min.   :0.00000   Min.   :12.32   Min.   :4288
##  1st Qu.:0.708   1st Qu.:0.00833   1st Qu.:20.32   1st Qu.:5390
##  Median :1.272   Median :0.02222   Median :22.20   Median :6291
##  Mean   :1.741   Mean   :0.15007   Mean   :21.60   Mean   :6296
##  3rd Qu.:2.228   3rd Qu.:0.10714   3rd Qu.:23.09   3rd Qu.:7033
##  Max.   :8.500   Max.   :5.03512   Max.   :24.00   Max.   :9577
##  NA's   :44      NA's   :44        NA's   :44      NA's   :44
##  p_rpaq_PAEEHOME  p_rpaq_PAEEJOB    p_rpaq_PAEETRANSPORT p_rpaq_PAEERECREA
##  Min.   : 106.2   Min.   :    0.0   Min.   :   0.00      Min.   :    0.0
##  1st Qu.: 788.9   1st Qu.:    0.0   1st Qu.:   0.00      1st Qu.:  753.7
##  Median :1141.9   Median :    0.0   Median :   0.00      Median : 1335.7
##  Mean   :1291.4   Mean   :  791.1   Mean   :  84.91      Mean   : 1848.9
##  3rd Qu.:1705.7   3rd Qu.: 1521.7   3rd Qu.:  62.28      3rd Qu.: 2494.5
##  Max.   :4570.8   Max.   :11230.5   Max.   :1900.91      Max.   :16467.7
##  NA's   :44       NA's   :44        NA's   :44           NA's   :44
##   p_rpaq_PAEE      p_rpaq_PAEE_kj_kg_day      vat              sat
##  Min.   :  555.3   Min.   :  9.59       Min.   : 1.315   Min.   : 0.215
##  1st Qu.: 2652.3   1st Qu.: 36.28       1st Qu.: 6.147   1st Qu.: 1.826
##  Median : 3681.8   Median : 46.82       Median : 7.810   Median : 2.482
##  Mean   : 4016.3   Mean   : 50.64       Mean   : 8.184   Mean   : 2.562
##  3rd Qu.: 4939.1   3rd Qu.: 60.31       3rd Qu.: 9.730   3rd Qu.: 3.180
##  Max.   :18690.4   Max.   :195.62       Max.   :21.210   Max.   :10.155
##  NA's   :44        NA's   :44           NA's   :10       NA's   :4
##        id             pro_id          IDX           Glycaemia_Status
##  Min.   :   136   Min.   :10007   Length:746         Length:746
```

```
##  1st Qu.:  8728   1st Qu.:10264   Class :character   Class :character  
##  Median : 17245   Median :10512   Mode  :character   Mode  :character  
##  Mean   : 31177   Mean   :10480                                        
##  3rd Qu.: 57384   3rd Qu.:10706                                        
##  Max.   :178857   Max.   :10897                                        
##                                                                        
##      Risk       sex_cat             BMI_cat      Fat_cat           Waist_cat  
##  Low    :421   Female:339   Underweight  :  3   Length:746         Low   :197  
##  High   :301   Male  :407   Healthyweight:251   Class :character   Medium:220  
##  KDM    : 21                Overweight   :337   Mode  :character   High  :329  
##  unclass:  0                Obese        :155                                  
##  NA's   :  3                UA           :  0                                  
##                                                                                
##                                                                                
##     whratio       whratio_cat          Alcprweek       Alcprweek_cat
##  Min.   :0.6883   Length:746         Min.   : 0.00   Abstinence: 79  
##  1st Qu.:0.8741   Class :character   1st Qu.: 3.00   Moderate  :302  
##  Median :0.9374   Mode  :character   Median : 8.00   High      :262  
##  Mean   :0.9339                      Mean   :10.98   NA's      :103  
##  3rd Qu.:0.9912                      3rd Qu.:16.00                   
##  Max.   :1.3977                      Max.   :54.00                   
##                                      NA's   :103                     
##    Smoking                        bp_cat               sbp_cat   
##  Length:746         Normal           :157   Low            :175  
##  Class :character   Mild_elevated    : 93   Normal         :347  
##  Mode  :character   Moderate_elevated:356   Mild_elevated  :179  
##                     Severely_elevated:138   Moderate_severe: 43  
##                     NA's             :  2   NA's           :  2  
##                                                                  
##                                                                  
##             dbp_cat       hr_cat       pp_cat            trig_cat        
##  Low            :342   Low   :345   Length:746         Length:746        
##  Normal         :267   Medium:344   Class :character   Class :character  
##  Mild_elevated  :103   High  : 55   Mode  :character   Mode  :character  
##  Moderate_severe: 32   NA's  :  2                                        
##  NA's           :  2                                                     
##                                                                          
##                                                                          
##    hdlc_cat           ldlc_cat           chol_cat              act         
##  Length:746         Length:746         Length:746         Min.   : 0.0000  
##  Class :character   Class :character   Class :character   1st Qu.: 0.7864  
##  Mode  :character   Mode  :character   Mode  :character   Median : 1.4261  
##                                                           Mean   : 1.8912  
##                                                           3rd Qu.: 2.4146  
##                                                           Max.   :10.0222  
##                                                           NA's   :44       
##    act_cat     hba1c_cat  
##  Low   :100   Low   :600  
##  Medium:149   Medium:119  
##  High  :453   High  : 26  
##  NA's  : 44   NA's  :  1  
##                           
##                           
##
```

```r
apply(Phe, MARGIN=2, class)
```

```
##             #FAM_ID              IND_ID              FAT_ID
##         "character"         "character"         "character"
##              MOT_ID                 sex                age2
##         "character"         "character"         "character"
##           log10_bmi          p_gv_dob.x              height
##         "character"         "character"         "character"
##              weight                 bmi           impedance
##         "character"         "character"         "character"
##              fat_pc       hours_fasting             fasting
##         "character"         "character"         "character"
##            waist_av              hip_av              sbp_av
##         "character"         "character"         "character"
##              dbp_av               pp_av               hr_av
##         "character"         "character"         "character"
##          crf_pw_sbp          crf_pw_dbp              centre
##         "character"         "character"         "character"
##          pwv_c_mean         pwv_hr_mean          crf_pw_mbp
##         "character"         "character"         "character"
##          crf_pw_map            operator             mean_hr
##         "character"         "character"         "character"
##           mean_c_sp           mean_c_dp           mean_c_pp
##         "character"         "character"         "character"
##      p_gq_high_chol p_gq_high_chol_year p_gq_high_chol_code
##         "character"         "character"         "character"
##        p_gq_high_bp   p_gq_high_bp_year   p_gq_high_bp_code
##         "character"         "character"         "character"
##         p_gq_angina    p_gq_angina_year    p_gq_angina_code
##         "character"         "character"         "character"
##             p_gq_mi        p_gq_mi_year        p_gq_mi_code
##         "character"         "character"         "character"
##         p_gq_stroke    p_gq_stroke_year    p_gq_stroke_code
##         "character"         "character"         "character"
##           p_gq_palp      p_gq_palp_year      p_gq_palp_code
##         "character"         "character"         "character"
##       p_gq_card_sur          p_gq_smoke        p_gq_dia_met
##         "character"         "character"         "character"
##        p_gq_dia_ins        p_gq_dia_sul        p_gq_dia_tzd
##         "character"         "character"         "character"
##        p_gq_dia_oth        p_gq_dia_any        p_gq_dia_num
##         "character"         "character"         "character"
##    p_gq_hyp_ace_arb        p_gq_hyp_bet        p_gq_hyp_cal
##         "character"         "character"         "character"
##        p_gq_hyp_diu        p_gq_hyp_oth        p_gq_hyp_any
##         "character"         "character"         "character"
##        p_gq_hyp_num        p_gq_lip_any        p_gq_lip_sta
##         "character"         "character"         "character"
##            p_gq_asa          target_pop         p_lab_glp1_0
##         "character"         "character"         "character"
##       p_lab_glp1_30      p_lab_glp1_120      p_lab_glucagon_0
##         "character"         "character"         "character"
##   p_lab_glucagon_30  p_lab_glucagon_120         p_lab_gip_0
```

```
##            "character"           "character"           "character"
##            p_lab_gip_30         p_lab_gip_120         auc_glp1_120
##            "character"           "character"           "character"
##            auc_gip_120      auc_glucagon_120        iauc_glp1_120
##            "character"           "character"           "character"
##           iauc_gip_120     iauc_glucagon_120          auc_glp1_30
##            "character"           "character"           "character"
##             auc_gip_30       auc_glucagon_30         iauc_glp1_30
##            "character"           "character"           "character"
##            iauc_gip_30      iauc_glucagon_30     diff_glp1_pct_30
##            "character"           "character"           "character"
##        diff_gip_pct_30  diff_glucagon_pct_30             fup_date
##            "character"           "character"           "character"
##                age_fup                inc_dm             gly_stat
##            "character"           "character"           "character"
##         p_gq_beer_week        p_gq_wine_week    p_gq_liquour_week
##            "character"           "character"           "character"
##        p_gq_occupation  p_gq_occupation_code            provedato
##            "character"           "character"           "character"
##            p_lab_hba1c           p_lab_insu0          p_lab_insu30
##            "character"           "character"           "character"
##          p_lab_insu120           p_lab_pglu0          p_lab_pglu30
##            "character"           "character"           "character"
##          p_lab_pglu120             p_lab_bas           p_lab_alat
##            "character"           "character"           "character"
##            p_lab_creae            p_lab_hdlc           p_lab_trig
##            "character"           "character"           "character"
##             p_lab_chol             p_lab_ldl        p_lab_ualbcre
##            "character"           "character"           "character"
##               p_ah_RMR           p_ah_vo2max           p_ah_Pwear
##            "character"           "character"           "character"
##               p_ah_SHR              p_ah_ACC            p_ah_HRaS
##            "character"           "character"           "character"
##      p_ah_PAEE_Branch4     p_ah_PAEE_Branch7            p_ah_PAEE
##            "character"           "character"           "character"
##            p_ah_sl_min           p_ah_sb_min       p_ah_light_min
##            "character"           "character"           "character"
##           p_ah_mod_min          p_ah_vig_min         p_ah_tot_min
##            "character"           "character"           "character"
##            p_ah_sl_hrs           p_ah_sb_hrs       p_ah_light_hrs
##            "character"           "character"           "character"
##           p_ah_mod_hrs          p_ah_vig_hrs         p_ah_tot_hrs
##            "character"           "character"           "character"
##          p_ah_mvpa_hrs       p_ah_sedent_hrs      p_ah_sl_min_std
##            "character"           "character"           "character"
##        p_ah_sb_min_std    p_ah_light_min_std     p_ah_mod_min_std
##            "character"           "character"           "character"
##       p_ah_vig_min_std      p_ah_tot_min_std      p_ah_sl_hrs_std
##            "character"           "character"           "character"
##        p_ah_sb_hrs_std    p_ah_light_hrs_std     p_ah_mod_hrs_std
##            "character"           "character"           "character"
##       p_ah_vig_hrs_std      p_ah_tot_hrs_std    p_ah_mvpa_hrs_std
##            "character"           "character"           "character"
##    p_ah_sedent_hrs_std    p_rpaq_SCORERECREA        p_rpaq_SCORE1
```

```
##            "character"             "character"             "character"
##          p_rpaq_SCORE2            p_rpaq_LIGHT         p_rpaq_MODERATE
##            "character"             "character"             "character"
##           p_rpaq_VIGOR           p_rpaq_SEDENT          p_rpaq_rmrrpaq
##            "character"             "character"             "character"
##        p_rpaq_PAEEHOME          p_rpaq_PAEEJOB   p_rpaq_PAEETRANSPORT
##            "character"             "character"             "character"
##      p_rpaq_PAEERECREA             p_rpaq_PAEE p_rpaq_PAEE_kj_kg_day
##            "character"             "character"             "character"
##                    vat                     sat                      id
##            "character"             "character"             "character"
##                 pro_id                     IDX       Glycaemia_Status
##            "character"             "character"             "character"
##                   Risk                 sex_cat                 BMI_cat
##            "character"             "character"             "character"
##                Fat_cat               Waist_cat                 whratio
##            "character"             "character"             "character"
##            whratio_cat               Alcprweek           Alcprweek_cat
##            "character"             "character"             "character"
##                Smoking                  bp_cat                 sbp_cat
##            "character"             "character"             "character"
##                dbp_cat                  hr_cat                  pp_cat
##            "character"             "character"             "character"
##               trig_cat                hdlc_cat                ldlc_cat
##            "character"             "character"             "character"
##               chol_cat                     act                 act_cat
##            "character"             "character"             "character"
##              hba1c_cat
##            "character"
```

```r
#########################
#Testing for loops
mosaicplot(table(Phe$sex_cat, Phe$BMI_cat, useNA="always"))
```

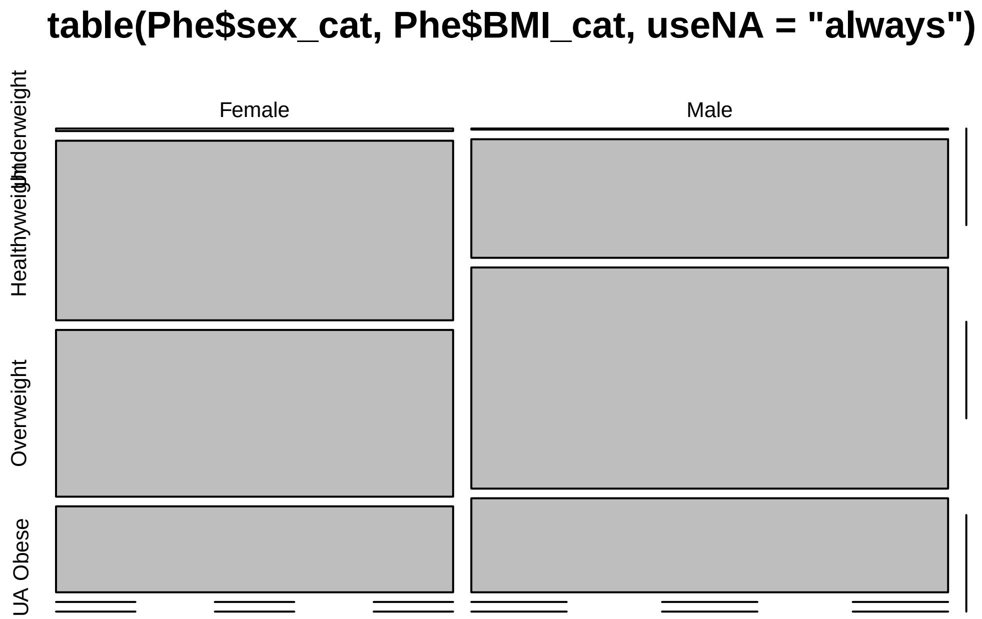


```
ggplot(data = Phe) +
  geom_mosaic(aes(x = product(sex_cat, BMI_cat), fill=sex_cat))
```

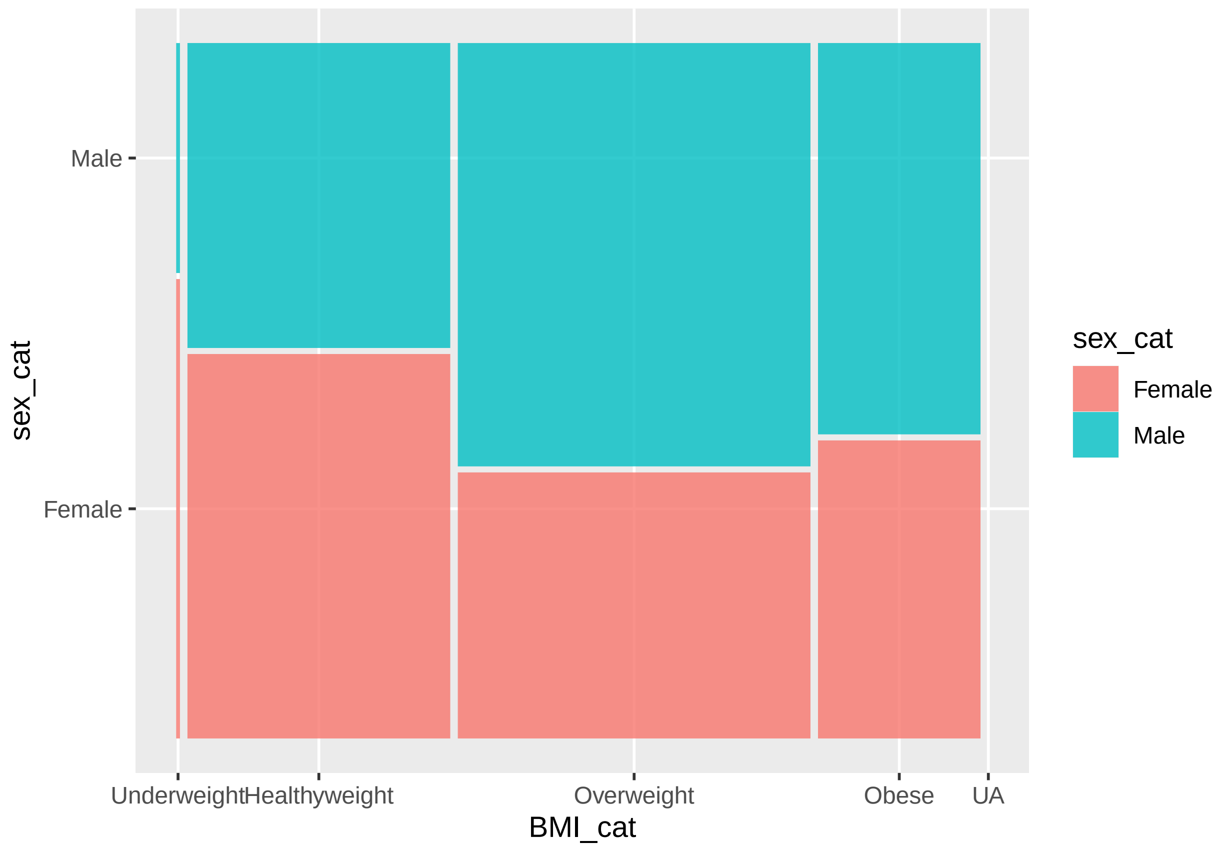


```
chisq.test(Phe$sex_cat, Phe$BMI_cat)
```

```
##
##  Pearson's Chi-squared test
##
## data:  Phe$sex_cat and Phe$BMI_cat
## X-squared = 18.074, df = 3, p-value = 0.0004246
```

```
#fisher.test(table(Phe$sex_cat, Phe$BMI_cat))

mosaicplot(table(Phe$sex_cat, Phe$Fat_cat, useNA="always"))
```

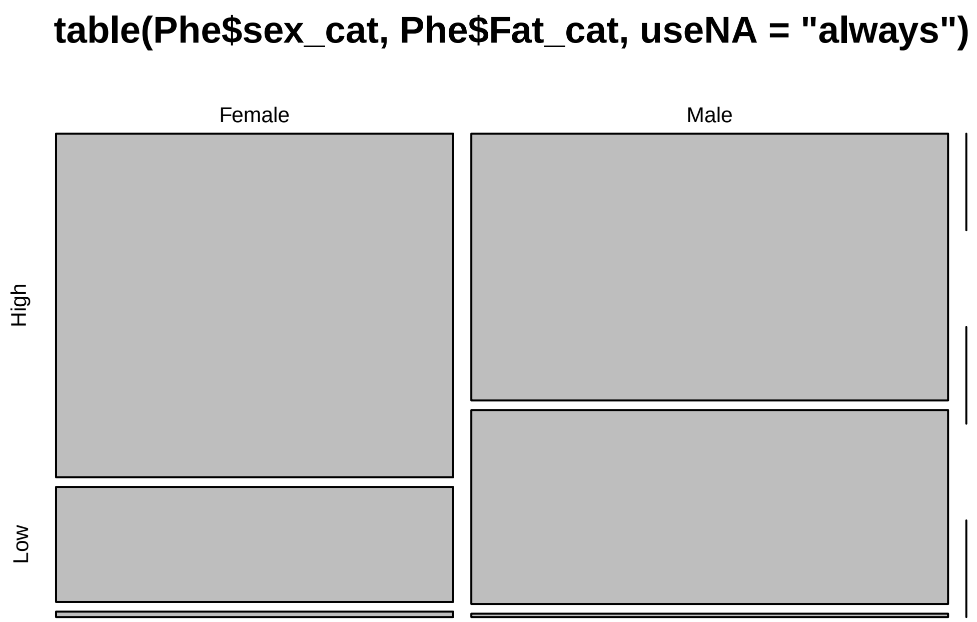


```
ggplot(data = Phe) +
  geom_mosaic(aes(x = product(sex_cat, Fat_cat), fill=sex_cat))
```

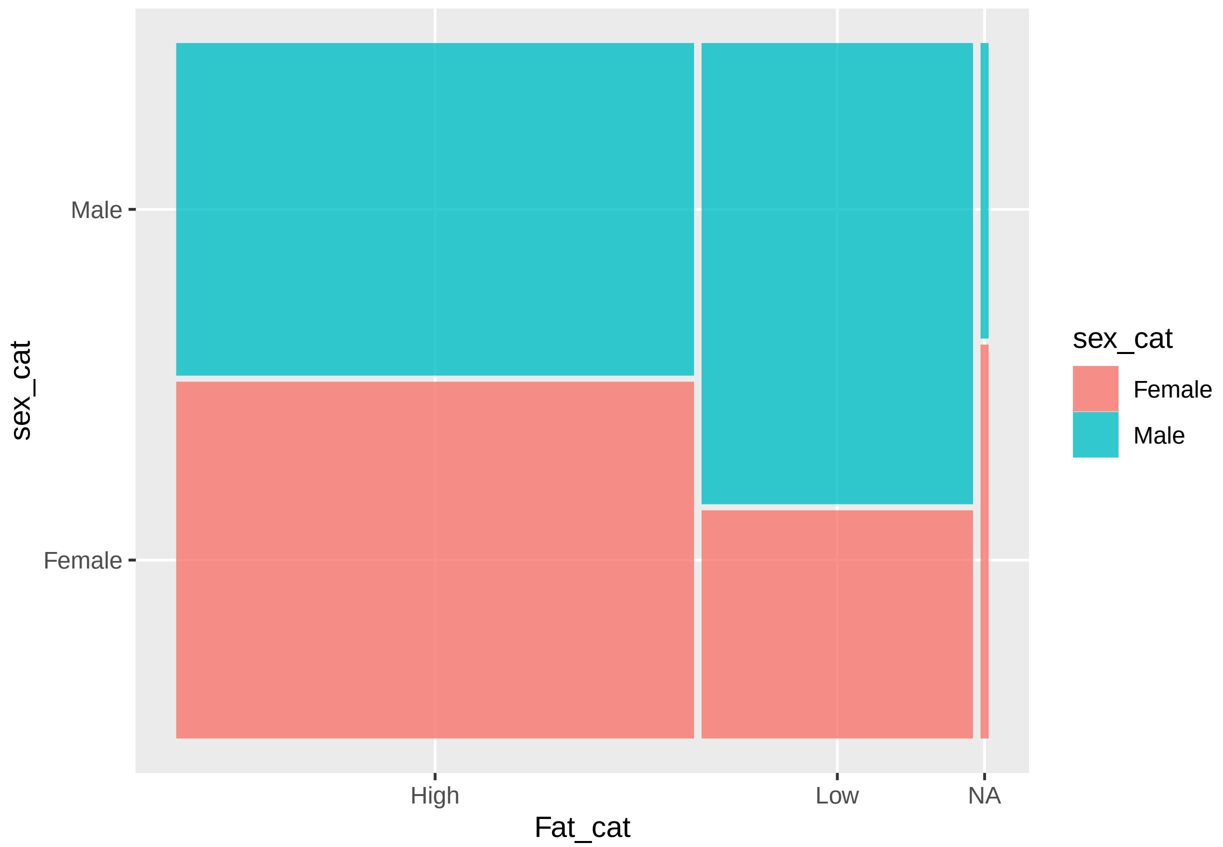


```r
chisq.test(Phe$sex_cat, Phe$Fat_cat)
```

```
##
##  Pearson's Chi-squared test with Yates' continuity correction
##
## data:  Phe$sex_cat and Phe$Fat_cat
## X-squared = 22.728, df = 1, p-value = 1.866e-06
```

```r
#chisq.test(Phe$Fat_cat, Phe$sex_cat) #Get the same
fisher.test(table(Phe$sex_cat, Phe$Fat_cat))
```

```
##
##  Fisher's Exact Test for Count Data
##
## data:  table(Phe$sex_cat, Phe$Fat_cat)
## p-value = 1.315e-06
## alternative hypothesis: true odds ratio is not equal to 1
## 95 percent confidence interval:
##  1.564330 3.020311
## sample estimates:
## odds ratio
##   2.168565
```

```r
mosaicplot(table(Phe$sex_cat, Phe$Smoking, useNA="always"))
```

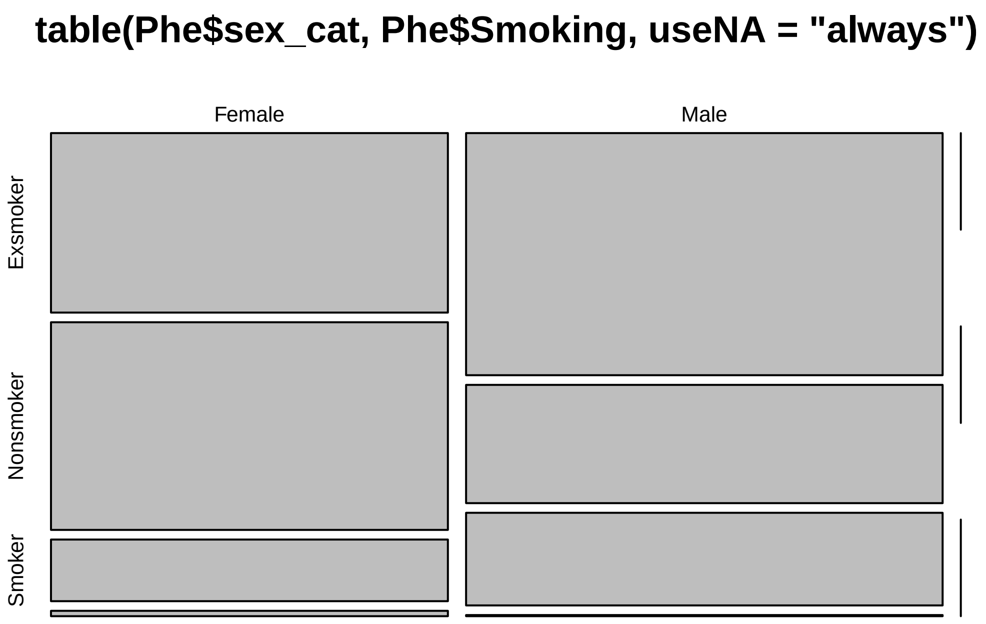


```r
ggplot(data = Phe) +
  geom_mosaic(aes(x = product(sex_cat, Smoking), fill=sex_cat))
```

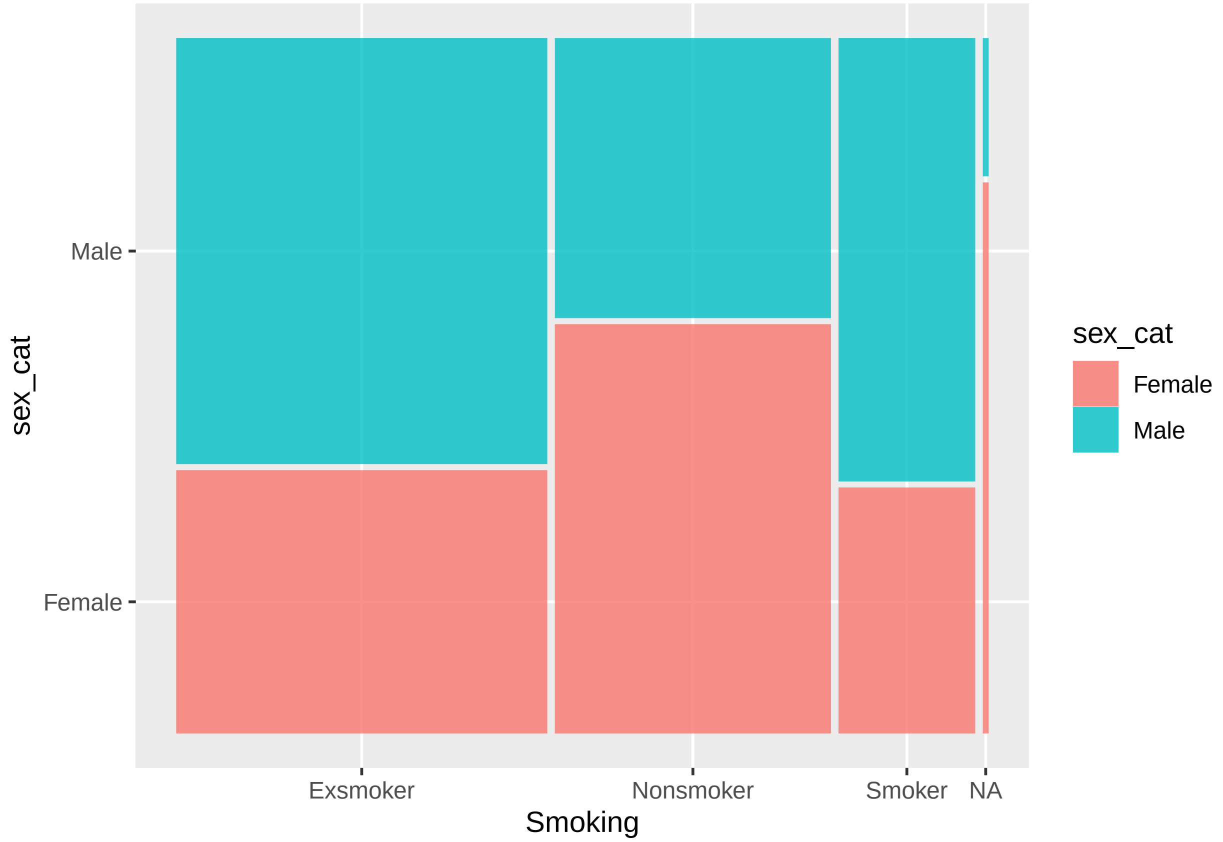


```
chisq.test(Phe$sex_cat, Phe$Smoking)
```

```
##
##  Pearson's Chi-squared test
##
## data:  Phe$sex_cat and Phe$Smoking
## X-squared = 32.938, df = 2, p-value = 7.041e-08
```

```
#chisq.test(Phe$Fat_cat, Phe$sex_cat) #Get the same
fisher.test(table(Phe$sex_cat, Phe$Smoking))
```

```
##
##  Fisher's Exact Test for Count Data
##
## data:  table(Phe$sex_cat, Phe$Smoking)
## p-value = 7.231e-08
## alternative hypothesis: two.sided
```

```
#Phe2<-Phe[!(is.na(Phe$sex_cat)|is.na(Phe$Smoking)),]
#chisq.test(Phe2$sex_cat, Phe2$Smoking) #get the same
#########################


links<-data.frame(matrix(ncol = 4, nrow = 0))
```

```r
colnames(links)<-c("from", "to", "pvalue", "sig")
nodes<-data.frame(matrix(ncol = 3, nrow = 0))
colnames(nodes)<-c("id", "name", "class")

#Corresponding to var in following loops in script
looper<-c("Risk", "sex_cat", "hba1c_cat",
       "BMI_cat", "Waist_cat", "whratio_cat",
       "Alcprweek_cat", "Smoking", "bp_cat",
       "sbp_cat", "dbp_cat", "hr_cat",
       "pp_cat", "trig_cat", "hdlc_cat",
       "ldlc_cat", "chol_cat", "act_cat")

# #Corresponding to var2 in following loops in script
# looper<-c("Glycaemia_Status", "sex_cat",
#        "BMI_cat", "Waist_cat", "whratio_cat",
#        "Smoking", "bp_cat",
#        "sbp_cat", "dbp_cat", "hr_cat",
#        "pp_cat", "trig_cat", "hdlc_cat",
#        "ldlc_cat", "chol_cat", "act_cat",
#        "hba1c_cat")



for (i in looper) {
  nodes<-rbind(nodes, c(paste("s", match(i, names(Phe)), sep=""), i, class(Phe[,match(i, names(Phe))]) )
  for (j in looper) {
    #Consider adding subset of NAs, but chisq.test runs with NA and provides exactly same result
    #Phe2<-Phe[!(is.na(Phe$Smoking)|is.na(Phe$Risk)),] #just example
    #Phe2<-Phe[!(is.na(Phe[,match(i, names(Phe))])|is.na(Phe[,match(j, names(Phe))])),] #So if in eithe

    #print(match(i, names(Phe)))
    #print(match(j, names(Phe)))
    #print(mosaicplot(table(Phe[,c(match(i, names(Phe)), match(j, names(Phe)))], useNA="always")))
    cht<-chisq.test(Phe[,match(i, names(Phe))], Phe[,match(j, names(Phe))])
    links<-rbind(links, c(paste("s", match(i, names(Phe)), sep=""),
                          paste("s", match(j, names(Phe)), sep=""),
                          as.numeric(ifelse(cht$p.value==0, 1e-16, cht$p.value)),
                          cht$p.value<0.05 ) )
  }
}
colnames(nodes)<-c("id", "name", "class")
colnames(links)<-c("from", "to", "pvalue", "sig")

links$pvalue<-as.numeric(links$pvalue)

#Consider getting values more close
links$pvalclose<-ifelse(links$pvalue<1e-3, 1e-3, links$pvalue)
#links$pvalclose<-as.numeric(links$pvalclose)

###########
#Heatmap
links$mlog10pval<- (-log10(links$pvalue))
links$mlog10pvalclose<- (-log10(links$pvalclose))
```

```
net <- graph_from_data_frame(d=links, vertices=nodes, directed=F)
#netm <- get.adjacency(net, attr="mlog10pval", sparse=F)
netm <- get.adjacency(net, attr="mlog10pvalclose", sparse=F)
heatmap(netm, Rowv = NA, Colv = NA,
        scale="none", margins=c(10,10) )
```

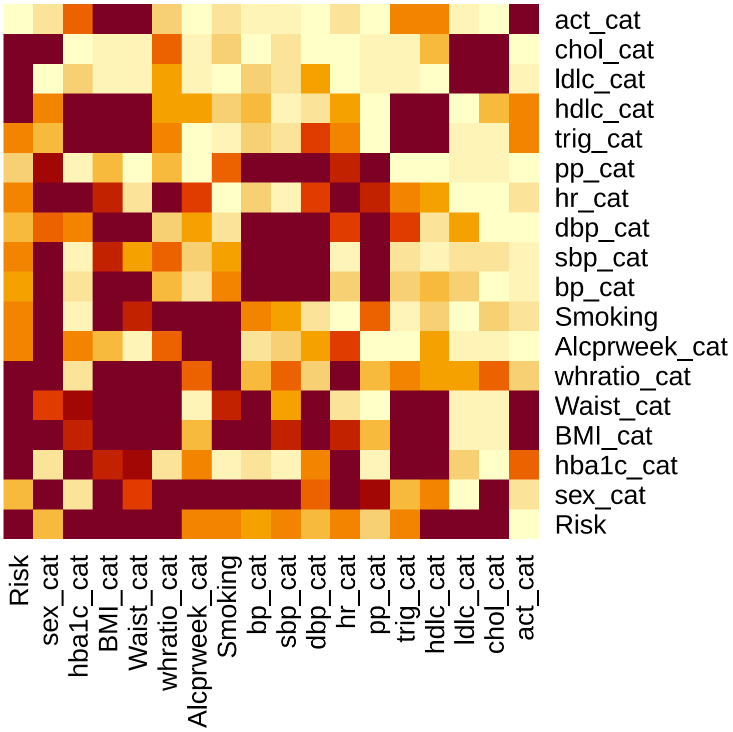


```
library(pheatmap)
pdf("ADDPRO_varheatmap.pdf", width=3.4, height=3)
pheatmap(netm,
         color = colorRampPalette(rev(brewer.pal(n = 7, name ="RdYlBu")))(100),
         margins=c(8,8),
         treeheight_row = 50,
         treeheight_col = 50,
         cellwidth=4,
         cellheight=4,
         fontsize_row=5,
         fontsize_col=5
)
dev.off()
```

```
## pdf
##   3
```

```r
detach('package:pheatmap')
###########

#Remove duplicate rows
links<-links[links$from!=links$to,]

#Remove reversed comparison


#Consider removing non significant edges/links
links<-filter(links, pvalue<0.1)

#Make a column in links representing -log10(pvalue)
links$pvalue<-as.numeric(links$pvalue)
links$pvalclose<-as.numeric(links$pvalclose)
links$mlog10pval<- (-log10(links$pvalue))
links$mlog10pvalclose<- (-log10(links$pvalclose))
#links$scalemlog10pval<-links$mlog10pval/10


detach('package:ggmosaic')
```

Correlation network of sample variables Inspired by https://kateto.net/network-visualization Ognyanova, K. (2021) Network visualization with R. Retrieved from www.kateto.net/network-visualization date: 05072022

```r
head(nodes) #First column id then information on nodes.
```

```
##     id        name     class
## 1 s178        Risk    factor
## 2 s179     sex_cat    factor
## 3 s199   hba1c_cat    factor
## 4 s180     BMI_cat    factor
## 5 s182   Waist_cat    factor
## 6 s184 whratio_cat character
```

```r
head(links) #From (id) to (id) can also have information and size measure.
```

```
##   from   to       pvalue   sig  pvalclose mlog10pval mlog10pvalclose
## 1 s178 s179 6.574492e-02 FALSE 0.06574492   1.182138        1.182138
## 2 s178 s199 4.547017e-18  TRUE 0.00100000  17.342273        3.000000
## 3 s178 s180 5.610627e-06  TRUE 0.00100000   5.250989        3.000000
## 4 s178 s182 3.036695e-07  TRUE 0.00100000   6.517599        3.000000
## 5 s178 s184 8.222591e-05  TRUE 0.00100000   4.084991        3.000000
## 6 s178 s186 2.148222e-02  TRUE 0.02148222   1.667921        1.667921
```

```r
net <- graph_from_data_frame(d=links, vertices=nodes, directed=F)


# Examine the resulting object:
class(net)
```

```
## [1] "igraph"
```

```
net
```

```
## IGRAPH 57b6237 UN-- 18 182 --
## + attr: name (v/c), class (v/c), pvalue (e/n), sig (e/c), pvalclose
## | (e/n), mlog10pval (e/n), mlog10pvalclose (e/n)
## + edges from 57b6237 (vertex names):
##  [1] Risk   --sex_cat       Risk   --hba1c_cat     Risk   --BMI_cat
##  [4] Risk   --Waist_cat     Risk   --whratio_cat   Risk   --Alcprweek_cat
##  [7] Risk   --Smoking       Risk   --bp_cat        Risk   --sbp_cat
## [10] Risk   --dbp_cat       Risk   --hr_cat        Risk   --trig_cat
## [13] Risk   --hdlc_cat      Risk   --ldlc_cat      Risk   --chol_cat
## [16] Risk   --sex_cat       sex_cat--BMI_cat       sex_cat--Waist_cat
## [19] sex_cat--whratio_cat   sex_cat--Alcprweek_cat sex_cat--Smoking
## + ... omitted several edges
```

```
# # We can access the nodes, edges, and their attributes:
# E(net)
# V(net)
# E(net)$pvalue
# E(net)$sig
# V(net)$name
#
# # Or find specific nodes and edges by attribute:
# # (that returns objects of type vertex sequence / edge sequence)
# V(net)[name=="sex_cat"]
# E(net)[sig=="TRUE"]
#
#
# # If you need them, you can extract an edge list
# # or a matrix back from the igraph networks.
# as_edgelist(net, names=T)
# as_adjacency_matrix(net, attr="pvalue")
#
# # Or data frames describing nodes and edges:
# as_data_frame(net, what="edges")
# as_data_frame(net, what="vertices")
#
#
# # You can also look at the network matrix directly:
# net[1,]
# net[1,3]
#
# # First attempt to plot the graph:
# plot(net) # not pretty!
#
# #net <- simplify(net, remove.multiple = T, remove.loops = T) #also removes
# #plot(net)
#
# # Generate colors based on node variable
# colrs <- c("gray50", "tomato", "gold", "red")
# V(net)$color <- colrs[V(net)$name] #Change to class when making other tests
```

```r
# V(net)$color <- c("gray50", "tomato", "gold", "red")
# E(net)$width <- E(net)$mlog10pval
#
# set.seed(99)
# plot(net, edge.curved=.1)


# We can add node labels with geom_node_text() or geom_node_label():
set.seed(99)
ggraph(net,  layout = 'lgl') +
  #geom_edge_arc(strength=0.3, width=1, aes(color = sig, width=mlog10pval)) +
  geom_edge_arc(strength=0.3, aes(color = sig, width=mlog10pval)) +
  scale_edge_width(range = c(0.2, 2)) +
  geom_node_point(color="gray50", size = 2) +
  geom_node_text(aes(label = name), color="black", repel=T) +
  theme_void()
```

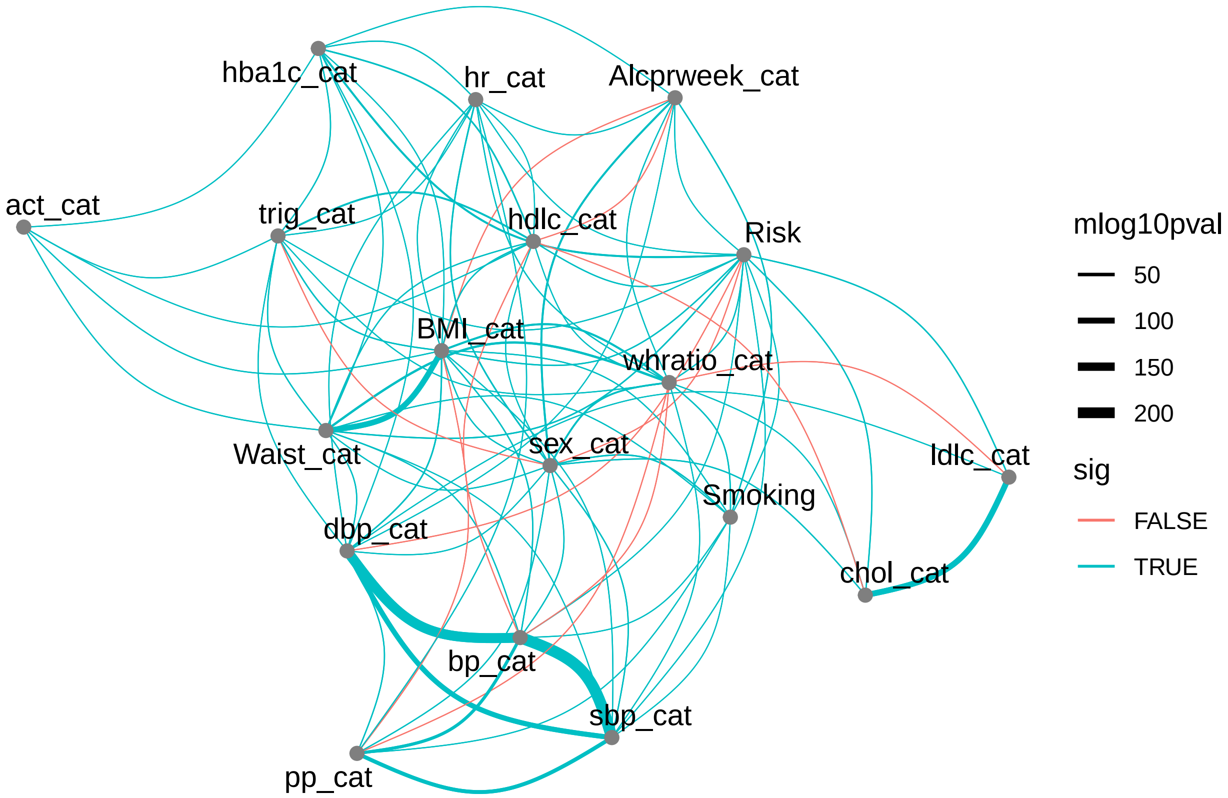


```r
#ggsave("ADDPRO_varnetwork_cat.pdf")

ggraph(net,  layout = 'linear') +
  #geom_edge_arc(strength=0.3, width=1, aes(color = sig, width=mlog10pval)) +
  geom_edge_arc(strength=0.3, aes(color = sig, width=mlog10pval)) +
  scale_edge_width(range = c(0.2, 2)) +
  geom_node_point(color="gray50", size = 2) +
```

```
  geom_node_text(aes(label = name), color="black", repel=T) +
  theme_void()
```

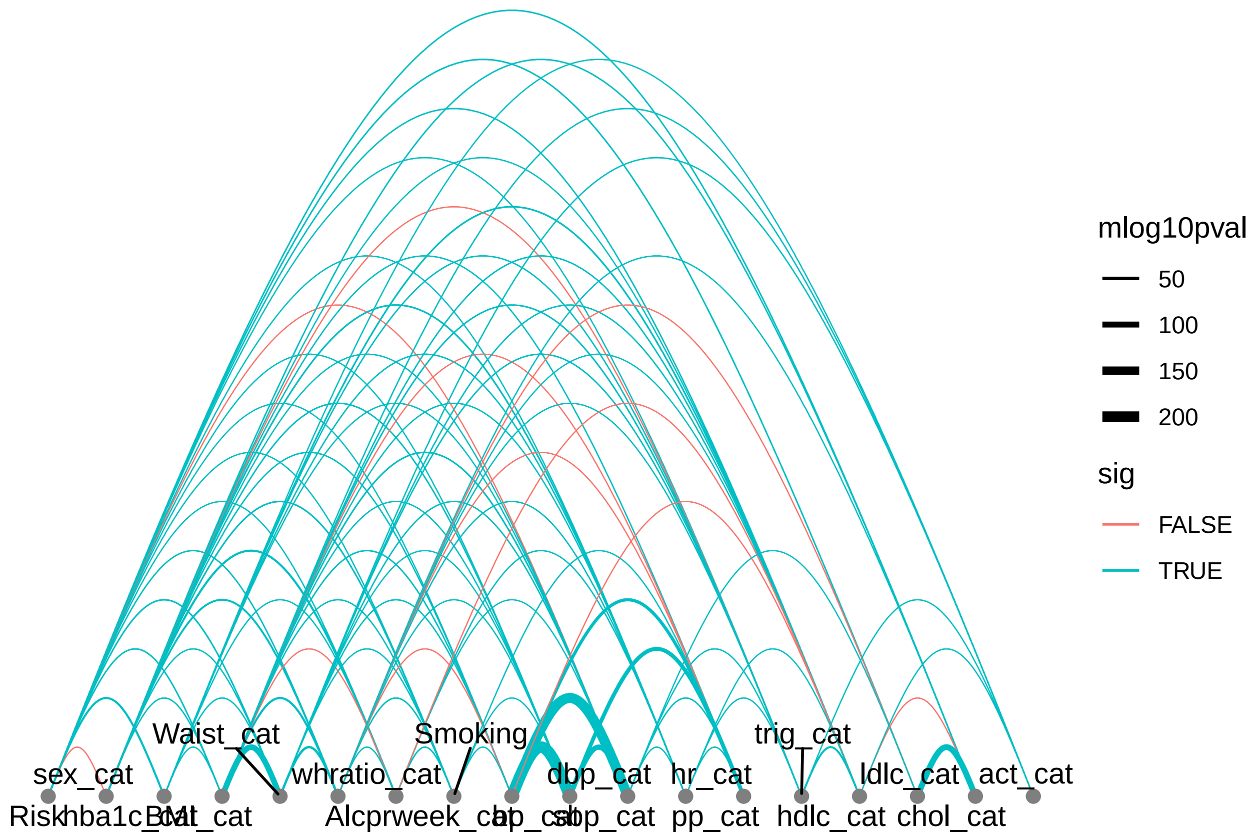


```
# ggraph(net,  layout = 'linear', circular=TRUE) +
#   #geom_edge_arc(strength=0.3, width=1, aes(color = sig, width=mlog10pval)) +
#   geom_edge_arc(strength=0.3, aes(color = sig, width=mlog10pvalclose)) +
#   scale_edge_width(range = c(0.1, 1)) +
#   geom_node_point(color="gray50", size = 2) +
#   geom_node_text(aes(label = name), color="black", repel=T) +
#   theme_void()
# ggsave("ADDPRO_varnetwork_cat_circular.pdf")
```

Stacked bar charts and bubble plots

Inspired by https://jkzorz.github.io/2019/06/05/stacked-bar-plots.html

```
rm(list=setdiff(ls(), c("FeaturePic2", "Pic2", "Phe", "Microbio", "FeatureMic", "Metabo", "paretoscale"

#Phenotype selector
selector<-c("gly_stat")

##Implement aggregation to higher taxonomic rank
#Changing names of low abundant to other
Ninclude <- 10
```

```r
#Most abundant organisms
Mostabun <- row.names(rankabundance(t(Microbio)))[1:Ninclude]
#Add row names to df
#Maks TSS
Taxonomy2<-sweep(Microbio, 2, colSums(Microbio), FUN="/")
#Taxonomy2<-Microbio

Taxonomy2$TaxID <- row.names(Taxonomy2)
Taxonomy2$TaxID2 <-
  ifelse(Taxonomy2$TaxID==Mostabun[1], Mostabun[1],
         ifelse(Taxonomy2$TaxID==Mostabun[2], Mostabun[2],
                ifelse(Taxonomy2$TaxID==Mostabun[3], Mostabun[3],
                       ifelse(Taxonomy2$TaxID==Mostabun[4],
                              Mostabun[4],
                              ifelse(Taxonomy2$TaxID==Mostabun[5],
                                     Mostabun[5],
                                     ifelse(Taxonomy2$TaxID==Mostabun[6],
                                            Mostabun[6],
                                            ifelse(Taxonomy2$TaxID==Mostabun[7],
                                                   Mostabun[7],
                                                   ifelse(Taxonomy2$TaxID==Mostabun[8],
                                                          Mostabun[8],
                                                          ifelse(Taxonomy2$TaxID==
                                                                   Mostabun[9],
                                                                 Mostabun[9],
                                                                 ifelse(Taxonomy2$TaxID==
                                                                          Mostabun[10],
                                                                        Mostabun[10],
                                                                        "other"))))))))))

#Does not work
#Taxonomy$TaxID2 <-
#  ifelse(for (i in Ninclude) {Taxonomy$TaxID==Mostabun[i], Mostabun[i]}, "other")


##Then need to aggregate
Taxonomy2<-Taxonomy2 %>% group_by(TaxID2) %>%
  summarise_if(is.numeric, funs(sum))
#colSums(Taxonomy2[,2:length(Taxonomy2)])

##Long format
row.names(Taxonomy2)<-Taxonomy2$TaxID2
Taxonomy2 <- data.frame(t(Taxonomy2))
Taxonomy2<-Taxonomy2[-c(1), ]
#Add row names to df
Taxonomy2$IDX <- row.names(Taxonomy2)
##Select phenotypes
Phe2<-Phe
#table(Phe2$Smoking)
Phe2<-dplyr::select(Phe2, one_of(c("IDX", selector)))
Phe2<-merge(Phe2, Taxonomy2, by="IDX")
Phe2<-dplyr::select(Phe2, -one_of(c("IDX")))
```

```r
Phe2 <- data.frame(sapply(Phe2, as.numeric))
Phe2$gly_stat<-as.factor(Phe2$gly_stat)

##If columns are factors have only got this manual conversion to work
##Consequently these can also change
#colnames(Phe2)
##Phe2$Actinomyces<-as.numeric(levels(Phe2$Actinomyces)[Phe2$Actinomyces])
#Phe2$Fusobacterium<-as.numeric(levels(Phe2$Fusobacterium)[Phe2$Fusobacterium])
#Phe2$Haemophilus<-as.numeric(levels(Phe2$Haemophilus)[Phe2$Haemophilus])
#Phe2$Leptotrichia<-as.numeric(levels(Phe2$Leptotrichia)[Phe2$Leptotrichia])
#Phe2$Neisseria<-as.numeric(levels(Phe2$Neisseria)[Phe2$Neisseria])
#Phe2$other<-as.numeric(levels(Phe2$other)[Phe2$other])
#Phe2$Porphyromonas<-as.numeric(levels(Phe2$Porphyromonas)[Phe2$Porphyromonas])
#Phe2$Prevotella<-as.numeric(levels(Phe2$Prevotella)[Phe2$Prevotella])
#Phe2$Rothia<-as.numeric(levels(Phe2$Rothia)[Phe2$Rothia])
#Phe2$Streptococcus<-as.numeric(levels(Phe2$Streptococcus)[Phe2$Streptococcus])
#Phe2$Veillonella<-as.numeric(levels(Phe2$Veillonella)[Phe2$Veillonella])

#rowSums(Phe2[, -1])
Phe2<-aggregate(formula(paste(".", "~ ", selector)), Phe2, mean)
#rowSums(Phe22[, -1])



Phe2<-melt(Phe2, id=c(selector))
#Phe2<-dplyr::select(Phe2, -one_of(c("IDX")))

#define the colours to use in the figure
colours = c( "#A54657",  "#582630", "#F7EE7F", "#4DAA57","#F1A66A","#F26157",
             "#F9ECCC", "#679289", "#33658A", "#F6AE2D","#86BBD8")

#Define order of selector in plot
##Smoking
#Phe2$Smoking <- factor(Phe2$Smoking,levels=c("Nonsmoker", "Exsmoker", "Smoker", "UA"))
##gly_stat
#Don't need to define right order

#make the plot!
barp<-ggplot(Phe2, aes_string(x = selector, fill = "variable", y = "value")) +
    geom_bar(stat = "identity", colour = "black") +
    theme(axis.text.x = element_text(angle = 90, size = 14, colour = "black", vjust = 0.5,
                                     hjust = 1, face= "bold"),
    axis.title.y = element_text(size = 16, face = "bold"),
    legend.title = element_text(size = 16, face = "bold"),
    legend.text = element_text(size = 12, face = "bold", colour = "black"),
    axis.text.y = element_text(colour = "black", size = 12, face = "bold")) +
    scale_y_continuous(expand = c(0,0)) +
    labs(x = "", y = "Relative Abundance (%)", fill = "Genus") +
    scale_fill_manual(values = colours)

barp
```

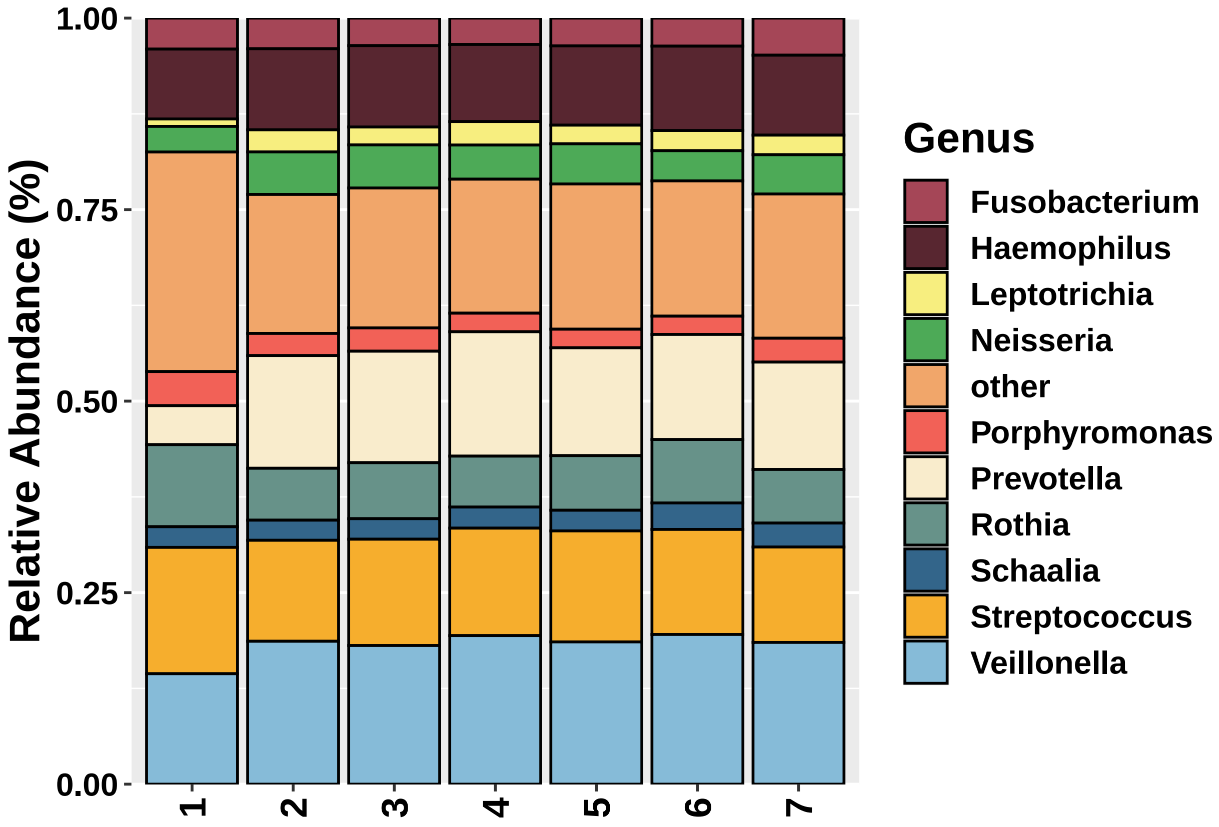


```r
#ggsave("ADDPRO_Microbio_smoke_stackbarrel.pdf")
#ggsave("ADDPRO_Microbio_glystat_stackbarrel.pdf")

#Make bubble plot
Phe2$value<-Phe2$value*100
dotmax<-max(Phe2$value)+1
bubp = ggplot(Phe2, aes_string(x = selector, fill = "Smoking", y = "variable")) +
  geom_point(aes(size = value, fill = variable), alpha = 0.75, shape = 21) +
  scale_size_continuous(limits = c(0.000001, dotmax), range = c(1,10),
                        breaks = c(1,3,9,18)) +
  labs( x= "", y = "", size = "Relative Abundance (%)", fill = "")  +
  theme(legend.key=element_blank(),
  axis.text.x = element_text(colour = "black", size = 12, face = "bold", angle = 90,
                             vjust = 0.3, hjust = 1),
  axis.text.y = element_text(colour = "black", face = "bold", size = 11),
  legend.text = element_text(size = 10, face ="bold", colour ="black"),
  legend.title = element_text(size = 12, face = "bold"),
  panel.background = element_blank(), panel.border = element_rect(colour = "black",
                                                                  fill = NA, size = 1.2),
  legend.position = "right") +
  scale_fill_manual(values = colours, guide = FALSE) +
  scale_y_discrete(limits = rev(levels(Phe2$variable)))

bubp
```

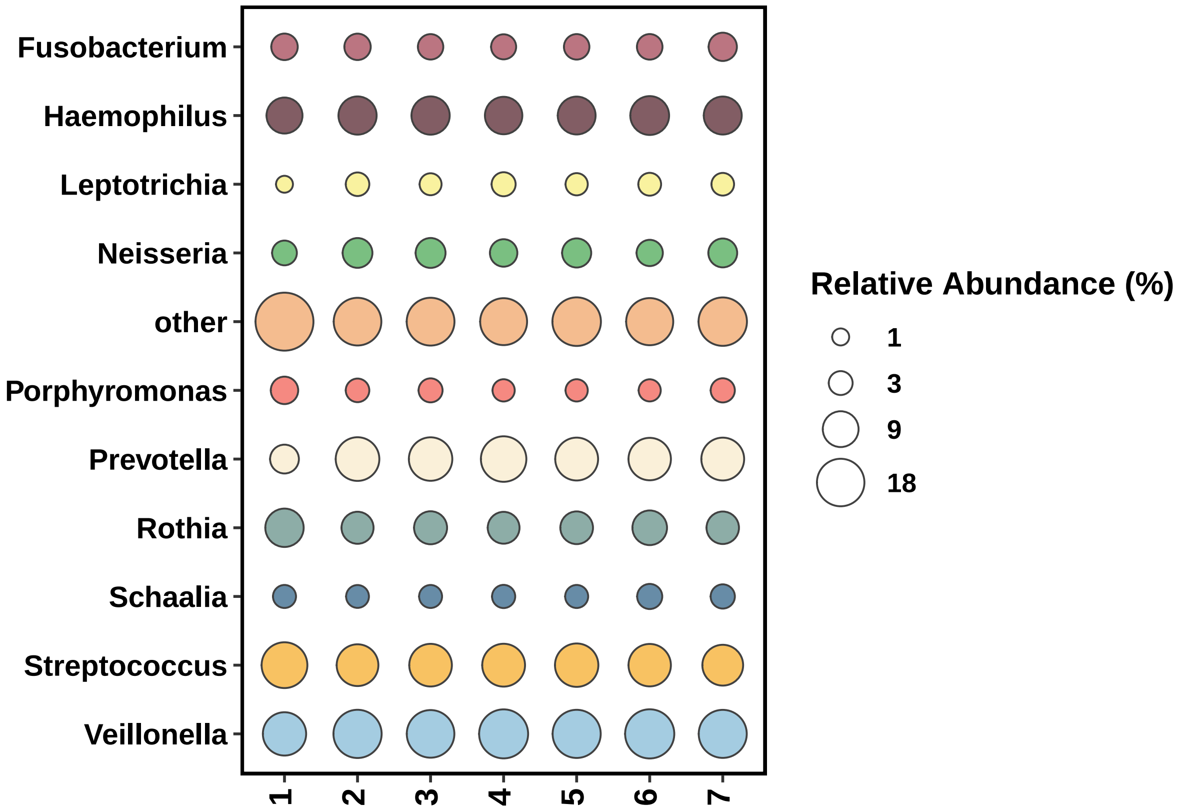


```
#ggsave("ADDPRO_Microbio_smoke_bubbleperc.pdf")
#ggsave("ADDPRO_Microbio_glystat_bubbleperc.pdf")
```

Alpha diversity

```
rm(list=setdiff(ls(), c("FeaturePic2", "Pic2", "Phe", "Microbio", "FeatureMic", "Metabo", "paretoscale"
#Calculate alpha diversity stats
#Use vegan to calculate various diversity and richness indices for each sample
set.seed(1)
diversityCalc <- data.frame(Shannon=vegan::diversity(t(Microbio), index="shannon"),
                            Simpson=vegan::diversity(t(Microbio), index="simpson"),
                            invSimpson=vegan::diversity(t(Microbio), index="invsimpson"),
                            fisher=fisher.alpha(t(Microbio)),
                            #richness=specnumber(t(Microbio)),
                            richness=specnumber(rrarefy(data.frame(t(Microbio)),
                                                         min(rowSums(t(Microbio))))),
                            rarefy_min_count=rarefy(t(Microbio),
                                                     sample=min(rowSums(t(Microbio)))),
                            chao1=estimateR(t(Microbio))["S.chao1",],
                            chao1SE=estimateR(t(Microbio))["se.chao1",],
                            ShannonRar=vegan::diversity(rrarefy(data.frame(t(Microbio)),
                                                          min(rowSums(t(Microbio)))),
```

```r
                                           index="shannon"),
                            SimpsonRar=vegan::diversity(rrarefy(data.frame(t(Microbio)),
                                                   min(rowSums(t(Microbio)))),
                                           index="simpson"),
                            invSimpsonRar=vegan::diversity(rrarefy(data.frame(t(Microbio)),
                                                      min(rowSums(t(Microbio)))),
                                              index="invsimpson"),
                            #Pielou=vegan::diversity(t(Microbio))/log(specnumber(t(Microbio)))
                            Pielou=vegan::diversity(rrarefy(data.frame(t(Microbio)),
                                                  min(rowSums(t(Microbio)))))/
                              log(specnumber(rrarefy(data.frame(t(Microbio)),
                                               min(rowSums(t(Microbio)))))))
#Merge with Phe.
diversityCalc<-add_rownames(diversityCalc, "IDX")
Phe2 <- merge(Phe, diversityCalc, by="IDX")


# #Create a list to hold the plot objects.
AlphaList <- list()
# #Create vector to loop
# AlphaDiv<-c("fisher", "Shannon", "Simpson", "invSimpson", "richness", "chao1",
#             "ShannonRar", "SimpsonRar", "invSimpsonRar", "Pielou")
# #Plot
# for (i in AlphaDiv) {
# #Create plot name
# pltName <- paste('Alpha', i, sep = '')
# #create boxplots
# AlphaList[[ pltName ]]<-
# ggplot(Phe2, aes_string(x="gly_stat", y=i, group="gly_stat")) +
#     geom_violin(aes(fill=gly_stat, trim=FALSE)) +
#     stat_summary(fun.data="mean_sdl",
#                  mult=1, #mean plus minus a constant (mult=1) times the st.dev
#                  geom="pointrange",
#                  width=0.2 ) +
#     #stat_summary(fun.y = mean, geom = "point") +
#     #facet_grid(. ~ Metformin, scales="fixed") + #Scales can also be "free"
#     #ggtitle(paste("Genus", i, sep=" ")) +
#     #xlab("gly_stat") +
#     ylab(paste(i)) +
#     scale_fill_manual(values=c("1"="#0000FF","2"="#FF0000", "3"="#228B22",
#                                "4"="#FFD700", "5"="#800080")) +
#     theme_bw() +
#     theme(legend.position="none", panel.grid.major = element_blank(),
#           panel.grid.minor = element_blank(), axis.title=element_text(size=20),
#           axis.title.x = element_blank(),
#           axis.text.x = element_text(angle = 45, hjust = 1, size=16),
#           axis.text.y = element_text(angle = 45, hjust = 1, size=12))
# }
#
#
# #Have the plots stored in lists
# lay <- rbind(c(1,2,3))
# pdf(paste("ADDPRO_Microbio_Alpha", ".pdf", sep=""), width=12, height=6)
```

```r
# grid.arrange(AlphaList$Alpharichness,
#              AlphaList$AlphaPielou, AlphaList$AlphaShannon, layout_matrix = lay)
# dev.off()
#
# grid.arrange(AlphaList$Alpharichness,
#              AlphaList$AlphaPielou, AlphaList$AlphaShannon, layout_matrix = lay)
#
#
# #Kruskal test
# kruskal.test(richness ~ gly_stat, data=Phe2)
# kruskal.test(Pielou ~ gly_stat, data=Phe2)
# kruskal.test(Shannon ~ gly_stat, data=Phe2)
# #If significant Mann-Whitney pairwise post-test
# pairwise.wilcox.test(Phe2$richness, Phe2$gly_stat,
#                      p.adjust.method="bonferroni")
# pairwise.wilcox.test(Phe2$Pielou, Phe2$gly_stat,
#                      p.adjust.method="bonferroni")
# pairwise.wilcox.test(Phe2$Shannon, Phe2$gly_stat,
#                      p.adjust.method="bonferroni")



############## New ####################
##For all variables
#readRDS("dada2/phyloseq.RDS") -> ps
#ps
#sample_data(ps)<-cbind(sample_data(ps),
#                       estimate_richness(ps,
#                                         measures=c("Observed", "Chao1", "ACE", "Shannon", "Simpson",
#alpha<-data.frame(sample_data(ps))
##Add row names to df
#alpha$IDX <- paste("X", row.names(alpha), sep="")
##Merge according to Sample
#alpha<-merge(Phe2, alpha, by="IDX")

var<-c("Risk", "gly_stat", "sex_cat",
       "BMI_cat", "Waist_cat", "whratio_cat",
       "Alcprweek_cat", "Smoking", "bp_cat",
       "sbp_cat", "dbp_cat", "hr_cat",
       "pp_cat", "trig_cat", "hdlc_cat",
       "ldlc_cat", "chol_cat", "act_cat",
       "Glycaemia_Status", "hba1c_cat")

#Plot
for (i in var) {
#Create plot name
pltName <- paste('Alpharichness', i, sep = '')
#create boxplots
AlphaList[[ pltName ]]<- ggplot(Phe2, aes_string(x=i, y="richness", group=i, color=i, fill=i)) +
    geom_violin() +
    stat_summary(fun.data="mean_sdl",
                 mult=1, #mean plus minus a constant (mult=1) times the st.dev
                 geom="pointrange",
```

```
                 width=0.2 ) +
    stat_summary(fun.y = mean, geom = "point") +
    stat_compare_means(method="anova") +
    ggtitle("Richness") +
    ylab(paste(i)) +
    scale_color_brewer(palette="Dark2") +
    theme_bw() +
    theme(legend.position="none", panel.grid.major = element_blank(),
          panel.grid.minor = element_blank(), axis.title=element_text(size=20),
          axis.title.x = element_blank(),
          axis.text.x = element_text(angle = 45, hjust = 1, size=16),
          axis.text.y = element_text(angle = 45, hjust = 1, size=12))

print(ggplot(Phe2, aes_string(x=i, y="richness", group=i, color=i, fill=i)) +
    geom_violin() +
    stat_summary(fun.data="mean_sdl",
                 mult=1, #mean plus minus a constant (mult=1) times the st.dev
                 geom="pointrange",
                 width=0.2 ) +
    stat_summary(fun.y = mean, geom = "point") +
    stat_compare_means(method="anova") +
    #ggtitle(i) +
    ylab(paste(i)) +
    scale_color_brewer(palette="Dark2") +
    theme_bw() +
    theme(legend.position="none", panel.grid.major = element_blank(),
          panel.grid.minor = element_blank(), axis.title=element_text(size=20),
          axis.title.x = element_blank(),
          axis.text.x = element_text(angle = 45, hjust = 1, size=16),
          axis.text.y = element_text(angle = 45, hjust = 1, size=12)))


print(kruskal.test(formula(paste("richness ~", i)), data=Phe2))
print(pairwise.wilcox.test(Phe2$richness, Phe2[,which( colnames(Phe2) %in% i )],
                      p.adjust.method="bonferroni")) #If variable binary result same as kruskal
}
```

Microbiome

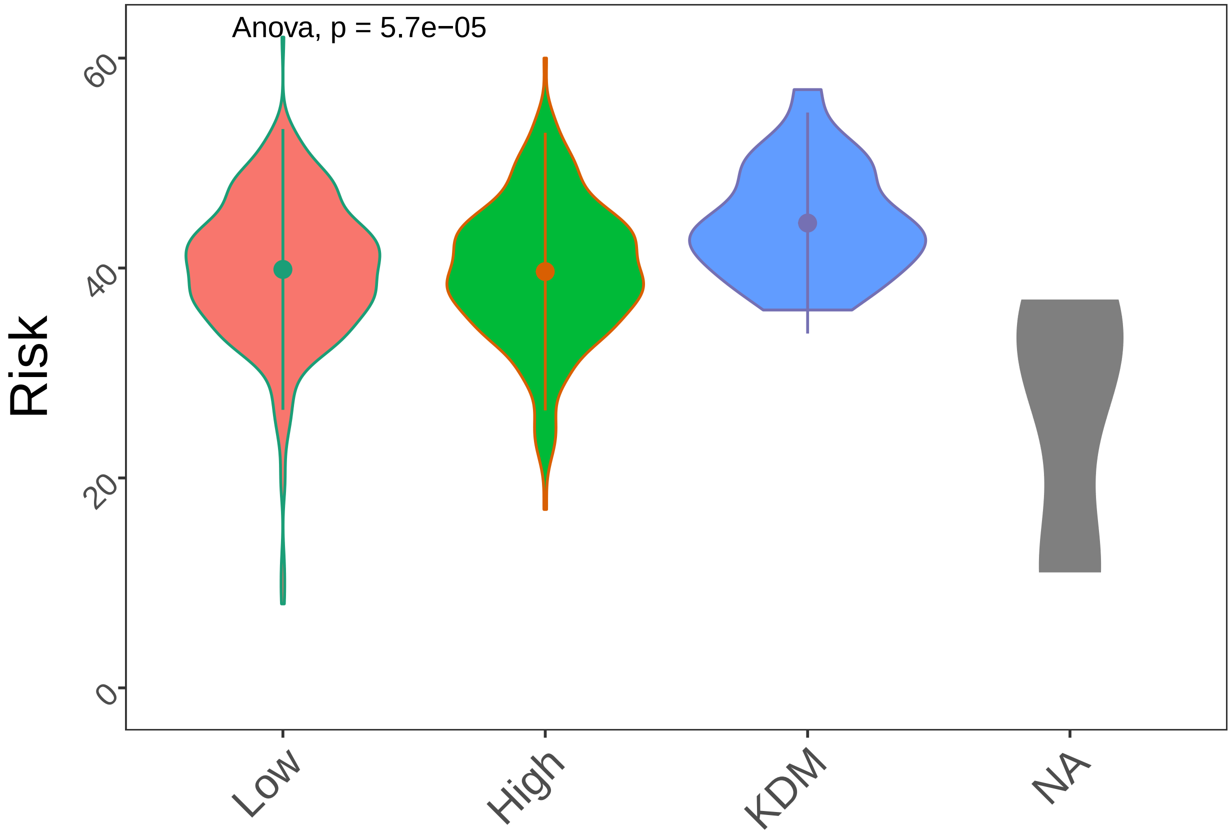


```
##
##  Kruskal-Wallis rank sum test
##
## data:  richness by Risk
## Kruskal-Wallis chi-squared = 9.9575, df = 2, p-value = 0.006883
##
##
##  Pairwise comparisons using Wilcoxon rank sum test with continuity correction
##
## data:  Phe2$richness and Phe2[, which(colnames(Phe2) %in% i)]
##
##      Low    High
## High 1.0000 -
## KDM  0.0077 0.0055
##
## P value adjustment method: bonferroni
```

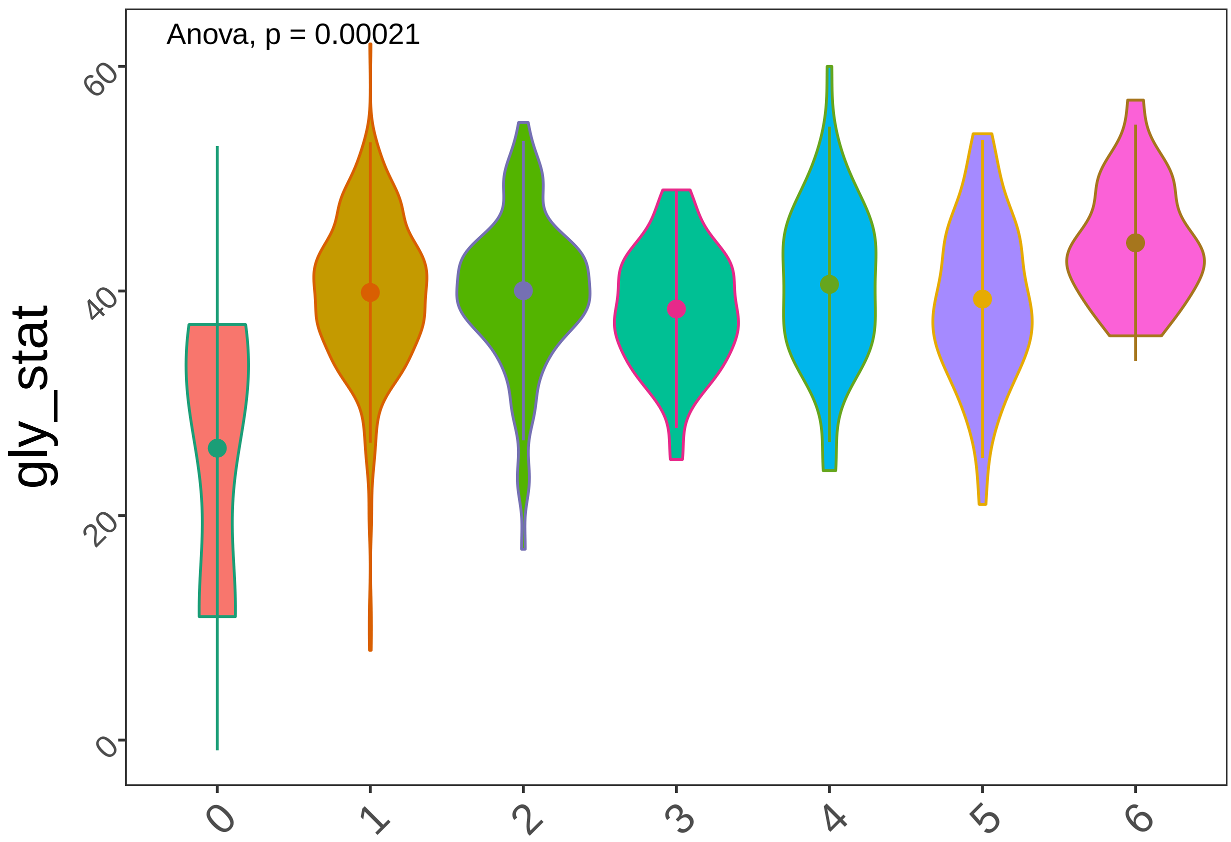


```
##
##  Kruskal-Wallis rank sum test
##
## data:  richness by gly_stat
## Kruskal-Wallis chi-squared = 20.203, df = 6, p-value = 0.002549
##
##
##  Pairwise comparisons using Wilcoxon rank sum test with continuity correction
##
## data:  Phe2$richness and Phe2[, which(colnames(Phe2) %in% i)]
##
##   0      1      2      3      4      5
## 1 0.5181 -      -      -      -      -
## 2 0.4678 1.0000 -      -      -      -
## 3 0.9045 1.0000 0.9178 -      -      -
## 4 0.6492 1.0000 1.0000 1.0000 -      -
## 5 0.9792 1.0000 1.0000 1.0000 1.0000 -
## 6 0.1793 0.0540 0.1432 0.0021 0.7120 0.1020
##
## P value adjustment method: bonferroni
```

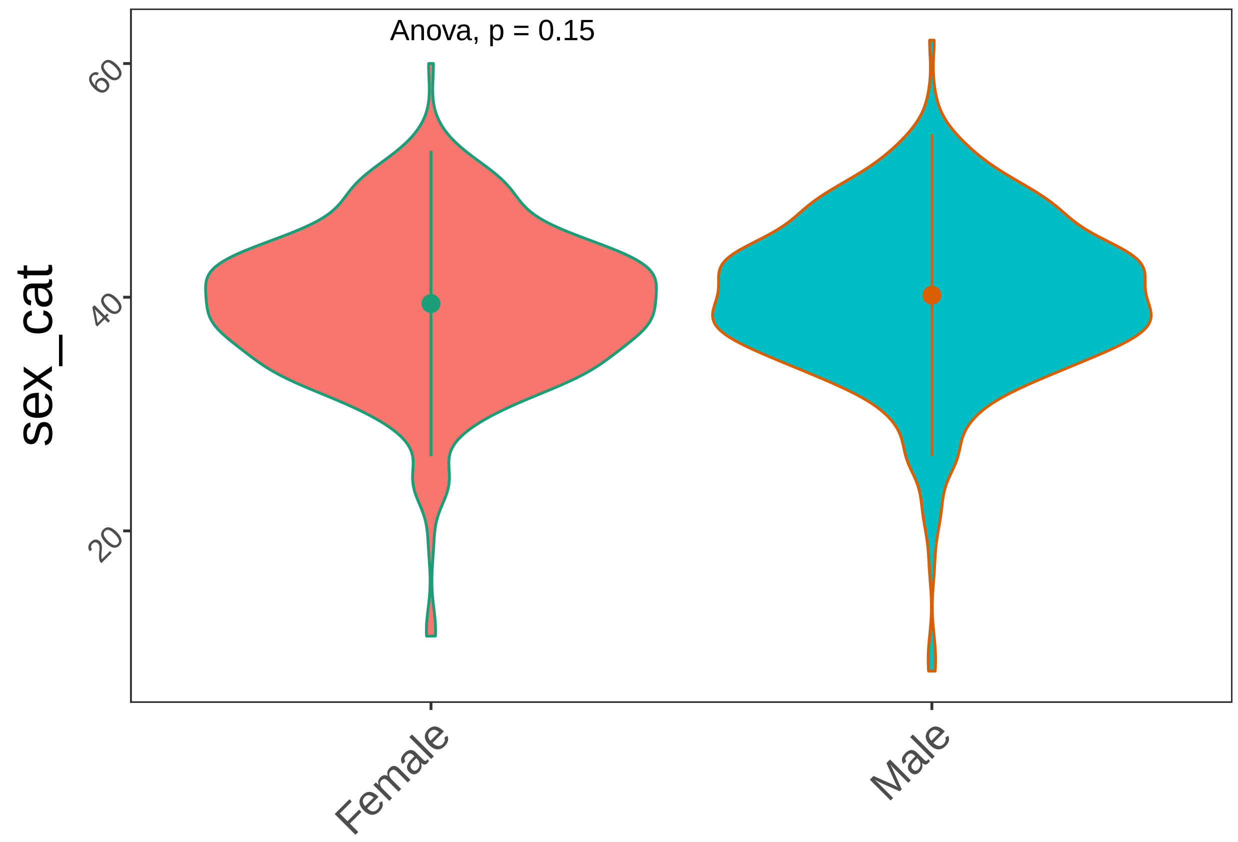


```
##
##  Kruskal-Wallis rank sum test
##
## data:  richness by sex_cat
## Kruskal-Wallis chi-squared = 2.8634, df = 1, p-value = 0.09062
##
##
##  Pairwise comparisons using Wilcoxon rank sum test with continuity correction
##
## data:  Phe2$richness and Phe2[, which(colnames(Phe2) %in% i)]
##
##      Female
## Male 0.091
##
## P value adjustment method: bonferroni
```

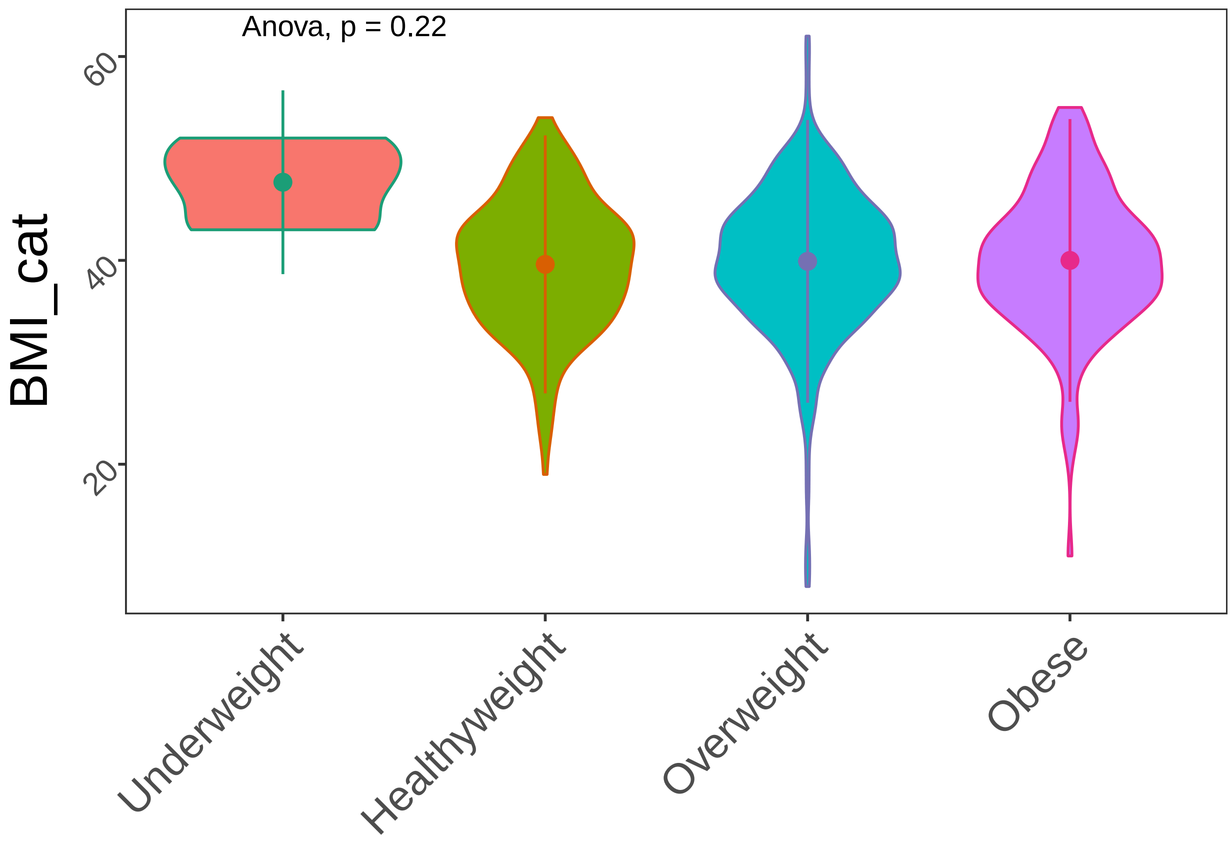


```
##
##  Kruskal-Wallis rank sum test
##
## data:  richness by BMI_cat
## Kruskal-Wallis chi-squared = 5.0888, df = 3, p-value = 0.1654
##
##
##  Pairwise comparisons using Wilcoxon rank sum test with continuity correction
##
## data:  Phe2$richness and Phe2[, which(colnames(Phe2) %in% i)]
##
##               Underweight Healthyweight Overweight
## Healthyweight 0.19        -             -
## Overweight    0.23        1.00          -
## Obese         0.28        1.00          1.00
##
## P value adjustment method: bonferroni
```

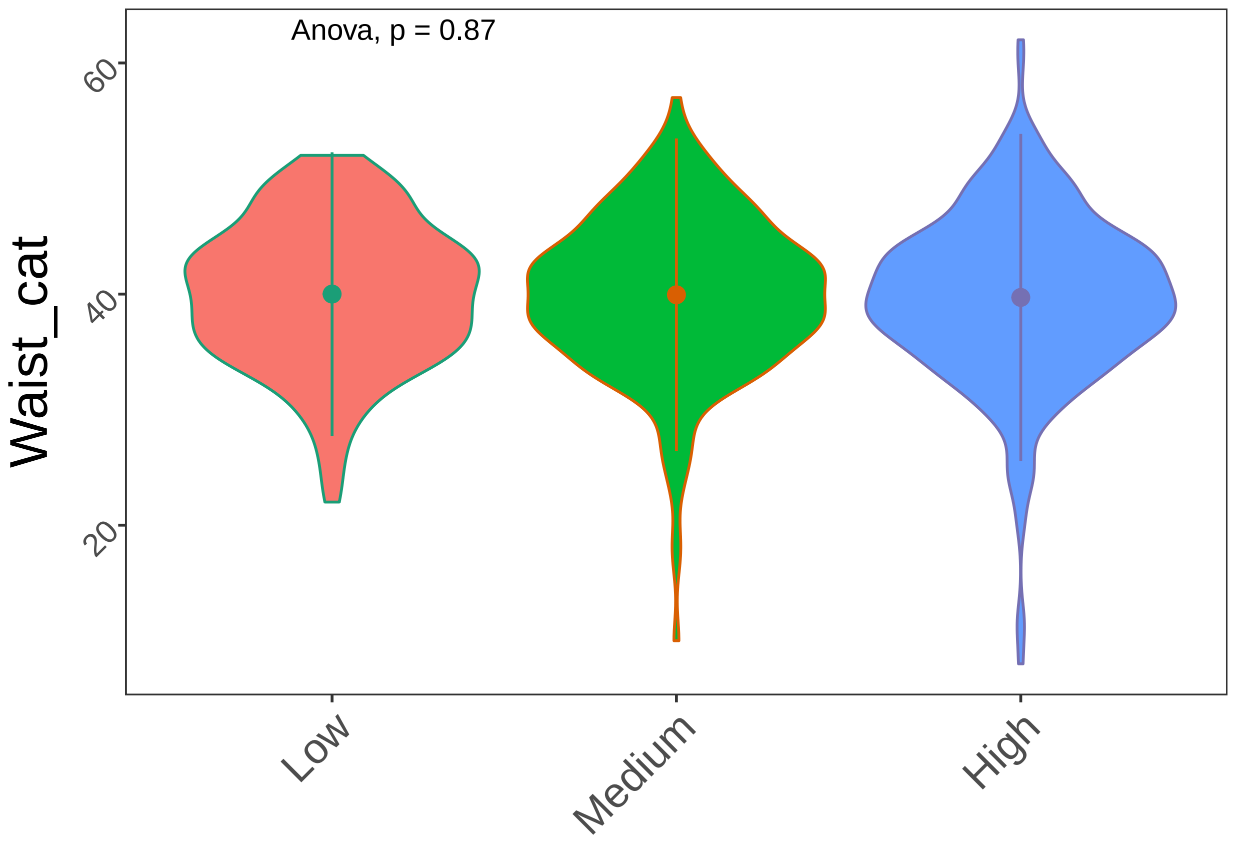


```
##
##  Kruskal-Wallis rank sum test
##
## data:  richness by Waist_cat
## Kruskal-Wallis chi-squared = 0.21195, df = 2, p-value = 0.8994
##
##
##  Pairwise comparisons using Wilcoxon rank sum test with continuity correction
##
## data:  Phe2$richness and Phe2[, which(colnames(Phe2) %in% i)]
##
##        Low Medium
## Medium 1   -
## High   1   1
##
## P value adjustment method: bonferroni
```

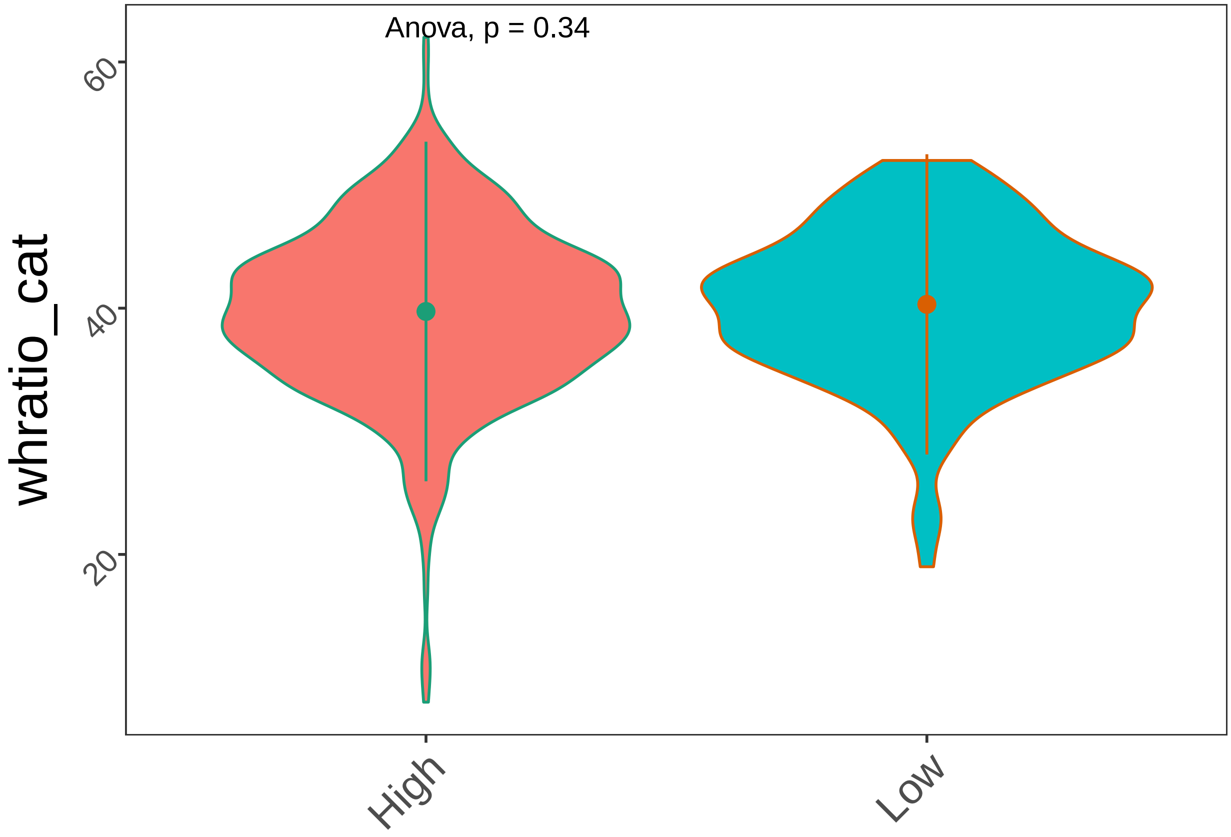


```
##
##  Kruskal-Wallis rank sum test
##
## data:  richness by whratio_cat
## Kruskal-Wallis chi-squared = 1.1009, df = 1, p-value = 0.2941
##
##
##  Pairwise comparisons using Wilcoxon rank sum test with continuity correction
##
## data:  Phe2$richness and Phe2[, which(colnames(Phe2) %in% i)]
##
##     High
## Low 0.29
##
## P value adjustment method: bonferroni
```

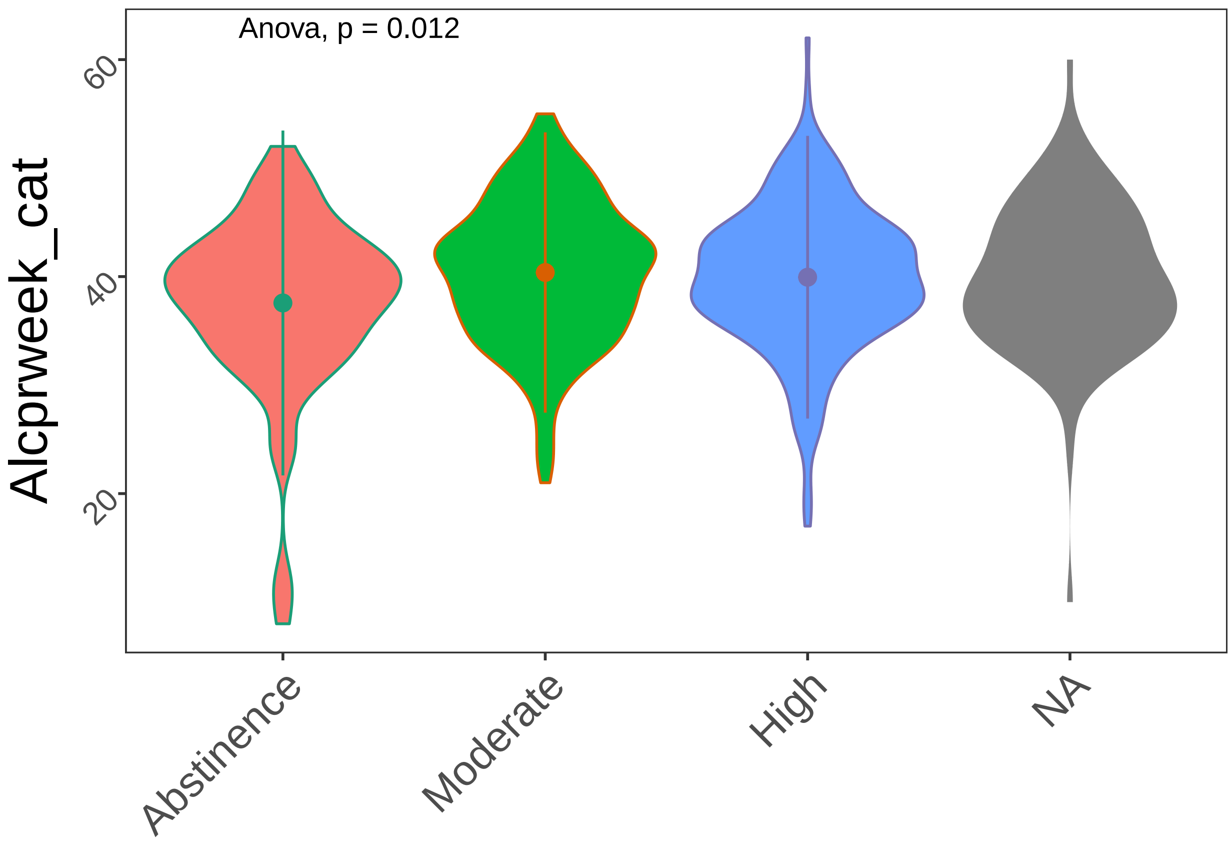


```
##
##  Kruskal-Wallis rank sum test
##
## data:  richness by Alcprweek_cat
## Kruskal-Wallis chi-squared = 7.1977, df = 2, p-value = 0.02736
##
##
##  Pairwise comparisons using Wilcoxon rank sum test with continuity correction
##
## data:  Phe2$richness and Phe2[, which(colnames(Phe2) %in% i)]
##
##          Abstinence Moderate
## Moderate 0.022      -
## High     0.096      1.000
##
## P value adjustment method: bonferroni
```

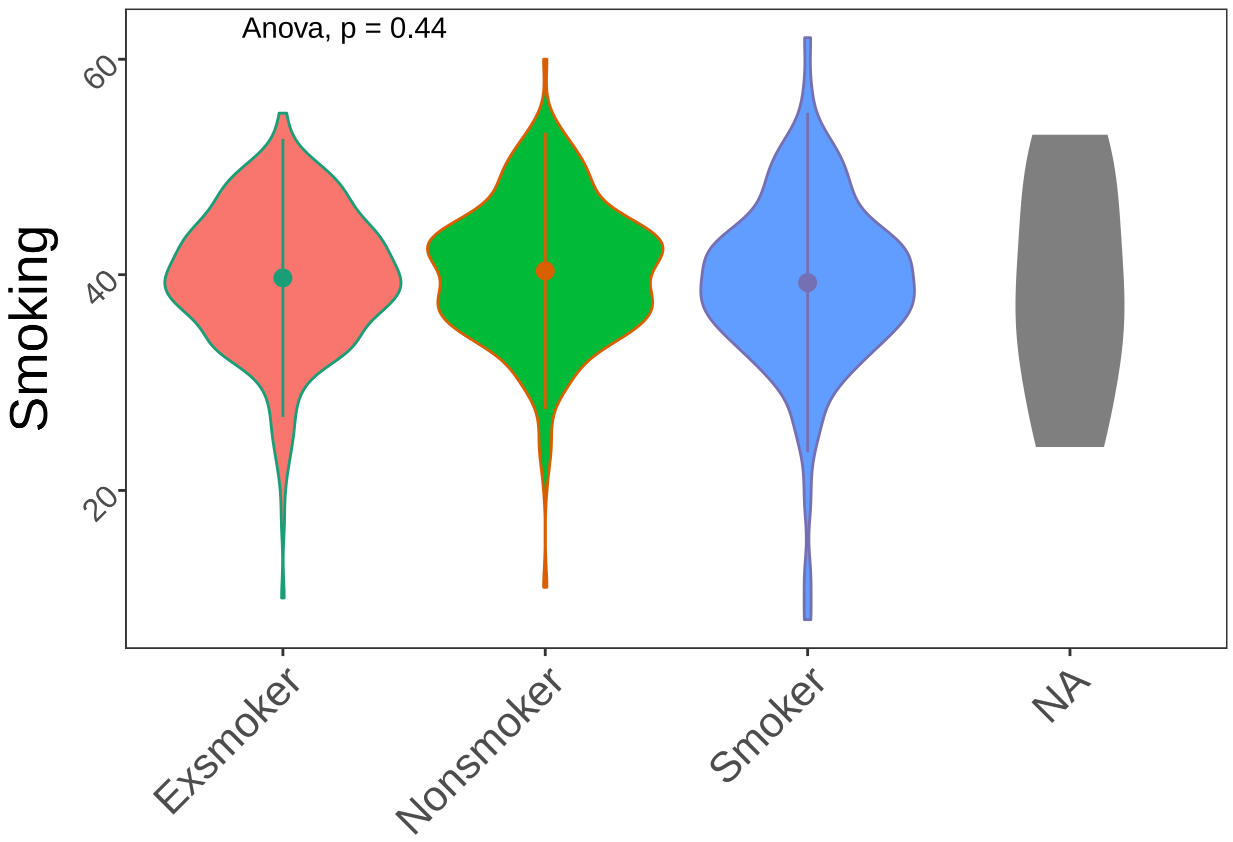


```
##
##  Kruskal-Wallis rank sum test
##
## data:  richness by Smoking
## Kruskal-Wallis chi-squared = 2.101, df = 2, p-value = 0.3498
##
##
##  Pairwise comparisons using Wilcoxon rank sum test with continuity correction
##
## data:  Phe2$richness and Phe2[, which(colnames(Phe2) %in% i)]
##
##           Exsmoker Nonsmoker
## Nonsmoker 0.97     -
## Smoker    1.00     0.50
##
## P value adjustment method: bonferroni
```

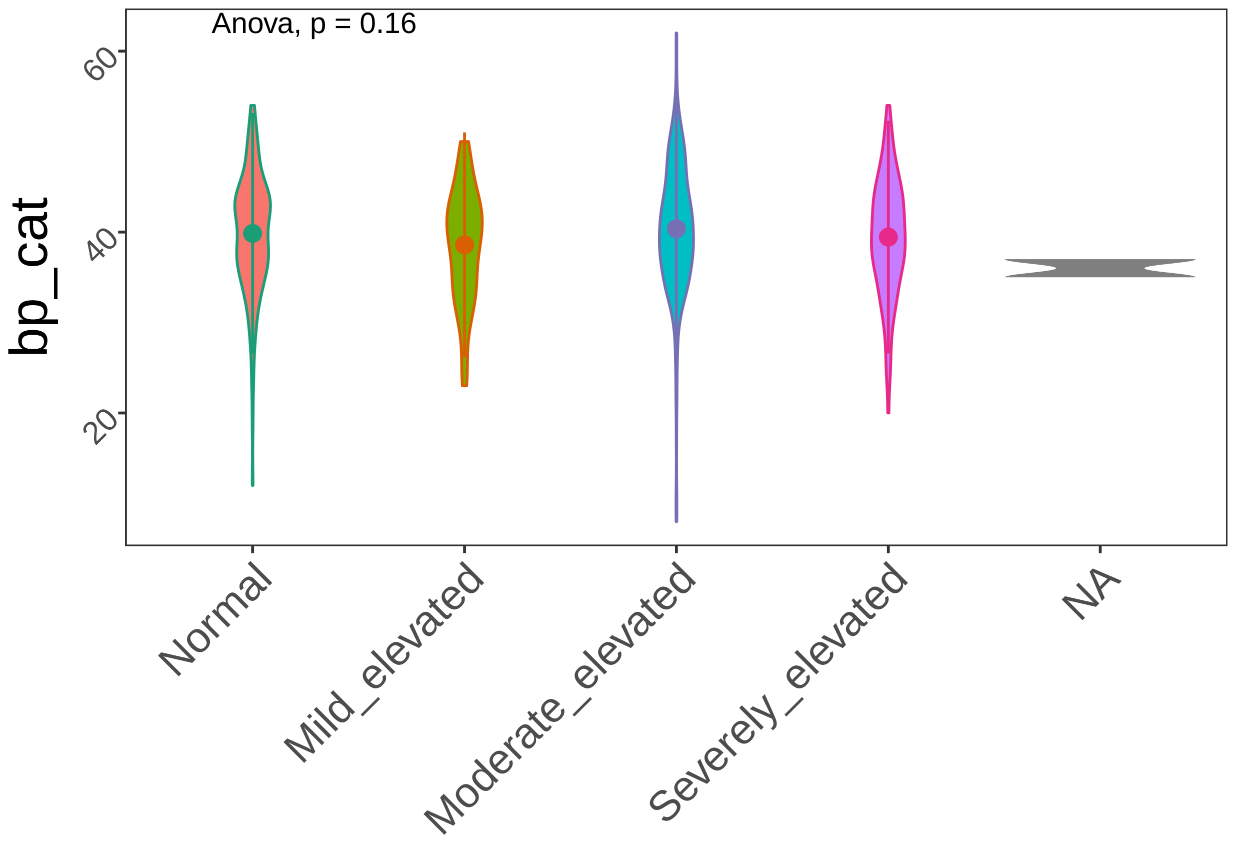


```
##
##  Kruskal-Wallis rank sum test
##
## data:  richness by bp_cat
## Kruskal-Wallis chi-squared = 5.3476, df = 3, p-value = 0.148
##
##
##  Pairwise comparisons using Wilcoxon rank sum test with continuity correction
##
## data:  Phe2$richness and Phe2[, which(colnames(Phe2) %in% i)]
##
##                   Normal Mild_elevated Moderate_elevated
## Mild_elevated     0.73   -             -
## Moderate_elevated 1.00   0.19          -
## Severely_elevated 1.00   1.00          1.00
##
## P value adjustment method: bonferroni
```

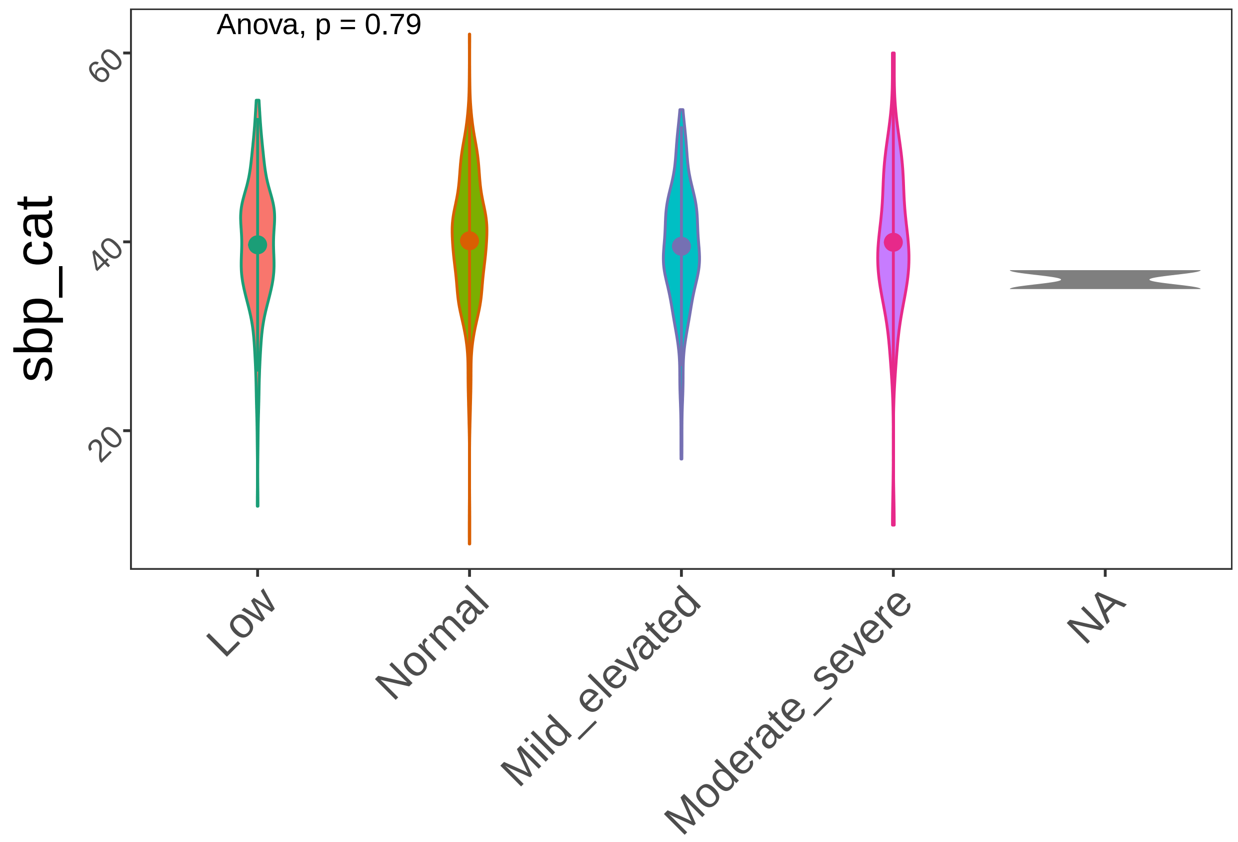


```
##
##  Kruskal-Wallis rank sum test
##
## data:  richness by sbp_cat
## Kruskal-Wallis chi-squared = 1.1038, df = 3, p-value = 0.7762
##
##
##  Pairwise comparisons using Wilcoxon rank sum test with continuity correction
##
## data:  Phe2$richness and Phe2[, which(colnames(Phe2) %in% i)]
##
##                 Low Normal Mild_elevated
## Normal          1   -      -
## Mild_elevated   1   1      -
## Moderate_severe 1   1      1
##
## P value adjustment method: bonferroni
```

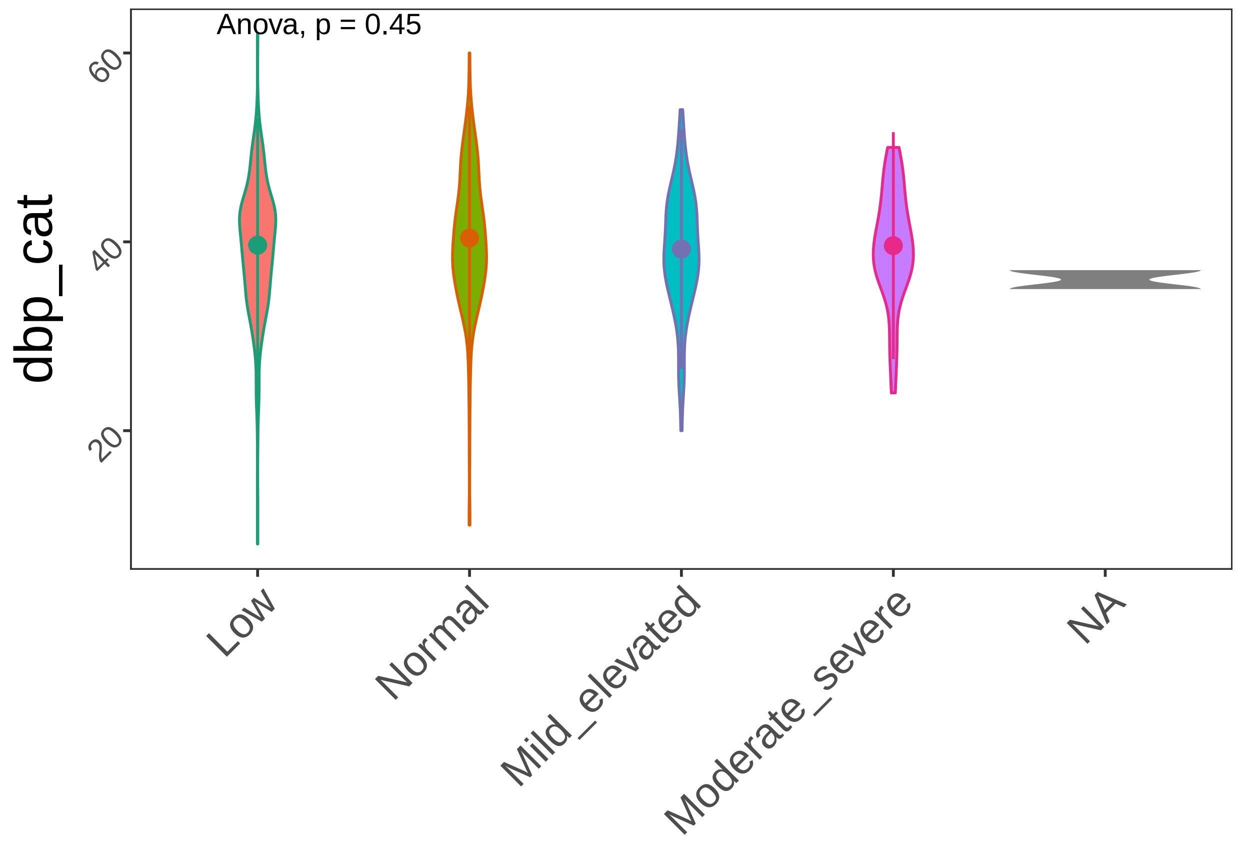


```
##
##  Kruskal-Wallis rank sum test
##
## data:  richness by dbp_cat
## Kruskal-Wallis chi-squared = 2.1321, df = 3, p-value = 0.5455
##
##
##  Pairwise comparisons using Wilcoxon rank sum test with continuity correction
##
## data:  Phe2$richness and Phe2[, which(colnames(Phe2) %in% i)]
##
##                 Low Normal Mild_elevated
## Normal          1   -      -
## Mild_elevated   1   1      -
## Moderate_severe 1   1      1
##
## P value adjustment method: bonferroni
```

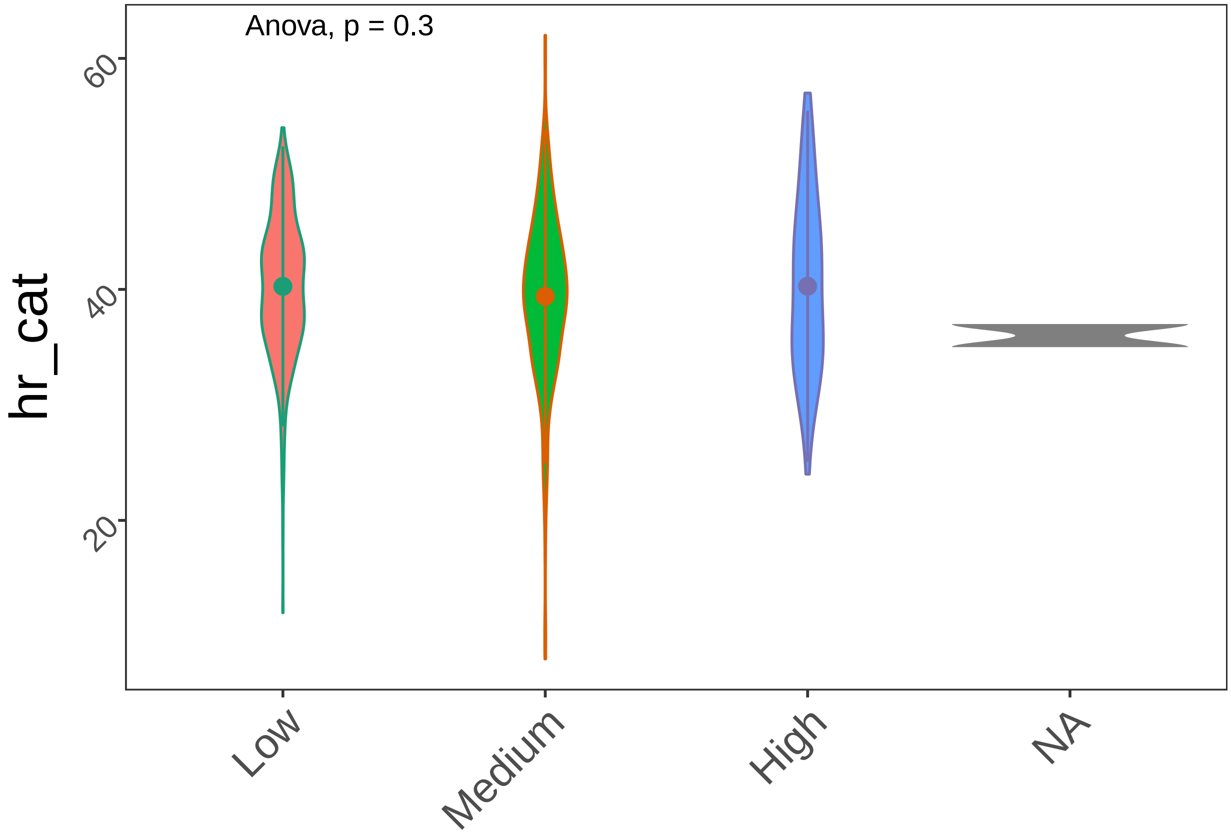


```
##
##  Kruskal-Wallis rank sum test
##
## data:  richness by hr_cat
## Kruskal-Wallis chi-squared = 2.0629, df = 2, p-value = 0.3565
##
##
##  Pairwise comparisons using Wilcoxon rank sum test with continuity correction
##
## data:  Phe2$richness and Phe2[, which(colnames(Phe2) %in% i)]
##
##        Low  Medium
## Medium 0.45 -
## High   1.00 1.00
##
## P value adjustment method: bonferroni
```

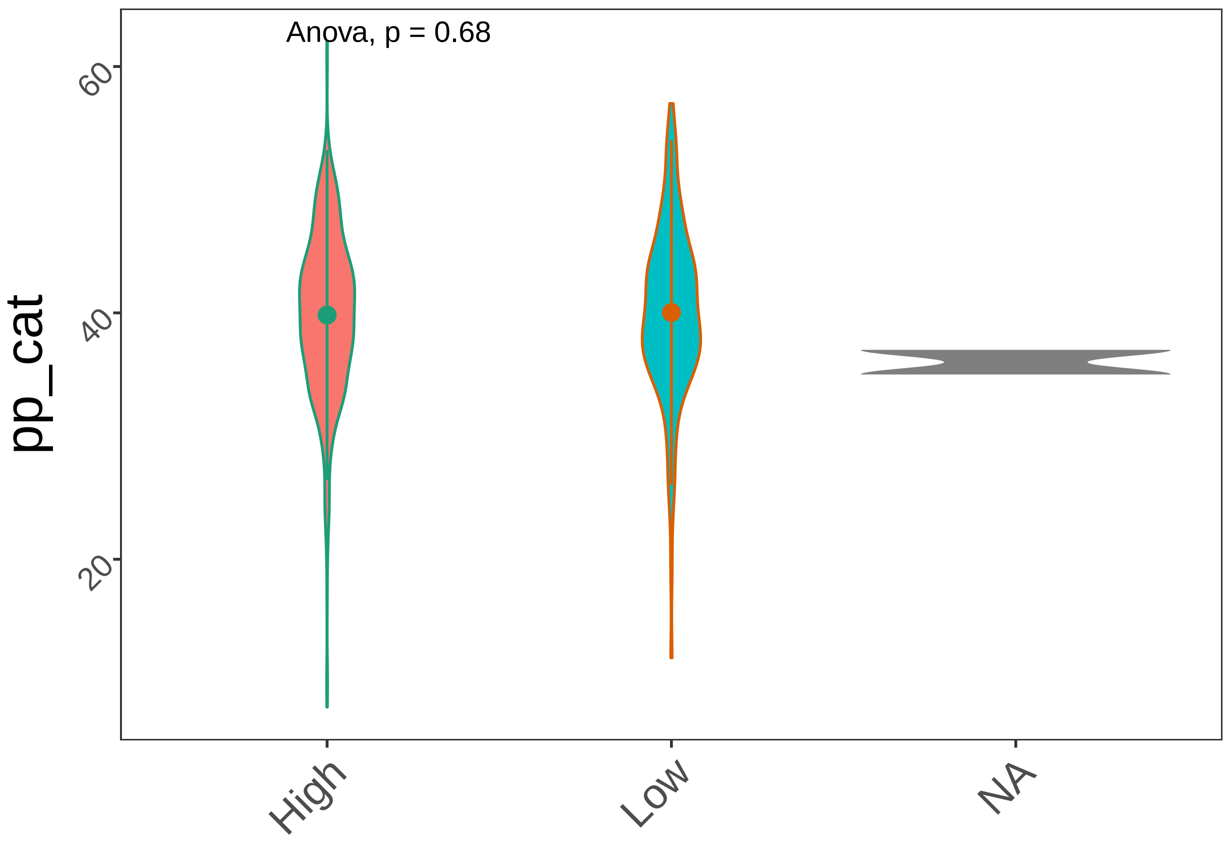


```
##
##  Kruskal-Wallis rank sum test
##
## data:  richness by pp_cat
## Kruskal-Wallis chi-squared = 0.083894, df = 1, p-value = 0.7721
##
##
##  Pairwise comparisons using Wilcoxon rank sum test with continuity correction
##
## data:  Phe2$richness and Phe2[, which(colnames(Phe2) %in% i)]
##
##     High
## Low 0.77
##
## P value adjustment method: bonferroni
```

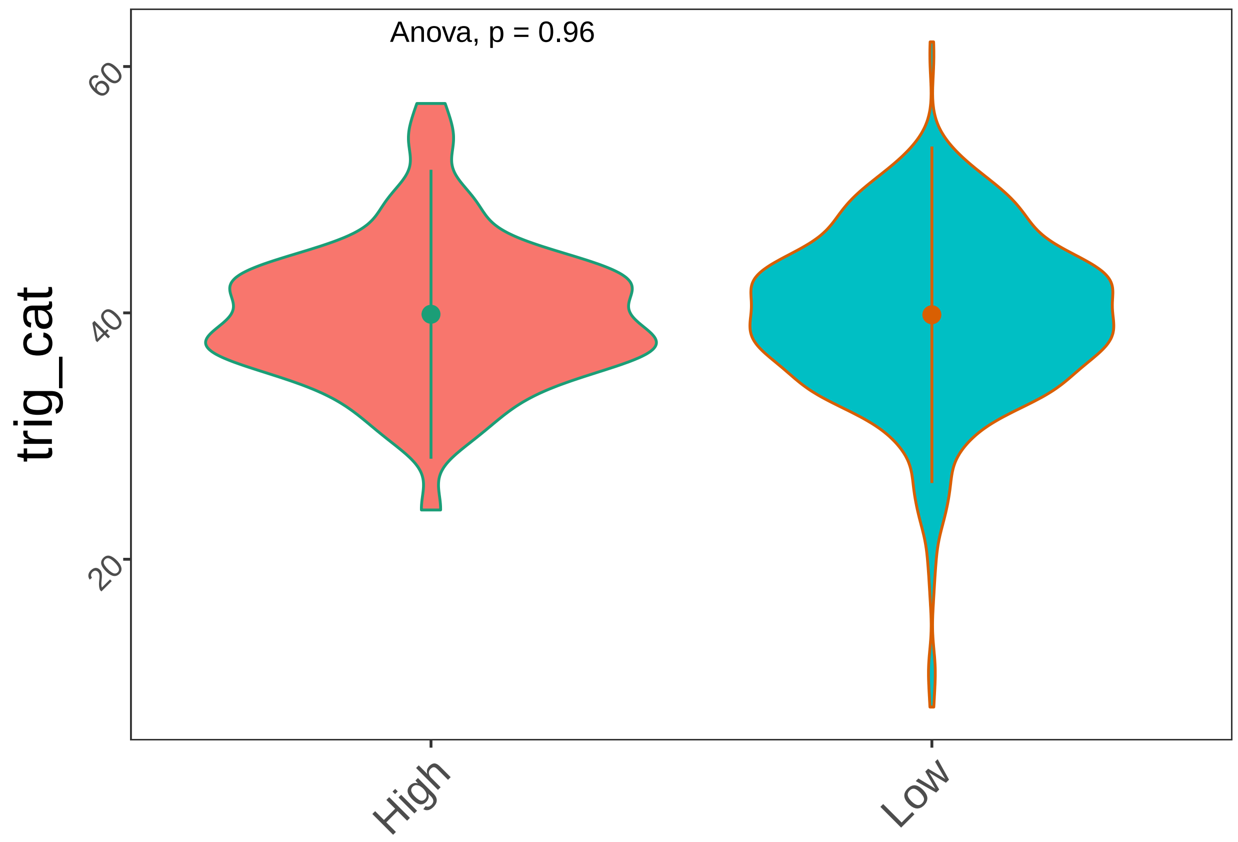


```
##
##  Kruskal-Wallis rank sum test
##
## data:  richness by trig_cat
## Kruskal-Wallis chi-squared = 0.19727, df = 1, p-value = 0.6569
##
##
##  Pairwise comparisons using Wilcoxon rank sum test with continuity correction
##
## data:  Phe2$richness and Phe2[, which(colnames(Phe2) %in% i)]
##
##     High
## Low 0.66
##
## P value adjustment method: bonferroni
```

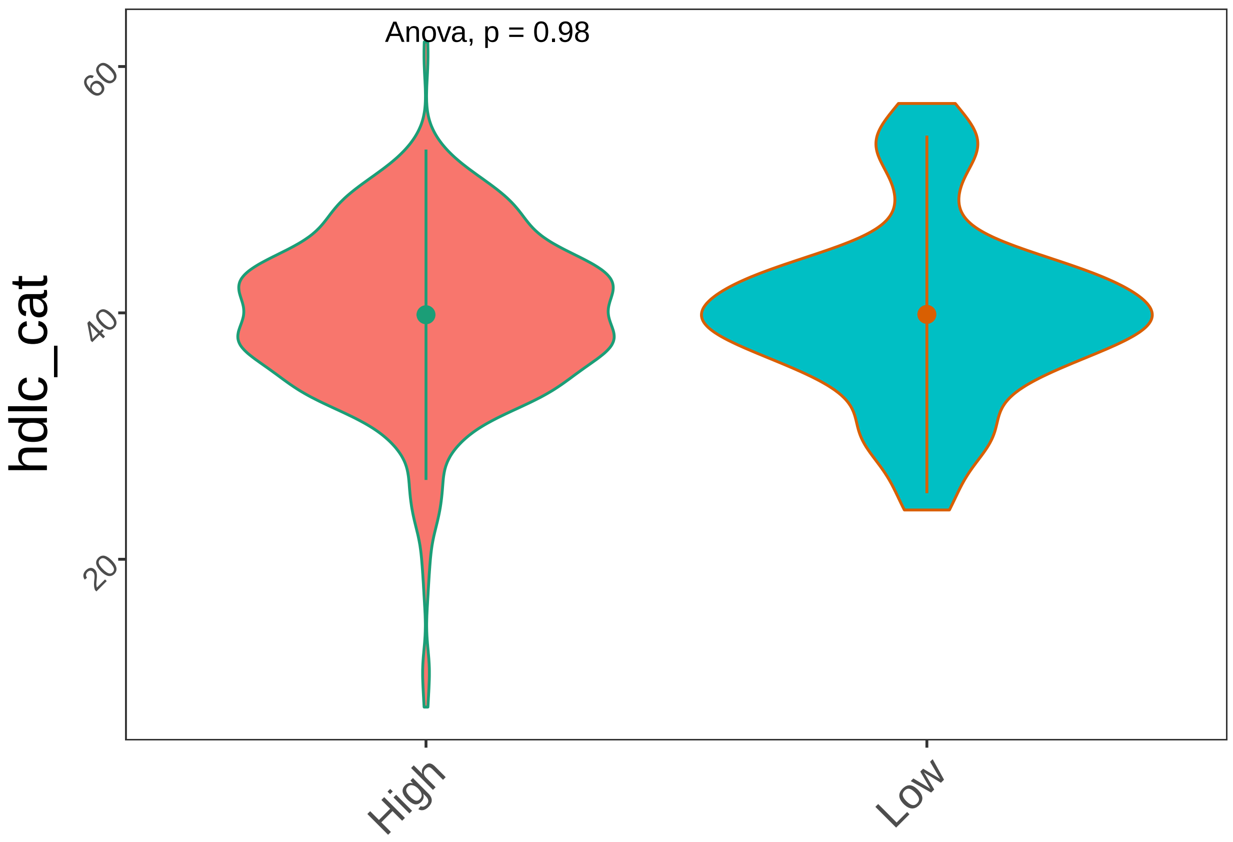


```
##
##  Kruskal-Wallis rank sum test
##
## data:  richness by hdlc_cat
## Kruskal-Wallis chi-squared = 0.11859, df = 1, p-value = 0.7306
##
##
##  Pairwise comparisons using Wilcoxon rank sum test with continuity correction
##
## data:  Phe2$richness and Phe2[, which(colnames(Phe2) %in% i)]
##
##     High
## Low 0.73
##
## P value adjustment method: bonferroni
```

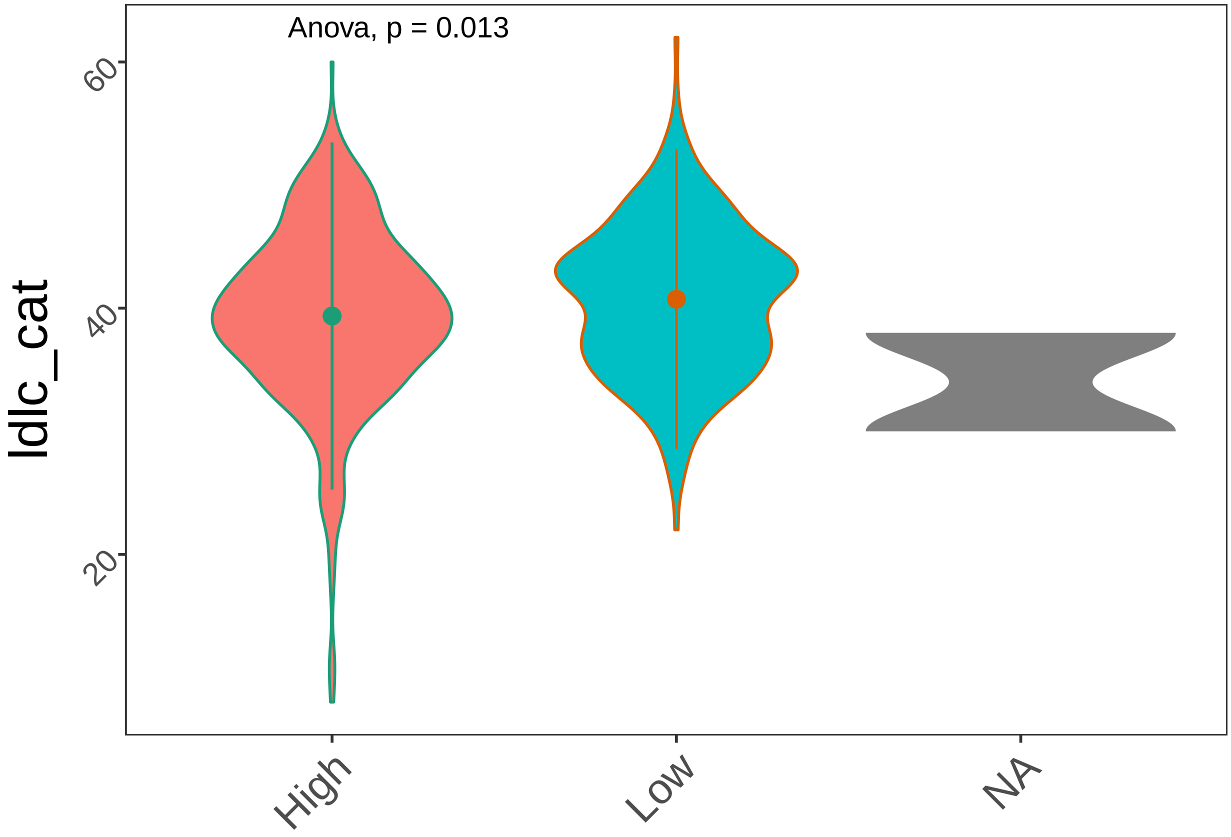


```
##
##  Kruskal-Wallis rank sum test
##
## data:  richness by ldlc_cat
## Kruskal-Wallis chi-squared = 5.3189, df = 1, p-value = 0.02109
##
##
##  Pairwise comparisons using Wilcoxon rank sum test with continuity correction
##
## data:  Phe2$richness and Phe2[, which(colnames(Phe2) %in% i)]
##
##     High
## Low 0.021
##
## P value adjustment method: bonferroni
```

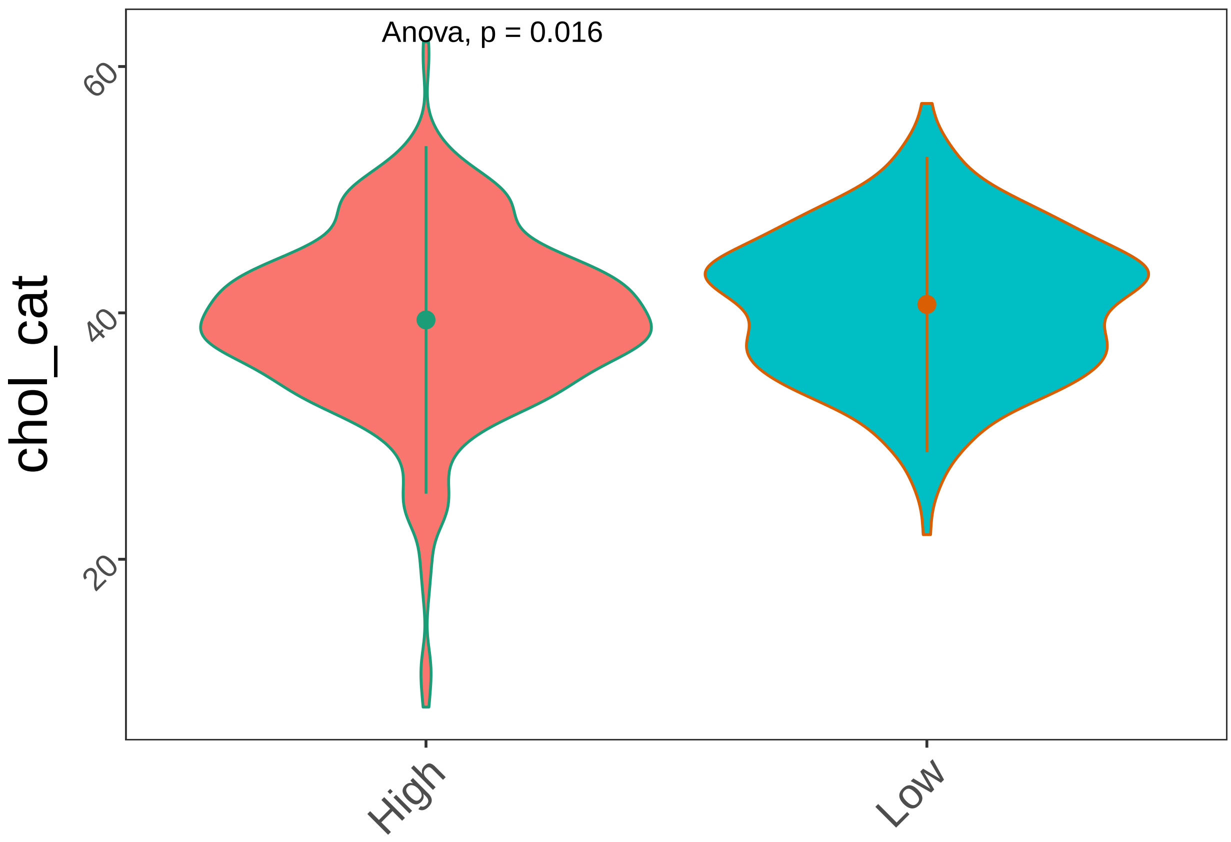


```
##
##  Kruskal-Wallis rank sum test
##
## data:  richness by chol_cat
## Kruskal-Wallis chi-squared = 5.0696, df = 1, p-value = 0.02435
##
##
##  Pairwise comparisons using Wilcoxon rank sum test with continuity correction
##
## data:  Phe2$richness and Phe2[, which(colnames(Phe2) %in% i)]
##
##     High
## Low 0.024
##
## P value adjustment method: bonferroni
```

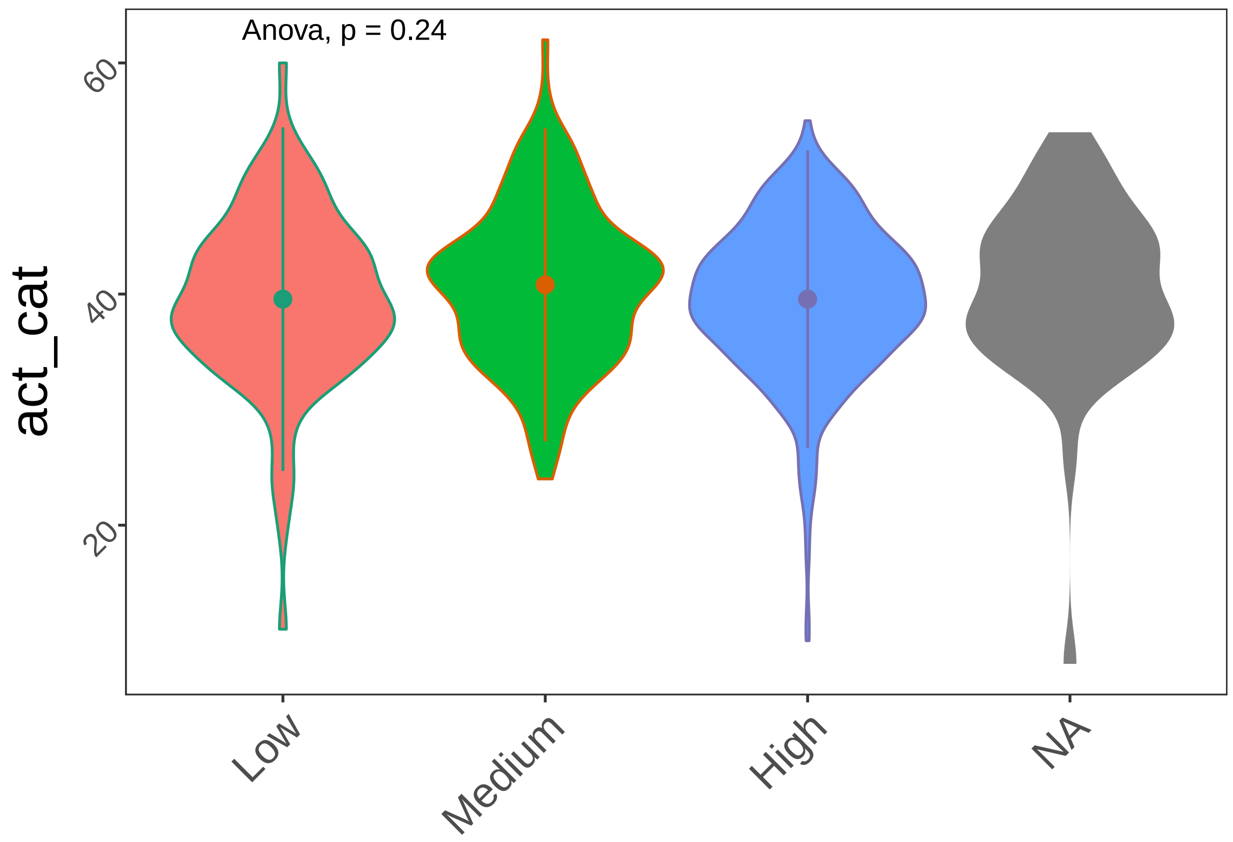


```
##
##  Kruskal-Wallis rank sum test
##
## data:  richness by act_cat
## Kruskal-Wallis chi-squared = 2.442, df = 2, p-value = 0.2949
##
##
##  Pairwise comparisons using Wilcoxon rank sum test with continuity correction
##
## data:  Phe2$richness and Phe2[, which(colnames(Phe2) %in% i)]
##
##        Low  Medium
## Medium 0.83 -
## High   1.00 0.38
##
## P value adjustment method: bonferroni
```

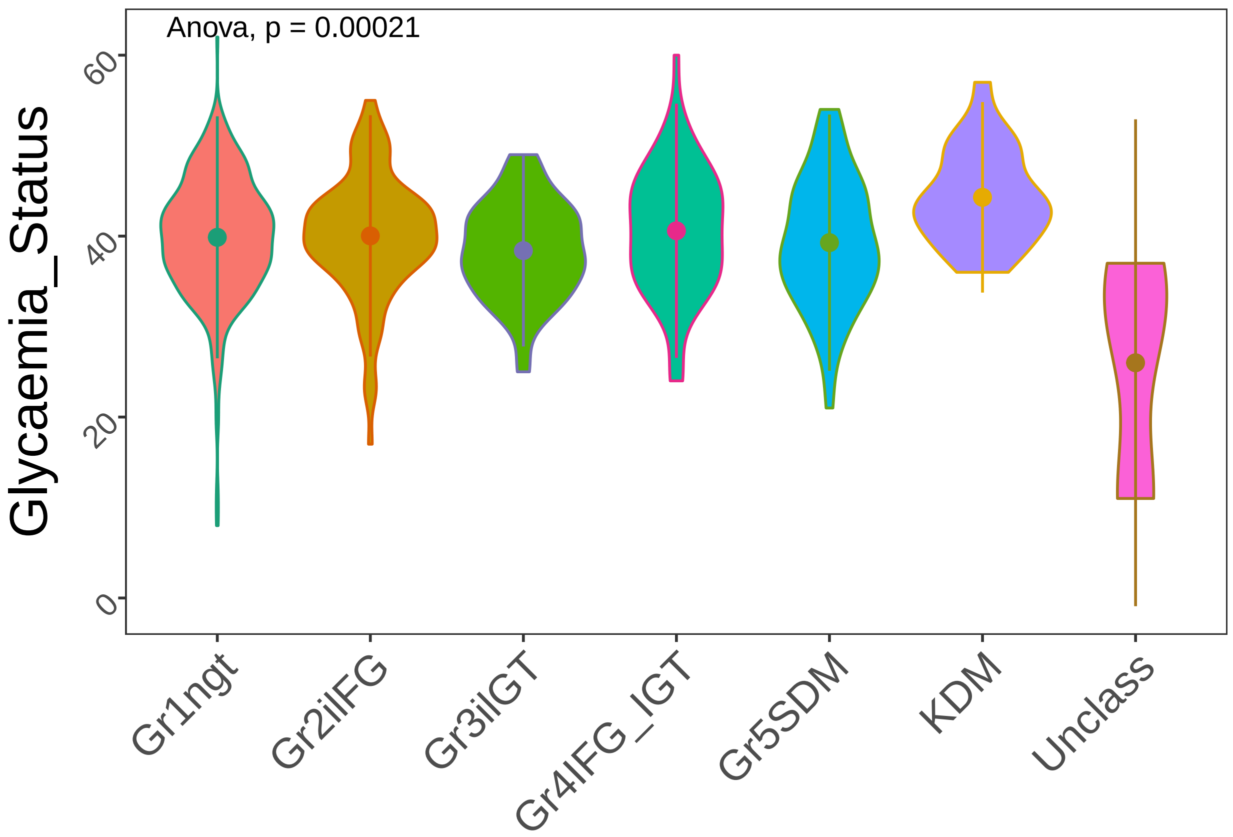


```
##
##  Kruskal-Wallis rank sum test
##
## data:  richness by Glycaemia_Status
## Kruskal-Wallis chi-squared = 20.203, df = 6, p-value = 0.002549
##
##
##  Pairwise comparisons using Wilcoxon rank sum test with continuity correction
##
## data:  Phe2$richness and Phe2[, which(colnames(Phe2) %in% i)]
##
##            Gr1ngt Gr2ilFG Gr3ilGT Gr4IFG_IGT Gr5SDM KDM
## Gr2ilFG    1.0000 -       -       -          -      -
## Gr3ilGT    1.0000 0.9178  -       -          -      -
## Gr4IFG_IGT 1.0000 1.0000  1.0000  -          -      -
## Gr5SDM     1.0000 1.0000  1.0000  1.0000     -      -
## KDM        0.0540 0.1432  0.0021  0.7120     0.1020 -
## Unclass    0.5181 0.4678  0.9045  0.6492     0.9792 0.1793
##
## P value adjustment method: bonferroni
```

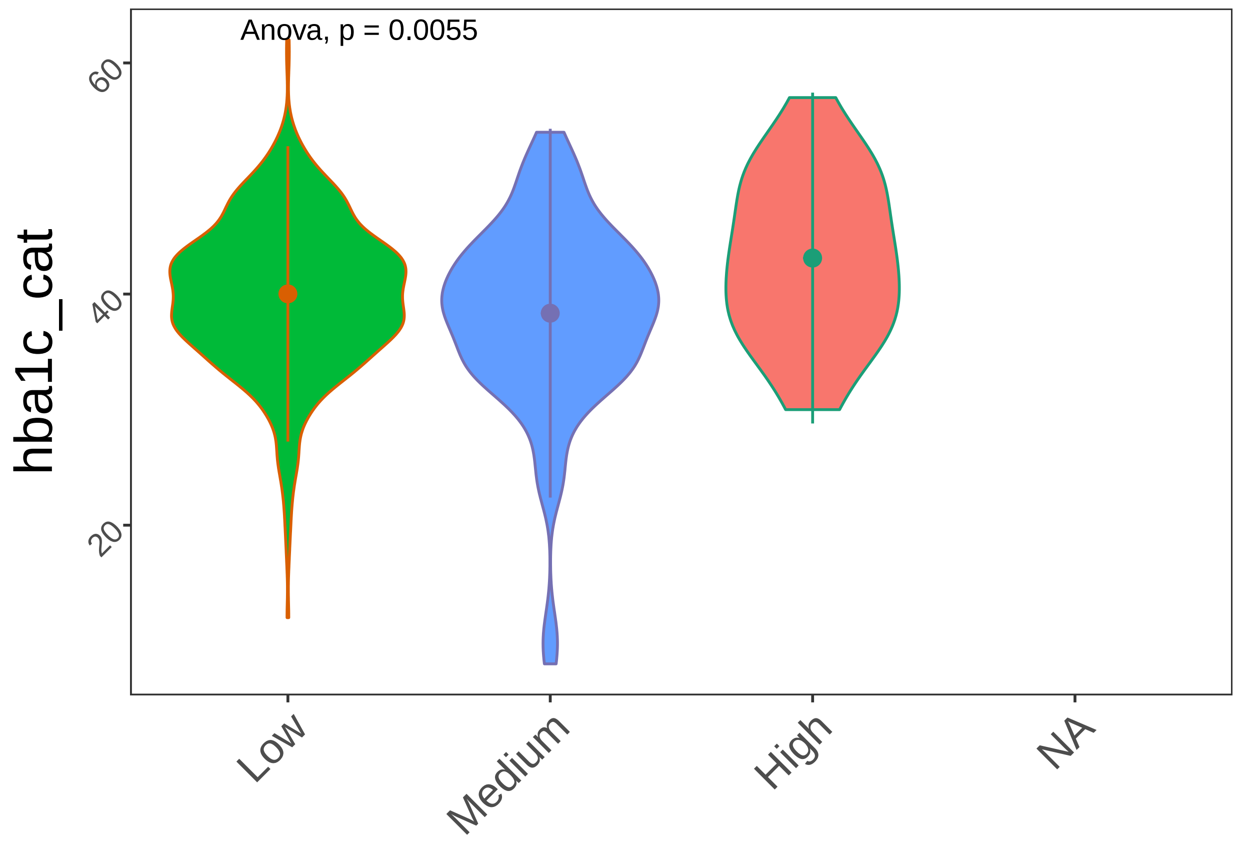


```
##
##  Kruskal-Wallis rank sum test
##
## data:  richness by hba1c_cat
## Kruskal-Wallis chi-squared = 8.566, df = 2, p-value = 0.0138
##
##
##  Pairwise comparisons using Wilcoxon rank sum test with continuity correction
##
## data:  Phe2$richness and Phe2[, which(colnames(Phe2) %in% i)]
##
##        Low   Medium
## Medium 0.149 -
## High   0.122 0.027
##
## P value adjustment method: bonferroni
```

```
for (i in var) {
#Create plot name
pltName <- paste('AlphaShannon', i, sep = '')
#create boxplots
AlphaList[[ pltName ]]<- ggplot(Phe2, aes_string(x=i, y="Shannon", group=i, color=i, fill=i)) +
    geom_violin() +
    stat_summary(fun.data="mean_sdl",
                 mult=1, #mean plus minus a constant (mult=1) times the st.dev
```

```
                 geom="pointrange",
                 width=0.2 ) +
    stat_summary(fun.y = mean, geom = "point") +
    stat_compare_means(method="anova") +
    ggtitle("Shannon") +
    ylab(paste(i)) +
    scale_color_brewer(palette="Dark2") +
    theme_bw() +
    theme(legend.position="none", panel.grid.major = element_blank(),
          panel.grid.minor = element_blank(), axis.title=element_text(size=20),
          axis.title.x = element_blank(),
          axis.text.x = element_text(angle = 45, hjust = 1, size=16),
          axis.text.y = element_text(angle = 45, hjust = 1, size=12))
print(ggplot(Phe2, aes_string(x=i, y="Shannon", group=i, color=i, fill=i)) +
    geom_violin() +
    stat_summary(fun.data="mean_sdl",
                 mult=1, #mean plus minus a constant (mult=1) times the st.dev
                 geom="pointrange",
                 width=0.2 ) +
    stat_summary(fun.y = mean, geom = "point") +
    stat_compare_means(method="anova") +
    #ggtitle(i) +
    ylab(paste(i)) +
    scale_color_brewer(palette="Dark2") +
    theme_bw() +
    theme(legend.position="none", panel.grid.major = element_blank(),
          panel.grid.minor = element_blank(), axis.title=element_text(size=20),
          axis.title.x = element_blank(),
          axis.text.x = element_text(angle = 45, hjust = 1, size=16),
          axis.text.y = element_text(angle = 45, hjust = 1, size=12)))

print(kruskal.test(formula(paste("Shannon ~", i)), data=Phe2))
print(pairwise.wilcox.test(Phe2$Shannon, Phe2[,which( colnames(Phe2) %in% i )],
                      p.adjust.method="bonferroni")) #If variable binary result same as kruskal
}
```

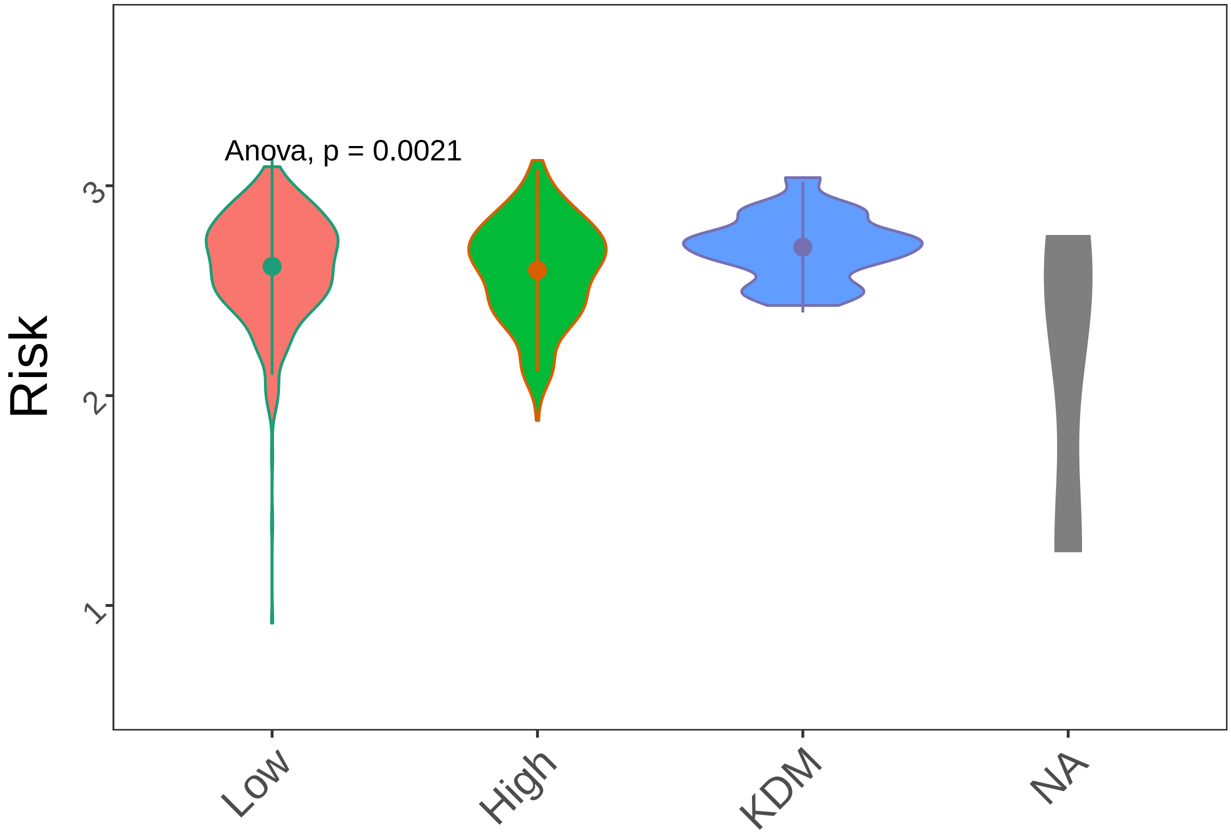


```
##
##  Kruskal-Wallis rank sum test
##
## data:  Shannon by Risk
## Kruskal-Wallis chi-squared = 5.0073, df = 2, p-value = 0.08179
##
##
##  Pairwise comparisons using Wilcoxon rank sum test with continuity correction
##
## data:  Phe2$Shannon and Phe2[, which(colnames(Phe2) %in% i)]
##
##      Low  High
## High 0.50 -
## KDM  0.38 0.13
##
## P value adjustment method: bonferroni
```

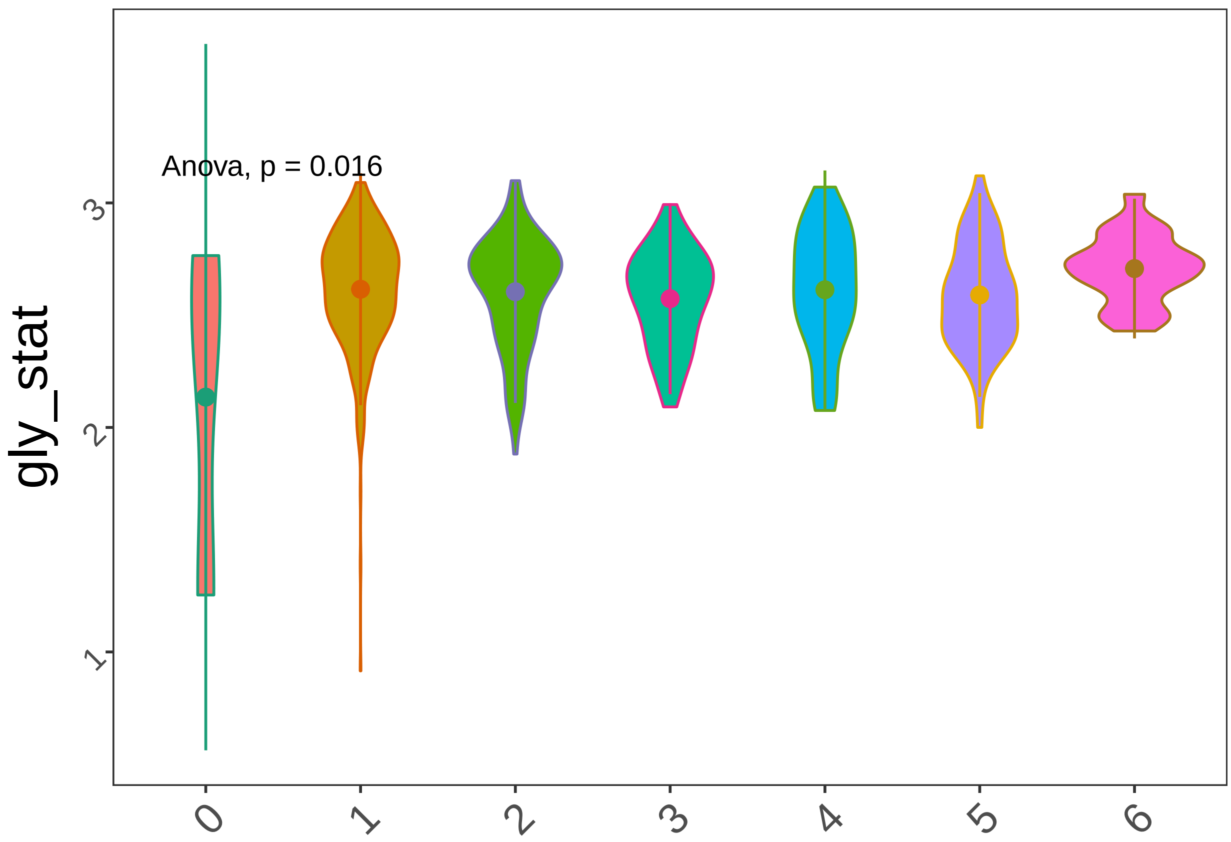


```
##
##  Kruskal-Wallis rank sum test
##
## data:  Shannon by gly_stat
## Kruskal-Wallis chi-squared = 8.759, df = 6, p-value = 0.1876
##
##
##  Pairwise comparisons using Wilcoxon rank sum test with continuity correction
##
## data:  Phe2$Shannon and Phe2[, which(colnames(Phe2) %in% i)]
##
##   0   1   2   3   4   5
## 1 1.0 -   -   -   -   -
## 2 1.0 1.0 -   -   -   -
## 3 1.0 1.0 1.0 -   -   -
## 4 1.0 1.0 1.0 1.0 -   -
## 5 1.0 1.0 1.0 1.0 1.0 -
## 6 1.0 1.0 1.0 0.3 1.0 0.4
##
## P value adjustment method: bonferroni
```

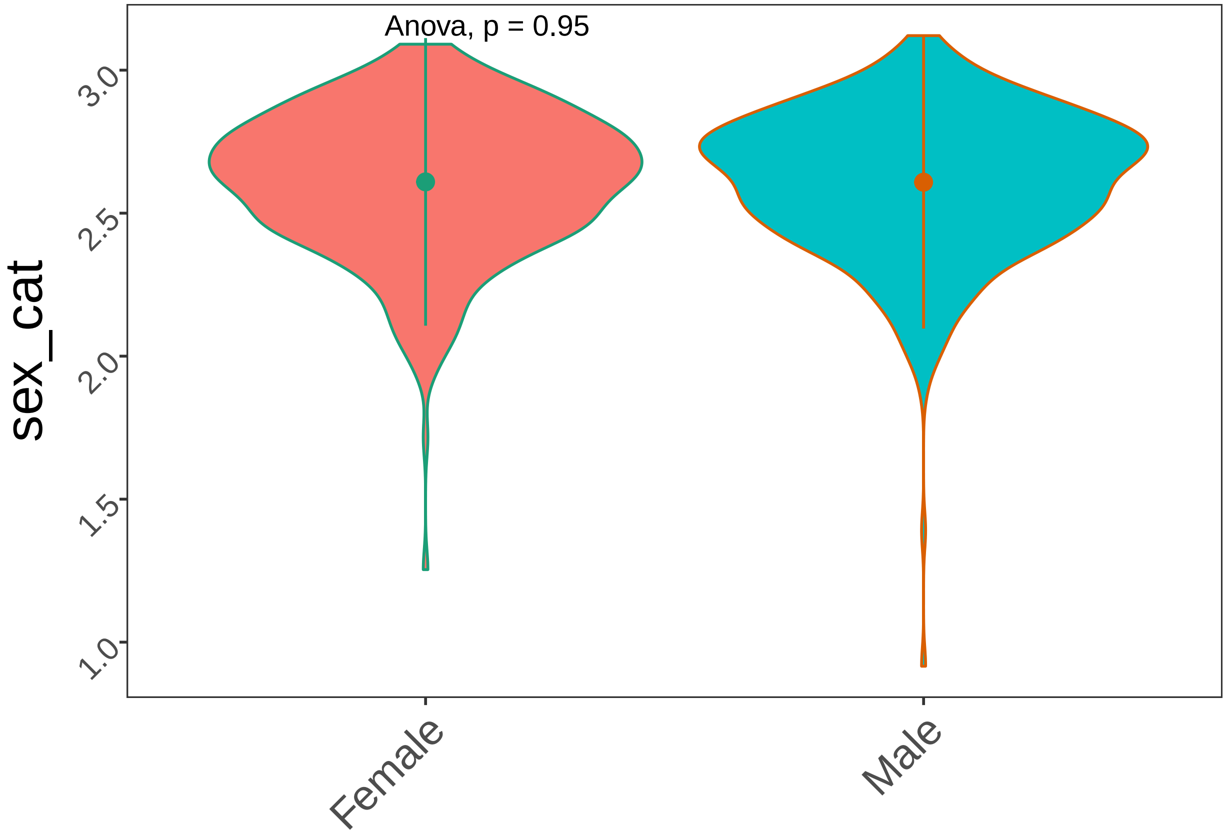


```
##
##  Kruskal-Wallis rank sum test
##
## data:  Shannon by sex_cat
## Kruskal-Wallis chi-squared = 3.9848e-05, df = 1, p-value = 0.995
##
##
##  Pairwise comparisons using Wilcoxon rank sum test with continuity correction
##
## data:  Phe2$Shannon and Phe2[, which(colnames(Phe2) %in% i)]
##
##      Female
## Male 1
##
## P value adjustment method: bonferroni
```

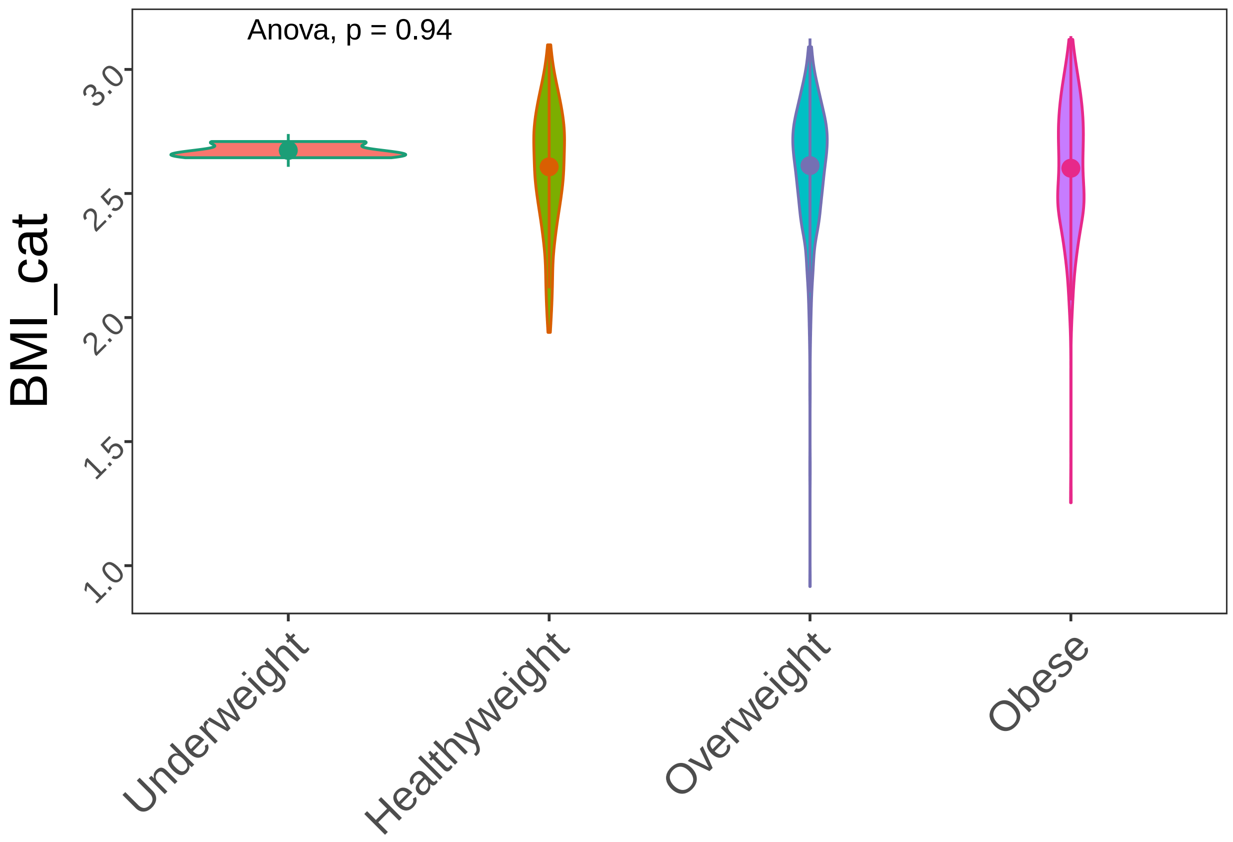


```
##
##  Kruskal-Wallis rank sum test
##
## data:  Shannon by BMI_cat
## Kruskal-Wallis chi-squared = 0.58511, df = 3, p-value = 0.8998
##
##
##  Pairwise comparisons using Wilcoxon rank sum test with continuity correction
##
## data:  Phe2$Shannon and Phe2[, which(colnames(Phe2) %in% i)]
##
##               Underweight Healthyweight Overweight
## Healthyweight 1           -             -
## Overweight    1           1             -
## Obese         1           1             1
##
## P value adjustment method: bonferroni
```

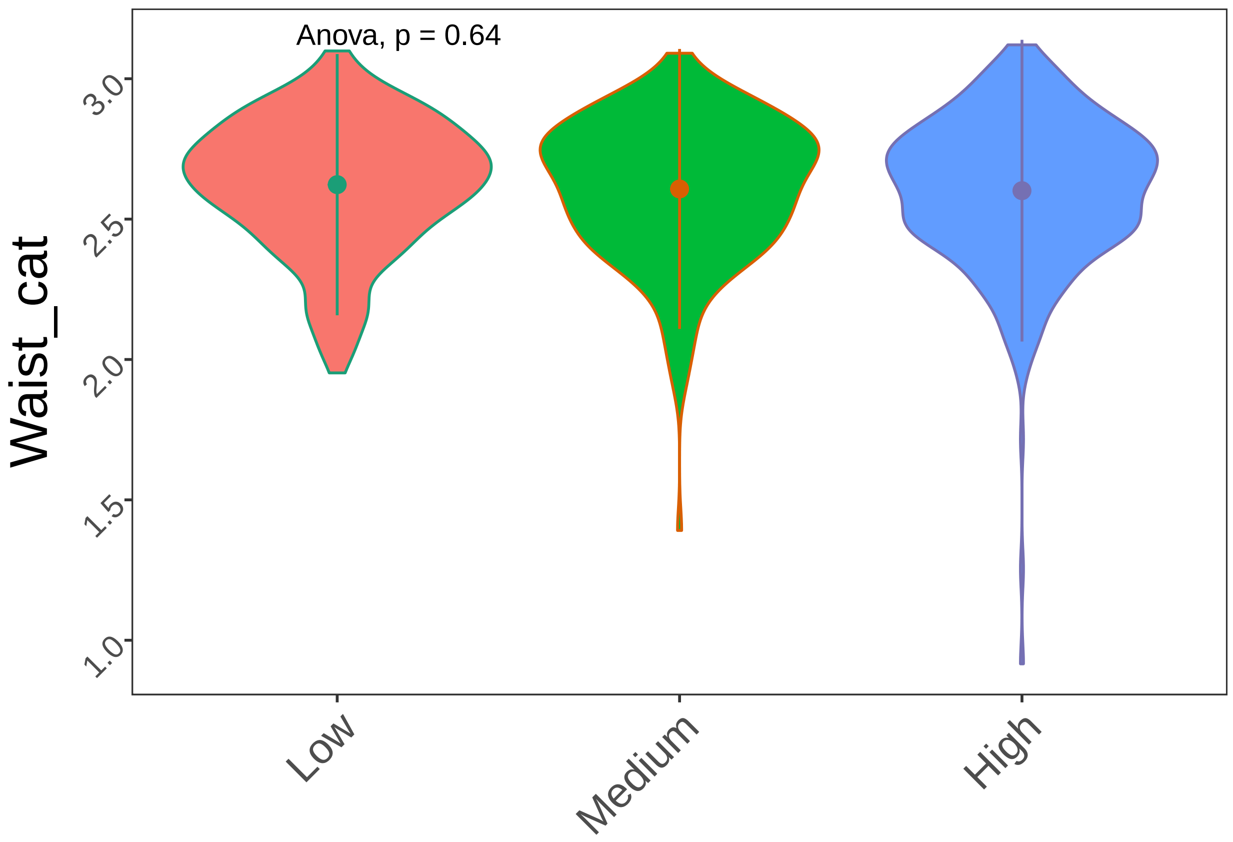


```
##
##  Kruskal-Wallis rank sum test
##
## data:  Shannon by Waist_cat
## Kruskal-Wallis chi-squared = 0.73957, df = 2, p-value = 0.6909
##
##
##  Pairwise comparisons using Wilcoxon rank sum test with continuity correction
##
## data:  Phe2$Shannon and Phe2[, which(colnames(Phe2) %in% i)]
##
##        Low Medium
## Medium 1   -
## High   1   1
##
## P value adjustment method: bonferroni
```

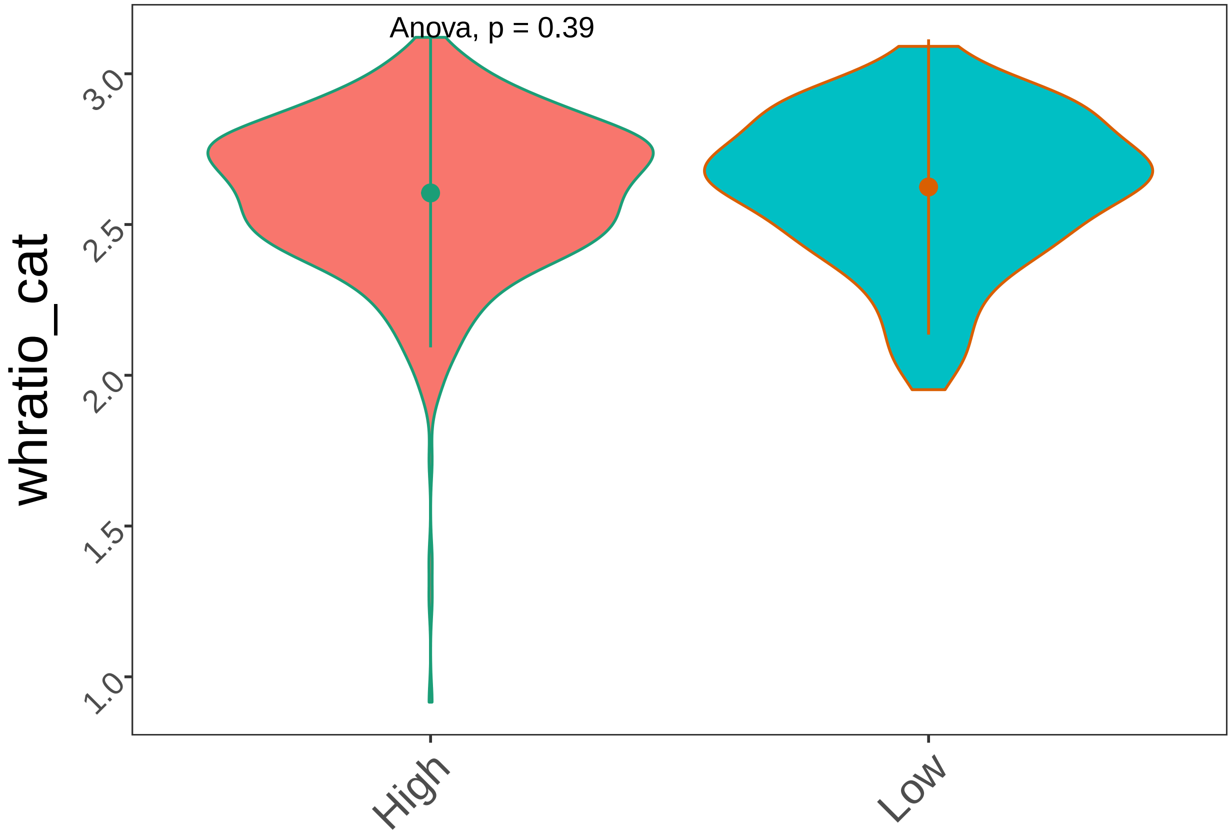


```
##
##  Kruskal-Wallis rank sum test
##
## data:  Shannon by whratio_cat
## Kruskal-Wallis chi-squared = 0.75721, df = 1, p-value = 0.3842
##
##
##  Pairwise comparisons using Wilcoxon rank sum test with continuity correction
##
## data:  Phe2$Shannon and Phe2[, which(colnames(Phe2) %in% i)]
##
##     High
## Low 0.38
##
## P value adjustment method: bonferroni
```

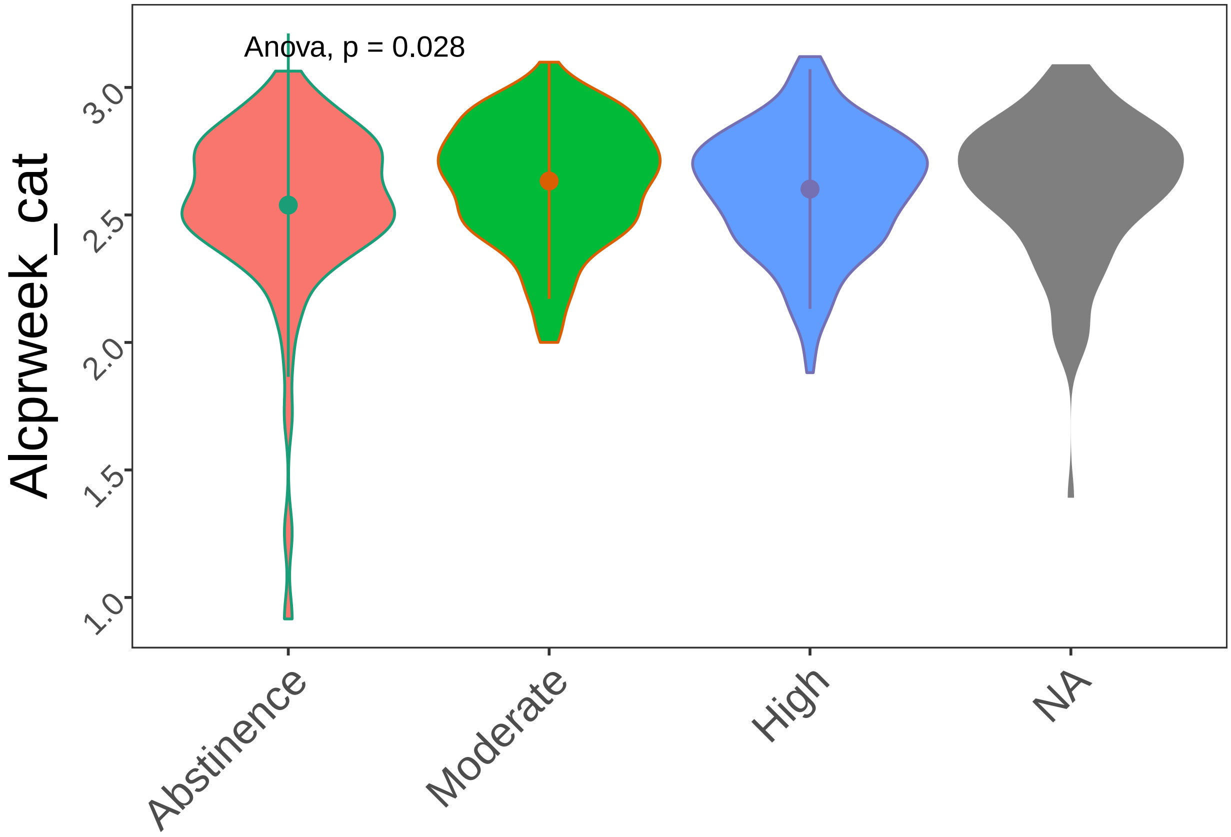


```
##
##  Kruskal-Wallis rank sum test
##
## data:  Shannon by Alcprweek_cat
## Kruskal-Wallis chi-squared = 6.0788, df = 2, p-value = 0.04786
##
##
##  Pairwise comparisons using Wilcoxon rank sum test with continuity correction
##
## data:  Phe2$Shannon and Phe2[, which(colnames(Phe2) %in% i)]
##
##          Abstinence Moderate
## Moderate 0.087      -
## High     0.879      0.245
##
## P value adjustment method: bonferroni
```

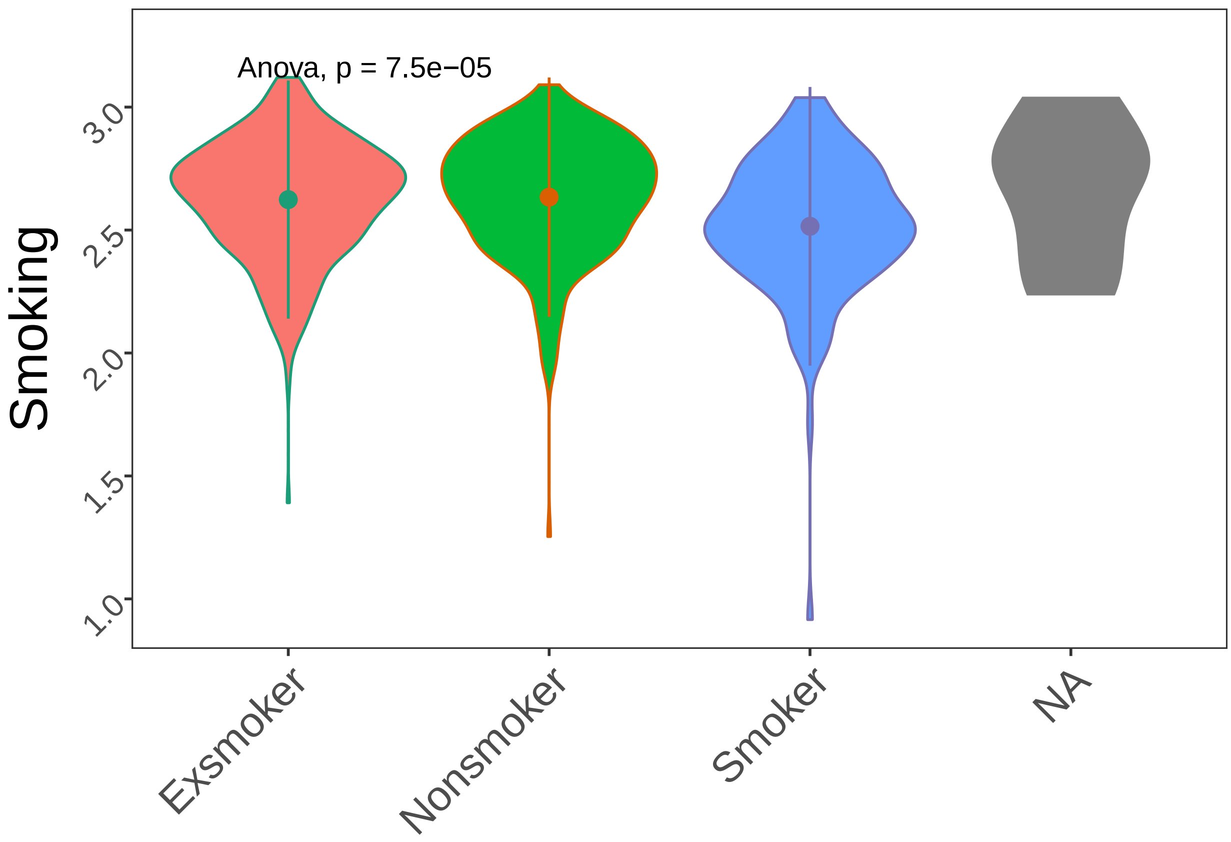


```
##
##  Kruskal-Wallis rank sum test
##
## data:  Shannon by Smoking
## Kruskal-Wallis chi-squared = 21.463, df = 2, p-value = 2.185e-05
##
##
##  Pairwise comparisons using Wilcoxon rank sum test with continuity correction
##
## data:  Phe2$Shannon and Phe2[, which(colnames(Phe2) %in% i)]
##
##           Exsmoker Nonsmoker
## Nonsmoker 1.00000  -
## Smoker    0.00013  3.3e-05
##
## P value adjustment method: bonferroni
```

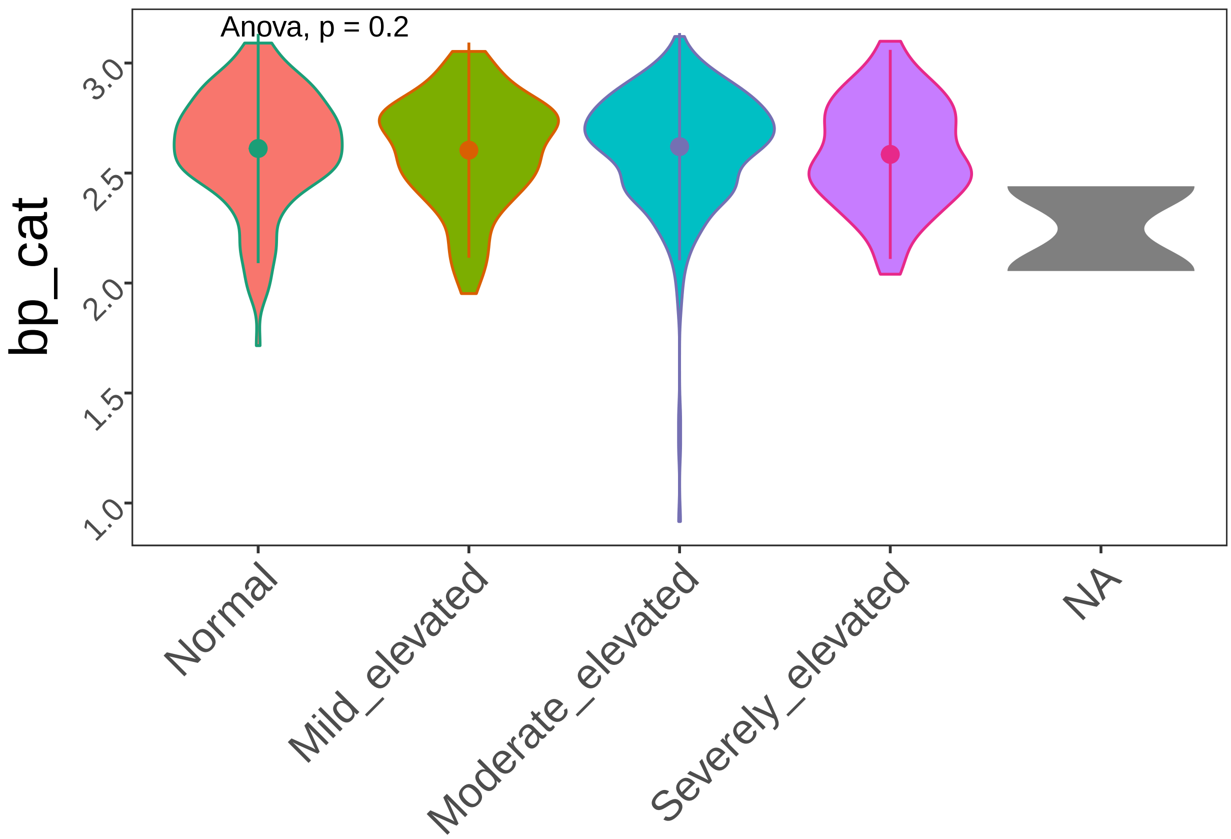


```
##
##  Kruskal-Wallis rank sum test
##
## data:  Shannon by bp_cat
## Kruskal-Wallis chi-squared = 4.2687, df = 3, p-value = 0.2339
##
##
##  Pairwise comparisons using Wilcoxon rank sum test with continuity correction
##
## data:  Phe2$Shannon and Phe2[, which(colnames(Phe2) %in% i)]
##
##                   Normal Mild_elevated Moderate_elevated
## Mild_elevated     1.00   -             -
## Moderate_elevated 1.00   1.00          -
## Severely_elevated 0.84   1.00          0.25
##
## P value adjustment method: bonferroni
```

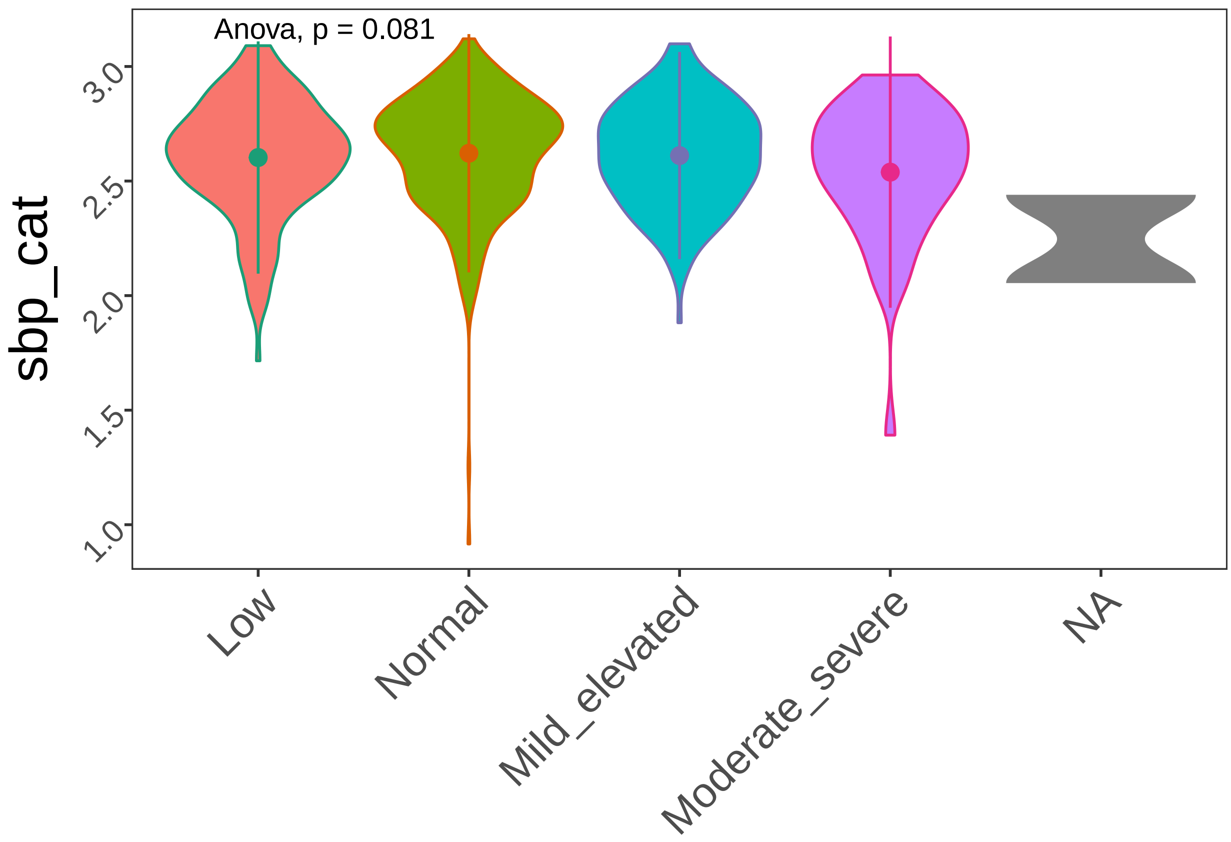


```
##
##  Kruskal-Wallis rank sum test
##
## data:  Shannon by sbp_cat
## Kruskal-Wallis chi-squared = 3.9413, df = 3, p-value = 0.2679
##
##
##  Pairwise comparisons using Wilcoxon rank sum test with continuity correction
##
## data:  Phe2$Shannon and Phe2[, which(colnames(Phe2) %in% i)]
##
##                 Low  Normal Mild_elevated
## Normal          1.00 -      -
## Mild_elevated   1.00 1.00   -
## Moderate_severe 1.00 0.48   1.00
##
## P value adjustment method: bonferroni
```

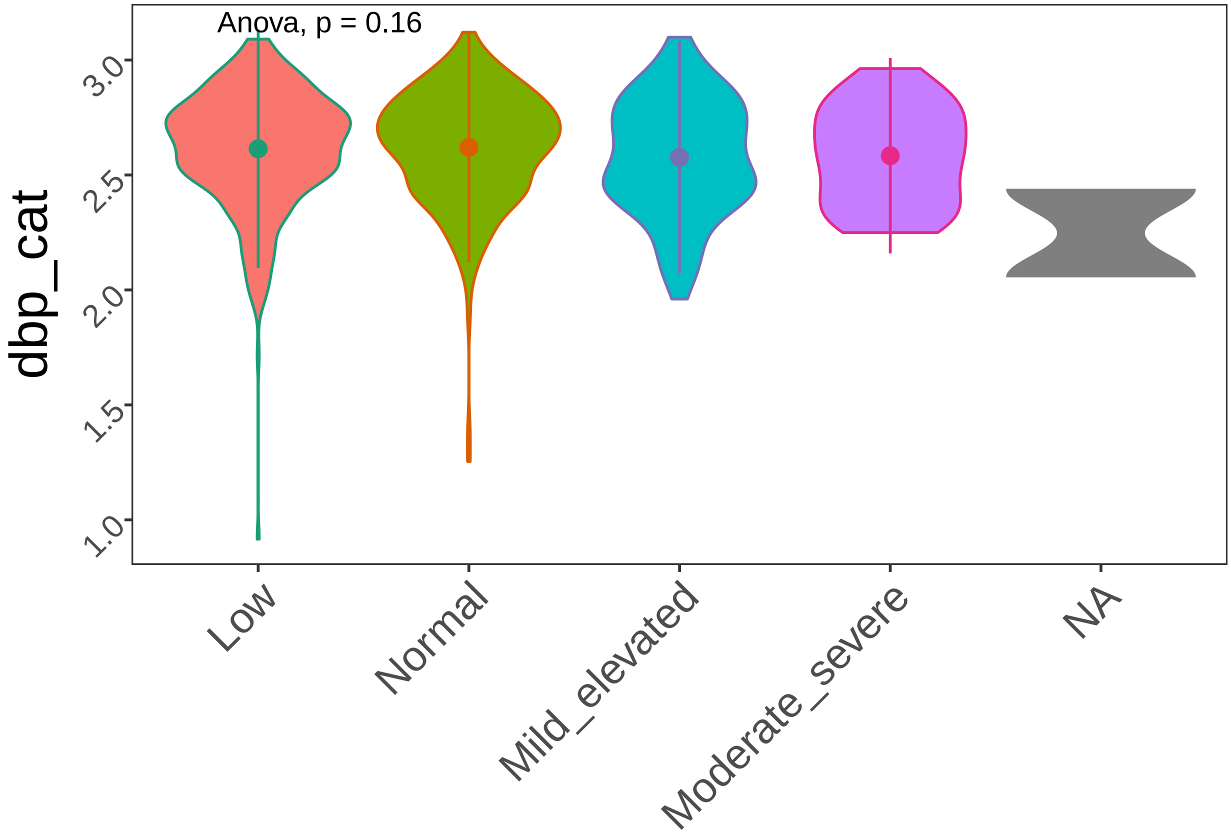


```
##
##  Kruskal-Wallis rank sum test
##
## data:  Shannon by dbp_cat
## Kruskal-Wallis chi-squared = 4.2245, df = 3, p-value = 0.2382
##
##
##  Pairwise comparisons using Wilcoxon rank sum test with continuity correction
##
## data:  Phe2$Shannon and Phe2[, which(colnames(Phe2) %in% i)]
##
##                 Low  Normal Mild_elevated
## Normal          1.00 -      -
## Mild_elevated   0.57 0.52   -
## Moderate_severe 1.00 1.00   1.00
##
## P value adjustment method: bonferroni
```

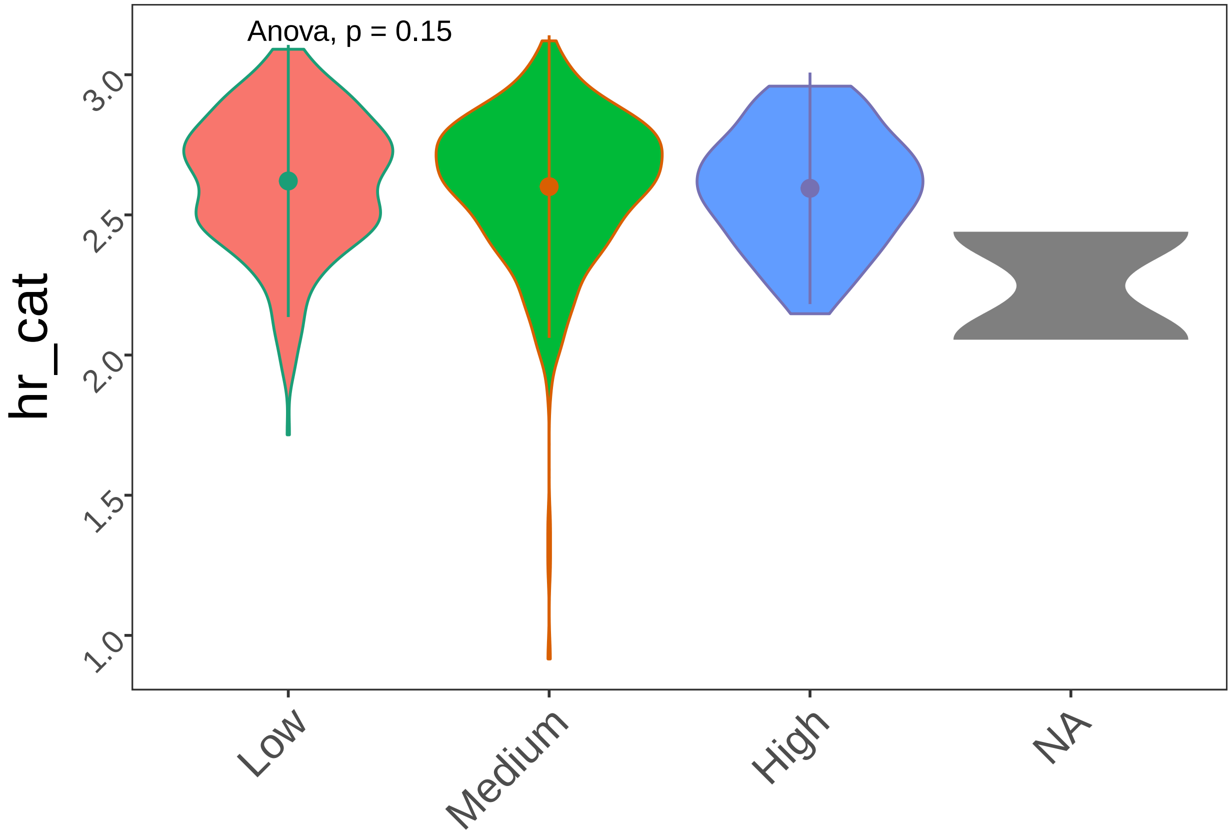


```
##
##  Kruskal-Wallis rank sum test
##
## data:  Shannon by hr_cat
## Kruskal-Wallis chi-squared = 1.182, df = 2, p-value = 0.5538
##
##
##  Pairwise comparisons using Wilcoxon rank sum test with continuity correction
##
## data:  Phe2$Shannon and Phe2[, which(colnames(Phe2) %in% i)]
##
##        Low  Medium
## Medium 1.00 -
## High   0.92 1.00
##
## P value adjustment method: bonferroni
```

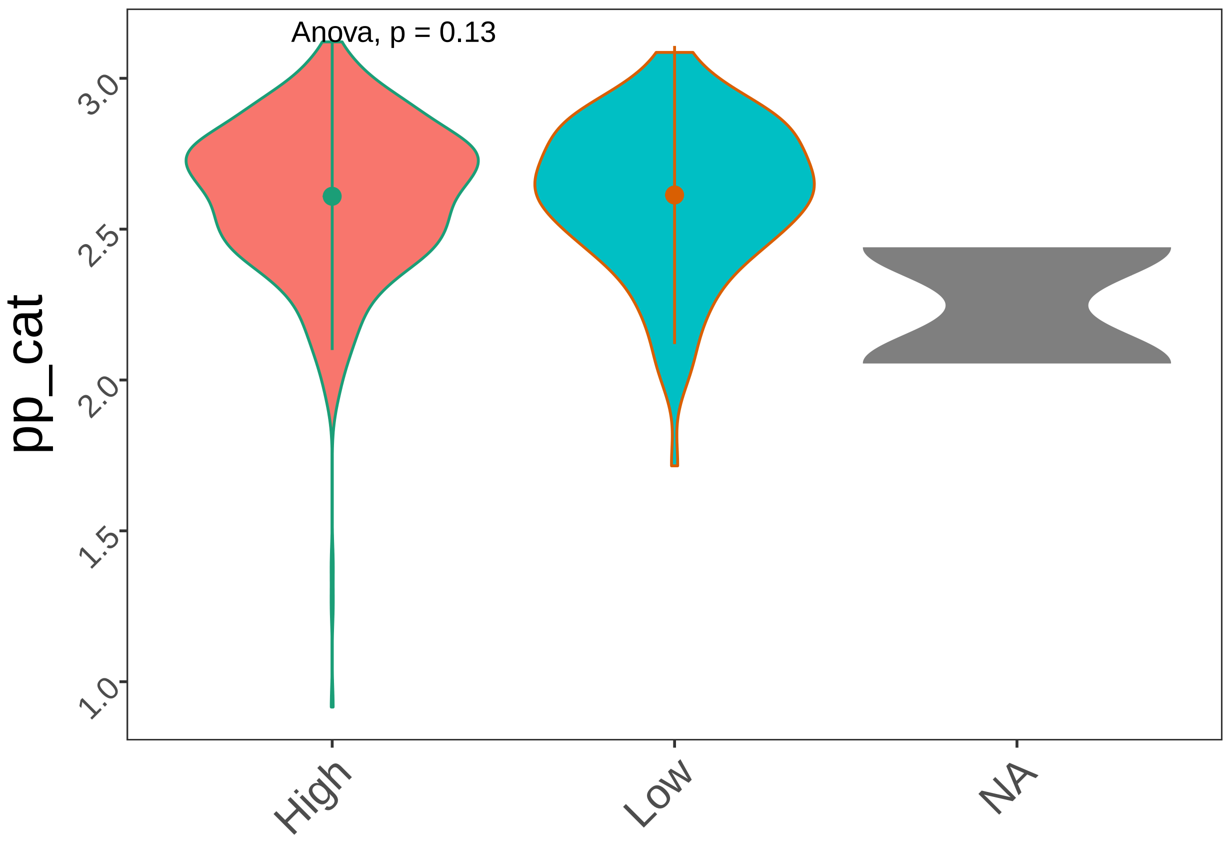


```
##
##  Kruskal-Wallis rank sum test
##
## data:  Shannon by pp_cat
## Kruskal-Wallis chi-squared = 0.039566, df = 1, p-value = 0.8423
##
##
##  Pairwise comparisons using Wilcoxon rank sum test with continuity correction
##
## data:  Phe2$Shannon and Phe2[, which(colnames(Phe2) %in% i)]
##
##     High
## Low 0.84
##
## P value adjustment method: bonferroni
```

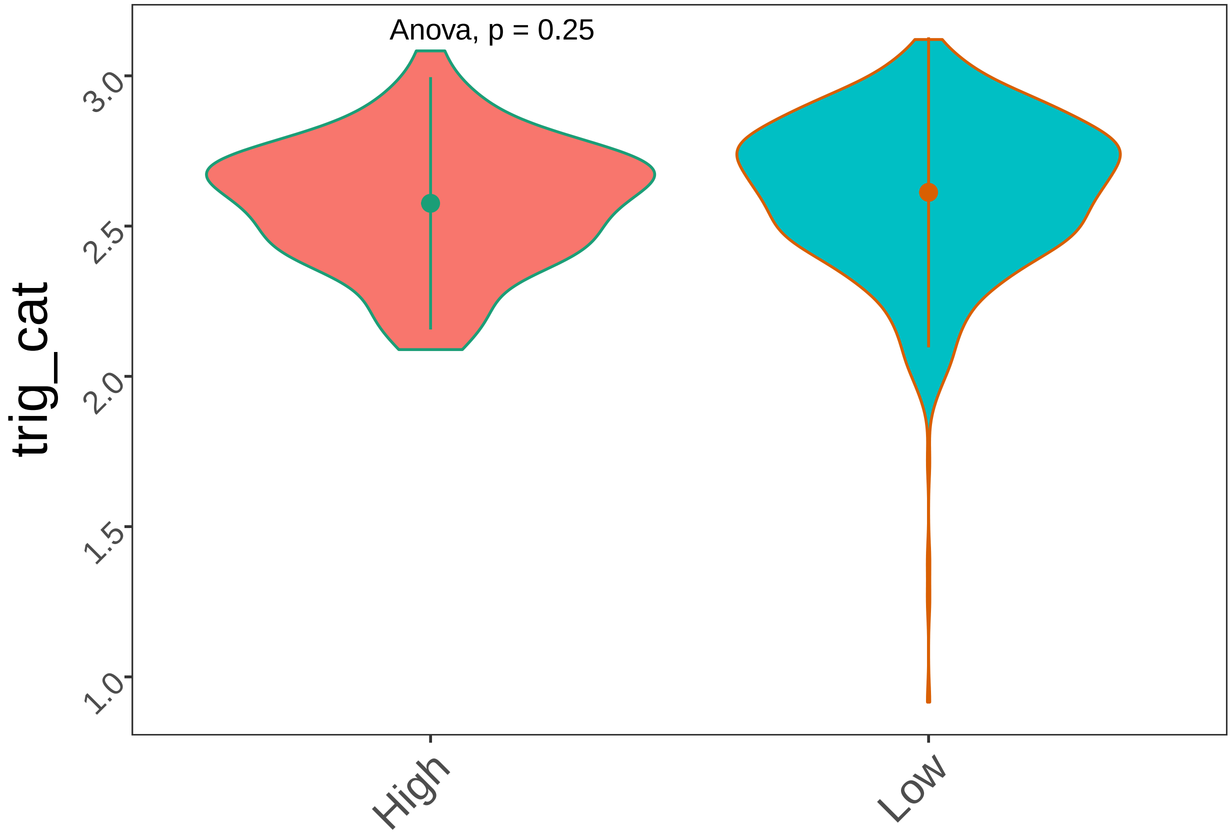


```
##
##  Kruskal-Wallis rank sum test
##
## data:  Shannon by trig_cat
## Kruskal-Wallis chi-squared = 3.1362, df = 1, p-value = 0.07657
##
##
##  Pairwise comparisons using Wilcoxon rank sum test with continuity correction
##
## data:  Phe2$Shannon and Phe2[, which(colnames(Phe2) %in% i)]
##
##     High
## Low 0.077
##
## P value adjustment method: bonferroni
```

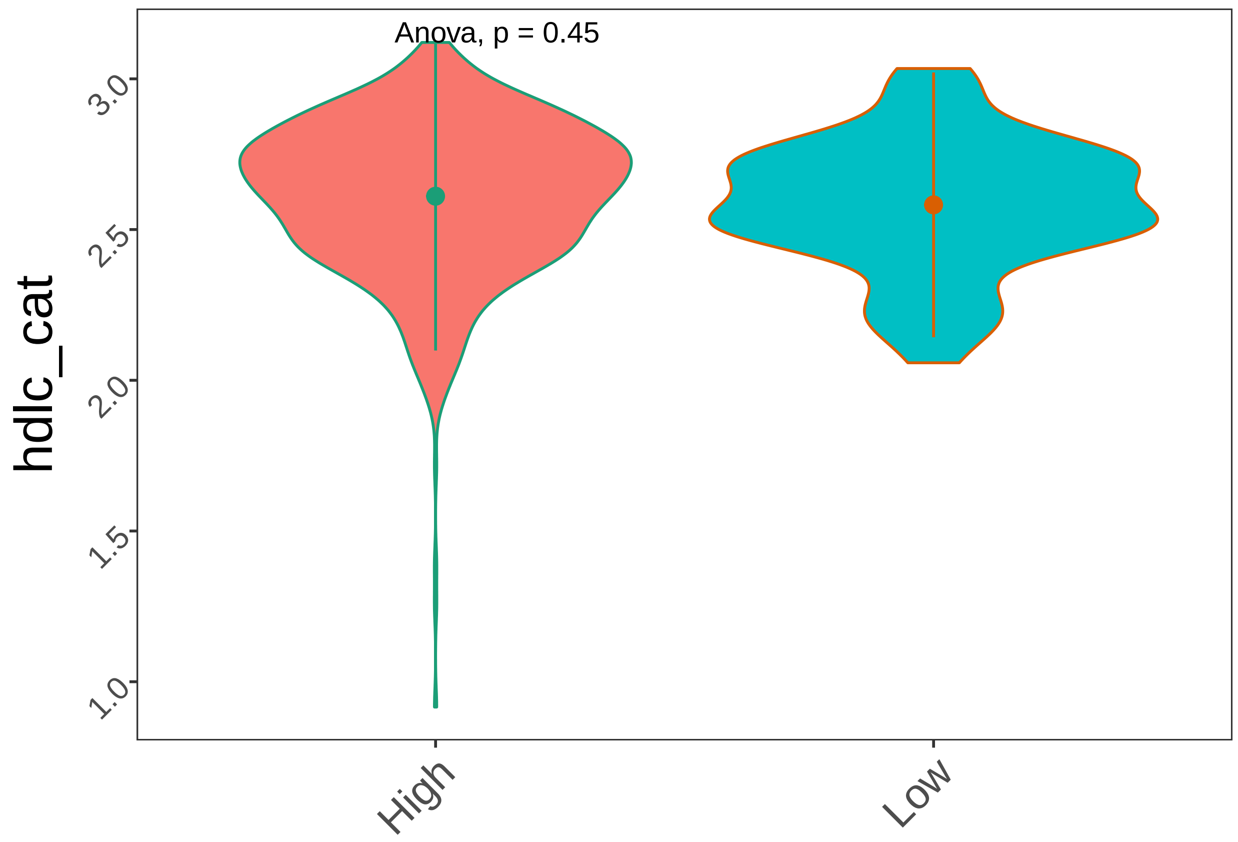


```
##
##  Kruskal-Wallis rank sum test
##
## data:  Shannon by hdlc_cat
## Kruskal-Wallis chi-squared = 1.3331, df = 1, p-value = 0.2483
##
##
##  Pairwise comparisons using Wilcoxon rank sum test with continuity correction
##
## data:  Phe2$Shannon and Phe2[, which(colnames(Phe2) %in% i)]
##
##     High
## Low 0.25
##
## P value adjustment method: bonferroni
```

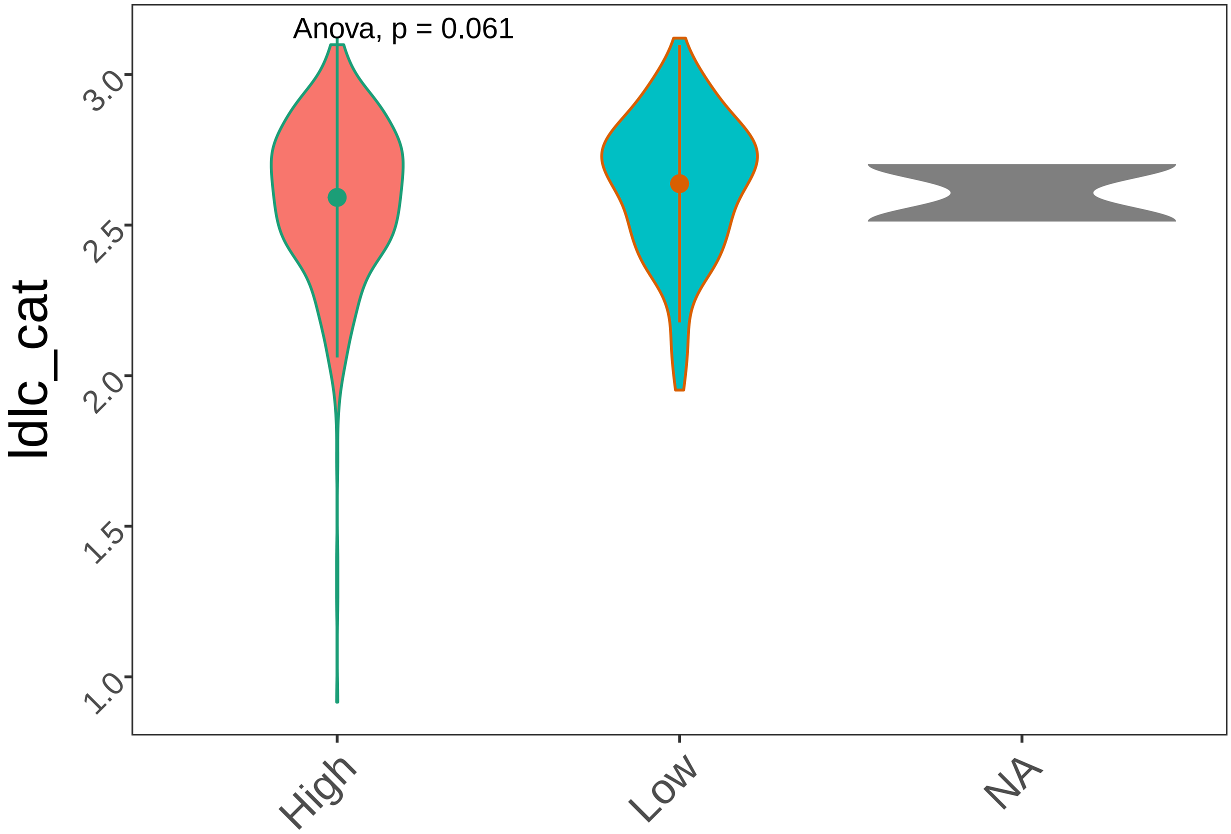


```
##
##  Kruskal-Wallis rank sum test
##
## data:  Shannon by ldlc_cat
## Kruskal-Wallis chi-squared = 4.578, df = 1, p-value = 0.03238
##
##
##  Pairwise comparisons using Wilcoxon rank sum test with continuity correction
##
## data:  Phe2$Shannon and Phe2[, which(colnames(Phe2) %in% i)]
##
##     High
## Low 0.032
##
## P value adjustment method: bonferroni
```

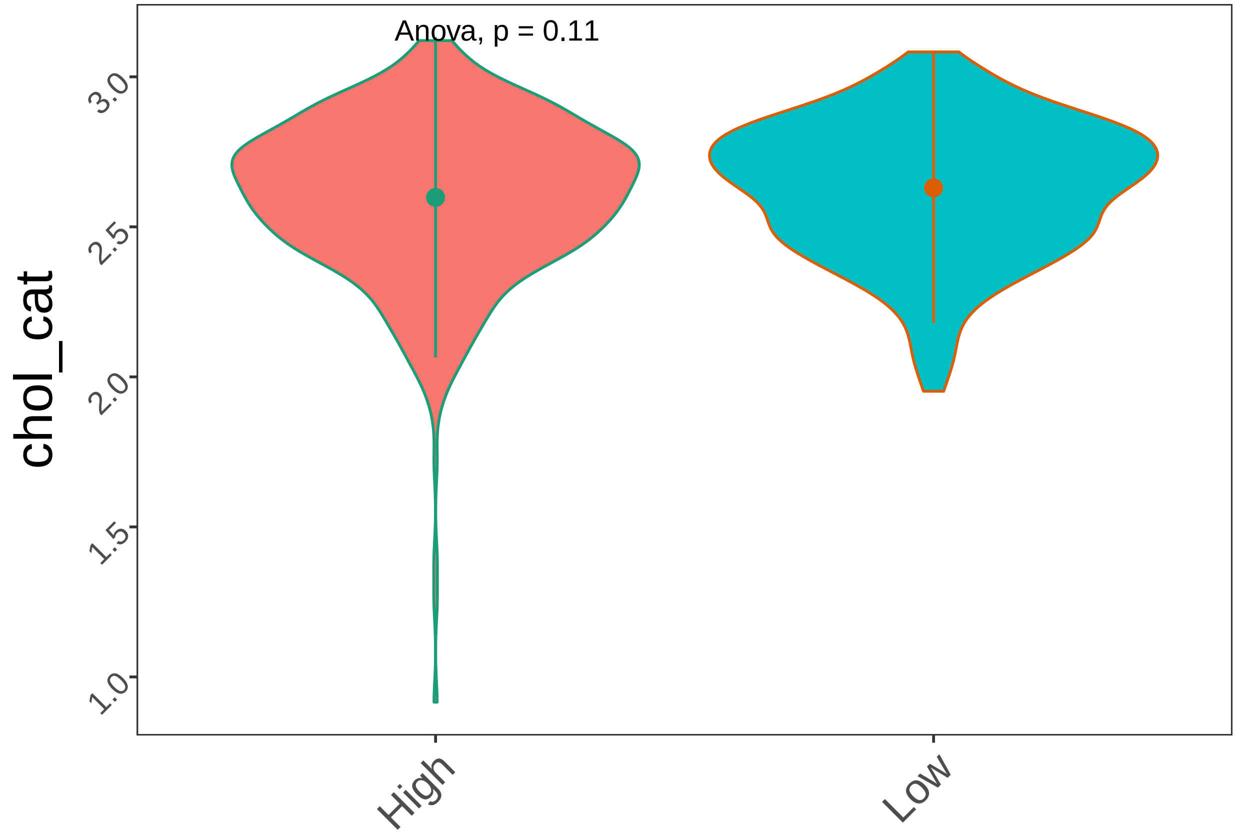


```
##
##  Kruskal-Wallis rank sum test
##
## data:  Shannon by chol_cat
## Kruskal-Wallis chi-squared = 1.6345, df = 1, p-value = 0.2011
##
##
##  Pairwise comparisons using Wilcoxon rank sum test with continuity correction
##
## data:  Phe2$Shannon and Phe2[, which(colnames(Phe2) %in% i)]
##
##     High
## Low 0.2
##
## P value adjustment method: bonferroni
```

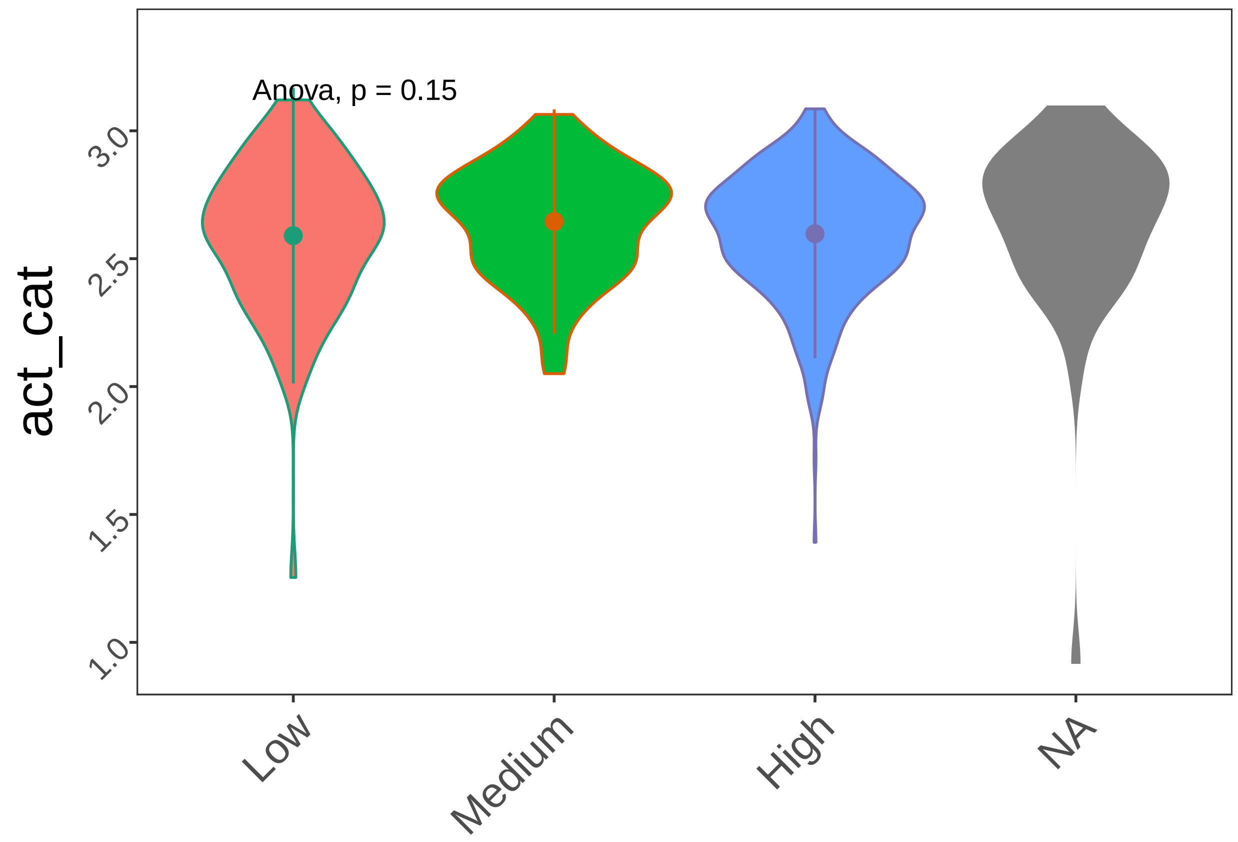


```
##
##  Kruskal-Wallis rank sum test
##
## data:  Shannon by act_cat
## Kruskal-Wallis chi-squared = 4.1929, df = 2, p-value = 0.1229
##
##
##  Pairwise comparisons using Wilcoxon rank sum test with continuity correction
##
## data:  Phe2$Shannon and Phe2[, which(colnames(Phe2) %in% i)]
##
##        Low  Medium
## Medium 0.43 -
## High   1.00 0.14
##
## P value adjustment method: bonferroni
```

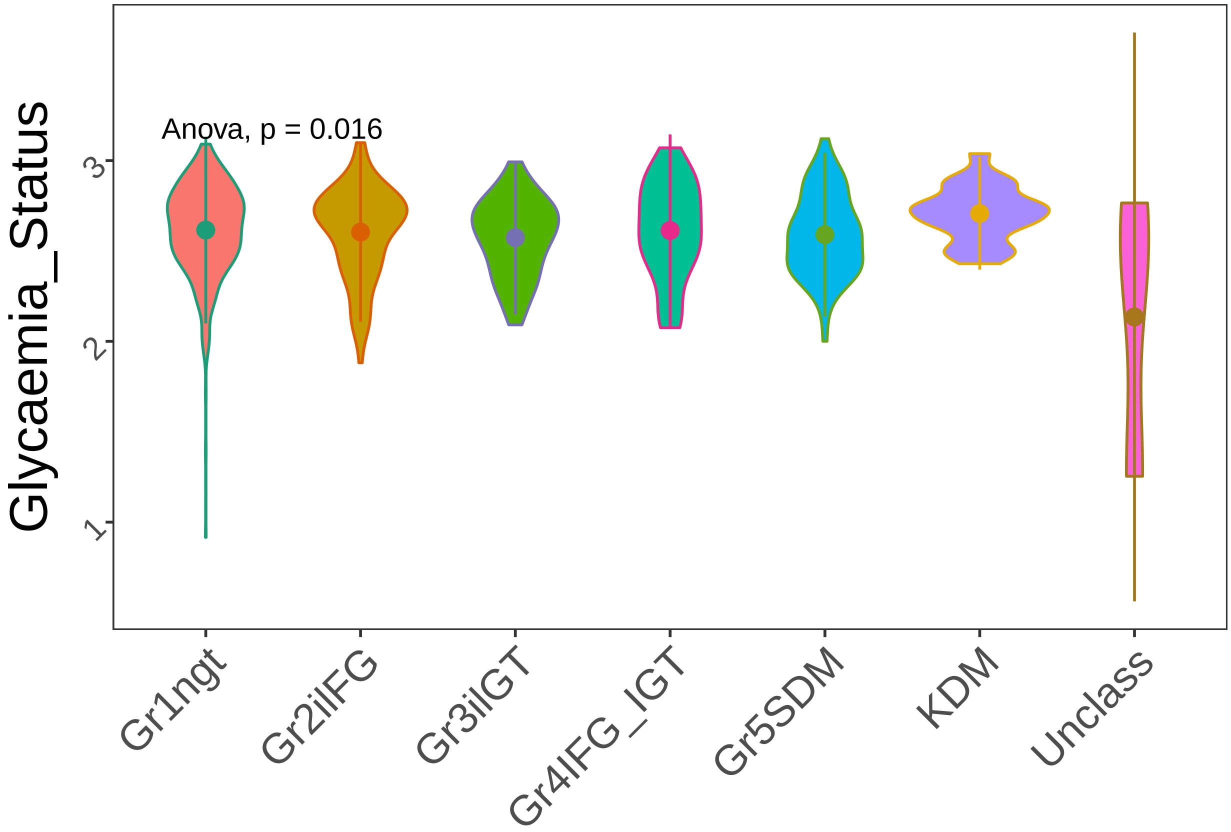


```
##
##  Kruskal-Wallis rank sum test
##
## data:  Shannon by Glycaemia_Status
## Kruskal-Wallis chi-squared = 8.759, df = 6, p-value = 0.1876
##
##
##  Pairwise comparisons using Wilcoxon rank sum test with continuity correction
##
## data:  Phe2$Shannon and Phe2[, which(colnames(Phe2) %in% i)]
##
##            Gr1ngt Gr2ilFG Gr3ilGT Gr4IFG_IGT Gr5SDM KDM
## Gr2ilFG    1.0    -       -       -          -      -
## Gr3ilGT    1.0    1.0     -       -          -      -
## Gr4IFG_IGT 1.0    1.0     1.0     -          -      -
## Gr5SDM     1.0    1.0     1.0     1.0        -      -
## KDM        1.0    1.0     0.3     1.0        0.4    -
## Unclass    1.0    1.0     1.0     1.0        1.0    1.0
##
## P value adjustment method: bonferroni
```

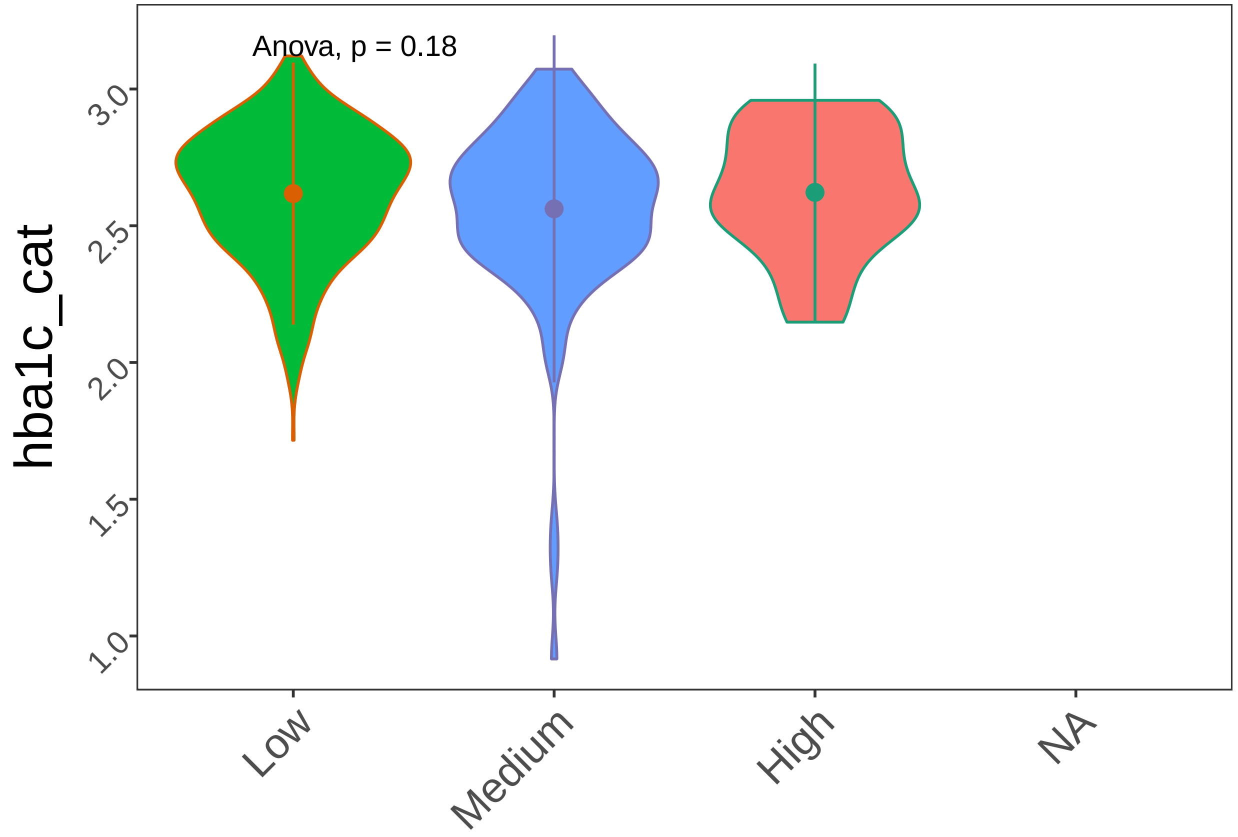


```
##
##  Kruskal-Wallis rank sum test
##
## data:  Shannon by hba1c_cat
## Kruskal-Wallis chi-squared = 2.7908, df = 2, p-value = 0.2477
##
##
##  Pairwise comparisons using Wilcoxon rank sum test with continuity correction
##
## data:  Phe2$Shannon and Phe2[, which(colnames(Phe2) %in% i)]
##
##        Low  Medium
## Medium 0.29 -
## High   1.00 1.00
##
## P value adjustment method: bonferroni
```

```
#Have the plots stored in list
lay <- rbind(c(1,2),
             c(3,4),
             c(5,6),
             c(7,8),
             c(9,10),
             c(11,12))
```

```
pdf(paste("ADDPRO_alpha_main.pdf", sep=""), width=15, height=30)
grid.arrange(AlphaList$AlpharichnessRisk,
             AlphaList$AlphaShannonRisk,
             AlphaList$AlpharichnessSmoking,
             AlphaList$AlphaShannonSmoking,
             AlphaList$Alpharichnesssex_cat,
             AlphaList$AlphaShannonsex_cat,
             AlphaList$AlpharichnessAlcprweek_cat,
             AlphaList$AlphaShannonAlcprweek_cat,
             AlphaList$Alpharichnesshba1c_cat,
             AlphaList$AlphaShannonhba1c_cat,
             AlphaList$Alpharichnessact_cat,
             AlphaList$AlphaShannonact_cat, layout_matrix = lay)
dev.off()
```

```
## pdf
##   2
```

```
#Have the plots stored in list
lay <- rbind(c(1,2),
             c(3,4),
             c(5,6),
             c(7,8),
             c(9,10),
             c(11,12),
             c(13,14),
             c(15,16),
             c(17,18),
             c(19,20),
             c(21,22),
             c(23,24))


pdf(paste("ADDPRO_alpha_secondary.pdf", sep=""), width=15, height=60)
grid.arrange(AlphaList$AlpharichnessBMI_cat,
             AlphaList$AlphaShannonBMI_cat,
             AlphaList$AlpharichnessWaist_cat,
             AlphaList$AlphaShannonWaist_cat,
             AlphaList$Alpharichnesswhratio_cat,
             AlphaList$AlphaShannonwhratio_cat,
             AlphaList$Alpharichnessbp_cat,
             AlphaList$AlphaShannonbp_cat,
             AlphaList$Alpharichnesssbp_cat,
             AlphaList$AlphaShannonsbp_cat,
             AlphaList$Alpharichnessdbp_cat,
             AlphaList$AlphaShannondbp_cat,
             AlphaList$Alpharichnesshr_cat,
             AlphaList$AlphaShannonhr_cat,
             AlphaList$Alpharichnesspp_cat,
             AlphaList$AlphaShannonpp_cat,
             AlphaList$Alpharichnesstrig_cat,
             AlphaList$AlphaShannontrig_cat,
             AlphaList$Alpharichnesshdlc_cat,
```

```
              AlphaList$AlphaShannonhdlc_cat,
              AlphaList$Alpharichnessldlc_cat,
              AlphaList$AlphaShannonldlc_cat,
              AlphaList$Alpharichnesschol_cat,
              AlphaList$AlphaShannonchol_cat, layout_matrix = lay)
dev.off()
```

```
## pdf
##   2
```

PCoA, rabuplot and PERMANOVA

```
rm(list=setdiff(ls(), c("FeaturePic2", "Pic2", "Phe", "Microbio", "FeatureMic", "Metabo", "paretoscale"[illegible]

i <- c("All")
Phe2 <- Phe

#Hellinger transformation
Microbio2 <- data.frame(t(decostand(t(Microbio), method="hellinger")))
#Maks TSS
Microbio2<-sweep(Microbio2, 2, colSums(Microbio2), FUN="/")
#Dissimilarity
distmatrix <- vegdist(t(Microbio2), method="bray")

#####New######
# #save dist matrix for Evelina
# test<-as.matrix(distmatrix)
# write.table(test, file="ADDPRO_Evelina_HelTSSBray.txt")
# #test2<-read.table("ADDPRO_Evelina_HelTSSBray.txt", header=TRUE, row.names=1)

#Multi dimensional scaling with capscale
PCoAcsObject<-capscale(distmatrix~1)


##Add eig to plot axes. with cmdscale there are negative values not with capscale
eig <- PCoAcsObject$CA$eig
# Calculate the variation explained by PCoA1, 2, 3 and 4
# and use it to generate axis labels
eig_1_2 <- eig[1:4] / sum(eig) * 100 #Vector with variance explained
#  by the first 4 axes
eig_1 <- paste("PCoA1", round(eig_1_2[1], digits = 2), "% variance")
eig_2 <- paste("PCoA2", round(eig_1_2[2], digits = 2), "% variance")
eig_3 <- paste("PCoA3", round(eig_1_2[3], digits = 2), "% variance")
eig_4 <- paste("PCoA4", round(eig_1_2[4], digits = 2), "% variance")

##Pull out coordinates for plotting from the ca object
#Structuring to add to Phe2
PCoACA<-PCoAcsObject$CA #The ca object contains the actual ordination results:
#u ((Weighted) orthonormal site scores),
#v ((Weighted) orthonormal species scores) all na in mine (unconstrained),
```

```
#Xbar (The standardized data matrix after previous stages of analysis),
#and imaginary.u.eig ???.
#Info http://cc.oulu.fi/~jarioksa/softhelp/vegan/html/cca.object.html
PCoA<-as.data.frame(PCoACA$u)
#Change colnames. Now add dis and trans info to names

colnames(PCoA) <- paste("MDS", 1:length(PCoA), "BrayHel", sep="")
#Add row names to df
PCoA$IDX <- row.names(PCoA)
#Merge according to Sample
Phe2<-merge(Phe2, PCoA, by="IDX")


Phe2$gly_stat<-as.factor(Phe2$gly_stat)
Phe2$p_gq_smoke<-as.factor(Phe2$p_gq_smoke)

# #PCoA MDS1 and MDS2 pdf
# pdf(paste("ADDPRO_Microbio_glystat_PCoA", i, ".pdf", sep=""), width=9, height=6)
# print(ggplot(Phe2) +
#   geom_point(aes(x=MDS1BrayHel, y=MDS2BrayHel, color = gly_stat,
#                  group = gly_stat), size=3) +
#   stat_ellipse(aes(MDS1BrayHel, y=MDS2BrayHel, color = gly_stat,
#                    group = gly_stat)) +
#   scale_color_manual(values=c("1"="#0000FF","2"="#FF0000", "3"="#228B22",
#                               "4"="#FFD700", "5"="#800080")) +
#   ggtitle(paste("PCoA", i, sep=" ")) +
#   labs(colour="gly_stat", x = eig_1, y = eig_2) +
#   theme_bw() +
#   theme(panel.grid.major = element_blank(), panel.grid.minor = element_blank(),
#         axis.title=element_text(size=12), legend.position="bottom"))
# dev.off()
#
# #PCoA MDS1 and MDS2 pdf
# pdf(paste("ADDPRO_Microbio_smoke_PCoA", i, ".pdf", sep=""), width=9, height=6)
# print(ggplot(Phe2[!is.na(Phe2$p_gq_smoke),]) + #Removed the samples wNAs smoke
#   geom_point(aes(x=MDS1BrayHel, y=MDS2BrayHel, color = p_gq_smoke,
#                  group = p_gq_smoke), size=3) +
#   stat_ellipse(aes(MDS1BrayHel, y=MDS2BrayHel, color = p_gq_smoke,
#                    group = p_gq_smoke)) +
#   scale_color_manual(values=c("1"="#0000FF","2"="#FF0000", "3"="#228B22")) +
#   ggtitle(paste("PCoA", i, sep=" ")) +
#   labs(colour="p_gq_smoke", x = eig_1, y = eig_2) +
#   theme_bw() +
#   theme(panel.grid.major = element_blank(), panel.grid.minor = element_blank(),
#         axis.title=element_text(size=12), legend.position="bottom"))
# dev.off()
#
# #coloring<-"p_gq_smoke" #Remember NAs are not plottet
# coloring<-"gly_stat"
#
# #PCoA MDS1 and MDS2
# print(ggplot(Phe2) +
#   geom_point(aes_string(x="MDS1BrayHel", y="MDS2BrayHel", color = coloring,
```

```
#                   group = coloring), size=3) +
#   stat_ellipse(aes_string("MDS1BrayHel", y="MDS2BrayHel", color = coloring,
#                     group = coloring)) +
#   scale_color_manual(values=c("1"="#0000FF","2"="#FF0000", "3"="#228B22",
#                                 "4"="#FFD700", "5"="#800080")) +
#   ggtitle(paste("PCoA", i, sep=" ")) +
#   labs(colour="gly_stat", x = eig_1, y = eig_2) +
#   theme_bw() +
#   theme(panel.grid.major = element_blank(), panel.grid.minor = element_blank(),
#         axis.title=element_text(size=12), legend.position="bottom"))
#
# #PCoA MDS1 and MDS3
# print(ggplot(Phe2) +
#   geom_point(aes_string(x="MDS1BrayHel", y="MDS3BrayHel", color = coloring,
#                   group = coloring), size=3) +
#   stat_ellipse(aes_string("MDS1BrayHel", y="MDS3BrayHel", color = coloring,
#                     group = coloring)) +
#   scale_color_manual(values=c("1"="#0000FF","2"="#FF0000", "3"="#228B22",
#                                 "4"="#FFD700", "5"="#800080")) +
#   ggtitle(paste("PCoA", i, sep=" ")) +
#   labs(colour="gly_stat", x = eig_1, y = eig_3) +
#   theme_bw() +
#   theme(panel.grid.major = element_blank(), panel.grid.minor = element_blank(),
#         axis.title=element_text(size=12), legend.position="bottom"))
#
# #PCoA MDS2 and MDS3
# print(ggplot(Phe2) +
#   geom_point(aes_string(x="MDS2BrayHel", y="MDS3BrayHel", color = coloring,
#                   group = coloring), size=3) +
#   stat_ellipse(aes_string("MDS2BrayHel", y="MDS3BrayHel", color = coloring,
#                     group = coloring)) +
#   scale_color_manual(values=c("1"="#0000FF","2"="#FF0000", "3"="#228B22",
#                                 "4"="#FFD700", "5"="#800080")) +
#   ggtitle(paste("PCoA", i, sep=" ")) +
#   labs(colour="gly_stat", x = eig_2, y = eig_3) +
#   theme_bw() +
#   theme(panel.grid.major = element_blank(), panel.grid.minor = element_blank(),
#         axis.title=element_text(size=12), legend.position="bottom"))
#
#
#
#
# #PCoA MDS1 and MDS2 pdf to poster Risk
# png(paste("ADDPROposter_Microbio_risk_PCoA", i, ".png", sep=""), width=1350, height=900)
# print(ggplot(Phe2) +
#   geom_point(aes(x=MDS1BrayHel, y=MDS2BrayHel, color = Risk,
#                   group = Risk), size=5) +
#   stat_ellipse(aes(MDS1BrayHel, y=MDS2BrayHel, color = Risk,
#                     group = Risk)) +
#   scale_color_manual(values=c(Low="#0000FF",High="#FF0000")) +
#   #ggtitle(paste("PCoA", i, sep=" ")) +
#   labs(colour="Risk", x = eig_1, y = eig_2, size=30) +
#   theme_bw() +
```

```r
#   theme(panel.grid.major = element_blank(), panel.grid.minor = element_blank(),
#         axis.title=element_text(size=30), legend.position="bottom",
#         axis.text=element_text(size=24), legend.text=element_text(size=24),
#         legend.title=element_text(size=30)))
# dev.off()



##NEW beta-diversity##
PCoAlist<-list()
adonislist<-list()
Rabulist<-list()
var<-c("Risk", "gly_stat", "sex_cat",
       "BMI_cat", "Waist_cat", "whratio_cat",
       "Alcprweek_cat", "Smoking", "bp_cat",
       "sbp_cat", "dbp_cat", "hr_cat",
       "pp_cat", "trig_cat", "hdlc_cat",
       "ldlc_cat", "chol_cat", "act_cat",
       "Glycaemia_Status", "hba1c_cat")


##Some massaging to perform rabuplot
readRDS("dada2/phyloseq.RDS") -> ps
#The rownames must match the sample names in the otu_table if you plan to combine them as a phyloseq-ob

rownames(Phe2)<-gsub("X", "", Phe2$IDX)
ps2<-prune_samples(rownames(Phe2), ps)
#colnames(otu_table(ps2))==rownames(Phe2)
sample_data(ps2)<-Phe2

#Make ps2 according to DAtest. PCoA and PERMANOVA on all genera
ps2<-tax_glom(ps2, "Genus")
ps2<-filter_phy(ps2, abundance=0, prevalence=20)
```

Microbiome in loop

```
## [1] "presence/absence"
## [1] "x/max(sum(x))"
```

```r
ps2
```

```
## phyloseq-class experiment-level object
## otu_table()   OTU Table:         [ 96 taxa and 746 samples ]
## sample_data() Sample Data:       [ 746 samples by 474 sample variables ]
## tax_table()   Taxonomy Table:    [ 96 taxa by 7 taxonomic ranks ]
## refseq()      DNAStringSet:      [ 96 reference sequences ]
```

```r
for (i in var) {
  print(i)
  plotvar<-ggplot(Phe2, aes_string(x="MDS1BrayHel", y="MDS2BrayHel", color = i, group=i), size=5) +
    geom_point() +
    stat_ellipse() +
```

```
    ggtitle(i) +
    #labs(colour="Risk", x = eig_1, y = eig_2, size=30) +
    theme_bw() #+
    #theme(panel.grid.major = element_blank(), panel.grid.minor = element_blank(),
    #      axis.title=element_text(size=30), legend.position="bottom",
    #      axis.text=element_text(size=24), legend.text=element_text(size=24),
    #      legend.title=element_text(size=30))
  PCoAlist[[i]] <- plotvar
  print(plotvar)

  #Remove NAs for adonis
  #Only includes rows without NAs from the i'th column
  Phe3<-Phe2[complete.cases(Phe2[,which( colnames(Phe2)==i )]),]
  #Also subset columns
  Microbio3<-dplyr::select(Microbio, one_of(Phe3$IDX))
  #Hellinger transformation
  Microbio3 <- data.frame(t(decostand(t(Microbio3), method="hellinger")))
  #Maks TSS
  Microbio3<-sweep(Microbio3, 2, colSums(Microbio3), FUN="/")
  #Dissimilarity
  distmatrix <- vegdist(t(Microbio3), method="bray")
  set.seed(1)
  adonisObject<-adonis2(formula(paste("distmatrix ~ ", i)), Phe3, by="terms",
                      perm=999) #, perm=99 can increase to get exact p-values
  adonislist[[i]]<-adonisObject
  print(adonisObject) #If significant then difference between groups

  #Stats are non-parametric
  print(rabuplot(ps2, i, p_adjust=T, N_taxa=20, p_adjust_full=T))
  Rabulist[[i]] <- rabuplot(ps2, i, p_adjust=T, N_taxa=20)
  print(rabuplot(ps2, i, p_adjust=T, N_taxa=500, Only_sig=T, p_adjust_full=T))
  Rabulist[[paste("Sig", i, sep="")]] <- rabuplot(ps2, i, p_adjust=T, N_taxa=500, Only_sig=T)
  if (i=="Smoking") {
      print(rabuplot(ps2, i, p_adjust=T, N_taxa=30, Only_sig=T, p_adjust_full=T))
      Rabulist[[paste("Sigmod", i, sep="")]] <- rabuplot(ps2, i, p_adjust=T, N_taxa=30, Only_sig=T)
  }
  #test<-rabuplot(ps2, i, p_adjust=T, N_taxa=30, Only_sig=T)
  print(rabuplot(ps2, i, N_taxa=10, bar_chart=TRUE, bar_chart_stacked=FALSE, percent=TRUE))
  #Rabulist[[paste("Barpercent", i, sep="")]] <- rabuplot(ps2, i, N_taxa=10, bar_chart=TRUE, bar_chart_

  ##Stats are metagenomeseq as assessed in DAtest
  #print(rabuplot(ps2, i, p_adjust=T, N_taxa=10, stats="mgs_feature", p_adjust_full=T))
  #print(rabuplot(ps2, i, p_adjust=T, N_taxa=500, stats="mgs_feature", Only_sig=T, p_adjust_full=T))

  print(rabuplot(ps2, i, N_taxa=20, bar_chart_stacked=TRUE))
  Rabulist[[paste("Bar", i, sep="")]] <- rabuplot(ps2, i, N_taxa=20, bar_chart_stacked=TRUE)

}
```

```
## [1] "Risk"
```

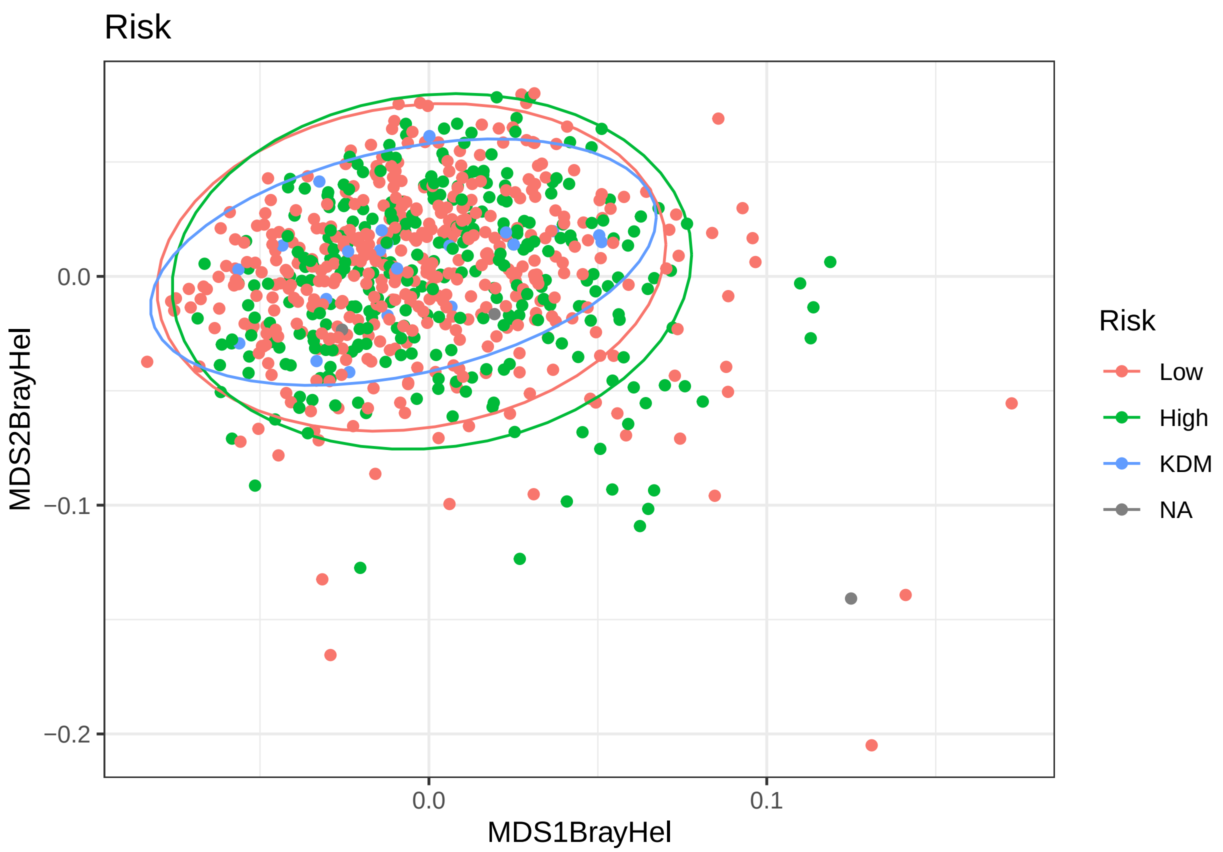


```
## Permutation test for adonis under reduced model
## Terms added sequentially (first to last)
## Permutation: free
## Number of permutations: 999
##
## adonis2(formula = formula(paste("distmatrix ~ ", i)), data = Phe3, permutations = 999, by = "terms")
##           Df SumOfSqs     R2      F Pr(>F)
## Risk       2   0.1138 0.0043 1.5997  0.043 *
## Residual 740  26.3240 0.9957
## Total    742  26.4378 1.0000
## ---
## Signif. codes:  0 '***' 0.001 '**' 0.01 '*' 0.05 '.' 0.1 ' ' 1
```

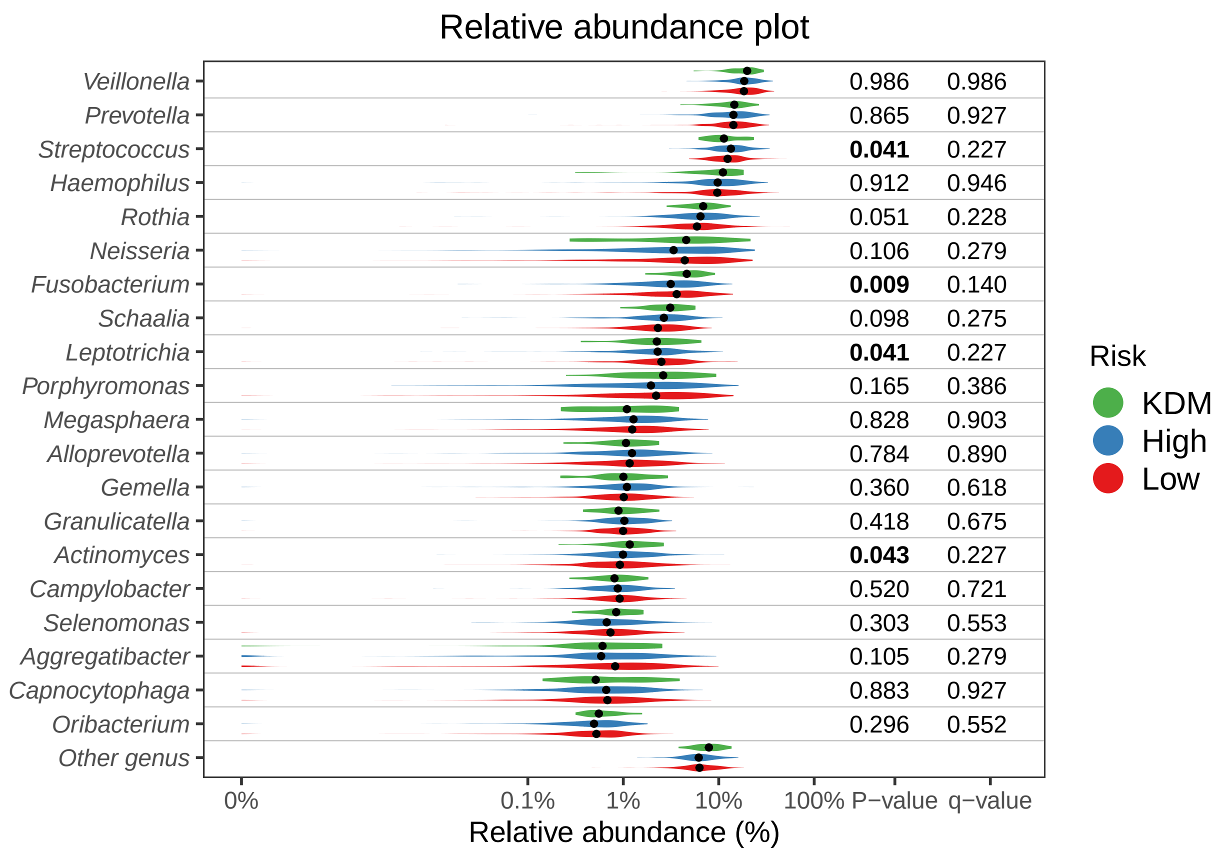
Relative abundance plot
Veillonella
Prevotella
Streptococcus
Haemophilus
Rothia
Neisseria
Fusobacterium
Schaalia
Leptotrichia
Porphyromonas
Megasphaera
Alloprevotella
Gemella
Granulicatella
Actinomyces
Campylobacter
Selenomonas
Aggregatibacter
Capnocytophaga
Oribacterium
Other genus
0.986 0.986
0.865 0.927
0.041 0.227
0.912 0.946
0.051 0.228
0.106 0.279
0.009 0.140
0.098 0.275
0.041 0.227
0.165 0.386
0.828 0.903
0.784 0.890
0.360 0.618
0.418 0.675
0.043 0.227
0.520 0.721
0.303 0.553
0.105 0.279
0.883 0.927
0.296 0.552
0%
0.1%
1%
10%
100%
P−value
q−value
Relative abundance (%)
Risk
KDM
High
Low

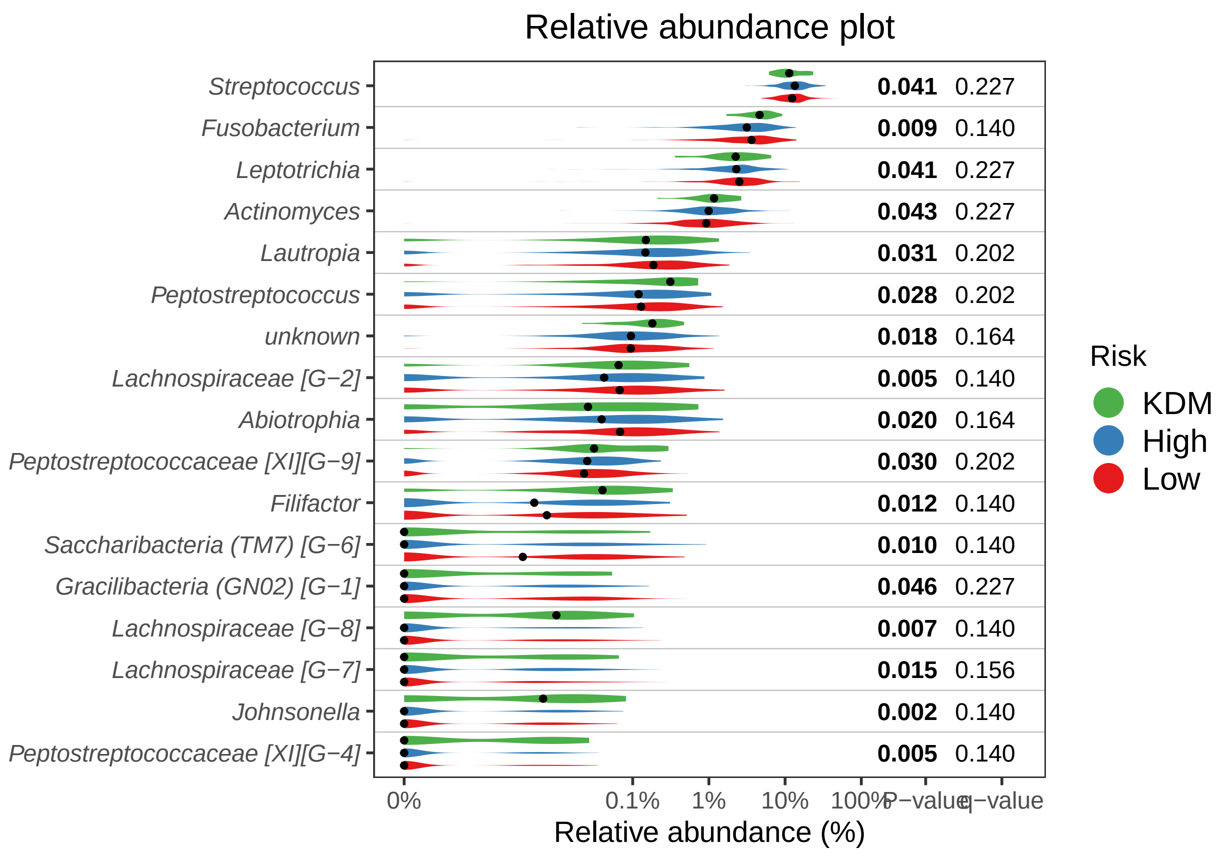

Relative abundance plot
Streptococcus
Fusobacterium
Leptotrichia
Actinomyces
Lautropia
Peptostreptococcus
unknown
Lachnospiraceae [G−2]
Abiotrophia
Peptostreptococcaceae [XI][G−9]
Filifactor
Saccharibacteria (TM7) [G−6]
Gracilibacteria (GN02) [G−1]
Lachnospiraceae [G−8]
Lachnospiraceae [G−7]
Johnsonella
Peptostreptococcaceae [XI][G−4]
0.041 0.227
0.009 0.140
0.041 0.227
0.043 0.227
0.031 0.202
0.028 0.202
0.018 0.164
0.005 0.140
0.020 0.164
0.030 0.202
0.012 0.140
0.010 0.140
0.046 0.227
0.007 0.140
0.015 0.156
0.002 0.140
0.005 0.140
0% 0.1% 1% 10% 100%
P−value
q−value
Relative abundance (%)
Risk
KDM
High
Low

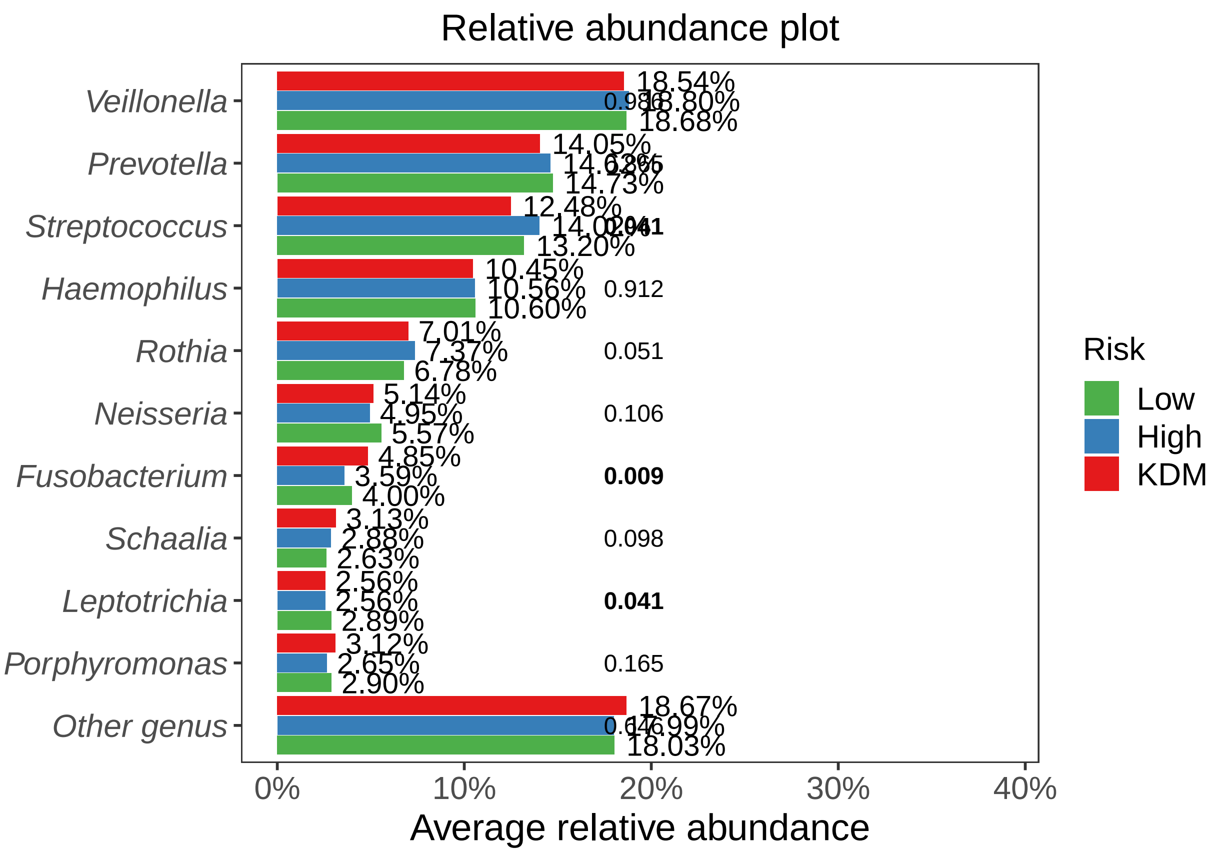
Relative abundance plot
Veillonella
Prevotella
Streptococcus
Haemophilus
Rothia
Neisseria
Fusobacterium
Schaalia
Leptotrichia
Porphyromonas
Other genus
18.54%
18.80%
18.68%
14.05%
14.73%
12.48%
13.20%
10.45%
10.56%
10.60%
0.912
7.01%
7.37%
6.78%
0.051
5.14%
4.95%
5.57%
0.106
4.85%
3.59%
4.00%
0.009
3.13%
2.88%
2.63%
0.098
2.56%
2.56%
2.89%
0.041
3.12%
2.65%
2.90%
0.165
18.67%
16.99%
18.03%
0%
10%
20%
30%
40%
Average relative abundance
Risk
Low
High
KDM

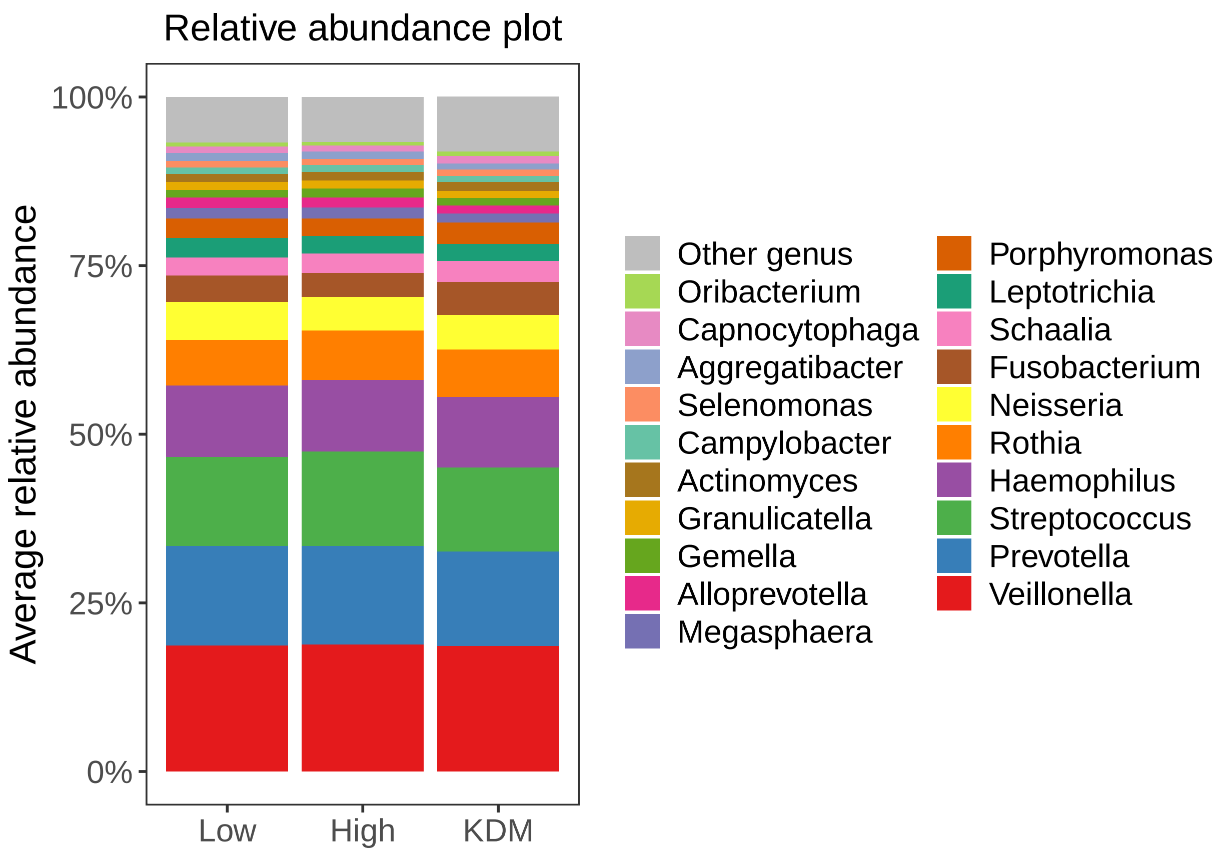
Relative abundance plot
Average relative abundance
100%
75%
50%
25%
0%
Low
High
KDM
Other genus
Oribacterium
Capnocytophaga
Aggregatibacter
Selenomonas
Campylobacter
Actinomyces
Granulicatella
Gemella
Alloprevotella
Megasphaera
Porphyromonas
Leptotrichia
Schaalia
Fusobacterium
Neisseria
Rothia
Haemophilus
Streptococcus
Prevotella
Veillonella

```
## [1] "gly_stat"
```

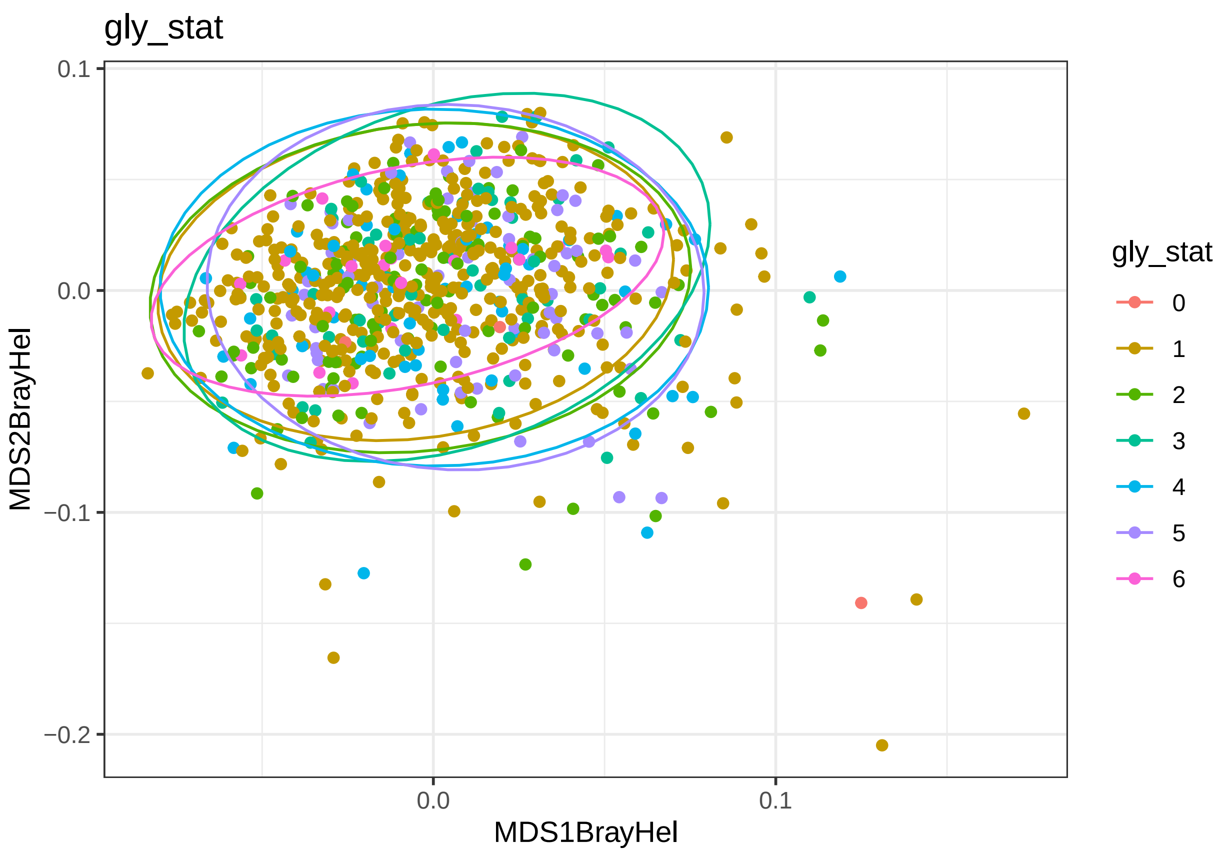


```
## Permutation test for adonis under reduced model
## Terms added sequentially (first to last)
## Permutation: free
## Number of permutations: 999
##
## adonis2(formula = formula(paste("distmatrix ~ ", i)), data = Phe3, permutations = 999, by = "terms")
##           Df SumOfSqs      R2      F Pr(>F)
## gly_stat   6   0.3601 0.01339 1.6714  0.005 **
## Residual 739  26.5323 0.98661
## Total    745  26.8923 1.00000
## ---
## Signif. codes:  0 '***' 0.001 '**' 0.01 '*' 0.05 '.' 0.1 ' ' 1
```

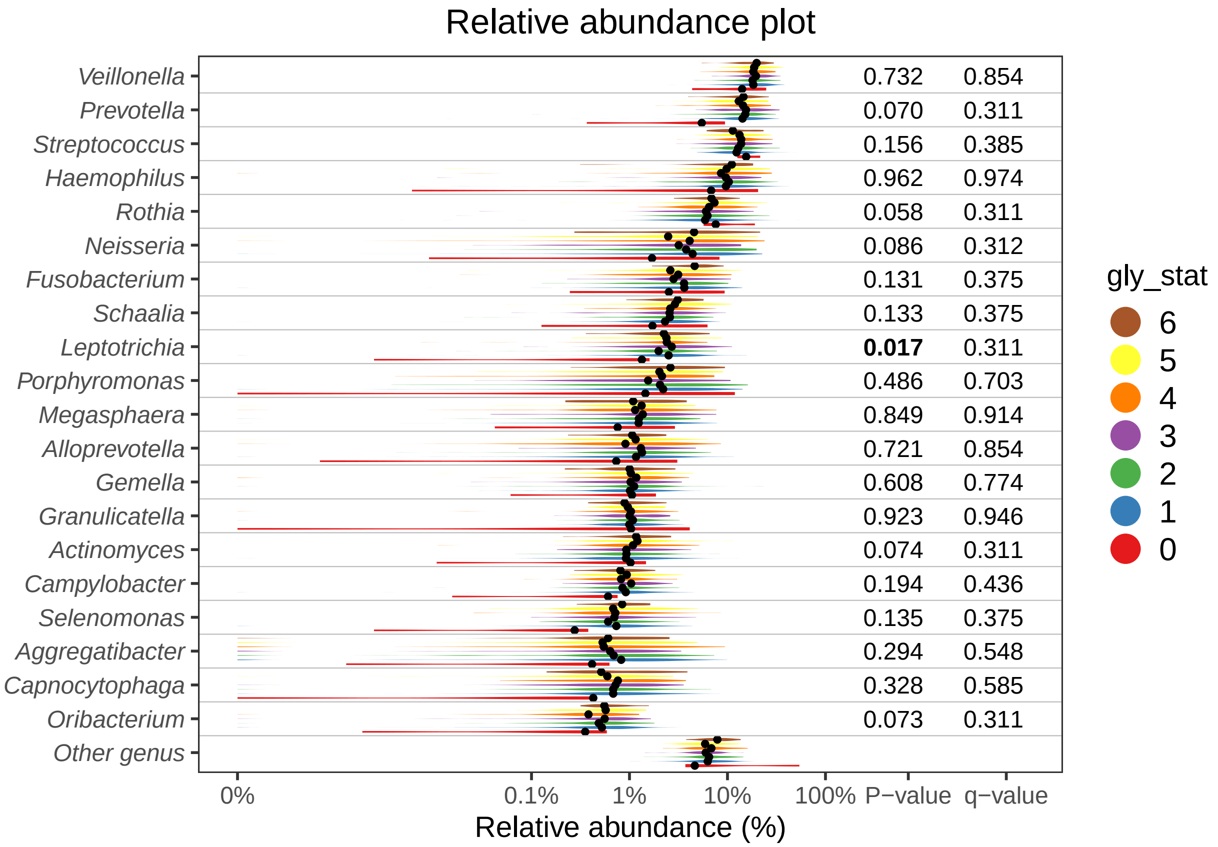

Relative abundance plot
Veillonella
Prevotella
Streptococcus
Haemophilus
Rothia
Neisseria
Fusobacterium
Schaalia
Leptotrichia
Porphyromonas
Megasphaera
Alloprevotella
Gemella
Granulicatella
Actinomyces
Campylobacter
Selenomonas
Aggregatibacter
Capnocytophaga
Oribacterium
Other genus
0.732 0.854
0.070 0.311
0.156 0.385
0.962 0.974
0.058 0.311
0.086 0.312
0.131 0.375
0.133 0.375
0.017 0.311
0.486 0.703
0.849 0.914
0.721 0.854
0.608 0.774
0.923 0.946
0.074 0.311
0.194 0.436
0.135 0.375
0.294 0.548
0.328 0.585
0.073 0.311
0%
0.1%
1%
10%
100%
P−value
q−value
Relative abundance (%)
gly_stat
6
5
4
3
2
1
0

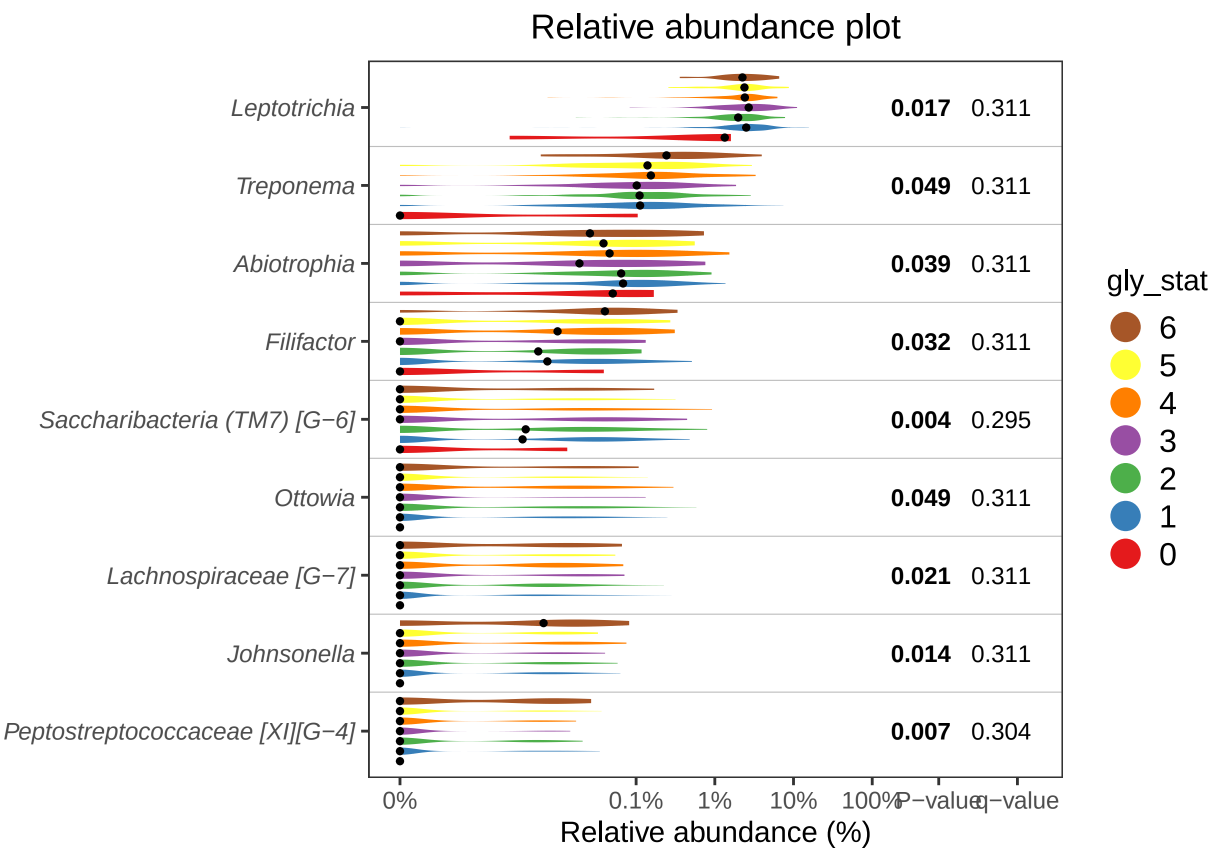
Relative abundance plot
Leptotrichia
Treponema
Abiotrophia
Filifactor
Saccharibacteria (TM7) [G−6]
Ottowia
Lachnospiraceae [G−7]
Johnsonella
Peptostreptococcaceae [XI][G−4]
0.017 0.311
0.049 0.311
0.039 0.311
0.032 0.311
0.004 0.295
0.049 0.311
0.021 0.311
0.014 0.311
0.007 0.304
0%
0.1%
1%
10%
100%
P−value
q−value
Relative abundance (%)
gly_stat
6
5
4
3
2
1
0

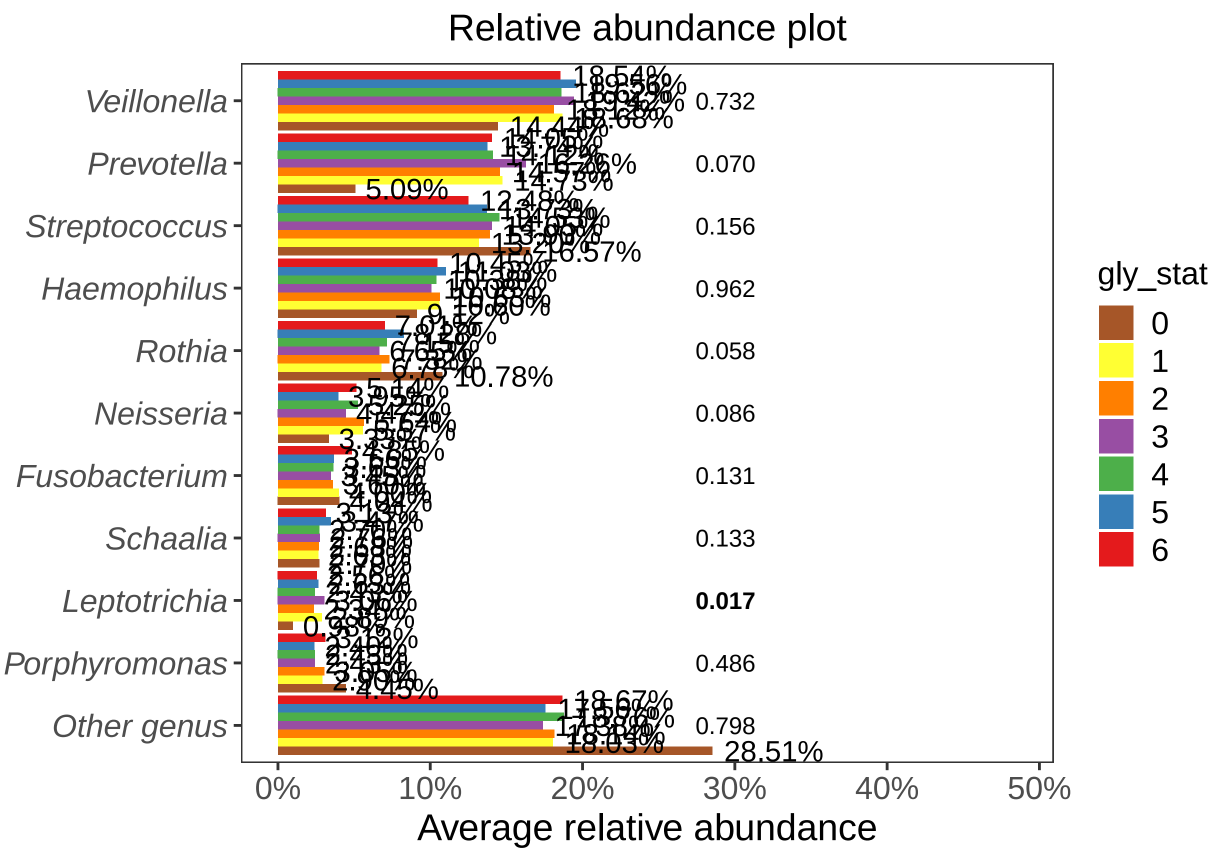
Relative abundance plot
Veillonella
Prevotella
Streptococcus
Haemophilus
Rothia
Neisseria
Fusobacterium
Schaalia
Leptotrichia
Porphyromonas
Other genus
0.732
0.070
0.156
0.962
0.058
0.086
0.131
0.133
0.017
0.486
0.798
5.09%
16.57%
10.78%
28.51%
0%
10%
20%
30%
40%
50%
Average relative abundance
gly_stat
0
1
2
3
4
5
6

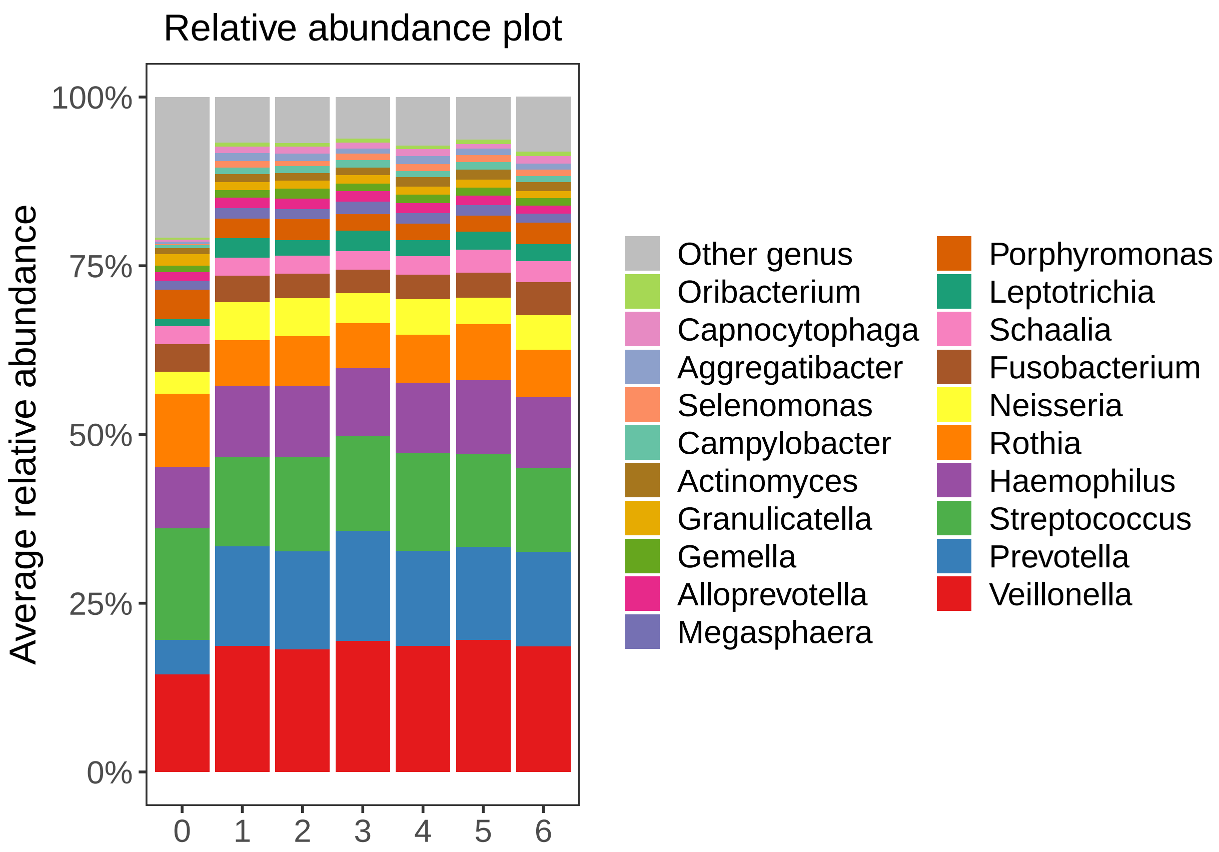
Relative abundance plot
Average relative abundance
100%
75%
50%
25%
0%
0
1
2
3
4
5
6
Other genus
Oribacterium
Capnocytophaga
Aggregatibacter
Selenomonas
Campylobacter
Actinomyces
Granulicatella
Gemella
Alloprevotella
Megasphaera
Porphyromonas
Leptotrichia
Schaalia
Fusobacterium
Neisseria
Rothia
Haemophilus
Streptococcus
Prevotella
Veillonella

```
## [1] "sex_cat"
```

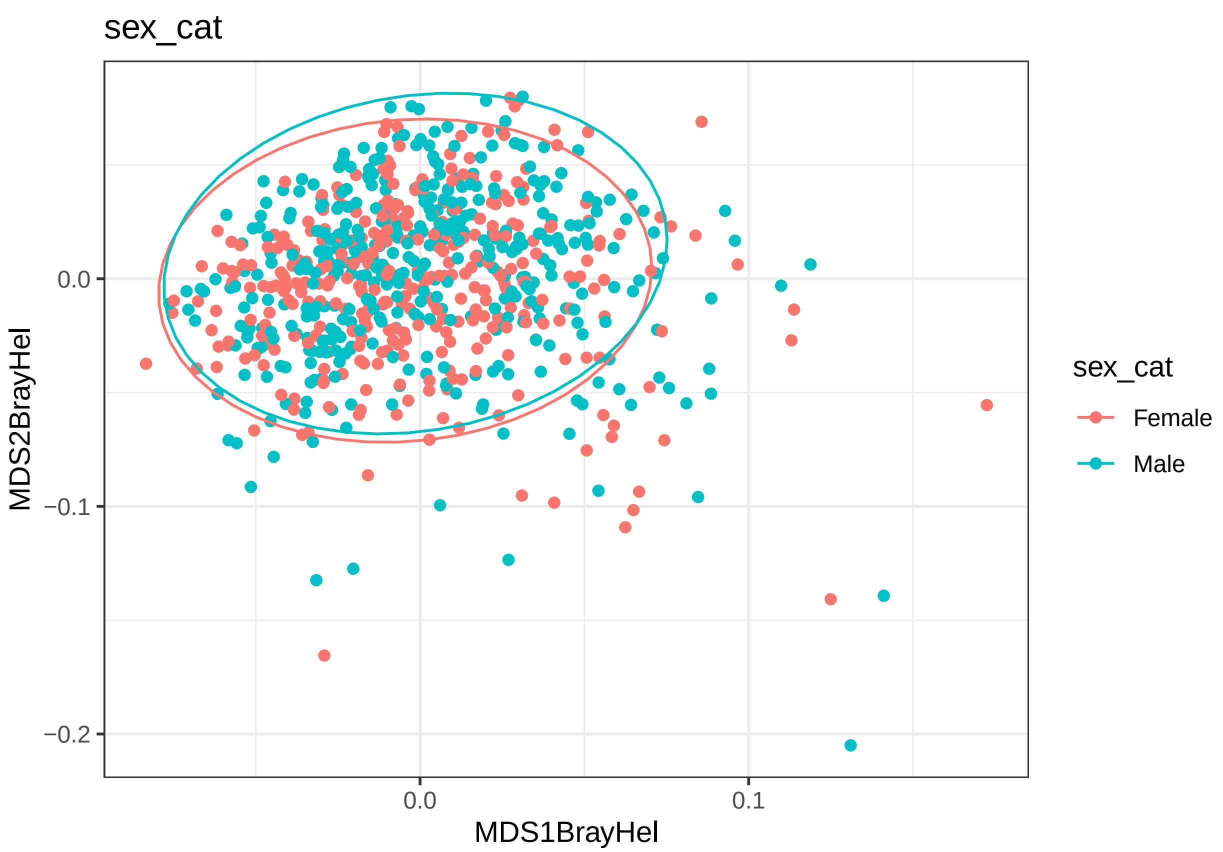


```
## Permutation test for adonis under reduced model
## Terms added sequentially (first to last)
## Permutation: free
## Number of permutations: 999
##
## adonis2(formula = formula(paste("distmatrix ~ ", i)), data = Phe3, permutations = 999, by = "terms")
##           Df SumOfSqs      R2      F Pr(>F)
## sex_cat    1   0.0899 0.00334 2.4955  0.011 *
## Residual 744  26.8024 0.99666
## Total    745  26.8923 1.00000
## ---
## Signif. codes:  0 '***' 0.001 '**' 0.01 '*' 0.05 '.' 0.1 ' ' 1
```

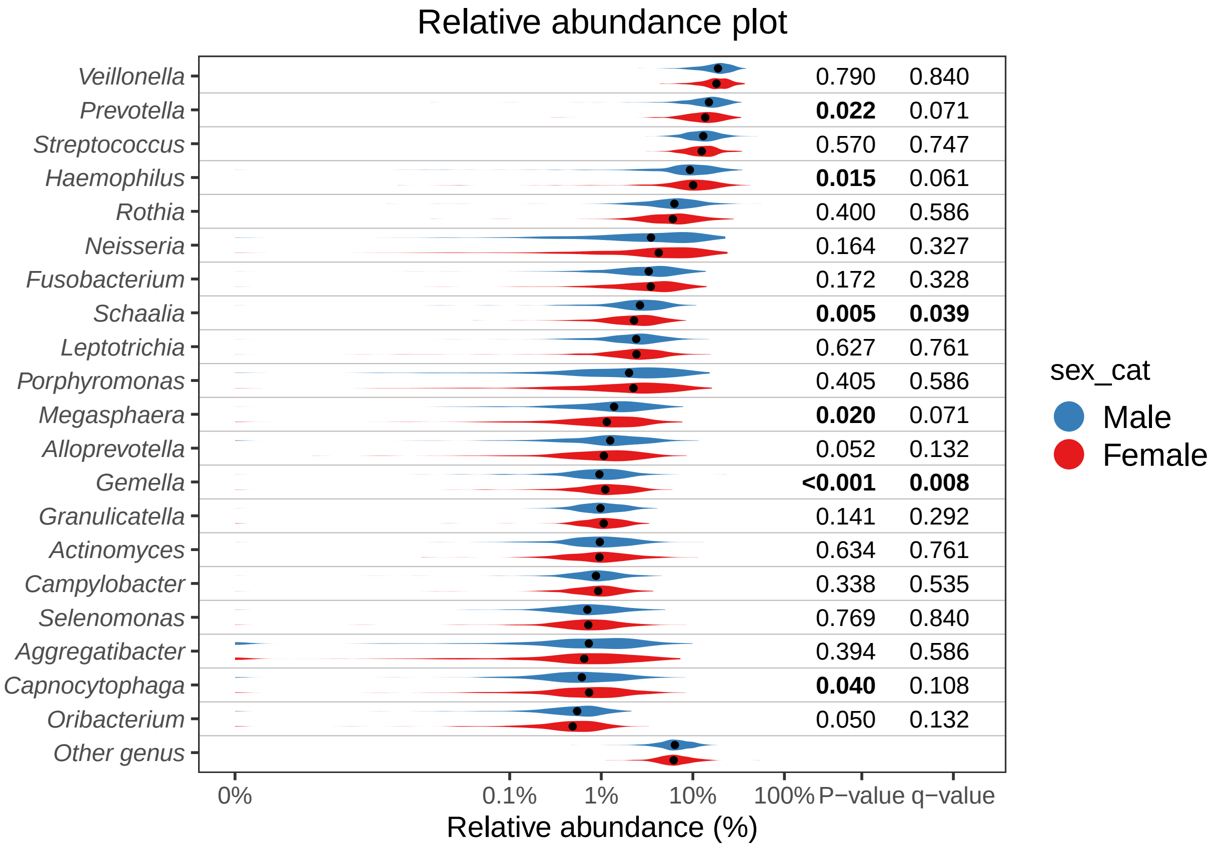

Relative abundance plot
Veillonella
Prevotella
Streptococcus
Haemophilus
Rothia
Neisseria
Fusobacterium
Schaalia
Leptotrichia
Porphyromonas
Megasphaera
Alloprevotella
Gemella
Granulicatella
Actinomyces
Campylobacter
Selenomonas
Aggregatibacter
Capnocytophaga
Oribacterium
Other genus
0.790 0.840
0.022 0.071
0.570 0.747
0.015 0.061
0.400 0.586
0.164 0.327
0.172 0.328
0.005 0.039
0.627 0.761
0.405 0.586
0.020 0.071
0.052 0.132
<0.001 0.008
0.141 0.292
0.634 0.761
0.338 0.535
0.769 0.840
0.394 0.586
0.040 0.108
0.050 0.132
0% 0.1% 1% 10% 100% P−value q−value
Relative abundance (%)
sex_cat
Male
Female

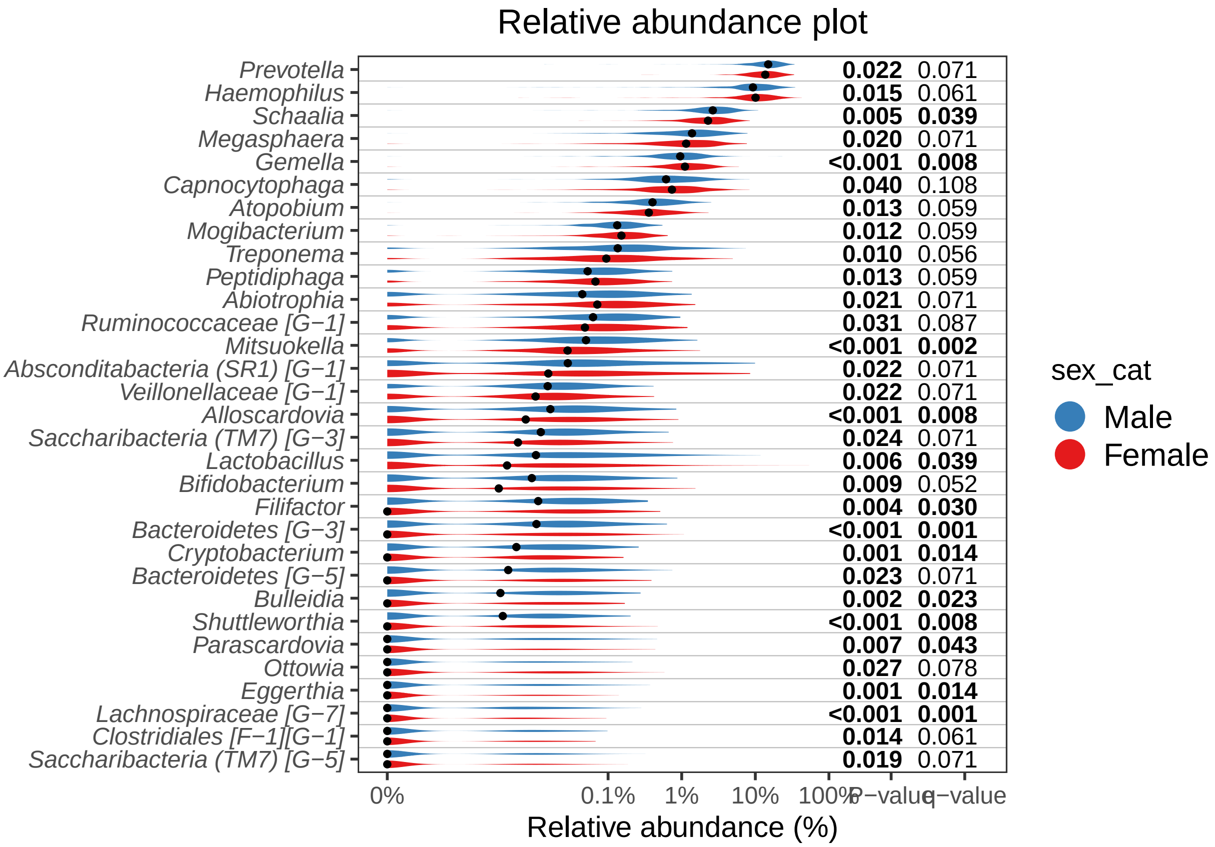
Relative abundance plot
Prevotella
Haemophilus
Schaalia
Megasphaera
Gemella
Capnocytophaga
Atopobium
Mogibacterium
Treponema
Peptidiphaga
Abiotrophia
Ruminococcaceae [G−1]
Mitsuokella
Absconditabacteria (SR1) [G−1]
Veillonellaceae [G−1]
Alloscardovia
Saccharibacteria (TM7) [G−3]
Lactobacillus
Bifidobacterium
Filifactor
Bacteroidetes [G−3]
Cryptobacterium
Bacteroidetes [G−5]
Bulleidia
Shuttleworthia
Parascardovia
Ottowia
Eggerthia
Lachnospiraceae [G−7]
Clostridiales [F−1][G−1]
Saccharibacteria (TM7) [G−5]
0.022 0.071
0.015 0.061
0.005 0.039
0.020 0.071
<0.001 0.008
0.040 0.108
0.013 0.059
0.012 0.059
0.010 0.056
0.013 0.059
0.021 0.071
0.031 0.087
<0.001 0.002
0.022 0.071
0.022 0.071
<0.001 0.008
0.024 0.071
0.006 0.039
0.009 0.052
0.004 0.030
<0.001 0.001
0.001 0.014
0.023 0.071
0.002 0.023
<0.001 0.008
0.007 0.043
0.027 0.078
0.001 0.014
<0.001 0.001
0.014 0.061
0.019 0.071
0%
0.1%
1%
10%
100%
P−value
q−value
Relative abundance (%)
sex_cat
Male
Female

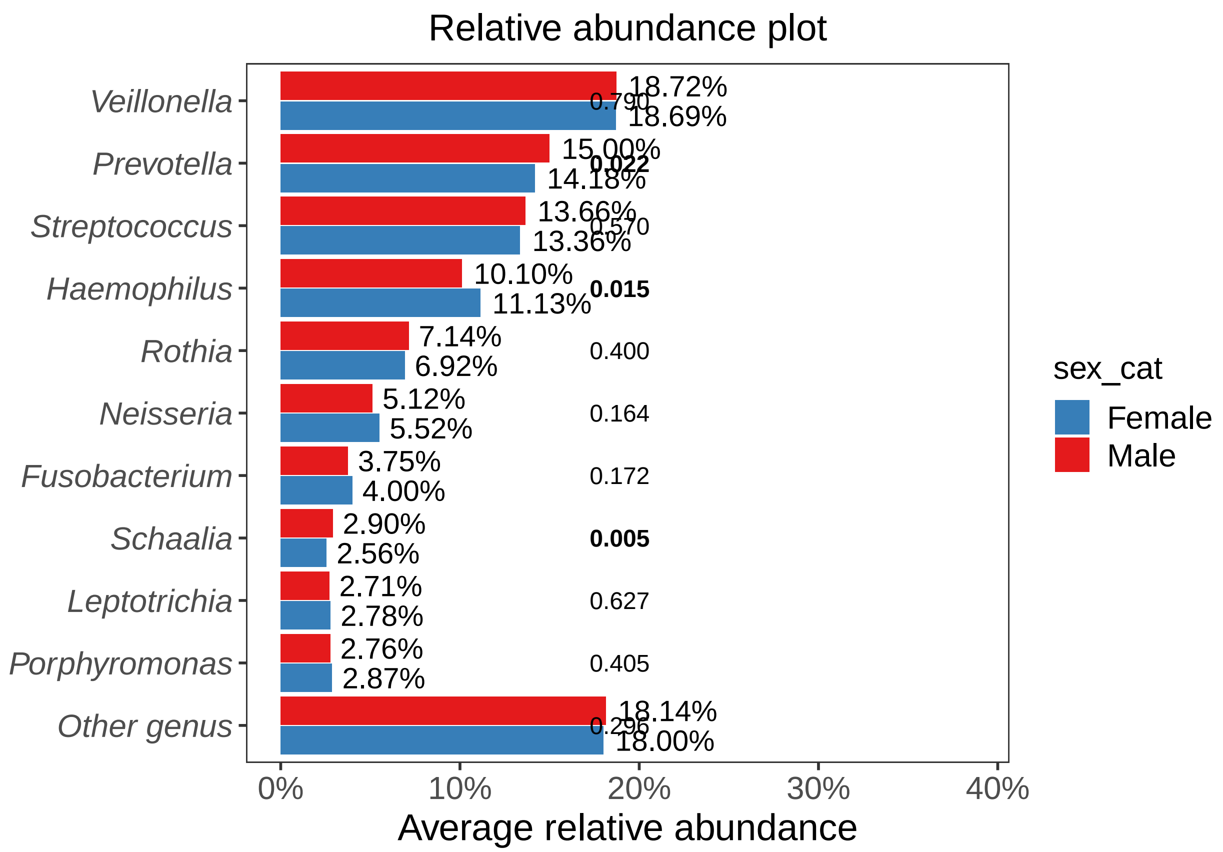

Relative abundance plot
Veillonella
Prevotella
Streptococcus
Haemophilus
Rothia
Neisseria
Fusobacterium
Schaalia
Leptotrichia
Porphyromonas
Other genus
18.72%
18.69%
0.790
15.00%
14.18%
0.022
13.66%
13.36%
0.570
10.10%
11.13%
0.015
7.14%
6.92%
0.400
5.12%
5.52%
0.164
3.75%
4.00%
0.172
2.90%
2.56%
0.005
2.71%
2.78%
0.627
2.76%
2.87%
0.405
18.14%
18.00%
0.296
0%
10%
20%
30%
40%
Average relative abundance
sex_cat
Female
Male

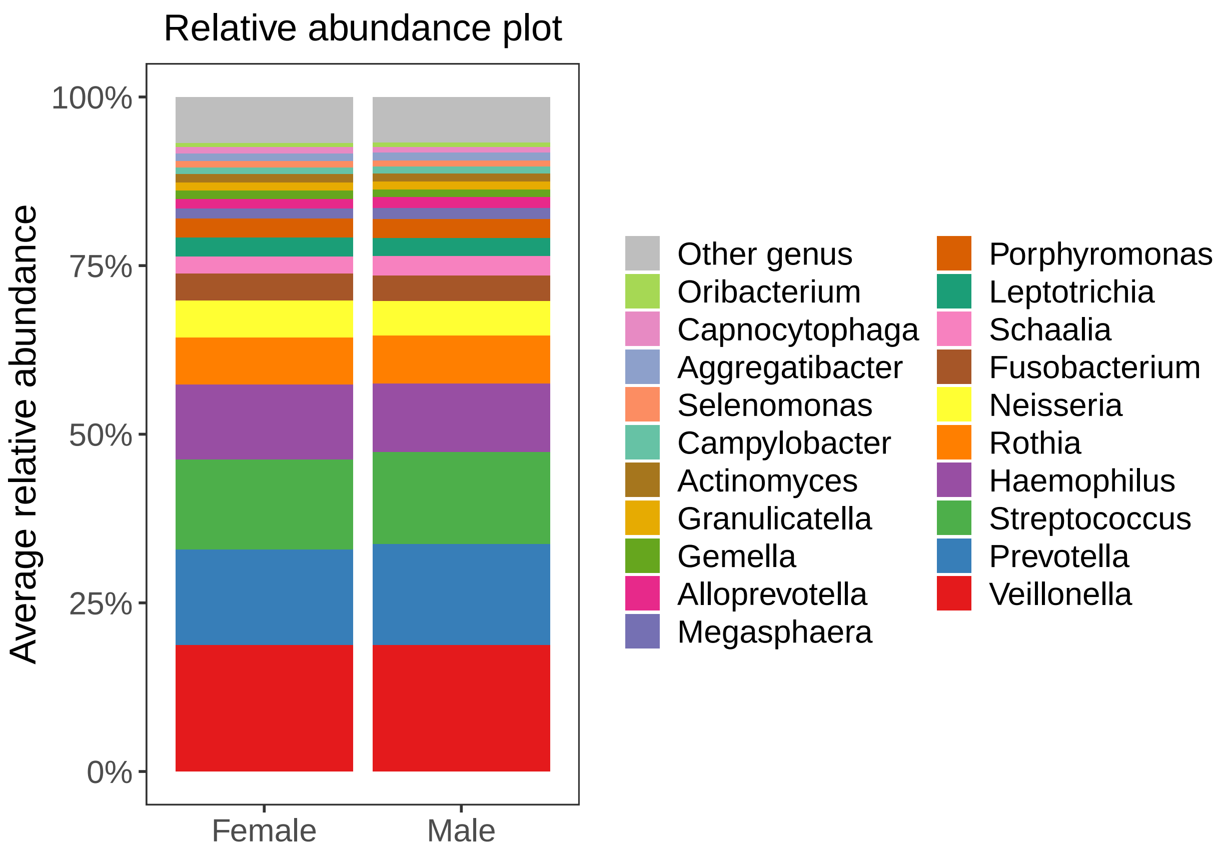
Relative abundance plot
Average relative abundance
100%
75%
50%
25%
0%
Female
Male
Other genus
Oribacterium
Capnocytophaga
Aggregatibacter
Selenomonas
Campylobacter
Actinomyces
Granulicatella
Gemella
Alloprevotella
Megasphaera
Porphyromonas
Leptotrichia
Schaalia
Fusobacterium
Neisseria
Rothia
Haemophilus
Streptococcus
Prevotella
Veillonella

```
## [1] "BMI_cat"
```

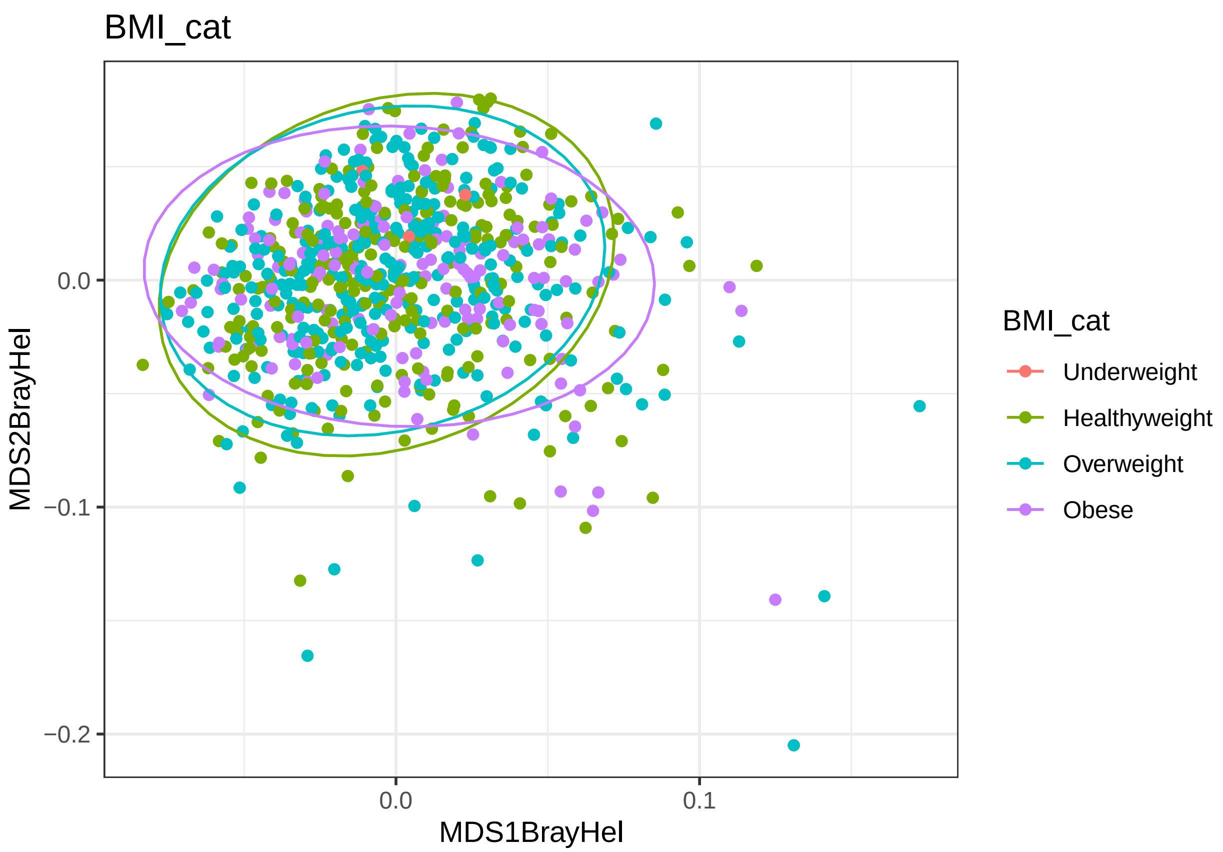


```
## Permutation test for adonis under reduced model
## Terms added sequentially (first to last)
## Permutation: free
## Number of permutations: 999
##
## adonis2(formula = formula(paste("distmatrix ~ ", i)), data = Phe3, permutations = 999, by = "terms")
##           Df SumOfSqs      R2     F Pr(>F)
## BMI_cat    3   0.1361 0.00506 1.258  0.129
## Residual 742  26.7562 0.99494
## Total    745  26.8923 1.00000
```

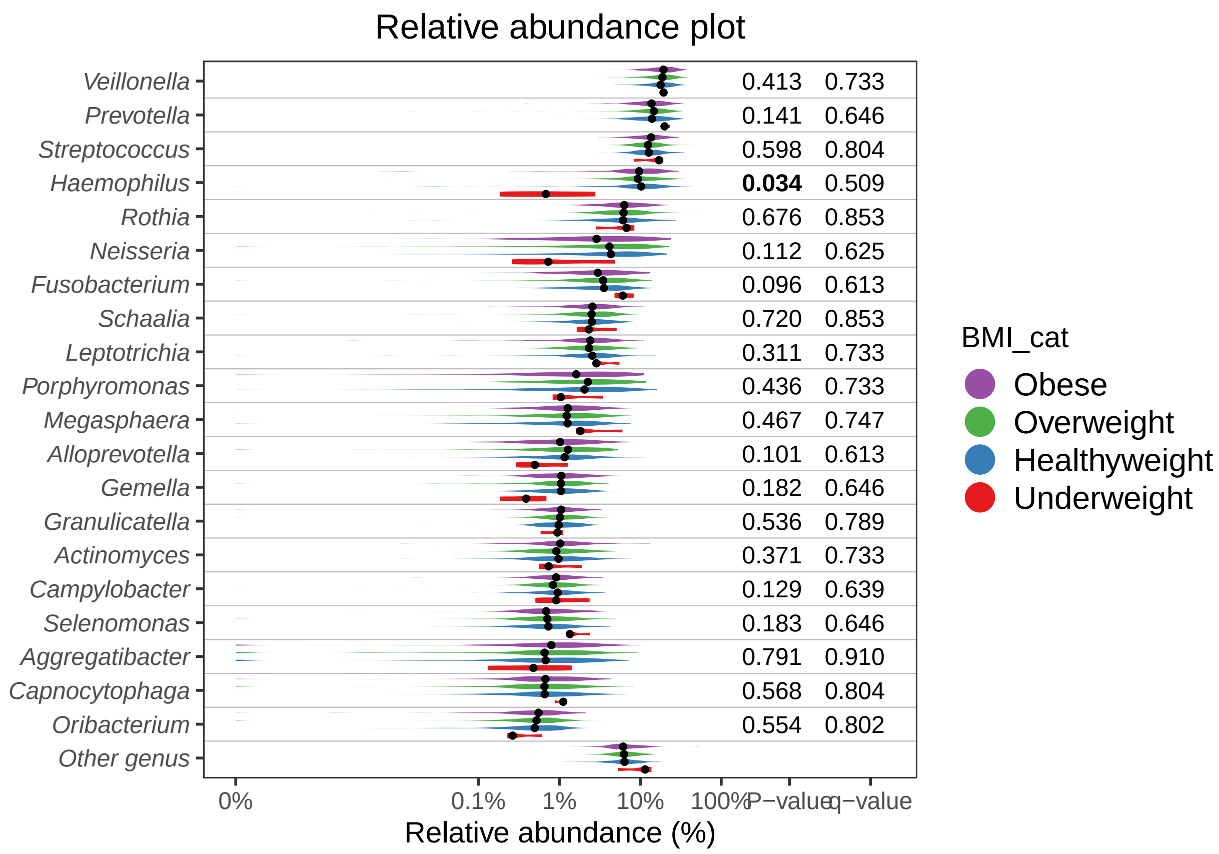
Relative abundance plot
Veillonella 0.413 0.733
Prevotella 0.141 0.646
Streptococcus 0.598 0.804
Haemophilus **0.034** 0.509
Rothia 0.676 0.853
Neisseria 0.112 0.625
Fusobacterium 0.096 0.613
Schaalia 0.720 0.853
Leptotrichia 0.311 0.733
Porphyromonas 0.436 0.733
Megasphaera 0.467 0.747
Alloprevotella 0.101 0.613
Gemella 0.182 0.646
Granulicatella 0.536 0.789
Actinomyces 0.371 0.733
Campylobacter 0.129 0.639
Selenomonas 0.183 0.646
Aggregatibacter 0.791 0.910
Capnocytophaga 0.568 0.804
Oribacterium 0.554 0.802
Other genus
0%
0.1%
1%
10%
100%
P−value
q−value
Relative abundance (%)
BMI_cat
Obese
Overweight
Healthyweight
Underweight

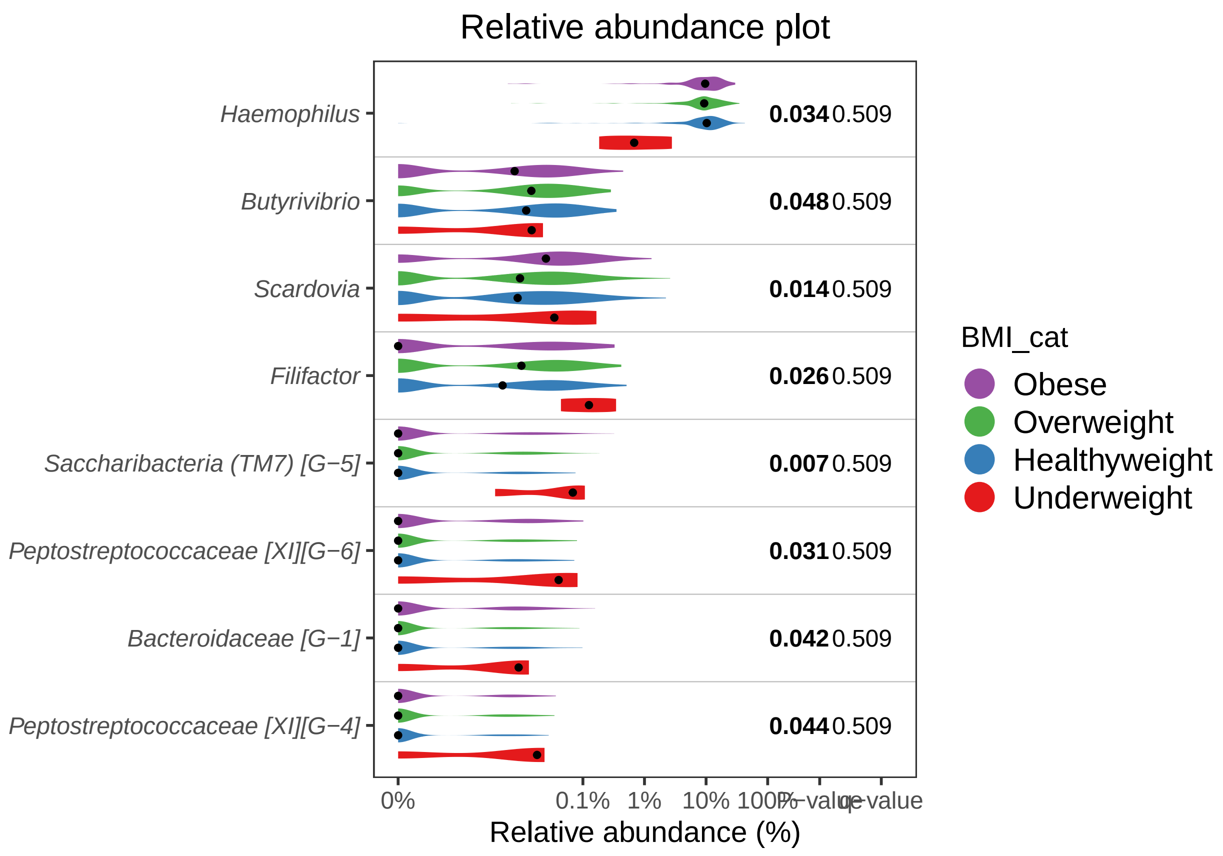
Relative abundance plot
Haemophilus
0.034 0.509
Butyrivibrio
0.048 0.509
Scardovia
0.014 0.509
Filifactor
0.026 0.509
Saccharibacteria (TM7) [G−5]
0.007 0.509
Peptostreptococcaceae [XI][G−6]
0.031 0.509
Bacteroidaceae [G−1]
0.042 0.509
Peptostreptococcaceae [XI][G−4]
0.044 0.509
0%
0.1%
1%
10%
100%
P−value
q−value
Relative abundance (%)
BMI_cat
Obese
Overweight
Healthyweight
Underweight

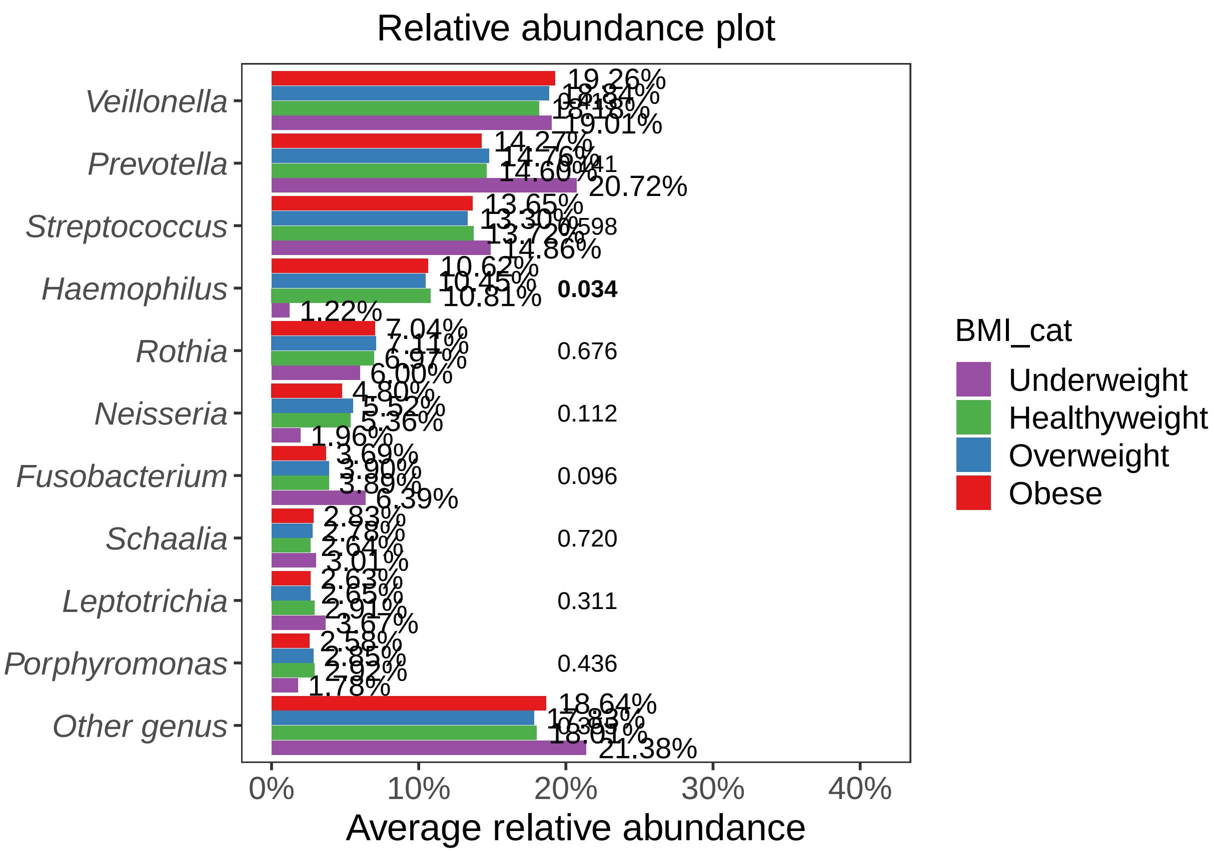
Relative abundance plot
Veillonella
Prevotella
Streptococcus
Haemophilus
Rothia
Neisseria
Fusobacterium
Schaalia
Leptotrichia
Porphyromonas
Other genus
19.26%
19.01%
14.27%
20.72%
13.65%
14.86%
10.62%
10.81%
1.22%
0.034
7.04%
6.00%
0.676
4.80%
5.52%
5.36%
1.96%
0.112
3.69%
6.39%
0.096
2.83%
3.01%
0.720
3.67%
0.311
2.58%
1.78%
0.436
18.64%
21.38%
0%
10%
20%
30%
40%
Average relative abundance
BMI_cat
Underweight
Healthyweight
Overweight
Obese

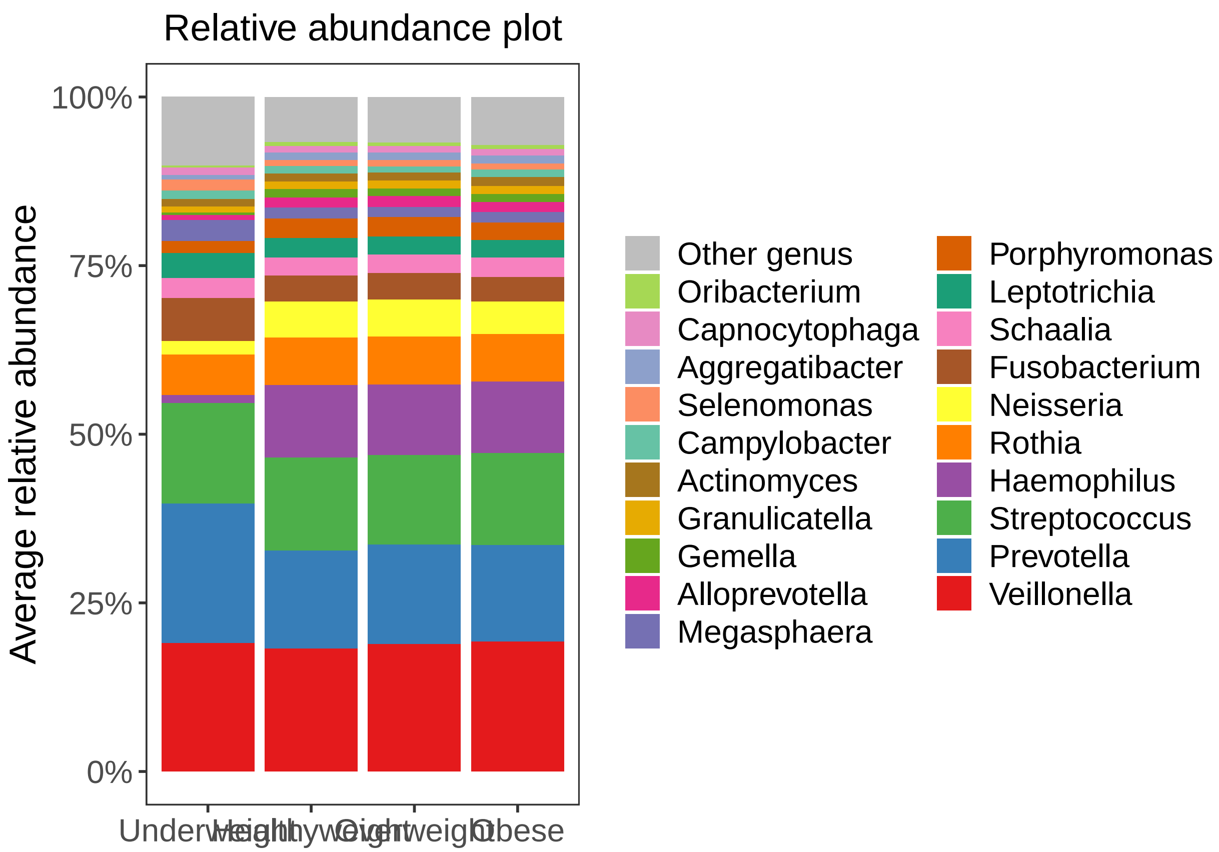
Relative abundance plot
Average relative abundance
100%
75%
50%
25%
0%
Underweight
Healthyweight
Overweight
Obese
Other genus
Oribacterium
Capnocytophaga
Aggregatibacter
Selenomonas
Campylobacter
Actinomyces
Granulicatella
Gemella
Alloprevotella
Megasphaera
Porphyromonas
Leptotrichia
Schaalia
Fusobacterium
Neisseria
Rothia
Haemophilus
Streptococcus
Prevotella
Veillonella

```
## [1] "Waist_cat"
```

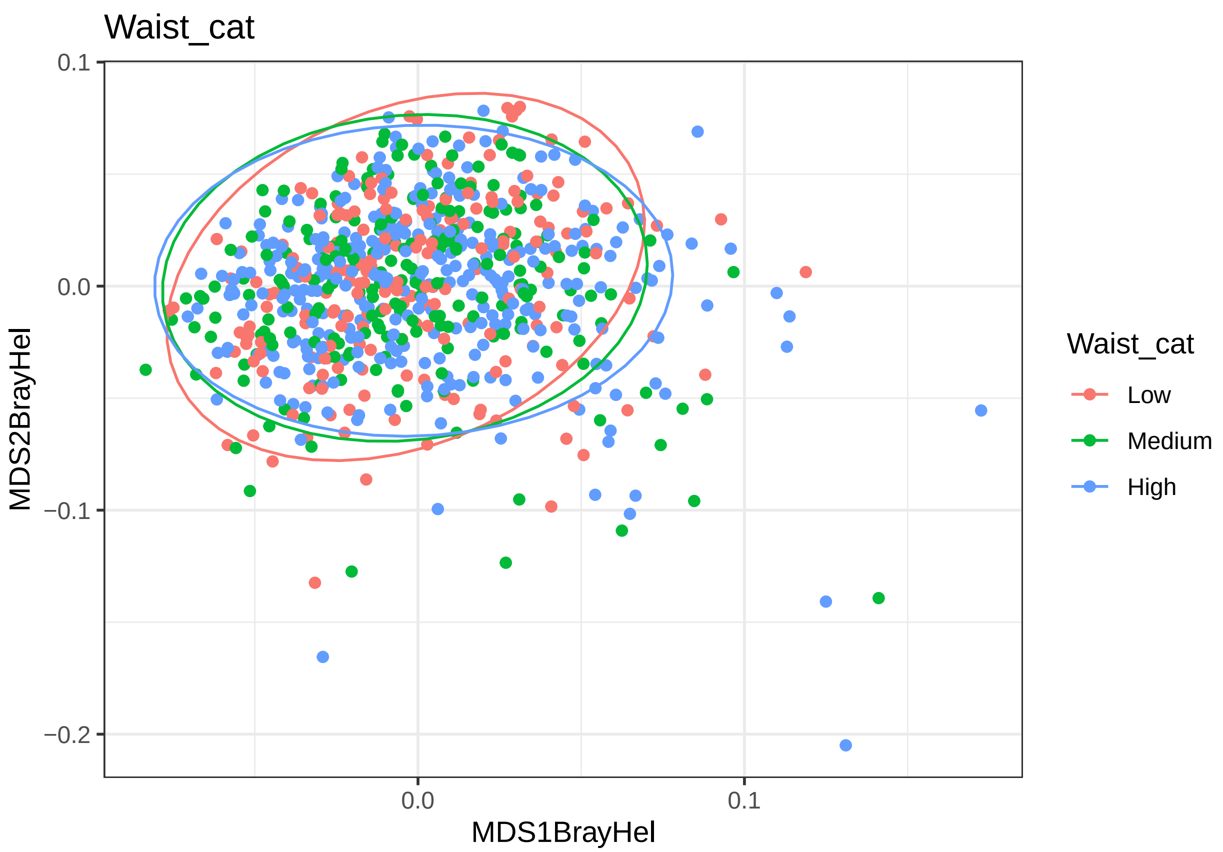


```
## Permutation test for adonis under reduced model
## Terms added sequentially (first to last)
## Permutation: free
## Number of permutations: 999
##
## adonis2(formula = formula(paste("distmatrix ~ ", i)), data = Phe3, permutations = 999, by = "terms")
##            Df SumOfSqs      R2      F Pr(>F)
## Waist_cat   2   0.0661 0.00246 0.9158   0.54
## Residual  743  26.8262 0.99754
## Total     745  26.8923 1.00000
```

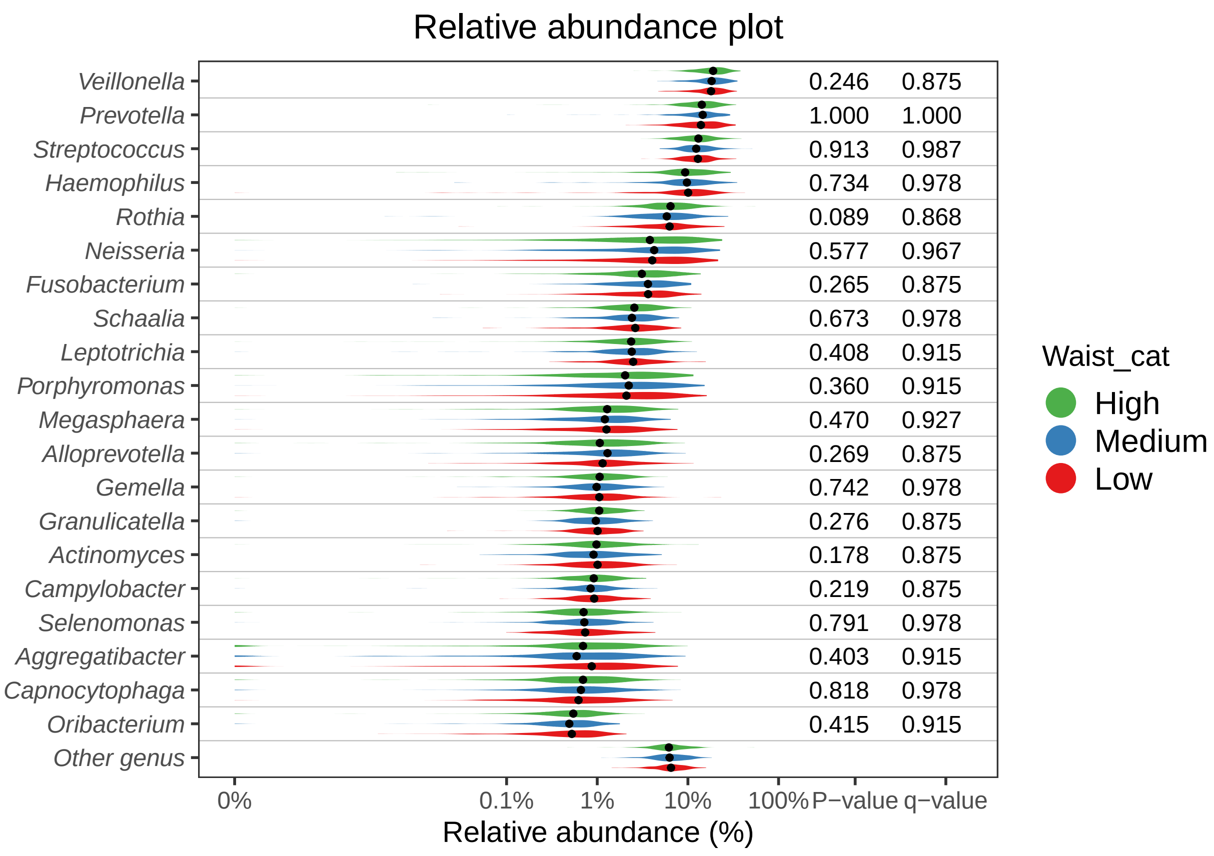
Relative abundance plot
Veillonella
Prevotella
Streptococcus
Haemophilus
Rothia
Neisseria
Fusobacterium
Schaalia
Leptotrichia
Porphyromonas
Megasphaera
Alloprevotella
Gemella
Granulicatella
Actinomyces
Campylobacter
Selenomonas
Aggregatibacter
Capnocytophaga
Oribacterium
Other genus
0.246 0.875
1.000 1.000
0.913 0.987
0.734 0.978
0.089 0.868
0.577 0.967
0.265 0.875
0.673 0.978
0.408 0.915
0.360 0.915
0.470 0.927
0.269 0.875
0.742 0.978
0.276 0.875
0.178 0.875
0.219 0.875
0.791 0.978
0.403 0.915
0.818 0.978
0.415 0.915
0%
0.1%
1%
10%
100%
P−value
q−value
Relative abundance (%)
Waist_cat
High
Medium
Low

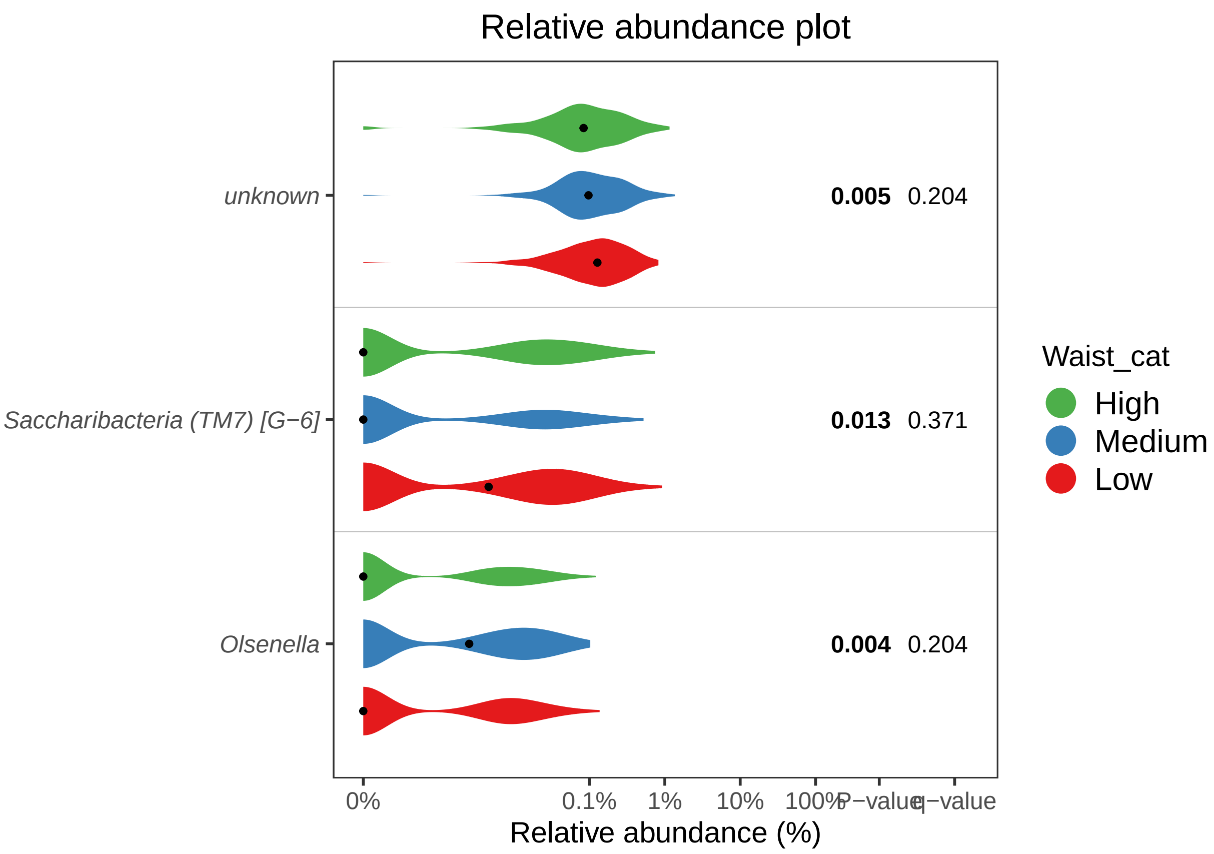

Relative abundance plot
unknown
0.005 0.204
Saccharibacteria (TM7) [G−6]
0.013 0.371
Olsenella
0.004 0.204
0%
0.1%
1%
10%
100%
P−value
q−value
Relative abundance (%)
Waist_cat
High
Medium
Low

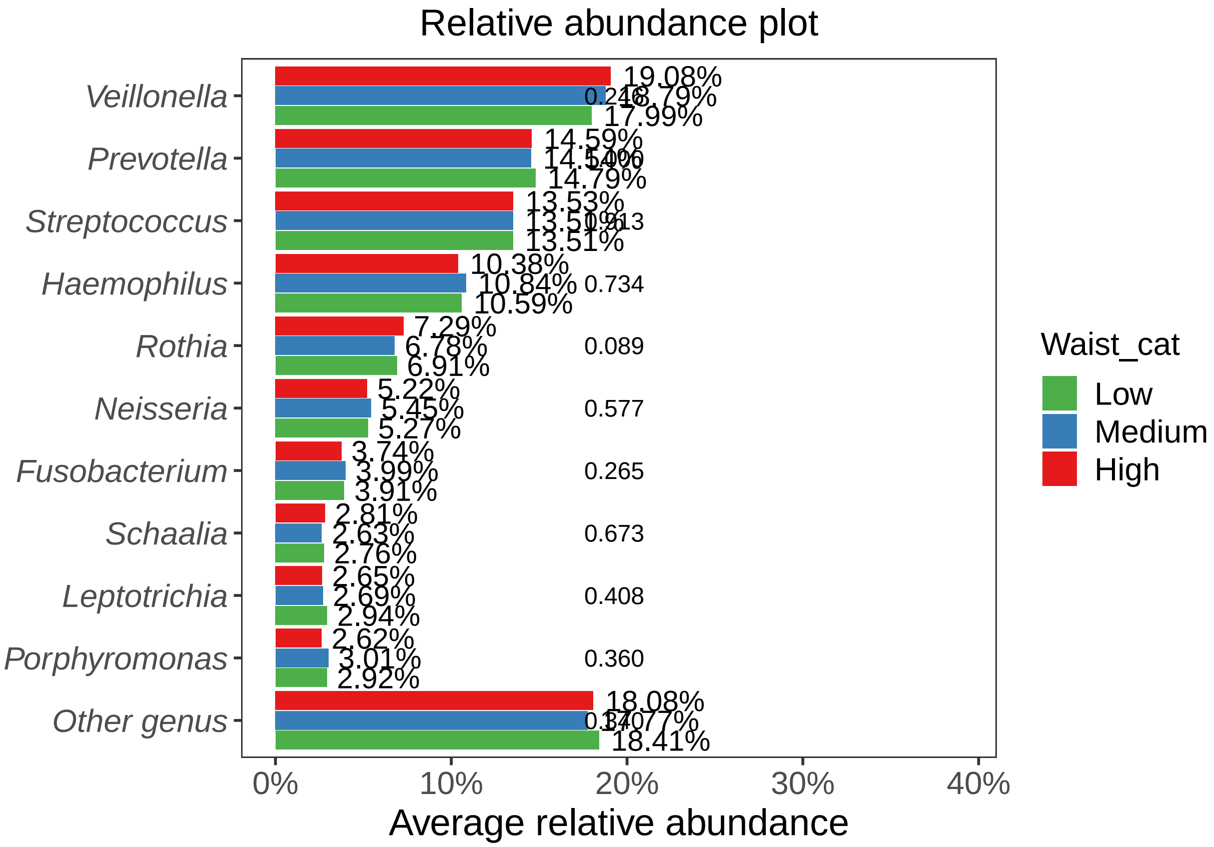

Relative abundance plot
Veillonella
Prevotella
Streptococcus
Haemophilus
Rothia
Neisseria
Fusobacterium
Schaalia
Leptotrichia
Porphyromonas
Other genus
19.08%
18.79%
17.99%
14.59%
14.79%
13.53%
13.51%
10.38%
10.84%
10.59%
0.734
7.29%
6.78%
6.91%
0.089
5.22%
5.45%
5.27%
0.577
3.74%
3.99%
3.91%
0.265
2.81%
2.63%
2.76%
0.673
2.65%
2.69%
2.94%
0.408
2.62%
3.01%
2.92%
0.360
18.08%
18.41%
0%
10%
20%
30%
40%
Average relative abundance
Waist_cat
Low
Medium
High

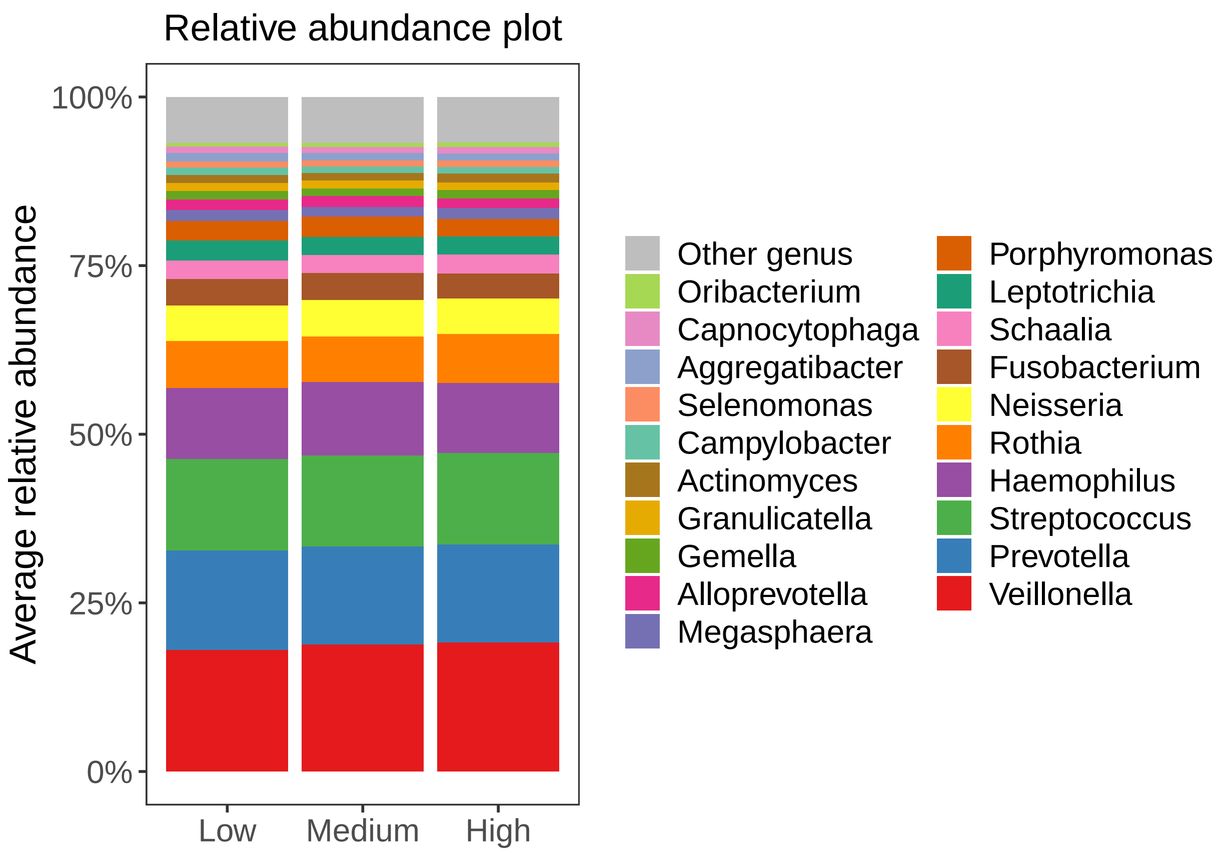


```
## [1] "whratio_cat"
```

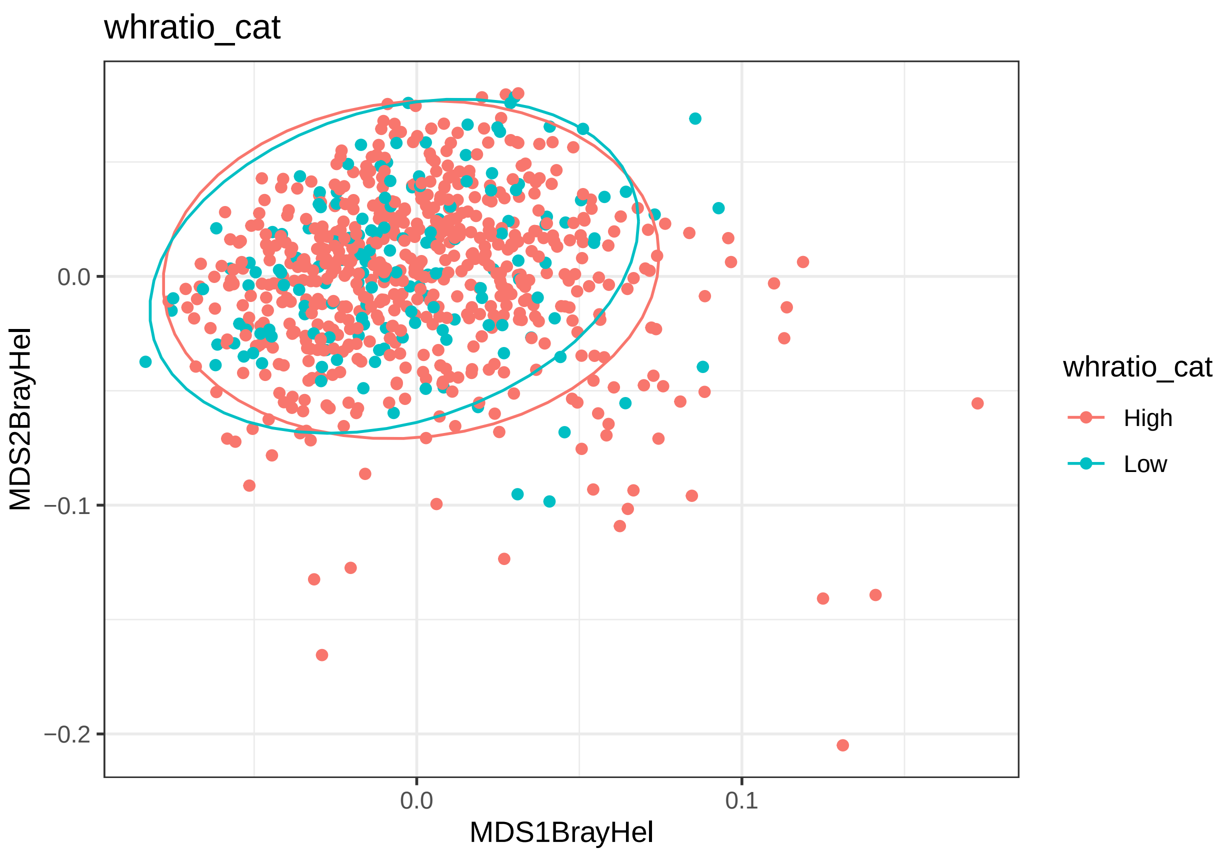


```
## Permutation test for adonis under reduced model
## Terms added sequentially (first to last)
## Permutation: free
## Number of permutations: 999
##
## adonis2(formula = formula(paste("distmatrix ~ ", i)), data = Phe3, permutations = 999, by = "terms")
##              Df SumOfSqs      R2      F Pr(>F)
## whratio_cat   1   0.0507 0.00189 1.4051  0.152
## Residual    744  26.8416 0.99811
## Total       745  26.8923 1.00000
```

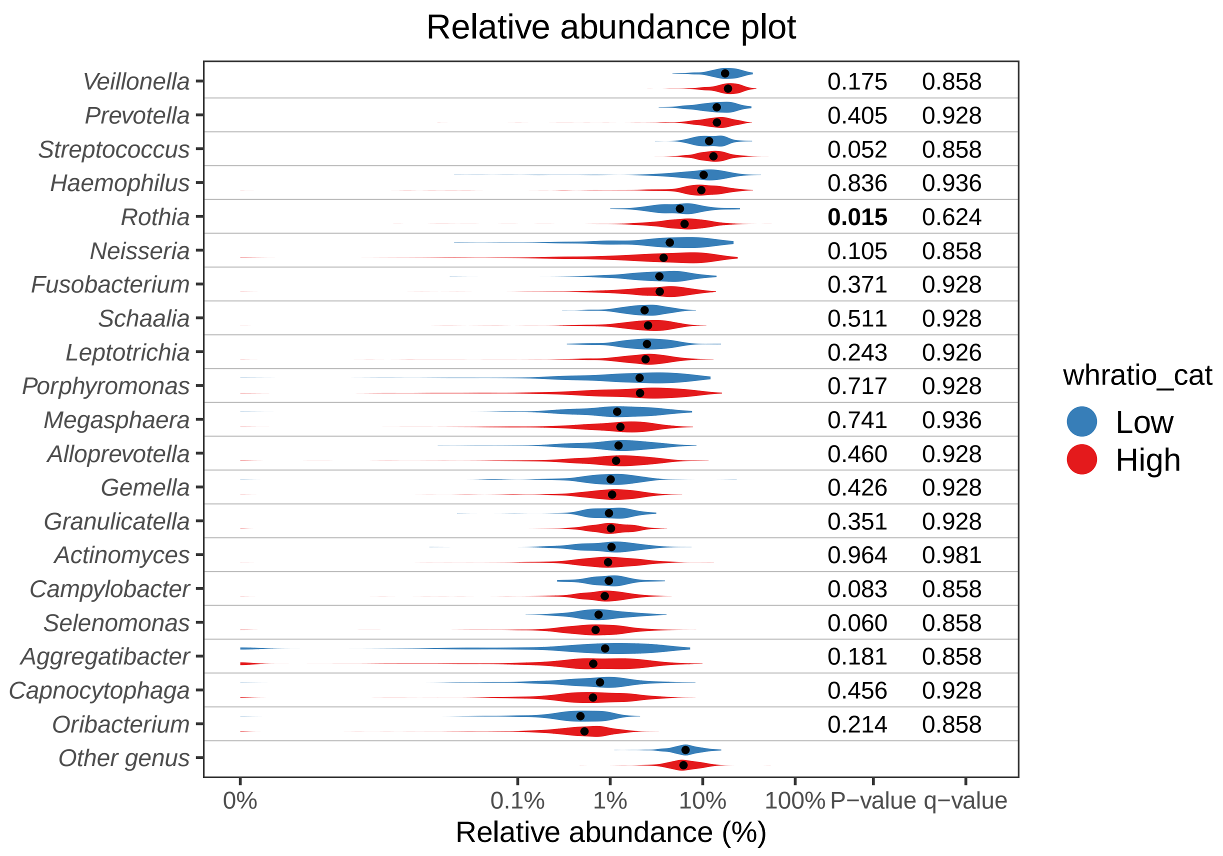
Relative abundance plot
Veillonella
Prevotella
Streptococcus
Haemophilus
Rothia
Neisseria
Fusobacterium
Schaalia
Leptotrichia
Porphyromonas
Megasphaera
Alloprevotella
Gemella
Granulicatella
Actinomyces
Campylobacter
Selenomonas
Aggregatibacter
Capnocytophaga
Oribacterium
Other genus
0.175 0.858
0.405 0.928
0.052 0.858
0.836 0.936
0.015 0.624
0.105 0.858
0.371 0.928
0.511 0.928
0.243 0.926
0.717 0.928
0.741 0.936
0.460 0.928
0.426 0.928
0.351 0.928
0.964 0.981
0.083 0.858
0.060 0.858
0.181 0.858
0.456 0.928
0.214 0.858
0%
0.1%
1%
10%
100%
P−value
q−value
Relative abundance (%)
whratio_cat
Low
High

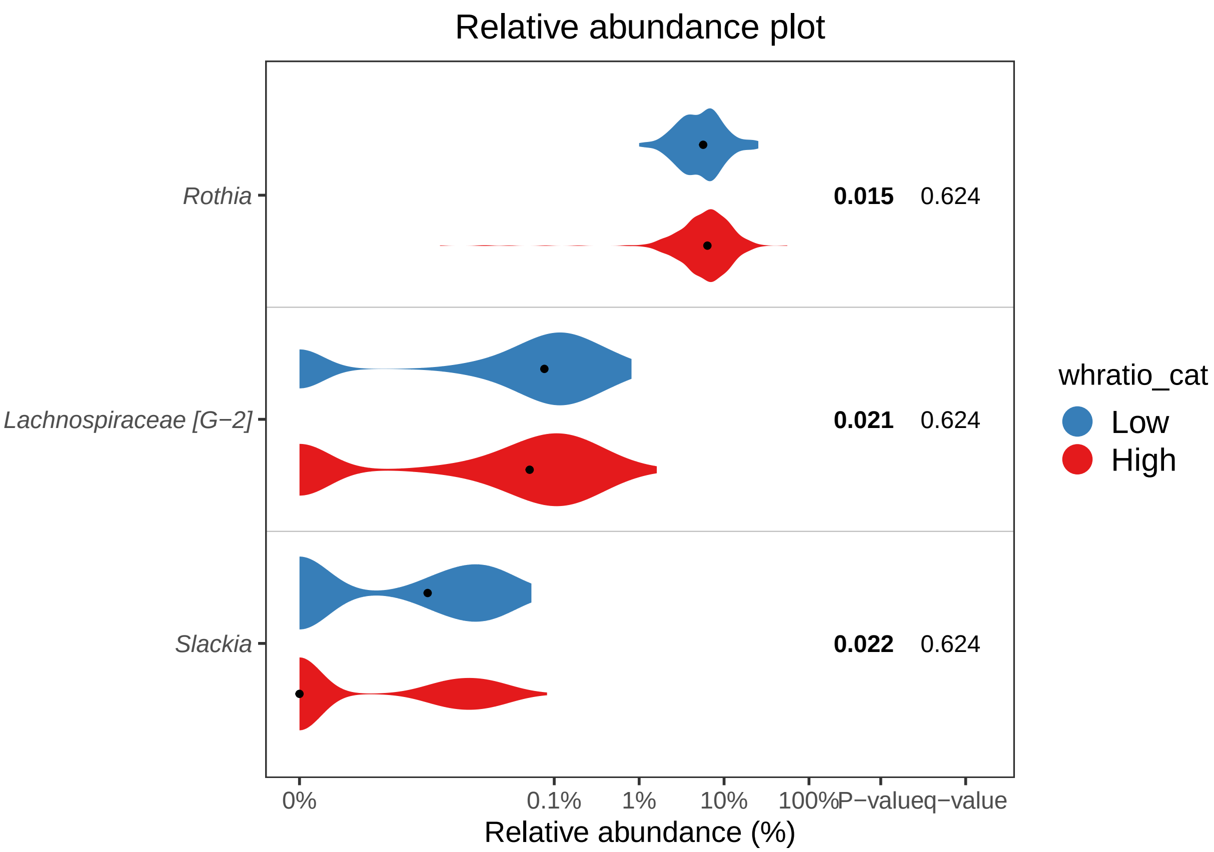
Relative abundance plot
Rothia
Lachnospiraceae [G−2]
Slackia
0.015
0.624
0.021
0.624
0.022
0.624
0%
0.1%
1%
10%
100%
P−value
q−value
Relative abundance (%)
whratio_cat
Low
High

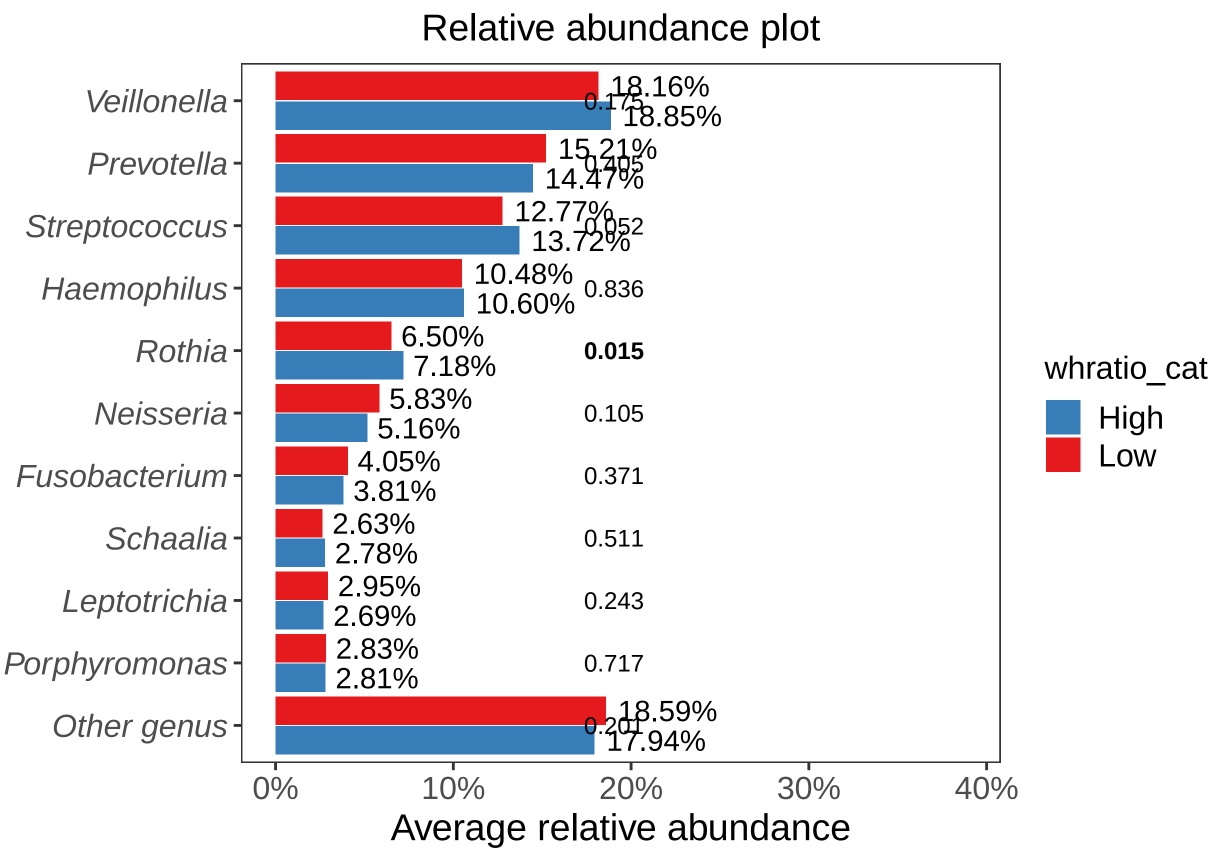
Relative abundance plot
Veillonella
Prevotella
Streptococcus
Haemophilus
Rothia
Neisseria
Fusobacterium
Schaalia
Leptotrichia
Porphyromonas
Other genus
18.16%
18.85%
0.175
15.21%
14.47%
0.405
12.77%
13.72%
0.052
10.48%
10.60%
0.836
6.50%
7.18%
0.015
5.83%
5.16%
0.105
4.05%
3.81%
0.371
2.63%
2.78%
0.511
2.95%
2.69%
0.243
2.83%
2.81%
0.717
18.59%
17.94%
0.201
0%
10%
20%
30%
40%
Average relative abundance
whratio_cat
High
Low

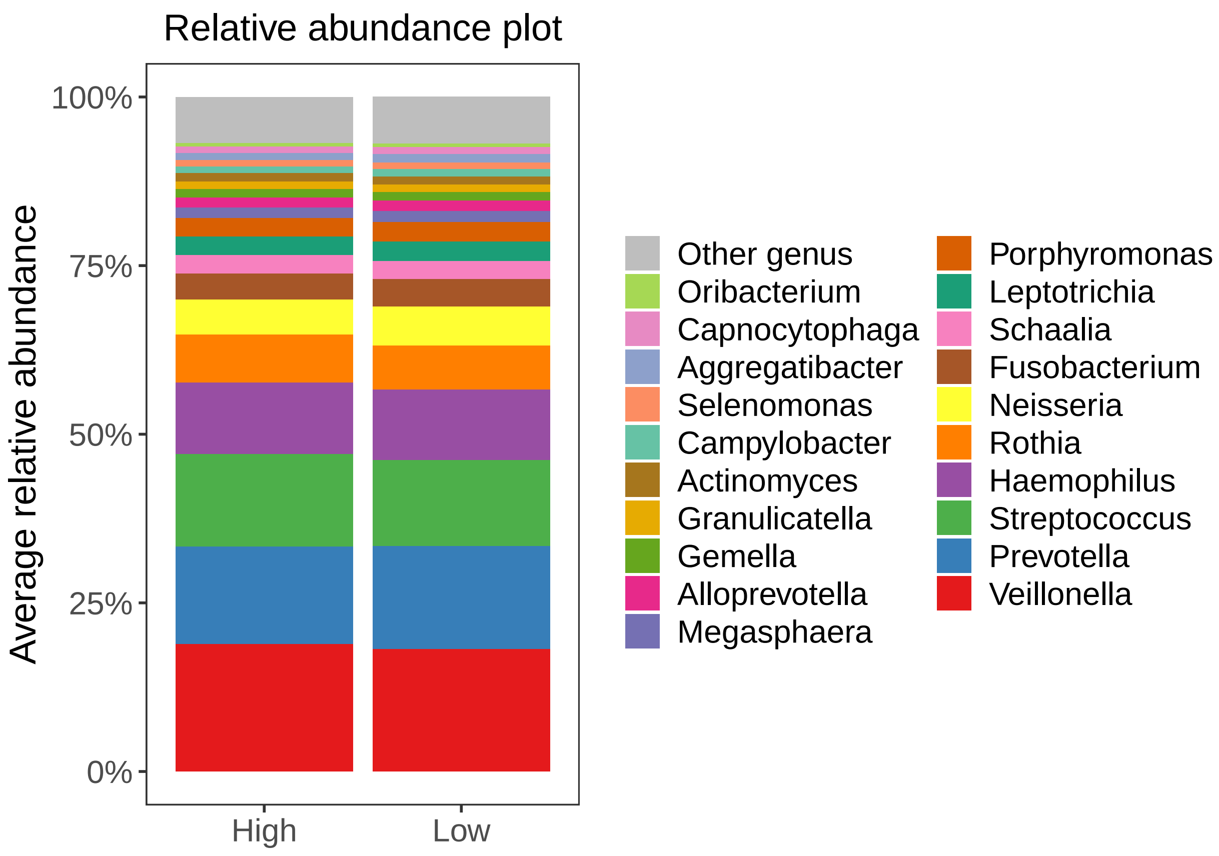


```
## [1] "Alcprweek_cat"
```

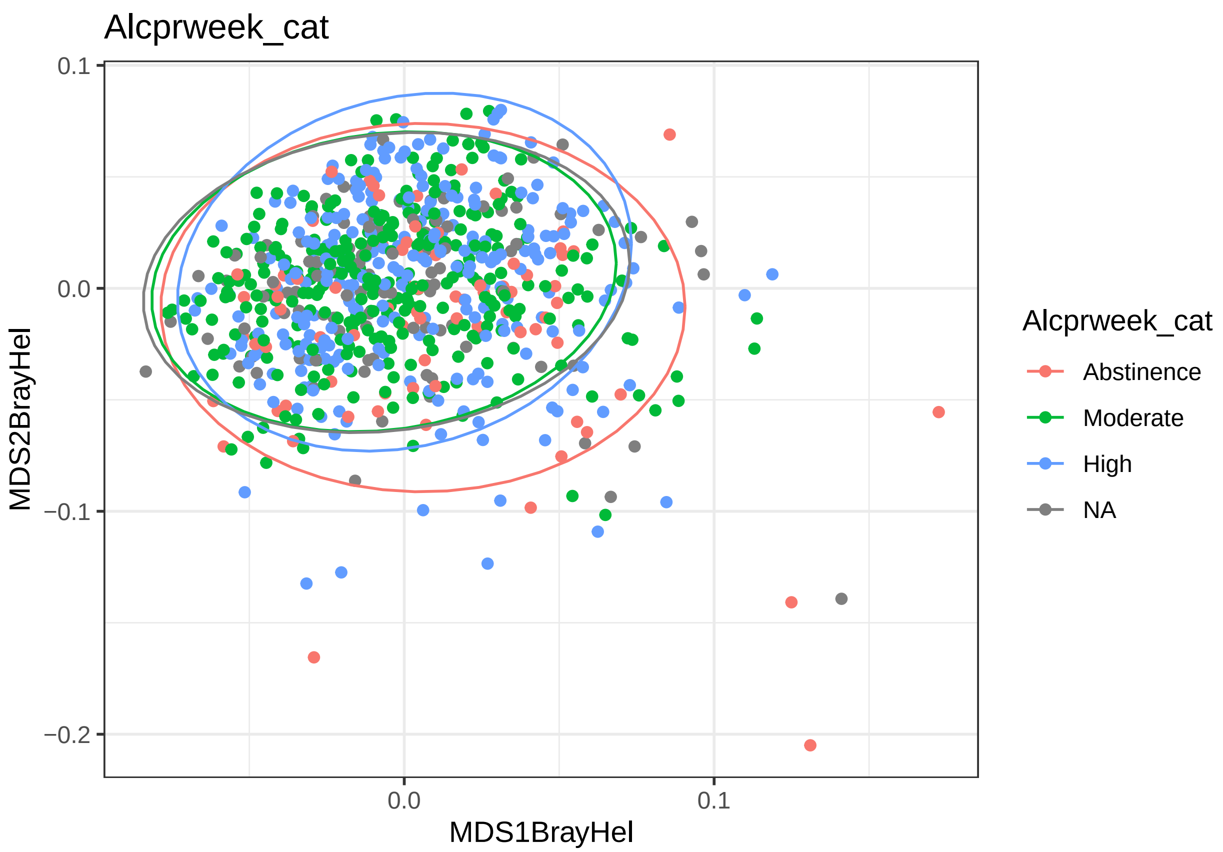


```
## Permutation test for adonis under reduced model
## Terms added sequentially (first to last)
## Permutation: free
## Number of permutations: 999
##
## adonis2(formula = formula(paste("distmatrix ~ ", i)), data = Phe3, permutations = 999, by = "terms")
##                Df SumOfSqs      R2      F Pr(>F)
## Alcprweek_cat   2   0.2475 0.01066 3.4486  0.001 ***
## Residual      640  22.9684 0.98934
## Total         642  23.2159 1.00000
## ---
## Signif. codes:  0 '***' 0.001 '**' 0.01 '*' 0.05 '.' 0.1 ' ' 1
```

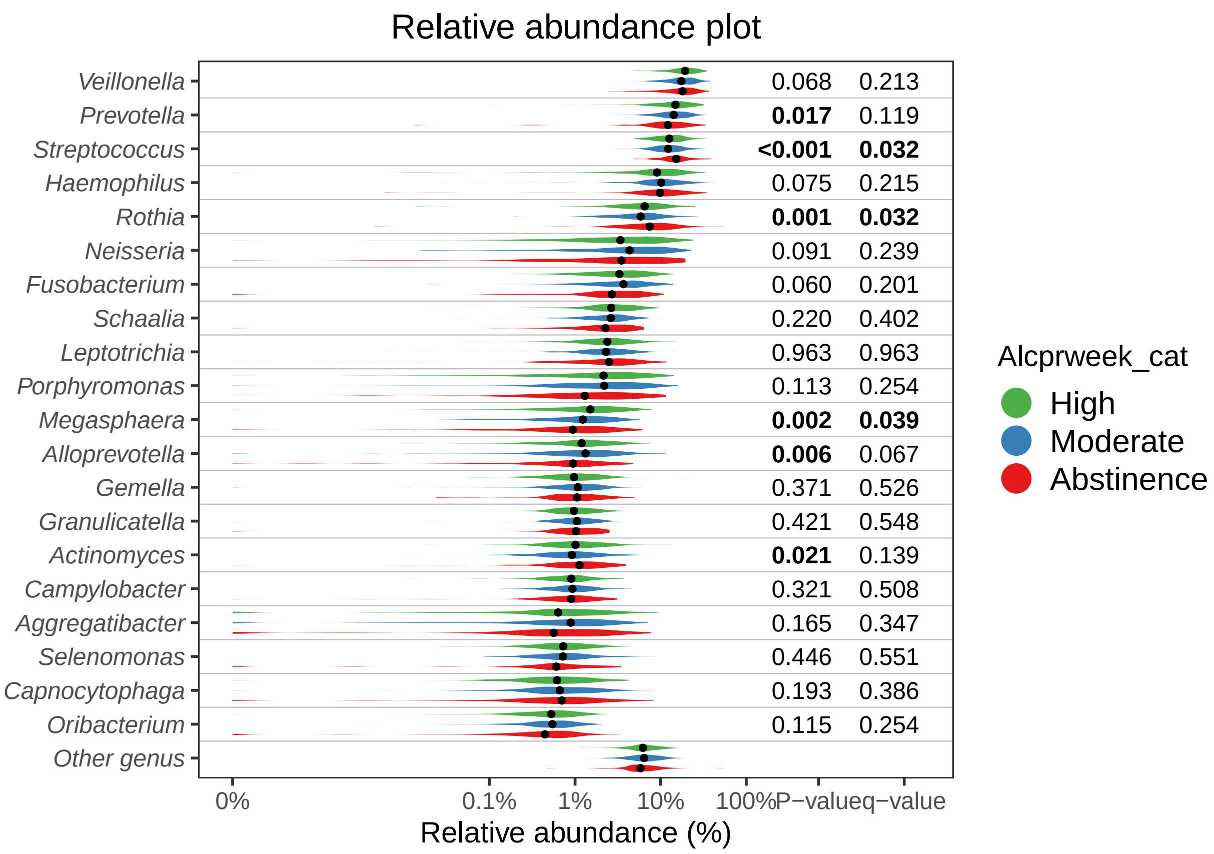

Relative abundance plot
Veillonella
Prevotella
Streptococcus
Haemophilus
Rothia
Neisseria
Fusobacterium
Schaalia
Leptotrichia
Porphyromonas
Megasphaera
Alloprevotella
Gemella
Granulicatella
Actinomyces
Campylobacter
Aggregatibacter
Selenomonas
Capnocytophaga
Oribacterium
Other genus
0.068 0.213
0.017 0.119
<0.001 0.032
0.075 0.215
0.001 0.032
0.091 0.239
0.060 0.201
0.220 0.402
0.963 0.963
0.113 0.254
0.002 0.039
0.006 0.067
0.371 0.526
0.421 0.548
0.021 0.139
0.321 0.508
0.165 0.347
0.446 0.551
0.193 0.386
0.115 0.254
0%
0.1%
1%
10%
100%
P−value
q−value
Relative abundance (%)
Alcprweek_cat
High
Moderate
Abstinence

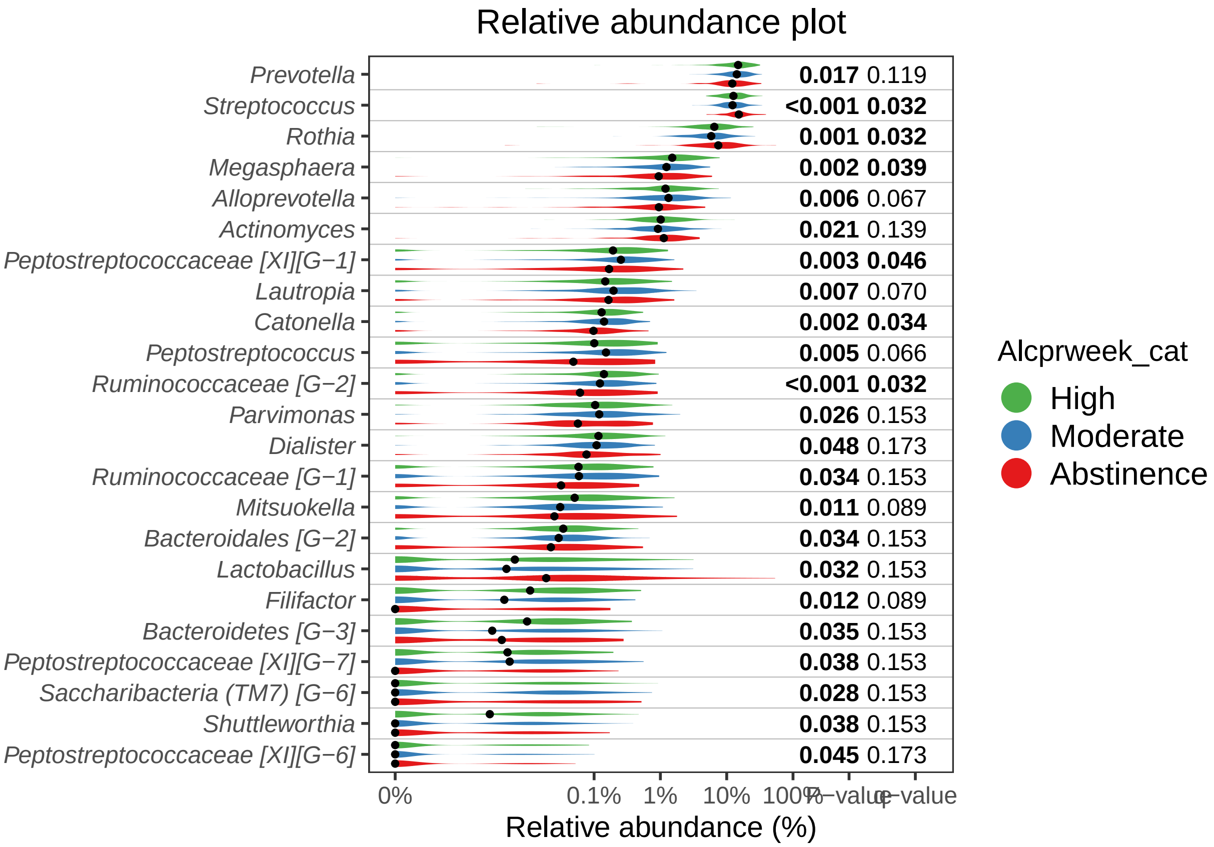
Relative abundance plot
Prevotella
Streptococcus
Rothia
Megasphaera
Alloprevotella
Actinomyces
Peptostreptococcaceae [XI][G−1]
Lautropia
Catonella
Peptostreptococcus
Ruminococcaceae [G−2]
Parvimonas
Dialister
Ruminococcaceae [G−1]
Mitsuokella
Bacteroidales [G−2]
Lactobacillus
Filifactor
Bacteroidetes [G−3]
Peptostreptococcaceae [XI][G−7]
Saccharibacteria (TM7) [G−6]
Shuttleworthia
Peptostreptococcaceae [XI][G−6]
0.017 0.119
<0.001 0.032
0.001 0.032
0.002 0.039
0.006 0.067
0.021 0.139
0.003 0.046
0.007 0.070
0.002 0.034
0.005 0.066
<0.001 0.032
0.026 0.153
0.048 0.173
0.034 0.153
0.011 0.089
0.034 0.153
0.032 0.153
0.012 0.089
0.035 0.153
0.038 0.153
0.028 0.153
0.038 0.153
0.045 0.173
0% 0.1% 1% 10% 100% P−value q−value
Relative abundance (%)
Alcprweek_cat
High
Moderate
Abstinence

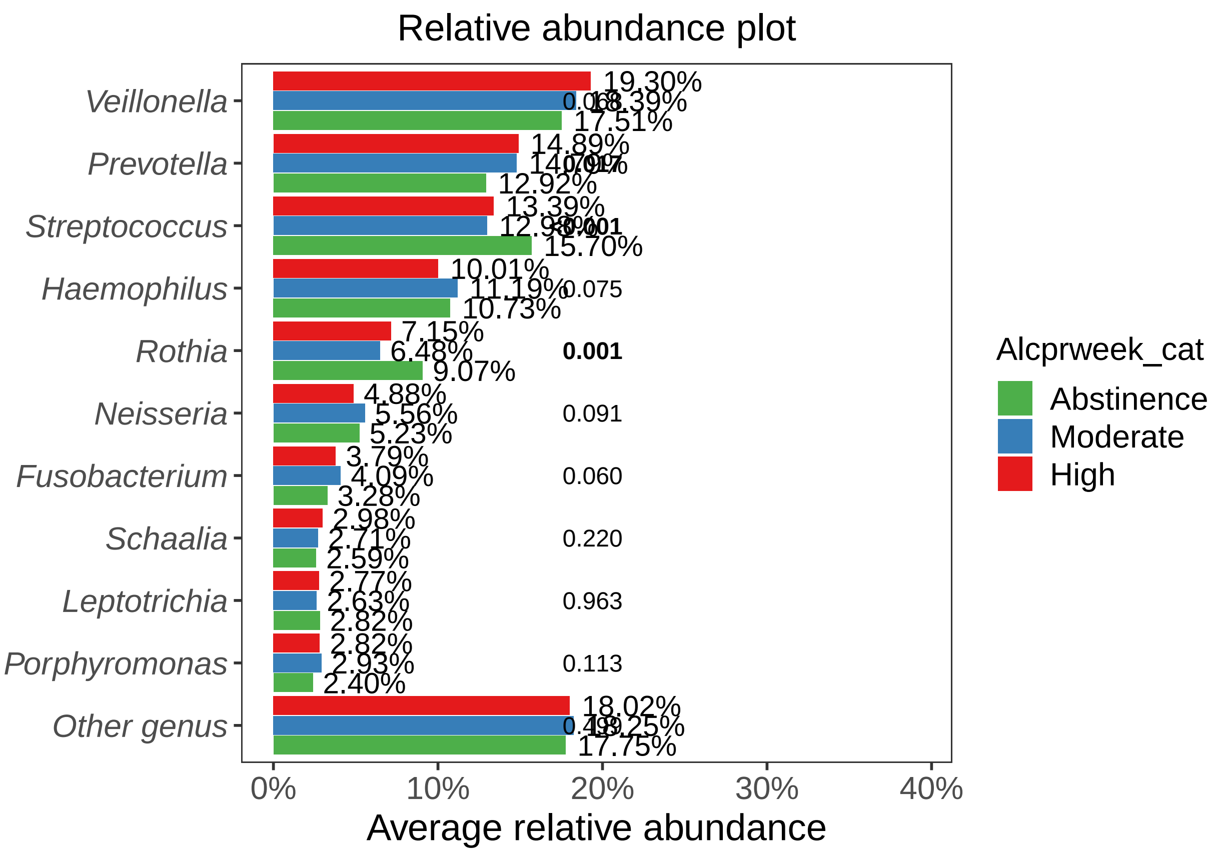
Relative abundance plot
Veillonella
Prevotella
Streptococcus
Haemophilus
Rothia
Neisseria
Fusobacterium
Schaalia
Leptotrichia
Porphyromonas
Other genus
19.30%
18.39%
17.51%
14.89%
12.92%
13.39%
15.70%
10.01%
11.19%
10.73%
0.075
7.15%
6.48%
9.07%
0.001
4.88%
5.56%
5.23%
0.091
3.79%
4.09%
3.28%
0.060
2.98%
2.71%
2.59%
0.220
2.77%
2.63%
2.82%
0.963
2.82%
2.93%
2.40%
0.113
18.02%
18.25%
17.75%
0%
10%
20%
30%
40%
Average relative abundance
Alcprweek_cat
Abstinence
Moderate
High

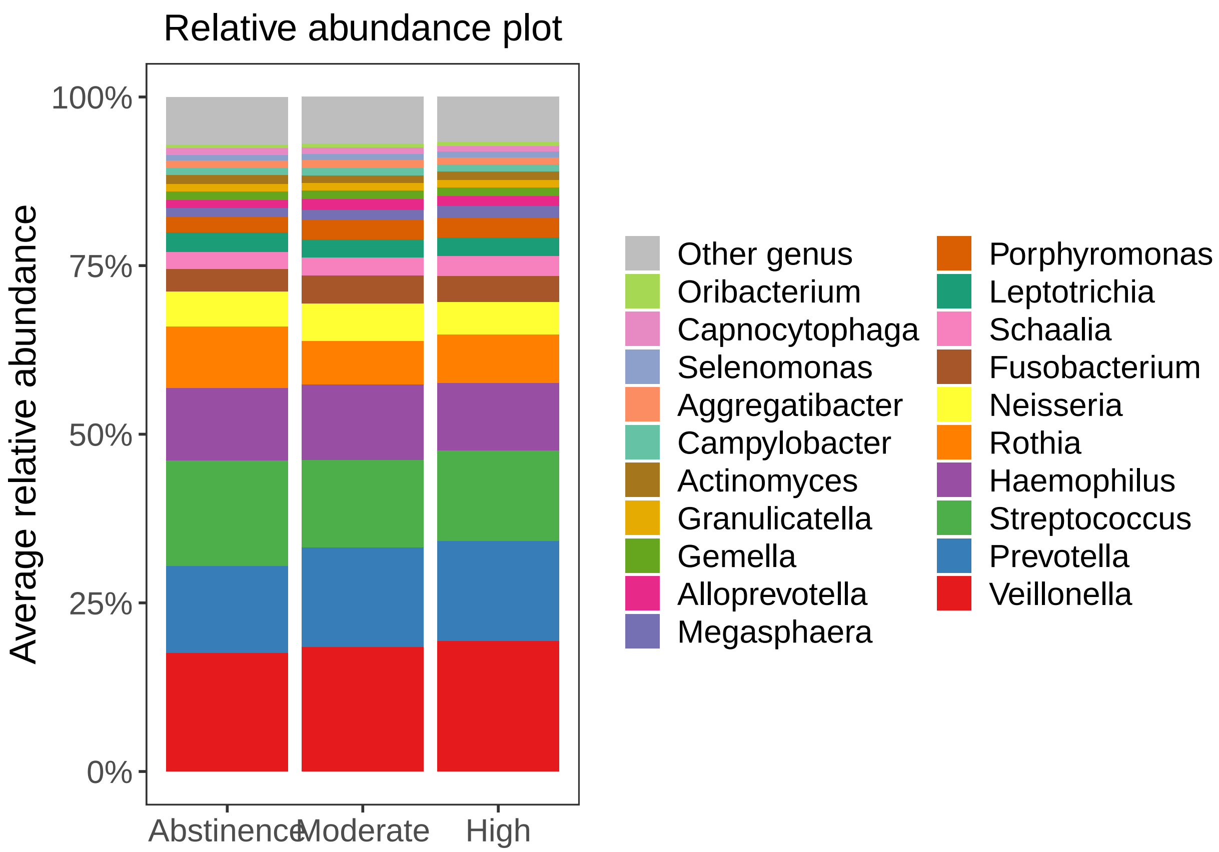
Relative abundance plot
100%
75%
50%
25%
0%
Average relative abundance
Abstinence
Moderate
High
Other genus
Oribacterium
Capnocytophaga
Selenomonas
Aggregatibacter
Campylobacter
Actinomyces
Granulicatella
Gemella
Alloprevotella
Megasphaera
Porphyromonas
Leptotrichia
Schaalia
Fusobacterium
Neisseria
Rothia
Haemophilus
Streptococcus
Prevotella
Veillonella

```
## [1] "Smoking"
```

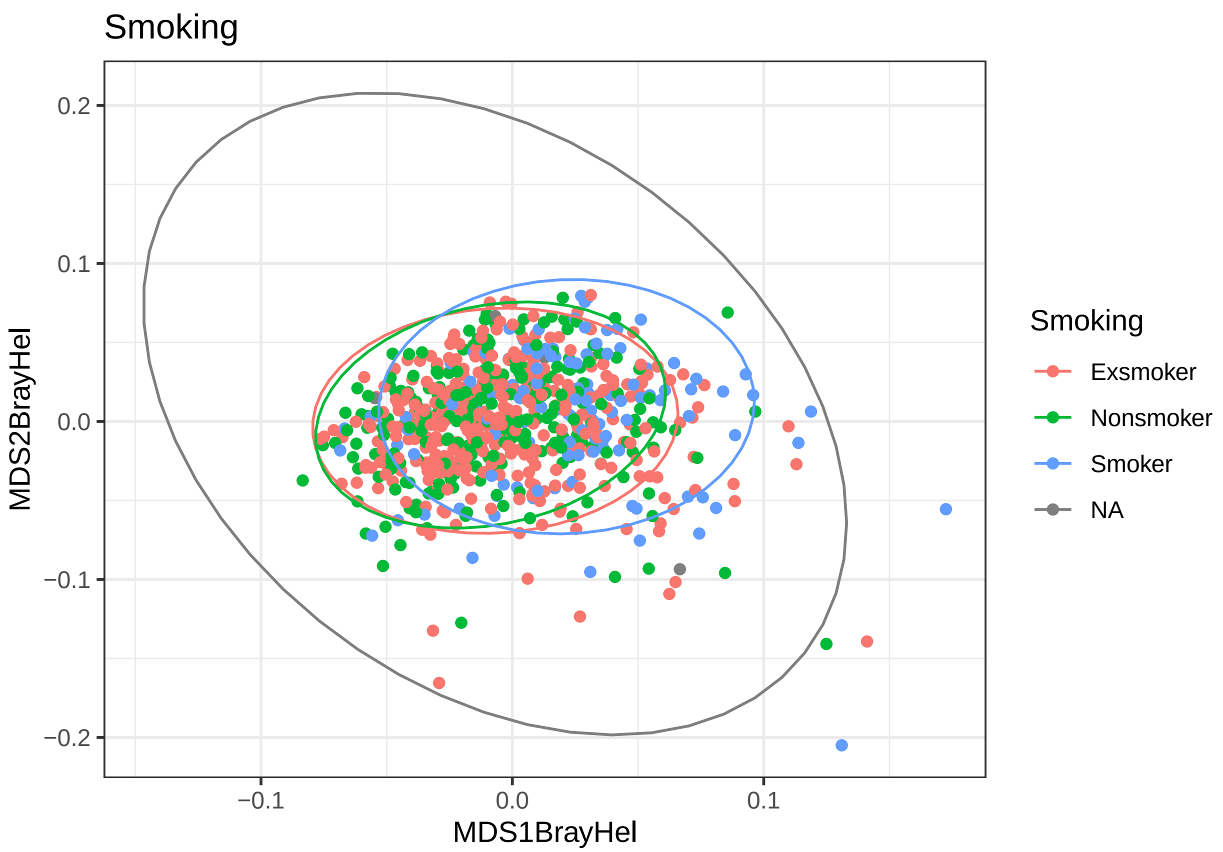


```
## Permutation test for adonis under reduced model
## Terms added sequentially (first to last)
## Permutation: free
## Number of permutations: 999
##
## adonis2(formula = formula(paste("distmatrix ~ ", i)), data = Phe3, permutations = 999, by = "terms")
##           Df SumOfSqs      R2      F Pr(>F)
## Smoking    2   0.8677 0.03254 12.412  0.001 ***
## Residual 738  25.7961 0.96746
## Total    740  26.6638 1.00000
## ---
## Signif. codes:  0 '***' 0.001 '**' 0.01 '*' 0.05 '.' 0.1 ' ' 1
```

Relative abundance plot

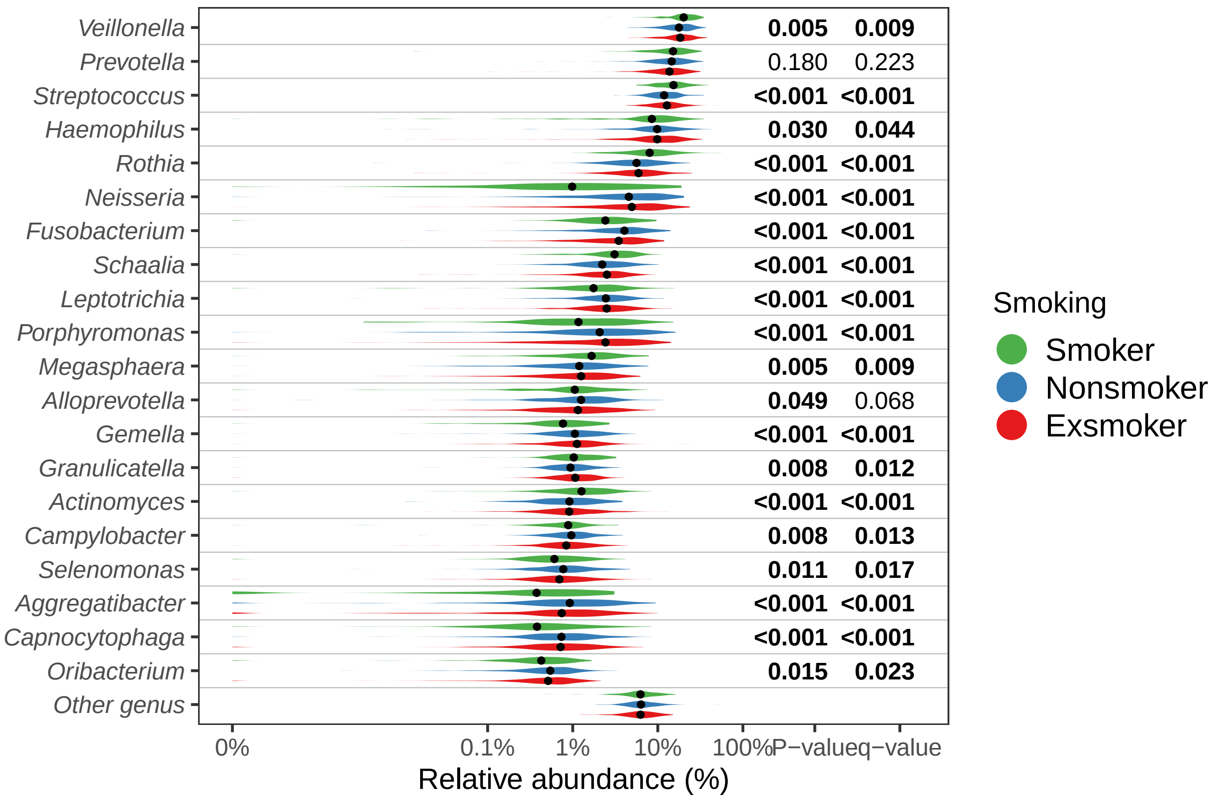

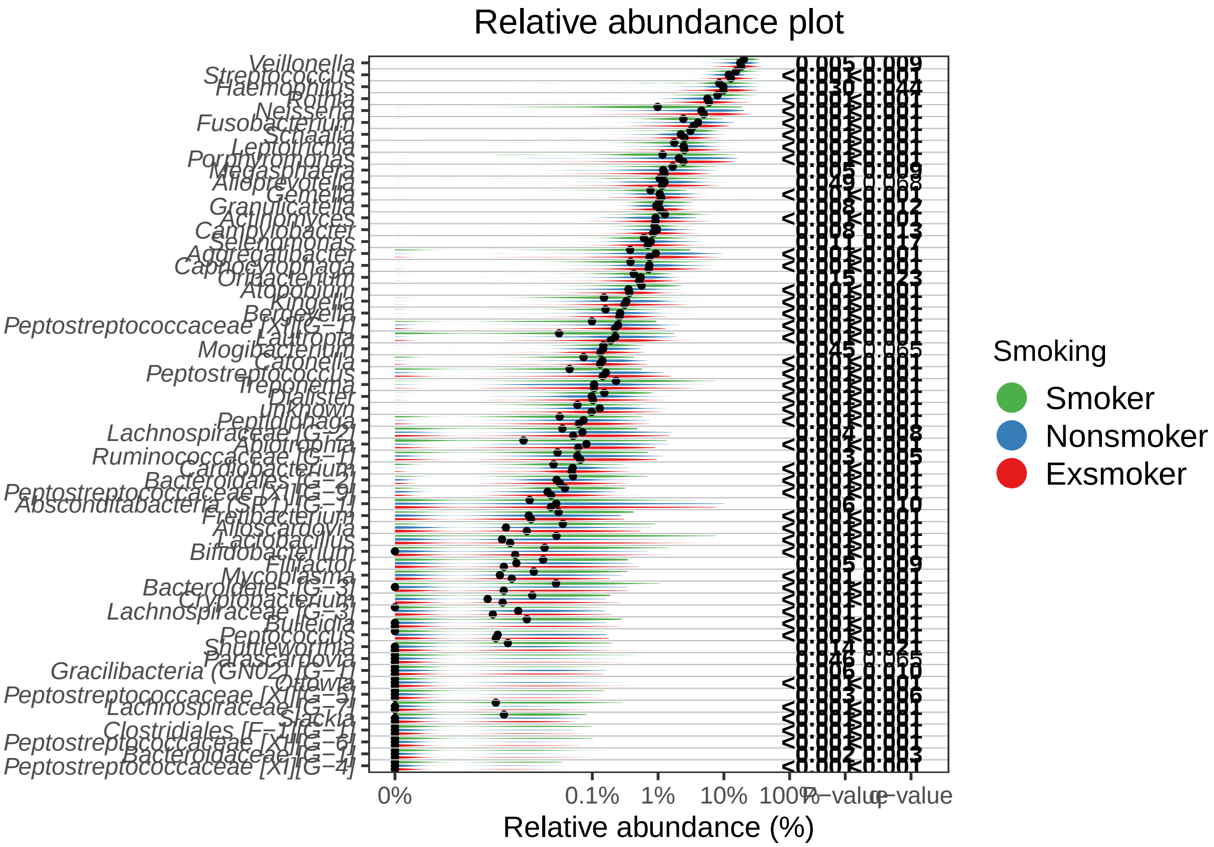
Relative abundance plot
Relative abundance (%)
0%
0.1%
1%
10%
100%
P−value
q−value
Smoking
Smoker
Nonsmoker
Exsmoker
Veillonella
Streptococcus
Haemophilus
Rothia
Neisseria
Fusobacterium
Schaalia
Leptotrichia
Porphyromonas
Megasphaera
Alloprevotella
Gemella
Granulicatella
Actinomyces
Campylobacter
Selenomonas
Aggregatibacter
Capnocytophaga
Oribacterium
Atopobium
Kingella
Bergeyella
Lautropia
Mogibacterium
Catonella
Peptostreptococcus
Treponema
Dialister
Peptidiphaga
Abiotrophia
Cardiobacterium
Freibacterium
Alloscardovia
Lactobacillus
Bifidobacterium
Filifactor
Mycoplasma
Cryptobacterium
Bulleidia
Peptococcus
Shuttleworthia
Parascardovia
Gracilibacteria (GN02) [G−1]
Ottowia
Slackia

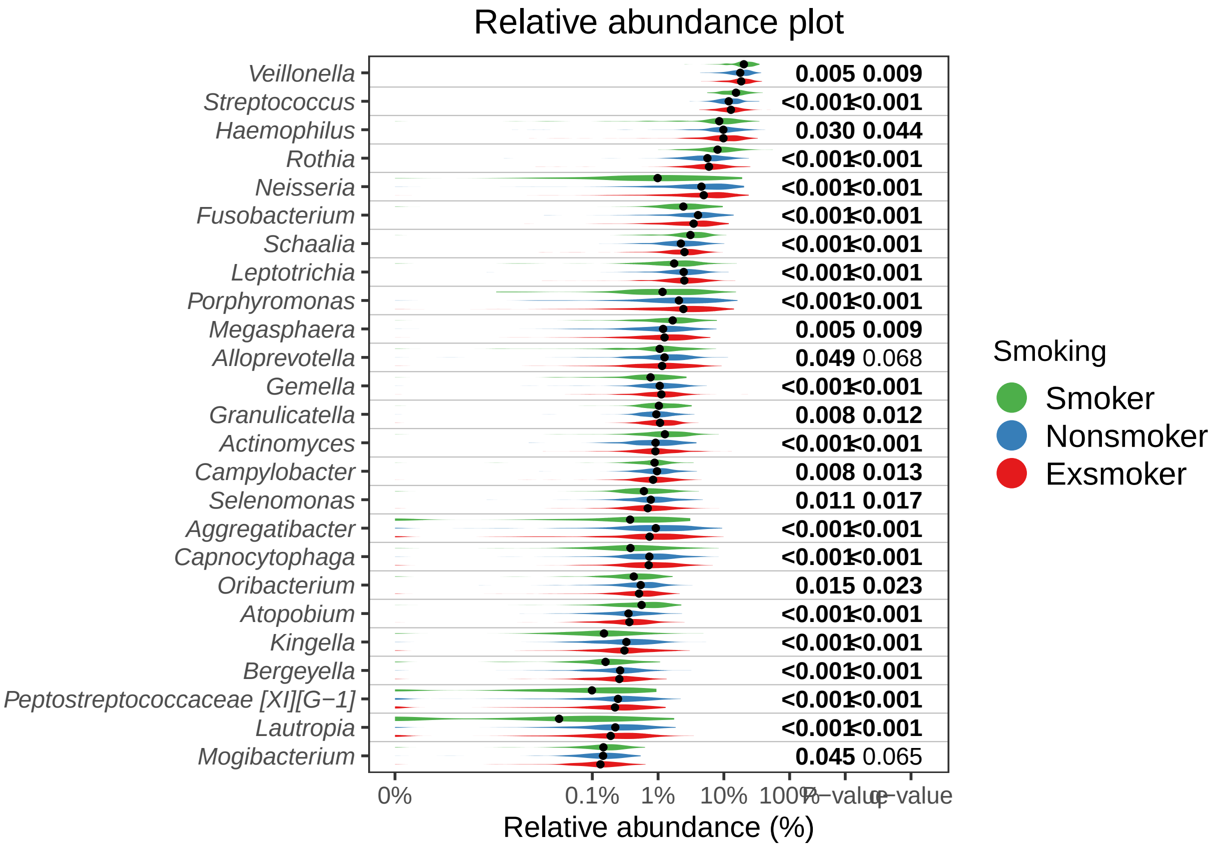

Relative abundance plot
Veillonella
Streptococcus
Haemophilus
Rothia
Neisseria
Fusobacterium
Schaalia
Leptotrichia
Porphyromonas
Megasphaera
Alloprevotella
Gemella
Granulicatella
Actinomyces
Campylobacter
Selenomonas
Aggregatibacter
Capnocytophaga
Oribacterium
Atopobium
Kingella
Bergeyella
Peptostreptococcaceae [XI][G−1]
Lautropia
Mogibacterium
0.005 0.009
<0.001 <0.001
0.030 0.044
<0.001 <0.001
<0.001 <0.001
<0.001 <0.001
<0.001 <0.001
<0.001 <0.001
<0.001 <0.001
0.005 0.009
0.049 0.068
<0.001 <0.001
0.008 0.012
<0.001 <0.001
0.008 0.013
0.011 0.017
<0.001 <0.001
<0.001 <0.001
0.015 0.023
<0.001 <0.001
<0.001 <0.001
<0.001 <0.001
<0.001 <0.001
<0.001 <0.001
0.045 0.065
0%
0.1%
1%
10%
100%
P−value
q−value
Relative abundance (%)
Smoking
Smoker
Nonsmoker
Exsmoker

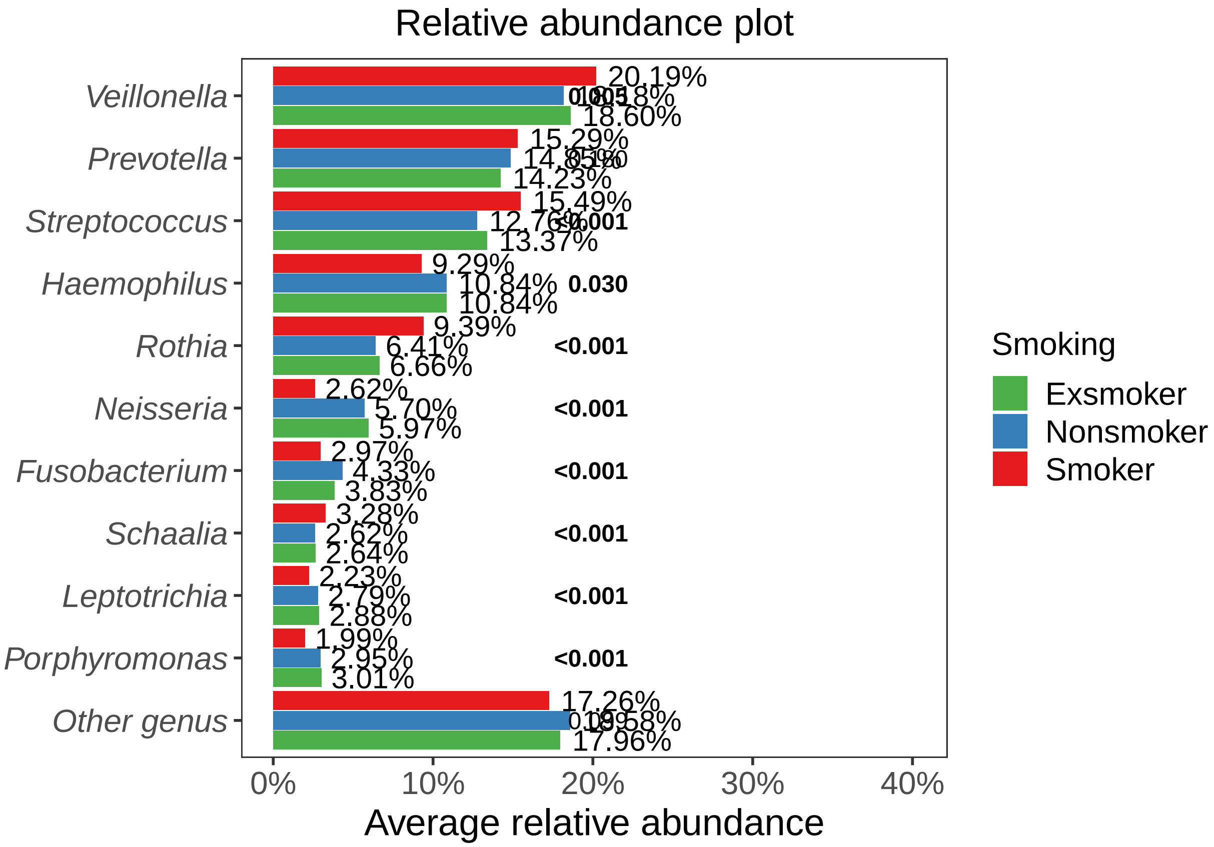

Relative abundance plot
Veillonella
Prevotella
Streptococcus
Haemophilus
Rothia
Neisseria
Fusobacterium
Schaalia
Leptotrichia
Porphyromonas
Other genus
20.19%
18.60%
15.29%
14.23%
15.49%
13.37%
9.29%
10.84%
10.84%
0.030
9.39%
6.41%
6.66%
<0.001
2.62%
5.70%
5.97%
<0.001
2.97%
4.33%
3.83%
<0.001
3.28%
2.62%
2.64%
<0.001
2.23%
2.79%
2.88%
<0.001
1.99%
2.95%
3.01%
<0.001
17.26%
17.96%
0%
10%
20%
30%
40%
Average relative abundance
Smoking
Exsmoker
Nonsmoker
Smoker

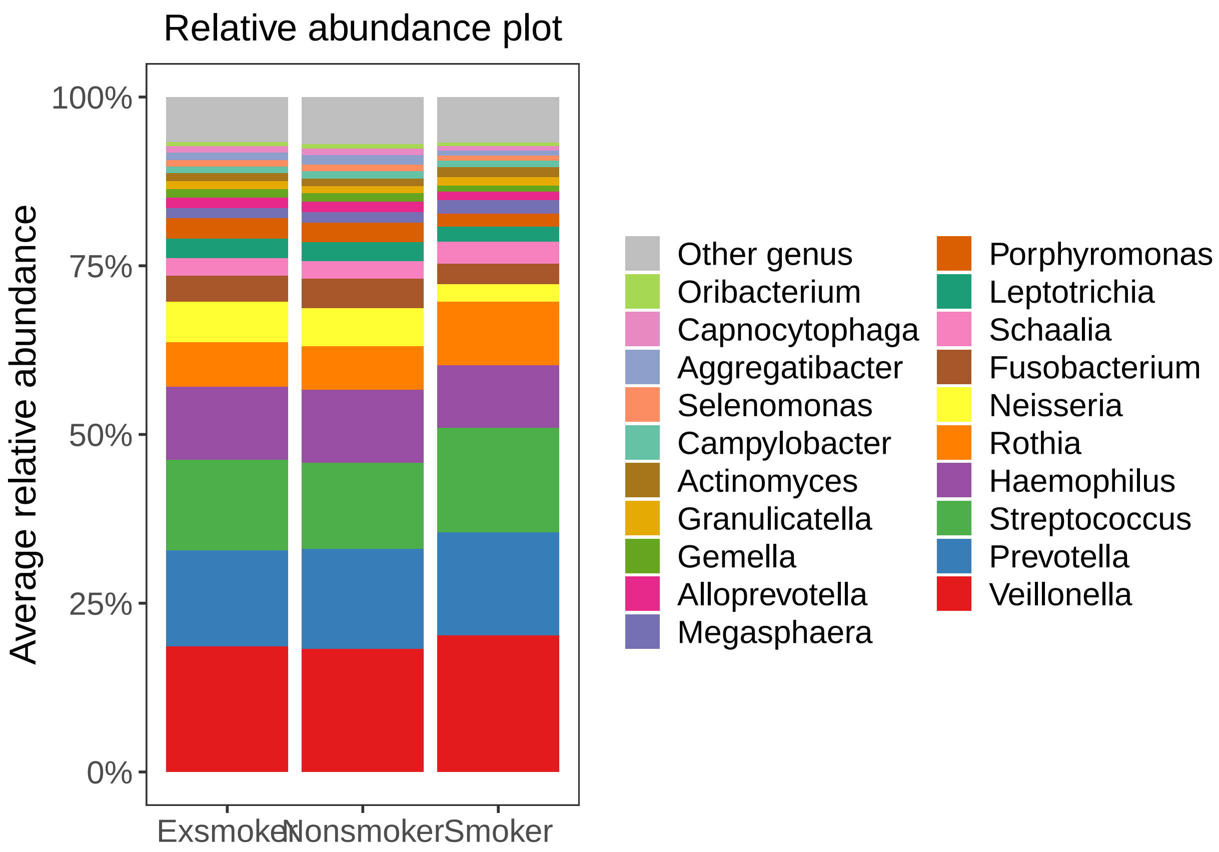

Relative abundance plot
Average relative abundance
100%
75%
50%
25%
0%
Exsmoker
Nonsmoker
Smoker
Other genus
Oribacterium
Capnocytophaga
Aggregatibacter
Selenomonas
Campylobacter
Actinomyces
Granulicatella
Gemella
Alloprevotella
Megasphaera
Porphyromonas
Leptotrichia
Schaalia
Fusobacterium
Neisseria
Rothia
Haemophilus
Streptococcus
Prevotella
Veillonella


```
## [1] "bp_cat"
```

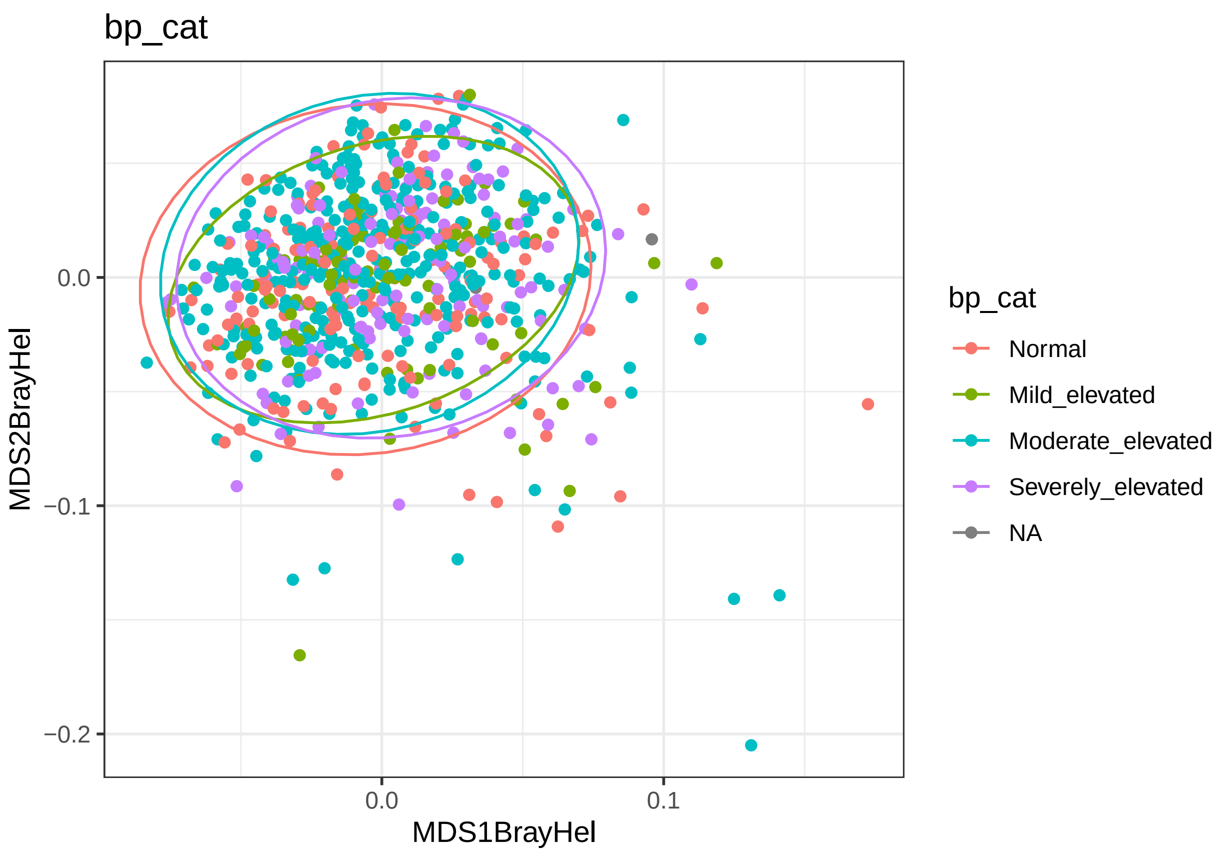


```
## Permutation test for adonis under reduced model
## Terms added sequentially (first to last)
## Permutation: free
## Number of permutations: 999
##
## adonis2(formula = formula(paste("distmatrix ~ ", i)), data = Phe3, permutations = 999, by = "terms")
##           Df SumOfSqs      R2      F Pr(>F)
## bp_cat     3   0.1107 0.00413 1.0236   0.39
## Residual 740  26.6783 0.99587
## Total    743  26.7891 1.00000
```

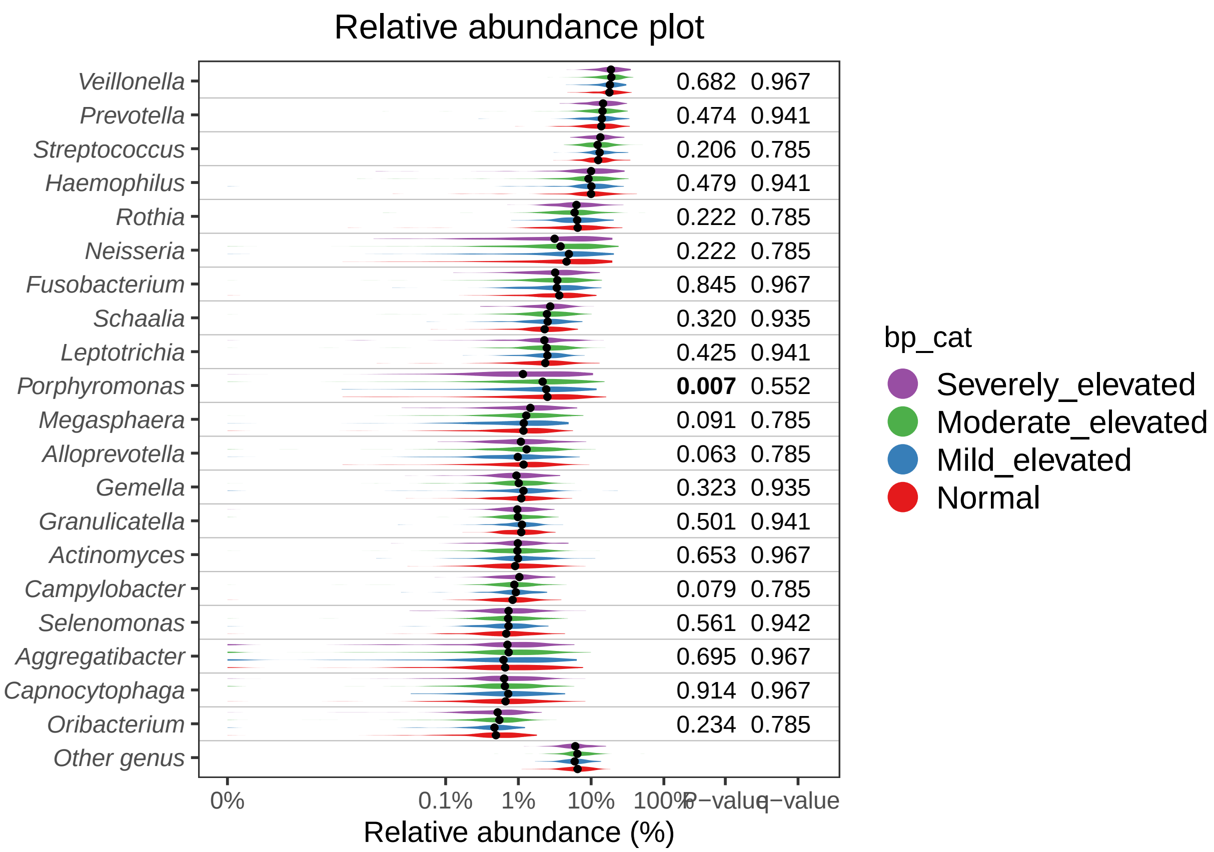
Relative abundance plot
Veillonella
Prevotella
Streptococcus
Haemophilus
Rothia
Neisseria
Fusobacterium
Schaalia
Leptotrichia
Porphyromonas
Megasphaera
Alloprevotella
Gemella
Granulicatella
Actinomyces
Campylobacter
Selenomonas
Aggregatibacter
Capnocytophaga
Oribacterium
Other genus
0.682 0.967
0.474 0.941
0.206 0.785
0.479 0.941
0.222 0.785
0.222 0.785
0.845 0.967
0.320 0.935
0.425 0.941
0.007 0.552
0.091 0.785
0.063 0.785
0.323 0.935
0.501 0.941
0.653 0.967
0.079 0.785
0.561 0.942
0.695 0.967
0.914 0.967
0.234 0.785
0%
0.1%
1%
10%
100%
P−value
q−value
Relative abundance (%)
bp_cat
Severely_elevated
Moderate_elevated
Mild_elevated
Normal

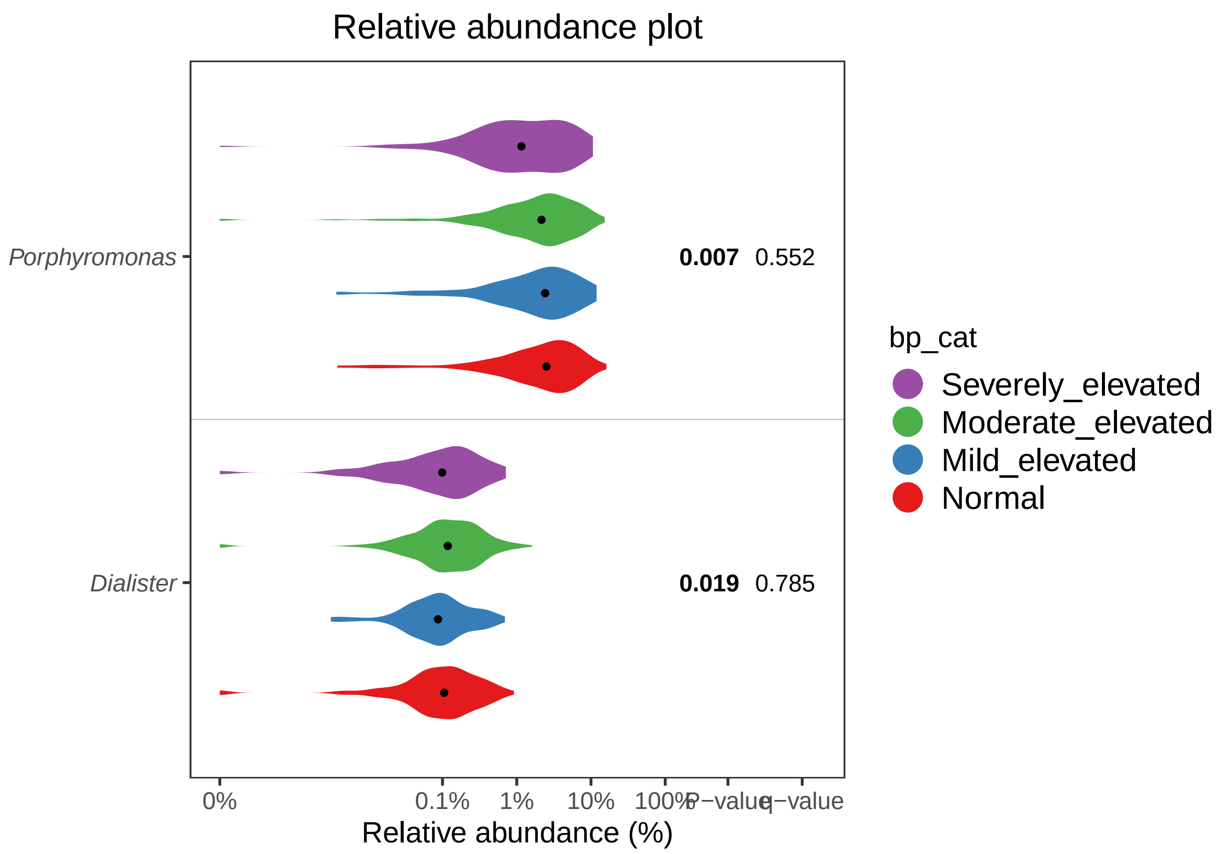
Relative abundance plot
Porphyromonas
Dialister
0.007
0.552
0.019
0.785
0%
0.1%
1%
10%
100%
P−value
q−value
Relative abundance (%)
bp_cat
Severely_elevated
Moderate_elevated
Mild_elevated
Normal

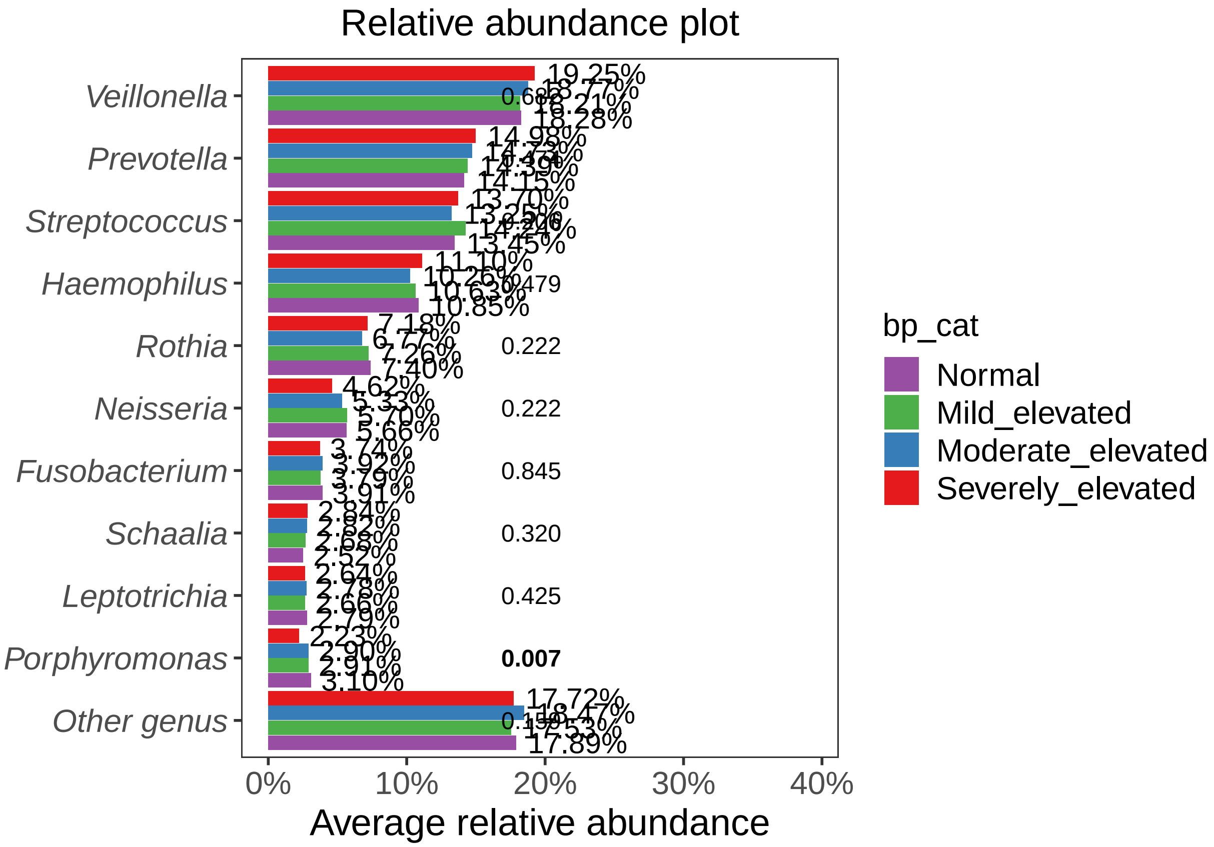

Relative abundance plot
Veillonella
Prevotella
Streptococcus
Haemophilus
Rothia
Neisseria
Fusobacterium
Schaalia
Leptotrichia
Porphyromonas
Other genus
19.25%
18.77%
18.21%
18.28%
14.98%
14.15%
13.70%
13.45%
11.10%
10.85%
0.479
7.18%
6.77%
7.26%
7.40%
0.222
4.62%
5.33%
5.70%
5.66%
0.222
3.74%
3.92%
3.91%
0.845
2.84%
2.82%
2.52%
0.320
2.79%
0.425
2.23%
2.90%
2.91%
3.10%
0.007
17.72%
18.47%
17.53%
17.89%
0%
10%
20%
30%
40%
Average relative abundance
bp_cat
Normal
Mild_elevated
Moderate_elevated
Severely_elevated

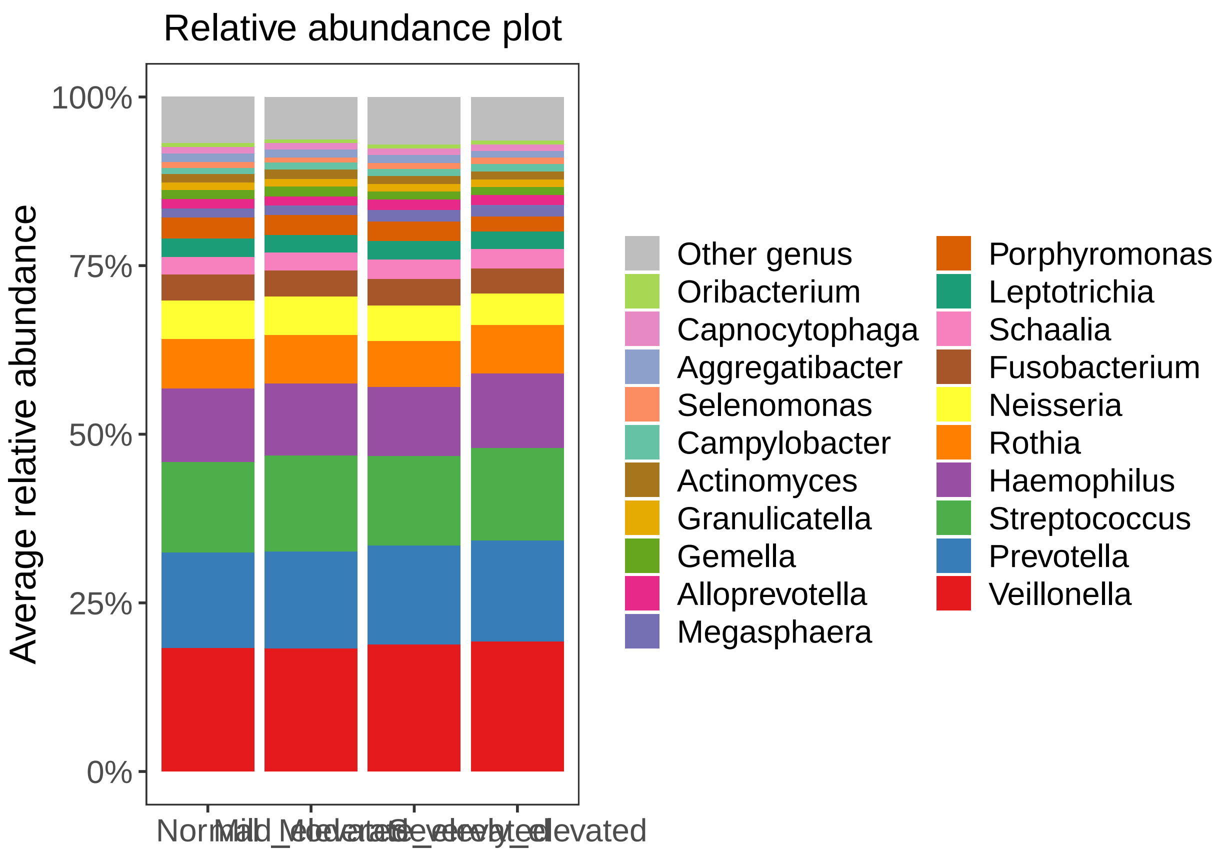
Relative abundance plot
Average relative abundance
100%
75%
50%
25%
0%
Other genus
Oribacterium
Capnocytophaga
Aggregatibacter
Selenomonas
Campylobacter
Actinomyces
Granulicatella
Gemella
Alloprevotella
Megasphaera
Porphyromonas
Leptotrichia
Schaalia
Fusobacterium
Neisseria
Rothia
Haemophilus
Streptococcus
Prevotella
Veillonella

```
## [1] "sbp_cat"
```

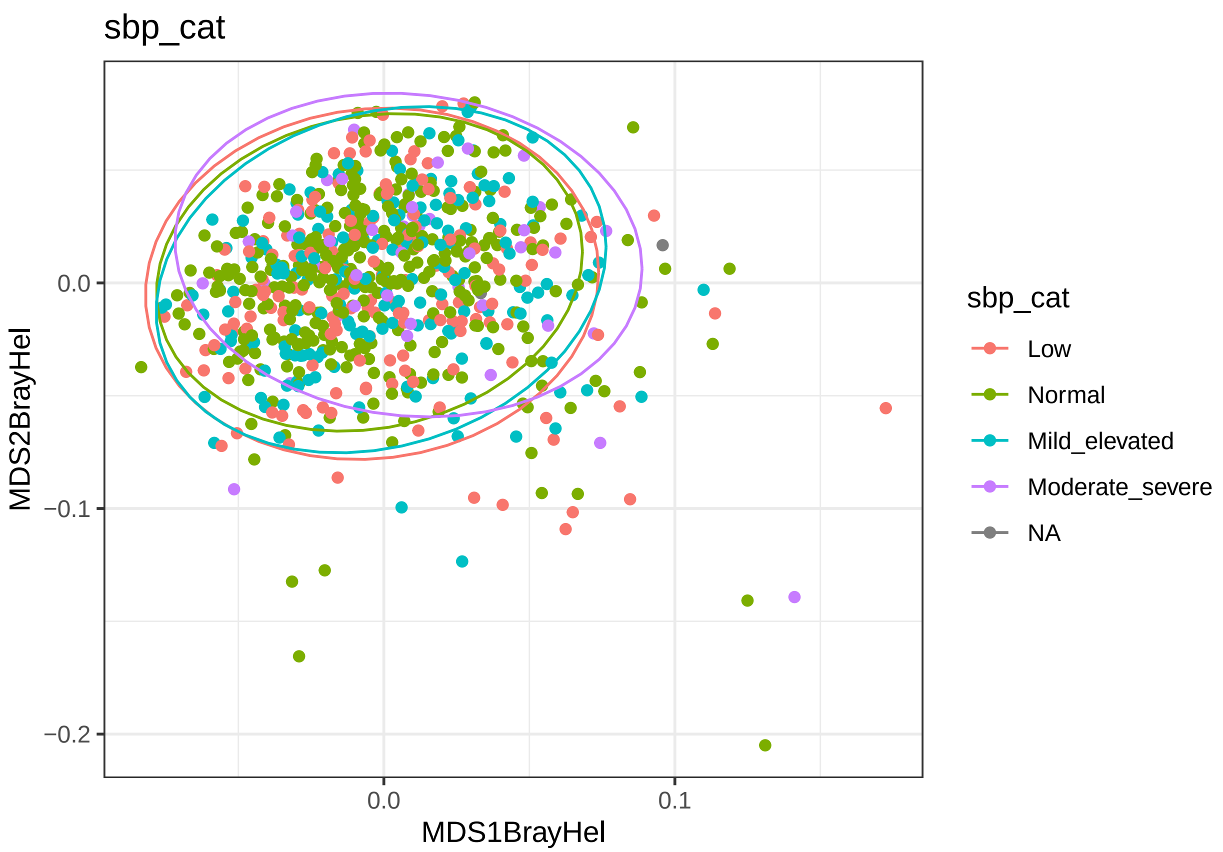


```
## Permutation test for adonis under reduced model
## Terms added sequentially (first to last)
## Permutation: free
## Number of permutations: 999
##
## adonis2(formula = formula(paste("distmatrix ~ ", i)), data = Phe3, permutations = 999, by = "terms")
##           Df SumOfSqs      R2      F Pr(>F)
## sbp_cat    3   0.1271 0.00474 1.1756  0.212
## Residual 740  26.6620 0.99526
## Total    743  26.7891 1.00000
```

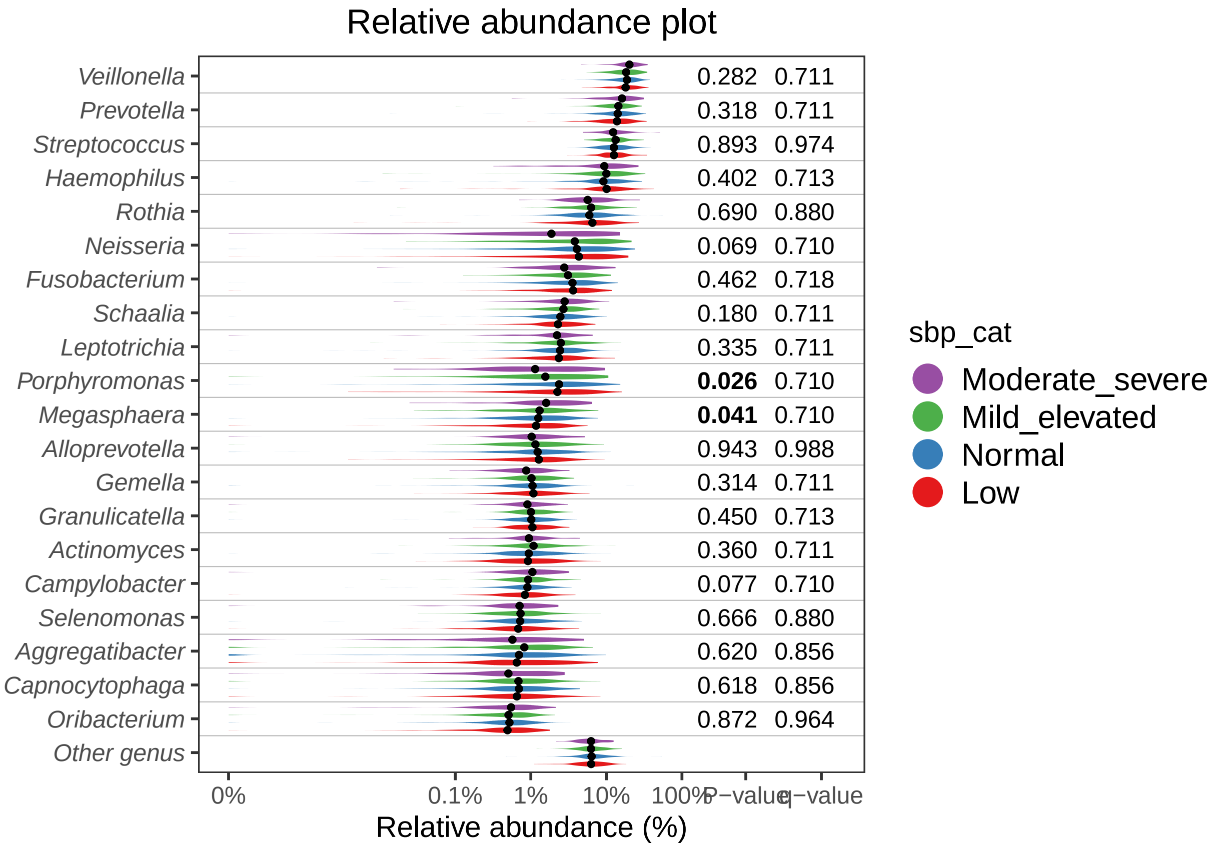

Relative abundance plot
Veillonella
Prevotella
Streptococcus
Haemophilus
Rothia
Neisseria
Fusobacterium
Schaalia
Leptotrichia
Porphyromonas
Megasphaera
Alloprevotella
Gemella
Granulicatella
Actinomyces
Campylobacter
Selenomonas
Aggregatibacter
Capnocytophaga
Oribacterium
Other genus
0.282 0.711
0.318 0.711
0.893 0.974
0.402 0.713
0.690 0.880
0.069 0.710
0.462 0.718
0.180 0.711
0.335 0.711
0.026 0.710
0.041 0.710
0.943 0.988
0.314 0.711
0.450 0.713
0.360 0.711
0.077 0.710
0.666 0.880
0.620 0.856
0.618 0.856
0.872 0.964
0%
0.1%
1%
10%
100%
P−value
q−value
Relative abundance (%)
sbp_cat
Moderate_severe
Mild_elevated
Normal
Low

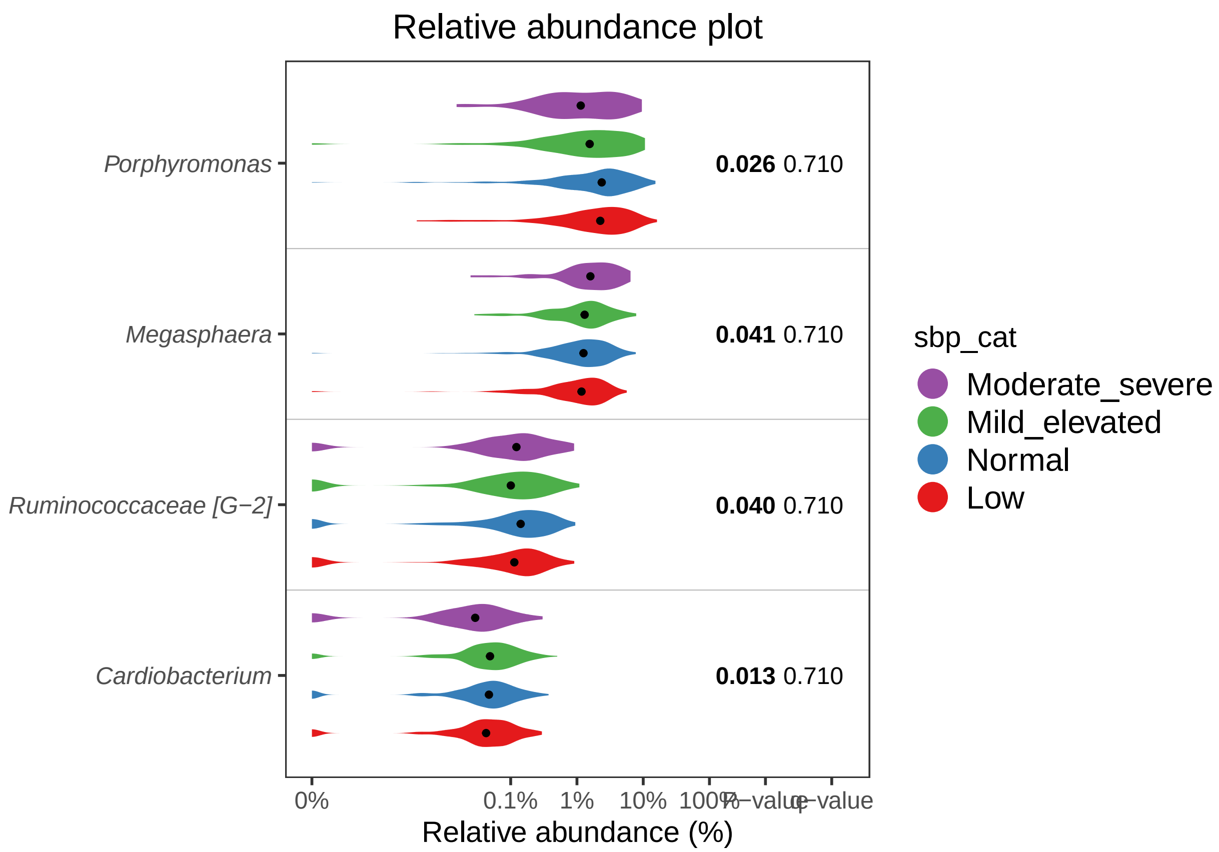
Relative abundance plot
Porphyromonas
Megasphaera
Ruminococcaceae [G−2]
Cardiobacterium
0.026 0.710
0.041 0.710
0.040 0.710
0.013 0.710
0%
0.1%
1%
10%
100%
P−value
q−value
Relative abundance (%)
sbp_cat
Moderate_severe
Mild_elevated
Normal
Low

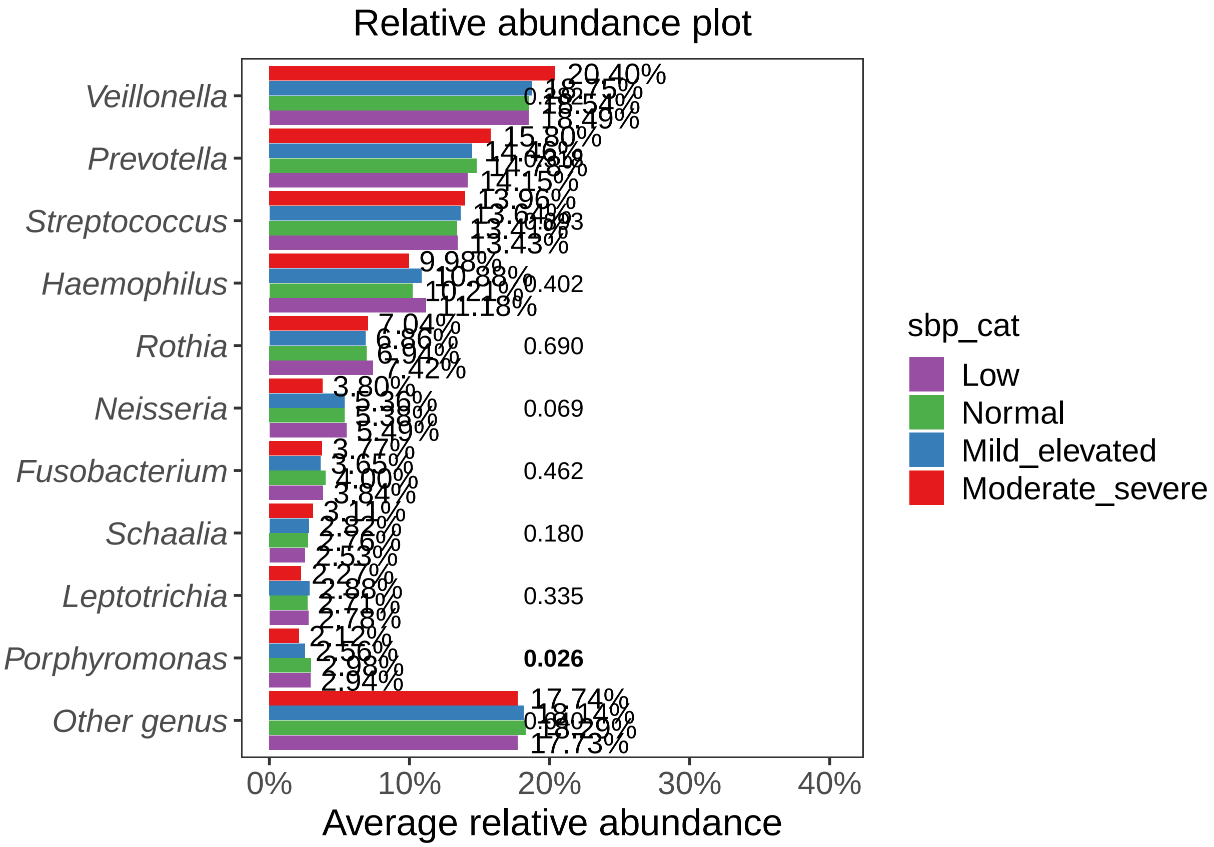
Relative abundance plot
Veillonella
Prevotella
Streptococcus
Haemophilus
Rothia
Neisseria
Fusobacterium
Schaalia
Leptotrichia
Porphyromonas
Other genus
0.402
0.690
0.069
0.462
0.180
0.335
0.026
0%
10%
20%
30%
40%
Average relative abundance
sbp_cat
Low
Normal
Mild_elevated
Moderate_severe

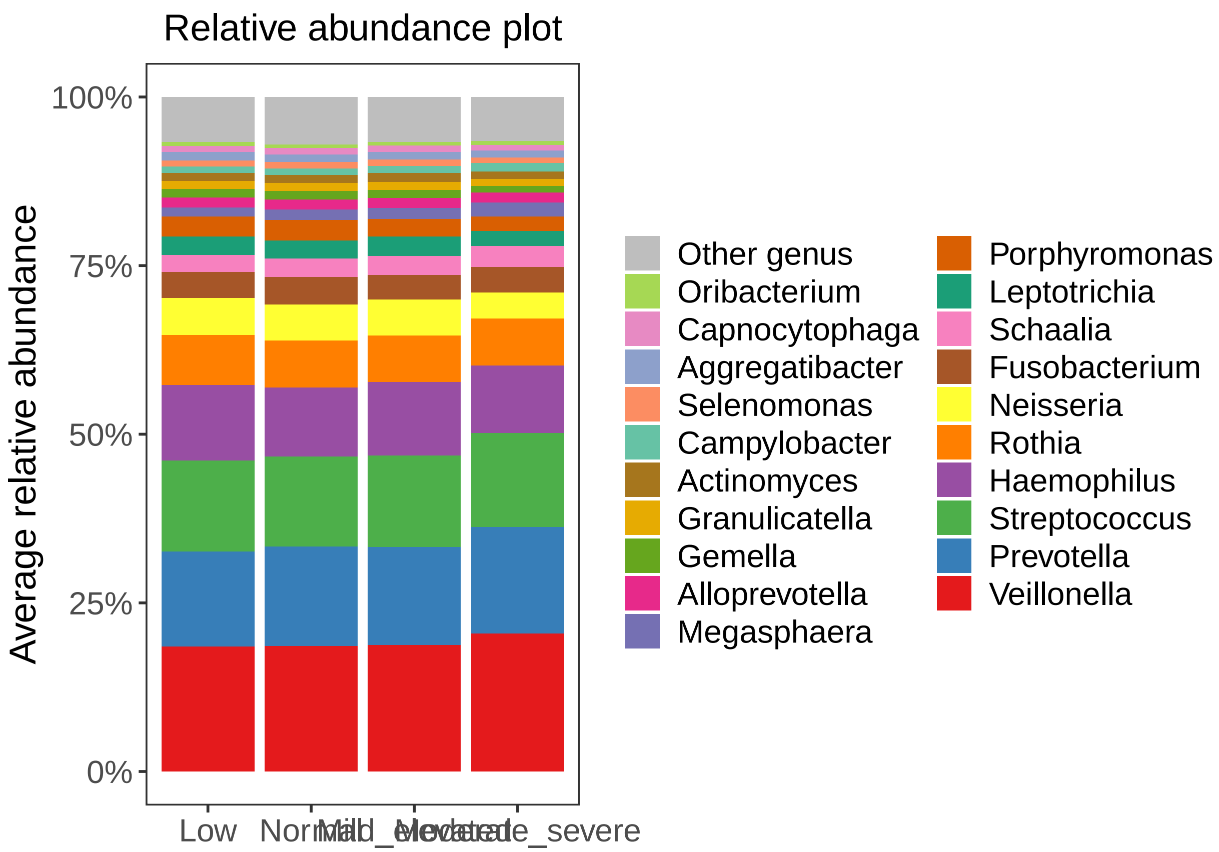
Relative abundance plot
Average relative abundance
100%
75%
50%
25%
0%
Low
Normal
Mild_elevated
Moderate_severe
Other genus
Oribacterium
Capnocytophaga
Aggregatibacter
Selenomonas
Campylobacter
Actinomyces
Granulicatella
Gemella
Alloprevotella
Megasphaera
Porphyromonas
Leptotrichia
Schaalia
Fusobacterium
Neisseria
Rothia
Haemophilus
Streptococcus
Prevotella
Veillonella

```
## [1] "dbp_cat"
```

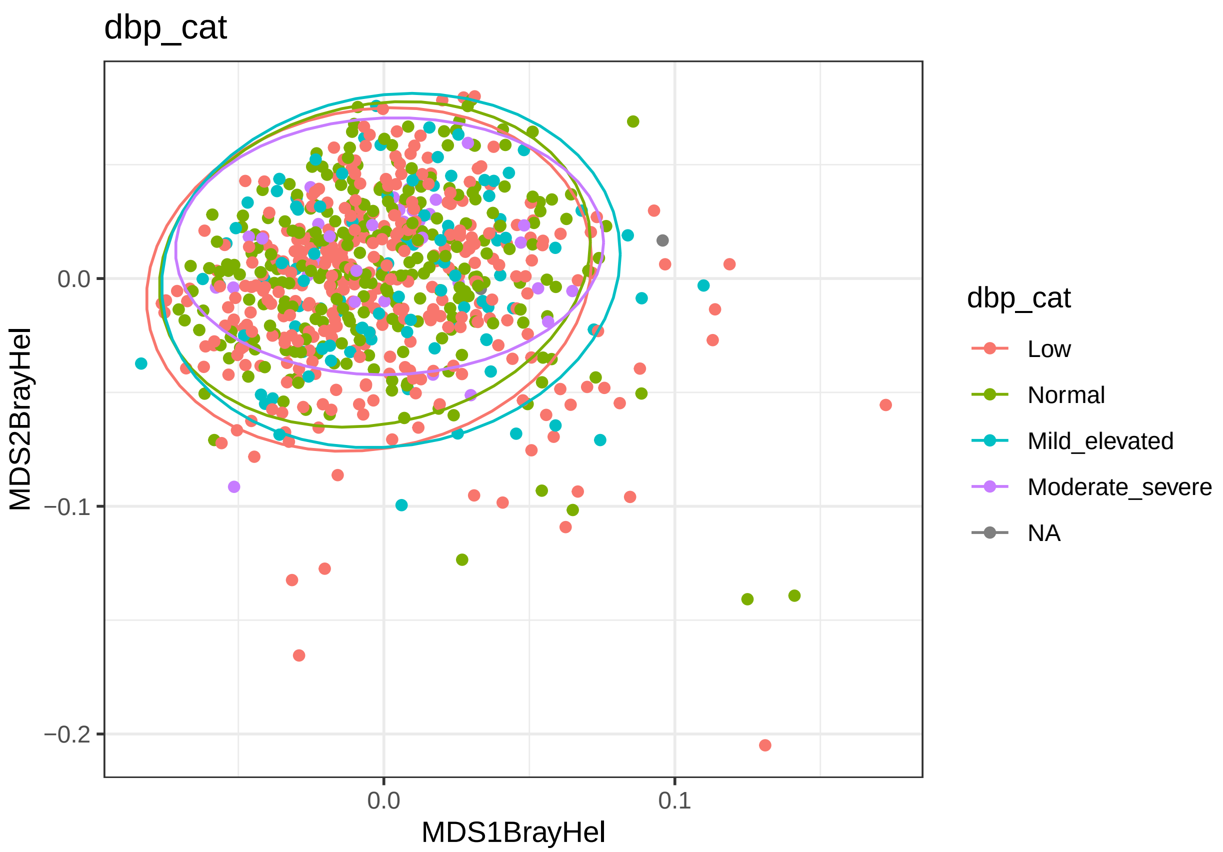


```
## Permutation test for adonis under reduced model
## Terms added sequentially (first to last)
## Permutation: free
## Number of permutations: 999
##
## adonis2(formula = formula(paste("distmatrix ~ ", i)), data = Phe3, permutations = 999, by = "terms")
##           Df SumOfSqs      R2      F Pr(>F)
## dbp_cat    3   0.1509 0.00563 1.3976  0.083 .
## Residual 740  26.6381 0.99437
## Total    743  26.7891 1.00000
## ---
## Signif. codes:  0 '***' 0.001 '**' 0.01 '*' 0.05 '.' 0.1 ' ' 1
```

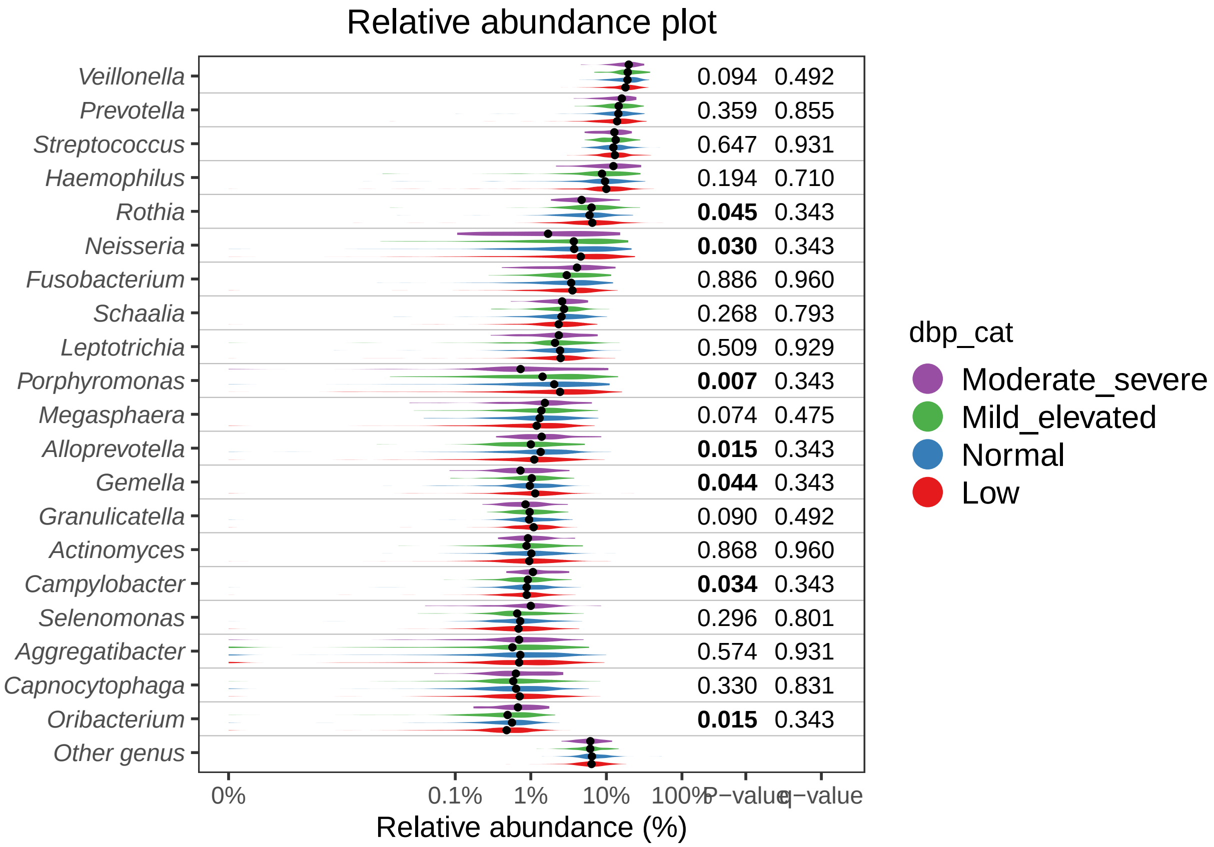
Relative abundance plot
Veillonella 0.094 0.492
Prevotella 0.359 0.855
Streptococcus 0.647 0.931
Haemophilus 0.194 0.710
Rothia 0.045 0.343
Neisseria 0.030 0.343
Fusobacterium 0.886 0.960
Schaalia 0.268 0.793
Leptotrichia 0.509 0.929
Porphyromonas 0.007 0.343
Megasphaera 0.074 0.475
Alloprevotella 0.015 0.343
Gemella 0.044 0.343
Granulicatella 0.090 0.492
Actinomyces 0.868 0.960
Campylobacter 0.034 0.343
Selenomonas 0.296 0.801
Aggregatibacter 0.574 0.931
Capnocytophaga 0.330 0.831
Oribacterium 0.015 0.343
Other genus
0%
0.1%
1%
10%
100%
P−value
q−value
Relative abundance (%)
dbp_cat
Moderate_severe
Mild_elevated
Normal
Low

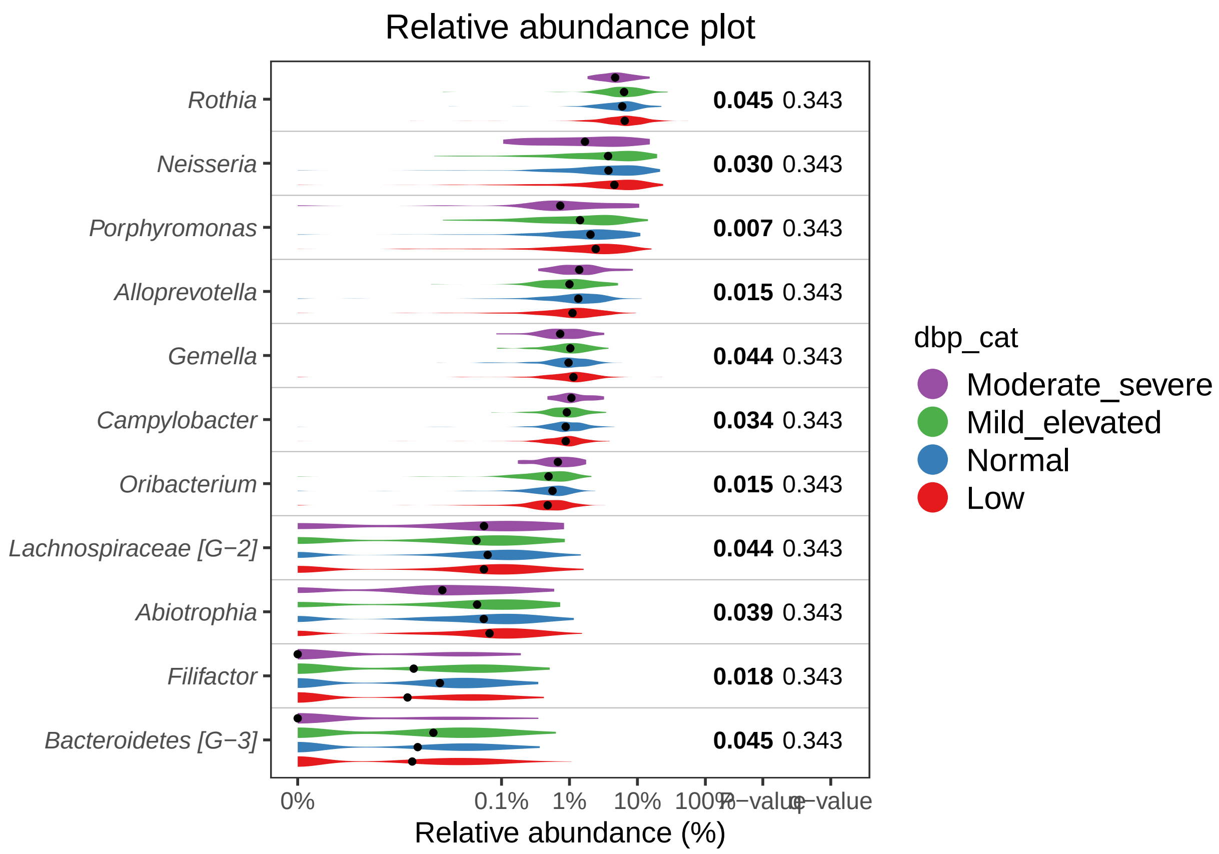
Relative abundance plot
Rothia
Neisseria
Porphyromonas
Alloprevotella
Gemella
Campylobacter
Oribacterium
Lachnospiraceae [G−2]
Abiotrophia
Filifactor
Bacteroidetes [G−3]
0.045 0.343
0.030 0.343
0.007 0.343
0.015 0.343
0.044 0.343
0.034 0.343
0.015 0.343
0.044 0.343
0.039 0.343
0.018 0.343
0.045 0.343
0%
0.1%
1%
10%
100%
P−value
q−value
Relative abundance (%)
dbp_cat
Moderate_severe
Mild_elevated
Normal
Low

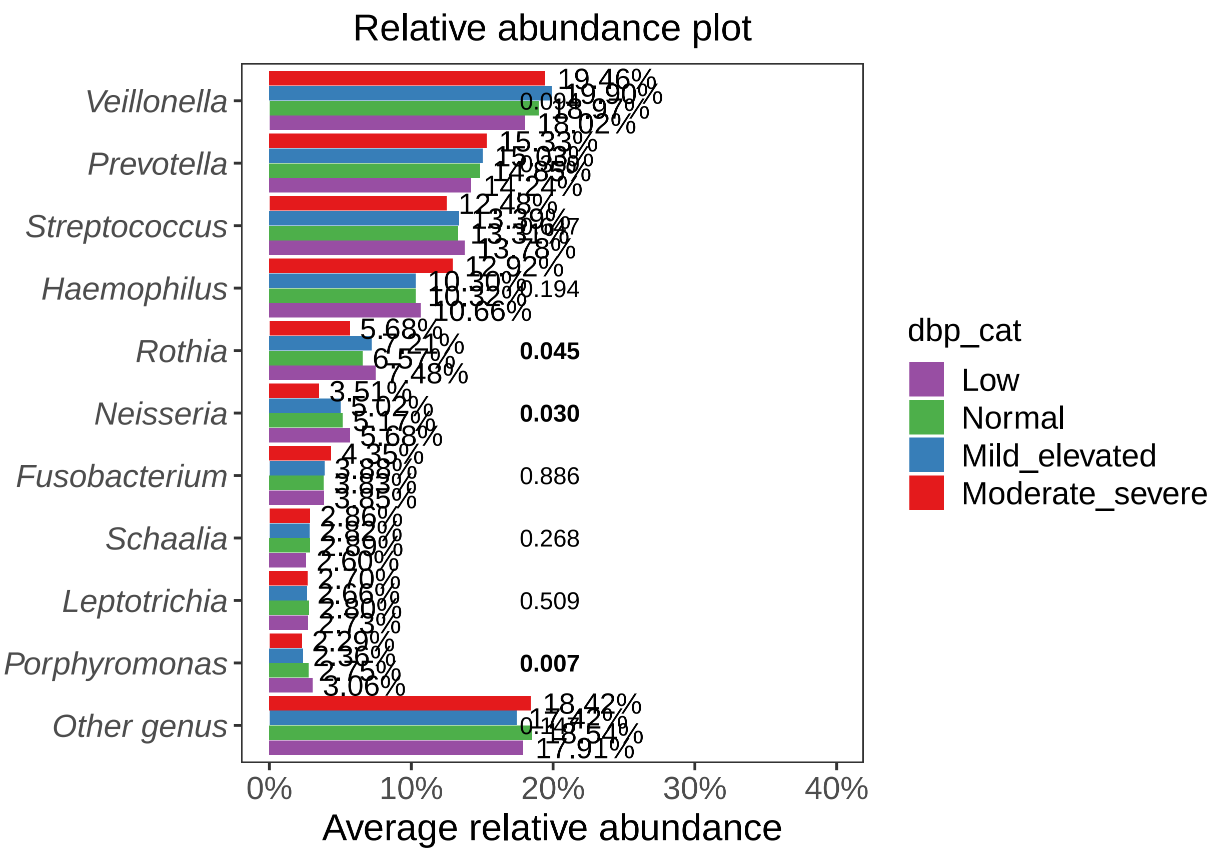

Relative abundance plot
Veillonella
Prevotella
Streptococcus
Haemophilus
Rothia
Neisseria
Fusobacterium
Schaalia
Leptotrichia
Porphyromonas
Other genus
19.46%
18.02%
14.24%
12.48%
13.78%
12.92%
0.194
10.66%
5.68%
0.045
7.48%
3.51%
0.030
5.68%
4.35%
0.886
3.85%
0.268
2.60%
0.509
2.73%
2.29%
0.007
3.06%
18.42%
17.91%
0%
10%
20%
30%
40%
Average relative abundance
dbp_cat
Low
Normal
Mild_elevated
Moderate_severe

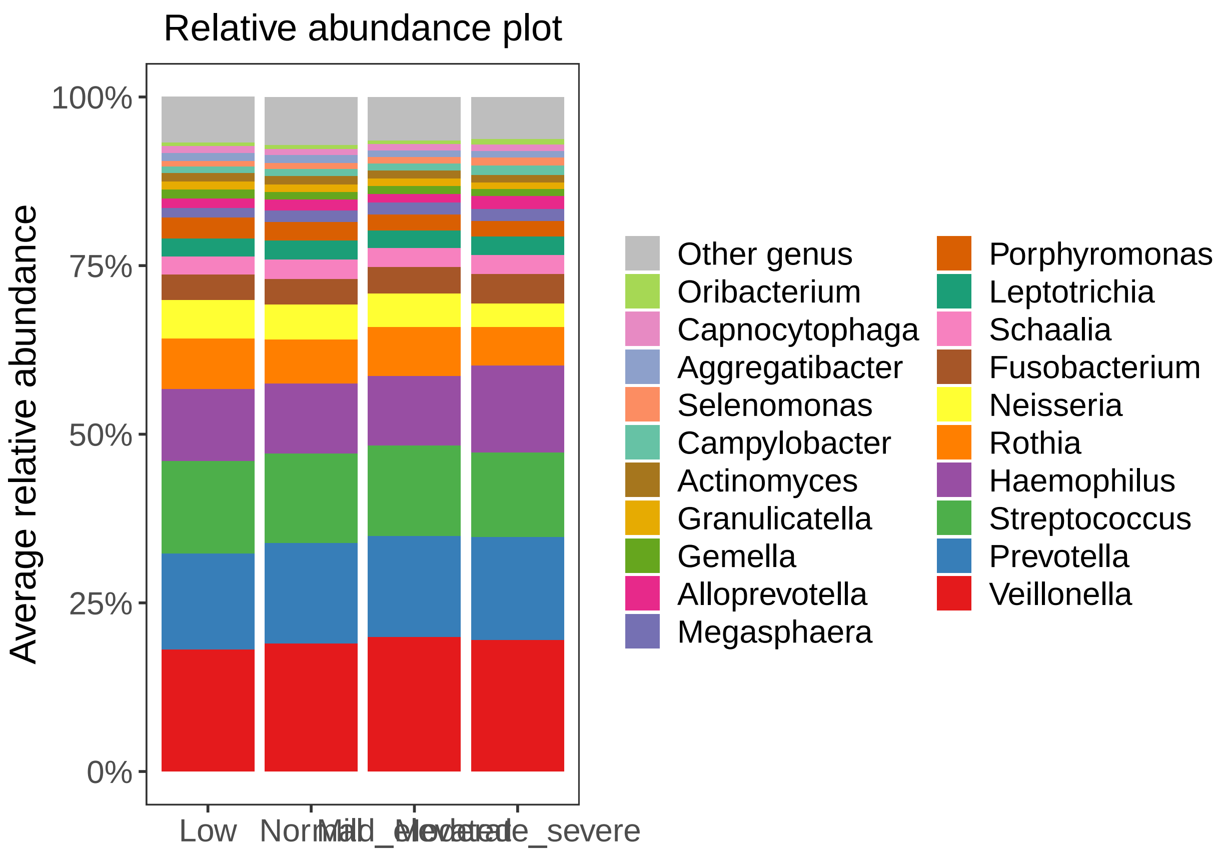
Relative abundance plot
Average relative abundance
100%
75%
50%
25%
0%
Low
Normal
Mild_elevated
Moderate_severe
Other genus
Oribacterium
Capnocytophaga
Aggregatibacter
Selenomonas
Campylobacter
Actinomyces
Granulicatella
Gemella
Alloprevotella
Megasphaera
Porphyromonas
Leptotrichia
Schaalia
Fusobacterium
Neisseria
Rothia
Haemophilus
Streptococcus
Prevotella
Veillonella

```
## [1] "hr_cat"
```

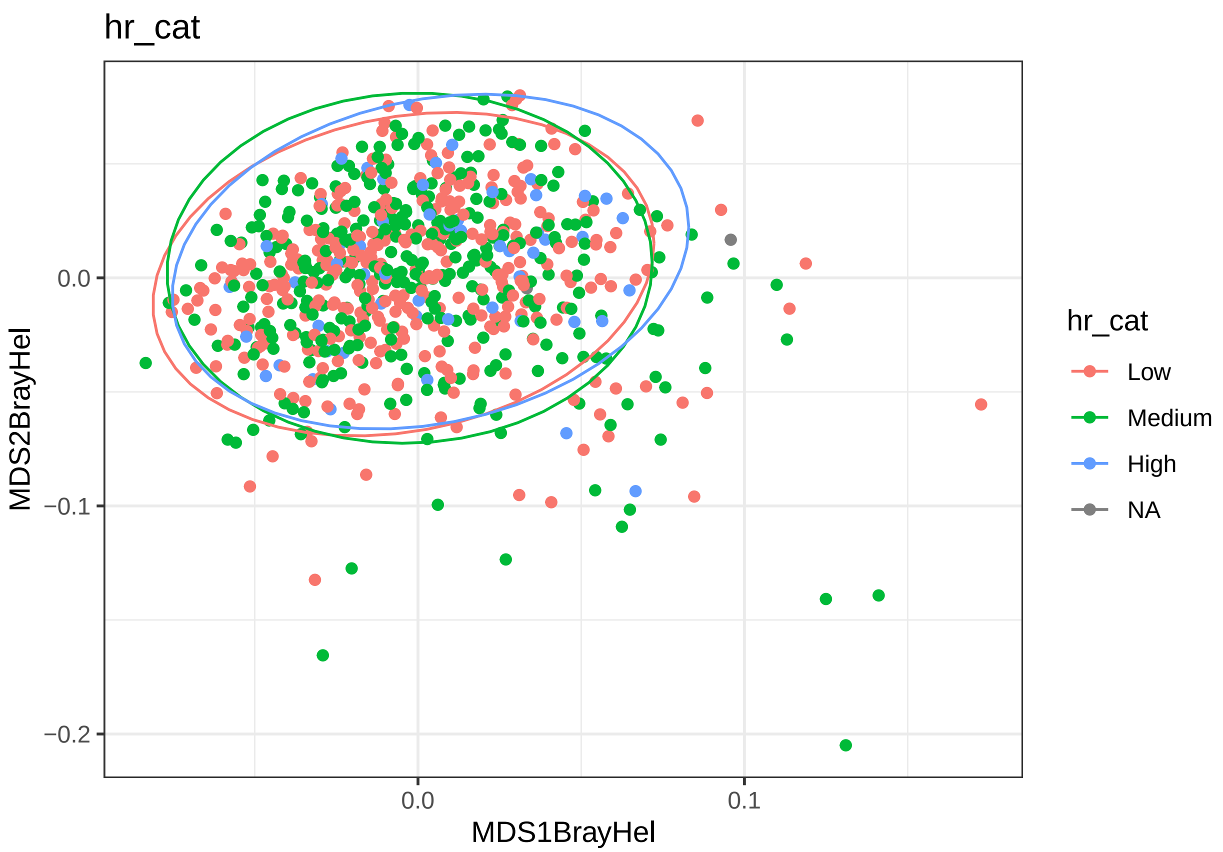


```
## Permutation test for adonis under reduced model
## Terms added sequentially (first to last)
## Permutation: free
## Number of permutations: 999
##
## adonis2(formula = formula(paste("distmatrix ~ ", i)), data = Phe3, permutations = 999, by = "terms")
##           Df SumOfSqs      R2      F Pr(>F)
## hr_cat     2    0.101 0.00377 1.4016  0.103
## Residual 741   26.688 0.99623
## Total    743   26.789 1.00000
```

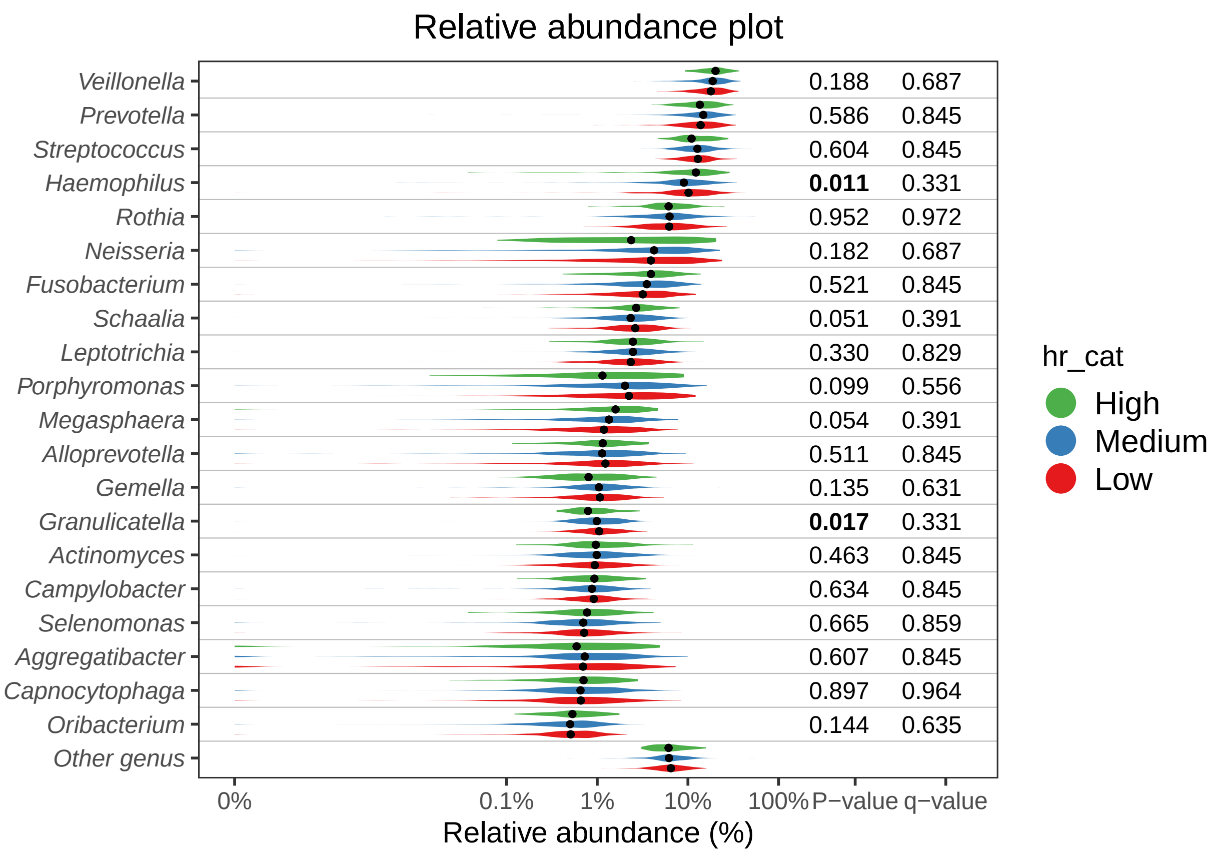

Relative abundance plot
Veillonella
Prevotella
Streptococcus
Haemophilus
Rothia
Neisseria
Fusobacterium
Schaalia
Leptotrichia
Porphyromonas
Megasphaera
Alloprevotella
Gemella
Granulicatella
Actinomyces
Campylobacter
Selenomonas
Aggregatibacter
Capnocytophaga
Oribacterium
Other genus
0.188 0.687
0.586 0.845
0.604 0.845
0.011 0.331
0.952 0.972
0.182 0.687
0.521 0.845
0.051 0.391
0.330 0.829
0.099 0.556
0.054 0.391
0.511 0.845
0.135 0.631
0.017 0.331
0.463 0.845
0.634 0.845
0.665 0.859
0.607 0.845
0.897 0.964
0.144 0.635
0%
0.1%
1%
10%
100%
P−value
q−value
Relative abundance (%)
hr_cat
High
Medium
Low

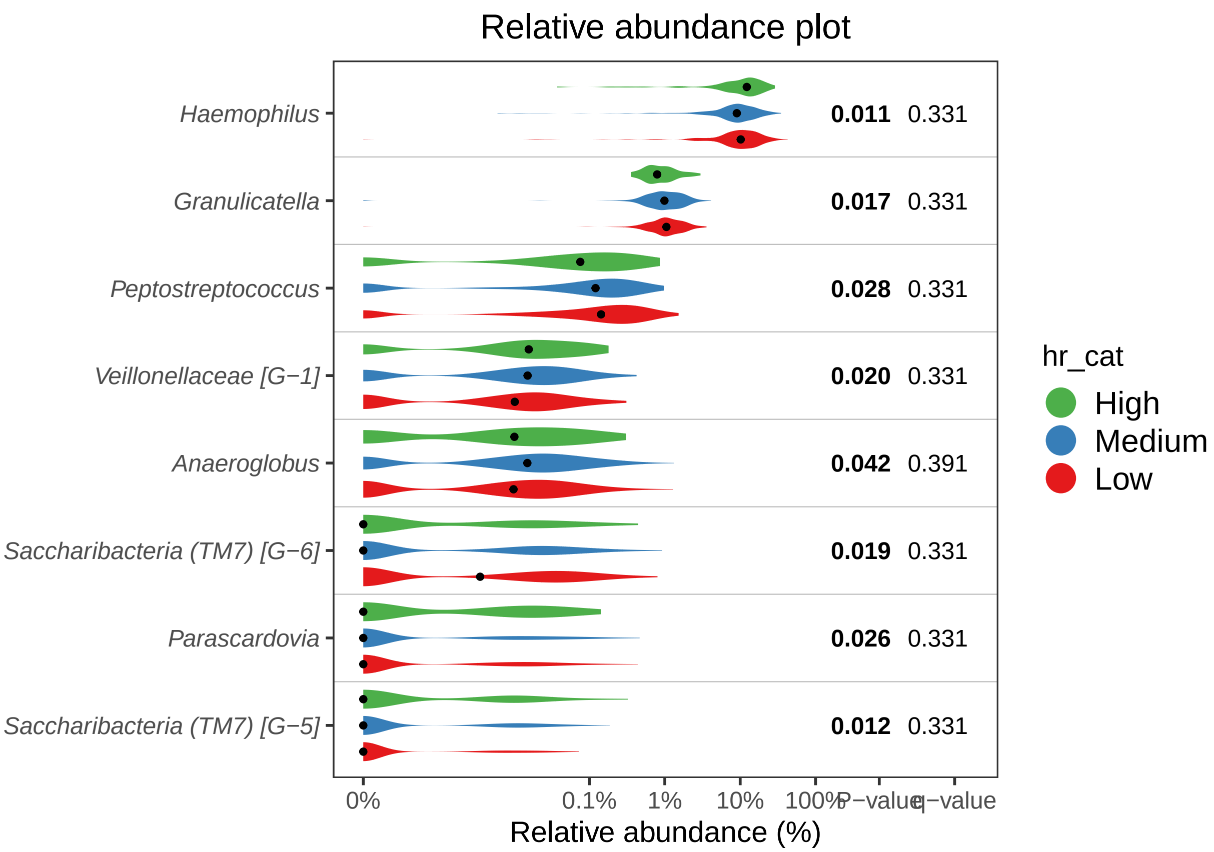

Relative abundance plot
Haemophilus
Granulicatella
Peptostreptococcus
Veillonellaceae [G−1]
Anaeroglobus
Saccharibacteria (TM7) [G−6]
Parascardovia
Saccharibacteria (TM7) [G−5]
0.011 0.331
0.017 0.331
0.028 0.331
0.020 0.331
0.042 0.391
0.019 0.331
0.026 0.331
0.012 0.331
0%
0.1%
1%
10%
100%
P−value
q−value
Relative abundance (%)
hr_cat
High
Medium
Low

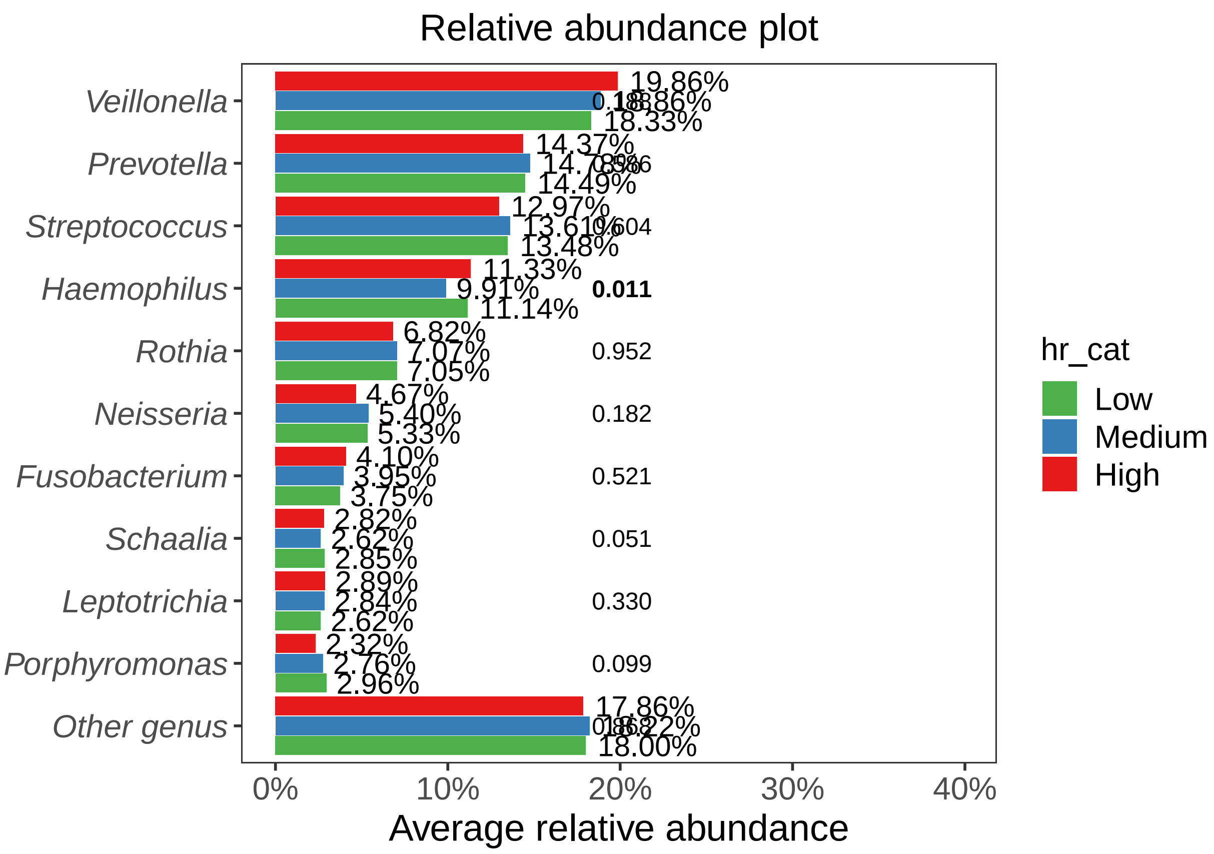
Relative abundance plot
Veillonella
Prevotella
Streptococcus
Haemophilus
Rothia
Neisseria
Fusobacterium
Schaalia
Leptotrichia
Porphyromonas
Other genus
19.86%
18.33%
14.37%
14.49%
12.97%
13.48%
11.33%
9.91%
11.14%
0.011
6.82%
7.07%
7.05%
0.952
4.67%
5.40%
5.33%
0.182
4.10%
3.95%
3.75%
0.521
2.82%
2.62%
2.85%
0.051
2.89%
2.84%
2.62%
0.330
2.32%
2.76%
2.96%
0.099
17.86%
18.00%
0%
10%
20%
30%
40%
Average relative abundance
hr_cat
Low
Medium
High

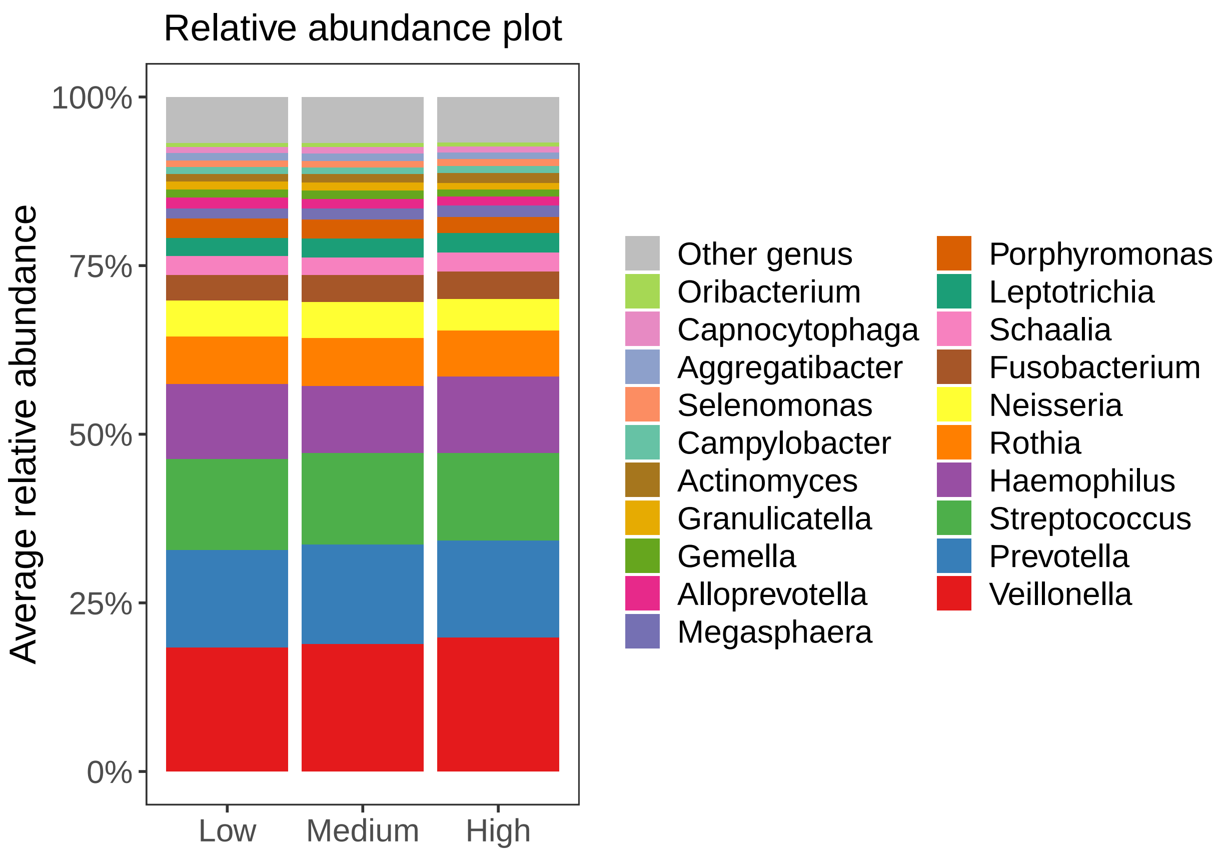
Relative abundance plot
Average relative abundance
100%
75%
50%
25%
0%
Low
Medium
High
Other genus
Oribacterium
Capnocytophaga
Aggregatibacter
Selenomonas
Campylobacter
Actinomyces
Granulicatella
Gemella
Alloprevotella
Megasphaera
Porphyromonas
Leptotrichia
Schaalia
Fusobacterium
Neisseria
Rothia
Haemophilus
Streptococcus
Prevotella
Veillonella

```
## [1] "pp_cat"
```

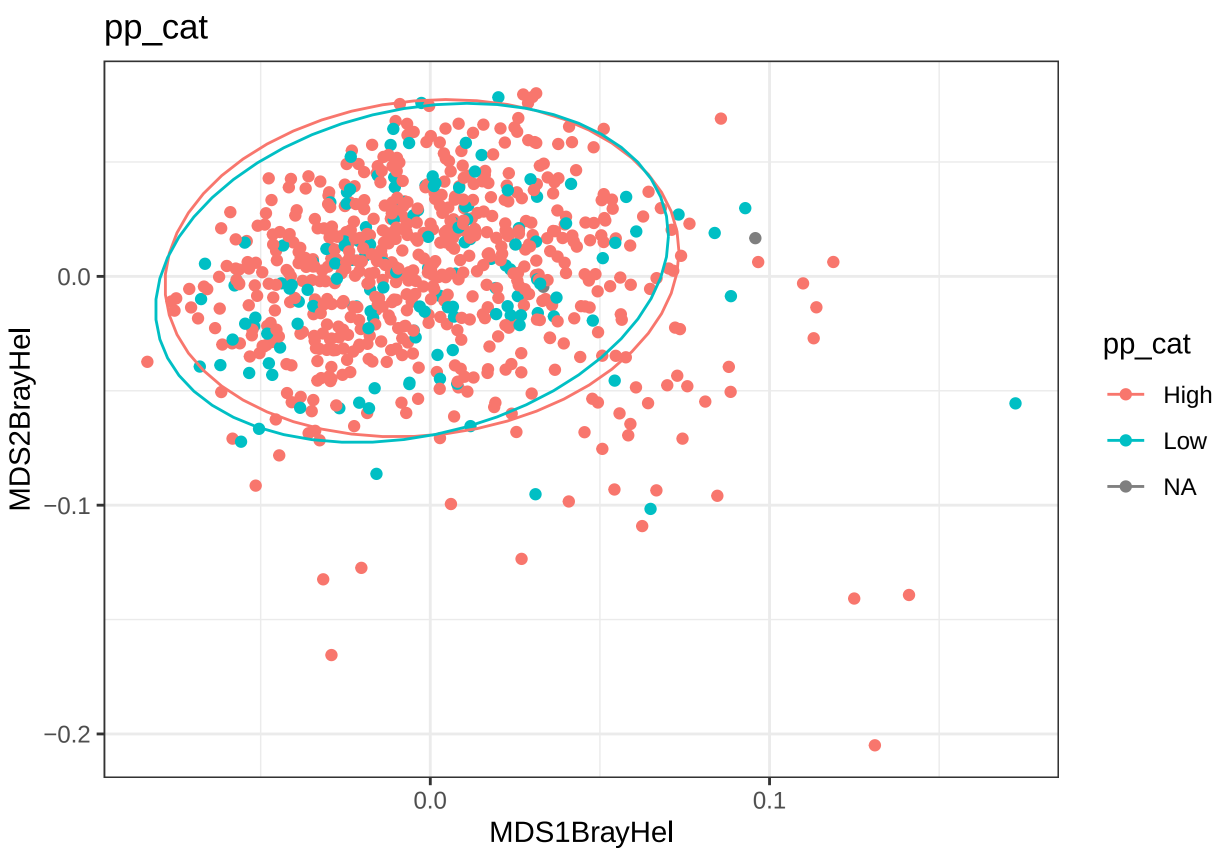


```
## Permutation test for adonis under reduced model
## Terms added sequentially (first to last)
## Permutation: free
## Number of permutations: 999
##
## adonis2(formula = formula(paste("distmatrix ~ ", i)), data = Phe3, permutations = 999, by = "terms")
##           Df SumOfSqs      R2     F Pr(>F)
## pp_cat     1   0.0243 0.00091 0.675  0.773
## Residual 742  26.7647 0.99909
## Total    743  26.7891 1.00000
```

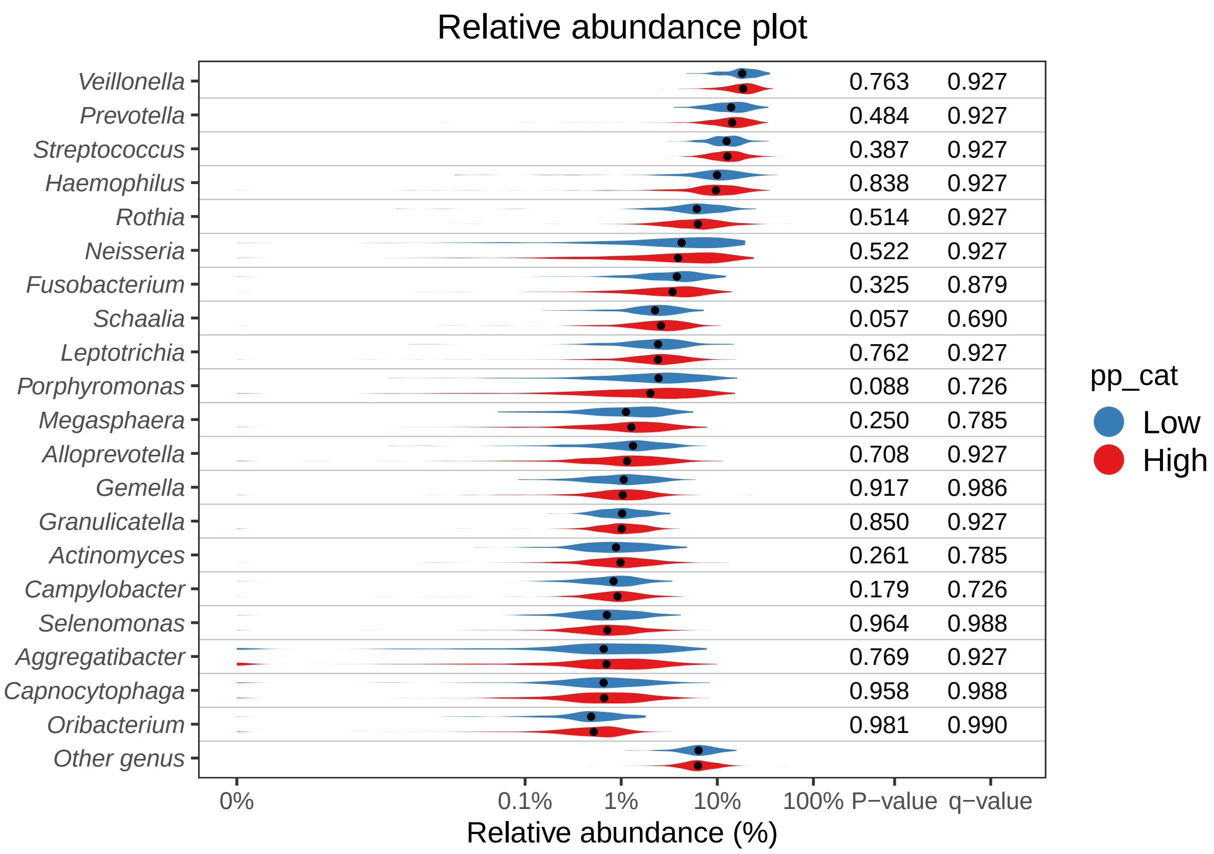
Relative abundance plot
Veillonella
Prevotella
Streptococcus
Haemophilus
Rothia
Neisseria
Fusobacterium
Schaalia
Leptotrichia
Porphyromonas
Megasphaera
Alloprevotella
Gemella
Granulicatella
Actinomyces
Campylobacter
Selenomonas
Aggregatibacter
Capnocytophaga
Oribacterium
Other genus
0.763 0.927
0.484 0.927
0.387 0.927
0.838 0.927
0.514 0.927
0.522 0.927
0.325 0.879
0.057 0.690
0.762 0.927
0.088 0.726
0.250 0.785
0.708 0.927
0.917 0.986
0.850 0.927
0.261 0.785
0.179 0.726
0.964 0.988
0.769 0.927
0.958 0.988
0.981 0.990
0%
0.1%
1%
10%
100%
P−value
q−value
Relative abundance (%)
pp_cat
Low
High

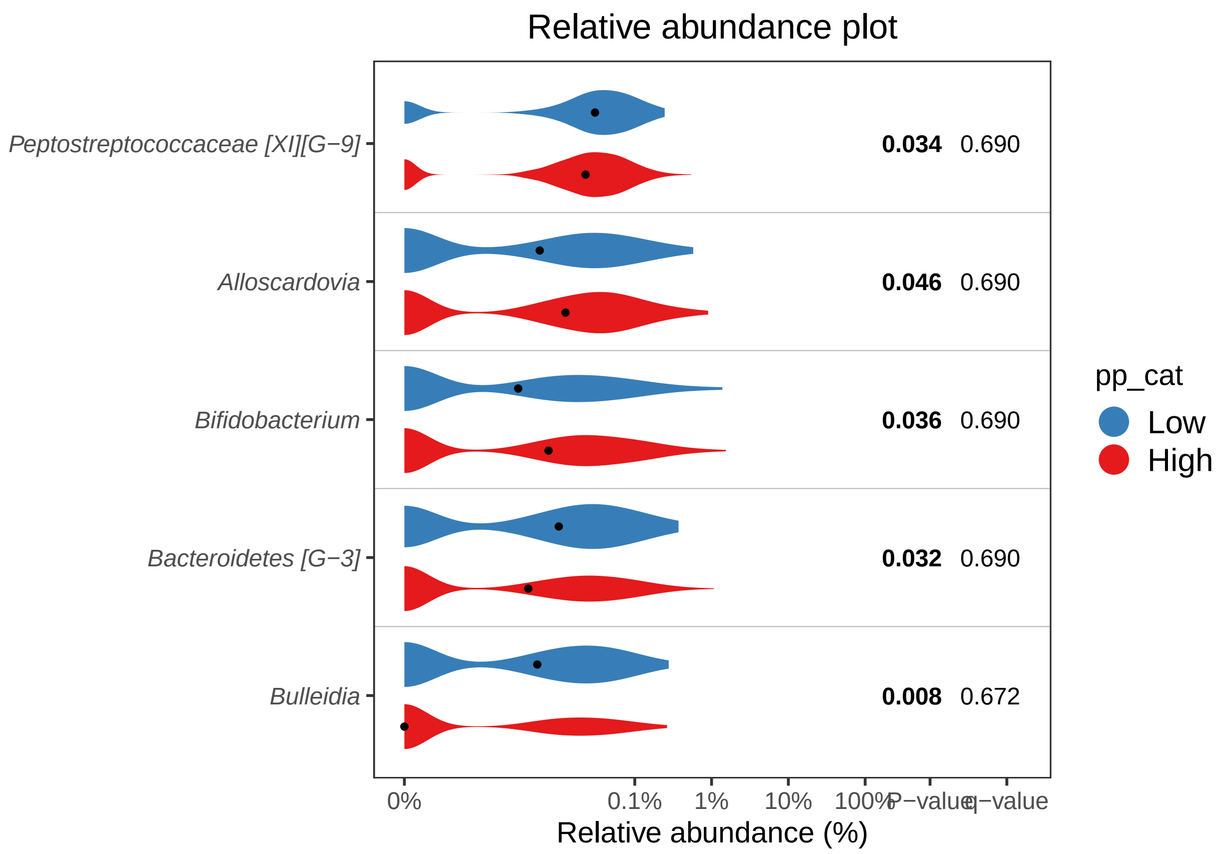
Relative abundance plot
Peptostreptococcaceae [XI][G−9]
Alloscardovia
Bifidobacterium
Bacteroidetes [G−3]
Bulleidia
0.034 0.690
0.046 0.690
0.036 0.690
0.032 0.690
0.008 0.672
0%
0.1%
1%
10%
100%
P−value
q−value
Relative abundance (%)
pp_cat
Low
High

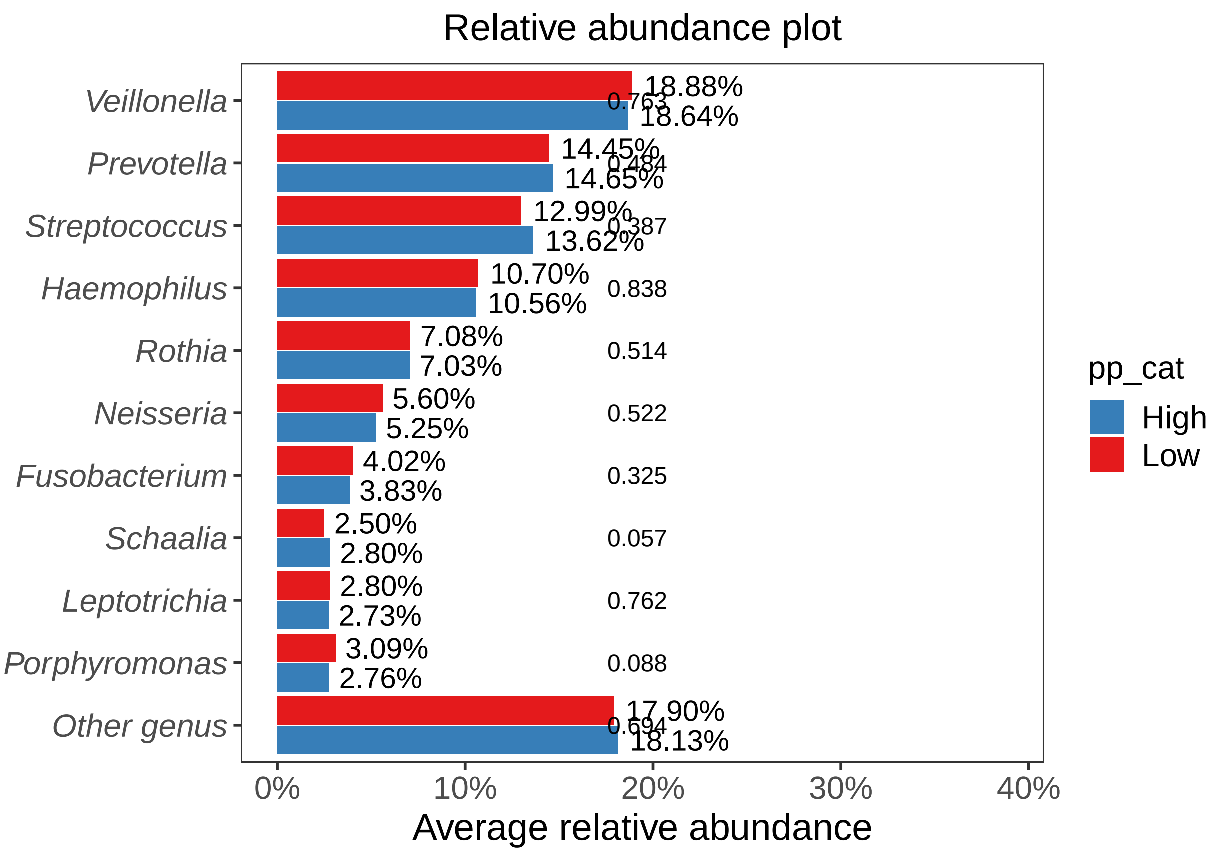
Relative abundance plot
Veillonella
Prevotella
Streptococcus
Haemophilus
Rothia
Neisseria
Fusobacterium
Schaalia
Leptotrichia
Porphyromonas
Other genus
18.88%
18.64%
0.763
14.45%
14.65%
0.484
12.99%
13.62%
0.387
10.70%
10.56%
0.838
7.08%
7.03%
0.514
5.60%
5.25%
0.522
4.02%
3.83%
0.325
2.50%
2.80%
0.057
2.80%
2.73%
0.762
3.09%
2.76%
0.088
17.90%
18.13%
0.694
0%
10%
20%
30%
40%
Average relative abundance
pp_cat
High
Low

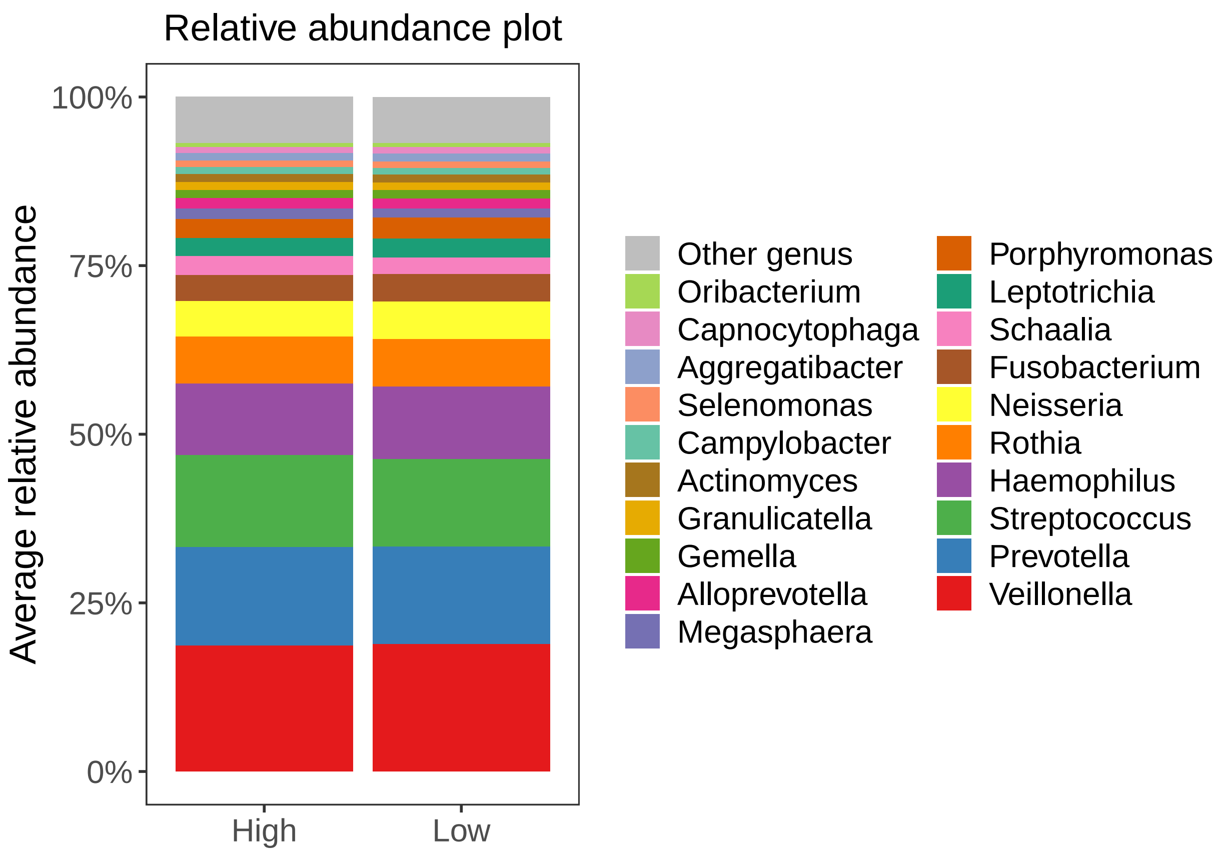
Relative abundance plot
Average relative abundance
100%
75%
50%
25%
0%
High
Low
Other genus
Oribacterium
Capnocytophaga
Aggregatibacter
Selenomonas
Campylobacter
Actinomyces
Granulicatella
Gemella
Alloprevotella
Megasphaera
Porphyromonas
Leptotrichia
Schaalia
Fusobacterium
Neisseria
Rothia
Haemophilus
Streptococcus
Prevotella
Veillonella

```
## [1] "trig_cat"
```

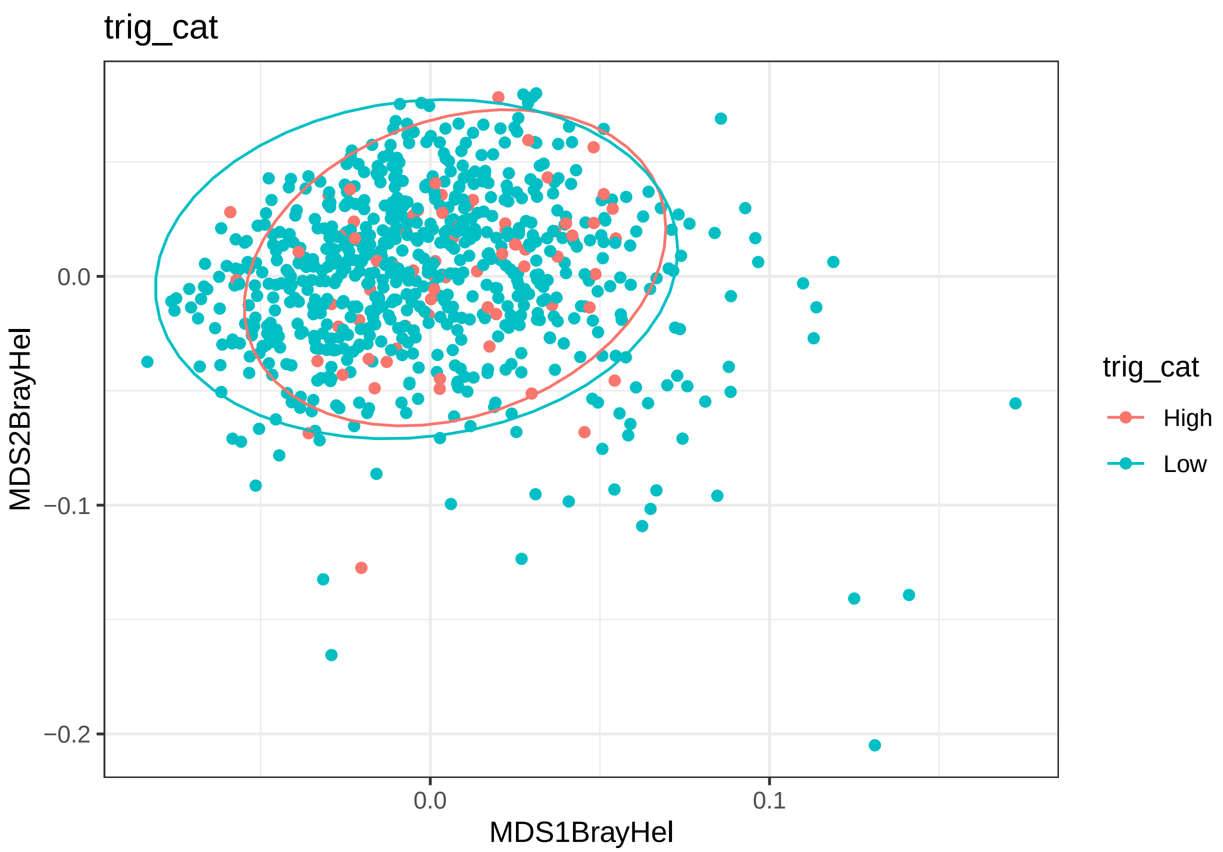


```
## Permutation test for adonis under reduced model
## Terms added sequentially (first to last)
## Permutation: free
## Number of permutations: 999
##
## adonis2(formula = formula(paste("distmatrix ~ ", i)), data = Phe3, permutations = 999, by = "terms")
##           Df SumOfSqs      R2      F Pr(>F)
## trig_cat   1   0.0622 0.00231 1.7245  0.081 .
## Residual 744  26.8301 0.99769
## Total    745  26.8923 1.00000
## ---
## Signif. codes:  0 '***' 0.001 '**' 0.01 '*' 0.05 '.' 0.1 ' ' 1
```

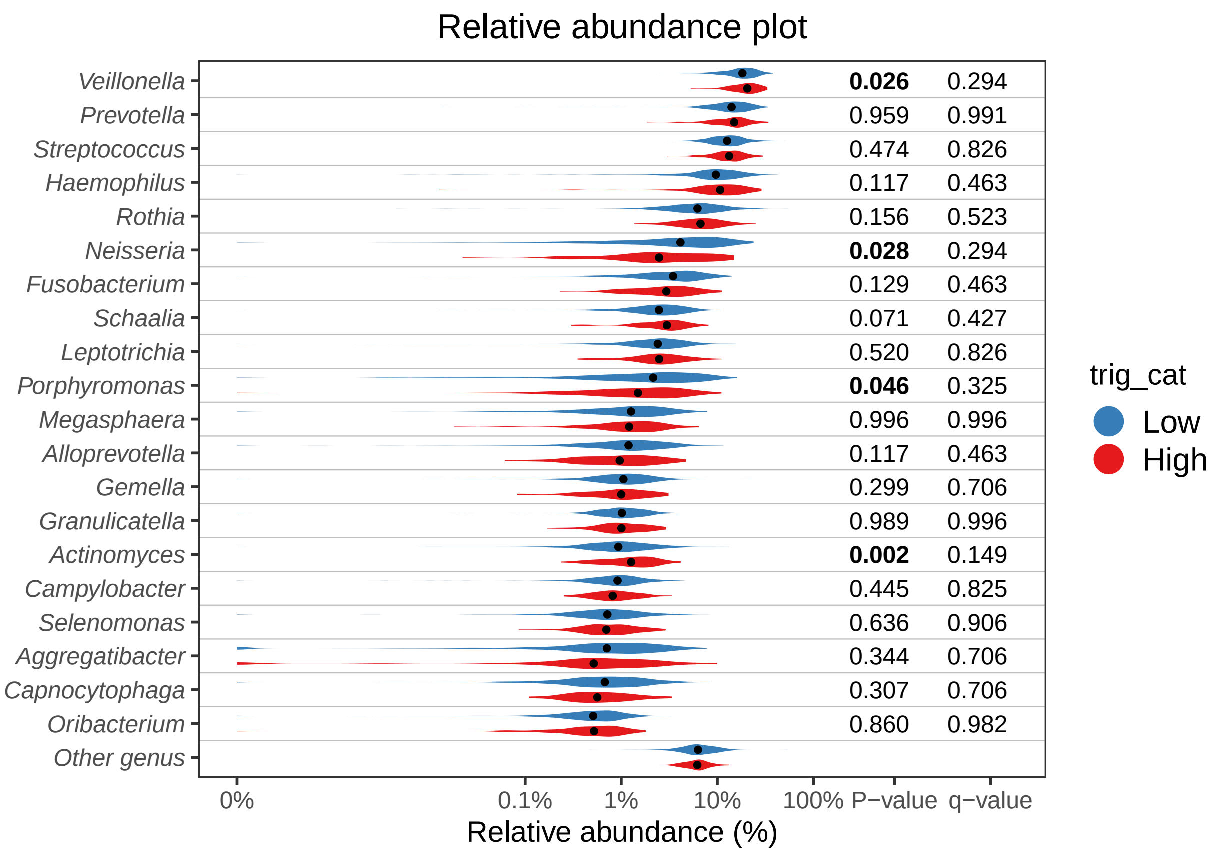
Relative abundance plot
Veillonella
Prevotella
Streptococcus
Haemophilus
Rothia
Neisseria
Fusobacterium
Schaalia
Leptotrichia
Porphyromonas
Megasphaera
Alloprevotella
Gemella
Granulicatella
Actinomyces
Campylobacter
Selenomonas
Aggregatibacter
Capnocytophaga
Oribacterium
Other genus
0.026 0.294
0.959 0.991
0.474 0.826
0.117 0.463
0.156 0.523
0.028 0.294
0.129 0.463
0.071 0.427
0.520 0.826
0.046 0.325
0.996 0.996
0.117 0.463
0.299 0.706
0.989 0.996
0.002 0.149
0.445 0.825
0.636 0.906
0.344 0.706
0.307 0.706
0.860 0.982
0%
0.1%
1%
10%
100%
P−value
q−value
Relative abundance (%)
trig_cat
Low
High

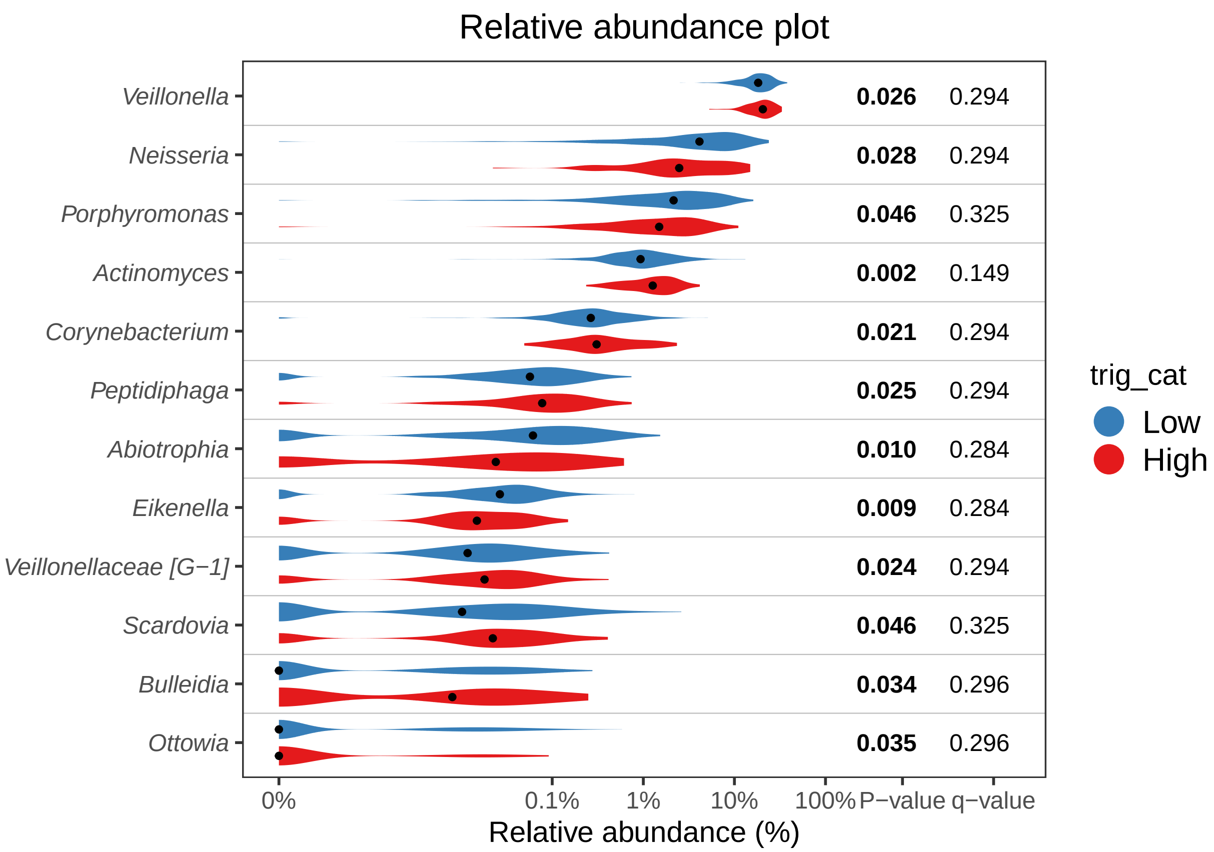

Relative abundance plot
Veillonella
Neisseria
Porphyromonas
Actinomyces
Corynebacterium
Peptidiphaga
Abiotrophia
Eikenella
Veillonellaceae [G−1]
Scardovia
Bulleidia
Ottowia
0.026 0.294
0.028 0.294
0.046 0.325
0.002 0.149
0.021 0.294
0.025 0.294
0.010 0.284
0.009 0.284
0.024 0.294
0.046 0.325
0.034 0.296
0.035 0.296
0%
0.1%
1%
10%
100%
P−value
q−value
Relative abundance (%)
trig_cat
Low
High

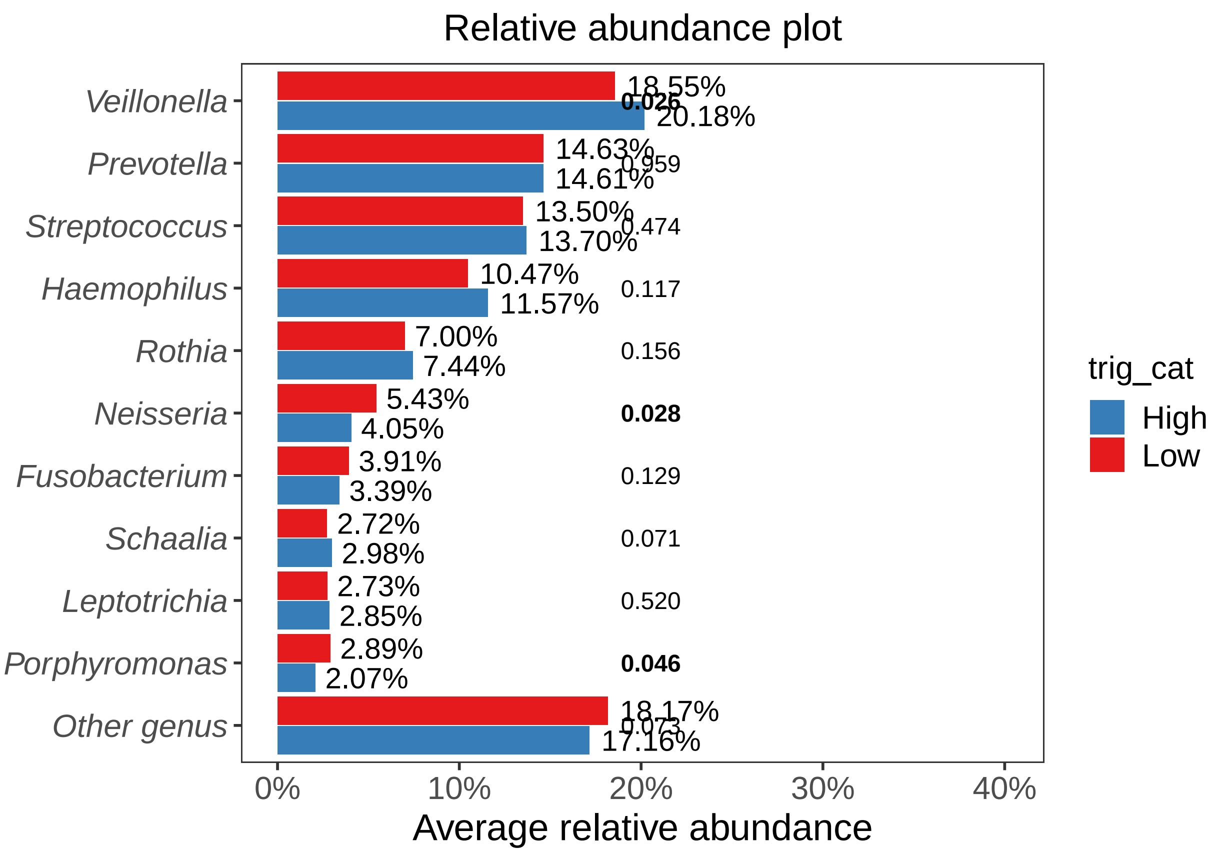

Relative abundance plot
Veillonella
18.55%
0.026
20.18%
Prevotella
14.63%
0.959
14.61%
Streptococcus
13.50%
0.474
13.70%
Haemophilus
10.47%
0.117
11.57%
Rothia
7.00%
0.156
7.44%
Neisseria
5.43%
0.028
4.05%
Fusobacterium
3.91%
0.129
3.39%
Schaalia
2.72%
0.071
2.98%
Leptotrichia
2.73%
0.520
2.85%
Porphyromonas
2.89%
0.046
2.07%
Other genus
18.17%
0.073
17.16%
0%
10%
20%
30%
40%
Average relative abundance
trig_cat
High
Low

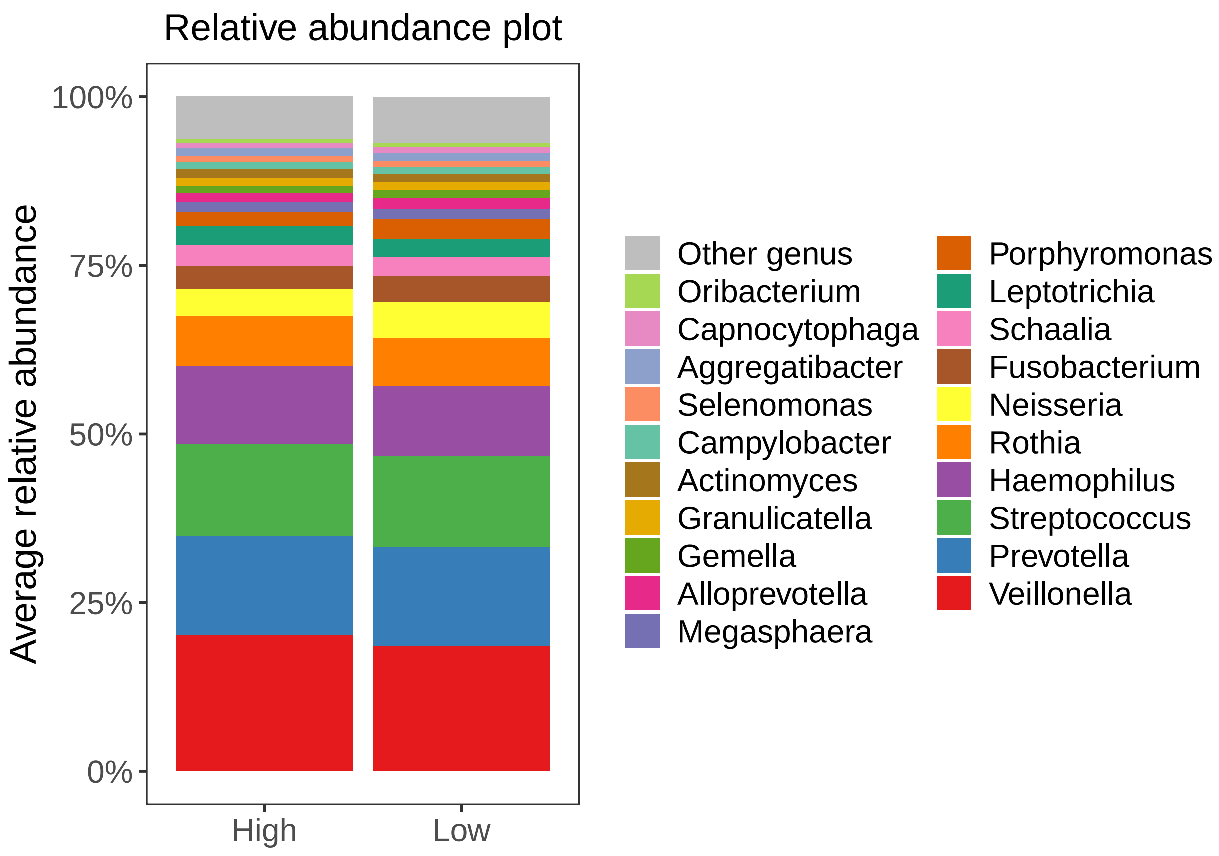
Relative abundance plot
Average relative abundance
100%
75%
50%
25%
0%
High
Low
Other genus
Oribacterium
Capnocytophaga
Aggregatibacter
Selenomonas
Campylobacter
Actinomyces
Granulicatella
Gemella
Alloprevotella
Megasphaera
Porphyromonas
Leptotrichia
Schaalia
Fusobacterium
Neisseria
Rothia
Haemophilus
Streptococcus
Prevotella
Veillonella

```
## [1] "hdlc_cat"
```

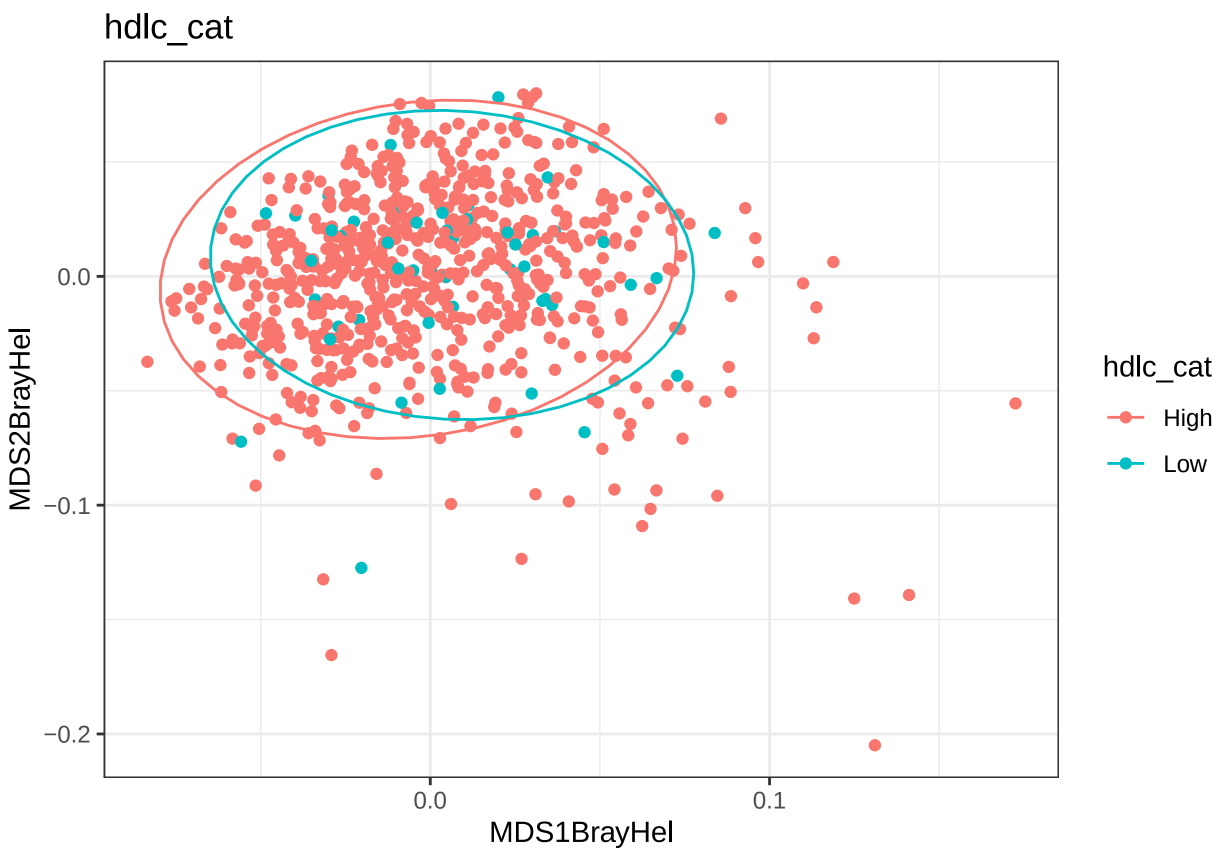


```
## Permutation test for adonis under reduced model
## Terms added sequentially (first to last)
## Permutation: free
## Number of permutations: 999
##
## adonis2(formula = formula(paste("distmatrix ~ ", i)), data = Phe3, permutations = 999, by = "terms")
##           Df SumOfSqs      R2      F Pr(>F)
## hdlc_cat   1   0.0546 0.00203 1.5135  0.122
## Residual 744  26.8377 0.99797
## Total    745  26.8923 1.00000
```

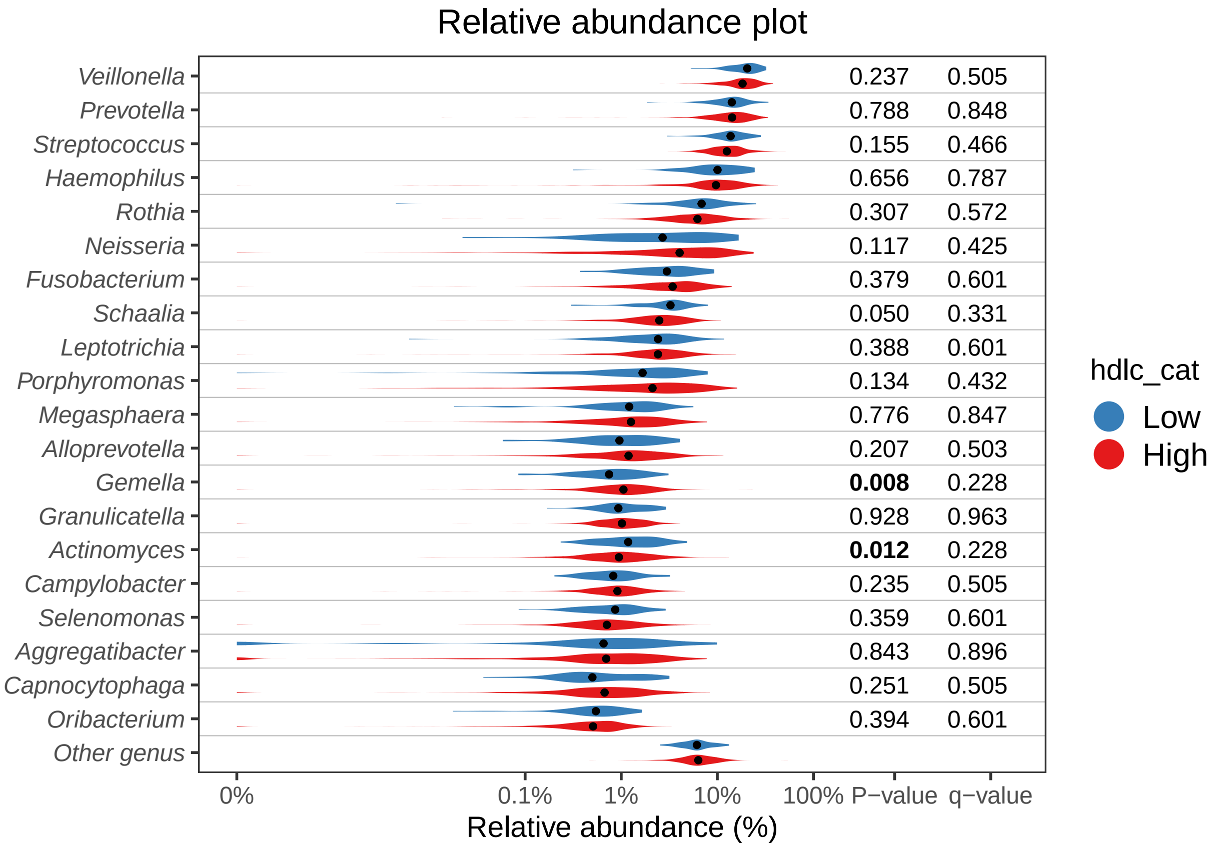
Relative abundance plot
Veillonella 0.237 0.505
Prevotella 0.788 0.848
Streptococcus 0.155 0.466
Haemophilus 0.656 0.787
Rothia 0.307 0.572
Neisseria 0.117 0.425
Fusobacterium 0.379 0.601
Schaalia 0.050 0.331
Leptotrichia 0.388 0.601
Porphyromonas 0.134 0.432
Megasphaera 0.776 0.847
Alloprevotella 0.207 0.503
Gemella 0.008 0.228
Granulicatella 0.928 0.963
Actinomyces 0.012 0.228
Campylobacter 0.235 0.505
Selenomonas 0.359 0.601
Aggregatibacter 0.843 0.896
Capnocytophaga 0.251 0.505
Oribacterium 0.394 0.601
Other genus
0%
0.1%
1%
10%
100%
P−value
q−value
Relative abundance (%)
hdlc_cat
Low
High

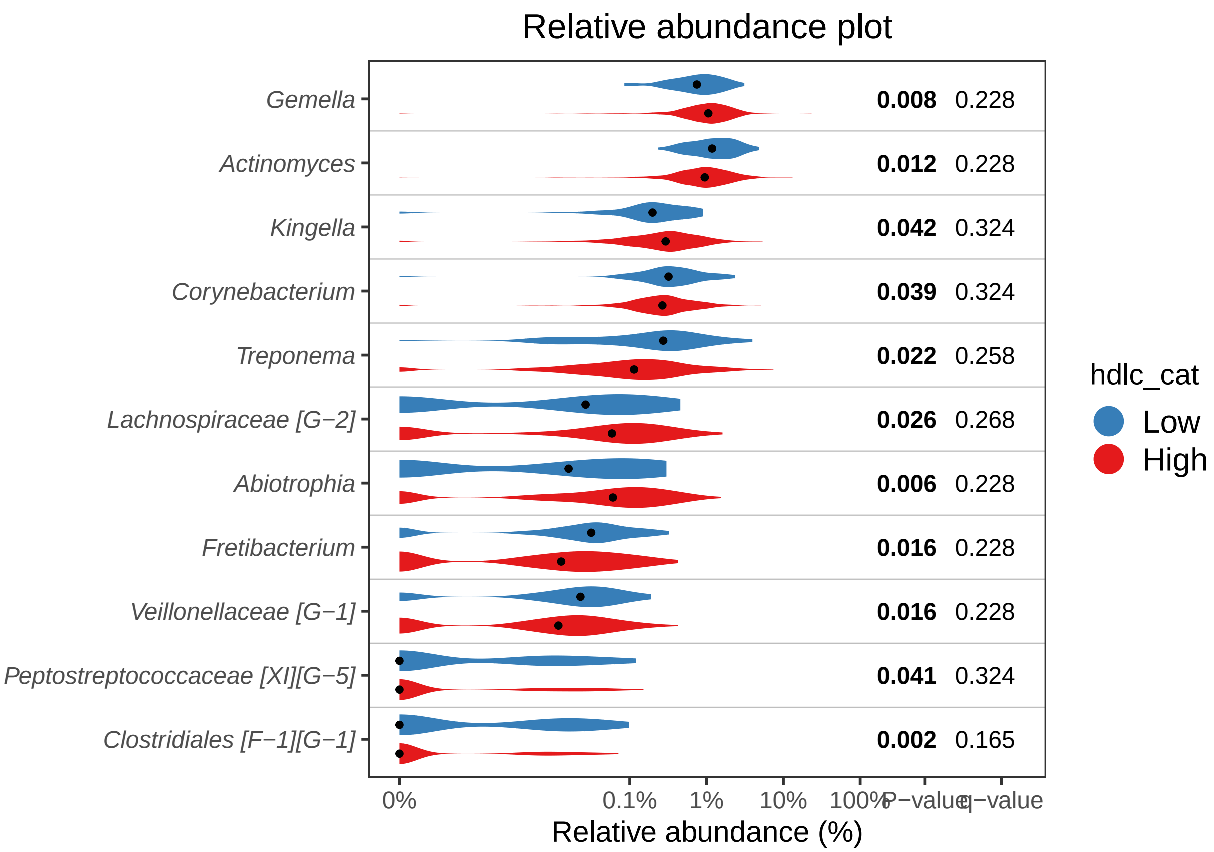

Relative abundance plot
Gemella
Actinomyces
Kingella
Corynebacterium
Treponema
Lachnospiraceae [G−2]
Abiotrophia
Fretibacterium
Veillonellaceae [G−1]
Peptostreptococcaceae [XI][G−5]
Clostridiales [F−1][G−1]
0.008 0.228
0.012 0.228
0.042 0.324
0.039 0.324
0.022 0.258
0.026 0.268
0.006 0.228
0.016 0.228
0.016 0.228
0.041 0.324
0.002 0.165
0%
0.1%
1%
10%
100%
P−value
q−value
Relative abundance (%)
hdlc_cat
Low
High

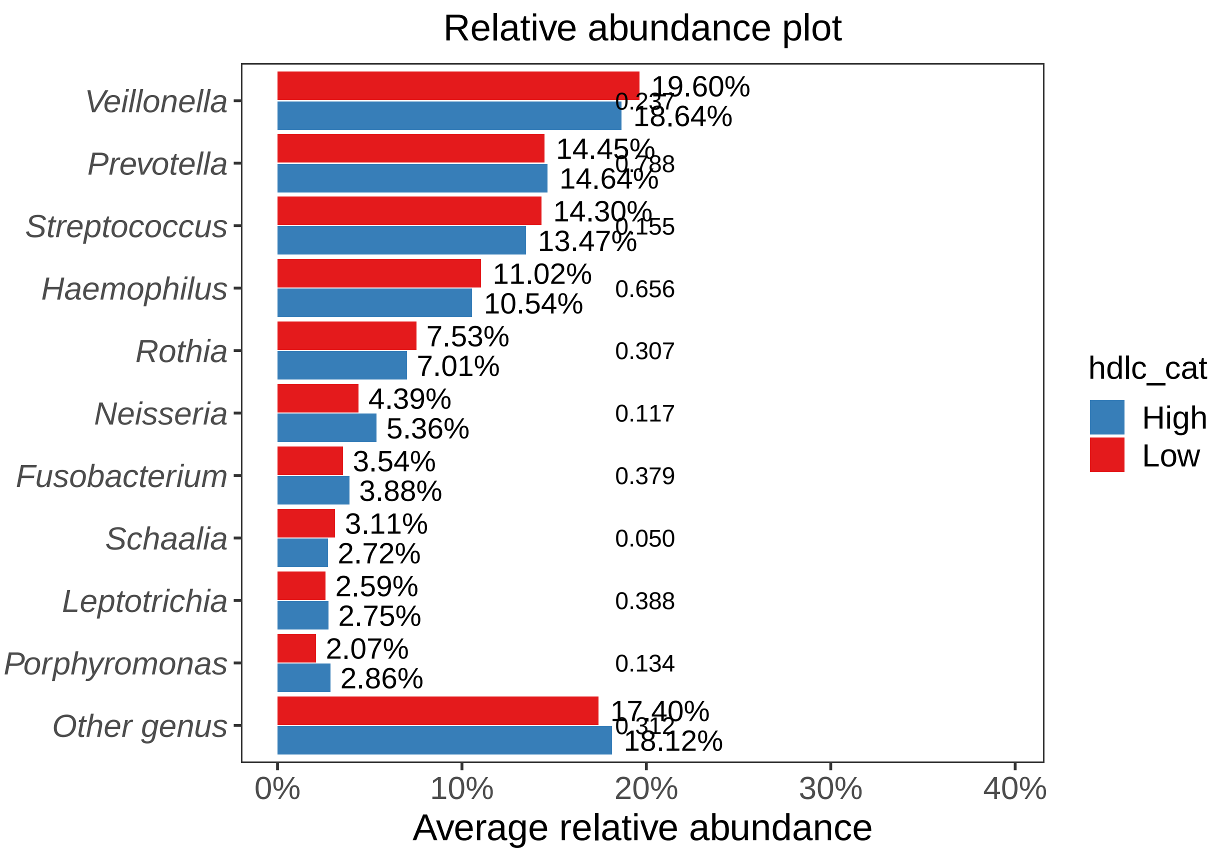

Relative abundance plot
Veillonella
Prevotella
Streptococcus
Haemophilus
Rothia
Neisseria
Fusobacterium
Schaalia
Leptotrichia
Porphyromonas
Other genus
19.60%
18.64%
0.237
14.45%
14.64%
0.788
14.30%
13.47%
0.155
11.02%
10.54%
0.656
7.53%
7.01%
0.307
4.39%
5.36%
0.117
3.54%
3.88%
0.379
3.11%
2.72%
0.050
2.59%
2.75%
0.388
2.07%
2.86%
0.134
17.40%
18.12%
0.312
0%
10%
20%
30%
40%
Average relative abundance
hdlc_cat
High
Low

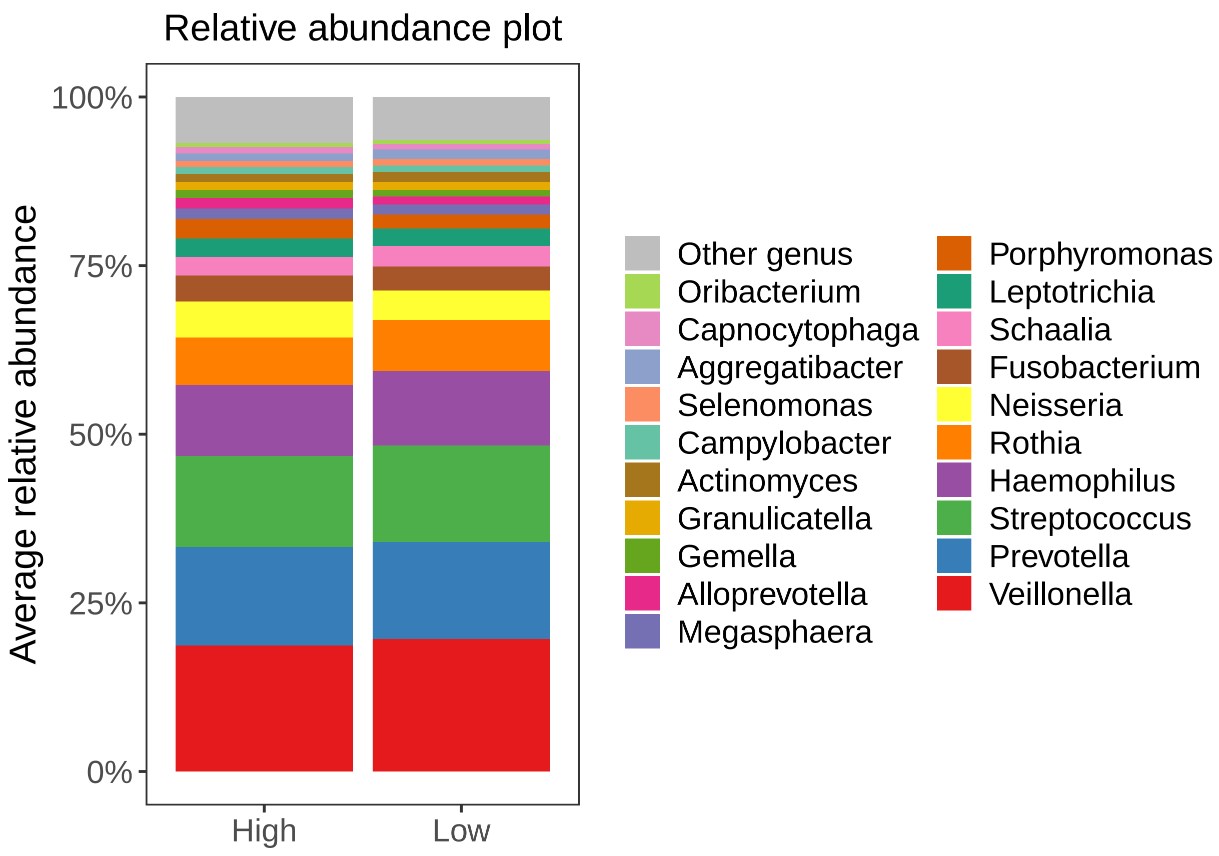
Relative abundance plot
Average relative abundance
100%
75%
50%
25%
0%
High
Low
Other genus
Oribacterium
Capnocytophaga
Aggregatibacter
Selenomonas
Campylobacter
Actinomyces
Granulicatella
Gemella
Alloprevotella
Megasphaera
Porphyromonas
Leptotrichia
Schaalia
Fusobacterium
Neisseria
Rothia
Haemophilus
Streptococcus
Prevotella
Veillonella

```
## [1] "ldlc_cat"
```

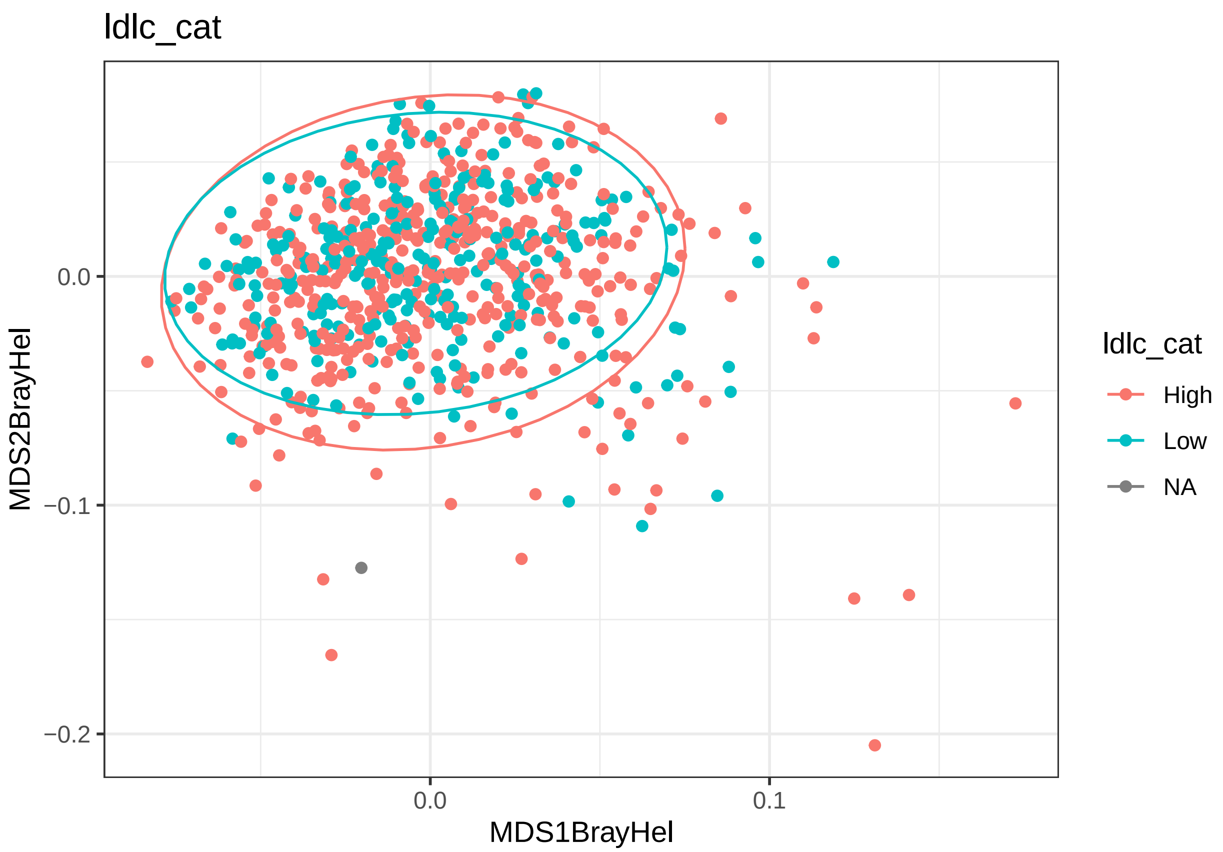


```
## Permutation test for adonis under reduced model
## Terms added sequentially (first to last)
## Permutation: free
## Number of permutations: 999
##
## adonis2(formula = formula(paste("distmatrix ~ ", i)), data = Phe3, permutations = 999, by = "terms")
##            Df SumOfSqs      R2      F Pr(>F)
## ldlc_cat    1   0.0523 0.00195 1.4521  0.126
## Residual 742  26.7247 0.99805
## Total    743  26.7770 1.00000
```

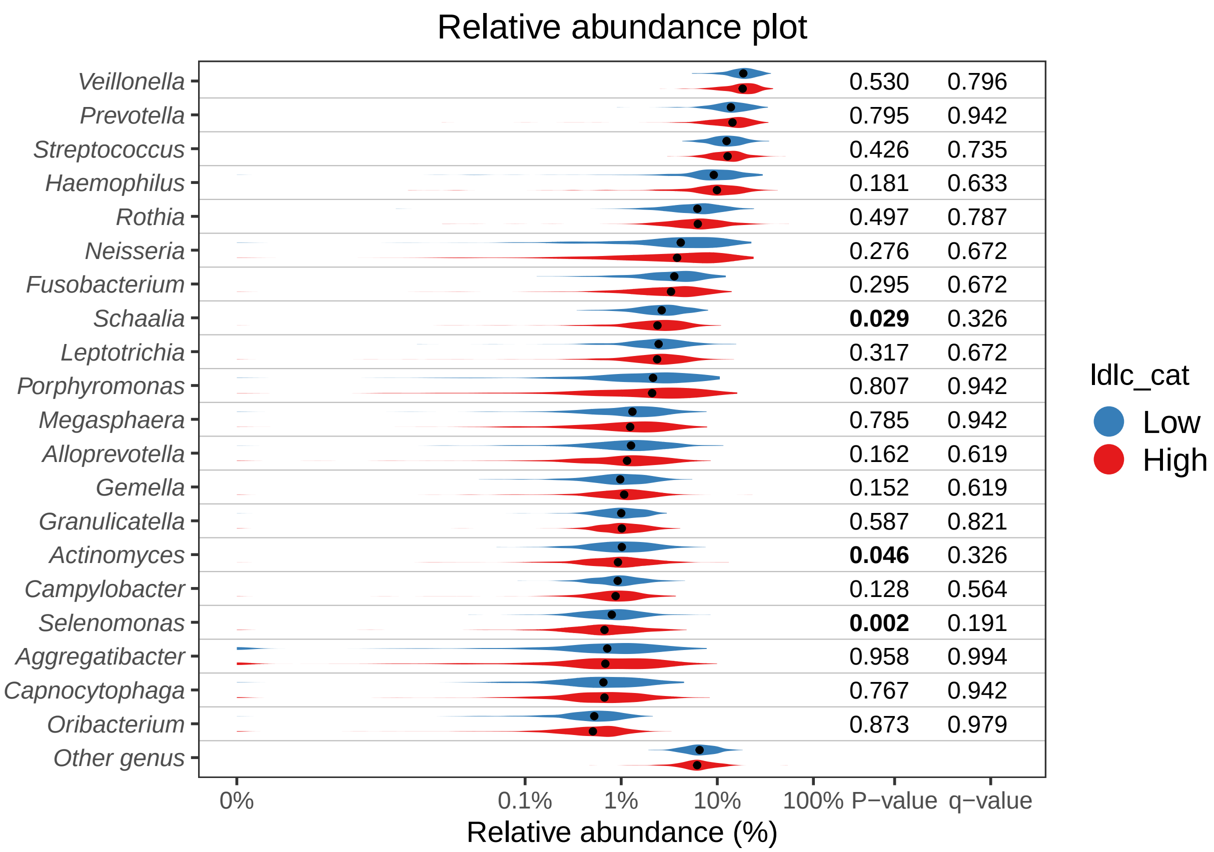

Relative abundance plot
Veillonella
Prevotella
Streptococcus
Haemophilus
Rothia
Neisseria
Fusobacterium
Schaalia
Leptotrichia
Porphyromonas
Megasphaera
Alloprevotella
Gemella
Granulicatella
Actinomyces
Campylobacter
Selenomonas
Aggregatibacter
Capnocytophaga
Oribacterium
Other genus
0.530 0.796
0.795 0.942
0.426 0.735
0.181 0.633
0.497 0.787
0.276 0.672
0.295 0.672
0.029 0.326
0.317 0.672
0.807 0.942
0.785 0.942
0.162 0.619
0.152 0.619
0.587 0.821
0.046 0.326
0.128 0.564
0.002 0.191
0.958 0.994
0.767 0.942
0.873 0.979
0% 0.1% 1% 10% 100% P−value q−value
Relative abundance (%)
ldlc_cat
Low
High

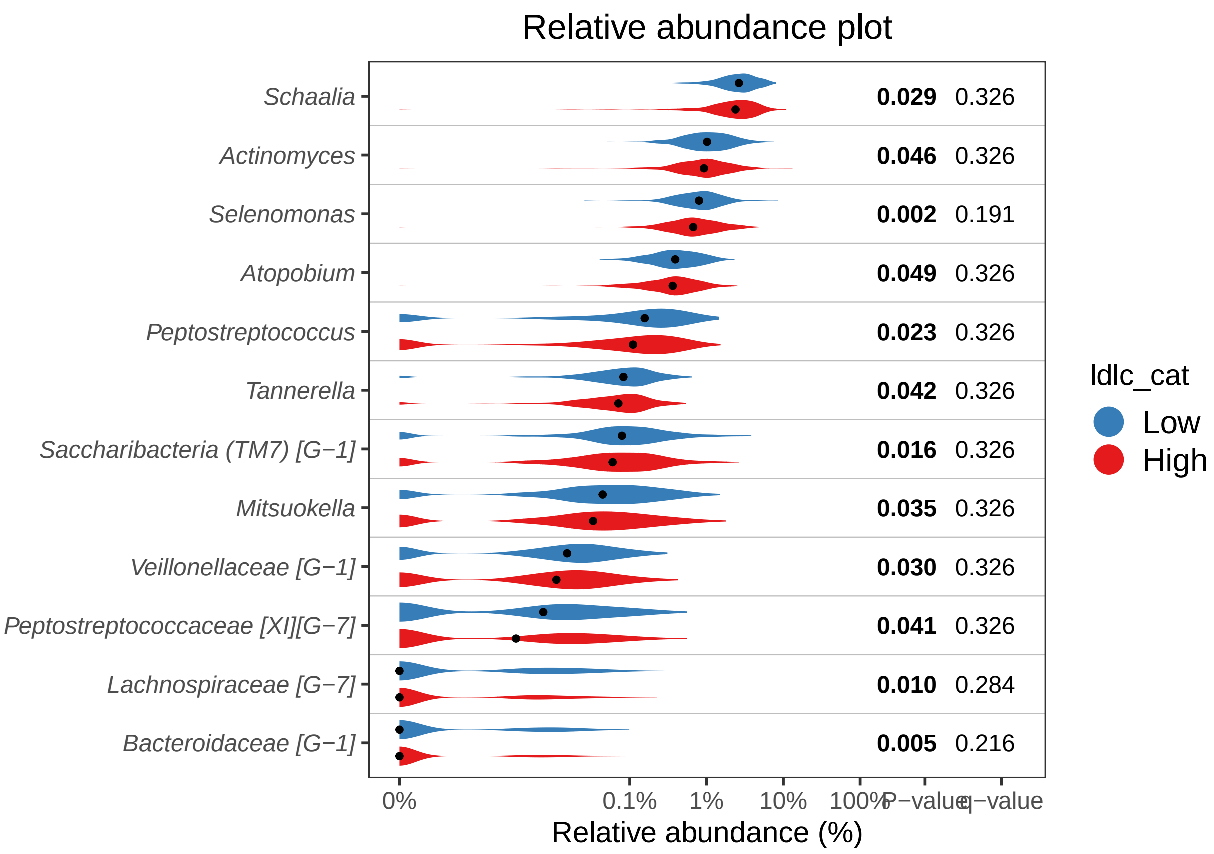
Relative abundance plot
Schaalia
Actinomyces
Selenomonas
Atopobium
Peptostreptococcus
Tannerella
Saccharibacteria (TM7) [G−1]
Mitsuokella
Veillonellaceae [G−1]
Peptostreptococcaceae [XI][G−7]
Lachnospiraceae [G−7]
Bacteroidaceae [G−1]
0.029 0.326
0.046 0.326
0.002 0.191
0.049 0.326
0.023 0.326
0.042 0.326
0.016 0.326
0.035 0.326
0.030 0.326
0.041 0.326
0.010 0.284
0.005 0.216
0%
0.1%
1%
10%
100%
P−value
q−value
Relative abundance (%)
ldlc_cat
Low
High

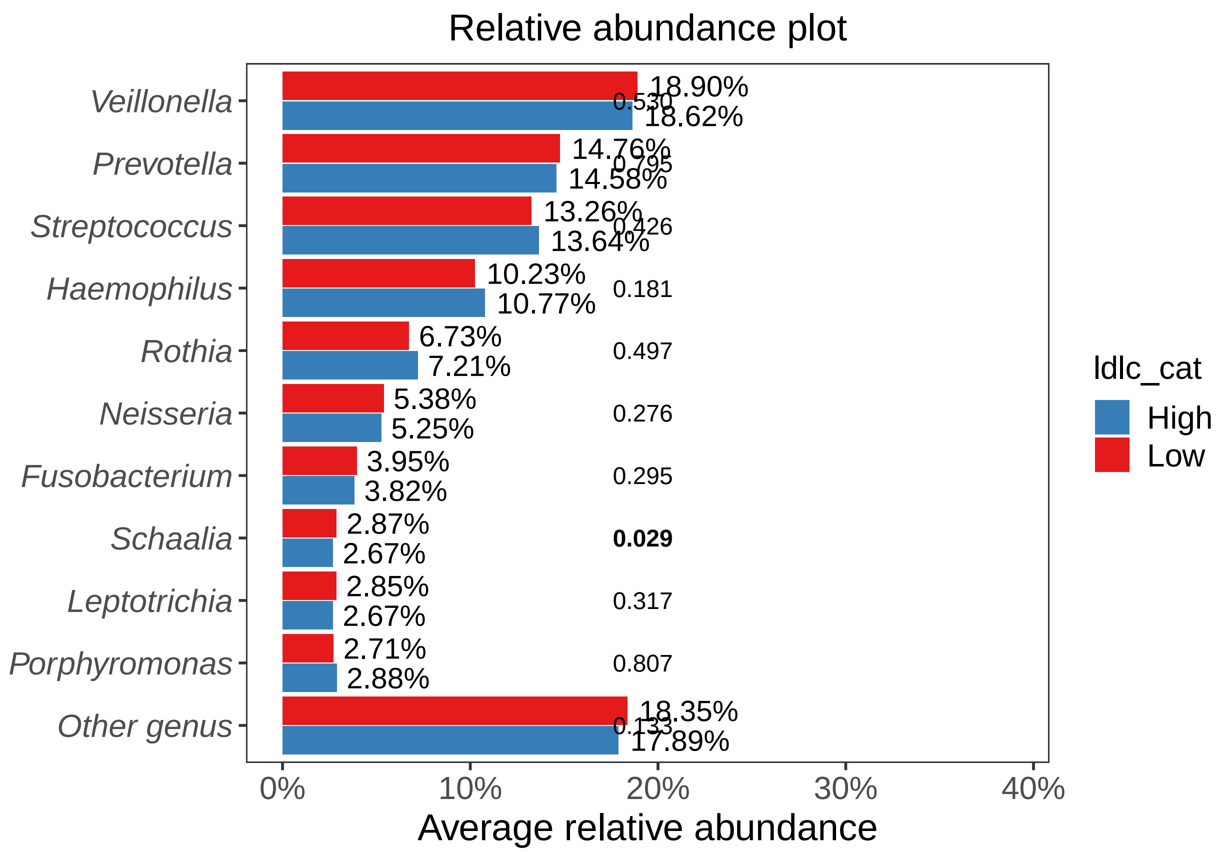

Relative abundance plot
Veillonella
Prevotella
Streptococcus
Haemophilus
Rothia
Neisseria
Fusobacterium
Schaalia
Leptotrichia
Porphyromonas
Other genus
18.90%
18.62%
0.530
14.76%
14.58%
0.795
13.26%
13.64%
0.426
10.23%
10.77%
0.181
6.73%
7.21%
0.497
5.38%
5.25%
0.276
3.95%
3.82%
0.295
2.87%
2.67%
0.029
2.85%
2.67%
0.317
2.71%
2.88%
0.807
18.35%
17.89%
0.133
0%
10%
20%
30%
40%
Average relative abundance
ldlc_cat
High
Low

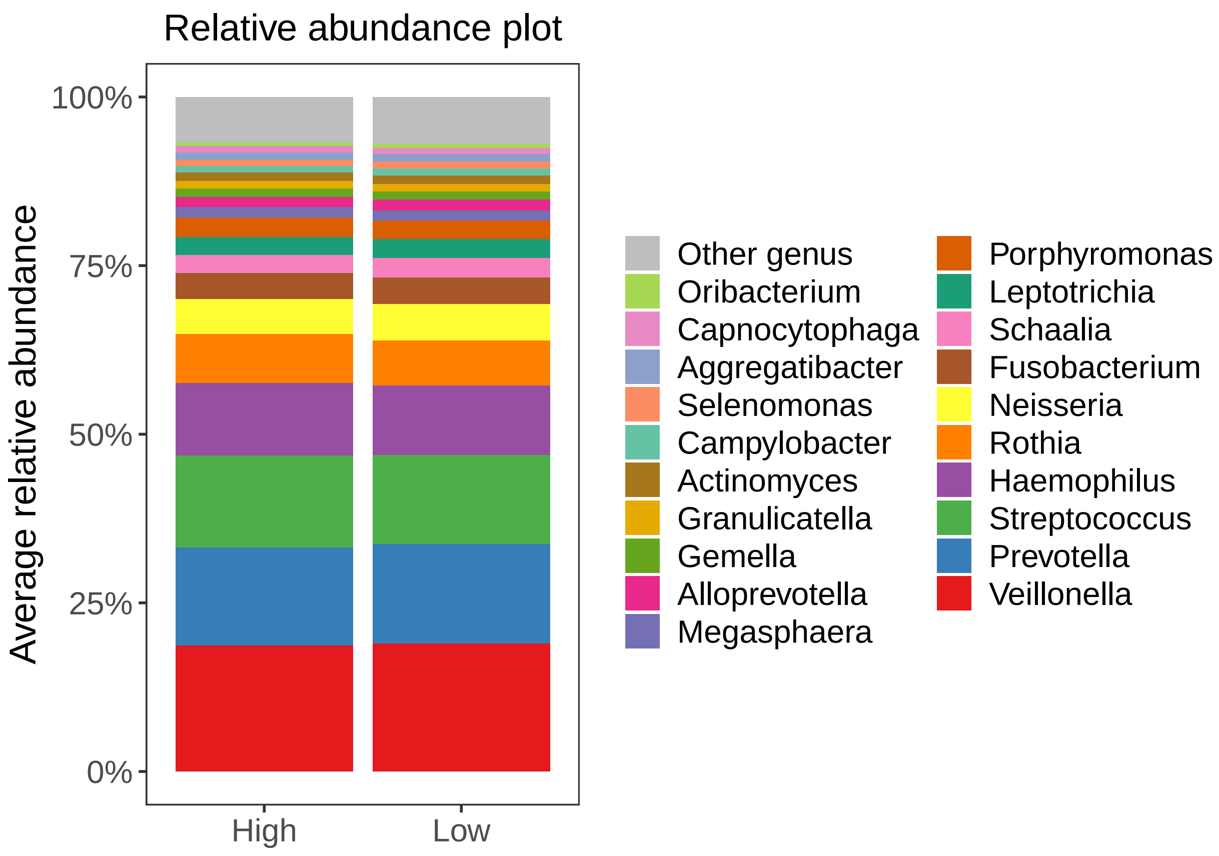
Relative abundance plot
Average relative abundance
100%
75%
50%
25%
0%
High
Low
Other genus
Oribacterium
Capnocytophaga
Aggregatibacter
Selenomonas
Campylobacter
Actinomyces
Granulicatella
Gemella
Alloprevotella
Megasphaera
Porphyromonas
Leptotrichia
Schaalia
Fusobacterium
Neisseria
Rothia
Haemophilus
Streptococcus
Prevotella
Veillonella

```
## [1] "chol_cat"
```

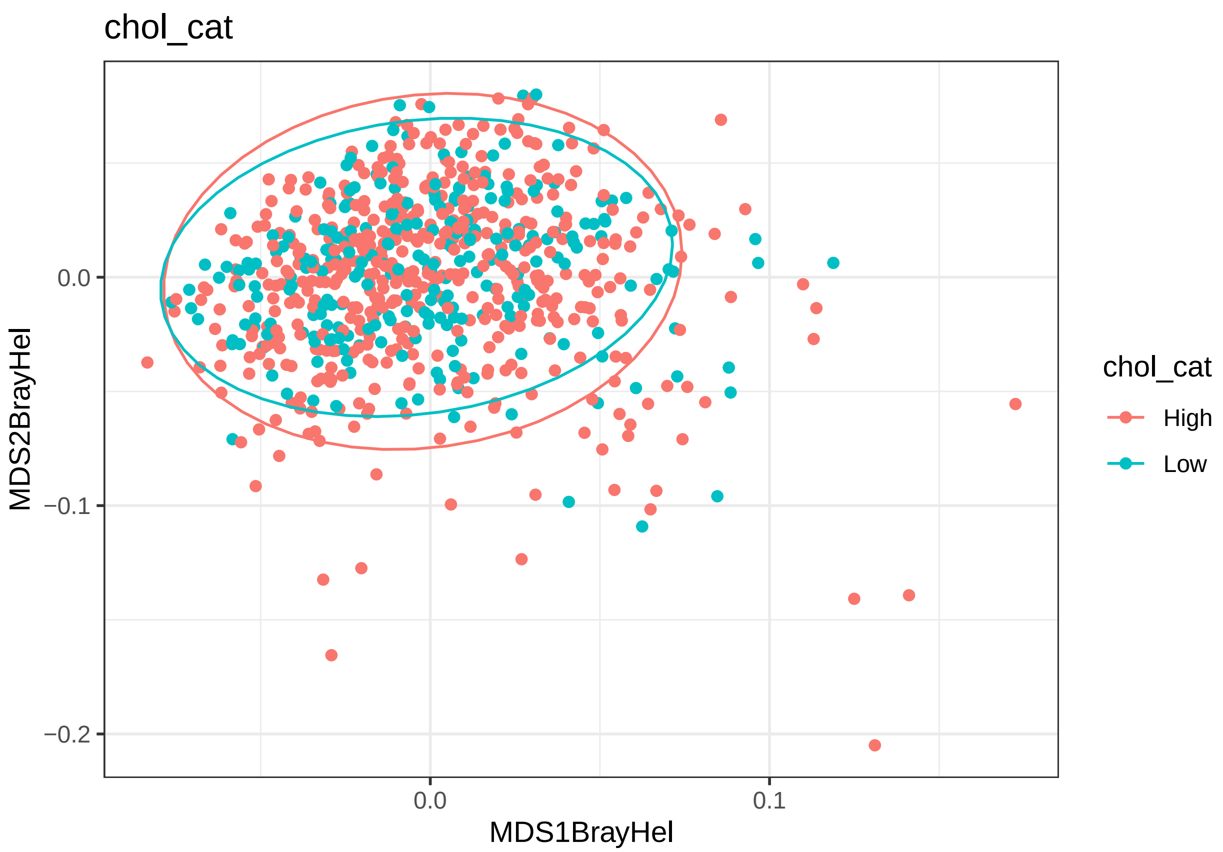


```
## Permutation test for adonis under reduced model
## Terms added sequentially (first to last)
## Permutation: free
## Number of permutations: 999
##
## adonis2(formula = formula(paste("distmatrix ~ ", i)), data = Phe3, permutations = 999, by = "terms")
##           Df SumOfSqs      R2      F Pr(>F)
## chol_cat   1   0.0373 0.00139 1.0335  0.389
## Residual 744  26.8550 0.99861
## Total    745  26.8923 1.00000
```

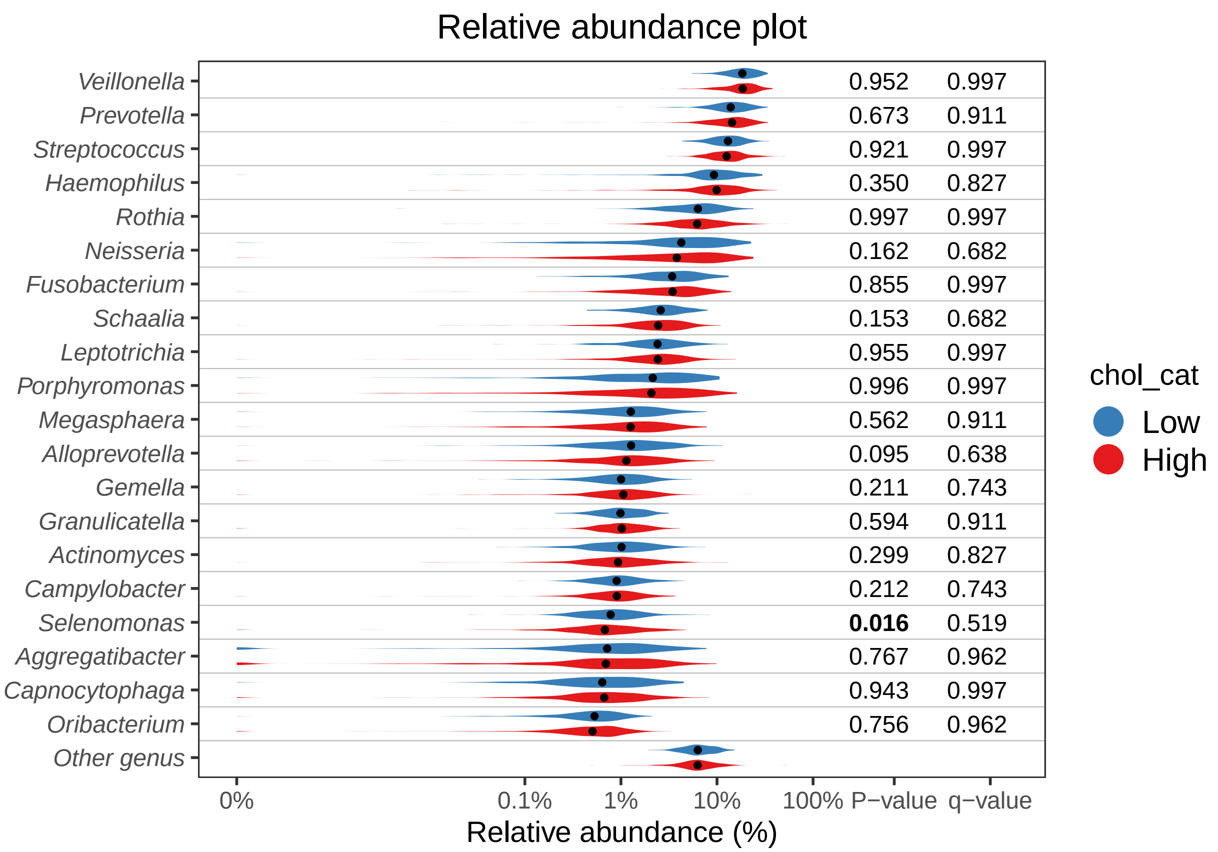
Relative abundance plot
Veillonella
Prevotella
Streptococcus
Haemophilus
Rothia
Neisseria
Fusobacterium
Schaalia
Leptotrichia
Porphyromonas
Megasphaera
Alloprevotella
Gemella
Granulicatella
Actinomyces
Campylobacter
Selenomonas
Aggregatibacter
Capnocytophaga
Oribacterium
Other genus
0.952 0.997
0.673 0.911
0.921 0.997
0.350 0.827
0.997 0.997
0.162 0.682
0.855 0.997
0.153 0.682
0.955 0.997
0.996 0.997
0.562 0.911
0.095 0.638
0.211 0.743
0.594 0.911
0.299 0.827
0.212 0.743
0.016 0.519
0.767 0.962
0.943 0.997
0.756 0.962
0% 0.1% 1% 10% 100% P−value q−value
Relative abundance (%)
chol_cat
Low
High

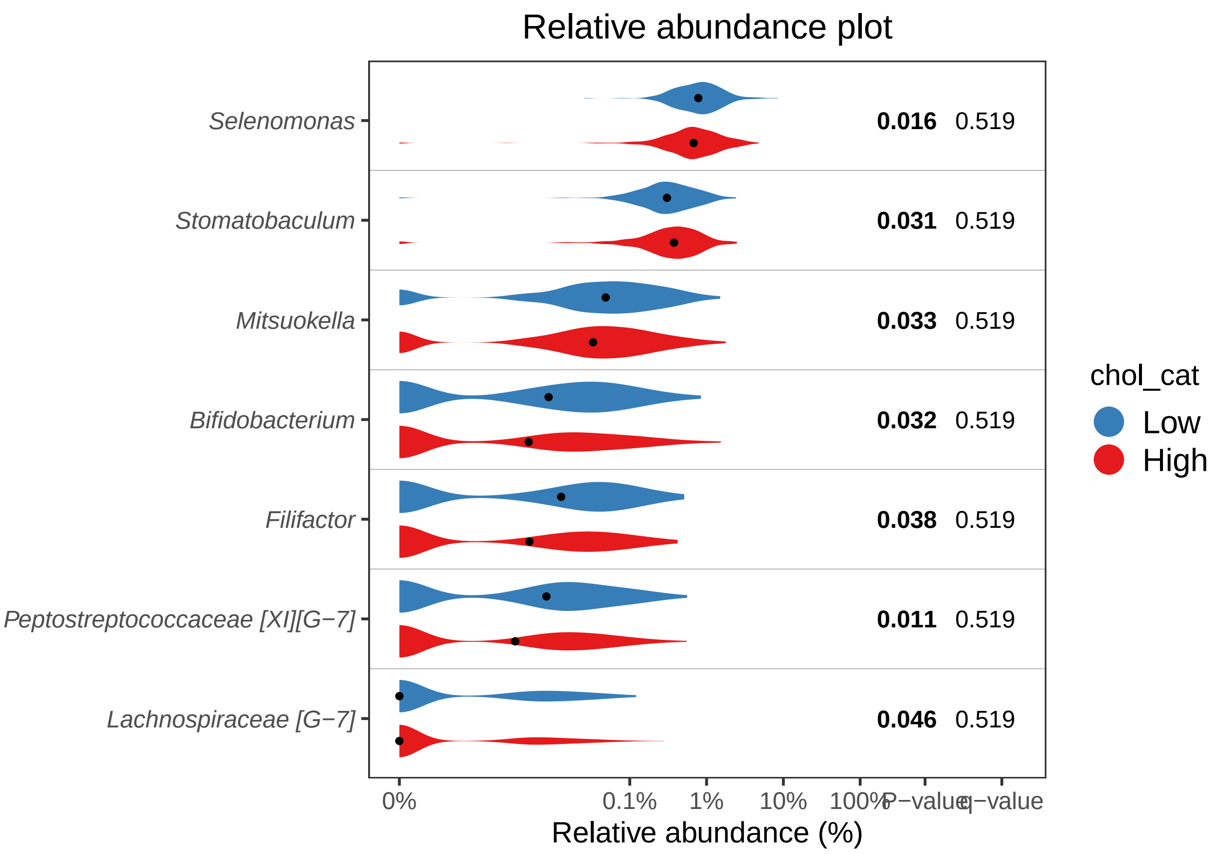
Relative abundance plot
Selenomonas
Stomatobaculum
Mitsuokella
Bifidobacterium
Filifactor
Peptostreptococcaceae [XI][G−7]
Lachnospiraceae [G−7]
0.016 0.519
0.031 0.519
0.033 0.519
0.032 0.519
0.038 0.519
0.011 0.519
0.046 0.519
0%
0.1%
1%
10%
100%
P−value
q−value
Relative abundance (%)
chol_cat
Low
High

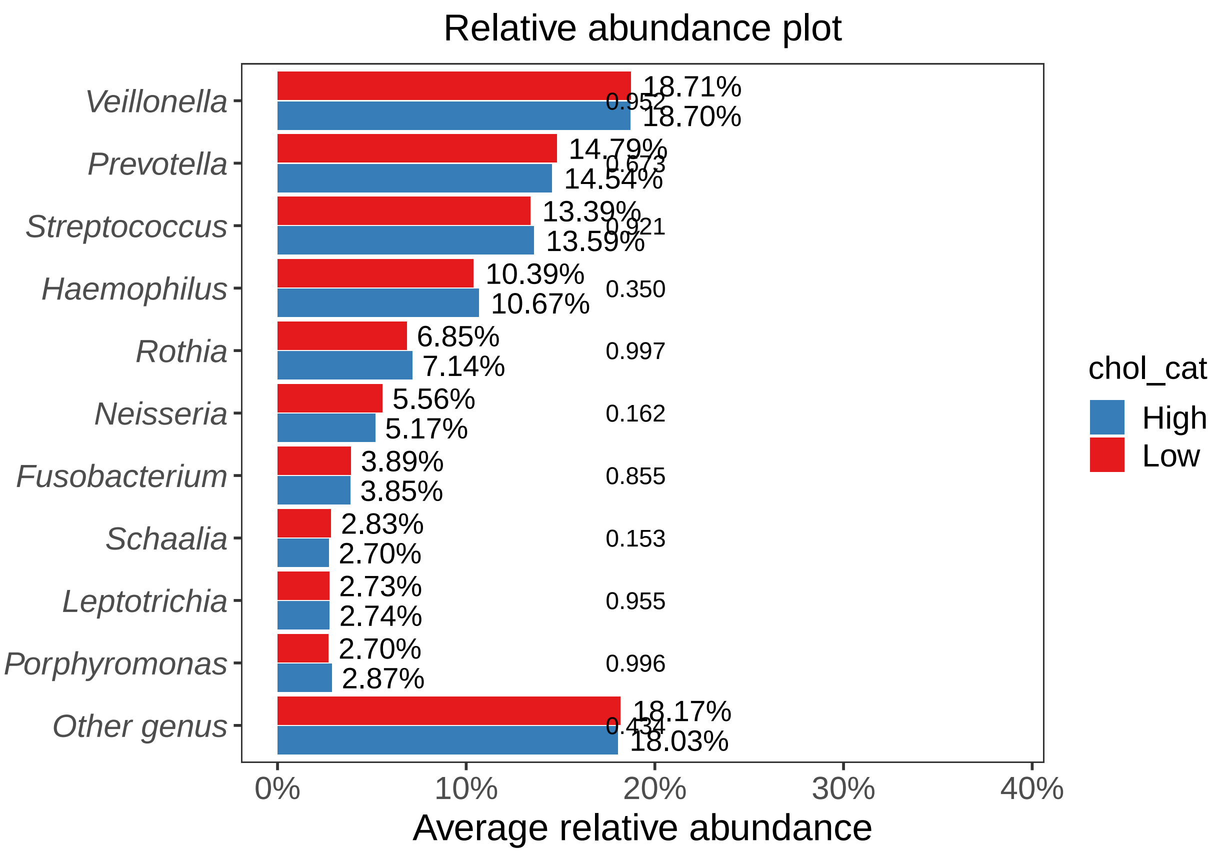
Relative abundance plot
Veillonella
Prevotella
Streptococcus
Haemophilus
Rothia
Neisseria
Fusobacterium
Schaalia
Leptotrichia
Porphyromonas
Other genus
18.71%
18.70%
0.952
14.79%
14.54%
0.673
13.39%
13.59%
0.921
10.39%
10.67%
0.350
6.85%
7.14%
0.997
5.56%
5.17%
0.162
3.89%
3.85%
0.855
2.83%
2.70%
0.153
2.73%
2.74%
0.955
2.70%
2.87%
0.996
18.17%
18.03%
0.434
0%
10%
20%
30%
40%
Average relative abundance
chol_cat
High
Low

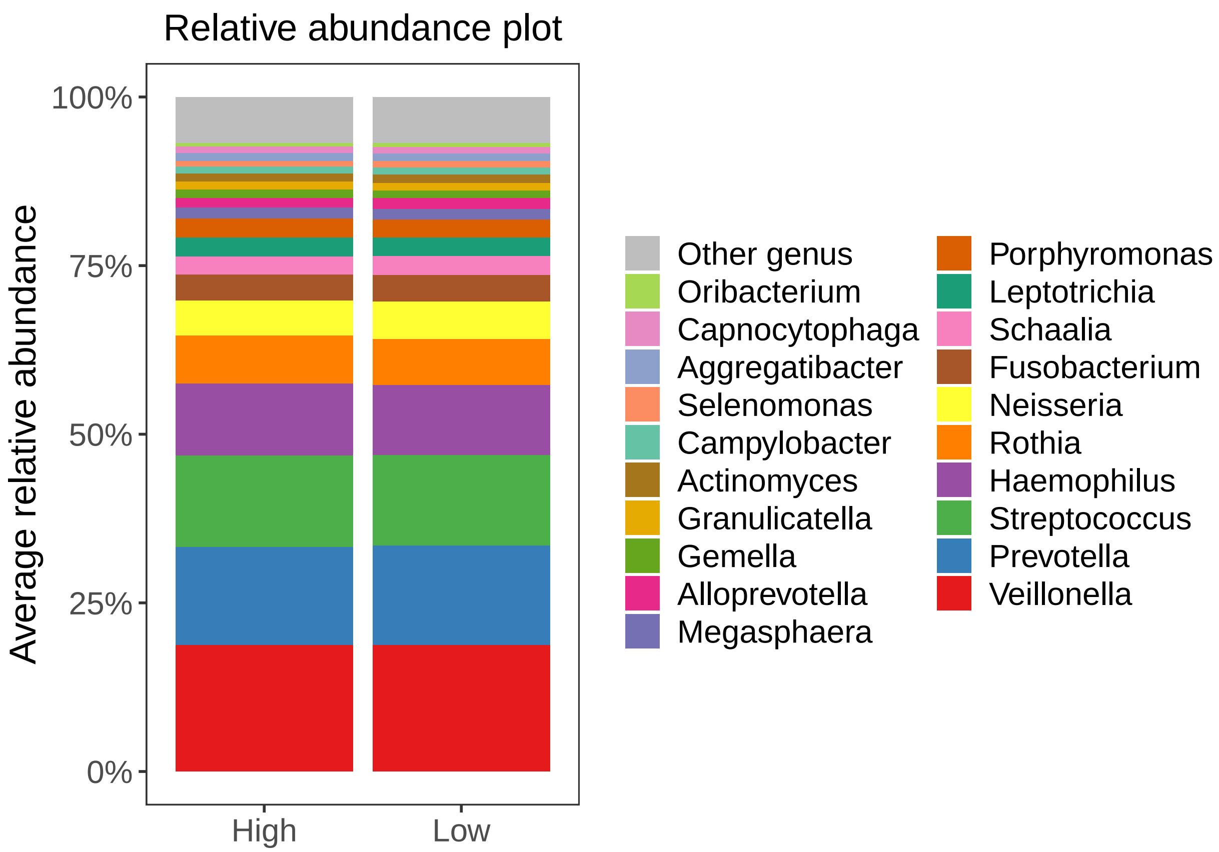
Relative abundance plot
Average relative abundance
100%
75%
50%
25%
0%
High
Low
Other genus
Oribacterium
Capnocytophaga
Aggregatibacter
Selenomonas
Campylobacter
Actinomyces
Granulicatella
Gemella
Alloprevotella
Megasphaera
Porphyromonas
Leptotrichia
Schaalia
Fusobacterium
Neisseria
Rothia
Haemophilus
Streptococcus
Prevotella
Veillonella

```
## [1] "act_cat"
```

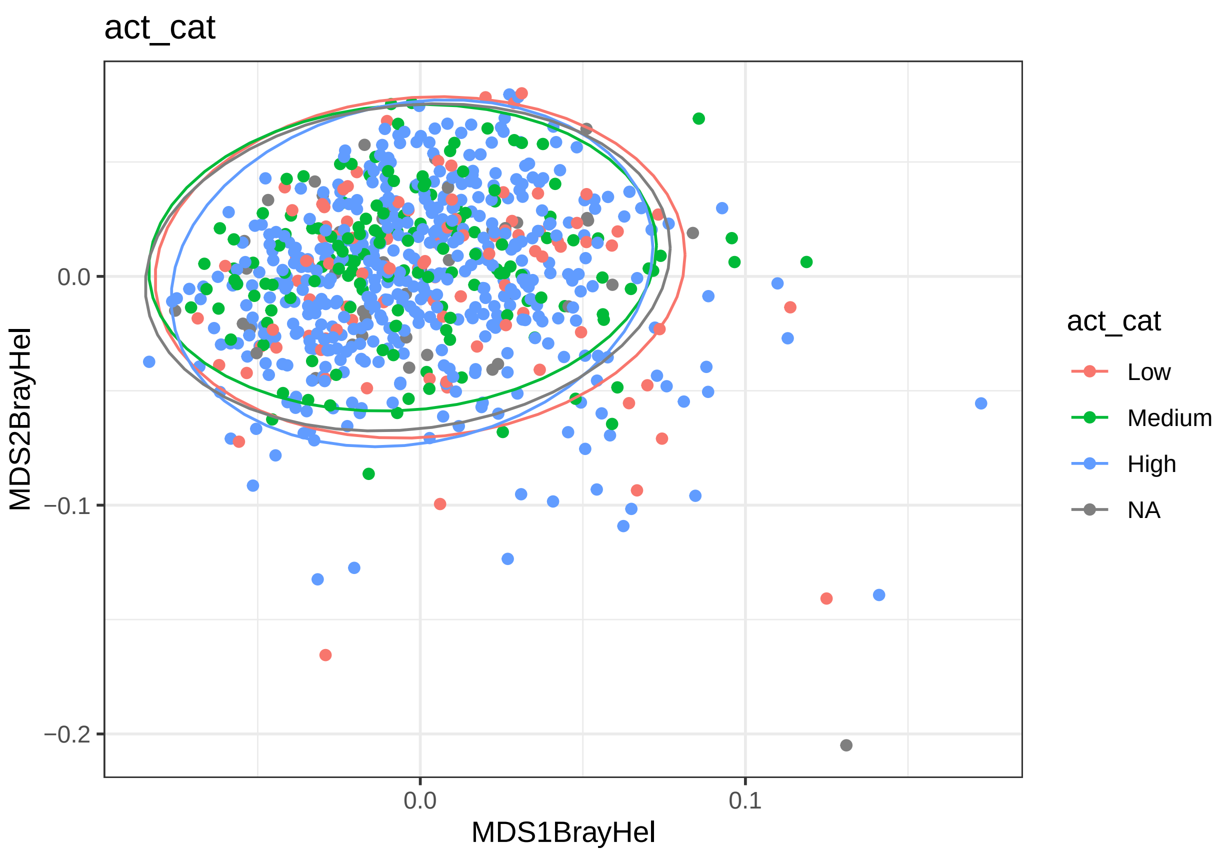


```
## Permutation test for adonis under reduced model
## Terms added sequentially (first to last)
## Permutation: free
## Number of permutations: 999
##
## adonis2(formula = formula(paste("distmatrix ~ ", i)), data = Phe3, permutations = 999, by = "terms")
##           Df SumOfSqs      R2      F Pr(>F)
## act_cat    2   0.1058 0.00423 1.4844  0.075 .
## Residual 699  24.9047 0.99577
## Total    701  25.0105 1.00000
## ---
## Signif. codes:  0 '***' 0.001 '**' 0.01 '*' 0.05 '.' 0.1 ' ' 1
```

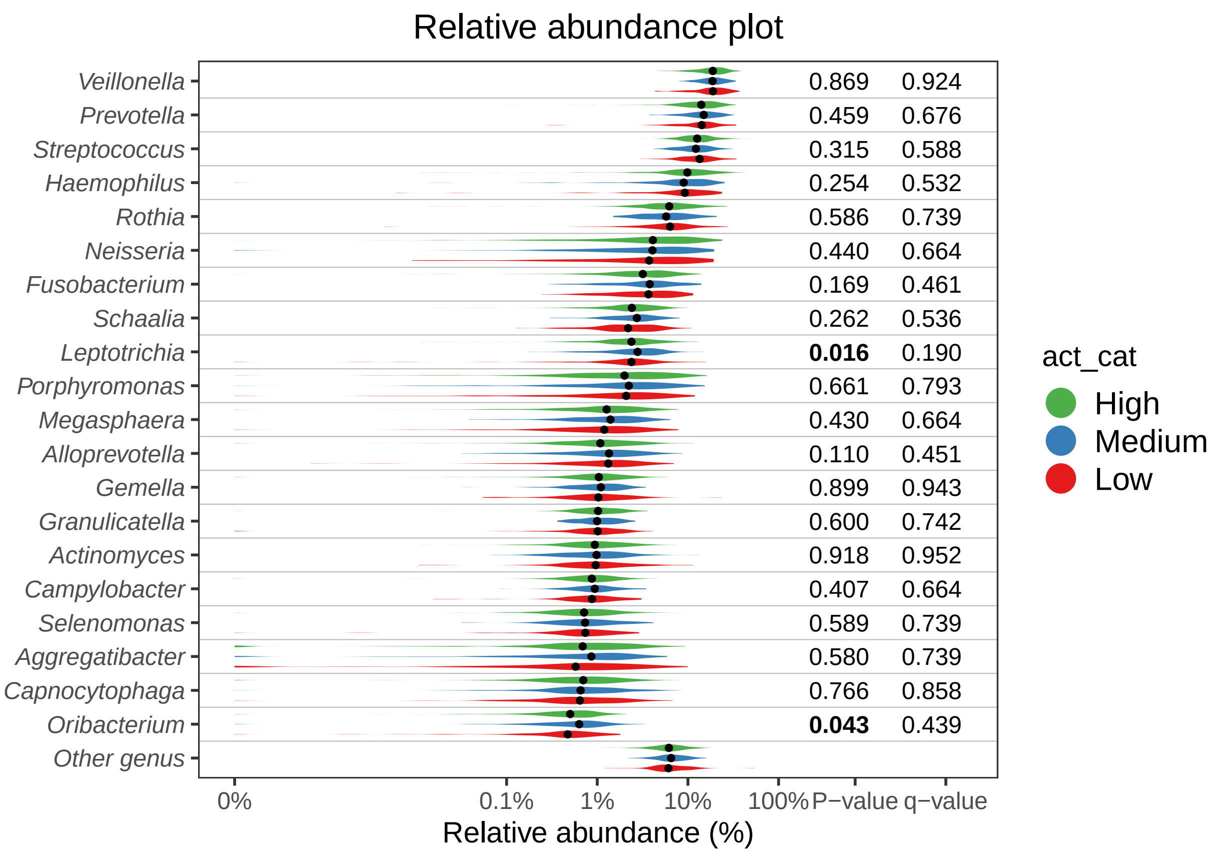
Relative abundance plot
Veillonella
Prevotella
Streptococcus
Haemophilus
Rothia
Neisseria
Fusobacterium
Schaalia
Leptotrichia
Porphyromonas
Megasphaera
Alloprevotella
Gemella
Granulicatella
Actinomyces
Campylobacter
Selenomonas
Aggregatibacter
Capnocytophaga
Oribacterium
Other genus
0.869 0.924
0.459 0.676
0.315 0.588
0.254 0.532
0.586 0.739
0.440 0.664
0.169 0.461
0.262 0.536
0.016 0.190
0.661 0.793
0.430 0.664
0.110 0.451
0.899 0.943
0.600 0.742
0.918 0.952
0.407 0.664
0.589 0.739
0.580 0.739
0.766 0.858
0.043 0.439
0%
0.1%
1%
10%
100%
P−value
q−value
Relative abundance (%)
act_cat
High
Medium
Low

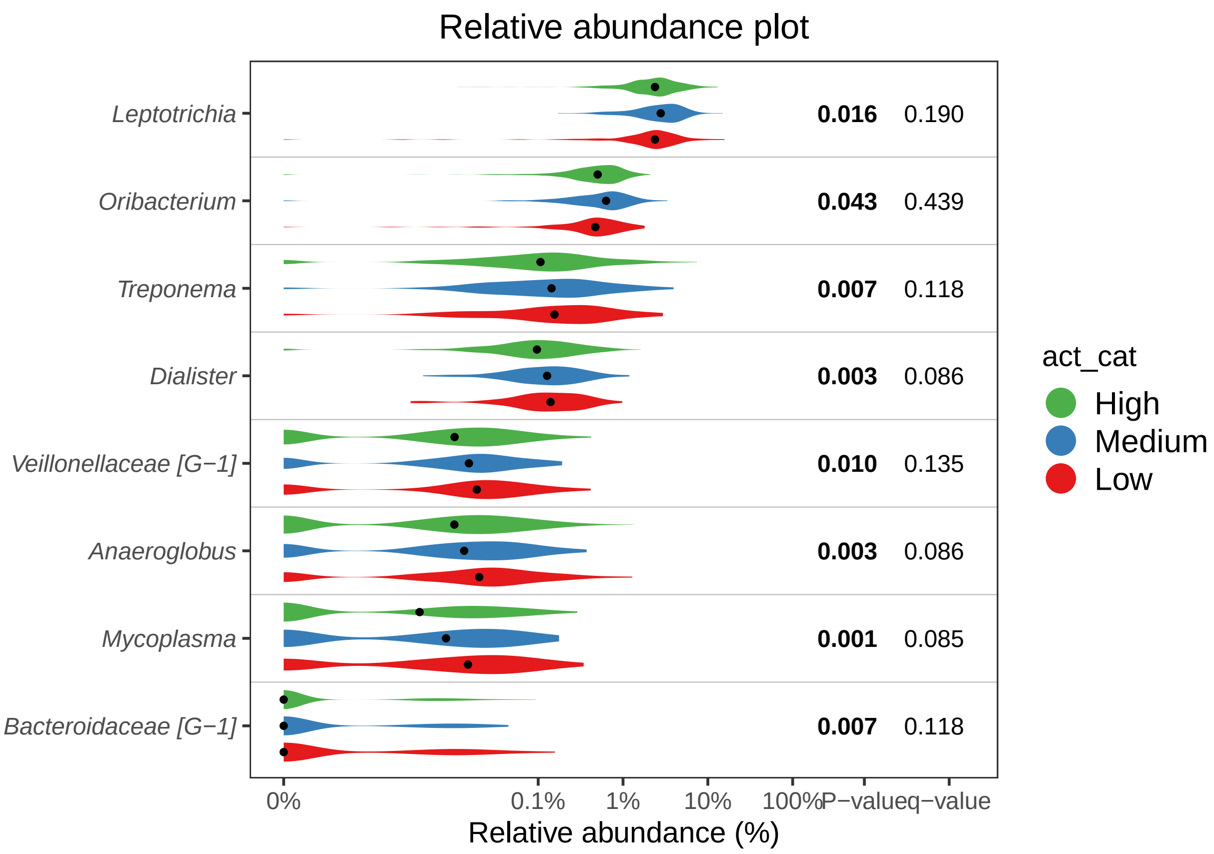
Relative abundance plot
Leptotrichia
Oribacterium
Treponema
Dialister
Veillonellaceae [G−1]
Anaeroglobus
Mycoplasma
Bacteroidaceae [G−1]
0.016 0.190
0.043 0.439
0.007 0.118
0.003 0.086
0.010 0.135
0.003 0.086
0.001 0.085
0.007 0.118
0%
0.1%
1%
10%
100%
P−value
q−value
Relative abundance (%)
act_cat
High
Medium
Low

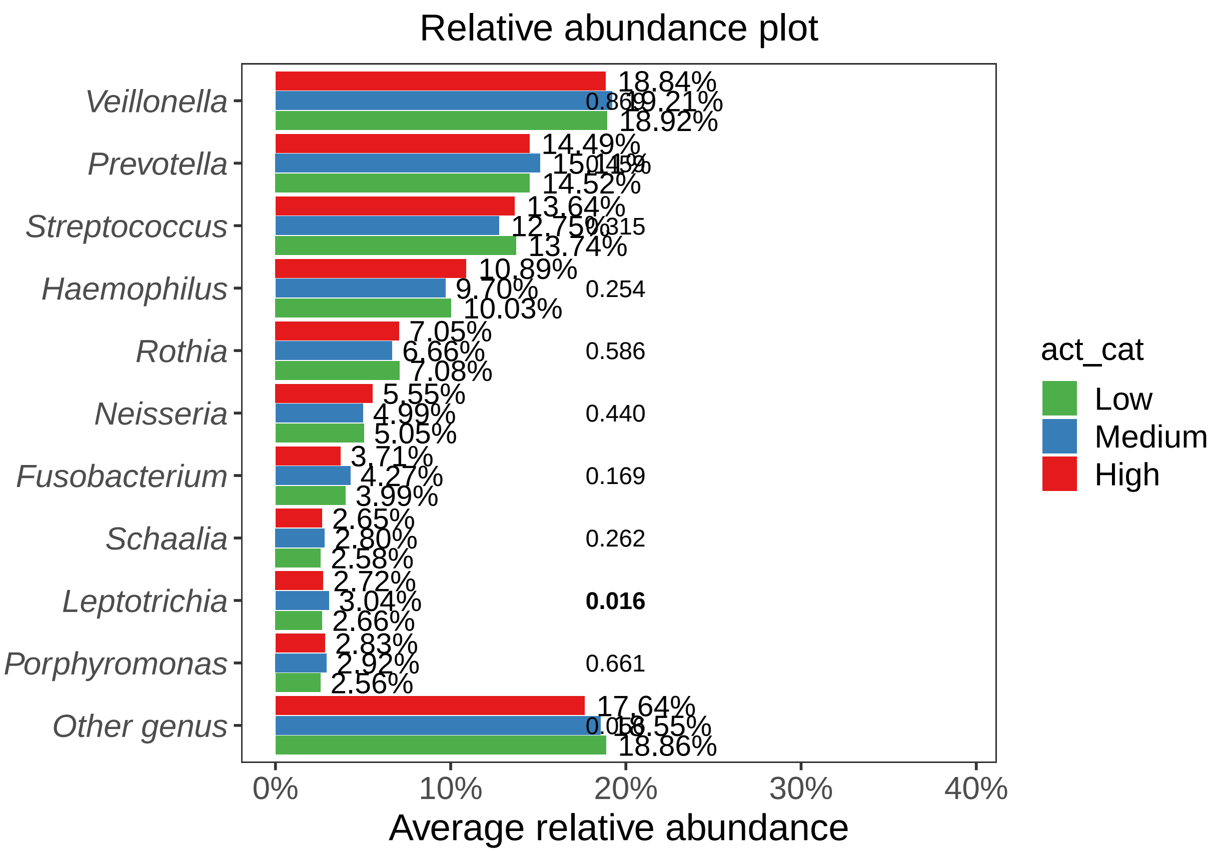
Relative abundance plot
Veillonella
Prevotella
Streptococcus
Haemophilus
Rothia
Neisseria
Fusobacterium
Schaalia
Leptotrichia
Porphyromonas
Other genus
18.84%
19.21%
18.92%
14.49%
14.52%
13.64%
13.74%
10.89%
9.70%
10.03%
0.254
7.05%
6.66%
7.08%
0.586
5.55%
4.99%
5.05%
0.440
3.71%
4.27%
3.99%
0.169
2.65%
2.80%
2.58%
0.262
2.72%
3.04%
2.66%
0.016
2.83%
2.92%
2.56%
0.661
17.64%
18.55%
18.86%
0%
10%
20%
30%
40%
Average relative abundance
act_cat
Low
Medium
High

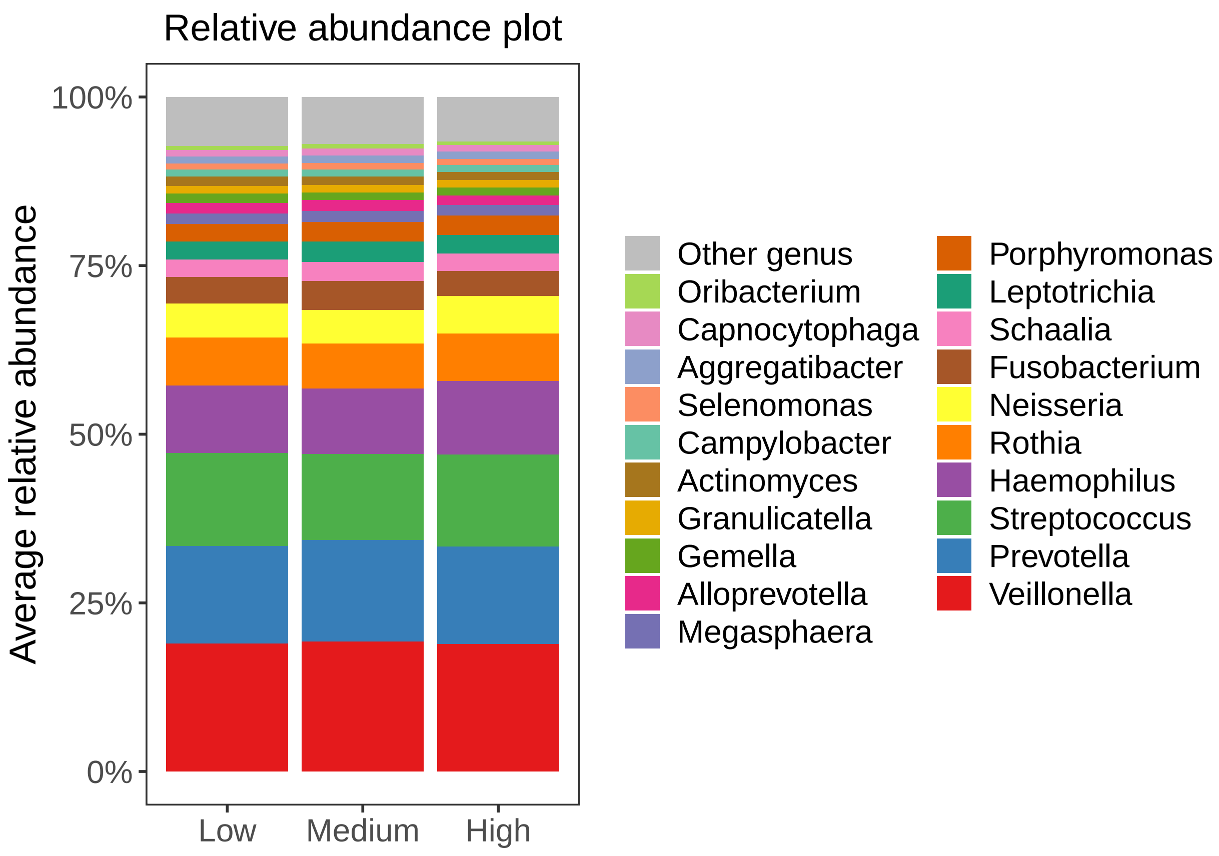


```
## [1] "Glycaemia_Status"
```

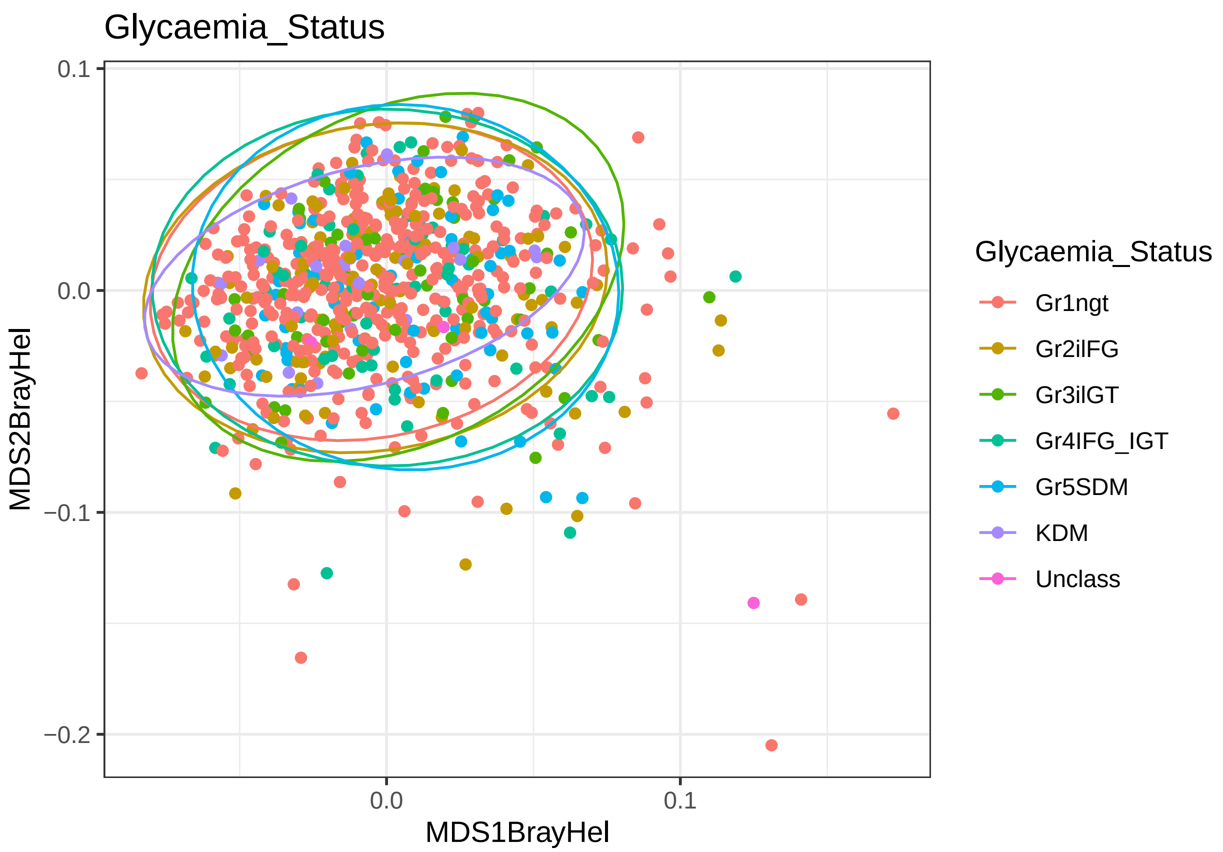


```
## Permutation test for adonis under reduced model
## Terms added sequentially (first to last)
## Permutation: free
## Number of permutations: 999
##
## adonis2(formula = formula(paste("distmatrix ~ ", i)), data = Phe3, permutations = 999, by = "terms")
##                    Df SumOfSqs      R2      F Pr(>F)
## Glycaemia_Status    6   0.3601 0.01339 1.6714  0.005 **
## Residual          739  26.5323 0.98661
## Total             745  26.8923 1.00000
## ---
## Signif. codes:  0 '***' 0.001 '**' 0.01 '*' 0.05 '.' 0.1 ' ' 1
```

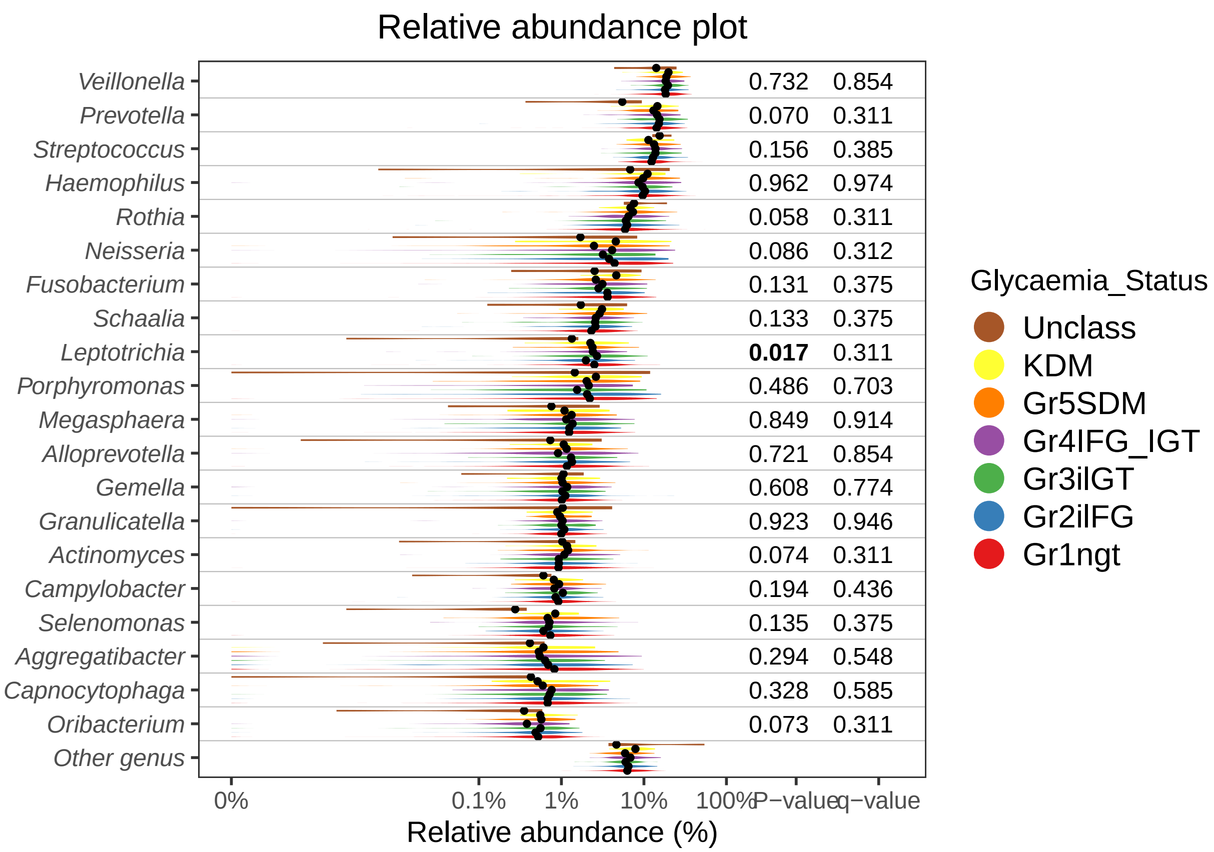
Relative abundance plot
Veillonella 0.732 0.854
Prevotella 0.070 0.311
Streptococcus 0.156 0.385
Haemophilus 0.962 0.974
Rothia 0.058 0.311
Neisseria 0.086 0.312
Fusobacterium 0.131 0.375
Schaalia 0.133 0.375
Leptotrichia 0.017 0.311
Porphyromonas 0.486 0.703
Megasphaera 0.849 0.914
Alloprevotella 0.721 0.854
Gemella 0.608 0.774
Granulicatella 0.923 0.946
Actinomyces 0.074 0.311
Campylobacter 0.194 0.436
Selenomonas 0.135 0.375
Aggregatibacter 0.294 0.548
Capnocytophaga 0.328 0.585
Oribacterium 0.073 0.311
Other genus
0%
0.1%
1%
10%
100%
P−value
q−value
Relative abundance (%)
Glycaemia_Status
Unclass
KDM
Gr5SDM
Gr4IFG_IGT
Gr3ilGT
Gr2ilFG
Gr1ngt

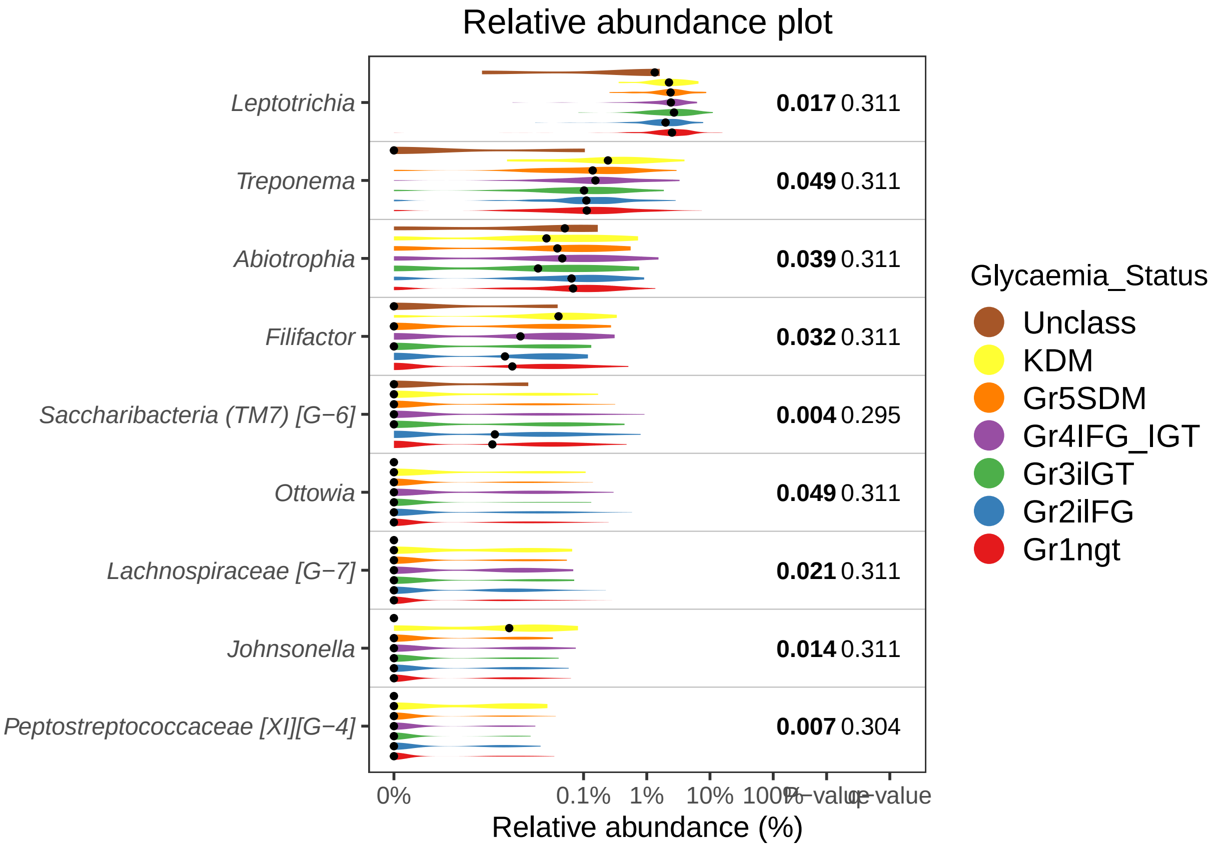

Relative abundance plot
Leptotrichia
Treponema
Abiotrophia
Filifactor
Saccharibacteria (TM7) [G−6]
Ottowia
Lachnospiraceae [G−7]
Johnsonella
Peptostreptococcaceae [XI][G−4]
0.017 0.311
0.049 0.311
0.039 0.311
0.032 0.311
0.004 0.295
0.049 0.311
0.021 0.311
0.014 0.311
0.007 0.304
0%
0.1%
1%
10%
100%
P−value
q−value
Relative abundance (%)
Glycaemia_Status
Unclass
KDM
Gr5SDM
Gr4IFG_IGT
Gr3iIGT
Gr2iIFG
Gr1ngt

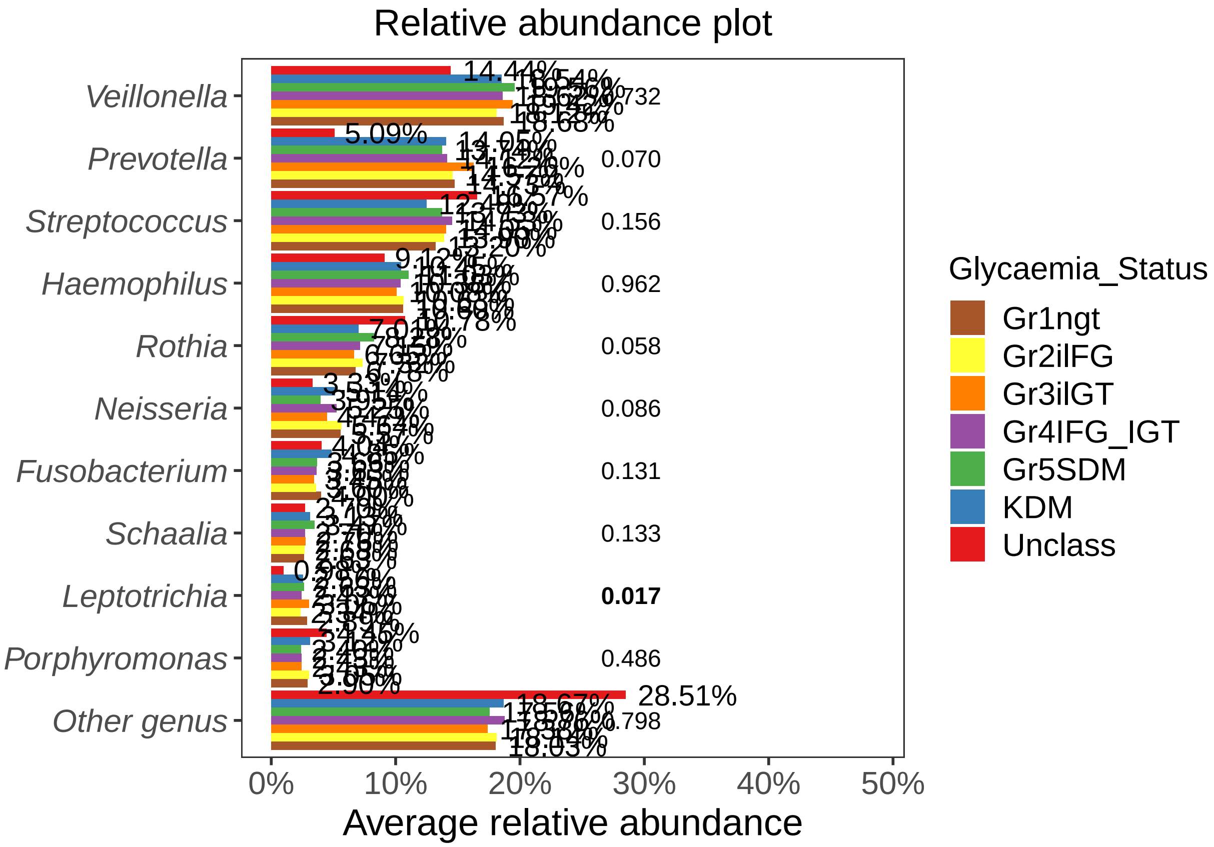
Relative abundance plot
Veillonella
Prevotella
Streptococcus
Haemophilus
Rothia
Neisseria
Fusobacterium
Schaalia
Leptotrichia
Porphyromonas
Other genus
14.44%
5.09%
28.51%
0.070
0.156
0.962
0.058
0.086
0.131
0.133
0.017
0.486
0%
10%
20%
30%
40%
50%
Average relative abundance
Glycaemia_Status
Gr1ngt
Gr2ilFG
Gr3ilGT
Gr4IFG_IGT
Gr5SDM
KDM
Unclass

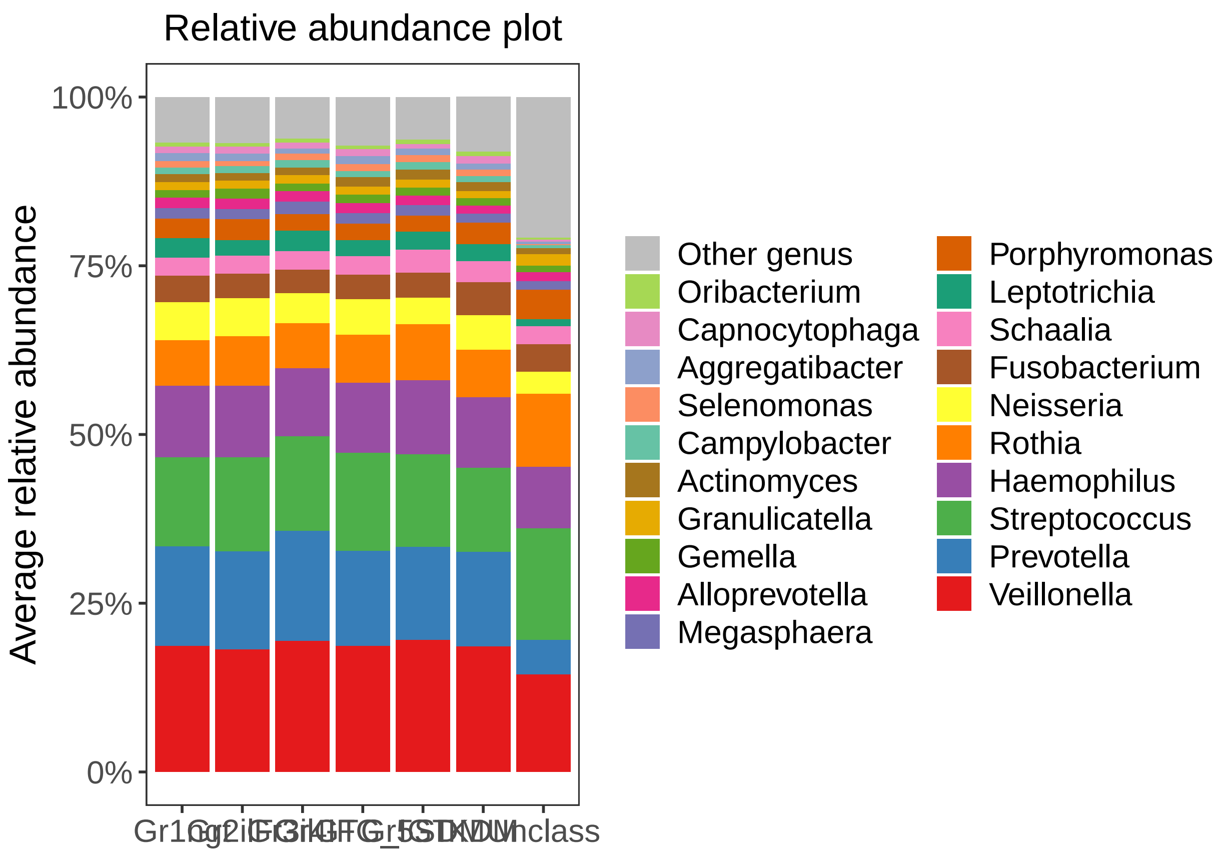
Relative abundance plot
Average relative abundance
100%
75%
50%
25%
0%
Other genus
Oribacterium
Capnocytophaga
Aggregatibacter
Selenomonas
Campylobacter
Actinomyces
Granulicatella
Gemella
Alloprevotella
Megasphaera
Porphyromonas
Leptotrichia
Schaalia
Fusobacterium
Neisseria
Rothia
Haemophilus
Streptococcus
Prevotella
Veillonella

```
## [1] "hba1c_cat"
```

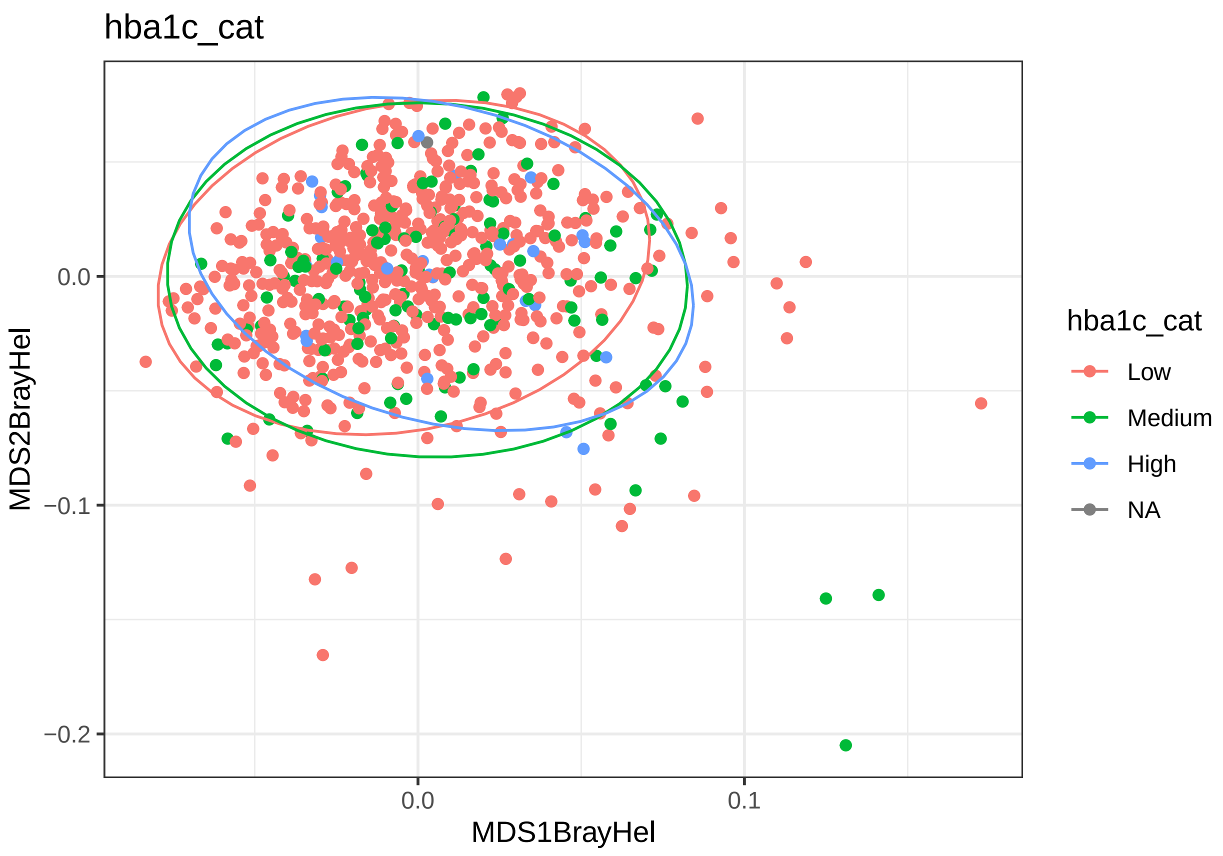


```
## Permutation test for adonis under reduced model
## Terms added sequentially (first to last)
## Permutation: free
## Number of permutations: 999
##
## adonis2(formula = formula(paste("distmatrix ~ ", i)), data = Phe3, permutations = 999, by = "terms")
##             Df SumOfSqs      R2      F Pr(>F)
## hba1c_cat    2   0.1876 0.00698 2.6087  0.002 **
## Residual   742  26.6785 0.99302
## Total      744  26.8661 1.00000
## ---
## Signif. codes:  0 '***' 0.001 '**' 0.01 '*' 0.05 '.' 0.1 ' ' 1
```

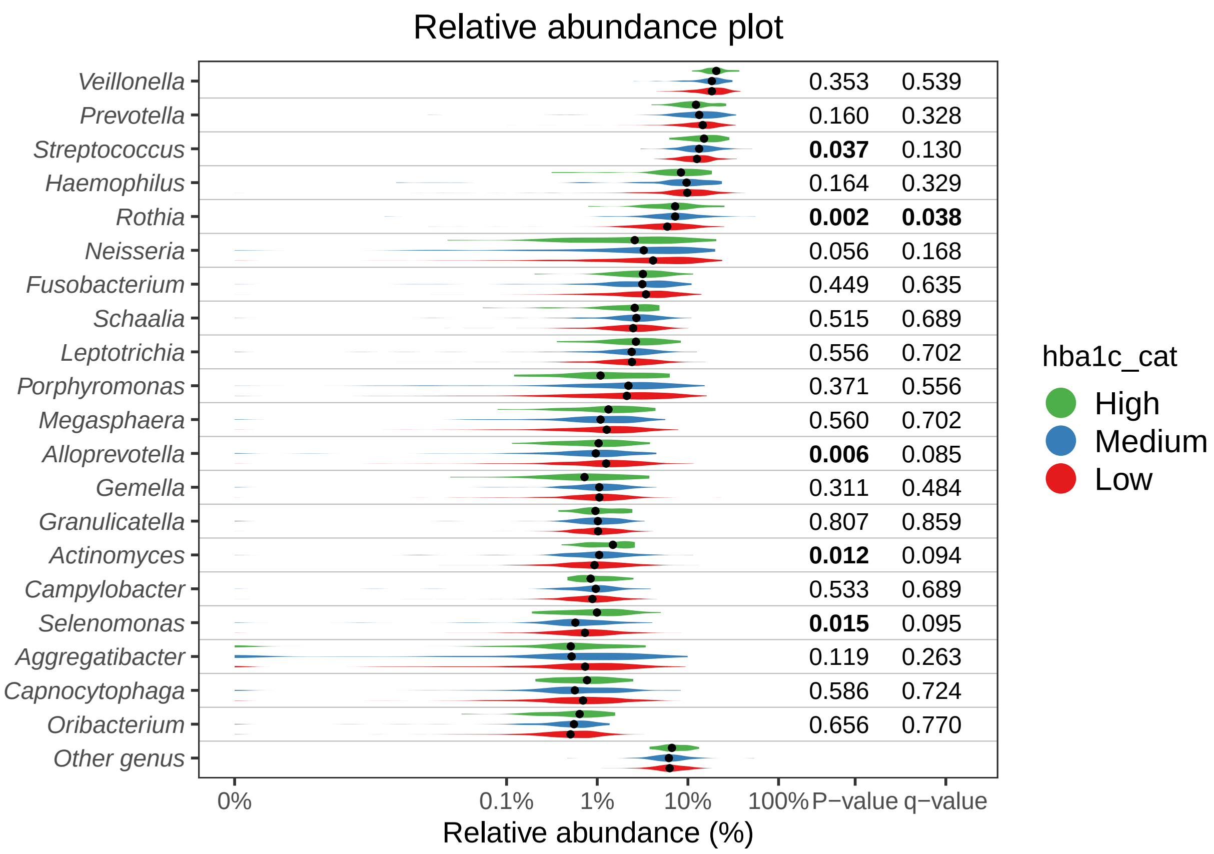
Relative abundance plot
Veillonella
Prevotella
Streptococcus
Haemophilus
Rothia
Neisseria
Fusobacterium
Schaalia
Leptotrichia
Porphyromonas
Megasphaera
Alloprevotella
Gemella
Granulicatella
Actinomyces
Campylobacter
Selenomonas
Aggregatibacter
Capnocytophaga
Oribacterium
Other genus
0.353 0.539
0.160 0.328
0.037 0.130
0.164 0.329
0.002 0.038
0.056 0.168
0.449 0.635
0.515 0.689
0.556 0.702
0.371 0.556
0.560 0.702
0.006 0.085
0.311 0.484
0.807 0.859
0.012 0.094
0.533 0.689
0.015 0.095
0.119 0.263
0.586 0.724
0.656 0.770
0%
0.1%
1%
10%
100%
P−value
q−value
Relative abundance (%)
hba1c_cat
High
Medium
Low

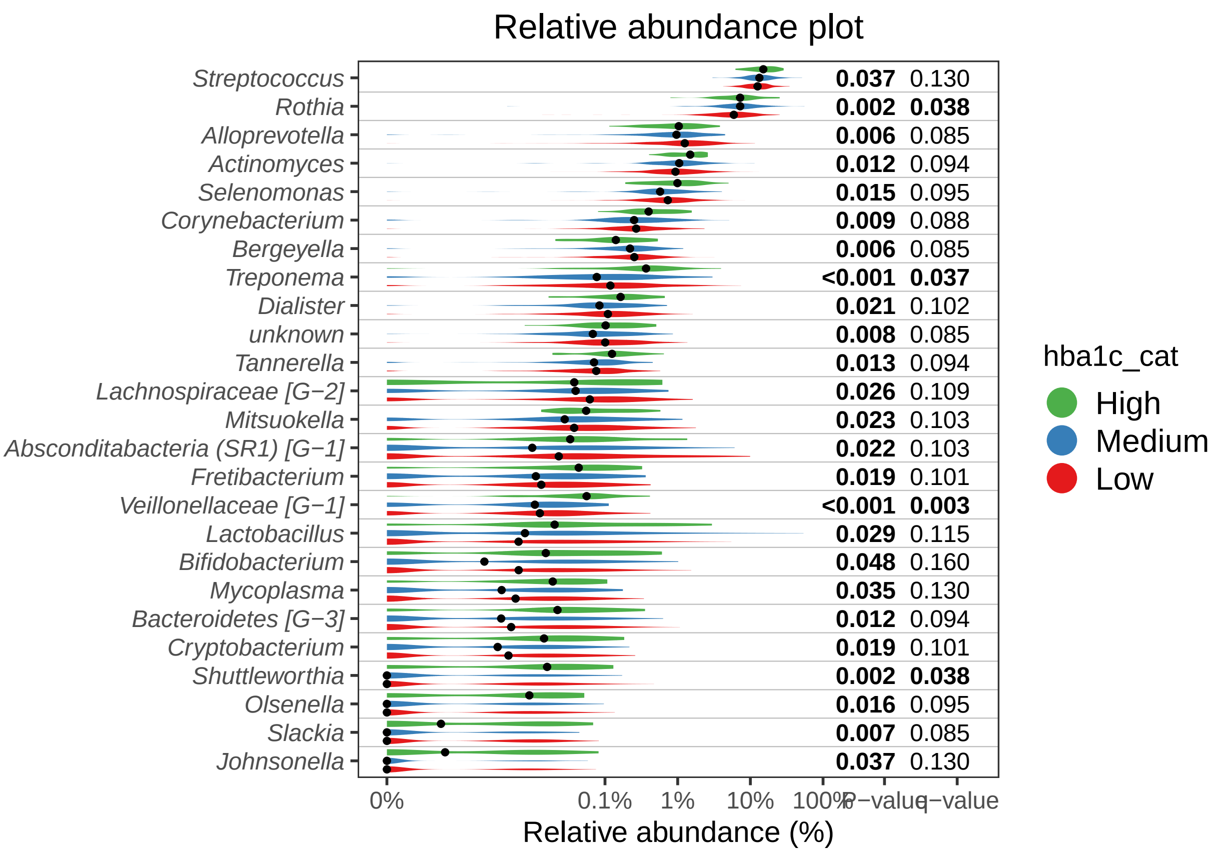
Relative abundance plot
Streptococcus
Rothia
Alloprevotella
Actinomyces
Selenomonas
Corynebacterium
Bergeyella
Treponema
Dialister
unknown
Tannerella
Lachnospiraceae [G−2]
Mitsuokella
Absconditabacteria (SR1) [G−1]
Fretibacterium
Veillonellaceae [G−1]
Lactobacillus
Bifidobacterium
Mycoplasma
Bacteroidetes [G−3]
Cryptobacterium
Shuttleworthia
Olsenella
Slackia
Johnsonella
0.037 0.130
0.002 0.038
0.006 0.085
0.012 0.094
0.015 0.095
0.009 0.088
0.006 0.085
<0.001 0.037
0.021 0.102
0.008 0.085
0.013 0.094
0.026 0.109
0.023 0.103
0.022 0.103
0.019 0.101
<0.001 0.003
0.029 0.115
0.048 0.160
0.035 0.130
0.012 0.094
0.019 0.101
0.002 0.038
0.016 0.095
0.007 0.085
0.037 0.130
0%
0.1%
1%
10%
100%
P−value
q−value
Relative abundance (%)
hba1c_cat
High
Medium
Low

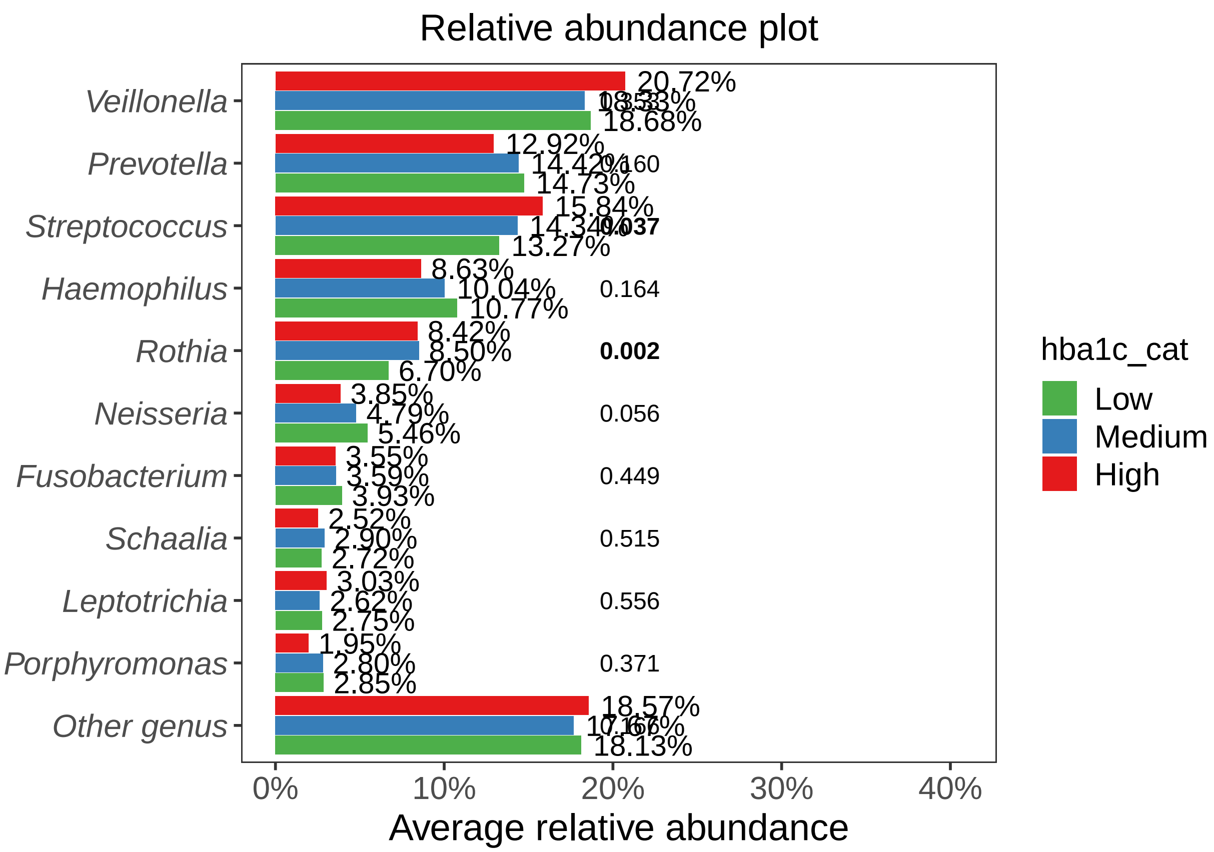
Relative abundance plot
Veillonella
Prevotella
Streptococcus
Haemophilus
Rothia
Neisseria
Fusobacterium
Schaalia
Leptotrichia
Porphyromonas
Other genus
20.72%
18.68%
12.92%
14.73%
15.84%
13.27%
8.63%
10.04%
10.77%
0.164
8.42%
8.50%
6.70%
0.002
3.85%
4.79%
5.46%
0.056
3.55%
3.59%
3.93%
0.449
2.52%
2.90%
2.72%
0.515
3.03%
2.62%
2.75%
0.556
1.95%
2.80%
2.85%
0.371
18.57%
18.13%
0%
10%
20%
30%
40%
Average relative abundance
hba1c_cat
Low
Medium
High

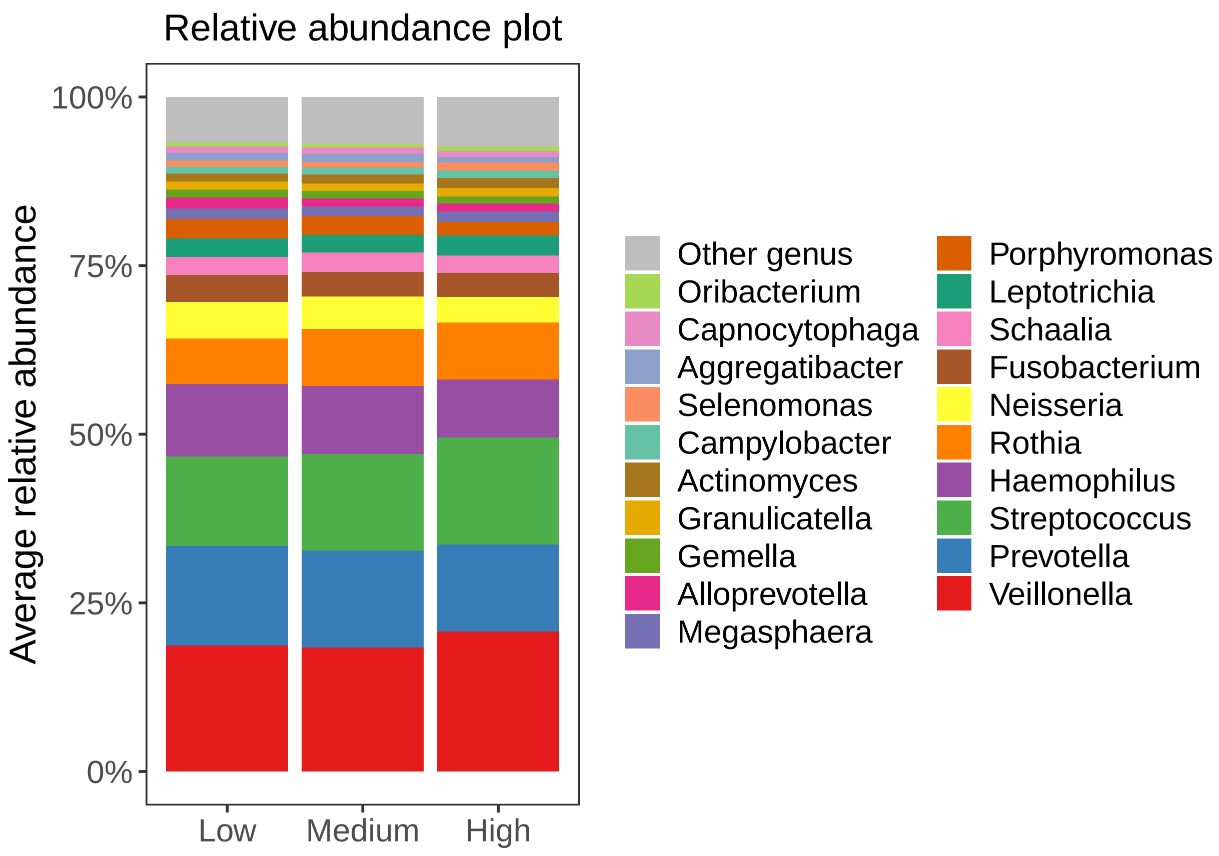


```
# ###########Investigating rabuplot mgs_feature
# var<-c("Risk", "gly_stat",
#        "BMI_cat", "Waist_cat",
#        "Alcprweek_cat", "Smoking", "bp_cat",
#        "sbp_cat", "dbp_cat", "hr_cat",
#        "hdlc_cat",
#        "ldlc_cat", "chol_cat", "act_cat",
#        "Glycaemia_Status", "hba1c_cat")
# #Variables sex_cat, whratio_cat, pp_cat, trig_cat with "Error in mgsfit$taxa : $ operator not defined
# table(Phe3$whratio_cat, useNA="always")
# for (i in var) {
#   ##Stats are metagenomeseq as assessed in DAtest
#   print(rabuplot(ps2, i, p_adjust=T, N_taxa=10, stats="mgs_feature", p_adjust_full=T))
#   print(rabuplot(ps2, i, p_adjust=T, N_taxa=500, stats="mgs_feature", Only_sig=T, p_adjust_full=T))
# }
# rabuplot(ps2, "hdlc_cat", p_adjust=T, N_taxa=10, stats="mgs_feature")
# rabuplot(ps2, "Risk", p_adjust=T, N_taxa=10, stats="mgs_feature")
# class(Phe2$hdlc_cat)
# class(Phe2$Risk)
# table(Phe2$Risk, useNA="always")
# ###########################################


#PCoAlist$Risk
#PCoAlist$gly_stat
#adonislist$Risk
```

```r
#adonislist$gly_stat


#Remove NAs for adonis
var2<-c("Glycaemia_Status", "sex_cat",
       "BMI_cat", "Waist_cat", "whratio_cat",
       "Smoking", "bp_cat",
       "sbp_cat", "dbp_cat", "hr_cat",
       "pp_cat", "trig_cat", "hdlc_cat",
       "ldlc_cat", "chol_cat", "act_cat",
       "hba1c_cat", "Risk", "bmi", "waist_av",
       "whratio", "bp_av", "sbp_av", "dbp_av",
       "hr_av", "pp_av", "p_lab_trig", "p_lab_chol",
       "p_lab_hdlc","p_lab_ldlc", "p_lab_hba1c", "act_cat",
       "Alcprweek_cat", "Alcprweek", "age_fup")
#Only includes rows without NAs from across the var columns
Phe3<-Phe2[complete.cases(Phe2[,which( colnames(Phe2)%in%var2 )]),]
  #var2=692 var=599 samples includes alc
#Also subset columns
Microbio3<-dplyr::select(Microbio, one_of(Phe3$IDX))
#Hellinger transformation
Microbio3 <- data.frame(t(decostand(t(Microbio3), method="hellinger")))
#Maks TSS
Microbio3<-sweep(Microbio3, 2, colSums(Microbio3), FUN="/")
#Dissimilarity
distmatrix <- vegdist(t(Microbio3), method="bray")
set.seed(1)
adonisObject<-adonis2(distmatrix ~ Risk + sex_cat + BMI_cat + Waist_cat + whratio_cat + Smoking + bp_ca
                      perm=999) #, perm=99 can increase to get exact p-values
i<-"all"
adonislist[[i]]<-adonisObject
print(adonisObject)
```

```
## Permutation test for adonis under reduced model
## Marginal effects of terms
## Permutation: free
## Number of permutations: 999
##
## adonis2(formula = distmatrix ~ Risk + sex_cat + BMI_cat + Waist_cat + whratio_cat + Smoking + bp_cat
##                  Df SumOfSqs      R2      F Pr(>F)
## Risk              2   0.0708 0.00339 1.0588  0.372
## sex_cat           1   0.0564 0.00270 1.6851  0.083 .
## BMI_cat           3   0.1237 0.00593 1.2329  0.180
## Waist_cat         2   0.0542 0.00260 0.8105  0.695
## whratio_cat       1   0.0393 0.00188 1.1750  0.278
## Smoking           2   0.6303 0.03020 9.4239  0.001 ***
## bp_cat            3   0.1016 0.00487 1.0131  0.413
## sbp_cat           3   0.1261 0.00604 1.2571  0.126
## dbp_cat           3   0.0970 0.00464 0.9664  0.480
## hr_cat            2   0.0629 0.00301 0.9404  0.502
## pp_cat            1   0.0538 0.00258 1.6082  0.085 .
## trig_cat          1   0.0492 0.00236 1.4718  0.132
## hdlc_cat          1   0.0177 0.00085 0.5303  0.919
```

```
## ldlc_cat        1   0.0443 0.00212 1.3255  0.214
## chol_cat        1   0.0249 0.00119 0.7444  0.696
## act_cat         2   0.0850 0.00407 1.2703  0.176
## Alcprweek_cat   2   0.1263 0.00605 1.8876  0.010 **
## hba1c_cat       2   0.0851 0.00408 1.2727  0.159
## age_fup         1   0.1320 0.00633 3.9478  0.001 ***
## Residual      561  18.7622 0.89884
## Total         595  20.8738 1.00000
## ---
## Signif. codes:  0 '***' 0.001 '**' 0.01 '*' 0.05 '.' 0.1 ' ' 1
```

```
#Significant in individual test
adonisObject<-adonis2(distmatrix ~ Risk + sex_cat + Smoking + hba1c_cat + Alcprweek_cat + act_cat, Phe3
                      perm=999)
i<-"allsig"
adonislist[[i]]<-adonisObject
print(adonisObject)
```

```
## Permutation test for adonis under reduced model
## Marginal effects of terms
## Permutation: free
## Number of permutations: 999
##
## adonis2(formula = distmatrix ~ Risk + sex_cat + Smoking + hba1c_cat + Alcprweek_cat + act_cat, data =
##                  Df SumOfSqs      R2      F Pr(>F)
## Risk            2   0.1007 0.00482 1.4924  0.084 .
## sex_cat         1   0.0605 0.00290 1.7938  0.047 *
## Smoking         2   0.6131 0.02937 9.0858  0.001 ***
## hba1c_cat       2   0.0918 0.00440 1.3609  0.120
## Alcprweek_cat   2   0.1355 0.00649 2.0077  0.012 *
## act_cat         2   0.0845 0.00405 1.2528  0.173
## Residual      584  19.7049 0.94400
## Total         595  20.8738 1.00000
## ---
## Signif. codes:  0 '***' 0.001 '**' 0.01 '*' 0.05 '.' 0.1 ' ' 1
```

```
#adonisObject<-adonis2(distmatrix ~ Glycaemia_Status * sex_cat * BMI_cat * Smoking * sbp_cat * hba1c_ca
#                       perm=99)
#i<-"allsigint"
#adonislist[[i]]<-adonisObject
#print(adonisObject)






# #Have the plots stored in list
lay <- rbind(c(1,2),
             c(3,4),
             c(5,6))

pdf(paste("ADDPRO_PCoA_main.pdf", sep=""), width=13, height=13)
grid.arrange(PCoAlist$Risk,
```

```r
             PCoAlist$Smoking,
             PCoAlist$sex_cat,
             PCoAlist$Alcprweek_cat,
             PCoAlist$hba1c_cat,
             PCoAlist$act_cat, layout_matrix = lay)
dev.off()
```

```
## pdf
##   2
```

```r
pdf(paste("ADDPRO_Stackbar_main.pdf", sep=""), width=16, height=16)
grid.arrange(Rabulist$BarRisk+theme(axis.text.x = element_text(angle = -20)),
             Rabulist$BarSmoking+theme(axis.text.x = element_text(angle = -20)),
             Rabulist$Barsex_cat+theme(axis.text.x = element_text(angle = -20)),
             Rabulist$BarAlcprweek_cat+theme(axis.text.x = element_text(angle = -20)),
             Rabulist$Barhba1c_cat+theme(axis.text.x = element_text(angle = -20)),
             Rabulist$Baract_cat+theme(axis.text.x = element_text(angle = -20)), layout_matrix = lay)
dev.off()
```

```
## pdf
##   2
```

```r
pdf(paste("ADDPRO_Rabu_main1of2.pdf", sep=""), width=16, height=16)
grid.arrange(Rabulist$Risk,
             Rabulist$SigRisk,
             Rabulist$Smoking,
             Rabulist$SigmodSmoking,
             Rabulist$sex_cat,
             Rabulist$Sigsex_cat, layout_matrix = lay)
dev.off()
```

```
## pdf
##   2
```

```r
pdf(paste("ADDPRO_Rabu_main2of2.pdf", sep=""), width=16, height=16)
grid.arrange(Rabulist$Alcprweek_cat,
             Rabulist$SigAlcprweek_cat,
             Rabulist$hba1c_cat,
             Rabulist$Sighba1c_cat,
             Rabulist$act_cat,
             Rabulist$Sigact_cat, layout_matrix = lay)
dev.off()
```

```
## pdf
##   2
```

```r
lay <- rbind(c(1,2),
             c(3,4),
             c(5,6),
             c(7,8),
             c(9,10),
```

```
             c(11,12))

pdf(paste("ADDPRO_PCoA_secondary.pdf", sep=""), width=13, height=26)
grid.arrange(PCoAlist$BMI_cat,
             PCoAlist$Waist_cat,
             PCoAlist$whratio_cat,
             PCoAlist$bp_cat,
             PCoAlist$sbp_cat,
             PCoAlist$dbp_cat,
             PCoAlist$hr_cat,
             PCoAlist$pp_cat,
             PCoAlist$trig_cat,
             PCoAlist$hdlc_cat,
             PCoAlist$ldlc_cat,
             PCoAlist$chol_cat, layout_matrix = lay)
dev.off()
```

```
## pdf
##   2
```

```
pdf(paste("ADDPRO_Stackbar_secondary.pdf", sep=""), width=16, height=32)
grid.arrange(Rabulist$BarBMI_cat+theme(axis.text.x = element_text(angle = -20)),
             Rabulist$BarWaist_cat+theme(axis.text.x = element_text(angle = -20)),
             Rabulist$Barwhratio_cat+theme(axis.text.x = element_text(angle = -20)),
             Rabulist$Barbp_cat+theme(axis.text.x = element_text(angle = -20)),
             Rabulist$Barsbp_cat+theme(axis.text.x = element_text(angle = -20)),
             Rabulist$Bardbp_cat+theme(axis.text.x = element_text(angle = -20)),
             Rabulist$Barhr_cat+theme(axis.text.x = element_text(angle = -20)),
             Rabulist$Barpp_cat+theme(axis.text.x = element_text(angle = -20)),
             Rabulist$Bartrig_cat+theme(axis.text.x = element_text(angle = -20)),
             Rabulist$Barhdlc_cat+theme(axis.text.x = element_text(angle = -20)),
             Rabulist$Barldlc_cat+theme(axis.text.x = element_text(angle = -20)),
             Rabulist$Barchol_cat+theme(axis.text.x = element_text(angle = -20)), layout_matrix = lay)
dev.off()
```

```
## pdf
##   2
```

```
lay <- rbind(c(1,2),
             c(3,4),
             c(5,6),
             c(7,8),
             c(9,10),
             c(11,12),
             c(13,14),
             c(15,16),
             c(17,18),
             c(19,20),
             c(21,22),
             c(23,24))
#
pdf(paste("ADDPRO_Rabu_secondary.pdf", sep=""), width=16, height=64)
```

```r
grid.arrange(Rabulist$BMI_cat,
             Rabulist$SigBMI_cat,
             Rabulist$Waist_cat,
             Rabulist$SigWaist_cat,
             Rabulist$whratio_cat,
             Rabulist$Sigwhratio_cat,
             Rabulist$bp_cat,
             Rabulist$Sigbp_cat,
             Rabulist$sbp_cat,
             Rabulist$Sigsbp_cat,
             Rabulist$dbp_cat,
             Rabulist$Sigdbp_cat,
             Rabulist$hr_cat,
             Rabulist$Sighr_cat,
             Rabulist$pp_cat,
             Rabulist$Sigpp_cat,
             Rabulist$trig_cat,
             Rabulist$Sigtrig_cat,
             Rabulist$hdlc_cat,
             Rabulist$Sighdlc_cat,
             Rabulist$ldlc_cat,
             Rabulist$Sigldlc_cat,
             Rabulist$chol_cat,
             Rabulist$Sigchol_cat, layout_matrix = lay)
dev.off()
```

```
## pdf
##   2
```

```r
# ##Just checking if the test results correspond to the one without hellinger transformation
# #Only includes rows without NAs from the i'th column
# i<-"Risk"
# Phe3<-Phe2[complete.cases(Phe2[,which( colnames(Phe2)==i )]),]
# #Also subset columns
# Microbio3<-dplyr::select(Microbio, one_of(Phe3$IDX))
# #Hellinger transformation
# #Microbio3 <- data.frame(t(decostand(t(Microbio3), method="hellinger")))
# #Maks TSS
# Microbio3<-sweep(Microbio3, 2, colSums(Microbio3), FUN="/")
# #is scaled
# krutest<-data.frame(Value=as.numeric(Microbio3["Prevotella",]), Group=Phe3$Risk)
# kruskal.test(Value ~ Group, data=krutest)
# #colSums(Microbio3)
#
# Microbio3<-dplyr::select(Microbio, one_of(Phe3$IDX))
# #Hellinger transformation
# Microbio3 <- data.frame(t(decostand(t(Microbio3), method="hellinger")))
# #Maks TSS
# Microbio3<-sweep(Microbio3, 2, colSums(Microbio3), FUN="/")
# #is scaled and hellinger transformed
# krutest<-data.frame(Value=as.numeric(Microbio3["Prevotella",]), Group=Phe3$Risk)
# kruskal.test(Value ~ Group, data=krutest)
# #colSums(Microbio3)
```

PERMANOVA

Permutational Multivariate ANOVA based on dissimilarities (Bray-Curtis, Hellinger transformed data) using vegan::adonis ##### Microbiome Only used to validate assumptions of the PERMANOVA. For PERMANOVA results see loop in PCoA.

```
rm(list=setdiff(ls(), c("FeaturePic2", "Pic2", "Phe", "Microbio", "FeatureMic", "Metabo", "paretoscale"

##Consider moving to after test with Risk and gly_stat to keep these samples in test
#Remove smoke values that are not 1:3 so the NA values
Phe2<-subset(Phe, p_gq_smoke %in% c(1:3))
#Subset shared samples of all datasets
Microbio2<-dplyr::select(Microbio, one_of(Phe2$IDX))

#Hellinger transformation
Taxonomy2 <- data.frame(t(decostand(t(Microbio2), method="hellinger")))
#Maks TSS
Taxonomy2<-sweep(Taxonomy2, 2, colSums(Taxonomy2), FUN="/")

#Dissimilarity
distmatrix <- vegdist(t(Taxonomy2), method="bray")

#adonis can handle both continous and factor predictors
set.seed(1)
adonisObject<-adonis2(distmatrix ~ Risk, Phe2, by="terms",
                      perm=999) #, perm=999 can increase to get exact p-values
adonisObject #If significant then difference between groups

set.seed(1)
adonisObject<-adonis2(distmatrix ~ gly_stat, Phe2, by="terms",
                      perm=999) #, perm=999 can increase to get exact p-values
adonisObject #If significant then difference between groups


set.seed(1)
adonisObject<-adonis2(distmatrix ~ p_gq_smoke, Phe2, by="terms",
                      perm=999) #, perm=999 can increase to get exact p-values
adonisObject #If significant then difference between groups

#With both risk and smoking
set.seed(1)
adonisObject<-adonis2(distmatrix ~ Risk + p_gq_smoke, Phe2, by="terms",
                      perm=999) #, perm=999 can increase to get exact p-values
adonisObject #If significant then difference between groups

#Swithcing order
set.seed(1)
adonisObject<-adonis2(distmatrix ~ p_gq_smoke + Risk, Phe2, by="terms",
                      perm=999) #, perm=999 can increase to get exact p-values
adonisObject #If significant then difference between groups

#Test with by = margin does not matter the order
set.seed(1)
adonisObject<-adonis2(distmatrix ~ p_gq_smoke + Risk, Phe2, by="margin",
```

```
                        perm=999) #, perm=999 can increase to get exact p-values
adonisObject #If significant then difference between groups


## Evaluating the model assumptions
TestModel <- with(Phe2, betadisper(distmatrix, Risk)) #Can not run
  #betadisper with multiple independant variables
#TestModel
#plot(TestModel)
plot(TestModel, label=FALSE)
boxplot(TestModel)
anova(TestModel) #p>0.05 -> Assumption met
#permutest(TestModel)

## Evaluating the model assumptions
TestModel <- with(Phe2, betadisper(distmatrix, p_gq_smoke)) #Can not run
  #betadisper with multiple independant variables
#TestModel
#plot(TestModel)
plot(TestModel, label=FALSE)
boxplot(TestModel)
anova(TestModel) #p>0.05 -> Assumption met
#permutest(TestModel)

# table(Phe2$gly_stat, Phe2$sex, useNA="always")
# table(Phe2$gly_stat, Phe2$p_gq_smoke, useNA="always")
# table(Phe2$p_gq_smoke, useNA="always")
# table(Phe2$p_gq_high_bp_code, useNA="always")

#Test with Risk and categorical variables, using by = margin.
set.seed(1)
adonisObject<-adonis2(distmatrix ~ Risk + Smoking + age_fup + sex + BMI_cat + whratio_cat + Waist_cat +
                        perm=999) #, perm=999 can increase to get exact p-values
adonisObject #If significant then difference between groups

# #Test with Risk and categorical variables, reduced, using by = margin.
# set.seed(1)
# adonisObject<-adonis2(distmatrix ~ Risk + Smoking + age_fup + sex + hba1c_cat, Phe3, by="margin",
#                         perm=999) #, perm=999 can increase to get exact p-values
# adonisObject #If significant then difference between groups
#
# #Test with Risk and continuous variables, using by = margin.
# set.seed(1)
# adonisObject<-adonis2(distmatrix ~ Risk + Smoking + age_fup + sex + bmi + whratio + Waist_av + hr_av
```

MaAsLin: Multivariate Association with Linear Models

Run default, but also with min_abundance = 0.0001, min_prevalence = 0.1 (Remember did already perform filtering if running additional filtering after alpha diversity estimates)

```
rm(list=setdiff(ls(), c("FeaturePic2", "Pic2", "Phe", "Microbio", "FeatureMic", "Metabo", "paretoscale")
```

```
Metadata2 <- Phe
Taxonomy2 <- Microbio
#Metabo2 <- Metabo


row.names(Metadata2) <-Metadata2$IDX

#Check order, removes smokers afterwards become false
sum((rownames( Metadata2 ) == colnames( Taxonomy2 ))) == length(Taxonomy2)

#Remove blank smokers, will remove samples automatically from taxonomy running Maaslin2
Metadata2<-subset(Metadata2, p_gq_smoke %in% c(1:3))
Metadata2$Smoking<-as.character(ifelse(grepl("1", Metadata2$p_gq_smoke), "Smoker",
                              ifelse(grepl("2", Metadata2$p_gq_smoke), "Exsmoker",
                                     ifelse(grepl("3", Metadata2$p_gq_smoke),
                                            "Nonsmoker", "NA"))))

Metadata2$HOMAIR<-Metadata2$p_lab_insu0*Metadata2$p_lab_pglu0/22.5
#fasting insulin (microU/L) x fasting glucose (nmol/L)/22.5.
#Values provided are in pmol/L and mmol/L can not get the calculations to correspond to
#Some provide conversion factor to be 6 others 6.945 (1 µIU/mL = 6.00 pmol/L)

Metadata2$whratio<-Metadata2$waist_av/Metadata2$hip_av

##Select columns used in model
Metadata2 <- dplyr::select(Metadata2, one_of(c('Risk', 'Smoking', 'bmi', 'age_fup', 'sex_cat',
                                        'waist_av', 'whratio', 'p_lab_trig', 'p_lab_chol',
                                        'p_lab_hdlc', 'p_lab_ldl', 'dbp_av', 'sbp_av')))
#

#Check order
sum((rownames( Metadata2 ) == colnames( Taxonomy2 ))) == length(Taxonomy2)
##Comments
#It is okay to provide the taxonomy file like this with sample names as column.
  #However the standard is as rows.
#If samples are missing in either of the files they are removed from the analysis,
  #so the analysis will still run.
#Same order also not needed


#In description it is stated that data is expected to be normalized before using MaAsLin
  #"so are proportional data ranging from 0 to 1.0."
#Consider performing the filtering before hand to keep TSS data
#Total sum scaling (Use relative abundances)
Taxonomy2<-sweep(Taxonomy2, 2, colSums(Taxonomy2), FUN="/")

#In demo example https://github.com/biobakery/Maaslin2 about tweeking
#Have site (place of sampling) and subject (multiple sample from the same person) as
  #random effects (don't see a reason why we should specify any variables as random
  #effects)
#Can change to run other than the linear model
#Can change transformation
#Can change normalization from TSS to CLR, but also CSS, None and TMM.
```

```
#Microbiome
fit_data <- Maaslin2(
    Taxonomy2, Metadata2, 'Maaslin2_ADDPRO_Microbiome', transform = "NONE",
    fixed_effects = c('Risk', 'Smoking', 'bmi', 'age_fup', 'sex_cat', 'waist_av', 'whratio',
                      'p_lab_trig', 'p_lab_chol', 'p_lab_hdlc', 'p_lab_ldl', 'dbp_av',
                      'sbp_av'), #The order does not change results
    #random_effects = c('centre', 'operator'), #Consider including centre
      #and operator as random effect
    reference = "Smoking,Nonsmoker",
    normalization = 'NONE',
    standardize = FALSE,
    min_abundanc=0.0001,
    min_prevalence=0.25)

fit_data <- Maaslin2(
    Taxonomy2, Metadata2, 'Maaslin2_ADDPRO_Microbiome_Reduced', transform = "NONE",
    fixed_effects = c('Risk', 'Smoking', 'bmi', 'age_fup', 'sex_cat'), #The order does not change resul
    #random_effects = c('centre', 'operator'), #Consider including centre
      #and operator as random effect
    reference = "Smoking,Nonsmoker",
    normalization = 'NONE',
    standardize = FALSE,
    min_abundanc=0.0001,
    min_prevalence=0.25)




##Metabolome
#fit_data <- Maaslin2(
#    Metabo2, Metadata2, 'Maaslin2_ADDPRO_Metabolome', transform = "LOG",
#    fixed_effects = c('Risk', 'Smoking', 'bmi', 'age_fup', 'sex_cat', 'waist_av', 'whratio',
#                      'p_lab_trig', 'p_lab_chol', 'p_lab_hdlc', 'p_lab_ldl', 'dbp_av',
#                      'sbp_av'), #The order does not change results
#    #random_effects = c('centre', 'operator'), #Consider including centre
#      #and operator as random effect
#    reference = "Smoking,Nonsmoker",
#    normalization = 'NONE',
#    standardize = FALSE,
#    min_abundanc=0,
#    min_prevalence=0.1)

#fit_data <- Maaslin2(
#    Metabo2, Metadata2, 'Maaslin2_ADDPRO_Metabolome_Reduced', transform = "LOG",
#    fixed_effects = c('Risk', 'Smoking', 'bmi', 'age_fup', 'sex_cat'), #The order does not change resu
#    #random_effects = c('centre', 'operator'), #Consider including centre
#      #and operator as random effect
#    reference = "Smoking,Nonsmoker",
#    normalization = 'NONE',
#    standardize = FALSE,
#    min_abundanc=0,
#    min_prevalence=0.1)
```

```
#fit_data <- Maaslin2(
#    Metabo2, Metadata2, 'Maaslin2_ADDPRO_Metabolome_OnlyRisk', transform = "LOG",
#    fixed_effects = c('Risk'), #The order does not change results
#    #random_effects = c('centre', 'operator'), #Consider including centre
#      #and operator as random effect
#    normalization = 'NONE',
#    standardize = FALSE,
#    min_abundanc=0,
#    min_prevalence=0.1)
```

Filtration for both DAtest and sPLSDA also core microbiome

Decided to do prevalence filtering on full dataset to make sure the same genera included Did not want to perform for alpha-diversity and beta-diversity measures. In beta-diversity measures are robust to these. Also consider what the DADA2 pipeline provides.

```
rm(list=setdiff(ls(), c("FeaturePic2", "Pic2", "Phe", "Microbio", "FeatureMic", "Metabo", "paretoscale"
library("DAtest")
library("eulerr")

#Core microbiome
Microbiocore<-preDA(Microbio, min.samples=round(ncol(Microbio)*0.95), min.abundance=0)
Microbiocore<-Microbiocore[!(row.names(Microbiocore) %in% "Others"),]
#Percentage from core
sum(Microbiocore)/sum(Microbio)
```

```
## [1] 0.9471407
```

```
range(colSums(Microbiocore)/colSums(Microbio))
```

```
## [1] 0.4542169 0.9982464
```

```
#Most prevalent Veilonella, Streptococcus, Haemophilus and Prevotella
Microbiofan4<-Microbiocore[row.names(Microbiocore) %in% c("Veillonella", "Streptococcus", "Haemophilus"
sum(Microbiofan4)/sum(Microbio)
```

```
## [1] 0.5665774
```

```
#Microbio<-preDA(Microbio, min.samples=round(ncol(Microbio)*0.2))
Microbio<-preDA(Microbio, min.samples=round(ncol(Microbio)*0.2), min.abundance=0)

ROClist<-list()
```

DAtest

Validated on chol_cat, Risk and Smoking. Afterwards DAtest was used to run tests with selected methods DESeq2 Wald binary and LRT multiclass

```
rm(list=setdiff(ls(), c("FeaturePic2", "Pic2", "Phe", "Microbio", "FeatureMic", "Metabo", "paretoscale"

# # Developmental version (recommended):
# devtools::install_github("Russel88/DAtest")
# # Version associated with bioRxiv paper:
# # devtools::install_github("Russel88/DAtest@v2.7.5")
#
# BiocManager::install(c("DESeq2","limma","edgeR","metagenomeSeq","baySeq","ALDEx2","impute","ANCOMBC"),
#
# install.packages(c("samr","pscl","statmod","mvabund"))
#
# # For drawing Venn diagrams
# install.packages("eulerr")
#
# # For post-hoc testing (generalized) linear models
# install.packages("lsmeans")


# #Some pruning/subsetting of taxa
# See filtration above


##The following variables where selected to validate differential abundance performance
#Selected cholestorol binary, but not significant p-value in PERMANOVA
table(Phe$chol_cat, useNA="always")
```

```
##
## High  Low <NA>
##  495  251    0
```

```
#Selected Risk binary and significant p-value in PERMANOVA
table(Phe$Risk, useNA="always")
```

```
##
##      Low     High      KDM unclass     <NA>
##      421      301       21        0        3
```

```
#Selected Smoking non-binary (called multi-class predictor in DAtest see DAtest wiki for more informati
table(Phe$Smoking, useNA="always")
```

```
##
##  Exsmoker Nonsmoker    Smoker      <NA>
##       351       261       129         5
```

```
# ################chol_cat no NA
# #runtimeDA(Microbio, predictor = varvec)
# #Check order
# sum(colnames(Microbio)!=Phe$IDX)==0
#
# test <- testDA(Microbio, predictor = Phe$chol_cat, R=20, tests=c("abc", "aov", "ds2", "ere", "vli", "
# summary(test)
```

```
# plot(test)
# pdf(paste("ADDPRO_DAtest1_Chol", ".pdf", sep=""), width=12, height=6)
# plot(test)
# dev.off()
# test$run.times
#
# fpr_plot <- ggplot(test$table, aes(x=fct_reorder(Method, -FPR), y=FPR)) +
#   geom_hline(yintercept=0.05, color="red") +
#   geom_hline(yintercept=0.1, color="red") +
#   geom_boxplot() + xlab("") + ylab("") +
#   geom_point() +
#   ylim(0,1) +
#   coord_flip() + theme_minimal() + ggtitle("False positive rate")
# fdr_plot <- ggplot(test$table, aes(x=fct_reorder(Method, -FPR), y=FDR)) +
#   geom_hline(yintercept=0.05, color="red") +
#   geom_hline(yintercept=0.1, color="red") +
#   geom_boxplot() + xlab("") + ylab("") +
#   geom_point() +
#   ylim(0,1) +
#   coord_flip() + theme_minimal() + ggtitle("False discovery rate")
# auc_plot <- ggplot(test$table, aes(x=fct_reorder(Method, -FPR), y=AUC)) +
#   geom_hline(yintercept=0.5, color="red") +
#   geom_boxplot() + xlab("") + ylab("") +
#   geom_point() +
#   ylim(0,1) +
#   coord_flip() + theme_minimal() + ggtitle("AUC")
# power_plot <- ggplot(test$table, aes(x=fct_reorder(Method, -FPR), y=Power)) +
#   geom_hline(yintercept=0.8, color="red") +
#   geom_boxplot() + xlab("") + ylab("") +
#   geom_point() +
#   ylim(0,1) +
#   coord_flip() + theme_minimal() + ggtitle("Power")
# cowplot::plot_grid(fpr_plot, fdr_plot, auc_plot, power_plot, nrow=2)
#
# pdf(paste("ADDPRO_DAtest2_Chol", ".pdf", sep=""), width=12, height=6)
# cowplot::plot_grid(fpr_plot, fdr_plot, auc_plot, power_plot, nrow=2)
# dev.off()
#
#
#
# #################Risk
# #Only includes rows high vs low
# Phe2<-subset(Phe, Risk %in% c("High", "Low"))
# #Also subset columns
# Microbio2<-dplyr::select(Microbio, one_of(Phe2$IDX))
#
#
# #runtimeDA(Microbio2, predictor = varvec)
# #Check order
# sum(colnames(Microbio2)!=Phe2$IDX)==0
# table(Phe2$Risk, useNA="always")
# Phe2$Risk<-droplevels(Phe2$Risk)
# test <- testDA(Microbio2, predictor = Phe2$Risk, R=20, tests=c("abc", "aov", "ds2", "ere", "vli", "lr
```

```
# summary(test)
# plot(test)
# pdf(paste("ADDPRO_DAtest1_Risk", ".pdf", sep=""), width=12, height=6)
# plot(test)
# dev.off()
# test$run.times
#
#
# fpr_plot <- ggplot(test$table, aes(x=fct_reorder(Method, -FPR), y=FPR)) +
#   geom_hline(yintercept=0.05, color="red") +
#   geom_hline(yintercept=0.1, color="red") +
#   geom_boxplot() + xlab("") + ylab("") +
#   geom_point() +
#   ylim(0,1) +
#   coord_flip() + theme_minimal() + ggtitle("False positive rate")
# fdr_plot <- ggplot(test$table, aes(x=fct_reorder(Method, -FPR), y=FDR)) +
#   geom_hline(yintercept=0.05, color="red") +
#   geom_hline(yintercept=0.1, color="red") +
#   geom_boxplot() + xlab("") + ylab("") +
#   geom_point() +
#   ylim(0,1) +
#   coord_flip() + theme_minimal() + ggtitle("False discovery rate")
# auc_plot <- ggplot(test$table, aes(x=fct_reorder(Method, -FPR), y=AUC)) +
#   geom_hline(yintercept=0.5, color="red") +
#   geom_boxplot() + xlab("") + ylab("") +
#   geom_point() +
#   ylim(0,1) +
#   coord_flip() + theme_minimal() + ggtitle("AUC")
# power_plot <- ggplot(test$table, aes(x=fct_reorder(Method, -FPR), y=Power)) +
#   geom_hline(yintercept=0.8, color="red") +
#   geom_boxplot() + xlab("") + ylab("") +
#   geom_point() +
#   ylim(0,1) +
#   coord_flip() + theme_minimal() + ggtitle("Power")
# cowplot::plot_grid(fpr_plot, fdr_plot, auc_plot, power_plot, nrow=2)
#
# pdf(paste("ADDPRO_DAtest2_Risk", ".pdf", sep=""), width=12, height=6)
# cowplot::plot_grid(fpr_plot, fdr_plot, auc_plot, power_plot, nrow=2)
# dev.off()
#
#
# ################Smoking
# #Remove NA
# Phe2<-Phe[complete.cases(Phe$Smoking),]
# #Also subset columns
# Microbio2<-dplyr::select(Microbio, one_of(Phe2$IDX))
#
#
# #runtimeDA(Microbio2, predictor = varvec)
# #Check order
# sum(colnames(Microbio2)!=Phe2$IDX)==0
#
# test <- testDA(Microbio2, predictor = Phe2$Smoking, R=20, tests=c("abc", "aov", "ds2", "erq", "vli",
```

```r
# summary(test)
# plot(test)
# pdf(paste("ADDPRO_DAtest1_Smoking", ".pdf", sep=""), width=12, height=6)
# plot(test)
# dev.off()
# test$run.times
#
#
#
# fpr_plot <- ggplot(test$table, aes(x=fct_reorder(Method, -FPR), y=FPR)) +
#   geom_hline(yintercept=0.05, color="red") +
#   geom_hline(yintercept=0.1, color="red") +
#   geom_boxplot() + xlab("") + ylab("") +
#   geom_point() +
#   ylim(0,1) +
#   coord_flip() + theme_minimal() + ggtitle("False positive rate")
# fdr_plot <- ggplot(test$table, aes(x=fct_reorder(Method, -FPR), y=FDR)) +
#   geom_hline(yintercept=0.05, color="red") +
#   geom_hline(yintercept=0.1, color="red") +
#   geom_boxplot() + xlab("") + ylab("") +
#   geom_point() +
#   ylim(0,1) +
#   coord_flip() + theme_minimal() + ggtitle("False discovery rate")
# auc_plot <- ggplot(test$table, aes(x=fct_reorder(Method, -FPR), y=AUC)) +
#   geom_hline(yintercept=0.5, color="red") +
#   geom_boxplot() + xlab("") + ylab("") +
#   geom_point() +
#   ylim(0,1) +
#   coord_flip() + theme_minimal() + ggtitle("AUC")
# power_plot <- ggplot(test$table, aes(x=fct_reorder(Method, -FPR), y=Power)) +
#   geom_hline(yintercept=0.8, color="red") +
#   geom_boxplot() + xlab("") + ylab("") +
#   geom_point() +
#   ylim(0,1) +
#   coord_flip() + theme_minimal() + ggtitle("Power")
# cowplot::plot_grid(fpr_plot, fdr_plot, auc_plot, power_plot, nrow=2)
#
# pdf(paste("ADDPRO_DAtest2_Smoking", ".pdf", sep=""), width=12, height=6)
# cowplot::plot_grid(fpr_plot, fdr_plot, auc_plot, power_plot, nrow=2)
# dev.off()


##Extract test for all variables
#################chol_cat
# res <- allDA(Microbio, predictor = Phe$chol_cat)
# vennDA(res, tests = c("aov", "kru"))

final <- DA.ds2(Microbio, predictor = Phe$chol_cat)
#kable(final[,1:10])
final
```

```
##        baseMean log2FoldChange      lfcSE        stat         pval ordering
## 1    65.8596889    0.315863862 0.26082233  1.21103073 2.258836e-01 Low>High
```

```
## 2   323.9127377   -0.089140992 0.08790900 -1.01401440 3.105759e-01 High>Low
## 3    22.0460481   -0.030074115 0.12546902 -0.23969355 8.105678e-01 High>Low
## 4   704.3072222    0.024613212 0.07232494  0.34031431 7.336198e-01 Low>High
## 5     5.4421107   -0.238579080 0.18073065 -1.32008091 1.868080e-01 High>Low
## 6  1939.2520922   -0.157409077 0.08454560 -1.86182453 6.262783e-02 High>Low
## 7    12.4599091    0.208916948 0.22272500  0.93800405 3.482423e-01 Low>High
## 8    11.4235529   -0.011490327 0.24576183 -0.04675391 9.627094e-01 High>Low
## 9     2.8038208   -0.077361131 0.33495708 -0.23095834 8.173472e-01 High>Low
## 10   12.5460084    0.137856776 0.21111046  0.65300780 5.137513e-01 Low>High
## 11   93.7260406   -0.027757619 0.09267017 -0.29953133 7.645347e-01 High>Low
## 12    1.5669010    0.083996117 0.20473471  0.41026808 6.816093e-01 Low>High
## 13  120.4914295   -0.010414568 0.08496657 -0.12257254 9.024456e-01 High>Low
## 14    1.3927613   -0.181435227 0.19685376 -0.92167518 3.566980e-01 High>Low
## 15    4.3017007    0.234411445 0.21037377  1.11426173 2.651669e-01 Low>High
## 16    6.5912121    0.033878967 0.22290131  0.15199088 8.791941e-01 Low>High
## 17    4.6746313    0.105921285 0.24298694  0.43591350 6.628995e-01 Low>High
## 18    0.8680201    0.731485097 0.34537073  2.11797071 3.417755e-02 Low>High
## 19    0.7921891   -0.227969095 0.33042909 -0.68991836 4.902455e-01 High>Low
## 20   12.1604713    0.158603105 0.12767785  1.24221317 2.141579e-01 Low>High
## 21  680.1285750   -0.146660006 0.11649702 -1.25891640 2.080605e-01 High>Low
## 22   22.0547383    0.069348125 0.08603010  0.80609140 4.201902e-01 Low>High
## 23  361.8645217    0.231853380 0.09209662  2.51750148 1.181905e-02 Low>High
## 24 3811.4256813   -0.020081238 0.05871713 -0.34199963 7.323512e-01 High>Low
## 25   72.5563323   -0.079723127 0.09066548 -0.87931070 3.792328e-01 High>Low
## 26  225.1152973   -0.025502316 0.09689024 -0.26320830 7.923900e-01 High>Low
## 27  309.0561286   -0.170552601 0.08074904 -2.11213155 3.467517e-02 High>Low
## 28   29.9768046   -0.142699544 0.17499893 -0.81543094 4.148257e-01 High>Low
## 29  306.2864969   -0.164380189 0.07040314 -2.33484166 1.955169e-02 High>Low
## 30   43.8440303   -1.619705234 0.29155727 -5.55535870 2.770422e-08 High>Low
## 31 3803.9265541   -0.194840251 0.06593274 -2.95513655 3.125307e-03 High>Low
## 32    0.2860988   -0.195063744 0.23910210 -0.81581780 4.146044e-01 High>Low
## 33    0.5750915   -0.080985697 0.19116350 -0.42364624 6.718238e-01 High>Low
## 34    1.0542291    0.407581182 0.28767915  1.41679083 1.565441e-01 Low>High
## 35    6.9441711    0.038587385 0.18480823  0.20879690 8.346068e-01 Low>High
## 36   36.8211521   -0.026182392 0.10420480 -0.25125897 8.016139e-01 High>Low
## 37    0.9388209    0.110227685 0.25105558  0.43905689 6.606203e-01 Low>High
## 38   87.7811267   -0.073695759 0.07283793 -1.01177723 3.116446e-01 High>Low
## 39   27.9872677   -0.190539534 0.19114597 -0.99682734 3.188483e-01 High>Low
## 40    2.8891780    0.461451577 0.19622972  2.35158862 1.869344e-02 Low>High
## 41    1.4688223    0.203358397 0.28329263  0.71783865 4.728568e-01 Low>High
## 42    1.4317578    0.090252198 0.30205376  0.29879515 7.650964e-01 Low>High
## 43  147.0483774   -0.055763508 0.08071686 -0.69085333 4.896577e-01 High>Low
## 44    2.8039987    0.010495227 0.22427994  0.04679521 9.626764e-01 Low>High
## 45  112.6769059   -0.241459146 0.09049549 -2.66818970 7.626119e-03 High>Low
## 46    1.5085797    0.199845262 0.25170205  0.79397550 4.272097e-01 Low>High
## 47    3.7020760    0.213523470 0.22498065  0.94907482 3.425826e-01 Low>High
## 48   40.8491501    0.008107239 0.11192546  0.07243427 9.422563e-01 Low>High
## 49    6.8375183    0.317617116 0.21800895  1.45689941 1.451442e-01 Low>High
## 50   41.0770621   -0.101741694 0.07738298 -1.31478133 1.885834e-01 High>Low
## 51   67.4763877   -0.101079173 0.13840141 -0.73033340 4.651864e-01 High>Low
## 52    0.4176611    0.385715860 0.29476667  1.30854640 1.906880e-01 Low>High
## 53    1.6627493    0.632146813 0.31090819  2.03322663 4.202963e-02 Low>High
## 54    1.0137786    0.275737961 0.30821367  0.89463250 3.709836e-01 Low>High
## 55    4.7083982    0.474907214 0.21400697  2.21912036 2.647854e-02 Low>High
```

```
## 56    8.7265107    0.133055956 0.12763699  1.04245609 2.972003e-01 Low>High
## 57   42.8338056    0.224939264 0.16140126  1.39366486 1.634188e-01 Low>High
## 58   25.1020535    0.152157135 0.16377250  0.92907623 3.528496e-01 Low>High
## 59   41.4593204   -0.011692463 0.13642418 -0.08570668 9.316996e-01 High>Low
## 60    3.8570393    0.160141201 0.24036668  0.66623711 5.052596e-01 Low>High
## 61    1.4537243    0.284868551 0.31597294  0.90155995 3.672907e-01 Low>High
## 62   74.0587622    0.045441898 0.09094740  0.49965033 6.173213e-01 Low>High
## 63    4.4957386   -0.166141128 0.19232428 -0.86385934 3.876652e-01 High>Low
## 64   26.8689347    0.279046018 0.17233298  1.61922582 1.053987e-01 Low>High
## 65  221.9840267    0.131275410 0.07742462  1.69552530 8.997581e-02 Low>High
## 66    5.0346400    0.276656191 0.20222689  1.36804852 1.712969e-01 Low>High
## 67    0.7371549    0.087772716 0.18508624  0.47422604 6.353387e-01 Low>High
## 68    8.7009758   -0.418461743 0.18556378 -2.25508305 2.412812e-02 High>Low
## 69   36.4394005    0.007146658 0.09535820  0.07494540 9.402582e-01 Low>High
## 70  407.9853210   -0.116497334 0.10030732 -1.16140411 2.454776e-01 High>Low
## 71    0.5266292   -0.066723682 0.21887745 -0.30484494 7.604843e-01 High>Low
## 72 5067.7924258   -0.105791636 0.05637168 -1.87668066 6.056188e-02 High>Low
## 73    6.1442803    0.085901670 0.15682625  0.54775057 5.838632e-01 Low>High
## 74    0.8912626   -0.317581238 0.26038246 -1.21967214 2.225892e-01 High>Low
## 75  927.1066406   -0.018730051 0.07509454 -0.24941962 8.030362e-01 High>Low
## 76  683.2517404   -0.034819718 0.07746809 -0.44947175 6.530914e-01 High>Low
## 77    2.4068360    0.057751928 0.25039440  0.23064385 8.175915e-01 Low>High
## 78   68.4201506   -0.013245569 0.14419878 -0.09185632 9.268122e-01 High>Low
## 79    2.0156485   -0.145509435 0.31466418 -0.46242771 6.437746e-01 High>Low
## 80    8.7736001    0.110240698 0.12032107  0.91622105 3.595510e-01 Low>High
## 81   99.8204227   -0.037652106 0.10377106 -0.36283821 7.167257e-01 High>Low
## 82 1329.0853342    0.051576371 0.11859204  0.43490584 6.636308e-01 Low>High
## 83    5.6824349   -0.440563890 0.27225833 -1.61818332 1.056231e-01 High>Low
## 84  257.8128378    0.045737247 0.06645702  0.68822295 4.913124e-01 Low>High
## 85   14.7982481   -0.020784799 0.10378045 -0.20027664 8.412642e-01 High>Low
## 86  275.5218474   -0.009596881 0.13680532 -0.07014992 9.440743e-01 High>Low
## 87 2905.9316825   -0.125111885 0.09126144 -1.37091731 1.704007e-01 High>Low
## 88    2.3978345    0.662288914 0.36999664  1.78998630 7.345611e-02 Low>High
## 89   36.0392035    0.415148592 0.15181463  2.73457559 6.246075e-03 Low>High
## 90    7.1137273   -0.139661476 0.21251399 -0.65718722 5.110605e-01 High>Low
## 91    7.2376188   -0.089043346 0.27059462 -0.32906547 7.421062e-01 High>Low
## 92    1.1003857    0.096343523 0.33586833  0.28684909 7.742279e-01 Low>High
## 93    0.9162822    0.154947938 0.31669200  0.48927013 6.246505e-01 Low>High
## 94   63.1946030    0.284583492 0.14416200  1.97405338 4.837567e-02 Low>High
## 95    8.2679474    0.215545290 0.17477010  1.23330757 2.174610e-01 Low>High
## 96    9.3689884    0.288947109 0.22101309  1.30737554 1.910852e-01 Low>High
## 97   33.1524239    0.328441995 0.14681953  2.23704567 2.528336e-02 Low>High
##        pval.adj                         Feature                    Method
## 1  6.444327e-01     Absconditabacteria_(SR1)_[G.1] DESeq2 man. geoMeans (ds2)
## 2  7.656470e-01                    Actinomyces DESeq2 man. geoMeans (ds2)
## 3  9.221672e-01                   Peptidiphaga DESeq2 man. geoMeans (ds2)
## 4  9.221672e-01                       Schaalia DESeq2 man. geoMeans (ds2)
## 5  6.391471e-01        Family.Actinomycetaceae. DESeq2 man. geoMeans (ds2)
## 6  3.796812e-01                        Rothia DESeq2 man. geoMeans (ds2)
## 7  7.656470e-01                  Alloscardovia DESeq2 man. geoMeans (ds2)
## 8  9.627094e-01                Bifidobacterium DESeq2 man. geoMeans (ds2)
## 9  9.221672e-01                  Parascardovia DESeq2 man. geoMeans (ds2)
## 10 8.169487e-01                      Scardovia DESeq2 man. geoMeans (ds2)
## 11 9.221672e-01                Corynebacterium DESeq2 man. geoMeans (ds2)
```

```
## 12 9.182792e-01                          Olsenella DESeq2 man. geoMeans (ds2)
## 13 9.627094e-01                          Atopobium DESeq2 man. geoMeans (ds2)
## 14 7.656470e-01                            Slackia DESeq2 man. geoMeans (ds2)
## 15 7.144775e-01                     Cryptobacterium DESeq2 man. geoMeans (ds2)
## 16 9.582228e-01                 Bacteroidetes_[G.3] DESeq2 man. geoMeans (ds2)
## 17 9.178438e-01                 Bacteroidetes_[G.5] DESeq2 man. geoMeans (ds2)
## 18 2.802909e-01         Family.Bacteroidetes_[F-1]. DESeq2 man. geoMeans (ds2)
## 19 8.169487e-01                Bacteroidaceae_[G.1] DESeq2 man. geoMeans (ds2)
## 20 6.444327e-01                 Bacteroidales_[G.2] DESeq2 man. geoMeans (ds2)
## 21 6.444327e-01                       Porphyromonas DESeq2 man. geoMeans (ds2)
## 22 7.818743e-01                         Tannerella DESeq2 man. geoMeans (ds2)
## 23 2.292895e-01                      Alloprevotella DESeq2 man. geoMeans (ds2)
## 24 9.221672e-01                          Prevotella DESeq2 man. geoMeans (ds2)
## 25 7.663664e-01                          Bergeyella DESeq2 man. geoMeans (ds2)
## 26 9.221672e-01                      Capnocytophaga DESeq2 man. geoMeans (ds2)
## 27 2.802909e-01                            Gemella DESeq2 man. geoMeans (ds2)
## 28 7.818743e-01                         Abiotrophia DESeq2 man. geoMeans (ds2)
## 29 2.568418e-01                      Granulicatella DESeq2 man. geoMeans (ds2)
## 30 2.687310e-06                       Lactobacillus DESeq2 man. geoMeans (ds2)
## 31 1.515774e-01                       Streptococcus DESeq2 man. geoMeans (ds2)
## 32 7.818743e-01               Order.Lactobacillales. DESeq2 man. geoMeans (ds2)
## 33 9.178438e-01                      Class.Bacilli. DESeq2 man. geoMeans (ds2)
## 34 6.391471e-01            Clostridiales_[F.1][G-1] DESeq2 man. geoMeans (ds2)
## 35 9.273026e-01                        Butyrivibrio DESeq2 man. geoMeans (ds2)
## 36 9.221672e-01                          Catonella DESeq2 man. geoMeans (ds2)
## 37 9.178438e-01                         Johnsonella DESeq2 man. geoMeans (ds2)
## 38 7.656470e-01                 Lachnoanaerobaculum DESeq2 man. geoMeans (ds2)
## 39 7.656470e-01               Lachnospiraceae_[G.2] DESeq2 man. geoMeans (ds2)
## 40 2.568418e-01               Lachnospiraceae_[G.3] DESeq2 man. geoMeans (ds2)
## 41 8.169487e-01               Lachnospiraceae_[G.7] DESeq2 man. geoMeans (ds2)
## 42 9.221672e-01               Lachnospiraceae_[G.8] DESeq2 man. geoMeans (ds2)
## 43 8.169487e-01                        Oribacterium DESeq2 man. geoMeans (ds2)
## 44 9.627094e-01                      Shuttleworthia DESeq2 man. geoMeans (ds2)
## 45 1.849334e-01                      Stomatobaculum DESeq2 man. geoMeans (ds2)
## 46 7.818743e-01       Family.Lachnospiraceae_[XIV]. DESeq2 man. geoMeans (ds2)
## 47 7.656470e-01                         Peptococcus DESeq2 man. geoMeans (ds2)
## 48 9.627094e-01                          Parvimonas DESeq2 man. geoMeans (ds2)
## 49 6.391471e-01                          Filifactor DESeq2 man. geoMeans (ds2)
## 50 6.391471e-01                        Mogibacterium DESeq2 man. geoMeans (ds2)
## 51 8.169487e-01   Peptostreptococcaceae_[XI][G.1] DESeq2 man. geoMeans (ds2)
## 52 6.391471e-01   Peptostreptococcaceae_[XI][G.4] DESeq2 man. geoMeans (ds2)
## 53 3.136057e-01   Peptostreptococcaceae_[XI][G.5] DESeq2 man. geoMeans (ds2)
## 54 7.656470e-01   Peptostreptococcaceae_[XI][G.6] DESeq2 man. geoMeans (ds2)
## 55 2.568418e-01   Peptostreptococcaceae_[XI][G.7] DESeq2 man. geoMeans (ds2)
## 56 7.656470e-01   Peptostreptococcaceae_[XI][G.9] DESeq2 man. geoMeans (ds2)
## 57 6.391471e-01                 Peptostreptococcus DESeq2 man. geoMeans (ds2)
## 58 7.656470e-01               Ruminococcaceae_[G.1] DESeq2 man. geoMeans (ds2)
## 59 9.627094e-01               Ruminococcaceae_[G.2] DESeq2 man. geoMeans (ds2)
## 60 8.169487e-01                          Bulleidia DESeq2 man. geoMeans (ds2)
## 61 7.656470e-01                          Eggerthia DESeq2 man. geoMeans (ds2)
## 62 9.178438e-01                       Solobacterium DESeq2 man. geoMeans (ds2)
## 63 7.674188e-01                          Mycoplasma DESeq2 man. geoMeans (ds2)
## 64 5.122720e-01                         Mitsuokella DESeq2 man. geoMeans (ds2)
## 65 4.848697e-01                         Selenomonas DESeq2 man. geoMeans (ds2)
```

```
## 66 6.391471e-01             Family.Selenomonadaceae. DESeq2 man. geoMeans (ds2)
## 67 9.178438e-01                 Class.Negativicutes. DESeq2 man. geoMeans (ds2)
## 68 2.568418e-01                         Anaeroglobus DESeq2 man. geoMeans (ds2)
## 69 9.627094e-01                            Dialister DESeq2 man. geoMeans (ds2)
## 70 6.803236e-01                          Megasphaera DESeq2 man. geoMeans (ds2)
## 71 9.221672e-01              Family.Veillonellaceae. DESeq2 man. geoMeans (ds2)
## 72 3.796812e-01                          Veillonella DESeq2 man. geoMeans (ds2)
## 73 9.134634e-01                Veillonellaceae_[G.1] DESeq2 man. geoMeans (ds2)
## 74 6.444327e-01                   Phylum.Firmicutes. DESeq2 man. geoMeans (ds2)
## 75 9.221672e-01                         Fusobacterium DESeq2 man. geoMeans (ds2)
## 76 9.178438e-01                         Leptotrichia DESeq2 man. geoMeans (ds2)
## 77 9.221672e-01          Gracilibacteria_(GN02)_[G.1] DESeq2 man. geoMeans (ds2)
## 78 9.627094e-01                            Lautropia DESeq2 man. geoMeans (ds2)
## 79 9.178438e-01                             Ottowia DESeq2 man. geoMeans (ds2)
## 80 7.656470e-01                            Eikenella DESeq2 man. geoMeans (ds2)
## 81 9.221672e-01                             Kingella DESeq2 man. geoMeans (ds2)
## 82 9.178438e-01                            Neisseria DESeq2 man. geoMeans (ds2)
## 83 5.122720e-01               Family.Neisseriaceae. DESeq2 man. geoMeans (ds2)
## 84 8.169487e-01                        Campylobacter DESeq2 man. geoMeans (ds2)
## 85 9.273026e-01                      Cardiobacterium DESeq2 man. geoMeans (ds2)
## 86 9.627094e-01                      Aggregatibacter DESeq2 man. geoMeans (ds2)
## 87 6.391471e-01                          Haemophilus DESeq2 man. geoMeans (ds2)
## 88 4.191319e-01             Family.Pasteurellaceae. DESeq2 man. geoMeans (ds2)
## 89 1.849334e-01          Saccharibacteria_(TM7)_[G.1] DESeq2 man. geoMeans (ds2)
## 90 8.169487e-01          Saccharibacteria_(TM7)_[G.3] DESeq2 man. geoMeans (ds2)
## 91 9.221672e-01          Saccharibacteria_(TM7)_[G.6] DESeq2 man. geoMeans (ds2)
## 92 9.221672e-01 Family.Saccharibacteria_(TM7)_[F-1]. DESeq2 man. geoMeans (ds2)
## 93 9.178438e-01          Saccharibacteria_(TM7)_[G.5] DESeq2 man. geoMeans (ds2)
## 94 3.351743e-01                            Treponema DESeq2 man. geoMeans (ds2)
## 95 6.444327e-01                       Fretibacterium DESeq2 man. geoMeans (ds2)
## 96 6.391471e-01                    Kingdom.Bacteria. DESeq2 man. geoMeans (ds2)
## 97 2.568418e-01                               Others DESeq2 man. geoMeans (ds2)
```

```
write.table(final, file="chol_cat_ds2.txt", sep="\t", dec=",", row.names=F)
write.table(final[final$pval<0.05,], file="chol_cat_ds2_sig.txt", sep="\t", dec=",", row.names=F)
final <- DA.kru(Microbio, predictor = Phe$chol_cat)
#kable(final[,1:10])
final
```

```
##                                             pval  pval.adj
## Absconditabacteria_(SR1)_[G.1]        0.41371702 0.8488822
## Actinomyces                           0.30503729 0.7996924
## Peptidiphaga                          0.98751425 0.9998566
## Schaalia                              0.15629913 0.6593235
## Family.Actinomycetaceae.              0.37693118 0.8124961
## Rothia                                0.99985655 0.9998566
## Alloscardovia                         0.24172440 0.7563634
## Bifidobacterium                       0.03188351 0.5243064
## Parascardovia                         0.72231278 0.9218992
## Scardovia                             0.63899951 0.9040208
## Corynebacterium                       0.29340391 0.7995022
## Olsenella                             0.65579485 0.9040208
## Atopobium                             0.37281681 0.8124961
## Slackia                               0.89803314 0.9998566
```

```
## Cryptobacterium                     0.32323907 0.8124961
## Bacteroidetes_[G.3]                 0.99609249 0.9998566
## Bacteroidetes_[G.5]                 0.70604204 0.9131477
## Family.Bacteroidetes_[F-1].         0.10882232 0.6396312
## Bacteroidaceae_[G.1]                0.19611723 0.7045693
## Bacteroidales_[G.2]                 0.40945027 0.8488822
## Porphyromonas                       0.99813515 0.9998566
## Tannerella                          0.42983721 0.8488822
## Alloprevotella                      0.09612466 0.6396312
## Prevotella                          0.68966532 0.9040208
## Bergeyella                          0.93337314 0.9998566
## Capnocytophaga                      0.94653312 0.9998566
## Gemella                             0.20840002 0.7190577
## Abiotrophia                         0.61819358 0.9040208
## Granulicatella                      0.58381943 0.9040208
## Lactobacillus                       0.17300129 0.6712450
## Streptococcus                       0.93480292 0.9998566
## Order.Lactobacillales.              0.10550880 0.6396312
## Class.Bacilli.                      0.95662755 0.9998566
## Clostridiales_[F.1][G-1]            0.05951576 0.5438280
## Butyrivibrio                        0.34470503 0.8124961
## Catonella                           0.59752858 0.9040208
## Johnsonella                         0.88219156 0.9998566
## Lachnoanaerobaculum                 0.53200691 0.9040208
## Lachnospiraceae_[G.2]               0.63392751 0.9040208
## Lachnospiraceae_[G.3]               0.35772112 0.8124961
## Lachnospiraceae_[G.7]               0.04582212 0.5438280
## Lachnospiraceae_[G.8]               0.15679790 0.6593235
## Oribacterium                        0.76371508 0.9509806
## Shuttleworthia                      0.63157879 0.9040208
## Stomatobaculum                      0.03123662 0.5243064
## Family.Lachnospiraceae_[XIV].       0.68359493 0.9040208
## Peptococcus                         0.57547873 0.9040208
## Parvimonas                          0.95985008 0.9998566
## Filifactor                          0.03783654 0.5243064
## Mogibacterium                       0.49323492 0.9040208
## Peptostreptococcaceae_[XI][G.1]     0.54970158 0.9040208
## Peptostreptococcaceae_[XI][G.4]     0.12058556 0.6498222
## Peptostreptococcaceae_[XI][G.5]     0.06167122 0.5438280
## Peptostreptococcaceae_[XI][G.6]     0.11210032 0.6396312
## Peptostreptococcaceae_[XI][G.7]     0.01051457 0.5243064
## Peptostreptococcaceae_[XI][G.9]     0.46452874 0.8835154
## Peptostreptococcus                  0.08566732 0.6396312
## Ruminococcaceae_[G.1]               0.23227030 0.7510073
## Ruminococcaceae_[G.2]               0.43756815 0.8488822
## Bulleidia                           0.55821754 0.9040208
## Eggerthia                           0.58221931 0.9040208
## Solobacterium                       0.34293304 0.8124961
## Mycoplasma                          0.67549116 0.9040208
## Mitsuokella                         0.03360585 0.5243064
## Selenomonas                         0.01631512 0.5243064
## Family.Selenomonadaceae.            0.28643382 0.7995022
## Class.Negativicutes.                0.18455926 0.6885480
## Anaeroglobus                        0.61966259 0.9040208
```

```
## Dialister                              0.29431319 0.7995022
## Megasphaera                            0.55575023 0.9040208
## Family.Veillonellaceae.                0.97709474 0.9998566
## Veillonella                            0.96085383 0.9998566
## Veillonellaceae_[G.1]                  0.14362819 0.6593235
## Phylum.Firmicutes.                     0.52119210 0.9040208
## Fusobacterium                          0.86282724 0.9998566
## Leptotrichia                           0.95168687 0.9998566
## Gracilibacteria_(GN02)_[G.1]           0.60292212 0.9040208
## Lautropia                              0.57581200 0.9040208
## Ottowia                                0.85558833 0.9998566
## Eikenella                              0.68196307 0.9040208
## Kingella                               0.67659818 0.9040208
## Neisseria                              0.16313159 0.6593235
## Family.Neisseriaceae.                  0.15440010 0.6593235
## Campylobacter                          0.21497602 0.7190577
## Cardiobacterium                        0.77450998 0.9509806
## Aggregatibacter                        0.77261540 0.9509806
## Haemophilus                            0.33965132 0.8124961
## Family.Pasteurellaceae.                0.27824877 0.7995022
## Saccharibacteria_(TM7)_[G.1]           0.14346263 0.6593235
## Saccharibacteria_(TM7)_[G.3]           0.36336748 0.8124961
## Saccharibacteria_(TM7)_[G.6]           0.99298598 0.9998566
## Family.Saccharibacteria_(TM7)_[F-1]. 0.09271670 0.6396312
## Saccharibacteria_(TM7)_[G.5]           0.91465465 0.9998566
## Treponema                              0.06018042 0.5438280
## Fretibacterium                         0.29672246 0.7995022
## Kingdom.Bacteria.                      0.43399780 0.8488822
## Others                                 0.02398808 0.5243064
##                                                                 Feature
## Absconditabacteria_(SR1)_[G.1]             Absconditabacteria_(SR1)_[G.1]
## Actinomyces                                                  Actinomyces
## Peptidiphaga                                                Peptidiphaga
## Schaalia                                                        Schaalia
## Family.Actinomycetaceae.                        Family.Actinomycetaceae.
## Rothia                                                            Rothia
## Alloscardovia                                              Alloscardovia
## Bifidobacterium                                          Bifidobacterium
## Parascardovia                                              Parascardovia
## Scardovia                                                      Scardovia
## Corynebacterium                                          Corynebacterium
## Olsenella                                                      Olsenella
## Atopobium                                                      Atopobium
## Slackia                                                          Slackia
## Cryptobacterium                                          Cryptobacterium
## Bacteroidetes_[G.3]                                  Bacteroidetes_[G.3]
## Bacteroidetes_[G.5]                                  Bacteroidetes_[G.5]
## Family.Bacteroidetes_[F-1].                  Family.Bacteroidetes_[F-1].
## Bacteroidaceae_[G.1]                                Bacteroidaceae_[G.1]
## Bacteroidales_[G.2]                                  Bacteroidales_[G.2]
## Porphyromonas                                              Porphyromonas
## Tannerella                                                    Tannerella
## Alloprevotella                                            Alloprevotella
## Prevotella                                                    Prevotella
```

```
## Bergeyella                                                       Bergeyella
## Capnocytophaga                                               Capnocytophaga
## Gemella                                                             Gemella
## Abiotrophia                                                     Abiotrophia
## Granulicatella                                               Granulicatella
## Lactobacillus                                                 Lactobacillus
## Streptococcus                                                 Streptococcus
## Order.Lactobacillales.                               Order.Lactobacillales.
## Class.Bacilli.                                               Class.Bacilli.
## Clostridiales_[F.1][G-1]                           Clostridiales_[F.1][G-1]
## Butyrivibrio                                                   Butyrivibrio
## Catonella                                                         Catonella
## Johnsonella                                                     Johnsonella
## Lachnoanaerobaculum                                     Lachnoanaerobaculum
## Lachnospiraceae_[G.2]                                 Lachnospiraceae_[G.2]
## Lachnospiraceae_[G.3]                                 Lachnospiraceae_[G.3]
## Lachnospiraceae_[G.7]                                 Lachnospiraceae_[G.7]
## Lachnospiraceae_[G.8]                                 Lachnospiraceae_[G.8]
## Oribacterium                                                   Oribacterium
## Shuttleworthia                                               Shuttleworthia
## Stomatobaculum                                               Stomatobaculum
## Family.Lachnospiraceae_[XIV].                 Family.Lachnospiraceae_[XIV].
## Peptococcus                                                     Peptococcus
## Parvimonas                                                       Parvimonas
## Filifactor                                                       Filifactor
## Mogibacterium                                                 Mogibacterium
## Peptostreptococcaceae_[XI][G.1]             Peptostreptococcaceae_[XI][G.1]
## Peptostreptococcaceae_[XI][G.4]             Peptostreptococcaceae_[XI][G.4]
## Peptostreptococcaceae_[XI][G.5]             Peptostreptococcaceae_[XI][G.5]
## Peptostreptococcaceae_[XI][G.6]             Peptostreptococcaceae_[XI][G.6]
## Peptostreptococcaceae_[XI][G.7]             Peptostreptococcaceae_[XI][G.7]
## Peptostreptococcaceae_[XI][G.9]             Peptostreptococcaceae_[XI][G.9]
## Peptostreptococcus                                       Peptostreptococcus
## Ruminococcaceae_[G.1]                                 Ruminococcaceae_[G.1]
## Ruminococcaceae_[G.2]                                 Ruminococcaceae_[G.2]
## Bulleidia                                                         Bulleidia
## Eggerthia                                                         Eggerthia
## Solobacterium                                                 Solobacterium
## Mycoplasma                                                       Mycoplasma
## Mitsuokella                                                     Mitsuokella
## Selenomonas                                                     Selenomonas
## Family.Selenomonadaceae.                           Family.Selenomonadaceae.
## Class.Negativicutes.                                   Class.Negativicutes.
## Anaeroglobus                                                   Anaeroglobus
## Dialister                                                         Dialister
## Megasphaera                                                     Megasphaera
## Family.Veillonellaceae.                             Family.Veillonellaceae.
## Veillonella                                                     Veillonella
## Veillonellaceae_[G.1]                                 Veillonellaceae_[G.1]
## Phylum.Firmicutes.                                       Phylum.Firmicutes.
## Fusobacterium                                                 Fusobacterium
## Leptotrichia                                                   Leptotrichia
## Gracilibacteria_(GN02)_[G.1]                   Gracilibacteria_(GN02)_[G.1]
## Lautropia                                                         Lautropia
```

```
## Ottowia                                                              Ottowia
## Eikenella                                                          Eikenella
## Kingella                                                            Kingella
## Neisseria                                                          Neisseria
## Family.Neisseriaceae.                                  Family.Neisseriaceae.
## Campylobacter                                                  Campylobacter
## Cardiobacterium                                              Cardiobacterium
## Aggregatibacter                                              Aggregatibacter
## Haemophilus                                                      Haemophilus
## Family.Pasteurellaceae.                              Family.Pasteurellaceae.
## Saccharibacteria_(TM7)_[G.1]                    Saccharibacteria_(TM7)_[G.1]
## Saccharibacteria_(TM7)_[G.3]                    Saccharibacteria_(TM7)_[G.3]
## Saccharibacteria_(TM7)_[G.6]                    Saccharibacteria_(TM7)_[G.6]
## Family.Saccharibacteria_(TM7)_[F-1]. Family.Saccharibacteria_(TM7)_[F-1].
## Saccharibacteria_(TM7)_[G.5]                    Saccharibacteria_(TM7)_[G.5]
## Treponema                                                          Treponema
## Fretibacterium                                                Fretibacterium
## Kingdom.Bacteria.                                          Kingdom.Bacteria.
## Others                                                                Others
##                                                  Method
## Absconditabacteria_(SR1)_[G.1]       Kruskal-Wallis (kru)
## Actinomyces                          Kruskal-Wallis (kru)
## Peptidiphaga                         Kruskal-Wallis (kru)
## Schaalia                             Kruskal-Wallis (kru)
## Family.Actinomycetaceae.             Kruskal-Wallis (kru)
## Rothia                               Kruskal-Wallis (kru)
## Alloscardovia                        Kruskal-Wallis (kru)
## Bifidobacterium                      Kruskal-Wallis (kru)
## Parascardovia                        Kruskal-Wallis (kru)
## Scardovia                            Kruskal-Wallis (kru)
## Corynebacterium                      Kruskal-Wallis (kru)
## Olsenella                            Kruskal-Wallis (kru)
## Atopobium                            Kruskal-Wallis (kru)
## Slackia                              Kruskal-Wallis (kru)
## Cryptobacterium                      Kruskal-Wallis (kru)
## Bacteroidetes_[G.3]                  Kruskal-Wallis (kru)
## Bacteroidetes_[G.5]                  Kruskal-Wallis (kru)
## Family.Bacteroidetes_[F-1].          Kruskal-Wallis (kru)
## Bacteroidaceae_[G.1]                 Kruskal-Wallis (kru)
## Bacteroidales_[G.2]                  Kruskal-Wallis (kru)
## Porphyromonas                        Kruskal-Wallis (kru)
## Tannerella                           Kruskal-Wallis (kru)
## Alloprevotella                       Kruskal-Wallis (kru)
## Prevotella                           Kruskal-Wallis (kru)
## Bergeyella                           Kruskal-Wallis (kru)
## Capnocytophaga                       Kruskal-Wallis (kru)
## Gemella                              Kruskal-Wallis (kru)
## Abiotrophia                          Kruskal-Wallis (kru)
## Granulicatella                       Kruskal-Wallis (kru)
## Lactobacillus                        Kruskal-Wallis (kru)
## Streptococcus                        Kruskal-Wallis (kru)
## Order.Lactobacillales.               Kruskal-Wallis (kru)
## Class.Bacilli.                       Kruskal-Wallis (kru)
## Clostridiales_[F.1][G-1]             Kruskal-Wallis (kru)
```

```
## Butyrivibrio                          Kruskal-Wallis (kru)
## Catonella                             Kruskal-Wallis (kru)
## Johnsonella                           Kruskal-Wallis (kru)
## Lachnoanaerobaculum                   Kruskal-Wallis (kru)
## Lachnospiraceae_[G.2]                 Kruskal-Wallis (kru)
## Lachnospiraceae_[G.3]                 Kruskal-Wallis (kru)
## Lachnospiraceae_[G.7]                 Kruskal-Wallis (kru)
## Lachnospiraceae_[G.8]                 Kruskal-Wallis (kru)
## Oribacterium                          Kruskal-Wallis (kru)
## Shuttleworthia                        Kruskal-Wallis (kru)
## Stomatobaculum                        Kruskal-Wallis (kru)
## Family.Lachnospiraceae_[XIV].         Kruskal-Wallis (kru)
## Peptococcus                           Kruskal-Wallis (kru)
## Parvimonas                            Kruskal-Wallis (kru)
## Filifactor                            Kruskal-Wallis (kru)
## Mogibacterium                         Kruskal-Wallis (kru)
## Peptostreptococcaceae_[XI][G.1]       Kruskal-Wallis (kru)
## Peptostreptococcaceae_[XI][G.4]       Kruskal-Wallis (kru)
## Peptostreptococcaceae_[XI][G.5]       Kruskal-Wallis (kru)
## Peptostreptococcaceae_[XI][G.6]       Kruskal-Wallis (kru)
## Peptostreptococcaceae_[XI][G.7]       Kruskal-Wallis (kru)
## Peptostreptococcaceae_[XI][G.9]       Kruskal-Wallis (kru)
## Peptostreptococcus                    Kruskal-Wallis (kru)
## Ruminococcaceae_[G.1]                 Kruskal-Wallis (kru)
## Ruminococcaceae_[G.2]                 Kruskal-Wallis (kru)
## Bulleidia                             Kruskal-Wallis (kru)
## Eggerthia                             Kruskal-Wallis (kru)
## Solobacterium                         Kruskal-Wallis (kru)
## Mycoplasma                            Kruskal-Wallis (kru)
## Mitsuokella                           Kruskal-Wallis (kru)
## Selenomonas                           Kruskal-Wallis (kru)
## Family.Selenomonadaceae.              Kruskal-Wallis (kru)
## Class.Negativicutes.                  Kruskal-Wallis (kru)
## Anaeroglobus                          Kruskal-Wallis (kru)
## Dialister                             Kruskal-Wallis (kru)
## Megasphaera                           Kruskal-Wallis (kru)
## Family.Veillonellaceae.               Kruskal-Wallis (kru)
## Veillonella                           Kruskal-Wallis (kru)
## Veillonellaceae_[G.1]                 Kruskal-Wallis (kru)
## Phylum.Firmicutes.                    Kruskal-Wallis (kru)
## Fusobacterium                         Kruskal-Wallis (kru)
## Leptotrichia                          Kruskal-Wallis (kru)
## Gracilibacteria_(GN02)_[G.1]          Kruskal-Wallis (kru)
## Lautropia                             Kruskal-Wallis (kru)
## Ottowia                               Kruskal-Wallis (kru)
## Eikenella                             Kruskal-Wallis (kru)
## Kingella                              Kruskal-Wallis (kru)
## Neisseria                             Kruskal-Wallis (kru)
## Family.Neisseriaceae.                 Kruskal-Wallis (kru)
## Campylobacter                         Kruskal-Wallis (kru)
## Cardiobacterium                       Kruskal-Wallis (kru)
## Aggregatibacter                       Kruskal-Wallis (kru)
## Haemophilus                           Kruskal-Wallis (kru)
## Family.Pasteurellaceae.               Kruskal-Wallis (kru)
```

```
## Saccharibacteria_(TM7)_[G.1]         Kruskal-Wallis (kru)
## Saccharibacteria_(TM7)_[G.3]         Kruskal-Wallis (kru)
## Saccharibacteria_(TM7)_[G.6]         Kruskal-Wallis (kru)
## Family.Saccharibacteria_(TM7)_[F-1]. Kruskal-Wallis (kru)
## Saccharibacteria_(TM7)_[G.5]         Kruskal-Wallis (kru)
## Treponema                            Kruskal-Wallis (kru)
## Fretibacterium                       Kruskal-Wallis (kru)
## Kingdom.Bacteria.                    Kruskal-Wallis (kru)
## Others                               Kruskal-Wallis (kru)
```

```
write.table(final, file="chol_cat_kru.txt", sep="\t", dec=",", row.names=F)
write.table(final[final$pval<0.05,], file="chol_cat_kru_sig.txt", sep="\t", dec=",", row.names=F)
##################Risk
#Only includes rows high vs low
Phe2<-subset(Phe, Risk %in% c("High", "Low"))
Phe2$Risk<-droplevels(Phe2$Risk)
#Also subset columns
Microbio2<-dplyr::select(Microbio, one_of(Phe2$IDX))
final <- DA.ds2(Microbio2, predictor = Phe2$Risk)
#kable(final[,1:10])
final
```

```
##        baseMean log2FoldChange      lfcSE        stat        pval ordering
## 1    64.2597725   -0.485012785 0.25354379 -1.91293497 0.055756379 Low>High
## 2   324.2145221    0.143585352 0.08623781  1.66499294 0.095914165 High>Low
## 3    21.8070254   -0.005774917 0.12137256 -0.04758009 0.962050899 Low>High
## 4   700.4515512    0.112284878 0.07068671  1.58848639 0.112176397 High>Low
## 5     5.3549126    0.016368699 0.17792680  0.09199681 0.926700571 High>Low
## 6  1945.8951087    0.070731122 0.08335094  0.84859421 0.396107128 High>Low
## 7    12.3058511   -0.055106943 0.21660599 -0.25441098 0.799178066 Low>High
## 8    10.8994425    0.212573792 0.23644988  0.89902262 0.368640611 High>Low
## 9     2.9990083    0.612204622 0.32700349  1.87216542 0.061183722 High>Low
## 10   12.4584166    0.179737981 0.20441411  0.87928361 0.379247519 High>Low
## 11   93.4333155    0.258361848 0.09048573  2.85527719 0.004299928 High>Low
## 12    1.5645842    0.177095244 0.19829331  0.89309740 0.371805015 High>Low
## 13  120.7894166    0.072246312 0.08291053  0.87137684 0.383548427 High>Low
## 14    1.3952328    0.061681015 0.19380196  0.31826827 0.750281454 High>Low
## 15    4.2229867    0.303265519 0.20276486  1.49565125 0.134744560 High>Low
## 16    6.4900827    0.056950958 0.21547176  0.26430822 0.791542425 High>Low
## 17    4.5443282    0.072796297 0.23767260  0.30628814 0.759385272 High>Low
## 18    0.8440599   -0.895407727 0.34399572 -2.60296180 0.009242225 Low>High
## 19    0.7927548    0.952933928 0.32006542  2.97730986 0.002907900 High>Low
## 20   12.0958316    0.159112539 0.12353105  1.28803683 0.197733142 High>Low
## 21  674.8647664   -0.145170610 0.11343783 -1.27973724 0.200637562 Low>High
## 22   21.8833160   -0.023858954 0.08379512 -0.28472963 0.775851290 Low>High
## 23  363.2895934   -0.038962557 0.08999779 -0.43292791 0.665067141 Low>High
## 24 3825.2557819   -0.045549468 0.05731141 -0.79477135 0.426746518 Low>High
## 25   72.7009790   -0.158351959 0.08806901 -1.79804405 0.072170027 Low>High
## 26  225.1643864   -0.089866849 0.09421104 -0.95388877 0.340139948 Low>High
## 27  308.9669193    0.045173007 0.07888726  0.57262743 0.566896985 High>Low
## 28   30.0443330   -0.086069013 0.16967186 -0.50726746 0.611967167 Low>High
## 29  306.4850456    0.015954485 0.06817115  0.23403573 0.814957237 High>Low
## 30   19.9090913    0.007959283 0.25306762  0.03145121 0.974909700 High>Low
## 31 3824.6130729   -0.034200749 0.06491855 -0.52682556 0.598314730 Low>High
```

```
## 32    0.2873798   -0.135909605 0.23674674 -0.57407171 0.565919273 Low>High
## 33    0.5789536    0.231176698 0.18503270  1.24938292 0.211525052 High>Low
## 34    1.0076262    0.016742894 0.28287047  0.05918926 0.952801364 High>Low
## 35    6.9558967   -0.545300320 0.18075265 -3.01683162 0.002554317 Low>High
## 36   36.5379681   -0.076218612 0.10201485 -0.74713250 0.454983584 Low>High
## 37    0.8914323    0.215322758 0.24768818  0.86932996 0.384666679 High>Low
## 38   87.7547810   -0.017745418 0.07097842 -0.25001145 0.802578495 Low>High
## 39   28.1084549   -0.544924513 0.18688359 -2.91584999 0.003547210 Low>High
## 40    2.8328544    0.012410576 0.19313434  0.06425877 0.948764179 High>Low
## 41    1.4579041    0.267475646 0.27766211  0.96331346 0.335390241 High>Low
## 42    1.3988933   -0.164326286 0.30165710 -0.54474530 0.585928705 Low>High
## 43  146.7103250   -0.077861003 0.07915297 -0.98367762 0.325274048 Low>High
## 44    2.8423211    0.243737097 0.21581038  1.12940394 0.258727472 High>Low
## 45  112.6897043   -0.072071900 0.08890900 -0.81062552 0.417580757 Low>High
## 46    1.3131169   -0.481071361 0.23769785 -2.02387767 0.042982740 Low>High
## 47    3.6055204   -0.018871964 0.22135627 -0.08525606 0.932057821 Low>High
## 48   40.6411433   -0.050444610 0.10978569 -0.45948255 0.645887678 Low>High
## 49    6.6654780   -0.372718376 0.21479575 -1.73522229 0.082701439 Low>High
## 50   41.1748713    0.043152296 0.07550299  0.57153094 0.567639794 High>Low
## 51   67.6290897   -0.228681171 0.13504457 -1.69337555 0.090384006 Low>High
## 52    0.3957527   -0.123070405 0.29760689 -0.41353345 0.679215820 Low>High
## 53    1.6496546   -0.202668514 0.30748935 -0.65910742 0.509826793 Low>High
## 54    0.9654537   -0.009265266 0.30297599 -0.03058086 0.975603807 Low>High
## 55    4.6973679    0.018150442 0.21022825  0.08633684 0.931198655 High>Low
## 56    8.4373976    0.012637918 0.12388231  0.10201552 0.918744357 High>Low
## 57   42.1549900   -0.170598911 0.15836099 -1.07727865 0.281355797 Low>High
## 58   25.0346257   -0.065106295 0.16120363 -0.40387611 0.686303824 Low>High
## 59   41.5306426    0.017962564 0.13363140  0.13441873 0.893071468 High>Low
## 60    3.6956815    0.192649192 0.23478004  0.82055183 0.411901594 High>Low
## 61    1.4314481    0.191911529 0.31015971  0.61875067 0.536080621 High>Low
## 62   73.8194362   -0.245072514 0.08832094 -2.77479499 0.005523652 Low>High
## 63    4.4517892   -0.075397486 0.18943480 -0.39801286 0.690620706 Low>High
## 64   27.3626869    0.099442074 0.16815938  0.59135608 0.554281861 High>Low
## 65  222.8112213   -0.036158505 0.07605791 -0.47540756 0.634496505 Low>High
## 66    5.0846973   -0.210961628 0.19772940 -1.06692090 0.286007557 Low>High
## 67    0.7328303    0.030721101 0.18022420  0.17046047 0.864648021 High>Low
## 68    7.6738251    0.443258291 0.17133599  2.58707050 0.009679579 High>Low
## 69   36.2070092    0.111817327 0.09265257  1.20684541 0.227491683 High>Low
## 70  409.5436984    0.017287000 0.09799649  0.17640429 0.859976338 High>Low
## 71    0.5271752    0.216689372 0.21289323  1.01783120 0.308758183 High>Low
## 72 5024.6971995   -0.009306253 0.05369756 -0.17330867 0.862408806 Low>High
## 73    6.0232614    0.296460159 0.15142152  1.95784699 0.050247970 High>Low
## 74    0.8939468   -0.231177054 0.25319669 -0.91303347 0.361224938 Low>High
## 75  915.0726073   -0.162967116 0.07316290 -2.22745557 0.025916842 Low>High
## 76  687.2657246   -0.140327579 0.07525272 -1.86475092 0.062216326 Low>High
## 77    2.4187883   -0.524233601 0.24280349 -2.15908595 0.030843499 Low>High
## 78   68.8304165   -0.106023818 0.14000594 -0.75728084 0.448881628 Low>High
## 79    2.1636390    0.208244079 0.30869674  0.67459113 0.499935571 High>Low
## 80    8.5319122   -0.061122087 0.11587177 -0.52749764 0.597848055 Low>High
## 81  100.8731479    0.070722455 0.10029414  0.70515039 0.480716650 High>Low
## 82 1333.2266191   -0.168875557 0.11536738 -1.46380675 0.143246758 Low>High
## 83    5.6579594    0.091278191 0.26468440  0.34485670 0.730202101 High>Low
## 84  259.3835376   -0.056148062 0.06506245 -0.86298725 0.388144475 Low>High
## 85   14.7423032    0.020063660 0.10087584  0.19889460 0.842345190 High>Low
```

```
## 86  277.1805098   -0.223875605 0.13351494 -1.67678322 0.093584880 Low>High
## 87 2911.4822527   -0.014642314 0.08859188 -0.16527828 0.868724954 Low>High
## 88    2.3201793    0.120218849 0.36185910  0.33222558 0.739718928 High>Low
## 89   34.7344972    0.275007961 0.14681778  1.87312439 0.061051210 High>Low
## 90    7.1544682    0.437376518 0.20659174  2.11710554 0.034250892 High>Low
## 91    7.3635144    0.221336429 0.26131476  0.84701080 0.396989101 High>Low
## 92    1.1063076   -0.155846803 0.32887992 -0.47387145 0.635591582 Low>High
## 93    0.8970041    0.650552719 0.30921381  2.10389285 0.035387794 High>Low
## 94   61.7408338   -0.073508033 0.14002958 -0.52494647 0.599620406 Low>High
## 95    8.1453806    0.009805393 0.17076030  0.05742197 0.954209061 High>Low
## 96    9.2099049   -0.353483824 0.21576062 -1.63831484 0.101356034 Low>High
## 97   32.9571263    0.291459769 0.14346980  2.03150608 0.042203684 High>Low
##     pval.adj                        Feature                    Method
## 1  0.3352769      Absconditabacteria_(SR1)_[G.1] DESeq2 man. geoMeans (ds2)
## 2  0.4045076                        Actinomyces DESeq2 man. geoMeans (ds2)
## 3  0.9756038                       Peptidiphaga DESeq2 man. geoMeans (ds2)
## 4  0.4352444                           Schaalia DESeq2 man. geoMeans (ds2)
## 5  0.9756038             Family.Actinomycetaceae. DESeq2 man. geoMeans (ds2)
## 6  0.8193179                             Rothia DESeq2 man. geoMeans (ds2)
## 7  0.9731264                      Alloscardovia DESeq2 man. geoMeans (ds2)
## 8  0.8193179                    Bifidobacterium DESeq2 man. geoMeans (ds2)
## 9  0.3352769                      Parascardovia DESeq2 man. geoMeans (ds2)
## 10 0.8193179                          Scardovia DESeq2 man. geoMeans (ds2)
## 11 0.1042733                    Corynebacterium DESeq2 man. geoMeans (ds2)
## 12 0.8193179                          Olsenella DESeq2 man. geoMeans (ds2)
## 13 0.8193179                          Atopobium DESeq2 man. geoMeans (ds2)
## 14 0.9692154                            Slackia DESeq2 man. geoMeans (ds2)
## 15 0.5027009                    Cryptobacterium DESeq2 man. geoMeans (ds2)
## 16 0.9731264                Bacteroidetes_[G.3] DESeq2 man. geoMeans (ds2)
## 17 0.9692154                Bacteroidetes_[G.5] DESeq2 man. geoMeans (ds2)
## 18 0.1341313        Family.Bacteroidetes_[F-1]. DESeq2 man. geoMeans (ds2)
## 19 0.1042733               Bacteroidaceae_[G.1] DESeq2 man. geoMeans (ds2)
## 20 0.6710981                Bacteroidales_[G.2] DESeq2 man. geoMeans (ds2)
## 21 0.6710981                      Porphyromonas DESeq2 man. geoMeans (ds2)
## 22 0.9731264                         Tannerella DESeq2 man. geoMeans (ds2)
## 23 0.9304196                     Alloprevotella DESeq2 man. geoMeans (ds2)
## 24 0.8278882                         Prevotella DESeq2 man. geoMeans (ds2)
## 25 0.3684470                         Bergeyella DESeq2 man. geoMeans (ds2)
## 26 0.8193179                     Capnocytophaga DESeq2 man. geoMeans (ds2)
## 27 0.9087997                            Gemella DESeq2 man. geoMeans (ds2)
## 28 0.9132433                        Abiotrophia DESeq2 man. geoMeans (ds2)
## 29 0.9756038                     Granulicatella DESeq2 man. geoMeans (ds2)
## 30 0.9756038                      Lactobacillus DESeq2 man. geoMeans (ds2)
## 31 0.9087997                      Streptococcus DESeq2 man. geoMeans (ds2)
## 32 0.9087997              Order.Lactobacillales. DESeq2 man. geoMeans (ds2)
## 33 0.6839310                     Class.Bacilli. DESeq2 man. geoMeans (ds2)
## 34 0.9756038             Clostridiales_[F.1][G-1] DESeq2 man. geoMeans (ds2)
## 35 0.1042733                       Butyrivibrio DESeq2 man. geoMeans (ds2)
## 36 0.8487194                          Catonella DESeq2 man. geoMeans (ds2)
## 37 0.8193179                        Johnsonella DESeq2 man. geoMeans (ds2)
## 38 0.9731264                Lachnoanaerobaculum DESeq2 man. geoMeans (ds2)
## 39 0.1042733              Lachnospiraceae_[G.2] DESeq2 man. geoMeans (ds2)
## 40 0.9756038              Lachnospiraceae_[G.3] DESeq2 man. geoMeans (ds2)
## 41 0.8193179              Lachnospiraceae_[G.7] DESeq2 man. geoMeans (ds2)
```

```
## 42 0.9087997             Lachnospiraceae_[G.8] DESeq2 man. geoMeans (ds2)
## 43 0.8193179                      Oribacterium DESeq2 man. geoMeans (ds2)
## 44 0.7842677                    Shuttleworthia DESeq2 man. geoMeans (ds2)
## 45 0.8266395                    Stomatobaculum DESeq2 man. geoMeans (ds2)
## 46 0.3207174      Family.Lachnospiraceae_[XIV]. DESeq2 man. geoMeans (ds2)
## 47 0.9756038                       Peptococcus DESeq2 man. geoMeans (ds2)
## 48 0.9213398                        Parvimonas DESeq2 man. geoMeans (ds2)
## 49 0.4011020                        Filifactor DESeq2 man. geoMeans (ds2)
## 50 0.9087997                      Mogibacterium DESeq2 man. geoMeans (ds2)
## 51 0.4045076    Peptostreptococcaceae_[XI][G.1] DESeq2 man. geoMeans (ds2)
## 52 0.9304196    Peptostreptococcaceae_[XI][G.4] DESeq2 man. geoMeans (ds2)
## 53 0.8991491    Peptostreptococcaceae_[XI][G.5] DESeq2 man. geoMeans (ds2)
## 54 0.9756038    Peptostreptococcaceae_[XI][G.6] DESeq2 man. geoMeans (ds2)
## 55 0.9756038    Peptostreptococcaceae_[XI][G.7] DESeq2 man. geoMeans (ds2)
## 56 0.9756038    Peptostreptococcaceae_[XI][G.9] DESeq2 man. geoMeans (ds2)
## 57 0.8159627               Peptostreptococcus DESeq2 man. geoMeans (ds2)
## 58 0.9304196             Ruminococcaceae_[G.1] DESeq2 man. geoMeans (ds2)
## 59 0.9756038             Ruminococcaceae_[G.2] DESeq2 man. geoMeans (ds2)
## 60 0.8266395                        Bulleidia DESeq2 man. geoMeans (ds2)
## 61 0.9087997                        Eggerthia DESeq2 man. geoMeans (ds2)
## 62 0.1071588                     Solobacterium DESeq2 man. geoMeans (ds2)
## 63 0.9304196                        Mycoplasma DESeq2 man. geoMeans (ds2)
## 64 0.9087997                       Mitsuokella DESeq2 man. geoMeans (ds2)
## 65 0.9201848                       Selenomonas DESeq2 man. geoMeans (ds2)
## 66 0.8159627          Family.Selenomonadaceae. DESeq2 man. geoMeans (ds2)
## 67 0.9756038             Class.Negativicutes. DESeq2 man. geoMeans (ds2)
## 68 0.1341313                      Anaeroglobus DESeq2 man. geoMeans (ds2)
## 69 0.7118288                         Dialister DESeq2 man. geoMeans (ds2)
## 70 0.9756038                       Megasphaera DESeq2 man. geoMeans (ds2)
## 71 0.8193179           Family.Veillonellaceae. DESeq2 man. geoMeans (ds2)
## 72 0.9756038                        Veillonella DESeq2 man. geoMeans (ds2)
## 73 0.3352769             Veillonellaceae_[G.1] DESeq2 man. geoMeans (ds2)
## 74 0.8193179               Phylum.Firmicutes. DESeq2 man. geoMeans (ds2)
## 75 0.3120560                     Fusobacterium DESeq2 man. geoMeans (ds2)
## 76 0.3352769                      Leptotrichia DESeq2 man. geoMeans (ds2)
## 77 0.3120560       Gracilibacteria_(GN02)_[G.1] DESeq2 man. geoMeans (ds2)
## 78 0.8487194                         Lautropia DESeq2 man. geoMeans (ds2)
## 79 0.8980324                           Ottowia DESeq2 man. geoMeans (ds2)
## 80 0.9087997                         Eikenella DESeq2 man. geoMeans (ds2)
## 81 0.8798022                          Kingella DESeq2 man. geoMeans (ds2)
## 82 0.5146272                         Neisseria DESeq2 man. geoMeans (ds2)
## 83 0.9692154             Family.Neisseriaceae. DESeq2 man. geoMeans (ds2)
## 84 0.8193179                     Campylobacter DESeq2 man. geoMeans (ds2)
## 85 0.9756038                    Cardiobacterium DESeq2 man. geoMeans (ds2)
## 86 0.4045076                    Aggregatibacter DESeq2 man. geoMeans (ds2)
## 87 0.9756038                        Haemophilus DESeq2 man. geoMeans (ds2)
## 88 0.9692154           Family.Pasteurellaceae. DESeq2 man. geoMeans (ds2)
## 89 0.3352769       Saccharibacteria_(TM7)_[G.1] DESeq2 man. geoMeans (ds2)
## 90 0.3120560       Saccharibacteria_(TM7)_[G.3] DESeq2 man. geoMeans (ds2)
## 91 0.8193179       Saccharibacteria_(TM7)_[G.6] DESeq2 man. geoMeans (ds2)
## 92 0.9201848 Family.Saccharibacteria_(TM7)_[F-1]. DESeq2 man. geoMeans (ds2)
## 93 0.3120560       Saccharibacteria_(TM7)_[G.5] DESeq2 man. geoMeans (ds2)
## 94 0.9087997                         Treponema DESeq2 man. geoMeans (ds2)
## 95 0.9756038                     Fretibacterium DESeq2 man. geoMeans (ds2)
```

```
## 96 0.4096473                  Kingdom.Bacteria. DESeq2 man. geoMeans (ds2)
## 97 0.3207174                             Others DESeq2 man. geoMeans (ds2)
```

```r
write.table(final, file="Risk_ds2.txt", sep="\t", dec=",", row.names=F)
write.table(final[final$pval<0.05,], file="Risk_ds2_sig.txt", sep="\t", dec=",", row.names=F)
final <- DA.kru(Microbio2, predictor = Phe2$Risk)
#kable(final[,1:10])
final
```

```
##                                            pval   pval.adj
## Absconditabacteria_(SR1)_[G.1]       0.5294446472 0.89067795
## Actinomyces                          0.0486265982 0.28228056
## Peptidiphaga                         0.9124957562 0.96752734
## Schaalia                             0.1073310873 0.43379648
## Family.Actinomycetaceae.             0.9394420691 0.96752734
## Rothia                               0.0233358367 0.24200257
## Alloscardovia                        0.2793670418 0.66094154
## Bifidobacterium                      0.5876638046 0.89067795
## Parascardovia                        0.2558875639 0.66094154
## Scardovia                            0.6233571220 0.90247225
## Corynebacterium                      0.1937961820 0.62039951
## Olsenella                            0.2549800783 0.66094154
## Atopobium                            0.2721139818 0.66094154
## Slackia                              0.5654072699 0.89067795
## Cryptobacterium                      0.0962788608 0.42450225
## Bacteroidetes_[G.3]                  0.7045765709 0.92356659
## Bacteroidetes_[G.5]                  0.9392084032 0.96752734
## Family.Bacteroidetes_[F-1].          0.0003956684 0.03837983
## Bacteroidaceae_[G.1]                 0.4231885026 0.86116263
## Bacteroidales_[G.2]                  0.2121490771 0.62039951
## Porphyromonas                        0.1152100446 0.44701497
## Tannerella                           0.5262661842 0.89067795
## Alloprevotella                       0.7590592690 0.95621752
## Prevotella                           0.6758127929 0.92010595
## Bergeyella                           0.0822953805 0.38012628
## Capnocytophaga                       0.7477899744 0.95441615
## Gemella                              0.1697850329 0.60629471
## Abiotrophia                          0.0064341075 0.14883489
## Granulicatella                       0.2347064920 0.65047228
## Lactobacillus                        0.8269978313 0.96752734
## Streptococcus                        0.0249487187 0.24200257
## Order.Lactobacillales.               0.0493362953 0.28228056
## Class.Bacilli.                       0.2169770558 0.62039951
## Clostridiales_[F.1][G-1]             0.6924508695 0.92010595
## Butyrivibrio                         0.0721813594 0.36850483
## Catonella                            0.4261423309 0.86116263
## Johnsonella                          0.7862059985 0.96534154
## Lachnoanaerobaculum                  0.8164105456 0.96752734
## Lachnospiraceae_[G.2]                0.0015896793 0.07709945
## Lachnospiraceae_[G.3]                0.4874627339 0.87851599
## Lachnospiraceae_[G.7]                0.0080864740 0.14883489
## Lachnospiraceae_[G.8]                0.2174596209 0.62039951
## Oribacterium                         0.5615778388 0.89067795
## Shuttleworthia                       0.5517225267 0.89067795
```

```
## Stomatobaculum                        0.6885578911 0.92010595
## Family.Lachnospiraceae_[XIV].         0.0317780724 0.26566521
## Peptococcus                           0.8790183250 0.96752734
## Parvimonas                            0.8432122157 0.96752734
## Filifactor                            0.4412912027 0.87357646
## Mogibacterium                         0.5841142925 0.89067795
## Peptostreptococcaceae_[XI][G.1]       0.0780133178 0.37836459
## Peptostreptococcaceae_[XI][G.4]       0.6022193384 0.89869655
## Peptostreptococcaceae_[XI][G.5]       0.1811615210 0.60629471
## Peptostreptococcaceae_[XI][G.6]       0.4253279111 0.86116263
## Peptostreptococcaceae_[XI][G.7]       0.5330615014 0.89067795
## Peptostreptococcaceae_[XI][G.9]       0.6183138617 0.90247225
## Peptostreptococcus                    0.2752491741 0.66094154
## Ruminococcaceae_[G.1]                 0.7699901905 0.95755190
## Ruminococcaceae_[G.2]                 0.9448259266 0.96752734
## Bulleidia                             0.9475783241 0.96752734
## Eggerthia                             0.9296761765 0.96752734
## Solobacterium                         0.0492488054 0.28228056
## Mycoplasma                            0.6452873553 0.90393944
## Mitsuokella                           0.6334281092 0.90356657
## Selenomonas                           0.3160230657 0.72986279
## Family.Selenomonadaceae.              0.1029970875 0.43379648
## Class.Negativicutes.                  0.5624001174 0.89067795
## Anaeroglobus                          0.2766018612 0.66094154
## Dialister                             0.4890707570 0.87851599
## Megasphaera                           0.9276207549 0.96752734
## Family.Veillonellaceae.               0.9748522435 0.98500695
## Veillonella                           0.8824697700 0.96752734
## Veillonellaceae_[G.1]                 0.8671075528 0.96752734
## Phylum.Firmicutes.                    0.6523274293 0.90393944
## Fusobacterium                         0.0660171142 0.35575889
## Leptotrichia                          0.0122722867 0.15902120
## Gracilibacteria_(GN02)_[G.1]          0.0131151508 0.15902120
## Lautropia                             0.0092062817 0.14883489
## Ottowia                               0.4578236013 0.87851599
## Eikenella                             0.4228295247 0.86116263
## Kingella                              0.8899007425 0.96752734
## Neisseria                             0.0356046159 0.26566521
## Family.Neisseriaceae.                 0.1812633681 0.60629471
## Campylobacter                         0.3730179700 0.84145914
## Cardiobacterium                       0.4661803155 0.87851599
## Aggregatibacter                       0.0339937979 0.26566521
## Haemophilus                           0.9929254693 0.99292547
## Family.Pasteurellaceae.               0.9285172098 0.96752734
## Saccharibacteria_(TM7)_[G.1]          0.4787792864 0.87851599
## Saccharibacteria_(TM7)_[G.3]          0.7209230499 0.93239381
## Saccharibacteria_(TM7)_[G.6]          0.0071887800 0.14883489
## Family.Saccharibacteria_(TM7)_[F-1].  0.2102348517 0.62039951
## Saccharibacteria_(TM7)_[G.5]          0.1287531901 0.48034844
## Treponema                             0.5709812587 0.89067795
## Fretibacterium                        0.9199335645 0.96752734
## Kingdom.Bacteria.                     0.4207221289 0.86116263
## Others                                0.0494718504 0.28228056
##                                                                    Feature
```

```
## Absconditabacteria_(SR1)_[G.1]             Absconditabacteria_(SR1)_[G.1]
## Actinomyces                                                    Actinomyces
## Peptidiphaga                                                  Peptidiphaga
## Schaalia                                                          Schaalia
## Family.Actinomycetaceae.                          Family.Actinomycetaceae.
## Rothia                                                              Rothia
## Alloscardovia                                                Alloscardovia
## Bifidobacterium                                            Bifidobacterium
## Parascardovia                                                Parascardovia
## Scardovia                                                        Scardovia
## Corynebacterium                                            Corynebacterium
## Olsenella                                                        Olsenella
## Atopobium                                                        Atopobium
## Slackia                                                            Slackia
## Cryptobacterium                                            Cryptobacterium
## Bacteroidetes_[G.3]                                    Bacteroidetes_[G.3]
## Bacteroidetes_[G.5]                                    Bacteroidetes_[G.5]
## Family.Bacteroidetes_[F-1].                      Family.Bacteroidetes_[F-1].
## Bacteroidaceae_[G.1]                                  Bacteroidaceae_[G.1]
## Bacteroidales_[G.2]                                    Bacteroidales_[G.2]
## Porphyromonas                                                Porphyromonas
## Tannerella                                                      Tannerella
## Alloprevotella                                              Alloprevotella
## Prevotella                                                      Prevotella
## Bergeyella                                                      Bergeyella
## Capnocytophaga                                              Capnocytophaga
## Gemella                                                            Gemella
## Abiotrophia                                                    Abiotrophia
## Granulicatella                                              Granulicatella
## Lactobacillus                                                Lactobacillus
## Streptococcus                                                Streptococcus
## Order.Lactobacillales.                              Order.Lactobacillales.
## Class.Bacilli.                                              Class.Bacilli.
## Clostridiales_[F.1][G-1]                          Clostridiales_[F.1][G-1]
## Butyrivibrio                                                  Butyrivibrio
## Catonella                                                        Catonella
## Johnsonella                                                    Johnsonella
## Lachnoanaerobaculum                                    Lachnoanaerobaculum
## Lachnospiraceae_[G.2]                                Lachnospiraceae_[G.2]
## Lachnospiraceae_[G.3]                                Lachnospiraceae_[G.3]
## Lachnospiraceae_[G.7]                                Lachnospiraceae_[G.7]
## Lachnospiraceae_[G.8]                                Lachnospiraceae_[G.8]
## Oribacterium                                                  Oribacterium
## Shuttleworthia                                              Shuttleworthia
## Stomatobaculum                                              Stomatobaculum
## Family.Lachnospiraceae_[XIV].                  Family.Lachnospiraceae_[XIV].
## Peptococcus                                                    Peptococcus
## Parvimonas                                                      Parvimonas
## Filifactor                                                      Filifactor
## Mogibacterium                                                Mogibacterium
## Peptostreptococcaceae_[XI][G.1]            Peptostreptococcaceae_[XI][G.1]
## Peptostreptococcaceae_[XI][G.4]            Peptostreptococcaceae_[XI][G.4]
## Peptostreptococcaceae_[XI][G.5]            Peptostreptococcaceae_[XI][G.5]
## Peptostreptococcaceae_[XI][G.6]            Peptostreptococcaceae_[XI][G.6]
```

```
## Peptostreptococcaceae_[XI][G.7]          Peptostreptococcaceae_[XI][G.7]
## Peptostreptococcaceae_[XI][G.9]          Peptostreptococcaceae_[XI][G.9]
## Peptostreptococcus                                    Peptostreptococcus
## Ruminococcaceae_[G.1]                              Ruminococcaceae_[G.1]
## Ruminococcaceae_[G.2]                              Ruminococcaceae_[G.2]
## Bulleidia                                                      Bulleidia
## Eggerthia                                                      Eggerthia
## Solobacterium                                              Solobacterium
## Mycoplasma                                                    Mycoplasma
## Mitsuokella                                                  Mitsuokella
## Selenomonas                                                  Selenomonas
## Family.Selenomonadaceae.                        Family.Selenomonadaceae.
## Class.Negativicutes.                                Class.Negativicutes.
## Anaeroglobus                                                Anaeroglobus
## Dialister                                                      Dialister
## Megasphaera                                                  Megasphaera
## Family.Veillonellaceae.                          Family.Veillonellaceae.
## Veillonella                                                  Veillonella
## Veillonellaceae_[G.1]                              Veillonellaceae_[G.1]
## Phylum.Firmicutes.                                    Phylum.Firmicutes.
## Fusobacterium                                              Fusobacterium
## Leptotrichia                                                Leptotrichia
## Gracilibacteria_(GN02)_[G.1]                Gracilibacteria_(GN02)_[G.1]
## Lautropia                                                      Lautropia
## Ottowia                                                          Ottowia
## Eikenella                                                      Eikenella
## Kingella                                                        Kingella
## Neisseria                                                      Neisseria
## Family.Neisseriaceae.                              Family.Neisseriaceae.
## Campylobacter                                              Campylobacter
## Cardiobacterium                                          Cardiobacterium
## Aggregatibacter                                          Aggregatibacter
## Haemophilus                                                  Haemophilus
## Family.Pasteurellaceae.                          Family.Pasteurellaceae.
## Saccharibacteria_(TM7)_[G.1]                Saccharibacteria_(TM7)_[G.1]
## Saccharibacteria_(TM7)_[G.3]                Saccharibacteria_(TM7)_[G.3]
## Saccharibacteria_(TM7)_[G.6]                Saccharibacteria_(TM7)_[G.6]
## Family.Saccharibacteria_(TM7)_[F-1]. Family.Saccharibacteria_(TM7)_[F-1].
## Saccharibacteria_(TM7)_[G.5]                Saccharibacteria_(TM7)_[G.5]
## Treponema                                                      Treponema
## Fretibacterium                                            Fretibacterium
## Kingdom.Bacteria.                                      Kingdom.Bacteria.
## Others                                                            Others
##                                                  Method
## Absconditabacteria_(SR1)_[G.1]       Kruskal-Wallis (kru)
## Actinomyces                          Kruskal-Wallis (kru)
## Peptidiphaga                         Kruskal-Wallis (kru)
## Schaalia                             Kruskal-Wallis (kru)
## Family.Actinomycetaceae.             Kruskal-Wallis (kru)
## Rothia                               Kruskal-Wallis (kru)
## Alloscardovia                        Kruskal-Wallis (kru)
## Bifidobacterium                      Kruskal-Wallis (kru)
## Parascardovia                        Kruskal-Wallis (kru)
## Scardovia                            Kruskal-Wallis (kru)
```

```
## Corynebacterium                      Kruskal-Wallis (kru)
## Olsenella                            Kruskal-Wallis (kru)
## Atopobium                            Kruskal-Wallis (kru)
## Slackia                              Kruskal-Wallis (kru)
## Cryptobacterium                      Kruskal-Wallis (kru)
## Bacteroidetes_[G.3]                  Kruskal-Wallis (kru)
## Bacteroidetes_[G.5]                  Kruskal-Wallis (kru)
## Family.Bacteroidetes_[F-1].          Kruskal-Wallis (kru)
## Bacteroidaceae_[G.1]                 Kruskal-Wallis (kru)
## Bacteroidales_[G.2]                  Kruskal-Wallis (kru)
## Porphyromonas                        Kruskal-Wallis (kru)
## Tannerella                           Kruskal-Wallis (kru)
## Alloprevotella                       Kruskal-Wallis (kru)
## Prevotella                           Kruskal-Wallis (kru)
## Bergeyella                           Kruskal-Wallis (kru)
## Capnocytophaga                       Kruskal-Wallis (kru)
## Gemella                              Kruskal-Wallis (kru)
## Abiotrophia                          Kruskal-Wallis (kru)
## Granulicatella                       Kruskal-Wallis (kru)
## Lactobacillus                        Kruskal-Wallis (kru)
## Streptococcus                        Kruskal-Wallis (kru)
## Order.Lactobacillales.               Kruskal-Wallis (kru)
## Class.Bacilli.                       Kruskal-Wallis (kru)
## Clostridiales_[F.1][G-1]             Kruskal-Wallis (kru)
## Butyrivibrio                         Kruskal-Wallis (kru)
## Catonella                            Kruskal-Wallis (kru)
## Johnsonella                          Kruskal-Wallis (kru)
## Lachnoanaerobaculum                  Kruskal-Wallis (kru)
## Lachnospiraceae_[G.2]                Kruskal-Wallis (kru)
## Lachnospiraceae_[G.3]                Kruskal-Wallis (kru)
## Lachnospiraceae_[G.7]                Kruskal-Wallis (kru)
## Lachnospiraceae_[G.8]                Kruskal-Wallis (kru)
## Oribacterium                         Kruskal-Wallis (kru)
## Shuttleworthia                       Kruskal-Wallis (kru)
## Stomatobaculum                       Kruskal-Wallis (kru)
## Family.Lachnospiraceae_[XIV].        Kruskal-Wallis (kru)
## Peptococcus                          Kruskal-Wallis (kru)
## Parvimonas                           Kruskal-Wallis (kru)
## Filifactor                           Kruskal-Wallis (kru)
## Mogibacterium                        Kruskal-Wallis (kru)
## Peptostreptococcaceae_[XI][G.1]      Kruskal-Wallis (kru)
## Peptostreptococcaceae_[XI][G.4]      Kruskal-Wallis (kru)
## Peptostreptococcaceae_[XI][G.5]      Kruskal-Wallis (kru)
## Peptostreptococcaceae_[XI][G.6]      Kruskal-Wallis (kru)
## Peptostreptococcaceae_[XI][G.7]      Kruskal-Wallis (kru)
## Peptostreptococcaceae_[XI][G.9]      Kruskal-Wallis (kru)
## Peptostreptococcus                   Kruskal-Wallis (kru)
## Ruminococcaceae_[G.1]                Kruskal-Wallis (kru)
## Ruminococcaceae_[G.2]                Kruskal-Wallis (kru)
## Bulleidia                            Kruskal-Wallis (kru)
## Eggerthia                            Kruskal-Wallis (kru)
## Solobacterium                        Kruskal-Wallis (kru)
## Mycoplasma                           Kruskal-Wallis (kru)
## Mitsuokella                          Kruskal-Wallis (kru)
```

```
## Selenomonas                          Kruskal-Wallis (kru)
## Family.Selenomonadaceae.             Kruskal-Wallis (kru)
## Class.Negativicutes.                 Kruskal-Wallis (kru)
## Anaeroglobus                         Kruskal-Wallis (kru)
## Dialister                            Kruskal-Wallis (kru)
## Megasphaera                          Kruskal-Wallis (kru)
## Family.Veillonellaceae.              Kruskal-Wallis (kru)
## Veillonella                          Kruskal-Wallis (kru)
## Veillonellaceae_[G.1]                Kruskal-Wallis (kru)
## Phylum.Firmicutes.                   Kruskal-Wallis (kru)
## Fusobacterium                        Kruskal-Wallis (kru)
## Leptotrichia                         Kruskal-Wallis (kru)
## Gracilibacteria_(GN02)_[G.1]         Kruskal-Wallis (kru)
## Lautropia                            Kruskal-Wallis (kru)
## Ottowia                              Kruskal-Wallis (kru)
## Eikenella                            Kruskal-Wallis (kru)
## Kingella                             Kruskal-Wallis (kru)
## Neisseria                            Kruskal-Wallis (kru)
## Family.Neisseriaceae.                Kruskal-Wallis (kru)
## Campylobacter                        Kruskal-Wallis (kru)
## Cardiobacterium                      Kruskal-Wallis (kru)
## Aggregatibacter                      Kruskal-Wallis (kru)
## Haemophilus                          Kruskal-Wallis (kru)
## Family.Pasteurellaceae.              Kruskal-Wallis (kru)
## Saccharibacteria_(TM7)_[G.1]         Kruskal-Wallis (kru)
## Saccharibacteria_(TM7)_[G.3]         Kruskal-Wallis (kru)
## Saccharibacteria_(TM7)_[G.6]         Kruskal-Wallis (kru)
## Family.Saccharibacteria_(TM7)_[F-1]. Kruskal-Wallis (kru)
## Saccharibacteria_(TM7)_[G.5]         Kruskal-Wallis (kru)
## Treponema                            Kruskal-Wallis (kru)
## Fretibacterium                       Kruskal-Wallis (kru)
## Kingdom.Bacteria.                    Kruskal-Wallis (kru)
## Others                               Kruskal-Wallis (kru)
```

```r
write.table(final, file="Risk_kru.txt", sep="\t", dec=",", row.names=F)
write.table(final[final$pval<0.05,], file="Risk_kru_sig.txt", sep="\t", dec=",", row.names=F)
#################Smoking
#Remove NA
Phe2<-Phe[complete.cases(Phe$Smoking),]
#Also subset columns
Microbio2<-dplyr::select(Microbio, one_of(Phe2$IDX))

# res <- allDA(Microbio, predictor = Phe$Smoking)
# vennDA(res, tests = c("kru", "aov", "lma")) #ds2 does not seem implemented
final <- DA.ds2(Microbio2, predictor = Phe2$Smoking, out.all=TRUE) #It is LRT so don't look at ordering
##kable(final[,1:10])
final
```

```
##                                         baseMean log2FoldChange      lfcSE
## Absconditabacteria_(SR1)_[G.1]        68.1884770   0.2399373798 0.26903770
## Actinomyces                          318.3322149  -0.1773160281 0.08952894
## Peptidiphaga                          22.1074345   0.0667680513 0.13103890
## Schaalia                             706.1094247  -0.0299286668 0.07579547
## Family.Actinomycetaceae.               5.4741804   0.2199928043 0.18299137
```

```
## Rothia                              1941.7150356  -0.1558838769 0.08814460
## Alloscardovia                         12.5452557  -0.3766665203 0.22912222
## Bifidobacterium                       11.4961263  -0.5486130171 0.25564970
## Parascardovia                          2.8812493  -0.6249425075 0.35392192
## Scardovia                             14.4319520  -0.7211020247 0.22971180
## Corynebacterium                       93.4829621  -0.0370359911 0.09737466
## Olsenella                              1.5665904  -0.1908810873 0.21808142
## Atopobium                            120.8668570  -0.1021123068 0.08827095
## Slackia                                1.3972260   0.0544307738 0.20639957
## Cryptobacterium                        4.3318660  -0.3303049136 0.22051063
## Bacteroidetes_[G.3]                    6.6121458  -0.2002265199 0.22709283
## Bacteroidetes_[G.5]                    4.7038352  -0.3827682168 0.25416503
## Family.Bacteroidetes_[F-1].            0.8691456  -0.1598950115 0.36700333
## Bacteroidaceae_[G.1]                   0.7970647  -1.0668454662 0.34526474
## Bacteroidales_[G.2]                   12.2333676  -0.2670269506 0.13075177
## Porphyromonas                        681.9545694  -0.1112430753 0.12189719
## Tannerella                            22.1260422   0.0024729938 0.09066616
## Alloprevotella                       360.8075942   0.0412522855 0.09724188
## Prevotella                          3819.3424178   0.0237177646 0.06202395
## Bergeyella                            72.7699971   0.0309669796 0.09477809
## Capnocytophaga                       225.4066835  -0.0228137821 0.10164860
## Gemella                              307.2225040  -0.0407253890 0.08402132
## Abiotrophia                           30.1267760   0.1488191927 0.18432682
## Granulicatella                       307.0660429  -0.2120572823 0.07374464
## Lactobacillus                         20.8870563  -0.9650172761 0.26627619
## Streptococcus                       3692.7041122  -0.1830134109 0.06420999
## Order.Lactobacillales.                 0.2875429   0.0269506124 0.25334821
## Class.Bacilli.                         0.5744446  -0.1725278277 0.20418408
## Clostridiales_[F.1][G-1]               1.0576823  -0.1324087493 0.30487153
## Butyrivibrio                           6.9179411   0.1192372825 0.19455837
## Catonella                             36.8658026   0.1139330335 0.10724577
## Johnsonella                            0.9430037   0.4949537214 0.26449797
## Lachnoanaerobaculum                   88.0618361   0.0614259011 0.07649999
## Lachnospiraceae_[G.2]                 27.7960142   0.2141702879 0.19948223
## Lachnospiraceae_[G.3]                  2.9045386   0.0449916863 0.20622076
## Lachnospiraceae_[G.7]                  1.4681485  -1.0256563660 0.28846892
## Lachnospiraceae_[G.8]                  1.4290881   0.0475994554 0.31915825
## Oribacterium                         147.3021292   0.0826130587 0.08505630
## Shuttleworthia                         2.8032786  -0.4613055963 0.23778744
## Stomatobaculum                       112.6166927   0.0394714274 0.09589849
## Family.Lachnospiraceae_[XIV].          1.5157956  -0.6264330290 0.26481761
## Peptococcus                            3.7065969  -0.0168131739 0.23326667
## Parvimonas                            40.9718954   0.1350280479 0.11771612
## Filifactor                             6.8447682  -0.1404738498 0.22881461
## Mogibacterium                         41.1395525   0.1102993113 0.08136403
## Peptostreptococcaceae_[XI][G.1]       67.6626888   0.1397255447 0.14442259
## Peptostreptococcaceae_[XI][G.4]        0.4194042   0.0259391496 0.30695805
## Peptostreptococcaceae_[XI][G.5]        1.6651247   0.1017019132 0.32627455
## Peptostreptococcaceae_[XI][G.6]        1.0140859  -0.6644037066 0.32267141
## Peptostreptococcaceae_[XI][G.7]        4.7273128  -0.1858308381 0.22523548
## Peptostreptococcaceae_[XI][G.9]        8.7571036  -0.1134163275 0.13138794
## Peptostreptococcus                    42.9442215   0.0279318276 0.16612036
## Ruminococcaceae_[G.1]                 25.0919901   0.0527786407 0.17178019
## Ruminococcaceae_[G.2]                 41.5536975   0.1878548622 0.14365659
```

```
## Bulleidia                              3.8808855  -0.0956938715 0.24888681
## Eggerthia                              1.4534090  -0.6969928644 0.33522902
## Solobacterium                         74.3636600  -0.0332042637 0.09575634
## Mycoplasma                             4.5188931   0.0528926585 0.20041611
## Mitsuokella                           27.0484273  -0.1499844719 0.18133169
## Selenomonas                          222.0971743   0.1281262549 0.08143691
## Family.Selenomonadaceae.               5.0079108   0.5782450798 0.21017066
## Class.Negativicutes.                   0.7429172  -0.3052297688 0.19569504
## Anaeroglobus                           8.7510601   0.0490845769 0.19557068
## Dialister                             36.2908084  -0.0863616562 0.09920270
## Megasphaera                          408.7473606  -0.0122580730 0.10488109
## Family.Veillonellaceae.                0.5200049  -0.4709698963 0.22935799
## Veillonella                         5079.2462825  -0.0414566933 0.05948954
## Veillonellaceae_[G.1]                  6.1567857  -0.0613763384 0.16564896
## Phylum.Firmicutes.                     0.8477043  -0.2482695290 0.26805509
## Fusobacterium                        927.8324686   0.1547901823 0.07798954
## Leptotrichia                         682.8458772  -0.1079380341 0.08077243
## Gracilibacteria_(GN02)_[G.1]           2.2806113   0.2509662195 0.25661057
## Lautropia                             68.6447354   0.1079727899 0.14902135
## Ottowia                                2.3492850  -0.3302334162 0.33767975
## Eikenella                              8.7739190  -0.0623361730 0.12678681
## Kingella                              99.1526054  -0.0802411710 0.10739899
## Neisseria                           1330.0337272  -0.1170996880 0.12169297
## Family.Neisseriaceae.                  6.1025523   0.5554519968 0.28607787
## Campylobacter                        258.1977958   0.0862054786 0.07000254
## Cardiobacterium                       14.8344819  -0.1207760042 0.10739592
## Aggregatibacter                      277.1687266   0.2075289907 0.14183434
## Haemophilus                         2907.3468459  -0.0439154681 0.09630622
## Family.Pasteurellaceae.                2.4084294  -0.1731143302 0.39227417
## Saccharibacteria_(TM7)_[G.1]          36.1909685   0.2304868195 0.16092537
## Saccharibacteria_(TM7)_[G.3]           7.1351673  -0.0619486094 0.22427332
## Saccharibacteria_(TM7)_[G.6]           7.2904011  -0.1792898298 0.28520701
## Family.Saccharibacteria_(TM7)_[F-1].   1.0990853   0.0006650367 0.35853136
## Saccharibacteria_(TM7)_[G.5]           0.9155241  -0.1171308054 0.33778824
## Treponema                             63.3259190  -0.0975166715 0.14799320
## Fretibacterium                         8.3058756   0.0143662164 0.17948025
## Kingdom.Bacteria.                      9.3830109   0.2408553824 0.23254891
## Others                                32.3266608   0.0174493098 0.15414325
##                                            stat         pval           ordering
## Absconditabacteria_(SR1)_[G.1]       46.2004114 9.283423e-11 Nonsmoker>Exsmoker
## Actinomyces                          19.7009374 5.272248e-05 Exsmoker>Nonsmoker
## Peptidiphaga                         17.3209509 1.733019e-04 Nonsmoker>Exsmoker
## Schaalia                             12.4720248 1.957646e-03 Exsmoker>Nonsmoker
## Family.Actinomycetaceae.             43.2554286 4.047651e-10 Nonsmoker>Exsmoker
## Rothia                               26.3610796 1.886967e-06 Exsmoker>Nonsmoker
## Alloscardovia                        40.3256658 1.751431e-09 Exsmoker>Nonsmoker
## Bifidobacterium                      19.7003392 5.273825e-05 Exsmoker>Nonsmoker
## Parascardovia                         4.3178770 1.154476e-01 Exsmoker>Nonsmoker
## Scardovia                            10.4675607 5.333325e-03 Exsmoker>Nonsmoker
## Corynebacterium                       0.2116874 8.995652e-01 Exsmoker>Nonsmoker
## Olsenella                             5.2808379 7.133138e-02 Exsmoker>Nonsmoker
## Atopobium                            30.1609782 2.822454e-07 Exsmoker>Nonsmoker
## Slackia                              17.5116332 1.575423e-04 Nonsmoker>Exsmoker
## Cryptobacterium                      19.0092746 7.450552e-05 Exsmoker>Nonsmoker
```

```
## Bacteroidetes_[G.3]                  51.6103597 6.208015e-12 Exsmoker>Nonsmoker
## Bacteroidetes_[G.5]                  12.1271539 2.326066e-03 Exsmoker>Nonsmoker
## Family.Bacteroidetes_[F-1].           7.1565538 2.792377e-02 Exsmoker>Nonsmoker
## Bacteroidaceae_[G.1]                 19.0655320 7.243898e-05 Exsmoker>Nonsmoker
## Bacteroidales_[G.2]                  53.8052567 2.071748e-12 Exsmoker>Nonsmoker
## Porphyromonas                        16.2009635 3.033929e-04 Exsmoker>Nonsmoker
## Tannerella                            2.0143436 3.652505e-01 Nonsmoker>Exsmoker
## Alloprevotella                        5.2039077 7.412860e-02 Nonsmoker>Exsmoker
## Prevotella                            1.6707698 4.337075e-01 Nonsmoker>Exsmoker
## Bergeyella                           18.3080849 1.057913e-04 Nonsmoker>Exsmoker
## Capnocytophaga                       10.1627538 6.211351e-03 Exsmoker>Nonsmoker
## Gemella                              16.8880548 2.151818e-04 Exsmoker>Nonsmoker
## Abiotrophia                           6.2525963 4.387993e-02 Nonsmoker>Exsmoker
## Granulicatella                       16.7925558 2.257059e-04 Exsmoker>Nonsmoker
## Lactobacillus                        58.7699299 1.730900e-13 Exsmoker>Nonsmoker
## Streptococcus                        16.2819781 2.913489e-04 Exsmoker>Nonsmoker
## Order.Lactobacillales.               -3.7103373 1.000000e+00 Nonsmoker>Exsmoker
## Class.Bacilli.                        4.8531842 8.833736e-02 Exsmoker>Nonsmoker
## Clostridiales_[F.1][G-1]             15.7790924 3.746395e-04 Exsmoker>Nonsmoker
## Butyrivibrio                          1.8179087 4.029454e-01 Nonsmoker>Exsmoker
## Catonella                            35.7774262 1.702278e-08 Nonsmoker>Exsmoker
## Johnsonella                           7.9792300 1.850684e-02 Nonsmoker>Exsmoker
## Lachnoanaerobaculum                   2.3132414 3.145473e-01 Nonsmoker>Exsmoker
## Lachnospiraceae_[G.2]                12.4845916 1.945384e-03 Nonsmoker>Exsmoker
## Lachnospiraceae_[G.3]                10.7540418 4.621570e-03 Nonsmoker>Exsmoker
## Lachnospiraceae_[G.7]                64.9444344 7.897602e-15 Exsmoker>Nonsmoker
## Lachnospiraceae_[G.8]                 6.1457463 4.628797e-02 Nonsmoker>Exsmoker
## Oribacterium                          7.1948037 2.739481e-02 Nonsmoker>Exsmoker
## Shuttleworthia                        7.2384109 2.680396e-02 Exsmoker>Nonsmoker
## Stomatobaculum                        0.9354884 6.264137e-01 Nonsmoker>Exsmoker
## Family.Lachnospiraceae_[XIV].         7.6924343 2.136039e-02 Exsmoker>Nonsmoker
## Peptococcus                          17.7920568 1.369317e-04 Exsmoker>Nonsmoker
## Parvimonas                            1.3589613 5.068802e-01 Nonsmoker>Exsmoker
## Filifactor                           18.2923143 1.066288e-04 Exsmoker>Nonsmoker
## Mogibacterium                         3.5585953 1.687566e-01 Nonsmoker>Exsmoker
## Peptostreptococcaceae_[XI][G.1]      13.0444139 1.470420e-03 Nonsmoker>Exsmoker
## Peptostreptococcaceae_[XI][G.4]      21.2231291 2.462953e-05 Nonsmoker>Exsmoker
## Peptostreptococcaceae_[XI][G.5]      16.3480782 2.818772e-04 Nonsmoker>Exsmoker
## Peptostreptococcaceae_[XI][G.6]      29.1955015 4.573803e-07 Exsmoker>Nonsmoker
## Peptostreptococcaceae_[XI][G.7]       8.5358637 1.401073e-02 Exsmoker>Nonsmoker
## Peptostreptococcaceae_[XI][G.9]      47.0148219 6.178188e-11 Exsmoker>Nonsmoker
## Peptostreptococcus                   37.1565446 8.541986e-09 Nonsmoker>Exsmoker
## Ruminococcaceae_[G.1]                 6.0048817 4.966569e-02 Nonsmoker>Exsmoker
## Ruminococcaceae_[G.2]                 3.5816088 1.668259e-01 Nonsmoker>Exsmoker
## Bulleidia                            27.3058716 1.176536e-06 Exsmoker>Nonsmoker
## Eggerthia                             8.3696449 1.522491e-02 Exsmoker>Nonsmoker
## Solobacterium                         0.2827409 8.681676e-01 Exsmoker>Nonsmoker
## Mycoplasma                           21.2977618 2.372738e-05 Nonsmoker>Exsmoker
## Mitsuokella                           3.2455149 1.973538e-01 Exsmoker>Nonsmoker
## Selenomonas                          11.1728964 3.748318e-03 Nonsmoker>Exsmoker
## Family.Selenomonadaceae.             20.9712082 2.793573e-05 Nonsmoker>Exsmoker
## Class.Negativicutes.                  2.9113056 2.332481e-01 Exsmoker>Nonsmoker
## Anaeroglobus                          5.0108817 8.163960e-02 Nonsmoker>Exsmoker
## Dialister                            17.3267848 1.727971e-04 Exsmoker>Nonsmoker
```

```
## Megasphaera                             8.8121712 1.220285e-02 Exsmoker>Nonsmoker
## Family.Veillonellaceae.                18.1967148 1.118494e-04 Exsmoker>Nonsmoker
## Veillonella                             3.8367703 1.468439e-01 Exsmoker>Nonsmoker
## Veillonellaceae_[G.1]                   3.0137504 2.216014e-01 Exsmoker>Nonsmoker
## Phylum.Firmicutes.                      1.5651107 4.572361e-01 Exsmoker>Nonsmoker
## Fusobacterium                          28.5070609 6.453129e-07 Nonsmoker>Exsmoker
## Leptotrichia                           21.0997819 2.619634e-05 Exsmoker>Nonsmoker
## Gracilibacteria_(GN02)_[G.1]            9.4608551 8.822698e-03 Nonsmoker>Exsmoker
## Lautropia                              37.9667064 5.696846e-09 Nonsmoker>Exsmoker
## Ottowia                                 6.7484271 3.424504e-02 Exsmoker>Nonsmoker
## Eikenella                               3.9583168 1.381855e-01 Exsmoker>Nonsmoker
## Kingella                               22.7945498 1.122604e-05 Exsmoker>Nonsmoker
## Neisseria                              58.1932757 2.309355e-13 Exsmoker>Nonsmoker
## Family.Neisseriaceae.                  15.3838816 4.564913e-04 Nonsmoker>Exsmoker
## Campylobacter                           5.7783570 5.562189e-02 Nonsmoker>Exsmoker
## Cardiobacterium                        25.0638218 3.609610e-06 Exsmoker>Nonsmoker
## Aggregatibacter                        26.6984391 1.594071e-06 Nonsmoker>Exsmoker
## Haemophilus                             3.1930613 2.025982e-01 Exsmoker>Nonsmoker
## Family.Pasteurellaceae.                 3.5449399 1.699128e-01 Exsmoker>Nonsmoker
## Saccharibacteria_(TM7)_[G.1]            2.4794140 2.894690e-01 Nonsmoker>Exsmoker
## Saccharibacteria_(TM7)_[G.3]            0.8898626 6.408683e-01 Exsmoker>Nonsmoker
## Saccharibacteria_(TM7)_[G.6]            1.4396161 4.868457e-01 Exsmoker>Nonsmoker
## Family.Saccharibacteria_(TM7)_[F-1].   0.4566712 7.958571e-01 Nonsmoker>Exsmoker
## Saccharibacteria_(TM7)_[G.5]            1.0808196 5.825095e-01 Exsmoker>Nonsmoker
## Treponema                              58.4612554 2.019760e-13 Exsmoker>Nonsmoker
## Fretibacterium                         48.2395645 3.348973e-11 Nonsmoker>Exsmoker
## Kingdom.Bacteria.                       6.6705008 3.560567e-02 Nonsmoker>Exsmoker
## Others                                  3.0647458 2.160225e-01 Nonsmoker>Exsmoker
##                                           pval.adj
## Absconditabacteria_(SR1)_[G.1]         1.000547e-09
## Actinomyces                            1.827004e-04
## Peptidiphaga                           4.543320e-04
## Schaalia                               4.040249e-03
## Family.Actinomycetaceae.               3.926221e-09
## Rothia                                 9.151790e-06
## Alloscardovia                          1.544443e-08
## Bifidobacterium                        1.827004e-04
## Parascardovia                          1.555336e-01
## Scardovia                              1.014378e-02
## Corynebacterium                        9.089357e-01
## Olsenella                              1.017521e-01
## Atopobium                              1.825187e-06
## Slackia                                4.366172e-04
## Cryptobacterium                        2.409012e-04
## Bacteroidetes_[G.3]                    1.003629e-10
## Bacteroidetes_[G.5]                    4.700591e-03
## Family.Bacteroidetes_[F-1].            4.440338e-02
## Bacteroidaceae_[G.1]                   2.409012e-04
## Bacteroidales_[G.2]                    4.019191e-11
## Porphyromonas                          7.006932e-04
## Tannerella                             4.168153e-01
## Alloprevotella                         1.042098e-01
## Prevotella                             4.835590e-01
## Bergeyella                             3.232185e-04
```

```
## Capnocytophaga                        1.158656e-02
## Gemella                               5.492798e-04
## Abiotrophia                           6.650553e-02
## Granulicatella                        5.613710e-04
## Lactobacillus                         5.600187e-12
## Streptococcus                         6.892889e-04
## Order.Lactobacillales.                1.000000e+00
## Class.Bacilli.                        1.206863e-01
## Clostridiales_[F.1][G-1]              8.451171e-04
## Butyrivibrio                          4.544849e-01
## Catonella                             1.179436e-07
## Johnsonella                           3.149409e-02
## Lachnoanaerobaculum                   3.632273e-01
## Lachnospiraceae_[G.2]                 4.040249e-03
## Lachnospiraceae_[G.3]                 8.965845e-03
## Lachnospiraceae_[G.7]                 7.660674e-13
## Lachnospiraceae_[G.8]                 6.907590e-02
## Oribacterium                          4.428827e-02
## Shuttleworthia                        4.406754e-02
## Stomatobaculum                        6.604580e-01
## Family.Lachnospiraceae_[XIV].         3.572341e-02
## Peptococcus                           3.906581e-04
## Parvimonas                            5.463042e-01
## Filifactor                            3.232185e-04
## Mogibacterium                         2.140460e-01
## Peptostreptococcaceae_[XI][G.1]       3.169573e-03
## Peptostreptococcaceae_[XI][G.4]       9.954434e-05
## Peptostreptococcaceae_[XI][G.5]       6.835522e-04
## Peptostreptococcaceae_[XI][G.6]       2.772868e-06
## Peptostreptococcaceae_[XI][G.7]       2.470983e-02
## Peptostreptococcaceae_[XI][G.9]       7.491053e-10
## Peptostreptococcus                    6.373636e-08
## Ruminococcaceae_[G.1]                 7.299352e-02
## Ruminococcaceae_[G.2]                 2.140460e-01
## Bulleidia                             6.340223e-06
## Eggerthia                             2.637172e-02
## Solobacterium                         8.864449e-01
## Mycoplasma                            9.954434e-05
## Mitsuokella                           2.454271e-01
## Selenomonas                           7.420139e-03
## Family.Selenomonadaceae.              1.042218e-04
## Class.Negativicutes.                  2.759154e-01
## Anaeroglobus                          1.131292e-01
## Dialister                             4.543320e-04
## Megasphaera                           2.191994e-02
## Family.Veillonellaceae.               3.287694e-04
## Veillonella                           1.924846e-01
## Veillonellaceae_[G.1]                 2.653745e-01
## Phylum.Firmicutes.                    5.039989e-01
## Fusobacterium                         3.682080e-06
## Leptotrichia                          1.016418e-04
## Gracilibacteria_(GN02)_[G.1]          1.614720e-02
## Lautropia                             4.604950e-08
## Ottowia                               5.357692e-02
```

```
## Eikenella                           1.836163e-01
## Kingella                            4.949661e-05
## Neisseria                           5.600187e-12
## Family.Neisseriaceae.               1.006356e-03
## Campylobacter                       8.052721e-02
## Cardiobacterium                     1.667296e-05
## Aggregatibacter                     8.138151e-06
## Haemophilus                         2.487598e-01
## Family.Pasteurellaceae.             2.140460e-01
## Saccharibacteria_(TM7)_[G.1]        3.382951e-01
## Saccharibacteria_(TM7)_[G.3]        6.684325e-01
## Saccharibacteria_(TM7)_[G.6]        5.306071e-01
## Family.Saccharibacteria_(TM7)_[F-1]. 8.212568e-01
## Saccharibacteria_(TM7)_[G.5]        6.209167e-01
## Treponema                           5.600187e-12
## Fretibacterium                      4.640720e-10
## Kingdom.Bacteria.                   5.482143e-02
## Others                              2.619272e-01
##                                                              Feature
## Absconditabacteria_(SR1)_[G.1]            Absconditabacteria_(SR1)_[G.1]
## Actinomyces                                                Actinomyces
## Peptidiphaga                                              Peptidiphaga
## Schaalia                                                      Schaalia
## Family.Actinomycetaceae.                        Family.Actinomycetaceae.
## Rothia                                                          Rothia
## Alloscardovia                                            Alloscardovia
## Bifidobacterium                                        Bifidobacterium
## Parascardovia                                            Parascardovia
## Scardovia                                                    Scardovia
## Corynebacterium                                        Corynebacterium
## Olsenella                                                    Olsenella
## Atopobium                                                    Atopobium
## Slackia                                                        Slackia
## Cryptobacterium                                        Cryptobacterium
## Bacteroidetes_[G.3]                                Bacteroidetes_[G.3]
## Bacteroidetes_[G.5]                                Bacteroidetes_[G.5]
## Family.Bacteroidetes_[F-1].                Family.Bacteroidetes_[F-1].
## Bacteroidaceae_[G.1]                              Bacteroidaceae_[G.1]
## Bacteroidales_[G.2]                                Bacteroidales_[G.2]
## Porphyromonas                                            Porphyromonas
## Tannerella                                                  Tannerella
## Alloprevotella                                          Alloprevotella
## Prevotella                                                  Prevotella
## Bergeyella                                                  Bergeyella
## Capnocytophaga                                          Capnocytophaga
## Gemella                                                        Gemella
## Abiotrophia                                                Abiotrophia
## Granulicatella                                          Granulicatella
## Lactobacillus                                            Lactobacillus
## Streptococcus                                            Streptococcus
## Order.Lactobacillales.                          Order.Lactobacillales.
## Class.Bacilli.                                          Class.Bacilli.
## Clostridiales_[F.1][G-1]                      Clostridiales_[F.1][G-1]
## Butyrivibrio                                              Butyrivibrio
```

```
## Catonella                                                     Catonella
## Johnsonella                                                 Johnsonella
## Lachnoanaerobaculum                                 Lachnoanaerobaculum
## Lachnospiraceae_[G.2]                             Lachnospiraceae_[G.2]
## Lachnospiraceae_[G.3]                             Lachnospiraceae_[G.3]
## Lachnospiraceae_[G.7]                             Lachnospiraceae_[G.7]
## Lachnospiraceae_[G.8]                             Lachnospiraceae_[G.8]
## Oribacterium                                               Oribacterium
## Shuttleworthia                                           Shuttleworthia
## Stomatobaculum                                           Stomatobaculum
## Family.Lachnospiraceae_[XIV].         Family.Lachnospiraceae_[XIV].
## Peptococcus                                                 Peptococcus
## Parvimonas                                                   Parvimonas
## Filifactor                                                   Filifactor
## Mogibacterium                                             Mogibacterium
## Peptostreptococcaceae_[XI][G.1]     Peptostreptococcaceae_[XI][G.1]
## Peptostreptococcaceae_[XI][G.4]     Peptostreptococcaceae_[XI][G.4]
## Peptostreptococcaceae_[XI][G.5]     Peptostreptococcaceae_[XI][G.5]
## Peptostreptococcaceae_[XI][G.6]     Peptostreptococcaceae_[XI][G.6]
## Peptostreptococcaceae_[XI][G.7]     Peptostreptococcaceae_[XI][G.7]
## Peptostreptococcaceae_[XI][G.9]     Peptostreptococcaceae_[XI][G.9]
## Peptostreptococcus                                   Peptostreptococcus
## Ruminococcaceae_[G.1]                             Ruminococcaceae_[G.1]
## Ruminococcaceae_[G.2]                             Ruminococcaceae_[G.2]
## Bulleidia                                                     Bulleidia
## Eggerthia                                                     Eggerthia
## Solobacterium                                             Solobacterium
## Mycoplasma                                                   Mycoplasma
## Mitsuokella                                                 Mitsuokella
## Selenomonas                                                 Selenomonas
## Family.Selenomonadaceae.                       Family.Selenomonadaceae.
## Class.Negativicutes.                               Class.Negativicutes.
## Anaeroglobus                                               Anaeroglobus
## Dialister                                                     Dialister
## Megasphaera                                                 Megasphaera
## Family.Veillonellaceae.                         Family.Veillonellaceae.
## Veillonella                                                 Veillonella
## Veillonellaceae_[G.1]                             Veillonellaceae_[G.1]
## Phylum.Firmicutes.                                   Phylum.Firmicutes.
## Fusobacterium                                             Fusobacterium
## Leptotrichia                                               Leptotrichia
## Gracilibacteria_(GN02)_[G.1]               Gracilibacteria_(GN02)_[G.1]
## Lautropia                                                     Lautropia
## Ottowia                                                         Ottowia
## Eikenella                                                     Eikenella
## Kingella                                                       Kingella
## Neisseria                                                     Neisseria
## Family.Neisseriaceae.                             Family.Neisseriaceae.
## Campylobacter                                             Campylobacter
## Cardiobacterium                                         Cardiobacterium
## Aggregatibacter                                         Aggregatibacter
## Haemophilus                                                 Haemophilus
## Family.Pasteurellaceae.                         Family.Pasteurellaceae.
## Saccharibacteria_(TM7)_[G.1]               Saccharibacteria_(TM7)_[G.1]
```

```
## Saccharibacteria_(TM7)_[G.3]                Saccharibacteria_(TM7)_[G.3]
## Saccharibacteria_(TM7)_[G.6]                Saccharibacteria_(TM7)_[G.6]
## Family.Saccharibacteria_(TM7)_[F-1]. Family.Saccharibacteria_(TM7)_[F-1].
## Saccharibacteria_(TM7)_[G.5]                Saccharibacteria_(TM7)_[G.5]
## Treponema                                                        Treponema
## Fretibacterium                                              Fretibacterium
## Kingdom.Bacteria.                                        Kingdom.Bacteria.
## Others                                                              Others
##                                                          Method
## Absconditabacteria_(SR1)_[G.1]       DESeq2 man. geoMeans (ds2)
## Actinomyces                          DESeq2 man. geoMeans (ds2)
## Peptidiphaga                         DESeq2 man. geoMeans (ds2)
## Schaalia                             DESeq2 man. geoMeans (ds2)
## Family.Actinomycetaceae.             DESeq2 man. geoMeans (ds2)
## Rothia                               DESeq2 man. geoMeans (ds2)
## Alloscardovia                        DESeq2 man. geoMeans (ds2)
## Bifidobacterium                      DESeq2 man. geoMeans (ds2)
## Parascardovia                        DESeq2 man. geoMeans (ds2)
## Scardovia                            DESeq2 man. geoMeans (ds2)
## Corynebacterium                      DESeq2 man. geoMeans (ds2)
## Olsenella                            DESeq2 man. geoMeans (ds2)
## Atopobium                            DESeq2 man. geoMeans (ds2)
## Slackia                              DESeq2 man. geoMeans (ds2)
## Cryptobacterium                      DESeq2 man. geoMeans (ds2)
## Bacteroidetes_[G.3]                  DESeq2 man. geoMeans (ds2)
## Bacteroidetes_[G.5]                  DESeq2 man. geoMeans (ds2)
## Family.Bacteroidetes_[F-1].          DESeq2 man. geoMeans (ds2)
## Bacteroidaceae_[G.1]                 DESeq2 man. geoMeans (ds2)
## Bacteroidales_[G.2]                  DESeq2 man. geoMeans (ds2)
## Porphyromonas                        DESeq2 man. geoMeans (ds2)
## Tannerella                           DESeq2 man. geoMeans (ds2)
## Alloprevotella                       DESeq2 man. geoMeans (ds2)
## Prevotella                           DESeq2 man. geoMeans (ds2)
## Bergeyella                           DESeq2 man. geoMeans (ds2)
## Capnocytophaga                       DESeq2 man. geoMeans (ds2)
## Gemella                              DESeq2 man. geoMeans (ds2)
## Abiotrophia                          DESeq2 man. geoMeans (ds2)
## Granulicatella                       DESeq2 man. geoMeans (ds2)
## Lactobacillus                        DESeq2 man. geoMeans (ds2)
## Streptococcus                        DESeq2 man. geoMeans (ds2)
## Order.Lactobacillales.               DESeq2 man. geoMeans (ds2)
## Class.Bacilli.                       DESeq2 man. geoMeans (ds2)
## Clostridiales_[F.1][G-1]             DESeq2 man. geoMeans (ds2)
## Butyrivibrio                         DESeq2 man. geoMeans (ds2)
## Catonella                            DESeq2 man. geoMeans (ds2)
## Johnsonella                          DESeq2 man. geoMeans (ds2)
## Lachnoanaerobaculum                  DESeq2 man. geoMeans (ds2)
## Lachnospiraceae_[G.2]                DESeq2 man. geoMeans (ds2)
## Lachnospiraceae_[G.3]                DESeq2 man. geoMeans (ds2)
## Lachnospiraceae_[G.7]                DESeq2 man. geoMeans (ds2)
## Lachnospiraceae_[G.8]                DESeq2 man. geoMeans (ds2)
## Oribacterium                         DESeq2 man. geoMeans (ds2)
## Shuttleworthia                       DESeq2 man. geoMeans (ds2)
## Stomatobaculum                       DESeq2 man. geoMeans (ds2)
```

```
## Family.Lachnospiraceae_[XIV].        DESeq2 man. geoMeans (ds2)
## Peptococcus                          DESeq2 man. geoMeans (ds2)
## Parvimonas                           DESeq2 man. geoMeans (ds2)
## Filifactor                           DESeq2 man. geoMeans (ds2)
## Mogibacterium                        DESeq2 man. geoMeans (ds2)
## Peptostreptococcaceae_[XI][G.1]      DESeq2 man. geoMeans (ds2)
## Peptostreptococcaceae_[XI][G.4]      DESeq2 man. geoMeans (ds2)
## Peptostreptococcaceae_[XI][G.5]      DESeq2 man. geoMeans (ds2)
## Peptostreptococcaceae_[XI][G.6]      DESeq2 man. geoMeans (ds2)
## Peptostreptococcaceae_[XI][G.7]      DESeq2 man. geoMeans (ds2)
## Peptostreptococcaceae_[XI][G.9]      DESeq2 man. geoMeans (ds2)
## Peptostreptococcus                   DESeq2 man. geoMeans (ds2)
## Ruminococcaceae_[G.1]                DESeq2 man. geoMeans (ds2)
## Ruminococcaceae_[G.2]                DESeq2 man. geoMeans (ds2)
## Bulleidia                            DESeq2 man. geoMeans (ds2)
## Eggerthia                            DESeq2 man. geoMeans (ds2)
## Solobacterium                        DESeq2 man. geoMeans (ds2)
## Mycoplasma                           DESeq2 man. geoMeans (ds2)
## Mitsuokella                          DESeq2 man. geoMeans (ds2)
## Selenomonas                          DESeq2 man. geoMeans (ds2)
## Family.Selenomonadaceae.             DESeq2 man. geoMeans (ds2)
## Class.Negativicutes.                 DESeq2 man. geoMeans (ds2)
## Anaeroglobus                         DESeq2 man. geoMeans (ds2)
## Dialister                            DESeq2 man. geoMeans (ds2)
## Megasphaera                          DESeq2 man. geoMeans (ds2)
## Family.Veillonellaceae.              DESeq2 man. geoMeans (ds2)
## Veillonella                          DESeq2 man. geoMeans (ds2)
## Veillonellaceae_[G.1]                DESeq2 man. geoMeans (ds2)
## Phylum.Firmicutes.                   DESeq2 man. geoMeans (ds2)
## Fusobacterium                        DESeq2 man. geoMeans (ds2)
## Leptotrichia                         DESeq2 man. geoMeans (ds2)
## Gracilibacteria_(GN02)_[G.1]         DESeq2 man. geoMeans (ds2)
## Lautropia                            DESeq2 man. geoMeans (ds2)
## Ottowia                              DESeq2 man. geoMeans (ds2)
## Eikenella                            DESeq2 man. geoMeans (ds2)
## Kingella                             DESeq2 man. geoMeans (ds2)
## Neisseria                            DESeq2 man. geoMeans (ds2)
## Family.Neisseriaceae.                DESeq2 man. geoMeans (ds2)
## Campylobacter                        DESeq2 man. geoMeans (ds2)
## Cardiobacterium                      DESeq2 man. geoMeans (ds2)
## Aggregatibacter                      DESeq2 man. geoMeans (ds2)
## Haemophilus                          DESeq2 man. geoMeans (ds2)
## Family.Pasteurellaceae.              DESeq2 man. geoMeans (ds2)
## Saccharibacteria_(TM7)_[G.1]         DESeq2 man. geoMeans (ds2)
## Saccharibacteria_(TM7)_[G.3]         DESeq2 man. geoMeans (ds2)
## Saccharibacteria_(TM7)_[G.6]         DESeq2 man. geoMeans (ds2)
## Family.Saccharibacteria_(TM7)_[F-1]. DESeq2 man. geoMeans (ds2)
## Saccharibacteria_(TM7)_[G.5]         DESeq2 man. geoMeans (ds2)
## Treponema                            DESeq2 man. geoMeans (ds2)
## Fretibacterium                       DESeq2 man. geoMeans (ds2)
## Kingdom.Bacteria.                    DESeq2 man. geoMeans (ds2)
## Others                               DESeq2 man. geoMeans (ds2)
```

```
write.table(final, file="Smoking_ds2.txt", sep="\t", dec=",", row.names=F)
write.table(final[final$pval<0.05,], file="Smoking_ds2_sig.txt", sep="\t", dec=",", row.names=F)
final <- DA.kru(Microbio2, predictor = Phe2$Smoking)
##kable(final[,1:10])
final
```

```
##                                             pval     pval.adj
## Absconditabacteria_(SR1)_[G.1]      6.016788e-03 1.101186e-02
## Actinomyces                         8.923618e-05 2.623003e-04
## Peptidiphaga                        2.670431e-06 1.295159e-05
## Schaalia                            5.401477e-05 1.637323e-04
## Family.Actinomycetaceae.            7.913267e-12 1.535174e-10
## Rothia                              5.682416e-08 4.239957e-07
## Alloscardovia                       2.149890e-10 3.475656e-09
## Bifidobacterium                     7.682356e-07 4.139936e-06
## Parascardovia                       4.578680e-02 7.049714e-02
## Scardovia                           7.272448e-01 7.751950e-01
## Corynebacterium                     9.170274e-01 9.265798e-01
## Olsenella                           4.357504e-01 5.101309e-01
## Atopobium                           6.727318e-07 3.880255e-06
## Slackia                             4.406396e-04 1.017668e-03
## Cryptobacterium                     3.412012e-05 1.182018e-04
## Bacteroidetes_[G.3]                 9.248374e-10 1.096839e-08
## Bacteroidetes_[G.5]                 1.151931e-01 1.619381e-01
## Family.Bacteroidetes_[F-1].         3.624467e-02 5.763497e-02
## Bacteroidaceae_[G.1]                1.634358e-03 3.522949e-03
## Bacteroidales_[G.2]                 2.318402e-04 5.622124e-04
## Porphyromonas                       1.596249e-04 4.074635e-04
## Tannerella                          6.489402e-01 7.319442e-01
## Alloprevotella                      4.874936e-02 7.388575e-02
## Prevotella                          1.787446e-01 2.375100e-01
## Bergeyella                          4.836918e-06 2.039918e-05
## Capnocytophaga                      3.999701e-07 2.730067e-06
## Gemella                             1.381795e-06 7.054429e-06
## Abiotrophia                         1.017686e-09 1.096839e-08
## Granulicatella                      7.576902e-03 1.361036e-02
## Lactobacillus                       6.800446e-07 3.880255e-06
## Streptococcus                       9.110004e-06 3.534682e-05
## Order.Lactobacillales.              9.131400e-01 9.265798e-01
## Class.Bacilli.                      3.513679e-01 4.260336e-01
## Clostridiales_[F.1][G-1]            1.445440e-04 3.789397e-04
## Butyrivibrio                        4.495095e-01 5.190764e-01
## Catonella                           2.085192e-09 2.022637e-08
## Johnsonella                         1.397182e-01 1.936095e-01
## Lachnoanaerobaculum                 2.342860e-01 2.990229e-01
## Lachnospiraceae_[G.2]               4.319221e-03 8.728426e-03
## Lachnospiraceae_[G.3]               5.879437e-04 1.326291e-03
## Lachnospiraceae_[G.7]               4.332835e-12 1.063279e-10
## Lachnospiraceae_[G.8]               1.580350e-01 2.159070e-01
## Oribacterium                        1.542018e-02 2.535183e-02
## Shuttleworthia                      1.370062e-02 2.291310e-02
## Stomatobaculum                      7.077563e-01 7.628041e-01
## Family.Lachnospiraceae_[XIV].       8.701102e-01 9.173988e-01
```

```
## Peptococcus                          1.982785e-04 4.931542e-04
## Parvimonas                          2.270215e-01 2.936145e-01
## Filifactor                          5.296131e-03 1.007303e-02
## Mogibacterium                       4.558941e-02 7.049714e-02
## Peptostreptococcaceae_[XI][G.1]     1.342557e-04 3.720800e-04
## Peptostreptococcaceae_[XI][G.4]     4.892309e-05 1.581847e-04
## Peptostreptococcaceae_[XI][G.5]     3.335396e-03 6.883690e-03
## Peptostreptococcaceae_[XI][G.6]     3.954472e-05 1.322703e-04
## Peptostreptococcaceae_[XI][G.7]     4.680242e-01 5.340982e-01
## Peptostreptococcaceae_[XI][G.9]     3.665950e-06 1.616351e-05
## Peptostreptococcus                  7.149200e-10 9.906749e-09
## Ruminococcaceae_[G.1]               2.576501e-03 5.433056e-03
## Ruminococcaceae_[G.2]               1.697361e-01 2.286722e-01
## Bulleidia                           1.185926e-05 4.424417e-05
## Eggerthia                           6.829623e-02 1.003748e-01
## Solobacterium                       9.063518e-01 9.265798e-01
## Mycoplasma                          6.862233e-04 1.512810e-03
## Mitsuokella                         6.682041e-02 9.971661e-02
## Selenomonas                         1.094610e-02 1.896021e-02
## Family.Selenomonadaceae.            1.292705e-02 2.199867e-02
## Class.Negativicutes.                6.893967e-01 7.599032e-01
## Anaeroglobus                        3.185576e-01 3.911404e-01
## Dialister                           4.092570e-04 9.682422e-04
## Megasphaera                         5.076170e-03 9.847769e-03
## Family.Veillonellaceae.             5.108115e-05 1.598346e-04
## Veillonella                         4.872323e-03 9.645211e-03
## Veillonellaceae_[G.1]               6.983202e-01 7.610906e-01
## Phylum.Firmicutes.                  2.755938e-01 3.427256e-01
## Fusobacterium                       7.900723e-06 3.193209e-05
## Leptotrichia                        2.639686e-05 9.483316e-05
## Gracilibacteria_(GN02)_[G.1]        5.938748e-03 1.101186e-02
## Lautropia                           1.017275e-16 4.933783e-15
## Ottowia                             9.924522e-05 2.831408e-04
## Eikenella                           8.968257e-02 1.298389e-01
## Kingella                            4.384655e-12 1.063279e-10
## Neisseria                           3.179603e-19 3.084214e-17
## Family.Neisseriaceae.               4.221754e-07 2.730067e-06
## Campylobacter                       8.361429e-03 1.474652e-02
## Cardiobacterium                     8.478889e-09 7.476838e-08
## Aggregatibacter                     2.780206e-08 2.247333e-07
## Haemophilus                         2.979463e-02 4.816799e-02
## Family.Pasteurellaceae.             4.365038e-01 5.101309e-01
## Saccharibacteria_(TM7)_[G.1]        2.566536e-01 3.233169e-01
## Saccharibacteria_(TM7)_[G.3]        9.161724e-01 9.265798e-01
## Saccharibacteria_(TM7)_[G.6]        2.102851e-01 2.756440e-01
## Family.Saccharibacteria_(TM7)_[F-1]. 9.848806e-01 9.848806e-01
## Saccharibacteria_(TM7)_[G.5]        3.871403e-01 4.636124e-01
## Treponema                           1.412473e-04 3.789397e-04
## Fretibacterium                      3.153794e-06 1.456753e-05
## Kingdom.Bacteria.                   1.085972e-01 1.549107e-01
## Others                              6.693069e-01 7.462387e-01
##                                                               Feature
## Absconditabacteria_(SR1)_[G.1]           Absconditabacteria_(SR1)_[G.1]
## Actinomyces                                                Actinomyces
```

```
## Peptidiphaga                                                     Peptidiphaga
## Schaalia                                                             Schaalia
## Family.Actinomycetaceae.                             Family.Actinomycetaceae.
## Rothia                                                                 Rothia
## Alloscardovia                                                   Alloscardovia
## Bifidobacterium                                               Bifidobacterium
## Parascardovia                                                   Parascardovia
## Scardovia                                                           Scardovia
## Corynebacterium                                               Corynebacterium
## Olsenella                                                           Olsenella
## Atopobium                                                           Atopobium
## Slackia                                                               Slackia
## Cryptobacterium                                               Cryptobacterium
## Bacteroidetes_[G.3]                                       Bacteroidetes_[G.3]
## Bacteroidetes_[G.5]                                       Bacteroidetes_[G.5]
## Family.Bacteroidetes_[F-1].                       Family.Bacteroidetes_[F-1].
## Bacteroidaceae_[G.1]                                     Bacteroidaceae_[G.1]
## Bacteroidales_[G.2]                                       Bacteroidales_[G.2]
## Porphyromonas                                                   Porphyromonas
## Tannerella                                                         Tannerella
## Alloprevotella                                                 Alloprevotella
## Prevotella                                                         Prevotella
## Bergeyella                                                         Bergeyella
## Capnocytophaga                                                 Capnocytophaga
## Gemella                                                               Gemella
## Abiotrophia                                                       Abiotrophia
## Granulicatella                                                 Granulicatella
## Lactobacillus                                                   Lactobacillus
## Streptococcus                                                   Streptococcus
## Order.Lactobacillales.                                 Order.Lactobacillales.
## Class.Bacilli.                                                 Class.Bacilli.
## Clostridiales_[F.1][G-1]                             Clostridiales_[F.1][G-1]
## Butyrivibrio                                                     Butyrivibrio
## Catonella                                                           Catonella
## Johnsonella                                                       Johnsonella
## Lachnoanaerobaculum                                       Lachnoanaerobaculum
## Lachnospiraceae_[G.2]                                   Lachnospiraceae_[G.2]
## Lachnospiraceae_[G.3]                                   Lachnospiraceae_[G.3]
## Lachnospiraceae_[G.7]                                   Lachnospiraceae_[G.7]
## Lachnospiraceae_[G.8]                                   Lachnospiraceae_[G.8]
## Oribacterium                                                     Oribacterium
## Shuttleworthia                                                 Shuttleworthia
## Stomatobaculum                                                 Stomatobaculum
## Family.Lachnospiraceae_[XIV].                   Family.Lachnospiraceae_[XIV].
## Peptococcus                                                       Peptococcus
## Parvimonas                                                         Parvimonas
## Filifactor                                                         Filifactor
## Mogibacterium                                                   Mogibacterium
## Peptostreptococcaceae_[XI][G.1]               Peptostreptococcaceae_[XI][G.1]
## Peptostreptococcaceae_[XI][G.4]               Peptostreptococcaceae_[XI][G.4]
## Peptostreptococcaceae_[XI][G.5]               Peptostreptococcaceae_[XI][G.5]
## Peptostreptococcaceae_[XI][G.6]               Peptostreptococcaceae_[XI][G.6]
## Peptostreptococcaceae_[XI][G.7]               Peptostreptococcaceae_[XI][G.7]
## Peptostreptococcaceae_[XI][G.9]               Peptostreptococcaceae_[XI][G.9]
```

```
## Peptostreptococcus                                            Peptostreptococcus
## Ruminococcaceae_[G.1]                                      Ruminococcaceae_[G.1]
## Ruminococcaceae_[G.2]                                      Ruminococcaceae_[G.2]
## Bulleidia                                                              Bulleidia
## Eggerthia                                                              Eggerthia
## Solobacterium                                                      Solobacterium
## Mycoplasma                                                            Mycoplasma
## Mitsuokella                                                          Mitsuokella
## Selenomonas                                                          Selenomonas
## Family.Selenomonadaceae.                                Family.Selenomonadaceae.
## Class.Negativicutes.                                        Class.Negativicutes.
## Anaeroglobus                                                        Anaeroglobus
## Dialister                                                              Dialister
## Megasphaera                                                          Megasphaera
## Family.Veillonellaceae.                                  Family.Veillonellaceae.
## Veillonella                                                          Veillonella
## Veillonellaceae_[G.1]                                      Veillonellaceae_[G.1]
## Phylum.Firmicutes.                                            Phylum.Firmicutes.
## Fusobacterium                                                      Fusobacterium
## Leptotrichia                                                        Leptotrichia
## Gracilibacteria_(GN02)_[G.1]                        Gracilibacteria_(GN02)_[G.1]
## Lautropia                                                              Lautropia
## Ottowia                                                                  Ottowia
## Eikenella                                                              Eikenella
## Kingella                                                                Kingella
## Neisseria                                                              Neisseria
## Family.Neisseriaceae.                                      Family.Neisseriaceae.
## Campylobacter                                                      Campylobacter
## Cardiobacterium                                                  Cardiobacterium
## Aggregatibacter                                                  Aggregatibacter
## Haemophilus                                                          Haemophilus
## Family.Pasteurellaceae.                                  Family.Pasteurellaceae.
## Saccharibacteria_(TM7)_[G.1]                        Saccharibacteria_(TM7)_[G.1]
## Saccharibacteria_(TM7)_[G.3]                        Saccharibacteria_(TM7)_[G.3]
## Saccharibacteria_(TM7)_[G.6]                        Saccharibacteria_(TM7)_[G.6]
## Family.Saccharibacteria_(TM7)_[F-1]. Family.Saccharibacteria_(TM7)_[F-1].
## Saccharibacteria_(TM7)_[G.5]                        Saccharibacteria_(TM7)_[G.5]
## Treponema                                                              Treponema
## Fretibacterium                                                    Fretibacterium
## Kingdom.Bacteria.                                              Kingdom.Bacteria.
## Others                                                                    Others
##                                                Method
## Absconditabacteria_(SR1)_[G.1]       Kruskal-Wallis (kru)
## Actinomyces                          Kruskal-Wallis (kru)
## Peptidiphaga                         Kruskal-Wallis (kru)
## Schaalia                             Kruskal-Wallis (kru)
## Family.Actinomycetaceae.             Kruskal-Wallis (kru)
## Rothia                               Kruskal-Wallis (kru)
## Alloscardovia                        Kruskal-Wallis (kru)
## Bifidobacterium                      Kruskal-Wallis (kru)
## Parascardovia                        Kruskal-Wallis (kru)
## Scardovia                            Kruskal-Wallis (kru)
## Corynebacterium                      Kruskal-Wallis (kru)
## Olsenella                            Kruskal-Wallis (kru)
```

```
## Atopobium                              Kruskal-Wallis (kru)
## Slackia                                Kruskal-Wallis (kru)
## Cryptobacterium                        Kruskal-Wallis (kru)
## Bacteroidetes_[G.3]                    Kruskal-Wallis (kru)
## Bacteroidetes_[G.5]                    Kruskal-Wallis (kru)
## Family.Bacteroidetes_[F-1].            Kruskal-Wallis (kru)
## Bacteroidaceae_[G.1]                   Kruskal-Wallis (kru)
## Bacteroidales_[G.2]                    Kruskal-Wallis (kru)
## Porphyromonas                          Kruskal-Wallis (kru)
## Tannerella                             Kruskal-Wallis (kru)
## Alloprevotella                         Kruskal-Wallis (kru)
## Prevotella                             Kruskal-Wallis (kru)
## Bergeyella                             Kruskal-Wallis (kru)
## Capnocytophaga                         Kruskal-Wallis (kru)
## Gemella                                Kruskal-Wallis (kru)
## Abiotrophia                            Kruskal-Wallis (kru)
## Granulicatella                         Kruskal-Wallis (kru)
## Lactobacillus                          Kruskal-Wallis (kru)
## Streptococcus                          Kruskal-Wallis (kru)
## Order.Lactobacillales.                 Kruskal-Wallis (kru)
## Class.Bacilli.                         Kruskal-Wallis (kru)
## Clostridiales_[F.1][G-1]               Kruskal-Wallis (kru)
## Butyrivibrio                           Kruskal-Wallis (kru)
## Catonella                              Kruskal-Wallis (kru)
## Johnsonella                            Kruskal-Wallis (kru)
## Lachnoanaerobaculum                    Kruskal-Wallis (kru)
## Lachnospiraceae_[G.2]                  Kruskal-Wallis (kru)
## Lachnospiraceae_[G.3]                  Kruskal-Wallis (kru)
## Lachnospiraceae_[G.7]                  Kruskal-Wallis (kru)
## Lachnospiraceae_[G.8]                  Kruskal-Wallis (kru)
## Oribacterium                           Kruskal-Wallis (kru)
## Shuttleworthia                         Kruskal-Wallis (kru)
## Stomatobaculum                         Kruskal-Wallis (kru)
## Family.Lachnospiraceae_[XIV].          Kruskal-Wallis (kru)
## Peptococcus                            Kruskal-Wallis (kru)
## Parvimonas                             Kruskal-Wallis (kru)
## Filifactor                             Kruskal-Wallis (kru)
## Mogibacterium                          Kruskal-Wallis (kru)
## Peptostreptococcaceae_[XI][G.1]        Kruskal-Wallis (kru)
## Peptostreptococcaceae_[XI][G.4]        Kruskal-Wallis (kru)
## Peptostreptococcaceae_[XI][G.5]        Kruskal-Wallis (kru)
## Peptostreptococcaceae_[XI][G.6]        Kruskal-Wallis (kru)
## Peptostreptococcaceae_[XI][G.7]        Kruskal-Wallis (kru)
## Peptostreptococcaceae_[XI][G.9]        Kruskal-Wallis (kru)
## Peptostreptococcus                     Kruskal-Wallis (kru)
## Ruminococcaceae_[G.1]                  Kruskal-Wallis (kru)
## Ruminococcaceae_[G.2]                  Kruskal-Wallis (kru)
## Bulleidia                              Kruskal-Wallis (kru)
## Eggerthia                              Kruskal-Wallis (kru)
## Solobacterium                          Kruskal-Wallis (kru)
## Mycoplasma                             Kruskal-Wallis (kru)
## Mitsuokella                            Kruskal-Wallis (kru)
## Selenomonas                            Kruskal-Wallis (kru)
## Family.Selenomonadaceae.               Kruskal-Wallis (kru)
```

```
## Class.Negativicutes.                  Kruskal-Wallis (kru)
## Anaeroglobus                          Kruskal-Wallis (kru)
## Dialister                             Kruskal-Wallis (kru)
## Megasphaera                           Kruskal-Wallis (kru)
## Family.Veillonellaceae.               Kruskal-Wallis (kru)
## Veillonella                           Kruskal-Wallis (kru)
## Veillonellaceae_[G.1]                 Kruskal-Wallis (kru)
## Phylum.Firmicutes.                    Kruskal-Wallis (kru)
## Fusobacterium                         Kruskal-Wallis (kru)
## Leptotrichia                          Kruskal-Wallis (kru)
## Gracilibacteria_(GN02)_[G.1]          Kruskal-Wallis (kru)
## Lautropia                             Kruskal-Wallis (kru)
## Ottowia                               Kruskal-Wallis (kru)
## Eikenella                             Kruskal-Wallis (kru)
## Kingella                              Kruskal-Wallis (kru)
## Neisseria                             Kruskal-Wallis (kru)
## Family.Neisseriaceae.                 Kruskal-Wallis (kru)
## Campylobacter                         Kruskal-Wallis (kru)
## Cardiobacterium                       Kruskal-Wallis (kru)
## Aggregatibacter                       Kruskal-Wallis (kru)
## Haemophilus                           Kruskal-Wallis (kru)
## Family.Pasteurellaceae.               Kruskal-Wallis (kru)
## Saccharibacteria_(TM7)_[G.1]          Kruskal-Wallis (kru)
## Saccharibacteria_(TM7)_[G.3]          Kruskal-Wallis (kru)
## Saccharibacteria_(TM7)_[G.6]          Kruskal-Wallis (kru)
## Family.Saccharibacteria_(TM7)_[F-1]. Kruskal-Wallis (kru)
## Saccharibacteria_(TM7)_[G.5]          Kruskal-Wallis (kru)
## Treponema                             Kruskal-Wallis (kru)
## Fretibacterium                        Kruskal-Wallis (kru)
## Kingdom.Bacteria.                     Kruskal-Wallis (kru)
## Others                                Kruskal-Wallis (kru)
```

```
write.table(final, file="Smoking_kru.txt", sep="\t", dec=",", row.names=F)
write.table(final[final$pval<0.05,], file="Smoking_kru_sig.txt", sep="\t", dec=",", row.names=F)
#################gly_stat
table(Phe$gly_stat, useNA="always")
```

```
##
##    0    1    2    3    4    5    6 <NA>
##    3  421  112   58   63   68   21    0
```

```
#Only includes rows with risk groups and low risk
Phe2<-subset(Phe, gly_stat %in% c(1:5))
Phe2$gly_stat<-droplevels(Phe2$gly_stat)
#Also subset columns
Microbio2<-dplyr::select(Microbio, one_of(Phe2$IDX))
final <- DA.ds2(Microbio2, predictor = as.factor(Phe2$gly_stat), out.all=TRUE) #It is LRT so don't look
final
```

```
##                                          baseMean log2FoldChange      lfcSE
## Absconditabacteria_(SR1)_[G.1]         66.7673997    0.056805534 0.35604247
## Actinomyces                           324.2145221   -0.021146139 0.12104582
## Peptidiphaga                           21.8070254    0.059981960 0.17053353
```

```
## Schaalia                              700.4515512  0.024707001 0.09932797
## Family.Actinomycetaceae.                5.3549126 -0.028036394 0.25115739
## Rothia                               1945.8951087  0.098979755 0.11726619
## Alloscardovia                          12.3058511 -0.119483015 0.30565018
## Bifidobacterium                        12.7731806  0.459896686 0.34316575
## Parascardovia                           2.9990083  0.838492447 0.45358924
## Scardovia                              13.3462660 -0.177372283 0.29341634
## Corynebacterium                        93.4333155  0.245035699 0.12758057
## Olsenella                               1.5645842  0.078143840 0.28108351
## Atopobium                             120.7894166 -0.047220106 0.11688106
## Slackia                                 1.3952328  0.163522941 0.27199568
## Cryptobacterium                         4.2229867  0.355067160 0.28565748
## Bacteroidetes_[G.3]                     6.4900827 -0.218723563 0.30376062
## Bacteroidetes_[G.5]                     4.5443282 -0.621680900 0.33673048
## Family.Bacteroidetes_[F-1].             0.8440599 -0.921198636 0.49179572
## Bacteroidaceae_[G.1]                    0.7927548  1.267425831 0.44470652
## Bacteroidales_[G.2]                    12.0958316  0.193580802 0.17402194
## Porphyromonas                         674.8647664  0.039618062 0.15970489
## Tannerella                             21.8833160 -0.135040494 0.11846958
## Alloprevotella                        363.2895934  0.057079392 0.12682312
## Prevotella                           3825.2557819 -0.037279225 0.08065964
## Bergeyella                             72.7009790 -0.039552540 0.12390566
## Capnocytophaga                        225.1643864 -0.014745344 0.13244868
## Gemella                               308.9669193  0.115132132 0.11115496
## Abiotrophia                            30.0443330  0.133445958 0.23842572
## Granulicatella                        306.4850456  0.062375003 0.09612814
## Lactobacillus                          19.1183009  0.360649853 0.35295531
## Streptococcus                        3824.6130729 -0.013730113 0.09159795
## Order.Lactobacillales.                  0.2873798  0.026851015 0.33632506
## Class.Bacilli.                          0.5789536  0.029312805 0.26576520
## Clostridiales_[F.1][G-1]                1.0076262 -0.139468082 0.40260101
## Butyrivibrio                            6.9558967 -0.751710652 0.25665832
## Catonella                              36.5379681  0.077111075 0.14329035
## Johnsonella                             0.8914323  0.203339362 0.34838183
## Lachnoanaerobaculum                    87.7547810 -0.179493898 0.09965950
## Lachnospiraceae_[G.2]                  28.1084549 -0.542384705 0.26384674
## Lachnospiraceae_[G.3]                   2.8328544 -0.235136530 0.27498793
## Lachnospiraceae_[G.7]                   1.4579041  0.232932143 0.39224234
## Lachnospiraceae_[G.8]                   1.3988933 -0.218929776 0.42650324
## Oribacterium                          146.7103250 -0.063746533 0.11125229
## Shuttleworthia                          2.8423211  0.312472866 0.30382304
## Stomatobaculum                        112.6897043 -0.120921430 0.12531868
## Family.Lachnospiraceae_[XIV].           1.4111153 -1.001362515 0.35126930
## Peptococcus                             3.6055204 -0.146981092 0.31349900
## Parvimonas                             40.6411433 -0.053564138 0.15419299
## Filifactor                              6.6654780 -0.605551679 0.30353376
## Mogibacterium                          41.1748713 -0.040689955 0.10652247
## Peptostreptococcaceae_[XI][G.1]        67.6290897 -0.162005974 0.19047981
## Peptostreptococcaceae_[XI][G.4]         0.3957527  0.123553781 0.42040428
## Peptostreptococcaceae_[XI][G.5]         1.6496546 -0.649970371 0.43955084
## Peptostreptococcaceae_[XI][G.6]         0.9654537 -0.524785623 0.43521417
## Peptostreptococcaceae_[XI][G.7]         4.6973679  0.237208197 0.29474995
## Peptostreptococcaceae_[XI][G.9]         8.4373976  0.019759342 0.17464525
## Peptostreptococcus                     42.1549900 -0.072688473 0.22297706
```

```
## Ruminococcaceae_[G.1]                     25.0346257    0.085762207 0.22711740
## Ruminococcaceae_[G.2]                     41.5306426   -0.015506836 0.18847371
## Bulleidia                                  3.6956815   -0.128712656 0.33300017
## Eggerthia                                  1.5610026   -0.043570492 0.44424802
## Solobacterium                             73.8194362   -0.247651314 0.12465031
## Mycoplasma                                 4.4517892   -0.168757176 0.26827084
## Mitsuokella                               27.3626869    0.052819132 0.23723551
## Selenomonas                              222.8112213   -0.235363731 0.10695229
## Family.Selenomonadaceae.                   5.0846973   -0.282990020 0.27737589
## Class.Negativicutes.                       0.7328303   -0.401274747 0.26575402
## Anaeroglobus                               7.6738251    0.211085272 0.24157536
## Dialister                                 36.2070092    0.024113425 0.13072938
## Megasphaera                              409.5436984   -0.085933023 0.13795763
## Family.Veillonellaceae.                    0.5271752    0.124506041 0.30000180
## Veillonella                             5024.6971995   -0.049812525 0.07574710
## Veillonellaceae_[G.1]                      6.0232614    0.366677335 0.21297177
## Phylum.Firmicutes.                         0.8529235    0.086923283 0.34358406
## Fusobacterium                            915.0726073   -0.162662358 0.10319943
## Leptotrichia                             687.2657246   -0.230327920 0.10594828
## Gracilibacteria_(GN02)_[G.1]               2.4187883   -0.626271707 0.34397206
## Lautropia                                 68.8304165    0.175721859 0.19673534
## Ottowia                                    2.3471068    0.478982029 0.43598734
## Eikenella                                  8.5319122   -0.071692271 0.16293030
## Kingella                                 100.8731479    0.214050596 0.14094399
## Neisseria                               1333.2266191    0.016295562 0.16234006
## Family.Neisseriaceae.                      5.6579594    0.582767649 0.36915577
## Campylobacter                            259.3835376   -0.107389210 0.09155752
## Cardiobacterium                           14.7423032    0.190809953 0.14153856
## Aggregatibacter                          277.1805098   -0.064852654 0.18789742
## Haemophilus                             2911.4822527   -0.002667696 0.12491861
## Family.Pasteurellaceae.                    2.4025379   -0.037806031 0.51502064
## Saccharibacteria_(TM7)_[G.1]              34.7344972    0.252273724 0.20666024
## Saccharibacteria_(TM7)_[G.3]               7.1544682    0.627691190 0.28988014
## Saccharibacteria_(TM7)_[G.6]               7.3635144    0.633774967 0.36425453
## Family.Saccharibacteria_(TM7)_[F-1].       1.1063076    0.340350374 0.45570008
## Saccharibacteria_(TM7)_[G.5]               0.9747886    0.492254844 0.44313185
## Treponema                                 61.7408338   -0.194896354 0.19696243
## Fretibacterium                             8.1453806   -0.054773644 0.23985559
## Kingdom.Bacteria.                          9.2099049   -0.415767901 0.30480630
## Others                                    32.9571263    0.227281085 0.20098806
##                                             stat         pval ordering
## Absconditabacteria_(SR1)_[G.1]       20.1893572 0.0004581962      2>1
## Actinomyces                          12.0757736 0.0167965994      1>2
## Peptidiphaga                          5.4758351 0.2418617747      2>1
## Schaalia                              9.4289272 0.0512283001      2>1
## Family.Actinomycetaceae.              1.6931492 0.7919616957      1>2
## Rothia                                5.6702680 0.2251632414      2>1
## Alloscardovia                         1.2943373 0.8623355609      1>2
## Bifidobacterium                       7.1041787 0.1304842161      2>1
## Parascardovia                        14.8061104 0.0051207219      2>1
## Scardovia                             4.6672453 0.3231744316      1>2
## Corynebacterium                       8.9697840 0.0618591906      2>1
## Olsenella                             2.2207951 0.6952239772      2>1
## Atopobium                             2.8992660 0.5748220451      1>2
```

```
## Slackia                              1.9545446 0.7441192169       2>1
## Cryptobacterium                      2.8276788 0.5870643706       2>1
## Bacteroidetes_[G.3]                  5.7179947 0.2212216918       1>2
## Bacteroidetes_[G.5]                  8.2640402 0.0823710915       1>2
## Family.Bacteroidetes_[F-1].         10.0003893 0.0404211251       1>2
## Bacteroidaceae_[G.1]                13.0334895 0.0111133125       2>1
## Bacteroidales_[G.2]                  2.5360629 0.6381900245       2>1
## Porphyromonas                        5.3600731 0.2523056946       2>1
## Tannerella                           2.0965871 0.7179994138       1>2
## Alloprevotella                       2.1373561 0.7105128245       2>1
## Prevotella                           4.8789991 0.2999397796       1>2
## Bergeyella                           6.9605265 0.1379889808       1>2
## Capnocytophaga                       6.8806924 0.1423290186       1>2
## Gemella                              2.4258035 0.6579692457       2>1
## Abiotrophia                          5.4771207 0.2417479200       2>1
## Granulicatella                       1.0255338 0.9058998338       2>1
## Lactobacillus                        7.1781090 0.1267700490       2>1
## Streptococcus                        0.4910186 0.9743694121       1>2
## Order.Lactobacillales.              -0.5961281 1.0000000000       2>1
## Class.Bacilli.                       7.5309857 0.1103506912       2>1
## Clostridiales_[F.1][G-1]             1.8054023 0.7714938140       1>2
## Butyrivibrio                        10.1937058 0.0372881320       1>2
## Catonella                            5.6465545 0.2271444138       2>1
## Johnsonella                          4.0184477 0.4035149875       2>1
## Lachnoanaerobaculum                  9.7576624 0.0447137933       1>2
## Lachnospiraceae_[G.2]                8.6412746 0.0707187732       1>2
## Lachnospiraceae_[G.3]                1.6014826 0.8085256320       1>2
## Lachnospiraceae_[G.7]                1.7383161 0.7837462555       2>1
## Lachnospiraceae_[G.8]                2.9717152 0.5625699061       1>2
## Oribacterium                         7.1959823 0.1258868693       1>2
## Shuttleworthia                       2.2823937 0.6839763670       2>1
## Stomatobaculum                       2.8082713 0.5904057867       1>2
## Family.Lachnospiraceae_[XIV].       11.5663409 0.0208849635       1>2
## Peptococcus                          0.8327565 0.9340034313       1>2
## Parvimonas                           7.8129279 0.0986763061       1>2
## Filifactor                           7.0554305 0.1329878105       1>2
## Mogibacterium                        2.3525929 0.6712108872       1>2
## Peptostreptococcaceae_[XI][G.1]      3.4363286 0.4876255279       1>2
## Peptostreptococcaceae_[XI][G.4]      1.1505899 0.8861647232       2>1
## Peptostreptococcaceae_[XI][G.5]      3.1290266 0.5364700230       1>2
## Peptostreptococcaceae_[XI][G.6]      4.2595067 0.3720231674       1>2
## Peptostreptococcaceae_[XI][G.7]      4.2249693 0.3764148864       2>1
## Peptostreptococcaceae_[XI][G.9]      1.7101863 0.7888664534       2>1
## Peptostreptococcus                   4.2403653 0.3744521993       1>2
## Ruminococcaceae_[G.1]                1.6263821 0.8040428213       2>1
## Ruminococcaceae_[G.2]                1.0210831 0.9065824341       1>2
## Bulleidia                            2.3404350 0.6734172892       1>2
## Eggerthia                            5.1015548 0.2770351639       1>2
## Solobacterium                        7.8776228 0.0961652978       1>2
## Mycoplasma                           0.4768142 0.9757185135       1>2
## Mitsuokella                          0.7635789 0.9432630412       2>1
## Selenomonas                          7.0536776 0.1330786553       1>2
## Family.Selenomonadaceae.            12.0941826 0.0166644706       1>2
## Class.Negativicutes.                 5.1364823 0.2735780384       1>2
```

```
## Anaeroglobus                          10.4272078 0.0338145933       2>1
## Dialister                              2.5559989 0.6346371634       2>1
## Megasphaera                            4.0799531 0.3952935056       1>2
## Family.Veillonellaceae.                6.9928757 0.1362651902       2>1
## Veillonella                            0.7337351 0.9471044561       1>2
## Veillonellaceae_[G.1]                  4.6934277 0.3202238771       2>1
## Phylum.Firmicutes.                    12.0259065 0.0171596540       2>1
## Fusobacterium                          5.6309553 0.2284559636       1>2
## Leptotrichia                           7.3180353 0.1200060627       1>2
## Gracilibacteria_(GN02)_[G.1]           9.4911020 0.0499304031       1>2
## Lautropia                              6.2596516 0.1805783430       2>1
## Ottowia                                8.1129297 0.0875279355       2>1
## Eikenella                              6.9535355 0.1383641290       1>2
## Kingella                               6.2712697 0.1797849224       2>1
## Neisseria                              6.8737122 0.1427143690       2>1
## Family.Neisseriaceae.                  8.7440370 0.0678256548       2>1
## Campylobacter                          5.2739924 0.2603214444       1>2
## Cardiobacterium                        3.3146581 0.5066131762       2>1
## Aggregatibacter                        7.1265659 0.1293490619       1>2
## Haemophilus                            1.1788565 0.8815673323       1>2
## Family.Pasteurellaceae.                1.0092396 0.9083917492       1>2
## Saccharibacteria_(TM7)_[G.1]           7.0146731 0.1351148470       2>1
## Saccharibacteria_(TM7)_[G.3]           8.1509493 0.0862026291       2>1
## Saccharibacteria_(TM7)_[G.6]          11.0118176 0.0264315217       2>1
## Family.Saccharibacteria_(TM7)_[F-1].  4.7784429 0.3107951618       2>1
## Saccharibacteria_(TM7)_[G.5]          11.8065345 0.0188494335       2>1
## Treponema                              6.4063656 0.1707865470       1>2
## Fretibacterium                         7.7068418 0.1029268111       1>2
## Kingdom.Bacteria.                      3.7823840 0.4362572822       1>2
## Others                                16.9445815 0.0019814627       2>1
##                                         pval.adj
## Absconditabacteria_(SR1)_[G.1]        0.04444504
## Actinomyces                           0.22509350
## Peptidiphaga                          0.48876234
## Schaalia                              0.31057157
## Family.Actinomycetaceae.              0.92267043
## Rothia                                0.48174410
## Alloscardovia                         0.96828571
## Bifidobacterium                       0.35495625
## Parascardovia                         0.16557001
## Scardovia                             0.55978428
## Corynebacterium                       0.35296126
## Olsenella                             0.88732534
## Atopobium                             0.82999074
## Slackia                               0.91366537
## Cryptobacterium                       0.82999074
## Bacteroidetes_[G.3]                   0.48174410
## Bacteroidetes_[G.5]                   0.35495625
## Family.Bacteroidetes_[F-1].           0.30160378
## Bacteroidaceae_[G.1]                  0.22509350
## Bacteroidales_[G.2]                   0.87189341
## Porphyromonas                         0.49946229
## Tannerella                            0.89289671
## Alloprevotella                        0.89289671
```

```
## Prevotella                            0.54894639
## Bergeyella                            0.35495625
## Capnocytophaga                        0.35495625
## Gemella                               0.88272266
## Abiotrophia                           0.48876234
## Granulicatella                        0.96828571
## Lactobacillus                         0.35495625
## Streptococcus                         0.98588225
## Order.Lactobacillales.                1.00000000
## Class.Bacilli.                        0.35495625
## Clostridiales_[F.1][G-1]              0.92267043
## Butyrivibrio                          0.30141240
## Catonella                             0.48174410
## Johnsonella                           0.64165498
## Lachnoanaerobaculum                   0.30980271
## Lachnospiraceae_[G.2]                 0.35495625
## Lachnospiraceae_[G.3]                 0.92267043
## Lachnospiraceae_[G.7]                 0.92267043
## Lachnospiraceae_[G.8]                 0.82680729
## Oribacterium                          0.35495625
## Shuttleworthia                        0.88460943
## Stomatobaculum                        0.82999074
## Family.Lachnospiraceae_[XIV].         0.22509350
## Peptococcus                           0.97733119
## Parvimonas                            0.35495625
## Filifactor                            0.35495625
## Mogibacterium                         0.88272266
## Peptostreptococcaceae_[XI][G.1]       0.75078851
## Peptostreptococcaceae_[XI][G.4]       0.96828571
## Peptostreptococcaceae_[XI][G.5]       0.80057834
## Peptostreptococcaceae_[XI][G.6]       0.61885159
## Peptostreptococcaceae_[XI][G.7]       0.61885159
## Peptostreptococcaceae_[XI][G.9]       0.92267043
## Peptostreptococcus                    0.61885159
## Ruminococcaceae_[G.1]                 0.92267043
## Ruminococcaceae_[G.2]                 0.96828571
## Bulleidia                             0.88272266
## Eggerthia                             0.51677713
## Solobacterium                         0.35495625
## Mycoplasma                            0.98588225
## Mitsuokella                           0.97733119
## Selenomonas                           0.35495625
## Family.Selenomonadaceae.              0.22509350
## Class.Negativicutes.                  0.51677713
## Anaeroglobus                          0.29818323
## Dialister                             0.87189341
## Megasphaera                           0.63905783
## Family.Veillonellaceae.               0.35495625
## Veillonella                           0.97733119
## Veillonellaceae_[G.1]                 0.55978428
## Phylum.Firmicutes.                    0.22509350
## Fusobacterium                         0.48174410
## Leptotrichia                          0.35495625
## Gracilibacteria_(GN02)_[G.1]          0.31057157
```

```
## Lautropia                            0.41704998
## Ottowia                              0.35495625
## Eikenella                            0.35495625
## Kingella                             0.41704998
## Neisseria                            0.35495625
## Family.Neisseriaceae.                0.35495625
## Campylobacter                        0.50502360
## Cardiobacterium                      0.76783560
## Aggregatibacter                      0.35495625
## Haemophilus                          0.96828571
## Family.Pasteurellaceae.              0.96828571
## Saccharibacteria_(TM7)_[G.1]         0.35495625
## Saccharibacteria_(TM7)_[G.3]         0.35495625
## Saccharibacteria_(TM7)_[G.6]         0.25638576
## Family.Saccharibacteria_(TM7)_[F-1]. 0.55828020
## Saccharibacteria_(TM7)_[G.5]         0.22509350
## Treponema                            0.41415738
## Fretibacterium                       0.35495625
## Kingdom.Bacteria.                    0.68253155
## Others                               0.09610094
##                                                                    Feature
## Absconditabacteria_(SR1)_[G.1]            Absconditabacteria_(SR1)_[G.1]
## Actinomyces                                                    Actinomyces
## Peptidiphaga                                                  Peptidiphaga
## Schaalia                                                          Schaalia
## Family.Actinomycetaceae.                          Family.Actinomycetaceae.
## Rothia                                                              Rothia
## Alloscardovia                                                Alloscardovia
## Bifidobacterium                                            Bifidobacterium
## Parascardovia                                                Parascardovia
## Scardovia                                                        Scardovia
## Corynebacterium                                            Corynebacterium
## Olsenella                                                        Olsenella
## Atopobium                                                        Atopobium
## Slackia                                                            Slackia
## Cryptobacterium                                            Cryptobacterium
## Bacteroidetes_[G.3]                                    Bacteroidetes_[G.3]
## Bacteroidetes_[G.5]                                    Bacteroidetes_[G.5]
## Family.Bacteroidetes_[F-1].                    Family.Bacteroidetes_[F-1].
## Bacteroidaceae_[G.1]                                  Bacteroidaceae_[G.1]
## Bacteroidales_[G.2]                                    Bacteroidales_[G.2]
## Porphyromonas                                                Porphyromonas
## Tannerella                                                      Tannerella
## Alloprevotella                                              Alloprevotella
## Prevotella                                                      Prevotella
## Bergeyella                                                      Bergeyella
## Capnocytophaga                                              Capnocytophaga
## Gemella                                                            Gemella
## Abiotrophia                                                    Abiotrophia
## Granulicatella                                              Granulicatella
## Lactobacillus                                                Lactobacillus
## Streptococcus                                                Streptococcus
## Order.Lactobacillales.                              Order.Lactobacillales.
## Class.Bacilli.                                              Class.Bacilli.
```

```
## Clostridiales_[F.1][G-1]                       Clostridiales_[F.1][G-1]
## Butyrivibrio                                               Butyrivibrio
## Catonella                                                     Catonella
## Johnsonella                                                 Johnsonella
## Lachnoanaerobaculum                                 Lachnoanaerobaculum
## Lachnospiraceae_[G.2]                             Lachnospiraceae_[G.2]
## Lachnospiraceae_[G.3]                             Lachnospiraceae_[G.3]
## Lachnospiraceae_[G.7]                             Lachnospiraceae_[G.7]
## Lachnospiraceae_[G.8]                             Lachnospiraceae_[G.8]
## Oribacterium                                               Oribacterium
## Shuttleworthia                                           Shuttleworthia
## Stomatobaculum                                           Stomatobaculum
## Family.Lachnospiraceae_[XIV].           Family.Lachnospiraceae_[XIV].
## Peptococcus                                                 Peptococcus
## Parvimonas                                                   Parvimonas
## Filifactor                                                   Filifactor
## Mogibacterium                                             Mogibacterium
## Peptostreptococcaceae_[XI][G.1]       Peptostreptococcaceae_[XI][G.1]
## Peptostreptococcaceae_[XI][G.4]       Peptostreptococcaceae_[XI][G.4]
## Peptostreptococcaceae_[XI][G.5]       Peptostreptococcaceae_[XI][G.5]
## Peptostreptococcaceae_[XI][G.6]       Peptostreptococcaceae_[XI][G.6]
## Peptostreptococcaceae_[XI][G.7]       Peptostreptococcaceae_[XI][G.7]
## Peptostreptococcaceae_[XI][G.9]       Peptostreptococcaceae_[XI][G.9]
## Peptostreptococcus                                   Peptostreptococcus
## Ruminococcaceae_[G.1]                             Ruminococcaceae_[G.1]
## Ruminococcaceae_[G.2]                             Ruminococcaceae_[G.2]
## Bulleidia                                                     Bulleidia
## Eggerthia                                                     Eggerthia
## Solobacterium                                             Solobacterium
## Mycoplasma                                                   Mycoplasma
## Mitsuokella                                                 Mitsuokella
## Selenomonas                                                 Selenomonas
## Family.Selenomonadaceae.                       Family.Selenomonadaceae.
## Class.Negativicutes.                               Class.Negativicutes.
## Anaeroglobus                                               Anaeroglobus
## Dialister                                                     Dialister
## Megasphaera                                                 Megasphaera
## Family.Veillonellaceae.                         Family.Veillonellaceae.
## Veillonella                                                 Veillonella
## Veillonellaceae_[G.1]                             Veillonellaceae_[G.1]
## Phylum.Firmicutes.                                   Phylum.Firmicutes.
## Fusobacterium                                             Fusobacterium
## Leptotrichia                                               Leptotrichia
## Gracilibacteria_(GN02)_[G.1]               Gracilibacteria_(GN02)_[G.1]
## Lautropia                                                     Lautropia
## Ottowia                                                         Ottowia
## Eikenella                                                     Eikenella
## Kingella                                                       Kingella
## Neisseria                                                     Neisseria
## Family.Neisseriaceae.                             Family.Neisseriaceae.
## Campylobacter                                             Campylobacter
## Cardiobacterium                                         Cardiobacterium
## Aggregatibacter                                         Aggregatibacter
## Haemophilus                                                 Haemophilus
```

```
## Family.Pasteurellaceae.                            Family.Pasteurellaceae.
## Saccharibacteria_(TM7)_[G.1]                  Saccharibacteria_(TM7)_[G.1]
## Saccharibacteria_(TM7)_[G.3]                  Saccharibacteria_(TM7)_[G.3]
## Saccharibacteria_(TM7)_[G.6]                  Saccharibacteria_(TM7)_[G.6]
## Family.Saccharibacteria_(TM7)_[F-1]. Family.Saccharibacteria_(TM7)_[F-1].
## Saccharibacteria_(TM7)_[G.5]                  Saccharibacteria_(TM7)_[G.5]
## Treponema                                                        Treponema
## Fretibacterium                                              Fretibacterium
## Kingdom.Bacteria.                                        Kingdom.Bacteria.
## Others                                                              Others
##                                                         Method
## Absconditabacteria_(SR1)_[G.1]       DESeq2 man. geoMeans (ds2)
## Actinomyces                          DESeq2 man. geoMeans (ds2)
## Peptidiphaga                         DESeq2 man. geoMeans (ds2)
## Schaalia                             DESeq2 man. geoMeans (ds2)
## Family.Actinomycetaceae.             DESeq2 man. geoMeans (ds2)
## Rothia                               DESeq2 man. geoMeans (ds2)
## Alloscardovia                        DESeq2 man. geoMeans (ds2)
## Bifidobacterium                      DESeq2 man. geoMeans (ds2)
## Parascardovia                        DESeq2 man. geoMeans (ds2)
## Scardovia                            DESeq2 man. geoMeans (ds2)
## Corynebacterium                      DESeq2 man. geoMeans (ds2)
## Olsenella                            DESeq2 man. geoMeans (ds2)
## Atopobium                            DESeq2 man. geoMeans (ds2)
## Slackia                              DESeq2 man. geoMeans (ds2)
## Cryptobacterium                      DESeq2 man. geoMeans (ds2)
## Bacteroidetes_[G.3]                  DESeq2 man. geoMeans (ds2)
## Bacteroidetes_[G.5]                  DESeq2 man. geoMeans (ds2)
## Family.Bacteroidetes_[F-1].          DESeq2 man. geoMeans (ds2)
## Bacteroidaceae_[G.1]                 DESeq2 man. geoMeans (ds2)
## Bacteroidales_[G.2]                  DESeq2 man. geoMeans (ds2)
## Porphyromonas                        DESeq2 man. geoMeans (ds2)
## Tannerella                           DESeq2 man. geoMeans (ds2)
## Alloprevotella                       DESeq2 man. geoMeans (ds2)
## Prevotella                           DESeq2 man. geoMeans (ds2)
## Bergeyella                           DESeq2 man. geoMeans (ds2)
## Capnocytophaga                       DESeq2 man. geoMeans (ds2)
## Gemella                              DESeq2 man. geoMeans (ds2)
## Abiotrophia                          DESeq2 man. geoMeans (ds2)
## Granulicatella                       DESeq2 man. geoMeans (ds2)
## Lactobacillus                        DESeq2 man. geoMeans (ds2)
## Streptococcus                        DESeq2 man. geoMeans (ds2)
## Order.Lactobacillales.               DESeq2 man. geoMeans (ds2)
## Class.Bacilli.                       DESeq2 man. geoMeans (ds2)
## Clostridiales_[F.1][G-1]             DESeq2 man. geoMeans (ds2)
## Butyrivibrio                         DESeq2 man. geoMeans (ds2)
## Catonella                            DESeq2 man. geoMeans (ds2)
## Johnsonella                          DESeq2 man. geoMeans (ds2)
## Lachnoanaerobaculum                  DESeq2 man. geoMeans (ds2)
## Lachnospiraceae_[G.2]                DESeq2 man. geoMeans (ds2)
## Lachnospiraceae_[G.3]                DESeq2 man. geoMeans (ds2)
## Lachnospiraceae_[G.7]                DESeq2 man. geoMeans (ds2)
## Lachnospiraceae_[G.8]                DESeq2 man. geoMeans (ds2)
## Oribacterium                         DESeq2 man. geoMeans (ds2)
```

```
## Shuttleworthia                        DESeq2 man. geoMeans (ds2)
## Stomatobaculum                        DESeq2 man. geoMeans (ds2)
## Family.Lachnospiraceae_[XIV].         DESeq2 man. geoMeans (ds2)
## Peptococcus                           DESeq2 man. geoMeans (ds2)
## Parvimonas                            DESeq2 man. geoMeans (ds2)
## Filifactor                            DESeq2 man. geoMeans (ds2)
## Mogibacterium                         DESeq2 man. geoMeans (ds2)
## Peptostreptococcaceae_[XI][G.1]       DESeq2 man. geoMeans (ds2)
## Peptostreptococcaceae_[XI][G.4]       DESeq2 man. geoMeans (ds2)
## Peptostreptococcaceae_[XI][G.5]       DESeq2 man. geoMeans (ds2)
## Peptostreptococcaceae_[XI][G.6]       DESeq2 man. geoMeans (ds2)
## Peptostreptococcaceae_[XI][G.7]       DESeq2 man. geoMeans (ds2)
## Peptostreptococcaceae_[XI][G.9]       DESeq2 man. geoMeans (ds2)
## Peptostreptococcus                    DESeq2 man. geoMeans (ds2)
## Ruminococcaceae_[G.1]                 DESeq2 man. geoMeans (ds2)
## Ruminococcaceae_[G.2]                 DESeq2 man. geoMeans (ds2)
## Bulleidia                             DESeq2 man. geoMeans (ds2)
## Eggerthia                             DESeq2 man. geoMeans (ds2)
## Solobacterium                         DESeq2 man. geoMeans (ds2)
## Mycoplasma                            DESeq2 man. geoMeans (ds2)
## Mitsuokella                           DESeq2 man. geoMeans (ds2)
## Selenomonas                           DESeq2 man. geoMeans (ds2)
## Family.Selenomonadaceae.              DESeq2 man. geoMeans (ds2)
## Class.Negativicutes.                  DESeq2 man. geoMeans (ds2)
## Anaeroglobus                          DESeq2 man. geoMeans (ds2)
## Dialister                             DESeq2 man. geoMeans (ds2)
## Megasphaera                           DESeq2 man. geoMeans (ds2)
## Family.Veillonellaceae.               DESeq2 man. geoMeans (ds2)
## Veillonella                           DESeq2 man. geoMeans (ds2)
## Veillonellaceae_[G.1]                 DESeq2 man. geoMeans (ds2)
## Phylum.Firmicutes.                    DESeq2 man. geoMeans (ds2)
## Fusobacterium                         DESeq2 man. geoMeans (ds2)
## Leptotrichia                          DESeq2 man. geoMeans (ds2)
## Gracilibacteria_(GN02)_[G.1]          DESeq2 man. geoMeans (ds2)
## Lautropia                             DESeq2 man. geoMeans (ds2)
## Ottowia                               DESeq2 man. geoMeans (ds2)
## Eikenella                             DESeq2 man. geoMeans (ds2)
## Kingella                              DESeq2 man. geoMeans (ds2)
## Neisseria                             DESeq2 man. geoMeans (ds2)
## Family.Neisseriaceae.                 DESeq2 man. geoMeans (ds2)
## Campylobacter                         DESeq2 man. geoMeans (ds2)
## Cardiobacterium                       DESeq2 man. geoMeans (ds2)
## Aggregatibacter                       DESeq2 man. geoMeans (ds2)
## Haemophilus                           DESeq2 man. geoMeans (ds2)
## Family.Pasteurellaceae.               DESeq2 man. geoMeans (ds2)
## Saccharibacteria_(TM7)_[G.1]          DESeq2 man. geoMeans (ds2)
## Saccharibacteria_(TM7)_[G.3]          DESeq2 man. geoMeans (ds2)
## Saccharibacteria_(TM7)_[G.6]          DESeq2 man. geoMeans (ds2)
## Family.Saccharibacteria_(TM7)_[F-1]. DESeq2 man. geoMeans (ds2)
## Saccharibacteria_(TM7)_[G.5]          DESeq2 man. geoMeans (ds2)
## Treponema                             DESeq2 man. geoMeans (ds2)
## Fretibacterium                        DESeq2 man. geoMeans (ds2)
## Kingdom.Bacteria.                     DESeq2 man. geoMeans (ds2)
## Others                                DESeq2 man. geoMeans (ds2)
```

```r
final <- DA.kru(Microbio2, predictor = as.factor(Phe2$gly_stat))
final
```

```
##                                           pval  pval.adj
## Absconditabacteria_(SR1)_[G.1]       0.725514181 0.8813442
## Actinomyces                          0.064721243 0.4829200
## Peptidiphaga                         0.332357788 0.7305597
## Schaalia                             0.111672654 0.5701183
## Family.Actinomycetaceae.             0.504660010 0.7976156
## Rothia                               0.041311821 0.4007247
## Alloscardovia                        0.510668032 0.7976156
## Bifidobacterium                      0.487742807 0.7976156
## Parascardovia                        0.429353211 0.7844969
## Scardovia                            0.448712329 0.7844969
## Corynebacterium                      0.711580325 0.8813442
## Olsenella                            0.549362355 0.8326273
## Atopobium                            0.573843308 0.8437662
## Slackia                              0.441575586 0.7844969
## Cryptobacterium                      0.297999172 0.6882362
## Bacteroidetes_[G.3]                  0.197071749 0.6374516
## Bacteroidetes_[G.5]                  0.982800034 0.9858884
## Family.Bacteroidetes_[F-1].          0.007629216 0.3305830
## Bacteroidaceae_[G.1]                 0.903593633 0.9681601
## Bacteroidales_[G.2]                  0.699696573 0.8813442
## Porphyromonas                        0.363288832 0.7658947
## Tannerella                           0.809806874 0.9133868
## Alloprevotella                       0.574108937 0.8437662
## Prevotella                           0.268749235 0.6638626
## Bergeyella                           0.089452932 0.5146724
## Capnocytophaga                       0.400244908 0.7844969
## Gemella                              0.371103599 0.7658947
## Abiotrophia                          0.012917921 0.3305830
## Granulicatella                       0.801023882 0.9133868
## Lactobacillus                        0.757309315 0.8951908
## Streptococcus                        0.175013477 0.6374516
## Order.Lactobacillales.               0.057293452 0.4631221
## Class.Bacilli.                       0.503958691 0.7976156
## Clostridiales_[F.1][G-1]             0.856503283 0.9549519
## Butyrivibrio                         0.338919457 0.7305597
## Catonella                            0.184101260 0.6374516
## Johnsonella                          0.720300778 0.8813442
## Lachnoanaerobaculum                  0.178802910 0.6374516
## Lachnospiraceae_[G.2]                0.031514925 0.3821185
## Lachnospiraceae_[G.3]                0.961114893 0.9813489
## Lachnospiraceae_[G.7]                0.016468625 0.3305830
## Lachnospiraceae_[G.8]                0.518039008 0.7976156
## Oribacterium                         0.095506212 0.5146724
## Shuttleworthia                       0.906593232 0.9681601
## Stomatobaculum                       0.927604678 0.9681601
## Family.Lachnospiraceae_[XIV].        0.085336274 0.5146724
## Peptococcus                          0.928235934 0.9681601
## Parvimonas                           0.460993045 0.7844969
## Filifactor                           0.273757755 0.6638626
```

```
## Mogibacterium                          0.795314964 0.9133868
## Peptostreptococcaceae_[XI][G.1]       0.249635988 0.6638626
## Peptostreptococcaceae_[XI][G.4]       0.145740212 0.6374516
## Peptostreptococcaceae_[XI][G.5]       0.628717498 0.8712228
## Peptostreptococcaceae_[XI][G.6]       0.210662628 0.6490632
## Peptostreptococcaceae_[XI][G.7]       0.072410129 0.5016988
## Peptostreptococcaceae_[XI][G.9]       0.431015319 0.7844969
## Peptostreptococcus                    0.245349102 0.6638626
## Ruminococcaceae_[G.1]                 0.765987978 0.8951908
## Ruminococcaceae_[G.2]                 0.626679214 0.8712228
## Bulleidia                             0.454636368 0.7844969
## Eggerthia                             0.288712264 0.6830510
## Solobacterium                         0.319025338 0.7196618
## Mycoplasma                            0.947771148 0.9780192
## Mitsuokella                           0.985888357 0.9858884
## Selenomonas                           0.624493611 0.8712228
## Family.Selenomonadaceae.              0.144132644 0.6374516
## Class.Negativicutes.                  0.194525806 0.6374516
## Anaeroglobus                          0.726881815 0.8813442
## Dialister                             0.713782139 0.8813442
## Megasphaera                           0.746404835 0.8938428
## Family.Veillonellaceae.               0.174973130 0.6374516
## Veillonella                           0.589109343 0.8528896
## Veillonellaceae_[G.1]                 0.247967362 0.6638626
## Phylum.Firmicutes.                    0.260454311 0.6638626
## Fusobacterium                         0.454124146 0.7844969
## Leptotrichia                          0.023856505 0.3305830
## Gracilibacteria_(GN02)_[G.1]          0.051200934 0.4514991
## Lautropia                             0.022418493 0.3305830
## Ottowia                               0.022864930 0.3305830
## Eikenella                             0.381727373 0.7714074
## Kingella                              0.705531035 0.8813442
## Neisseria                             0.037581329 0.4007247
## Family.Neisseriaceae.                 0.192532108 0.6374516
## Campylobacter                         0.266457800 0.6638626
## Cardiobacterium                       0.925038621 0.9681601
## Aggregatibacter                       0.229682288 0.6638626
## Haemophilus                           0.912865978 0.9681601
## Family.Pasteurellaceae.               0.445780528 0.7844969
## Saccharibacteria_(TM7)_[G.1]          0.723052752 0.8813442
## Saccharibacteria_(TM7)_[G.3]          0.662135219 0.8813442
## Saccharibacteria_(TM7)_[G.6]          0.002088644 0.2025985
## Family.Saccharibacteria_(TM7)_[F-1].  0.090841107 0.5146724
## Saccharibacteria_(TM7)_[G.5]          0.214123947 0.6490632
## Treponema                             0.479629535 0.7976156
## Fretibacterium                        0.197149965 0.6374516
## Kingdom.Bacteria.                     0.683003280 0.8813442
## Others                                0.174258768 0.6374516
##                                                              Feature
## Absconditabacteria_(SR1)_[G.1]            Absconditabacteria_(SR1)_[G.1]
## Actinomyces                                              Actinomyces
## Peptidiphaga                                            Peptidiphaga
## Schaalia                                                    Schaalia
## Family.Actinomycetaceae.                      Family.Actinomycetaceae.
```

```
## Rothia                                                              Rothia
## Alloscardovia                                                Alloscardovia
## Bifidobacterium                                            Bifidobacterium
## Parascardovia                                                Parascardovia
## Scardovia                                                        Scardovia
## Corynebacterium                                            Corynebacterium
## Olsenella                                                        Olsenella
## Atopobium                                                        Atopobium
## Slackia                                                            Slackia
## Cryptobacterium                                            Cryptobacterium
## Bacteroidetes_[G.3]                                    Bacteroidetes_[G.3]
## Bacteroidetes_[G.5]                                    Bacteroidetes_[G.5]
## Family.Bacteroidetes_[F-1].                    Family.Bacteroidetes_[F-1].
## Bacteroidaceae_[G.1]                                  Bacteroidaceae_[G.1]
## Bacteroidales_[G.2]                                    Bacteroidales_[G.2]
## Porphyromonas                                                Porphyromonas
## Tannerella                                                      Tannerella
## Alloprevotella                                              Alloprevotella
## Prevotella                                                      Prevotella
## Bergeyella                                                      Bergeyella
## Capnocytophaga                                              Capnocytophaga
## Gemella                                                            Gemella
## Abiotrophia                                                    Abiotrophia
## Granulicatella                                              Granulicatella
## Lactobacillus                                                Lactobacillus
## Streptococcus                                                Streptococcus
## Order.Lactobacillales.                              Order.Lactobacillales.
## Class.Bacilli.                                              Class.Bacilli.
## Clostridiales_[F.1][G-1]                          Clostridiales_[F.1][G-1]
## Butyrivibrio                                                  Butyrivibrio
## Catonella                                                        Catonella
## Johnsonella                                                    Johnsonella
## Lachnoanaerobaculum                                    Lachnoanaerobaculum
## Lachnospiraceae_[G.2]                                Lachnospiraceae_[G.2]
## Lachnospiraceae_[G.3]                                Lachnospiraceae_[G.3]
## Lachnospiraceae_[G.7]                                Lachnospiraceae_[G.7]
## Lachnospiraceae_[G.8]                                Lachnospiraceae_[G.8]
## Oribacterium                                                  Oribacterium
## Shuttleworthia                                              Shuttleworthia
## Stomatobaculum                                              Stomatobaculum
## Family.Lachnospiraceae_[XIV].                Family.Lachnospiraceae_[XIV].
## Peptococcus                                                    Peptococcus
## Parvimonas                                                      Parvimonas
## Filifactor                                                      Filifactor
## Mogibacterium                                                Mogibacterium
## Peptostreptococcaceae_[XI][G.1]            Peptostreptococcaceae_[XI][G.1]
## Peptostreptococcaceae_[XI][G.4]            Peptostreptococcaceae_[XI][G.4]
## Peptostreptococcaceae_[XI][G.5]            Peptostreptococcaceae_[XI][G.5]
## Peptostreptococcaceae_[XI][G.6]            Peptostreptococcaceae_[XI][G.6]
## Peptostreptococcaceae_[XI][G.7]            Peptostreptococcaceae_[XI][G.7]
## Peptostreptococcaceae_[XI][G.9]            Peptostreptococcaceae_[XI][G.9]
## Peptostreptococcus                                      Peptostreptococcus
## Ruminococcaceae_[G.1]                                Ruminococcaceae_[G.1]
## Ruminococcaceae_[G.2]                                Ruminococcaceae_[G.2]
```

```
## Bulleidia                                                         Bulleidia
## Eggerthia                                                         Eggerthia
## Solobacterium                                                 Solobacterium
## Mycoplasma                                                       Mycoplasma
## Mitsuokella                                                     Mitsuokella
## Selenomonas                                                     Selenomonas
## Family.Selenomonadaceae.                           Family.Selenomonadaceae.
## Class.Negativicutes.                                   Class.Negativicutes.
## Anaeroglobus                                                   Anaeroglobus
## Dialister                                                         Dialister
## Megasphaera                                                     Megasphaera
## Family.Veillonellaceae.                             Family.Veillonellaceae.
## Veillonella                                                     Veillonella
## Veillonellaceae_[G.1]                                 Veillonellaceae_[G.1]
## Phylum.Firmicutes.                                       Phylum.Firmicutes.
## Fusobacterium                                                 Fusobacterium
## Leptotrichia                                                   Leptotrichia
## Gracilibacteria_(GN02)_[G.1]                   Gracilibacteria_(GN02)_[G.1]
## Lautropia                                                         Lautropia
## Ottowia                                                             Ottowia
## Eikenella                                                         Eikenella
## Kingella                                                           Kingella
## Neisseria                                                         Neisseria
## Family.Neisseriaceae.                                 Family.Neisseriaceae.
## Campylobacter                                                 Campylobacter
## Cardiobacterium                                             Cardiobacterium
## Aggregatibacter                                             Aggregatibacter
## Haemophilus                                                     Haemophilus
## Family.Pasteurellaceae.                             Family.Pasteurellaceae.
## Saccharibacteria_(TM7)_[G.1]                   Saccharibacteria_(TM7)_[G.1]
## Saccharibacteria_(TM7)_[G.3]                   Saccharibacteria_(TM7)_[G.3]
## Saccharibacteria_(TM7)_[G.6]                   Saccharibacteria_(TM7)_[G.6]
## Family.Saccharibacteria_(TM7)_[F-1]. Family.Saccharibacteria_(TM7)_[F-1].
## Saccharibacteria_(TM7)_[G.5]                   Saccharibacteria_(TM7)_[G.5]
## Treponema                                                         Treponema
## Fretibacterium                                               Fretibacterium
## Kingdom.Bacteria.                                         Kingdom.Bacteria.
## Others                                                               Others
##                                                    Method
## Absconditabacteria_(SR1)_[G.1]       Kruskal-Wallis (kru)
## Actinomyces                          Kruskal-Wallis (kru)
## Peptidiphaga                         Kruskal-Wallis (kru)
## Schaalia                             Kruskal-Wallis (kru)
## Family.Actinomycetaceae.             Kruskal-Wallis (kru)
## Rothia                               Kruskal-Wallis (kru)
## Alloscardovia                        Kruskal-Wallis (kru)
## Bifidobacterium                      Kruskal-Wallis (kru)
## Parascardovia                        Kruskal-Wallis (kru)
## Scardovia                            Kruskal-Wallis (kru)
## Corynebacterium                      Kruskal-Wallis (kru)
## Olsenella                            Kruskal-Wallis (kru)
## Atopobium                            Kruskal-Wallis (kru)
## Slackia                              Kruskal-Wallis (kru)
## Cryptobacterium                      Kruskal-Wallis (kru)
```

```
## Bacteroidetes_[G.3]                  Kruskal-Wallis (kru)
## Bacteroidetes_[G.5]                  Kruskal-Wallis (kru)
## Family.Bacteroidetes_[F-1].          Kruskal-Wallis (kru)
## Bacteroidaceae_[G.1]                 Kruskal-Wallis (kru)
## Bacteroidales_[G.2]                  Kruskal-Wallis (kru)
## Porphyromonas                        Kruskal-Wallis (kru)
## Tannerella                           Kruskal-Wallis (kru)
## Alloprevotella                       Kruskal-Wallis (kru)
## Prevotella                           Kruskal-Wallis (kru)
## Bergeyella                           Kruskal-Wallis (kru)
## Capnocytophaga                       Kruskal-Wallis (kru)
## Gemella                              Kruskal-Wallis (kru)
## Abiotrophia                          Kruskal-Wallis (kru)
## Granulicatella                       Kruskal-Wallis (kru)
## Lactobacillus                        Kruskal-Wallis (kru)
## Streptococcus                        Kruskal-Wallis (kru)
## Order.Lactobacillales.               Kruskal-Wallis (kru)
## Class.Bacilli.                       Kruskal-Wallis (kru)
## Clostridiales_[F.1][G-1]             Kruskal-Wallis (kru)
## Butyrivibrio                         Kruskal-Wallis (kru)
## Catonella                            Kruskal-Wallis (kru)
## Johnsonella                          Kruskal-Wallis (kru)
## Lachnoanaerobaculum                  Kruskal-Wallis (kru)
## Lachnospiraceae_[G.2]                Kruskal-Wallis (kru)
## Lachnospiraceae_[G.3]                Kruskal-Wallis (kru)
## Lachnospiraceae_[G.7]                Kruskal-Wallis (kru)
## Lachnospiraceae_[G.8]                Kruskal-Wallis (kru)
## Oribacterium                         Kruskal-Wallis (kru)
## Shuttleworthia                       Kruskal-Wallis (kru)
## Stomatobaculum                       Kruskal-Wallis (kru)
## Family.Lachnospiraceae_[XIV].        Kruskal-Wallis (kru)
## Peptococcus                          Kruskal-Wallis (kru)
## Parvimonas                           Kruskal-Wallis (kru)
## Filifactor                           Kruskal-Wallis (kru)
## Mogibacterium                        Kruskal-Wallis (kru)
## Peptostreptococcaceae_[XI][G.1]      Kruskal-Wallis (kru)
## Peptostreptococcaceae_[XI][G.4]      Kruskal-Wallis (kru)
## Peptostreptococcaceae_[XI][G.5]      Kruskal-Wallis (kru)
## Peptostreptococcaceae_[XI][G.6]      Kruskal-Wallis (kru)
## Peptostreptococcaceae_[XI][G.7]      Kruskal-Wallis (kru)
## Peptostreptococcaceae_[XI][G.9]      Kruskal-Wallis (kru)
## Peptostreptococcus                   Kruskal-Wallis (kru)
## Ruminococcaceae_[G.1]                Kruskal-Wallis (kru)
## Ruminococcaceae_[G.2]                Kruskal-Wallis (kru)
## Bulleidia                            Kruskal-Wallis (kru)
## Eggerthia                            Kruskal-Wallis (kru)
## Solobacterium                        Kruskal-Wallis (kru)
## Mycoplasma                           Kruskal-Wallis (kru)
## Mitsuokella                          Kruskal-Wallis (kru)
## Selenomonas                          Kruskal-Wallis (kru)
## Family.Selenomonadaceae.             Kruskal-Wallis (kru)
## Class.Negativicutes.                 Kruskal-Wallis (kru)
## Anaeroglobus                         Kruskal-Wallis (kru)
## Dialister                            Kruskal-Wallis (kru)
```

```
## Megasphaera                          Kruskal-Wallis (kru)
## Family.Veillonellaceae.              Kruskal-Wallis (kru)
## Veillonella                          Kruskal-Wallis (kru)
## Veillonellaceae_[G.1]                Kruskal-Wallis (kru)
## Phylum.Firmicutes.                   Kruskal-Wallis (kru)
## Fusobacterium                        Kruskal-Wallis (kru)
## Leptotrichia                         Kruskal-Wallis (kru)
## Gracilibacteria_(GN02)_[G.1]         Kruskal-Wallis (kru)
## Lautropia                            Kruskal-Wallis (kru)
## Ottowia                              Kruskal-Wallis (kru)
## Eikenella                            Kruskal-Wallis (kru)
## Kingella                             Kruskal-Wallis (kru)
## Neisseria                            Kruskal-Wallis (kru)
## Family.Neisseriaceae.                Kruskal-Wallis (kru)
## Campylobacter                        Kruskal-Wallis (kru)
## Cardiobacterium                      Kruskal-Wallis (kru)
## Aggregatibacter                      Kruskal-Wallis (kru)
## Haemophilus                          Kruskal-Wallis (kru)
## Family.Pasteurellaceae.              Kruskal-Wallis (kru)
## Saccharibacteria_(TM7)_[G.1]         Kruskal-Wallis (kru)
## Saccharibacteria_(TM7)_[G.3]         Kruskal-Wallis (kru)
## Saccharibacteria_(TM7)_[G.6]         Kruskal-Wallis (kru)
## Family.Saccharibacteria_(TM7)_[F-1]. Kruskal-Wallis (kru)
## Saccharibacteria_(TM7)_[G.5]         Kruskal-Wallis (kru)
## Treponema                            Kruskal-Wallis (kru)
## Fretibacterium                       Kruskal-Wallis (kru)
## Kingdom.Bacteria.                    Kruskal-Wallis (kru)
## Others                               Kruskal-Wallis (kru)
```

```
#################sex_cat
table(Phe$sex_cat, useNA="always")
```

```
##
## Female   Male   <NA>
##    339    407      0
```

```
final <- DA.ds2(Microbio, predictor = Phe$sex_cat, out.all=TRUE)
final
```

```
##                                         baseMean log2FoldChange      lfcSE
## Absconditabacteria_(SR1)_[G.1]        63.1989360    0.175352526 0.24622108
## Actinomyces                          323.9127377   -0.113305914 0.08337014
## Peptidiphaga                          22.0460481   -0.238112734 0.11870249
## Schaalia                             704.3072222    0.125060516 0.06849926
## Family.Actinomycetaceae.               5.4421107   -0.314921242 0.17083627
## Rothia                              1939.2520922    0.010955960 0.08038236
## Alloscardovia                         12.4599091    0.403098655 0.21109951
## Bifidobacterium                       11.4235529   -0.038718097 0.23316015
## Parascardovia                          2.8111480    0.646181333 0.31677394
## Scardovia                             12.5460084    0.266425112 0.20025161
## Corynebacterium                       93.7260406   -0.034495570 0.08791709
## Olsenella                              1.5669010    0.195950376 0.19500265
## Atopobium                            120.4914295    0.152313687 0.08045811
```

```
## Slackia                                  1.3927613    0.345831164 0.18641600
## Cryptobacterium                          4.3017007    0.679043571 0.19884473
## Bacteroidetes_[G.3]                      6.5912121    0.463606378 0.21091781
## Bacteroidetes_[G.5]                      4.6746313    0.099457423 0.23077968
## Family.Bacteroidetes_[F-1].              0.8680201    0.062800355 0.33254254
## Bacteroidaceae_[G.1]                     0.7921891   -0.524771069 0.31056120
## Bacteroidales_[G.2]                     12.1604713    0.070579598 0.12141349
## Porphyromonas                          680.1285750   -0.083727127 0.11059974
## Tannerella                              22.0547383   -0.108236581 0.08160007
## Alloprevotella                         361.8645217    0.225902095 0.08738709
## Prevotella                            3811.4256813    0.040915587 0.05570439
## Bergeyella                              72.5563323   -0.187501518 0.08578159
## Capnocytophaga                         225.1152973   -0.178186902 0.09173720
## Gemella                                309.0561286   -0.249920012 0.07631675
## Abiotrophia                             29.9768046   -0.316954197 0.16565530
## Granulicatella                         306.2864969   -0.036413446 0.06699244
## Lactobacillus                           29.0689605   -0.357600291 0.26301466
## Streptococcus                         3803.9265541    0.099513106 0.06279391
## Order.Lactobacillales.                   0.2860988   -0.255553356 0.22637573
## Class.Bacilli.                           0.5750915   -0.292192619 0.17984924
## Clostridiales_[F.1][G-1]                 1.0542291    0.473265378 0.27514786
## Butyrivibrio                             6.9441711   -0.076706394 0.17537998
## Catonella                               36.8211521   -0.033061891 0.09885303
## Johnsonella                              0.9388209   -0.051638332 0.23905881
## Lachnoanaerobaculum                     87.7811267   -0.068740301 0.06908805
## Lachnospiraceae_[G.2]                   27.9872677   -0.200966445 0.18126684
## Lachnospiraceae_[G.3]                    2.8891780   -0.223749492 0.18779065
## Lachnospiraceae_[G.7]                    1.4688223    0.958732493 0.26738398
## Lachnospiraceae_[G.8]                    1.4317578   -0.287916801 0.28643926
## Oribacterium                           147.0483774    0.072697147 0.07656036
## Shuttleworthia                           2.8039987    0.315572842 0.21281966
## Stomatobaculum                         112.6769059   -0.081013499 0.08612902
## Family.Lachnospiraceae_[XIV].            1.5085797   -0.183801612 0.23953878
## Peptococcus                              3.7020760   -0.187552454 0.21380374
## Parvimonas                              40.8491501    0.065092516 0.10618280
## Filifactor                               6.8375183    0.237794908 0.20739775
## Mogibacterium                           41.0770621   -0.257622617 0.07285947
## Peptostreptococcaceae_[XI][G.1]         67.4763877   -0.224598394 0.13110296
## Peptostreptococcaceae_[XI][G.4]          0.4176611   -0.102268862 0.28258471
## Peptostreptococcaceae_[XI][G.5]          1.6627493    0.399590157 0.29801242
## Peptostreptococcaceae_[XI][G.6]          1.0137786    0.181843744 0.29401609
## Peptostreptococcaceae_[XI][G.7]          4.7083982    0.250992858 0.20440282
## Peptostreptococcaceae_[XI][G.9]          8.7265107    0.059662278 0.12139876
## Peptostreptococcus                      42.8338056   -0.326955365 0.15291247
## Ruminococcaceae_[G.1]                   25.1020535    0.243603608 0.15530577
## Ruminococcaceae_[G.2]                   41.4593204   -0.000106972 0.12943652
## Bulleidia                                3.8570393    0.581034798 0.22745041
## Eggerthia                                1.5796110    1.151671764 0.30271060
## Solobacterium                           74.0587622   -0.031490850 0.08630569
## Mycoplasma                               4.4957386    0.086389368 0.18238792
## Mitsuokella                             26.8689347    0.521134856 0.16296379
## Selenomonas                            221.9840267   -0.027364405 0.07358903
## Family.Selenomonadaceae.                 5.0346400    0.654619616 0.19137532
## Class.Negativicutes.                     0.7371549   -0.033355543 0.17646942
```

```
## Anaeroglobus                             8.1958528   -0.218456616 0.17221489
## Dialister                               36.4394005    0.169301238 0.09031457
## Megasphaera                            407.9853210    0.148473444 0.09512046
## Family.Veillonellaceae.                  0.5266292    0.299014026 0.20701934
## Veillonella                           5067.7924258   -0.066708792 0.05355709
## Veillonellaceae_[G.1]                    6.1442803    0.170129296 0.14896161
## Phylum.Firmicutes.                       0.8912626    0.060114707 0.24613650
## Fusobacterium                          927.1066406   -0.112221562 0.07115393
## Leptotrichia                           683.2517404   -0.038723900 0.07350515
## Gracilibacteria_(GN02)_[G.1]             2.4068360    0.065609836 0.23786387
## Lautropia                               68.4201506   -0.156195553 0.13670665
## Ottowia                                  2.3342613   -0.609015814 0.30435958
## Eikenella                                8.7736001   -0.027427974 0.11439515
## Kingella                                99.8204227    0.023313136 0.09846023
## Neisseria                             1329.0853342   -0.158842351 0.11242940
## Family.Neisseriaceae.                    5.6824349   -0.137544130 0.25843807
## Campylobacter                          257.8128378   -0.067850879 0.06302697
## Cardiobacterium                         14.7982481   -0.063238635 0.09841967
## Aggregatibacter                        275.5218474    0.083632565 0.12978670
## Haemophilus                           2905.9316825   -0.176354872 0.08648759
## Family.Pasteurellaceae.                  2.3978345    0.998281869 0.35046324
## Saccharibacteria_(TM7)_[G.1]            36.0392035   -0.215714865 0.14459586
## Saccharibacteria_(TM7)_[G.3]             7.1137273    0.240982477 0.20145685
## Saccharibacteria_(TM7)_[G.6]             7.2376188   -0.553967246 0.25547652
## Family.Saccharibacteria_(TM7)_[F-1].     1.1003857   -0.258223290 0.31873811
## Saccharibacteria_(TM7)_[G.5]             0.9162822    0.327480086 0.30191987
## Treponema                               63.1946030    0.350011623 0.13666168
## Fretibacterium                           8.2679474    0.194159290 0.16614101
## Kingdom.Bacteria.                        9.3689884    0.142679703 0.21007564
## Others                                  33.1524239   -0.060289688 0.13973719
##                                               stat         pval    ordering
## Absconditabacteria_(SR1)_[G.1]        0.5051620387 0.4772407060 Male>Female
## Actinomyces                           1.8503068954 0.1737477048 Female>Male
## Peptidiphaga                          4.0593395065 0.0439276780 Female>Male
## Schaalia                              3.3228119098 0.0683248747 Male>Female
## Family.Actinomycetaceae.              3.4398625311 0.0636409877 Female>Male
## Rothia                                0.0186081008 0.8914959730 Male>Female
## Alloscardovia                         3.5958210613 0.0579250080 Male>Female
## Bifidobacterium                       0.0304473723 0.8614790105 Female>Male
## Parascardovia                         4.0730870065 0.0435715770 Male>Female
## Scardovia                             1.7583110876 0.1848353150 Male>Female
## Corynebacterium                       0.1546593642 0.6941218843 Female>Male
## Olsenella                             1.2472781263 0.2640729778 Male>Female
## Atopobium                             3.5689510470 0.0588694872 Male>Female
## Slackia                               3.4438171617 0.0634888508 Male>Female
## Cryptobacterium                      11.3846017494 0.0007405543 Male>Female
## Bacteroidetes_[G.3]                   4.8003203259 0.0284544460 Male>Female
## Bacteroidetes_[G.5]                   0.1961755219 0.6578257274 Male>Female
## Family.Bacteroidetes_[F-1].           0.0665064758 0.7964929644 Male>Female
## Bacteroidaceae_[G.1]                  2.9502480130 0.0858643799 Female>Male
## Bacteroidales_[G.2]                   0.3559291122 0.5507758412 Male>Female
## Porphyromonas                         0.5740848939 0.4486402389 Female>Male
## Tannerella                            1.8356531798 0.1754612768 Female>Male
## Alloprevotella                        6.6432110877 0.0099534370 Male>Female
```

```
## Prevotella                              0.5390003510 0.4628473089 Male>Female
## Bergeyella                              4.7956203396 0.0285321802 Female>Male
## Capnocytophaga                          3.7856425079 0.0516940602 Female>Male
## Gemella                                10.7596487089 0.0010373707 Female>Male
## Abiotrophia                             3.6802865846 0.0550593856 Female>Male
## Granulicatella                          0.2997471910 0.5840409527 Female>Male
## Lactobacillus                           1.8541508369 0.1733013982 Female>Male
## Streptococcus                           2.5052977239 0.1134640402 Male>Female
## Order.Lactobacillales.                 -2.7678694377 1.0000000000 Female>Male
## Class.Bacilli.                          6.7544044111 0.0093516556 Female>Male
## Clostridiales_[F.1][G-1]                3.1064524800 0.0779826425 Male>Female
## Butyrivibrio                            0.1967378572 0.6573669393 Female>Male
## Catonella                               0.1163777214 0.7329968417 Female>Male
## Johnsonella                             0.2427473924 0.6222288072 Female>Male
## Lachnoanaerobaculum                     0.9917597402 0.3193126586 Female>Male
## Lachnospiraceae_[G.2]                   1.2330150346 0.2668214859 Female>Male
## Lachnospiraceae_[G.3]                   1.5352956018 0.2153194060 Female>Male
## Lachnospiraceae_[G.7]                  12.1424441464 0.0004928714 Male>Female
## Lachnospiraceae_[G.8]                   1.0953257927 0.2952941907 Female>Male
## Oribacterium                            0.9000684951 0.3427633458 Male>Female
## Shuttleworthia                          2.2447049342 0.1340724809 Male>Female
## Stomatobaculum                          0.8860088939 0.3465611563 Female>Male
## Family.Lachnospiraceae_[XIV].           0.7558058296 0.3846442731 Female>Male
## Peptococcus                             0.8194749803 0.3653337140 Female>Male
## Parvimonas                              0.3882803638 0.5332047032 Male>Female
## Filifactor                              1.3218801244 0.2502549057 Male>Female
## Mogibacterium                          12.5345356170 0.0003994989 Female>Male
## Peptostreptococcaceae_[XI][G.1]         2.9471094211 0.0860313097 Female>Male
## Peptostreptococcaceae_[XI][G.4]        -0.4693801438 1.0000000000 Female>Male
## Peptostreptococcaceae_[XI][G.5]         1.8317475805 0.1759212694 Male>Female
## Peptostreptococcaceae_[XI][G.6]         0.4404490036 0.5069058200 Male>Female
## Peptostreptococcaceae_[XI][G.7]         1.5033947915 0.2201497558 Male>Female
## Peptostreptococcaceae_[XI][G.9]         0.2882434219 0.5913483918 Male>Female
## Peptostreptococcus                      4.5993429620 0.0319842117 Female>Male
## Ruminococcaceae_[G.1]                   2.4557529255 0.1170950092 Male>Female
## Ruminococcaceae_[G.2]                   0.0004181484 0.9836854524 Female>Male
## Bulleidia                               6.4695327121 0.0109739383 Male>Female
## Eggerthia                              14.2764193115 0.0001578298 Male>Female
## Solobacterium                           0.1337760988 0.7145485351 Female>Male
## Mycoplasma                              0.2391987847 0.6247853944 Male>Female
## Mitsuokella                            10.0624717829 0.0015132007 Male>Female
## Selenomonas                             0.1418976934 0.7064016894 Female>Male
## Family.Selenomonadaceae.               11.4954880225 0.0006976534 Male>Female
## Class.Negativicutes.                    0.1659515840 0.6837351153 Female>Male
## Anaeroglobus                            1.6352921514 0.2009726294 Female>Male
## Dialister                               3.5370147304 0.0600133823 Male>Female
## Megasphaera                             2.4274343579 0.1192278957 Male>Female
## Family.Veillonellaceae.                 2.3640829152 0.1241568577 Male>Female
## Veillonella                             1.5533047801 0.2126482282 Female>Male
## Veillonellaceae_[G.1]                   1.3060747673 0.2531065717 Male>Female
## Phylum.Firmicutes.                      0.1288200246 0.7196586286 Male>Female
## Fusobacterium                           2.4931321073 0.1143439664 Female>Male
## Leptotrichia                            0.2777934311 0.5981511407 Female>Male
## Gracilibacteria_(GN02)_[G.1]            0.0840943091 0.7718237441 Male>Female
```

```
## Lautropia                              1.3100053425 0.2523936881 Female>Male
## Ottowia                                4.1017259238 0.0428394610 Female>Male
## Eikenella                              0.0805280503 0.7765830848 Female>Male
## Kingella                               0.0563807392 0.8123105844 Male>Female
## Neisseria                              2.0017690119 0.1571157462 Female>Male
## Family.Neisseriaceae.                  0.2923926476 0.5886913295 Female>Male
## Campylobacter                          1.1647471730 0.2804831898 Female>Male
## Cardiobacterium                        0.4320936135 0.5109634282 Female>Male
## Aggregatibacter                        0.4144681390 0.5197103222 Male>Female
## Haemophilus                            4.1704236143 0.0411355135 Female>Male
## Family.Pasteurellaceae.                7.9148926069 0.0049029491 Male>Female
## Saccharibacteria_(TM7)_[G.1]           2.2337137091 0.1350289524 Female>Male
## Saccharibacteria_(TM7)_[G.3]           1.4183303651 0.2336784124 Male>Female
## Saccharibacteria_(TM7)_[G.6]           4.7511727547 0.0292783341 Female>Male
## Family.Saccharibacteria_(TM7)_[F-1].   0.6779968912 0.4102773419 Female>Male
## Saccharibacteria_(TM7)_[G.5]           1.3162765478 0.2512613888 Male>Female
## Treponema                              6.5144957976 0.0106998654 Male>Female
## Fretibacterium                         1.3773427085 0.2405542998 Male>Female
## Kingdom.Bacteria.                      0.4561965734 0.4994068949 Male>Female
## Others                                 0.1884652800 0.6641970329 Female>Male
##                                          pval.adj
## Absconditabacteria_(SR1)_[G.1]         0.69093057
## Actinomyces                            0.39684565
## Peptidiphaga                           0.21304924
## Schaalia                               0.23669689
## Family.Actinomycetaceae.               0.22863614
## Rothia                                 0.91994797
## Alloscardovia                          0.22863614
## Bifidobacterium                        0.89853187
## Parascardovia                          0.21304924
## Scardovia                              0.40747785
## Corynebacterium                        0.80154551
## Olsenella                              0.46217293
## Atopobium                              0.22863614
## Slackia                                0.22863614
## Cryptobacterium                        0.01436675
## Bacteroidetes_[G.3]                    0.18933323
## Bacteroidetes_[G.5]                    0.78569649
## Family.Bacteroidetes_[F-1].            0.84900898
## Bacteroidaceae_[G.1]                   0.26919474
## Bacteroidales_[G.2]                    0.73185283
## Porphyromonas                          0.66950928
## Tannerella                             0.39684565
## Alloprevotella                         0.08870600
## Prevotella                             0.68024529
## Bergeyella                             0.18933323
## Capnocytophaga                         0.22863614
## Gemella                                0.01677083
## Abiotrophia                            0.22863614
## Granulicatella                         0.75351507
## Lactobacillus                          0.39684565
## Streptococcus                          0.33043160
## Order.Lactobacillales.                 1.00000000
## Class.Bacilli.                         0.08870600
```

```
## Clostridiales_[F.1][G-1]            0.26083849
## Butyrivibrio                        0.78569649
## Catonella                           0.80796243
## Johnsonella                         0.76714156
## Lachnoanaerobaculum                 0.52497166
## Lachnospiraceae_[G.2]               0.46217293
## Lachnospiraceae_[G.3]               0.44438260
## Lachnospiraceae_[G.7]               0.01436675
## Lachnospiraceae_[G.8]               0.49385408
## Oribacterium                        0.55108905
## Shuttleworthia                      0.34467917
## Stomatobaculum                      0.55108905
## Family.Lachnospiraceae_[XIV].       0.59223007
## Peptococcus                         0.57157049
## Parvimonas                          0.71834523
## Filifactor                          0.45465440
## Mogibacterium                       0.01436675
## Peptostreptococcaceae_[XI][G.1]     0.26919474
## Peptostreptococcaceae_[XI][G.4]     1.00000000
## Peptostreptococcaceae_[XI][G.5]     0.39684565
## Peptostreptococcaceae_[XI][G.6]     0.70804932
## Peptostreptococcaceae_[XI][G.7]     0.44488596
## Peptostreptococcaceae_[XI][G.9]     0.75351507
## Peptostreptococcus                  0.19390428
## Ruminococcaceae_[G.1]               0.33043160
## Ruminococcaceae_[G.2]               1.00000000
## Bulleidia                           0.08870600
## Eggerthia                           0.01436675
## Solobacterium                       0.80237801
## Mycoplasma                          0.76714156
## Mitsuokella                         0.02096864
## Selenomonas                         0.80237801
## Family.Selenomonadaceae.            0.01436675
## Class.Negativicutes.                0.79906393
## Anaeroglobus                        0.43320767
## Dialister                           0.22863614
## Megasphaera                         0.33043160
## Family.Veillonellaceae.             0.33453376
## Veillonella                         0.44438260
## Veillonellaceae_[G.1]               0.45465440
## Phylum.Firmicutes.                  0.80237801
## Fusobacterium                       0.33043160
## Leptotrichia                        0.75351507
## Gracilibacteria_(GN02)_[G.1]        0.83698399
## Lautropia                           0.45465440
## Ottowia                             0.21304924
## Eikenella                           0.83698399
## Kingella                            0.85645790
## Neisseria                           0.39077506
## Family.Neisseriaceae.               0.75351507
## Campylobacter                       0.47731350
## Cardiobacterium                     0.70804932
## Aggregatibacter                     0.71002678
## Haemophilus                         0.21304924
```

```
## Family.Pasteurellaceae.             0.05944826
## Saccharibacteria_(TM7)_[G.1]        0.34467917
## Saccharibacteria_(TM7)_[G.3]        0.45465440
## Saccharibacteria_(TM7)_[G.6]        0.18933323
## Family.Saccharibacteria_(TM7)_[F-1]. 0.62182660
## Saccharibacteria_(TM7)_[G.5]        0.45465440
## Treponema                           0.08870600
## Fretibacterium                      0.45465440
## Kingdom.Bacteria.                   0.70804932
## Others                              0.78569649
##                                                                 Feature
## Absconditabacteria_(SR1)_[G.1]           Absconditabacteria_(SR1)_[G.1]
## Actinomyces                                                 Actinomyces
## Peptidiphaga                                               Peptidiphaga
## Schaalia                                                       Schaalia
## Family.Actinomycetaceae.                       Family.Actinomycetaceae.
## Rothia                                                           Rothia
## Alloscardovia                                             Alloscardovia
## Bifidobacterium                                         Bifidobacterium
## Parascardovia                                             Parascardovia
## Scardovia                                                     Scardovia
## Corynebacterium                                         Corynebacterium
## Olsenella                                                     Olsenella
## Atopobium                                                     Atopobium
## Slackia                                                         Slackia
## Cryptobacterium                                         Cryptobacterium
## Bacteroidetes_[G.3]                                 Bacteroidetes_[G.3]
## Bacteroidetes_[G.5]                                 Bacteroidetes_[G.5]
## Family.Bacteroidetes_[F-1].                   Family.Bacteroidetes_[F-1].
## Bacteroidaceae_[G.1]                               Bacteroidaceae_[G.1]
## Bacteroidales_[G.2]                                 Bacteroidales_[G.2]
## Porphyromonas                                             Porphyromonas
## Tannerella                                                   Tannerella
## Alloprevotella                                           Alloprevotella
## Prevotella                                                   Prevotella
## Bergeyella                                                   Bergeyella
## Capnocytophaga                                           Capnocytophaga
## Gemella                                                         Gemella
## Abiotrophia                                                 Abiotrophia
## Granulicatella                                           Granulicatella
## Lactobacillus                                             Lactobacillus
## Streptococcus                                             Streptococcus
## Order.Lactobacillales.                           Order.Lactobacillales.
## Class.Bacilli.                                           Class.Bacilli.
## Clostridiales_[F.1][G-1]                       Clostridiales_[F.1][G-1]
## Butyrivibrio                                               Butyrivibrio
## Catonella                                                     Catonella
## Johnsonella                                                 Johnsonella
## Lachnoanaerobaculum                                 Lachnoanaerobaculum
## Lachnospiraceae_[G.2]                             Lachnospiraceae_[G.2]
## Lachnospiraceae_[G.3]                             Lachnospiraceae_[G.3]
## Lachnospiraceae_[G.7]                             Lachnospiraceae_[G.7]
## Lachnospiraceae_[G.8]                             Lachnospiraceae_[G.8]
## Oribacterium                                               Oribacterium
```

```
## Shuttleworthia                                       Shuttleworthia
## Stomatobaculum                                       Stomatobaculum
## Family.Lachnospiraceae_[XIV].         Family.Lachnospiraceae_[XIV].
## Peptococcus                                             Peptococcus
## Parvimonas                                               Parvimonas
## Filifactor                                               Filifactor
## Mogibacterium                                         Mogibacterium
## Peptostreptococcaceae_[XI][G.1]     Peptostreptococcaceae_[XI][G.1]
## Peptostreptococcaceae_[XI][G.4]     Peptostreptococcaceae_[XI][G.4]
## Peptostreptococcaceae_[XI][G.5]     Peptostreptococcaceae_[XI][G.5]
## Peptostreptococcaceae_[XI][G.6]     Peptostreptococcaceae_[XI][G.6]
## Peptostreptococcaceae_[XI][G.7]     Peptostreptococcaceae_[XI][G.7]
## Peptostreptococcaceae_[XI][G.9]     Peptostreptococcaceae_[XI][G.9]
## Peptostreptococcus                               Peptostreptococcus
## Ruminococcaceae_[G.1]                         Ruminococcaceae_[G.1]
## Ruminococcaceae_[G.2]                         Ruminococcaceae_[G.2]
## Bulleidia                                                 Bulleidia
## Eggerthia                                                 Eggerthia
## Solobacterium                                         Solobacterium
## Mycoplasma                                               Mycoplasma
## Mitsuokella                                             Mitsuokella
## Selenomonas                                             Selenomonas
## Family.Selenomonadaceae.                   Family.Selenomonadaceae.
## Class.Negativicutes.                           Class.Negativicutes.
## Anaeroglobus                                           Anaeroglobus
## Dialister                                                 Dialister
## Megasphaera                                             Megasphaera
## Family.Veillonellaceae.                     Family.Veillonellaceae.
## Veillonella                                             Veillonella
## Veillonellaceae_[G.1]                         Veillonellaceae_[G.1]
## Phylum.Firmicutes.                               Phylum.Firmicutes.
## Fusobacterium                                         Fusobacterium
## Leptotrichia                                           Leptotrichia
## Gracilibacteria_(GN02)_[G.1]           Gracilibacteria_(GN02)_[G.1]
## Lautropia                                                 Lautropia
## Ottowia                                                     Ottowia
## Eikenella                                                 Eikenella
## Kingella                                                   Kingella
## Neisseria                                                 Neisseria
## Family.Neisseriaceae.                         Family.Neisseriaceae.
## Campylobacter                                         Campylobacter
## Cardiobacterium                                     Cardiobacterium
## Aggregatibacter                                     Aggregatibacter
## Haemophilus                                             Haemophilus
## Family.Pasteurellaceae.                     Family.Pasteurellaceae.
## Saccharibacteria_(TM7)_[G.1]           Saccharibacteria_(TM7)_[G.1]
## Saccharibacteria_(TM7)_[G.3]           Saccharibacteria_(TM7)_[G.3]
## Saccharibacteria_(TM7)_[G.6]           Saccharibacteria_(TM7)_[G.6]
## Family.Saccharibacteria_(TM7)_[F-1]. Family.Saccharibacteria_(TM7)_[F-1].
## Saccharibacteria_(TM7)_[G.5]           Saccharibacteria_(TM7)_[G.5]
## Treponema                                                 Treponema
## Fretibacterium                                       Fretibacterium
## Kingdom.Bacteria.                                 Kingdom.Bacteria.
## Others                                                       Others
```

```
##                                                      Method
## Absconditabacteria_(SR1)_[G.1]       DESeq2 man. geoMeans (ds2)
## Actinomyces                          DESeq2 man. geoMeans (ds2)
## Peptidiphaga                         DESeq2 man. geoMeans (ds2)
## Schaalia                             DESeq2 man. geoMeans (ds2)
## Family.Actinomycetaceae.             DESeq2 man. geoMeans (ds2)
## Rothia                               DESeq2 man. geoMeans (ds2)
## Alloscardovia                        DESeq2 man. geoMeans (ds2)
## Bifidobacterium                      DESeq2 man. geoMeans (ds2)
## Parascardovia                        DESeq2 man. geoMeans (ds2)
## Scardovia                            DESeq2 man. geoMeans (ds2)
## Corynebacterium                      DESeq2 man. geoMeans (ds2)
## Olsenella                            DESeq2 man. geoMeans (ds2)
## Atopobium                            DESeq2 man. geoMeans (ds2)
## Slackia                              DESeq2 man. geoMeans (ds2)
## Cryptobacterium                      DESeq2 man. geoMeans (ds2)
## Bacteroidetes_[G.3]                  DESeq2 man. geoMeans (ds2)
## Bacteroidetes_[G.5]                  DESeq2 man. geoMeans (ds2)
## Family.Bacteroidetes_[F-1].          DESeq2 man. geoMeans (ds2)
## Bacteroidaceae_[G.1]                 DESeq2 man. geoMeans (ds2)
## Bacteroidales_[G.2]                  DESeq2 man. geoMeans (ds2)
## Porphyromonas                        DESeq2 man. geoMeans (ds2)
## Tannerella                           DESeq2 man. geoMeans (ds2)
## Alloprevotella                       DESeq2 man. geoMeans (ds2)
## Prevotella                           DESeq2 man. geoMeans (ds2)
## Bergeyella                           DESeq2 man. geoMeans (ds2)
## Capnocytophaga                       DESeq2 man. geoMeans (ds2)
## Gemella                              DESeq2 man. geoMeans (ds2)
## Abiotrophia                          DESeq2 man. geoMeans (ds2)
## Granulicatella                       DESeq2 man. geoMeans (ds2)
## Lactobacillus                        DESeq2 man. geoMeans (ds2)
## Streptococcus                        DESeq2 man. geoMeans (ds2)
## Order.Lactobacillales.               DESeq2 man. geoMeans (ds2)
## Class.Bacilli.                       DESeq2 man. geoMeans (ds2)
## Clostridiales_[F.1][G-1]             DESeq2 man. geoMeans (ds2)
## Butyrivibrio                         DESeq2 man. geoMeans (ds2)
## Catonella                            DESeq2 man. geoMeans (ds2)
## Johnsonella                          DESeq2 man. geoMeans (ds2)
## Lachnoanaerobaculum                  DESeq2 man. geoMeans (ds2)
## Lachnospiraceae_[G.2]                DESeq2 man. geoMeans (ds2)
## Lachnospiraceae_[G.3]                DESeq2 man. geoMeans (ds2)
## Lachnospiraceae_[G.7]                DESeq2 man. geoMeans (ds2)
## Lachnospiraceae_[G.8]                DESeq2 man. geoMeans (ds2)
## Oribacterium                         DESeq2 man. geoMeans (ds2)
## Shuttleworthia                       DESeq2 man. geoMeans (ds2)
## Stomatobaculum                       DESeq2 man. geoMeans (ds2)
## Family.Lachnospiraceae_[XIV].        DESeq2 man. geoMeans (ds2)
## Peptococcus                          DESeq2 man. geoMeans (ds2)
## Parvimonas                           DESeq2 man. geoMeans (ds2)
## Filifactor                           DESeq2 man. geoMeans (ds2)
## Mogibacterium                        DESeq2 man. geoMeans (ds2)
## Peptostreptococcaceae_[XI][G.1]      DESeq2 man. geoMeans (ds2)
## Peptostreptococcaceae_[XI][G.4]      DESeq2 man. geoMeans (ds2)
## Peptostreptococcaceae_[XI][G.5]      DESeq2 man. geoMeans (ds2)
```

```
## Peptostreptococcaceae_[XI][G.6]      DESeq2 man. geoMeans (ds2)
## Peptostreptococcaceae_[XI][G.7]      DESeq2 man. geoMeans (ds2)
## Peptostreptococcaceae_[XI][G.9]      DESeq2 man. geoMeans (ds2)
## Peptostreptococcus                   DESeq2 man. geoMeans (ds2)
## Ruminococcaceae_[G.1]                DESeq2 man. geoMeans (ds2)
## Ruminococcaceae_[G.2]                DESeq2 man. geoMeans (ds2)
## Bulleidia                            DESeq2 man. geoMeans (ds2)
## Eggerthia                            DESeq2 man. geoMeans (ds2)
## Solobacterium                        DESeq2 man. geoMeans (ds2)
## Mycoplasma                           DESeq2 man. geoMeans (ds2)
## Mitsuokella                          DESeq2 man. geoMeans (ds2)
## Selenomonas                          DESeq2 man. geoMeans (ds2)
## Family.Selenomonadaceae.             DESeq2 man. geoMeans (ds2)
## Class.Negativicutes.                 DESeq2 man. geoMeans (ds2)
## Anaeroglobus                         DESeq2 man. geoMeans (ds2)
## Dialister                            DESeq2 man. geoMeans (ds2)
## Megasphaera                          DESeq2 man. geoMeans (ds2)
## Family.Veillonellaceae.              DESeq2 man. geoMeans (ds2)
## Veillonella                          DESeq2 man. geoMeans (ds2)
## Veillonellaceae_[G.1]                DESeq2 man. geoMeans (ds2)
## Phylum.Firmicutes.                   DESeq2 man. geoMeans (ds2)
## Fusobacterium                        DESeq2 man. geoMeans (ds2)
## Leptotrichia                         DESeq2 man. geoMeans (ds2)
## Gracilibacteria_(GN02)_[G.1]         DESeq2 man. geoMeans (ds2)
## Lautropia                            DESeq2 man. geoMeans (ds2)
## Ottowia                              DESeq2 man. geoMeans (ds2)
## Eikenella                            DESeq2 man. geoMeans (ds2)
## Kingella                             DESeq2 man. geoMeans (ds2)
## Neisseria                            DESeq2 man. geoMeans (ds2)
## Family.Neisseriaceae.                DESeq2 man. geoMeans (ds2)
## Campylobacter                        DESeq2 man. geoMeans (ds2)
## Cardiobacterium                      DESeq2 man. geoMeans (ds2)
## Aggregatibacter                      DESeq2 man. geoMeans (ds2)
## Haemophilus                          DESeq2 man. geoMeans (ds2)
## Family.Pasteurellaceae.              DESeq2 man. geoMeans (ds2)
## Saccharibacteria_(TM7)_[G.1]         DESeq2 man. geoMeans (ds2)
## Saccharibacteria_(TM7)_[G.3]         DESeq2 man. geoMeans (ds2)
## Saccharibacteria_(TM7)_[G.6]         DESeq2 man. geoMeans (ds2)
## Family.Saccharibacteria_(TM7)_[F-1]. DESeq2 man. geoMeans (ds2)
## Saccharibacteria_(TM7)_[G.5]         DESeq2 man. geoMeans (ds2)
## Treponema                            DESeq2 man. geoMeans (ds2)
## Fretibacterium                       DESeq2 man. geoMeans (ds2)
## Kingdom.Bacteria.                    DESeq2 man. geoMeans (ds2)
## Others                               DESeq2 man. geoMeans (ds2)
```

```
write.table(final, file="sex_cat_ds2.txt", sep="\t", dec=",", row.names=F)
write.table(final[final$pval<0.05,], file="sex_cat_ds2_sig.txt", sep="\t", dec=",", row.names=F)
final <- DA.kru(Microbio, predictor = Phe$sex_cat)
final
```

```
##                                              pval    pval.adj
## Absconditabacteria_(SR1)_[G.1]       2.235808e-02 0.074589294
## Actinomyces                          6.346818e-01 0.769551714
## Peptidiphaga                         1.266799e-02 0.065427209
```

```
## Schaalia                               5.620048e-03 0.045428719
## Family.Actinomycetaceae.               1.276794e-01 0.283103630
## Rothia                                 3.948640e-01 0.580378411
## Alloscardovia                          3.888821e-04 0.008977511
## Bifidobacterium                        8.609732e-03 0.059653143
## Parascardovia                          6.569075e-03 0.049015404
## Scardovia                              6.951176e-01 0.809836995
## Corynebacterium                        7.813330e-01 0.841518867
## Olsenella                              1.284181e-01 0.283103630
## Atopobium                              1.281564e-02 0.065427209
## Slackia                                2.096772e-01 0.391128596
## Cryptobacterium                        1.350423e-03 0.016373877
## Bacteroidetes_[G.3]                    2.877401e-05 0.001395540
## Bacteroidetes_[G.5]                    2.333275e-02 0.074589294
## Family.Bacteroidetes_[F-1].            4.902066e-01 0.679286253
## Bacteroidaceae_[G.1]                   6.044934e-01 0.753679998
## Bacteroidales_[G.2]                    1.151615e-01 0.265968176
## Porphyromonas                          4.051810e-01 0.586605256
## Tannerella                             9.629858e-01 0.962985844
## Alloprevotella                         5.184277e-02 0.135912131
## Prevotella                             2.166253e-02 0.074589294
## Bergeyella                             2.418463e-01 0.434427614
## Capnocytophaga                         3.955114e-02 0.109613162
## Gemella                                4.687485e-04 0.008977511
## Abiotrophia                            2.106913e-02 0.074589294
## Granulicatella                         1.409236e-01 0.292824485
## Lactobacillus                          5.595088e-03 0.045428719
## Streptococcus                          5.798335e-01 0.749918023
## Order.Lactobacillales.                 2.579038e-01 0.446820846
## Class.Bacilli.                         2.625648e-01 0.446820846
## Clostridiales_[F.1][G-1]               1.363196e-02 0.066115022
## Butyrivibrio                           2.242044e-01 0.410336434
## Catonella                              5.084602e-01 0.694656838
## Johnsonella                            8.956969e-01 0.905027036
## Lachnoanaerobaculum                    5.640525e-01 0.749494389
## Lachnospiraceae_[G.2]                  8.095202e-01 0.849687625
## Lachnospiraceae_[G.3]                  6.449775e-01 0.772380509
## Lachnospiraceae_[G.7]                  1.545290e-05 0.001395540
## Lachnospiraceae_[G.8]                  7.013021e-01 0.809836995
## Oribacterium                           5.122904e-02 0.135912131
## Shuttleworthia                         5.553099e-04 0.008977511
## Stomatobaculum                         3.665057e-01 0.564302404
## Family.Lachnospiraceae_[XIV].          8.909751e-01 0.905027036
## Peptococcus                            4.280026e-01 0.610533086
## Parvimonas                             5.513215e-01 0.742752580
## Filifactor                             3.628566e-03 0.035197094
## Mogibacterium                          1.152983e-02 0.065427209
## Peptostreptococcaceae_[XI][G.1]        2.056563e-01 0.391128596
## Peptostreptococcaceae_[XI][G.4]        5.986908e-01 0.753679998
## Peptostreptococcaceae_[XI][G.5]        3.248106e-01 0.516502175
## Peptostreptococcaceae_[XI][G.6]        8.283025e-02 0.195964246
## Peptostreptococcaceae_[XI][G.7]        2.949299e-01 0.484884734
## Peptostreptococcaceae_[XI][G.9]        6.060520e-01 0.753679998
## Peptostreptococcus                     2.677487e-01 0.447786580
```

```
## Ruminococcaceae_[G.1]              3.108599e-02 0.091373964
## Ruminococcaceae_[G.2]              3.948967e-01 0.580378411
## Bulleidia                          2.361946e-03 0.025456529
## Eggerthia                          1.241319e-03 0.016373877
## Solobacterium                      7.498247e-01 0.836011471
## Mycoplasma                         1.347634e-01 0.290489958
## Mitsuokella                        5.885140e-05 0.001902862
## Selenomonas                        7.667032e-01 0.841518867
## Family.Selenomonadaceae.           2.195629e-02 0.074589294
## Class.Negativicutes.               7.338495e-01 0.827714005
## Anaeroglobus                       5.776014e-01 0.749918023
## Dialister                          5.859611e-02 0.149574280
## Megasphaera                        2.047325e-02 0.074589294
## Family.Veillonellaceae.            7.390677e-02 0.179223920
## Veillonella                        7.894662e-01 0.841518867
## Veillonellaceae_[G.1]              2.255054e-02 0.074589294
## Phylum.Firmicutes.                 7.116242e-02 0.176993708
## Fusobacterium                      1.739065e-01 0.337378692
## Leptotrichia                       6.263153e-01 0.769020074
## Gracilibacteria_(GN02)_[G.1]       7.822327e-01 0.841518867
## Lautropia                          4.782109e-01 0.672267546
## Ottowia                            2.701276e-02 0.081882435
## Eikenella                          2.622972e-01 0.446820846
## Kingella                           8.872562e-01 0.905027036
## Neisseria                          1.625823e-01 0.328551710
## Family.Neisseriaceae.              6.797007e-01 0.804036166
## Campylobacter                      3.409234e-01 0.533380119
## Cardiobacterium                    1.677672e-01 0.332110580
## Aggregatibacter                    3.936684e-01 0.580378411
## Haemophilus                        1.443028e-02 0.066654168
## Family.Pasteurellaceae.            3.899916e-02 0.109613162
## Saccharibacteria_(TM7)_[G.1]       8.146490e-01 0.849687625
## Saccharibacteria_(TM7)_[G.3]       2.383782e-02 0.074589294
## Saccharibacteria_(TM7)_[G.6]       7.137185e-01 0.814478800
## Family.Saccharibacteria_(TM7)_[F-1]. 3.200330e-01 0.516502175
## Saccharibacteria_(TM7)_[G.5]       1.908881e-02 0.074589294
## Treponema                          1.001798e-02 0.064782964
## Fretibacterium                     1.418840e-01 0.292824485
## Kingdom.Bacteria.                  1.945374e-02 0.074589294
## Others                             1.226361e-02 0.065427209
##                                                               Feature
## Absconditabacteria_(SR1)_[G.1]         Absconditabacteria_(SR1)_[G.1]
## Actinomyces                                               Actinomyces
## Peptidiphaga                                             Peptidiphaga
## Schaalia                                                     Schaalia
## Family.Actinomycetaceae.                     Family.Actinomycetaceae.
## Rothia                                                         Rothia
## Alloscardovia                                           Alloscardovia
## Bifidobacterium                                       Bifidobacterium
## Parascardovia                                           Parascardovia
## Scardovia                                                   Scardovia
## Corynebacterium                                       Corynebacterium
## Olsenella                                                   Olsenella
## Atopobium                                                   Atopobium
```

```
## Slackia                                                       Slackia
## Cryptobacterium                                       Cryptobacterium
## Bacteroidetes_[G.3]                               Bacteroidetes_[G.3]
## Bacteroidetes_[G.5]                               Bacteroidetes_[G.5]
## Family.Bacteroidetes_[F-1].               Family.Bacteroidetes_[F-1].
## Bacteroidaceae_[G.1]                             Bacteroidaceae_[G.1]
## Bacteroidales_[G.2]                               Bacteroidales_[G.2]
## Porphyromonas                                           Porphyromonas
## Tannerella                                                 Tannerella
## Alloprevotella                                         Alloprevotella
## Prevotella                                                 Prevotella
## Bergeyella                                                 Bergeyella
## Capnocytophaga                                         Capnocytophaga
## Gemella                                                       Gemella
## Abiotrophia                                               Abiotrophia
## Granulicatella                                         Granulicatella
## Lactobacillus                                           Lactobacillus
## Streptococcus                                           Streptococcus
## Order.Lactobacillales.                       Order.Lactobacillales.
## Class.Bacilli.                                         Class.Bacilli.
## Clostridiales_[F.1][G-1]                   Clostridiales_[F.1][G-1]
## Butyrivibrio                                             Butyrivibrio
## Catonella                                                   Catonella
## Johnsonella                                               Johnsonella
## Lachnoanaerobaculum                               Lachnoanaerobaculum
## Lachnospiraceae_[G.2]                           Lachnospiraceae_[G.2]
## Lachnospiraceae_[G.3]                           Lachnospiraceae_[G.3]
## Lachnospiraceae_[G.7]                           Lachnospiraceae_[G.7]
## Lachnospiraceae_[G.8]                           Lachnospiraceae_[G.8]
## Oribacterium                                             Oribacterium
## Shuttleworthia                                         Shuttleworthia
## Stomatobaculum                                         Stomatobaculum
## Family.Lachnospiraceae_[XIV].           Family.Lachnospiraceae_[XIV].
## Peptococcus                                               Peptococcus
## Parvimonas                                                 Parvimonas
## Filifactor                                                 Filifactor
## Mogibacterium                                           Mogibacterium
## Peptostreptococcaceae_[XI][G.1]       Peptostreptococcaceae_[XI][G.1]
## Peptostreptococcaceae_[XI][G.4]       Peptostreptococcaceae_[XI][G.4]
## Peptostreptococcaceae_[XI][G.5]       Peptostreptococcaceae_[XI][G.5]
## Peptostreptococcaceae_[XI][G.6]       Peptostreptococcaceae_[XI][G.6]
## Peptostreptococcaceae_[XI][G.7]       Peptostreptococcaceae_[XI][G.7]
## Peptostreptococcaceae_[XI][G.9]       Peptostreptococcaceae_[XI][G.9]
## Peptostreptococcus                                 Peptostreptococcus
## Ruminococcaceae_[G.1]                           Ruminococcaceae_[G.1]
## Ruminococcaceae_[G.2]                           Ruminococcaceae_[G.2]
## Bulleidia                                                   Bulleidia
## Eggerthia                                                   Eggerthia
## Solobacterium                                           Solobacterium
## Mycoplasma                                                 Mycoplasma
## Mitsuokella                                               Mitsuokella
## Selenomonas                                               Selenomonas
## Family.Selenomonadaceae.                   Family.Selenomonadaceae.
## Class.Negativicutes.                           Class.Negativicutes.
```

```
## Anaeroglobus                                               Anaeroglobus
## Dialister                                                     Dialister
## Megasphaera                                                 Megasphaera
## Family.Veillonellaceae.                         Family.Veillonellaceae.
## Veillonella                                                 Veillonella
## Veillonellaceae_[G.1]                             Veillonellaceae_[G.1]
## Phylum.Firmicutes.                                   Phylum.Firmicutes.
## Fusobacterium                                             Fusobacterium
## Leptotrichia                                               Leptotrichia
## Gracilibacteria_(GN02)_[G.1]               Gracilibacteria_(GN02)_[G.1]
## Lautropia                                                     Lautropia
## Ottowia                                                         Ottowia
## Eikenella                                                     Eikenella
## Kingella                                                       Kingella
## Neisseria                                                     Neisseria
## Family.Neisseriaceae.                             Family.Neisseriaceae.
## Campylobacter                                             Campylobacter
## Cardiobacterium                                         Cardiobacterium
## Aggregatibacter                                         Aggregatibacter
## Haemophilus                                                 Haemophilus
## Family.Pasteurellaceae.                         Family.Pasteurellaceae.
## Saccharibacteria_(TM7)_[G.1]               Saccharibacteria_(TM7)_[G.1]
## Saccharibacteria_(TM7)_[G.3]               Saccharibacteria_(TM7)_[G.3]
## Saccharibacteria_(TM7)_[G.6]               Saccharibacteria_(TM7)_[G.6]
## Family.Saccharibacteria_(TM7)_[F-1]. Family.Saccharibacteria_(TM7)_[F-1].
## Saccharibacteria_(TM7)_[G.5]               Saccharibacteria_(TM7)_[G.5]
## Treponema                                                     Treponema
## Fretibacterium                                           Fretibacterium
## Kingdom.Bacteria.                                     Kingdom.Bacteria.
## Others                                                           Others
##                                                Method
## Absconditabacteria_(SR1)_[G.1]       Kruskal-Wallis (kru)
## Actinomyces                          Kruskal-Wallis (kru)
## Peptidiphaga                         Kruskal-Wallis (kru)
## Schaalia                             Kruskal-Wallis (kru)
## Family.Actinomycetaceae.             Kruskal-Wallis (kru)
## Rothia                               Kruskal-Wallis (kru)
## Alloscardovia                        Kruskal-Wallis (kru)
## Bifidobacterium                      Kruskal-Wallis (kru)
## Parascardovia                        Kruskal-Wallis (kru)
## Scardovia                            Kruskal-Wallis (kru)
## Corynebacterium                      Kruskal-Wallis (kru)
## Olsenella                            Kruskal-Wallis (kru)
## Atopobium                            Kruskal-Wallis (kru)
## Slackia                              Kruskal-Wallis (kru)
## Cryptobacterium                      Kruskal-Wallis (kru)
## Bacteroidetes_[G.3]                  Kruskal-Wallis (kru)
## Bacteroidetes_[G.5]                  Kruskal-Wallis (kru)
## Family.Bacteroidetes_[F-1].          Kruskal-Wallis (kru)
## Bacteroidaceae_[G.1]                 Kruskal-Wallis (kru)
## Bacteroidales_[G.2]                  Kruskal-Wallis (kru)
## Porphyromonas                        Kruskal-Wallis (kru)
## Tannerella                           Kruskal-Wallis (kru)
## Alloprevotella                       Kruskal-Wallis (kru)
```

```
## Prevotella                              Kruskal-Wallis (kru)
## Bergeyella                              Kruskal-Wallis (kru)
## Capnocytophaga                          Kruskal-Wallis (kru)
## Gemella                                 Kruskal-Wallis (kru)
## Abiotrophia                             Kruskal-Wallis (kru)
## Granulicatella                          Kruskal-Wallis (kru)
## Lactobacillus                           Kruskal-Wallis (kru)
## Streptococcus                           Kruskal-Wallis (kru)
## Order.Lactobacillales.                  Kruskal-Wallis (kru)
## Class.Bacilli.                          Kruskal-Wallis (kru)
## Clostridiales_[F.1][G-1]                Kruskal-Wallis (kru)
## Butyrivibrio                            Kruskal-Wallis (kru)
## Catonella                               Kruskal-Wallis (kru)
## Johnsonella                             Kruskal-Wallis (kru)
## Lachnoanaerobaculum                     Kruskal-Wallis (kru)
## Lachnospiraceae_[G.2]                   Kruskal-Wallis (kru)
## Lachnospiraceae_[G.3]                   Kruskal-Wallis (kru)
## Lachnospiraceae_[G.7]                   Kruskal-Wallis (kru)
## Lachnospiraceae_[G.8]                   Kruskal-Wallis (kru)
## Oribacterium                            Kruskal-Wallis (kru)
## Shuttleworthia                          Kruskal-Wallis (kru)
## Stomatobaculum                          Kruskal-Wallis (kru)
## Family.Lachnospiraceae_[XIV].           Kruskal-Wallis (kru)
## Peptococcus                             Kruskal-Wallis (kru)
## Parvimonas                              Kruskal-Wallis (kru)
## Filifactor                              Kruskal-Wallis (kru)
## Mogibacterium                           Kruskal-Wallis (kru)
## Peptostreptococcaceae_[XI][G.1]         Kruskal-Wallis (kru)
## Peptostreptococcaceae_[XI][G.4]         Kruskal-Wallis (kru)
## Peptostreptococcaceae_[XI][G.5]         Kruskal-Wallis (kru)
## Peptostreptococcaceae_[XI][G.6]         Kruskal-Wallis (kru)
## Peptostreptococcaceae_[XI][G.7]         Kruskal-Wallis (kru)
## Peptostreptococcaceae_[XI][G.9]         Kruskal-Wallis (kru)
## Peptostreptococcus                      Kruskal-Wallis (kru)
## Ruminococcaceae_[G.1]                   Kruskal-Wallis (kru)
## Ruminococcaceae_[G.2]                   Kruskal-Wallis (kru)
## Bulleidia                               Kruskal-Wallis (kru)
## Eggerthia                               Kruskal-Wallis (kru)
## Solobacterium                           Kruskal-Wallis (kru)
## Mycoplasma                              Kruskal-Wallis (kru)
## Mitsuokella                             Kruskal-Wallis (kru)
## Selenomonas                             Kruskal-Wallis (kru)
## Family.Selenomonadaceae.                Kruskal-Wallis (kru)
## Class.Negativicutes.                    Kruskal-Wallis (kru)
## Anaeroglobus                            Kruskal-Wallis (kru)
## Dialister                               Kruskal-Wallis (kru)
## Megasphaera                             Kruskal-Wallis (kru)
## Family.Veillonellaceae.                 Kruskal-Wallis (kru)
## Veillonella                             Kruskal-Wallis (kru)
## Veillonellaceae_[G.1]                   Kruskal-Wallis (kru)
## Phylum.Firmicutes.                      Kruskal-Wallis (kru)
## Fusobacterium                           Kruskal-Wallis (kru)
## Leptotrichia                            Kruskal-Wallis (kru)
## Gracilibacteria_(GN02)_[G.1]            Kruskal-Wallis (kru)
```

```
## Lautropia                              Kruskal-Wallis (kru)
## Ottowia                                Kruskal-Wallis (kru)
## Eikenella                              Kruskal-Wallis (kru)
## Kingella                               Kruskal-Wallis (kru)
## Neisseria                              Kruskal-Wallis (kru)
## Family.Neisseriaceae.                  Kruskal-Wallis (kru)
## Campylobacter                          Kruskal-Wallis (kru)
## Cardiobacterium                        Kruskal-Wallis (kru)
## Aggregatibacter                        Kruskal-Wallis (kru)
## Haemophilus                            Kruskal-Wallis (kru)
## Family.Pasteurellaceae.                Kruskal-Wallis (kru)
## Saccharibacteria_(TM7)_[G.1]           Kruskal-Wallis (kru)
## Saccharibacteria_(TM7)_[G.3]           Kruskal-Wallis (kru)
## Saccharibacteria_(TM7)_[G.6]           Kruskal-Wallis (kru)
## Family.Saccharibacteria_(TM7)_[F-1]. Kruskal-Wallis (kru)
## Saccharibacteria_(TM7)_[G.5]           Kruskal-Wallis (kru)
## Treponema                              Kruskal-Wallis (kru)
## Fretibacterium                         Kruskal-Wallis (kru)
## Kingdom.Bacteria.                      Kruskal-Wallis (kru)
## Others                                 Kruskal-Wallis (kru)
```

```
write.table(final, file="sex_cat_kru.txt", sep="\t", dec=",", row.names=F)
write.table(final[final$pval<0.05,], file="sex_cat_kru_sig.txt", sep="\t", dec=",", row.names=F)
#################BMI_cat
table(Phe$BMI_cat, useNA="always")
```

```
##
##    Underweight Healthyweight    Overweight         Obese            UA
##              3           251           337           155             0
##           <NA>
##              0
```

```
Phe2<-subset(Phe, BMI_cat %in% c("Healthyweight", "Overweight", "Obese"))
Phe2$BMI_cat<-droplevels(Phe2$BMI_cat)
#Also subset columns
Microbio2<-dplyr::select(Microbio, one_of(Phe2$IDX))
final <- DA.ds2(Microbio2, predictor = Phe2$BMI_cat, out.all=TRUE) #It is LRT so don't look at ordering
final
```

```
##                                         baseMean log2FoldChange      lfcSE
## Absconditabacteria_(SR1)_[G.1]        66.0149989  -0.0252408744 0.28143864
## Actinomyces                          324.0513694  -0.1467885207 0.09414497
## Peptidiphaga                          21.8499552  -0.0943274737 0.13484123
## Schaalia                             703.9026075   0.0649538972 0.07788265
## Family.Actinomycetaceae.               5.4429073  -0.3771891796 0.19426253
## Rothia                              1939.9892563   0.0148959408 0.09127626
## Alloscardovia                         12.4532857   0.0261468755 0.24006597
## Bifidobacterium                       11.4146226  -0.1288412000 0.26455960
## Parascardovia                          2.8099908  -0.4122544882 0.35986258
## Scardovia                             12.5367587  -0.1011675610 0.22719391
## Corynebacterium                       93.7032092  -0.0552497092 0.09976446
## Olsenella                              1.5479302  -0.2103066386 0.21986949
## Atopobium                            120.3433492  -0.0378455006 0.09149833
```

```
## Slackia                                1.3789738   0.1076320058 0.20912742
## Cryptobacterium                        4.2815372  -0.4658634747 0.22629199
## Bacteroidetes_[G.3]                    6.2814650   0.2096086490 0.23656075
## Bacteroidetes_[G.5]                    4.6043413   0.1281795260 0.26055304
## Family.Bacteroidetes_[F-1].            0.8532789   0.0158640459 0.37629328
## Bacteroidaceae_[G.1]                   0.7884424  -0.1260724314 0.35831761
## Bacteroidales_[G.2]                   11.9555179  -0.0216722683 0.13642878
## Porphyromonas                        680.5675842  -0.0280024068 0.12533094
## Tannerella                            21.8786158  -0.0078619461 0.09211753
## Alloprevotella                       362.3831427   0.0444840923 0.09935655
## Prevotella                          3803.4341471  -0.0062947429 0.06325686
## Bergeyella                            72.6688914   0.1301276430 0.09727780
## Capnocytophaga                       224.8283089  -0.0712783234 0.10441209
## Gemella                              309.5600715  -0.0477260623 0.08685188
## Abiotrophia                           29.9454115   0.0195690714 0.18798242
## Granulicatella                       306.4982963   0.1561227058 0.07593733
## Lactobacillus                         21.3286482   0.6144854995 0.28284338
## Streptococcus                       3803.1928514   0.0711867088 0.07140724
## Order.Lactobacillales.                 0.2870253  -0.0224616803 0.25699136
## Class.Bacilli.                         0.5705518   0.2925385816 0.20547693
## Clostridiales_[F.1][G-1]               1.0553422   0.2274741183 0.31355919
## Butyrivibrio                           6.9540598  -0.0717130765 0.19734054
## Catonella                             36.7608947  -0.0447454932 0.11197855
## Johnsonella                            0.9256177   0.4969417280 0.26998531
## Lachnoanaerobaculum                   87.8278442  -0.0486844225 0.07811212
## Lachnospiraceae_[G.2]                 27.9765495  -0.4168220706 0.20469849
## Lachnospiraceae_[G.3]                  2.8963095  -0.1380365020 0.21335627
## Lachnospiraceae_[G.7]                  1.4445878  -0.5248863516 0.30383672
## Lachnospiraceae_[G.8]                  1.4242266   0.4120210764 0.32599331
## Oribacterium                         147.1225771   0.0509319856 0.08683987
## Shuttleworthia                         2.8097236   0.2225178131 0.24217102
## Stomatobaculum                       112.7416039  -0.1492593021 0.09773901
## Family.Lachnospiraceae_[XIV].          1.4359143   0.0527852692 0.26955924
## Peptococcus                            3.7061841  -0.0854646829 0.24310115
## Parvimonas                            40.7092362   0.0467148269 0.12044205
## Filifactor                             6.7070439   0.2888220393 0.23509201
## Mogibacterium                         41.0929336  -0.0199438036 0.08336378
## Peptostreptococcaceae_[XI][G.1]       67.5610760  -0.0356318027 0.14878479
## Peptostreptococcaceae_[XI][G.4]        0.4077938   0.4464652778 0.32185167
## Peptostreptococcaceae_[XI][G.5]        1.6379223   0.3314261529 0.33863673
## Peptostreptococcaceae_[XI][G.6]        0.9831369   0.2861657424 0.33411237
## Peptostreptococcaceae_[XI][G.7]        4.6720763   0.4720942238 0.23166369
## Peptostreptococcaceae_[XI][G.9]        8.6093953   0.0015922784 0.13689011
## Peptostreptococcus                    42.9344245  -0.0433724114 0.17410975
## Ruminococcaceae_[G.1]                 24.9520363   0.0658958975 0.17630805
## Ruminococcaceae_[G.2]                 41.5133748   0.0114117621 0.14682909
## Bulleidia                              3.7820436   0.3705520904 0.25843920
## Eggerthia                              1.4571098   0.1623276874 0.34231790
## Solobacterium                         73.9527042  -0.1220874954 0.09782189
## Mycoplasma                             4.4631120   0.2725965251 0.20678434
## Mitsuokella                           26.6824734  -0.3477710043 0.18536228
## Selenomonas                          220.9369826  -0.0583456550 0.08332306
## Family.Selenomonadaceae.               4.9103154  -0.0096746664 0.21605756
## Class.Negativicutes.                   0.7345592   0.0938502353 0.20048229
```

```
## Anaeroglobus                            8.1368294  -0.2772079946 0.19470272
## Dialister                              36.2551202   0.1471253739 0.10245864
## Megasphaera                           405.7597713  -0.0384000709 0.10790066
## Family.Veillonellaceae.                 0.5244498  -0.2066585576 0.23532501
## Veillonella                          5065.4751266   0.0512961164 0.06076920
## Veillonellaceae_[G.1]                   6.0701907  -0.0781825323 0.16843000
## Phylum.Firmicutes.                      0.8927399  -0.0697146320 0.27344332
## Fusobacterium                         923.5000260   0.0068553688 0.08072987
## Leptotrichia                          681.5692371  -0.1325442832 0.08324744
## Gracilibacteria_(GN02)_[G.1]            2.4062692   0.3566455838 0.27058756
## Lautropia                              68.5720452   0.0008552874 0.15478912
## Ottowia                                 2.2016380  -0.7700636342 0.33754852
## Eikenella                               8.7535351   0.2522528340 0.12939377
## Kingella                               99.6224781   0.1201223481 0.11177483
## Neisseria                            1330.9687057   0.0177875651 0.12725400
## Family.Neisseriaceae.                   5.6952181  -0.2907197921 0.29261725
## Campylobacter                         257.2735466  -0.2064415296 0.07095964
## Cardiobacterium                        14.8034460  -0.1100509044 0.11164436
## Aggregatibacter                       275.5999071   0.0483097798 0.14730331
## Haemophilus                          2914.0087049  -0.0840792804 0.09748713
## Family.Pasteurellaceae.                 2.3966745   0.3244309711 0.40192109
## Saccharibacteria_(TM7)_[G.1]           35.9760211   0.3412261081 0.16406590
## Saccharibacteria_(TM7)_[G.3]            7.1315107   0.3165423864 0.22788603
## Saccharibacteria_(TM7)_[G.6]            7.2432549  -0.0929625900 0.29050169
## Family.Saccharibacteria_(TM7)_[F-1].    1.1018253  -0.2380068152 0.36289297
## Saccharibacteria_(TM7)_[G.5]            0.8725887   0.1782078886 0.34267486
## Treponema                              61.7774069   0.3701764102 0.15389742
## Fretibacterium                          8.0757991   0.1806483982 0.18736163
## Kingdom.Bacteria.                       9.3970711  -0.0020300273 0.23816820
## Others                                 33.1988976   0.2224876527 0.15822136
##                                             stat        pval
## Absconditabacteria_(SR1)_[G.1]        0.75254483 0.686415319
## Actinomyces                          12.48392687 0.001946031
## Peptidiphaga                          0.50200750 0.778019455
## Schaalia                              2.10943315 0.348291128
## Family.Actinomycetaceae.              4.41402627 0.110028800
## Rothia                                0.39785295 0.819610151
## Alloscardovia                         0.04996968 0.975324699
## Bifidobacterium                       0.77041264 0.680310262
## Parascardovia                         1.41513674 0.492841148
## Scardovia                             2.56175100 0.277793985
## Corynebacterium                       1.51859228 0.467995714
## Olsenella                             1.30475431 0.520806268
## Atopobium                             1.11967748 0.571301186
## Slackia                               3.64008001 0.162019269
## Cryptobacterium                       4.67949827 0.096351807
## Bacteroidetes_[G.3]                   2.32616825 0.312520840
## Bacteroidetes_[G.5]                   0.69440354 0.706662732
## Family.Bacteroidetes_[F-1].           0.26285669 0.876842105
## Bacteroidaceae_[G.1]                  3.14496438 0.207529416
## Bacteroidales_[G.2]                   0.27500841 0.871530683
## Porphyromonas                         4.12293176 0.127267275
## Tannerella                            0.15339555 0.926169722
## Alloprevotella                        2.00239657 0.367438880
```

```
## Prevotella                            0.52588280 0.768786946
## Bergeyella                            3.63325469 0.162573130
## Capnocytophaga                        1.02353109 0.599436311
## Gemella                               0.92120459 0.630903540
## Abiotrophia                           3.50490332 0.173348431
## Granulicatella                        4.20450890 0.122180667
## Lactobacillus                         5.95044766 0.051036009
## Streptococcus                         1.90949530 0.384909265
## Order.Lactobacillales.               -3.67755152 1.000000000
## Class.Bacilli.                        6.21940619 0.044614199
## Clostridiales_[F.1][G-1]              0.75921401 0.684130217
## Butyrivibrio                          7.55453590 0.022885129
## Catonella                             0.45005905 0.798492643
## Johnsonella                           5.19587219 0.074427030
## Lachnoanaerobaculum                   2.28126328 0.319617075
## Lachnospiraceae_[G.2]                 6.62473918 0.036429748
## Lachnospiraceae_[G.3]                 0.72791806 0.694919665
## Lachnospiraceae_[G.7]                 3.19543953 0.202357415
## Lachnospiraceae_[G.8]                 1.70148987 0.427096654
## Oribacterium                          0.68089697 0.711451177
## Shuttleworthia                        1.28187145 0.526799255
## Stomatobaculum                        2.35843094 0.307519903
## Family.Lachnospiraceae_[XIV].         0.17218044 0.917511449
## Peptococcus                           1.22516778 0.541948722
## Parvimonas                            0.55643174 0.757133360
## Filifactor                            1.61213913 0.446609992
## Mogibacterium                         0.99264605 0.608764963
## Peptostreptococcaceae_[XI][G.1]       3.59405153 0.165791258
## Peptostreptococcaceae_[XI][G.4]       2.40552305 0.300363603
## Peptostreptococcaceae_[XI][G.5]       1.33513736 0.512954218
## Peptostreptococcaceae_[XI][G.6]       3.39674974 0.182980650
## Peptostreptococcaceae_[XI][G.7]       4.67622212 0.096509767
## Peptostreptococcaceae_[XI][G.9]       0.09607590 0.953097614
## Peptostreptococcus                    0.19448695 0.907335064
## Ruminococcaceae_[G.1]                 0.36589829 0.832810505
## Ruminococcaceae_[G.2]                 0.10780246 0.947525689
## Bulleidia                             2.21822414 0.329851717
## Eggerthia                             1.64707052 0.438877363
## Solobacterium                         1.98375275 0.370880127
## Mycoplasma                            3.20180324 0.201714566
## Mitsuokella                           5.01075363 0.081644827
## Selenomonas                           0.75024889 0.687203754
## Family.Selenomonadaceae.              1.46705089 0.480213039
## Class.Negativicutes.                  0.46129969 0.794017447
## Anaeroglobus                          7.77977323 0.020447664
## Dialister                             2.20268369 0.332424722
## Megasphaera                           0.23301498 0.890023429
## Family.Veillonellaceae.               0.92313365 0.630295310
## Veillonella                           3.63407125 0.162506768
## Veillonellaceae_[G.1]                 3.65025443 0.161197136
## Phylum.Firmicutes.                    7.53546230 0.023104424
## Fusobacterium                         1.44011082 0.486725286
## Leptotrichia                          3.35566340 0.186778529
## Gracilibacteria_(GN02)_[G.1]          2.28502094 0.319017133
```

```
## Lautropia                              1.37312012 0.503304432
## Ottowia                                8.67484450 0.013070176
## Eikenella                              6.06774141 0.048128984
## Kingella                               1.71246234 0.424759917
## Neisseria                              3.31143732 0.190954775
## Family.Neisseriaceae.                  1.26595676 0.531007900
## Campylobacter                          9.77134016 0.007554060
## Cardiobacterium                        1.13215332 0.567748545
## Aggregatibacter                        0.19605085 0.906625854
## Haemophilus                            0.98613549 0.610749893
## Family.Pasteurellaceae.                0.62749538 0.730703369
## Saccharibacteria_(TM7)_[G.1]           4.88110547 0.087112688
## Saccharibacteria_(TM7)_[G.3]           6.52019871 0.038384584
## Saccharibacteria_(TM7)_[G.6]           2.60542873 0.271793045
## Family.Saccharibacteria_(TM7)_[F-1].  1.52373869 0.466793015
## Saccharibacteria_(TM7)_[G.5]           0.59770737 0.741667920
## Treponema                              8.45880728 0.014561072
## Fretibacterium                         1.09552788 0.578241348
## Kingdom.Bacteria.                      0.03353396 0.983372806
## Others                                 7.28607772 0.026172688
##                                                      ordering  pval.adj
## Absconditabacteria_(SR1)_[G.1]       Healthyweight>Overweight 0.9080364
## Actinomyces                          Healthyweight>Overweight 0.1887650
## Peptidiphaga                         Healthyweight>Overweight 0.9317023
## Schaalia                             Overweight>Healthyweight 0.7856800
## Family.Actinomycetaceae.             Healthyweight>Overweight 0.5617260
## Rothia                               Overweight>Healthyweight 0.9464546
## Alloscardovia                        Overweight>Healthyweight 0.9936163
## Bifidobacterium                      Healthyweight>Overweight 0.9080364
## Parascardovia                        Healthyweight>Overweight 0.8584628
## Scardovia                            Healthyweight>Overweight 0.7677428
## Corynebacterium                      Healthyweight>Overweight 0.8584628
## Olsenella                            Healthyweight>Overweight 0.8584628
## Atopobium                            Healthyweight>Overweight 0.8763970
## Slackia                              Overweight>Healthyweight 0.6100107
## Cryptobacterium                      Healthyweight>Overweight 0.5200804
## Bacteroidetes_[G.3]                  Overweight>Healthyweight 0.7677428
## Bacteroidetes_[G.5]                  Overweight>Healthyweight 0.9080364
## Family.Bacteroidetes_[F-1].          Overweight>Healthyweight 0.9765050
## Bacteroidaceae_[G.1]                 Healthyweight>Overweight 0.6100107
## Bacteroidales_[G.2]                  Healthyweight>Overweight 0.9765050
## Porphyromonas                        Healthyweight>Overweight 0.5878536
## Tannerella                           Healthyweight>Overweight 0.9765050
## Alloprevotella                       Overweight>Healthyweight 0.7994527
## Prevotella                           Healthyweight>Overweight 0.9317023
## Bergeyella                           Overweight>Healthyweight 0.6100107
## Capnocytophaga                       Healthyweight>Overweight 0.8842200
## Gemella                              Healthyweight>Overweight 0.8869224
## Abiotrophia                          Overweight>Healthyweight 0.6100107
## Granulicatella                       Overweight>Healthyweight 0.5878536
## Lactobacillus                        Overweight>Healthyweight 0.3808071
## Streptococcus                        Overweight>Healthyweight 0.8116565
## Order.Lactobacillales.               Healthyweight>Overweight 1.0000000
## Class.Bacilli.                       Overweight>Healthyweight 0.3808071
```

```
## Clostridiales_[F.1][G-1]             Overweight>Healthyweight 0.9080364
## Butyrivibrio                         Healthyweight>Overweight 0.3173438
## Catonella                            Healthyweight>Overweight 0.9331781
## Johnsonella                          Overweight>Healthyweight 0.5156730
## Lachnoanaerobaculum                  Healthyweight>Overweight 0.7677428
## Lachnospiraceae_[G.2]                Healthyweight>Overweight 0.3723305
## Lachnospiraceae_[G.3]                Healthyweight>Overweight 0.9080364
## Lachnospiraceae_[G.7]                Healthyweight>Overweight 0.6100107
## Lachnospiraceae_[G.8]                Overweight>Healthyweight 0.8584628
## Oribacterium                         Overweight>Healthyweight 0.9080364
## Shuttleworthia                       Overweight>Healthyweight 0.8584628
## Stomatobaculum                       Healthyweight>Overweight 0.7677428
## Family.Lachnospiraceae_[XIV].        Overweight>Healthyweight 0.9765050
## Peptococcus                          Healthyweight>Overweight 0.8617873
## Parvimonas                           Overweight>Healthyweight 0.9296448
## Filifactor                           Overweight>Healthyweight 0.8584628
## Mogibacterium                        Healthyweight>Overweight 0.8842200
## Peptostreptococcaceae_[XI][G.1]      Healthyweight>Overweight 0.6100107
## Peptostreptococcaceae_[XI][G.4]      Overweight>Healthyweight 0.7677428
## Peptostreptococcaceae_[XI][G.5]      Overweight>Healthyweight 0.8584628
## Peptostreptococcaceae_[XI][G.6]      Overweight>Healthyweight 0.6100107
## Peptostreptococcaceae_[XI][G.7]      Overweight>Healthyweight 0.5200804
## Peptostreptococcaceae_[XI][G.9]      Overweight>Healthyweight 0.9835156
## Peptostreptococcus                   Healthyweight>Overweight 0.9765050
## Ruminococcaceae_[G.1]                Overweight>Healthyweight 0.9503838
## Ruminococcaceae_[G.2]                Overweight>Healthyweight 0.9835156
## Bulleidia                            Overweight>Healthyweight 0.7677428
## Eggerthia                            Overweight>Healthyweight 0.8584628
## Solobacterium                        Healthyweight>Overweight 0.7994527
## Mycoplasma                           Overweight>Healthyweight 0.6100107
## Mitsuokella                          Healthyweight>Overweight 0.5200804
## Selenomonas                          Healthyweight>Overweight 0.9080364
## Family.Selenomonadaceae.             Healthyweight>Overweight 0.8584628
## Class.Negativicutes.                 Overweight>Healthyweight 0.9331781
## Anaeroglobus                         Healthyweight>Overweight 0.3173438
## Dialister                            Overweight>Healthyweight 0.7677428
## Megasphaera                          Healthyweight>Overweight 0.9765050
## Family.Veillonellaceae.              Healthyweight>Overweight 0.8869224
## Veillonella                          Overweight>Healthyweight 0.6100107
## Veillonellaceae_[G.1]                Healthyweight>Overweight 0.6100107
## Phylum.Firmicutes.                   Healthyweight>Overweight 0.3173438
## Fusobacterium                        Overweight>Healthyweight 0.8584628
## Leptotrichia                         Healthyweight>Overweight 0.6100107
## Gracilibacteria_(GN02)_[G.1]         Overweight>Healthyweight 0.7677428
## Lautropia                            Overweight>Healthyweight 0.8584628
## Ottowia                              Healthyweight>Overweight 0.3173438
## Eikenella                            Overweight>Healthyweight 0.3808071
## Kingella                             Overweight>Healthyweight 0.8584628
## Neisseria                            Overweight>Healthyweight 0.6100107
## Family.Neisseriaceae.                Healthyweight>Overweight 0.8584628
## Campylobacter                        Healthyweight>Overweight 0.3173438
## Cardiobacterium                      Healthyweight>Overweight 0.8763970
## Aggregatibacter                      Overweight>Healthyweight 0.9765050
## Haemophilus                          Healthyweight>Overweight 0.8842200
```

```
## Family.Pasteurellaceae.               Overweight>Healthyweight 0.9204965
## Saccharibacteria_(TM7)_[G.1]          Overweight>Healthyweight 0.5200804
## Saccharibacteria_(TM7)_[G.3]          Overweight>Healthyweight 0.3723305
## Saccharibacteria_(TM7)_[G.6]          Healthyweight>Overweight 0.7677428
## Family.Saccharibacteria_(TM7)_[F-1]. Healthyweight>Overweight 0.8584628
## Saccharibacteria_(TM7)_[G.5]          Overweight>Healthyweight 0.9223306
## Treponema                             Overweight>Healthyweight 0.3173438
## Fretibacterium                        Overweight>Healthyweight 0.8763970
## Kingdom.Bacteria.                     Healthyweight>Overweight 0.9936163
## Others                                Overweight>Healthyweight 0.3173438
##                                                                   Feature
## Absconditabacteria_(SR1)_[G.1]           Absconditabacteria_(SR1)_[G.1]
## Actinomyces                                                   Actinomyces
## Peptidiphaga                                                 Peptidiphaga
## Schaalia                                                         Schaalia
## Family.Actinomycetaceae.                       Family.Actinomycetaceae.
## Rothia                                                             Rothia
## Alloscardovia                                               Alloscardovia
## Bifidobacterium                                           Bifidobacterium
## Parascardovia                                               Parascardovia
## Scardovia                                                       Scardovia
## Corynebacterium                                           Corynebacterium
## Olsenella                                                       Olsenella
## Atopobium                                                       Atopobium
## Slackia                                                           Slackia
## Cryptobacterium                                           Cryptobacterium
## Bacteroidetes_[G.3]                                   Bacteroidetes_[G.3]
## Bacteroidetes_[G.5]                                   Bacteroidetes_[G.5]
## Family.Bacteroidetes_[F-1].                   Family.Bacteroidetes_[F-1].
## Bacteroidaceae_[G.1]                                 Bacteroidaceae_[G.1]
## Bacteroidales_[G.2]                                   Bacteroidales_[G.2]
## Porphyromonas                                               Porphyromonas
## Tannerella                                                     Tannerella
## Alloprevotella                                             Alloprevotella
## Prevotella                                                     Prevotella
## Bergeyella                                                     Bergeyella
## Capnocytophaga                                             Capnocytophaga
## Gemella                                                           Gemella
## Abiotrophia                                                   Abiotrophia
## Granulicatella                                             Granulicatella
## Lactobacillus                                               Lactobacillus
## Streptococcus                                               Streptococcus
## Order.Lactobacillales.                           Order.Lactobacillales.
## Class.Bacilli.                                             Class.Bacilli.
## Clostridiales_[F.1][G-1]                       Clostridiales_[F.1][G-1]
## Butyrivibrio                                                 Butyrivibrio
## Catonella                                                       Catonella
## Johnsonella                                                   Johnsonella
## Lachnoanaerobaculum                                   Lachnoanaerobaculum
## Lachnospiraceae_[G.2]                               Lachnospiraceae_[G.2]
## Lachnospiraceae_[G.3]                               Lachnospiraceae_[G.3]
## Lachnospiraceae_[G.7]                               Lachnospiraceae_[G.7]
## Lachnospiraceae_[G.8]                               Lachnospiraceae_[G.8]
## Oribacterium                                                 Oribacterium
```

```
## Shuttleworthia                                                   Shuttleworthia
## Stomatobaculum                                                   Stomatobaculum
## Family.Lachnospiraceae_[XIV].                     Family.Lachnospiraceae_[XIV].
## Peptococcus                                                         Peptococcus
## Parvimonas                                                           Parvimonas
## Filifactor                                                           Filifactor
## Mogibacterium                                                     Mogibacterium
## Peptostreptococcaceae_[XI][G.1]                 Peptostreptococcaceae_[XI][G.1]
## Peptostreptococcaceae_[XI][G.4]                 Peptostreptococcaceae_[XI][G.4]
## Peptostreptococcaceae_[XI][G.5]                 Peptostreptococcaceae_[XI][G.5]
## Peptostreptococcaceae_[XI][G.6]                 Peptostreptococcaceae_[XI][G.6]
## Peptostreptococcaceae_[XI][G.7]                 Peptostreptococcaceae_[XI][G.7]
## Peptostreptococcaceae_[XI][G.9]                 Peptostreptococcaceae_[XI][G.9]
## Peptostreptococcus                                           Peptostreptococcus
## Ruminococcaceae_[G.1]                                     Ruminococcaceae_[G.1]
## Ruminococcaceae_[G.2]                                     Ruminococcaceae_[G.2]
## Bulleidia                                                             Bulleidia
## Eggerthia                                                             Eggerthia
## Solobacterium                                                     Solobacterium
## Mycoplasma                                                           Mycoplasma
## Mitsuokella                                                         Mitsuokella
## Selenomonas                                                         Selenomonas
## Family.Selenomonadaceae.                               Family.Selenomonadaceae.
## Class.Negativicutes.                                       Class.Negativicutes.
## Anaeroglobus                                                       Anaeroglobus
## Dialister                                                             Dialister
## Megasphaera                                                         Megasphaera
## Family.Veillonellaceae.                                 Family.Veillonellaceae.
## Veillonella                                                         Veillonella
## Veillonellaceae_[G.1]                                     Veillonellaceae_[G.1]
## Phylum.Firmicutes.                                           Phylum.Firmicutes.
## Fusobacterium                                                     Fusobacterium
## Leptotrichia                                                       Leptotrichia
## Gracilibacteria_(GN02)_[G.1]                       Gracilibacteria_(GN02)_[G.1]
## Lautropia                                                             Lautropia
## Ottowia                                                                 Ottowia
## Eikenella                                                             Eikenella
## Kingella                                                               Kingella
## Neisseria                                                             Neisseria
## Family.Neisseriaceae.                                     Family.Neisseriaceae.
## Campylobacter                                                     Campylobacter
## Cardiobacterium                                                 Cardiobacterium
## Aggregatibacter                                                 Aggregatibacter
## Haemophilus                                                         Haemophilus
## Family.Pasteurellaceae.                                 Family.Pasteurellaceae.
## Saccharibacteria_(TM7)_[G.1]                       Saccharibacteria_(TM7)_[G.1]
## Saccharibacteria_(TM7)_[G.3]                       Saccharibacteria_(TM7)_[G.3]
## Saccharibacteria_(TM7)_[G.6]                       Saccharibacteria_(TM7)_[G.6]
## Family.Saccharibacteria_(TM7)_[F-1]. Family.Saccharibacteria_(TM7)_[F-1].
## Saccharibacteria_(TM7)_[G.5]                       Saccharibacteria_(TM7)_[G.5]
## Treponema                                                             Treponema
## Fretibacterium                                                   Fretibacterium
## Kingdom.Bacteria.                                             Kingdom.Bacteria.
## Others                                                                   Others
```

```
##                                                            Method
## Absconditabacteria_(SR1)_[G.1]       DESeq2 man. geoMeans (ds2)
## Actinomyces                          DESeq2 man. geoMeans (ds2)
## Peptidiphaga                         DESeq2 man. geoMeans (ds2)
## Schaalia                             DESeq2 man. geoMeans (ds2)
## Family.Actinomycetaceae.             DESeq2 man. geoMeans (ds2)
## Rothia                               DESeq2 man. geoMeans (ds2)
## Alloscardovia                        DESeq2 man. geoMeans (ds2)
## Bifidobacterium                      DESeq2 man. geoMeans (ds2)
## Parascardovia                        DESeq2 man. geoMeans (ds2)
## Scardovia                            DESeq2 man. geoMeans (ds2)
## Corynebacterium                      DESeq2 man. geoMeans (ds2)
## Olsenella                            DESeq2 man. geoMeans (ds2)
## Atopobium                            DESeq2 man. geoMeans (ds2)
## Slackia                              DESeq2 man. geoMeans (ds2)
## Cryptobacterium                      DESeq2 man. geoMeans (ds2)
## Bacteroidetes_[G.3]                  DESeq2 man. geoMeans (ds2)
## Bacteroidetes_[G.5]                  DESeq2 man. geoMeans (ds2)
## Family.Bacteroidetes_[F-1].          DESeq2 man. geoMeans (ds2)
## Bacteroidaceae_[G.1]                 DESeq2 man. geoMeans (ds2)
## Bacteroidales_[G.2]                  DESeq2 man. geoMeans (ds2)
## Porphyromonas                        DESeq2 man. geoMeans (ds2)
## Tannerella                           DESeq2 man. geoMeans (ds2)
## Alloprevotella                       DESeq2 man. geoMeans (ds2)
## Prevotella                           DESeq2 man. geoMeans (ds2)
## Bergeyella                           DESeq2 man. geoMeans (ds2)
## Capnocytophaga                       DESeq2 man. geoMeans (ds2)
## Gemella                              DESeq2 man. geoMeans (ds2)
## Abiotrophia                          DESeq2 man. geoMeans (ds2)
## Granulicatella                       DESeq2 man. geoMeans (ds2)
## Lactobacillus                        DESeq2 man. geoMeans (ds2)
## Streptococcus                        DESeq2 man. geoMeans (ds2)
## Order.Lactobacillales.               DESeq2 man. geoMeans (ds2)
## Class.Bacilli.                       DESeq2 man. geoMeans (ds2)
## Clostridiales_[F.1][G-1]             DESeq2 man. geoMeans (ds2)
## Butyrivibrio                         DESeq2 man. geoMeans (ds2)
## Catonella                            DESeq2 man. geoMeans (ds2)
## Johnsonella                          DESeq2 man. geoMeans (ds2)
## Lachnoanaerobaculum                  DESeq2 man. geoMeans (ds2)
## Lachnospiraceae_[G.2]                DESeq2 man. geoMeans (ds2)
## Lachnospiraceae_[G.3]                DESeq2 man. geoMeans (ds2)
## Lachnospiraceae_[G.7]                DESeq2 man. geoMeans (ds2)
## Lachnospiraceae_[G.8]                DESeq2 man. geoMeans (ds2)
## Oribacterium                         DESeq2 man. geoMeans (ds2)
## Shuttleworthia                       DESeq2 man. geoMeans (ds2)
## Stomatobaculum                       DESeq2 man. geoMeans (ds2)
## Family.Lachnospiraceae_[XIV].        DESeq2 man. geoMeans (ds2)
## Peptococcus                          DESeq2 man. geoMeans (ds2)
## Parvimonas                           DESeq2 man. geoMeans (ds2)
## Filifactor                           DESeq2 man. geoMeans (ds2)
## Mogibacterium                        DESeq2 man. geoMeans (ds2)
## Peptostreptococcaceae_[XI][G.1]      DESeq2 man. geoMeans (ds2)
## Peptostreptococcaceae_[XI][G.4]      DESeq2 man. geoMeans (ds2)
## Peptostreptococcaceae_[XI][G.5]      DESeq2 man. geoMeans (ds2)
```

```
## Peptostreptococcaceae_[XI][G.6]      DESeq2 man. geoMeans (ds2)
## Peptostreptococcaceae_[XI][G.7]      DESeq2 man. geoMeans (ds2)
## Peptostreptococcaceae_[XI][G.9]      DESeq2 man. geoMeans (ds2)
## Peptostreptococcus                   DESeq2 man. geoMeans (ds2)
## Ruminococcaceae_[G.1]                DESeq2 man. geoMeans (ds2)
## Ruminococcaceae_[G.2]                DESeq2 man. geoMeans (ds2)
## Bulleidia                            DESeq2 man. geoMeans (ds2)
## Eggerthia                            DESeq2 man. geoMeans (ds2)
## Solobacterium                        DESeq2 man. geoMeans (ds2)
## Mycoplasma                           DESeq2 man. geoMeans (ds2)
## Mitsuokella                          DESeq2 man. geoMeans (ds2)
## Selenomonas                          DESeq2 man. geoMeans (ds2)
## Family.Selenomonadaceae.             DESeq2 man. geoMeans (ds2)
## Class.Negativicutes.                 DESeq2 man. geoMeans (ds2)
## Anaeroglobus                         DESeq2 man. geoMeans (ds2)
## Dialister                            DESeq2 man. geoMeans (ds2)
## Megasphaera                          DESeq2 man. geoMeans (ds2)
## Family.Veillonellaceae.              DESeq2 man. geoMeans (ds2)
## Veillonella                          DESeq2 man. geoMeans (ds2)
## Veillonellaceae_[G.1]                DESeq2 man. geoMeans (ds2)
## Phylum.Firmicutes.                   DESeq2 man. geoMeans (ds2)
## Fusobacterium                        DESeq2 man. geoMeans (ds2)
## Leptotrichia                         DESeq2 man. geoMeans (ds2)
## Gracilibacteria_(GN02)_[G.1]         DESeq2 man. geoMeans (ds2)
## Lautropia                            DESeq2 man. geoMeans (ds2)
## Ottowia                              DESeq2 man. geoMeans (ds2)
## Eikenella                            DESeq2 man. geoMeans (ds2)
## Kingella                             DESeq2 man. geoMeans (ds2)
## Neisseria                            DESeq2 man. geoMeans (ds2)
## Family.Neisseriaceae.                DESeq2 man. geoMeans (ds2)
## Campylobacter                        DESeq2 man. geoMeans (ds2)
## Cardiobacterium                      DESeq2 man. geoMeans (ds2)
## Aggregatibacter                      DESeq2 man. geoMeans (ds2)
## Haemophilus                          DESeq2 man. geoMeans (ds2)
## Family.Pasteurellaceae.              DESeq2 man. geoMeans (ds2)
## Saccharibacteria_(TM7)_[G.1]         DESeq2 man. geoMeans (ds2)
## Saccharibacteria_(TM7)_[G.3]         DESeq2 man. geoMeans (ds2)
## Saccharibacteria_(TM7)_[G.6]         DESeq2 man. geoMeans (ds2)
## Family.Saccharibacteria_(TM7)_[F-1]. DESeq2 man. geoMeans (ds2)
## Saccharibacteria_(TM7)_[G.5]         DESeq2 man. geoMeans (ds2)
## Treponema                            DESeq2 man. geoMeans (ds2)
## Fretibacterium                       DESeq2 man. geoMeans (ds2)
## Kingdom.Bacteria.                    DESeq2 man. geoMeans (ds2)
## Others                               DESeq2 man. geoMeans (ds2)
```

```
write.table(final, file="BMI_cat_ds2.txt", sep="\t", dec=",", row.names=F)
write.table(final[final$pval<0.05,], file="BMI_cat_ds2_sig.txt", sep="\t", dec=",", row.names=F)
final <- DA.kru(Microbio2, predictor = Phe2$BMI_cat)
final
```

```
##                                             pval  pval.adj
## Absconditabacteria_(SR1)_[G.1]       0.321213769 0.8281972
## Actinomyces                          0.213257827 0.8281972
## Peptidiphaga                         0.691505804 0.9216569
```

```
## Schaalia                               0.539934831 0.9091490
## Family.Actinomycetaceae.               0.033905529 0.8281972
## Rothia                                 0.485937371 0.8895242
## Alloscardovia                          0.890568231 0.9700502
## Bifidobacterium                        0.580407600 0.9216569
## Parascardovia                          0.244526608 0.8281972
## Scardovia                              0.005879027 0.5702656
## Corynebacterium                        0.861416572 0.9700502
## Olsenella                              0.479309476 0.8895242
## Atopobium                              0.612705086 0.9216569
## Slackia                                0.283183245 0.8281972
## Cryptobacterium                        0.136069346 0.8281972
## Bacteroidetes_[G.3]                    0.212108305 0.8281972
## Bacteroidetes_[G.5]                    0.457750808 0.8895242
## Family.Bacteroidetes_[F-1].            0.209289188 0.8281972
## Bacteroidaceae_[G.1]                   0.097340508 0.8281972
## Bacteroidales_[G.2]                    0.697861519 0.9216569
## Porphyromonas                          0.277088050 0.8281972
## Tannerella                             0.991690097 0.9965199
## Alloprevotella                         0.105360981 0.8281972
## Prevotella                             0.498068879 0.8895242
## Bergeyella                             0.408588230 0.8895242
## Capnocytophaga                         0.670967013 0.9216569
## Gemella                                0.940048621 0.9700502
## Abiotrophia                            0.115933641 0.8281972
## Granulicatella                         0.427010696 0.8895242
## Lactobacillus                          0.159329008 0.8281972
## Streptococcus                          0.492769259 0.8895242
## Order.Lactobacillales.                 0.807660160 0.9646106
## Class.Bacilli.                         0.246143937 0.8281972
## Clostridiales_[F.1][G-1]               0.760129438 0.9216569
## Butyrivibrio                           0.020245730 0.8281972
## Catonella                              0.754624447 0.9216569
## Johnsonella                            0.418930988 0.8895242
## Lachnoanaerobaculum                    0.367188392 0.8687140
## Lachnospiraceae_[G.2]                  0.110192348 0.8281972
## Lachnospiraceae_[G.3]                  0.718402089 0.9216569
## Lachnospiraceae_[G.7]                  0.679663078 0.9216569
## Lachnospiraceae_[G.8]                  0.726836627 0.9216569
## Oribacterium                           0.670926449 0.9216569
## Shuttleworthia                         0.961007138 0.9812389
## Stomatobaculum                         0.745209120 0.9216569
## Family.Lachnospiraceae_[XIV].          0.912090723 0.9700502
## Peptococcus                            0.290888515 0.8281972
## Parvimonas                             0.879127767 0.9700502
## Filifactor                             0.242174354 0.8281972
## Mogibacterium                          0.436608182 0.8895242
## Peptostreptococcaceae_[XI][G.1]        0.107548941 0.8281972
## Peptostreptococcaceae_[XI][G.4]        0.315787653 0.8281972
## Peptostreptococcaceae_[XI][G.5]        0.854035953 0.9700502
## Peptostreptococcaceae_[XI][G.6]        0.119542766 0.8281972
## Peptostreptococcaceae_[XI][G.7]        0.222340350 0.8281972
## Peptostreptococcaceae_[XI][G.9]        0.996519948 0.9965199
## Peptostreptococcus                     0.326508688 0.8281972
```

```
## Ruminococcaceae_[G.1]                0.308653385 0.8281972
## Ruminococcaceae_[G.2]                0.498805265 0.8895242
## Bulleidia                            0.200863622 0.8281972
## Eggerthia                            0.652677149 0.9216569
## Solobacterium                        0.622130593 0.9216569
## Mycoplasma                           0.332986488 0.8281972
## Mitsuokella                          0.267408978 0.8281972
## Selenomonas                          0.900623609 0.9700502
## Family.Selenomonadaceae.             0.269516040 0.8281972
## Class.Negativicutes.                 0.522353919 0.9047916
## Anaeroglobus                         0.680060409 0.9216569
## Dialister                            0.114410562 0.8281972
## Megasphaera                          0.926295477 0.9700502
## Family.Veillonellaceae.              0.172666246 0.8281972
## Veillonella                          0.257308847 0.8281972
## Veillonellaceae_[G.1]                0.573156464 0.9216569
## Phylum.Firmicutes.                   0.620484034 0.9216569
## Fusobacterium                        0.243148967 0.8281972
## Leptotrichia                         0.353687412 0.8576920
## Gracilibacteria_(GN02)_[G.1]         0.887541835 0.9700502
## Lautropia                            0.305582674 0.8281972
## Ottowia                              0.401350183 0.8895242
## Eikenella                            0.260939229 0.8281972
## Kingella                             0.825388417 0.9646106
## Neisseria                            0.132950254 0.8281972
## Family.Neisseriaceae.                0.816716582 0.9646106
## Campylobacter                        0.061438205 0.8281972
## Cardiobacterium                      0.910764310 0.9700502
## Aggregatibacter                      0.680930052 0.9216569
## Haemophilus                          0.543614882 0.9091490
## Family.Pasteurellaceae.              0.266035344 0.8281972
## Saccharibacteria_(TM7)_[G.1]         0.453577226 0.8895242
## Saccharibacteria_(TM7)_[G.3]         0.403732985 0.8895242
## Saccharibacteria_(TM7)_[G.6]         0.504369405 0.8895242
## Family.Saccharibacteria_(TM7)_[F-1]. 0.556789148 0.9153991
## Saccharibacteria_(TM7)_[G.5]         0.615578846 0.9216569
## Treponema                            0.256826505 0.8281972
## Fretibacterium                       0.937048128 0.9700502
## Kingdom.Bacteria.                    0.715193334 0.9216569
## Others                               0.750027883 0.9216569
##                                                                    Feature
## Absconditabacteria_(SR1)_[G.1]            Absconditabacteria_(SR1)_[G.1]
## Actinomyces                                                    Actinomyces
## Peptidiphaga                                                  Peptidiphaga
## Schaalia                                                          Schaalia
## Family.Actinomycetaceae.                          Family.Actinomycetaceae.
## Rothia                                                              Rothia
## Alloscardovia                                                Alloscardovia
## Bifidobacterium                                            Bifidobacterium
## Parascardovia                                                Parascardovia
## Scardovia                                                        Scardovia
## Corynebacterium                                            Corynebacterium
## Olsenella                                                        Olsenella
## Atopobium                                                        Atopobium
```

```
## Slackia                                                            Slackia
## Cryptobacterium                                            Cryptobacterium
## Bacteroidetes_[G.3]                                    Bacteroidetes_[G.3]
## Bacteroidetes_[G.5]                                    Bacteroidetes_[G.5]
## Family.Bacteroidetes_[F-1].                    Family.Bacteroidetes_[F-1].
## Bacteroidaceae_[G.1]                                  Bacteroidaceae_[G.1]
## Bacteroidales_[G.2]                                    Bacteroidales_[G.2]
## Porphyromonas                                                Porphyromonas
## Tannerella                                                      Tannerella
## Alloprevotella                                              Alloprevotella
## Prevotella                                                      Prevotella
## Bergeyella                                                      Bergeyella
## Capnocytophaga                                              Capnocytophaga
## Gemella                                                            Gemella
## Abiotrophia                                                    Abiotrophia
## Granulicatella                                              Granulicatella
## Lactobacillus                                                Lactobacillus
## Streptococcus                                                Streptococcus
## Order.Lactobacillales.                              Order.Lactobacillales.
## Class.Bacilli.                                              Class.Bacilli.
## Clostridiales_[F.1][G-1]                          Clostridiales_[F.1][G-1]
## Butyrivibrio                                                  Butyrivibrio
## Catonella                                                        Catonella
## Johnsonella                                                    Johnsonella
## Lachnoanaerobaculum                                    Lachnoanaerobaculum
## Lachnospiraceae_[G.2]                                Lachnospiraceae_[G.2]
## Lachnospiraceae_[G.3]                                Lachnospiraceae_[G.3]
## Lachnospiraceae_[G.7]                                Lachnospiraceae_[G.7]
## Lachnospiraceae_[G.8]                                Lachnospiraceae_[G.8]
## Oribacterium                                                  Oribacterium
## Shuttleworthia                                              Shuttleworthia
## Stomatobaculum                                              Stomatobaculum
## Family.Lachnospiraceae_[XIV].                Family.Lachnospiraceae_[XIV].
## Peptococcus                                                    Peptococcus
## Parvimonas                                                      Parvimonas
## Filifactor                                                      Filifactor
## Mogibacterium                                                Mogibacterium
## Peptostreptococcaceae_[XI][G.1]            Peptostreptococcaceae_[XI][G.1]
## Peptostreptococcaceae_[XI][G.4]            Peptostreptococcaceae_[XI][G.4]
## Peptostreptococcaceae_[XI][G.5]            Peptostreptococcaceae_[XI][G.5]
## Peptostreptococcaceae_[XI][G.6]            Peptostreptococcaceae_[XI][G.6]
## Peptostreptococcaceae_[XI][G.7]            Peptostreptococcaceae_[XI][G.7]
## Peptostreptococcaceae_[XI][G.9]            Peptostreptococcaceae_[XI][G.9]
## Peptostreptococcus                                      Peptostreptococcus
## Ruminococcaceae_[G.1]                                Ruminococcaceae_[G.1]
## Ruminococcaceae_[G.2]                                Ruminococcaceae_[G.2]
## Bulleidia                                                        Bulleidia
## Eggerthia                                                        Eggerthia
## Solobacterium                                                Solobacterium
## Mycoplasma                                                      Mycoplasma
## Mitsuokella                                                    Mitsuokella
## Selenomonas                                                    Selenomonas
## Family.Selenomonadaceae.                          Family.Selenomonadaceae.
## Class.Negativicutes.                                  Class.Negativicutes.
```

```
## Anaeroglobus                                                 Anaeroglobus
## Dialister                                                       Dialister
## Megasphaera                                                   Megasphaera
## Family.Veillonellaceae.                           Family.Veillonellaceae.
## Veillonella                                                   Veillonella
## Veillonellaceae_[G.1]                               Veillonellaceae_[G.1]
## Phylum.Firmicutes.                                     Phylum.Firmicutes.
## Fusobacterium                                               Fusobacterium
## Leptotrichia                                                 Leptotrichia
## Gracilibacteria_(GN02)_[G.1]                 Gracilibacteria_(GN02)_[G.1]
## Lautropia                                                       Lautropia
## Ottowia                                                           Ottowia
## Eikenella                                                       Eikenella
## Kingella                                                         Kingella
## Neisseria                                                       Neisseria
## Family.Neisseriaceae.                               Family.Neisseriaceae.
## Campylobacter                                               Campylobacter
## Cardiobacterium                                           Cardiobacterium
## Aggregatibacter                                           Aggregatibacter
## Haemophilus                                                   Haemophilus
## Family.Pasteurellaceae.                           Family.Pasteurellaceae.
## Saccharibacteria_(TM7)_[G.1]                 Saccharibacteria_(TM7)_[G.1]
## Saccharibacteria_(TM7)_[G.3]                 Saccharibacteria_(TM7)_[G.3]
## Saccharibacteria_(TM7)_[G.6]                 Saccharibacteria_(TM7)_[G.6]
## Family.Saccharibacteria_(TM7)_[F-1]. Family.Saccharibacteria_(TM7)_[F-1].
## Saccharibacteria_(TM7)_[G.5]                 Saccharibacteria_(TM7)_[G.5]
## Treponema                                                       Treponema
## Fretibacterium                                             Fretibacterium
## Kingdom.Bacteria.                                       Kingdom.Bacteria.
## Others                                                             Others
##                                                 Method
## Absconditabacteria_(SR1)_[G.1]       Kruskal-Wallis (kru)
## Actinomyces                          Kruskal-Wallis (kru)
## Peptidiphaga                         Kruskal-Wallis (kru)
## Schaalia                             Kruskal-Wallis (kru)
## Family.Actinomycetaceae.             Kruskal-Wallis (kru)
## Rothia                               Kruskal-Wallis (kru)
## Alloscardovia                        Kruskal-Wallis (kru)
## Bifidobacterium                      Kruskal-Wallis (kru)
## Parascardovia                        Kruskal-Wallis (kru)
## Scardovia                            Kruskal-Wallis (kru)
## Corynebacterium                      Kruskal-Wallis (kru)
## Olsenella                            Kruskal-Wallis (kru)
## Atopobium                            Kruskal-Wallis (kru)
## Slackia                              Kruskal-Wallis (kru)
## Cryptobacterium                      Kruskal-Wallis (kru)
## Bacteroidetes_[G.3]                  Kruskal-Wallis (kru)
## Bacteroidetes_[G.5]                  Kruskal-Wallis (kru)
## Family.Bacteroidetes_[F-1].          Kruskal-Wallis (kru)
## Bacteroidaceae_[G.1]                 Kruskal-Wallis (kru)
## Bacteroidales_[G.2]                  Kruskal-Wallis (kru)
## Porphyromonas                        Kruskal-Wallis (kru)
## Tannerella                           Kruskal-Wallis (kru)
## Alloprevotella                       Kruskal-Wallis (kru)
```

```
## Prevotella                              Kruskal-Wallis (kru)
## Bergeyella                              Kruskal-Wallis (kru)
## Capnocytophaga                          Kruskal-Wallis (kru)
## Gemella                                 Kruskal-Wallis (kru)
## Abiotrophia                             Kruskal-Wallis (kru)
## Granulicatella                          Kruskal-Wallis (kru)
## Lactobacillus                           Kruskal-Wallis (kru)
## Streptococcus                           Kruskal-Wallis (kru)
## Order.Lactobacillales.                  Kruskal-Wallis (kru)
## Class.Bacilli.                          Kruskal-Wallis (kru)
## Clostridiales_[F.1][G-1]                Kruskal-Wallis (kru)
## Butyrivibrio                            Kruskal-Wallis (kru)
## Catonella                               Kruskal-Wallis (kru)
## Johnsonella                             Kruskal-Wallis (kru)
## Lachnoanaerobaculum                     Kruskal-Wallis (kru)
## Lachnospiraceae_[G.2]                   Kruskal-Wallis (kru)
## Lachnospiraceae_[G.3]                   Kruskal-Wallis (kru)
## Lachnospiraceae_[G.7]                   Kruskal-Wallis (kru)
## Lachnospiraceae_[G.8]                   Kruskal-Wallis (kru)
## Oribacterium                            Kruskal-Wallis (kru)
## Shuttleworthia                          Kruskal-Wallis (kru)
## Stomatobaculum                          Kruskal-Wallis (kru)
## Family.Lachnospiraceae_[XIV].           Kruskal-Wallis (kru)
## Peptococcus                             Kruskal-Wallis (kru)
## Parvimonas                              Kruskal-Wallis (kru)
## Filifactor                              Kruskal-Wallis (kru)
## Mogibacterium                           Kruskal-Wallis (kru)
## Peptostreptococcaceae_[XI][G.1]         Kruskal-Wallis (kru)
## Peptostreptococcaceae_[XI][G.4]         Kruskal-Wallis (kru)
## Peptostreptococcaceae_[XI][G.5]         Kruskal-Wallis (kru)
## Peptostreptococcaceae_[XI][G.6]         Kruskal-Wallis (kru)
## Peptostreptococcaceae_[XI][G.7]         Kruskal-Wallis (kru)
## Peptostreptococcaceae_[XI][G.9]         Kruskal-Wallis (kru)
## Peptostreptococcus                      Kruskal-Wallis (kru)
## Ruminococcaceae_[G.1]                   Kruskal-Wallis (kru)
## Ruminococcaceae_[G.2]                   Kruskal-Wallis (kru)
## Bulleidia                               Kruskal-Wallis (kru)
## Eggerthia                               Kruskal-Wallis (kru)
## Solobacterium                           Kruskal-Wallis (kru)
## Mycoplasma                              Kruskal-Wallis (kru)
## Mitsuokella                             Kruskal-Wallis (kru)
## Selenomonas                             Kruskal-Wallis (kru)
## Family.Selenomonadaceae.                Kruskal-Wallis (kru)
## Class.Negativicutes.                    Kruskal-Wallis (kru)
## Anaeroglobus                            Kruskal-Wallis (kru)
## Dialister                               Kruskal-Wallis (kru)
## Megasphaera                             Kruskal-Wallis (kru)
## Family.Veillonellaceae.                 Kruskal-Wallis (kru)
## Veillonella                             Kruskal-Wallis (kru)
## Veillonellaceae_[G.1]                   Kruskal-Wallis (kru)
## Phylum.Firmicutes.                      Kruskal-Wallis (kru)
## Fusobacterium                           Kruskal-Wallis (kru)
## Leptotrichia                            Kruskal-Wallis (kru)
## Gracilibacteria_(GN02)_[G.1]            Kruskal-Wallis (kru)
```

```
## Lautropia                              Kruskal-Wallis (kru)
## Ottowia                                Kruskal-Wallis (kru)
## Eikenella                              Kruskal-Wallis (kru)
## Kingella                               Kruskal-Wallis (kru)
## Neisseria                              Kruskal-Wallis (kru)
## Family.Neisseriaceae.                  Kruskal-Wallis (kru)
## Campylobacter                          Kruskal-Wallis (kru)
## Cardiobacterium                        Kruskal-Wallis (kru)
## Aggregatibacter                        Kruskal-Wallis (kru)
## Haemophilus                            Kruskal-Wallis (kru)
## Family.Pasteurellaceae.                Kruskal-Wallis (kru)
## Saccharibacteria_(TM7)_[G.1]           Kruskal-Wallis (kru)
## Saccharibacteria_(TM7)_[G.3]           Kruskal-Wallis (kru)
## Saccharibacteria_(TM7)_[G.6]           Kruskal-Wallis (kru)
## Family.Saccharibacteria_(TM7)_[F-1]. Kruskal-Wallis (kru)
## Saccharibacteria_(TM7)_[G.5]           Kruskal-Wallis (kru)
## Treponema                              Kruskal-Wallis (kru)
## Fretibacterium                         Kruskal-Wallis (kru)
## Kingdom.Bacteria.                      Kruskal-Wallis (kru)
## Others                                 Kruskal-Wallis (kru)
```

```
write.table(final, file="BMI_cat_kru.txt", sep="\t", dec=",", row.names=F)
write.table(final[final$pval<0.05,], file="BMI_cat_kru_sig.txt", sep="\t", dec=",", row.names=F)
##################Waist_cat
table(Phe$Waist_cat, useNA="always")
```

```
##
##    Low Medium   High   <NA>
##    197    220    329      0
```

```
final <- DA.ds2(Microbio, predictor = Phe$Waist_cat, out.all=TRUE) #It is LRT so don't look at ordering
final
```

```
##                                          baseMean log2FoldChange      lfcSE
## Absconditabacteria_(SR1)_[G.1]         63.1989360    0.104769950 0.32807899
## Actinomyces                           323.9127377   -0.081989687 0.11108270
## Peptidiphaga                           22.0460481   -0.068241322 0.15850299
## Schaalia                              704.3072222   -0.052779955 0.09145073
## Family.Actinomycetaceae.                5.4421107   -0.220307751 0.22732637
## Rothia                               1939.2520922    0.110441548 0.10718824
## Alloscardovia                          12.4599091    0.173666992 0.28188477
## Bifidobacterium                        10.2458170   -0.227974802 0.30354329
## Parascardovia                           2.9486113    0.463622771 0.42673328
## Scardovia                              12.5460084    0.322980867 0.26710444
## Corynebacterium                        93.7260406   -0.150707742 0.11720482
## Olsenella                               1.5669010    0.495912926 0.25451097
## Atopobium                             120.4914295   -0.079417473 0.10752671
## Slackia                                 1.3927613   -0.142218179 0.24594290
## Cryptobacterium                         4.3017007   -0.646097537 0.26558264
## Bacteroidetes_[G.3]                     6.5912121   -0.167210913 0.28185695
## Bacteroidetes_[G.5]                     4.6746313   -0.026490672 0.30759526
## Family.Bacteroidetes_[F-1].             0.8680201   -0.065746951 0.44008946
## Bacteroidaceae_[G.1]                    0.7921891    0.221361735 0.41966881
```

```
## Bacteroidales_[G.2]                        12.1604713   -0.252048668 0.16155121
## Porphyromonas                             680.1285750    0.009523129 0.14751063
## Tannerella                                 22.0547383   -0.109969647 0.10851015
## Alloprevotella                            361.8645217    0.016139572 0.11691736
## Prevotella                               3811.4256813   -0.058981544 0.07434358
## Bergeyella                                 72.5563323    0.193919129 0.11459586
## Capnocytophaga                            225.1152973   -0.015902531 0.12268511
## Gemella                                   309.0561286    0.066638755 0.10244199
## Abiotrophia                                29.9768046   -0.046866055 0.22102202
## Granulicatella                            306.2864969    0.052107544 0.08919195
## Lactobacillus                              26.9558702    0.455509604 0.34374512
## Streptococcus                            3803.9265541    0.095929055 0.08374331
## Order.Lactobacillales.                      0.2860988    0.152534047 0.30250728
## Class.Bacilli.                              0.5750915   -0.090720703 0.24253193
## Clostridiales_[F.1][G-1]                    1.0542291    0.200795940 0.36545735
## Butyrivibrio                                6.9441711   -0.372848550 0.23272622
## Catonella                                  36.8211521   -0.070895728 0.13180725
## Johnsonella                                 0.9388209   -0.194689638 0.31804443
## Lachnoanaerobaculum                        87.7811267   -0.072134728 0.09213268
## Lachnospiraceae_[G.2]                      27.9872677   -0.413192224 0.24109813
## Lachnospiraceae_[G.3]                       2.8891780    0.081639582 0.25081297
## Lachnospiraceae_[G.7]                       1.4688223   -1.371936255 0.35131828
## Lachnospiraceae_[G.8]                       1.4317578    0.091903485 0.38401516
## Oribacterium                              147.0483774   -0.062244633 0.10205949
## Shuttleworthia                              2.8039987   -0.041392315 0.28423042
## Stomatobaculum                            112.6769059   -0.180875214 0.11474418
## Family.Lachnospiraceae_[XIV].               1.6039609   -0.655381107 0.32297139
## Peptococcus                                 3.7020760    0.239384325 0.28328971
## Parvimonas                                 40.8491501    0.072677087 0.14162833
## Filifactor                                  6.8375183    0.362986981 0.27615886
## Mogibacterium                              41.0770621    0.008207512 0.09781346
## Peptostreptococcaceae_[XI][G.1]            67.4763877    0.025609082 0.17523886
## Peptostreptococcaceae_[XI][G.4]             0.4176611    0.139628872 0.37737550
## Peptostreptococcaceae_[XI][G.5]             1.6627493    0.230899827 0.39586971
## Peptostreptococcaceae_[XI][G.6]             1.0137786    0.207643874 0.39358344
## Peptostreptococcaceae_[XI][G.7]             4.7083982    0.723519048 0.27119649
## Peptostreptococcaceae_[XI][G.9]             8.7265107    0.187957795 0.16134650
## Peptostreptococcus                         42.8338056    0.151143260 0.20454061
## Ruminococcaceae_[G.1]                      25.1020535   -0.080961603 0.20736153
## Ruminococcaceae_[G.2]                      41.4593204   -0.069784881 0.17268590
## Bulleidia                                   3.8570393    0.112992470 0.30484110
## Eggerthia                                   1.4537243   -0.064152137 0.40261232
## Solobacterium                              74.0587622   -0.062600680 0.11508253
## Mycoplasma                                  4.4957386    0.154121922 0.24345926
## Mitsuokella                                26.8689347   -0.339360835 0.21813493
## Selenomonas                               221.9840267   -0.198002921 0.09796231
## Family.Selenomonadaceae.                    5.0346400   -0.416828780 0.25343319
## Class.Negativicutes.                        0.7371549    0.080112289 0.23590697
## Anaeroglobus                                8.7009758    0.277648743 0.23481907
## Dialister                                  36.4394005   -0.015443758 0.12066556
## Megasphaera                               407.9853210   -0.177054538 0.12695330
## Family.Veillonellaceae.                     0.5266292    0.027918321 0.27693031
## Veillonella                              5067.7924258    0.080394855 0.07131889
## Veillonellaceae_[G.1]                       6.1442803    0.099802662 0.19845264
```

```
## Phylum.Firmicutes.                       0.8912626   -0.860064839 0.32424219
## Fusobacterium                          927.1066406    0.017554768 0.09508209
## Leptotrichia                           683.2517404   -0.123397867 0.09791369
## Gracilibacteria_(GN02)_[G.1]             2.4068360    0.302019099 0.31748261
## Lautropia                               68.4201506   -0.096045835 0.18242725
## Ottowia                                  2.2320032   -0.568133622 0.40367737
## Eikenella                                8.7736001    0.063101414 0.15256410
## Kingella                                99.8204227    0.223129908 0.13117152
## Neisseria                             1329.0853342    0.034298831 0.15019438
## Family.Neisseriaceae.                    5.6824349   -0.165236928 0.34484596
## Campylobacter                          257.8128378   -0.173101459 0.08392443
## Cardiobacterium                         14.7982481    0.119400531 0.13080639
## Aggregatibacter                        275.5218474   -0.128124947 0.17318722
## Haemophilus                           2905.9316825    0.108278025 0.11561001
## Family.Pasteurellaceae.                  2.1629761    0.537074442 0.46661252
## Saccharibacteria_(TM7)_[G.1]            36.0392035   -0.175133822 0.19300860
## Saccharibacteria_(TM7)_[G.3]             7.1137273   -0.007101074 0.26901986
## Saccharibacteria_(TM7)_[G.6]             7.2376188   -0.777957946 0.34107382
## Family.Saccharibacteria_(TM7)_[F-1].     1.1003857    0.253780909 0.42246952
## Saccharibacteria_(TM7)_[G.5]             0.9162822    0.479608816 0.40246903
## Treponema                               63.1946030   -0.056778307 0.18294291
## Fretibacterium                           8.2679474    0.031268962 0.22117304
## Kingdom.Bacteria.                        9.3689884   -0.047851262 0.27995841
## Others                                  33.1524239    0.396039540 0.18608324
##                                             stat         pval   ordering
## Absconditabacteria_(SR1)_[G.1]        2.91410339 0.2329219880 Medium>Low
## Actinomyces                           4.96555933 0.0835107709 Low>Medium
## Peptidiphaga                          1.35798608 0.5071273925 Low>Medium
## Schaalia                              2.85842190 0.2394978233 Low>Medium
## Family.Actinomycetaceae.              3.13475349 0.2085916541 Low>Medium
## Rothia                                1.59943785 0.4494552770 Medium>Low
## Alloscardovia                         4.28402334 0.1174183983 Medium>Low
## Bifidobacterium                       2.41263401 0.2992975633 Low>Medium
## Parascardovia                         1.14285011 0.5647201065 Medium>Low
## Scardovia                             3.67186950 0.1594643730 Medium>Low
## Corynebacterium                       1.74442257 0.4180261516 Low>Medium
## Olsenella                            10.28979126 0.0058290827 Medium>Low
## Atopobium                             0.57515295 0.7500792025 Low>Medium
## Slackia                               1.39154836 0.4986882236 Low>Medium
## Cryptobacterium                       6.06009921 0.0483132415 Low>Medium
## Bacteroidetes_[G.3]                   0.64461544 0.7244752198 Low>Medium
## Bacteroidetes_[G.5]                   0.19486849 0.9071619888 Low>Medium
## Family.Bacteroidetes_[F-1].           1.91640724 0.3835813257 Low>Medium
## Bacteroidaceae_[G.1]                  6.50877475 0.0386044635 Medium>Low
## Bacteroidales_[G.2]                   2.47178746 0.2905749506 Low>Medium
## Porphyromonas                         1.77596286 0.4114855279 Medium>Low
## Tannerella                            3.06389399 0.2161144841 Low>Medium
## Alloprevotella                        2.10069328 0.3498164682 Medium>Low
## Prevotella                            0.65212162 0.7217612972 Low>Medium
## Bergeyella                            3.22004205 0.1998834119 Medium>Low
## Capnocytophaga                        0.09864141 0.9518758115 Low>Medium
## Gemella                               1.16319919 0.5590034708 Medium>Low
## Abiotrophia                           3.18343706 0.2035754607 Low>Medium
## Granulicatella                        4.62064592 0.0992291993 Medium>Low
```

```
## Lactobacillus                            17.13563159 0.0001901275 Medium>Low
## Streptococcus                             3.85690469 0.1453730114 Medium>Low
## Order.Lactobacillales.                   -3.62406876 1.0000000000 Medium>Low
## Class.Bacilli.                            5.56384308 0.0619194125 Low>Medium
## Clostridiales_[F.1][G-1]                  0.99210211 0.6089305507 Medium>Low
## Butyrivibrio                              4.29025399 0.1170531711 Low>Medium
## Catonella                                 0.44329617 0.8011972680 Low>Medium
## Johnsonella                               0.60383891 0.7393976187 Low>Medium
## Lachnoanaerobaculum                       1.22616912 0.5416774525 Low>Medium
## Lachnospiraceae_[G.2]                     5.39234900 0.0674631000 Low>Medium
## Lachnospiraceae_[G.3]                     0.17232669 0.9174443591 Medium>Low
## Lachnospiraceae_[G.7]                    17.72985150 0.0001412575 Low>Medium
## Lachnospiraceae_[G.8]                     0.95032568 0.6217837976 Medium>Low
## Oribacterium                              2.90779860 0.2336574096 Low>Medium
## Shuttleworthia                            0.46456679 0.7927214384 Low>Medium
## Stomatobaculum                            3.92805750 0.1402920791 Low>Medium
## Family.Lachnospiraceae_[XIV].             4.66215568 0.0971909345 Low>Medium
## Peptococcus                               4.20117845 0.1223842949 Medium>Low
## Parvimonas                                0.34716615 0.8406473116 Medium>Low
## Filifactor                                2.32508086 0.3126908020 Medium>Low
## Mogibacterium                             3.65293527 0.1609812089 Medium>Low
## Peptostreptococcaceae_[XI][G.1]           0.26395180 0.8763621163 Medium>Low
## Peptostreptococcaceae_[XI][G.4]          -0.29616246 1.0000000000 Medium>Low
## Peptostreptococcaceae_[XI][G.5]           1.53371797 0.4644696861 Medium>Low
## Peptostreptococcaceae_[XI][G.6]           1.01259087 0.6027242793 Medium>Low
## Peptostreptococcaceae_[XI][G.7]           7.43922641 0.0242433432 Medium>Low
## Peptostreptococcaceae_[XI][G.9]           2.10481482 0.3490963194 Medium>Low
## Peptostreptococcus                        0.95130686 0.6214788313 Medium>Low
## Ruminococcaceae_[G.1]                     0.69623113 0.7060172818 Low>Medium
## Ruminococcaceae_[G.2]                     0.58197629 0.7475245398 Low>Medium
## Bulleidia                                 0.19561981 0.9068212703 Medium>Low
## Eggerthia                                 0.78781103 0.6744177752 Low>Medium
## Solobacterium                             0.71439812 0.6996332132 Low>Medium
## Mycoplasma                                1.43092551 0.4889657885 Medium>Low
## Mitsuokella                               3.32626219 0.1895445676 Low>Medium
## Selenomonas                               4.13179514 0.1267045133 Low>Medium
## Family.Selenomonadaceae.                 10.63667930 0.0049008842 Low>Medium
## Class.Negativicutes.                      0.64610152 0.7239371051 Medium>Low
## Anaeroglobus                              1.40671112 0.4949217739 Medium>Low
## Dialister                                 0.89621283 0.6388366973 Low>Medium
## Megasphaera                               2.38404949 0.3036059163 Low>Medium
## Family.Veillonellaceae.                  -0.03096705 1.0000000000 Medium>Low
## Veillonella                               5.28683122 0.0711179430 Medium>Low
## Veillonellaceae_[G.1]                     0.30402667 0.8589768271 Medium>Low
## Phylum.Firmicutes.                        7.12920373 0.0283082537 Low>Medium
## Fusobacterium                             0.42125445 0.8100759867 Medium>Low
## Leptotrichia                              3.42599544 0.1803244199 Low>Medium
## Gracilibacteria_(GN02)_[G.1]              1.20293109 0.5480079175 Medium>Low
## Lautropia                                 1.16481380 0.5585523683 Low>Medium
## Ottowia                                   2.81590358 0.2446438525 Low>Medium
## Eikenella                                 2.07660929 0.3540544210 Medium>Low
## Kingella                                  2.90194510 0.2343422675 Medium>Low
## Neisseria                                 0.17707641 0.9152681438 Medium>Low
## Family.Neisseriaceae.                     0.28055671 0.8691162801 Low>Medium
```

```
## Campylobacter                          4.57057287 0.1017449147 Low>Medium
## Cardiobacterium                        2.69682737 0.2596518241 Medium>Low
## Aggregatibacter                        0.71017133 0.7011133786 Low>Medium
## Haemophilus                            1.44413837 0.4857461159 Medium>Low
## Family.Pasteurellaceae.                1.67213698 0.4334111386 Medium>Low
## Saccharibacteria_(TM7)_[G.1]           2.56986766 0.2766688902 Low>Medium
## Saccharibacteria_(TM7)_[G.3]           0.47822742 0.7873253501 Low>Medium
## Saccharibacteria_(TM7)_[G.6]           6.04840036 0.0485966743 Low>Medium
## Family.Saccharibacteria_(TM7)_[F-1].   2.01987590 0.3642415800 Medium>Low
## Saccharibacteria_(TM7)_[G.5]           1.48222635 0.4765830998 Medium>Low
## Treponema                              0.10706773 0.9478738412 Low>Medium
## Fretibacterium                         1.20133270 0.5484460566 Medium>Low
## Kingdom.Bacteria.                      0.69760213 0.7055334704 Low>Medium
## Others                                 4.84450899 0.0887213698 Medium>Low
##                                          pval.adj
## Absconditabacteria_(SR1)_[G.1]        0.659179269
## Actinomyces                           0.580544513
## Peptidiphaga                          0.848126846
## Schaalia                              0.659179269
## Family.Actinomycetaceae.              0.659179269
## Rothia                                0.848126846
## Alloscardovia                         0.585254181
## Bifidobacterium                       0.718287168
## Parascardovia                         0.855903911
## Scardovia                             0.624607090
## Corynebacterium                       0.827521157
## Olsenella                             0.141355256
## Atopobium                             0.909471033
## Slackia                               0.848126846
## Cryptobacterium                       0.523764156
## Bacteroidetes_[G.3]                   0.909471033
## Bacteroidetes_[G.5]                   0.967305466
## Family.Bacteroidetes_[F-1].           0.791646566
## Bacteroidaceae_[G.1]                  0.523764156
## Bacteroidales_[G.2]                   0.718287168
## Porphyromonas                         0.827521157
## Tannerella                            0.659179269
## Alloprevotella                        0.763183974
## Prevotella                            0.909471033
## Bergeyella                            0.659179269
## Capnocytophaga                        0.982254827
## Gemella                               0.855903911
## Abiotrophia                           0.659179269
## Granulicatella                        0.580544513
## Lactobacillus                         0.009221183
## Streptococcus                         0.613094874
## Order.Lactobacillales.                1.000000000
## Class.Bacilli.                        0.574870039
## Clostridiales_[F.1][G-1]              0.886956300
## Butyrivibrio                          0.585254181
## Catonella                             0.935444889
## Johnsonella                           0.909471033
## Lachnoanaerobaculum                   0.855903911
## Lachnospiraceae_[G.2]                 0.574870039
```

```
## Lachnospiraceae_[G.3]                0.967305466
## Lachnospiraceae_[G.7]                0.009221183
## Lachnospiraceae_[G.8]                0.886956300
## Oribacterium                         0.659179269
## Shuttleworthia                       0.935444889
## Stomatobaculum                       0.613094874
## Family.Lachnospiraceae_[XIV].        0.580544513
## Peptococcus                          0.585254181
## Parvimonas                           0.959326932
## Filifactor                           0.722166852
## Mogibacterium                        0.624607090
## Peptostreptococcaceae_[XI][G.1]      0.965990060
## Peptostreptococcaceae_[XI][G.4]      1.000000000
## Peptostreptococcaceae_[XI][G.5]      0.848126846
## Peptostreptococcaceae_[XI][G.6]      0.886956300
## Peptostreptococcaceae_[XI][G.7]      0.457650102
## Peptostreptococcaceae_[XI][G.9]      0.763183974
## Peptostreptococcus                   0.886956300
## Ruminococcaceae_[G.1]                0.909471033
## Ruminococcaceae_[G.2]                0.909471033
## Bulleidia                            0.967305466
## Eggerthia                            0.909471033
## Solobacterium                        0.909471033
## Mycoplasma                           0.848126846
## Mitsuokella                          0.659179269
## Selenomonas                          0.585254181
## Family.Selenomonadaceae.             0.141355256
## Class.Negativicutes.                 0.909471033
## Anaeroglobus                         0.848126846
## Dialister                            0.898074777
## Megasphaera                          0.718287168
## Family.Veillonellaceae.              1.000000000
## Veillonella                          0.574870039
## Veillonellaceae_[G.1]                0.965990060
## Phylum.Firmicutes.                   0.457650102
## Fusobacterium                        0.935444889
## Leptotrichia                         0.659179269
## Gracilibacteria_(GN02)_[G.1]         0.855903911
## Lautropia                            0.855903911
## Ottowia                              0.659179269
## Eikenella                            0.763183974
## Kingella                             0.659179269
## Neisseria                            0.967305466
## Family.Neisseriaceae.                0.965990060
## Campylobacter                        0.580544513
## Cardiobacterium                      0.680708836
## Aggregatibacter                      0.909471033
## Haemophilus                          0.848126846
## Family.Pasteurellaceae.              0.840817609
## Saccharibacteria_(TM7)_[G.1]         0.706233746
## Saccharibacteria_(TM7)_[G.3]         0.935444889
## Saccharibacteria_(TM7)_[G.6]         0.523764156
## Family.Saccharibacteria_(TM7)_[F-1]. 0.768074636
## Saccharibacteria_(TM7)_[G.5]         0.848126846
```

```
## Treponema                              0.982254827
## Fretibacterium                         0.855903911
## Kingdom.Bacteria.                      0.909471033
## Others                                 0.580544513
##                                                                    Feature
## Absconditabacteria_(SR1)_[G.1]          Absconditabacteria_(SR1)_[G.1]
## Actinomyces                                                    Actinomyces
## Peptidiphaga                                                  Peptidiphaga
## Schaalia                                                          Schaalia
## Family.Actinomycetaceae.                        Family.Actinomycetaceae.
## Rothia                                                              Rothia
## Alloscardovia                                                Alloscardovia
## Bifidobacterium                                            Bifidobacterium
## Parascardovia                                                Parascardovia
## Scardovia                                                        Scardovia
## Corynebacterium                                            Corynebacterium
## Olsenella                                                        Olsenella
## Atopobium                                                        Atopobium
## Slackia                                                            Slackia
## Cryptobacterium                                            Cryptobacterium
## Bacteroidetes_[G.3]                                    Bacteroidetes_[G.3]
## Bacteroidetes_[G.5]                                    Bacteroidetes_[G.5]
## Family.Bacteroidetes_[F-1].                    Family.Bacteroidetes_[F-1].
## Bacteroidaceae_[G.1]                                  Bacteroidaceae_[G.1]
## Bacteroidales_[G.2]                                    Bacteroidales_[G.2]
## Porphyromonas                                                Porphyromonas
## Tannerella                                                      Tannerella
## Alloprevotella                                              Alloprevotella
## Prevotella                                                      Prevotella
## Bergeyella                                                      Bergeyella
## Capnocytophaga                                              Capnocytophaga
## Gemella                                                            Gemella
## Abiotrophia                                                    Abiotrophia
## Granulicatella                                              Granulicatella
## Lactobacillus                                                Lactobacillus
## Streptococcus                                                Streptococcus
## Order.Lactobacillales.                            Order.Lactobacillales.
## Class.Bacilli.                                              Class.Bacilli.
## Clostridiales_[F.1][G-1]                        Clostridiales_[F.1][G-1]
## Butyrivibrio                                                  Butyrivibrio
## Catonella                                                        Catonella
## Johnsonella                                                    Johnsonella
## Lachnoanaerobaculum                                    Lachnoanaerobaculum
## Lachnospiraceae_[G.2]                                Lachnospiraceae_[G.2]
## Lachnospiraceae_[G.3]                                Lachnospiraceae_[G.3]
## Lachnospiraceae_[G.7]                                Lachnospiraceae_[G.7]
## Lachnospiraceae_[G.8]                                Lachnospiraceae_[G.8]
## Oribacterium                                                  Oribacterium
## Shuttleworthia                                              Shuttleworthia
## Stomatobaculum                                              Stomatobaculum
## Family.Lachnospiraceae_[XIV].                Family.Lachnospiraceae_[XIV].
## Peptococcus                                                    Peptococcus
## Parvimonas                                                      Parvimonas
## Filifactor                                                      Filifactor
```

```
## Mogibacterium                                                        Mogibacterium
## Peptostreptococcaceae_[XI][G.1]          Peptostreptococcaceae_[XI][G.1]
## Peptostreptococcaceae_[XI][G.4]          Peptostreptococcaceae_[XI][G.4]
## Peptostreptococcaceae_[XI][G.5]          Peptostreptococcaceae_[XI][G.5]
## Peptostreptococcaceae_[XI][G.6]          Peptostreptococcaceae_[XI][G.6]
## Peptostreptococcaceae_[XI][G.7]          Peptostreptococcaceae_[XI][G.7]
## Peptostreptococcaceae_[XI][G.9]          Peptostreptococcaceae_[XI][G.9]
## Peptostreptococcus                                        Peptostreptococcus
## Ruminococcaceae_[G.1]                                  Ruminococcaceae_[G.1]
## Ruminococcaceae_[G.2]                                  Ruminococcaceae_[G.2]
## Bulleidia                                                              Bulleidia
## Eggerthia                                                              Eggerthia
## Solobacterium                                                      Solobacterium
## Mycoplasma                                                            Mycoplasma
## Mitsuokella                                                          Mitsuokella
## Selenomonas                                                          Selenomonas
## Family.Selenomonadaceae.                              Family.Selenomonadaceae.
## Class.Negativicutes.                                      Class.Negativicutes.
## Anaeroglobus                                                        Anaeroglobus
## Dialister                                                              Dialister
## Megasphaera                                                          Megasphaera
## Family.Veillonellaceae.                                Family.Veillonellaceae.
## Veillonella                                                          Veillonella
## Veillonellaceae_[G.1]                                  Veillonellaceae_[G.1]
## Phylum.Firmicutes.                                          Phylum.Firmicutes.
## Fusobacterium                                                      Fusobacterium
## Leptotrichia                                                        Leptotrichia
## Gracilibacteria_(GN02)_[G.1]                    Gracilibacteria_(GN02)_[G.1]
## Lautropia                                                              Lautropia
## Ottowia                                                                  Ottowia
## Eikenella                                                              Eikenella
## Kingella                                                                Kingella
## Neisseria                                                              Neisseria
## Family.Neisseriaceae.                                    Family.Neisseriaceae.
## Campylobacter                                                      Campylobacter
## Cardiobacterium                                                  Cardiobacterium
## Aggregatibacter                                                  Aggregatibacter
## Haemophilus                                                          Haemophilus
## Family.Pasteurellaceae.                                Family.Pasteurellaceae.
## Saccharibacteria_(TM7)_[G.1]                    Saccharibacteria_(TM7)_[G.1]
## Saccharibacteria_(TM7)_[G.3]                    Saccharibacteria_(TM7)_[G.3]
## Saccharibacteria_(TM7)_[G.6]                    Saccharibacteria_(TM7)_[G.6]
## Family.Saccharibacteria_(TM7)_[F-1]. Family.Saccharibacteria_(TM7)_[F-1].
## Saccharibacteria_(TM7)_[G.5]                    Saccharibacteria_(TM7)_[G.5]
## Treponema                                                              Treponema
## Fretibacterium                                                    Fretibacterium
## Kingdom.Bacteria.                                            Kingdom.Bacteria.
## Others                                                                    Others
##                                                           Method
## Absconditabacteria_(SR1)_[G.1]       DESeq2 man. geoMeans (ds2)
## Actinomyces                          DESeq2 man. geoMeans (ds2)
## Peptidiphaga                         DESeq2 man. geoMeans (ds2)
## Schaalia                             DESeq2 man. geoMeans (ds2)
## Family.Actinomycetaceae.             DESeq2 man. geoMeans (ds2)
```

```
## Rothia                             DESeq2 man. geoMeans (ds2)
## Alloscardovia                      DESeq2 man. geoMeans (ds2)
## Bifidobacterium                    DESeq2 man. geoMeans (ds2)
## Parascardovia                      DESeq2 man. geoMeans (ds2)
## Scardovia                          DESeq2 man. geoMeans (ds2)
## Corynebacterium                    DESeq2 man. geoMeans (ds2)
## Olsenella                          DESeq2 man. geoMeans (ds2)
## Atopobium                          DESeq2 man. geoMeans (ds2)
## Slackia                            DESeq2 man. geoMeans (ds2)
## Cryptobacterium                    DESeq2 man. geoMeans (ds2)
## Bacteroidetes_[G.3]                DESeq2 man. geoMeans (ds2)
## Bacteroidetes_[G.5]                DESeq2 man. geoMeans (ds2)
## Family.Bacteroidetes_[F-1].        DESeq2 man. geoMeans (ds2)
## Bacteroidaceae_[G.1]               DESeq2 man. geoMeans (ds2)
## Bacteroidales_[G.2]                DESeq2 man. geoMeans (ds2)
## Porphyromonas                      DESeq2 man. geoMeans (ds2)
## Tannerella                         DESeq2 man. geoMeans (ds2)
## Alloprevotella                     DESeq2 man. geoMeans (ds2)
## Prevotella                         DESeq2 man. geoMeans (ds2)
## Bergeyella                         DESeq2 man. geoMeans (ds2)
## Capnocytophaga                     DESeq2 man. geoMeans (ds2)
## Gemella                            DESeq2 man. geoMeans (ds2)
## Abiotrophia                        DESeq2 man. geoMeans (ds2)
## Granulicatella                     DESeq2 man. geoMeans (ds2)
## Lactobacillus                      DESeq2 man. geoMeans (ds2)
## Streptococcus                      DESeq2 man. geoMeans (ds2)
## Order.Lactobacillales.             DESeq2 man. geoMeans (ds2)
## Class.Bacilli.                     DESeq2 man. geoMeans (ds2)
## Clostridiales_[F.1][G-1]           DESeq2 man. geoMeans (ds2)
## Butyrivibrio                       DESeq2 man. geoMeans (ds2)
## Catonella                          DESeq2 man. geoMeans (ds2)
## Johnsonella                        DESeq2 man. geoMeans (ds2)
## Lachnoanaerobaculum                DESeq2 man. geoMeans (ds2)
## Lachnospiraceae_[G.2]              DESeq2 man. geoMeans (ds2)
## Lachnospiraceae_[G.3]              DESeq2 man. geoMeans (ds2)
## Lachnospiraceae_[G.7]              DESeq2 man. geoMeans (ds2)
## Lachnospiraceae_[G.8]              DESeq2 man. geoMeans (ds2)
## Oribacterium                       DESeq2 man. geoMeans (ds2)
## Shuttleworthia                     DESeq2 man. geoMeans (ds2)
## Stomatobaculum                     DESeq2 man. geoMeans (ds2)
## Family.Lachnospiraceae_[XIV].      DESeq2 man. geoMeans (ds2)
## Peptococcus                        DESeq2 man. geoMeans (ds2)
## Parvimonas                         DESeq2 man. geoMeans (ds2)
## Filifactor                         DESeq2 man. geoMeans (ds2)
## Mogibacterium                      DESeq2 man. geoMeans (ds2)
## Peptostreptococcaceae_[XI][G.1]    DESeq2 man. geoMeans (ds2)
## Peptostreptococcaceae_[XI][G.4]    DESeq2 man. geoMeans (ds2)
## Peptostreptococcaceae_[XI][G.5]    DESeq2 man. geoMeans (ds2)
## Peptostreptococcaceae_[XI][G.6]    DESeq2 man. geoMeans (ds2)
## Peptostreptococcaceae_[XI][G.7]    DESeq2 man. geoMeans (ds2)
## Peptostreptococcaceae_[XI][G.9]    DESeq2 man. geoMeans (ds2)
## Peptostreptococcus                 DESeq2 man. geoMeans (ds2)
## Ruminococcaceae_[G.1]              DESeq2 man. geoMeans (ds2)
## Ruminococcaceae_[G.2]              DESeq2 man. geoMeans (ds2)
```

```
## Bulleidia                              DESeq2 man. geoMeans (ds2)
## Eggerthia                              DESeq2 man. geoMeans (ds2)
## Solobacterium                          DESeq2 man. geoMeans (ds2)
## Mycoplasma                             DESeq2 man. geoMeans (ds2)
## Mitsuokella                            DESeq2 man. geoMeans (ds2)
## Selenomonas                            DESeq2 man. geoMeans (ds2)
## Family.Selenomonadaceae.               DESeq2 man. geoMeans (ds2)
## Class.Negativicutes.                   DESeq2 man. geoMeans (ds2)
## Anaeroglobus                           DESeq2 man. geoMeans (ds2)
## Dialister                              DESeq2 man. geoMeans (ds2)
## Megasphaera                            DESeq2 man. geoMeans (ds2)
## Family.Veillonellaceae.                DESeq2 man. geoMeans (ds2)
## Veillonella                            DESeq2 man. geoMeans (ds2)
## Veillonellaceae_[G.1]                  DESeq2 man. geoMeans (ds2)
## Phylum.Firmicutes.                     DESeq2 man. geoMeans (ds2)
## Fusobacterium                          DESeq2 man. geoMeans (ds2)
## Leptotrichia                           DESeq2 man. geoMeans (ds2)
## Gracilibacteria_(GN02)_[G.1]           DESeq2 man. geoMeans (ds2)
## Lautropia                              DESeq2 man. geoMeans (ds2)
## Ottowia                                DESeq2 man. geoMeans (ds2)
## Eikenella                              DESeq2 man. geoMeans (ds2)
## Kingella                               DESeq2 man. geoMeans (ds2)
## Neisseria                              DESeq2 man. geoMeans (ds2)
## Family.Neisseriaceae.                  DESeq2 man. geoMeans (ds2)
## Campylobacter                          DESeq2 man. geoMeans (ds2)
## Cardiobacterium                        DESeq2 man. geoMeans (ds2)
## Aggregatibacter                        DESeq2 man. geoMeans (ds2)
## Haemophilus                            DESeq2 man. geoMeans (ds2)
## Family.Pasteurellaceae.                DESeq2 man. geoMeans (ds2)
## Saccharibacteria_(TM7)_[G.1]           DESeq2 man. geoMeans (ds2)
## Saccharibacteria_(TM7)_[G.3]           DESeq2 man. geoMeans (ds2)
## Saccharibacteria_(TM7)_[G.6]           DESeq2 man. geoMeans (ds2)
## Family.Saccharibacteria_(TM7)_[F-1]. DESeq2 man. geoMeans (ds2)
## Saccharibacteria_(TM7)_[G.5]           DESeq2 man. geoMeans (ds2)
## Treponema                              DESeq2 man. geoMeans (ds2)
## Fretibacterium                         DESeq2 man. geoMeans (ds2)
## Kingdom.Bacteria.                      DESeq2 man. geoMeans (ds2)
## Others                                 DESeq2 man. geoMeans (ds2)
```

```
write.table(final, file="Waist_cat_ds2.txt", sep="\t", dec=",", row.names=F)
write.table(final[final$pval<0.05,], file="Waist_cat_ds2_sig.txt", sep="\t", dec=",", row.names=F)
final <- DA.kru(Microbio, predictor = Phe$Waist_cat)
final
```

```
##                                            pval  pval.adj
## Absconditabacteria_(SR1)_[G.1]      0.769349516 0.9700471
## Actinomyces                         0.174643011 0.9352968
## Peptidiphaga                        0.965169834 0.9854892
## Schaalia                            0.667978987 0.9700471
## Family.Actinomycetaceae.            0.346653762 0.9352968
## Rothia                              0.090701596 0.9073390
## Alloscardovia                       0.903533049 0.9802937
## Bifidobacterium                     0.826433219 0.9700471
## Parascardovia                       0.151216124 0.9352968
```

```
## Scardovia                              0.275891564 0.9352968
## Corynebacterium                        0.475895073 0.9477067
## Olsenella                              0.004183123 0.4057629
## Atopobium                              0.826503301 0.9700471
## Slackia                                0.279005459 0.9352968
## Cryptobacterium                        0.684319961 0.9700471
## Bacteroidetes_[G.3]                    0.919656930 0.9802937
## Bacteroidetes_[G.5]                    0.982358508 0.9925914
## Family.Bacteroidetes_[F-1].            0.009099309 0.4255043
## Bacteroidaceae_[G.1]                   0.089482941 0.9073390
## Bacteroidales_[G.2]                    0.467106511 0.9477067
## Porphyromonas                          0.361148894 0.9352968
## Tannerella                             0.324550213 0.9352968
## Alloprevotella                         0.269635620 0.9352968
## Prevotella                             0.999994072 0.9999941
## Bergeyella                             0.426700402 0.9406804
## Capnocytophaga                         0.820510965 0.9700471
## Gemella                                0.744234591 0.9700471
## Abiotrophia                            0.611535944 0.9700471
## Granulicatella                         0.273806930 0.9352968
## Lactobacillus                          0.568666714 0.9700471
## Streptococcus                          0.908590797 0.9802937
## Order.Lactobacillales.                 0.665041654 0.9700471
## Class.Bacilli.                         0.701478456 0.9700471
## Clostridiales_[F.1][G-1]               0.824685389 0.9700471
## Butyrivibrio                           0.285162367 0.9352968
## Catonella                              0.950048501 0.9854892
## Johnsonella                            0.490659971 0.9477067
## Lachnoanaerobaculum                    0.762208366 0.9700471
## Lachnospiraceae_[G.2]                  0.102894113 0.9073390
## Lachnospiraceae_[G.3]                  0.637925212 0.9700471
## Lachnospiraceae_[G.7]                  0.403677202 0.9352968
## Lachnospiraceae_[G.8]                  0.516954530 0.9477067
## Oribacterium                           0.412679596 0.9352968
## Shuttleworthia                         0.379690971 0.9352968
## Stomatobaculum                         0.343028341 0.9352968
## Family.Lachnospiraceae_[XIV].          0.314957155 0.9352968
## Peptococcus                            0.081668346 0.9073390
## Parvimonas                             0.135331668 0.9352968
## Filifactor                             0.222295789 0.9352968
## Mogibacterium                          0.097340892 0.9073390
## Peptostreptococcaceae_[XI][G.1]        0.756818813 0.9700471
## Peptostreptococcaceae_[XI][G.4]        0.848834185 0.9700471
## Peptostreptococcaceae_[XI][G.5]        0.185771075 0.9352968
## Peptostreptococcaceae_[XI][G.6]        0.309961598 0.9352968
## Peptostreptococcaceae_[XI][G.7]        0.288291889 0.9352968
## Peptostreptococcaceae_[XI][G.9]        0.066932618 0.9073390
## Peptostreptococcus                     0.272001534 0.9352968
## Ruminococcaceae_[G.1]                  0.786990021 0.9700471
## Ruminococcaceae_[G.2]                  0.903687045 0.9802937
## Bulleidia                              0.662575687 0.9700471
## Eggerthia                              0.947516271 0.9854892
## Solobacterium                          0.864833106 0.9754513
## Mycoplasma                             0.128921437 0.9352968
```

```
## Mitsuokella                          0.290914868 0.9352968
## Selenomonas                          0.787367636 0.9700471
## Family.Selenomonadaceae.             0.023694343 0.5745878
## Class.Negativicutes.                 0.838495353 0.9700471
## Anaeroglobus                         0.543681572 0.9700471
## Dialister                            0.147579452 0.9352968
## Megasphaera                          0.468318465 0.9477067
## Family.Veillonellaceae.              0.703854795 0.9700471
## Veillonella                          0.253652955 0.9352968
## Veillonellaceae_[G.1]                0.504554470 0.9477067
## Phylum.Firmicutes.                   0.500430339 0.9477067
## Fusobacterium                        0.262273399 0.9352968
## Leptotrichia                         0.402235013 0.9352968
## Gracilibacteria_(GN02)_[G.1]         0.850041245 0.9700471
## Lautropia                            0.958959085 0.9854892
## Ottowia                              0.827105390 0.9700471
## Eikenella                            0.828960825 0.9700471
## Kingella                             0.683852666 0.9700471
## Neisseria                            0.575320523 0.9700471
## Family.Neisseriaceae.                0.729416320 0.9700471
## Campylobacter                        0.216188797 0.9352968
## Cardiobacterium                      0.414616108 0.9352968
## Aggregatibacter                      0.396405920 0.9352968
## Haemophilus                          0.733405809 0.9700471
## Family.Pasteurellaceae.              0.377212590 0.9352968
## Saccharibacteria_(TM7)_[G.1]         0.599576666 0.9700471
## Saccharibacteria_(TM7)_[G.3]         0.441167573 0.9477067
## Saccharibacteria_(TM7)_[G.6]         0.013159926 0.4255043
## Family.Saccharibacteria_(TM7)_[F-1]. 0.700562759 0.9700471
## Saccharibacteria_(TM7)_[G.5]         0.056976054 0.9073390
## Treponema                            0.600126032 0.9700471
## Fretibacterium                       0.517819113 0.9477067
## Kingdom.Bacteria.                    0.253203546 0.9352968
## Others                               0.913001900 0.9802937
##                                                                    Feature
## Absconditabacteria_(SR1)_[G.1]            Absconditabacteria_(SR1)_[G.1]
## Actinomyces                                                    Actinomyces
## Peptidiphaga                                                  Peptidiphaga
## Schaalia                                                          Schaalia
## Family.Actinomycetaceae.                          Family.Actinomycetaceae.
## Rothia                                                              Rothia
## Alloscardovia                                                Alloscardovia
## Bifidobacterium                                            Bifidobacterium
## Parascardovia                                                Parascardovia
## Scardovia                                                        Scardovia
## Corynebacterium                                            Corynebacterium
## Olsenella                                                        Olsenella
## Atopobium                                                        Atopobium
## Slackia                                                            Slackia
## Cryptobacterium                                            Cryptobacterium
## Bacteroidetes_[G.3]                                    Bacteroidetes_[G.3]
## Bacteroidetes_[G.5]                                    Bacteroidetes_[G.5]
## Family.Bacteroidetes_[F-1].                    Family.Bacteroidetes_[F-1].
## Bacteroidaceae_[G.1]                                  Bacteroidaceae_[G.1]
```

```
## Bacteroidales_[G.2]                                          Bacteroidales_[G.2]
## Porphyromonas                                                      Porphyromonas
## Tannerella                                                            Tannerella
## Alloprevotella                                                    Alloprevotella
## Prevotella                                                            Prevotella
## Bergeyella                                                            Bergeyella
## Capnocytophaga                                                    Capnocytophaga
## Gemella                                                                  Gemella
## Abiotrophia                                                          Abiotrophia
## Granulicatella                                                    Granulicatella
## Lactobacillus                                                      Lactobacillus
## Streptococcus                                                      Streptococcus
## Order.Lactobacillales.                                    Order.Lactobacillales.
## Class.Bacilli.                                                    Class.Bacilli.
## Clostridiales_[F.1][G-1]                                Clostridiales_[F.1][G-1]
## Butyrivibrio                                                        Butyrivibrio
## Catonella                                                              Catonella
## Johnsonella                                                          Johnsonella
## Lachnoanaerobaculum                                          Lachnoanaerobaculum
## Lachnospiraceae_[G.2]                                      Lachnospiraceae_[G.2]
## Lachnospiraceae_[G.3]                                      Lachnospiraceae_[G.3]
## Lachnospiraceae_[G.7]                                      Lachnospiraceae_[G.7]
## Lachnospiraceae_[G.8]                                      Lachnospiraceae_[G.8]
## Oribacterium                                                        Oribacterium
## Shuttleworthia                                                    Shuttleworthia
## Stomatobaculum                                                    Stomatobaculum
## Family.Lachnospiraceae_[XIV].                      Family.Lachnospiraceae_[XIV].
## Peptococcus                                                          Peptococcus
## Parvimonas                                                            Parvimonas
## Filifactor                                                            Filifactor
## Mogibacterium                                                      Mogibacterium
## Peptostreptococcaceae_[XI][G.1]                Peptostreptococcaceae_[XI][G.1]
## Peptostreptococcaceae_[XI][G.4]                Peptostreptococcaceae_[XI][G.4]
## Peptostreptococcaceae_[XI][G.5]                Peptostreptococcaceae_[XI][G.5]
## Peptostreptococcaceae_[XI][G.6]                Peptostreptococcaceae_[XI][G.6]
## Peptostreptococcaceae_[XI][G.7]                Peptostreptococcaceae_[XI][G.7]
## Peptostreptococcaceae_[XI][G.9]                Peptostreptococcaceae_[XI][G.9]
## Peptostreptococcus                                            Peptostreptococcus
## Ruminococcaceae_[G.1]                                      Ruminococcaceae_[G.1]
## Ruminococcaceae_[G.2]                                      Ruminococcaceae_[G.2]
## Bulleidia                                                              Bulleidia
## Eggerthia                                                              Eggerthia
## Solobacterium                                                      Solobacterium
## Mycoplasma                                                            Mycoplasma
## Mitsuokella                                                          Mitsuokella
## Selenomonas                                                          Selenomonas
## Family.Selenomonadaceae.                                Family.Selenomonadaceae.
## Class.Negativicutes.                                        Class.Negativicutes.
## Anaeroglobus                                                        Anaeroglobus
## Dialister                                                              Dialister
## Megasphaera                                                          Megasphaera
## Family.Veillonellaceae.                                  Family.Veillonellaceae.
## Veillonella                                                          Veillonella
## Veillonellaceae_[G.1]                                      Veillonellaceae_[G.1]
```

```
## Phylum.Firmicutes.                                    Phylum.Firmicutes.
## Fusobacterium                                              Fusobacterium
## Leptotrichia                                                Leptotrichia
## Gracilibacteria_(GN02)_[G.1]                Gracilibacteria_(GN02)_[G.1]
## Lautropia                                                      Lautropia
## Ottowia                                                          Ottowia
## Eikenella                                                      Eikenella
## Kingella                                                        Kingella
## Neisseria                                                      Neisseria
## Family.Neisseriaceae.                              Family.Neisseriaceae.
## Campylobacter                                              Campylobacter
## Cardiobacterium                                          Cardiobacterium
## Aggregatibacter                                          Aggregatibacter
## Haemophilus                                                  Haemophilus
## Family.Pasteurellaceae.                          Family.Pasteurellaceae.
## Saccharibacteria_(TM7)_[G.1]                Saccharibacteria_(TM7)_[G.1]
## Saccharibacteria_(TM7)_[G.3]                Saccharibacteria_(TM7)_[G.3]
## Saccharibacteria_(TM7)_[G.6]                Saccharibacteria_(TM7)_[G.6]
## Family.Saccharibacteria_(TM7)_[F-1]. Family.Saccharibacteria_(TM7)_[F-1].
## Saccharibacteria_(TM7)_[G.5]                Saccharibacteria_(TM7)_[G.5]
## Treponema                                                      Treponema
## Fretibacterium                                            Fretibacterium
## Kingdom.Bacteria.                                      Kingdom.Bacteria.
## Others                                                            Others
##                                                Method
## Absconditabacteria_(SR1)_[G.1]       Kruskal-Wallis (kru)
## Actinomyces                          Kruskal-Wallis (kru)
## Peptidiphaga                         Kruskal-Wallis (kru)
## Schaalia                             Kruskal-Wallis (kru)
## Family.Actinomycetaceae.             Kruskal-Wallis (kru)
## Rothia                               Kruskal-Wallis (kru)
## Alloscardovia                        Kruskal-Wallis (kru)
## Bifidobacterium                      Kruskal-Wallis (kru)
## Parascardovia                        Kruskal-Wallis (kru)
## Scardovia                            Kruskal-Wallis (kru)
## Corynebacterium                      Kruskal-Wallis (kru)
## Olsenella                            Kruskal-Wallis (kru)
## Atopobium                            Kruskal-Wallis (kru)
## Slackia                              Kruskal-Wallis (kru)
## Cryptobacterium                      Kruskal-Wallis (kru)
## Bacteroidetes_[G.3]                  Kruskal-Wallis (kru)
## Bacteroidetes_[G.5]                  Kruskal-Wallis (kru)
## Family.Bacteroidetes_[F-1].          Kruskal-Wallis (kru)
## Bacteroidaceae_[G.1]                 Kruskal-Wallis (kru)
## Bacteroidales_[G.2]                  Kruskal-Wallis (kru)
## Porphyromonas                        Kruskal-Wallis (kru)
## Tannerella                           Kruskal-Wallis (kru)
## Alloprevotella                       Kruskal-Wallis (kru)
## Prevotella                           Kruskal-Wallis (kru)
## Bergeyella                           Kruskal-Wallis (kru)
## Capnocytophaga                       Kruskal-Wallis (kru)
## Gemella                              Kruskal-Wallis (kru)
## Abiotrophia                          Kruskal-Wallis (kru)
## Granulicatella                       Kruskal-Wallis (kru)
```

```
## Lactobacillus                          Kruskal-Wallis (kru)
## Streptococcus                          Kruskal-Wallis (kru)
## Order.Lactobacillales.                 Kruskal-Wallis (kru)
## Class.Bacilli.                         Kruskal-Wallis (kru)
## Clostridiales_[F.1][G-1]               Kruskal-Wallis (kru)
## Butyrivibrio                           Kruskal-Wallis (kru)
## Catonella                              Kruskal-Wallis (kru)
## Johnsonella                            Kruskal-Wallis (kru)
## Lachnoanaerobaculum                    Kruskal-Wallis (kru)
## Lachnospiraceae_[G.2]                  Kruskal-Wallis (kru)
## Lachnospiraceae_[G.3]                  Kruskal-Wallis (kru)
## Lachnospiraceae_[G.7]                  Kruskal-Wallis (kru)
## Lachnospiraceae_[G.8]                  Kruskal-Wallis (kru)
## Oribacterium                           Kruskal-Wallis (kru)
## Shuttleworthia                         Kruskal-Wallis (kru)
## Stomatobaculum                         Kruskal-Wallis (kru)
## Family.Lachnospiraceae_[XIV].          Kruskal-Wallis (kru)
## Peptococcus                            Kruskal-Wallis (kru)
## Parvimonas                             Kruskal-Wallis (kru)
## Filifactor                             Kruskal-Wallis (kru)
## Mogibacterium                          Kruskal-Wallis (kru)
## Peptostreptococcaceae_[XI][G.1]        Kruskal-Wallis (kru)
## Peptostreptococcaceae_[XI][G.4]        Kruskal-Wallis (kru)
## Peptostreptococcaceae_[XI][G.5]        Kruskal-Wallis (kru)
## Peptostreptococcaceae_[XI][G.6]        Kruskal-Wallis (kru)
## Peptostreptococcaceae_[XI][G.7]        Kruskal-Wallis (kru)
## Peptostreptococcaceae_[XI][G.9]        Kruskal-Wallis (kru)
## Peptostreptococcus                     Kruskal-Wallis (kru)
## Ruminococcaceae_[G.1]                  Kruskal-Wallis (kru)
## Ruminococcaceae_[G.2]                  Kruskal-Wallis (kru)
## Bulleidia                              Kruskal-Wallis (kru)
## Eggerthia                              Kruskal-Wallis (kru)
## Solobacterium                          Kruskal-Wallis (kru)
## Mycoplasma                             Kruskal-Wallis (kru)
## Mitsuokella                            Kruskal-Wallis (kru)
## Selenomonas                            Kruskal-Wallis (kru)
## Family.Selenomonadaceae.               Kruskal-Wallis (kru)
## Class.Negativicutes.                   Kruskal-Wallis (kru)
## Anaeroglobus                           Kruskal-Wallis (kru)
## Dialister                              Kruskal-Wallis (kru)
## Megasphaera                            Kruskal-Wallis (kru)
## Family.Veillonellaceae.                Kruskal-Wallis (kru)
## Veillonella                            Kruskal-Wallis (kru)
## Veillonellaceae_[G.1]                  Kruskal-Wallis (kru)
## Phylum.Firmicutes.                     Kruskal-Wallis (kru)
## Fusobacterium                          Kruskal-Wallis (kru)
## Leptotrichia                           Kruskal-Wallis (kru)
## Gracilibacteria_(GN02)_[G.1]           Kruskal-Wallis (kru)
## Lautropia                              Kruskal-Wallis (kru)
## Ottowia                                Kruskal-Wallis (kru)
## Eikenella                              Kruskal-Wallis (kru)
## Kingella                               Kruskal-Wallis (kru)
## Neisseria                              Kruskal-Wallis (kru)
## Family.Neisseriaceae.                  Kruskal-Wallis (kru)
```

```
## Campylobacter                          Kruskal-Wallis (kru)
## Cardiobacterium                        Kruskal-Wallis (kru)
## Aggregatibacter                        Kruskal-Wallis (kru)
## Haemophilus                            Kruskal-Wallis (kru)
## Family.Pasteurellaceae.                Kruskal-Wallis (kru)
## Saccharibacteria_(TM7)_[G.1]           Kruskal-Wallis (kru)
## Saccharibacteria_(TM7)_[G.3]           Kruskal-Wallis (kru)
## Saccharibacteria_(TM7)_[G.6]           Kruskal-Wallis (kru)
## Family.Saccharibacteria_(TM7)_[F-1]. Kruskal-Wallis (kru)
## Saccharibacteria_(TM7)_[G.5]           Kruskal-Wallis (kru)
## Treponema                              Kruskal-Wallis (kru)
## Fretibacterium                         Kruskal-Wallis (kru)
## Kingdom.Bacteria.                      Kruskal-Wallis (kru)
## Others                                 Kruskal-Wallis (kru)
```

```
write.table(final, file="Waist_cat_kru.txt", sep="\t", dec=",", row.names=F)
write.table(final[final$pval<0.05,], file="Waist_cat_kru_sig.txt", sep="\t", dec=",", row.names=F)
#################whratio_cat
table(Phe$whratio_cat, useNA="always")
```

```
##
## High  Low <NA>
##  592  154    0
```

```
final <- DA.ds2(Microbio, predictor = Phe$whratio_cat)
final
```

```
##        baseMean log2FoldChange      lfcSE        stat         pval ordering
## 1    61.0271812    0.139135713 0.30145666  0.46154467 6.444079e-01 Low>High
## 2   323.9127377   -0.065201149 0.10263472 -0.63527377 5.252499e-01 High>Low
## 3    22.0460481    0.167068782 0.14602053  1.14414580 2.525632e-01 Low>High
## 4   704.3072222   -0.083175062 0.08437627 -0.98576368 3.242491e-01 High>Low
## 5     5.4421107    0.347712024 0.20920226  1.66208539 9.649566e-02 Low>High
## 6  1939.2520922   -0.146285997 0.09876190 -1.48119873 1.385536e-01 High>Low
## 7    12.4599091   -0.515378893 0.26016894 -1.98093939 4.759807e-02 High>Low
## 8    10.8467069   -0.098279958 0.28373096 -0.34638432 7.290539e-01 High>Low
## 9     2.9486113   -0.567920200 0.39465362 -1.43903457 1.501407e-01 High>Low
## 10   13.4028767   -0.386294955 0.25088653 -1.53971977 1.236287e-01 High>Low
## 11   93.7260406   -0.070179877 0.10811910 -0.64909784 5.162751e-01 High>Low
## 12    1.5669010    0.338650509 0.23608224  1.43445993 1.514411e-01 Low>High
## 13  120.4914295   -0.004553044 0.09913485 -0.04592778 9.633678e-01 High>Low
## 14    1.3927613    0.303261398 0.22537745  1.34557118 1.784409e-01 Low>High
## 15    4.3017007   -0.077223409 0.24630913 -0.31352231 7.538839e-01 High>Low
## 16    6.2701667   -0.173648906 0.25656393 -0.67682510 4.985169e-01 High>Low
## 17    4.6746313    0.346947104 0.28243038  1.22843409 2.192841e-01 Low>High
## 18    0.8680201    0.112938663 0.40772707  0.27699575 7.817834e-01 Low>High
## 19    0.7921891   -0.489409090 0.38845462 -1.25988743 2.077100e-01 High>Low
## 20   12.1604713    0.153704535 0.14878506  1.03306429 3.015738e-01 Low>High
## 21  680.1285750   -0.044741743 0.13609115 -0.32876306 7.423348e-01 High>Low
## 22   22.0547383    0.227125616 0.09967248  2.27871944 2.268375e-02 Low>High
## 23  361.8645217    0.045886109 0.10788230  0.42533490 6.705926e-01 Low>High
## 24 3811.4256813    0.091461719 0.06846308  1.33592755 1.815730e-01 Low>High
## 25   72.5563323    0.188890446 0.10549653  1.79048968 7.337523e-02 Low>High
```

```
## 26  225.1152973    0.057938300 0.11305528  0.51247759 6.083168e-01 Low>High
## 27  309.0561286   -0.178020630 0.09429803 -1.88785098 5.904596e-02 High>Low
## 28   29.9768046    0.334290674 0.20369226  1.64115553 1.007651e-01 Low>High
## 29  306.2864969   -0.136239198 0.08229438 -1.65551027 9.782103e-02 High>Low
## 30   45.9200607   -2.459725557 0.34088904 -7.21561945 5.368901e-13 High>Low
## 31 3803.9265541   -0.186754625 0.07709339 -2.42244673 1.541639e-02 High>Low
## 32    0.2860988    0.225486000 0.27801811  0.81104790 4.173382e-01 Low>High
## 33    0.5750915    0.326952954 0.21594082  1.51408593 1.300040e-01 Low>High
## 34    1.0542291    0.198866735 0.33652563  0.59094083 5.545601e-01 Low>High
## 35    6.9441711    0.284262157 0.21470628  1.32395827 1.855169e-01 Low>High
## 36   36.8211521    0.073946194 0.12144566  0.60888296 5.426020e-01 Low>High
## 37    0.9388209    0.011903746 0.29361225  0.04054240 9.676607e-01 Low>High
## 38   87.7811267    0.122899550 0.08482064  1.44893456 1.473559e-01 Low>High
## 39   27.9872677    0.387238402 0.22244940  1.74079321 8.171983e-02 Low>High
## 40    2.8891780    0.052661478 0.23094135  0.22802966 8.196232e-01 Low>High
## 41    1.4688223    0.081793520 0.33105864  0.24706656 8.048567e-01 Low>High
## 42    1.4317578   -0.297796787 0.35473961 -0.83947993 4.012000e-01 High>Low
## 43  147.0483774   -0.123672515 0.09413029 -1.31384397 1.888987e-01 High>Low
## 44    2.8039987   -0.245347527 0.26271766 -0.93388290 3.503643e-01 High>Low
## 45  112.6769059   -0.227732505 0.10576580 -2.15317712 3.130476e-02 High>Low
## 46    1.6039609   -0.072807336 0.30018952 -0.24253790 8.083634e-01 High>Low
## 47    3.7020760    0.211498476 0.26221148  0.80659501 4.198998e-01 Low>High
## 48   40.8491501    0.029608426 0.13054357  0.22680877 8.205724e-01 Low>High
## 49    6.8375183    0.190373908 0.25465735  0.74756886 4.547203e-01 Low>High
## 50   41.0770621    0.049836178 0.09020014  0.55250668 5.806013e-01 Low>High
## 51   67.4763877   -0.024404805 0.16154874 -0.15106775 8.799223e-01 High>Low
## 52    0.4176611   -0.059240765 0.34792013 -0.17027116 8.647969e-01 High>Low
## 53    1.6627493   -0.291514512 0.36815710 -0.79182098 4.284651e-01 High>Low
## 54    1.0137786    0.091575797 0.36034499  0.25413368 7.993923e-01 Low>High
## 55    4.7083982    0.076619899 0.25126483  0.30493682 7.604143e-01 Low>High
## 56    8.7265107    0.042038771 0.14899837  0.28214249 7.778343e-01 Low>High
## 57   42.8338056    0.083340778 0.18858956  0.44191617 6.585499e-01 Low>High
## 58   25.1020535    0.157861664 0.19105107  0.82627991 4.086453e-01 Low>High
## 59   41.4593204    0.056706466 0.15912852  0.35635638 7.215737e-01 Low>High
## 60    3.8570393   -0.193787279 0.28137759 -0.68870901 4.910064e-01 High>Low
## 61    1.5796110   -0.276513823 0.37815694 -0.73121447 4.646482e-01 High>Low
## 62   74.0587622    0.042101486 0.10609652  0.39682249 6.914984e-01 Low>High
## 63    4.4957386   -0.146310390 0.22456747 -0.65152084 5.147103e-01 High>Low
## 64   26.8689347    0.237744080 0.20118932  1.18169333 2.373274e-01 Low>High
## 65  221.9840267    0.141789985 0.09034338  1.56945635 1.165416e-01 Low>High
## 66    5.0346400    0.124599091 0.23623651  0.52743368 5.978925e-01 Low>High
## 67    0.7371549   -0.107164195 0.21830122 -0.49090058 6.234968e-01 High>Low
## 68    8.7009758   -0.077958370 0.21673457 -0.35969513 7.190751e-01 High>Low
## 69   36.4394005   -0.011340530 0.11122345 -0.10196168 9.187871e-01 High>Low
## 70  407.9853210    0.148058801 0.11704458  1.26497787 2.058793e-01 Low>High
## 71    0.5266292    0.021623294 0.25436662  0.08500838 9.322547e-01 Low>High
## 72 5067.7924258   -0.077299184 0.06588541 -1.17323679 2.407009e-01 High>Low
## 73    6.1442803    0.102801114 0.18271638  0.56262671 5.736891e-01 Low>High
## 74    0.8420814   -0.485697145 0.30148860 -1.61099674 1.071804e-01 High>Low
## 75  927.1066406    0.056889736 0.08762788  0.64921962 5.161964e-01 Low>High
## 76  683.2517404    0.128403467 0.09030967  1.42181298 1.550805e-01 Low>High
## 77    2.4068360   -0.124284542 0.29288999 -0.42433864 6.713189e-01 High>Low
## 78   68.4201506    0.175494855 0.16812037  1.04386434 2.965482e-01 Low>High
## 79    2.2320032   -0.094027846 0.37451270 -0.25106718 8.017622e-01 High>Low
```

```
## 80    8.7736001   -0.223639953 0.14098288 -1.58629154 1.126732e-01 High>Low
## 81   99.8204227   -0.004590786 0.12109000 -0.03791218 9.697577e-01 High>Low
## 82 1329.0853342    0.190063843 0.13831096  1.37417779 1.693865e-01 Low>High
## 83    5.6824349    0.176069634 0.31749839  0.55455284 5.792006e-01 Low>High
## 84  257.8128378    0.109073981 0.07744592  1.40838899 1.590159e-01 Low>High
## 85   14.7982481   -0.071744846 0.12108732 -0.59250503 5.535125e-01 High>Low
## 86  275.5218474    0.205828360 0.15952147  1.29028627 1.969513e-01 Low>High
## 87 2905.9316825   -0.029274170 0.10663835 -0.27451822 7.836864e-01 High>Low
## 88    2.4746904   -1.293649778 0.43963390 -2.94256148 3.255091e-03 High>Low
## 89   36.0392035   -0.151555761 0.17805924 -0.85115357 3.946841e-01 High>Low
## 90    7.1137273   -0.555935018 0.24821549 -2.23972730 2.510863e-02 High>Low
## 91    6.9428465   -0.310513359 0.31327827 -0.99117425 3.216005e-01 High>Low
## 92    1.1003857   -0.046071366 0.39286041 -0.11727159 9.066448e-01 High>Low
## 93    0.9916119   -0.011223280 0.37804716 -0.02968751 9.763163e-01 High>Low
## 94   63.1946030    0.009444112 0.16864365  0.05600040 9.553415e-01 Low>High
## 95    8.2679474    0.303404841 0.20338283  1.49179181 1.357537e-01 Low>High
## 96    9.3689884    0.156330863 0.25805766  0.60579819 5.446488e-01 Low>High
## 97   33.1524239   -0.419046649 0.17166589 -2.44105945 1.464424e-02 High>Low
##        pval.adj                            Feature                     Method
## 1  9.148911e-01     Absconditabacteria_(SR1)_[G.1] DESeq2 man. geoMeans (ds2)
## 2  8.799738e-01                        Actinomyces DESeq2 man. geoMeans (ds2)
## 3  6.447007e-01                       Peptidiphaga DESeq2 man. geoMeans (ds2)
## 4  7.488610e-01                           Schaalia DESeq2 man. geoMeans (ds2)
## 5  5.910702e-01           Family.Actinomycetaceae. DESeq2 man. geoMeans (ds2)
## 6  5.910702e-01                             Rothia DESeq2 man. geoMeans (ds2)
## 7  5.771266e-01                      Alloscardovia DESeq2 man. geoMeans (ds2)
## 8  9.148911e-01                    Bifidobacterium DESeq2 man. geoMeans (ds2)
## 9  5.910702e-01                      Parascardovia DESeq2 man. geoMeans (ds2)
## 10 5.910702e-01                          Scardovia DESeq2 man. geoMeans (ds2)
## 11 8.799738e-01                    Corynebacterium DESeq2 man. geoMeans (ds2)
## 12 5.910702e-01                          Olsenella DESeq2 man. geoMeans (ds2)
## 13 9.763163e-01                          Atopobium DESeq2 man. geoMeans (ds2)
## 14 5.910702e-01                            Slackia DESeq2 man. geoMeans (ds2)
## 15 9.148911e-01                    Cryptobacterium DESeq2 man. geoMeans (ds2)
## 16 8.799738e-01                Bacteroidetes_[G.3] DESeq2 man. geoMeans (ds2)
## 17 6.077301e-01                Bacteroidetes_[G.5] DESeq2 man. geoMeans (ds2)
## 18 9.148911e-01        Family.Bacteroidetes_[F-1]. DESeq2 man. geoMeans (ds2)
## 19 5.925843e-01               Bacteroidaceae_[G.1] DESeq2 man. geoMeans (ds2)
## 20 7.313165e-01                Bacteroidales_[G.2] DESeq2 man. geoMeans (ds2)
## 21 9.148911e-01                      Porphyromonas DESeq2 man. geoMeans (ds2)
## 22 4.059229e-01                         Tannerella DESeq2 man. geoMeans (ds2)
## 23 9.148911e-01                     Alloprevotella DESeq2 man. geoMeans (ds2)
## 24 5.910702e-01                         Prevotella DESeq2 man. geoMeans (ds2)
## 25 5.910702e-01                         Bergeyella DESeq2 man. geoMeans (ds2)
## 26 8.940414e-01                      Capnocytophaga DESeq2 man. geoMeans (ds2)
## 27 5.910702e-01                            Gemella DESeq2 man. geoMeans (ds2)
## 28 5.910702e-01                        Abiotrophia DESeq2 man. geoMeans (ds2)
## 29 5.910702e-01                     Granulicatella DESeq2 man. geoMeans (ds2)
## 30 5.207834e-11                      Lactobacillus DESeq2 man. geoMeans (ds2)
## 31 3.738474e-01                      Streptococcus DESeq2 man. geoMeans (ds2)
## 32 8.481860e-01              Order.Lactobacillales. DESeq2 man. geoMeans (ds2)
## 33 5.910702e-01                     Class.Bacilli. DESeq2 man. geoMeans (ds2)
## 34 8.799738e-01          Clostridiales_[F.1][G-1] DESeq2 man. geoMeans (ds2)
## 35 5.910702e-01                       Butyrivibrio DESeq2 man. geoMeans (ds2)
```

```
## 36 8.799738e-01                          Catonella DESeq2 man. geoMeans (ds2)
## 37 9.763163e-01                         Johnsonella DESeq2 man. geoMeans (ds2)
## 38 5.910702e-01                 Lachnoanaerobaculum DESeq2 man. geoMeans (ds2)
## 39 5.910702e-01               Lachnospiraceae_[G.2] DESeq2 man. geoMeans (ds2)
## 40 9.148911e-01               Lachnospiraceae_[G.3] DESeq2 man. geoMeans (ds2)
## 41 9.148911e-01               Lachnospiraceae_[G.7] DESeq2 man. geoMeans (ds2)
## 42 8.481860e-01               Lachnospiraceae_[G.8] DESeq2 man. geoMeans (ds2)
## 43 5.910702e-01                        Oribacterium DESeq2 man. geoMeans (ds2)
## 44 7.903567e-01                      Shuttleworthia DESeq2 man. geoMeans (ds2)
## 45 4.337945e-01                      Stomatobaculum DESeq2 man. geoMeans (ds2)
## 46 9.148911e-01       Family.Lachnospiraceae_[XIV]. DESeq2 man. geoMeans (ds2)
## 47 8.481860e-01                         Peptococcus DESeq2 man. geoMeans (ds2)
## 48 9.148911e-01                         Parvimonas DESeq2 man. geoMeans (ds2)
## 49 8.799738e-01                         Filifactor DESeq2 man. geoMeans (ds2)
## 50 8.799738e-01                       Mogibacterium DESeq2 man. geoMeans (ds2)
## 51 9.590164e-01     Peptostreptococcaceae_[XI][G.1] DESeq2 man. geoMeans (ds2)
## 52 9.532420e-01     Peptostreptococcaceae_[XI][G.4] DESeq2 man. geoMeans (ds2)
## 53 8.481860e-01     Peptostreptococcaceae_[XI][G.5] DESeq2 man. geoMeans (ds2)
## 54 9.148911e-01     Peptostreptococcaceae_[XI][G.6] DESeq2 man. geoMeans (ds2)
## 55 9.148911e-01     Peptostreptococcaceae_[XI][G.7] DESeq2 man. geoMeans (ds2)
## 56 9.148911e-01     Peptostreptococcaceae_[XI][G.9] DESeq2 man. geoMeans (ds2)
## 57 9.148911e-01                 Peptostreptococcus DESeq2 man. geoMeans (ds2)
## 58 8.481860e-01               Ruminococcaceae_[G.1] DESeq2 man. geoMeans (ds2)
## 59 9.148911e-01               Ruminococcaceae_[G.2] DESeq2 man. geoMeans (ds2)
## 60 8.799738e-01                          Bulleidia DESeq2 man. geoMeans (ds2)
## 61 8.799738e-01                          Eggerthia DESeq2 man. geoMeans (ds2)
## 62 9.148911e-01                       Solobacterium DESeq2 man. geoMeans (ds2)
## 63 8.799738e-01                          Mycoplasma DESeq2 man. geoMeans (ds2)
## 64 6.310266e-01                         Mitsuokella DESeq2 man. geoMeans (ds2)
## 65 5.910702e-01                         Selenomonas DESeq2 man. geoMeans (ds2)
## 66 8.922395e-01            Family.Selenomonadaceae. DESeq2 man. geoMeans (ds2)
## 67 9.026744e-01                Class.Negativicutes. DESeq2 man. geoMeans (ds2)
## 68 9.148911e-01                        Anaeroglobus DESeq2 man. geoMeans (ds2)
## 69 9.763163e-01                          Dialister DESeq2 man. geoMeans (ds2)
## 70 5.925843e-01                         Megasphaera DESeq2 man. geoMeans (ds2)
## 71 9.763163e-01             Family.Veillonellaceae. DESeq2 man. geoMeans (ds2)
## 72 6.310266e-01                         Veillonella DESeq2 man. geoMeans (ds2)
## 73 8.799738e-01               Veillonellaceae_[G.1] DESeq2 man. geoMeans (ds2)
## 74 5.910702e-01                  Phylum.Firmicutes. DESeq2 man. geoMeans (ds2)
## 75 8.799738e-01                       Fusobacterium DESeq2 man. geoMeans (ds2)
## 76 5.910702e-01                        Leptotrichia DESeq2 man. geoMeans (ds2)
## 77 9.148911e-01        Gracilibacteria_(GN02)_[G.1] DESeq2 man. geoMeans (ds2)
## 78 7.313165e-01                          Lautropia DESeq2 man. geoMeans (ds2)
## 79 9.148911e-01                            Ottowia DESeq2 man. geoMeans (ds2)
## 80 5.910702e-01                          Eikenella DESeq2 man. geoMeans (ds2)
## 81 9.763163e-01                           Kingella DESeq2 man. geoMeans (ds2)
## 82 5.910702e-01                          Neisseria DESeq2 man. geoMeans (ds2)
## 83 8.799738e-01               Family.Neisseriaceae. DESeq2 man. geoMeans (ds2)
## 84 5.910702e-01                       Campylobacter DESeq2 man. geoMeans (ds2)
## 85 8.799738e-01                      Cardiobacterium DESeq2 man. geoMeans (ds2)
## 86 5.925843e-01                     Aggregatibacter DESeq2 man. geoMeans (ds2)
## 87 9.148911e-01                         Haemophilus DESeq2 man. geoMeans (ds2)
## 88 1.578719e-01             Family.Pasteurellaceae. DESeq2 man. geoMeans (ds2)
## 89 8.481860e-01        Saccharibacteria_(TM7)_[G.1] DESeq2 man. geoMeans (ds2)
```

```
## 90 4.059229e-01         Saccharibacteria_(TM7)_[G.3] DESeq2 man. geoMeans (ds2)
## 91 7.488610e-01         Saccharibacteria_(TM7)_[G.6] DESeq2 man. geoMeans (ds2)
## 92 9.763163e-01 Family.Saccharibacteria_(TM7)_[F-1]. DESeq2 man. geoMeans (ds2)
## 93 9.763163e-01         Saccharibacteria_(TM7)_[G.5] DESeq2 man. geoMeans (ds2)
## 94 9.763163e-01                            Treponema DESeq2 man. geoMeans (ds2)
## 95 5.910702e-01                       Fretibacterium DESeq2 man. geoMeans (ds2)
## 96 8.799738e-01                    Kingdom.Bacteria. DESeq2 man. geoMeans (ds2)
## 97 3.738474e-01                               Others DESeq2 man. geoMeans (ds2)
```

```
write.table(final, file="whratio_cat_ds2.txt", sep="\t", dec=",", row.names=F)
write.table(final[final$pval<0.05,], file="whratio_cat_ds2_sig.txt", sep="\t", dec=",", row.names=F)
final <- DA.kru(Microbio, predictor = Phe$whratio_cat)
final
```

```
##                                          pval  pval.adj
## Absconditabacteria_(SR1)_[G.1]       0.91532051 0.9650662
## Actinomyces                          0.97287620 0.9830103
## Peptidiphaga                         0.47343980 0.9296589
## Schaalia                             0.51500786 0.9296589
## Family.Actinomycetaceae.             0.36010932 0.9296589
## Rothia                               0.01516742 0.7153647
## Alloscardovia                        0.46432461 0.9296589
## Bifidobacterium                      0.71880844 0.9296589
## Parascardovia                        0.65303063 0.9296589
## Scardovia                            0.75698496 0.9509585
## Corynebacterium                      0.68064497 0.9296589
## Olsenella                            0.61640786 0.9296589
## Atopobium                            0.92742139 0.9673105
## Slackia                              0.02212468 0.7153647
## Cryptobacterium                      0.63107318 0.9296589
## Bacteroidetes_[G.3]                  0.57799014 0.9296589
## Bacteroidetes_[G.5]                  0.69875223 0.9296589
## Family.Bacteroidetes_[F-1].          0.08276303 0.8372974
## Bacteroidaceae_[G.1]                 0.18091604 0.8372974
## Bacteroidales_[G.2]                  0.54394641 0.9296589
## Porphyromonas                        0.71308454 0.9296589
## Tannerella                           0.13900389 0.8372974
## Alloprevotella                       0.45825107 0.9296589
## Prevotella                           0.39414271 0.9296589
## Bergeyella                           0.18247077 0.8372974
## Capnocytophaga                       0.45027702 0.9296589
## Gemella                              0.42659011 0.9296589
## Abiotrophia                          0.12121647 0.8372974
## Granulicatella                       0.35466011 0.9296589
## Lactobacillus                        0.20734379 0.8380145
## Streptococcus                        0.05422247 0.8372974
## Order.Lactobacillales.               0.16126805 0.8372974
## Class.Bacilli.                       0.15923398 0.8372974
## Clostridiales_[F.1][G-1]             0.81463198 0.9509585
## Butyrivibrio                         0.77822780 0.9509585
## Catonella                            0.96734762 0.9830103
## Johnsonella                          0.44830910 0.9296589
## Lachnoanaerobaculum                  0.53180950 0.9296589
## Lachnospiraceae_[G.2]                0.02066171 0.7153647
```

```
## Lachnospiraceae_[G.3]                0.40650963 0.9296589
## Lachnospiraceae_[G.7]                0.18103394 0.8372974
## Lachnospiraceae_[G.8]                0.62331196 0.9296589
## Oribacterium                         0.21739360 0.8434872
## Shuttleworthia                       0.90369965 0.9650662
## Stomatobaculum                       0.17209362 0.8372974
## Family.Lachnospiraceae_[XIV].        0.19813582 0.8372974
## Peptococcus                          0.16390457 0.8372974
## Parvimonas                           0.82818923 0.9509585
## Filifactor                           0.19853443 0.8372974
## Mogibacterium                        0.44763700 0.9296589
## Peptostreptococcaceae_[XI][G.1]      0.78579353 0.9509585
## Peptostreptococcaceae_[XI][G.4]      0.91131974 0.9650662
## Peptostreptococcaceae_[XI][G.5]      0.59654314 0.9296589
## Peptostreptococcaceae_[XI][G.6]      0.34264723 0.9296589
## Peptostreptococcaceae_[XI][G.7]      0.71418479 0.9296589
## Peptostreptococcaceae_[XI][G.9]      0.47572997 0.9296589
## Peptostreptococcus                   0.43565090 0.9296589
## Ruminococcaceae_[G.1]                0.65332458 0.9296589
## Ruminococcaceae_[G.2]                0.59893280 0.9296589
## Bulleidia                            0.85790699 0.9565170
## Eggerthia                            0.41581945 0.9296589
## Solobacterium                        0.65908433 0.9296589
## Mycoplasma                           0.36424538 0.9296589
## Mitsuokella                          0.53924334 0.9296589
## Selenomonas                          0.05929269 0.8372974
## Family.Selenomonadaceae.             0.44748053 0.9296589
## Class.Negativicutes.                 0.68421870 0.9296589
## Anaeroglobus                         0.70191929 0.9296589
## Dialister                            0.98693843 0.9869384
## Megasphaera                          0.73716923 0.9408607
## Family.Veillonellaceae.              0.89253764 0.9650662
## Veillonella                          0.17851037 0.8372974
## Veillonellaceae_[G.1]                0.76518595 0.9509585
## Phylum.Firmicutes.                   0.49882461 0.9296589
## Fusobacterium                        0.36790640 0.9296589
## Leptotrichia                         0.23851449 0.8898425
## Gracilibacteria_(GN02)_[G.1]         0.88910971 0.9650662
## Lautropia                            0.71760592 0.9296589
## Ottowia                              0.31264502 0.9296589
## Eikenella                            0.59081546 0.9296589
## Kingella                             0.49768478 0.9296589
## Neisseria                            0.10319901 0.8372974
## Family.Neisseriaceae.                0.68754054 0.9296589
## Campylobacter                        0.08179631 0.8372974
## Cardiobacterium                      0.83043780 0.9509585
## Aggregatibacter                      0.17967851 0.8372974
## Haemophilus                          0.84261464 0.9509585
## Family.Pasteurellaceae.              0.28702047 0.9296589
## Saccharibacteria_(TM7)_[G.1]         0.53217437 0.9296589
## Saccharibacteria_(TM7)_[G.3]         0.09984536 0.8372974
## Saccharibacteria_(TM7)_[G.6]         0.11835500 0.8372974
## Family.Saccharibacteria_(TM7)_[F-1]. 0.84311781 0.9509585
## Saccharibacteria_(TM7)_[G.5]         0.46684023 0.9296589
```

```
## Treponema                              0.40978187 0.9296589
## Fretibacterium                         0.81698587 0.9509585
## Kingdom.Bacteria.                      0.26895195 0.9296589
## Others                                 0.95714853 0.9830103
##                                                                  Feature
## Absconditabacteria_(SR1)_[G.1]             Absconditabacteria_(SR1)_[G.1]
## Actinomyces                                                  Actinomyces
## Peptidiphaga                                                Peptidiphaga
## Schaalia                                                        Schaalia
## Family.Actinomycetaceae.                         Family.Actinomycetaceae.
## Rothia                                                            Rothia
## Alloscardovia                                              Alloscardovia
## Bifidobacterium                                          Bifidobacterium
## Parascardovia                                              Parascardovia
## Scardovia                                                      Scardovia
## Corynebacterium                                          Corynebacterium
## Olsenella                                                      Olsenella
## Atopobium                                                      Atopobium
## Slackia                                                          Slackia
## Cryptobacterium                                          Cryptobacterium
## Bacteroidetes_[G.3]                                  Bacteroidetes_[G.3]
## Bacteroidetes_[G.5]                                  Bacteroidetes_[G.5]
## Family.Bacteroidetes_[F-1].                   Family.Bacteroidetes_[F-1].
## Bacteroidaceae_[G.1]                                Bacteroidaceae_[G.1]
## Bacteroidales_[G.2]                                  Bacteroidales_[G.2]
## Porphyromonas                                              Porphyromonas
## Tannerella                                                    Tannerella
## Alloprevotella                                            Alloprevotella
## Prevotella                                                    Prevotella
## Bergeyella                                                    Bergeyella
## Capnocytophaga                                            Capnocytophaga
## Gemella                                                          Gemella
## Abiotrophia                                                  Abiotrophia
## Granulicatella                                            Granulicatella
## Lactobacillus                                              Lactobacillus
## Streptococcus                                              Streptococcus
## Order.Lactobacillales.                             Order.Lactobacillales.
## Class.Bacilli.                                             Class.Bacilli.
## Clostridiales_[F.1][G-1]                         Clostridiales_[F.1][G-1]
## Butyrivibrio                                                Butyrivibrio
## Catonella                                                      Catonella
## Johnsonella                                                  Johnsonella
## Lachnoanaerobaculum                                  Lachnoanaerobaculum
## Lachnospiraceae_[G.2]                              Lachnospiraceae_[G.2]
## Lachnospiraceae_[G.3]                              Lachnospiraceae_[G.3]
## Lachnospiraceae_[G.7]                              Lachnospiraceae_[G.7]
## Lachnospiraceae_[G.8]                              Lachnospiraceae_[G.8]
## Oribacterium                                                Oribacterium
## Shuttleworthia                                            Shuttleworthia
## Stomatobaculum                                            Stomatobaculum
## Family.Lachnospiraceae_[XIV].                 Family.Lachnospiraceae_[XIV].
## Peptococcus                                                  Peptococcus
## Parvimonas                                                    Parvimonas
## Filifactor                                                    Filifactor
```

```
## Mogibacterium                                               Mogibacterium
## Peptostreptococcaceae_[XI][G.1]          Peptostreptococcaceae_[XI][G.1]
## Peptostreptococcaceae_[XI][G.4]          Peptostreptococcaceae_[XI][G.4]
## Peptostreptococcaceae_[XI][G.5]          Peptostreptococcaceae_[XI][G.5]
## Peptostreptococcaceae_[XI][G.6]          Peptostreptococcaceae_[XI][G.6]
## Peptostreptococcaceae_[XI][G.7]          Peptostreptococcaceae_[XI][G.7]
## Peptostreptococcaceae_[XI][G.9]          Peptostreptococcaceae_[XI][G.9]
## Peptostreptococcus                                     Peptostreptococcus
## Ruminococcaceae_[G.1]                               Ruminococcaceae_[G.1]
## Ruminococcaceae_[G.2]                               Ruminococcaceae_[G.2]
## Bulleidia                                                       Bulleidia
## Eggerthia                                                       Eggerthia
## Solobacterium                                               Solobacterium
## Mycoplasma                                                     Mycoplasma
## Mitsuokella                                                   Mitsuokella
## Selenomonas                                                   Selenomonas
## Family.Selenomonadaceae.                         Family.Selenomonadaceae.
## Class.Negativicutes.                                 Class.Negativicutes.
## Anaeroglobus                                                 Anaeroglobus
## Dialister                                                       Dialister
## Megasphaera                                                   Megasphaera
## Family.Veillonellaceae.                           Family.Veillonellaceae.
## Veillonella                                                   Veillonella
## Veillonellaceae_[G.1]                               Veillonellaceae_[G.1]
## Phylum.Firmicutes.                                     Phylum.Firmicutes.
## Fusobacterium                                               Fusobacterium
## Leptotrichia                                                 Leptotrichia
## Gracilibacteria_(GN02)_[G.1]                 Gracilibacteria_(GN02)_[G.1]
## Lautropia                                                       Lautropia
## Ottowia                                                           Ottowia
## Eikenella                                                       Eikenella
## Kingella                                                         Kingella
## Neisseria                                                       Neisseria
## Family.Neisseriaceae.                               Family.Neisseriaceae.
## Campylobacter                                               Campylobacter
## Cardiobacterium                                           Cardiobacterium
## Aggregatibacter                                           Aggregatibacter
## Haemophilus                                                   Haemophilus
## Family.Pasteurellaceae.                           Family.Pasteurellaceae.
## Saccharibacteria_(TM7)_[G.1]                 Saccharibacteria_(TM7)_[G.1]
## Saccharibacteria_(TM7)_[G.3]                 Saccharibacteria_(TM7)_[G.3]
## Saccharibacteria_(TM7)_[G.6]                 Saccharibacteria_(TM7)_[G.6]
## Family.Saccharibacteria_(TM7)_[F-1]. Family.Saccharibacteria_(TM7)_[F-1].
## Saccharibacteria_(TM7)_[G.5]                 Saccharibacteria_(TM7)_[G.5]
## Treponema                                                       Treponema
## Fretibacterium                                             Fretibacterium
## Kingdom.Bacteria.                                       Kingdom.Bacteria.
## Others                                                             Others
##                                                     Method
## Absconditabacteria_(SR1)_[G.1]       Kruskal-Wallis (kru)
## Actinomyces                          Kruskal-Wallis (kru)
## Peptidiphaga                         Kruskal-Wallis (kru)
## Schaalia                             Kruskal-Wallis (kru)
## Family.Actinomycetaceae.             Kruskal-Wallis (kru)
```

```
## Rothia                                  Kruskal-Wallis (kru)
## Alloscardovia                           Kruskal-Wallis (kru)
## Bifidobacterium                         Kruskal-Wallis (kru)
## Parascardovia                           Kruskal-Wallis (kru)
## Scardovia                               Kruskal-Wallis (kru)
## Corynebacterium                         Kruskal-Wallis (kru)
## Olsenella                               Kruskal-Wallis (kru)
## Atopobium                               Kruskal-Wallis (kru)
## Slackia                                 Kruskal-Wallis (kru)
## Cryptobacterium                         Kruskal-Wallis (kru)
## Bacteroidetes_[G.3]                     Kruskal-Wallis (kru)
## Bacteroidetes_[G.5]                     Kruskal-Wallis (kru)
## Family.Bacteroidetes_[F-1].             Kruskal-Wallis (kru)
## Bacteroidaceae_[G.1]                    Kruskal-Wallis (kru)
## Bacteroidales_[G.2]                     Kruskal-Wallis (kru)
## Porphyromonas                           Kruskal-Wallis (kru)
## Tannerella                              Kruskal-Wallis (kru)
## Alloprevotella                          Kruskal-Wallis (kru)
## Prevotella                              Kruskal-Wallis (kru)
## Bergeyella                              Kruskal-Wallis (kru)
## Capnocytophaga                          Kruskal-Wallis (kru)
## Gemella                                 Kruskal-Wallis (kru)
## Abiotrophia                             Kruskal-Wallis (kru)
## Granulicatella                          Kruskal-Wallis (kru)
## Lactobacillus                           Kruskal-Wallis (kru)
## Streptococcus                           Kruskal-Wallis (kru)
## Order.Lactobacillales.                  Kruskal-Wallis (kru)
## Class.Bacilli.                          Kruskal-Wallis (kru)
## Clostridiales_[F.1][G-1]                Kruskal-Wallis (kru)
## Butyrivibrio                            Kruskal-Wallis (kru)
## Catonella                               Kruskal-Wallis (kru)
## Johnsonella                             Kruskal-Wallis (kru)
## Lachnoanaerobaculum                     Kruskal-Wallis (kru)
## Lachnospiraceae_[G.2]                   Kruskal-Wallis (kru)
## Lachnospiraceae_[G.3]                   Kruskal-Wallis (kru)
## Lachnospiraceae_[G.7]                   Kruskal-Wallis (kru)
## Lachnospiraceae_[G.8]                   Kruskal-Wallis (kru)
## Oribacterium                            Kruskal-Wallis (kru)
## Shuttleworthia                          Kruskal-Wallis (kru)
## Stomatobaculum                          Kruskal-Wallis (kru)
## Family.Lachnospiraceae_[XIV].           Kruskal-Wallis (kru)
## Peptococcus                             Kruskal-Wallis (kru)
## Parvimonas                              Kruskal-Wallis (kru)
## Filifactor                              Kruskal-Wallis (kru)
## Mogibacterium                           Kruskal-Wallis (kru)
## Peptostreptococcaceae_[XI][G.1]         Kruskal-Wallis (kru)
## Peptostreptococcaceae_[XI][G.4]         Kruskal-Wallis (kru)
## Peptostreptococcaceae_[XI][G.5]         Kruskal-Wallis (kru)
## Peptostreptococcaceae_[XI][G.6]         Kruskal-Wallis (kru)
## Peptostreptococcaceae_[XI][G.7]         Kruskal-Wallis (kru)
## Peptostreptococcaceae_[XI][G.9]         Kruskal-Wallis (kru)
## Peptostreptococcus                      Kruskal-Wallis (kru)
## Ruminococcaceae_[G.1]                   Kruskal-Wallis (kru)
## Ruminococcaceae_[G.2]                   Kruskal-Wallis (kru)
```

```
## Bulleidia                            Kruskal-Wallis (kru)
## Eggerthia                            Kruskal-Wallis (kru)
## Solobacterium                        Kruskal-Wallis (kru)
## Mycoplasma                           Kruskal-Wallis (kru)
## Mitsuokella                          Kruskal-Wallis (kru)
## Selenomonas                          Kruskal-Wallis (kru)
## Family.Selenomonadaceae.             Kruskal-Wallis (kru)
## Class.Negativicutes.                 Kruskal-Wallis (kru)
## Anaeroglobus                         Kruskal-Wallis (kru)
## Dialister                            Kruskal-Wallis (kru)
## Megasphaera                          Kruskal-Wallis (kru)
## Family.Veillonellaceae.              Kruskal-Wallis (kru)
## Veillonella                          Kruskal-Wallis (kru)
## Veillonellaceae_[G.1]                Kruskal-Wallis (kru)
## Phylum.Firmicutes.                   Kruskal-Wallis (kru)
## Fusobacterium                        Kruskal-Wallis (kru)
## Leptotrichia                         Kruskal-Wallis (kru)
## Gracilibacteria_(GN02)_[G.1]         Kruskal-Wallis (kru)
## Lautropia                            Kruskal-Wallis (kru)
## Ottowia                              Kruskal-Wallis (kru)
## Eikenella                            Kruskal-Wallis (kru)
## Kingella                             Kruskal-Wallis (kru)
## Neisseria                            Kruskal-Wallis (kru)
## Family.Neisseriaceae.                Kruskal-Wallis (kru)
## Campylobacter                        Kruskal-Wallis (kru)
## Cardiobacterium                      Kruskal-Wallis (kru)
## Aggregatibacter                      Kruskal-Wallis (kru)
## Haemophilus                          Kruskal-Wallis (kru)
## Family.Pasteurellaceae.              Kruskal-Wallis (kru)
## Saccharibacteria_(TM7)_[G.1]         Kruskal-Wallis (kru)
## Saccharibacteria_(TM7)_[G.3]         Kruskal-Wallis (kru)
## Saccharibacteria_(TM7)_[G.6]         Kruskal-Wallis (kru)
## Family.Saccharibacteria_(TM7)_[F-1]. Kruskal-Wallis (kru)
## Saccharibacteria_(TM7)_[G.5]         Kruskal-Wallis (kru)
## Treponema                            Kruskal-Wallis (kru)
## Fretibacterium                       Kruskal-Wallis (kru)
## Kingdom.Bacteria.                    Kruskal-Wallis (kru)
## Others                               Kruskal-Wallis (kru)
```

```
write.table(final, file="whratio_cat_kru.txt", sep="\t", dec=",", row.names=F)
write.table(final[final$pval<0.05,], file="whratio_cat_kru_sig.txt", sep="\t", dec=",", row.names=F)
#################Alcprweek_cat
table(Phe$Alcprweek_cat, useNA="always")
```

```
##
## Abstinence   Moderate       High       <NA>
##         79        302        262        103
```

```
#Remove NA
Phe2<-Phe[complete.cases(Phe$Alcprweek_cat),]
#Also subset columns
Microbio2<-dplyr::select(Microbio, one_of(Phe2$IDX))
final <- DA.ds2(Microbio2, predictor = Phe2$Alcprweek_cat, out.all=TRUE) #It is LRT so don't look at or
final
```

```
##                                       baseMean log2FoldChange      lfcSE
## Absconditabacteria_(SR1)_[G.1]      67.4525953    0.306290441 0.42601445
## Actinomyces                        320.6243769   -0.260123892 0.14214435
## Peptidiphaga                        21.8788508    0.096638508 0.20411461
## Schaalia                           718.2385650   -0.005310581 0.11829535
## Family.Actinomycetaceae.             5.4245465   -0.228488378 0.29408868
## Rothia                            2115.3596729   -1.145221591 0.14724984
## Alloscardovia                       11.6020875   -0.732848904 0.35574130
## Bifidobacterium                     12.7930534   -1.017315918 0.40976134
## Parascardovia                        2.7228811   -0.235779451 0.54706343
## Scardovia                           12.6265928    0.565276436 0.35181165
## Corynebacterium                     93.2795594   -0.378002828 0.15274279
## Olsenella                            1.5187686    0.342781753 0.34474699
## Atopobium                          120.9405608   -0.051689925 0.13985169
## Slackia                              1.3750812    0.056820121 0.32892272
## Cryptobacterium                      4.3752325   -0.796427519 0.34244951
## Bacteroidetes_[G.3]                  6.6714509   -0.154764806 0.36404500
## Bacteroidetes_[G.5]                  4.5435785   -0.041787578 0.39424231
## Family.Bacteroidetes_[F-1].          0.9078927    0.111324321 0.57638877
## Bacteroidaceae_[G.1]                 0.7290243    0.420284139 0.56029127
## Bacteroidales_[G.2]                 12.1112184   -0.085042258 0.21212143
## Porphyromonas                      681.9271472    0.087197739 0.19184215
## Tannerella                          22.2027084   -0.049324792 0.14377240
## Alloprevotella                     368.5066460    0.477599275 0.15165276
## Prevotella                        3797.6491678    0.108586863 0.09656463
## Bergeyella                          71.0186814   -0.144071417 0.14535873
## Capnocytophaga                     220.4029252   -0.229207690 0.15781812
## Gemella                            304.0674457   -0.239855068 0.13047048
## Abiotrophia                         29.5020730    0.165490658 0.28639859
## Granulicatella                     305.2408448   -0.316230339 0.11495194
## Lactobacillus                       26.5895530   -2.688609811 0.42739935
## Streptococcus                     3749.4451834   -0.653859545 0.10200338
## Order.Lactobacillales.               0.2770175    0.199008496 0.40503286
## Class.Bacilli.                       0.5750674   -0.420972921 0.29771678
## Clostridiales_[F.1][G-1]             1.0490462   -0.300130127 0.46978399
## Butyrivibrio                         6.8355094    0.228199745 0.30114695
## Catonella                           37.1962299    0.393978887 0.16955878
## Johnsonella                          0.9592888   -0.032110206 0.40984627
## Lachnoanaerobaculum                 89.1665564    0.150233056 0.12002340
## Lachnospiraceae_[G.2]               27.5035917    0.398240289 0.31765139
## Lachnospiraceae_[G.3]                2.9341877   -0.188986371 0.32477771
## Lachnospiraceae_[G.7]                1.4439746   -0.237294480 0.45916827
## Lachnospiraceae_[G.8]                1.4202629    0.317871594 0.50281409
## Oribacterium                       149.2674133    0.109653323 0.13269049
## Shuttleworthia                       2.7926002   -0.252976826 0.37570701
## Stomatobaculum                     113.8629752    0.014213779 0.14944178
## Family.Lachnospiraceae_[XIV].        1.4913692    0.269835073 0.42282548
## Peptococcus                          3.7832046    0.263056058 0.36222715
## Parvimonas                          41.2315278    0.460624698 0.18514982
## Filifactor                           6.7236878    0.258489253 0.35860030
## Mogibacterium                       41.2918319    0.087823581 0.12653623
## Peptostreptococcaceae_[XI][G.1]     68.8423415    0.364554673 0.22680022
## Peptostreptococcaceae_[XI][G.4]      0.4069864   -0.210378117 0.49712246
## Peptostreptococcaceae_[XI][G.5]      1.6753842    0.154885765 0.50838204
```

```
## Peptostreptococcaceae_[XI][G.6]         0.9974995    0.432219173 0.52280954
## Peptostreptococcaceae_[XI][G.7]         4.7845292    1.138571755 0.35678992
## Peptostreptococcaceae_[XI][G.9]         8.7120111    0.215201286 0.21256865
## Peptostreptococcus                     41.8868129    0.391070354 0.26374406
## Ruminococcaceae_[G.1]                  24.2574062    0.648540209 0.26461528
## Ruminococcaceae_[G.2]                  41.3219120    0.395822110 0.22344350
## Bulleidia                               3.8831840   -0.023488720 0.39181329
## Eggerthia                               1.4274281    0.678689734 0.53592051
## Solobacterium                          75.7476573    0.262436190 0.15100667
## Mycoplasma                              4.6442885    0.194944193 0.32081718
## Mitsuokella                            26.0116728   -0.067728154 0.28092005
## Selenomonas                           219.7749831    0.051099844 0.12762405
## Family.Selenomonadaceae.                4.8512044    1.090505626 0.33755583
## Class.Negativicutes.                    0.7356692    0.179408159 0.31347578
## Anaeroglobus                            7.2188584    0.726324486 0.29136787
## Dialister                              36.1011507   -0.001412040 0.15649409
## Megasphaera                           412.9464681    0.135063334 0.16243778
## Family.Veillonellaceae.                 0.5150875    0.191054175 0.36925571
## Veillonella                          5045.2472165   -0.177439420 0.09268026
## Veillonellaceae_[G.1]                   6.2982730   -0.049746289 0.25757923
## Phylum.Firmicutes.                      0.8735652    1.077390100 0.43987864
## Fusobacterium                         930.5323286    0.187323782 0.12283273
## Leptotrichia                          678.5709542   -0.240568825 0.12746853
## Gracilibacteria_(GN02)_[G.1]            2.3012085   -0.216454416 0.40801055
## Lautropia                              69.8161607    0.186123825 0.22840837
## Ottowia                                 2.0378315    0.247834622 0.53846619
## Eikenella                               8.6699490   -0.127499920 0.19623634
## Kingella                               96.5505729    0.314968460 0.16606022
## Neisseria                            1316.0166063   -0.071240848 0.18973126
## Family.Neisseriaceae.                   5.4775195    0.208944146 0.44182425
## Campylobacter                         259.1326399   -0.006335512 0.10900371
## Cardiobacterium                        14.6134974   -0.228805821 0.17066884
## Aggregatibacter                       273.6996566   -0.006240690 0.22240131
## Haemophilus                          2937.0569321   -0.035030300 0.15189985
## Family.Pasteurellaceae.                 2.2862692    0.161739367 0.60137237
## Saccharibacteria_(TM7)_[G.1]           37.0382821    0.006534423 0.25056752
## Saccharibacteria_(TM7)_[G.3]            7.1725741   -0.035675477 0.34063405
## Saccharibacteria_(TM7)_[G.6]            7.1449440   -0.427223568 0.44382923
## Family.Saccharibacteria_(TM7)_[F-1].    1.1504620   -0.369229267 0.55569314
## Saccharibacteria_(TM7)_[G.5]            0.9126204    0.390498675 0.52379393
## Treponema                              64.3874725    0.311753081 0.23757578
## Fretibacterium                          8.3464247   -0.144390539 0.28875664
## Kingdom.Bacteria.                       9.2061485    1.011485413 0.36448564
## Others                                 32.7487451   -0.090802746 0.23974225
##                                             stat         pval
## Absconditabacteria_(SR1)_[G.1]         3.4035271 1.823616e-01
## Actinomyces                            7.2363204 2.683200e-02
## Peptidiphaga                           2.7182112 2.568904e-01
## Schaalia                               3.3190251 1.902317e-01
## Family.Actinomycetaceae.               2.2881029 3.185259e-01
## Rothia                                71.4748315 3.016043e-16
## Alloscardovia                          4.6823198 9.621597e-02
## Bifidobacterium                       10.8720985 4.356661e-03
## Parascardovia                          3.9984155 1.354425e-01
```

```
## Scardovia                              3.0070835 2.223413e-01
## Corynebacterium                        8.1526726 1.696952e-02
## Olsenella                              3.2559887 1.963229e-01
## Atopobium                              6.4748525 3.926482e-02
## Slackia                                5.5023753 6.385198e-02
## Cryptobacterium                       12.6001359 1.836180e-03
## Bacteroidetes_[G.3]                    0.5826097 7.472878e-01
## Bacteroidetes_[G.5]                    0.1230045 9.403508e-01
## Family.Bacteroidetes_[F-1].            1.0163686 6.015869e-01
## Bacteroidaceae_[G.1]                   0.9011669 6.372562e-01
## Bacteroidales_[G.2]                    1.8386748 3.987832e-01
## Porphyromonas                          0.2251324 8.935382e-01
## Tannerella                             0.3083477 8.571230e-01
## Alloprevotella                         9.5295202 8.524933e-03
## Prevotella                             1.8580822 3.949322e-01
## Bergeyella                             2.7930750 2.474523e-01
## Capnocytophaga                         8.3130064 1.566223e-02
## Gemella                                4.9911048 8.245089e-02
## Abiotrophia                            1.0877298 5.805003e-01
## Granulicatella                         8.5643667 1.381247e-02
## Lactobacillus                         67.5326053 2.165116e-15
## Streptococcus                         45.5820511 1.264688e-10
## Order.Lactobacillales.                -2.9194359 1.000000e+00
## Class.Bacilli.                         6.7293471 3.457330e-02
## Clostridiales_[F.1][G-1]               0.5029888 7.776378e-01
## Butyrivibrio                           1.8098933 4.045635e-01
## Catonella                              6.3125313 4.258447e-02
## Johnsonella                            0.9918748 6.089997e-01
## Lachnoanaerobaculum                    1.5614189 4.580809e-01
## Lachnospiraceae_[G.2]                  2.0009094 3.677122e-01
## Lachnospiraceae_[G.3]                  5.2303313 7.315567e-02
## Lachnospiraceae_[G.7]                  6.0125334 4.947604e-02
## Lachnospiraceae_[G.8]                  3.4138476 1.814230e-01
## Oribacterium                           0.6916497 7.076364e-01
## Shuttleworthia                         4.2314185 1.205478e-01
## Stomatobaculum                         2.5665640 2.771263e-01
## Family.Lachnospiraceae_[XIV].          1.5029469 4.716711e-01
## Peptococcus                            1.0578126 5.892491e-01
## Parvimonas                             8.1950376 1.661385e-02
## Filifactor                             4.9024398 8.618838e-02
## Mogibacterium                          1.3305062 5.141434e-01
## Peptostreptococcaceae_[XI][G.1]        5.1660123 7.554656e-02
## Peptostreptococcaceae_[XI][G.4]        1.9740310 3.726873e-01
## Peptostreptococcaceae_[XI][G.5]        0.3987080 8.192598e-01
## Peptostreptococcaceae_[XI][G.6]        3.0615522 2.163677e-01
## Peptostreptococcaceae_[XI][G.7]       14.4226234 7.381882e-04
## Peptostreptococcaceae_[XI][G.9]        1.6681705 4.342715e-01
## Peptostreptococcus                     5.3322247 6.952198e-02
## Ruminococcaceae_[G.1]                  5.6890646 5.816146e-02
## Ruminococcaceae_[G.2]                  4.8183905 8.988760e-02
## Bulleidia                              0.3703087 8.309760e-01
## Eggerthia                              5.9741778 5.043404e-02
## Solobacterium                          2.9752742 2.259058e-01
## Mycoplasma                             0.4240159 8.089583e-01
```

```
## Mitsuokella                           10.8347843 4.438707e-03
## Selenomonas                            0.8669229 6.482613e-01
## Family.Selenomonadaceae.              11.0902585 3.906438e-03
## Class.Negativicutes.                   0.4918721 7.819722e-01
## Anaeroglobus                           5.9653944 5.065602e-02
## Dialister                              1.5001134 4.723398e-01
## Megasphaera                           12.1762860 2.269620e-03
## Family.Veillonellaceae.                1.7459812 4.177005e-01
## Veillonella                            4.3352347 1.144500e-01
## Veillonellaceae_[G.1]                  0.1678313 9.195088e-01
## Phylum.Firmicutes.                     6.7513702 3.419468e-02
## Fusobacterium                          3.1501326 2.069938e-01
## Leptotrichia                           4.1615685 1.248323e-01
## Gracilibacteria_(GN02)_[G.1]           7.8588681 1.965479e-02
## Lautropia                              9.0254016 1.096880e-02
## Ottowia                                0.2381862 8.877252e-01
## Eikenella                              0.5896517 7.446613e-01
## Kingella                               3.4578159 1.774781e-01
## Neisseria                              2.2067671 3.317467e-01
## Family.Neisseriaceae.                  0.6383266 7.267568e-01
## Campylobacter                          1.3552081 5.078323e-01
## Cardiobacterium                        2.0486652 3.590360e-01
## Aggregatibacter                        0.8665333 6.483876e-01
## Haemophilus                            1.7930059 4.079940e-01
## Family.Pasteurellaceae.                3.1319310 2.088862e-01
## Saccharibacteria_(TM7)_[G.1]           0.3350715 8.457464e-01
## Saccharibacteria_(TM7)_[G.3]           0.3737460 8.295491e-01
## Saccharibacteria_(TM7)_[G.6]           8.4798501 1.440867e-02
## Family.Saccharibacteria_(TM7)_[F-1].  1.8832731 3.899891e-01
## Saccharibacteria_(TM7)_[G.5]           0.8647339 6.489712e-01
## Treponema                              3.4995245 1.738153e-01
## Fretibacterium                         7.0619670 2.927611e-02
## Kingdom.Bacteria.                      6.8185073 3.306587e-02
## Others                                 1.4183618 4.920471e-01
##                                                  ordering     pval.adj
## Absconditabacteria_(SR1)_[G.1]       Moderate>Abstinence 4.020245e-01
## Actinomyces                          Abstinence>Moderate 1.445946e-01
## Peptidiphaga                         Moderate>Abstinence 4.701580e-01
## Schaalia                             Abstinence>Moderate 4.100550e-01
## Family.Actinomycetaceae.             Abstinence>Moderate 5.617639e-01
## Rothia                               Abstinence>Moderate 2.925562e-14
## Alloscardovia                        Abstinence>Moderate 2.592486e-01
## Bifidobacterium                      Abstinence>Moderate 4.783940e-02
## Parascardovia                        Abstinence>Moderate 3.284482e-01
## Scardovia                            Moderate>Abstinence 4.296640e-01
## Corynebacterium                      Abstinence>Moderate 1.028777e-01
## Olsenella                            Moderate>Abstinence 4.139853e-01
## Atopobium                            Abstinence>Moderate 1.655951e-01
## Slackia                              Moderate>Abstinence 2.135739e-01
## Cryptobacterium                      Abstinence>Moderate 3.562189e-02
## Bacteroidetes_[G.3]                  Abstinence>Moderate 8.629395e-01
## Bacteroidetes_[G.5]                  Abstinence>Moderate 9.501461e-01
## Family.Bacteroidetes_[F-1].          Moderate>Abstinence 7.772760e-01
## Bacteroidaceae_[G.1]                 Moderate>Abstinence 7.868776e-01
```

```
## Bacteroidales_[G.2]                    Abstinence>Moderate 6.183658e-01
## Porphyromonas                          Moderate>Abstinence 9.220554e-01
## Tannerella                             Abstinence>Moderate 9.037058e-01
## Alloprevotella                         Moderate>Abstinence 8.269185e-02
## Prevotella                             Moderate>Abstinence 6.183658e-01
## Bergeyella                             Abstinence>Moderate 4.615937e-01
## Capnocytophaga                         Abstinence>Moderate 1.028777e-01
## Gemella                                Abstinence>Moderate 2.423557e-01
## Abiotrophia                            Moderate>Abstinence 7.713497e-01
## Granulicatella                         Abstinence>Moderate 1.028777e-01
## Lactobacillus                          Abstinence>Moderate 1.050081e-13
## Streptococcus                          Abstinence>Moderate 4.089158e-09
## Order.Lactobacillales.                 Moderate>Abstinence 1.000000e+00
## Class.Bacilli.                         Abstinence>Moderate 1.524368e-01
## Clostridiales_[F.1][G-1]               Abstinence>Moderate 8.819919e-01
## Butyrivibrio                           Moderate>Abstinence 6.183658e-01
## Catonella                              Moderate>Abstinence 1.721122e-01
## Johnsonella                            Abstinence>Moderate 7.772760e-01
## Lachnoanaerobaculum                    Moderate>Abstinence 6.631918e-01
## Lachnospiraceae_[G.2]                  Moderate>Abstinence 6.127232e-01
## Lachnospiraceae_[G.3]                  Abstinence>Moderate 2.289065e-01
## Lachnospiraceae_[G.7]                  Abstinence>Moderate 1.819864e-01
## Lachnospiraceae_[G.8]                  Moderate>Abstinence 4.020245e-01
## Oribacterium                           Moderate>Abstinence 8.474165e-01
## Shuttleworthia                         Abstinence>Moderate 3.077140e-01
## Stomatobaculum                         Moderate>Abstinence 4.978009e-01
## Family.Lachnospiraceae_[XIV].          Moderate>Abstinence 6.640139e-01
## Peptococcus                            Moderate>Abstinence 7.723941e-01
## Parvimonas                             Moderate>Abstinence 1.028777e-01
## Filifactor                             Moderate>Abstinence 2.458904e-01
## Mogibacterium                          Moderate>Abstinence 6.926654e-01
## Peptostreptococcaceae_[XI][G.1]        Moderate>Abstinence 2.290005e-01
## Peptostreptococcaceae_[XI][G.4]        Abstinence>Moderate 6.127232e-01
## Peptostreptococcaceae_[XI][G.5]        Moderate>Abstinence 8.956075e-01
## Peptostreptococcaceae_[XI][G.6]        Moderate>Abstinence 4.283197e-01
## Peptostreptococcaceae_[XI][G.7]        Moderate>Abstinence 1.790106e-02
## Peptostreptococcaceae_[XI][G.9]        Moderate>Abstinence 6.382476e-01
## Peptostreptococcus                     Moderate>Abstinence 2.247877e-01
## Ruminococcaceae_[G.1]                  Moderate>Abstinence 2.014879e-01
## Ruminococcaceae_[G.2]                  Moderate>Abstinence 2.491171e-01
## Bulleidia                              Abstinence>Moderate 8.956075e-01
## Eggerthia                              Moderate>Abstinence 1.819864e-01
## Solobacterium                          Moderate>Abstinence 4.296640e-01
## Mycoplasma                             Moderate>Abstinence 8.956075e-01
## Mitsuokella                            Abstinence>Moderate 4.783940e-02
## Selenomonas                            Moderate>Abstinence 7.868776e-01
## Family.Selenomonadaceae.               Moderate>Abstinence 4.783940e-02
## Class.Negativicutes.                   Moderate>Abstinence 8.819919e-01
## Anaeroglobus                           Moderate>Abstinence 1.819864e-01
## Dialister                              Abstinence>Moderate 6.640139e-01
## Megasphaera                            Moderate>Abstinence 3.669219e-02
## Family.Veillonellaceae.                Moderate>Abstinence 6.233377e-01
## Veillonella                            Abstinence>Moderate 3.000446e-01
## Veillonellaceae_[G.1]                  Abstinence>Moderate 9.388669e-01
```

```
## Phylum.Firmicutes.                    Moderate>Abstinence 1.524368e-01
## Fusobacterium                         Moderate>Abstinence 4.221243e-01
## Leptotrichia                          Abstinence>Moderate 3.104803e-01
## Gracilibacteria_(GN02)_[G.1]          Abstinence>Moderate 1.121479e-01
## Lautropia                             Moderate>Abstinence 9.672483e-02
## Ottowia                               Moderate>Abstinence 9.220554e-01
## Eikenella                             Abstinence>Moderate 8.629395e-01
## Kingella                              Moderate>Abstinence 4.020245e-01
## Neisseria                             Abstinence>Moderate 5.746327e-01
## Family.Neisseriaceae.                 Moderate>Abstinence 8.597002e-01
## Campylobacter                         Abstinence>Moderate 6.926654e-01
## Cardiobacterium                       Abstinence>Moderate 6.109911e-01
## Aggregatibacter                       Abstinence>Moderate 7.868776e-01
## Haemophilus                           Abstinence>Moderate 6.183658e-01
## Family.Pasteurellaceae.               Moderate>Abstinence 4.221243e-01
## Saccharibacteria_(TM7)_[G.1]          Moderate>Abstinence 9.015099e-01
## Saccharibacteria_(TM7)_[G.3]          Abstinence>Moderate 8.956075e-01
## Saccharibacteria_(TM7)_[G.6]          Abstinence>Moderate 1.028777e-01
## Family.Saccharibacteria_(TM7)_[F-1]. Abstinence>Moderate 6.183658e-01
## Saccharibacteria_(TM7)_[G.5]          Moderate>Abstinence 7.868776e-01
## Treponema                             Moderate>Abstinence 4.020245e-01
## Fretibacterium                        Abstinence>Moderate 1.494622e-01
## Kingdom.Bacteria.                     Moderate>Abstinence 1.524368e-01
## Others                                Abstinence>Moderate 6.818367e-01
##                                                                 Feature
## Absconditabacteria_(SR1)_[G.1]           Absconditabacteria_(SR1)_[G.1]
## Actinomyces                                                 Actinomyces
## Peptidiphaga                                               Peptidiphaga
## Schaalia                                                       Schaalia
## Family.Actinomycetaceae.                       Family.Actinomycetaceae.
## Rothia                                                           Rothia
## Alloscardovia                                             Alloscardovia
## Bifidobacterium                                         Bifidobacterium
## Parascardovia                                             Parascardovia
## Scardovia                                                     Scardovia
## Corynebacterium                                         Corynebacterium
## Olsenella                                                     Olsenella
## Atopobium                                                     Atopobium
## Slackia                                                         Slackia
## Cryptobacterium                                         Cryptobacterium
## Bacteroidetes_[G.3]                                 Bacteroidetes_[G.3]
## Bacteroidetes_[G.5]                                 Bacteroidetes_[G.5]
## Family.Bacteroidetes_[F-1].                 Family.Bacteroidetes_[F-1].
## Bacteroidaceae_[G.1]                               Bacteroidaceae_[G.1]
## Bacteroidales_[G.2]                                 Bacteroidales_[G.2]
## Porphyromonas                                             Porphyromonas
## Tannerella                                                   Tannerella
## Alloprevotella                                           Alloprevotella
## Prevotella                                                   Prevotella
## Bergeyella                                                   Bergeyella
## Capnocytophaga                                           Capnocytophaga
## Gemella                                                         Gemella
## Abiotrophia                                                 Abiotrophia
## Granulicatella                                           Granulicatella
```

```
## Lactobacillus                                            Lactobacillus
## Streptococcus                                            Streptococcus
## Order.Lactobacillales.                          Order.Lactobacillales.
## Class.Bacilli.                                          Class.Bacilli.
## Clostridiales_[F.1][G-1]                      Clostridiales_[F.1][G-1]
## Butyrivibrio                                              Butyrivibrio
## Catonella                                                    Catonella
## Johnsonella                                                Johnsonella
## Lachnoanaerobaculum                                Lachnoanaerobaculum
## Lachnospiraceae_[G.2]                            Lachnospiraceae_[G.2]
## Lachnospiraceae_[G.3]                            Lachnospiraceae_[G.3]
## Lachnospiraceae_[G.7]                            Lachnospiraceae_[G.7]
## Lachnospiraceae_[G.8]                            Lachnospiraceae_[G.8]
## Oribacterium                                              Oribacterium
## Shuttleworthia                                          Shuttleworthia
## Stomatobaculum                                          Stomatobaculum
## Family.Lachnospiraceae_[XIV].            Family.Lachnospiraceae_[XIV].
## Peptococcus                                                Peptococcus
## Parvimonas                                                  Parvimonas
## Filifactor                                                  Filifactor
## Mogibacterium                                            Mogibacterium
## Peptostreptococcaceae_[XI][G.1]        Peptostreptococcaceae_[XI][G.1]
## Peptostreptococcaceae_[XI][G.4]        Peptostreptococcaceae_[XI][G.4]
## Peptostreptococcaceae_[XI][G.5]        Peptostreptococcaceae_[XI][G.5]
## Peptostreptococcaceae_[XI][G.6]        Peptostreptococcaceae_[XI][G.6]
## Peptostreptococcaceae_[XI][G.7]        Peptostreptococcaceae_[XI][G.7]
## Peptostreptococcaceae_[XI][G.9]        Peptostreptococcaceae_[XI][G.9]
## Peptostreptococcus                                  Peptostreptococcus
## Ruminococcaceae_[G.1]                            Ruminococcaceae_[G.1]
## Ruminococcaceae_[G.2]                            Ruminococcaceae_[G.2]
## Bulleidia                                                    Bulleidia
## Eggerthia                                                    Eggerthia
## Solobacterium                                            Solobacterium
## Mycoplasma                                                  Mycoplasma
## Mitsuokella                                                Mitsuokella
## Selenomonas                                                Selenomonas
## Family.Selenomonadaceae.                      Family.Selenomonadaceae.
## Class.Negativicutes.                              Class.Negativicutes.
## Anaeroglobus                                              Anaeroglobus
## Dialister                                                    Dialister
## Megasphaera                                                Megasphaera
## Family.Veillonellaceae.                        Family.Veillonellaceae.
## Veillonella                                                Veillonella
## Veillonellaceae_[G.1]                            Veillonellaceae_[G.1]
## Phylum.Firmicutes.                                  Phylum.Firmicutes.
## Fusobacterium                                            Fusobacterium
## Leptotrichia                                              Leptotrichia
## Gracilibacteria_(GN02)_[G.1]              Gracilibacteria_(GN02)_[G.1]
## Lautropia                                                    Lautropia
## Ottowia                                                        Ottowia
## Eikenella                                                    Eikenella
## Kingella                                                      Kingella
## Neisseria                                                    Neisseria
## Family.Neisseriaceae.                            Family.Neisseriaceae.
```

```
## Campylobacter                                                 Campylobacter
## Cardiobacterium                                             Cardiobacterium
## Aggregatibacter                                             Aggregatibacter
## Haemophilus                                                     Haemophilus
## Family.Pasteurellaceae.                             Family.Pasteurellaceae.
## Saccharibacteria_(TM7)_[G.1]                   Saccharibacteria_(TM7)_[G.1]
## Saccharibacteria_(TM7)_[G.3]                   Saccharibacteria_(TM7)_[G.3]
## Saccharibacteria_(TM7)_[G.6]                   Saccharibacteria_(TM7)_[G.6]
## Family.Saccharibacteria_(TM7)_[F-1]. Family.Saccharibacteria_(TM7)_[F-1].
## Saccharibacteria_(TM7)_[G.5]                   Saccharibacteria_(TM7)_[G.5]
## Treponema                                                         Treponema
## Fretibacterium                                               Fretibacterium
## Kingdom.Bacteria.                                         Kingdom.Bacteria.
## Others                                                               Others
##                                                    Method
## Absconditabacteria_(SR1)_[G.1]       DESeq2 man. geoMeans (ds2)
## Actinomyces                          DESeq2 man. geoMeans (ds2)
## Peptidiphaga                         DESeq2 man. geoMeans (ds2)
## Schaalia                             DESeq2 man. geoMeans (ds2)
## Family.Actinomycetaceae.             DESeq2 man. geoMeans (ds2)
## Rothia                               DESeq2 man. geoMeans (ds2)
## Alloscardovia                        DESeq2 man. geoMeans (ds2)
## Bifidobacterium                      DESeq2 man. geoMeans (ds2)
## Parascardovia                        DESeq2 man. geoMeans (ds2)
## Scardovia                            DESeq2 man. geoMeans (ds2)
## Corynebacterium                      DESeq2 man. geoMeans (ds2)
## Olsenella                            DESeq2 man. geoMeans (ds2)
## Atopobium                            DESeq2 man. geoMeans (ds2)
## Slackia                              DESeq2 man. geoMeans (ds2)
## Cryptobacterium                      DESeq2 man. geoMeans (ds2)
## Bacteroidetes_[G.3]                  DESeq2 man. geoMeans (ds2)
## Bacteroidetes_[G.5]                  DESeq2 man. geoMeans (ds2)
## Family.Bacteroidetes_[F-1].          DESeq2 man. geoMeans (ds2)
## Bacteroidaceae_[G.1]                 DESeq2 man. geoMeans (ds2)
## Bacteroidales_[G.2]                  DESeq2 man. geoMeans (ds2)
## Porphyromonas                        DESeq2 man. geoMeans (ds2)
## Tannerella                           DESeq2 man. geoMeans (ds2)
## Alloprevotella                       DESeq2 man. geoMeans (ds2)
## Prevotella                           DESeq2 man. geoMeans (ds2)
## Bergeyella                           DESeq2 man. geoMeans (ds2)
## Capnocytophaga                       DESeq2 man. geoMeans (ds2)
## Gemella                              DESeq2 man. geoMeans (ds2)
## Abiotrophia                          DESeq2 man. geoMeans (ds2)
## Granulicatella                       DESeq2 man. geoMeans (ds2)
## Lactobacillus                        DESeq2 man. geoMeans (ds2)
## Streptococcus                        DESeq2 man. geoMeans (ds2)
## Order.Lactobacillales.               DESeq2 man. geoMeans (ds2)
## Class.Bacilli.                       DESeq2 man. geoMeans (ds2)
## Clostridiales_[F.1][G-1]             DESeq2 man. geoMeans (ds2)
## Butyrivibrio                         DESeq2 man. geoMeans (ds2)
## Catonella                            DESeq2 man. geoMeans (ds2)
## Johnsonella                          DESeq2 man. geoMeans (ds2)
## Lachnoanaerobaculum                  DESeq2 man. geoMeans (ds2)
## Lachnospiraceae_[G.2]                DESeq2 man. geoMeans (ds2)
```

```
## Lachnospiraceae_[G.3]                DESeq2 man. geoMeans (ds2)
## Lachnospiraceae_[G.7]                DESeq2 man. geoMeans (ds2)
## Lachnospiraceae_[G.8]                DESeq2 man. geoMeans (ds2)
## Oribacterium                         DESeq2 man. geoMeans (ds2)
## Shuttleworthia                       DESeq2 man. geoMeans (ds2)
## Stomatobaculum                       DESeq2 man. geoMeans (ds2)
## Family.Lachnospiraceae_[XIV].        DESeq2 man. geoMeans (ds2)
## Peptococcus                          DESeq2 man. geoMeans (ds2)
## Parvimonas                           DESeq2 man. geoMeans (ds2)
## Filifactor                           DESeq2 man. geoMeans (ds2)
## Mogibacterium                        DESeq2 man. geoMeans (ds2)
## Peptostreptococcaceae_[XI][G.1]      DESeq2 man. geoMeans (ds2)
## Peptostreptococcaceae_[XI][G.4]      DESeq2 man. geoMeans (ds2)
## Peptostreptococcaceae_[XI][G.5]      DESeq2 man. geoMeans (ds2)
## Peptostreptococcaceae_[XI][G.6]      DESeq2 man. geoMeans (ds2)
## Peptostreptococcaceae_[XI][G.7]      DESeq2 man. geoMeans (ds2)
## Peptostreptococcaceae_[XI][G.9]      DESeq2 man. geoMeans (ds2)
## Peptostreptococcus                   DESeq2 man. geoMeans (ds2)
## Ruminococcaceae_[G.1]                DESeq2 man. geoMeans (ds2)
## Ruminococcaceae_[G.2]                DESeq2 man. geoMeans (ds2)
## Bulleidia                            DESeq2 man. geoMeans (ds2)
## Eggerthia                            DESeq2 man. geoMeans (ds2)
## Solobacterium                        DESeq2 man. geoMeans (ds2)
## Mycoplasma                           DESeq2 man. geoMeans (ds2)
## Mitsuokella                          DESeq2 man. geoMeans (ds2)
## Selenomonas                          DESeq2 man. geoMeans (ds2)
## Family.Selenomonadaceae.             DESeq2 man. geoMeans (ds2)
## Class.Negativicutes.                 DESeq2 man. geoMeans (ds2)
## Anaeroglobus                         DESeq2 man. geoMeans (ds2)
## Dialister                            DESeq2 man. geoMeans (ds2)
## Megasphaera                          DESeq2 man. geoMeans (ds2)
## Family.Veillonellaceae.              DESeq2 man. geoMeans (ds2)
## Veillonella                          DESeq2 man. geoMeans (ds2)
## Veillonellaceae_[G.1]                DESeq2 man. geoMeans (ds2)
## Phylum.Firmicutes.                   DESeq2 man. geoMeans (ds2)
## Fusobacterium                        DESeq2 man. geoMeans (ds2)
## Leptotrichia                         DESeq2 man. geoMeans (ds2)
## Gracilibacteria_(GN02)_[G.1]         DESeq2 man. geoMeans (ds2)
## Lautropia                            DESeq2 man. geoMeans (ds2)
## Ottowia                              DESeq2 man. geoMeans (ds2)
## Eikenella                            DESeq2 man. geoMeans (ds2)
## Kingella                             DESeq2 man. geoMeans (ds2)
## Neisseria                            DESeq2 man. geoMeans (ds2)
## Family.Neisseriaceae.                DESeq2 man. geoMeans (ds2)
## Campylobacter                        DESeq2 man. geoMeans (ds2)
## Cardiobacterium                      DESeq2 man. geoMeans (ds2)
## Aggregatibacter                      DESeq2 man. geoMeans (ds2)
## Haemophilus                          DESeq2 man. geoMeans (ds2)
## Family.Pasteurellaceae.              DESeq2 man. geoMeans (ds2)
## Saccharibacteria_(TM7)_[G.1]         DESeq2 man. geoMeans (ds2)
## Saccharibacteria_(TM7)_[G.3]         DESeq2 man. geoMeans (ds2)
## Saccharibacteria_(TM7)_[G.6]         DESeq2 man. geoMeans (ds2)
## Family.Saccharibacteria_(TM7)_[F-1]. DESeq2 man. geoMeans (ds2)
## Saccharibacteria_(TM7)_[G.5]         DESeq2 man. geoMeans (ds2)
```

```
## Treponema                                 DESeq2 man. geoMeans (ds2)
## Fretibacterium                            DESeq2 man. geoMeans (ds2)
## Kingdom.Bacteria.                         DESeq2 man. geoMeans (ds2)
## Others                                    DESeq2 man. geoMeans (ds2)
```

```
write.table(final, file="Alcprweek_cat_ds2.txt", sep="\t", dec=",", row.names=F)
write.table(final[final$pval<0.05,], file="Alcprweek_cat_ds2_sig.txt", sep="\t", dec=",", row.names=F)
final <- DA.kru(Microbio2, predictor = Phe2$Alcprweek_cat)
final
```

```
##                                                  pval   pval.adj
## Absconditabacteria_(SR1)_[G.1]       0.2929689782 0.53618851
## Actinomyces                          0.0213774150 0.13824062
## Peptidiphaga                         0.7818602315 0.85213980
## Schaalia                             0.2173617040 0.43028745
## Family.Actinomycetaceae.             0.3726023972 0.54870552
## Rothia                               0.0011159595 0.03608269
## Alloscardovia                        0.8813495903 0.90947777
## Bifidobacterium                      0.3511768516 0.54870552
## Parascardovia                        0.3483245552 0.54870552
## Scardovia                            0.6347905518 0.74927922
## Corynebacterium                      0.4060806719 0.56949487
## Olsenella                            0.2030141393 0.42136027
## Atopobium                            0.0675783821 0.22603804
## Slackia                              0.0726157671 0.23240790
## Cryptobacterium                      0.4694680879 0.58382570
## Bacteroidetes_[G.3]                  0.0345827382 0.15973931
## Bacteroidetes_[G.5]                  0.7322182057 0.80710416
## Family.Bacteroidetes_[F-1].          0.0076519762 0.06747652
## Bacteroidaceae_[G.1]                 0.1425572996 0.32923948
## Bacteroidales_[G.2]                  0.0341183580 0.15973931
## Porphyromonas                        0.1117966184 0.27110680
## Tannerella                           0.6503238529 0.74927922
## Alloprevotella                       0.0063329540 0.06747652
## Prevotella                           0.0170309665 0.11800027
## Bergeyella                           0.4227178410 0.56949487
## Capnocytophaga                       0.1942399659 0.41869504
## Gemella                              0.3714686944 0.54870552
## Abiotrophia                          0.2041642550 0.42136027
## Granulicatella                       0.4220334952 0.56949487
## Lactobacillus                        0.0320897250 0.15973931
## Streptococcus                        0.0006856958 0.03608269
## Order.Lactobacillales.               0.2710128873 0.50554327
## Class.Bacilli.                       0.5554817285 0.67352160
## Clostridiales_[F.1][G-1]             0.2094904727 0.42334533
## Butyrivibrio                         0.3317978406 0.54549814
## Catonella                            0.0016048653 0.03891798
## Johnsonella                          0.8139249307 0.86021502
## Lachnoanaerobaculum                  0.0950896169 0.25621369
## Lachnospiraceae_[G.2]                0.3184326883 0.54549814
## Lachnospiraceae_[G.3]                0.1782626158 0.39298804
## Lachnospiraceae_[G.7]                0.4131410572 0.56949487
## Lachnospiraceae_[G.8]                0.3746339636 0.54870552
## Oribacterium                         0.1148994998 0.27183540
```

```
## Shuttleworthia                        0.0382430262 0.16128581
## Stomatobaculum                        0.1103773852 0.27110680
## Family.Lachnospiraceae_[XIV].         0.3790027798 0.54870552
## Peptococcus                           0.3317529590 0.54549814
## Parvimonas                            0.0263027436 0.15946038
## Filifactor                            0.0115581046 0.08624124
## Mogibacterium                         0.4847324086 0.59517777
## Peptostreptococcaceae_[XI][G.1]       0.0032910494 0.05320530
## Peptostreptococcaceae_[XI][G.4]       0.3746696093 0.54870552
## Peptostreptococcaceae_[XI][G.5]       0.4330742919 0.57518380
## Peptostreptococcaceae_[XI][G.6]       0.0450555892 0.18209967
## Peptostreptococcaceae_[XI][G.7]       0.0377699068 0.16128581
## Peptostreptococcaceae_[XI][G.9]       0.0840948395 0.24718786
## Peptostreptococcus                    0.0054349079 0.06589826
## Ruminococcaceae_[G.1]                 0.0333312914 0.15973931
## Ruminococcaceae_[G.2]                 0.0009946202 0.03608269
## Bulleidia                             0.2509249058 0.48679432
## Eggerthia                             0.3105756617 0.54549814
## Solobacterium                         0.0765515339 0.23240790
## Mycoplasma                            0.8145817756 0.86021502
## Mitsuokella                           0.0105686672 0.08543006
## Selenomonas                           0.4445187664 0.57518380
## Family.Selenomonadaceae.              0.0041989031 0.05818480
## Class.Negativicutes.                  0.7097678390 0.80003765
## Anaeroglobus                          0.1044749915 0.26668616
## Dialister                             0.0473152913 0.18358333
## Megasphaera                           0.0022608882 0.04386123
## Family.Veillonellaceae.               0.0994879197 0.26081968
## Veillonella                           0.0660486572 0.22603804
## Veillonellaceae_[G.1]                 0.4689605497 0.58382570
## Phylum.Firmicutes.                    0.3051630693 0.54549814
## Fusobacterium                         0.0599753038 0.21546683
## Leptotrichia                          0.9646076849 0.96460768
## Gracilibacteria_(GN02)_[G.1]          0.0579846177 0.21546683
## Lautropia                             0.0075102555 0.06747652
## Ottowia                               0.6450526602 0.74927922
## Eikenella                             0.6565848842 0.74927922
## Kingella                              0.2612161410 0.49682286
## Neisseria                             0.0907998932 0.25621369
## Family.Neisseriaceae.                 0.9616157014 0.96460768
## Campylobacter                         0.3214286753 0.54549814
## Cardiobacterium                       0.7175595436 0.80003765
## Aggregatibacter                       0.1659318054 0.37431128
## Haemophilus                           0.0766706459 0.23240790
## Family.Pasteurellaceae.               0.8563497060 0.89318195
## Saccharibacteria_(TM7)_[G.1]          0.9572454852 0.96460768
## Saccharibacteria_(TM7)_[G.3]          0.6273557241 0.74927922
## Saccharibacteria_(TM7)_[G.6]          0.0280050160 0.15973931
## Family.Saccharibacteria_(TM7)_[F-1].  0.4506594687 0.57518380
## Saccharibacteria_(TM7)_[G.5]          0.4450947793 0.57518380
## Treponema                             0.4000746557 0.56949487
## Fretibacterium                        0.0942461469 0.25621369
## Kingdom.Bacteria.                     0.3418754893 0.54870552
## Others                                0.8158740389 0.86021502
```

```
##                                                                          Feature
## Absconditabacteria_(SR1)_[G.1]              Absconditabacteria_(SR1)_[G.1]
## Actinomyces                                                      Actinomyces
## Peptidiphaga                                                    Peptidiphaga
## Schaalia                                                            Schaalia
## Family.Actinomycetaceae.                          Family.Actinomycetaceae.
## Rothia                                                                Rothia
## Alloscardovia                                                  Alloscardovia
## Bifidobacterium                                              Bifidobacterium
## Parascardovia                                                  Parascardovia
## Scardovia                                                          Scardovia
## Corynebacterium                                              Corynebacterium
## Olsenella                                                          Olsenella
## Atopobium                                                          Atopobium
## Slackia                                                              Slackia
## Cryptobacterium                                              Cryptobacterium
## Bacteroidetes_[G.3]                                      Bacteroidetes_[G.3]
## Bacteroidetes_[G.5]                                      Bacteroidetes_[G.5]
## Family.Bacteroidetes_[F-1].                        Family.Bacteroidetes_[F-1].
## Bacteroidaceae_[G.1]                                    Bacteroidaceae_[G.1]
## Bacteroidales_[G.2]                                      Bacteroidales_[G.2]
## Porphyromonas                                                  Porphyromonas
## Tannerella                                                        Tannerella
## Alloprevotella                                                Alloprevotella
## Prevotella                                                        Prevotella
## Bergeyella                                                        Bergeyella
## Capnocytophaga                                                Capnocytophaga
## Gemella                                                              Gemella
## Abiotrophia                                                      Abiotrophia
## Granulicatella                                                Granulicatella
## Lactobacillus                                                  Lactobacillus
## Streptococcus                                                  Streptococcus
## Order.Lactobacillales.                              Order.Lactobacillales.
## Class.Bacilli.                                                Class.Bacilli.
## Clostridiales_[F.1][G-1]                          Clostridiales_[F.1][G-1]
## Butyrivibrio                                                    Butyrivibrio
## Catonella                                                          Catonella
## Johnsonella                                                      Johnsonella
## Lachnoanaerobaculum                                      Lachnoanaerobaculum
## Lachnospiraceae_[G.2]                                  Lachnospiraceae_[G.2]
## Lachnospiraceae_[G.3]                                  Lachnospiraceae_[G.3]
## Lachnospiraceae_[G.7]                                  Lachnospiraceae_[G.7]
## Lachnospiraceae_[G.8]                                  Lachnospiraceae_[G.8]
## Oribacterium                                                    Oribacterium
## Shuttleworthia                                                Shuttleworthia
## Stomatobaculum                                                Stomatobaculum
## Family.Lachnospiraceae_[XIV].                    Family.Lachnospiraceae_[XIV].
## Peptococcus                                                      Peptococcus
## Parvimonas                                                        Parvimonas
## Filifactor                                                        Filifactor
## Mogibacterium                                                  Mogibacterium
## Peptostreptococcaceae_[XI][G.1]            Peptostreptococcaceae_[XI][G.1]
## Peptostreptococcaceae_[XI][G.4]            Peptostreptococcaceae_[XI][G.4]
## Peptostreptococcaceae_[XI][G.5]            Peptostreptococcaceae_[XI][G.5]
```

```
## Peptostreptococcaceae_[XI][G.6]          Peptostreptococcaceae_[XI][G.6]
## Peptostreptococcaceae_[XI][G.7]          Peptostreptococcaceae_[XI][G.7]
## Peptostreptococcaceae_[XI][G.9]          Peptostreptococcaceae_[XI][G.9]
## Peptostreptococcus                                    Peptostreptococcus
## Ruminococcaceae_[G.1]                              Ruminococcaceae_[G.1]
## Ruminococcaceae_[G.2]                              Ruminococcaceae_[G.2]
## Bulleidia                                                        Bulleidia
## Eggerthia                                                        Eggerthia
## Solobacterium                                                Solobacterium
## Mycoplasma                                                      Mycoplasma
## Mitsuokella                                                    Mitsuokella
## Selenomonas                                                    Selenomonas
## Family.Selenomonadaceae.                        Family.Selenomonadaceae.
## Class.Negativicutes.                                Class.Negativicutes.
## Anaeroglobus                                                  Anaeroglobus
## Dialister                                                        Dialister
## Megasphaera                                                    Megasphaera
## Family.Veillonellaceae.                          Family.Veillonellaceae.
## Veillonella                                                    Veillonella
## Veillonellaceae_[G.1]                              Veillonellaceae_[G.1]
## Phylum.Firmicutes.                                    Phylum.Firmicutes.
## Fusobacterium                                                Fusobacterium
## Leptotrichia                                                  Leptotrichia
## Gracilibacteria_(GN02)_[G.1]                Gracilibacteria_(GN02)_[G.1]
## Lautropia                                                        Lautropia
## Ottowia                                                            Ottowia
## Eikenella                                                        Eikenella
## Kingella                                                          Kingella
## Neisseria                                                        Neisseria
## Family.Neisseriaceae.                              Family.Neisseriaceae.
## Campylobacter                                                Campylobacter
## Cardiobacterium                                            Cardiobacterium
## Aggregatibacter                                            Aggregatibacter
## Haemophilus                                                    Haemophilus
## Family.Pasteurellaceae.                          Family.Pasteurellaceae.
## Saccharibacteria_(TM7)_[G.1]                Saccharibacteria_(TM7)_[G.1]
## Saccharibacteria_(TM7)_[G.3]                Saccharibacteria_(TM7)_[G.3]
## Saccharibacteria_(TM7)_[G.6]                Saccharibacteria_(TM7)_[G.6]
## Family.Saccharibacteria_(TM7)_[F-1]. Family.Saccharibacteria_(TM7)_[F-1].
## Saccharibacteria_(TM7)_[G.5]                Saccharibacteria_(TM7)_[G.5]
## Treponema                                                        Treponema
## Fretibacterium                                              Fretibacterium
## Kingdom.Bacteria.                                      Kingdom.Bacteria.
## Others                                                              Others
##                                                   Method
## Absconditabacteria_(SR1)_[G.1]       Kruskal-Wallis (kru)
## Actinomyces                          Kruskal-Wallis (kru)
## Peptidiphaga                         Kruskal-Wallis (kru)
## Schaalia                             Kruskal-Wallis (kru)
## Family.Actinomycetaceae.             Kruskal-Wallis (kru)
## Rothia                               Kruskal-Wallis (kru)
## Alloscardovia                        Kruskal-Wallis (kru)
## Bifidobacterium                      Kruskal-Wallis (kru)
## Parascardovia                        Kruskal-Wallis (kru)
```

```
## Scardovia                           Kruskal-Wallis (kru)
## Corynebacterium                     Kruskal-Wallis (kru)
## Olsenella                           Kruskal-Wallis (kru)
## Atopobium                           Kruskal-Wallis (kru)
## Slackia                             Kruskal-Wallis (kru)
## Cryptobacterium                     Kruskal-Wallis (kru)
## Bacteroidetes_[G.3]                 Kruskal-Wallis (kru)
## Bacteroidetes_[G.5]                 Kruskal-Wallis (kru)
## Family.Bacteroidetes_[F-1].         Kruskal-Wallis (kru)
## Bacteroidaceae_[G.1]                Kruskal-Wallis (kru)
## Bacteroidales_[G.2]                 Kruskal-Wallis (kru)
## Porphyromonas                       Kruskal-Wallis (kru)
## Tannerella                          Kruskal-Wallis (kru)
## Alloprevotella                      Kruskal-Wallis (kru)
## Prevotella                          Kruskal-Wallis (kru)
## Bergeyella                          Kruskal-Wallis (kru)
## Capnocytophaga                      Kruskal-Wallis (kru)
## Gemella                             Kruskal-Wallis (kru)
## Abiotrophia                         Kruskal-Wallis (kru)
## Granulicatella                      Kruskal-Wallis (kru)
## Lactobacillus                       Kruskal-Wallis (kru)
## Streptococcus                       Kruskal-Wallis (kru)
## Order.Lactobacillales.              Kruskal-Wallis (kru)
## Class.Bacilli.                      Kruskal-Wallis (kru)
## Clostridiales_[F.1][G-1]            Kruskal-Wallis (kru)
## Butyrivibrio                        Kruskal-Wallis (kru)
## Catonella                           Kruskal-Wallis (kru)
## Johnsonella                         Kruskal-Wallis (kru)
## Lachnoanaerobaculum                 Kruskal-Wallis (kru)
## Lachnospiraceae_[G.2]               Kruskal-Wallis (kru)
## Lachnospiraceae_[G.3]               Kruskal-Wallis (kru)
## Lachnospiraceae_[G.7]               Kruskal-Wallis (kru)
## Lachnospiraceae_[G.8]               Kruskal-Wallis (kru)
## Oribacterium                        Kruskal-Wallis (kru)
## Shuttleworthia                      Kruskal-Wallis (kru)
## Stomatobaculum                      Kruskal-Wallis (kru)
## Family.Lachnospiraceae_[XIV].       Kruskal-Wallis (kru)
## Peptococcus                         Kruskal-Wallis (kru)
## Parvimonas                          Kruskal-Wallis (kru)
## Filifactor                          Kruskal-Wallis (kru)
## Mogibacterium                       Kruskal-Wallis (kru)
## Peptostreptococcaceae_[XI][G.1]     Kruskal-Wallis (kru)
## Peptostreptococcaceae_[XI][G.4]     Kruskal-Wallis (kru)
## Peptostreptococcaceae_[XI][G.5]     Kruskal-Wallis (kru)
## Peptostreptococcaceae_[XI][G.6]     Kruskal-Wallis (kru)
## Peptostreptococcaceae_[XI][G.7]     Kruskal-Wallis (kru)
## Peptostreptococcaceae_[XI][G.9]     Kruskal-Wallis (kru)
## Peptostreptococcus                  Kruskal-Wallis (kru)
## Ruminococcaceae_[G.1]               Kruskal-Wallis (kru)
## Ruminococcaceae_[G.2]               Kruskal-Wallis (kru)
## Bulleidia                           Kruskal-Wallis (kru)
## Eggerthia                           Kruskal-Wallis (kru)
## Solobacterium                       Kruskal-Wallis (kru)
## Mycoplasma                          Kruskal-Wallis (kru)
```

```
## Mitsuokella                            Kruskal-Wallis (kru)
## Selenomonas                            Kruskal-Wallis (kru)
## Family.Selenomonadaceae.               Kruskal-Wallis (kru)
## Class.Negativicutes.                   Kruskal-Wallis (kru)
## Anaeroglobus                           Kruskal-Wallis (kru)
## Dialister                              Kruskal-Wallis (kru)
## Megasphaera                            Kruskal-Wallis (kru)
## Family.Veillonellaceae.                Kruskal-Wallis (kru)
## Veillonella                            Kruskal-Wallis (kru)
## Veillonellaceae_[G.1]                  Kruskal-Wallis (kru)
## Phylum.Firmicutes.                     Kruskal-Wallis (kru)
## Fusobacterium                          Kruskal-Wallis (kru)
## Leptotrichia                           Kruskal-Wallis (kru)
## Gracilibacteria_(GN02)_[G.1]           Kruskal-Wallis (kru)
## Lautropia                              Kruskal-Wallis (kru)
## Ottowia                                Kruskal-Wallis (kru)
## Eikenella                              Kruskal-Wallis (kru)
## Kingella                               Kruskal-Wallis (kru)
## Neisseria                              Kruskal-Wallis (kru)
## Family.Neisseriaceae.                  Kruskal-Wallis (kru)
## Campylobacter                          Kruskal-Wallis (kru)
## Cardiobacterium                        Kruskal-Wallis (kru)
## Aggregatibacter                        Kruskal-Wallis (kru)
## Haemophilus                            Kruskal-Wallis (kru)
## Family.Pasteurellaceae.                Kruskal-Wallis (kru)
## Saccharibacteria_(TM7)_[G.1]           Kruskal-Wallis (kru)
## Saccharibacteria_(TM7)_[G.3]           Kruskal-Wallis (kru)
## Saccharibacteria_(TM7)_[G.6]           Kruskal-Wallis (kru)
## Family.Saccharibacteria_(TM7)_[F-1]. Kruskal-Wallis (kru)
## Saccharibacteria_(TM7)_[G.5]           Kruskal-Wallis (kru)
## Treponema                              Kruskal-Wallis (kru)
## Fretibacterium                         Kruskal-Wallis (kru)
## Kingdom.Bacteria.                      Kruskal-Wallis (kru)
## Others                                 Kruskal-Wallis (kru)
```

```
write.table(final, file="Alcprweek_cat_kru.txt", sep="\t", dec=",", row.names=F)
write.table(final[final$pval<0.05,], file="Alcprweek_cat_kru_sig.txt", sep="\t", dec=",", row.names=F)
#################bp_cat
table(Phe$bp_cat, useNA="always")
```

```
##
##              Normal     Mild_elevated Moderate_elevated Severely_elevated
##                 157                93               356               138
##                <NA>
##                   2
```

```
#Remove NA
Phe2<-Phe[complete.cases(Phe$bp_cat),]
#Also subset columns
Microbio2<-dplyr::select(Microbio, one_of(Phe2$IDX))
final <- DA.ds2(Microbio2, predictor = Phe2$bp_cat, out.all=TRUE) #It is LRT so don't look at ordering
final
```

```
##                                          baseMean log2FoldChange      lfcSE
```

```
## Absconditabacteria_(SR1)_[G.1]          66.1058777   -1.631801260 0.43743672
## Actinomyces                            323.7803206    0.285465847 0.14786897
## Peptidiphaga                            22.0416883    0.269031021 0.21181294
## Schaalia                               703.5570032    0.111243645 0.12206644
## Family.Actinomycetaceae.                 5.4318947    0.007436356 0.30665914
## Rothia                                1938.6926786   -0.069117621 0.14258326
## Alloscardovia                           12.0844404    0.206858666 0.37346154
## Bifidobacterium                         10.7284828    0.439005755 0.40899188
## Parascardovia                            2.9264171    1.055140752 0.56870279
## Scardovia                               14.3693609    0.224342267 0.36916094
## Corynebacterium                         93.8520152    0.164430358 0.15567203
## Olsenella                                1.5705319   -0.005079829 0.34732878
## Atopobium                              120.2270723    0.083895953 0.14325484
## Slackia                                  1.3929535    0.158787096 0.33629371
## Cryptobacterium                          4.2513998    0.295842283 0.35439488
## Bacteroidetes_[G.3]                      6.6074797    0.547557811 0.37561691
## Bacteroidetes_[G.5]                      4.6913133    0.014265842 0.41051713
## Family.Bacteroidetes_[F-1].              0.8714947   -1.088144910 0.59820608
## Bacteroidaceae_[G.1]                     0.7948120   -0.688920472 0.56119433
## Bacteroidales_[G.2]                     12.0719990    0.131892325 0.21556046
## Porphyromonas                          682.2933394    0.005644740 0.19551945
## Tannerella                              22.0983847    0.055008456 0.14530440
## Alloprevotella                         362.5219516   -0.095809577 0.15589130
## Prevotella                            3807.2361865    0.105011315 0.09900078
## Bergeyella                              72.7155861   -0.014766525 0.15297157
## Capnocytophaga                         225.4613668    0.090451817 0.16319753
## Gemella                                309.8006772    0.020865250 0.13591425
## Abiotrophia                             30.0741451    0.050237607 0.29346030
## Granulicatella                         306.3404328   -0.035324994 0.11930420
## Lactobacillus                           37.0583312    0.113508650 0.47803212
## Streptococcus                         3797.5698295    0.119793597 0.11184783
## Order.Lactobacillales.                   0.2871119   -0.287620884 0.40726339
## Class.Bacilli.                           0.5755295   -0.133548406 0.32119895
## Clostridiales_[F.1][G-1]                 1.0579178    0.070296628 0.49360222
## Butyrivibrio                             6.9656228   -0.222080728 0.31338955
## Catonella                               36.9095724    0.019647361 0.17610813
## Johnsonella                              0.9421596    0.644601320 0.42317763
## Lachnoanaerobaculum                     87.8820004    0.287496285 0.12241918
## Lachnospiraceae_[G.2]                   28.0534664   -0.102303628 0.32273673
## Lachnospiraceae_[G.3]                    2.8995123    0.669526254 0.33059257
## Lachnospiraceae_[G.7]                    1.4732956    0.285764378 0.47845963
## Lachnospiraceae_[G.8]                    1.4367939   -0.208026693 0.51335580
## Oribacterium                           147.1321858   -0.142020031 0.13628973
## Shuttleworthia                           2.8108966   -0.497992027 0.38299279
## Stomatobaculum                         112.9182555    0.144250642 0.15316165
## Family.Lachnospiraceae_[XIV].            1.6095271    0.151918360 0.42617466
## Peptococcus                              3.7157473    0.089138535 0.38100655
## Parvimonas                              40.9495178   -0.116651999 0.18906536
## Filifactor                               6.8621336   -0.356292328 0.36977902
## Mogibacterium                           41.1723089    0.203952846 0.13029531
## Peptostreptococcaceae_[XI][G.1]         67.7192574   -0.066292675 0.23261779
## Peptostreptococcaceae_[XI][G.4]          0.4191905   -0.053110537 0.50602664
## Peptostreptococcaceae_[XI][G.5]          1.6690812    0.118527387 0.53196042
## Peptostreptococcaceae_[XI][G.6]          1.0175853   -0.002662948 0.52396701
```

```
## Peptostreptococcaceae_[XI][G.7]         4.7195993   -0.265006458 0.36511020
## Peptostreptococcaceae_[XI][G.9]         8.7363992   -0.045207528 0.21594839
## Peptostreptococcus                     42.9864069   -0.143793997 0.27206231
## Ruminococcaceae_[G.1]                  25.1836759   -0.153113515 0.27672398
## Ruminococcaceae_[G.2]                  41.5234417    0.531217582 0.22985626
## Bulleidia                               3.8709078   -0.067680738 0.40602954
## Eggerthia                               1.4587509   -0.532124160 0.54344470
## Solobacterium                          74.1630338   -0.296153027 0.15369210
## Mycoplasma                              4.5000508   -0.099490151 0.32508007
## Mitsuokella                            26.7544753   -0.171471206 0.29135947
## Selenomonas                           222.3185156   -0.050342908 0.13104378
## Family.Selenomonadaceae.                5.0504886   -0.210128778 0.34462927
## Class.Negativicutes.                    0.7322999   -0.004003693 0.31905977
## Anaeroglobus                            8.7028189    0.462309991 0.31374690
## Dialister                              36.4852787   -0.114194566 0.16075580
## Megasphaera                           407.7957370    0.267560169 0.16892088
## Family.Veillonellaceae.                 0.5239301    0.327646336 0.37188070
## Veillonella                          5061.2445718    0.054852917 0.09534691
## Veillonellaceae_[G.1]                   6.1469526    0.012451647 0.26649497
## Phylum.Firmicutes.                      0.8344489   -0.876788762 0.42136391
## Fusobacterium                         929.2571372    0.077170355 0.12692644
## Leptotrichia                          684.6054135    0.069170432 0.13085897
## Gracilibacteria_(GN02)_[G.1]            2.4161467   -0.502971987 0.42295018
## Lautropia                              68.4994208   -0.099587603 0.24326899
## Ottowia                                 2.3379102    0.408614543 0.54418746
## Eikenella                               8.7703431    0.346889068 0.20279386
## Kingella                              100.0995471   -0.154026814 0.17489628
## Neisseria                            1333.1334336    0.165176303 0.19892948
## Family.Neisseriaceae.                   6.1192335   -0.634231678 0.46646763
## Campylobacter                         257.7162863    0.104142921 0.11200655
## Cardiobacterium                        14.8139218   -0.076724452 0.17504777
## Aggregatibacter                       276.1135518   -0.012675262 0.23131421
## Haemophilus                          2911.7582507   -0.047862433 0.15394483
## Family.Pasteurellaceae.                 2.4849937    0.031810453 0.63529349
## Saccharibacteria_(TM7)_[G.1]           36.1574542    0.159532413 0.25738668
## Saccharibacteria_(TM7)_[G.3]            7.1401803   -0.231799530 0.35890651
## Saccharibacteria_(TM7)_[G.6]            6.8567864   -0.224781419 0.45318358
## Family.Saccharibacteria_(TM7)_[F-1].    1.1040897    0.732982002 0.56432829
## Saccharibacteria_(TM7)_[G.5]            0.9151491    0.313601616 0.53724781
## Treponema                              63.3764075   -0.041691419 0.24422832
## Fretibacterium                          8.2967790    0.043838328 0.29474706
## Kingdom.Bacteria.                       9.4046214   -1.188507240 0.37380053
## Others                                 32.3002188   -1.005684893 0.24446394
##                                            stat         pval
## Absconditabacteria_(SR1)_[G.1]       13.0632805 4.501737e-03
## Actinomyces                           8.9507833 2.995246e-02
## Peptidiphaga                          2.0920160 5.535297e-01
## Schaalia                              3.3015761 3.474233e-01
## Family.Actinomycetaceae.              0.4160474 9.369079e-01
## Rothia                                9.0527366 2.859788e-02
## Alloscardovia                         3.0563752 3.830146e-01
## Bifidobacterium                       3.0181985 3.888278e-01
## Parascardovia                         5.9791123 1.126309e-01
## Scardovia                             9.2921547 2.564845e-02
```

```
## Corynebacterium                        9.9775389 1.875803e-02
## Olsenella                              0.4833339 9.225373e-01
## Atopobium                              3.3874187 3.356597e-01
## Slackia                                3.0133298 3.895745e-01
## Cryptobacterium                        1.7357512 6.290149e-01
## Bacteroidetes_[G.3]                    2.8104141 4.217884e-01
## Bacteroidetes_[G.5]                    3.6270754 3.046510e-01
## Family.Bacteroidetes_[F-1].            6.1315005 1.053848e-01
## Bacteroidaceae_[G.1]                   1.3472357 7.179482e-01
## Bacteroidales_[G.2]                    1.4041377 7.045651e-01
## Porphyromonas                          8.2345061 4.140582e-02
## Tannerella                             1.0077395 7.993792e-01
## Alloprevotella                         4.5862133 2.047280e-01
## Prevotella                             3.3940599 3.347643e-01
## Bergeyella                             2.0104595 5.702386e-01
## Capnocytophaga                         6.0472822 1.093324e-01
## Gemella                                4.1418250 2.465512e-01
## Abiotrophia                            9.1461533 2.740951e-02
## Granulicatella                         0.8910946 8.275763e-01
## Lactobacillus                         39.9243447 1.105589e-08
## Streptococcus                          1.4697199 6.892764e-01
## Order.Lactobacillales.                -2.4930934 1.000000e+00
## Class.Bacilli.                         7.3771948 6.079924e-02
## Clostridiales_[F.1][G-1]               2.5682256 4.630873e-01
## Butyrivibrio                           2.2599998 5.202272e-01
## Catonella                              0.1736595 9.817252e-01
## Johnsonella                            2.8187122 4.204288e-01
## Lachnoanaerobaculum                    8.6015303 3.508583e-02
## Lachnospiraceae_[G.2]                  3.2715454 3.516227e-01
## Lachnospiraceae_[G.3]                  5.2129675 1.568505e-01
## Lachnospiraceae_[G.7]                  4.2365992 2.370231e-01
## Lachnospiraceae_[G.8]                  0.3441321 9.515255e-01
## Oribacterium                           3.1017074 3.762082e-01
## Shuttleworthia                         3.4471077 3.276871e-01
## Stomatobaculum                         2.7015967 4.399560e-01
## Family.Lachnospiraceae_[XIV].          5.0512053 1.680858e-01
## Peptococcus                            0.1428538 9.862399e-01
## Parvimonas                             3.4670143 3.250655e-01
## Filifactor                             1.6076711 6.576517e-01
## Mogibacterium                          3.8349448 2.798475e-01
## Peptostreptococcaceae_[XI][G.1]        1.1405855 7.672867e-01
## Peptostreptococcaceae_[XI][G.4]        0.5346649 9.112123e-01
## Peptostreptococcaceae_[XI][G.5]        0.1775279 9.811328e-01
## Peptostreptococcaceae_[XI][G.6]        0.2474303 9.695919e-01
## Peptostreptococcaceae_[XI][G.7]        1.7021122 6.364643e-01
## Peptostreptococcaceae_[XI][G.9]        0.8546149 8.363641e-01
## Peptostreptococcus                     0.6495672 8.849971e-01
## Ruminococcaceae_[G.1]                  2.1459145 5.426799e-01
## Ruminococcaceae_[G.2]                  6.2014911 1.022083e-01
## Bulleidia                              0.3319753 9.539068e-01
## Eggerthia                              3.5999768 3.080251e-01
## Solobacterium                          3.7441101 2.904543e-01
## Mycoplasma                             5.6047349 1.325068e-01
## Mitsuokella                            2.6969645 4.407434e-01
```

```
## Selenomonas                              3.1671920 3.665588e-01
## Family.Selenomonadaceae.                 1.5990017 6.596162e-01
## Class.Negativicutes.                     1.3362512 7.205434e-01
## Anaeroglobus                             7.8038422 5.024452e-02
## Dialister                               10.4337297 1.521725e-02
## Megasphaera                              9.8033088 2.031425e-02
## Family.Veillonellaceae.                  5.1485761 1.612354e-01
## Veillonella                              1.2769941 7.346032e-01
## Veillonellaceae_[G.1]                    1.0457959 7.901726e-01
## Phylum.Firmicutes.                      12.0987475 7.052436e-03
## Fusobacterium                            0.7555708 8.600617e-01
## Leptotrichia                             0.7769156 8.549802e-01
## Gracilibacteria_(GN02)_[G.1]             2.5560362 4.652491e-01
## Lautropia                                0.1888276 9.793724e-01
## Ottowia                                  1.7089039 6.349561e-01
## Eikenella                                3.4495282 3.273674e-01
## Kingella                                 4.3745076 2.237608e-01
## Neisseria                                4.9615547 1.746340e-01
## Family.Neisseriaceae.                    6.9420905 7.376663e-02
## Campylobacter                            6.2309995 1.008968e-01
## Cardiobacterium                          1.3045171 7.280607e-01
## Aggregatibacter                          0.9361411 8.166985e-01
## Haemophilus                              4.7353855 1.922311e-01
## Family.Pasteurellaceae.                  8.1303796 4.339262e-02
## Saccharibacteria_(TM7)_[G.1]             1.1402986 7.673558e-01
## Saccharibacteria_(TM7)_[G.3]             4.8418221 1.837530e-01
## Saccharibacteria_(TM7)_[G.6]             2.0904808 5.538410e-01
## Family.Saccharibacteria_(TM7)_[F-1].  5.6889107 1.277663e-01
## Saccharibacteria_(TM7)_[G.5]             0.8918050 8.274050e-01
## Treponema                                0.5623469 9.049954e-01
## Fretibacterium                           0.6360992 8.881208e-01
## Kingdom.Bacteria.                       19.1750260 2.515359e-04
## Others                                  18.6794636 3.184539e-04
##                                                     ordering     pval.adj
## Absconditabacteria_(SR1)_[G.1]       Normal>Mild_elevated 1.091671e-01
## Actinomyces                          Mild_elevated>Normal 2.421157e-01
## Peptidiphaga                         Mild_elevated>Normal 8.806979e-01
## Schaalia                             Mild_elevated>Normal 7.409555e-01
## Family.Actinomycetaceae.             Mild_elevated>Normal 9.965133e-01
## Rothia                               Normal>Mild_elevated 2.421157e-01
## Alloscardovia                        Mild_elevated>Normal 7.409555e-01
## Bifidobacterium                      Mild_elevated>Normal 7.409555e-01
## Parascardovia                        Mild_elevated>Normal 4.750085e-01
## Scardovia                            Mild_elevated>Normal 2.421157e-01
## Corynebacterium                      Mild_elevated>Normal 2.421157e-01
## Olsenella                            Normal>Mild_elevated 9.965133e-01
## Atopobium                            Mild_elevated>Normal 7.399771e-01
## Slackia                              Mild_elevated>Normal 7.409555e-01
## Cryptobacterium                      Mild_elevated>Normal 9.498006e-01
## Bacteroidetes_[G.3]                  Mild_elevated>Normal 7.719524e-01
## Bacteroidetes_[G.5]                  Mild_elevated>Normal 7.399771e-01
## Family.Bacteroidetes_[F-1].          Normal>Mild_elevated 4.750085e-01
## Bacteroidaceae_[G.1]                 Normal>Mild_elevated 9.761165e-01
## Bacteroidales_[G.2]                  Mild_elevated>Normal 9.761165e-01
```

```
## Porphyromonas                          Mild_elevated>Normal 2.806056e-01
## Tannerella                             Mild_elevated>Normal 9.965133e-01
## Alloprevotella                         Normal>Mild_elevated 6.205817e-01
## Prevotella                             Mild_elevated>Normal 7.399771e-01
## Bergeyella                             Normal>Mild_elevated 8.921475e-01
## Capnocytophaga                         Mild_elevated>Normal 4.750085e-01
## Gemella                                Mild_elevated>Normal 6.832991e-01
## Abiotrophia                            Mild_elevated>Normal 2.421157e-01
## Granulicatella                         Normal>Mild_elevated 9.965133e-01
## Lactobacillus                          Mild_elevated>Normal 1.072421e-06
## Streptococcus                          Mild_elevated>Normal 9.761165e-01
## Order.Lactobacillales.                 Normal>Mild_elevated 1.000000e+00
## Class.Bacilli.                         Normal>Mild_elevated 3.469133e-01
## Clostridiales_[F.1][G-1]               Mild_elevated>Normal 7.917398e-01
## Butyrivibrio                           Normal>Mild_elevated 8.700351e-01
## Catonella                              Mild_elevated>Normal 9.965133e-01
## Johnsonella                            Mild_elevated>Normal 7.719524e-01
## Lachnoanaerobaculum                    Mild_elevated>Normal 2.617943e-01
## Lachnospiraceae_[G.2]                  Normal>Mild_elevated 7.409555e-01
## Lachnospiraceae_[G.3]                  Mild_elevated>Normal 5.792531e-01
## Lachnospiraceae_[G.7]                  Mild_elevated>Normal 6.762128e-01
## Lachnospiraceae_[G.8]                  Normal>Mild_elevated 9.965133e-01
## Oribacterium                           Normal>Mild_elevated 7.409555e-01
## Shuttleworthia                         Normal>Mild_elevated 7.399771e-01
## Stomatobaculum                         Mild_elevated>Normal 7.773111e-01
## Family.Lachnospiraceae_[XIV].          Mild_elevated>Normal 5.822973e-01
## Peptococcus                            Mild_elevated>Normal 9.965133e-01
## Parvimonas                             Normal>Mild_elevated 7.399771e-01
## Filifactor                             Normal>Mild_elevated 9.549667e-01
## Mogibacterium                          Mild_elevated>Normal 7.399771e-01
## Peptostreptococcaceae_[XI][G.1]        Normal>Mild_elevated 9.924468e-01
## Peptostreptococcaceae_[XI][G.4]        Normal>Mild_elevated 9.965133e-01
## Peptostreptococcaceae_[XI][G.5]        Mild_elevated>Normal 9.965133e-01
## Peptostreptococcaceae_[XI][G.6]        Normal>Mild_elevated 9.965133e-01
## Peptostreptococcaceae_[XI][G.7]        Normal>Mild_elevated 9.498006e-01
## Peptostreptococcaceae_[XI][G.9]        Normal>Mild_elevated 9.965133e-01
## Peptostreptococcus                     Normal>Mild_elevated 9.965133e-01
## Ruminococcaceae_[G.1]                  Normal>Mild_elevated 8.806979e-01
## Ruminococcaceae_[G.2]                  Mild_elevated>Normal 4.750085e-01
## Bulleidia                              Normal>Mild_elevated 9.965133e-01
## Eggerthia                              Normal>Mild_elevated 7.399771e-01
## Solobacterium                          Normal>Mild_elevated 7.399771e-01
## Mycoplasma                             Normal>Mild_elevated 5.141264e-01
## Mitsuokella                            Normal>Mild_elevated 7.773111e-01
## Selenomonas                            Normal>Mild_elevated 7.409555e-01
## Family.Selenomonadaceae.               Normal>Mild_elevated 9.549667e-01
## Class.Negativicutes.                   Normal>Mild_elevated 9.761165e-01
## Anaeroglobus                           Mild_elevated>Normal 3.046074e-01
## Dialister                              Normal>Mild_elevated 2.421157e-01
## Megasphaera                            Mild_elevated>Normal 2.421157e-01
## Family.Veillonellaceae.                Mild_elevated>Normal 5.792531e-01
## Veillonella                            Mild_elevated>Normal 9.761165e-01
## Veillonellaceae_[G.1]                  Mild_elevated>Normal 9.965133e-01
## Phylum.Firmicutes.                     Normal>Mild_elevated 1.368173e-01
```

```
## Fusobacterium                          Mild_elevated>Normal 9.965133e-01
## Leptotrichia                           Mild_elevated>Normal 9.965133e-01
## Gracilibacteria_(GN02)_[G.1]           Normal>Mild_elevated 7.917398e-01
## Lautropia                              Normal>Mild_elevated 9.965133e-01
## Ottowia                                Mild_elevated>Normal 9.498006e-01
## Eikenella                              Mild_elevated>Normal 7.399771e-01
## Kingella                               Normal>Mild_elevated 6.577211e-01
## Neisseria                              Mild_elevated>Normal 5.841208e-01
## Family.Neisseriaceae.                  Normal>Mild_elevated 3.975202e-01
## Campylobacter                          Mild_elevated>Normal 4.750085e-01
## Cardiobacterium                        Normal>Mild_elevated 9.761165e-01
## Aggregatibacter                        Normal>Mild_elevated 9.965133e-01
## Haemophilus                            Normal>Mild_elevated 6.014973e-01
## Family.Pasteurellaceae.                Mild_elevated>Normal 2.806056e-01
## Saccharibacteria_(TM7)_[G.1]           Mild_elevated>Normal 9.924468e-01
## Saccharibacteria_(TM7)_[G.3]           Normal>Mild_elevated 5.941345e-01
## Saccharibacteria_(TM7)_[G.6]           Normal>Mild_elevated 8.806979e-01
## Family.Saccharibacteria_(TM7)_[F-1]. Mild_elevated>Normal 5.141264e-01
## Saccharibacteria_(TM7)_[G.5]           Mild_elevated>Normal 9.965133e-01
## Treponema                              Normal>Mild_elevated 9.965133e-01
## Fretibacterium                         Mild_elevated>Normal 9.965133e-01
## Kingdom.Bacteria.                      Normal>Mild_elevated 1.029668e-02
## Others                                 Normal>Mild_elevated 1.029668e-02
##                                                                   Feature
## Absconditabacteria_(SR1)_[G.1]             Absconditabacteria_(SR1)_[G.1]
## Actinomyces                                                   Actinomyces
## Peptidiphaga                                                 Peptidiphaga
## Schaalia                                                         Schaalia
## Family.Actinomycetaceae.                         Family.Actinomycetaceae.
## Rothia                                                             Rothia
## Alloscardovia                                               Alloscardovia
## Bifidobacterium                                           Bifidobacterium
## Parascardovia                                               Parascardovia
## Scardovia                                                       Scardovia
## Corynebacterium                                           Corynebacterium
## Olsenella                                                       Olsenella
## Atopobium                                                       Atopobium
## Slackia                                                           Slackia
## Cryptobacterium                                           Cryptobacterium
## Bacteroidetes_[G.3]                                   Bacteroidetes_[G.3]
## Bacteroidetes_[G.5]                                   Bacteroidetes_[G.5]
## Family.Bacteroidetes_[F-1].                   Family.Bacteroidetes_[F-1].
## Bacteroidaceae_[G.1]                                 Bacteroidaceae_[G.1]
## Bacteroidales_[G.2]                                   Bacteroidales_[G.2]
## Porphyromonas                                               Porphyromonas
## Tannerella                                                     Tannerella
## Alloprevotella                                             Alloprevotella
## Prevotella                                                     Prevotella
## Bergeyella                                                     Bergeyella
## Capnocytophaga                                             Capnocytophaga
## Gemella                                                           Gemella
## Abiotrophia                                                   Abiotrophia
## Granulicatella                                             Granulicatella
## Lactobacillus                                               Lactobacillus
```

```
## Streptococcus                                              Streptococcus
## Order.Lactobacillales.                            Order.Lactobacillales.
## Class.Bacilli.                                            Class.Bacilli.
## Clostridiales_[F.1][G-1]                        Clostridiales_[F.1][G-1]
## Butyrivibrio                                                Butyrivibrio
## Catonella                                                      Catonella
## Johnsonella                                                  Johnsonella
## Lachnoanaerobaculum                                  Lachnoanaerobaculum
## Lachnospiraceae_[G.2]                              Lachnospiraceae_[G.2]
## Lachnospiraceae_[G.3]                              Lachnospiraceae_[G.3]
## Lachnospiraceae_[G.7]                              Lachnospiraceae_[G.7]
## Lachnospiraceae_[G.8]                              Lachnospiraceae_[G.8]
## Oribacterium                                                Oribacterium
## Shuttleworthia                                            Shuttleworthia
## Stomatobaculum                                            Stomatobaculum
## Family.Lachnospiraceae_[XIV].              Family.Lachnospiraceae_[XIV].
## Peptococcus                                                  Peptococcus
## Parvimonas                                                    Parvimonas
## Filifactor                                                    Filifactor
## Mogibacterium                                              Mogibacterium
## Peptostreptococcaceae_[XI][G.1]          Peptostreptococcaceae_[XI][G.1]
## Peptostreptococcaceae_[XI][G.4]          Peptostreptococcaceae_[XI][G.4]
## Peptostreptococcaceae_[XI][G.5]          Peptostreptococcaceae_[XI][G.5]
## Peptostreptococcaceae_[XI][G.6]          Peptostreptococcaceae_[XI][G.6]
## Peptostreptococcaceae_[XI][G.7]          Peptostreptococcaceae_[XI][G.7]
## Peptostreptococcaceae_[XI][G.9]          Peptostreptococcaceae_[XI][G.9]
## Peptostreptococcus                                    Peptostreptococcus
## Ruminococcaceae_[G.1]                              Ruminococcaceae_[G.1]
## Ruminococcaceae_[G.2]                              Ruminococcaceae_[G.2]
## Bulleidia                                                      Bulleidia
## Eggerthia                                                      Eggerthia
## Solobacterium                                              Solobacterium
## Mycoplasma                                                    Mycoplasma
## Mitsuokella                                                  Mitsuokella
## Selenomonas                                                  Selenomonas
## Family.Selenomonadaceae.                        Family.Selenomonadaceae.
## Class.Negativicutes.                                Class.Negativicutes.
## Anaeroglobus                                                Anaeroglobus
## Dialister                                                      Dialister
## Megasphaera                                                  Megasphaera
## Family.Veillonellaceae.                          Family.Veillonellaceae.
## Veillonella                                                  Veillonella
## Veillonellaceae_[G.1]                              Veillonellaceae_[G.1]
## Phylum.Firmicutes.                                    Phylum.Firmicutes.
## Fusobacterium                                              Fusobacterium
## Leptotrichia                                                Leptotrichia
## Gracilibacteria_(GN02)_[G.1]                Gracilibacteria_(GN02)_[G.1]
## Lautropia                                                      Lautropia
## Ottowia                                                          Ottowia
## Eikenella                                                      Eikenella
## Kingella                                                        Kingella
## Neisseria                                                      Neisseria
## Family.Neisseriaceae.                              Family.Neisseriaceae.
## Campylobacter                                              Campylobacter
```

```
## Cardiobacterium                                          Cardiobacterium
## Aggregatibacter                                          Aggregatibacter
## Haemophilus                                                   Haemophilus
## Family.Pasteurellaceae.                           Family.Pasteurellaceae.
## Saccharibacteria_(TM7)_[G.1]                 Saccharibacteria_(TM7)_[G.1]
## Saccharibacteria_(TM7)_[G.3]                 Saccharibacteria_(TM7)_[G.3]
## Saccharibacteria_(TM7)_[G.6]                 Saccharibacteria_(TM7)_[G.6]
## Family.Saccharibacteria_(TM7)_[F-1]. Family.Saccharibacteria_(TM7)_[F-1].
## Saccharibacteria_(TM7)_[G.5]                 Saccharibacteria_(TM7)_[G.5]
## Treponema                                                       Treponema
## Fretibacterium                                             Fretibacterium
## Kingdom.Bacteria.                                       Kingdom.Bacteria.
## Others                                                             Others
##                                                Method
## Absconditabacteria_(SR1)_[G.1]       DESeq2 man. geoMeans (ds2)
## Actinomyces                          DESeq2 man. geoMeans (ds2)
## Peptidiphaga                         DESeq2 man. geoMeans (ds2)
## Schaalia                             DESeq2 man. geoMeans (ds2)
## Family.Actinomycetaceae.             DESeq2 man. geoMeans (ds2)
## Rothia                               DESeq2 man. geoMeans (ds2)
## Alloscardovia                        DESeq2 man. geoMeans (ds2)
## Bifidobacterium                      DESeq2 man. geoMeans (ds2)
## Parascardovia                        DESeq2 man. geoMeans (ds2)
## Scardovia                            DESeq2 man. geoMeans (ds2)
## Corynebacterium                      DESeq2 man. geoMeans (ds2)
## Olsenella                            DESeq2 man. geoMeans (ds2)
## Atopobium                            DESeq2 man. geoMeans (ds2)
## Slackia                              DESeq2 man. geoMeans (ds2)
## Cryptobacterium                      DESeq2 man. geoMeans (ds2)
## Bacteroidetes_[G.3]                  DESeq2 man. geoMeans (ds2)
## Bacteroidetes_[G.5]                  DESeq2 man. geoMeans (ds2)
## Family.Bacteroidetes_[F-1].          DESeq2 man. geoMeans (ds2)
## Bacteroidaceae_[G.1]                 DESeq2 man. geoMeans (ds2)
## Bacteroidales_[G.2]                  DESeq2 man. geoMeans (ds2)
## Porphyromonas                        DESeq2 man. geoMeans (ds2)
## Tannerella                           DESeq2 man. geoMeans (ds2)
## Alloprevotella                       DESeq2 man. geoMeans (ds2)
## Prevotella                           DESeq2 man. geoMeans (ds2)
## Bergeyella                           DESeq2 man. geoMeans (ds2)
## Capnocytophaga                       DESeq2 man. geoMeans (ds2)
## Gemella                              DESeq2 man. geoMeans (ds2)
## Abiotrophia                          DESeq2 man. geoMeans (ds2)
## Granulicatella                       DESeq2 man. geoMeans (ds2)
## Lactobacillus                        DESeq2 man. geoMeans (ds2)
## Streptococcus                        DESeq2 man. geoMeans (ds2)
## Order.Lactobacillales.               DESeq2 man. geoMeans (ds2)
## Class.Bacilli.                       DESeq2 man. geoMeans (ds2)
## Clostridiales_[F.1][G-1]             DESeq2 man. geoMeans (ds2)
## Butyrivibrio                         DESeq2 man. geoMeans (ds2)
## Catonella                            DESeq2 man. geoMeans (ds2)
## Johnsonella                          DESeq2 man. geoMeans (ds2)
## Lachnoanaerobaculum                  DESeq2 man. geoMeans (ds2)
## Lachnospiraceae_[G.2]                DESeq2 man. geoMeans (ds2)
## Lachnospiraceae_[G.3]                DESeq2 man. geoMeans (ds2)
```

```
## Lachnospiraceae_[G.7]                DESeq2 man. geoMeans (ds2)
## Lachnospiraceae_[G.8]                DESeq2 man. geoMeans (ds2)
## Oribacterium                         DESeq2 man. geoMeans (ds2)
## Shuttleworthia                       DESeq2 man. geoMeans (ds2)
## Stomatobaculum                       DESeq2 man. geoMeans (ds2)
## Family.Lachnospiraceae_[XIV].        DESeq2 man. geoMeans (ds2)
## Peptococcus                          DESeq2 man. geoMeans (ds2)
## Parvimonas                           DESeq2 man. geoMeans (ds2)
## Filifactor                           DESeq2 man. geoMeans (ds2)
## Mogibacterium                        DESeq2 man. geoMeans (ds2)
## Peptostreptococcaceae_[XI][G.1]      DESeq2 man. geoMeans (ds2)
## Peptostreptococcaceae_[XI][G.4]      DESeq2 man. geoMeans (ds2)
## Peptostreptococcaceae_[XI][G.5]      DESeq2 man. geoMeans (ds2)
## Peptostreptococcaceae_[XI][G.6]      DESeq2 man. geoMeans (ds2)
## Peptostreptococcaceae_[XI][G.7]      DESeq2 man. geoMeans (ds2)
## Peptostreptococcaceae_[XI][G.9]      DESeq2 man. geoMeans (ds2)
## Peptostreptococcus                   DESeq2 man. geoMeans (ds2)
## Ruminococcaceae_[G.1]                DESeq2 man. geoMeans (ds2)
## Ruminococcaceae_[G.2]                DESeq2 man. geoMeans (ds2)
## Bulleidia                            DESeq2 man. geoMeans (ds2)
## Eggerthia                            DESeq2 man. geoMeans (ds2)
## Solobacterium                        DESeq2 man. geoMeans (ds2)
## Mycoplasma                           DESeq2 man. geoMeans (ds2)
## Mitsuokella                          DESeq2 man. geoMeans (ds2)
## Selenomonas                          DESeq2 man. geoMeans (ds2)
## Family.Selenomonadaceae.             DESeq2 man. geoMeans (ds2)
## Class.Negativicutes.                 DESeq2 man. geoMeans (ds2)
## Anaeroglobus                         DESeq2 man. geoMeans (ds2)
## Dialister                            DESeq2 man. geoMeans (ds2)
## Megasphaera                          DESeq2 man. geoMeans (ds2)
## Family.Veillonellaceae.              DESeq2 man. geoMeans (ds2)
## Veillonella                          DESeq2 man. geoMeans (ds2)
## Veillonellaceae_[G.1]                DESeq2 man. geoMeans (ds2)
## Phylum.Firmicutes.                   DESeq2 man. geoMeans (ds2)
## Fusobacterium                        DESeq2 man. geoMeans (ds2)
## Leptotrichia                         DESeq2 man. geoMeans (ds2)
## Gracilibacteria_(GN02)_[G.1]         DESeq2 man. geoMeans (ds2)
## Lautropia                            DESeq2 man. geoMeans (ds2)
## Ottowia                              DESeq2 man. geoMeans (ds2)
## Eikenella                            DESeq2 man. geoMeans (ds2)
## Kingella                             DESeq2 man. geoMeans (ds2)
## Neisseria                            DESeq2 man. geoMeans (ds2)
## Family.Neisseriaceae.                DESeq2 man. geoMeans (ds2)
## Campylobacter                        DESeq2 man. geoMeans (ds2)
## Cardiobacterium                      DESeq2 man. geoMeans (ds2)
## Aggregatibacter                      DESeq2 man. geoMeans (ds2)
## Haemophilus                          DESeq2 man. geoMeans (ds2)
## Family.Pasteurellaceae.              DESeq2 man. geoMeans (ds2)
## Saccharibacteria_(TM7)_[G.1]         DESeq2 man. geoMeans (ds2)
## Saccharibacteria_(TM7)_[G.3]         DESeq2 man. geoMeans (ds2)
## Saccharibacteria_(TM7)_[G.6]         DESeq2 man. geoMeans (ds2)
## Family.Saccharibacteria_(TM7)_[F-1]. DESeq2 man. geoMeans (ds2)
## Saccharibacteria_(TM7)_[G.5]         DESeq2 man. geoMeans (ds2)
## Treponema                            DESeq2 man. geoMeans (ds2)
```

```
## Fretibacterium                          DESeq2 man. geoMeans (ds2)
## Kingdom.Bacteria.                       DESeq2 man. geoMeans (ds2)
## Others                                  DESeq2 man. geoMeans (ds2)
```

```
write.table(final, file="bp_cat_ds2.txt", sep="\t", dec=",", row.names=F)
write.table(final[final$pval<0.05,], file="bp_cat_ds2_sig.txt", sep="\t", dec=",", row.names=F)
final <- DA.kru(Microbio2, predictor = Phe2$bp_cat)
final
```

```
##                                               pval  pval.adj
## Absconditabacteria_(SR1)_[G.1]         0.483947286 0.8952019
## Actinomyces                            0.649628975 0.9670887
## Peptidiphaga                           0.832365644 0.9670887
## Schaalia                               0.316899480 0.8952019
## Family.Actinomycetaceae.               0.909923467 0.9670887
## Rothia                                 0.221938066 0.7770259
## Alloscardovia                          0.682484878 0.9670887
## Bifidobacterium                        0.534325044 0.8952019
## Parascardovia                          0.680520081 0.9670887
## Scardovia                              0.387648185 0.8952019
## Corynebacterium                        0.833261566 0.9670887
## Olsenella                              0.976874941 0.9768749
## Atopobium                              0.177201416 0.7770259
## Slackia                                0.393178322 0.8952019
## Cryptobacterium                        0.903608894 0.9670887
## Bacteroidetes_[G.3]                    0.808459622 0.9670887
## Bacteroidetes_[G.5]                    0.349644829 0.8952019
## Family.Bacteroidetes_[F-1].            0.052258294 0.7770259
## Bacteroidaceae_[G.1]                   0.965643949 0.9757027
## Bacteroidales_[G.2]                    0.202648584 0.7770259
## Porphyromonas                          0.006561679 0.6364829
## Tannerella                             0.366090443 0.8952019
## Alloprevotella                         0.062852556 0.7770259
## Prevotella                             0.466580032 0.8952019
## Bergeyella                             0.161004314 0.7770259
## Capnocytophaga                         0.914498838 0.9670887
## Gemella                                0.324624439 0.8952019
## Abiotrophia                            0.129066444 0.7770259
## Granulicatella                         0.506092863 0.8952019
## Lactobacillus                          0.228940291 0.7770259
## Streptococcus                          0.205343652 0.7770259
## Order.Lactobacillales.                 0.495898270 0.8952019
## Class.Bacilli.                         0.508956243 0.8952019
## Clostridiales_[F.1][G-1]               0.378146060 0.8952019
## Butyrivibrio                           0.089011278 0.7770259
## Catonella                              0.902715092 0.9670887
## Johnsonella                            0.072613128 0.7770259
## Lachnoanaerobaculum                    0.511461802 0.8952019
## Lachnospiraceae_[G.2]                  0.071087015 0.7770259
## Lachnospiraceae_[G.3]                  0.407883515 0.8952019
## Lachnospiraceae_[G.7]                  0.732372908 0.9670887
## Lachnospiraceae_[G.8]                  0.827081312 0.9670887
## Oribacterium                           0.232306720 0.7770259
## Shuttleworthia                         0.542791010 0.8952019
```

```
## Stomatobaculum                        0.484391258 0.8952019
## Family.Lachnospiraceae_[XIV].         0.060681624 0.7770259
## Peptococcus                           0.894743113 0.9670887
## Parvimonas                            0.136926312 0.7770259
## Filifactor                            0.127204563 0.7770259
## Mogibacterium                         0.842186690 0.9670887
## Peptostreptococcaceae_[XI][G.1]       0.519207361 0.8952019
## Peptostreptococcaceae_[XI][G.4]       0.920515697 0.9670887
## Peptostreptococcaceae_[XI][G.5]       0.853094518 0.9670887
## Peptostreptococcaceae_[XI][G.6]       0.916403346 0.9670887
## Peptostreptococcaceae_[XI][G.7]       0.839746160 0.9670887
## Peptostreptococcaceae_[XI][G.9]       0.279766317 0.8480416
## Peptostreptococcus                    0.173878575 0.7770259
## Ruminococcaceae_[G.1]                 0.750875577 0.9670887
## Ruminococcaceae_[G.2]                 0.544504245 0.8952019
## Bulleidia                             0.522004784 0.8952019
## Eggerthia                             0.174707839 0.7770259
## Solobacterium                         0.179830123 0.7770259
## Mycoplasma                            0.157532092 0.7770259
## Mitsuokella                           0.653038327 0.9670887
## Selenomonas                           0.557282605 0.9009402
## Family.Selenomonadaceae.              0.392569613 0.8952019
## Class.Negativicutes.                  0.927208734 0.9670887
## Anaeroglobus                          0.247873567 0.8014579
## Dialister                             0.018587227 0.7770259
## Megasphaera                           0.091095804 0.7770259
## Family.Veillonellaceae.               0.268454254 0.8400020
## Veillonella                           0.684118326 0.9670887
## Veillonellaceae_[G.1]                 0.954649676 0.9747476
## Phylum.Firmicutes.                    0.455196550 0.8952019
## Fusobacterium                         0.846198696 0.9670887
## Leptotrichia                          0.421994879 0.8952019
## Gracilibacteria_(GN02)_[G.1]          0.947000421 0.9747476
## Lautropia                             0.613133246 0.9670887
## Ottowia                               0.727874378 0.9670887
## Eikenella                             0.429835386 0.8952019
## Kingella                              0.917080937 0.9670887
## Neisseria                             0.221373952 0.7770259
## Family.Neisseriaceae.                 0.296346720 0.8710798
## Campylobacter                         0.079375219 0.7770259
## Cardiobacterium                       0.836699541 0.9670887
## Aggregatibacter                       0.694050227 0.9670887
## Haemophilus                           0.483178340 0.8952019
## Family.Pasteurellaceae.               0.209094893 0.7770259
## Saccharibacteria_(TM7)_[G.1]          0.892665619 0.9670887
## Saccharibacteria_(TM7)_[G.3]          0.125310773 0.7770259
## Saccharibacteria_(TM7)_[G.6]          0.852022596 0.9670887
## Family.Saccharibacteria_(TM7)_[F-1]. 0.736388416 0.9670887
## Saccharibacteria_(TM7)_[G.5]          0.724115731 0.9670887
## Treponema                             0.844080344 0.9670887
## Fretibacterium                        0.472925098 0.8952019
## Kingdom.Bacteria.                     0.026716598 0.7770259
## Others                                0.182595584 0.7770259
##                                                                       Feature
```

```
## Absconditabacteria_(SR1)_[G.1]            Absconditabacteria_(SR1)_[G.1]
## Actinomyces                                                   Actinomyces
## Peptidiphaga                                                 Peptidiphaga
## Schaalia                                                         Schaalia
## Family.Actinomycetaceae.                         Family.Actinomycetaceae.
## Rothia                                                             Rothia
## Alloscardovia                                               Alloscardovia
## Bifidobacterium                                           Bifidobacterium
## Parascardovia                                               Parascardovia
## Scardovia                                                       Scardovia
## Corynebacterium                                           Corynebacterium
## Olsenella                                                       Olsenella
## Atopobium                                                       Atopobium
## Slackia                                                           Slackia
## Cryptobacterium                                           Cryptobacterium
## Bacteroidetes_[G.3]                                   Bacteroidetes_[G.3]
## Bacteroidetes_[G.5]                                   Bacteroidetes_[G.5]
## Family.Bacteroidetes_[F-1].                   Family.Bacteroidetes_[F-1].
## Bacteroidaceae_[G.1]                                 Bacteroidaceae_[G.1]
## Bacteroidales_[G.2]                                   Bacteroidales_[G.2]
## Porphyromonas                                               Porphyromonas
## Tannerella                                                     Tannerella
## Alloprevotella                                             Alloprevotella
## Prevotella                                                     Prevotella
## Bergeyella                                                     Bergeyella
## Capnocytophaga                                             Capnocytophaga
## Gemella                                                           Gemella
## Abiotrophia                                                   Abiotrophia
## Granulicatella                                             Granulicatella
## Lactobacillus                                               Lactobacillus
## Streptococcus                                               Streptococcus
## Order.Lactobacillales.                               Order.Lactobacillales.
## Class.Bacilli.                                             Class.Bacilli.
## Clostridiales_[F.1][G-1]                         Clostridiales_[F.1][G-1]
## Butyrivibrio                                                 Butyrivibrio
## Catonella                                                       Catonella
## Johnsonella                                                   Johnsonella
## Lachnoanaerobaculum                                   Lachnoanaerobaculum
## Lachnospiraceae_[G.2]                               Lachnospiraceae_[G.2]
## Lachnospiraceae_[G.3]                               Lachnospiraceae_[G.3]
## Lachnospiraceae_[G.7]                               Lachnospiraceae_[G.7]
## Lachnospiraceae_[G.8]                               Lachnospiraceae_[G.8]
## Oribacterium                                                 Oribacterium
## Shuttleworthia                                             Shuttleworthia
## Stomatobaculum                                             Stomatobaculum
## Family.Lachnospiraceae_[XIV].               Family.Lachnospiraceae_[XIV].
## Peptococcus                                                   Peptococcus
## Parvimonas                                                     Parvimonas
## Filifactor                                                     Filifactor
## Mogibacterium                                               Mogibacterium
## Peptostreptococcaceae_[XI][G.1]           Peptostreptococcaceae_[XI][G.1]
## Peptostreptococcaceae_[XI][G.4]           Peptostreptococcaceae_[XI][G.4]
## Peptostreptococcaceae_[XI][G.5]           Peptostreptococcaceae_[XI][G.5]
## Peptostreptococcaceae_[XI][G.6]           Peptostreptococcaceae_[XI][G.6]
```

```
## Peptostreptococcaceae_[XI][G.7]          Peptostreptococcaceae_[XI][G.7]
## Peptostreptococcaceae_[XI][G.9]          Peptostreptococcaceae_[XI][G.9]
## Peptostreptococcus                                    Peptostreptococcus
## Ruminococcaceae_[G.1]                              Ruminococcaceae_[G.1]
## Ruminococcaceae_[G.2]                              Ruminococcaceae_[G.2]
## Bulleidia                                                      Bulleidia
## Eggerthia                                                      Eggerthia
## Solobacterium                                              Solobacterium
## Mycoplasma                                                    Mycoplasma
## Mitsuokella                                                  Mitsuokella
## Selenomonas                                                  Selenomonas
## Family.Selenomonadaceae.                        Family.Selenomonadaceae.
## Class.Negativicutes.                                Class.Negativicutes.
## Anaeroglobus                                                Anaeroglobus
## Dialister                                                      Dialister
## Megasphaera                                                  Megasphaera
## Family.Veillonellaceae.                          Family.Veillonellaceae.
## Veillonella                                                  Veillonella
## Veillonellaceae_[G.1]                              Veillonellaceae_[G.1]
## Phylum.Firmicutes.                                    Phylum.Firmicutes.
## Fusobacterium                                              Fusobacterium
## Leptotrichia                                                Leptotrichia
## Gracilibacteria_(GN02)_[G.1]                Gracilibacteria_(GN02)_[G.1]
## Lautropia                                                      Lautropia
## Ottowia                                                          Ottowia
## Eikenella                                                      Eikenella
## Kingella                                                        Kingella
## Neisseria                                                      Neisseria
## Family.Neisseriaceae.                              Family.Neisseriaceae.
## Campylobacter                                              Campylobacter
## Cardiobacterium                                          Cardiobacterium
## Aggregatibacter                                          Aggregatibacter
## Haemophilus                                                  Haemophilus
## Family.Pasteurellaceae.                          Family.Pasteurellaceae.
## Saccharibacteria_(TM7)_[G.1]                Saccharibacteria_(TM7)_[G.1]
## Saccharibacteria_(TM7)_[G.3]                Saccharibacteria_(TM7)_[G.3]
## Saccharibacteria_(TM7)_[G.6]                Saccharibacteria_(TM7)_[G.6]
## Family.Saccharibacteria_(TM7)_[F-1]. Family.Saccharibacteria_(TM7)_[F-1].
## Saccharibacteria_(TM7)_[G.5]                Saccharibacteria_(TM7)_[G.5]
## Treponema                                                      Treponema
## Fretibacterium                                            Fretibacterium
## Kingdom.Bacteria.                                      Kingdom.Bacteria.
## Others                                                            Others
##                                                 Method
## Absconditabacteria_(SR1)_[G.1]       Kruskal-Wallis (kru)
## Actinomyces                          Kruskal-Wallis (kru)
## Peptidiphaga                         Kruskal-Wallis (kru)
## Schaalia                             Kruskal-Wallis (kru)
## Family.Actinomycetaceae.             Kruskal-Wallis (kru)
## Rothia                               Kruskal-Wallis (kru)
## Alloscardovia                        Kruskal-Wallis (kru)
## Bifidobacterium                      Kruskal-Wallis (kru)
## Parascardovia                        Kruskal-Wallis (kru)
## Scardovia                            Kruskal-Wallis (kru)
```

```
## Corynebacterium                        Kruskal-Wallis (kru)
## Olsenella                              Kruskal-Wallis (kru)
## Atopobium                              Kruskal-Wallis (kru)
## Slackia                                Kruskal-Wallis (kru)
## Cryptobacterium                        Kruskal-Wallis (kru)
## Bacteroidetes_[G.3]                    Kruskal-Wallis (kru)
## Bacteroidetes_[G.5]                    Kruskal-Wallis (kru)
## Family.Bacteroidetes_[F-1].            Kruskal-Wallis (kru)
## Bacteroidaceae_[G.1]                   Kruskal-Wallis (kru)
## Bacteroidales_[G.2]                    Kruskal-Wallis (kru)
## Porphyromonas                          Kruskal-Wallis (kru)
## Tannerella                             Kruskal-Wallis (kru)
## Alloprevotella                         Kruskal-Wallis (kru)
## Prevotella                             Kruskal-Wallis (kru)
## Bergeyella                             Kruskal-Wallis (kru)
## Capnocytophaga                         Kruskal-Wallis (kru)
## Gemella                                Kruskal-Wallis (kru)
## Abiotrophia                            Kruskal-Wallis (kru)
## Granulicatella                         Kruskal-Wallis (kru)
## Lactobacillus                          Kruskal-Wallis (kru)
## Streptococcus                          Kruskal-Wallis (kru)
## Order.Lactobacillales.                 Kruskal-Wallis (kru)
## Class.Bacilli.                         Kruskal-Wallis (kru)
## Clostridiales_[F.1][G-1]               Kruskal-Wallis (kru)
## Butyrivibrio                           Kruskal-Wallis (kru)
## Catonella                              Kruskal-Wallis (kru)
## Johnsonella                            Kruskal-Wallis (kru)
## Lachnoanaerobaculum                    Kruskal-Wallis (kru)
## Lachnospiraceae_[G.2]                  Kruskal-Wallis (kru)
## Lachnospiraceae_[G.3]                  Kruskal-Wallis (kru)
## Lachnospiraceae_[G.7]                  Kruskal-Wallis (kru)
## Lachnospiraceae_[G.8]                  Kruskal-Wallis (kru)
## Oribacterium                           Kruskal-Wallis (kru)
## Shuttleworthia                         Kruskal-Wallis (kru)
## Stomatobaculum                         Kruskal-Wallis (kru)
## Family.Lachnospiraceae_[XIV].          Kruskal-Wallis (kru)
## Peptococcus                            Kruskal-Wallis (kru)
## Parvimonas                             Kruskal-Wallis (kru)
## Filifactor                             Kruskal-Wallis (kru)
## Mogibacterium                          Kruskal-Wallis (kru)
## Peptostreptococcaceae_[XI][G.1]        Kruskal-Wallis (kru)
## Peptostreptococcaceae_[XI][G.4]        Kruskal-Wallis (kru)
## Peptostreptococcaceae_[XI][G.5]        Kruskal-Wallis (kru)
## Peptostreptococcaceae_[XI][G.6]        Kruskal-Wallis (kru)
## Peptostreptococcaceae_[XI][G.7]        Kruskal-Wallis (kru)
## Peptostreptococcaceae_[XI][G.9]        Kruskal-Wallis (kru)
## Peptostreptococcus                     Kruskal-Wallis (kru)
## Ruminococcaceae_[G.1]                  Kruskal-Wallis (kru)
## Ruminococcaceae_[G.2]                  Kruskal-Wallis (kru)
## Bulleidia                              Kruskal-Wallis (kru)
## Eggerthia                              Kruskal-Wallis (kru)
## Solobacterium                          Kruskal-Wallis (kru)
## Mycoplasma                             Kruskal-Wallis (kru)
## Mitsuokella                            Kruskal-Wallis (kru)
```

```
## Selenomonas                          Kruskal-Wallis (kru)
## Family.Selenomonadaceae.             Kruskal-Wallis (kru)
## Class.Negativicutes.                 Kruskal-Wallis (kru)
## Anaeroglobus                         Kruskal-Wallis (kru)
## Dialister                            Kruskal-Wallis (kru)
## Megasphaera                          Kruskal-Wallis (kru)
## Family.Veillonellaceae.              Kruskal-Wallis (kru)
## Veillonella                          Kruskal-Wallis (kru)
## Veillonellaceae_[G.1]                Kruskal-Wallis (kru)
## Phylum.Firmicutes.                   Kruskal-Wallis (kru)
## Fusobacterium                        Kruskal-Wallis (kru)
## Leptotrichia                         Kruskal-Wallis (kru)
## Gracilibacteria_(GN02)_[G.1]         Kruskal-Wallis (kru)
## Lautropia                            Kruskal-Wallis (kru)
## Ottowia                              Kruskal-Wallis (kru)
## Eikenella                            Kruskal-Wallis (kru)
## Kingella                             Kruskal-Wallis (kru)
## Neisseria                            Kruskal-Wallis (kru)
## Family.Neisseriaceae.                Kruskal-Wallis (kru)
## Campylobacter                        Kruskal-Wallis (kru)
## Cardiobacterium                      Kruskal-Wallis (kru)
## Aggregatibacter                      Kruskal-Wallis (kru)
## Haemophilus                          Kruskal-Wallis (kru)
## Family.Pasteurellaceae.              Kruskal-Wallis (kru)
## Saccharibacteria_(TM7)_[G.1]         Kruskal-Wallis (kru)
## Saccharibacteria_(TM7)_[G.3]         Kruskal-Wallis (kru)
## Saccharibacteria_(TM7)_[G.6]         Kruskal-Wallis (kru)
## Family.Saccharibacteria_(TM7)_[F-1]. Kruskal-Wallis (kru)
## Saccharibacteria_(TM7)_[G.5]         Kruskal-Wallis (kru)
## Treponema                            Kruskal-Wallis (kru)
## Fretibacterium                       Kruskal-Wallis (kru)
## Kingdom.Bacteria.                    Kruskal-Wallis (kru)
## Others                               Kruskal-Wallis (kru)
```

```
write.table(final, file="bp_cat_kru.txt", sep="\t", dec=",", row.names=F)
write.table(final[final$pval<0.05,], file="bp_cat_kru_sig.txt", sep="\t", dec=",", row.names=F)
#################sbp_cat
table(Phe$sbp_cat, useNA="always")
```

```
##
##            Low          Normal   Mild_elevated Moderate_severe            <NA>
##            175             347             179              43               2
```

```
#Remove NA
Phe2<-Phe[complete.cases(Phe$sbp_cat),]
#Also subset columns
Microbio2<-dplyr::select(Microbio, one_of(Phe2$IDX))
final <- DA.ds2(Microbio2, predictor = Phe2$sbp_cat, out.all=TRUE) #It is LRT so don't look at ordering
final
```

```
##                                          baseMean log2FoldChange      lfcSE
## Absconditabacteria_(SR1)_[G.1]         60.8782745   -0.4581857709 0.30582130
## Actinomyces                           323.7803206   -0.0734477379 0.10513779
```

```
## Peptidiphaga                              22.0416883   0.1559515655 0.15025119
## Schaalia                                 703.5570032   0.0842229955 0.08646227
## Family.Actinomycetaceae.                   5.4318947  -0.1235790191 0.21658069
## Rothia                                  1938.6926786  -0.2715934834 0.10104499
## Alloscardovia                             12.0844404  -0.0621284779 0.26450130
## Bifidobacterium                           10.7284828   0.2118211303 0.29071461
## Parascardovia                              2.9264171   0.9323335207 0.40454351
## Scardovia                                 13.5083615   0.5152311834 0.25796925
## Corynebacterium                           93.8520152  -0.0642111925 0.11088270
## Olsenella                                  1.5705319   0.0740339832 0.24623224
## Atopobium                                120.2270723   0.0047115178 0.10143731
## Slackia                                    1.3929535   0.3326524422 0.23900241
## Cryptobacterium                            4.2513998   0.1012108815 0.25235409
## Bacteroidetes_[G.3]                        6.6074797   0.2419044808 0.26627153
## Bacteroidetes_[G.5]                        4.6913133   0.2880964445 0.28980003
## Family.Bacteroidetes_[F-1].                0.8456413  -0.2905019563 0.41206323
## Bacteroidaceae_[G.1]                       0.7948120  -0.2422017061 0.39413394
## Bacteroidales_[G.2]                       12.0719990   0.1760513466 0.15277080
## Porphyromonas                            682.2933394  -0.0268262300 0.13872295
## Tannerella                                22.0983847   0.0178364109 0.10291105
## Alloprevotella                           362.5219516   0.0682376391 0.11069161
## Prevotella                              3807.2361865   0.0514262038 0.07019611
## Bergeyella                                72.7155861  -0.0064019544 0.10832779
## Capnocytophaga                           225.4613668  -0.0499264049 0.11599433
## Gemella                                  309.8006772  -0.2160442844 0.09622728
## Abiotrophia                               30.0741451  -0.0903151582 0.20880539
## Granulicatella                           306.3404328  -0.0767733618 0.08444716
## Lactobacillus                             22.7382734   0.7019858025 0.31623988
## Streptococcus                           3731.9524647   0.0143768820 0.07640881
## Order.Lactobacillales.                     0.2871119  -0.0432341339 0.28660489
## Class.Bacilli.                             0.5755295  -0.3020829209 0.22655830
## Clostridiales_[F.1][G-1]                   1.0579178   0.4235949593 0.34854712
## Butyrivibrio                               6.9656228  -0.0445285744 0.22115297
## Catonella                                 36.9095724   0.0365949928 0.12462368
## Johnsonella                                0.9421596   0.4471660932 0.30532019
## Lachnoanaerobaculum                       87.8820004   0.1126615666 0.08725974
## Lachnospiraceae_[G.2]                     28.0534664   0.1819176249 0.22855528
## Lachnospiraceae_[G.3]                      2.8995123   0.3470184287 0.23814614
## Lachnospiraceae_[G.7]                      1.4732956   0.2075522271 0.34247711
## Lachnospiraceae_[G.8]                      1.4367939  -0.0199839317 0.36022726
## Oribacterium                             147.1321858   0.0348710914 0.09667494
## Shuttleworthia                             2.8108966   0.0675469902 0.26961378
## Stomatobaculum                           112.9182555   0.0787101386 0.10851488
## Family.Lachnospiraceae_[XIV].              1.5138789   0.3526785810 0.29954417
## Peptococcus                                3.7157473   0.0648705310 0.27012854
## Parvimonas                                40.9495178   0.1348015972 0.13390291
## Filifactor                                 6.8621336  -0.0521263184 0.26109184
## Mogibacterium                             41.1723089   0.0840296995 0.09259546
## Peptostreptococcaceae_[XI][G.1]           67.7192574   0.1185931350 0.16469758
## Peptostreptococcaceae_[XI][G.4]            0.4191905   0.0058078145 0.35677059
## Peptostreptococcaceae_[XI][G.5]            1.6690812   0.3450778527 0.37466016
## Peptostreptococcaceae_[XI][G.6]            1.0175853  -0.1224318960 0.37065231
## Peptostreptococcaceae_[XI][G.7]            4.7195993   0.1037319800 0.25730420
## Peptostreptococcaceae_[XI][G.9]            8.7363992  -0.0379006635 0.15293699
```

```
## Peptostreptococcus                     42.9864069  -0.0149790349 0.19278484
## Ruminococcaceae_[G.1]                  25.1836759  -0.0331915887 0.19585200
## Ruminococcaceae_[G.2]                  41.5234417   0.4033362899 0.16300061
## Bulleidia                               3.8709078  -0.2185630199 0.28748325
## Eggerthia                               1.5849632   0.4400078485 0.38872846
## Solobacterium                          74.1630338  -0.1145612903 0.10883459
## Mycoplasma                              4.5000508   0.1439625711 0.23043587
## Mitsuokella                            26.7544753   0.0580037022 0.20622511
## Selenomonas                           222.3185156   0.0680886504 0.09296291
## Family.Selenomonadaceae.                5.0504886  -0.0536984602 0.24249544
## Class.Negativicutes.                    0.7322999   0.1344628735 0.21946308
## Anaeroglobus                            8.7028189   0.5225132542 0.22282371
## Dialister                              36.4852787   0.1912497060 0.11414346
## Megasphaera                           407.7957370   0.2538601870 0.11942174
## Family.Veillonellaceae.                 0.5239301   0.4279254535 0.26494573
## Veillonella                          5061.2445718   0.0005203703 0.06753038
## Veillonellaceae_[G.1]                   6.1469526   0.2196985218 0.18823161
## Phylum.Firmicutes.                      0.8918217  -0.4089982592 0.30101780
## Fusobacterium                         929.2571372   0.0671064098 0.08990014
## Leptotrichia                          684.6054135  -0.0406357445 0.09248538
## Gracilibacteria_(GN02)_[G.1]            2.4161467  -0.3510203092 0.29715562
## Lautropia                              68.4994208   0.0042394131 0.17217959
## Ottowia                                 2.2349835   0.7537869579 0.38420073
## Eikenella                               8.7703431   0.2499101249 0.14470559
## Kingella                              100.0995471  -0.1443564812 0.12373641
## Neisseria                            1333.1334336  -0.0591378820 0.14102398
## Family.Neisseriaceae.                   6.1192335  -0.0950703786 0.33072175
## Campylobacter                         257.7162863   0.0091811759 0.07932330
## Cardiobacterium                        14.8139218   0.0046805885 0.12317669
## Aggregatibacter                       276.1135518  -0.0736243339 0.16394324
## Haemophilus                          2911.7582507  -0.2441107550 0.10899524
## Family.Pasteurellaceae.                 2.5966784  -0.2124275372 0.44906010
## Saccharibacteria_(TM7)_[G.1]           36.1574542   0.2139979744 0.18203444
## Saccharibacteria_(TM7)_[G.3]            7.1401803   0.0353815412 0.25377430
## Saccharibacteria_(TM7)_[G.6]            7.2298310   0.0132822806 0.32411786
## Family.Saccharibacteria_(TM7)_[F-1].    1.1040897   0.2028593579 0.40551173
## Saccharibacteria_(TM7)_[G.5]            0.9908292   0.4132138912 0.38942406
## Treponema                              63.3764075   0.1295510142 0.17270840
## Fretibacterium                          8.2967790  -0.1073327732 0.20909253
## Kingdom.Bacteria.                       9.4046214   0.2210888706 0.26500144
## Others                                 33.2400828  -0.4743836754 0.17508680
##                                            stat        pval   ordering
## Absconditabacteria_(SR1)_[G.1]       15.6376892 0.001345370 Low>Normal
## Actinomyces                           3.3128216 0.345862106 Low>Normal
## Peptidiphaga                          2.5794321 0.461106784 Normal>Low
## Schaalia                              3.8823512 0.274451521 Normal>Low
## Family.Actinomycetaceae.              2.5077999 0.473883107 Low>Normal
## Rothia                                8.7988775 0.032087954 Low>Normal
## Alloscardovia                         5.8852070 0.117330695 Low>Normal
## Bifidobacterium                       1.1817422 0.757386533 Normal>Low
## Parascardovia                         6.7572897 0.080049586 Normal>Low
## Scardovia                             5.4388254 0.142343665 Normal>Low
## Corynebacterium                       2.5245680 0.470867446 Low>Normal
## Olsenella                             2.0642273 0.559183647 Normal>Low
```

```
## Atopobium                            4.6791889 0.196853188 Normal>Low
## Slackia                              4.2139476 0.239269173 Normal>Low
## Cryptobacterium                      0.9141409 0.822014023 Normal>Low
## Bacteroidetes_[G.3]                  4.5162091 0.210849020 Normal>Low
## Bacteroidetes_[G.5]                  7.5465176 0.056375185 Normal>Low
## Family.Bacteroidetes_[F-1].          8.4625822 0.037359059 Low>Normal
## Bacteroidaceae_[G.1]                 1.0287643 0.794292310 Low>Normal
## Bacteroidales_[G.2]                  1.5523208 0.670248414 Normal>Low
## Porphyromonas                        5.7785107 0.122897721 Low>Normal
## Tannerella                           3.0453242 0.384689731 Normal>Low
## Alloprevotella                       0.6126305 0.893534914 Normal>Low
## Prevotella                           2.3007666 0.512374057 Normal>Low
## Bergeyella                           3.3452302 0.341396889 Low>Normal
## Capnocytophaga                       1.1250397 0.771033092 Low>Normal
## Gemella                              5.6392748 0.130541729 Low>Normal
## Abiotrophia                          3.8056677 0.283227566 Low>Normal
## Granulicatella                       2.6293738 0.452363196 Low>Normal
## Lactobacillus                       15.6906252 0.001312204 Normal>Low
## Streptococcus                        1.3879711 0.708356790 Normal>Low
## Order.Lactobacillales.              -3.7663483 1.000000000 Low>Normal
## Class.Bacilli.                      14.5686784 0.002224928 Low>Normal
## Clostridiales_[F.1][G-1]             4.5960907 0.203877699 Normal>Low
## Butyrivibrio                         0.3783719 0.944673211 Low>Normal
## Catonella                            2.0322641 0.565737255 Normal>Low
## Johnsonella                          2.8376083 0.417346279 Normal>Low
## Lachnoanaerobaculum                  1.8823438 0.597181973 Normal>Low
## Lachnospiraceae_[G.2]                3.1203526 0.373438823 Normal>Low
## Lachnospiraceae_[G.3]                2.7542710 0.431083151 Normal>Low
## Lachnospiraceae_[G.7]                3.5781697 0.310764393 Normal>Low
## Lachnospiraceae_[G.8]                3.2269623 0.357938172 Low>Normal
## Oribacterium                         0.5882418 0.899118787 Normal>Low
## Shuttleworthia                       0.7604843 0.858893399 Normal>Low
## Stomatobaculum                       3.5807545 0.310438573 Normal>Low
## Family.Lachnospiraceae_[XIV].        7.9503171 0.047049739 Normal>Low
## Peptococcus                          0.4569815 0.928235475 Normal>Low
## Parvimonas                           2.9958093 0.392271756 Normal>Low
## Filifactor                           1.6376345 0.650886882 Low>Normal
## Mogibacterium                        2.4394629 0.486330885 Normal>Low
## Peptostreptococcaceae_[XI][G.1]      1.8137075 0.611956572 Normal>Low
## Peptostreptococcaceae_[XI][G.4]      0.2332576 0.972049797 Normal>Low
## Peptostreptococcaceae_[XI][G.5]      4.4269998 0.218894899 Normal>Low
## Peptostreptococcaceae_[XI][G.6]      0.9617238 0.810512522 Low>Normal
## Peptostreptococcaceae_[XI][G.7]      2.9585030 0.398067550 Normal>Low
## Peptostreptococcaceae_[XI][G.9]      1.9755459 0.577497727 Low>Normal
## Peptostreptococcus                   0.0844978 0.993630514 Low>Normal
## Ruminococcaceae_[G.1]                2.8696411 0.412163688 Low>Normal
## Ruminococcaceae_[G.2]                6.7591396 0.079984208 Normal>Low
## Bulleidia                            0.6378488 0.887715670 Low>Normal
## Eggerthia                            1.7681416 0.621892106 Normal>Low
## Solobacterium                        2.7692124 0.428593211 Low>Normal
## Mycoplasma                           2.0490558 0.562287670 Normal>Low
## Mitsuokella                          3.8186089 0.281729009 Normal>Low
## Selenomonas                          0.6223678 0.891293221 Normal>Low
## Family.Selenomonadaceae.             3.8606544 0.276909399 Low>Normal
```

```
## Class.Negativicutes.                    8.8299697 0.031639070 Normal>Low
## Anaeroglobus                            7.0624428 0.069933917 Normal>Low
## Dialister                               3.6855875 0.297477639 Normal>Low
## Megasphaera                            14.0513097 0.002836137 Normal>Low
## Family.Veillonellaceae.                 3.3991207 0.334083425 Normal>Low
## Veillonella                             1.9453421 0.583828288 Normal>Low
## Veillonellaceae_[G.1]                   2.9031324 0.406802634 Normal>Low
## Phylum.Firmicutes.                     11.2782127 0.010312784 Low>Normal
## Fusobacterium                           1.4730874 0.688495518 Normal>Low
## Leptotrichia                            5.0995612 0.164650273 Low>Normal
## Gracilibacteria_(GN02)_[G.1]            6.9747044 0.072708413 Low>Normal
## Lautropia                               2.1009003 0.551730645 Normal>Low
## Ottowia                                 6.1368671 0.105137930 Normal>Low
## Eikenella                               3.4295748 0.330011549 Normal>Low
## Kingella                                7.9224644 0.047641609 Low>Normal
## Neisseria                               4.0589361 0.255170018 Low>Normal
## Family.Neisseriaceae.                   1.2625419 0.738046504 Low>Normal
## Campylobacter                           7.4199453 0.059651474 Normal>Low
## Cardiobacterium                        12.7588964 0.005188106 Normal>Low
## Aggregatibacter                         0.5080559 0.917118041 Low>Normal
## Haemophilus                             6.0917558 0.107230447 Low>Normal
## Family.Pasteurellaceae.                 5.5914164 0.133272002 Low>Normal
## Saccharibacteria_(TM7)_[G.1]            4.2204651 0.238620906 Normal>Low
## Saccharibacteria_(TM7)_[G.3]            2.9746357 0.395552411 Normal>Low
## Saccharibacteria_(TM7)_[G.6]            0.7012100 0.872919314 Normal>Low
## Family.Saccharibacteria_(TM7)_[F-1].    1.1439885 0.766467067 Normal>Low
## Saccharibacteria_(TM7)_[G.5]            2.2387413 0.524357905 Normal>Low
## Treponema                               4.2459437 0.236102116 Normal>Low
## Fretibacterium                          0.5337438 0.911417907 Low>Normal
## Kingdom.Bacteria.                       1.6762619 0.642224159 Normal>Low
## Others                                 13.0692237 0.004489272 Low>Normal
##                                          pval.adj
## Absconditabacteria_(SR1)_[G.1]         0.06525047
## Actinomyces                            0.74552498
## Peptidiphaga                           0.76611102
## Schaalia                               0.72297563
## Family.Actinomycetaceae.               0.76611102
## Rothia                                 0.34583684
## Alloscardovia                          0.53864101
## Bifidobacterium                        0.92333593
## Parascardovia                          0.43137833
## Scardovia                              0.55229342
## Corynebacterium                        0.76611102
## Olsenella                              0.81905244
## Atopobium                              0.70330636
## Slackia                                0.70330636
## Cryptobacterium                        0.94923048
## Bacteroidetes_[G.3]                    0.70330636
## Bacteroidetes_[G.5]                    0.41329950
## Family.Bacteroidetes_[F-1].            0.36238287
## Bacteroidaceae_[G.1]                   0.93958968
## Bacteroidales_[G.2]                    0.86685461
## Porphyromonas                          0.53864101
## Tannerella                             0.74669760
```

```
## Alloprevotella                        0.96696141
## Prevotella                            0.80161748
## Bergeyella                            0.74552498
## Capnocytophaga                        0.92333593
## Gemella                               0.53864101
## Abiotrophia                           0.72297563
## Granulicatella                        0.76611102
## Lactobacillus                         0.06525047
## Streptococcus                         0.89234557
## Order.Lactobacillales.                1.00000000
## Class.Bacilli.                        0.06877632
## Clostridiales_[F.1][G-1]              0.70330636
## Butyrivibrio                          0.97482236
## Catonella                             0.81905244
## Johnsonella                           0.74669760
## Lachnoanaerobaculum                   0.82752359
## Lachnospiraceae_[G.2]                 0.74669760
## Lachnospiraceae_[G.3]                 0.74669760
## Lachnospiraceae_[G.7]                 0.73522308
## Lachnospiraceae_[G.8]                 0.74669760
## Oribacterium                          0.96696141
## Shuttleworthia                        0.96696141
## Stomatobaculum                        0.73522308
## Family.Lachnospiraceae_[XIV].         0.38510301
## Peptococcus                           0.96815958
## Parvimonas                            0.74669760
## Filifactor                            0.85318956
## Mogibacterium                         0.77334583
## Peptostreptococcaceae_[XI][G.1]       0.83605335
## Peptostreptococcaceae_[XI][G.4]       0.99251400
## Peptostreptococcaceae_[XI][G.5]       0.70330636
## Peptostreptococcaceae_[XI][G.6]       0.94722548
## Peptostreptococcaceae_[XI][G.7]       0.74669760
## Peptostreptococcaceae_[XI][G.9]       0.82074412
## Peptostreptococcus                    1.00000000
## Ruminococcaceae_[G.1]                 0.74669760
## Ruminococcaceae_[G.2]                 0.43137833
## Bulleidia                             0.96696141
## Eggerthia                             0.83782686
## Solobacterium                         0.74669760
## Mycoplasma                            0.81905244
## Mitsuokella                           0.72297563
## Selenomonas                           0.96696141
## Family.Selenomonadaceae.              0.72297563
## Class.Negativicutes.                  0.34583684
## Anaeroglobus                          0.43137833
## Dialister                             0.73522308
## Megasphaera                           0.06877632
## Family.Veillonellaceae.               0.74552498
## Veillonella                           0.82074412
## Veillonellaceae_[G.1]                 0.74669760
## Phylum.Firmicutes.                    0.14290572
## Fusobacterium                         0.87873770
## Leptotrichia                          0.61427217
```

```
## Gracilibacteria_(GN02)_[G.1]         0.43137833
## Lautropia                            0.81905244
## Ottowia                              0.52006767
## Eikenella                            0.74552498
## Kingella                             0.38510301
## Neisseria                            0.72297563
## Family.Neisseriaceae.                0.91782706
## Campylobacter                        0.41329950
## Cardiobacterium                      0.08387439
## Aggregatibacter                      0.96696141
## Haemophilus                          0.52006767
## Family.Pasteurellaceae.              0.53864101
## Saccharibacteria_(TM7)_[G.1]         0.70330636
## Saccharibacteria_(TM7)_[G.3]         0.74669760
## Saccharibacteria_(TM7)_[G.6]         0.96696141
## Family.Saccharibacteria_(TM7)_[F-1]. 0.92333593
## Saccharibacteria_(TM7)_[G.5]         0.80734471
## Treponema                            0.70330636
## Fretibacterium                       0.96696141
## Kingdom.Bacteria.                    0.85318956
## Others                               0.08387439
##                                                                   Feature
## Absconditabacteria_(SR1)_[G.1]         Absconditabacteria_(SR1)_[G.1]
## Actinomyces                                                   Actinomyces
## Peptidiphaga                                                 Peptidiphaga
## Schaalia                                                         Schaalia
## Family.Actinomycetaceae.                         Family.Actinomycetaceae.
## Rothia                                                             Rothia
## Alloscardovia                                               Alloscardovia
## Bifidobacterium                                           Bifidobacterium
## Parascardovia                                               Parascardovia
## Scardovia                                                       Scardovia
## Corynebacterium                                           Corynebacterium
## Olsenella                                                       Olsenella
## Atopobium                                                       Atopobium
## Slackia                                                           Slackia
## Cryptobacterium                                           Cryptobacterium
## Bacteroidetes_[G.3]                                   Bacteroidetes_[G.3]
## Bacteroidetes_[G.5]                                   Bacteroidetes_[G.5]
## Family.Bacteroidetes_[F-1].                   Family.Bacteroidetes_[F-1].
## Bacteroidaceae_[G.1]                                 Bacteroidaceae_[G.1]
## Bacteroidales_[G.2]                                   Bacteroidales_[G.2]
## Porphyromonas                                               Porphyromonas
## Tannerella                                                     Tannerella
## Alloprevotella                                             Alloprevotella
## Prevotella                                                     Prevotella
## Bergeyella                                                     Bergeyella
## Capnocytophaga                                             Capnocytophaga
## Gemella                                                           Gemella
## Abiotrophia                                                   Abiotrophia
## Granulicatella                                             Granulicatella
## Lactobacillus                                               Lactobacillus
## Streptococcus                                               Streptococcus
## Order.Lactobacillales.                             Order.Lactobacillales.
```

```
## Class.Bacilli.                                                      Class.Bacilli.
## Clostridiales_[F.1][G-1]                                Clostridiales_[F.1][G-1]
## Butyrivibrio                                                         Butyrivibrio
## Catonella                                                               Catonella
## Johnsonella                                                           Johnsonella
## Lachnoanaerobaculum                                           Lachnoanaerobaculum
## Lachnospiraceae_[G.2]                                       Lachnospiraceae_[G.2]
## Lachnospiraceae_[G.3]                                       Lachnospiraceae_[G.3]
## Lachnospiraceae_[G.7]                                       Lachnospiraceae_[G.7]
## Lachnospiraceae_[G.8]                                       Lachnospiraceae_[G.8]
## Oribacterium                                                         Oribacterium
## Shuttleworthia                                                     Shuttleworthia
## Stomatobaculum                                                     Stomatobaculum
## Family.Lachnospiraceae_[XIV].                       Family.Lachnospiraceae_[XIV].
## Peptococcus                                                           Peptococcus
## Parvimonas                                                             Parvimonas
## Filifactor                                                             Filifactor
## Mogibacterium                                                       Mogibacterium
## Peptostreptococcaceae_[XI][G.1]                 Peptostreptococcaceae_[XI][G.1]
## Peptostreptococcaceae_[XI][G.4]                 Peptostreptococcaceae_[XI][G.4]
## Peptostreptococcaceae_[XI][G.5]                 Peptostreptococcaceae_[XI][G.5]
## Peptostreptococcaceae_[XI][G.6]                 Peptostreptococcaceae_[XI][G.6]
## Peptostreptococcaceae_[XI][G.7]                 Peptostreptococcaceae_[XI][G.7]
## Peptostreptococcaceae_[XI][G.9]                 Peptostreptococcaceae_[XI][G.9]
## Peptostreptococcus                                             Peptostreptococcus
## Ruminococcaceae_[G.1]                                       Ruminococcaceae_[G.1]
## Ruminococcaceae_[G.2]                                       Ruminococcaceae_[G.2]
## Bulleidia                                                               Bulleidia
## Eggerthia                                                               Eggerthia
## Solobacterium                                                       Solobacterium
## Mycoplasma                                                             Mycoplasma
## Mitsuokella                                                           Mitsuokella
## Selenomonas                                                           Selenomonas
## Family.Selenomonadaceae.                                 Family.Selenomonadaceae.
## Class.Negativicutes.                                         Class.Negativicutes.
## Anaeroglobus                                                         Anaeroglobus
## Dialister                                                               Dialister
## Megasphaera                                                           Megasphaera
## Family.Veillonellaceae.                                   Family.Veillonellaceae.
## Veillonella                                                           Veillonella
## Veillonellaceae_[G.1]                                       Veillonellaceae_[G.1]
## Phylum.Firmicutes.                                             Phylum.Firmicutes.
## Fusobacterium                                                       Fusobacterium
## Leptotrichia                                                         Leptotrichia
## Gracilibacteria_(GN02)_[G.1]                       Gracilibacteria_(GN02)_[G.1]
## Lautropia                                                               Lautropia
## Ottowia                                                                   Ottowia
## Eikenella                                                               Eikenella
## Kingella                                                                 Kingella
## Neisseria                                                               Neisseria
## Family.Neisseriaceae.                                       Family.Neisseriaceae.
## Campylobacter                                                       Campylobacter
## Cardiobacterium                                                   Cardiobacterium
## Aggregatibacter                                                   Aggregatibacter
```

```
## Haemophilus                                                         Haemophilus
## Family.Pasteurellaceae.                                 Family.Pasteurellaceae.
## Saccharibacteria_(TM7)_[G.1]                       Saccharibacteria_(TM7)_[G.1]
## Saccharibacteria_(TM7)_[G.3]                       Saccharibacteria_(TM7)_[G.3]
## Saccharibacteria_(TM7)_[G.6]                       Saccharibacteria_(TM7)_[G.6]
## Family.Saccharibacteria_(TM7)_[F-1]. Family.Saccharibacteria_(TM7)_[F-1].
## Saccharibacteria_(TM7)_[G.5]                       Saccharibacteria_(TM7)_[G.5]
## Treponema                                                             Treponema
## Fretibacterium                                                   Fretibacterium
## Kingdom.Bacteria.                                             Kingdom.Bacteria.
## Others                                                                   Others
##                                                              Method
## Absconditabacteria_(SR1)_[G.1]       DESeq2 man. geoMeans (ds2)
## Actinomyces                          DESeq2 man. geoMeans (ds2)
## Peptidiphaga                         DESeq2 man. geoMeans (ds2)
## Schaalia                             DESeq2 man. geoMeans (ds2)
## Family.Actinomycetaceae.             DESeq2 man. geoMeans (ds2)
## Rothia                               DESeq2 man. geoMeans (ds2)
## Alloscardovia                        DESeq2 man. geoMeans (ds2)
## Bifidobacterium                      DESeq2 man. geoMeans (ds2)
## Parascardovia                        DESeq2 man. geoMeans (ds2)
## Scardovia                            DESeq2 man. geoMeans (ds2)
## Corynebacterium                      DESeq2 man. geoMeans (ds2)
## Olsenella                            DESeq2 man. geoMeans (ds2)
## Atopobium                            DESeq2 man. geoMeans (ds2)
## Slackia                              DESeq2 man. geoMeans (ds2)
## Cryptobacterium                      DESeq2 man. geoMeans (ds2)
## Bacteroidetes_[G.3]                  DESeq2 man. geoMeans (ds2)
## Bacteroidetes_[G.5]                  DESeq2 man. geoMeans (ds2)
## Family.Bacteroidetes_[F-1].          DESeq2 man. geoMeans (ds2)
## Bacteroidaceae_[G.1]                 DESeq2 man. geoMeans (ds2)
## Bacteroidales_[G.2]                  DESeq2 man. geoMeans (ds2)
## Porphyromonas                        DESeq2 man. geoMeans (ds2)
## Tannerella                           DESeq2 man. geoMeans (ds2)
## Alloprevotella                       DESeq2 man. geoMeans (ds2)
## Prevotella                           DESeq2 man. geoMeans (ds2)
## Bergeyella                           DESeq2 man. geoMeans (ds2)
## Capnocytophaga                       DESeq2 man. geoMeans (ds2)
## Gemella                              DESeq2 man. geoMeans (ds2)
## Abiotrophia                          DESeq2 man. geoMeans (ds2)
## Granulicatella                       DESeq2 man. geoMeans (ds2)
## Lactobacillus                        DESeq2 man. geoMeans (ds2)
## Streptococcus                        DESeq2 man. geoMeans (ds2)
## Order.Lactobacillales.               DESeq2 man. geoMeans (ds2)
## Class.Bacilli.                       DESeq2 man. geoMeans (ds2)
## Clostridiales_[F.1][G-1]             DESeq2 man. geoMeans (ds2)
## Butyrivibrio                         DESeq2 man. geoMeans (ds2)
## Catonella                            DESeq2 man. geoMeans (ds2)
## Johnsonella                          DESeq2 man. geoMeans (ds2)
## Lachnoanaerobaculum                  DESeq2 man. geoMeans (ds2)
## Lachnospiraceae_[G.2]                DESeq2 man. geoMeans (ds2)
## Lachnospiraceae_[G.3]                DESeq2 man. geoMeans (ds2)
## Lachnospiraceae_[G.7]                DESeq2 man. geoMeans (ds2)
## Lachnospiraceae_[G.8]                DESeq2 man. geoMeans (ds2)
```

```
## Oribacterium                           DESeq2 man. geoMeans (ds2)
## Shuttleworthia                         DESeq2 man. geoMeans (ds2)
## Stomatobaculum                         DESeq2 man. geoMeans (ds2)
## Family.Lachnospiraceae_[XIV].          DESeq2 man. geoMeans (ds2)
## Peptococcus                            DESeq2 man. geoMeans (ds2)
## Parvimonas                             DESeq2 man. geoMeans (ds2)
## Filifactor                             DESeq2 man. geoMeans (ds2)
## Mogibacterium                          DESeq2 man. geoMeans (ds2)
## Peptostreptococcaceae_[XI][G.1]        DESeq2 man. geoMeans (ds2)
## Peptostreptococcaceae_[XI][G.4]        DESeq2 man. geoMeans (ds2)
## Peptostreptococcaceae_[XI][G.5]        DESeq2 man. geoMeans (ds2)
## Peptostreptococcaceae_[XI][G.6]        DESeq2 man. geoMeans (ds2)
## Peptostreptococcaceae_[XI][G.7]        DESeq2 man. geoMeans (ds2)
## Peptostreptococcaceae_[XI][G.9]        DESeq2 man. geoMeans (ds2)
## Peptostreptococcus                     DESeq2 man. geoMeans (ds2)
## Ruminococcaceae_[G.1]                  DESeq2 man. geoMeans (ds2)
## Ruminococcaceae_[G.2]                  DESeq2 man. geoMeans (ds2)
## Bulleidia                              DESeq2 man. geoMeans (ds2)
## Eggerthia                              DESeq2 man. geoMeans (ds2)
## Solobacterium                          DESeq2 man. geoMeans (ds2)
## Mycoplasma                             DESeq2 man. geoMeans (ds2)
## Mitsuokella                            DESeq2 man. geoMeans (ds2)
## Selenomonas                            DESeq2 man. geoMeans (ds2)
## Family.Selenomonadaceae.               DESeq2 man. geoMeans (ds2)
## Class.Negativicutes.                   DESeq2 man. geoMeans (ds2)
## Anaeroglobus                           DESeq2 man. geoMeans (ds2)
## Dialister                              DESeq2 man. geoMeans (ds2)
## Megasphaera                            DESeq2 man. geoMeans (ds2)
## Family.Veillonellaceae.                DESeq2 man. geoMeans (ds2)
## Veillonella                            DESeq2 man. geoMeans (ds2)
## Veillonellaceae_[G.1]                  DESeq2 man. geoMeans (ds2)
## Phylum.Firmicutes.                     DESeq2 man. geoMeans (ds2)
## Fusobacterium                          DESeq2 man. geoMeans (ds2)
## Leptotrichia                           DESeq2 man. geoMeans (ds2)
## Gracilibacteria_(GN02)_[G.1]           DESeq2 man. geoMeans (ds2)
## Lautropia                              DESeq2 man. geoMeans (ds2)
## Ottowia                                DESeq2 man. geoMeans (ds2)
## Eikenella                              DESeq2 man. geoMeans (ds2)
## Kingella                               DESeq2 man. geoMeans (ds2)
## Neisseria                              DESeq2 man. geoMeans (ds2)
## Family.Neisseriaceae.                  DESeq2 man. geoMeans (ds2)
## Campylobacter                          DESeq2 man. geoMeans (ds2)
## Cardiobacterium                        DESeq2 man. geoMeans (ds2)
## Aggregatibacter                        DESeq2 man. geoMeans (ds2)
## Haemophilus                            DESeq2 man. geoMeans (ds2)
## Family.Pasteurellaceae.                DESeq2 man. geoMeans (ds2)
## Saccharibacteria_(TM7)_[G.1]           DESeq2 man. geoMeans (ds2)
## Saccharibacteria_(TM7)_[G.3]           DESeq2 man. geoMeans (ds2)
## Saccharibacteria_(TM7)_[G.6]           DESeq2 man. geoMeans (ds2)
## Family.Saccharibacteria_(TM7)_[F-1].   DESeq2 man. geoMeans (ds2)
## Saccharibacteria_(TM7)_[G.5]           DESeq2 man. geoMeans (ds2)
## Treponema                              DESeq2 man. geoMeans (ds2)
## Fretibacterium                         DESeq2 man. geoMeans (ds2)
## Kingdom.Bacteria.                      DESeq2 man. geoMeans (ds2)
```

```
## Others                               DESeq2 man. geoMeans (ds2)
```

```r
write.table(final, file="sbp_cat_ds2.txt", sep="\t", dec=",", row.names=F)
write.table(final[final$pval<0.05,], file="sbp_cat_ds2_sig.txt", sep="\t", dec=",", row.names=F)
final <- DA.kru(Microbio2, predictor = Phe2$sbp_cat)
final
```

```
##                                             pval  pval.adj
## Absconditabacteria_(SR1)_[G.1]        0.97652513 0.9866973
## Actinomyces                           0.35488625 0.7120224
## Peptidiphaga                          0.68790940 0.9102037
## Schaalia                              0.17997868 0.6677292
## Family.Actinomycetaceae.              0.77094403 0.9455247
## Rothia                                0.69438220 0.9102037
## Alloscardovia                         0.15830240 0.6677292
## Bifidobacterium                       0.38904318 0.7120224
## Parascardovia                         0.56869204 0.8408974
## Scardovia                             0.22397759 0.6921648
## Corynebacterium                       0.78953453 0.9455247
## Olsenella                             0.32041388 0.7119749
## Atopobium                             0.44674861 0.7284329
## Slackia                               0.28379084 0.7119749
## Cryptobacterium                       0.94847425 0.9866973
## Bacteroidetes_[G.3]                   0.18586277 0.6677292
## Bacteroidetes_[G.5]                   0.20539980 0.6921648
## Family.Bacteroidetes_[F-1].           0.12022569 0.6677292
## Bacteroidaceae_[G.1]                  0.32637338 0.7119749
## Bacteroidales_[G.2]                   0.06265757 0.6436951
## Porphyromonas                         0.02553244 0.6436951
## Tannerella                            0.13865977 0.6677292
## Alloprevotella                        0.94061179 0.9866973
## Prevotella                            0.31485061 0.7119749
## Bergeyella                            0.17654623 0.6677292
## Capnocytophaga                        0.61675627 0.8739572
## Gemella                               0.31361707 0.7119749
## Abiotrophia                           0.31972979 0.7119749
## Granulicatella                        0.45063809 0.7284329
## Lactobacillus                         0.57215698 0.8408974
## Streptococcus                         0.89063186 0.9866973
## Order.Lactobacillales.                0.89049105 0.9866973
## Class.Bacilli.                        0.01127999 0.6119866
## Clostridiales_[F.1][G-1]              0.37770452 0.7120224
## Butyrivibrio                          0.99630180 0.9963018
## Catonella                             0.16552465 0.6677292
## Johnsonella                           0.12967458 0.6677292
## Lachnoanaerobaculum                   0.67884728 0.9102037
## Lachnospiraceae_[G.2]                 0.47079564 0.7365674
## Lachnospiraceae_[G.3]                 0.75810710 0.9455247
## Lachnospiraceae_[G.7]                 0.35820061 0.7120224
## Lachnospiraceae_[G.8]                 0.08432830 0.6436951
## Oribacterium                          0.87341040 0.9866973
## Shuttleworthia                        0.78613689 0.9455247
## Stomatobaculum                        0.33763757 0.7119749
## Family.Lachnospiraceae_[XIV].         0.25107388 0.6921648
```

```
## Peptococcus                             0.96483664 0.9866973
## Parvimonas                             0.42379967 0.7284329
## Filifactor                             0.62168093 0.8739572
## Mogibacterium                          0.84446855 0.9751601
## Peptostreptococcaceae_[XI][G.1]        0.37395430 0.7120224
## Peptostreptococcaceae_[XI][G.4]        0.77736051 0.9455247
## Peptostreptococcaceae_[XI][G.5]        0.43619535 0.7284329
## Peptostreptococcaceae_[XI][G.6]        0.83358934 0.9751601
## Peptostreptococcaceae_[XI][G.7]        0.66212998 0.9054599
## Peptostreptococcaceae_[XI][G.9]        0.13471276 0.6677292
## Peptostreptococcus                     0.92718822 0.9866973
## Ruminococcaceae_[G.1]                  0.83901614 0.9751601
## Ruminococcaceae_[G.2]                  0.03932028 0.6436951
## Bulleidia                              0.18296067 0.6677292
## Eggerthia                              0.08742123 0.6436951
## Solobacterium                          0.55629483 0.8408974
## Mycoplasma                             0.09290445 0.6436951
## Mitsuokella                            0.76949753 0.9455247
## Selenomonas                            0.66275932 0.9054599
## Family.Selenomonadaceae.               0.23047423 0.6921648
## Class.Negativicutes.                   0.04772649 0.6436951
## Anaeroglobus                           0.13356372 0.6677292
## Dialister                              0.25688589 0.6921648
## Megasphaera                            0.04069955 0.6436951
## Family.Veillonellaceae.                0.41345996 0.7284329
## Veillonella                            0.29027517 0.7119749
## Veillonellaceae_[G.1]                  0.17289810 0.6677292
## Phylum.Firmicutes.                     0.97380503 0.9866973
## Fusobacterium                          0.45808668 0.7284329
## Leptotrichia                           0.33215710 0.7119749
## Gracilibacteria_(GN02)_[G.1]           0.30907972 0.7119749
## Lautropia                              0.43199430 0.7284329
## Ottowia                                0.24324419 0.6921648
## Eikenella                              0.25144181 0.6921648
## Kingella                               0.08965630 0.6436951
## Neisseria                              0.06863166 0.6436951
## Family.Neisseriaceae.                  0.08963399 0.6436951
## Campylobacter                          0.07733154 0.6436951
## Cardiobacterium                        0.01261828 0.6119866
## Aggregatibacter                        0.61830202 0.8739572
## Haemophilus                            0.40699559 0.7284329
## Family.Pasteurellaceae.                0.11084194 0.6677292
## Saccharibacteria_(TM7)_[G.1]           0.95906362 0.9866973
## Saccharibacteria_(TM7)_[G.3]           0.21258228 0.6921648
## Saccharibacteria_(TM7)_[G.6]           0.78956184 0.9455247
## Family.Saccharibacteria_(TM7)_[F-1].   0.94514465 0.9866973
## Saccharibacteria_(TM7)_[G.5]           0.38392913 0.7120224
## Treponema                              0.24963255 0.6921648
## Fretibacterium                         0.37683284 0.7120224
## Kingdom.Bacteria.                      0.90381620 0.9866973
## Others                                 0.53974164 0.8310308
##                                                                Feature
## Absconditabacteria_(SR1)_[G.1]            Absconditabacteria_(SR1)_[G.1]
## Actinomyces                                                Actinomyces
```

```
## Peptidiphaga                                                    Peptidiphaga
## Schaalia                                                            Schaalia
## Family.Actinomycetaceae.                            Family.Actinomycetaceae.
## Rothia                                                                Rothia
## Alloscardovia                                                  Alloscardovia
## Bifidobacterium                                              Bifidobacterium
## Parascardovia                                                  Parascardovia
## Scardovia                                                          Scardovia
## Corynebacterium                                              Corynebacterium
## Olsenella                                                          Olsenella
## Atopobium                                                          Atopobium
## Slackia                                                              Slackia
## Cryptobacterium                                              Cryptobacterium
## Bacteroidetes_[G.3]                                      Bacteroidetes_[G.3]
## Bacteroidetes_[G.5]                                      Bacteroidetes_[G.5]
## Family.Bacteroidetes_[F-1].                      Family.Bacteroidetes_[F-1].
## Bacteroidaceae_[G.1]                                    Bacteroidaceae_[G.1]
## Bacteroidales_[G.2]                                      Bacteroidales_[G.2]
## Porphyromonas                                                  Porphyromonas
## Tannerella                                                        Tannerella
## Alloprevotella                                                Alloprevotella
## Prevotella                                                        Prevotella
## Bergeyella                                                        Bergeyella
## Capnocytophaga                                                Capnocytophaga
## Gemella                                                              Gemella
## Abiotrophia                                                      Abiotrophia
## Granulicatella                                                Granulicatella
## Lactobacillus                                                  Lactobacillus
## Streptococcus                                                  Streptococcus
## Order.Lactobacillales.                                Order.Lactobacillales.
## Class.Bacilli.                                                Class.Bacilli.
## Clostridiales_[F.1][G-1]                            Clostridiales_[F.1][G-1]
## Butyrivibrio                                                    Butyrivibrio
## Catonella                                                          Catonella
## Johnsonella                                                      Johnsonella
## Lachnoanaerobaculum                                      Lachnoanaerobaculum
## Lachnospiraceae_[G.2]                                  Lachnospiraceae_[G.2]
## Lachnospiraceae_[G.3]                                  Lachnospiraceae_[G.3]
## Lachnospiraceae_[G.7]                                  Lachnospiraceae_[G.7]
## Lachnospiraceae_[G.8]                                  Lachnospiraceae_[G.8]
## Oribacterium                                                    Oribacterium
## Shuttleworthia                                                Shuttleworthia
## Stomatobaculum                                                Stomatobaculum
## Family.Lachnospiraceae_[XIV].                  Family.Lachnospiraceae_[XIV].
## Peptococcus                                                      Peptococcus
## Parvimonas                                                        Parvimonas
## Filifactor                                                        Filifactor
## Mogibacterium                                                  Mogibacterium
## Peptostreptococcaceae_[XI][G.1]              Peptostreptococcaceae_[XI][G.1]
## Peptostreptococcaceae_[XI][G.4]              Peptostreptococcaceae_[XI][G.4]
## Peptostreptococcaceae_[XI][G.5]              Peptostreptococcaceae_[XI][G.5]
## Peptostreptococcaceae_[XI][G.6]              Peptostreptococcaceae_[XI][G.6]
## Peptostreptococcaceae_[XI][G.7]              Peptostreptococcaceae_[XI][G.7]
## Peptostreptococcaceae_[XI][G.9]              Peptostreptococcaceae_[XI][G.9]
```

```
## Peptostreptococcus                                         Peptostreptococcus
## Ruminococcaceae_[G.1]                                   Ruminococcaceae_[G.1]
## Ruminococcaceae_[G.2]                                   Ruminococcaceae_[G.2]
## Bulleidia                                                           Bulleidia
## Eggerthia                                                           Eggerthia
## Solobacterium                                                   Solobacterium
## Mycoplasma                                                         Mycoplasma
## Mitsuokella                                                       Mitsuokella
## Selenomonas                                                       Selenomonas
## Family.Selenomonadaceae.                             Family.Selenomonadaceae.
## Class.Negativicutes.                                     Class.Negativicutes.
## Anaeroglobus                                                     Anaeroglobus
## Dialister                                                           Dialister
## Megasphaera                                                       Megasphaera
## Family.Veillonellaceae.                               Family.Veillonellaceae.
## Veillonella                                                       Veillonella
## Veillonellaceae_[G.1]                                   Veillonellaceae_[G.1]
## Phylum.Firmicutes.                                         Phylum.Firmicutes.
## Fusobacterium                                                   Fusobacterium
## Leptotrichia                                                     Leptotrichia
## Gracilibacteria_(GN02)_[G.1]                     Gracilibacteria_(GN02)_[G.1]
## Lautropia                                                           Lautropia
## Ottowia                                                               Ottowia
## Eikenella                                                           Eikenella
## Kingella                                                             Kingella
## Neisseria                                                           Neisseria
## Family.Neisseriaceae.                                   Family.Neisseriaceae.
## Campylobacter                                                   Campylobacter
## Cardiobacterium                                               Cardiobacterium
## Aggregatibacter                                               Aggregatibacter
## Haemophilus                                                       Haemophilus
## Family.Pasteurellaceae.                               Family.Pasteurellaceae.
## Saccharibacteria_(TM7)_[G.1]                     Saccharibacteria_(TM7)_[G.1]
## Saccharibacteria_(TM7)_[G.3]                     Saccharibacteria_(TM7)_[G.3]
## Saccharibacteria_(TM7)_[G.6]                     Saccharibacteria_(TM7)_[G.6]
## Family.Saccharibacteria_(TM7)_[F-1]. Family.Saccharibacteria_(TM7)_[F-1].
## Saccharibacteria_(TM7)_[G.5]                     Saccharibacteria_(TM7)_[G.5]
## Treponema                                                           Treponema
## Fretibacterium                                                 Fretibacterium
## Kingdom.Bacteria.                                           Kingdom.Bacteria.
## Others                                                                 Others
##                                                      Method
## Absconditabacteria_(SR1)_[G.1]       Kruskal-Wallis (kru)
## Actinomyces                          Kruskal-Wallis (kru)
## Peptidiphaga                         Kruskal-Wallis (kru)
## Schaalia                             Kruskal-Wallis (kru)
## Family.Actinomycetaceae.             Kruskal-Wallis (kru)
## Rothia                               Kruskal-Wallis (kru)
## Alloscardovia                        Kruskal-Wallis (kru)
## Bifidobacterium                      Kruskal-Wallis (kru)
## Parascardovia                        Kruskal-Wallis (kru)
## Scardovia                            Kruskal-Wallis (kru)
## Corynebacterium                      Kruskal-Wallis (kru)
## Olsenella                            Kruskal-Wallis (kru)
```

```
## Atopobium                              Kruskal-Wallis (kru)
## Slackia                                Kruskal-Wallis (kru)
## Cryptobacterium                        Kruskal-Wallis (kru)
## Bacteroidetes_[G.3]                    Kruskal-Wallis (kru)
## Bacteroidetes_[G.5]                    Kruskal-Wallis (kru)
## Family.Bacteroidetes_[F-1].            Kruskal-Wallis (kru)
## Bacteroidaceae_[G.1]                   Kruskal-Wallis (kru)
## Bacteroidales_[G.2]                    Kruskal-Wallis (kru)
## Porphyromonas                          Kruskal-Wallis (kru)
## Tannerella                             Kruskal-Wallis (kru)
## Alloprevotella                         Kruskal-Wallis (kru)
## Prevotella                             Kruskal-Wallis (kru)
## Bergeyella                             Kruskal-Wallis (kru)
## Capnocytophaga                         Kruskal-Wallis (kru)
## Gemella                                Kruskal-Wallis (kru)
## Abiotrophia                            Kruskal-Wallis (kru)
## Granulicatella                         Kruskal-Wallis (kru)
## Lactobacillus                          Kruskal-Wallis (kru)
## Streptococcus                          Kruskal-Wallis (kru)
## Order.Lactobacillales.                 Kruskal-Wallis (kru)
## Class.Bacilli.                         Kruskal-Wallis (kru)
## Clostridiales_[F.1][G-1]               Kruskal-Wallis (kru)
## Butyrivibrio                           Kruskal-Wallis (kru)
## Catonella                              Kruskal-Wallis (kru)
## Johnsonella                            Kruskal-Wallis (kru)
## Lachnoanaerobaculum                    Kruskal-Wallis (kru)
## Lachnospiraceae_[G.2]                  Kruskal-Wallis (kru)
## Lachnospiraceae_[G.3]                  Kruskal-Wallis (kru)
## Lachnospiraceae_[G.7]                  Kruskal-Wallis (kru)
## Lachnospiraceae_[G.8]                  Kruskal-Wallis (kru)
## Oribacterium                           Kruskal-Wallis (kru)
## Shuttleworthia                         Kruskal-Wallis (kru)
## Stomatobaculum                         Kruskal-Wallis (kru)
## Family.Lachnospiraceae_[XIV].          Kruskal-Wallis (kru)
## Peptococcus                            Kruskal-Wallis (kru)
## Parvimonas                             Kruskal-Wallis (kru)
## Filifactor                             Kruskal-Wallis (kru)
## Mogibacterium                          Kruskal-Wallis (kru)
## Peptostreptococcaceae_[XI][G.1]        Kruskal-Wallis (kru)
## Peptostreptococcaceae_[XI][G.4]        Kruskal-Wallis (kru)
## Peptostreptococcaceae_[XI][G.5]        Kruskal-Wallis (kru)
## Peptostreptococcaceae_[XI][G.6]        Kruskal-Wallis (kru)
## Peptostreptococcaceae_[XI][G.7]        Kruskal-Wallis (kru)
## Peptostreptococcaceae_[XI][G.9]        Kruskal-Wallis (kru)
## Peptostreptococcus                     Kruskal-Wallis (kru)
## Ruminococcaceae_[G.1]                  Kruskal-Wallis (kru)
## Ruminococcaceae_[G.2]                  Kruskal-Wallis (kru)
## Bulleidia                              Kruskal-Wallis (kru)
## Eggerthia                              Kruskal-Wallis (kru)
## Solobacterium                          Kruskal-Wallis (kru)
## Mycoplasma                             Kruskal-Wallis (kru)
## Mitsuokella                            Kruskal-Wallis (kru)
## Selenomonas                            Kruskal-Wallis (kru)
## Family.Selenomonadaceae.               Kruskal-Wallis (kru)
```

```
## Class.Negativicutes.                     Kruskal-Wallis (kru)
## Anaeroglobus                             Kruskal-Wallis (kru)
## Dialister                                Kruskal-Wallis (kru)
## Megasphaera                              Kruskal-Wallis (kru)
## Family.Veillonellaceae.                  Kruskal-Wallis (kru)
## Veillonella                              Kruskal-Wallis (kru)
## Veillonellaceae_[G.1]                    Kruskal-Wallis (kru)
## Phylum.Firmicutes.                       Kruskal-Wallis (kru)
## Fusobacterium                            Kruskal-Wallis (kru)
## Leptotrichia                             Kruskal-Wallis (kru)
## Gracilibacteria_(GN02)_[G.1]             Kruskal-Wallis (kru)
## Lautropia                                Kruskal-Wallis (kru)
## Ottowia                                  Kruskal-Wallis (kru)
## Eikenella                                Kruskal-Wallis (kru)
## Kingella                                 Kruskal-Wallis (kru)
## Neisseria                                Kruskal-Wallis (kru)
## Family.Neisseriaceae.                    Kruskal-Wallis (kru)
## Campylobacter                            Kruskal-Wallis (kru)
## Cardiobacterium                          Kruskal-Wallis (kru)
## Aggregatibacter                          Kruskal-Wallis (kru)
## Haemophilus                              Kruskal-Wallis (kru)
## Family.Pasteurellaceae.                  Kruskal-Wallis (kru)
## Saccharibacteria_(TM7)_[G.1]             Kruskal-Wallis (kru)
## Saccharibacteria_(TM7)_[G.3]             Kruskal-Wallis (kru)
## Saccharibacteria_(TM7)_[G.6]             Kruskal-Wallis (kru)
## Family.Saccharibacteria_(TM7)_[F-1]. Kruskal-Wallis (kru)
## Saccharibacteria_(TM7)_[G.5]             Kruskal-Wallis (kru)
## Treponema                                Kruskal-Wallis (kru)
## Fretibacterium                           Kruskal-Wallis (kru)
## Kingdom.Bacteria.                        Kruskal-Wallis (kru)
## Others                                   Kruskal-Wallis (kru)
```

```
write.table(final, file="sbp_cat_kru.txt", sep="\t", dec=",", row.names=F)
write.table(final[final$pval<0.05,], file="sbp_cat_kru_sig.txt", sep="\t", dec=",", row.names=F)
#################dbp_cat
table(Phe$dbp_cat, useNA="always")
```

```
##
##             Low          Normal    Mild_elevated Moderate_severe            <NA>
##             342             267             103              32               2
```

```
#Remove NA
Phe2<-Phe[complete.cases(Phe$dbp_cat),]
#Also subset columns
Microbio2<-dplyr::select(Microbio, one_of(Phe2$IDX))
final <- DA.ds2(Microbio2, predictor = Phe2$dbp_cat, out.all=TRUE) #It is LRT so don't look at ordering
final
```

```
##                                          baseMean log2FoldChange      lfcSE
## Absconditabacteria_(SR1)_[G.1]         69.3302739  -0.1302873487 0.27654559
## Actinomyces                           323.7803206  -0.0858214739 0.09274011
## Peptidiphaga                           22.0416883  -0.0508371802 0.13240878
## Schaalia                              703.5570032   0.1583817556 0.07607899
```

```
## Family.Actinomycetaceae.                    5.4318947  0.1980879461 0.19050286
## Rothia                                   1938.6926786 -0.1931288522 0.08910156
## Alloscardovia                              12.0844404 -0.0497551592 0.23396583
## Bifidobacterium                            10.7284828 -0.0571976887 0.25570804
## Parascardovia                               2.9264171 -0.1187060623 0.35560256
## Scardovia                                  12.5045148 -0.1395055834 0.22301963
## Corynebacterium                            93.8520152 -0.1993848742 0.09752453
## Olsenella                                   1.5705319  0.0353020834 0.21594013
## Atopobium                                 120.2270723  0.2854036055 0.08897510
## Slackia                                     1.3929535  0.0889612441 0.20667542
## Cryptobacterium                             4.2513998  0.0896881776 0.22177288
## Bacteroidetes_[G.3]                         6.6074797  0.0754628043 0.23495710
## Bacteroidetes_[G.5]                         4.6913133  0.2205031291 0.25540882
## Family.Bacteroidetes_[F-1].                 0.8456413  0.4578897900 0.36530554
## Bacteroidaceae_[G.1]                        0.7948120 -0.4397028076 0.34560712
## Bacteroidales_[G.2]                        12.0719990  0.0808544388 0.13434365
## Porphyromonas                             682.2933394 -0.2561114064 0.12204022
## Tannerella                                 22.0983847 -0.1048235941 0.09081650
## Alloprevotella                            362.5219516  0.2031244946 0.09699272
## Prevotella                               3807.2361865  0.0233927371 0.06180197
## Bergeyella                                 72.7155861  0.0016679984 0.09560346
## Capnocytophaga                            225.4613668 -0.2450880313 0.10191029
## Gemella                                   327.3165902 -0.3288663698 0.09100465
## Abiotrophia                                30.0741451 -0.0942362948 0.18350859
## Granulicatella                            306.3404328 -0.0834505784 0.07441864
## Lactobacillus                              35.4616642  1.5183795073 0.29533430
## Streptococcus                            3797.5698295 -0.1353123155 0.06967840
## Order.Lactobacillales.                      0.2871119  0.0995452388 0.25277966
## Class.Bacilli.                              0.5755295 -0.0734715424 0.20220105
## Clostridiales_[F.1][G-1]                    1.0579178 -0.0963459400 0.30640238
## Butyrivibrio                                6.9656228  0.1126622606 0.19481190
## Catonella                                  36.9095724  0.0043125233 0.10977788
## Johnsonella                                 0.9421596 -0.0845787573 0.26451710
## Lachnoanaerobaculum                        87.8820004 -0.0154176402 0.07686499
## Lachnospiraceae_[G.2]                      28.0534664  0.3797175987 0.20068137
## Lachnospiraceae_[G.3]                       2.8995123 -0.2122854561 0.20830835
## Lachnospiraceae_[G.7]                       1.3864469 -0.1988102382 0.29350051
## Lachnospiraceae_[G.8]                       1.3853264 -0.0874859731 0.31562717
## Oribacterium                              147.1321858  0.0929990815 0.08476698
## Shuttleworthia                              2.8108966  0.1582827034 0.23639758
## Stomatobaculum                            112.9182555  0.0353382973 0.09553464
## Family.Lachnospiraceae_[XIV].               1.6095271 -0.4162453681 0.26789499
## Peptococcus                                 3.7157473  0.1332154986 0.23756078
## Parvimonas                                 40.9495178 -0.0003004017 0.11809352
## Filifactor                                  6.8621336  0.2155621825 0.22968523
## Mogibacterium                              41.1723089  0.0112178738 0.08154608
## Peptostreptococcaceae_[XI][G.1]            67.7192574  0.0560959804 0.14515924
## Peptostreptococcaceae_[XI][G.4]             0.4191905 -0.0027129245 0.31456282
## Peptostreptococcaceae_[XI][G.5]             1.6690812 -0.2042683070 0.33189983
## Peptostreptococcaceae_[XI][G.6]             1.0175853  0.0733545142 0.32699190
## Peptostreptococcaceae_[XI][G.7]             4.7195993  0.0236915317 0.22635936
## Peptostreptococcaceae_[XI][G.9]             8.7363992 -0.1946211445 0.13443807
## Peptostreptococcus                         42.9864069  0.1482945034 0.16953601
## Ruminococcaceae_[G.1]                      25.1836759 -0.0592141319 0.17282994
```

```
## Ruminococcaceae_[G.2]                  41.5234417   0.0484773330 0.14391237
## Bulleidia                               3.8709078  -0.3232549268 0.25209206
## Eggerthia                               1.4587509   0.6401201510 0.33035606
## Solobacterium                          74.1630338   0.1364498875 0.09589894
## Mycoplasma                              4.5000508   0.1875786658 0.20205970
## Mitsuokella                            26.7544753   0.0897910668 0.18141615
## Selenomonas                           222.3185156   0.0373633783 0.08149824
## Family.Selenomonadaceae.                5.0504886   0.1209133946 0.21424909
## Class.Negativicutes.                    0.7322999   0.1428953834 0.19686715
## Anaeroglobus                            8.7028189  -0.0899950840 0.19593072
## Dialister                              36.4852787   0.0913735698 0.10051669
## Megasphaera                           407.7957370   0.2424635526 0.10542926
## Family.Veillonellaceae.                 0.5239301   0.2126347066 0.22983593
## Veillonella                          5061.2445718   0.0956000863 0.05932513
## Veillonellaceae_[G.1]                   6.1469526   0.0767382334 0.16581193
## Phylum.Firmicutes.                      0.8918217  -0.7743121691 0.26703970
## Fusobacterium                         929.2571372  -0.0536870685 0.07913212
## Leptotrichia                          684.6054135   0.0143814454 0.08171210
## Gracilibacteria_(GN02)_[G.1]            2.4161467  -0.0077897958 0.26344125
## Lautropia                              68.4994208  -0.0364069840 0.15174917
## Ottowia                                 2.3379102  -0.2186232241 0.33882284
## Eikenella                               8.7703431  -0.0313457797 0.12697340
## Kingella                              100.0995471  -0.1599512715 0.10911690
## Neisseria                            1333.1334336  -0.1855174765 0.12402676
## Family.Neisseriaceae.                   6.1192335  -0.0383912021 0.29128883
## Campylobacter                         257.7162863   0.1125871730 0.06967912
## Cardiobacterium                        14.8139218  -0.1636601852 0.10898429
## Aggregatibacter                       276.1135518   0.0249491620 0.14437408
## Haemophilus                          2911.7582507  -0.0743270372 0.09612404
## Family.Pasteurellaceae.                 2.5966784   0.6358631399 0.39605091
## Saccharibacteria_(TM7)_[G.1]           36.1574542   0.1931714102 0.16056188
## Saccharibacteria_(TM7)_[G.3]            7.1401803   0.4278477034 0.22249205
## Saccharibacteria_(TM7)_[G.6]            7.2298310  -0.3274416514 0.28521083
## Family.Saccharibacteria_(TM7)_[F-1].    1.1040897  -0.0488291657 0.35470725
## Saccharibacteria_(TM7)_[G.5]            0.9151491   0.0599180440 0.33616373
## Treponema                              63.3764075   0.0992445711 0.15242780
## Fretibacterium                          8.2967790  -0.0186898961 0.18390228
## Kingdom.Bacteria.                       9.4046214   0.3821998069 0.23301608
## Others                                 33.2400828  -0.2932773349 0.15472366
##                                             stat         pval   ordering
## Absconditabacteria_(SR1)_[G.1]        1.35055106 7.171656e-01 Low>Normal
## Actinomyces                           0.95099837 8.131065e-01 Low>Normal
## Peptidiphaga                          0.21403530 9.752923e-01 Low>Normal
## Schaalia                              5.56790537 1.346331e-01 Normal>Low
## Family.Actinomycetaceae.              2.24685483 5.227785e-01 Normal>Low
## Rothia                                6.91049973 7.480583e-02 Low>Normal
## Alloscardovia                         0.09549269 9.923728e-01 Low>Normal
## Bifidobacterium                       2.61135510 4.555023e-01 Low>Normal
## Parascardovia                         1.38039831 7.101358e-01 Low>Normal
## Scardovia                             1.17296676 7.594950e-01 Low>Normal
## Corynebacterium                       5.92940491 1.150958e-01 Low>Normal
## Olsenella                             1.36437126 7.139071e-01 Normal>Low
## Atopobium                            11.30826338 1.017059e-02 Normal>Low
## Slackia                               0.38675699 9.429644e-01 Normal>Low
```

```
## Cryptobacterium                        1.55490106 6.696584e-01 Normal>Low
## Bacteroidetes_[G.3]                    2.56124275 4.643247e-01 Normal>Low
## Bacteroidetes_[G.5]                    3.94911873 2.670111e-01 Normal>Low
## Family.Bacteroidetes_[F-1].            4.44356712 2.173795e-01 Normal>Low
## Bacteroidaceae_[G.1]                   5.20147417 1.576249e-01 Low>Normal
## Bacteroidales_[G.2]                    2.34368210 5.042043e-01 Normal>Low
## Porphyromonas                          8.17986073 4.243716e-02 Low>Normal
## Tannerella                             1.86218624 6.014969e-01 Low>Normal
## Alloprevotella                         9.68294442 2.146271e-02 Normal>Low
## Prevotella                             3.06260024 3.820738e-01 Normal>Low
## Bergeyella                             0.64616325 8.857877e-01 Normal>Low
## Capnocytophaga                         5.95757930 1.136924e-01 Low>Normal
## Gemella                               16.96681841 7.179348e-04 Low>Normal
## Abiotrophia                            7.53435032 5.668238e-02 Low>Normal
## Granulicatella                         2.27662915 5.170129e-01 Low>Normal
## Lactobacillus                         44.74808448 1.046654e-09 Normal>Low
## Streptococcus                          4.55420469 2.075060e-01 Low>Normal
## Order.Lactobacillales.                -3.11824848 1.000000e+00 Normal>Low
## Class.Bacilli.                         7.48108809 5.804638e-02 Low>Normal
## Clostridiales_[F.1][G-1]               4.56823730 2.062838e-01 Low>Normal
## Butyrivibrio                           0.64295483 8.865322e-01 Normal>Low
## Catonella                              2.45394413 4.836719e-01 Normal>Low
## Johnsonella                            1.87313772 5.991501e-01 Low>Normal
## Lachnoanaerobaculum                    1.43035276 6.984356e-01 Low>Normal
## Lachnospiraceae_[G.2]                  6.69055671 8.244287e-02 Normal>Low
## Lachnospiraceae_[G.3]                  3.71782466 2.935903e-01 Low>Normal
## Lachnospiraceae_[G.7]                  3.56674756 3.122078e-01 Low>Normal
## Lachnospiraceae_[G.8]                  2.20612901 5.307422e-01 Low>Normal
## Oribacterium                           8.18438225 4.235088e-02 Normal>Low
## Shuttleworthia                         0.76305782 8.582811e-01 Normal>Low
## Stomatobaculum                         4.15919684 2.447789e-01 Normal>Low
## Family.Lachnospiraceae_[XIV].          7.68483087 5.299479e-02 Low>Normal
## Peptococcus                            0.42656250 9.347023e-01 Normal>Low
## Parvimonas                             0.40839813 9.385022e-01 Low>Normal
## Filifactor                             2.84117070 4.167673e-01 Normal>Low
## Mogibacterium                          1.42886559 6.987826e-01 Normal>Low
## Peptostreptococcaceae_[XI][G.1]        1.00641818 7.996990e-01 Normal>Low
## Peptostreptococcaceae_[XI][G.4]        0.09845393 9.920223e-01 Low>Normal
## Peptostreptococcaceae_[XI][G.5]        0.60218694 8.959315e-01 Low>Normal
## Peptostreptococcaceae_[XI][G.6]        0.33917279 9.525004e-01 Normal>Low
## Peptostreptococcaceae_[XI][G.7]        5.51534048 1.377240e-01 Normal>Low
## Peptostreptococcaceae_[XI][G.9]        9.56738852 2.262516e-02 Low>Normal
## Peptostreptococcus                     2.59772273 4.578889e-01 Normal>Low
## Ruminococcaceae_[G.1]                  0.84866871 8.377941e-01 Low>Normal
## Ruminococcaceae_[G.2]                  1.73559580 6.290492e-01 Normal>Low
## Bulleidia                              8.71533427 3.332517e-02 Low>Normal
## Eggerthia                             10.01153642 1.846833e-02 Normal>Low
## Solobacterium                          2.79494858 4.243321e-01 Normal>Low
## Mycoplasma                             4.02504892 2.587720e-01 Normal>Low
## Mitsuokella                            5.95510522 1.138150e-01 Normal>Low
## Selenomonas                            9.43995170 2.397883e-02 Normal>Low
## Family.Selenomonadaceae.               0.47343140 9.246884e-01 Normal>Low
## Class.Negativicutes.                   3.31560159 3.454771e-01 Normal>Low
## Anaeroglobus                           2.06313581 5.594066e-01 Low>Normal
```

```
## Dialister                             2.15961779 5.399461e-01 Normal>Low
## Megasphaera                           9.56234881 2.267724e-02 Normal>Low
## Family.Veillonellaceae.               1.29960005 7.292281e-01 Normal>Low
## Veillonella                           6.22910887 1.009803e-01 Normal>Low
## Veillonellaceae_[G.1]                 1.45740325 6.921359e-01 Normal>Low
## Phylum.Firmicutes.                   18.29557315 3.822267e-04 Low>Normal
## Fusobacterium                         2.69025856 4.418853e-01 Low>Normal
## Leptotrichia                          0.07628037 9.945233e-01 Normal>Low
## Gracilibacteria_(GN02)_[G.1]          1.66376147 6.450203e-01 Low>Normal
## Lautropia                             1.11264420 7.740226e-01 Low>Normal
## Ottowia                               4.98883072 1.726168e-01 Low>Normal
## Eikenella                             2.71997741 4.368430e-01 Low>Normal
## Kingella                              6.46381911 9.110036e-02 Low>Normal
## Neisseria                             7.00502358 7.173783e-02 Low>Normal
## Family.Neisseriaceae.                 0.72931987 8.662869e-01 Low>Normal
## Campylobacter                        11.86632103 7.855403e-03 Normal>Low
## Cardiobacterium                       4.30638008 2.302247e-01 Low>Normal
## Aggregatibacter                       0.89399128 8.268776e-01 Normal>Low
## Haemophilus                           3.91120996 2.712128e-01 Low>Normal
## Family.Pasteurellaceae.               3.00306290 3.911532e-01 Normal>Low
## Saccharibacteria_(TM7)_[G.1]          1.50682983 6.806949e-01 Normal>Low
## Saccharibacteria_(TM7)_[G.3]          7.32718595 6.216906e-02 Normal>Low
## Saccharibacteria_(TM7)_[G.6]          1.75516821 6.247391e-01 Low>Normal
## Family.Saccharibacteria_(TM7)_[F-1].  2.76675920 4.290012e-01 Low>Normal
## Saccharibacteria_(TM7)_[G.5]          2.13893280 5.440766e-01 Normal>Low
## Treponema                             0.59226843 8.982000e-01 Normal>Low
## Fretibacterium                        3.31842730 3.450861e-01 Low>Normal
## Kingdom.Bacteria.                     3.45006317 3.272967e-01 Normal>Low
## Others                                8.66495155 3.409372e-02 Low>Normal
##                                          pval.adj
## Absconditabacteria_(SR1)_[G.1]       9.400685e-01
## Actinomyces                          9.983713e-01
## Peptidiphaga                         1.000000e+00
## Schaalia                             4.771151e-01
## Family.Actinomycetaceae.             8.795906e-01
## Rothia                               3.628083e-01
## Alloscardovia                        1.000000e+00
## Bifidobacterium                      8.498019e-01
## Parascardovia                        9.400685e-01
## Scardovia                            9.693555e-01
## Corynebacterium                      4.293959e-01
## Olsenella                            9.400685e-01
## Atopobium                            1.973094e-01
## Slackia                              1.000000e+00
## Cryptobacterium                      9.400685e-01
## Bacteroidetes_[G.3]                  8.498019e-01
## Bacteroidetes_[G.5]                  6.923065e-01
## Family.Bacteroidetes_[F-1].          6.389641e-01
## Bacteroidaceae_[G.1]                 5.272280e-01
## Bacteroidales_[G.2]                  8.795906e-01
## Porphyromonas                        2.940289e-01
## Tannerella                           9.261143e-01
## Alloprevotella                       2.325946e-01
## Prevotella                           8.422990e-01
```

```
## Bergeyella                          1.000000e+00
## Capnocytophaga                      4.293959e-01
## Gemella                             2.321323e-02
## Abiotrophia                         3.312058e-01
## Granulicatella                      8.795906e-01
## Lactobacillus                       1.015255e-07
## Streptococcus                       6.290026e-01
## Order.Lactobacillales.              1.000000e+00
## Class.Bacilli.                      3.312058e-01
## Clostridiales_[F.1][G-1]            6.290026e-01
## Butyrivibrio                        1.000000e+00
## Catonella                           8.688181e-01
## Johnsonella                         9.261143e-01
## Lachnoanaerobaculum                 9.400685e-01
## Lachnospiraceae_[G.2]               3.808076e-01
## Lachnospiraceae_[G.3]               7.302117e-01
## Lachnospiraceae_[G.7]               7.571039e-01
## Lachnospiraceae_[G.8]               8.795906e-01
## Oribacterium                        2.940289e-01
## Shuttleworthia                      1.000000e+00
## Stomatobaculum                      6.783873e-01
## Family.Lachnospiraceae_[XIV].       3.312058e-01
## Peptococcus                         1.000000e+00
## Parvimonas                          1.000000e+00
## Filifactor                          8.498019e-01
## Mogibacterium                       9.400685e-01
## Peptostreptococcaceae_[XI][G.1]     9.944974e-01
## Peptostreptococcaceae_[XI][G.4]     1.000000e+00
## Peptostreptococcaceae_[XI][G.5]     1.000000e+00
## Peptostreptococcaceae_[XI][G.6]     1.000000e+00
## Peptostreptococcaceae_[XI][G.7]     4.771151e-01
## Peptostreptococcaceae_[XI][G.9]     2.325946e-01
## Peptostreptococcus                  8.498019e-01
## Ruminococcaceae_[G.1]               1.000000e+00
## Ruminococcaceae_[G.2]               9.387350e-01
## Bulleidia                           2.755909e-01
## Eggerthia                           2.325946e-01
## Solobacterium                       8.498019e-01
## Mycoplasma                          6.923065e-01
## Mitsuokella                         4.293959e-01
## Selenomonas                         2.325946e-01
## Family.Selenomonadaceae.            1.000000e+00
## Class.Negativicutes.                7.793320e-01
## Anaeroglobus                        8.895481e-01
## Dialister                           8.795906e-01
## Megasphaera                         2.325946e-01
## Family.Veillonellaceae.             9.431349e-01
## Veillonella                         4.258735e-01
## Veillonellaceae_[G.1]               9.400685e-01
## Phylum.Firmicutes.                  1.853800e-02
## Fusobacterium                       8.498019e-01
## Leptotrichia                        1.000000e+00
## Gracilibacteria_(GN02)_[G.1]        9.400685e-01
## Lautropia                           9.750675e-01
```

```
## Ottowia                              5.581278e-01
## Eikenella                            8.498019e-01
## Kingella                             4.016698e-01
## Neisseria                            3.628083e-01
## Family.Neisseriaceae.                1.000000e+00
## Campylobacter                        1.904935e-01
## Cardiobacterium                      6.568175e-01
## Aggregatibacter                      1.000000e+00
## Haemophilus                          6.923065e-01
## Family.Pasteurellaceae.              8.431524e-01
## Saccharibacteria_(TM7)_[G.1]         9.400685e-01
## Saccharibacteria_(TM7)_[G.3]         3.350222e-01
## Saccharibacteria_(TM7)_[G.6]         9.387350e-01
## Family.Saccharibacteria_(TM7)_[F-1]. 8.498019e-01
## Saccharibacteria_(TM7)_[G.5]         8.795906e-01
## Treponema                            1.000000e+00
## Fretibacterium                       7.793320e-01
## Kingdom.Bacteria.                    7.743362e-01
## Others                               2.755909e-01
##                                                                   Feature
## Absconditabacteria_(SR1)_[G.1]            Absconditabacteria_(SR1)_[G.1]
## Actinomyces                                                   Actinomyces
## Peptidiphaga                                                 Peptidiphaga
## Schaalia                                                         Schaalia
## Family.Actinomycetaceae.                         Family.Actinomycetaceae.
## Rothia                                                             Rothia
## Alloscardovia                                               Alloscardovia
## Bifidobacterium                                           Bifidobacterium
## Parascardovia                                               Parascardovia
## Scardovia                                                       Scardovia
## Corynebacterium                                           Corynebacterium
## Olsenella                                                       Olsenella
## Atopobium                                                       Atopobium
## Slackia                                                           Slackia
## Cryptobacterium                                           Cryptobacterium
## Bacteroidetes_[G.3]                                   Bacteroidetes_[G.3]
## Bacteroidetes_[G.5]                                   Bacteroidetes_[G.5]
## Family.Bacteroidetes_[F-1].                   Family.Bacteroidetes_[F-1].
## Bacteroidaceae_[G.1]                                 Bacteroidaceae_[G.1]
## Bacteroidales_[G.2]                                   Bacteroidales_[G.2]
## Porphyromonas                                               Porphyromonas
## Tannerella                                                     Tannerella
## Alloprevotella                                             Alloprevotella
## Prevotella                                                     Prevotella
## Bergeyella                                                     Bergeyella
## Capnocytophaga                                             Capnocytophaga
## Gemella                                                           Gemella
## Abiotrophia                                                   Abiotrophia
## Granulicatella                                             Granulicatella
## Lactobacillus                                               Lactobacillus
## Streptococcus                                               Streptococcus
## Order.Lactobacillales.                             Order.Lactobacillales.
## Class.Bacilli.                                             Class.Bacilli.
## Clostridiales_[F.1][G-1]                         Clostridiales_[F.1][G-1]
```

```
## Butyrivibrio                                                     Butyrivibrio
## Catonella                                                           Catonella
## Johnsonella                                                       Johnsonella
## Lachnoanaerobaculum                                       Lachnoanaerobaculum
## Lachnospiraceae_[G.2]                                   Lachnospiraceae_[G.2]
## Lachnospiraceae_[G.3]                                   Lachnospiraceae_[G.3]
## Lachnospiraceae_[G.7]                                   Lachnospiraceae_[G.7]
## Lachnospiraceae_[G.8]                                   Lachnospiraceae_[G.8]
## Oribacterium                                                     Oribacterium
## Shuttleworthia                                                 Shuttleworthia
## Stomatobaculum                                                 Stomatobaculum
## Family.Lachnospiraceae_[XIV].                   Family.Lachnospiraceae_[XIV].
## Peptococcus                                                       Peptococcus
## Parvimonas                                                         Parvimonas
## Filifactor                                                         Filifactor
## Mogibacterium                                                   Mogibacterium
## Peptostreptococcaceae_[XI][G.1]               Peptostreptococcaceae_[XI][G.1]
## Peptostreptococcaceae_[XI][G.4]               Peptostreptococcaceae_[XI][G.4]
## Peptostreptococcaceae_[XI][G.5]               Peptostreptococcaceae_[XI][G.5]
## Peptostreptococcaceae_[XI][G.6]               Peptostreptococcaceae_[XI][G.6]
## Peptostreptococcaceae_[XI][G.7]               Peptostreptococcaceae_[XI][G.7]
## Peptostreptococcaceae_[XI][G.9]               Peptostreptococcaceae_[XI][G.9]
## Peptostreptococcus                                         Peptostreptococcus
## Ruminococcaceae_[G.1]                                   Ruminococcaceae_[G.1]
## Ruminococcaceae_[G.2]                                   Ruminococcaceae_[G.2]
## Bulleidia                                                           Bulleidia
## Eggerthia                                                           Eggerthia
## Solobacterium                                                   Solobacterium
## Mycoplasma                                                         Mycoplasma
## Mitsuokella                                                       Mitsuokella
## Selenomonas                                                       Selenomonas
## Family.Selenomonadaceae.                             Family.Selenomonadaceae.
## Class.Negativicutes.                                     Class.Negativicutes.
## Anaeroglobus                                                     Anaeroglobus
## Dialister                                                           Dialister
## Megasphaera                                                       Megasphaera
## Family.Veillonellaceae.                               Family.Veillonellaceae.
## Veillonella                                                       Veillonella
## Veillonellaceae_[G.1]                                   Veillonellaceae_[G.1]
## Phylum.Firmicutes.                                         Phylum.Firmicutes.
## Fusobacterium                                                   Fusobacterium
## Leptotrichia                                                     Leptotrichia
## Gracilibacteria_(GN02)_[G.1]                     Gracilibacteria_(GN02)_[G.1]
## Lautropia                                                           Lautropia
## Ottowia                                                               Ottowia
## Eikenella                                                           Eikenella
## Kingella                                                             Kingella
## Neisseria                                                           Neisseria
## Family.Neisseriaceae.                                   Family.Neisseriaceae.
## Campylobacter                                                   Campylobacter
## Cardiobacterium                                               Cardiobacterium
## Aggregatibacter                                               Aggregatibacter
## Haemophilus                                                       Haemophilus
## Family.Pasteurellaceae.                               Family.Pasteurellaceae.
```

```
## Saccharibacteria_(TM7)_[G.1]                 Saccharibacteria_(TM7)_[G.1]
## Saccharibacteria_(TM7)_[G.3]                 Saccharibacteria_(TM7)_[G.3]
## Saccharibacteria_(TM7)_[G.6]                 Saccharibacteria_(TM7)_[G.6]
## Family.Saccharibacteria_(TM7)_[F-1]. Family.Saccharibacteria_(TM7)_[F-1].
## Saccharibacteria_(TM7)_[G.5]                 Saccharibacteria_(TM7)_[G.5]
## Treponema                                                        Treponema
## Fretibacterium                                              Fretibacterium
## Kingdom.Bacteria.                                        Kingdom.Bacteria.
## Others                                                              Others
##                                                          Method
## Absconditabacteria_(SR1)_[G.1]       DESeq2 man. geoMeans (ds2)
## Actinomyces                          DESeq2 man. geoMeans (ds2)
## Peptidiphaga                         DESeq2 man. geoMeans (ds2)
## Schaalia                             DESeq2 man. geoMeans (ds2)
## Family.Actinomycetaceae.             DESeq2 man. geoMeans (ds2)
## Rothia                               DESeq2 man. geoMeans (ds2)
## Alloscardovia                        DESeq2 man. geoMeans (ds2)
## Bifidobacterium                      DESeq2 man. geoMeans (ds2)
## Parascardovia                        DESeq2 man. geoMeans (ds2)
## Scardovia                            DESeq2 man. geoMeans (ds2)
## Corynebacterium                      DESeq2 man. geoMeans (ds2)
## Olsenella                            DESeq2 man. geoMeans (ds2)
## Atopobium                            DESeq2 man. geoMeans (ds2)
## Slackia                              DESeq2 man. geoMeans (ds2)
## Cryptobacterium                      DESeq2 man. geoMeans (ds2)
## Bacteroidetes_[G.3]                  DESeq2 man. geoMeans (ds2)
## Bacteroidetes_[G.5]                  DESeq2 man. geoMeans (ds2)
## Family.Bacteroidetes_[F-1].          DESeq2 man. geoMeans (ds2)
## Bacteroidaceae_[G.1]                 DESeq2 man. geoMeans (ds2)
## Bacteroidales_[G.2]                  DESeq2 man. geoMeans (ds2)
## Porphyromonas                        DESeq2 man. geoMeans (ds2)
## Tannerella                           DESeq2 man. geoMeans (ds2)
## Alloprevotella                       DESeq2 man. geoMeans (ds2)
## Prevotella                           DESeq2 man. geoMeans (ds2)
## Bergeyella                           DESeq2 man. geoMeans (ds2)
## Capnocytophaga                       DESeq2 man. geoMeans (ds2)
## Gemella                              DESeq2 man. geoMeans (ds2)
## Abiotrophia                          DESeq2 man. geoMeans (ds2)
## Granulicatella                       DESeq2 man. geoMeans (ds2)
## Lactobacillus                        DESeq2 man. geoMeans (ds2)
## Streptococcus                        DESeq2 man. geoMeans (ds2)
## Order.Lactobacillales.               DESeq2 man. geoMeans (ds2)
## Class.Bacilli.                       DESeq2 man. geoMeans (ds2)
## Clostridiales_[F.1][G-1]             DESeq2 man. geoMeans (ds2)
## Butyrivibrio                         DESeq2 man. geoMeans (ds2)
## Catonella                            DESeq2 man. geoMeans (ds2)
## Johnsonella                          DESeq2 man. geoMeans (ds2)
## Lachnoanaerobaculum                  DESeq2 man. geoMeans (ds2)
## Lachnospiraceae_[G.2]                DESeq2 man. geoMeans (ds2)
## Lachnospiraceae_[G.3]                DESeq2 man. geoMeans (ds2)
## Lachnospiraceae_[G.7]                DESeq2 man. geoMeans (ds2)
## Lachnospiraceae_[G.8]                DESeq2 man. geoMeans (ds2)
## Oribacterium                         DESeq2 man. geoMeans (ds2)
## Shuttleworthia                       DESeq2 man. geoMeans (ds2)
```

```
## Stomatobaculum                        DESeq2 man. geoMeans (ds2)
## Family.Lachnospiraceae_[XIV].         DESeq2 man. geoMeans (ds2)
## Peptococcus                           DESeq2 man. geoMeans (ds2)
## Parvimonas                            DESeq2 man. geoMeans (ds2)
## Filifactor                            DESeq2 man. geoMeans (ds2)
## Mogibacterium                         DESeq2 man. geoMeans (ds2)
## Peptostreptococcaceae_[XI][G.1]       DESeq2 man. geoMeans (ds2)
## Peptostreptococcaceae_[XI][G.4]       DESeq2 man. geoMeans (ds2)
## Peptostreptococcaceae_[XI][G.5]       DESeq2 man. geoMeans (ds2)
## Peptostreptococcaceae_[XI][G.6]       DESeq2 man. geoMeans (ds2)
## Peptostreptococcaceae_[XI][G.7]       DESeq2 man. geoMeans (ds2)
## Peptostreptococcaceae_[XI][G.9]       DESeq2 man. geoMeans (ds2)
## Peptostreptococcus                    DESeq2 man. geoMeans (ds2)
## Ruminococcaceae_[G.1]                 DESeq2 man. geoMeans (ds2)
## Ruminococcaceae_[G.2]                 DESeq2 man. geoMeans (ds2)
## Bulleidia                             DESeq2 man. geoMeans (ds2)
## Eggerthia                             DESeq2 man. geoMeans (ds2)
## Solobacterium                         DESeq2 man. geoMeans (ds2)
## Mycoplasma                            DESeq2 man. geoMeans (ds2)
## Mitsuokella                           DESeq2 man. geoMeans (ds2)
## Selenomonas                           DESeq2 man. geoMeans (ds2)
## Family.Selenomonadaceae.              DESeq2 man. geoMeans (ds2)
## Class.Negativicutes.                  DESeq2 man. geoMeans (ds2)
## Anaeroglobus                          DESeq2 man. geoMeans (ds2)
## Dialister                             DESeq2 man. geoMeans (ds2)
## Megasphaera                           DESeq2 man. geoMeans (ds2)
## Family.Veillonellaceae.               DESeq2 man. geoMeans (ds2)
## Veillonella                           DESeq2 man. geoMeans (ds2)
## Veillonellaceae_[G.1]                 DESeq2 man. geoMeans (ds2)
## Phylum.Firmicutes.                    DESeq2 man. geoMeans (ds2)
## Fusobacterium                         DESeq2 man. geoMeans (ds2)
## Leptotrichia                          DESeq2 man. geoMeans (ds2)
## Gracilibacteria_(GN02)_[G.1]          DESeq2 man. geoMeans (ds2)
## Lautropia                             DESeq2 man. geoMeans (ds2)
## Ottowia                               DESeq2 man. geoMeans (ds2)
## Eikenella                             DESeq2 man. geoMeans (ds2)
## Kingella                              DESeq2 man. geoMeans (ds2)
## Neisseria                             DESeq2 man. geoMeans (ds2)
## Family.Neisseriaceae.                 DESeq2 man. geoMeans (ds2)
## Campylobacter                         DESeq2 man. geoMeans (ds2)
## Cardiobacterium                       DESeq2 man. geoMeans (ds2)
## Aggregatibacter                       DESeq2 man. geoMeans (ds2)
## Haemophilus                           DESeq2 man. geoMeans (ds2)
## Family.Pasteurellaceae.               DESeq2 man. geoMeans (ds2)
## Saccharibacteria_(TM7)_[G.1]          DESeq2 man. geoMeans (ds2)
## Saccharibacteria_(TM7)_[G.3]          DESeq2 man. geoMeans (ds2)
## Saccharibacteria_(TM7)_[G.6]          DESeq2 man. geoMeans (ds2)
## Family.Saccharibacteria_(TM7)_[F-1]. DESeq2 man. geoMeans (ds2)
## Saccharibacteria_(TM7)_[G.5]          DESeq2 man. geoMeans (ds2)
## Treponema                             DESeq2 man. geoMeans (ds2)
## Fretibacterium                        DESeq2 man. geoMeans (ds2)
## Kingdom.Bacteria.                     DESeq2 man. geoMeans (ds2)
## Others                                DESeq2 man. geoMeans (ds2)
```

```
write.table(final, file="dbp_cat_ds2.txt", sep="\t", dec=",", row.names=F)
write.table(final[final$pval<0.05,], file="dbp_cat_ds2_sig.txt", sep="\t", dec=",", row.names=F)
final <- DA.kru(Microbio2, predictor = Phe2$dbp_cat)
final
```

```
##                                           pval  pval.adj
## Absconditabacteria_(SR1)_[G.1]        0.873912101 0.9595239
## Actinomyces                           0.870222327 0.9595239
## Peptidiphaga                          0.961129024 0.9813633
## Schaalia                              0.265078137 0.8064674
## Family.Actinomycetaceae.              0.566773062 0.9195889
## Rothia                                0.045562685 0.4017800
## Alloscardovia                         0.990995108 0.9931110
## Bifidobacterium                       0.364789959 0.8375713
## Parascardovia                         0.716186939 0.9195889
## Scardovia                             0.281304312 0.8064674
## Corynebacterium                       0.944842615 0.9749972
## Olsenella                             0.761102806 0.9195889
## Atopobium                             0.050261866 0.4062834
## Slackia                               0.786864732 0.9195889
## Cryptobacterium                       0.716667628 0.9195889
## Bacteroidetes_[G.3]                   0.044516585 0.4017800
## Bacteroidetes_[G.5]                   0.232496638 0.8064674
## Family.Bacteroidetes_[F-1].           0.324129151 0.8194541
## Bacteroidaceae_[G.1]                  0.475818131 0.8875838
## Bacteroidales_[G.2]                   0.177705860 0.7835213
## Porphyromonas                         0.007404321 0.4017800
## Tannerella                            0.418152802 0.8592575
## Alloprevotella                        0.014822048 0.4017800
## Prevotella                            0.348701169 0.8249759
## Bergeyella                            0.630453777 0.9195889
## Capnocytophaga                        0.330800002 0.8194541
## Gemella                               0.044622689 0.4017800
## Abiotrophia                           0.039406998 0.4017800
## Granulicatella                        0.092642041 0.5686213
## Lactobacillus                         0.938442113 0.9749972
## Streptococcus                         0.648135693 0.9195889
## Order.Lactobacillales.                0.326267957 0.8194541
## Class.Bacilli.                        0.277393535 0.8064674
## Clostridiales_[F.1][G-1]              0.536994115 0.9195889
## Butyrivibrio                          0.282551606 0.8064674
## Catonella                             0.635368880 0.9195889
## Johnsonella                           0.452910400 0.8614178
## Lachnoanaerobaculum                   0.743132521 0.9195889
## Lachnospiraceae_[G.2]                 0.043927282 0.4017800
## Lachnospiraceae_[G.3]                 0.658376331 0.9195889
## Lachnospiraceae_[G.7]                 0.603114092 0.9195889
## Lachnospiraceae_[G.8]                 0.701233465 0.9195889
## Oribacterium                          0.014519219 0.4017800
## Shuttleworthia                        0.434057931 0.8592575
## Stomatobaculum                        0.784014278 0.9195889
## Family.Lachnospiraceae_[XIV].         0.269329887 0.8064674
## Peptococcus                           0.933597080 0.9749972
```

```
## Parvimonas                             0.337919225 0.8194541
## Filifactor                             0.017808777 0.4017800
## Mogibacterium                          0.763154097 0.9195889
## Peptostreptococcaceae_[XI][G.1]        0.718208197 0.9195889
## Peptostreptococcaceae_[XI][G.4]        0.634326844 0.9195889
## Peptostreptococcaceae_[XI][G.5]        0.525031257 0.9195889
## Peptostreptococcaceae_[XI][G.6]        0.846221897 0.9595239
## Peptostreptococcaceae_[XI][G.7]        0.157998347 0.7662920
## Peptostreptococcaceae_[XI][G.9]        0.271975961 0.8064674
## Peptostreptococcus                     0.244732890 0.8064674
## Ruminococcaceae_[G.1]                  0.856813748 0.9595239
## Ruminococcaceae_[G.2]                  0.993111048 0.9931110
## Bulleidia                              0.093205349 0.5686213
## Eggerthia                              0.102819974 0.5866787
## Solobacterium                          0.379929272 0.8375713
## Mycoplasma                             0.587503117 0.9195889
## Mitsuokella                            0.281467711 0.8064674
## Selenomonas                            0.290993376 0.8064674
## Family.Selenomonadaceae.               0.376831543 0.8375713
## Class.Negativicutes.                   0.559108046 0.9195889
## Anaeroglobus                           0.306276779 0.8194541
## Dialister                              0.443242814 0.8598911
## Megasphaera                            0.073326542 0.5471288
## Family.Veillonellaceae.                0.673184433 0.9195889
## Veillonella                            0.093793196 0.5686213
## Veillonellaceae_[G.1]                  0.407312667 0.8592575
## Phylum.Firmicutes.                     0.566072305 0.9195889
## Fusobacterium                          0.887726236 0.9595239
## Leptotrichia                           0.509637115 0.9195889
## Gracilibacteria_(GN02)_[G.1]           0.418875358 0.8592575
## Lautropia                              0.744422237 0.9195889
## Ottowia                                0.129217575 0.6596897
## Eikenella                              0.719824266 0.9195889
## Kingella                               0.618874680 0.9195889
## Neisseria                              0.030577423 0.4017800
## Family.Neisseriaceae.                  0.290446171 0.8064674
## Campylobacter                          0.033422257 0.4017800
## Cardiobacterium                        0.609485269 0.9195889
## Aggregatibacter                        0.575603823 0.9195889
## Haemophilus                            0.195930673 0.8064674
## Family.Pasteurellaceae.                0.715073826 0.9195889
## Saccharibacteria_(TM7)_[G.1]           0.786308411 0.9195889
## Saccharibacteria_(TM7)_[G.3]           0.169219943 0.7816350
## Saccharibacteria_(TM7)_[G.6]           0.735110944 0.9195889
## Family.Saccharibacteria_(TM7)_[F-1]. 0.909137979 0.9690811
## Saccharibacteria_(TM7)_[G.5]           0.868690590 0.9595239
## Treponema                              0.890279905 0.9595239
## Fretibacterium                         0.114684144 0.6180201
## Kingdom.Bacteria.                      0.430580267 0.8592575
## Others                                 0.213946509 0.8064674
##                                                                Feature
## Absconditabacteria_(SR1)_[G.1]            Absconditabacteria_(SR1)_[G.1]
## Actinomyces                                                Actinomyces
## Peptidiphaga                                              Peptidiphaga
```

```
## Schaalia                                                                Schaalia
## Family.Actinomycetaceae.                                Family.Actinomycetaceae.
## Rothia                                                                    Rothia
## Alloscardovia                                                      Alloscardovia
## Bifidobacterium                                                  Bifidobacterium
## Parascardovia                                                      Parascardovia
## Scardovia                                                              Scardovia
## Corynebacterium                                                  Corynebacterium
## Olsenella                                                              Olsenella
## Atopobium                                                              Atopobium
## Slackia                                                                  Slackia
## Cryptobacterium                                                  Cryptobacterium
## Bacteroidetes_[G.3]                                          Bacteroidetes_[G.3]
## Bacteroidetes_[G.5]                                          Bacteroidetes_[G.5]
## Family.Bacteroidetes_[F-1].                          Family.Bacteroidetes_[F-1].
## Bacteroidaceae_[G.1]                                        Bacteroidaceae_[G.1]
## Bacteroidales_[G.2]                                          Bacteroidales_[G.2]
## Porphyromonas                                                      Porphyromonas
## Tannerella                                                            Tannerella
## Alloprevotella                                                    Alloprevotella
## Prevotella                                                            Prevotella
## Bergeyella                                                            Bergeyella
## Capnocytophaga                                                    Capnocytophaga
## Gemella                                                                  Gemella
## Abiotrophia                                                          Abiotrophia
## Granulicatella                                                    Granulicatella
## Lactobacillus                                                      Lactobacillus
## Streptococcus                                                      Streptococcus
## Order.Lactobacillales.                                    Order.Lactobacillales.
## Class.Bacilli.                                                    Class.Bacilli.
## Clostridiales_[F.1][G-1]                                Clostridiales_[F.1][G-1]
## Butyrivibrio                                                        Butyrivibrio
## Catonella                                                              Catonella
## Johnsonella                                                          Johnsonella
## Lachnoanaerobaculum                                          Lachnoanaerobaculum
## Lachnospiraceae_[G.2]                                      Lachnospiraceae_[G.2]
## Lachnospiraceae_[G.3]                                      Lachnospiraceae_[G.3]
## Lachnospiraceae_[G.7]                                      Lachnospiraceae_[G.7]
## Lachnospiraceae_[G.8]                                      Lachnospiraceae_[G.8]
## Oribacterium                                                        Oribacterium
## Shuttleworthia                                                    Shuttleworthia
## Stomatobaculum                                                    Stomatobaculum
## Family.Lachnospiraceae_[XIV].                      Family.Lachnospiraceae_[XIV].
## Peptococcus                                                          Peptococcus
## Parvimonas                                                            Parvimonas
## Filifactor                                                            Filifactor
## Mogibacterium                                                      Mogibacterium
## Peptostreptococcaceae_[XI][G.1]                  Peptostreptococcaceae_[XI][G.1]
## Peptostreptococcaceae_[XI][G.4]                  Peptostreptococcaceae_[XI][G.4]
## Peptostreptococcaceae_[XI][G.5]                  Peptostreptococcaceae_[XI][G.5]
## Peptostreptococcaceae_[XI][G.6]                  Peptostreptococcaceae_[XI][G.6]
## Peptostreptococcaceae_[XI][G.7]                  Peptostreptococcaceae_[XI][G.7]
## Peptostreptococcaceae_[XI][G.9]                  Peptostreptococcaceae_[XI][G.9]
## Peptostreptococcus                                            Peptostreptococcus
```

```
## Ruminococcaceae_[G.1]                                        Ruminococcaceae_[G.1]
## Ruminococcaceae_[G.2]                                        Ruminococcaceae_[G.2]
## Bulleidia                                                                Bulleidia
## Eggerthia                                                                Eggerthia
## Solobacterium                                                        Solobacterium
## Mycoplasma                                                              Mycoplasma
## Mitsuokella                                                            Mitsuokella
## Selenomonas                                                            Selenomonas
## Family.Selenomonadaceae.                                  Family.Selenomonadaceae.
## Class.Negativicutes.                                          Class.Negativicutes.
## Anaeroglobus                                                          Anaeroglobus
## Dialister                                                                Dialister
## Megasphaera                                                            Megasphaera
## Family.Veillonellaceae.                                    Family.Veillonellaceae.
## Veillonella                                                            Veillonella
## Veillonellaceae_[G.1]                                        Veillonellaceae_[G.1]
## Phylum.Firmicutes.                                              Phylum.Firmicutes.
## Fusobacterium                                                        Fusobacterium
## Leptotrichia                                                          Leptotrichia
## Gracilibacteria_(GN02)_[G.1]                          Gracilibacteria_(GN02)_[G.1]
## Lautropia                                                                Lautropia
## Ottowia                                                                    Ottowia
## Eikenella                                                                Eikenella
## Kingella                                                                  Kingella
## Neisseria                                                                Neisseria
## Family.Neisseriaceae.                                        Family.Neisseriaceae.
## Campylobacter                                                        Campylobacter
## Cardiobacterium                                                    Cardiobacterium
## Aggregatibacter                                                    Aggregatibacter
## Haemophilus                                                            Haemophilus
## Family.Pasteurellaceae.                                    Family.Pasteurellaceae.
## Saccharibacteria_(TM7)_[G.1]                          Saccharibacteria_(TM7)_[G.1]
## Saccharibacteria_(TM7)_[G.3]                          Saccharibacteria_(TM7)_[G.3]
## Saccharibacteria_(TM7)_[G.6]                          Saccharibacteria_(TM7)_[G.6]
## Family.Saccharibacteria_(TM7)_[F-1]. Family.Saccharibacteria_(TM7)_[F-1].
## Saccharibacteria_(TM7)_[G.5]                          Saccharibacteria_(TM7)_[G.5]
## Treponema                                                                Treponema
## Fretibacterium                                                      Fretibacterium
## Kingdom.Bacteria.                                                Kingdom.Bacteria.
## Others                                                                      Others
##                                                     Method
## Absconditabacteria_(SR1)_[G.1]       Kruskal-Wallis (kru)
## Actinomyces                          Kruskal-Wallis (kru)
## Peptidiphaga                         Kruskal-Wallis (kru)
## Schaalia                             Kruskal-Wallis (kru)
## Family.Actinomycetaceae.             Kruskal-Wallis (kru)
## Rothia                               Kruskal-Wallis (kru)
## Alloscardovia                        Kruskal-Wallis (kru)
## Bifidobacterium                      Kruskal-Wallis (kru)
## Parascardovia                        Kruskal-Wallis (kru)
## Scardovia                            Kruskal-Wallis (kru)
## Corynebacterium                      Kruskal-Wallis (kru)
## Olsenella                            Kruskal-Wallis (kru)
## Atopobium                            Kruskal-Wallis (kru)
```

```
## Slackia                              Kruskal-Wallis (kru)
## Cryptobacterium                      Kruskal-Wallis (kru)
## Bacteroidetes_[G.3]                  Kruskal-Wallis (kru)
## Bacteroidetes_[G.5]                  Kruskal-Wallis (kru)
## Family.Bacteroidetes_[F-1].          Kruskal-Wallis (kru)
## Bacteroidaceae_[G.1]                 Kruskal-Wallis (kru)
## Bacteroidales_[G.2]                  Kruskal-Wallis (kru)
## Porphyromonas                        Kruskal-Wallis (kru)
## Tannerella                           Kruskal-Wallis (kru)
## Alloprevotella                       Kruskal-Wallis (kru)
## Prevotella                           Kruskal-Wallis (kru)
## Bergeyella                           Kruskal-Wallis (kru)
## Capnocytophaga                       Kruskal-Wallis (kru)
## Gemella                              Kruskal-Wallis (kru)
## Abiotrophia                          Kruskal-Wallis (kru)
## Granulicatella                       Kruskal-Wallis (kru)
## Lactobacillus                        Kruskal-Wallis (kru)
## Streptococcus                        Kruskal-Wallis (kru)
## Order.Lactobacillales.               Kruskal-Wallis (kru)
## Class.Bacilli.                       Kruskal-Wallis (kru)
## Clostridiales_[F.1][G-1]             Kruskal-Wallis (kru)
## Butyrivibrio                         Kruskal-Wallis (kru)
## Catonella                            Kruskal-Wallis (kru)
## Johnsonella                          Kruskal-Wallis (kru)
## Lachnoanaerobaculum                  Kruskal-Wallis (kru)
## Lachnospiraceae_[G.2]                Kruskal-Wallis (kru)
## Lachnospiraceae_[G.3]                Kruskal-Wallis (kru)
## Lachnospiraceae_[G.7]                Kruskal-Wallis (kru)
## Lachnospiraceae_[G.8]                Kruskal-Wallis (kru)
## Oribacterium                         Kruskal-Wallis (kru)
## Shuttleworthia                       Kruskal-Wallis (kru)
## Stomatobaculum                       Kruskal-Wallis (kru)
## Family.Lachnospiraceae_[XIV].        Kruskal-Wallis (kru)
## Peptococcus                          Kruskal-Wallis (kru)
## Parvimonas                           Kruskal-Wallis (kru)
## Filifactor                           Kruskal-Wallis (kru)
## Mogibacterium                        Kruskal-Wallis (kru)
## Peptostreptococcaceae_[XI][G.1]      Kruskal-Wallis (kru)
## Peptostreptococcaceae_[XI][G.4]      Kruskal-Wallis (kru)
## Peptostreptococcaceae_[XI][G.5]      Kruskal-Wallis (kru)
## Peptostreptococcaceae_[XI][G.6]      Kruskal-Wallis (kru)
## Peptostreptococcaceae_[XI][G.7]      Kruskal-Wallis (kru)
## Peptostreptococcaceae_[XI][G.9]      Kruskal-Wallis (kru)
## Peptostreptococcus                   Kruskal-Wallis (kru)
## Ruminococcaceae_[G.1]                Kruskal-Wallis (kru)
## Ruminococcaceae_[G.2]                Kruskal-Wallis (kru)
## Bulleidia                            Kruskal-Wallis (kru)
## Eggerthia                            Kruskal-Wallis (kru)
## Solobacterium                        Kruskal-Wallis (kru)
## Mycoplasma                           Kruskal-Wallis (kru)
## Mitsuokella                          Kruskal-Wallis (kru)
## Selenomonas                          Kruskal-Wallis (kru)
## Family.Selenomonadaceae.             Kruskal-Wallis (kru)
## Class.Negativicutes.                 Kruskal-Wallis (kru)
```

```
## Anaeroglobus                           Kruskal-Wallis (kru)
## Dialister                              Kruskal-Wallis (kru)
## Megasphaera                            Kruskal-Wallis (kru)
## Family.Veillonellaceae.                Kruskal-Wallis (kru)
## Veillonella                            Kruskal-Wallis (kru)
## Veillonellaceae_[G.1]                  Kruskal-Wallis (kru)
## Phylum.Firmicutes.                     Kruskal-Wallis (kru)
## Fusobacterium                          Kruskal-Wallis (kru)
## Leptotrichia                           Kruskal-Wallis (kru)
## Gracilibacteria_(GN02)_[G.1]           Kruskal-Wallis (kru)
## Lautropia                              Kruskal-Wallis (kru)
## Ottowia                                Kruskal-Wallis (kru)
## Eikenella                              Kruskal-Wallis (kru)
## Kingella                               Kruskal-Wallis (kru)
## Neisseria                              Kruskal-Wallis (kru)
## Family.Neisseriaceae.                  Kruskal-Wallis (kru)
## Campylobacter                          Kruskal-Wallis (kru)
## Cardiobacterium                        Kruskal-Wallis (kru)
## Aggregatibacter                        Kruskal-Wallis (kru)
## Haemophilus                            Kruskal-Wallis (kru)
## Family.Pasteurellaceae.                Kruskal-Wallis (kru)
## Saccharibacteria_(TM7)_[G.1]           Kruskal-Wallis (kru)
## Saccharibacteria_(TM7)_[G.3]           Kruskal-Wallis (kru)
## Saccharibacteria_(TM7)_[G.6]           Kruskal-Wallis (kru)
## Family.Saccharibacteria_(TM7)_[F-1]. Kruskal-Wallis (kru)
## Saccharibacteria_(TM7)_[G.5]           Kruskal-Wallis (kru)
## Treponema                              Kruskal-Wallis (kru)
## Fretibacterium                         Kruskal-Wallis (kru)
## Kingdom.Bacteria.                      Kruskal-Wallis (kru)
## Others                                 Kruskal-Wallis (kru)
```

```
write.table(final, file="dbp_cat_kru.txt", sep="\t", dec=",", row.names=F)
write.table(final[final$pval<0.05,], file="dbp_cat_kru_sig.txt", sep="\t", dec=",", row.names=F)
#################hr_cat
table(Phe$hr_cat, useNA="always")
```

```
##
##    Low Medium   High   <NA>
##    345    344     55      2
```

```
#Remove NA
Phe2<-Phe[complete.cases(Phe$hr_cat),]
#Also subset columns
Microbio2<-dplyr::select(Microbio, one_of(Phe2$IDX))
final <- DA.ds2(Microbio2, predictor = Phe2$hr_cat, out.all=TRUE) #It is LRT so don't look at ordering
final
```

```
##                                          baseMean log2FoldChange      lfcSE
## Absconditabacteria_(SR1)_[G.1]         61.8342452   -0.604340193 0.25223042
## Actinomyces                           323.7803206    0.158803240 0.08619813
## Peptidiphaga                           22.0416883   -0.098873237 0.12342122
## Schaalia                              703.5570032   -0.112436983 0.07107378
## Family.Actinomycetaceae.                5.4318947   -0.320121705 0.17773984
```

```
## Rothia                              1938.6926786   -0.031744111 0.08339126
## Alloscardovia                         12.0844404   -0.191991089 0.21796605
## Bifidobacterium                       11.1408685    0.015048459 0.24088277
## Parascardovia                          2.9264171    0.760050484 0.32990509
## Scardovia                             14.3693609    0.628761547 0.21438683
## Corynebacterium                       93.8520152   -0.078412700 0.09119812
## Olsenella                              1.5705319    0.094619749 0.20231378
## Atopobium                            120.2270723    0.019545512 0.08347636
## Slackia                                1.3929535   -0.072889204 0.19344670
## Cryptobacterium                        4.2513998    0.342686110 0.20695146
## Bacteroidetes_[G.3]                    6.6074797    0.083293231 0.21928716
## Bacteroidetes_[G.5]                    4.6913133    0.056058764 0.23890950
## Family.Bacteroidetes_[F-1].            0.8714947    0.002641439 0.34224535
## Bacteroidaceae_[G.1]                   0.7948120   -0.083774359 0.32461610
## Bacteroidales_[G.2]                   12.0719990   -0.109032654 0.12543276
## Porphyromonas                        682.2933394   -0.071350544 0.11418328
## Tannerella                            22.0983847    0.148393814 0.08455254
## Alloprevotella                       362.5219516   -0.108132859 0.09081533
## Prevotella                          3807.2361865    0.010435041 0.05772246
## Bergeyella                            72.7155861   -0.052593119 0.08915730
## Capnocytophaga                       225.4613668    0.068967417 0.09529624
## Gemella                              309.8006772    0.017601452 0.07929317
## Abiotrophia                           30.0741451    0.163099361 0.17168938
## Granulicatella                       306.3404328    0.022161381 0.06930154
## Lactobacillus                         37.4327819    1.693259433 0.27817787
## Streptococcus                       3797.5698295    0.141846571 0.06490698
## Order.Lactobacillales.                 0.2871119    0.019676179 0.23513589
## Class.Bacilli.                         0.5755295   -0.101037264 0.18655690
## Clostridiales_[F.1][G-1]               1.0579178    0.107795663 0.28518761
## Butyrivibrio                           6.9656228   -0.281931206 0.18143040
## Catonella                             36.9095724   -0.109885199 0.10241277
## Johnsonella                            0.9421596   -0.072861113 0.24788605
## Lachnoanaerobaculum                   87.8820004    0.013124279 0.07171407
## Lachnospiraceae_[G.2]                 28.0534664   -0.121607183 0.18782266
## Lachnospiraceae_[G.3]                  2.8995123   -0.221003487 0.19445203
## Lachnospiraceae_[G.7]                  1.4732956    0.714102749 0.27797145
## Lachnospiraceae_[G.8]                  1.4367939    0.110979303 0.29749585
## Oribacterium                         147.1321858   -0.107677614 0.07925609
## Shuttleworthia                         2.8108966    0.449836431 0.22017204
## Stomatobaculum                       112.9182555   -0.012148350 0.08917874
## Family.Lachnospiraceae_[XIV].          1.5138789   -0.151558496 0.24822340
## Peptococcus                            3.7157473   -0.260050288 0.22125201
## Parvimonas                            40.9495178    0.013794987 0.11002506
## Filifactor                             6.8621336   -0.093832989 0.21468652
## Mogibacterium                         41.1723089   -0.153152038 0.07589570
## Peptostreptococcaceae_[XI][G.1]       67.7192574    0.010928397 0.13523251
## Peptostreptococcaceae_[XI][G.4]        0.4191905   -0.311566059 0.29499711
## Peptostreptococcaceae_[XI][G.5]        1.6690812    0.321297906 0.30843332
## Peptostreptococcaceae_[XI][G.6]        1.0175853   -0.001937727 0.30428564
## Peptostreptococcaceae_[XI][G.7]        4.7195993   -0.152993305 0.21204565
## Peptostreptococcaceae_[XI][G.9]        8.7363992   -0.076274122 0.12592437
## Peptostreptococcus                    42.9864069   -0.468940560 0.15731694
## Ruminococcaceae_[G.1]                 25.1836759   -0.058610287 0.16115984
## Ruminococcaceae_[G.2]                 41.5234417   -0.038200612 0.13422478
```

```
## Bulleidia                                3.8709078    0.118501637 0.23595245
## Eggerthia                                1.5130043    0.540032663 0.31403097
## Solobacterium                           74.1630338   -0.070134799 0.08945690
## Mycoplasma                               4.5000508   -0.020598800 0.18959663
## Mitsuokella                             26.7544753   -0.108170725 0.16933748
## Selenomonas                            222.3185156    0.001157420 0.07637647
## Family.Selenomonadaceae.                 5.0504886    0.103496260 0.19997191
## Class.Negativicutes.                     0.7322999    0.073032830 0.18397166
## Anaeroglobus                             8.7028189    0.639637096 0.18151200
## Dialister                               36.4852787    0.320384612 0.09327791
## Megasphaera                            407.7957370    0.129795073 0.09869525
## Family.Veillonellaceae.                  0.5239301    0.516581851 0.21435822
## Veillonella                           5061.2445718    0.059124144 0.05548965
## Veillonellaceae_[G.1]                    6.1469526    0.400862881 0.15417007
## Phylum.Firmicutes.                       0.8918217    0.468810226 0.25333568
## Fusobacterium                          929.2571372    0.056913765 0.07380672
## Leptotrichia                           684.6054135    0.140314733 0.07603802
## Gracilibacteria_(GN02)_[G.1]             2.4161467   -0.091747049 0.24597489
## Lautropia                               68.4994208   -0.016222581 0.14145430
## Ottowia                                  2.3379102   -0.534893567 0.31726987
## Eikenella                                8.7703431    0.281344054 0.11834023
## Kingella                               100.0995471    0.149210391 0.10196722
## Neisseria                             1333.1334336    0.094147597 0.11601324
## Family.Neisseriaceae.                    5.7037425   -0.834457513 0.26534172
## Campylobacter                          257.7162863   -0.029150776 0.06534222
## Cardiobacterium                         14.8139218   -0.122907453 0.10169000
## Aggregatibacter                        276.1135518    0.045870188 0.13466701
## Haemophilus                           2911.7582507   -0.167460290 0.08961995
## Family.Pasteurellaceae.                  2.5966784    0.317819029 0.36810986
## Saccharibacteria_(TM7)_[G.1]            36.1574542   -0.163771144 0.14949771
## Saccharibacteria_(TM7)_[G.3]             7.1401803    0.020301888 0.20856368
## Saccharibacteria_(TM7)_[G.6]             7.0191590   -0.290074300 0.26296232
## Family.Saccharibacteria_(TM7)_[F-1].     1.1040897   -0.300467504 0.32908581
## Saccharibacteria_(TM7)_[G.5]             0.9151491    0.699719339 0.31150605
## Treponema                               63.3764075   -0.050126636 0.14170193
## Fretibacterium                           8.2967790   -0.121295029 0.17182630
## Kingdom.Bacteria.                        9.4046214   -0.155854936 0.21771289
## Others                                  32.7138830   -0.031908023 0.14386329
##                                              stat         pval   ordering
## Absconditabacteria_(SR1)_[G.1]         10.87012889 4.360954e-03 Low>Medium
## Actinomyces                             6.86717027 3.227104e-02 Medium>Low
## Peptidiphaga                            1.57086030 4.559235e-01 Low>Medium
## Schaalia                                2.50652575 2.855715e-01 Low>Medium
## Family.Actinomycetaceae.                3.31929238 1.902063e-01 Low>Medium
## Rothia                                  0.48211123 7.857979e-01 Low>Medium
## Alloscardovia                           1.12408073 5.700448e-01 Low>Medium
## Bifidobacterium                         1.17552416 5.555692e-01 Medium>Low
## Parascardovia                           5.99905138 4.981069e-02 Medium>Low
## Scardovia                               9.45209884 8.861410e-03 Medium>Low
## Corynebacterium                         1.01581508 6.017534e-01 Low>Medium
## Olsenella                               1.17289400 5.563003e-01 Medium>Low
## Atopobium                               1.14132286 5.651515e-01 Medium>Low
## Slackia                                 0.34053503 8.434392e-01 Low>Medium
## Cryptobacterium                         2.81926506 2.442330e-01 Medium>Low
```

```
## Bacteroidetes_[G.3]                       1.22993014 5.406598e-01 Medium>Low
## Bacteroidetes_[G.5]                       0.15142670 9.270819e-01 Medium>Low
## Family.Bacteroidetes_[F-1].               4.18096707 1.236273e-01 Medium>Low
## Bacteroidaceae_[G.1]                      0.27499594 8.715361e-01 Low>Medium
## Bacteroidales_[G.2]                       0.84436209 6.556153e-01 Low>Medium
## Porphyromonas                             1.91031375 3.847518e-01 Low>Medium
## Tannerella                                3.76864975 1.519316e-01 Medium>Low
## Alloprevotella                            2.54872679 2.796089e-01 Low>Medium
## Prevotella                                0.39383386 8.212589e-01 Medium>Low
## Bergeyella                                0.70292401 7.036586e-01 Low>Medium
## Capnocytophaga                            0.92464475 6.298193e-01 Medium>Low
## Gemella                                   0.58859077 7.450564e-01 Medium>Low
## Abiotrophia                               2.61173324 2.709376e-01 Medium>Low
## Granulicatella                            4.29919176 1.165312e-01 Medium>Low
## Lactobacillus                            40.46312197 1.635101e-09 Medium>Low
## Streptococcus                             6.23308959 4.431000e-02 Medium>Low
## Order.Lactobacillales.                   -3.74390856 1.000000e+00 Medium>Low
## Class.Bacilli.                            5.56963579 6.174033e-02 Low>Medium
## Clostridiales_[F.1][G-1]                  1.97743058 3.720544e-01 Medium>Low
## Butyrivibrio                              2.77216630 2.500528e-01 Low>Medium
## Catonella                                 2.04761719 3.592242e-01 Low>Medium
## Johnsonella                               0.86926112 6.475038e-01 Low>Medium
## Lachnoanaerobaculum                       0.76170310 6.832793e-01 Medium>Low
## Lachnospiraceae_[G.2]                     1.63558965 4.414040e-01 Low>Medium
## Lachnospiraceae_[G.3]                     1.62604998 4.435144e-01 Low>Medium
## Lachnospiraceae_[G.7]                     6.98541965 3.041833e-02 Medium>Low
## Lachnospiraceae_[G.8]                     0.26895076 8.741744e-01 Medium>Low
## Oribacterium                              3.91208620 1.414169e-01 Low>Medium
## Shuttleworthia                            4.37736820 1.120641e-01 Medium>Low
## Stomatobaculum                            2.61258623 2.708221e-01 Low>Medium
## Family.Lachnospiraceae_[XIV].             0.58448942 7.465858e-01 Low>Medium
## Peptococcus                               1.50811117 4.704547e-01 Low>Medium
## Parvimonas                                2.65356355 2.653298e-01 Medium>Low
## Filifactor                                0.78124380 6.766359e-01 Low>Medium
## Mogibacterium                             4.37790548 1.120340e-01 Low>Medium
## Peptostreptococcaceae_[XI][G.1]           2.26250605 3.226287e-01 Medium>Low
## Peptostreptococcaceae_[XI][G.4]           1.34646243 5.100578e-01 Low>Medium
## Peptostreptococcaceae_[XI][G.5]           1.30322457 5.212048e-01 Medium>Low
## Peptostreptococcaceae_[XI][G.6]           2.27767224 3.201915e-01 Low>Medium
## Peptostreptococcaceae_[XI][G.7]           0.66457270 7.172819e-01 Low>Medium
## Peptostreptococcaceae_[XI][G.9]           2.03797934 3.609594e-01 Low>Medium
## Peptostreptococcus                       10.17497372 6.173515e-03 Low>Medium
## Ruminococcaceae_[G.1]                     1.06787665 5.862914e-01 Low>Medium
## Ruminococcaceae_[G.2]                     1.73872986 4.192177e-01 Low>Medium
## Bulleidia                                 3.56004428 1.686344e-01 Medium>Low
## Eggerthia                                 3.07031973 2.154213e-01 Medium>Low
## Solobacterium                             2.48534416 2.886120e-01 Low>Medium
## Mycoplasma                                0.07505709 9.631669e-01 Low>Medium
## Mitsuokella                               5.82322064 5.438808e-02 Low>Medium
## Selenomonas                               0.23745695 8.880489e-01 Medium>Low
## Family.Selenomonadaceae.                  0.28699184 8.663243e-01 Medium>Low
## Class.Negativicutes.                      2.77011065 2.503100e-01 Medium>Low
## Anaeroglobus                             13.64164894 1.090821e-03 Medium>Low
## Dialister                                11.85444418 2.665877e-03 Medium>Low
```

```
## Megasphaera                          2.60850689 2.713751e-01 Medium>Low
## Family.Veillonellaceae.              6.98335128 3.044981e-02 Medium>Low
## Veillonella                          1.23248880 5.399685e-01 Medium>Low
## Veillonellaceae_[G.1]                7.29238887 2.609023e-02 Medium>Low
## Phylum.Firmicutes.                   4.93074781 8.497706e-02 Medium>Low
## Fusobacterium                        2.26704531 3.218973e-01 Medium>Low
## Leptotrichia                         3.55432389 1.691174e-01 Medium>Low
## Gracilibacteria_(GN02)_[G.1]         0.70772436 7.019717e-01 Low>Medium
## Lautropia                            1.63227630 4.421358e-01 Low>Medium
## Ottowia                              2.82045072 2.440883e-01 Low>Medium
## Eikenella                            5.84516730 5.379452e-02 Medium>Low
## Kingella                             2.21023964 3.311712e-01 Medium>Low
## Neisseria                            1.13409273 5.671983e-01 Medium>Low
## Family.Neisseriaceae.                9.78242409 7.512312e-03 Low>Medium
## Campylobacter                        2.54695618 2.798566e-01 Low>Medium
## Cardiobacterium                      2.60471425 2.718902e-01 Low>Medium
## Aggregatibacter                      0.46266766 7.934745e-01 Medium>Low
## Haemophilus                          4.21434934 1.215810e-01 Low>Medium
## Family.Pasteurellaceae.              3.68117317 1.587243e-01 Medium>Low
## Saccharibacteria_(TM7)_[G.1]         4.23018215 1.206223e-01 Low>Medium
## Saccharibacteria_(TM7)_[G.3]         0.13737217 9.336197e-01 Medium>Low
## Saccharibacteria_(TM7)_[G.6]         8.38896056 1.507858e-02 Low>Medium
## Family.Saccharibacteria_(TM7)_[F-1]. 1.78887929 4.088366e-01 Low>Medium
## Saccharibacteria_(TM7)_[G.5]         5.58092265 6.139289e-02 Medium>Low
## Treponema                            6.89560608 3.181546e-02 Low>Medium
## Fretibacterium                       1.23911487 5.381826e-01 Low>Medium
## Kingdom.Bacteria.                    0.61010245 7.370856e-01 Low>Medium
## Others                               4.41977273 1.097131e-01 Low>Medium
##                                          pval.adj
## Absconditabacteria_(SR1)_[G.1]       1.057531e-01
## Actinomyces                          2.407916e-01
## Peptidiphaga                         7.249932e-01
## Schaalia                             5.956460e-01
## Family.Actinomycetaceae.             5.590911e-01
## Rothia                               8.846785e-01
## Alloscardovia                        7.679770e-01
## Bifidobacterium                      7.679770e-01
## Parascardovia                        3.103320e-01
## Scardovia                            1.227938e-01
## Corynebacterium                      7.887849e-01
## Olsenella                            7.679770e-01
## Atopobium                            7.679770e-01
## Slackia                              9.192539e-01
## Cryptobacterium                      5.956460e-01
## Bacteroidetes_[G.3]                  7.679770e-01
## Bacteroidetes_[G.5]                  9.532749e-01
## Family.Bacteroidetes_[F-1].          4.441427e-01
## Bacteroidaceae_[G.1]                 9.216839e-01
## Bacteroidales_[G.2]                  8.259050e-01
## Porphyromonas                        6.785622e-01
## Tannerella                           5.081850e-01
## Alloprevotella                       5.956460e-01
## Prevotella                           9.052512e-01
## Bergeyella                           8.426529e-01
```

```
## Capnocytophaga                        8.145663e-01
## Gemella                               8.519862e-01
## Abiotrophia                           5.956460e-01
## Granulicatella                        4.441427e-01
## Lactobacillus                         1.586048e-07
## Streptococcus                         3.070050e-01
## Order.Lactobacillales.                1.000000e+00
## Class.Bacilli.                        3.152006e-01
## Clostridiales_[F.1][G-1]              6.683199e-01
## Butyrivibrio                          5.956460e-01
## Catonella                             6.606239e-01
## Johnsonella                           8.259050e-01
## Lachnoanaerobaculum                   8.389632e-01
## Lachnospiraceae_[G.2]                 7.170150e-01
## Lachnospiraceae_[G.3]                 7.170150e-01
## Lachnospiraceae_[G.7]                 2.407916e-01
## Lachnospiraceae_[G.8]                 9.216839e-01
## Oribacterium                          4.899085e-01
## Shuttleworthia                        4.441427e-01
## Stomatobaculum                        5.956460e-01
## Family.Lachnospiraceae_[XIV].         8.519862e-01
## Peptococcus                           7.360340e-01
## Parvimonas                            5.956460e-01
## Filifactor                            8.389632e-01
## Mogibacterium                         4.441427e-01
## Peptostreptococcaceae_[XI][G.1]       6.258998e-01
## Peptostreptococcaceae_[XI][G.4]       7.679770e-01
## Peptostreptococcaceae_[XI][G.5]       7.679770e-01
## Peptostreptococcaceae_[XI][G.6]       6.258998e-01
## Peptostreptococcaceae_[XI][G.7]       8.484920e-01
## Peptostreptococcaceae_[XI][G.9]       6.606239e-01
## Peptostreptococcus                    1.197662e-01
## Ruminococcaceae_[G.1]                 7.790448e-01
## Ruminococcaceae_[G.2]                 7.134056e-01
## Bulleidia                             5.126372e-01
## Eggerthia                             5.956460e-01
## Solobacterium                         5.956460e-01
## Mycoplasma                            9.731999e-01
## Mitsuokella                           3.103320e-01
## Selenomonas                           9.262445e-01
## Family.Selenomonadaceae.              9.216839e-01
## Class.Negativicutes.                  5.956460e-01
## Anaeroglobus                          5.290483e-02
## Dialister                             8.619670e-02
## Megasphaera                           5.956460e-01
## Family.Veillonellaceae.               2.407916e-01
## Veillonella                           7.679770e-01
## Veillonellaceae_[G.1]                 2.407916e-01
## Phylum.Firmicutes.                    4.121388e-01
## Fusobacterium                         6.258998e-01
## Leptotrichia                          5.126372e-01
## Gracilibacteria_(GN02)_[G.1]          8.426529e-01
## Lautropia                             7.170150e-01
## Ottowia                               5.956460e-01
```

```
## Eikenella                            3.103320e-01
## Kingella                             6.298746e-01
## Neisseria                            7.679770e-01
## Family.Neisseriaceae.                1.214490e-01
## Campylobacter                        5.956460e-01
## Cardiobacterium                      5.956460e-01
## Aggregatibacter                      8.846785e-01
## Haemophilus                          4.441427e-01
## Family.Pasteurellaceae.              5.126372e-01
## Saccharibacteria_(TM7)_[G.1]         4.441427e-01
## Saccharibacteria_(TM7)_[G.3]         9.532749e-01
## Saccharibacteria_(TM7)_[G.6]         1.828277e-01
## Family.Saccharibacteria_(TM7)_[F-1]. 7.081634e-01
## Saccharibacteria_(TM7)_[G.5]         3.152006e-01
## Treponema                            2.407916e-01
## Fretibacterium                       7.679770e-01
## Kingdom.Bacteria.                    8.519862e-01
## Others                               4.441427e-01
##                                                                   Feature
## Absconditabacteria_(SR1)_[G.1]             Absconditabacteria_(SR1)_[G.1]
## Actinomyces                                                   Actinomyces
## Peptidiphaga                                                 Peptidiphaga
## Schaalia                                                         Schaalia
## Family.Actinomycetaceae.                         Family.Actinomycetaceae.
## Rothia                                                             Rothia
## Alloscardovia                                               Alloscardovia
## Bifidobacterium                                           Bifidobacterium
## Parascardovia                                               Parascardovia
## Scardovia                                                       Scardovia
## Corynebacterium                                           Corynebacterium
## Olsenella                                                       Olsenella
## Atopobium                                                       Atopobium
## Slackia                                                           Slackia
## Cryptobacterium                                           Cryptobacterium
## Bacteroidetes_[G.3]                                   Bacteroidetes_[G.3]
## Bacteroidetes_[G.5]                                   Bacteroidetes_[G.5]
## Family.Bacteroidetes_[F-1].                   Family.Bacteroidetes_[F-1].
## Bacteroidaceae_[G.1]                                 Bacteroidaceae_[G.1]
## Bacteroidales_[G.2]                                   Bacteroidales_[G.2]
## Porphyromonas                                               Porphyromonas
## Tannerella                                                     Tannerella
## Alloprevotella                                             Alloprevotella
## Prevotella                                                     Prevotella
## Bergeyella                                                     Bergeyella
## Capnocytophaga                                             Capnocytophaga
## Gemella                                                           Gemella
## Abiotrophia                                                   Abiotrophia
## Granulicatella                                             Granulicatella
## Lactobacillus                                               Lactobacillus
## Streptococcus                                               Streptococcus
## Order.Lactobacillales.                             Order.Lactobacillales.
## Class.Bacilli.                                             Class.Bacilli.
## Clostridiales_[F.1][G-1]                         Clostridiales_[F.1][G-1]
## Butyrivibrio                                                 Butyrivibrio
```

```
## Catonella                                                      Catonella
## Johnsonella                                                  Johnsonella
## Lachnoanaerobaculum                                  Lachnoanaerobaculum
## Lachnospiraceae_[G.2]                              Lachnospiraceae_[G.2]
## Lachnospiraceae_[G.3]                              Lachnospiraceae_[G.3]
## Lachnospiraceae_[G.7]                              Lachnospiraceae_[G.7]
## Lachnospiraceae_[G.8]                              Lachnospiraceae_[G.8]
## Oribacterium                                                Oribacterium
## Shuttleworthia                                            Shuttleworthia
## Stomatobaculum                                            Stomatobaculum
## Family.Lachnospiraceae_[XIV].          Family.Lachnospiraceae_[XIV].
## Peptococcus                                                  Peptococcus
## Parvimonas                                                    Parvimonas
## Filifactor                                                    Filifactor
## Mogibacterium                                              Mogibacterium
## Peptostreptococcaceae_[XI][G.1]      Peptostreptococcaceae_[XI][G.1]
## Peptostreptococcaceae_[XI][G.4]      Peptostreptococcaceae_[XI][G.4]
## Peptostreptococcaceae_[XI][G.5]      Peptostreptococcaceae_[XI][G.5]
## Peptostreptococcaceae_[XI][G.6]      Peptostreptococcaceae_[XI][G.6]
## Peptostreptococcaceae_[XI][G.7]      Peptostreptococcaceae_[XI][G.7]
## Peptostreptococcaceae_[XI][G.9]      Peptostreptococcaceae_[XI][G.9]
## Peptostreptococcus                                    Peptostreptococcus
## Ruminococcaceae_[G.1]                              Ruminococcaceae_[G.1]
## Ruminococcaceae_[G.2]                              Ruminococcaceae_[G.2]
## Bulleidia                                                      Bulleidia
## Eggerthia                                                      Eggerthia
## Solobacterium                                              Solobacterium
## Mycoplasma                                                    Mycoplasma
## Mitsuokella                                                  Mitsuokella
## Selenomonas                                                  Selenomonas
## Family.Selenomonadaceae.                        Family.Selenomonadaceae.
## Class.Negativicutes.                                Class.Negativicutes.
## Anaeroglobus                                                Anaeroglobus
## Dialister                                                      Dialister
## Megasphaera                                                  Megasphaera
## Family.Veillonellaceae.                          Family.Veillonellaceae.
## Veillonella                                                  Veillonella
## Veillonellaceae_[G.1]                              Veillonellaceae_[G.1]
## Phylum.Firmicutes.                                    Phylum.Firmicutes.
## Fusobacterium                                              Fusobacterium
## Leptotrichia                                                Leptotrichia
## Gracilibacteria_(GN02)_[G.1]                Gracilibacteria_(GN02)_[G.1]
## Lautropia                                                      Lautropia
## Ottowia                                                          Ottowia
## Eikenella                                                      Eikenella
## Kingella                                                        Kingella
## Neisseria                                                      Neisseria
## Family.Neisseriaceae.                              Family.Neisseriaceae.
## Campylobacter                                              Campylobacter
## Cardiobacterium                                          Cardiobacterium
## Aggregatibacter                                          Aggregatibacter
## Haemophilus                                                  Haemophilus
## Family.Pasteurellaceae.                          Family.Pasteurellaceae.
## Saccharibacteria_(TM7)_[G.1]                Saccharibacteria_(TM7)_[G.1]
```

```
## Saccharibacteria_(TM7)_[G.3]                      Saccharibacteria_(TM7)_[G.3]
## Saccharibacteria_(TM7)_[G.6]                      Saccharibacteria_(TM7)_[G.6]
## Family.Saccharibacteria_(TM7)_[F-1]. Family.Saccharibacteria_(TM7)_[F-1].
## Saccharibacteria_(TM7)_[G.5]                      Saccharibacteria_(TM7)_[G.5]
## Treponema                                                            Treponema
## Fretibacterium                                                  Fretibacterium
## Kingdom.Bacteria.                                            Kingdom.Bacteria.
## Others                                                                  Others
##                                                              Method
## Absconditabacteria_(SR1)_[G.1]       DESeq2 man. geoMeans (ds2)
## Actinomyces                          DESeq2 man. geoMeans (ds2)
## Peptidiphaga                         DESeq2 man. geoMeans (ds2)
## Schaalia                             DESeq2 man. geoMeans (ds2)
## Family.Actinomycetaceae.             DESeq2 man. geoMeans (ds2)
## Rothia                               DESeq2 man. geoMeans (ds2)
## Alloscardovia                        DESeq2 man. geoMeans (ds2)
## Bifidobacterium                      DESeq2 man. geoMeans (ds2)
## Parascardovia                        DESeq2 man. geoMeans (ds2)
## Scardovia                            DESeq2 man. geoMeans (ds2)
## Corynebacterium                      DESeq2 man. geoMeans (ds2)
## Olsenella                            DESeq2 man. geoMeans (ds2)
## Atopobium                            DESeq2 man. geoMeans (ds2)
## Slackia                              DESeq2 man. geoMeans (ds2)
## Cryptobacterium                      DESeq2 man. geoMeans (ds2)
## Bacteroidetes_[G.3]                  DESeq2 man. geoMeans (ds2)
## Bacteroidetes_[G.5]                  DESeq2 man. geoMeans (ds2)
## Family.Bacteroidetes_[F-1].          DESeq2 man. geoMeans (ds2)
## Bacteroidaceae_[G.1]                 DESeq2 man. geoMeans (ds2)
## Bacteroidales_[G.2]                  DESeq2 man. geoMeans (ds2)
## Porphyromonas                        DESeq2 man. geoMeans (ds2)
## Tannerella                           DESeq2 man. geoMeans (ds2)
## Alloprevotella                       DESeq2 man. geoMeans (ds2)
## Prevotella                           DESeq2 man. geoMeans (ds2)
## Bergeyella                           DESeq2 man. geoMeans (ds2)
## Capnocytophaga                       DESeq2 man. geoMeans (ds2)
## Gemella                              DESeq2 man. geoMeans (ds2)
## Abiotrophia                          DESeq2 man. geoMeans (ds2)
## Granulicatella                       DESeq2 man. geoMeans (ds2)
## Lactobacillus                        DESeq2 man. geoMeans (ds2)
## Streptococcus                        DESeq2 man. geoMeans (ds2)
## Order.Lactobacillales.               DESeq2 man. geoMeans (ds2)
## Class.Bacilli.                       DESeq2 man. geoMeans (ds2)
## Clostridiales_[F.1][G-1]             DESeq2 man. geoMeans (ds2)
## Butyrivibrio                         DESeq2 man. geoMeans (ds2)
## Catonella                            DESeq2 man. geoMeans (ds2)
## Johnsonella                          DESeq2 man. geoMeans (ds2)
## Lachnoanaerobaculum                  DESeq2 man. geoMeans (ds2)
## Lachnospiraceae_[G.2]                DESeq2 man. geoMeans (ds2)
## Lachnospiraceae_[G.3]                DESeq2 man. geoMeans (ds2)
## Lachnospiraceae_[G.7]                DESeq2 man. geoMeans (ds2)
## Lachnospiraceae_[G.8]                DESeq2 man. geoMeans (ds2)
## Oribacterium                         DESeq2 man. geoMeans (ds2)
## Shuttleworthia                       DESeq2 man. geoMeans (ds2)
## Stomatobaculum                       DESeq2 man. geoMeans (ds2)
```

```
## Family.Lachnospiraceae_[XIV].        DESeq2 man. geoMeans (ds2)
## Peptococcus                          DESeq2 man. geoMeans (ds2)
## Parvimonas                           DESeq2 man. geoMeans (ds2)
## Filifactor                           DESeq2 man. geoMeans (ds2)
## Mogibacterium                        DESeq2 man. geoMeans (ds2)
## Peptostreptococcaceae_[XI][G.1]      DESeq2 man. geoMeans (ds2)
## Peptostreptococcaceae_[XI][G.4]      DESeq2 man. geoMeans (ds2)
## Peptostreptococcaceae_[XI][G.5]      DESeq2 man. geoMeans (ds2)
## Peptostreptococcaceae_[XI][G.6]      DESeq2 man. geoMeans (ds2)
## Peptostreptococcaceae_[XI][G.7]      DESeq2 man. geoMeans (ds2)
## Peptostreptococcaceae_[XI][G.9]      DESeq2 man. geoMeans (ds2)
## Peptostreptococcus                   DESeq2 man. geoMeans (ds2)
## Ruminococcaceae_[G.1]                DESeq2 man. geoMeans (ds2)
## Ruminococcaceae_[G.2]                DESeq2 man. geoMeans (ds2)
## Bulleidia                            DESeq2 man. geoMeans (ds2)
## Eggerthia                            DESeq2 man. geoMeans (ds2)
## Solobacterium                        DESeq2 man. geoMeans (ds2)
## Mycoplasma                           DESeq2 man. geoMeans (ds2)
## Mitsuokella                          DESeq2 man. geoMeans (ds2)
## Selenomonas                          DESeq2 man. geoMeans (ds2)
## Family.Selenomonadaceae.             DESeq2 man. geoMeans (ds2)
## Class.Negativicutes.                 DESeq2 man. geoMeans (ds2)
## Anaeroglobus                         DESeq2 man. geoMeans (ds2)
## Dialister                            DESeq2 man. geoMeans (ds2)
## Megasphaera                          DESeq2 man. geoMeans (ds2)
## Family.Veillonellaceae.              DESeq2 man. geoMeans (ds2)
## Veillonella                          DESeq2 man. geoMeans (ds2)
## Veillonellaceae_[G.1]                DESeq2 man. geoMeans (ds2)
## Phylum.Firmicutes.                   DESeq2 man. geoMeans (ds2)
## Fusobacterium                        DESeq2 man. geoMeans (ds2)
## Leptotrichia                         DESeq2 man. geoMeans (ds2)
## Gracilibacteria_(GN02)_[G.1]         DESeq2 man. geoMeans (ds2)
## Lautropia                            DESeq2 man. geoMeans (ds2)
## Ottowia                              DESeq2 man. geoMeans (ds2)
## Eikenella                            DESeq2 man. geoMeans (ds2)
## Kingella                             DESeq2 man. geoMeans (ds2)
## Neisseria                            DESeq2 man. geoMeans (ds2)
## Family.Neisseriaceae.                DESeq2 man. geoMeans (ds2)
## Campylobacter                        DESeq2 man. geoMeans (ds2)
## Cardiobacterium                      DESeq2 man. geoMeans (ds2)
## Aggregatibacter                      DESeq2 man. geoMeans (ds2)
## Haemophilus                          DESeq2 man. geoMeans (ds2)
## Family.Pasteurellaceae.              DESeq2 man. geoMeans (ds2)
## Saccharibacteria_(TM7)_[G.1]         DESeq2 man. geoMeans (ds2)
## Saccharibacteria_(TM7)_[G.3]         DESeq2 man. geoMeans (ds2)
## Saccharibacteria_(TM7)_[G.6]         DESeq2 man. geoMeans (ds2)
## Family.Saccharibacteria_(TM7)_[F-1]. DESeq2 man. geoMeans (ds2)
## Saccharibacteria_(TM7)_[G.5]         DESeq2 man. geoMeans (ds2)
## Treponema                            DESeq2 man. geoMeans (ds2)
## Fretibacterium                       DESeq2 man. geoMeans (ds2)
## Kingdom.Bacteria.                    DESeq2 man. geoMeans (ds2)
## Others                               DESeq2 man. geoMeans (ds2)
```

```r
write.table(final, file="hr_cat_ds2.txt", sep="\t", dec=",", row.names=F)
write.table(final[final$pval<0.05,], file="hr_cat_ds2_sig.txt", sep="\t", dec=",", row.names=F)
final <- DA.kru(Microbio2, predictor = Phe2$hr_cat)
final
```

```
##                                           pval  pval.adj
## Absconditabacteria_(SR1)_[G.1]      0.56957008 0.8685866
## Actinomyces                         0.46307495 0.8685866
## Peptidiphaga                        0.38418386 0.8271121
## Schaalia                            0.05103492 0.4178948
## Family.Actinomycetaceae.            0.10155115 0.5472479
## Rothia                              0.95095060 0.9706076
## Alloscardovia                       0.63232010 0.8685866
## Bifidobacterium                     0.97933800 0.9793380
## Parascardovia                       0.02637329 0.3838627
## Scardovia                           0.50485886 0.8685866
## Corynebacterium                     0.56511464 0.8685866
## Olsenella                           0.55540749 0.8685866
## Atopobium                           0.37737120 0.8271121
## Slackia                             0.96060129 0.9706076
## Cryptobacterium                     0.19910298 0.6832463
## Bacteroidetes_[G.3]                 0.20426952 0.6832463
## Bacteroidetes_[G.5]                 0.41663674 0.8598673
## Family.Bacteroidetes_[F-1].         0.18425458 0.6832463
## Bacteroidaceae_[G.1]                0.83936747 0.9578078
## Bacteroidales_[G.2]                 0.84658917 0.9578078
## Porphyromonas                       0.09914622 0.5472479
## Tannerella                          0.63960290 0.8685866
## Alloprevotella                      0.50936023 0.8685866
## Prevotella                          0.58760454 0.8685866
## Bergeyella                          0.39223873 0.8271121
## Capnocytophaga                      0.89567411 0.9578078
## Gemella                             0.13564761 0.6251540
## Abiotrophia                         0.12882042 0.6247790
## Granulicatella                      0.01731613 0.3838627
## Lactobacillus                       0.31562751 0.7909073
## Streptococcus                       0.60151810 0.8685866
## Order.Lactobacillales.              0.81058417 0.9578078
## Class.Bacilli.                      0.69819589 0.8915674
## Clostridiales_[F.1][G-1]            0.69572223 0.8915674
## Butyrivibrio                        0.50627778 0.8685866
## Catonella                           0.23668194 0.6957015
## Johnsonella                         0.81282885 0.9578078
## Lachnoanaerobaculum                 0.88042428 0.9578078
## Lachnospiraceae_[G.2]               0.15404534 0.6471759
## Lachnospiraceae_[G.3]               0.35290450 0.8098617
## Lachnospiraceae_[G.7]               0.46079345 0.8685866
## Lachnospiraceae_[G.8]               0.78039611 0.9578078
## Oribacterium                        0.14178752 0.6251540
## Shuttleworthia                      0.07959319 0.4878959
## Stomatobaculum                      0.53421392 0.8685866
## Family.Lachnospiraceae_[XIV].       0.08047768 0.4878959
## Peptococcus                         0.90150802 0.9578078
```

```
## Parvimonas                             0.93260461 0.9623686
## Filifactor                             0.62474567 0.8685866
## Mogibacterium                          0.21769669 0.6862788
## Peptostreptococcaceae_[XI][G.1]        0.35901086 0.8098617
## Peptostreptococcaceae_[XI][G.4]        0.58469973 0.8685866
## Peptostreptococcaceae_[XI][G.5]        0.74278182 0.9357122
## Peptostreptococcaceae_[XI][G.6]        0.16012600 0.6471759
## Peptostreptococcaceae_[XI][G.7]        0.54683639 0.8685866
## Peptostreptococcaceae_[XI][G.9]        0.90109052 0.9578078
## Peptostreptococcus                     0.02770143 0.3838627
## Ruminococcaceae_[G.1]                  0.80858504 0.9578078
## Ruminococcaceae_[G.2]                  0.48435728 0.8685866
## Bulleidia                              0.22594252 0.6862788
## Eggerthia                              0.06031472 0.4178948
## Solobacterium                          0.32223843 0.7909073
## Mycoplasma                             0.82343838 0.9578078
## Mitsuokella                            0.10873591 0.5551254
## Selenomonas                            0.66463918 0.8831507
## Family.Selenomonadaceae.               0.47240441 0.8685866
## Class.Negativicutes.                   0.91441905 0.9578078
## Anaeroglobus                           0.04233987 0.4178948
## Dialister                              0.05506021 0.4178948
## Megasphaera                            0.05428614 0.4178948
## Family.Veillonellaceae.                0.22640125 0.6862788
## Veillonella                            0.19373486 0.6832463
## Veillonellaceae_[G.1]                  0.02036167 0.3838627
## Phylum.Firmicutes.                     0.79342688 0.9578078
## Fusobacterium                          0.51537970 0.8685866
## Leptotrichia                           0.32724975 0.7909073
## Gracilibacteria_(GN02)_[G.1]           0.30978031 0.7909073
## Lautropia                              0.05665333 0.4178948
## Ottowia                                0.28735505 0.7909073
## Eikenella                              0.69854767 0.8915674
## Kingella                               0.33430102 0.7909073
## Neisseria                              0.18152471 0.6832463
## Family.Neisseriaceae.                  0.64472406 0.8685866
## Campylobacter                          0.63374376 0.8685866
## Cardiobacterium                        0.59039439 0.8685866
## Aggregatibacter                        0.60724844 0.8685866
## Haemophilus                            0.01076618 0.3838627
## Family.Pasteurellaceae.                0.27850225 0.7909073
## Saccharibacteria_(TM7)_[G.1]           0.53183420 0.8685866
## Saccharibacteria_(TM7)_[G.3]           0.91805295 0.9578078
## Saccharibacteria_(TM7)_[G.6]           0.01861712 0.3838627
## Family.Saccharibacteria_(TM7)_[F-1]. 0.31536133 0.7909073
## Saccharibacteria_(TM7)_[G.5]           0.01189818 0.3838627
## Treponema                              0.91831061 0.9578078
## Fretibacterium                         0.58436118 0.8685866
## Kingdom.Bacteria.                      0.90550722 0.9578078
## Others                                 0.05618999 0.4178948
##                                                                Feature
## Absconditabacteria_(SR1)_[G.1]             Absconditabacteria_(SR1)_[G.1]
## Actinomyces                                                Actinomyces
## Peptidiphaga                                              Peptidiphaga
```

```
## Schaalia                                                              Schaalia
## Family.Actinomycetaceae.                              Family.Actinomycetaceae.
## Rothia                                                                  Rothia
## Alloscardovia                                                    Alloscardovia
## Bifidobacterium                                                Bifidobacterium
## Parascardovia                                                    Parascardovia
## Scardovia                                                            Scardovia
## Corynebacterium                                                Corynebacterium
## Olsenella                                                            Olsenella
## Atopobium                                                            Atopobium
## Slackia                                                                Slackia
## Cryptobacterium                                                Cryptobacterium
## Bacteroidetes_[G.3]                                        Bacteroidetes_[G.3]
## Bacteroidetes_[G.5]                                        Bacteroidetes_[G.5]
## Family.Bacteroidetes_[F-1].                        Family.Bacteroidetes_[F-1].
## Bacteroidaceae_[G.1]                                      Bacteroidaceae_[G.1]
## Bacteroidales_[G.2]                                        Bacteroidales_[G.2]
## Porphyromonas                                                    Porphyromonas
## Tannerella                                                          Tannerella
## Alloprevotella                                                  Alloprevotella
## Prevotella                                                          Prevotella
## Bergeyella                                                          Bergeyella
## Capnocytophaga                                                  Capnocytophaga
## Gemella                                                                Gemella
## Abiotrophia                                                        Abiotrophia
## Granulicatella                                                  Granulicatella
## Lactobacillus                                                    Lactobacillus
## Streptococcus                                                    Streptococcus
## Order.Lactobacillales.                                  Order.Lactobacillales.
## Class.Bacilli.                                                  Class.Bacilli.
## Clostridiales_[F.1][G-1]                              Clostridiales_[F.1][G-1]
## Butyrivibrio                                                      Butyrivibrio
## Catonella                                                            Catonella
## Johnsonella                                                        Johnsonella
## Lachnoanaerobaculum                                        Lachnoanaerobaculum
## Lachnospiraceae_[G.2]                                    Lachnospiraceae_[G.2]
## Lachnospiraceae_[G.3]                                    Lachnospiraceae_[G.3]
## Lachnospiraceae_[G.7]                                    Lachnospiraceae_[G.7]
## Lachnospiraceae_[G.8]                                    Lachnospiraceae_[G.8]
## Oribacterium                                                      Oribacterium
## Shuttleworthia                                                  Shuttleworthia
## Stomatobaculum                                                  Stomatobaculum
## Family.Lachnospiraceae_[XIV].                    Family.Lachnospiraceae_[XIV].
## Peptococcus                                                        Peptococcus
## Parvimonas                                                          Parvimonas
## Filifactor                                                          Filifactor
## Mogibacterium                                                    Mogibacterium
## Peptostreptococcaceae_[XI][G.1]                Peptostreptococcaceae_[XI][G.1]
## Peptostreptococcaceae_[XI][G.4]                Peptostreptococcaceae_[XI][G.4]
## Peptostreptococcaceae_[XI][G.5]                Peptostreptococcaceae_[XI][G.5]
## Peptostreptococcaceae_[XI][G.6]                Peptostreptococcaceae_[XI][G.6]
## Peptostreptococcaceae_[XI][G.7]                Peptostreptococcaceae_[XI][G.7]
## Peptostreptococcaceae_[XI][G.9]                Peptostreptococcaceae_[XI][G.9]
## Peptostreptococcus                                          Peptostreptococcus
```

```
## Ruminococcaceae_[G.1]                                    Ruminococcaceae_[G.1]
## Ruminococcaceae_[G.2]                                    Ruminococcaceae_[G.2]
## Bulleidia                                                            Bulleidia
## Eggerthia                                                            Eggerthia
## Solobacterium                                                    Solobacterium
## Mycoplasma                                                          Mycoplasma
## Mitsuokella                                                        Mitsuokella
## Selenomonas                                                        Selenomonas
## Family.Selenomonadaceae.                                Family.Selenomonadaceae.
## Class.Negativicutes.                                        Class.Negativicutes.
## Anaeroglobus                                                      Anaeroglobus
## Dialister                                                            Dialister
## Megasphaera                                                        Megasphaera
## Family.Veillonellaceae.                                  Family.Veillonellaceae.
## Veillonella                                                        Veillonella
## Veillonellaceae_[G.1]                                    Veillonellaceae_[G.1]
## Phylum.Firmicutes.                                          Phylum.Firmicutes.
## Fusobacterium                                                    Fusobacterium
## Leptotrichia                                                      Leptotrichia
## Gracilibacteria_(GN02)_[G.1]                      Gracilibacteria_(GN02)_[G.1]
## Lautropia                                                            Lautropia
## Ottowia                                                                Ottowia
## Eikenella                                                            Eikenella
## Kingella                                                              Kingella
## Neisseria                                                            Neisseria
## Family.Neisseriaceae.                                    Family.Neisseriaceae.
## Campylobacter                                                    Campylobacter
## Cardiobacterium                                                Cardiobacterium
## Aggregatibacter                                                Aggregatibacter
## Haemophilus                                                        Haemophilus
## Family.Pasteurellaceae.                                  Family.Pasteurellaceae.
## Saccharibacteria_(TM7)_[G.1]                      Saccharibacteria_(TM7)_[G.1]
## Saccharibacteria_(TM7)_[G.3]                      Saccharibacteria_(TM7)_[G.3]
## Saccharibacteria_(TM7)_[G.6]                      Saccharibacteria_(TM7)_[G.6]
## Family.Saccharibacteria_(TM7)_[F-1]. Family.Saccharibacteria_(TM7)_[F-1].
## Saccharibacteria_(TM7)_[G.5]                      Saccharibacteria_(TM7)_[G.5]
## Treponema                                                            Treponema
## Fretibacterium                                                  Fretibacterium
## Kingdom.Bacteria.                                            Kingdom.Bacteria.
## Others                                                                  Others
##                                                   Method
## Absconditabacteria_(SR1)_[G.1]       Kruskal-Wallis (kru)
## Actinomyces                          Kruskal-Wallis (kru)
## Peptidiphaga                         Kruskal-Wallis (kru)
## Schaalia                             Kruskal-Wallis (kru)
## Family.Actinomycetaceae.             Kruskal-Wallis (kru)
## Rothia                               Kruskal-Wallis (kru)
## Alloscardovia                        Kruskal-Wallis (kru)
## Bifidobacterium                      Kruskal-Wallis (kru)
## Parascardovia                        Kruskal-Wallis (kru)
## Scardovia                            Kruskal-Wallis (kru)
## Corynebacterium                      Kruskal-Wallis (kru)
## Olsenella                            Kruskal-Wallis (kru)
## Atopobium                            Kruskal-Wallis (kru)
```

```
## Slackia                                  Kruskal-Wallis (kru)
## Cryptobacterium                          Kruskal-Wallis (kru)
## Bacteroidetes_[G.3]                      Kruskal-Wallis (kru)
## Bacteroidetes_[G.5]                      Kruskal-Wallis (kru)
## Family.Bacteroidetes_[F-1].              Kruskal-Wallis (kru)
## Bacteroidaceae_[G.1]                     Kruskal-Wallis (kru)
## Bacteroidales_[G.2]                      Kruskal-Wallis (kru)
## Porphyromonas                            Kruskal-Wallis (kru)
## Tannerella                               Kruskal-Wallis (kru)
## Alloprevotella                           Kruskal-Wallis (kru)
## Prevotella                               Kruskal-Wallis (kru)
## Bergeyella                               Kruskal-Wallis (kru)
## Capnocytophaga                           Kruskal-Wallis (kru)
## Gemella                                  Kruskal-Wallis (kru)
## Abiotrophia                              Kruskal-Wallis (kru)
## Granulicatella                           Kruskal-Wallis (kru)
## Lactobacillus                            Kruskal-Wallis (kru)
## Streptococcus                            Kruskal-Wallis (kru)
## Order.Lactobacillales.                   Kruskal-Wallis (kru)
## Class.Bacilli.                           Kruskal-Wallis (kru)
## Clostridiales_[F.1][G-1]                 Kruskal-Wallis (kru)
## Butyrivibrio                             Kruskal-Wallis (kru)
## Catonella                                Kruskal-Wallis (kru)
## Johnsonella                              Kruskal-Wallis (kru)
## Lachnoanaerobaculum                      Kruskal-Wallis (kru)
## Lachnospiraceae_[G.2]                    Kruskal-Wallis (kru)
## Lachnospiraceae_[G.3]                    Kruskal-Wallis (kru)
## Lachnospiraceae_[G.7]                    Kruskal-Wallis (kru)
## Lachnospiraceae_[G.8]                    Kruskal-Wallis (kru)
## Oribacterium                             Kruskal-Wallis (kru)
## Shuttleworthia                           Kruskal-Wallis (kru)
## Stomatobaculum                           Kruskal-Wallis (kru)
## Family.Lachnospiraceae_[XIV].            Kruskal-Wallis (kru)
## Peptococcus                              Kruskal-Wallis (kru)
## Parvimonas                               Kruskal-Wallis (kru)
## Filifactor                               Kruskal-Wallis (kru)
## Mogibacterium                            Kruskal-Wallis (kru)
## Peptostreptococcaceae_[XI][G.1]          Kruskal-Wallis (kru)
## Peptostreptococcaceae_[XI][G.4]          Kruskal-Wallis (kru)
## Peptostreptococcaceae_[XI][G.5]          Kruskal-Wallis (kru)
## Peptostreptococcaceae_[XI][G.6]          Kruskal-Wallis (kru)
## Peptostreptococcaceae_[XI][G.7]          Kruskal-Wallis (kru)
## Peptostreptococcaceae_[XI][G.9]          Kruskal-Wallis (kru)
## Peptostreptococcus                       Kruskal-Wallis (kru)
## Ruminococcaceae_[G.1]                    Kruskal-Wallis (kru)
## Ruminococcaceae_[G.2]                    Kruskal-Wallis (kru)
## Bulleidia                                Kruskal-Wallis (kru)
## Eggerthia                                Kruskal-Wallis (kru)
## Solobacterium                            Kruskal-Wallis (kru)
## Mycoplasma                               Kruskal-Wallis (kru)
## Mitsuokella                              Kruskal-Wallis (kru)
## Selenomonas                              Kruskal-Wallis (kru)
## Family.Selenomonadaceae.                 Kruskal-Wallis (kru)
## Class.Negativicutes.                     Kruskal-Wallis (kru)
```

```
## Anaeroglobus                            Kruskal-Wallis (kru)
## Dialister                               Kruskal-Wallis (kru)
## Megasphaera                             Kruskal-Wallis (kru)
## Family.Veillonellaceae.                 Kruskal-Wallis (kru)
## Veillonella                             Kruskal-Wallis (kru)
## Veillonellaceae_[G.1]                   Kruskal-Wallis (kru)
## Phylum.Firmicutes.                      Kruskal-Wallis (kru)
## Fusobacterium                           Kruskal-Wallis (kru)
## Leptotrichia                            Kruskal-Wallis (kru)
## Gracilibacteria_(GN02)_[G.1]            Kruskal-Wallis (kru)
## Lautropia                               Kruskal-Wallis (kru)
## Ottowia                                 Kruskal-Wallis (kru)
## Eikenella                               Kruskal-Wallis (kru)
## Kingella                                Kruskal-Wallis (kru)
## Neisseria                               Kruskal-Wallis (kru)
## Family.Neisseriaceae.                   Kruskal-Wallis (kru)
## Campylobacter                           Kruskal-Wallis (kru)
## Cardiobacterium                         Kruskal-Wallis (kru)
## Aggregatibacter                         Kruskal-Wallis (kru)
## Haemophilus                             Kruskal-Wallis (kru)
## Family.Pasteurellaceae.                 Kruskal-Wallis (kru)
## Saccharibacteria_(TM7)_[G.1]            Kruskal-Wallis (kru)
## Saccharibacteria_(TM7)_[G.3]            Kruskal-Wallis (kru)
## Saccharibacteria_(TM7)_[G.6]            Kruskal-Wallis (kru)
## Family.Saccharibacteria_(TM7)_[F-1]. Kruskal-Wallis (kru)
## Saccharibacteria_(TM7)_[G.5]            Kruskal-Wallis (kru)
## Treponema                               Kruskal-Wallis (kru)
## Fretibacterium                          Kruskal-Wallis (kru)
## Kingdom.Bacteria.                       Kruskal-Wallis (kru)
## Others                                  Kruskal-Wallis (kru)
```

```
write.table(final, file="hr_cat_kru.txt", sep="\t", dec=",", row.names=F)
write.table(final[final$pval<0.05,], file="hr_cat_kru_sig.txt", sep="\t", dec=",", row.names=F)
#################pp_cat
table(Phe$pp_cat, useNA="always")
```

```
##
## High  Low <NA>
##  605  139    2
```

```
#Remove NA
Phe2<-Phe[complete.cases(Phe$pp_cat),]
#Also subset columns
Microbio2<-dplyr::select(Microbio, one_of(Phe2$IDX))
final <- DA.ds2(Microbio2, predictor = Phe2$pp_cat)
final
```

```
##         baseMean log2FoldChange      lfcSE         stat         pval ordering
## 1    67.1503320  -0.0924379568 0.31707380 -0.291534517 7.706425e-01 High>Low
## 2   323.7803206  -0.1460193028 0.10665354 -1.369099477 1.709682e-01 High>Low
## 3    22.0416883  -0.0256325132 0.15234559 -0.168252410 8.663847e-01 High>Low
## 4   703.5570032  -0.1720632286 0.08762736 -1.963578754 4.957896e-02 High>Low
## 5     5.4318947  -0.1229925308 0.22004073 -0.558953480 5.761935e-01 High>Low
```

```
## 6  1938.6926786  0.0381104654 0.10290634  0.370341264 7.111282e-01 Low>High
## 7    11.5794832 -0.7690433064 0.26629001 -2.887991603 3.877102e-03 High>Low
## 8    10.7550475 -0.8728680117 0.29479154 -2.960966928 3.066749e-03 High>Low
## 9     2.9264171 -0.6395838648 0.41094537 -1.556371979 1.196197e-01 High>Low
## 10   13.3655142 -0.1824436109 0.26143070 -0.697866060 4.852610e-01 High>Low
## 11   93.8520152 -0.0998875851 0.11254095 -0.887566600 3.747739e-01 High>Low
## 12    1.5705319  0.0379124926 0.24894083  0.152295196 8.789541e-01 Low>High
## 13  120.2270723 -0.2297813002 0.10283810 -2.234398583 2.545686e-02 High>Low
## 14    1.3929535 -0.4116783135 0.24331157 -1.691979982 9.064979e-02 High>Low
## 15    4.2513998 -0.4362798726 0.25709289 -1.696973681 8.970164e-02 High>Low
## 16    6.6074797  0.2353225655 0.26984808  0.872055747 3.831780e-01 Low>High
## 17    4.6913133 -0.0227698267 0.29461486 -0.077286755 9.383954e-01 High>Low
## 18    0.8714947 -0.1552113402 0.42605149 -0.364301836 7.156326e-01 High>Low
## 19    0.7948120 -0.6737295668 0.40501600 -1.663464069 9.621958e-02 High>Low
## 20   12.0719990  0.2622557982 0.15388392  1.704244297 8.833546e-02 Low>High
## 21  682.2933394  0.1380220680 0.14095055  0.979223318 3.274696e-01 Low>High
## 22   22.0983847  0.1115905562 0.10425710  1.070340117 2.844662e-01 Low>High
## 23  362.5219516 -0.0684841292 0.11218910 -0.610434799 5.415738e-01 High>Low
## 24 3807.2361865 -0.0387864358 0.07121612 -0.544630035 5.860080e-01 High>Low
## 25   72.7155861 -0.0193919744 0.11002907 -0.176244100 8.601022e-01 High>Low
## 26  225.4613668  0.0042282083 0.11762689  0.035945932 9.713255e-01 Low>High
## 27  309.8006772  0.0355509076 0.09784120  0.363353149 7.163411e-01 Low>High
## 28   30.0741451 -0.1765697315 0.21218594 -0.832146238 4.053264e-01 High>Low
## 29  306.3404328  0.0493471058 0.08568912  0.575885332 5.646927e-01 Low>High
## 30   41.5440135 -1.6274731819 0.35387970 -4.598944688 4.246365e-06 High>Low
## 31 3797.5698295 -0.1293426016 0.08025605 -1.611624391 1.070437e-01 High>Low
## 32    0.2871119 -0.1287831053 0.28877915 -0.445957069 6.556283e-01 High>Low
## 33    0.5755295 -0.1433895078 0.23262779 -0.616390267 5.376370e-01 High>Low
## 34    1.0579178  0.0465364831 0.35122111  0.132499107 8.945895e-01 Low>High
## 35    6.9656228  0.0219873226 0.22415526  0.098089702 9.218611e-01 Low>High
## 36   36.9095724  0.0397695231 0.12639782  0.314637740 7.530367e-01 Low>High
## 37    0.9421596 -0.0889055211 0.30652460 -0.290043674 7.717828e-01 High>Low
## 38   87.8820004 -0.1276390760 0.08844589 -1.443131794 1.489833e-01 High>Low
## 39   28.0534664 -0.2912777846 0.23192607 -1.255907881 2.091494e-01 High>Low
## 40    2.8995123 -0.4786013864 0.24260772 -1.972737649 4.852546e-02 High>Low
## 41    1.4732956 -0.4921147428 0.34837131 -1.412615596 1.577688e-01 High>Low
## 42    1.4367939  0.4726978024 0.36240839  1.304323574 1.921233e-01 Low>High
## 43  147.1321858 -0.0172642078 0.09798896 -0.176185236 8.601484e-01 High>Low
## 44    2.8108966 -0.0968411700 0.27286906 -0.354899777 7.226647e-01 High>Low
## 45  112.9182555 -0.0199344212 0.11016368 -0.180952757 8.564047e-01 High>Low
## 46    1.6095271  0.0936262058 0.31064498  0.301392947 7.631149e-01 Low>High
## 47    3.7157473 -0.0411590764 0.27335879 -0.150567963 8.803165e-01 High>Low
## 48   40.9495178  0.0506328406 0.13582733  0.372773588 7.093170e-01 Low>High
## 49    6.8621336  0.3623777911 0.26356630  1.374901840 1.691619e-01 Low>High
## 50   41.1723089 -0.0954173891 0.09389751 -1.016186537 3.095406e-01 High>Low
## 51   67.7192574 -0.0005669232 0.16707493 -0.003393227 9.972926e-01 High>Low
## 52    0.4191905  0.4583011433 0.35637973  1.285991033 1.984462e-01 Low>High
## 53    1.6690812 -0.0893251977 0.38150902 -0.234136531 8.148790e-01 High>Low
## 54    1.0175853  0.6272055433 0.36800025  1.704361733 8.831353e-02 Low>High
## 55    4.7195993 -0.3053964326 0.26245884 -1.163597420 2.445872e-01 High>Low
## 56    8.7363992  0.2481187772 0.15435818  1.607422291 1.079618e-01 Low>High
## 57   42.9864069  0.0771093573 0.19529740  0.394830441 6.929680e-01 Low>High
## 58   25.1836759 -0.0811120845 0.19898958 -0.407619768 6.835528e-01 High>Low
## 59   41.5234417 -0.2921658333 0.16560081 -1.764277768 7.768523e-02 High>Low
```

```
## 60    3.8709078   0.2979475844 0.29025351  1.026508134 3.046521e-01 Low>High
## 61    1.5130043  -0.0790735367 0.38899075 -0.203278705 8.389172e-01 High>Low
## 62   74.1630338   0.0157207009 0.11050580  0.142261315 8.868736e-01 Low>High
## 63    4.5000508   0.4143391643 0.23165690  1.788589808 7.368090e-02 Low>High
## 64   26.7544753  -0.2738415926 0.20955680 -1.306765458 1.912924e-01 High>Low
## 65  222.3185156  -0.0247375637 0.09422701 -0.262531547 7.929117e-01 High>Low
## 66    5.0504886  -0.0708907666 0.24685419 -0.287176684 7.739770e-01 High>Low
## 67    0.7322999   0.4086304862 0.21768326  1.877179159 6.049354e-02 Low>High
## 68    8.7028189  -0.3999278202 0.22622490 -1.767832863 7.708885e-02 High>Low
## 69   36.4852787  -0.0330654802 0.11582689 -0.285473261 7.752816e-01 High>Low
## 70  407.7957370  -0.2363322923 0.12171583 -1.941672679 5.217674e-02 High>Low
## 71    0.5239301  -0.1882841343 0.26730318 -0.704384191 4.811935e-01 High>Low
## 72 5061.2445718   0.0036795347 0.06850873  0.053708993 9.571670e-01 Low>High
## 73    6.1469526   0.3045674935 0.18945743  1.607577487 1.079277e-01 Low>High
## 74    0.8558641  -1.0366873927 0.32032200 -3.236391529 1.210512e-03 High>Low
## 75  929.2571372   0.0064500682 0.09117701  0.070742268 9.436029e-01 Low>High
## 76  684.6054135  -0.0224619829 0.09400370 -0.238947861 8.111460e-01 High>Low
## 77    2.2878325  -0.1449619560 0.29914911 -0.484580940 6.279736e-01 High>Low
## 78   68.4994208  -0.0157458575 0.17469465 -0.090133598 9.281811e-01 High>Low
## 79    2.2349835  -0.1380400605 0.39048817 -0.353506380 7.237088e-01 High>Low
## 80    8.7703431   0.0866175998 0.14616101  0.592617686 5.534370e-01 Low>High
## 81  100.0995471  -0.1168430973 0.12591243 -0.927971110 3.534226e-01 High>Low
## 82 1333.1334336   0.0697150242 0.14321189  0.486796335 6.264027e-01 Low>High
## 83    6.1192335   0.0083894744 0.33518253  0.025029569 9.800314e-01 Low>High
## 84  257.7162863  -0.1049874463 0.08065927 -1.301616577 1.930475e-01 High>Low
## 85   14.8139218   0.0220986589 0.12558370  0.175967579 8.603194e-01 Low>High
## 86  276.1135518   0.0286385651 0.16619766  0.172316300 8.631889e-01 Low>High
## 87 2911.7582507   0.0013593915 0.11083710  0.012264770 9.902144e-01 Low>High
## 88    2.3609083  -0.9056632771 0.45451903 -1.992575027 4.630800e-02 High>Low
## 89   36.1574542   0.1404015897 0.18472864  0.760042346 4.472293e-01 Low>High
## 90    7.1401803  -0.1928102694 0.25759975 -0.748487783 4.541660e-01 High>Low
## 91    7.2298310  -0.0876662915 0.32850739 -0.266862468 7.895751e-01 High>Low
## 92    1.1040897  -0.2363243828 0.40979326 -0.576691721 5.641477e-01 High>Low
## 93    0.9151491  -0.2484034403 0.38920028 -0.638240648 5.233170e-01 High>Low
## 94   63.3764075   0.5095556158 0.17441385  2.921531856 3.483147e-03 Low>High
## 95    8.2967790   0.2857554893 0.21104799  1.353983476 1.757416e-01 Low>High
## 96    9.4046214  -0.6249016864 0.26897117 -2.323303636 2.016285e-02 High>Low
## 97   32.3741773  -0.0637972836 0.17718711 -0.360056004 7.188053e-01 High>Low
##        pval.adj                           Feature                     Method
## 1  0.9860816406      Absconditabacteria_(SR1)_[G.1] DESeq2 man. geoMeans (ds2)
## 2  0.5833115313                       Actinomyces DESeq2 man. geoMeans (ds2)
## 3  0.9860816406                      Peptidiphaga DESeq2 man. geoMeans (ds2)
## 4  0.4553170023                          Schaalia DESeq2 man. geoMeans (ds2)
## 5  0.9860816406          Family.Actinomycetaceae. DESeq2 man. geoMeans (ds2)
## 6  0.9860816406                           Rothia DESeq2 man. geoMeans (ds2)
## 7  0.0752157833                    Alloscardovia DESeq2 man. geoMeans (ds2)
## 8  0.0752157833                  Bifidobacterium DESeq2 man. geoMeans (ds2)
## 9  0.4834628102                    Parascardovia DESeq2 man. geoMeans (ds2)
## 10 0.9860816406                        Scardovia DESeq2 man. geoMeans (ds2)
## 11 0.8849586364                  Corynebacterium DESeq2 man. geoMeans (ds2)
## 12 0.9860816406                        Olsenella DESeq2 man. geoMeans (ds2)
## 13 0.3527593301                        Atopobium DESeq2 man. geoMeans (ds2)
## 14 0.4553170023                          Slackia DESeq2 man. geoMeans (ds2)
## 15 0.4553170023                  Cryptobacterium DESeq2 man. geoMeans (ds2)
```

```
## 16 0.8849586364                  Bacteroidetes_[G.3] DESeq2 man. geoMeans (ds2)
## 17 0.9948856454                  Bacteroidetes_[G.5] DESeq2 man. geoMeans (ds2)
## 18 0.9860816406         Family.Bacteroidetes_[F-1]. DESeq2 man. geoMeans (ds2)
## 19 0.4553170023                 Bacteroidaceae_[G.1] DESeq2 man. geoMeans (ds2)
## 20 0.4553170023                  Bacteroidales_[G.2] DESeq2 man. geoMeans (ds2)
## 21 0.8144757952                        Porphyromonas DESeq2 man. geoMeans (ds2)
## 22 0.7664784883                           Tannerella DESeq2 man. geoMeans (ds2)
## 23 0.9860816406                       Alloprevotella DESeq2 man. geoMeans (ds2)
## 24 0.9860816406                           Prevotella DESeq2 man. geoMeans (ds2)
## 25 0.9860816406                           Bergeyella DESeq2 man. geoMeans (ds2)
## 26 0.9972926014                       Capnocytophaga DESeq2 man. geoMeans (ds2)
## 27 0.9860816406                              Gemella DESeq2 man. geoMeans (ds2)
## 28 0.9143409613                          Abiotrophia DESeq2 man. geoMeans (ds2)
## 29 0.9860816406                       Granulicatella DESeq2 man. geoMeans (ds2)
## 30 0.0004118974                        Lactobacillus DESeq2 man. geoMeans (ds2)
## 31 0.4553170023                        Streptococcus DESeq2 man. geoMeans (ds2)
## 32 0.9860816406              Order.Lactobacillales. DESeq2 man. geoMeans (ds2)
## 33 0.9860816406                       Class.Bacilli. DESeq2 man. geoMeans (ds2)
## 34 0.9860816406           Clostridiales_[F.1][G-1] DESeq2 man. geoMeans (ds2)
## 35 0.9948856454                         Butyrivibrio DESeq2 man. geoMeans (ds2)
## 36 0.9860816406                            Catonella DESeq2 man. geoMeans (ds2)
## 37 0.9860816406                          Johnsonella DESeq2 man. geoMeans (ds2)
## 38 0.5780553926                  Lachnoanaerobaculum DESeq2 man. geoMeans (ds2)
## 39 0.5966908527                Lachnospiraceae_[G.2] DESeq2 man. geoMeans (ds2)
## 40 0.4553170023                Lachnospiraceae_[G.3] DESeq2 man. geoMeans (ds2)
## 41 0.5833115313                Lachnospiraceae_[G.7] DESeq2 man. geoMeans (ds2)
## 42 0.5833115313                Lachnospiraceae_[G.8] DESeq2 man. geoMeans (ds2)
## 43 0.9860816406                         Oribacterium DESeq2 man. geoMeans (ds2)
## 44 0.9860816406                       Shuttleworthia DESeq2 man. geoMeans (ds2)
## 45 0.9860816406                       Stomatobaculum DESeq2 man. geoMeans (ds2)
## 46 0.9860816406      Family.Lachnospiraceae_[XIV]. DESeq2 man. geoMeans (ds2)
## 47 0.9860816406                          Peptococcus DESeq2 man. geoMeans (ds2)
## 48 0.9860816406                           Parvimonas DESeq2 man. geoMeans (ds2)
## 49 0.5833115313                           Filifactor DESeq2 man. geoMeans (ds2)
## 50 0.7901430241                         Mogibacterium DESeq2 man. geoMeans (ds2)
## 51 0.9972926014     Peptostreptococcaceae_[XI][G.1] DESeq2 man. geoMeans (ds2)
## 52 0.5833115313     Peptostreptococcaceae_[XI][G.4] DESeq2 man. geoMeans (ds2)
## 53 0.9860816406     Peptostreptococcaceae_[XI][G.5] DESeq2 man. geoMeans (ds2)
## 54 0.4553170023     Peptostreptococcaceae_[XI][G.6] DESeq2 man. geoMeans (ds2)
## 55 0.6778559477     Peptostreptococcaceae_[XI][G.7] DESeq2 man. geoMeans (ds2)
## 56 0.4553170023     Peptostreptococcaceae_[XI][G.9] DESeq2 man. geoMeans (ds2)
## 57 0.9860816406                  Peptostreptococcus DESeq2 man. geoMeans (ds2)
## 58 0.9860816406               Ruminococcaceae_[G.1] DESeq2 man. geoMeans (ds2)
## 59 0.4553170023               Ruminococcaceae_[G.2] DESeq2 man. geoMeans (ds2)
## 60 0.7901430241                            Bulleidia DESeq2 man. geoMeans (ds2)
## 61 0.9860816406                            Eggerthia DESeq2 man. geoMeans (ds2)
## 62 0.9860816406                         Solobacterium DESeq2 man. geoMeans (ds2)
## 63 0.4553170023                           Mycoplasma DESeq2 man. geoMeans (ds2)
## 64 0.5833115313                          Mitsuokella DESeq2 man. geoMeans (ds2)
## 65 0.9860816406                          Selenomonas DESeq2 man. geoMeans (ds2)
## 66 0.9860816406           Family.Selenomonadaceae. DESeq2 man. geoMeans (ds2)
## 67 0.4553170023               Class.Negativicutes. DESeq2 man. geoMeans (ds2)
## 68 0.4553170023                         Anaeroglobus DESeq2 man. geoMeans (ds2)
## 69 0.9860816406                            Dialister DESeq2 man. geoMeans (ds2)
```

```
## 70 0.4553170023                       Megasphaera DESeq2 man. geoMeans (ds2)
## 71 0.9860816406              Family.Veillonellaceae. DESeq2 man. geoMeans (ds2)
## 72 0.9972926014                       Veillonella DESeq2 man. geoMeans (ds2)
## 73 0.4553170023               Veillonellaceae_[G.1] DESeq2 man. geoMeans (ds2)
## 74 0.0587098432                  Phylum.Firmicutes. DESeq2 man. geoMeans (ds2)
## 75 0.9948856454                     Fusobacterium DESeq2 man. geoMeans (ds2)
## 76 0.9860816406                      Leptotrichia DESeq2 man. geoMeans (ds2)
## 77 0.9860816406        Gracilibacteria_(GN02)_[G.1] DESeq2 man. geoMeans (ds2)
## 78 0.9948856454                         Lautropia DESeq2 man. geoMeans (ds2)
## 79 0.9860816406                           Ottowia DESeq2 man. geoMeans (ds2)
## 80 0.9860816406                         Eikenella DESeq2 man. geoMeans (ds2)
## 81 0.8570496983                          Kingella DESeq2 man. geoMeans (ds2)
## 82 0.9860816406                         Neisseria DESeq2 man. geoMeans (ds2)
## 83 0.9972926014               Family.Neisseriaceae. DESeq2 man. geoMeans (ds2)
## 84 0.5833115313                     Campylobacter DESeq2 man. geoMeans (ds2)
## 85 0.9860816406                    Cardiobacterium DESeq2 man. geoMeans (ds2)
## 86 0.9860816406                   Aggregatibacter DESeq2 man. geoMeans (ds2)
## 87 0.9972926014                       Haemophilus DESeq2 man. geoMeans (ds2)
## 88 0.4553170023             Family.Pasteurellaceae. DESeq2 man. geoMeans (ds2)
## 89 0.9789800256        Saccharibacteria_(TM7)_[G.1] DESeq2 man. geoMeans (ds2)
## 90 0.9789800256        Saccharibacteria_(TM7)_[G.3] DESeq2 man. geoMeans (ds2)
## 91 0.9860816406        Saccharibacteria_(TM7)_[G.6] DESeq2 man. geoMeans (ds2)
## 92 0.9860816406 Family.Saccharibacteria_(TM7)_[F-1]. DESeq2 man. geoMeans (ds2)
## 93 0.9860816406        Saccharibacteria_(TM7)_[G.5] DESeq2 man. geoMeans (ds2)
## 94 0.0752157833                         Treponema DESeq2 man. geoMeans (ds2)
## 95 0.5833115313                    Fretibacterium DESeq2 man. geoMeans (ds2)
## 96 0.3259660207                  Kingdom.Bacteria. DESeq2 man. geoMeans (ds2)
## 97 0.9860816406                            Others DESeq2 man. geoMeans (ds2)
```

```
write.table(final, file="pp_cat_ds2.txt", sep="\t", dec=",", row.names=F)
write.table(final[final$pval<0.05,], file="pp_cat_ds2_sig.txt", sep="\t", dec=",", row.names=F)
final <- DA.kru(Microbio2, predictor = Phe2$pp_cat)
final
```

```
##                                        pval  pval.adj
## Absconditabacteria_(SR1)_[G.1]       0.655772515 0.9174503
## Actinomyces                          0.260968386 0.8501101
## Peptidiphaga                         0.583849660 0.9174503
## Schaalia                             0.057540259 0.7967817
## Family.Actinomycetaceae.             0.552752499 0.9174503
## Rothia                               0.513913803 0.9174503
## Alloscardovia                        0.045885136 0.7967817
## Bifidobacterium                      0.035463796 0.7967817
## Parascardovia                        0.818749517 0.9174503
## Scardovia                            0.751507875 0.9174503
## Corynebacterium                      0.831562080 0.9174503
## Olsenella                            0.521207885 0.9174503
## Atopobium                            0.153087611 0.7967817
## Slackia                              0.091978112 0.7967817
## Cryptobacterium                      0.679019999 0.9174503
## Bacteroidetes_[G.3]                  0.032002537 0.7967817
## Bacteroidetes_[G.5]                  0.797595785 0.9174503
## Family.Bacteroidetes_[F-1].          0.630748396 0.9174503
## Bacteroidaceae_[G.1]                 0.927218184 0.9776105
```

```
## Bacteroidales_[G.2]                  0.123389969 0.7967817
## Porphyromonas                        0.087243785 0.7967817
## Tannerella                           0.151759710 0.7967817
## Alloprevotella                       0.707122850 0.9174503
## Prevotella                           0.482548099 0.9174503
## Bergeyella                           0.423948653 0.9174503
## Capnocytophaga                       0.955326410 0.9832723
## Gemella                              0.911313395 0.9714000
## Abiotrophia                          0.744471144 0.9174503
## Granulicatella                       0.851242564 0.9174503
## Lactobacillus                        0.380919042 0.9174503
## Streptococcus                        0.389918955 0.9174503
## Order.Lactobacillales.               0.811714064 0.9174503
## Class.Bacilli.                       0.546899580 0.9174503
## Clostridiales_[F.1][G-1]             0.704890419 0.9174503
## Butyrivibrio                         0.312860656 0.9174503
## Catonella                            0.213484692 0.8501101
## Johnsonella                          0.514164811 0.9174503
## Lachnoanaerobaculum                  0.258284587 0.8501101
## Lachnospiraceae_[G.2]                0.262920642 0.8501101
## Lachnospiraceae_[G.3]                0.166061383 0.7967817
## Lachnospiraceae_[G.7]                0.161144586 0.7967817
## Lachnospiraceae_[G.8]                0.694605109 0.9174503
## Oribacterium                         0.980796142 0.9910128
## Shuttleworthia                       0.676143944 0.9174503
## Stomatobaculum                       0.542529809 0.9174503
## Family.Lachnospiraceae_[XIV].        0.831660819 0.9174503
## Peptococcus                          0.219512603 0.8501101
## Parvimonas                           0.663855571 0.9174503
## Filifactor                           0.204282480 0.8501101
## Mogibacterium                        0.621848400 0.9174503
## Peptostreptococcaceae_[XI][G.1]      0.452224192 0.9174503
## Peptostreptococcaceae_[XI][G.4]      0.180713374 0.7967817
## Peptostreptococcaceae_[XI][G.5]      0.845250116 0.9174503
## Peptostreptococcaceae_[XI][G.6]      0.160280359 0.7967817
## Peptostreptococcaceae_[XI][G.7]      0.560596268 0.9174503
## Peptostreptococcaceae_[XI][G.9]      0.033996003 0.7967817
## Peptostreptococcus                   0.286265203 0.8957331
## Ruminococcaceae_[G.1]                0.575310887 0.9174503
## Ruminococcaceae_[G.2]                0.171135032 0.7967817
## Bulleidia                            0.007914684 0.7677244
## Eggerthia                            0.636181029 0.9174503
## Solobacterium                        0.992143217 0.9921432
## Mycoplasma                           0.168327381 0.7967817
## Mitsuokella                          0.779708761 0.9174503
## Selenomonas                          0.962998647 0.9832723
## Family.Selenomonadaceae.             0.699722783 0.9174503
## Class.Negativicutes.                 0.680210404 0.9174503
## Anaeroglobus                         0.765042849 0.9174503
## Dialister                            0.625717905 0.9174503
## Megasphaera                          0.249813704 0.8501101
## Family.Veillonellaceae.              0.611511624 0.9174503
## Veillonella                          0.766507855 0.9174503
## Veillonellaceae_[G.1]                0.059110023 0.7967817
```

```
## Phylum.Firmicutes.                    0.101101247 0.7967817
## Fusobacterium                         0.326919767 0.9174503
## Leptotrichia                          0.761834266 0.9174503
## Gracilibacteria_(GN02)_[G.1]          0.463717221 0.9174503
## Lautropia                             0.747708884 0.9174503
## Ottowia                               0.837335045 0.9174503
## Eikenella                             0.171416271 0.7967817
## Kingella                              0.951665994 0.9832723
## Neisseria                             0.521704400 0.9174503
## Family.Neisseriaceae.                 0.369710803 0.9174503
## Campylobacter                         0.177810948 0.7967817
## Cardiobacterium                       0.700875419 0.9174503
## Aggregatibacter                       0.768819551 0.9174503
## Haemophilus                           0.840620076 0.9174503
## Family.Pasteurellaceae.               0.800812128 0.9174503
## Saccharibacteria_(TM7)_[G.1]          0.830238643 0.9174503
## Saccharibacteria_(TM7)_[G.3]          0.582260554 0.9174503
## Saccharibacteria_(TM7)_[G.6]          0.531732199 0.9174503
## Family.Saccharibacteria_(TM7)_[F-1]. 0.705136289 0.9174503
## Saccharibacteria_(TM7)_[G.5]          0.743990861 0.9174503
## Treponema                             0.364237662 0.9174503
## Fretibacterium                        0.066458803 0.7967817
## Kingdom.Bacteria.                     0.233080743 0.8501101
## Others                                0.674528702 0.9174503
##                                                                Feature
## Absconditabacteria_(SR1)_[G.1]             Absconditabacteria_(SR1)_[G.1]
## Actinomyces                                                Actinomyces
## Peptidiphaga                                              Peptidiphaga
## Schaalia                                                      Schaalia
## Family.Actinomycetaceae.                        Family.Actinomycetaceae.
## Rothia                                                          Rothia
## Alloscardovia                                            Alloscardovia
## Bifidobacterium                                        Bifidobacterium
## Parascardovia                                            Parascardovia
## Scardovia                                                    Scardovia
## Corynebacterium                                        Corynebacterium
## Olsenella                                                    Olsenella
## Atopobium                                                    Atopobium
## Slackia                                                        Slackia
## Cryptobacterium                                        Cryptobacterium
## Bacteroidetes_[G.3]                                Bacteroidetes_[G.3]
## Bacteroidetes_[G.5]                                Bacteroidetes_[G.5]
## Family.Bacteroidetes_[F-1].                  Family.Bacteroidetes_[F-1].
## Bacteroidaceae_[G.1]                              Bacteroidaceae_[G.1]
## Bacteroidales_[G.2]                                Bacteroidales_[G.2]
## Porphyromonas                                            Porphyromonas
## Tannerella                                                  Tannerella
## Alloprevotella                                          Alloprevotella
## Prevotella                                                  Prevotella
## Bergeyella                                                  Bergeyella
## Capnocytophaga                                          Capnocytophaga
## Gemella                                                        Gemella
## Abiotrophia                                                Abiotrophia
## Granulicatella                                          Granulicatella
```

```
## Lactobacillus                                                         Lactobacillus
## Streptococcus                                                         Streptococcus
## Order.Lactobacillales.                                       Order.Lactobacillales.
## Class.Bacilli.                                                       Class.Bacilli.
## Clostridiales_[F.1][G-1]                                   Clostridiales_[F.1][G-1]
## Butyrivibrio                                                           Butyrivibrio
## Catonella                                                                 Catonella
## Johnsonella                                                             Johnsonella
## Lachnoanaerobaculum                                             Lachnoanaerobaculum
## Lachnospiraceae_[G.2]                                         Lachnospiraceae_[G.2]
## Lachnospiraceae_[G.3]                                         Lachnospiraceae_[G.3]
## Lachnospiraceae_[G.7]                                         Lachnospiraceae_[G.7]
## Lachnospiraceae_[G.8]                                         Lachnospiraceae_[G.8]
## Oribacterium                                                           Oribacterium
## Shuttleworthia                                                       Shuttleworthia
## Stomatobaculum                                                       Stomatobaculum
## Family.Lachnospiraceae_[XIV].                         Family.Lachnospiraceae_[XIV].
## Peptococcus                                                             Peptococcus
## Parvimonas                                                               Parvimonas
## Filifactor                                                               Filifactor
## Mogibacterium                                                         Mogibacterium
## Peptostreptococcaceae_[XI][G.1]                     Peptostreptococcaceae_[XI][G.1]
## Peptostreptococcaceae_[XI][G.4]                     Peptostreptococcaceae_[XI][G.4]
## Peptostreptococcaceae_[XI][G.5]                     Peptostreptococcaceae_[XI][G.5]
## Peptostreptococcaceae_[XI][G.6]                     Peptostreptococcaceae_[XI][G.6]
## Peptostreptococcaceae_[XI][G.7]                     Peptostreptococcaceae_[XI][G.7]
## Peptostreptococcaceae_[XI][G.9]                     Peptostreptococcaceae_[XI][G.9]
## Peptostreptococcus                                               Peptostreptococcus
## Ruminococcaceae_[G.1]                                         Ruminococcaceae_[G.1]
## Ruminococcaceae_[G.2]                                         Ruminococcaceae_[G.2]
## Bulleidia                                                                 Bulleidia
## Eggerthia                                                                 Eggerthia
## Solobacterium                                                         Solobacterium
## Mycoplasma                                                               Mycoplasma
## Mitsuokella                                                             Mitsuokella
## Selenomonas                                                             Selenomonas
## Family.Selenomonadaceae.                                   Family.Selenomonadaceae.
## Class.Negativicutes.                                           Class.Negativicutes.
## Anaeroglobus                                                           Anaeroglobus
## Dialister                                                                 Dialister
## Megasphaera                                                             Megasphaera
## Family.Veillonellaceae.                                     Family.Veillonellaceae.
## Veillonella                                                             Veillonella
## Veillonellaceae_[G.1]                                         Veillonellaceae_[G.1]
## Phylum.Firmicutes.                                               Phylum.Firmicutes.
## Fusobacterium                                                         Fusobacterium
## Leptotrichia                                                           Leptotrichia
## Gracilibacteria_(GN02)_[G.1]                           Gracilibacteria_(GN02)_[G.1]
## Lautropia                                                                 Lautropia
## Ottowia                                                                     Ottowia
## Eikenella                                                                 Eikenella
## Kingella                                                                   Kingella
## Neisseria                                                                 Neisseria
## Family.Neisseriaceae.                                         Family.Neisseriaceae.
```

```
## Campylobacter                                                    Campylobacter
## Cardiobacterium                                                Cardiobacterium
## Aggregatibacter                                                Aggregatibacter
## Haemophilus                                                        Haemophilus
## Family.Pasteurellaceae.                                Family.Pasteurellaceae.
## Saccharibacteria_(TM7)_[G.1]                      Saccharibacteria_(TM7)_[G.1]
## Saccharibacteria_(TM7)_[G.3]                      Saccharibacteria_(TM7)_[G.3]
## Saccharibacteria_(TM7)_[G.6]                      Saccharibacteria_(TM7)_[G.6]
## Family.Saccharibacteria_(TM7)_[F-1]. Family.Saccharibacteria_(TM7)_[F-1].
## Saccharibacteria_(TM7)_[G.5]                      Saccharibacteria_(TM7)_[G.5]
## Treponema                                                            Treponema
## Fretibacterium                                                  Fretibacterium
## Kingdom.Bacteria.                                            Kingdom.Bacteria.
## Others                                                                  Others
##                                                      Method
## Absconditabacteria_(SR1)_[G.1]       Kruskal-Wallis (kru)
## Actinomyces                          Kruskal-Wallis (kru)
## Peptidiphaga                         Kruskal-Wallis (kru)
## Schaalia                             Kruskal-Wallis (kru)
## Family.Actinomycetaceae.             Kruskal-Wallis (kru)
## Rothia                               Kruskal-Wallis (kru)
## Alloscardovia                        Kruskal-Wallis (kru)
## Bifidobacterium                      Kruskal-Wallis (kru)
## Parascardovia                        Kruskal-Wallis (kru)
## Scardovia                            Kruskal-Wallis (kru)
## Corynebacterium                      Kruskal-Wallis (kru)
## Olsenella                            Kruskal-Wallis (kru)
## Atopobium                            Kruskal-Wallis (kru)
## Slackia                              Kruskal-Wallis (kru)
## Cryptobacterium                      Kruskal-Wallis (kru)
## Bacteroidetes_[G.3]                  Kruskal-Wallis (kru)
## Bacteroidetes_[G.5]                  Kruskal-Wallis (kru)
## Family.Bacteroidetes_[F-1].          Kruskal-Wallis (kru)
## Bacteroidaceae_[G.1]                 Kruskal-Wallis (kru)
## Bacteroidales_[G.2]                  Kruskal-Wallis (kru)
## Porphyromonas                        Kruskal-Wallis (kru)
## Tannerella                           Kruskal-Wallis (kru)
## Alloprevotella                       Kruskal-Wallis (kru)
## Prevotella                           Kruskal-Wallis (kru)
## Bergeyella                           Kruskal-Wallis (kru)
## Capnocytophaga                       Kruskal-Wallis (kru)
## Gemella                              Kruskal-Wallis (kru)
## Abiotrophia                          Kruskal-Wallis (kru)
## Granulicatella                       Kruskal-Wallis (kru)
## Lactobacillus                        Kruskal-Wallis (kru)
## Streptococcus                        Kruskal-Wallis (kru)
## Order.Lactobacillales.               Kruskal-Wallis (kru)
## Class.Bacilli.                       Kruskal-Wallis (kru)
## Clostridiales_[F.1][G-1]             Kruskal-Wallis (kru)
## Butyrivibrio                         Kruskal-Wallis (kru)
## Catonella                            Kruskal-Wallis (kru)
## Johnsonella                          Kruskal-Wallis (kru)
## Lachnoanaerobaculum                  Kruskal-Wallis (kru)
## Lachnospiraceae_[G.2]                Kruskal-Wallis (kru)
```

```
## Lachnospiraceae_[G.3]                Kruskal-Wallis (kru)
## Lachnospiraceae_[G.7]                Kruskal-Wallis (kru)
## Lachnospiraceae_[G.8]                Kruskal-Wallis (kru)
## Oribacterium                         Kruskal-Wallis (kru)
## Shuttleworthia                       Kruskal-Wallis (kru)
## Stomatobaculum                       Kruskal-Wallis (kru)
## Family.Lachnospiraceae_[XIV].        Kruskal-Wallis (kru)
## Peptococcus                          Kruskal-Wallis (kru)
## Parvimonas                           Kruskal-Wallis (kru)
## Filifactor                           Kruskal-Wallis (kru)
## Mogibacterium                        Kruskal-Wallis (kru)
## Peptostreptococcaceae_[XI][G.1]      Kruskal-Wallis (kru)
## Peptostreptococcaceae_[XI][G.4]      Kruskal-Wallis (kru)
## Peptostreptococcaceae_[XI][G.5]      Kruskal-Wallis (kru)
## Peptostreptococcaceae_[XI][G.6]      Kruskal-Wallis (kru)
## Peptostreptococcaceae_[XI][G.7]      Kruskal-Wallis (kru)
## Peptostreptococcaceae_[XI][G.9]      Kruskal-Wallis (kru)
## Peptostreptococcus                   Kruskal-Wallis (kru)
## Ruminococcaceae_[G.1]                Kruskal-Wallis (kru)
## Ruminococcaceae_[G.2]                Kruskal-Wallis (kru)
## Bulleidia                            Kruskal-Wallis (kru)
## Eggerthia                            Kruskal-Wallis (kru)
## Solobacterium                        Kruskal-Wallis (kru)
## Mycoplasma                           Kruskal-Wallis (kru)
## Mitsuokella                          Kruskal-Wallis (kru)
## Selenomonas                          Kruskal-Wallis (kru)
## Family.Selenomonadaceae.             Kruskal-Wallis (kru)
## Class.Negativicutes.                 Kruskal-Wallis (kru)
## Anaeroglobus                         Kruskal-Wallis (kru)
## Dialister                            Kruskal-Wallis (kru)
## Megasphaera                          Kruskal-Wallis (kru)
## Family.Veillonellaceae.              Kruskal-Wallis (kru)
## Veillonella                          Kruskal-Wallis (kru)
## Veillonellaceae_[G.1]                Kruskal-Wallis (kru)
## Phylum.Firmicutes.                   Kruskal-Wallis (kru)
## Fusobacterium                        Kruskal-Wallis (kru)
## Leptotrichia                         Kruskal-Wallis (kru)
## Gracilibacteria_(GN02)_[G.1]         Kruskal-Wallis (kru)
## Lautropia                            Kruskal-Wallis (kru)
## Ottowia                              Kruskal-Wallis (kru)
## Eikenella                            Kruskal-Wallis (kru)
## Kingella                             Kruskal-Wallis (kru)
## Neisseria                            Kruskal-Wallis (kru)
## Family.Neisseriaceae.                Kruskal-Wallis (kru)
## Campylobacter                        Kruskal-Wallis (kru)
## Cardiobacterium                      Kruskal-Wallis (kru)
## Aggregatibacter                      Kruskal-Wallis (kru)
## Haemophilus                          Kruskal-Wallis (kru)
## Family.Pasteurellaceae.              Kruskal-Wallis (kru)
## Saccharibacteria_(TM7)_[G.1]         Kruskal-Wallis (kru)
## Saccharibacteria_(TM7)_[G.3]         Kruskal-Wallis (kru)
## Saccharibacteria_(TM7)_[G.6]         Kruskal-Wallis (kru)
## Family.Saccharibacteria_(TM7)_[F-1]. Kruskal-Wallis (kru)
## Saccharibacteria_(TM7)_[G.5]         Kruskal-Wallis (kru)
```

```
## Treponema                            Kruskal-Wallis (kru)
## Fretibacterium                       Kruskal-Wallis (kru)
## Kingdom.Bacteria.                    Kruskal-Wallis (kru)
## Others                               Kruskal-Wallis (kru)
```

```
write.table(final, file="pp_cat_kru.txt", sep="\t", dec=",", row.names=F)
write.table(final[final$pval<0.05,], file="pp_cat_kru_sig.txt", sep="\t", dec=",", row.names=F)
#################trig_cat
table(Phe$trig_cat, useNA="always")
```

```
##
## High  Low <NA>
##   72  674    0
```

```
final <- DA.ds2(Microbio, predictor = Phe$trig_cat)
final
```

```
##        baseMean log2FoldChange      lfcSE        stat         pval ordering
## 1    65.1989841    2.285843060 0.41418464  5.51889867 3.411308e-08 Low>High
## 2   323.9127377   -0.163757697 0.14058405 -1.16483839 2.440844e-01 High>Low
## 3    22.0460481   -0.458557119 0.19915888 -2.30246887 2.130875e-02 High>Low
## 4   704.3072222   -0.119418898 0.11564703 -1.03261530 3.017840e-01 High>Low
## 5     5.4421107   -0.499476432 0.28583812 -1.74741016 8.056622e-02 High>Low
## 6  1939.2520922   -0.034253241 0.13554162 -0.25271382 8.004894e-01 High>Low
## 7    12.4599091    0.185989529 0.35715673  0.52075045 6.025406e-01 Low>High
## 8    12.6386956   -0.022425136 0.40191638 -0.05579553 9.555047e-01 High>Low
## 9     2.9486113    0.374627826 0.54219890  0.69094170 4.896022e-01 Low>High
## 10   13.4028767    0.153871273 0.34425170  0.44697316 6.548944e-01 Low>High
## 11   93.7260406   -0.460232405 0.14705524 -3.12965672 1.750107e-03 High>Low
## 12    1.5669010    0.104747455 0.33194196  0.31555955 7.523368e-01 Low>High
## 13  120.4914295    0.027511268 0.13595739  0.20235213 8.396414e-01 Low>High
## 14    1.3927613    0.024353679 0.31659743  0.07692317 9.386847e-01 Low>High
## 15    4.3017007   -0.082918913 0.33733594 -0.24580516 8.058331e-01 High>Low
## 16    6.5912121   -0.516735488 0.35426338 -1.45861956 1.446698e-01 High>Low
## 17    4.6746313   -0.421299266 0.38682289 -1.08912702 2.760979e-01 High>Low
## 18    0.8448145    1.827598373 0.57256366  3.19195664 1.413125e-03 Low>High
## 19    0.7921891    0.127726595 0.53280649  0.23972417 8.105441e-01 Low>High
## 20   12.1604713   -0.034033259 0.20472648 -0.16623770 8.679699e-01 High>Low
## 21  680.1285750    0.450153830 0.18606996  2.41927197 1.555161e-02 Low>High
## 22   22.0547383   -0.239376376 0.13689902 -1.74856160 8.036683e-02 High>Low
## 23  361.8645217    0.232786580 0.14776442  1.57538998 1.151665e-01 Low>High
## 24 3811.4256813   -0.045830539 0.09395002 -0.48781831 6.256785e-01 High>Low
## 25   72.5563323    0.147472142 0.14515825  1.01594050 3.096577e-01 Low>High
## 26  225.1152973    0.265432484 0.15486574  1.71395225 8.653750e-02 Low>High
## 27  326.5314927    0.232895451 0.13967377  1.66742438 9.543005e-02 Low>High
## 28   29.9768046    0.774661435 0.28005828  2.76607224 5.673596e-03 Low>High
## 29  306.2864969    0.008285240 0.11299398  0.07332462 9.415478e-01 Low>High
## 30   46.1736152    1.797051065 0.47515902  3.78199927 1.555738e-04 Low>High
## 31 3803.9265541    0.075364363 0.10601736  0.71086812 4.771660e-01 Low>High
## 32    0.2860988    0.205911196 0.38363536  0.53673675 5.914495e-01 Low>High
## 33    0.5750915   -0.244858286 0.30291366 -0.80834350 4.188929e-01 High>Low
## 34    1.0542291   -0.509060281 0.45662946 -1.11482138 2.649270e-01 High>Low
## 35    6.9441711    0.353404553 0.29757149  1.18762908 2.349796e-01 Low>High
```

```
## 36   36.8211521   -0.039802471 0.16666711 -0.23881419 8.112497e-01 High>Low
## 37    0.9388209   -0.368025872 0.39698144 -0.92706064 3.538950e-01 High>Low
## 38   87.7811267   -0.041335236 0.11654927 -0.35465891 7.228451e-01 High>Low
## 39   27.9872677    0.141710094 0.30607916  0.46298511 6.433750e-01 Low>High
## 40    2.8891780    0.446894132 0.32288525  1.38406489 1.663386e-01 Low>High
## 41    1.4688223    0.959004362 0.47293317  2.02777987 4.258272e-02 Low>High
## 42    1.4317578    0.035298837 0.48555332  0.07269817 9.420463e-01 Low>High
## 43  147.0483774   -0.060379323 0.12912198 -0.46761461 6.400602e-01 High>Low
## 44    2.8039987   -0.113892663 0.35860885 -0.31759580 7.507916e-01 High>Low
## 45  112.6769059   -0.110819092 0.14520379 -0.76319695 4.453459e-01 High>Low
## 46    1.6039609    0.603951170 0.42193299  1.43139120 1.523181e-01 Low>High
## 47    3.7020760    0.858338876 0.36798936  2.33251003 1.967388e-02 Low>High
## 48   40.8491501    0.264285077 0.17926287  1.47428789 1.404041e-01 Low>High
## 49    6.8375183    0.266347802 0.35109258  0.75862554 4.480766e-01 Low>High
## 50   41.0770621   -0.214308960 0.12329822 -1.73813504 8.218702e-02 High>Low
## 51   67.4763877    0.112132291 0.22152707  0.50617873 6.127312e-01 Low>High
## 52    0.4176611    0.173504018 0.48064236  0.36098362 7.181117e-01 Low>High
## 53    1.6627493   -0.080913829 0.50273739 -0.16094651 8.721355e-01 High>Low
## 54    1.0137786    0.283333721 0.50265054  0.56367933 5.729724e-01 Low>High
## 55    4.7083982   -0.003473173 0.34527664 -0.01005910 9.919741e-01 High>Low
## 56    8.7265107   -0.231168658 0.20356733 -1.13558817 2.561290e-01 High>Low
## 57   42.8338056    0.342722268 0.25871195  1.32472532 1.852623e-01 Low>High
## 58   25.1020535    0.571329873 0.26256433  2.17596149 2.955814e-02 Low>High
## 59   41.4593204    0.152614761 0.21837767  0.69885699 4.846414e-01 Low>High
## 60    3.8570393   -0.901822015 0.37830996 -2.38381782 1.713408e-02 High>Low
## 61    1.5287700    0.254085760 0.51880427  0.48975264 6.243089e-01 Low>High
## 62   74.0587622    0.087662009 0.14560733  0.60204392 5.471449e-01 Low>High
## 63    4.4957386    0.026174227 0.30803636  0.08497123 9.322843e-01 Low>High
## 64   26.8689347   -0.387003770 0.27549149 -1.40477576 1.600880e-01 High>Low
## 65  221.9840267   -0.002217997 0.12409837 -0.01787289 9.857403e-01 High>Low
## 66    5.0346400    0.053756956 0.32524168  0.16528311 8.687212e-01 Low>High
## 67    0.7371549    0.149839931 0.30766207  0.48702763 6.262388e-01 Low>High
## 68    8.7009758    0.095054882 0.29758471  0.31942126 7.494071e-01 Low>High
## 69   36.4394005    0.047290175 0.15268647  0.30972080 7.567733e-01 Low>High
## 70  407.9853210   -0.007468820 0.16060815 -0.04650337 9.629090e-01 High>Low
## 71    0.5266292    0.005431392 0.35407102  0.01533984 9.877611e-01 Low>High
## 72 5067.7924258   -0.099420553 0.09032862 -1.10065393 2.710473e-01 High>Low
## 73    6.1442803   -0.414818569 0.24865774 -1.66823107 9.526987e-02 High>Low
## 74    0.8912626    1.135193922 0.43631955  2.60174894 9.274972e-03 Low>High
## 75  927.1066406    0.154201436 0.12006343  1.28433311 1.990254e-01 Low>High
## 76  683.2517404   -0.115606223 0.12389126 -0.93312657 3.507546e-01 High>Low
## 77    2.4068360    0.243094115 0.40411615  0.60154516 5.474769e-01 Low>High
## 78   68.4201506    0.087160676 0.23075149  0.37772530 7.056347e-01 Low>High
## 79    2.3342613    1.041226808 0.52658557  1.97731740 4.800576e-02 Low>High
## 80    8.7736001    0.641850889 0.19656338  3.26536350 1.093237e-03 Low>High
## 81   99.8204227    0.431723178 0.16574623  2.60472399 9.194830e-03 Low>High
## 82 1329.0853342    0.424358906 0.18934530  2.24119058 2.501373e-02 Low>High
## 83    5.6824349    0.089823084 0.43643363  0.20581156 8.369381e-01 Low>High
## 84  257.8128378    0.078146069 0.10635895  0.73473904 4.624984e-01 Low>High
## 85   14.7982481    0.059864301 0.16643302  0.35969006 7.190789e-01 Low>High
## 86  275.5218474    0.018603575 0.21888307  0.08499321 9.322668e-01 Low>High
## 87 2905.9316825   -0.124925271 0.14612016 -0.85494888 3.925794e-01 High>Low
## 88    2.5859315   -1.022543312 0.59480975 -1.71910986 8.559437e-02 High>Low
## 89   36.0392035   -0.491558871 0.24303974 -2.02254521 4.312006e-02 High>Low
```

```
## 90    7.1137273    0.171178004 0.34071824  0.50240341 6.153838e-01 Low>High
## 91    6.8660111    0.707919197 0.43104294  1.64234033 1.005195e-01 Low>High
## 92    1.1003857    0.031422208 0.54014614  0.05817353 9.536104e-01 Low>High
## 93    0.9916119   -0.415802608 0.51287851 -0.81072340 4.175245e-01 High>Low
## 94   63.1946030   -0.137126523 0.23107836 -0.59342002 5.529001e-01 High>Low
## 95    8.2679474   -0.363536028 0.27866609 -1.30455783 1.920435e-01 High>Low
## 96    9.2007479    0.552064290 0.35461557  1.55679655 1.195188e-01 Low>High
## 97   33.1524239    1.026577533 0.23565570  4.35626014 1.323035e-05 Low>High
##          pval.adj                           Feature                    Method
## 1  3.308969e-06      Absconditabacteria_(SR1)_[G.1] DESeq2 man. geoMeans (ds2)
## 2  6.230576e-01                         Actinomyces DESeq2 man. geoMeans (ds2)
## 3  1.589960e-01                        Peptidiphaga DESeq2 man. geoMeans (ds2)
## 4  6.807685e-01                            Schaalia DESeq2 man. geoMeans (ds2)
## 5  3.649625e-01            Family.Actinomycetaceae. DESeq2 man. geoMeans (ds2)
## 6  9.714965e-01                              Rothia DESeq2 man. geoMeans (ds2)
## 7  9.177556e-01                       Alloscardovia DESeq2 man. geoMeans (ds2)
## 8  9.919741e-01                     Bifidobacterium DESeq2 man. geoMeans (ds2)
## 9  8.634802e-01                       Parascardovia DESeq2 man. geoMeans (ds2)
## 10 9.206487e-01                           Scardovia DESeq2 man. geoMeans (ds2)
## 11 2.829340e-02                     Corynebacterium DESeq2 man. geoMeans (ds2)
## 12 9.533378e-01                           Olsenella DESeq2 man. geoMeans (ds2)
## 13 9.812677e-01                           Atopobium DESeq2 man. geoMeans (ds2)
## 14 9.919741e-01                             Slackia DESeq2 man. geoMeans (ds2)
## 15 9.714965e-01                     Cryptobacterium DESeq2 man. geoMeans (ds2)
## 16 4.677658e-01                 Bacteroidetes_[G.3] DESeq2 man. geoMeans (ds2)
## 17 6.376546e-01                 Bacteroidetes_[G.5] DESeq2 man. geoMeans (ds2)
## 18 2.741463e-02         Family.Bacteroidetes_[F-1]. DESeq2 man. geoMeans (ds2)
## 19 9.714965e-01                Bacteroidaceae_[G.1] DESeq2 man. geoMeans (ds2)
## 20 9.836877e-01                 Bacteroidales_[G.2] DESeq2 man. geoMeans (ds2)
## 21 1.508506e-01                       Porphyromonas DESeq2 man. geoMeans (ds2)
## 22 3.649625e-01                         Tannerella DESeq2 man. geoMeans (ds2)
## 23 4.137462e-01                      Alloprevotella DESeq2 man. geoMeans (ds2)
## 24 9.177556e-01                          Prevotella DESeq2 man. geoMeans (ds2)
## 25 6.826545e-01                          Bergeyella DESeq2 man. geoMeans (ds2)
## 26 3.649625e-01                      Capnocytophaga DESeq2 man. geoMeans (ds2)
## 27 3.702686e-01                             Gemella DESeq2 man. geoMeans (ds2)
## 28 7.861984e-02                         Abiotrophia DESeq2 man. geoMeans (ds2)
## 29 9.919741e-01                      Granulicatella DESeq2 man. geoMeans (ds2)
## 30 5.030220e-03                       Lactobacillus DESeq2 man. geoMeans (ds2)
## 31 8.634802e-01                       Streptococcus DESeq2 man. geoMeans (ds2)
## 32 9.177556e-01               Order.Lactobacillales. DESeq2 man. geoMeans (ds2)
## 33 8.292369e-01                      Class.Bacilli. DESeq2 man. geoMeans (ds2)
## 34 6.376546e-01            Clostridiales_[F.1][G-1] DESeq2 man. geoMeans (ds2)
## 35 6.160275e-01                        Butyrivibrio DESeq2 man. geoMeans (ds2)
## 36 9.714965e-01                           Catonella DESeq2 man. geoMeans (ds2)
## 37 7.462569e-01                         Johnsonella DESeq2 man. geoMeans (ds2)
## 38 9.533378e-01                 Lachnoanaerobaculum DESeq2 man. geoMeans (ds2)
## 39 9.177556e-01               Lachnospiraceae_[G.2] DESeq2 man. geoMeans (ds2)
## 40 4.889346e-01               Lachnospiraceae_[G.3] DESeq2 man. geoMeans (ds2)
## 41 2.460380e-01               Lachnospiraceae_[G.7] DESeq2 man. geoMeans (ds2)
## 42 9.919741e-01               Lachnospiraceae_[G.8] DESeq2 man. geoMeans (ds2)
## 43 9.177556e-01                        Oribacterium DESeq2 man. geoMeans (ds2)
## 44 9.533378e-01                      Shuttleworthia DESeq2 man. geoMeans (ds2)
## 45 8.522241e-01                      Stomatobaculum DESeq2 man. geoMeans (ds2)
```

```
## 46 4.766083e-01        Family.Lachnospiraceae_[XIV]. DESeq2 man. geoMeans (ds2)
## 47 1.589960e-01                           Peptococcus DESeq2 man. geoMeans (ds2)
## 48 4.677658e-01                           Parvimonas DESeq2 man. geoMeans (ds2)
## 49 8.522241e-01                           Filifactor DESeq2 man. geoMeans (ds2)
## 50 3.649625e-01                         Mogibacterium DESeq2 man. geoMeans (ds2)
## 51 9.177556e-01      Peptostreptococcaceae_[XI][G.1] DESeq2 man. geoMeans (ds2)
## 52 9.533378e-01      Peptostreptococcaceae_[XI][G.4] DESeq2 man. geoMeans (ds2)
## 53 9.836877e-01      Peptostreptococcaceae_[XI][G.5] DESeq2 man. geoMeans (ds2)
## 54 9.177556e-01      Peptostreptococcaceae_[XI][G.6] DESeq2 man. geoMeans (ds2)
## 55 9.919741e-01      Peptostreptococcaceae_[XI][G.7] DESeq2 man. geoMeans (ds2)
## 56 6.370387e-01      Peptostreptococcaceae_[XI][G.9] DESeq2 man. geoMeans (ds2)
## 57 5.285424e-01                  Peptostreptococcus DESeq2 man. geoMeans (ds2)
## 58 1.911426e-01                Ruminococcaceae_[G.1] DESeq2 man. geoMeans (ds2)
## 59 8.634802e-01                Ruminococcaceae_[G.2] DESeq2 man. geoMeans (ds2)
## 60 1.510915e-01                            Bulleidia DESeq2 man. geoMeans (ds2)
## 61 9.177556e-01                            Eggerthia DESeq2 man. geoMeans (ds2)
## 62 9.177556e-01                         Solobacterium DESeq2 man. geoMeans (ds2)
## 63 9.919741e-01                           Mycoplasma DESeq2 man. geoMeans (ds2)
## 64 4.852667e-01                          Mitsuokella DESeq2 man. geoMeans (ds2)
## 65 9.919741e-01                          Selenomonas DESeq2 man. geoMeans (ds2)
## 66 9.836877e-01            Family.Selenomonadaceae. DESeq2 man. geoMeans (ds2)
## 67 9.177556e-01                Class.Negativicutes. DESeq2 man. geoMeans (ds2)
## 68 9.533378e-01                         Anaeroglobus DESeq2 man. geoMeans (ds2)
## 69 9.533378e-01                            Dialister DESeq2 man. geoMeans (ds2)
## 70 9.919741e-01                          Megasphaera DESeq2 man. geoMeans (ds2)
## 71 9.919741e-01             Family.Veillonellaceae. DESeq2 man. geoMeans (ds2)
## 72 6.376546e-01                          Veillonella DESeq2 man. geoMeans (ds2)
## 73 3.702686e-01                Veillonellaceae_[G.1] DESeq2 man. geoMeans (ds2)
## 74 9.996359e-02                  Phylum.Firmicutes. DESeq2 man. geoMeans (ds2)
## 75 5.362630e-01                         Fusobacterium DESeq2 man. geoMeans (ds2)
## 76 7.462569e-01                          Leptotrichia DESeq2 man. geoMeans (ds2)
## 77 9.177556e-01         Gracilibacteria_(GN02)_[G.1] DESeq2 man. geoMeans (ds2)
## 78 9.533378e-01                            Lautropia DESeq2 man. geoMeans (ds2)
## 79 2.586977e-01                              Ottowia DESeq2 man. geoMeans (ds2)
## 80 2.651099e-02                            Eikenella DESeq2 man. geoMeans (ds2)
## 81 9.996359e-02                             Kingella DESeq2 man. geoMeans (ds2)
## 82 1.733094e-01                            Neisseria DESeq2 man. geoMeans (ds2)
## 83 9.812677e-01                Family.Neisseriaceae. DESeq2 man. geoMeans (ds2)
## 84 8.627375e-01                         Campylobacter DESeq2 man. geoMeans (ds2)
## 85 9.533378e-01                        Cardiobacterium DESeq2 man. geoMeans (ds2)
## 86 9.919741e-01                       Aggregatibacter DESeq2 man. geoMeans (ds2)
## 87 8.102172e-01                          Haemophilus DESeq2 man. geoMeans (ds2)
## 88 3.649625e-01             Family.Pasteurellaceae. DESeq2 man. geoMeans (ds2)
## 89 2.460380e-01         Saccharibacteria_(TM7)_[G.1] DESeq2 man. geoMeans (ds2)
## 90 9.177556e-01         Saccharibacteria_(TM7)_[G.3] DESeq2 man. geoMeans (ds2)
## 91 3.750150e-01         Saccharibacteria_(TM7)_[G.6] DESeq2 man. geoMeans (ds2)
## 92 9.919741e-01 Family.Saccharibacteria_(TM7)_[F-1]. DESeq2 man. geoMeans (ds2)
## 93 8.292369e-01         Saccharibacteria_(TM7)_[G.5] DESeq2 man. geoMeans (ds2)
## 94 9.177556e-01                            Treponema DESeq2 man. geoMeans (ds2)
## 95 5.322347e-01                        Fretibacterium DESeq2 man. geoMeans (ds2)
## 96 4.140473e-01                   Kingdom.Bacteria. DESeq2 man. geoMeans (ds2)
## 97 6.416721e-04                               Others DESeq2 man. geoMeans (ds2)
```

```r
write.table(final, file="trig_cat_ds2.txt", sep="\t", dec=",", row.names=F)
write.table(final[final$pval<0.05,], file="trig_cat_ds2_sig.txt", sep="\t", dec=",", row.names=F)
final <- DA.kru(Microbio, predictor = Phe$trig_cat)
final
```

```
##                                                pval  pval.adj
## Absconditabacteria_(SR1)_[G.1]          0.225001589 0.6142857
## Actinomyces                             0.001728111 0.1676268
## Peptidiphaga                            0.024366347 0.2740090
## Schaalia                                0.070286565 0.4274652
## Family.Actinomycetaceae.                0.011647832 0.2259679
## Rothia                                  0.151799696 0.5258775
## Alloscardovia                           0.891607537 0.9769813
## Bifidobacterium                         0.348744401 0.7335800
## Parascardovia                           0.763074086 0.9369391
## Scardovia                               0.046027655 0.3240171
## Corynebacterium                         0.021013362 0.2740090
## Olsenella                               0.565768770 0.8574933
## Atopobium                               0.920255914 0.9769813
## Slackia                                 0.706705204 0.9019790
## Cryptobacterium                         0.677366653 0.8875264
## Bacteroidetes_[G.3]                     0.519331868 0.8125031
## Bacteroidetes_[G.5]                     0.477602012 0.8083845
## Family.Bacteroidetes_[F-1].             0.004537426 0.2200652
## Bacteroidaceae_[G.1]                    0.252740160 0.6625891
## Bacteroidales_[G.2]                     0.447228264 0.7973327
## Porphyromonas                           0.046765360 0.3240171
## Tannerella                              0.100033958 0.4726224
## Alloprevotella                          0.118404092 0.4726224
## Prevotella                              0.972461569 0.9839821
## Bergeyella                              0.308498878 0.7200042
## Capnocytophaga                          0.311515803 0.7200042
## Gemella                                 0.304689331 0.7200042
## Abiotrophia                             0.010257303 0.2259679
## Granulicatella                          0.973837983 0.9839821
## Lactobacillus                           0.302344606 0.7200042
## Streptococcus                           0.462156382 0.8005209
## Order.Lactobacillales.                  0.409255474 0.7783879
## Class.Bacilli.                          0.426188797 0.7950060
## Clostridiales_[F.1][G-1]                0.967307570 0.9839821
## Butyrivibrio                            0.751196788 0.9341806
## Catonella                               0.686231721 0.8875264
## Johnsonella                             0.491697799 0.8083845
## Lachnoanaerobaculum                     0.862960848 0.9733396
## Lachnospiraceae_[G.2]                   0.936693392 0.9769813
## Lachnospiraceae_[G.3]                   0.613771882 0.8686020
## Lachnospiraceae_[G.7]                   0.560320981 0.8574933
## Lachnospiraceae_[G.8]                   0.616200896 0.8686020
## Oribacterium                            0.853247866 0.9733396
## Shuttleworthia                          0.195129886 0.5914875
## Stomatobaculum                          0.685652880 0.8875264
## Family.Lachnospiraceae_[XIV].           0.595128850 0.8686020
## Peptococcus                             0.452095872 0.7973327
```

```
## Parvimonas                              0.192702492 0.5914875
## Filifactor                              0.656140238 0.8875264
## Mogibacterium                           0.070509719 0.4274652
## Peptostreptococcaceae_[XI][G.1]         0.355445968 0.7335800
## Peptostreptococcaceae_[XI][G.4]         0.915539067 0.9769813
## Peptostreptococcaceae_[XI][G.5]         0.489047531 0.8083845
## Peptostreptococcaceae_[XI][G.6]         0.928636512 0.9769813
## Peptostreptococcaceae_[XI][G.7]         0.932440516 0.9769813
## Peptostreptococcaceae_[XI][G.9]         0.510516445 0.8118048
## Peptostreptococcus                      0.103002228 0.4726224
## Ruminococcaceae_[G.1]                   0.184522444 0.5914875
## Ruminococcaceae_[G.2]                   0.318592621 0.7200042
## Bulleidia                               0.033558980 0.2855895
## Eggerthia                               0.351304570 0.7335800
## Solobacterium                           0.126049179 0.4726224
## Mycoplasma                              0.857921854 0.9733396
## Mitsuokella                             0.192201768 0.5914875
## Selenomonas                             0.634204982 0.8788269
## Family.Selenomonadaceae.                0.385155494 0.7472017
## Class.Negativicutes.                    0.818817393 0.9686133
## Anaeroglobus                            0.748527400 0.9341806
## Dialister                               0.917515555 0.9769813
## Megasphaera                             0.990359869 0.9903599
## Family.Veillonellaceae.                 0.382540475 0.7472017
## Veillonella                             0.025819481 0.2740090
## Veillonellaceae_[G.1]                   0.023891767 0.2740090
## Phylum.Firmicutes.                      0.226193694 0.6142857
## Fusobacterium                           0.131554697 0.4726224
## Leptotrichia                            0.509668375 0.8118048
## Gracilibacteria_(GN02)_[G.1]            0.210226902 0.6142857
## Lautropia                               0.678697352 0.8875264
## Ottowia                                 0.035330663 0.2855895
## Eikenella                               0.008998517 0.2259679
## Kingella                                0.088612655 0.4726224
## Neisseria                               0.028248352 0.2740090
## Family.Neisseriaceae.                   0.606744868 0.8686020
## Campylobacter                           0.451713589 0.7973327
## Cardiobacterium                         0.818827749 0.9686133
## Aggregatibacter                         0.347978530 0.7335800
## Haemophilus                             0.114389350 0.4726224
## Family.Pasteurellaceae.                 0.227982313 0.6142857
## Saccharibacteria_(TM7)_[G.1]            0.319177109 0.7200042
## Saccharibacteria_(TM7)_[G.3]            0.808428402 0.9686133
## Saccharibacteria_(TM7)_[G.6]            0.078012832 0.4451320
## Family.Saccharibacteria_(TM7)_[F-1]. 0.377995335 0.7472017
## Saccharibacteria_(TM7)_[G.5]            0.111729241 0.4726224
## Treponema                               0.617871543 0.8686020
## Fretibacterium                          0.131291278 0.4726224
## Kingdom.Bacteria.                       0.829352530 0.9692433
## Others                                  0.094290782 0.4726224
##                                                                   Feature
## Absconditabacteria_(SR1)_[G.1]             Absconditabacteria_(SR1)_[G.1]
## Actinomyces                                                   Actinomyces
## Peptidiphaga                                                 Peptidiphaga
```

```
## Schaalia                                                         Schaalia
## Family.Actinomycetaceae.                         Family.Actinomycetaceae.
## Rothia                                                             Rothia
## Alloscardovia                                               Alloscardovia
## Bifidobacterium                                           Bifidobacterium
## Parascardovia                                               Parascardovia
## Scardovia                                                       Scardovia
## Corynebacterium                                           Corynebacterium
## Olsenella                                                       Olsenella
## Atopobium                                                       Atopobium
## Slackia                                                           Slackia
## Cryptobacterium                                           Cryptobacterium
## Bacteroidetes_[G.3]                                   Bacteroidetes_[G.3]
## Bacteroidetes_[G.5]                                   Bacteroidetes_[G.5]
## Family.Bacteroidetes_[F-1].                   Family.Bacteroidetes_[F-1].
## Bacteroidaceae_[G.1]                                 Bacteroidaceae_[G.1]
## Bacteroidales_[G.2]                                   Bacteroidales_[G.2]
## Porphyromonas                                               Porphyromonas
## Tannerella                                                     Tannerella
## Alloprevotella                                             Alloprevotella
## Prevotella                                                     Prevotella
## Bergeyella                                                     Bergeyella
## Capnocytophaga                                             Capnocytophaga
## Gemella                                                           Gemella
## Abiotrophia                                                   Abiotrophia
## Granulicatella                                             Granulicatella
## Lactobacillus                                               Lactobacillus
## Streptococcus                                               Streptococcus
## Order.Lactobacillales.                             Order.Lactobacillales.
## Class.Bacilli.                                             Class.Bacilli.
## Clostridiales_[F.1][G-1]                         Clostridiales_[F.1][G-1]
## Butyrivibrio                                                 Butyrivibrio
## Catonella                                                       Catonella
## Johnsonella                                                   Johnsonella
## Lachnoanaerobaculum                                   Lachnoanaerobaculum
## Lachnospiraceae_[G.2]                               Lachnospiraceae_[G.2]
## Lachnospiraceae_[G.3]                               Lachnospiraceae_[G.3]
## Lachnospiraceae_[G.7]                               Lachnospiraceae_[G.7]
## Lachnospiraceae_[G.8]                               Lachnospiraceae_[G.8]
## Oribacterium                                                 Oribacterium
## Shuttleworthia                                             Shuttleworthia
## Stomatobaculum                                             Stomatobaculum
## Family.Lachnospiraceae_[XIV].               Family.Lachnospiraceae_[XIV].
## Peptococcus                                                   Peptococcus
## Parvimonas                                                     Parvimonas
## Filifactor                                                     Filifactor
## Mogibacterium                                               Mogibacterium
## Peptostreptococcaceae_[XI][G.1]           Peptostreptococcaceae_[XI][G.1]
## Peptostreptococcaceae_[XI][G.4]           Peptostreptococcaceae_[XI][G.4]
## Peptostreptococcaceae_[XI][G.5]           Peptostreptococcaceae_[XI][G.5]
## Peptostreptococcaceae_[XI][G.6]           Peptostreptococcaceae_[XI][G.6]
## Peptostreptococcaceae_[XI][G.7]           Peptostreptococcaceae_[XI][G.7]
## Peptostreptococcaceae_[XI][G.9]           Peptostreptococcaceae_[XI][G.9]
## Peptostreptococcus                                     Peptostreptococcus
```

```
## Ruminococcaceae_[G.1]                                 Ruminococcaceae_[G.1]
## Ruminococcaceae_[G.2]                                 Ruminococcaceae_[G.2]
## Bulleidia                                                          Bulleidia
## Eggerthia                                                          Eggerthia
## Solobacterium                                                  Solobacterium
## Mycoplasma                                                        Mycoplasma
## Mitsuokella                                                      Mitsuokella
## Selenomonas                                                      Selenomonas
## Family.Selenomonadaceae.                             Family.Selenomonadaceae.
## Class.Negativicutes.                                     Class.Negativicutes.
## Anaeroglobus                                                    Anaeroglobus
## Dialister                                                          Dialister
## Megasphaera                                                      Megasphaera
## Family.Veillonellaceae.                               Family.Veillonellaceae.
## Veillonella                                                      Veillonella
## Veillonellaceae_[G.1]                                 Veillonellaceae_[G.1]
## Phylum.Firmicutes.                                       Phylum.Firmicutes.
## Fusobacterium                                                  Fusobacterium
## Leptotrichia                                                    Leptotrichia
## Gracilibacteria_(GN02)_[G.1]                   Gracilibacteria_(GN02)_[G.1]
## Lautropia                                                          Lautropia
## Ottowia                                                              Ottowia
## Eikenella                                                          Eikenella
## Kingella                                                            Kingella
## Neisseria                                                          Neisseria
## Family.Neisseriaceae.                                 Family.Neisseriaceae.
## Campylobacter                                                  Campylobacter
## Cardiobacterium                                              Cardiobacterium
## Aggregatibacter                                              Aggregatibacter
## Haemophilus                                                      Haemophilus
## Family.Pasteurellaceae.                               Family.Pasteurellaceae.
## Saccharibacteria_(TM7)_[G.1]                   Saccharibacteria_(TM7)_[G.1]
## Saccharibacteria_(TM7)_[G.3]                   Saccharibacteria_(TM7)_[G.3]
## Saccharibacteria_(TM7)_[G.6]                   Saccharibacteria_(TM7)_[G.6]
## Family.Saccharibacteria_(TM7)_[F-1]. Family.Saccharibacteria_(TM7)_[F-1].
## Saccharibacteria_(TM7)_[G.5]                   Saccharibacteria_(TM7)_[G.5]
## Treponema                                                          Treponema
## Fretibacterium                                                Fretibacterium
## Kingdom.Bacteria.                                          Kingdom.Bacteria.
## Others                                                                Others
##                                                     Method
## Absconditabacteria_(SR1)_[G.1]       Kruskal-Wallis (kru)
## Actinomyces                          Kruskal-Wallis (kru)
## Peptidiphaga                         Kruskal-Wallis (kru)
## Schaalia                             Kruskal-Wallis (kru)
## Family.Actinomycetaceae.             Kruskal-Wallis (kru)
## Rothia                               Kruskal-Wallis (kru)
## Alloscardovia                        Kruskal-Wallis (kru)
## Bifidobacterium                      Kruskal-Wallis (kru)
## Parascardovia                        Kruskal-Wallis (kru)
## Scardovia                            Kruskal-Wallis (kru)
## Corynebacterium                      Kruskal-Wallis (kru)
## Olsenella                            Kruskal-Wallis (kru)
## Atopobium                            Kruskal-Wallis (kru)
```

```
## Slackia                              Kruskal-Wallis (kru)
## Cryptobacterium                      Kruskal-Wallis (kru)
## Bacteroidetes_[G.3]                  Kruskal-Wallis (kru)
## Bacteroidetes_[G.5]                  Kruskal-Wallis (kru)
## Family.Bacteroidetes_[F-1].          Kruskal-Wallis (kru)
## Bacteroidaceae_[G.1]                 Kruskal-Wallis (kru)
## Bacteroidales_[G.2]                  Kruskal-Wallis (kru)
## Porphyromonas                        Kruskal-Wallis (kru)
## Tannerella                           Kruskal-Wallis (kru)
## Alloprevotella                       Kruskal-Wallis (kru)
## Prevotella                           Kruskal-Wallis (kru)
## Bergeyella                           Kruskal-Wallis (kru)
## Capnocytophaga                       Kruskal-Wallis (kru)
## Gemella                              Kruskal-Wallis (kru)
## Abiotrophia                          Kruskal-Wallis (kru)
## Granulicatella                       Kruskal-Wallis (kru)
## Lactobacillus                        Kruskal-Wallis (kru)
## Streptococcus                        Kruskal-Wallis (kru)
## Order.Lactobacillales.               Kruskal-Wallis (kru)
## Class.Bacilli.                       Kruskal-Wallis (kru)
## Clostridiales_[F.1][G-1]             Kruskal-Wallis (kru)
## Butyrivibrio                         Kruskal-Wallis (kru)
## Catonella                            Kruskal-Wallis (kru)
## Johnsonella                          Kruskal-Wallis (kru)
## Lachnoanaerobaculum                  Kruskal-Wallis (kru)
## Lachnospiraceae_[G.2]                Kruskal-Wallis (kru)
## Lachnospiraceae_[G.3]                Kruskal-Wallis (kru)
## Lachnospiraceae_[G.7]                Kruskal-Wallis (kru)
## Lachnospiraceae_[G.8]                Kruskal-Wallis (kru)
## Oribacterium                         Kruskal-Wallis (kru)
## Shuttleworthia                       Kruskal-Wallis (kru)
## Stomatobaculum                       Kruskal-Wallis (kru)
## Family.Lachnospiraceae_[XIV].        Kruskal-Wallis (kru)
## Peptococcus                          Kruskal-Wallis (kru)
## Parvimonas                           Kruskal-Wallis (kru)
## Filifactor                           Kruskal-Wallis (kru)
## Mogibacterium                        Kruskal-Wallis (kru)
## Peptostreptococcaceae_[XI][G.1]      Kruskal-Wallis (kru)
## Peptostreptococcaceae_[XI][G.4]      Kruskal-Wallis (kru)
## Peptostreptococcaceae_[XI][G.5]      Kruskal-Wallis (kru)
## Peptostreptococcaceae_[XI][G.6]      Kruskal-Wallis (kru)
## Peptostreptococcaceae_[XI][G.7]      Kruskal-Wallis (kru)
## Peptostreptococcaceae_[XI][G.9]      Kruskal-Wallis (kru)
## Peptostreptococcus                   Kruskal-Wallis (kru)
## Ruminococcaceae_[G.1]                Kruskal-Wallis (kru)
## Ruminococcaceae_[G.2]                Kruskal-Wallis (kru)
## Bulleidia                            Kruskal-Wallis (kru)
## Eggerthia                            Kruskal-Wallis (kru)
## Solobacterium                        Kruskal-Wallis (kru)
## Mycoplasma                           Kruskal-Wallis (kru)
## Mitsuokella                          Kruskal-Wallis (kru)
## Selenomonas                          Kruskal-Wallis (kru)
## Family.Selenomonadaceae.             Kruskal-Wallis (kru)
## Class.Negativicutes.                 Kruskal-Wallis (kru)
```

```
## Anaeroglobus                            Kruskal-Wallis (kru)
## Dialister                               Kruskal-Wallis (kru)
## Megasphaera                             Kruskal-Wallis (kru)
## Family.Veillonellaceae.                 Kruskal-Wallis (kru)
## Veillonella                             Kruskal-Wallis (kru)
## Veillonellaceae_[G.1]                   Kruskal-Wallis (kru)
## Phylum.Firmicutes.                      Kruskal-Wallis (kru)
## Fusobacterium                           Kruskal-Wallis (kru)
## Leptotrichia                            Kruskal-Wallis (kru)
## Gracilibacteria_(GN02)_[G.1]            Kruskal-Wallis (kru)
## Lautropia                               Kruskal-Wallis (kru)
## Ottowia                                 Kruskal-Wallis (kru)
## Eikenella                               Kruskal-Wallis (kru)
## Kingella                                Kruskal-Wallis (kru)
## Neisseria                               Kruskal-Wallis (kru)
## Family.Neisseriaceae.                   Kruskal-Wallis (kru)
## Campylobacter                           Kruskal-Wallis (kru)
## Cardiobacterium                         Kruskal-Wallis (kru)
## Aggregatibacter                         Kruskal-Wallis (kru)
## Haemophilus                             Kruskal-Wallis (kru)
## Family.Pasteurellaceae.                 Kruskal-Wallis (kru)
## Saccharibacteria_(TM7)_[G.1]            Kruskal-Wallis (kru)
## Saccharibacteria_(TM7)_[G.3]            Kruskal-Wallis (kru)
## Saccharibacteria_(TM7)_[G.6]            Kruskal-Wallis (kru)
## Family.Saccharibacteria_(TM7)_[F-1]. Kruskal-Wallis (kru)
## Saccharibacteria_(TM7)_[G.5]            Kruskal-Wallis (kru)
## Treponema                               Kruskal-Wallis (kru)
## Fretibacterium                          Kruskal-Wallis (kru)
## Kingdom.Bacteria.                       Kruskal-Wallis (kru)
## Others                                  Kruskal-Wallis (kru)
```

```
write.table(final, file="trig_cat_kru.txt", sep="\t", dec=",", row.names=F)
write.table(final[final$pval<0.05,], file="trig_cat_kru_sig.txt", sep="\t", dec=",", row.names=F)
#################hdlc_cat
table(Phe$hdlc_cat, useNA="always")
```

```
##
## High  Low <NA>
##  697   49    0
```

```
final <- DA.ds2(Microbio, predictor = Phe$hdlc_cat)
final
```

```
##        baseMean log2FoldChange     lfcSE        stat         pval ordering
## 1    65.1989841   -2.702421739 0.4956063 -5.45275869 4.959435e-08 High>Low
## 2   323.9127377    0.274674097 0.1674040  1.64078599 1.008418e-01 Low>High
## 3    22.0460481    0.422662711 0.2376732  1.77833529 7.534880e-02 Low>High
## 4   704.3072222    0.207359980 0.1377280  1.50557655 1.321759e-01 Low>High
## 5     5.4421107    0.138810080 0.3436119  0.40397338 6.862323e-01 Low>High
## 6  1939.2520922    0.091595868 0.1615371  0.56702699 5.706958e-01 Low>High
## 7    12.4599091    0.198764198 0.4246168  0.46810249 6.397113e-01 Low>High
## 8    12.6386956    0.182387894 0.4786013  0.38108523 7.031400e-01 Low>High
## 9     2.9486113   -0.675250063 0.6492439 -1.04005606 2.983139e-01 High>Low
```

```
## 10   13.4028767  -0.287653244 0.4106651 -0.70045705 4.836419e-01 High>Low
## 11   93.7260406   0.415092342 0.1757533  2.36178932 1.818697e-02 Low>High
## 12    1.5669010  -0.062322087 0.3940616 -0.15815314 8.743361e-01 High>Low
## 13  120.4914295  -0.059000542 0.1620582 -0.36407005 7.158057e-01 High>Low
## 14    1.3927613  -0.471266163 0.3897949 -1.20901046 2.266588e-01 High>Low
## 15    4.3017007   0.205665610 0.4007991  0.51313894 6.078541e-01 Low>High
## 16    6.5912121   0.578603193 0.4219117  1.37138450 1.702551e-01 Low>High
## 17    4.6746313   1.220674930 0.4537945  2.68992886 7.146725e-03 Low>High
## 18    0.8680201  -1.134005980 0.6879402 -1.64840788 9.926900e-02 High>Low
## 19    0.7921891  -0.462080815 0.6410157 -0.72085725 4.709973e-01 High>Low
## 20   12.1604713   0.133272443 0.2433763  0.54759819 5.839678e-01 Low>High
## 21  680.1285750  -0.532890452 0.2218322 -2.40222280 1.629578e-02 High>Low
## 22   22.0547383   0.038895346 0.1641092  0.23700889 8.126499e-01 Low>High
## 23  361.8645217  -0.291152437 0.1761292 -1.65306182 9.831828e-02 High>Low
## 24 3811.4256813  -0.041865771 0.1119951 -0.37381783 7.085399e-01 High>Low
## 25   72.5563323  -0.225558956 0.1730486 -1.30344266 1.924237e-01 High>Low
## 26  225.1152973  -0.298831145 0.1846470 -1.61839179 1.055782e-01 High>Low
## 27  326.5314927  -0.506009679 0.1660395 -3.04752600 2.307336e-03 High>Low
## 28   29.9768046  -1.027985255 0.3343581 -3.07450418 2.108528e-03 High>Low
## 29  306.2864969   0.020342184 0.1346653  0.15105740 8.799304e-01 Low>High
## 30   45.9050583  -1.857810156 0.5672994 -3.27483165 1.057250e-03 High>Low
## 31 3803.9265541  -0.026550484 0.1264030 -0.21004627 8.336316e-01 High>Low
## 32    0.2860988  -0.080629315 0.4558993 -0.17685774 8.596201e-01 High>Low
## 33    0.5750915  -0.099217997 0.3695214 -0.26850404 7.883114e-01 High>Low
## 34    1.0542291   1.269145070 0.5270672  2.40793790 1.604291e-02 Low>High
## 35    6.9441711  -0.228469849 0.3540241 -0.64535106 5.186997e-01 High>Low
## 36   36.8211521   0.154148690 0.1983152  0.77729135 4.369869e-01 Low>High
## 37    0.9388209   0.891986234 0.4578544  1.94818734 5.139255e-02 Low>High
## 38   87.7811267  -0.242975618 0.1390125 -1.74786884 8.048674e-02 High>Low
## 39   27.9872677  -0.988529252 0.3652367 -2.70654399 6.798757e-03 High>Low
## 40    2.8891780  -0.375207227 0.3839506 -0.97722789 3.284563e-01 High>Low
## 41    1.4688223  -0.372211507 0.5505174 -0.67611214 4.989695e-01 High>Low
## 42    1.4317578   0.312087496 0.5725283  0.54510403 5.856820e-01 Low>High
## 43  147.0483774   0.092060587 0.1538590  0.59834373 5.496106e-01 Low>High
## 44    2.8039987  -0.233961505 0.4312773 -0.54248513 5.874844e-01 High>Low
## 45  112.6769059  -0.419108206 0.1730187 -2.42232869 1.542139e-02 High>Low
## 46    1.6039609  -0.184477644 0.4943416 -0.37317846 7.090156e-01 High>Low
## 47    3.7020760  -0.363855376 0.4339211 -0.83852892 4.017337e-01 High>Low
## 48   40.8491501  -0.226177622 0.2137131 -1.05832364 2.899079e-01 High>Low
## 49    6.8375183   0.334867766 0.4153368  0.80625589 4.200953e-01 Low>High
## 50   41.0770621  -0.101643523 0.1478524 -0.68746617 4.917890e-01 High>Low
## 51   67.4763877  -0.228638613 0.2640848 -0.86577738 3.866123e-01 High>Low
## 52    0.4176611   0.666674127 0.5551953  1.20079206 2.298319e-01 Low>High
## 53    1.6627493   0.708954939 0.5901567  1.20129957 2.296350e-01 Low>High
## 54    1.0137786   0.418839684 0.5828885  0.71855889 4.724128e-01 Low>High
## 55    4.5862907  -0.182012226 0.4096289 -0.44433440 6.568008e-01 High>Low
## 56    8.7265107   0.514158292 0.2402422  2.14016607 3.234135e-02 Low>High
## 57   42.8338056  -0.442794929 0.3084878 -1.43537271 1.511809e-01 High>Low
## 58   25.1020535  -0.314383482 0.3130494 -1.00426151 3.152526e-01 High>Low
## 59   41.4593204  -0.157695968 0.2603177 -0.60578271 5.446591e-01 High>Low
## 60    3.8570393   1.096656917 0.4492479  2.44109516 1.464280e-02 Low>High
## 61    1.5796110   0.363050767 0.6111356  0.59405930 5.524724e-01 Low>High
## 62   74.0587622  -0.230426140 0.1735857 -1.32744897 1.843602e-01 High>Low
## 63    4.4957386   0.370172915 0.3634538  1.01848672 3.084467e-01 Low>High
```

```
## 64   26.8689347   -0.192191666 0.3295940 -0.58311641 5.598149e-01 High>Low
## 65  221.9840267    0.005914854 0.1479102  0.03998949 9.681015e-01 Low>High
## 66    4.9080122   -0.769325732 0.3912627 -1.96626409 4.926811e-02 High>Low
## 67    0.7371549   -0.286379113 0.3692131 -0.77564725 4.379573e-01 High>Low
## 68    8.7009758   -0.030084420 0.3543149 -0.08490871 9.323340e-01 High>Low
## 69   36.4394005   -0.142685002 0.1821190 -0.78347138 4.333504e-01 High>Low
## 70  407.9853210   -0.206078594 0.1913677 -1.07687247 2.815372e-01 High>Low
## 71    0.5266292    0.041432021 0.4183264  0.09904234 9.211047e-01 Low>High
## 72 5067.7924258   -0.002690985 0.1077538 -0.02497346 9.800761e-01 High>Low
## 73    6.1442803    0.424907370 0.2961344  1.43484641 1.513309e-01 Low>High
## 74    0.8912626   -1.555271026 0.5228531 -2.97458504 2.933851e-03 High>Low
## 75  927.1066406   -0.120654268 0.1431865 -0.84263700 3.994315e-01 High>Low
## 76  683.2517404   -0.140510665 0.1477117 -0.95124911 3.414779e-01 High>Low
## 77    2.2788852   -0.683853622 0.4807322 -1.42252513 1.548739e-01 High>Low
## 78   68.4201506   -0.010124431 0.2750051 -0.03681543 9.706322e-01 High>Low
## 79    2.3342613   -1.570440865 0.6392567 -2.45666712 1.402326e-02 High>Low
## 80    8.7736001   -0.098521723 0.2307955 -0.42687887 6.694676e-01 High>Low
## 81   99.8204227   -0.567524661 0.1975907 -2.87222351 4.075946e-03 High>Low
## 82 1329.0853342   -0.325024412 0.2260135 -1.43807535 1.504127e-01 High>Low
## 83    5.6824349   -0.619815294 0.5229260 -1.18528306 2.359055e-01 High>Low
## 84  257.8128378   -0.162576672 0.1267279 -1.28287943 1.995343e-01 High>Low
## 85   14.7982481    0.172902433 0.1970289  0.87754876 3.801886e-01 Low>High
## 86  275.5218474    0.266689038 0.2606949  1.02299283 3.063112e-01 Low>High
## 87 2905.9316825    0.051795314 0.1742415  0.29726162 7.662668e-01 Low>High
## 88    2.5859315    0.798019831 0.7121278  1.12061327 2.624525e-01 Low>High
## 89   36.0392035    0.507402774 0.2897988  1.75087973 7.996663e-02 Low>High
## 90    7.1137273   -0.292305374 0.4067190 -0.71869121 4.723312e-01 High>Low
## 91    7.2376188   -0.697888079 0.5187619 -1.34529553 1.785298e-01 High>Low
## 92    1.1003857    0.063539344 0.6413559  0.09907033 9.210824e-01 Low>High
## 93    0.9916119    0.562977508 0.6071556  0.92723763 3.538032e-01 Low>High
## 94   63.1946030    0.642478063 0.2742191  2.34293670 1.913263e-02 Low>High
## 95    8.2679474    0.526440020 0.3310764  1.59008611 1.118154e-01 Low>High
## 96    9.3689884   -0.240427858 0.4232164 -0.56809676 5.699693e-01 High>Low
## 97   32.3921144   -0.730545393 0.2793031 -2.61560104 8.907056e-03 High>Low
##        pval.adj                          Feature                     Method
## 1  4.810652e-06     Absconditabacteria_(SR1)_[G.1] DESeq2 man. geoMeans (ds2)
## 2  3.912664e-01                      Actinomyces DESeq2 man. geoMeans (ds2)
## 3  3.548734e-01                     Peptidiphaga DESeq2 man. geoMeans (ds2)
## 4  4.578951e-01                         Schaalia DESeq2 man. geoMeans (ds2)
## 5  8.265852e-01         Family.Actinomycetaceae. DESeq2 man. geoMeans (ds2)
## 6  7.598131e-01                           Rothia DESeq2 man. geoMeans (ds2)
## 7  8.058701e-01                    Alloscardovia DESeq2 man. geoMeans (ds2)
## 8  8.265852e-01                  Bifidobacterium DESeq2 man. geoMeans (ds2)
## 9  6.365815e-01                    Parascardovia DESeq2 man. geoMeans (ds2)
## 10 7.446160e-01                        Scardovia DESeq2 man. geoMeans (ds2)
## 11 1.159916e-01                  Corynebacterium DESeq2 man. geoMeans (ds2)
## 12 9.379478e-01                        Olsenella DESeq2 man. geoMeans (ds2)
## 13 8.265852e-01                        Atopobium DESeq2 man. geoMeans (ds2)
## 14 5.573423e-01                          Slackia DESeq2 man. geoMeans (ds2)
## 15 7.758138e-01                  Cryptobacterium DESeq2 man. geoMeans (ds2)
## 16 5.004469e-01               Bacteroidetes_[G.3] DESeq2 man. geoMeans (ds2)
## 17 8.665404e-02               Bacteroidetes_[G.5] DESeq2 man. geoMeans (ds2)
## 18 3.912664e-01      Family.Bacteroidetes_[F-1]. DESeq2 man. geoMeans (ds2)
## 19 7.390974e-01              Bacteroidaceae_[G.1] DESeq2 man. geoMeans (ds2)
```

```
## 20 7.598131e-01              Bacteroidales_[G.2] DESeq2 man. geoMeans (ds2)
## 21 1.129065e-01                     Porphyromonas DESeq2 man. geoMeans (ds2)
## 22 9.060579e-01                        Tannerella DESeq2 man. geoMeans (ds2)
## 23 3.912664e-01                    Alloprevotella DESeq2 man. geoMeans (ds2)
## 24 8.265852e-01                        Prevotella DESeq2 man. geoMeans (ds2)
## 25 5.184749e-01                        Bergeyella DESeq2 man. geoMeans (ds2)
## 26 3.938879e-01                    Capnocytophaga DESeq2 man. geoMeans (ds2)
## 27 5.595289e-02                           Gemella DESeq2 man. geoMeans (ds2)
## 28 5.595289e-02                       Abiotrophia DESeq2 man. geoMeans (ds2)
## 29 9.379478e-01                    Granulicatella DESeq2 man. geoMeans (ds2)
## 30 5.127661e-02                     Lactobacillus DESeq2 man. geoMeans (ds2)
## 31 9.188893e-01                     Streptococcus DESeq2 man. geoMeans (ds2)
## 32 9.368894e-01           Order.Lactobacillales. DESeq2 man. geoMeans (ds2)
## 33 8.891419e-01                   Class.Bacilli. DESeq2 man. geoMeans (ds2)
## 34 1.129065e-01         Clostridiales_[F.1][G-1] DESeq2 man. geoMeans (ds2)
## 35 7.598131e-01                      Butyrivibrio DESeq2 man. geoMeans (ds2)
## 36 7.200315e-01                         Catonella DESeq2 man. geoMeans (ds2)
## 37 2.623725e-01                       Johnsonella DESeq2 man. geoMeans (ds2)
## 38 3.548734e-01              Lachnoanaerobaculum DESeq2 man. geoMeans (ds2)
## 39 8.665404e-02             Lachnospiraceae_[G.2] DESeq2 man. geoMeans (ds2)
## 40 6.502095e-01             Lachnospiraceae_[G.3] DESeq2 man. geoMeans (ds2)
## 41 7.446160e-01             Lachnospiraceae_[G.7] DESeq2 man. geoMeans (ds2)
## 42 7.598131e-01             Lachnospiraceae_[G.8] DESeq2 man. geoMeans (ds2)
## 43 7.598131e-01                      Oribacterium DESeq2 man. geoMeans (ds2)
## 44 7.598131e-01                    Shuttleworthia DESeq2 man. geoMeans (ds2)
## 45 1.129065e-01                    Stomatobaculum DESeq2 man. geoMeans (ds2)
## 46 8.265852e-01      Family.Lachnospiraceae_[XIV]. DESeq2 man. geoMeans (ds2)
## 47 7.085122e-01                       Peptococcus DESeq2 man. geoMeans (ds2)
## 48 6.365815e-01                        Parvimonas DESeq2 man. geoMeans (ds2)
## 49 7.200315e-01                        Filifactor DESeq2 man. geoMeans (ds2)
## 50 7.446160e-01                      Mogibacterium DESeq2 man. geoMeans (ds2)
## 51 7.075734e-01    Peptostreptococcaceae_[XI][G.1] DESeq2 man. geoMeans (ds2)
## 52 5.573423e-01    Peptostreptococcaceae_[XI][G.4] DESeq2 man. geoMeans (ds2)
## 53 5.573423e-01    Peptostreptococcaceae_[XI][G.5] DESeq2 man. geoMeans (ds2)
## 54 7.390974e-01    Peptostreptococcaceae_[XI][G.6] DESeq2 man. geoMeans (ds2)
## 55 8.167908e-01    Peptostreptococcaceae_[XI][G.7] DESeq2 man. geoMeans (ds2)
## 56 1.845359e-01    Peptostreptococcaceae_[XI][G.9] DESeq2 man. geoMeans (ds2)
## 57 4.694614e-01               Peptostreptococcus DESeq2 man. geoMeans (ds2)
## 58 6.370729e-01             Ruminococcaceae_[G.1] DESeq2 man. geoMeans (ds2)
## 59 7.598131e-01             Ruminococcaceae_[G.2] DESeq2 man. geoMeans (ds2)
## 60 1.129065e-01                         Bulleidia DESeq2 man. geoMeans (ds2)
## 61 7.598131e-01                         Eggerthia DESeq2 man. geoMeans (ds2)
## 62 5.109412e-01                     Solobacterium DESeq2 man. geoMeans (ds2)
## 63 6.365815e-01                        Mycoplasma DESeq2 man. geoMeans (ds2)
## 64 7.598131e-01                       Mitsuokella DESeq2 man. geoMeans (ds2)
## 65 9.800761e-01                       Selenomonas DESeq2 man. geoMeans (ds2)
## 66 2.623725e-01          Family.Selenomonadaceae. DESeq2 man. geoMeans (ds2)
## 67 7.200315e-01             Class.Negativicutes. DESeq2 man. geoMeans (ds2)
## 68 9.620893e-01                      Anaeroglobus DESeq2 man. geoMeans (ds2)
## 69 7.200315e-01                         Dialister DESeq2 man. geoMeans (ds2)
## 70 6.350956e-01                       Megasphaera DESeq2 man. geoMeans (ds2)
## 71 9.607221e-01           Family.Veillonellaceae. DESeq2 man. geoMeans (ds2)
## 72 9.800761e-01                        Veillonella DESeq2 man. geoMeans (ds2)
## 73 4.694614e-01             Veillonellaceae_[G.1] DESeq2 man. geoMeans (ds2)
```

```
## 74 5.691671e-02                   Phylum.Firmicutes. DESeq2 man. geoMeans (ds2)
## 75 7.085122e-01                        Fusobacterium DESeq2 man. geoMeans (ds2)
## 76 6.624672e-01                         Leptotrichia DESeq2 man. geoMeans (ds2)
## 77 4.694614e-01         Gracilibacteria_(GN02)_[G.1] DESeq2 man. geoMeans (ds2)
## 78 9.800761e-01                            Lautropia DESeq2 man. geoMeans (ds2)
## 79 1.129065e-01                              Ottowia DESeq2 man. geoMeans (ds2)
## 80 8.220045e-01                            Eikenella DESeq2 man. geoMeans (ds2)
## 81 6.589446e-02                             Kingella DESeq2 man. geoMeans (ds2)
## 82 4.694614e-01                            Neisseria DESeq2 man. geoMeans (ds2)
## 83 5.581180e-01                Family.Neisseriaceae. DESeq2 man. geoMeans (ds2)
## 84 5.231035e-01                        Campylobacter DESeq2 man. geoMeans (ds2)
## 85 7.075734e-01                       Cardiobacterium DESeq2 man. geoMeans (ds2)
## 86 6.365815e-01                      Aggregatibacter DESeq2 man. geoMeans (ds2)
## 87 8.744456e-01                          Haemophilus DESeq2 man. geoMeans (ds2)
## 88 6.061403e-01              Family.Pasteurellaceae. DESeq2 man. geoMeans (ds2)
## 89 3.548734e-01         Saccharibacteria_(TM7)_[G.1] DESeq2 man. geoMeans (ds2)
## 90 7.390974e-01         Saccharibacteria_(TM7)_[G.3] DESeq2 man. geoMeans (ds2)
## 91 5.093351e-01         Saccharibacteria_(TM7)_[G.6] DESeq2 man. geoMeans (ds2)
## 92 9.607221e-01 Family.Saccharibacteria_(TM7)_[F-1]. DESeq2 man. geoMeans (ds2)
## 93 6.729197e-01         Saccharibacteria_(TM7)_[G.5] DESeq2 man. geoMeans (ds2)
## 94 1.159916e-01                            Treponema DESeq2 man. geoMeans (ds2)
## 95 4.017072e-01                        Fretibacterium DESeq2 man. geoMeans (ds2)
## 96 7.598131e-01                    Kingdom.Bacteria. DESeq2 man. geoMeans (ds2)
## 97 9.599827e-02                               Others DESeq2 man. geoMeans (ds2)
```

```
write.table(final, file="hdlc_cat_ds2.txt", sep="\t", dec=",", row.names=F)
write.table(final[final$pval<0.05,], file="hdlc_cat_ds2_sig.txt", sep="\t", dec=",", row.names=F)
final <- DA.kru(Microbio, predictor = Phe$hdlc_cat)
final
```

```
##                                             pval  pval.adj
## Absconditabacteria_(SR1)_[G.1]       0.382195342 0.6255290
## Actinomyces                          0.012533349 0.2632404
## Peptidiphaga                         0.514494209 0.7397107
## Schaalia                             0.050588290 0.3536738
## Family.Actinomycetaceae.             0.610256945 0.7831186
## Rothia                               0.310910938 0.6212952
## Alloscardovia                        0.196203522 0.5419285
## Bifidobacterium                      0.362424149 0.6255290
## Parascardovia                        0.126983740 0.4562008
## Scardovia                            0.169961187 0.5318140
## Corynebacterium                      0.039245217 0.3465801
## Olsenella                            0.622571211 0.7831186
## Atopobium                            0.490445050 0.7397107
## Slackia                              0.410788757 0.6426856
## Cryptobacterium                      0.539454697 0.7397107
## Bacteroidetes_[G.3]                  0.102651170 0.4562008
## Bacteroidetes_[G.5]                  0.070671910 0.3808431
## Family.Bacteroidetes_[F-1].          0.042012449 0.3465801
## Bacteroidaceae_[G.1]                 0.695860704 0.8231523
## Bacteroidales_[G.2]                  0.765100846 0.8471152
## Porphyromonas                        0.134261125 0.4651189
## Tannerella                           0.749007967 0.8448113
## Alloprevotella                       0.207098960 0.5419285
```

```
## Prevotella                              0.777250050 0.8471152
## Bergeyella                              0.262583919 0.5419285
## Capnocytophaga                          0.249382181 0.5419285
## Gemella                                 0.008237996 0.2632404
## Abiotrophia                             0.005823997 0.2632404
## Granulicatella                          0.928683997 0.9669430
## Lactobacillus                           0.344766627 0.6255290
## Streptococcus                           0.158217098 0.5115686
## Order.Lactobacillales.                  0.633128818 0.7831186
## Class.Bacilli.                          0.639925284 0.7831186
## Clostridiales_[F.1][G-1]                0.001975625 0.1916357
## Butyrivibrio                            0.946511426 0.9669430
## Catonella                               0.386477328 0.6255290
## Johnsonella                             0.212187868 0.5419285
## Lachnoanaerobaculum                     0.123051300 0.4562008
## Lachnospiraceae_[G.2]                   0.025430004 0.3083388
## Lachnospiraceae_[G.3]                   0.259084470 0.5419285
## Lachnospiraceae_[G.7]                   0.710371276 0.8285822
## Lachnospiraceae_[G.8]                   0.638918633 0.7831186
## Oribacterium                            0.393373882 0.6255290
## Shuttleworthia                          0.432806846 0.6663851
## Stomatobaculum                          0.068833918 0.3808431
## Family.Lachnospiraceae_[XIV].           0.572155145 0.7602609
## Peptococcus                             0.197127069 0.5419285
## Parvimonas                              0.856049204 0.9124920
## Filifactor                              0.254788329 0.5419285
## Mogibacterium                           0.645871017 0.7831186
## Peptostreptococcaceae_[XI][G.1]         0.386993018 0.6255290
## Peptostreptococcaceae_[XI][G.4]         0.153442427 0.5115686
## Peptostreptococcaceae_[XI][G.5]         0.041324983 0.3465801
## Peptostreptococcaceae_[XI][G.6]         0.516363553 0.7397107
## Peptostreptococcaceae_[XI][G.7]         0.517568349 0.7397107
## Peptostreptococcaceae_[XI][G.9]         0.214383900 0.5419285
## Peptostreptococcus                      0.114335822 0.4562008
## Ruminococcaceae_[G.1]                   0.190614025 0.5419285
## Ruminococcaceae_[G.2]                   0.964676273 0.9692079
## Bulleidia                               0.097371094 0.4562008
## Eggerthia                               0.080367347 0.4102965
## Solobacterium                           0.551870790 0.7434926
## Mycoplasma                              0.065066964 0.3808431
## Mitsuokella                             0.124301387 0.4562008
## Selenomonas                             0.361868839 0.6255290
## Family.Selenomonadaceae.                0.245930387 0.5419285
## Class.Negativicutes.                    0.886225785 0.9343902
## Anaeroglobus                            0.184948259 0.5419285
## Dialister                               0.722137664 0.8285822
## Megasphaera                             0.773047344 0.8471152
## Family.Veillonellaceae.                 0.947006078 0.9669430
## Veillonella                             0.238558581 0.5419285
## Veillonellaceae_[G.1]                   0.016282909 0.2632404
## Phylum.Firmicutes.                      0.104134201 0.4562008
## Fusobacterium                           0.375383658 0.6255290
## Leptotrichia                            0.385064570 0.6255290
## Gracilibacteria_(GN02)_[G.1]            0.232763364 0.5419285
```

```
## Lautropia                              0.726077222 0.8285822
## Ottowia                                0.054691827 0.3536738
## Eikenella                              0.529268915 0.7397107
## Kingella                               0.042875887 0.3465801
## Neisseria                              0.116048555 0.4562008
## Family.Neisseriaceae.                  0.345939218 0.6255290
## Campylobacter                          0.235566465 0.5419285
## Cardiobacterium                        0.582427548 0.7634523
## Aggregatibacter                        0.845818181 0.9116040
## Haemophilus                            0.662452088 0.7933068
## Family.Pasteurellaceae.                0.541437731 0.7397107
## Saccharibacteria_(TM7)_[G.1]           0.253544471 0.5419285
## Saccharibacteria_(TM7)_[G.3]           0.322784555 0.6255290
## Saccharibacteria_(TM7)_[G.6]           0.539922613 0.7397107
## Family.Saccharibacteria_(TM7)_[F-1].   0.969207911 0.9692079
## Saccharibacteria_(TM7)_[G.5]           0.054529914 0.3536738
## Treponema                              0.021500615 0.2979371
## Fretibacterium                         0.015662605 0.2632404
## Kingdom.Bacteria.                      0.330669151 0.6255290
## Others                                 0.313850158 0.6212952
##                                                                Feature
## Absconditabacteria_(SR1)_[G.1]               Absconditabacteria_(SR1)_[G.1]
## Actinomyces                                                Actinomyces
## Peptidiphaga                                              Peptidiphaga
## Schaalia                                                      Schaalia
## Family.Actinomycetaceae.                      Family.Actinomycetaceae.
## Rothia                                                          Rothia
## Alloscardovia                                            Alloscardovia
## Bifidobacterium                                        Bifidobacterium
## Parascardovia                                            Parascardovia
## Scardovia                                                    Scardovia
## Corynebacterium                                        Corynebacterium
## Olsenella                                                    Olsenella
## Atopobium                                                    Atopobium
## Slackia                                                        Slackia
## Cryptobacterium                                        Cryptobacterium
## Bacteroidetes_[G.3]                                Bacteroidetes_[G.3]
## Bacteroidetes_[G.5]                                Bacteroidetes_[G.5]
## Family.Bacteroidetes_[F-1].                Family.Bacteroidetes_[F-1].
## Bacteroidaceae_[G.1]                              Bacteroidaceae_[G.1]
## Bacteroidales_[G.2]                                Bacteroidales_[G.2]
## Porphyromonas                                            Porphyromonas
## Tannerella                                                  Tannerella
## Alloprevotella                                          Alloprevotella
## Prevotella                                                  Prevotella
## Bergeyella                                                  Bergeyella
## Capnocytophaga                                          Capnocytophaga
## Gemella                                                        Gemella
## Abiotrophia                                                Abiotrophia
## Granulicatella                                          Granulicatella
## Lactobacillus                                            Lactobacillus
## Streptococcus                                            Streptococcus
## Order.Lactobacillales.                          Order.Lactobacillales.
## Class.Bacilli.                                          Class.Bacilli.
```

```
## Clostridiales_[F.1][G-1]                         Clostridiales_[F.1][G-1]
## Butyrivibrio                                                 Butyrivibrio
## Catonella                                                       Catonella
## Johnsonella                                                   Johnsonella
## Lachnoanaerobaculum                                   Lachnoanaerobaculum
## Lachnospiraceae_[G.2]                               Lachnospiraceae_[G.2]
## Lachnospiraceae_[G.3]                               Lachnospiraceae_[G.3]
## Lachnospiraceae_[G.7]                               Lachnospiraceae_[G.7]
## Lachnospiraceae_[G.8]                               Lachnospiraceae_[G.8]
## Oribacterium                                                 Oribacterium
## Shuttleworthia                                             Shuttleworthia
## Stomatobaculum                                             Stomatobaculum
## Family.Lachnospiraceae_[XIV].               Family.Lachnospiraceae_[XIV].
## Peptococcus                                                   Peptococcus
## Parvimonas                                                     Parvimonas
## Filifactor                                                     Filifactor
## Mogibacterium                                               Mogibacterium
## Peptostreptococcaceae_[XI][G.1]           Peptostreptococcaceae_[XI][G.1]
## Peptostreptococcaceae_[XI][G.4]           Peptostreptococcaceae_[XI][G.4]
## Peptostreptococcaceae_[XI][G.5]           Peptostreptococcaceae_[XI][G.5]
## Peptostreptococcaceae_[XI][G.6]           Peptostreptococcaceae_[XI][G.6]
## Peptostreptococcaceae_[XI][G.7]           Peptostreptococcaceae_[XI][G.7]
## Peptostreptococcaceae_[XI][G.9]           Peptostreptococcaceae_[XI][G.9]
## Peptostreptococcus                                     Peptostreptococcus
## Ruminococcaceae_[G.1]                               Ruminococcaceae_[G.1]
## Ruminococcaceae_[G.2]                               Ruminococcaceae_[G.2]
## Bulleidia                                                       Bulleidia
## Eggerthia                                                       Eggerthia
## Solobacterium                                               Solobacterium
## Mycoplasma                                                     Mycoplasma
## Mitsuokella                                                   Mitsuokella
## Selenomonas                                                   Selenomonas
## Family.Selenomonadaceae.                         Family.Selenomonadaceae.
## Class.Negativicutes.                                 Class.Negativicutes.
## Anaeroglobus                                                 Anaeroglobus
## Dialister                                                       Dialister
## Megasphaera                                                   Megasphaera
## Family.Veillonellaceae.                           Family.Veillonellaceae.
## Veillonella                                                   Veillonella
## Veillonellaceae_[G.1]                               Veillonellaceae_[G.1]
## Phylum.Firmicutes.                                     Phylum.Firmicutes.
## Fusobacterium                                               Fusobacterium
## Leptotrichia                                                 Leptotrichia
## Gracilibacteria_(GN02)_[G.1]                 Gracilibacteria_(GN02)_[G.1]
## Lautropia                                                       Lautropia
## Ottowia                                                           Ottowia
## Eikenella                                                       Eikenella
## Kingella                                                         Kingella
## Neisseria                                                       Neisseria
## Family.Neisseriaceae.                               Family.Neisseriaceae.
## Campylobacter                                               Campylobacter
## Cardiobacterium                                           Cardiobacterium
## Aggregatibacter                                           Aggregatibacter
## Haemophilus                                                   Haemophilus
```

```
## Family.Pasteurellaceae.                         Family.Pasteurellaceae.
## Saccharibacteria_(TM7)_[G.1]               Saccharibacteria_(TM7)_[G.1]
## Saccharibacteria_(TM7)_[G.3]               Saccharibacteria_(TM7)_[G.3]
## Saccharibacteria_(TM7)_[G.6]               Saccharibacteria_(TM7)_[G.6]
## Family.Saccharibacteria_(TM7)_[F-1]. Family.Saccharibacteria_(TM7)_[F-1].
## Saccharibacteria_(TM7)_[G.5]               Saccharibacteria_(TM7)_[G.5]
## Treponema                                                       Treponema
## Fretibacterium                                             Fretibacterium
## Kingdom.Bacteria.                                       Kingdom.Bacteria.
## Others                                                             Others
##                                                     Method
## Absconditabacteria_(SR1)_[G.1]       Kruskal-Wallis (kru)
## Actinomyces                          Kruskal-Wallis (kru)
## Peptidiphaga                         Kruskal-Wallis (kru)
## Schaalia                             Kruskal-Wallis (kru)
## Family.Actinomycetaceae.             Kruskal-Wallis (kru)
## Rothia                               Kruskal-Wallis (kru)
## Alloscardovia                        Kruskal-Wallis (kru)
## Bifidobacterium                      Kruskal-Wallis (kru)
## Parascardovia                        Kruskal-Wallis (kru)
## Scardovia                            Kruskal-Wallis (kru)
## Corynebacterium                      Kruskal-Wallis (kru)
## Olsenella                            Kruskal-Wallis (kru)
## Atopobium                            Kruskal-Wallis (kru)
## Slackia                              Kruskal-Wallis (kru)
## Cryptobacterium                      Kruskal-Wallis (kru)
## Bacteroidetes_[G.3]                  Kruskal-Wallis (kru)
## Bacteroidetes_[G.5]                  Kruskal-Wallis (kru)
## Family.Bacteroidetes_[F-1].          Kruskal-Wallis (kru)
## Bacteroidaceae_[G.1]                 Kruskal-Wallis (kru)
## Bacteroidales_[G.2]                  Kruskal-Wallis (kru)
## Porphyromonas                        Kruskal-Wallis (kru)
## Tannerella                           Kruskal-Wallis (kru)
## Alloprevotella                       Kruskal-Wallis (kru)
## Prevotella                           Kruskal-Wallis (kru)
## Bergeyella                           Kruskal-Wallis (kru)
## Capnocytophaga                       Kruskal-Wallis (kru)
## Gemella                              Kruskal-Wallis (kru)
## Abiotrophia                          Kruskal-Wallis (kru)
## Granulicatella                       Kruskal-Wallis (kru)
## Lactobacillus                        Kruskal-Wallis (kru)
## Streptococcus                        Kruskal-Wallis (kru)
## Order.Lactobacillales.               Kruskal-Wallis (kru)
## Class.Bacilli.                       Kruskal-Wallis (kru)
## Clostridiales_[F.1][G-1]             Kruskal-Wallis (kru)
## Butyrivibrio                         Kruskal-Wallis (kru)
## Catonella                            Kruskal-Wallis (kru)
## Johnsonella                          Kruskal-Wallis (kru)
## Lachnoanaerobaculum                  Kruskal-Wallis (kru)
## Lachnospiraceae_[G.2]                Kruskal-Wallis (kru)
## Lachnospiraceae_[G.3]                Kruskal-Wallis (kru)
## Lachnospiraceae_[G.7]                Kruskal-Wallis (kru)
## Lachnospiraceae_[G.8]                Kruskal-Wallis (kru)
## Oribacterium                         Kruskal-Wallis (kru)
```

```
## Shuttleworthia                          Kruskal-Wallis (kru)
## Stomatobaculum                          Kruskal-Wallis (kru)
## Family.Lachnospiraceae_[XIV].           Kruskal-Wallis (kru)
## Peptococcus                             Kruskal-Wallis (kru)
## Parvimonas                              Kruskal-Wallis (kru)
## Filifactor                              Kruskal-Wallis (kru)
## Mogibacterium                           Kruskal-Wallis (kru)
## Peptostreptococcaceae_[XI][G.1]         Kruskal-Wallis (kru)
## Peptostreptococcaceae_[XI][G.4]         Kruskal-Wallis (kru)
## Peptostreptococcaceae_[XI][G.5]         Kruskal-Wallis (kru)
## Peptostreptococcaceae_[XI][G.6]         Kruskal-Wallis (kru)
## Peptostreptococcaceae_[XI][G.7]         Kruskal-Wallis (kru)
## Peptostreptococcaceae_[XI][G.9]         Kruskal-Wallis (kru)
## Peptostreptococcus                      Kruskal-Wallis (kru)
## Ruminococcaceae_[G.1]                   Kruskal-Wallis (kru)
## Ruminococcaceae_[G.2]                   Kruskal-Wallis (kru)
## Bulleidia                               Kruskal-Wallis (kru)
## Eggerthia                               Kruskal-Wallis (kru)
## Solobacterium                           Kruskal-Wallis (kru)
## Mycoplasma                              Kruskal-Wallis (kru)
## Mitsuokella                             Kruskal-Wallis (kru)
## Selenomonas                             Kruskal-Wallis (kru)
## Family.Selenomonadaceae.                Kruskal-Wallis (kru)
## Class.Negativicutes.                    Kruskal-Wallis (kru)
## Anaeroglobus                            Kruskal-Wallis (kru)
## Dialister                               Kruskal-Wallis (kru)
## Megasphaera                             Kruskal-Wallis (kru)
## Family.Veillonellaceae.                 Kruskal-Wallis (kru)
## Veillonella                             Kruskal-Wallis (kru)
## Veillonellaceae_[G.1]                   Kruskal-Wallis (kru)
## Phylum.Firmicutes.                      Kruskal-Wallis (kru)
## Fusobacterium                           Kruskal-Wallis (kru)
## Leptotrichia                            Kruskal-Wallis (kru)
## Gracilibacteria_(GN02)_[G.1]            Kruskal-Wallis (kru)
## Lautropia                               Kruskal-Wallis (kru)
## Ottowia                                 Kruskal-Wallis (kru)
## Eikenella                               Kruskal-Wallis (kru)
## Kingella                                Kruskal-Wallis (kru)
## Neisseria                               Kruskal-Wallis (kru)
## Family.Neisseriaceae.                   Kruskal-Wallis (kru)
## Campylobacter                           Kruskal-Wallis (kru)
## Cardiobacterium                         Kruskal-Wallis (kru)
## Aggregatibacter                         Kruskal-Wallis (kru)
## Haemophilus                             Kruskal-Wallis (kru)
## Family.Pasteurellaceae.                 Kruskal-Wallis (kru)
## Saccharibacteria_(TM7)_[G.1]            Kruskal-Wallis (kru)
## Saccharibacteria_(TM7)_[G.3]            Kruskal-Wallis (kru)
## Saccharibacteria_(TM7)_[G.6]            Kruskal-Wallis (kru)
## Family.Saccharibacteria_(TM7)_[F-1].    Kruskal-Wallis (kru)
## Saccharibacteria_(TM7)_[G.5]            Kruskal-Wallis (kru)
## Treponema                               Kruskal-Wallis (kru)
## Fretibacterium                          Kruskal-Wallis (kru)
## Kingdom.Bacteria.                       Kruskal-Wallis (kru)
## Others                                  Kruskal-Wallis (kru)
```

```r
write.table(final, file="hdlc_cat_kru.txt", sep="\t", dec=",", row.names=F)
write.table(final[final$pval<0.05,], file="hdlc_cat_kru_sig.txt", sep="\t", dec=",", row.names=F)
##################ldlc_cat
table(Phe$ldlc_cat, useNA="always")
```

```
##
## High  Low <NA>
##  469  275    2
```

```r
#Remove NA
Phe2<-Phe[complete.cases(Phe$ldlc_cat),]
#Also subset columns
Microbio2<-dplyr::select(Microbio, one_of(Phe2$IDX))
final <- DA.ds2(Microbio2, predictor = Phe2$ldlc_cat)
final
```

```
##       baseMean log2FoldChange      lfcSE         stat         pval ordering
## 1    66.0445757   -0.128669389 0.25569132 -0.503221582 6.148085e-01 High>Low
## 2   323.0317176   -0.020138978 0.08618295 -0.233677050 8.152357e-01 High>Low
## 3    21.7811803    0.060471538 0.12234744  0.494260741 6.211220e-01 Low>High
## 4   704.5633545    0.032263448 0.07072972  0.456151256 6.482812e-01 Low>High
## 5     5.4458433   -0.165561605 0.17702613 -0.935238224 3.496655e-01 High>Low
## 6  1938.7351255   -0.225531597 0.08276477 -2.724971155 6.430710e-03 High>Low
## 7    12.4609233    0.176788127 0.21831836  0.809772133 4.180712e-01 Low>High
## 8    11.3440662    0.042882564 0.24052823  0.178284954 8.584992e-01 Low>High
## 9     2.8077998    0.091127131 0.32865336  0.277274302 7.815695e-01 Low>High
## 10   12.5622376    0.337447888 0.20647390  1.634336776 1.021882e-01 Low>High
## 11   93.1311330   -0.051809336 0.09042055 -0.572981871 5.666570e-01 High>Low
## 12    1.5644014    0.248320126 0.19994785  1.241924441 2.142644e-01 Low>High
## 13  120.2246048    0.055580146 0.08297791  0.669818563 5.029735e-01 Low>High
## 14    1.3967877   -0.032550133 0.19175782 -0.169746056 8.652099e-01 High>Low
## 15    4.2956678    0.213078231 0.20621382  1.033287832 3.014692e-01 Low>High
## 16    6.6101938   -0.193086342 0.21798842 -0.885764219 3.757446e-01 High>Low
## 17    4.6885569   -0.154957433 0.23786119 -0.651461602 5.147486e-01 High>Low
## 18    0.8707417    0.731362509 0.33871562  2.159222882 3.083288e-02 Low>High
## 19    0.7949952    0.449431692 0.32066493  1.401561719 1.610462e-01 Low>High
## 20   12.1959098    0.133854074 0.12463426  1.073974956 2.828339e-01 Low>High
## 21  680.9133853   -0.170560224 0.11422727 -1.493165570 1.353939e-01 High>Low
## 22   22.0635322    0.167090705 0.08414277  1.985799835 4.705555e-02 Low>High
## 23  362.6939638    0.163223740 0.09018727  1.809831275 7.032196e-02 Low>High
## 24 3816.8071886   -0.056004679 0.05724778 -0.978285683 3.279330e-01 High>Low
## 25   72.5673327   -0.185575446 0.08876327 -2.090678304 3.655691e-02 High>Low
## 26  224.6567711   -0.090772553 0.09479821 -0.957534452 3.382976e-01 High>Low
## 27  308.2244574   -0.197531136 0.07902370 -2.499644360 1.243180e-02 High>Low
## 28   29.9968341   -0.031901344 0.17179884 -0.185690098 8.526878e-01 High>Low
## 29  305.2608842   -0.201765334 0.06873250 -2.935515649 3.329938e-03 High>Low
## 30   42.4530683   -1.439469858 0.28548818 -5.042134714 4.603670e-07 High>Low
## 31 3798.5213708   -0.230321534 0.06445394 -3.573428093 3.523380e-04 High>Low
## 32    0.2848918   -0.279109315 0.23484603 -1.188477869 2.346452e-01 High>Low
## 33    0.5736915   -0.241249967 0.18804552 -1.282933904 1.995152e-01 High>Low
## 34    1.0572350    0.112218665 0.28320759  0.396241735 6.919267e-01 Low>High
## 35    6.9558454    0.200596299 0.18074579  1.109825576 2.670742e-01 Low>High
## 36   36.8665586   -0.070709840 0.10171637 -0.695166759 4.869508e-01 High>Low
```

```
## 37    0.9414850    0.131080592 0.24548334  0.533969395 5.933627e-01 Low>High
## 38   87.8457640   -0.010281167 0.07145964 -0.143873772 8.856001e-01 High>Low
## 39   28.0239032   -0.243197628 0.18710040 -1.299824210 1.936612e-01 High>Low
## 40    2.8955555    0.378178330 0.19285729  1.960923151 4.988799e-02 Low>High
## 41    1.4735262    0.587455156 0.27509132  2.135491463 3.272089e-02 Low>High
## 42    1.4362326   -0.001642209 0.29579962 -0.005551761 9.955704e-01 High>Low
## 43  147.1655087   -0.097647942 0.07885721 -1.238288052 2.156093e-01 High>Low
## 44    2.8084688    0.047216556 0.21978762  0.214828096 8.299013e-01 Low>High
## 45  112.8061515   -0.194151672 0.08825842 -2.199808975 2.782045e-02 High>Low
## 46    1.5130671    0.343998637 0.24565250  1.400346559 1.614096e-01 Low>High
## 47    3.7130976    0.266105672 0.21987775  1.210243747 2.261854e-01 Low>High
## 48   40.9530628    0.087655420 0.10953106  0.800279095 4.235491e-01 Low>High
## 49    6.8569889    0.041445702 0.21377300  0.193877159 8.462721e-01 Low>High
## 50   41.0817411   -0.034227562 0.07534720 -0.454264575 6.496384e-01 High>Low
## 51   67.6330957   -0.044144527 0.13532735 -0.326205509 7.442689e-01 High>Low
## 52    0.4187964    0.301130222 0.28963683  1.039682081 2.984876e-01 Low>High
## 53    1.6676249    0.367709868 0.30611409  1.201218361 2.296665e-01 Low>High
## 54    1.0154112    0.046016907 0.30370280  0.151519533 8.795659e-01 Low>High
## 55    4.7226151    0.375054695 0.20980284  1.787653101 7.383199e-02 Low>High
## 56    8.7484940    0.171914592 0.12471383  1.378472590 1.680574e-01 Low>High
## 57   42.9164000    0.295375136 0.15799717  1.869496388 6.155379e-02 Low>High
## 58   25.1497754    0.171240021 0.16067717  1.065739618 2.865414e-01 Low>High
## 59   41.5610659    0.011255285 0.13335820  0.084398896 9.327393e-01 Low>High
## 60    3.8684545    0.069811127 0.23529842  0.296691859 7.667018e-01 Low>High
## 61    1.4585855    0.043880538 0.31016867  0.141473147 8.874962e-01 Low>High
## 62   74.1707405    0.072456814 0.08863232  0.817498780 4.136434e-01 Low>High
## 63    4.5015741   -0.045739371 0.18833578 -0.242860760 8.081133e-01 High>Low
## 64   26.9287868    0.169166606 0.16918896  0.999867859 3.173745e-01 Low>High
## 65  222.1718573    0.125003294 0.07593084  1.646278407 9.970645e-02 Low>High
## 66    5.0502749    0.198217998 0.19802903  1.000954254 3.168489e-01 Low>High
## 67    0.7392918   -0.152807409 0.18238203 -0.837842466 4.021192e-01 High>Low
## 68    8.7199297   -0.041879048 0.18227701 -0.229754963 8.182822e-01 High>Low
## 69   36.5108556    0.086163028 0.09321690  0.924328410 3.553154e-01 Low>High
## 70  408.7802795   -0.117220668 0.09795721 -1.196651814 2.314423e-01 High>Low
## 71    0.5263274   -0.037174150 0.21442332 -0.173368042 8.623621e-01 High>Low
## 72 5074.3878565   -0.102724788 0.05514233 -1.862902493 6.247599e-02 High>Low
## 73    6.1434722    0.158434684 0.15343748  1.032568335 3.018060e-01 Low>High
## 74    0.8921685   -0.073974960 0.25421417 -0.290994631 7.710554e-01 High>Low
## 75  928.1297092   -0.029108399 0.07364673 -0.395243612 6.926631e-01 High>Low
## 76  682.9726580    0.021197400 0.07601304  0.278865317 7.803482e-01 Low>High
## 77    2.4085097   -0.082495649 0.24578151 -0.335646281 7.371376e-01 High>Low
## 78   68.5122787   -0.067056460 0.14149339 -0.473919375 6.355574e-01 High>Low
## 79    2.3408346    0.179396103 0.31530477  0.568960955 5.693826e-01 Low>High
## 80    8.7934693    0.007475347 0.11789042  0.063409288 9.494406e-01 Low>High
## 81   99.7429243   -0.061911478 0.10177207 -0.608334653 5.429655e-01 High>Low
## 82 1329.5715834   -0.072090880 0.11632450 -0.619739415 5.354294e-01 High>Low
## 83    5.6904829   -0.311928791 0.26764709 -1.165448095 2.438377e-01 High>Low
## 84  258.1615451    0.025608397 0.06513914  0.393133781 6.942207e-01 Low>High
## 85   14.7787415   -0.059513746 0.10172960 -0.585018988 5.585349e-01 High>Low
## 86  272.9135897   -0.058662920 0.13375898 -0.438571833 6.609718e-01 High>Low
## 87 2906.6918990   -0.184370567 0.08941820 -2.061890926 3.921812e-02 High>Low
## 88    2.3985632    0.233343605 0.36702968  0.635762227 5.249314e-01 Low>High
## 89   36.1302206    0.487863495 0.14826930  3.290387751 1.000494e-03 Low>High
## 90    7.1279587    0.005958152 0.20817488  0.028620899 9.771669e-01 Low>High
```

```
## 91    7.2587743   -0.141335889 0.26465156 -0.534045183 5.933103e-01 High>Low
## 92    1.1034398    0.311393315 0.32783475  0.949848414 3.421893e-01 Low>High
## 93    0.9190726    0.273253192 0.30948395  0.882931709 3.772732e-01 Low>High
## 94   63.3599891    0.164850796 0.14124442  1.167131407 2.431573e-01 Low>High
## 95    8.2907574    0.182237626 0.17087748  1.066481230 2.862062e-01 Low>High
## 96    9.3988296    0.252916888 0.21625772  1.169516132 2.421957e-01 Low>High
## 97   33.2317101    0.259296567 0.14410776  1.799324139 7.196742e-02 Low>High
##         pval.adj                              Feature                     Method
## 1  0.8860123320       Absconditabacteria_(SR1)_[G.1] DESeq2 man. geoMeans (ds2)
## 2  0.9256680504                          Actinomyces DESeq2 man. geoMeans (ds2)
## 3  0.8860123320                         Peptidiphaga DESeq2 man. geoMeans (ds2)
## 4  0.8875341660                             Schaalia DESeq2 man. geoMeans (ds2)
## 5  0.7033793768             Family.Actinomycetaceae. DESeq2 man. geoMeans (ds2)
## 6  0.1247557836                               Rothia DESeq2 man. geoMeans (ds2)
## 7  0.7469866162                         Alloscardovia DESeq2 man. geoMeans (ds2)
## 8  0.9256680504                      Bifidobacterium DESeq2 man. geoMeans (ds2)
## 9  0.9256680504                        Parascardovia DESeq2 man. geoMeans (ds2)
## 10 0.4956125547                            Scardovia DESeq2 man. geoMeans (ds2)
## 11 0.8629705601                      Corynebacterium DESeq2 man. geoMeans (ds2)
## 12 0.6757786549                            Olsenella DESeq2 man. geoMeans (ds2)
## 13 0.8559372893                            Atopobium DESeq2 man. geoMeans (ds2)
## 14 0.9256680504                              Slackia DESeq2 man. geoMeans (ds2)
## 15 0.6970280345                      Cryptobacterium DESeq2 man. geoMeans (ds2)
## 16 0.7175587742                   Bacteroidetes_[G.3] DESeq2 man. geoMeans (ds2)
## 17 0.8608725855                   Bacteroidetes_[G.5] DESeq2 man. geoMeans (ds2)
## 18 0.3458325269         Family.Bacteroidetes_[F-1]. DESeq2 man. geoMeans (ds2)
## 19 0.6757786549                 Bacteroidaceae_[G.1] DESeq2 man. geoMeans (ds2)
## 20 0.6970280345                  Bacteroidales_[G.2] DESeq2 man. geoMeans (ds2)
## 21 0.6253906509                        Porphyromonas DESeq2 man. geoMeans (ds2)
## 22 0.3722411427                           Tannerella DESeq2 man. geoMeans (ds2)
## 23 0.3978724051                       Alloprevotella DESeq2 man. geoMeans (ds2)
## 24 0.7033793768                           Prevotella DESeq2 man. geoMeans (ds2)
## 25 0.3458325269                           Bergeyella DESeq2 man. geoMeans (ds2)
## 26 0.7033793768                       Capnocytophaga DESeq2 man. geoMeans (ds2)
## 27 0.2009808270                              Gemella DESeq2 man. geoMeans (ds2)
## 28 0.9256680504                          Abiotrophia DESeq2 man. geoMeans (ds2)
## 29 0.0807510001                       Granulicatella DESeq2 man. geoMeans (ds2)
## 30 0.0000446556                        Lactobacillus DESeq2 man. geoMeans (ds2)
## 31 0.0170883945                        Streptococcus DESeq2 man. geoMeans (ds2)
## 32 0.6757786549               Order.Lactobacillales. DESeq2 man. geoMeans (ds2)
## 33 0.6757786549                       Class.Bacilli. DESeq2 man. geoMeans (ds2)
## 34 0.8978587395            Clostridiales_[F.1][G-1] DESeq2 man. geoMeans (ds2)
## 35 0.6970280345                         Butyrivibrio DESeq2 man. geoMeans (ds2)
## 36 0.8434683530                            Catonella DESeq2 man. geoMeans (ds2)
## 37 0.8720633750                          Johnsonella DESeq2 man. geoMeans (ds2)
## 38 0.9256680504                  Lachnoanaerobaculum DESeq2 man. geoMeans (ds2)
## 39 0.6757786549                Lachnospiraceae_[G.2] DESeq2 man. geoMeans (ds2)
## 40 0.3722411427                Lachnospiraceae_[G.3] DESeq2 man. geoMeans (ds2)
## 41 0.3458325269                Lachnospiraceae_[G.7] DESeq2 man. geoMeans (ds2)
## 42 0.9955703581                Lachnospiraceae_[G.8] DESeq2 man. geoMeans (ds2)
## 43 0.6757786549                          Oribacterium DESeq2 man. geoMeans (ds2)
## 44 0.9256680504                        Shuttleworthia DESeq2 man. geoMeans (ds2)
## 45 0.3458325269                        Stomatobaculum DESeq2 man. geoMeans (ds2)
## 46 0.6757786549       Family.Lachnospiraceae_[XIV]. DESeq2 man. geoMeans (ds2)
```

```
## 47 0.6757786549                           Peptococcus DESeq2 man. geoMeans (ds2)
## 48 0.7469866162                           Parvimonas DESeq2 man. geoMeans (ds2)
## 49 0.9256680504                           Filifactor DESeq2 man. geoMeans (ds2)
## 50 0.8875341660                         Mogibacterium DESeq2 man. geoMeans (ds2)
## 51 0.9256680504      Peptostreptococcaceae_[XI][G.1] DESeq2 man. geoMeans (ds2)
## 52 0.6970280345      Peptostreptococcaceae_[XI][G.4] DESeq2 man. geoMeans (ds2)
## 53 0.6757786549      Peptostreptococcaceae_[XI][G.5] DESeq2 man. geoMeans (ds2)
## 54 0.9256680504      Peptostreptococcaceae_[XI][G.6] DESeq2 man. geoMeans (ds2)
## 55 0.3978724051      Peptostreptococcaceae_[XI][G.7] DESeq2 man. geoMeans (ds2)
## 56 0.6757786549      Peptostreptococcaceae_[XI][G.9] DESeq2 man. geoMeans (ds2)
## 57 0.3978724051                    Peptostreptococcus DESeq2 man. geoMeans (ds2)
## 58 0.6970280345                  Ruminococcaceae_[G.1] DESeq2 man. geoMeans (ds2)
## 59 0.9625075604                  Ruminococcaceae_[G.2] DESeq2 man. geoMeans (ds2)
## 60 0.9256680504                            Bulleidia DESeq2 man. geoMeans (ds2)
## 61 0.9256680504                            Eggerthia DESeq2 man. geoMeans (ds2)
## 62 0.7469866162                         Solobacterium DESeq2 man. geoMeans (ds2)
## 63 0.9256680504                           Mycoplasma DESeq2 man. geoMeans (ds2)
## 64 0.6996664245                          Mitsuokella DESeq2 man. geoMeans (ds2)
## 65 0.4956125547                          Selenomonas DESeq2 man. geoMeans (ds2)
## 66 0.6996664245              Family.Selenomonadaceae. DESeq2 man. geoMeans (ds2)
## 67 0.7469866162                 Class.Negativicutes. DESeq2 man. geoMeans (ds2)
## 68 0.9256680504                          Anaeroglobus DESeq2 man. geoMeans (ds2)
## 69 0.7033793768                            Dialister DESeq2 man. geoMeans (ds2)
## 70 0.6757786549                          Megasphaera DESeq2 man. geoMeans (ds2)
## 71 0.9256680504              Family.Veillonellaceae. DESeq2 man. geoMeans (ds2)
## 72 0.3978724051                           Veillonella DESeq2 man. geoMeans (ds2)
## 73 0.6970280345                 Veillonellaceae_[G.1] DESeq2 man. geoMeans (ds2)
## 74 0.9256680504                    Phylum.Firmicutes. DESeq2 man. geoMeans (ds2)
## 75 0.8978587395                        Fusobacterium DESeq2 man. geoMeans (ds2)
## 76 0.9256680504                          Leptotrichia DESeq2 man. geoMeans (ds2)
## 77 0.9256680504         Gracilibacteria_(GN02)_[G.1] DESeq2 man. geoMeans (ds2)
## 78 0.8875341660                            Lautropia DESeq2 man. geoMeans (ds2)
## 79 0.8629705601                              Ottowia DESeq2 man. geoMeans (ds2)
## 80 0.9694288140                             Eikenella DESeq2 man. geoMeans (ds2)
## 81 0.8629705601                             Kingella DESeq2 man. geoMeans (ds2)
## 82 0.8629705601                            Neisseria DESeq2 man. geoMeans (ds2)
## 83 0.6757786549                 Family.Neisseriaceae. DESeq2 man. geoMeans (ds2)
## 84 0.8978587395                         Campylobacter DESeq2 man. geoMeans (ds2)
## 85 0.8629705601                        Cardiobacterium DESeq2 man. geoMeans (ds2)
## 86 0.8904759114                       Aggregatibacter DESeq2 man. geoMeans (ds2)
## 87 0.3458325269                          Haemophilus DESeq2 man. geoMeans (ds2)
## 88 0.8629705601              Family.Pasteurellaceae. DESeq2 man. geoMeans (ds2)
## 89 0.0323493093         Saccharibacteria_(TM7)_[G.1] DESeq2 man. geoMeans (ds2)
## 90 0.9873457662         Saccharibacteria_(TM7)_[G.3] DESeq2 man. geoMeans (ds2)
## 91 0.8720633750         Saccharibacteria_(TM7)_[G.6] DESeq2 man. geoMeans (ds2)
## 92 0.7033793768 Family.Saccharibacteria_(TM7)_[F-1]. DESeq2 man. geoMeans (ds2)
## 93 0.7175587742         Saccharibacteria_(TM7)_[G.5] DESeq2 man. geoMeans (ds2)
## 94 0.6757786549                            Treponema DESeq2 man. geoMeans (ds2)
## 95 0.6970280345                         Fretibacterium DESeq2 man. geoMeans (ds2)
## 96 0.6757786549                      Kingdom.Bacteria. DESeq2 man. geoMeans (ds2)
## 97 0.3978724051                               Others DESeq2 man. geoMeans (ds2)
```

```
write.table(final, file="ldlc_cat_ds2.txt", sep="\t", dec=",", row.names=F)
write.table(final[final$pval<0.05,], file="ldlc_cat_ds2_sig.txt", sep="\t", dec=",", row.names=F)
```

```r
final <- DA.kru(Microbio2, predictor = Phe2$ldlc_cat)
final
```

```
##                                           pval  pval.adj
## Absconditabacteria_(SR1)_[G.1]       0.580346261 0.8278469
## Actinomyces                          0.047280402 0.3055302
## Peptidiphaga                         0.260490957 0.6983177
## Schaalia                             0.029978677 0.3055302
## Family.Actinomycetaceae.             0.784435988 0.9252387
## Rothia                               0.492194839 0.7957150
## Alloscardovia                        0.171548521 0.6349670
## Bifidobacterium                      0.073080064 0.3544383
## Parascardovia                        0.923810634 0.9737339
## Scardovia                            0.923076506 0.9737339
## Corynebacterium                      0.342239523 0.6983177
## Olsenella                            0.567571462 0.8278469
## Atopobium                            0.050690031 0.3055302
## Slackia                              0.968906621 0.9893047
## Cryptobacterium                      0.324676846 0.6983177
## Bacteroidetes_[G.3]                  0.884951946 0.9737339
## Bacteroidetes_[G.5]                  0.482801663 0.7937587
## Family.Bacteroidetes_[F-1].          0.002571695 0.1247272
## Bacteroidaceae_[G.1]                 0.005151954 0.1665799
## Bacteroidales_[G.2]                  0.448410412 0.7767109
## Porphyromonas                        0.810776224 0.9252387
## Tannerella                           0.042703909 0.3055302
## Alloprevotella                       0.163328298 0.6337138
## Prevotella                           0.808858814 0.9252387
## Bergeyella                           0.594700542 0.8360283
## Capnocytophaga                       0.770495008 0.9252387
## Gemella                              0.151097696 0.6337138
## Abiotrophia                          0.285710919 0.6983177
## Granulicatella                       0.578528020 0.8278469
## Lactobacillus                        0.336480238 0.6983177
## Streptococcus                        0.420087515 0.7612655
## Order.Lactobacillales.               0.044451553 0.3055302
## Class.Bacilli.                       0.459991471 0.7770096
## Clostridiales_[F.1][G-1]             0.509585482 0.8103245
## Butyrivibrio                         0.254145824 0.6983177
## Catonella                            0.722817783 0.9252387
## Johnsonella                          0.915856295 0.9737339
## Lachnoanaerobaculum                  0.157640326 0.6337138
## Lachnospiraceae_[G.2]                0.622768569 0.8508247
## Lachnospiraceae_[G.3]                0.252125661 0.6983177
## Lachnospiraceae_[G.7]                0.010240166 0.2483240
## Lachnospiraceae_[G.8]                0.346259854 0.6983177
## Oribacterium                         0.876989349 0.9737339
## Shuttleworthia                       0.363694758 0.7055678
## Stomatobaculum                       0.352758410 0.6983177
## Family.Lachnospiraceae_[XIV].        0.259467405 0.6983177
## Peptococcus                          0.319614339 0.6983177
## Parvimonas                           0.401978595 0.7607545
## Filifactor                           0.428963558 0.7612655
```

```
## Mogibacterium                          0.325623656 0.6983177
## Peptostreptococcaceae_[XI][G.1]        0.772780437 0.9252387
## Peptostreptococcaceae_[XI][G.4]        0.607987792 0.8424974
## Peptostreptococcaceae_[XI][G.5]        0.565076614 0.8278469
## Peptostreptococcaceae_[XI][G.6]        0.464603676 0.7770096
## Peptostreptococcaceae_[XI][G.7]        0.041319196 0.3055302
## Peptostreptococcaceae_[XI][G.9]        0.282010724 0.6983177
## Peptostreptococcus                     0.022846235 0.3055302
## Ruminococcaceae_[G.1]                  0.050790297 0.3055302
## Ruminococcaceae_[G.2]                  0.199737540 0.6919479
## Bulleidia                              0.333920919 0.6983177
## Eggerthia                              0.407827162 0.7607545
## Solobacterium                          0.086048771 0.3974634
## Mycoplasma                             0.895320594 0.9737339
## Mitsuokella                            0.035762285 0.3055302
## Selenomonas                            0.002362411 0.1247272
## Family.Selenomonadaceae.               0.317514389 0.6983177
## Class.Negativicutes.                   0.727606952 0.9252387
## Anaeroglobus                           0.065947033 0.3366770
## Dialister                              0.057614892 0.3104803
## Megasphaera                            0.790565814 0.9252387
## Family.Veillonellaceae.                0.670048329 0.8903382
## Veillonella                            0.536867534 0.8224294
## Veillonellaceae_[G.1]                  0.030535231 0.3055302
## Phylum.Firmicutes.                     0.796592913 0.9252387
## Fusobacterium                          0.300873394 0.6983177
## Leptotrichia                           0.319401347 0.6983177
## Gracilibacteria_(GN02)_[G.1]           0.735030925 0.9252387
## Lautropia                              0.993933685 0.9939337
## Ottowia                                0.338467954 0.6983177
## Eikenella                              0.933579926 0.9737339
## Kingella                               0.536283803 0.8224294
## Neisseria                              0.276316312 0.6983177
## Family.Neisseriaceae.                  0.431645388 0.7612655
## Campylobacter                          0.129280403 0.5700090
## Cardiobacterium                        0.806890366 0.9252387
## Aggregatibacter                        0.959268201 0.9893047
## Haemophilus                            0.176743403 0.6349670
## Family.Pasteurellaceae.                0.542633840 0.8224294
## Saccharibacteria_(TM7)_[G.1]           0.015764954 0.3055302
## Saccharibacteria_(TM7)_[G.3]           0.899451336 0.9737339
## Saccharibacteria_(TM7)_[G.6]           0.638428120 0.8601046
## Family.Saccharibacteria_(TM7)_[F-1]. 0.047823872 0.3055302
## Saccharibacteria_(TM7)_[G.5]           0.992926191 0.9939337
## Treponema                              0.265363814 0.6983177
## Fretibacterium                         0.342155029 0.6983177
## Kingdom.Bacteria.                      0.734747357 0.9252387
## Others                                 0.053546536 0.3055302
##                                                                Feature
## Absconditabacteria_(SR1)_[G.1]            Absconditabacteria_(SR1)_[G.1]
## Actinomyces                                                Actinomyces
## Peptidiphaga                                              Peptidiphaga
## Schaalia                                                      Schaalia
## Family.Actinomycetaceae.                      Family.Actinomycetaceae.
```

```
## Rothia                                                          Rothia
## Alloscardovia                                            Alloscardovia
## Bifidobacterium                                        Bifidobacterium
## Parascardovia                                            Parascardovia
## Scardovia                                                    Scardovia
## Corynebacterium                                        Corynebacterium
## Olsenella                                                    Olsenella
## Atopobium                                                    Atopobium
## Slackia                                                        Slackia
## Cryptobacterium                                        Cryptobacterium
## Bacteroidetes_[G.3]                                Bacteroidetes_[G.3]
## Bacteroidetes_[G.5]                                Bacteroidetes_[G.5]
## Family.Bacteroidetes_[F-1].                Family.Bacteroidetes_[F-1].
## Bacteroidaceae_[G.1]                              Bacteroidaceae_[G.1]
## Bacteroidales_[G.2]                                Bacteroidales_[G.2]
## Porphyromonas                                            Porphyromonas
## Tannerella                                                  Tannerella
## Alloprevotella                                          Alloprevotella
## Prevotella                                                  Prevotella
## Bergeyella                                                  Bergeyella
## Capnocytophaga                                          Capnocytophaga
## Gemella                                                        Gemella
## Abiotrophia                                                Abiotrophia
## Granulicatella                                          Granulicatella
## Lactobacillus                                            Lactobacillus
## Streptococcus                                            Streptococcus
## Order.Lactobacillales.                          Order.Lactobacillales.
## Class.Bacilli.                                          Class.Bacilli.
## Clostridiales_[F.1][G-1]                      Clostridiales_[F.1][G-1]
## Butyrivibrio                                              Butyrivibrio
## Catonella                                                    Catonella
## Johnsonella                                                Johnsonella
## Lachnoanaerobaculum                                Lachnoanaerobaculum
## Lachnospiraceae_[G.2]                            Lachnospiraceae_[G.2]
## Lachnospiraceae_[G.3]                            Lachnospiraceae_[G.3]
## Lachnospiraceae_[G.7]                            Lachnospiraceae_[G.7]
## Lachnospiraceae_[G.8]                            Lachnospiraceae_[G.8]
## Oribacterium                                              Oribacterium
## Shuttleworthia                                          Shuttleworthia
## Stomatobaculum                                          Stomatobaculum
## Family.Lachnospiraceae_[XIV].            Family.Lachnospiraceae_[XIV].
## Peptococcus                                                Peptococcus
## Parvimonas                                                  Parvimonas
## Filifactor                                                  Filifactor
## Mogibacterium                                            Mogibacterium
## Peptostreptococcaceae_[XI][G.1]        Peptostreptococcaceae_[XI][G.1]
## Peptostreptococcaceae_[XI][G.4]        Peptostreptococcaceae_[XI][G.4]
## Peptostreptococcaceae_[XI][G.5]        Peptostreptococcaceae_[XI][G.5]
## Peptostreptococcaceae_[XI][G.6]        Peptostreptococcaceae_[XI][G.6]
## Peptostreptococcaceae_[XI][G.7]        Peptostreptococcaceae_[XI][G.7]
## Peptostreptococcaceae_[XI][G.9]        Peptostreptococcaceae_[XI][G.9]
## Peptostreptococcus                                  Peptostreptococcus
## Ruminococcaceae_[G.1]                            Ruminococcaceae_[G.1]
## Ruminococcaceae_[G.2]                            Ruminococcaceae_[G.2]
```

```
## Bulleidia                                                     Bulleidia
## Eggerthia                                                     Eggerthia
## Solobacterium                                             Solobacterium
## Mycoplasma                                                   Mycoplasma
## Mitsuokella                                                 Mitsuokella
## Selenomonas                                                 Selenomonas
## Family.Selenomonadaceae.                       Family.Selenomonadaceae.
## Class.Negativicutes.                               Class.Negativicutes.
## Anaeroglobus                                               Anaeroglobus
## Dialister                                                     Dialister
## Megasphaera                                                 Megasphaera
## Family.Veillonellaceae.                         Family.Veillonellaceae.
## Veillonella                                                 Veillonella
## Veillonellaceae_[G.1]                             Veillonellaceae_[G.1]
## Phylum.Firmicutes.                                   Phylum.Firmicutes.
## Fusobacterium                                             Fusobacterium
## Leptotrichia                                               Leptotrichia
## Gracilibacteria_(GN02)_[G.1]               Gracilibacteria_(GN02)_[G.1]
## Lautropia                                                     Lautropia
## Ottowia                                                         Ottowia
## Eikenella                                                     Eikenella
## Kingella                                                       Kingella
## Neisseria                                                     Neisseria
## Family.Neisseriaceae.                             Family.Neisseriaceae.
## Campylobacter                                             Campylobacter
## Cardiobacterium                                         Cardiobacterium
## Aggregatibacter                                         Aggregatibacter
## Haemophilus                                                 Haemophilus
## Family.Pasteurellaceae.                         Family.Pasteurellaceae.
## Saccharibacteria_(TM7)_[G.1]               Saccharibacteria_(TM7)_[G.1]
## Saccharibacteria_(TM7)_[G.3]               Saccharibacteria_(TM7)_[G.3]
## Saccharibacteria_(TM7)_[G.6]               Saccharibacteria_(TM7)_[G.6]
## Family.Saccharibacteria_(TM7)_[F-1]. Family.Saccharibacteria_(TM7)_[F-1].
## Saccharibacteria_(TM7)_[G.5]               Saccharibacteria_(TM7)_[G.5]
## Treponema                                                     Treponema
## Fretibacterium                                           Fretibacterium
## Kingdom.Bacteria.                                     Kingdom.Bacteria.
## Others                                                           Others
##                                                    Method
## Absconditabacteria_(SR1)_[G.1]       Kruskal-Wallis (kru)
## Actinomyces                          Kruskal-Wallis (kru)
## Peptidiphaga                         Kruskal-Wallis (kru)
## Schaalia                             Kruskal-Wallis (kru)
## Family.Actinomycetaceae.             Kruskal-Wallis (kru)
## Rothia                               Kruskal-Wallis (kru)
## Alloscardovia                        Kruskal-Wallis (kru)
## Bifidobacterium                      Kruskal-Wallis (kru)
## Parascardovia                        Kruskal-Wallis (kru)
## Scardovia                            Kruskal-Wallis (kru)
## Corynebacterium                      Kruskal-Wallis (kru)
## Olsenella                            Kruskal-Wallis (kru)
## Atopobium                            Kruskal-Wallis (kru)
## Slackia                              Kruskal-Wallis (kru)
## Cryptobacterium                      Kruskal-Wallis (kru)
```

```
## Bacteroidetes_[G.3]                 Kruskal-Wallis (kru)
## Bacteroidetes_[G.5]                 Kruskal-Wallis (kru)
## Family.Bacteroidetes_[F-1].         Kruskal-Wallis (kru)
## Bacteroidaceae_[G.1]                Kruskal-Wallis (kru)
## Bacteroidales_[G.2]                 Kruskal-Wallis (kru)
## Porphyromonas                       Kruskal-Wallis (kru)
## Tannerella                          Kruskal-Wallis (kru)
## Alloprevotella                      Kruskal-Wallis (kru)
## Prevotella                          Kruskal-Wallis (kru)
## Bergeyella                          Kruskal-Wallis (kru)
## Capnocytophaga                      Kruskal-Wallis (kru)
## Gemella                             Kruskal-Wallis (kru)
## Abiotrophia                         Kruskal-Wallis (kru)
## Granulicatella                      Kruskal-Wallis (kru)
## Lactobacillus                       Kruskal-Wallis (kru)
## Streptococcus                       Kruskal-Wallis (kru)
## Order.Lactobacillales.              Kruskal-Wallis (kru)
## Class.Bacilli.                      Kruskal-Wallis (kru)
## Clostridiales_[F.1][G-1]            Kruskal-Wallis (kru)
## Butyrivibrio                        Kruskal-Wallis (kru)
## Catonella                           Kruskal-Wallis (kru)
## Johnsonella                         Kruskal-Wallis (kru)
## Lachnoanaerobaculum                 Kruskal-Wallis (kru)
## Lachnospiraceae_[G.2]               Kruskal-Wallis (kru)
## Lachnospiraceae_[G.3]               Kruskal-Wallis (kru)
## Lachnospiraceae_[G.7]               Kruskal-Wallis (kru)
## Lachnospiraceae_[G.8]               Kruskal-Wallis (kru)
## Oribacterium                        Kruskal-Wallis (kru)
## Shuttleworthia                      Kruskal-Wallis (kru)
## Stomatobaculum                      Kruskal-Wallis (kru)
## Family.Lachnospiraceae_[XIV].       Kruskal-Wallis (kru)
## Peptococcus                         Kruskal-Wallis (kru)
## Parvimonas                          Kruskal-Wallis (kru)
## Filifactor                          Kruskal-Wallis (kru)
## Mogibacterium                       Kruskal-Wallis (kru)
## Peptostreptococcaceae_[XI][G.1]     Kruskal-Wallis (kru)
## Peptostreptococcaceae_[XI][G.4]     Kruskal-Wallis (kru)
## Peptostreptococcaceae_[XI][G.5]     Kruskal-Wallis (kru)
## Peptostreptococcaceae_[XI][G.6]     Kruskal-Wallis (kru)
## Peptostreptococcaceae_[XI][G.7]     Kruskal-Wallis (kru)
## Peptostreptococcaceae_[XI][G.9]     Kruskal-Wallis (kru)
## Peptostreptococcus                  Kruskal-Wallis (kru)
## Ruminococcaceae_[G.1]               Kruskal-Wallis (kru)
## Ruminococcaceae_[G.2]               Kruskal-Wallis (kru)
## Bulleidia                           Kruskal-Wallis (kru)
## Eggerthia                           Kruskal-Wallis (kru)
## Solobacterium                       Kruskal-Wallis (kru)
## Mycoplasma                          Kruskal-Wallis (kru)
## Mitsuokella                         Kruskal-Wallis (kru)
## Selenomonas                         Kruskal-Wallis (kru)
## Family.Selenomonadaceae.            Kruskal-Wallis (kru)
## Class.Negativicutes.                Kruskal-Wallis (kru)
## Anaeroglobus                        Kruskal-Wallis (kru)
## Dialister                           Kruskal-Wallis (kru)
```

```
## Megasphaera                          Kruskal-Wallis (kru)
## Family.Veillonellaceae.              Kruskal-Wallis (kru)
## Veillonella                          Kruskal-Wallis (kru)
## Veillonellaceae_[G.1]                Kruskal-Wallis (kru)
## Phylum.Firmicutes.                   Kruskal-Wallis (kru)
## Fusobacterium                        Kruskal-Wallis (kru)
## Leptotrichia                         Kruskal-Wallis (kru)
## Gracilibacteria_(GN02)_[G.1]         Kruskal-Wallis (kru)
## Lautropia                            Kruskal-Wallis (kru)
## Ottowia                              Kruskal-Wallis (kru)
## Eikenella                            Kruskal-Wallis (kru)
## Kingella                             Kruskal-Wallis (kru)
## Neisseria                            Kruskal-Wallis (kru)
## Family.Neisseriaceae.                Kruskal-Wallis (kru)
## Campylobacter                        Kruskal-Wallis (kru)
## Cardiobacterium                      Kruskal-Wallis (kru)
## Aggregatibacter                      Kruskal-Wallis (kru)
## Haemophilus                          Kruskal-Wallis (kru)
## Family.Pasteurellaceae.              Kruskal-Wallis (kru)
## Saccharibacteria_(TM7)_[G.1]         Kruskal-Wallis (kru)
## Saccharibacteria_(TM7)_[G.3]         Kruskal-Wallis (kru)
## Saccharibacteria_(TM7)_[G.6]         Kruskal-Wallis (kru)
## Family.Saccharibacteria_(TM7)_[F-1]. Kruskal-Wallis (kru)
## Saccharibacteria_(TM7)_[G.5]         Kruskal-Wallis (kru)
## Treponema                            Kruskal-Wallis (kru)
## Fretibacterium                       Kruskal-Wallis (kru)
## Kingdom.Bacteria.                    Kruskal-Wallis (kru)
## Others                               Kruskal-Wallis (kru)
```

```
write.table(final, file="ldlc_cat_kru.txt", sep="\t", dec=",", row.names=F)
write.table(final[final$pval<0.05,], file="ldlc_cat_kru_sig.txt", sep="\t", dec=",", row.names=F)
##################act_cat
table(Phe$act_cat, useNA="always")
```

```
##
##    Low Medium   High   <NA>
##    100    149    453     44
```

```
#Remove NA
Phe2<-Phe[complete.cases(Phe$act_cat),]
#Also subset columns
Microbio2<-dplyr::select(Microbio, one_of(Phe2$IDX))
final <- DA.ds2(Microbio2, predictor = Phe2$act_cat, out.all=TRUE) #It is LRT so don't look at ordering
final
```

```
##                                        baseMean log2FoldChange      lfcSE
## Absconditabacteria_(SR1)_[G.1]       59.2896717   0.0404937823 0.42948129
## Actinomyces                         323.5372999  -0.3503539390 0.14666460
## Peptidiphaga                         22.0247906   0.1824717457 0.21103981
## Schaalia                            694.1804888   0.0729400456 0.12033012
## Family.Actinomycetaceae.              5.3605886   0.1045761742 0.30700903
## Rothia                             1964.8708227  -0.1329279419 0.14266374
## Alloscardovia                        12.6660233  -0.5823943214 0.37175554
```

```
## Bifidobacterium                         10.9006386  -0.6673084357 0.40516866
## Parascardovia                            2.8857587   0.0915910576 0.56902261
## Scardovia                               14.9816389  -0.2487290590 0.36405242
## Corynebacterium                         94.8694307   0.0635484560 0.15476843
## Olsenella                                1.5540156   0.0125308847 0.34194153
## Atopobium                              120.6321232  -0.1936167820 0.14258391
## Slackia                                  1.3799708  -0.0395150260 0.31689367
## Cryptobacterium                          4.1489578  -0.2954312323 0.35084150
## Bacteroidetes_[G.3]                      6.6173873  -0.3923711047 0.36835186
## Bacteroidetes_[G.5]                      4.6936020   0.1113627346 0.40224729
## Family.Bacteroidetes_[F-1].              0.8495843   0.2017062053 0.59293251
## Bacteroidaceae_[G.1]                     0.8369362  -1.2494974953 0.52195496
## Bacteroidales_[G.2]                     12.4001183  -0.0768874702 0.21080942
## Porphyromonas                          686.5785230   0.0370398259 0.19532475
## Tannerella                              22.3978584  -0.1606448326 0.14369247
## Alloprevotella                         358.8560588  -0.0455709704 0.15437877
## Prevotella                            3850.1486946  -0.0315150277 0.09670165
## Bergeyella                              73.7672269  -0.2763180926 0.15155773
## Capnocytophaga                         230.9964219   0.0718977268 0.15994200
## Gemella                                311.4195577  -0.1703360083 0.13549401
## Abiotrophia                             30.5449620   0.3513454179 0.29196671
## Granulicatella                         305.7679662  -0.0934582482 0.11548843
## Lactobacillus                           23.5100282  -1.5009896289 0.43277138
## Streptococcus                         3735.5172582  -0.2211533783 0.10363767
## Order.Lactobacillales.                   0.2691685   0.1872309676 0.40359083
## Class.Bacilli.                           0.5979507   0.0339565478 0.32643491
## Clostridiales_[F.1][G-1]                 1.0879229  -0.5200936074 0.47393794
## Butyrivibrio                             6.9446372   0.4101017484 0.31036146
## Catonella                               36.7239936   0.1331415930 0.17430333
## Johnsonella                              0.9633830  -0.1025074507 0.41258971
## Lachnoanaerobaculum                     88.1189353   0.2022515375 0.12256220
## Lachnospiraceae_[G.2]                   28.4599257   0.3507676131 0.31713097
## Lachnospiraceae_[G.3]                    2.9317741  -0.0684027010 0.33032838
## Lachnospiraceae_[G.7]                    1.5350251  -0.3205858839 0.46668863
## Lachnospiraceae_[G.8]                    1.4849793   0.1410073533 0.48927529
## Oribacterium                           147.4730576   0.2953799855 0.13454907
## Shuttleworthia                           2.8325997  -0.3068256668 0.37774951
## Stomatobaculum                         113.5082371   0.0242727429 0.15166247
## Family.Lachnospiraceae_[XIV].            1.6670018   0.6830418690 0.43111360
## Peptococcus                              3.7605589   0.1459986084 0.37497741
## Parvimonas                              40.9086352  -0.0586528763 0.18670998
## Filifactor                               6.9928668   0.0190683653 0.35928248
## Mogibacterium                           40.4464151  -0.0361387653 0.12911465
## Peptostreptococcaceae_[XI][G.1]         67.8043481   0.3131035459 0.23441875
## Peptostreptococcaceae_[XI][G.4]          0.4235131  -0.3937210345 0.49753736
## Peptostreptococcaceae_[XI][G.5]          1.7005424  -0.5205422676 0.51032222
## Peptostreptococcaceae_[XI][G.6]          1.0463602   0.1051226537 0.50425064
## Peptostreptococcaceae_[XI][G.7]          4.6769866   0.2534062256 0.35460553
## Peptostreptococcaceae_[XI][G.9]          8.7147190  -0.1371627781 0.21230693
## Peptostreptococcus                      41.7432064   0.2902523457 0.27012375
## Ruminococcaceae_[G.1]                   24.6406703   0.2887542999 0.27101478
## Ruminococcaceae_[G.2]                   41.4458778   0.3283177079 0.23109923
## Bulleidia                                3.9052615  -0.1921058341 0.39748285
## Eggerthia                                1.5761319   0.4294689429 0.54175604
```

```
## Solobacterium                           74.0948717   0.3519443592 0.15322486
## Mycoplasma                               4.5602238  -0.5932037200 0.31438613
## Mitsuokella                             27.1663632  -0.0145733853 0.28765494
## Selenomonas                            224.9189619   0.0928242699 0.12918206
## Family.Selenomonadaceae.                 5.0762835  -0.0590621688 0.34006790
## Class.Negativicutes.                     0.7673063   0.1516143514 0.30538249
## Anaeroglobus                             8.9032881  -1.1516683241 0.30315997
## Dialister                               37.1152722  -0.1057896616 0.15814694
## Megasphaera                            415.6955543   0.0183431066 0.16743676
## Family.Veillonellaceae.                  0.5374127   0.0370321086 0.35448696
## Veillonella                           5171.5475423  -0.1561474892 0.09326939
## Veillonellaceae_[G.1]                    6.2348176  -0.6375138180 0.25520522
## Phylum.Firmicutes.                       0.9355293  -0.0512281330 0.43723004
## Fusobacterium                          937.2458774   0.0180350660 0.12379375
## Leptotrichia                           699.8496266   0.1176526160 0.12793711
## Gracilibacteria_(GN02)_[G.1]             2.2978066   0.3735197113 0.41634363
## Lautropia                               69.3687842   0.1420789107 0.23945095
## Ottowia                                  2.2919185   0.4679426199 0.53987880
## Eikenella                                9.0561712  -0.0002192479 0.19948186
## Kingella                               101.6396776   0.0190528014 0.17359794
## Neisseria                             1360.8568451  -0.1172732705 0.19761618
## Family.Neisseriaceae.                    6.1227287   0.1339823004 0.45875455
## Campylobacter                          260.4218217   0.0052594180 0.11070590
## Cardiobacterium                         14.9664891  -0.0433627358 0.17383446
## Aggregatibacter                        275.0584538  -0.0242693377 0.22770002
## Haemophilus                           2923.5363226  -0.1526623277 0.15201932
## Family.Pasteurellaceae.                  2.4311586   0.1329006226 0.61455889
## Saccharibacteria_(TM7)_[G.1]            33.0235717   0.5683647806 0.24729294
## Saccharibacteria_(TM7)_[G.3]             6.5756943   0.8428061844 0.34776244
## Saccharibacteria_(TM7)_[G.6]             6.7788368   1.3055732600 0.44526629
## Family.Saccharibacteria_(TM7)_[F-1].     1.0430542   1.1102755602 0.57259552
## Saccharibacteria_(TM7)_[G.5]             1.0073737   0.1820993386 0.54160350
## Treponema                               64.1196242  -0.2600881244 0.23928909
## Fretibacterium                           8.4704518  -0.3859944695 0.28858792
## Kingdom.Bacteria.                        9.4767701   0.2123758111 0.36440962
## Others                                  34.1894400   0.0253478190 0.24710901
##                                              stat         pval   ordering
## Absconditabacteria_(SR1)_[G.1]         1.57378213 4.552580e-01 Medium>Low
## Actinomyces                            7.40700341 2.463710e-02 Low>Medium
## Peptidiphaga                           4.40902994 1.103040e-01 Medium>Low
## Schaalia                               1.12716001 5.691678e-01 Medium>Low
## Family.Actinomycetaceae.               1.59278613 4.509526e-01 Medium>Low
## Rothia                                 4.99841525 8.215007e-02 Low>Medium
## Alloscardovia                          4.68470608 9.610124e-02 Low>Medium
## Bifidobacterium                        2.90086310 2.344691e-01 Low>Medium
## Parascardovia                          0.71289017 7.001609e-01 Medium>Low
## Scardovia                              0.47195716 7.897976e-01 Low>Medium
## Corynebacterium                        0.82982739 6.603973e-01 Medium>Low
## Olsenella                              1.84809133 3.969100e-01 Medium>Low
## Atopobium                              2.04813062 3.591320e-01 Low>Medium
## Slackia                                3.90457644 1.419489e-01 Low>Medium
## Cryptobacterium                        2.46166226 2.920497e-01 Low>Medium
## Bacteroidetes_[G.3]                    1.19233179 5.509199e-01 Low>Medium
## Bacteroidetes_[G.5]                    2.37946684 3.043024e-01 Medium>Low
```

```
## Family.Bacteroidetes_[F-1].           0.26435028 8.761875e-01 Medium>Low
## Bacteroidaceae_[G.1]                 11.69270018 2.890430e-03 Low>Medium
## Bacteroidales_[G.2]                   2.29410940 3.175707e-01 Low>Medium
## Porphyromonas                         0.16477103 9.209169e-01 Medium>Low
## Tannerella                            1.99789693 3.682665e-01 Low>Medium
## Alloprevotella                        0.92930058 6.283548e-01 Low>Medium
## Prevotella                            0.13531094 9.345824e-01 Low>Medium
## Bergeyella                            3.63697357 1.622711e-01 Low>Medium
## Capnocytophaga                        0.20227289 9.038097e-01 Medium>Low
## Gemella                               2.46797663 2.911291e-01 Low>Medium
## Abiotrophia                           7.52890005 2.318036e-02 Medium>Low
## Granulicatella                        6.88140979 3.204209e-02 Low>Medium
## Lactobacillus                        23.72960785 7.033656e-06 Low>Medium
## Streptococcus                        11.19676767 3.703845e-03 Low>Medium
## Order.Lactobacillales.               -3.73267203 1.000000e+00 Medium>Low
## Class.Bacilli.                        6.83693708 3.276257e-02 Medium>Low
## Clostridiales_[F.1][G-1]              1.65053026 4.381188e-01 Low>Medium
## Butyrivibrio                          2.34757999 3.091929e-01 Medium>Low
## Catonella                             1.62024153 4.448043e-01 Medium>Low
## Johnsonella                           1.40333938 4.957569e-01 Low>Medium
## Lachnoanaerobaculum                   3.38006358 1.845137e-01 Medium>Low
## Lachnospiraceae_[G.2]                 6.58939392 3.707928e-02 Medium>Low
## Lachnospiraceae_[G.3]                 0.56457071 7.540585e-01 Low>Medium
## Lachnospiraceae_[G.7]                 0.68699550 7.092851e-01 Low>Medium
## Lachnospiraceae_[G.8]                 3.85624710 1.454208e-01 Medium>Low
## Oribacterium                          5.51337652 6.350172e-02 Medium>Low
## Shuttleworthia                        1.03092955 5.972230e-01 Low>Medium
## Stomatobaculum                        0.63390810 7.283642e-01 Medium>Low
## Family.Lachnospiraceae_[XIV].         5.99566747 4.989504e-02 Medium>Low
## Peptococcus                           0.64094305 7.258067e-01 Medium>Low
## Parvimonas                            0.91144921 6.339884e-01 Low>Medium
## Filifactor                            1.33173783 5.138269e-01 Medium>Low
## Mogibacterium                         0.29511234 8.628140e-01 Low>Medium
## Peptostreptococcaceae_[XI][G.1]       1.85993534 3.945665e-01 Medium>Low
## Peptostreptococcaceae_[XI][G.4]       3.20519511 2.013728e-01 Low>Medium
## Peptostreptococcaceae_[XI][G.5]       4.48276948 1.063112e-01 Low>Medium
## Peptostreptococcaceae_[XI][G.6]       2.72264644 2.563214e-01 Medium>Low
## Peptostreptococcaceae_[XI][G.7]       3.60468192 1.649124e-01 Medium>Low
## Peptostreptococcaceae_[XI][G.9]       2.93660811 2.303158e-01 Low>Medium
## Peptostreptococcus                    1.28348390 5.263747e-01 Medium>Low
## Ruminococcaceae_[G.1]                 1.12689883 5.692421e-01 Medium>Low
## Ruminococcaceae_[G.2]                 2.09320367 3.511289e-01 Medium>Low
## Bulleidia                             1.60125346 4.490474e-01 Low>Medium
## Eggerthia                             0.66026127 7.188298e-01 Medium>Low
## Solobacterium                         5.23033063 7.315569e-02 Medium>Low
## Mycoplasma                            8.53789291 1.399652e-02 Low>Medium
## Mitsuokella                           1.56051168 4.582887e-01 Low>Medium
## Selenomonas                           0.55564066 7.574329e-01 Medium>Low
## Family.Selenomonadaceae.              4.12427866 1.271816e-01 Low>Medium
## Class.Negativicutes.                  0.52785943 7.680275e-01 Medium>Low
## Anaeroglobus                         28.68006205 5.918391e-07 Low>Medium
## Dialister                             6.26699455 4.356517e-02 Low>Medium
## Megasphaera                           0.06188895 9.695294e-01 Medium>Low
## Family.Veillonellaceae.               0.76599246 6.818155e-01 Medium>Low
```

```
## Veillonella                              3.15014803 2.069922e-01 Low>Medium
## Veillonellaceae_[G.1]                   14.78686007 6.152819e-04 Low>Medium
## Phylum.Firmicutes.                        5.40167251 6.714934e-02 Low>Medium
## Fusobacterium                             1.75641192 4.155277e-01 Medium>Low
## Leptotrichia                              0.85821473 6.510900e-01 Medium>Low
## Gracilibacteria_(GN02)_[G.1]              1.08783656 5.804693e-01 Medium>Low
## Lautropia                                 7.44505712 2.417277e-02 Medium>Low
## Ottowia                                   1.61071898 4.469272e-01 Medium>Low
## Eikenella                                 1.56785242 4.566097e-01 Low>Medium
## Kingella                                  9.91093154 7.044798e-03 Medium>Low
## Neisseria                                 2.42779009 2.970380e-01 Low>Medium
## Family.Neisseriaceae.                     4.06910552 1.307389e-01 Medium>Low
## Campylobacter                             0.01881336 9.906374e-01 Medium>Low
## Cardiobacterium                          11.55613122 3.094696e-03 Low>Medium
## Aggregatibacter                           0.84971446 6.538631e-01 Low>Medium
## Haemophilus                               8.27480496 1.596427e-02 Low>Medium
## Family.Pasteurellaceae.                   1.15818073 5.604079e-01 Medium>Low
## Saccharibacteria_(TM7)_[G.1]              9.34918497 9.329326e-03 Medium>Low
## Saccharibacteria_(TM7)_[G.3]              7.53961647 2.305648e-02 Medium>Low
## Saccharibacteria_(TM7)_[G.6]              8.88301880 1.177815e-02 Medium>Low
## Family.Saccharibacteria_(TM7)_[F-1].  3.97524567 1.370208e-01 Medium>Low
## Saccharibacteria_(TM7)_[G.5]              0.20131744 9.042416e-01 Medium>Low
## Treponema                                 5.60079158 6.078600e-02 Low>Medium
## Fretibacterium                            3.85159438 1.457595e-01 Low>Medium
## Kingdom.Bacteria.                         6.01021087 4.953353e-02 Medium>Low
## Others                                    0.13116802 9.365204e-01 Medium>Low
##                                              pval.adj
## Absconditabacteria_(SR1)_[G.1]         7.056192e-01
## Actinomyces                            1.593199e-01
## Peptidiphaga                           3.689479e-01
## Schaalia                               7.888069e-01
## Family.Actinomycetaceae.               7.056192e-01
## Rothia                                 3.064829e-01
## Alloscardovia                          3.452526e-01
## Bifidobacterium                        5.415119e-01
## Parascardovia                          8.512208e-01
## Scardovia                              8.805789e-01
## Corynebacterium                        8.319290e-01
## Olsenella                              7.056192e-01
## Atopobium                              6.830550e-01
## Slackia                                4.039621e-01
## Cryptobacterium                        6.248273e-01
## Bacteroidetes_[G.3]                    7.888069e-01
## Bacteroidetes_[G.5]                    6.248273e-01
## Family.Bacteroidetes_[F-1].            9.549460e-01
## Bacteroidaceae_[G.1]                   5.987883e-02
## Bacteroidales_[G.2]                    6.286604e-01
## Porphyromonas                          9.664093e-01
## Tannerella                             6.869586e-01
## Alloprevotella                         8.310389e-01
## Prevotella                             9.664093e-01
## Bergeyella                             4.323379e-01
## Capnocytophaga                         9.638619e-01
## Gemella                                6.248273e-01
```

```
## Abiotrophia                          1.593199e-01
## Granulicatella                       1.869394e-01
## Lactobacillus                        3.411323e-04
## Streptococcus                        5.987883e-02
## Order.Lactobacillales.               1.000000e+00
## Class.Bacilli.                       1.869394e-01
## Clostridiales_[F.1][G-1]             7.056192e-01
## Butyrivibrio                         6.248273e-01
## Catonella                            7.056192e-01
## Johnsonella                          7.513815e-01
## Lachnoanaerobaculum                  4.709954e-01
## Lachnospiraceae_[G.2]                1.998161e-01
## Lachnospiraceae_[G.3]                8.643646e-01
## Lachnospiraceae_[G.7]                8.512208e-01
## Lachnospiraceae_[G.8]                4.039621e-01
## Oribacterium                         2.678116e-01
## Shuttleworthia                       8.045920e-01
## Stomatobaculum                       8.512208e-01
## Family.Lachnospiraceae_[XIV].        2.304676e-01
## Peptococcus                          8.512208e-01
## Parvimonas                           8.310389e-01
## Filifactor                           7.667878e-01
## Mogibacterium                        9.510563e-01
## Peptostreptococcaceae_[XI][G.1]      7.056192e-01
## Peptostreptococcaceae_[XI][G.4]      5.008502e-01
## Peptostreptococcaceae_[XI][G.5]      3.682923e-01
## Peptostreptococcaceae_[XI][G.6]      5.782134e-01
## Peptostreptococcaceae_[XI][G.7]      4.323379e-01
## Peptostreptococcaceae_[XI][G.9]      5.415119e-01
## Peptostreptococcus                   7.736113e-01
## Ruminococcaceae_[G.1]                7.888069e-01
## Ruminococcaceae_[G.2]                6.811901e-01
## Bulleidia                            7.056192e-01
## Eggerthia                            8.512208e-01
## Solobacterium                        2.838441e-01
## Mycoplasma                           1.357663e-01
## Mitsuokella                          7.056192e-01
## Selenomonas                          8.643646e-01
## Family.Selenomonadaceae.             4.039621e-01
## Class.Negativicutes.                 8.662636e-01
## Anaeroglobus                         5.740839e-05
## Dialister                            2.224117e-01
## Megasphaera                          9.899406e-01
## Family.Veillonellaceae.              8.478987e-01
## Veillonella                          5.019562e-01
## Veillonellaceae_[G.1]                1.989411e-02
## Phylum.Firmicutes.                   2.713952e-01
## Fusobacterium                        7.056192e-01
## Leptotrichia                         8.319290e-01
## Gracilibacteria_(GN02)_[G.1]         7.930356e-01
## Lautropia                            1.593199e-01
## Ottowia                              7.056192e-01
## Eikenella                            7.056192e-01
## Kingella                             9.762078e-02
```

```
## Neisseria                               6.248273e-01
## Family.Neisseriaceae.                   4.039621e-01
## Campylobacter                           1.000000e+00
## Cardiobacterium                         5.987883e-02
## Aggregatibacter                         8.319290e-01
## Haemophilus                             1.407758e-01
## Family.Pasteurellaceae.                 7.888069e-01
## Saccharibacteria_(TM7)_[G.1]            1.131181e-01
## Saccharibacteria_(TM7)_[G.3]            1.593199e-01
## Saccharibacteria_(TM7)_[G.6]            1.269423e-01
## Family.Saccharibacteria_(TM7)_[F-1]. 4.039621e-01
## Saccharibacteria_(TM7)_[G.5]            9.638619e-01
## Treponema                               2.678116e-01
## Fretibacterium                          4.039621e-01
## Kingdom.Bacteria.                       2.304676e-01
## Others                                  9.664093e-01
##                                                               Feature
## Absconditabacteria_(SR1)_[G.1]         Absconditabacteria_(SR1)_[G.1]
## Actinomyces                                               Actinomyces
## Peptidiphaga                                             Peptidiphaga
## Schaalia                                                     Schaalia
## Family.Actinomycetaceae.                     Family.Actinomycetaceae.
## Rothia                                                         Rothia
## Alloscardovia                                           Alloscardovia
## Bifidobacterium                                       Bifidobacterium
## Parascardovia                                           Parascardovia
## Scardovia                                                   Scardovia
## Corynebacterium                                       Corynebacterium
## Olsenella                                                   Olsenella
## Atopobium                                                   Atopobium
## Slackia                                                       Slackia
## Cryptobacterium                                       Cryptobacterium
## Bacteroidetes_[G.3]                               Bacteroidetes_[G.3]
## Bacteroidetes_[G.5]                               Bacteroidetes_[G.5]
## Family.Bacteroidetes_[F-1].                 Family.Bacteroidetes_[F-1].
## Bacteroidaceae_[G.1]                             Bacteroidaceae_[G.1]
## Bacteroidales_[G.2]                               Bacteroidales_[G.2]
## Porphyromonas                                           Porphyromonas
## Tannerella                                                 Tannerella
## Alloprevotella                                         Alloprevotella
## Prevotella                                                 Prevotella
## Bergeyella                                                 Bergeyella
## Capnocytophaga                                         Capnocytophaga
## Gemella                                                       Gemella
## Abiotrophia                                               Abiotrophia
## Granulicatella                                         Granulicatella
## Lactobacillus                                           Lactobacillus
## Streptococcus                                           Streptococcus
## Order.Lactobacillales.                         Order.Lactobacillales.
## Class.Bacilli.                                         Class.Bacilli.
## Clostridiales_[F.1][G-1]                     Clostridiales_[F.1][G-1]
## Butyrivibrio                                             Butyrivibrio
## Catonella                                                   Catonella
## Johnsonella                                               Johnsonella
```

```
## Lachnoanaerobaculum                                          Lachnoanaerobaculum
## Lachnospiraceae_[G.2]                                      Lachnospiraceae_[G.2]
## Lachnospiraceae_[G.3]                                      Lachnospiraceae_[G.3]
## Lachnospiraceae_[G.7]                                      Lachnospiraceae_[G.7]
## Lachnospiraceae_[G.8]                                      Lachnospiraceae_[G.8]
## Oribacterium                                                        Oribacterium
## Shuttleworthia                                                    Shuttleworthia
## Stomatobaculum                                                    Stomatobaculum
## Family.Lachnospiraceae_[XIV].                      Family.Lachnospiraceae_[XIV].
## Peptococcus                                                          Peptococcus
## Parvimonas                                                            Parvimonas
## Filifactor                                                            Filifactor
## Mogibacterium                                                      Mogibacterium
## Peptostreptococcaceae_[XI][G.1]          Peptostreptococcaceae_[XI][G.1]
## Peptostreptococcaceae_[XI][G.4]          Peptostreptococcaceae_[XI][G.4]
## Peptostreptococcaceae_[XI][G.5]          Peptostreptococcaceae_[XI][G.5]
## Peptostreptococcaceae_[XI][G.6]          Peptostreptococcaceae_[XI][G.6]
## Peptostreptococcaceae_[XI][G.7]          Peptostreptococcaceae_[XI][G.7]
## Peptostreptococcaceae_[XI][G.9]          Peptostreptococcaceae_[XI][G.9]
## Peptostreptococcus                                          Peptostreptococcus
## Ruminococcaceae_[G.1]                                      Ruminococcaceae_[G.1]
## Ruminococcaceae_[G.2]                                      Ruminococcaceae_[G.2]
## Bulleidia                                                              Bulleidia
## Eggerthia                                                              Eggerthia
## Solobacterium                                                      Solobacterium
## Mycoplasma                                                            Mycoplasma
## Mitsuokella                                                          Mitsuokella
## Selenomonas                                                          Selenomonas
## Family.Selenomonadaceae.                                Family.Selenomonadaceae.
## Class.Negativicutes.                                        Class.Negativicutes.
## Anaeroglobus                                                        Anaeroglobus
## Dialister                                                              Dialister
## Megasphaera                                                          Megasphaera
## Family.Veillonellaceae.                                  Family.Veillonellaceae.
## Veillonella                                                          Veillonella
## Veillonellaceae_[G.1]                                      Veillonellaceae_[G.1]
## Phylum.Firmicutes.                                            Phylum.Firmicutes.
## Fusobacterium                                                      Fusobacterium
## Leptotrichia                                                        Leptotrichia
## Gracilibacteria_(GN02)_[G.1]                    Gracilibacteria_(GN02)_[G.1]
## Lautropia                                                              Lautropia
## Ottowia                                                                  Ottowia
## Eikenella                                                              Eikenella
## Kingella                                                                Kingella
## Neisseria                                                              Neisseria
## Family.Neisseriaceae.                                      Family.Neisseriaceae.
## Campylobacter                                                      Campylobacter
## Cardiobacterium                                                  Cardiobacterium
## Aggregatibacter                                                  Aggregatibacter
## Haemophilus                                                          Haemophilus
## Family.Pasteurellaceae.                                  Family.Pasteurellaceae.
## Saccharibacteria_(TM7)_[G.1]                    Saccharibacteria_(TM7)_[G.1]
## Saccharibacteria_(TM7)_[G.3]                    Saccharibacteria_(TM7)_[G.3]
## Saccharibacteria_(TM7)_[G.6]                    Saccharibacteria_(TM7)_[G.6]
```

```
## Family.Saccharibacteria_(TM7)_[F-1]. Family.Saccharibacteria_(TM7)_[F-1].
## Saccharibacteria_(TM7)_[G.5]                Saccharibacteria_(TM7)_[G.5]
## Treponema                                                        Treponema
## Fretibacterium                                              Fretibacterium
## Kingdom.Bacteria.                                        Kingdom.Bacteria.
## Others                                                              Others
##                                                    Method
## Absconditabacteria_(SR1)_[G.1]       DESeq2 man. geoMeans (ds2)
## Actinomyces                          DESeq2 man. geoMeans (ds2)
## Peptidiphaga                         DESeq2 man. geoMeans (ds2)
## Schaalia                             DESeq2 man. geoMeans (ds2)
## Family.Actinomycetaceae.             DESeq2 man. geoMeans (ds2)
## Rothia                               DESeq2 man. geoMeans (ds2)
## Alloscardovia                        DESeq2 man. geoMeans (ds2)
## Bifidobacterium                      DESeq2 man. geoMeans (ds2)
## Parascardovia                        DESeq2 man. geoMeans (ds2)
## Scardovia                            DESeq2 man. geoMeans (ds2)
## Corynebacterium                      DESeq2 man. geoMeans (ds2)
## Olsenella                            DESeq2 man. geoMeans (ds2)
## Atopobium                            DESeq2 man. geoMeans (ds2)
## Slackia                              DESeq2 man. geoMeans (ds2)
## Cryptobacterium                      DESeq2 man. geoMeans (ds2)
## Bacteroidetes_[G.3]                  DESeq2 man. geoMeans (ds2)
## Bacteroidetes_[G.5]                  DESeq2 man. geoMeans (ds2)
## Family.Bacteroidetes_[F-1].          DESeq2 man. geoMeans (ds2)
## Bacteroidaceae_[G.1]                 DESeq2 man. geoMeans (ds2)
## Bacteroidales_[G.2]                  DESeq2 man. geoMeans (ds2)
## Porphyromonas                        DESeq2 man. geoMeans (ds2)
## Tannerella                           DESeq2 man. geoMeans (ds2)
## Alloprevotella                       DESeq2 man. geoMeans (ds2)
## Prevotella                           DESeq2 man. geoMeans (ds2)
## Bergeyella                           DESeq2 man. geoMeans (ds2)
## Capnocytophaga                       DESeq2 man. geoMeans (ds2)
## Gemella                              DESeq2 man. geoMeans (ds2)
## Abiotrophia                          DESeq2 man. geoMeans (ds2)
## Granulicatella                       DESeq2 man. geoMeans (ds2)
## Lactobacillus                        DESeq2 man. geoMeans (ds2)
## Streptococcus                        DESeq2 man. geoMeans (ds2)
## Order.Lactobacillales.               DESeq2 man. geoMeans (ds2)
## Class.Bacilli.                       DESeq2 man. geoMeans (ds2)
## Clostridiales_[F.1][G-1]             DESeq2 man. geoMeans (ds2)
## Butyrivibrio                         DESeq2 man. geoMeans (ds2)
## Catonella                            DESeq2 man. geoMeans (ds2)
## Johnsonella                          DESeq2 man. geoMeans (ds2)
## Lachnoanaerobaculum                  DESeq2 man. geoMeans (ds2)
## Lachnospiraceae_[G.2]                DESeq2 man. geoMeans (ds2)
## Lachnospiraceae_[G.3]                DESeq2 man. geoMeans (ds2)
## Lachnospiraceae_[G.7]                DESeq2 man. geoMeans (ds2)
## Lachnospiraceae_[G.8]                DESeq2 man. geoMeans (ds2)
## Oribacterium                         DESeq2 man. geoMeans (ds2)
## Shuttleworthia                       DESeq2 man. geoMeans (ds2)
## Stomatobaculum                       DESeq2 man. geoMeans (ds2)
## Family.Lachnospiraceae_[XIV].        DESeq2 man. geoMeans (ds2)
## Peptococcus                          DESeq2 man. geoMeans (ds2)
```

```
## Parvimonas                              DESeq2 man. geoMeans (ds2)
## Filifactor                              DESeq2 man. geoMeans (ds2)
## Mogibacterium                           DESeq2 man. geoMeans (ds2)
## Peptostreptococcaceae_[XI][G.1]         DESeq2 man. geoMeans (ds2)
## Peptostreptococcaceae_[XI][G.4]         DESeq2 man. geoMeans (ds2)
## Peptostreptococcaceae_[XI][G.5]         DESeq2 man. geoMeans (ds2)
## Peptostreptococcaceae_[XI][G.6]         DESeq2 man. geoMeans (ds2)
## Peptostreptococcaceae_[XI][G.7]         DESeq2 man. geoMeans (ds2)
## Peptostreptococcaceae_[XI][G.9]         DESeq2 man. geoMeans (ds2)
## Peptostreptococcus                      DESeq2 man. geoMeans (ds2)
## Ruminococcaceae_[G.1]                   DESeq2 man. geoMeans (ds2)
## Ruminococcaceae_[G.2]                   DESeq2 man. geoMeans (ds2)
## Bulleidia                               DESeq2 man. geoMeans (ds2)
## Eggerthia                               DESeq2 man. geoMeans (ds2)
## Solobacterium                           DESeq2 man. geoMeans (ds2)
## Mycoplasma                              DESeq2 man. geoMeans (ds2)
## Mitsuokella                             DESeq2 man. geoMeans (ds2)
## Selenomonas                             DESeq2 man. geoMeans (ds2)
## Family.Selenomonadaceae.                DESeq2 man. geoMeans (ds2)
## Class.Negativicutes.                    DESeq2 man. geoMeans (ds2)
## Anaeroglobus                            DESeq2 man. geoMeans (ds2)
## Dialister                               DESeq2 man. geoMeans (ds2)
## Megasphaera                             DESeq2 man. geoMeans (ds2)
## Family.Veillonellaceae.                 DESeq2 man. geoMeans (ds2)
## Veillonella                             DESeq2 man. geoMeans (ds2)
## Veillonellaceae_[G.1]                   DESeq2 man. geoMeans (ds2)
## Phylum.Firmicutes.                      DESeq2 man. geoMeans (ds2)
## Fusobacterium                           DESeq2 man. geoMeans (ds2)
## Leptotrichia                            DESeq2 man. geoMeans (ds2)
## Gracilibacteria_(GN02)_[G.1]            DESeq2 man. geoMeans (ds2)
## Lautropia                               DESeq2 man. geoMeans (ds2)
## Ottowia                                 DESeq2 man. geoMeans (ds2)
## Eikenella                               DESeq2 man. geoMeans (ds2)
## Kingella                                DESeq2 man. geoMeans (ds2)
## Neisseria                               DESeq2 man. geoMeans (ds2)
## Family.Neisseriaceae.                   DESeq2 man. geoMeans (ds2)
## Campylobacter                           DESeq2 man. geoMeans (ds2)
## Cardiobacterium                         DESeq2 man. geoMeans (ds2)
## Aggregatibacter                         DESeq2 man. geoMeans (ds2)
## Haemophilus                             DESeq2 man. geoMeans (ds2)
## Family.Pasteurellaceae.                 DESeq2 man. geoMeans (ds2)
## Saccharibacteria_(TM7)_[G.1]            DESeq2 man. geoMeans (ds2)
## Saccharibacteria_(TM7)_[G.3]            DESeq2 man. geoMeans (ds2)
## Saccharibacteria_(TM7)_[G.6]            DESeq2 man. geoMeans (ds2)
## Family.Saccharibacteria_(TM7)_[F-1]. DESeq2 man. geoMeans (ds2)
## Saccharibacteria_(TM7)_[G.5]            DESeq2 man. geoMeans (ds2)
## Treponema                               DESeq2 man. geoMeans (ds2)
## Fretibacterium                          DESeq2 man. geoMeans (ds2)
## Kingdom.Bacteria.                       DESeq2 man. geoMeans (ds2)
## Others                                  DESeq2 man. geoMeans (ds2)
```

```
write.table(final, file="act_cat_ds2.txt", sep="\t", dec=",", row.names=F)
write.table(final[final$pval<0.05,], file="act_cat_ds2_sig.txt", sep="\t", dec=",", row.names=F)
final <- DA.kru(Microbio2, predictor = Phe2$act_cat)
```

```
final
```

```
##                                          pval   pval.adj
## Absconditabacteria_(SR1)_[G.1]       0.485141430 0.69579213
## Actinomyces                          0.919615515 0.94896495
## Peptidiphaga                         0.501509228 0.70502022
## Schaalia                             0.263990055 0.58105479
## Family.Actinomycetaceae.             0.487771805 0.69579213
## Rothia                               0.580933756 0.73801220
## Alloscardovia                        0.444903560 0.67430696
## Bifidobacterium                      0.702587907 0.81132175
## Parascardovia                        0.111291602 0.48267244
## Scardovia                            0.184139509 0.50305345
## Corynebacterium                      0.787969900 0.84925645
## Olsenella                            0.235109872 0.56481172
## Atopobium                            0.937620407 0.95330027
## Slackia                              0.129376117 0.48267244
## Cryptobacterium                      0.541271216 0.72829188
## Bacteroidetes_[G.3]                  0.360214650 0.67430696
## Bacteroidetes_[G.5]                  0.400568724 0.67430696
## Family.Bacteroidetes_[F-1].          0.366041446 0.67430696
## Bacteroidaceae_[G.1]                 0.006490391 0.13694040
## Bacteroidales_[G.2]                  0.072916268 0.47152520
## Porphyromonas                        0.661519212 0.77510723
## Tannerella                           0.202715017 0.53144207
## Alloprevotella                       0.110159870 0.48267244
## Prevotella                           0.462879788 0.69075907
## Bergeyella                           0.759841968 0.84546573
## Capnocytophaga                       0.767020459 0.84546573
## Gemella                              0.898144695 0.93677457
## Abiotrophia                          0.125484353 0.48267244
## Granulicatella                       0.598852100 0.73801220
## Lactobacillus                        0.736340263 0.83052332
## Streptococcus                        0.307675293 0.60907150
## Order.Lactobacillales.               0.646856339 0.77463043
## Class.Bacilli.                       0.421868130 0.67430696
## Clostridiales_[F.1][G-1]             0.536852205 0.72829188
## Butyrivibrio                         0.272289397 0.58105479
## Catonella                            0.178317072 0.50305345
## Johnsonella                          0.178661231 0.50305345
## Lachnoanaerobaculum                  0.051309610 0.47152520
## Lachnospiraceae_[G.2]                0.071141774 0.47152520
## Lachnospiraceae_[G.3]                0.556996952 0.73011763
## Lachnospiraceae_[G.7]                0.437767704 0.67430696
## Lachnospiraceae_[G.8]                0.164736585 0.50305345
## Oribacterium                         0.043764623 0.47152520
## Shuttleworthia                       0.155707815 0.50305345
## Stomatobaculum                       0.538932392 0.72829188
## Family.Lachnospiraceae_[XIV].        0.296892081 0.59996941
## Peptococcus                          0.943472434 0.95330027
## Parvimonas                           0.098339140 0.48267244
## Filifactor                           0.611352836 0.74126531
## Mogibacterium                        0.820528964 0.87462977
## Peptostreptococcaceae_[XI][G.1]      0.435962828 0.67430696
```

```
## Peptostreptococcaceae_[XI][G.4]      0.275551758 0.58105479
## Peptostreptococcaceae_[XI][G.5]      0.142557294 0.50305345
## Peptostreptococcaceae_[XI][G.6]      0.163296793 0.50305345
## Peptostreptococcaceae_[XI][G.7]      0.210305683 0.53683293
## Peptostreptococcaceae_[XI][G.9]      0.089856082 0.48267244
## Peptostreptococcus                   0.395106938 0.67430696
## Ruminococcaceae_[G.1]                0.151976813 0.50305345
## Ruminococcaceae_[G.2]                0.119830955 0.48267244
## Bulleidia                            0.372610966 0.67430696
## Eggerthia                            0.082594077 0.48267244
## Solobacterium                        0.072189476 0.47152520
## Mycoplasma                           0.001019721 0.09891294
## Mitsuokella                          0.971103977 0.97110398
## Selenomonas                          0.595928733 0.73801220
## Family.Selenomonadaceae.             0.119043174 0.48267244
## Class.Negativicutes.                 0.365040054 0.67430696
## Anaeroglobus                         0.003078794 0.09954766
## Dialister                            0.002950080 0.09954766
## Megasphaera                          0.429655613 0.67430696
## Family.Veillonellaceae.              0.233871689 0.56481172
## Veillonella                          0.871478743 0.91884172
## Veillonellaceae_[G.1]                0.009555736 0.15448440
## Phylum.Firmicutes.                   0.548095947 0.72829188
## Fusobacterium                        0.169546901 0.50305345
## Leptotrichia                         0.015925525 0.22068228
## Gracilibacteria_(GN02)_[G.1]         0.482367217 0.69579213
## Lautropia                            0.062890638 0.47152520
## Ottowia                              0.431742383 0.67430696
## Eikenella                            0.721361247 0.82320048
## Kingella                             0.244557652 0.56481172
## Neisseria                            0.439387695 0.67430696
## Family.Neisseriaceae.                0.663236083 0.77510723
## Campylobacter                        0.405849805 0.67430696
## Cardiobacterium                      0.282234859 0.58248471
## Aggregatibacter                      0.578580469 0.73801220
## Haemophilus                          0.252226039 0.56897502
## Family.Pasteurellaceae.              0.186700249 0.50305345
## Saccharibacteria_(TM7)_[G.1]         0.120948803 0.48267244
## Saccharibacteria_(TM7)_[G.3]         0.238851105 0.56481172
## Saccharibacteria_(TM7)_[G.6]         0.093183839 0.48267244
## Family.Saccharibacteria_(TM7)_[F-1]. 0.072107237 0.47152520
## Saccharibacteria_(TM7)_[G.5]         0.777143477 0.84699907
## Treponema                            0.007058783 0.13694040
## Fretibacterium                       0.056834822 0.47152520
## Kingdom.Bacteria.                    0.437499988 0.67430696
## Others                               0.601061479 0.73801220
##                                                                    Feature
## Absconditabacteria_(SR1)_[G.1]            Absconditabacteria_(SR1)_[G.1]
## Actinomyces                                                    Actinomyces
## Peptidiphaga                                                  Peptidiphaga
## Schaalia                                                          Schaalia
## Family.Actinomycetaceae.                          Family.Actinomycetaceae.
## Rothia                                                              Rothia
## Alloscardovia                                                Alloscardovia
```

```
## Bifidobacterium                                       Bifidobacterium
## Parascardovia                                           Parascardovia
## Scardovia                                                   Scardovia
## Corynebacterium                                       Corynebacterium
## Olsenella                                                   Olsenella
## Atopobium                                                   Atopobium
## Slackia                                                       Slackia
## Cryptobacterium                                       Cryptobacterium
## Bacteroidetes_[G.3]                               Bacteroidetes_[G.3]
## Bacteroidetes_[G.5]                               Bacteroidetes_[G.5]
## Family.Bacteroidetes_[F-1].               Family.Bacteroidetes_[F-1].
## Bacteroidaceae_[G.1]                             Bacteroidaceae_[G.1]
## Bacteroidales_[G.2]                               Bacteroidales_[G.2]
## Porphyromonas                                           Porphyromonas
## Tannerella                                                 Tannerella
## Alloprevotella                                         Alloprevotella
## Prevotella                                                 Prevotella
## Bergeyella                                                 Bergeyella
## Capnocytophaga                                         Capnocytophaga
## Gemella                                                       Gemella
## Abiotrophia                                               Abiotrophia
## Granulicatella                                         Granulicatella
## Lactobacillus                                           Lactobacillus
## Streptococcus                                           Streptococcus
## Order.Lactobacillales.                         Order.Lactobacillales.
## Class.Bacilli.                                         Class.Bacilli.
## Clostridiales_[F.1][G-1]                     Clostridiales_[F.1][G-1]
## Butyrivibrio                                             Butyrivibrio
## Catonella                                                   Catonella
## Johnsonella                                               Johnsonella
## Lachnoanaerobaculum                               Lachnoanaerobaculum
## Lachnospiraceae_[G.2]                           Lachnospiraceae_[G.2]
## Lachnospiraceae_[G.3]                           Lachnospiraceae_[G.3]
## Lachnospiraceae_[G.7]                           Lachnospiraceae_[G.7]
## Lachnospiraceae_[G.8]                           Lachnospiraceae_[G.8]
## Oribacterium                                             Oribacterium
## Shuttleworthia                                         Shuttleworthia
## Stomatobaculum                                         Stomatobaculum
## Family.Lachnospiraceae_[XIV].           Family.Lachnospiraceae_[XIV].
## Peptococcus                                               Peptococcus
## Parvimonas                                                 Parvimonas
## Filifactor                                                 Filifactor
## Mogibacterium                                           Mogibacterium
## Peptostreptococcaceae_[XI][G.1]       Peptostreptococcaceae_[XI][G.1]
## Peptostreptococcaceae_[XI][G.4]       Peptostreptococcaceae_[XI][G.4]
## Peptostreptococcaceae_[XI][G.5]       Peptostreptococcaceae_[XI][G.5]
## Peptostreptococcaceae_[XI][G.6]       Peptostreptococcaceae_[XI][G.6]
## Peptostreptococcaceae_[XI][G.7]       Peptostreptococcaceae_[XI][G.7]
## Peptostreptococcaceae_[XI][G.9]       Peptostreptococcaceae_[XI][G.9]
## Peptostreptococcus                                 Peptostreptococcus
## Ruminococcaceae_[G.1]                           Ruminococcaceae_[G.1]
## Ruminococcaceae_[G.2]                           Ruminococcaceae_[G.2]
## Bulleidia                                                   Bulleidia
## Eggerthia                                                   Eggerthia
```

```
## Solobacterium                                                     Solobacterium
## Mycoplasma                                                           Mycoplasma
## Mitsuokella                                                         Mitsuokella
## Selenomonas                                                         Selenomonas
## Family.Selenomonadaceae.                                Family.Selenomonadaceae.
## Class.Negativicutes.                                        Class.Negativicutes.
## Anaeroglobus                                                       Anaeroglobus
## Dialister                                                             Dialister
## Megasphaera                                                         Megasphaera
## Family.Veillonellaceae.                                  Family.Veillonellaceae.
## Veillonella                                                         Veillonella
## Veillonellaceae_[G.1]                                      Veillonellaceae_[G.1]
## Phylum.Firmicutes.                                            Phylum.Firmicutes.
## Fusobacterium                                                     Fusobacterium
## Leptotrichia                                                       Leptotrichia
## Gracilibacteria_(GN02)_[G.1]                        Gracilibacteria_(GN02)_[G.1]
## Lautropia                                                             Lautropia
## Ottowia                                                                 Ottowia
## Eikenella                                                             Eikenella
## Kingella                                                               Kingella
## Neisseria                                                             Neisseria
## Family.Neisseriaceae.                                      Family.Neisseriaceae.
## Campylobacter                                                     Campylobacter
## Cardiobacterium                                                 Cardiobacterium
## Aggregatibacter                                                 Aggregatibacter
## Haemophilus                                                         Haemophilus
## Family.Pasteurellaceae.                                  Family.Pasteurellaceae.
## Saccharibacteria_(TM7)_[G.1]                        Saccharibacteria_(TM7)_[G.1]
## Saccharibacteria_(TM7)_[G.3]                        Saccharibacteria_(TM7)_[G.3]
## Saccharibacteria_(TM7)_[G.6]                        Saccharibacteria_(TM7)_[G.6]
## Family.Saccharibacteria_(TM7)_[F-1]. Family.Saccharibacteria_(TM7)_[F-1].
## Saccharibacteria_(TM7)_[G.5]                        Saccharibacteria_(TM7)_[G.5]
## Treponema                                                             Treponema
## Fretibacterium                                                   Fretibacterium
## Kingdom.Bacteria.                                              Kingdom.Bacteria.
## Others                                                                   Others
##                                                   Method
## Absconditabacteria_(SR1)_[G.1]       Kruskal-Wallis (kru)
## Actinomyces                          Kruskal-Wallis (kru)
## Peptidiphaga                         Kruskal-Wallis (kru)
## Schaalia                             Kruskal-Wallis (kru)
## Family.Actinomycetaceae.             Kruskal-Wallis (kru)
## Rothia                               Kruskal-Wallis (kru)
## Alloscardovia                        Kruskal-Wallis (kru)
## Bifidobacterium                      Kruskal-Wallis (kru)
## Parascardovia                        Kruskal-Wallis (kru)
## Scardovia                            Kruskal-Wallis (kru)
## Corynebacterium                      Kruskal-Wallis (kru)
## Olsenella                            Kruskal-Wallis (kru)
## Atopobium                            Kruskal-Wallis (kru)
## Slackia                              Kruskal-Wallis (kru)
## Cryptobacterium                      Kruskal-Wallis (kru)
## Bacteroidetes_[G.3]                  Kruskal-Wallis (kru)
## Bacteroidetes_[G.5]                  Kruskal-Wallis (kru)
```

```
## Family.Bacteroidetes_[F-1].          Kruskal-Wallis (kru)
## Bacteroidaceae_[G.1]                 Kruskal-Wallis (kru)
## Bacteroidales_[G.2]                  Kruskal-Wallis (kru)
## Porphyromonas                        Kruskal-Wallis (kru)
## Tannerella                           Kruskal-Wallis (kru)
## Alloprevotella                       Kruskal-Wallis (kru)
## Prevotella                           Kruskal-Wallis (kru)
## Bergeyella                           Kruskal-Wallis (kru)
## Capnocytophaga                       Kruskal-Wallis (kru)
## Gemella                              Kruskal-Wallis (kru)
## Abiotrophia                          Kruskal-Wallis (kru)
## Granulicatella                       Kruskal-Wallis (kru)
## Lactobacillus                        Kruskal-Wallis (kru)
## Streptococcus                        Kruskal-Wallis (kru)
## Order.Lactobacillales.               Kruskal-Wallis (kru)
## Class.Bacilli.                       Kruskal-Wallis (kru)
## Clostridiales_[F.1][G-1]             Kruskal-Wallis (kru)
## Butyrivibrio                         Kruskal-Wallis (kru)
## Catonella                            Kruskal-Wallis (kru)
## Johnsonella                          Kruskal-Wallis (kru)
## Lachnoanaerobaculum                  Kruskal-Wallis (kru)
## Lachnospiraceae_[G.2]                Kruskal-Wallis (kru)
## Lachnospiraceae_[G.3]                Kruskal-Wallis (kru)
## Lachnospiraceae_[G.7]                Kruskal-Wallis (kru)
## Lachnospiraceae_[G.8]                Kruskal-Wallis (kru)
## Oribacterium                         Kruskal-Wallis (kru)
## Shuttleworthia                       Kruskal-Wallis (kru)
## Stomatobaculum                       Kruskal-Wallis (kru)
## Family.Lachnospiraceae_[XIV].        Kruskal-Wallis (kru)
## Peptococcus                          Kruskal-Wallis (kru)
## Parvimonas                           Kruskal-Wallis (kru)
## Filifactor                           Kruskal-Wallis (kru)
## Mogibacterium                        Kruskal-Wallis (kru)
## Peptostreptococcaceae_[XI][G.1]      Kruskal-Wallis (kru)
## Peptostreptococcaceae_[XI][G.4]      Kruskal-Wallis (kru)
## Peptostreptococcaceae_[XI][G.5]      Kruskal-Wallis (kru)
## Peptostreptococcaceae_[XI][G.6]      Kruskal-Wallis (kru)
## Peptostreptococcaceae_[XI][G.7]      Kruskal-Wallis (kru)
## Peptostreptococcaceae_[XI][G.9]      Kruskal-Wallis (kru)
## Peptostreptococcus                   Kruskal-Wallis (kru)
## Ruminococcaceae_[G.1]                Kruskal-Wallis (kru)
## Ruminococcaceae_[G.2]                Kruskal-Wallis (kru)
## Bulleidia                            Kruskal-Wallis (kru)
## Eggerthia                            Kruskal-Wallis (kru)
## Solobacterium                        Kruskal-Wallis (kru)
## Mycoplasma                           Kruskal-Wallis (kru)
## Mitsuokella                          Kruskal-Wallis (kru)
## Selenomonas                          Kruskal-Wallis (kru)
## Family.Selenomonadaceae.             Kruskal-Wallis (kru)
## Class.Negativicutes.                 Kruskal-Wallis (kru)
## Anaeroglobus                         Kruskal-Wallis (kru)
## Dialister                            Kruskal-Wallis (kru)
## Megasphaera                          Kruskal-Wallis (kru)
## Family.Veillonellaceae.              Kruskal-Wallis (kru)
```

```
## Veillonella                          Kruskal-Wallis (kru)
## Veillonellaceae_[G.1]                Kruskal-Wallis (kru)
## Phylum.Firmicutes.                   Kruskal-Wallis (kru)
## Fusobacterium                        Kruskal-Wallis (kru)
## Leptotrichia                         Kruskal-Wallis (kru)
## Gracilibacteria_(GN02)_[G.1]         Kruskal-Wallis (kru)
## Lautropia                            Kruskal-Wallis (kru)
## Ottowia                              Kruskal-Wallis (kru)
## Eikenella                            Kruskal-Wallis (kru)
## Kingella                             Kruskal-Wallis (kru)
## Neisseria                            Kruskal-Wallis (kru)
## Family.Neisseriaceae.                Kruskal-Wallis (kru)
## Campylobacter                        Kruskal-Wallis (kru)
## Cardiobacterium                      Kruskal-Wallis (kru)
## Aggregatibacter                      Kruskal-Wallis (kru)
## Haemophilus                          Kruskal-Wallis (kru)
## Family.Pasteurellaceae.              Kruskal-Wallis (kru)
## Saccharibacteria_(TM7)_[G.1]         Kruskal-Wallis (kru)
## Saccharibacteria_(TM7)_[G.3]         Kruskal-Wallis (kru)
## Saccharibacteria_(TM7)_[G.6]         Kruskal-Wallis (kru)
## Family.Saccharibacteria_(TM7)_[F-1]. Kruskal-Wallis (kru)
## Saccharibacteria_(TM7)_[G.5]         Kruskal-Wallis (kru)
## Treponema                            Kruskal-Wallis (kru)
## Fretibacterium                       Kruskal-Wallis (kru)
## Kingdom.Bacteria.                    Kruskal-Wallis (kru)
## Others                               Kruskal-Wallis (kru)
```

```
write.table(final, file="act_cat_kru.txt", sep="\t", dec=",", row.names=F)
write.table(final[final$pval<0.05,], file="act_cat_kru_sig.txt", sep="\t", dec=",", row.names=F)
#################Glycaemia_Status
table(Phe$Glycaemia_Status, useNA="always") #see gly_stat same test
```

```
##
##     Gr1ngt    Gr2ilFG    Gr3ilGT Gr4IFG_IGT     Gr5SDM        KDM    Unclass
##        421        112         58         63         68         21          3
##       <NA>
##          0
```

```
#################hba1c_cat
table(Phe$hba1c_cat, useNA="always")
```

```
##
##    Low Medium   High   <NA>
##    600    119     26      1
```

```
#Remove NA
Phe2<-Phe[complete.cases(Phe$hba1c_cat),]
#Also subset columns
Microbio2<-dplyr::select(Microbio, one_of(Phe2$IDX))
final <- DA.ds2(Microbio2, predictor = Phe2$hba1c_cat, out.all=TRUE) #It is LRT so don't look at orderi
final
```

```
##                                      baseMean log2FoldChange      lfcSE
## Absconditabacteria_(SR1)_[G.1]     65.8083996   -2.078175460 0.33353146
## Actinomyces                       323.5994877    0.197852141 0.11381062
## Peptidiphaga                       22.0050721    0.012125751 0.16287914
## Schaalia                          702.4964214    0.085626110 0.09342672
## Family.Actinomycetaceae.            5.4367511   -0.118075561 0.23568094
## Rothia                           1938.7037786    0.306133790 0.10921029
## Alloscardovia                      12.4584403    0.119928905 0.28896508
## Bifidobacterium                    12.6345225    0.118978461 0.32519458
## Parascardovia                       2.8066146    0.819766620 0.42854605
## Scardovia                          14.4042935    0.349528824 0.28284711
## Corynebacterium                    93.6716081    0.234369921 0.11959757
## Olsenella                           1.5661971   -0.188476995 0.27061169
## Atopobium                         119.8056644   -0.023264528 0.10979610
## Slackia                             1.3866219   -0.669227343 0.26708903
## Cryptobacterium                     4.2899669   -0.487918486 0.27638798
## Bacteroidetes_[G.3]                 6.5869763   -0.084824472 0.28968610
## Bacteroidetes_[G.5]                 4.6704170   -0.191015380 0.31686429
## Family.Bacteroidetes_[F-1].         0.8669826   -0.219060824 0.46049753
## Bacteroidaceae_[G.1]                0.7924375    0.197808867 0.42691558
## Bacteroidales_[G.2]                12.1392645    0.037855782 0.16611918
## Porphyromonas                     679.7792654   -0.041244478 0.15068348
## Tannerella                         22.0331730    0.006472271 0.11153548
## Alloprevotella                    360.5559989   -0.447556566 0.11860492
## Prevotella                       3802.7293750    0.006446805 0.07569930
## Bergeyella                         72.4816383   -0.162361789 0.11686027
## Capnocytophaga                    225.0697493    0.084810031 0.12545056
## Gemella                           326.4262635    0.052113623 0.11301145
## Abiotrophia                        29.9626473    0.334578341 0.22579556
## Granulicatella                    306.0513594    0.087144161 0.09152222
## Lactobacillus                      38.2200658    3.105608956 0.34925014
## Streptococcus                    3800.8272350    0.405743857 0.08469259
## Order.Lactobacillales.              0.2862023    0.146359705 0.31292208
## Class.Bacilli.                      0.5745982    0.192292445 0.24814758
## Clostridiales_[F.1][G-1]            1.0546936    0.340085890 0.37274081
## Butyrivibrio                        6.9099023    0.082042795 0.23972153
## Catonella                          36.7605802   -0.042396041 0.13532823
## Johnsonella                         0.9391664   -0.056014557 0.32895869
## Lachnoanaerobaculum                87.6094852    0.047154330 0.09424052
## Lachnospiraceae_[G.2]              27.8875160   -0.641406255 0.24761320
## Lachnospiraceae_[G.3]               2.8875675   -0.128266407 0.25949115
## Lachnospiraceae_[G.7]               1.4690851    0.474222532 0.36462723
## Lachnospiraceae_[G.8]               1.4324153   -0.384075619 0.39674706
## Oribacterium                      146.7632604   -0.059092744 0.10470733
## Shuttleworthia                      2.8046296   -0.457637483 0.29395219
## Stomatobaculum                    112.5219132    0.117244242 0.11748561
## Family.Lachnospiraceae_[XIV].       1.6037240   -0.798847355 0.34267422
## Peptococcus                         3.7013485   -0.258566978 0.29491048
## Parvimonas                         40.8300883   -0.412436451 0.14503726
## Filifactor                          6.8352717   -0.055410195 0.28440116
## Mogibacterium                      41.0236194    0.066901059 0.10022232
## Peptostreptococcaceae_[XI][G.1]    67.2115667   -0.138327887 0.17892627
## Peptostreptococcaceae_[XI][G.4]     0.4178474    0.260809590 0.38578621
## Peptostreptococcaceae_[XI][G.5]     1.6627884    0.044018906 0.40924692
```

```
## Peptostreptococcaceae_[XI][G.6]         1.0142543    0.130932666 0.40143767
## Peptostreptococcaceae_[XI][G.7]         4.7094117    0.009528119 0.27937117
## Peptostreptococcaceae_[XI][G.9]         8.7194835   -0.037462896 0.16631095
## Peptostreptococcus                     42.8126030   -0.180139980 0.20901465
## Ruminococcaceae_[G.1]                  25.0982275   -0.271695478 0.21264064
## Ruminococcaceae_[G.2]                  41.2189039   -0.055895496 0.17671319
## Bulleidia                               3.8583839    0.281023017 0.30993553
## Eggerthia                               1.5798155   -0.379103219 0.42218549
## Solobacterium                          73.9304904   -0.094934657 0.11804755
## Mycoplasma                              4.4971608    0.065171537 0.24912642
## Mitsuokella                            26.8554383   -0.310827359 0.22409629
## Selenomonas                           221.6646708   -0.179415765 0.10044022
## Family.Selenomonadaceae.                5.0121185    0.001181600 0.26382033
## Class.Negativicutes.                    0.7372433   -0.227830010 0.24890466
## Anaeroglobus                            8.6996690    0.516482901 0.23932363
## Dialister                              36.4272993   -0.179150889 0.12380625
## Megasphaera                           405.9085914   -0.121200296 0.12978468
## Family.Veillonellaceae.                 0.5255260   -0.371273342 0.29104165
## Veillonella                          5057.8970332    0.101023780 0.07312487
## Veillonellaceae_[G.1]                   6.1411684   -0.228581655 0.20336258
## Phylum.Firmicutes.                      0.8916355   -0.773263941 0.34687789
## Fusobacterium                         926.7032571   -0.078673601 0.09712840
## Leptotrichia                          682.0312738   -0.008600923 0.10045019
## Gracilibacteria_(GN02)_[G.1]            2.4054534    0.031502572 0.32626355
## Lautropia                              68.3118466    0.336975286 0.18640062
## Ottowia                                 2.1804469    0.404118135 0.41274348
## Eikenella                               8.7658458    0.184090394 0.15609523
## Kingella                               99.6715990   -0.111771735 0.13437293
## Neisseria                            1327.1726631   -0.125047978 0.15336057
## Family.Neisseriaceae.                   6.0974840   -0.472487451 0.35936182
## Campylobacter                         257.5630090    0.085241938 0.08613054
## Cardiobacterium                        14.7885081    0.150878412 0.13447442
## Aggregatibacter                       275.0083562    0.029941455 0.17700451
## Haemophilus                          2905.1713651   -0.087803484 0.11805978
## Family.Pasteurellaceae.                 2.4777183    0.060212622 0.48530647
## Saccharibacteria_(TM7)_[G.1]           35.9292444    0.303629262 0.19723629
## Saccharibacteria_(TM7)_[G.3]            7.1095886    0.071797372 0.27561975
## Saccharibacteria_(TM7)_[G.6]            7.2190288    0.176190031 0.35002826
## Family.Saccharibacteria_(TM7)_[F-1].    1.0911879   -0.208946008 0.43889079
## Saccharibacteria_(TM7)_[G.5]            0.9919980   -0.297795834 0.42345121
## Treponema                              63.1995602   -0.205409322 0.18683451
## Fretibacterium                          8.2673336    0.074167519 0.22680166
## Kingdom.Bacteria.                       9.3681949   -0.744615457 0.28756484
## Others                                 33.1435829    0.293375010 0.19032641
##                                             stat         pval   ordering
## Absconditabacteria_(SR1)_[G.1]        32.58017647 8.419854e-08 Low>Medium
## Actinomyces                            3.51298546 1.726493e-01 Medium>Low
## Peptidiphaga                           0.16067979 9.228026e-01 Medium>Low
## Schaalia                               4.99906898 8.212322e-02 Medium>Low
## Family.Actinomycetaceae.               0.66085413 7.186168e-01 Low>Medium
## Rothia                                 8.62406127 1.340630e-02 Medium>Low
## Alloscardovia                          1.16538609 5.583926e-01 Medium>Low
## Bifidobacterium                        2.41364549 2.991462e-01 Medium>Low
## Parascardovia                          4.89385129 8.655929e-02 Medium>Low
```

```
## Scardovia                               3.17214793 2.047278e-01 Medium>Low
## Corynebacterium                         7.18236820 2.756567e-02 Medium>Low
## Olsenella                               3.48022763 1.755004e-01 Low>Medium
## Atopobium                               0.09981281 9.513185e-01 Low>Medium
## Slackia                                 7.14949238 2.802254e-02 Low>Medium
## Cryptobacterium                         4.91530757 8.563563e-02 Low>Medium
## Bacteroidetes_[G.3]                     1.99061544 3.696097e-01 Low>Medium
## Bacteroidetes_[G.5]                     1.34628651 5.101027e-01 Low>Medium
## Family.Bacteroidetes_[F-1].             0.40522976 8.165927e-01 Low>Medium
## Bacteroidaceae_[G.1]                    0.24902450 8.829274e-01 Medium>Low
## Bacteroidales_[G.2]                     2.02841978 3.626889e-01 Medium>Low
## Porphyromonas                           5.65477124 5.916734e-02 Low>Medium
## Tannerella                              7.76973988 2.055050e-02 Medium>Low
## Alloprevotella                         17.38560921 1.677888e-04 Low>Medium
## Prevotella                              8.20339962 1.654453e-02 Medium>Low
## Bergeyella                             12.81418508 1.649814e-03 Low>Medium
## Capnocytophaga                          1.10723830 5.748655e-01 Medium>Low
## Gemella                                 3.39867958 1.828042e-01 Medium>Low
## Abiotrophia                             6.47348835 3.929161e-02 Medium>Low
## Granulicatella                          1.25771522 5.332006e-01 Medium>Low
## Lactobacillus                         112.81322252 3.183611e-25 Medium>Low
## Streptococcus                          24.39484844 5.043430e-06 Medium>Low
## Order.Lactobacillales.                 -3.30175200 1.000000e+00 Medium>Low
## Class.Bacilli.                          5.42001663 6.653625e-02 Medium>Low
## Clostridiales_[F.1][G-1]                3.35125598 1.871906e-01 Medium>Low
## Butyrivibrio                            0.75354465 6.860723e-01 Medium>Low
## Catonella                               0.10800427 9.474301e-01 Low>Medium
## Johnsonella                             2.95931705 2.277154e-01 Low>Medium
## Lachnoanaerobaculum                     4.58168694 1.011811e-01 Medium>Low
## Lachnospiraceae_[G.2]                   6.13569200 4.652125e-02 Low>Medium
## Lachnospiraceae_[G.3]                   1.66713588 4.344963e-01 Low>Medium
## Lachnospiraceae_[G.7]                   3.14306529 2.077266e-01 Medium>Low
## Lachnospiraceae_[G.8]                   1.49966306 4.724461e-01 Low>Medium
## Oribacterium                            0.33552107 8.455563e-01 Low>Medium
## Shuttleworthia                          5.85876670 5.342998e-02 Low>Medium
## Stomatobaculum                          3.91550476 1.411754e-01 Medium>Low
## Family.Lachnospiraceae_[XIV].           7.42255845 2.444623e-02 Low>Medium
## Peptococcus                             0.78410871 6.756674e-01 Low>Medium
## Parvimonas                              8.19855663 1.658464e-02 Low>Medium
## Filifactor                              0.38166270 8.262719e-01 Low>Medium
## Mogibacterium                           4.18464403 1.234003e-01 Medium>Low
## Peptostreptococcaceae_[XI][G.1]         4.70008084 9.536531e-02 Low>Medium
## Peptostreptococcaceae_[XI][G.4]        -0.10627830 1.000000e+00 Medium>Low
## Peptostreptococcaceae_[XI][G.5]         0.85976130 6.505867e-01 Medium>Low
## Peptostreptococcaceae_[XI][G.6]         0.15059128 9.274692e-01 Medium>Low
## Peptostreptococcaceae_[XI][G.7]         0.51728844 7.720977e-01 Medium>Low
## Peptostreptococcaceae_[XI][G.9]         4.63573215 9.848352e-02 Low>Medium
## Peptostreptococcus                      5.32588911 6.974256e-02 Low>Medium
## Ruminococcaceae_[G.1]                   1.64447262 4.394478e-01 Low>Medium
## Ruminococcaceae_[G.2]                   0.11458757 9.443166e-01 Low>Medium
## Bulleidia                               4.82107637 8.976697e-02 Medium>Low
## Eggerthia                               1.67424672 4.329542e-01 Low>Medium
## Solobacterium                           0.76690047 6.815060e-01 Low>Medium
## Mycoplasma                              1.05814653 5.891507e-01 Medium>Low
```

```
## Mitsuokella                              1.92402102 3.821238e-01 Low>Medium
## Selenomonas                              3.67802473 1.589744e-01 Low>Medium
## Family.Selenomonadaceae.                 0.17211178 9.175429e-01 Medium>Low
## Class.Negativicutes.                     1.10698187 5.749392e-01 Low>Medium
## Anaeroglobus                             5.05996627 7.966036e-02 Medium>Low
## Dialister                                2.95098312 2.286663e-01 Low>Medium
## Megasphaera                              1.31710796 5.175993e-01 Low>Medium
## Family.Veillonellaceae.                  4.15157132 1.254578e-01 Low>Medium
## Veillonella                              2.26488521 3.222452e-01 Medium>Low
## Veillonellaceae_[G.1]                   16.89930574 2.139747e-04 Low>Medium
## Phylum.Firmicutes.                       5.37648683 6.800028e-02 Low>Medium
## Fusobacterium                            3.45117047 1.780688e-01 Low>Medium
## Leptotrichia                             0.14998624 9.277499e-01 Low>Medium
## Gracilibacteria_(GN02)_[G.1]             0.23232020 8.903327e-01 Medium>Low
## Lautropia                                4.18837027 1.231706e-01 Medium>Low
## Ottowia                                  3.62235452 1.634616e-01 Medium>Low
## Eikenella                                1.89428018 3.878487e-01 Medium>Low
## Kingella                                 3.29091449 1.929243e-01 Low>Medium
## Neisseria                                4.99064844 8.246971e-02 Low>Medium
## Family.Neisseriaceae.                    1.85272465 3.959916e-01 Low>Medium
## Campylobacter                            1.71592579 4.240250e-01 Medium>Low
## Cardiobacterium                          1.38459002 5.004263e-01 Medium>Low
## Aggregatibacter                          3.35735350 1.866208e-01 Medium>Low
## Haemophilus                              4.83477568 8.915420e-02 Low>Medium
## Family.Pasteurellaceae.                  1.21076957 5.458643e-01 Medium>Low
## Saccharibacteria_(TM7)_[G.1]             3.56107574 1.685475e-01 Medium>Low
## Saccharibacteria_(TM7)_[G.3]             0.76347715 6.826735e-01 Medium>Low
## Saccharibacteria_(TM7)_[G.6]             2.54822279 2.796794e-01 Medium>Low
## Family.Saccharibacteria_(TM7)_[F-1].     2.21836660 3.298282e-01 Low>Medium
## Saccharibacteria_(TM7)_[G.5]             4.38864907 1.114338e-01 Low>Medium
## Treponema                                6.26113264 4.369305e-02 Low>Medium
## Fretibacterium                           4.69704489 9.551018e-02 Medium>Low
## Kingdom.Bacteria.                        8.53380881 1.402513e-02 Low>Medium
## Others                                   4.18026292 1.236709e-01 Medium>Low
##                                            pval.adj
## Absconditabacteria_(SR1)_[G.1]         4.083629e-06
## Actinomyces                            3.782810e-01
## Peptidiphaga                           9.713462e-01
## Schaalia                               2.974111e-01
## Family.Actinomycetaceae.               8.500711e-01
## Rothia                                 1.608710e-01
## Alloscardovia                          7.419737e-01
## Bifidobacterium                        5.275852e-01
## Parascardovia                          2.974111e-01
## Scardovia                              3.950878e-01
## Corynebacterium                        1.941562e-01
## Olsenella                              3.782810e-01
## Atopobium                              9.713462e-01
## Slackia                                1.941562e-01
## Cryptobacterium                        2.974111e-01
## Bacteroidetes_[G.3]                    6.076634e-01
## Bacteroidetes_[G.5]                    7.171009e-01
## Family.Bacteroidetes_[F-1].            9.429221e-01
## Bacteroidaceae_[G.1]                   9.713462e-01
```

```
## Bacteroidales_[G.2]                       6.065659e-01
## Porphyromonas                             2.974111e-01
## Tannerella                                1.812181e-01
## Alloprevotella                            4.068878e-03
## Prevotella                                1.608710e-01
## Bergeyella                                2.667200e-02
## Capnocytophaga                            7.435881e-01
## Gemella                                   3.782810e-01
## Abiotrophia                               2.540858e-01
## Granulicatella                            7.284571e-01
## Lactobacillus                             3.088103e-23
## Streptococcus                             1.630709e-04
## Order.Lactobacillales.                    1.000000e+00
## Class.Bacilli.                            2.974111e-01
## Clostridiales_[F.1][G-1]                  3.782810e-01
## Butyrivibrio                              8.215927e-01
## Catonella                                 9.713462e-01
## Johnsonella                               4.185025e-01
## Lachnoanaerobaculum                       2.974111e-01
## Lachnospiraceae_[G.2]                     2.654448e-01
## Lachnospiraceae_[G.3]                     6.458551e-01
## Lachnospiraceae_[G.7]                     3.950878e-01
## Lachnospiraceae_[G.8]                     6.839892e-01
## Oribacterium                              9.537088e-01
## Shuttleworthia                            2.879282e-01
## Stomatobaculum                            3.511285e-01
## Family.Lachnospiraceae_[XIV].             1.941562e-01
## Peptococcus                               8.215927e-01
## Parvimonas                                1.608710e-01
## Filifactor                                9.429221e-01
## Mogibacterium                             3.202476e-01
## Peptostreptococcaceae_[XI][G.1]           2.974111e-01
## Peptostreptococcaceae_[XI][G.4]           1.000000e+00
## Peptostreptococcaceae_[XI][G.5]           8.195703e-01
## Peptostreptococcaceae_[XI][G.6]           9.713462e-01
## Peptostreptococcaceae_[XI][G.7]           9.023310e-01
## Peptostreptococcaceae_[XI][G.9]           2.974111e-01
## Peptostreptococcus                        2.974111e-01
## Ruminococcaceae_[G.1]                     6.458551e-01
## Ruminococcaceae_[G.2]                     9.713462e-01
## Bulleidia                                 2.974111e-01
## Eggerthia                                 6.458551e-01
## Solobacterium                             8.215927e-01
## Mycoplasma                                7.519423e-01
## Mitsuokella                               6.167429e-01
## Selenomonas                               3.782810e-01
## Family.Selenomonadaceae.                  9.713462e-01
## Class.Negativicutes.                      7.435881e-01
## Anaeroglobus                              2.974111e-01
## Dialister                                 4.185025e-01
## Megasphaera                               7.172447e-01
## Family.Veillonellaceae.                   3.202476e-01
## Veillonella                               5.581747e-01
## Veillonellaceae_[G.1]                     4.151109e-03
```

```
## Phylum.Firmicutes.                     2.974111e-01
## Fusobacterium                          3.782810e-01
## Leptotrichia                           9.713462e-01
## Gracilibacteria_(GN02)_[G.1]           9.713462e-01
## Lautropia                              3.202476e-01
## Ottowia                                3.782810e-01
## Eikenella                              6.167429e-01
## Kingella                               3.819114e-01
## Neisseria                              2.974111e-01
## Family.Neisseriaceae.                  6.195352e-01
## Campylobacter                          6.458551e-01
## Cardiobacterium                        7.138433e-01
## Aggregatibacter                        3.782810e-01
## Haemophilus                            2.974111e-01
## Family.Pasteurellaceae.                7.354006e-01
## Saccharibacteria_(TM7)_[G.1]           3.782810e-01
## Saccharibacteria_(TM7)_[G.3]           8.215927e-01
## Saccharibacteria_(TM7)_[G.6]           5.023870e-01
## Family.Saccharibacteria_(TM7)_[F-1]. 5.612866e-01
## Saccharibacteria_(TM7)_[G.5]           3.179141e-01
## Treponema                              2.648891e-01
## Fretibacterium                         2.974111e-01
## Kingdom.Bacteria.                      1.608710e-01
## Others                                 3.202476e-01
##                                                              Feature
## Absconditabacteria_(SR1)_[G.1]            Absconditabacteria_(SR1)_[G.1]
## Actinomyces                                                Actinomyces
## Peptidiphaga                                              Peptidiphaga
## Schaalia                                                      Schaalia
## Family.Actinomycetaceae.                        Family.Actinomycetaceae.
## Rothia                                                          Rothia
## Alloscardovia                                            Alloscardovia
## Bifidobacterium                                        Bifidobacterium
## Parascardovia                                            Parascardovia
## Scardovia                                                    Scardovia
## Corynebacterium                                        Corynebacterium
## Olsenella                                                    Olsenella
## Atopobium                                                    Atopobium
## Slackia                                                        Slackia
## Cryptobacterium                                        Cryptobacterium
## Bacteroidetes_[G.3]                                Bacteroidetes_[G.3]
## Bacteroidetes_[G.5]                                Bacteroidetes_[G.5]
## Family.Bacteroidetes_[F-1].                  Family.Bacteroidetes_[F-1].
## Bacteroidaceae_[G.1]                              Bacteroidaceae_[G.1]
## Bacteroidales_[G.2]                                Bacteroidales_[G.2]
## Porphyromonas                                            Porphyromonas
## Tannerella                                                  Tannerella
## Alloprevotella                                          Alloprevotella
## Prevotella                                                  Prevotella
## Bergeyella                                                  Bergeyella
## Capnocytophaga                                          Capnocytophaga
## Gemella                                                        Gemella
## Abiotrophia                                                Abiotrophia
## Granulicatella                                          Granulicatella
```

```
## Lactobacillus                                                  Lactobacillus
## Streptococcus                                                  Streptococcus
## Order.Lactobacillales.                                Order.Lactobacillales.
## Class.Bacilli.                                                Class.Bacilli.
## Clostridiales_[F.1][G-1]                            Clostridiales_[F.1][G-1]
## Butyrivibrio                                                    Butyrivibrio
## Catonella                                                          Catonella
## Johnsonella                                                      Johnsonella
## Lachnoanaerobaculum                                      Lachnoanaerobaculum
## Lachnospiraceae_[G.2]                                  Lachnospiraceae_[G.2]
## Lachnospiraceae_[G.3]                                  Lachnospiraceae_[G.3]
## Lachnospiraceae_[G.7]                                  Lachnospiraceae_[G.7]
## Lachnospiraceae_[G.8]                                  Lachnospiraceae_[G.8]
## Oribacterium                                                    Oribacterium
## Shuttleworthia                                                Shuttleworthia
## Stomatobaculum                                                Stomatobaculum
## Family.Lachnospiraceae_[XIV].                  Family.Lachnospiraceae_[XIV].
## Peptococcus                                                      Peptococcus
## Parvimonas                                                        Parvimonas
## Filifactor                                                        Filifactor
## Mogibacterium                                                  Mogibacterium
## Peptostreptococcaceae_[XI][G.1]              Peptostreptococcaceae_[XI][G.1]
## Peptostreptococcaceae_[XI][G.4]              Peptostreptococcaceae_[XI][G.4]
## Peptostreptococcaceae_[XI][G.5]              Peptostreptococcaceae_[XI][G.5]
## Peptostreptococcaceae_[XI][G.6]              Peptostreptococcaceae_[XI][G.6]
## Peptostreptococcaceae_[XI][G.7]              Peptostreptococcaceae_[XI][G.7]
## Peptostreptococcaceae_[XI][G.9]              Peptostreptococcaceae_[XI][G.9]
## Peptostreptococcus                                        Peptostreptococcus
## Ruminococcaceae_[G.1]                                  Ruminococcaceae_[G.1]
## Ruminococcaceae_[G.2]                                  Ruminococcaceae_[G.2]
## Bulleidia                                                          Bulleidia
## Eggerthia                                                          Eggerthia
## Solobacterium                                                  Solobacterium
## Mycoplasma                                                        Mycoplasma
## Mitsuokella                                                      Mitsuokella
## Selenomonas                                                      Selenomonas
## Family.Selenomonadaceae.                            Family.Selenomonadaceae.
## Class.Negativicutes.                                    Class.Negativicutes.
## Anaeroglobus                                                    Anaeroglobus
## Dialister                                                          Dialister
## Megasphaera                                                      Megasphaera
## Family.Veillonellaceae.                              Family.Veillonellaceae.
## Veillonella                                                      Veillonella
## Veillonellaceae_[G.1]                                  Veillonellaceae_[G.1]
## Phylum.Firmicutes.                                        Phylum.Firmicutes.
## Fusobacterium                                                  Fusobacterium
## Leptotrichia                                                    Leptotrichia
## Gracilibacteria_(GN02)_[G.1]                    Gracilibacteria_(GN02)_[G.1]
## Lautropia                                                          Lautropia
## Ottowia                                                              Ottowia
## Eikenella                                                          Eikenella
## Kingella                                                            Kingella
## Neisseria                                                          Neisseria
## Family.Neisseriaceae.                                  Family.Neisseriaceae.
```

```
## Campylobacter                                              Campylobacter
## Cardiobacterium                                          Cardiobacterium
## Aggregatibacter                                          Aggregatibacter
## Haemophilus                                                  Haemophilus
## Family.Pasteurellaceae.                          Family.Pasteurellaceae.
## Saccharibacteria_(TM7)_[G.1]                Saccharibacteria_(TM7)_[G.1]
## Saccharibacteria_(TM7)_[G.3]                Saccharibacteria_(TM7)_[G.3]
## Saccharibacteria_(TM7)_[G.6]                Saccharibacteria_(TM7)_[G.6]
## Family.Saccharibacteria_(TM7)_[F-1]. Family.Saccharibacteria_(TM7)_[F-1].
## Saccharibacteria_(TM7)_[G.5]                Saccharibacteria_(TM7)_[G.5]
## Treponema                                                      Treponema
## Fretibacterium                                            Fretibacterium
## Kingdom.Bacteria.                                      Kingdom.Bacteria.
## Others                                                            Others
##                                                     Method
## Absconditabacteria_(SR1)_[G.1]       DESeq2 man. geoMeans (ds2)
## Actinomyces                          DESeq2 man. geoMeans (ds2)
## Peptidiphaga                         DESeq2 man. geoMeans (ds2)
## Schaalia                             DESeq2 man. geoMeans (ds2)
## Family.Actinomycetaceae.             DESeq2 man. geoMeans (ds2)
## Rothia                               DESeq2 man. geoMeans (ds2)
## Alloscardovia                        DESeq2 man. geoMeans (ds2)
## Bifidobacterium                      DESeq2 man. geoMeans (ds2)
## Parascardovia                        DESeq2 man. geoMeans (ds2)
## Scardovia                            DESeq2 man. geoMeans (ds2)
## Corynebacterium                      DESeq2 man. geoMeans (ds2)
## Olsenella                            DESeq2 man. geoMeans (ds2)
## Atopobium                            DESeq2 man. geoMeans (ds2)
## Slackia                              DESeq2 man. geoMeans (ds2)
## Cryptobacterium                      DESeq2 man. geoMeans (ds2)
## Bacteroidetes_[G.3]                  DESeq2 man. geoMeans (ds2)
## Bacteroidetes_[G.5]                  DESeq2 man. geoMeans (ds2)
## Family.Bacteroidetes_[F-1].          DESeq2 man. geoMeans (ds2)
## Bacteroidaceae_[G.1]                 DESeq2 man. geoMeans (ds2)
## Bacteroidales_[G.2]                  DESeq2 man. geoMeans (ds2)
## Porphyromonas                        DESeq2 man. geoMeans (ds2)
## Tannerella                           DESeq2 man. geoMeans (ds2)
## Alloprevotella                       DESeq2 man. geoMeans (ds2)
## Prevotella                           DESeq2 man. geoMeans (ds2)
## Bergeyella                           DESeq2 man. geoMeans (ds2)
## Capnocytophaga                       DESeq2 man. geoMeans (ds2)
## Gemella                              DESeq2 man. geoMeans (ds2)
## Abiotrophia                          DESeq2 man. geoMeans (ds2)
## Granulicatella                       DESeq2 man. geoMeans (ds2)
## Lactobacillus                        DESeq2 man. geoMeans (ds2)
## Streptococcus                        DESeq2 man. geoMeans (ds2)
## Order.Lactobacillales.               DESeq2 man. geoMeans (ds2)
## Class.Bacilli.                       DESeq2 man. geoMeans (ds2)
## Clostridiales_[F.1][G-1]             DESeq2 man. geoMeans (ds2)
## Butyrivibrio                         DESeq2 man. geoMeans (ds2)
## Catonella                            DESeq2 man. geoMeans (ds2)
## Johnsonella                          DESeq2 man. geoMeans (ds2)
## Lachnoanaerobaculum                  DESeq2 man. geoMeans (ds2)
## Lachnospiraceae_[G.2]                DESeq2 man. geoMeans (ds2)
```

```
## Lachnospiraceae_[G.3]               DESeq2 man. geoMeans (ds2)
## Lachnospiraceae_[G.7]               DESeq2 man. geoMeans (ds2)
## Lachnospiraceae_[G.8]               DESeq2 man. geoMeans (ds2)
## Oribacterium                        DESeq2 man. geoMeans (ds2)
## Shuttleworthia                      DESeq2 man. geoMeans (ds2)
## Stomatobaculum                      DESeq2 man. geoMeans (ds2)
## Family.Lachnospiraceae_[XIV].       DESeq2 man. geoMeans (ds2)
## Peptococcus                         DESeq2 man. geoMeans (ds2)
## Parvimonas                          DESeq2 man. geoMeans (ds2)
## Filifactor                          DESeq2 man. geoMeans (ds2)
## Mogibacterium                       DESeq2 man. geoMeans (ds2)
## Peptostreptococcaceae_[XI][G.1]     DESeq2 man. geoMeans (ds2)
## Peptostreptococcaceae_[XI][G.4]     DESeq2 man. geoMeans (ds2)
## Peptostreptococcaceae_[XI][G.5]     DESeq2 man. geoMeans (ds2)
## Peptostreptococcaceae_[XI][G.6]     DESeq2 man. geoMeans (ds2)
## Peptostreptococcaceae_[XI][G.7]     DESeq2 man. geoMeans (ds2)
## Peptostreptococcaceae_[XI][G.9]     DESeq2 man. geoMeans (ds2)
## Peptostreptococcus                  DESeq2 man. geoMeans (ds2)
## Ruminococcaceae_[G.1]               DESeq2 man. geoMeans (ds2)
## Ruminococcaceae_[G.2]               DESeq2 man. geoMeans (ds2)
## Bulleidia                           DESeq2 man. geoMeans (ds2)
## Eggerthia                           DESeq2 man. geoMeans (ds2)
## Solobacterium                       DESeq2 man. geoMeans (ds2)
## Mycoplasma                          DESeq2 man. geoMeans (ds2)
## Mitsuokella                         DESeq2 man. geoMeans (ds2)
## Selenomonas                         DESeq2 man. geoMeans (ds2)
## Family.Selenomonadaceae.            DESeq2 man. geoMeans (ds2)
## Class.Negativicutes.                DESeq2 man. geoMeans (ds2)
## Anaeroglobus                        DESeq2 man. geoMeans (ds2)
## Dialister                           DESeq2 man. geoMeans (ds2)
## Megasphaera                         DESeq2 man. geoMeans (ds2)
## Family.Veillonellaceae.             DESeq2 man. geoMeans (ds2)
## Veillonella                         DESeq2 man. geoMeans (ds2)
## Veillonellaceae_[G.1]               DESeq2 man. geoMeans (ds2)
## Phylum.Firmicutes.                  DESeq2 man. geoMeans (ds2)
## Fusobacterium                       DESeq2 man. geoMeans (ds2)
## Leptotrichia                        DESeq2 man. geoMeans (ds2)
## Gracilibacteria_(GN02)_[G.1]        DESeq2 man. geoMeans (ds2)
## Lautropia                           DESeq2 man. geoMeans (ds2)
## Ottowia                             DESeq2 man. geoMeans (ds2)
## Eikenella                           DESeq2 man. geoMeans (ds2)
## Kingella                            DESeq2 man. geoMeans (ds2)
## Neisseria                           DESeq2 man. geoMeans (ds2)
## Family.Neisseriaceae.               DESeq2 man. geoMeans (ds2)
## Campylobacter                       DESeq2 man. geoMeans (ds2)
## Cardiobacterium                     DESeq2 man. geoMeans (ds2)
## Aggregatibacter                     DESeq2 man. geoMeans (ds2)
## Haemophilus                         DESeq2 man. geoMeans (ds2)
## Family.Pasteurellaceae.             DESeq2 man. geoMeans (ds2)
## Saccharibacteria_(TM7)_[G.1]        DESeq2 man. geoMeans (ds2)
## Saccharibacteria_(TM7)_[G.3]        DESeq2 man. geoMeans (ds2)
## Saccharibacteria_(TM7)_[G.6]        DESeq2 man. geoMeans (ds2)
## Family.Saccharibacteria_(TM7)_[F-1]. DESeq2 man. geoMeans (ds2)
## Saccharibacteria_(TM7)_[G.5]        DESeq2 man. geoMeans (ds2)
```

```
## Treponema                                DESeq2 man. geoMeans (ds2)
## Fretibacterium                           DESeq2 man. geoMeans (ds2)
## Kingdom.Bacteria.                        DESeq2 man. geoMeans (ds2)
## Others                                   DESeq2 man. geoMeans (ds2)
```

```
write.table(final, file="hba1c_cat_ds2.txt", sep="\t", dec=",", row.names=F)
write.table(final[final$pval<0.05,], file="hba1c_cat_ds2_sig.txt", sep="\t", dec=",", row.names=F)
final <- DA.kru(Microbio2, predictor = Phe2$hba1c_cat)
final
```

```
##                                                 pval    pval.adj
## Absconditabacteria_(SR1)_[G.1]       2.198863e-02 0.113273080
## Actinomyces                          1.177370e-02 0.110393526
## Peptidiphaga                         4.611999e-01 0.612827230
## Schaalia                             5.180513e-01 0.674174607
## Family.Actinomycetaceae.             4.562423e-01 0.612827230
## Rothia                               1.777915e-03 0.044072761
## Alloscardovia                        2.966067e-01 0.496049119
## Bifidobacterium                      4.856537e-02 0.168244333
## Parascardovia                        7.783743e-02 0.222065611
## Scardovia                            9.247429e-02 0.239308083
## Corynebacterium                      9.438571e-03 0.110393526
## Olsenella                            1.593309e-02 0.110393526
## Atopobium                            3.975837e-01 0.573005463
## Slackia                              7.103438e-03 0.098433354
## Cryptobacterium                      1.907309e-02 0.113273080
## Bacteroidetes_[G.3]                  1.237882e-02 0.110393526
## Bacteroidetes_[G.5]                  3.093724e-01 0.502194138
## Family.Bacteroidetes_[F-1].          3.713007e-01 0.562752657
## Bacteroidaceae_[G.1]                 2.850746e-01 0.493789998
## Bacteroidales_[G.2]                  1.783696e-01 0.353099053
## Porphyromonas                        3.655960e-01 0.562752657
## Tannerella                           1.357569e-02 0.110393526
## Alloprevotella                       5.621952e-03 0.098433354
## Prevotella                           1.584479e-01 0.333879001
## Bergeyella                           6.091086e-03 0.098433354
## Capnocytophaga                       5.830601e-01 0.698232503
## Gemella                              3.106355e-01 0.502194138
## Abiotrophia                          1.510126e-01 0.332914146
## Granulicatella                       8.061586e-01 0.846859541
## Lactobacillus                        2.878684e-02 0.121405374
## Streptococcus                        3.811488e-02 0.142197807
## Order.Lactobacillales.               3.401902e-01 0.540958251
## Class.Bacilli.                       5.724851e-01 0.694138148
## Clostridiales_[F.1][G-1]             2.136932e-01 0.398619951
## Butyrivibrio                         8.119375e-01 0.846859541
## Catonella                            6.637623e-01 0.755270466
## Johnsonella                          3.706383e-02 0.142197807
## Lachnoanaerobaculum                  8.089161e-01 0.846859541
## Lachnospiraceae_[G.2]                2.569080e-02 0.113273080
## Lachnospiraceae_[G.3]                2.044085e-01 0.388776973
## Lachnospiraceae_[G.7]                1.131178e-01 0.267620177
## Lachnospiraceae_[G.8]                5.487865e-02 0.171717064
## Oribacterium                         6.577749e-01 0.755270466
```

```
## Shuttleworthia                          1.817433e-03 0.044072761
## Stomatobaculum                          5.265740e-01 0.674174607
## Family.Lachnospiraceae_[XIV].           3.996836e-02 0.143590029
## Peptococcus                             2.932465e-01 0.496049119
## Parvimonas                              5.131838e-02 0.171651117
## Filifactor                              8.009178e-01 0.846859541
## Mogibacterium                           6.774075e-01 0.755270466
## Peptostreptococcaceae_[XI][G.1]         1.772110e-01 0.353099053
## Peptostreptococcaceae_[XI][G.4]         7.908079e-01 0.846859541
## Peptostreptococcaceae_[XI][G.5]         8.407459e-01 0.866622285
## Peptostreptococcaceae_[XI][G.6]         4.016946e-01 0.573005463
## Peptostreptococcaceae_[XI][G.7]         8.487538e-01 0.866622285
## Peptostreptococcaceae_[XI][G.9]         7.265233e-02 0.213553832
## Peptostreptococcus                      8.343561e-02 0.231235846
## Ruminococcaceae_[G.1]                   1.076054e-01 0.260943110
## Ruminococcaceae_[G.2]                   5.986574e-01 0.708167957
## Bulleidia                               1.938649e-01 0.376097875
## Eggerthia                               2.454005e-01 0.449129198
## Solobacterium                           4.583523e-01 0.612827230
## Mycoplasma                              3.537388e-02 0.142197807
## Mitsuokella                             2.309069e-02 0.113273080
## Selenomonas                             1.493325e-02 0.110393526
## Family.Selenomonadaceae.                1.549843e-01 0.333879001
## Class.Negativicutes.                    3.788053e-01 0.565294002
## Anaeroglobus                            8.936380e-02 0.239308083
## Dialister                               2.064514e-02 0.113273080
## Megasphaera                             5.634727e-01 0.691858866
## Family.Veillonellaceae.                 2.415095e-02 0.113273080
## Veillonella                             3.621031e-01 0.562752657
## Veillonellaceae_[G.1]                   3.631622e-05 0.003522674
## Phylum.Firmicutes.                      3.881741e-01 0.570498247
## Fusobacterium                           4.469171e-01 0.612827230
## Leptotrichia                            5.580586e-01 0.691858866
## Gracilibacteria_(GN02)_[G.1]            5.282193e-01 0.674174607
## Lautropia                               6.382062e-01 0.745855383
## Ottowia                                 7.992970e-01 0.846859541
## Eikenella                               9.357603e-01 0.935760321
## Kingella                                1.055665e-01 0.260943110
## Neisseria                               5.417247e-02 0.171717064
## Family.Neisseriaceae.                   9.374956e-02 0.239308083
## Campylobacter                           5.387468e-01 0.678681002
## Cardiobacterium                         2.587215e-01 0.464740525
## Aggregatibacter                         1.175651e-01 0.271519511
## Haemophilus                             1.617764e-01 0.333879001
## Family.Pasteurellaceae.                 4.229776e-01 0.594620720
## Saccharibacteria_(TM7)_[G.1]            9.296640e-01 0.935760321
## Saccharibacteria_(TM7)_[G.3]            6.708352e-01 0.755270466
## Saccharibacteria_(TM7)_[G.6]            1.476043e-01 0.332914146
## Family.Saccharibacteria_(TM7)_[F-1]. 2.652662e-01 0.467833073
## Saccharibacteria_(TM7)_[G.5]            6.665571e-02 0.202050136
## Treponema                               8.930764e-04 0.043314207
## Fretibacterium                          1.906999e-02 0.113273080
## Kingdom.Bacteria.                       1.370908e-02 0.110393526
## Others                                  2.505155e-02 0.113273080
```

```
##                                                                   Feature
## Absconditabacteria_(SR1)_[G.1]             Absconditabacteria_(SR1)_[G.1]
## Actinomyces                                                   Actinomyces
## Peptidiphaga                                                 Peptidiphaga
## Schaalia                                                         Schaalia
## Family.Actinomycetaceae.                         Family.Actinomycetaceae.
## Rothia                                                             Rothia
## Alloscardovia                                               Alloscardovia
## Bifidobacterium                                           Bifidobacterium
## Parascardovia                                               Parascardovia
## Scardovia                                                       Scardovia
## Corynebacterium                                           Corynebacterium
## Olsenella                                                       Olsenella
## Atopobium                                                       Atopobium
## Slackia                                                           Slackia
## Cryptobacterium                                           Cryptobacterium
## Bacteroidetes_[G.3]                                   Bacteroidetes_[G.3]
## Bacteroidetes_[G.5]                                   Bacteroidetes_[G.5]
## Family.Bacteroidetes_[F-1].                   Family.Bacteroidetes_[F-1].
## Bacteroidaceae_[G.1]                                 Bacteroidaceae_[G.1]
## Bacteroidales_[G.2]                                   Bacteroidales_[G.2]
## Porphyromonas                                               Porphyromonas
## Tannerella                                                     Tannerella
## Alloprevotella                                             Alloprevotella
## Prevotella                                                     Prevotella
## Bergeyella                                                     Bergeyella
## Capnocytophaga                                             Capnocytophaga
## Gemella                                                           Gemella
## Abiotrophia                                                   Abiotrophia
## Granulicatella                                             Granulicatella
## Lactobacillus                                               Lactobacillus
## Streptococcus                                               Streptococcus
## Order.Lactobacillales.                             Order.Lactobacillales.
## Class.Bacilli.                                             Class.Bacilli.
## Clostridiales_[F.1][G-1]                         Clostridiales_[F.1][G-1]
## Butyrivibrio                                                 Butyrivibrio
## Catonella                                                       Catonella
## Johnsonella                                                   Johnsonella
## Lachnoanaerobaculum                                   Lachnoanaerobaculum
## Lachnospiraceae_[G.2]                               Lachnospiraceae_[G.2]
## Lachnospiraceae_[G.3]                               Lachnospiraceae_[G.3]
## Lachnospiraceae_[G.7]                               Lachnospiraceae_[G.7]
## Lachnospiraceae_[G.8]                               Lachnospiraceae_[G.8]
## Oribacterium                                                 Oribacterium
## Shuttleworthia                                             Shuttleworthia
## Stomatobaculum                                             Stomatobaculum
## Family.Lachnospiraceae_[XIV].                 Family.Lachnospiraceae_[XIV].
## Peptococcus                                                   Peptococcus
## Parvimonas                                                     Parvimonas
## Filifactor                                                     Filifactor
## Mogibacterium                                               Mogibacterium
## Peptostreptococcaceae_[XI][G.1]           Peptostreptococcaceae_[XI][G.1]
## Peptostreptococcaceae_[XI][G.4]           Peptostreptococcaceae_[XI][G.4]
## Peptostreptococcaceae_[XI][G.5]           Peptostreptococcaceae_[XI][G.5]
```

```
## Peptostreptococcaceae_[XI][G.6]          Peptostreptococcaceae_[XI][G.6]
## Peptostreptococcaceae_[XI][G.7]          Peptostreptococcaceae_[XI][G.7]
## Peptostreptococcaceae_[XI][G.9]          Peptostreptococcaceae_[XI][G.9]
## Peptostreptococcus                                   Peptostreptococcus
## Ruminococcaceae_[G.1]                             Ruminococcaceae_[G.1]
## Ruminococcaceae_[G.2]                             Ruminococcaceae_[G.2]
## Bulleidia                                                     Bulleidia
## Eggerthia                                                     Eggerthia
## Solobacterium                                             Solobacterium
## Mycoplasma                                                   Mycoplasma
## Mitsuokella                                                 Mitsuokella
## Selenomonas                                                 Selenomonas
## Family.Selenomonadaceae.                       Family.Selenomonadaceae.
## Class.Negativicutes.                               Class.Negativicutes.
## Anaeroglobus                                               Anaeroglobus
## Dialister                                                     Dialister
## Megasphaera                                                 Megasphaera
## Family.Veillonellaceae.                         Family.Veillonellaceae.
## Veillonella                                                 Veillonella
## Veillonellaceae_[G.1]                             Veillonellaceae_[G.1]
## Phylum.Firmicutes.                                   Phylum.Firmicutes.
## Fusobacterium                                             Fusobacterium
## Leptotrichia                                               Leptotrichia
## Gracilibacteria_(GN02)_[G.1]               Gracilibacteria_(GN02)_[G.1]
## Lautropia                                                     Lautropia
## Ottowia                                                         Ottowia
## Eikenella                                                     Eikenella
## Kingella                                                       Kingella
## Neisseria                                                     Neisseria
## Family.Neisseriaceae.                             Family.Neisseriaceae.
## Campylobacter                                             Campylobacter
## Cardiobacterium                                         Cardiobacterium
## Aggregatibacter                                         Aggregatibacter
## Haemophilus                                                 Haemophilus
## Family.Pasteurellaceae.                         Family.Pasteurellaceae.
## Saccharibacteria_(TM7)_[G.1]               Saccharibacteria_(TM7)_[G.1]
## Saccharibacteria_(TM7)_[G.3]               Saccharibacteria_(TM7)_[G.3]
## Saccharibacteria_(TM7)_[G.6]               Saccharibacteria_(TM7)_[G.6]
## Family.Saccharibacteria_(TM7)_[F-1]. Family.Saccharibacteria_(TM7)_[F-1].
## Saccharibacteria_(TM7)_[G.5]               Saccharibacteria_(TM7)_[G.5]
## Treponema                                                     Treponema
## Fretibacterium                                           Fretibacterium
## Kingdom.Bacteria.                                     Kingdom.Bacteria.
## Others                                                           Others
##                                                      Method
## Absconditabacteria_(SR1)_[G.1]       Kruskal-Wallis (kru)
## Actinomyces                          Kruskal-Wallis (kru)
## Peptidiphaga                         Kruskal-Wallis (kru)
## Schaalia                             Kruskal-Wallis (kru)
## Family.Actinomycetaceae.             Kruskal-Wallis (kru)
## Rothia                               Kruskal-Wallis (kru)
## Alloscardovia                        Kruskal-Wallis (kru)
## Bifidobacterium                      Kruskal-Wallis (kru)
## Parascardovia                        Kruskal-Wallis (kru)
```

```
## Scardovia                             Kruskal-Wallis (kru)
## Corynebacterium                       Kruskal-Wallis (kru)
## Olsenella                             Kruskal-Wallis (kru)
## Atopobium                             Kruskal-Wallis (kru)
## Slackia                               Kruskal-Wallis (kru)
## Cryptobacterium                       Kruskal-Wallis (kru)
## Bacteroidetes_[G.3]                   Kruskal-Wallis (kru)
## Bacteroidetes_[G.5]                   Kruskal-Wallis (kru)
## Family.Bacteroidetes_[F-1].           Kruskal-Wallis (kru)
## Bacteroidaceae_[G.1]                  Kruskal-Wallis (kru)
## Bacteroidales_[G.2]                   Kruskal-Wallis (kru)
## Porphyromonas                         Kruskal-Wallis (kru)
## Tannerella                            Kruskal-Wallis (kru)
## Alloprevotella                        Kruskal-Wallis (kru)
## Prevotella                            Kruskal-Wallis (kru)
## Bergeyella                            Kruskal-Wallis (kru)
## Capnocytophaga                        Kruskal-Wallis (kru)
## Gemella                               Kruskal-Wallis (kru)
## Abiotrophia                           Kruskal-Wallis (kru)
## Granulicatella                        Kruskal-Wallis (kru)
## Lactobacillus                         Kruskal-Wallis (kru)
## Streptococcus                         Kruskal-Wallis (kru)
## Order.Lactobacillales.                Kruskal-Wallis (kru)
## Class.Bacilli.                        Kruskal-Wallis (kru)
## Clostridiales_[F.1][G-1]              Kruskal-Wallis (kru)
## Butyrivibrio                          Kruskal-Wallis (kru)
## Catonella                             Kruskal-Wallis (kru)
## Johnsonella                           Kruskal-Wallis (kru)
## Lachnoanaerobaculum                   Kruskal-Wallis (kru)
## Lachnospiraceae_[G.2]                 Kruskal-Wallis (kru)
## Lachnospiraceae_[G.3]                 Kruskal-Wallis (kru)
## Lachnospiraceae_[G.7]                 Kruskal-Wallis (kru)
## Lachnospiraceae_[G.8]                 Kruskal-Wallis (kru)
## Oribacterium                          Kruskal-Wallis (kru)
## Shuttleworthia                        Kruskal-Wallis (kru)
## Stomatobaculum                        Kruskal-Wallis (kru)
## Family.Lachnospiraceae_[XIV].         Kruskal-Wallis (kru)
## Peptococcus                           Kruskal-Wallis (kru)
## Parvimonas                            Kruskal-Wallis (kru)
## Filifactor                            Kruskal-Wallis (kru)
## Mogibacterium                         Kruskal-Wallis (kru)
## Peptostreptococcaceae_[XI][G.1]       Kruskal-Wallis (kru)
## Peptostreptococcaceae_[XI][G.4]       Kruskal-Wallis (kru)
## Peptostreptococcaceae_[XI][G.5]       Kruskal-Wallis (kru)
## Peptostreptococcaceae_[XI][G.6]       Kruskal-Wallis (kru)
## Peptostreptococcaceae_[XI][G.7]       Kruskal-Wallis (kru)
## Peptostreptococcaceae_[XI][G.9]       Kruskal-Wallis (kru)
## Peptostreptococcus                    Kruskal-Wallis (kru)
## Ruminococcaceae_[G.1]                 Kruskal-Wallis (kru)
## Ruminococcaceae_[G.2]                 Kruskal-Wallis (kru)
## Bulleidia                             Kruskal-Wallis (kru)
## Eggerthia                             Kruskal-Wallis (kru)
## Solobacterium                         Kruskal-Wallis (kru)
## Mycoplasma                            Kruskal-Wallis (kru)
```

```
## Mitsuokella                            Kruskal-Wallis (kru)
## Selenomonas                            Kruskal-Wallis (kru)
## Family.Selenomonadaceae.               Kruskal-Wallis (kru)
## Class.Negativicutes.                   Kruskal-Wallis (kru)
## Anaeroglobus                           Kruskal-Wallis (kru)
## Dialister                              Kruskal-Wallis (kru)
## Megasphaera                            Kruskal-Wallis (kru)
## Family.Veillonellaceae.                Kruskal-Wallis (kru)
## Veillonella                            Kruskal-Wallis (kru)
## Veillonellaceae_[G.1]                  Kruskal-Wallis (kru)
## Phylum.Firmicutes.                     Kruskal-Wallis (kru)
## Fusobacterium                          Kruskal-Wallis (kru)
## Leptotrichia                           Kruskal-Wallis (kru)
## Gracilibacteria_(GN02)_[G.1]           Kruskal-Wallis (kru)
## Lautropia                              Kruskal-Wallis (kru)
## Ottowia                                Kruskal-Wallis (kru)
## Eikenella                              Kruskal-Wallis (kru)
## Kingella                               Kruskal-Wallis (kru)
## Neisseria                              Kruskal-Wallis (kru)
## Family.Neisseriaceae.                  Kruskal-Wallis (kru)
## Campylobacter                          Kruskal-Wallis (kru)
## Cardiobacterium                        Kruskal-Wallis (kru)
## Aggregatibacter                        Kruskal-Wallis (kru)
## Haemophilus                            Kruskal-Wallis (kru)
## Family.Pasteurellaceae.                Kruskal-Wallis (kru)
## Saccharibacteria_(TM7)_[G.1]           Kruskal-Wallis (kru)
## Saccharibacteria_(TM7)_[G.3]           Kruskal-Wallis (kru)
## Saccharibacteria_(TM7)_[G.6]           Kruskal-Wallis (kru)
## Family.Saccharibacteria_(TM7)_[F-1]. Kruskal-Wallis (kru)
## Saccharibacteria_(TM7)_[G.5]           Kruskal-Wallis (kru)
## Treponema                              Kruskal-Wallis (kru)
## Fretibacterium                         Kruskal-Wallis (kru)
## Kingdom.Bacteria.                      Kruskal-Wallis (kru)
## Others                                 Kruskal-Wallis (kru)
```

```
write.table(final, file="hba1c_cat_kru.txt", sep="\t", dec=",", row.names=F)
write.table(final[final$pval<0.05,], file="hba1c_cat_kru_sig.txt", sep="\t", dec=",", row.names=F)
```

sPLS-DA mixOmics

```
rm(list=setdiff(ls(), c("FeaturePic2", "Pic2", "Phe", "Microbio", "FeatureMic", "Metabo", "paretoscale"

set.seed(99) # for reproducibility, remove for normal use

Microbio2<-Microbio
#Subset select or just remove NA
table(Phe$Smoking, useNA="always")
```

Smoking

```
##
```

```
##  Exsmoker Nonsmoker    Smoker      <NA>
##       351       261       129         5
```

```
Phe2<-Phe[complete.cases(Phe$Smoking), ] #NA
Phe2<-subset(Phe2, Smoking %in% c("Smoker", "Nonsmoker")) #also removes NA, but above can be easier to
X<-dplyr::select(Microbio2, one_of(Phe2$IDX)) #Also in X
##Remove orgs that are not present after subsetting.
X <- X[rowSums(X)>0,]


#Hellinger transformation
X <- data.frame(t(decostand(t(X), method="hellinger")))
#Maks TSS
X<-sweep(X, 2, colSums(X), FUN="/")
#rowSums(X)
sum(colnames(X)!=Phe2$IDX)==0
```

```
## [1] TRUE
```

```
X<-t(X)
Y<-Phe2$Smoking

sum(rownames(X)!=Phe2$IDX)==0
```

```
## [1] TRUE
```

```
dim(X) # check the dimensions of the X dataframe
```

```
## [1] 390  97
```

```
summary(Y) # check the distribution of class labels
```

```
##    Length     Class      Mode
##       390 character character
```

```
# Barplot of the variance each principal component explains
pca.addp = pca(X, ncomp = 10, center = TRUE, scale = TRUE) # run pca method on data
plot(pca.addp)  # barplot of the eigenvalues (explained variance per component)
```

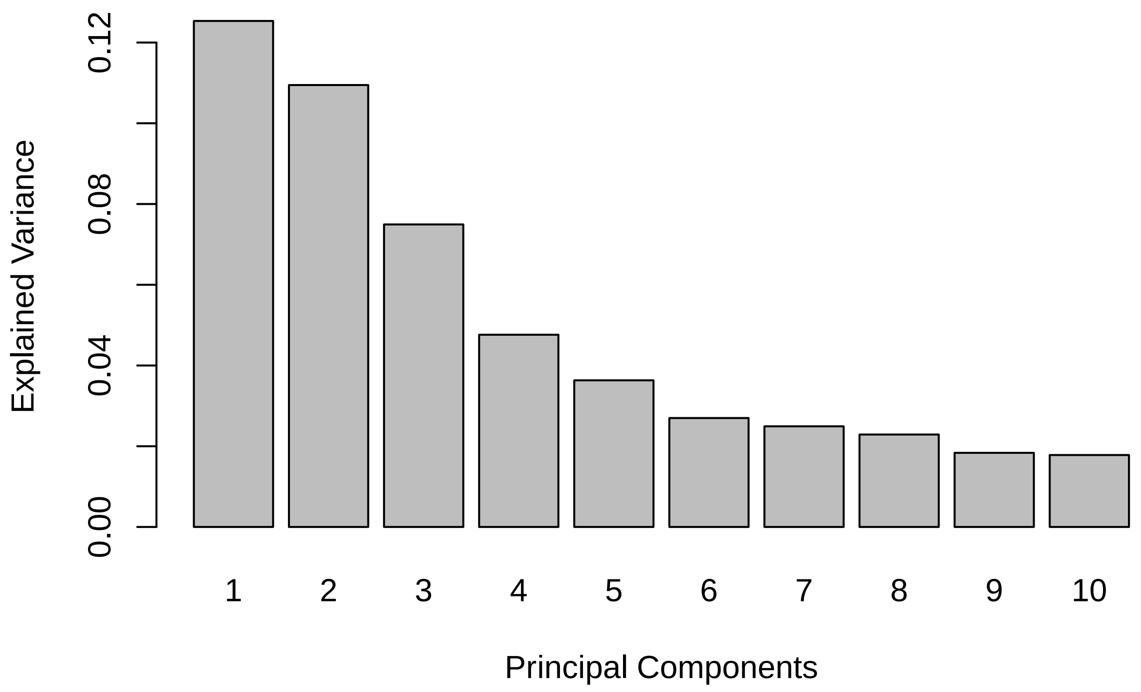


```
# Preliminary (unsupervised) analysis with PCA
plotIndiv(pca.addp, group = Y, ind.names = FALSE, # plot the samples projected
          legend = TRUE, title = 'PCA on addp, comp 1 - 2') # onto the PCA subspace
```

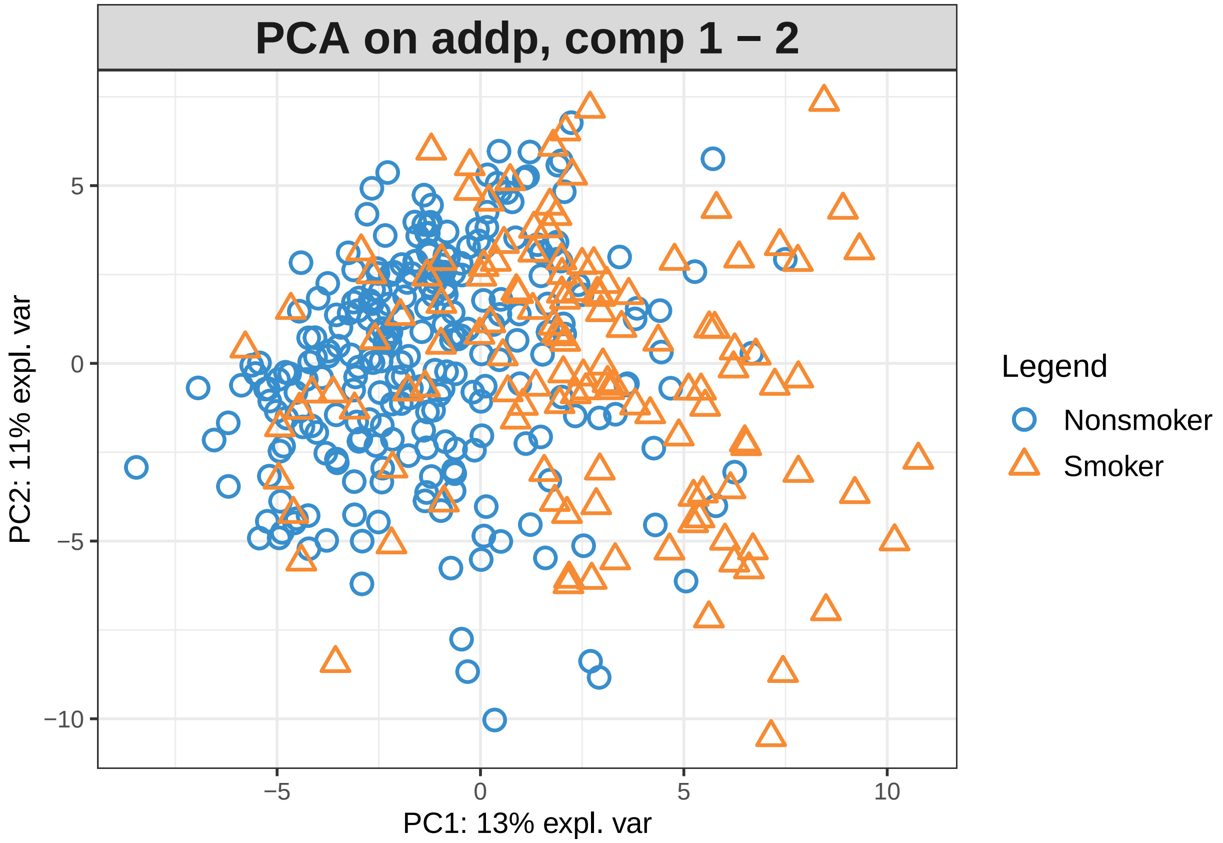


```
addp.splsda <- splsda(X, Y, ncomp = 10)  # set ncomp to 10 for performance assessment later

## Sample plots after a basic PLS-DA model was operated on this data.
# plot the samples projected onto the first two components of the PLS-DA subspace
plotIndiv(addp.splsda , comp = 1:2,
          group = Y, ind.names = FALSE,  # colour points by class
          ellipse = TRUE, # include 95% confidence ellipse for each class
          legend = TRUE, title = '(a) PLSDA with confidence ellipses')
```

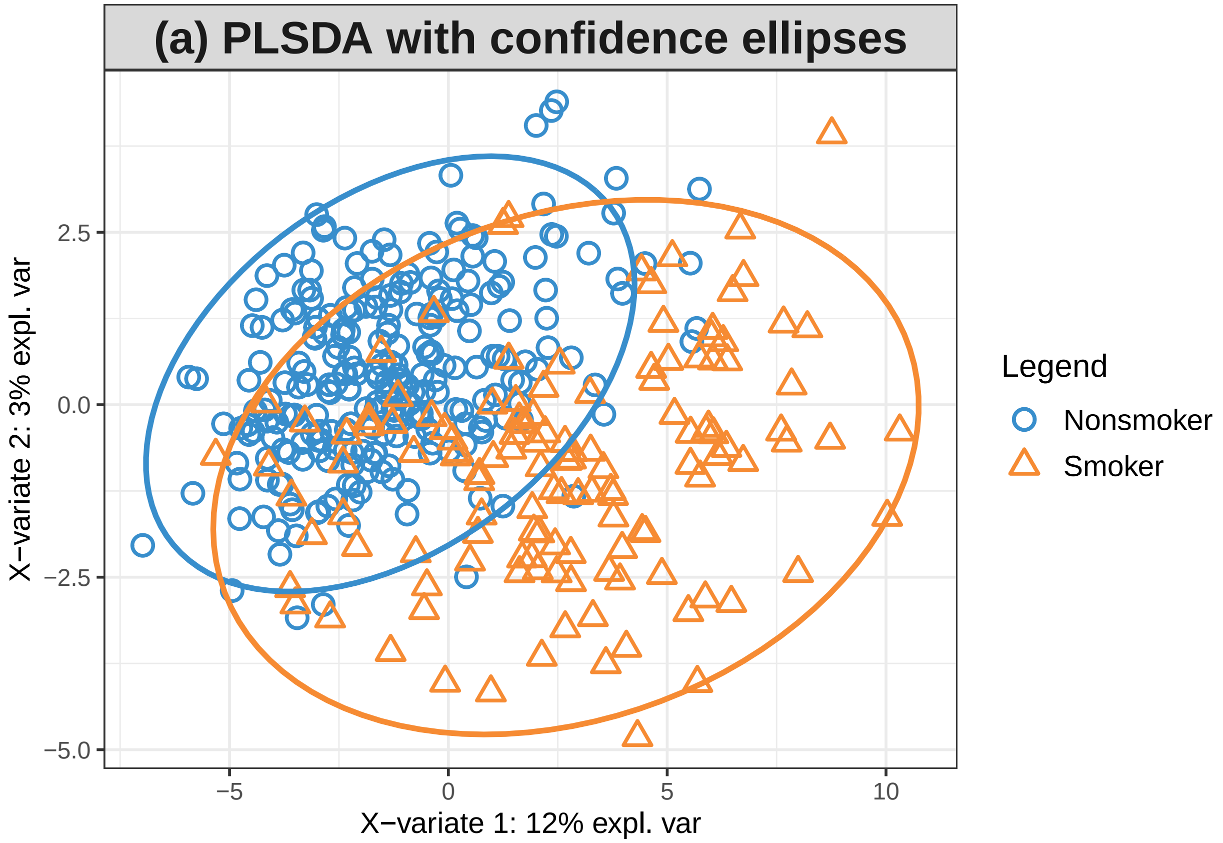


```
# use the max.dist measure to form decision boundaries between classes based on PLS-DA data
background = background.predict(addp.splsda, comp.predicted=2, dist = "max.dist")

# plot the samples projected onto the first two components of the PLS-DA subspace
plotIndiv(addp.splsda, comp = 1:2,
          group = Y, ind.names = FALSE, # colour points by class
          background = background, # include prediction background for each class
          legend = TRUE, title = " (b) PLSDA with prediction background")
```

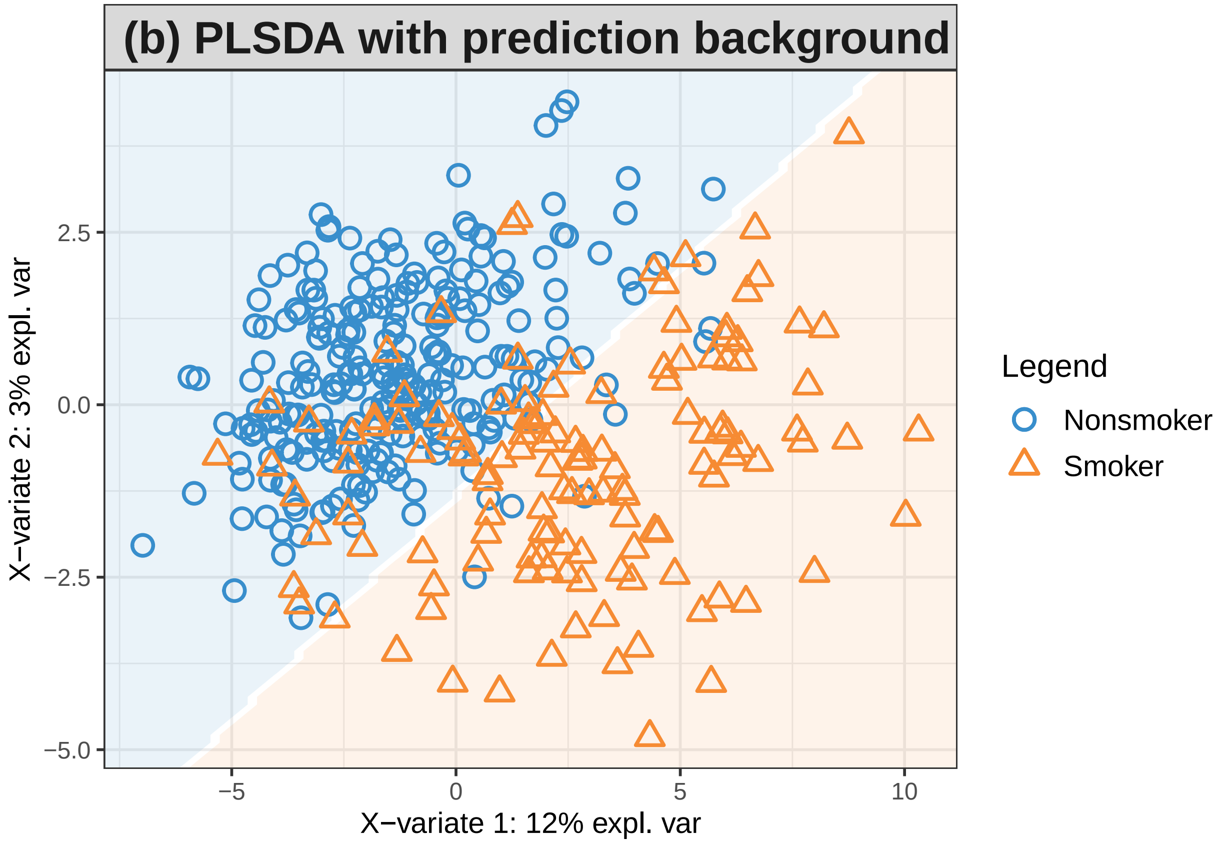


```r
## Tuning the number of components in PLS-DA. For each component, repeated cross-validation (10 × 3-fol
# undergo performance evaluation in order to tune the number of components to use
perf.splsda.addp <- perf(addp.splsda, validation = "Mfold",
                          folds = 5, nrepeat = 10, # use repeated cross-validation
                          progressBar = FALSE, auc = TRUE) # include AUC values

# plot the outcome of performance evaluation across all ten components
plot(perf.splsda.addp, col = color.mixo(5:7), sd = TRUE,
     legend.position = "horizontal")
```

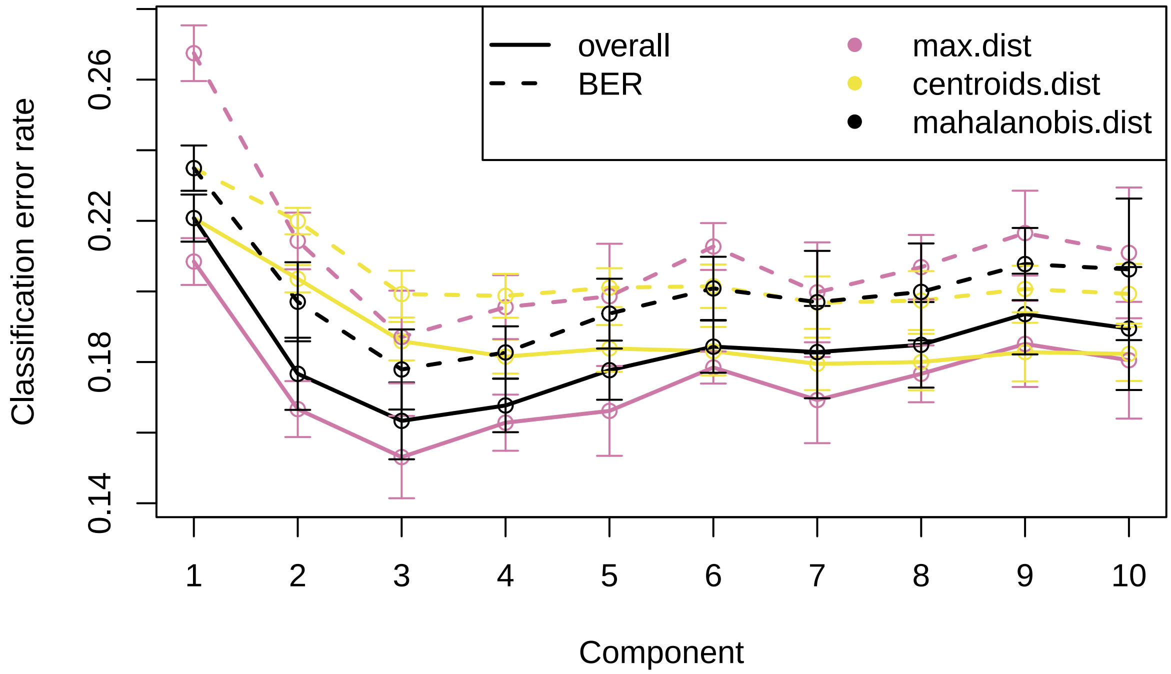


```
perf.splsda.addp$choice.ncomp # what is the optimal value of components according to perf()
```

```
##          max.dist centroids.dist mahalanobis.dist
## overall         3              3                3
## BER             3              3                3
```

```
## Tuning keepX for the sPLS-DA. Each coloured line represents the balanced error rate (y-axis) per comp
# grid of possible keepX values that will be tested for each component
list.keepX <- c(1:10,  seq(20, 300, 10))

# undergo the tuning process to determine the optimal number of variables
tune.splsda.addp <- tune.splsda(X, Y, ncomp = 4, # calculate for first 4 components
                                validation = 'Mfold',
                                folds = 5, nrepeat = 10, # use repeated cross-validation
                                dist = 'max.dist', # use max.dist measure
                                measure = "BER", # use balanced error rate of dist measure
                                test.keepX = list.keepX,
                                cpus = 2 # allow for parallelisation to decrease runtime
                                )

plot(tune.splsda.addp, col = color.jet(4))
```

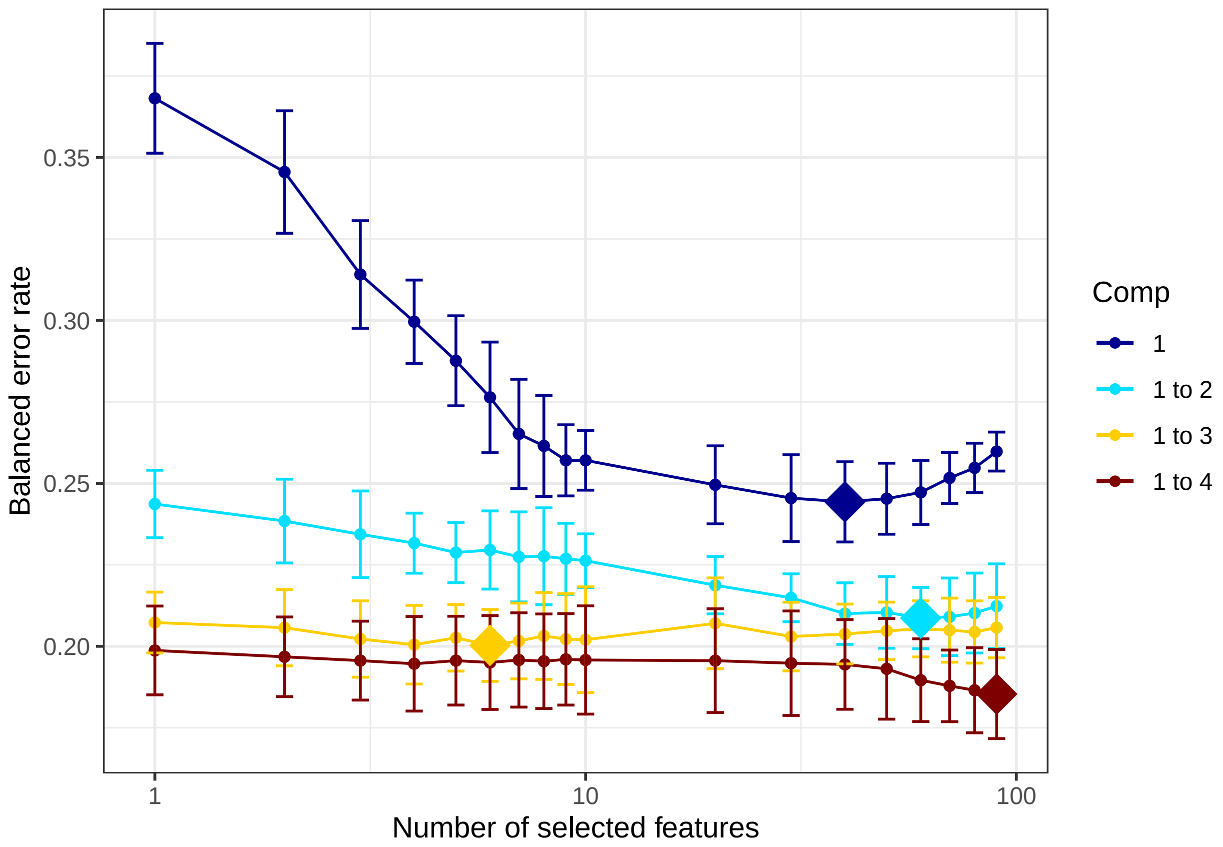


```
tune.splsda.addp$choice.ncomp$ncomp # what is the optimal value of components according to tune.splsda(
```

```
## [1] 4
```

```
tune.splsda.addp$choice.keepX # what are the optimal values of variables according to tune.splsda()
```

```
## comp1 comp2 comp3 comp4
##    40    60     6    90
```

```
optimal.ncomp <- tune.splsda.addp$choice.ncomp$ncomp
optimal.keepX <- tune.splsda.addp$choice.keepX[1:optimal.ncomp]

# form final model with optimised values for component and variable count
final.splsda <- splsda(X, Y,
                       ncomp = optimal.ncomp,
                       keepX = optimal.keepX)

#########Loadings
#plotLoadings(final.splsda, comp=1, contrib = 'max', method = 'mean', size.title = 1)
#plotLoadings(final.splsda, comp=2, contrib = 'max', method = 'mean', size.title = 1)

comp1 <- plotLoadings(final.splsda, comp = 1, method = 'mean', contrib = 'max',
                      size.title = 1)
```

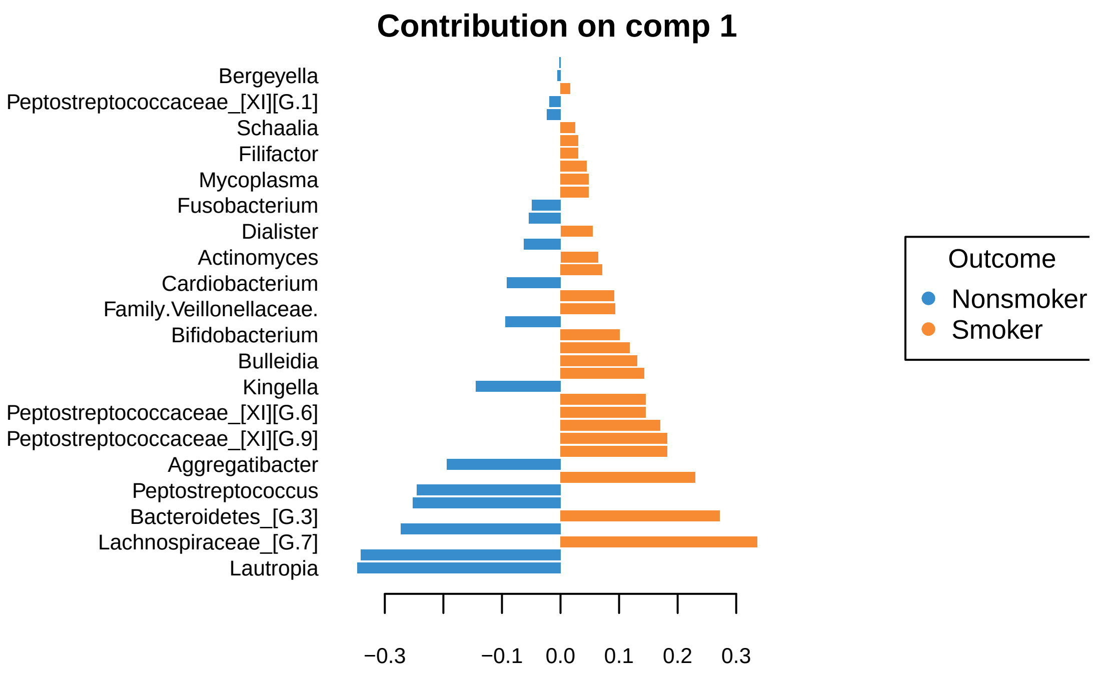


```r
head(comp1, n=20)
```

```
##                                  Nonsmoker     Smoker Contrib.Nonsmoker
## Lautropia                        0.2726922 -0.5517260              TRUE
## Neisseria                        0.2702474 -0.5467797              TRUE
## Lachnospiraceae_[G.7]           -0.2679546  0.5421406             FALSE
## Family.Actinomycetaceae.         0.2426594 -0.4909621              TRUE
## Bacteroidetes_[G.3]             -0.2424461  0.4905304             FALSE
## Catonella                        0.2346052 -0.4746663              TRUE
## Peptostreptococcus               0.2316714 -0.4687305              TRUE
## Alloscardovia                   -0.2256459  0.4565393             FALSE
## Aggregatibacter                  0.2114528 -0.4278231              TRUE
## Fretibacterium                  -0.2063973  0.4175945             FALSE
## Peptostreptococcaceae_[XI][G.9] -0.2063858  0.4175713             FALSE
## Atopobium                       -0.2018172  0.4083278             FALSE
## Peptostreptococcaceae_[XI][G.6] -0.1917780  0.3880159             FALSE
## Bacteroidales_[G.2]             -0.1916270  0.3877105             FALSE
## Kingella                         0.1914568 -0.3873660              TRUE
## Treponema                       -0.1906569  0.3857476             FALSE
## Bulleidia                       -0.1858314  0.3759844             FALSE
## Cryptobacterium                 -0.1806656  0.3655327             FALSE
## Bifidobacterium                 -0.1739692  0.3519843             FALSE
## Abiotrophia                      0.1712291 -0.3464402              TRUE
##                                 Contrib.Smoker Contrib GroupContrib   color
## Lautropia                                FALSE   FALSE    Nonsmoker #388ECC
```

```
## Neisseria                             FALSE   FALSE    Nonsmoker #388ECC
## Lachnospiraceae_[G.7]                  TRUE   FALSE       Smoker #F68B33
## Family.Actinomycetaceae.              FALSE   FALSE    Nonsmoker #388ECC
## Bacteroidetes_[G.3]                    TRUE   FALSE       Smoker #F68B33
## Catonella                             FALSE   FALSE    Nonsmoker #388ECC
## Peptostreptococcus                    FALSE   FALSE    Nonsmoker #388ECC
## Alloscardovia                          TRUE   FALSE       Smoker #F68B33
## Aggregatibacter                       FALSE   FALSE    Nonsmoker #388ECC
## Fretibacterium                         TRUE   FALSE       Smoker #F68B33
## Peptostreptococcaceae_[XI][G.9]        TRUE   FALSE       Smoker #F68B33
## Atopobium                              TRUE   FALSE       Smoker #F68B33
## Peptostreptococcaceae_[XI][G.6]        TRUE   FALSE       Smoker #F68B33
## Bacteroidales_[G.2]                    TRUE   FALSE       Smoker #F68B33
## Kingella                              FALSE   FALSE    Nonsmoker #388ECC
## Treponema                              TRUE   FALSE       Smoker #F68B33
## Bulleidia                              TRUE   FALSE       Smoker #F68B33
## Cryptobacterium                        TRUE   FALSE       Smoker #F68B33
## Bifidobacterium                        TRUE   FALSE       Smoker #F68B33
## Abiotrophia                           FALSE   FALSE    Nonsmoker #388ECC
##                                  importance
## Lautropia                       -0.34756805
## Neisseria                       -0.34146615
## Lachnospiraceae_[G.7]            0.33574340
## Family.Actinomycetaceae.        -0.27260899
## Bacteroidetes_[G.3]              0.27207646
## Catonella                       -0.25250622
## Peptostreptococcus              -0.24518383
## Alloscardovia                    0.23014457
## Aggregatibacter                 -0.19471986
## Fretibacterium                   0.18210182
## Peptostreptococcaceae_[XI][G.9]  0.18207311
## Atopobium                        0.17067028
## Peptostreptococcaceae_[XI][G.6]  0.14561325
## Bacteroidales_[G.2]              0.14523655
## Kingella                        -0.14481159
## Treponema                        0.14281506
## Bulleidia                        0.13077107
## Cryptobacterium                  0.11787776
## Bifidobacterium                  0.10116420
## Abiotrophia                     -0.09432499
```

```
write.table(comp1, file="Smoking_comp1.txt", sep="\t", dec=",", row.names=T)
comp2 <- plotLoadings(final.splsda, comp = 2, method = 'mean', contrib = 'max',
                      size.title = 1)
```

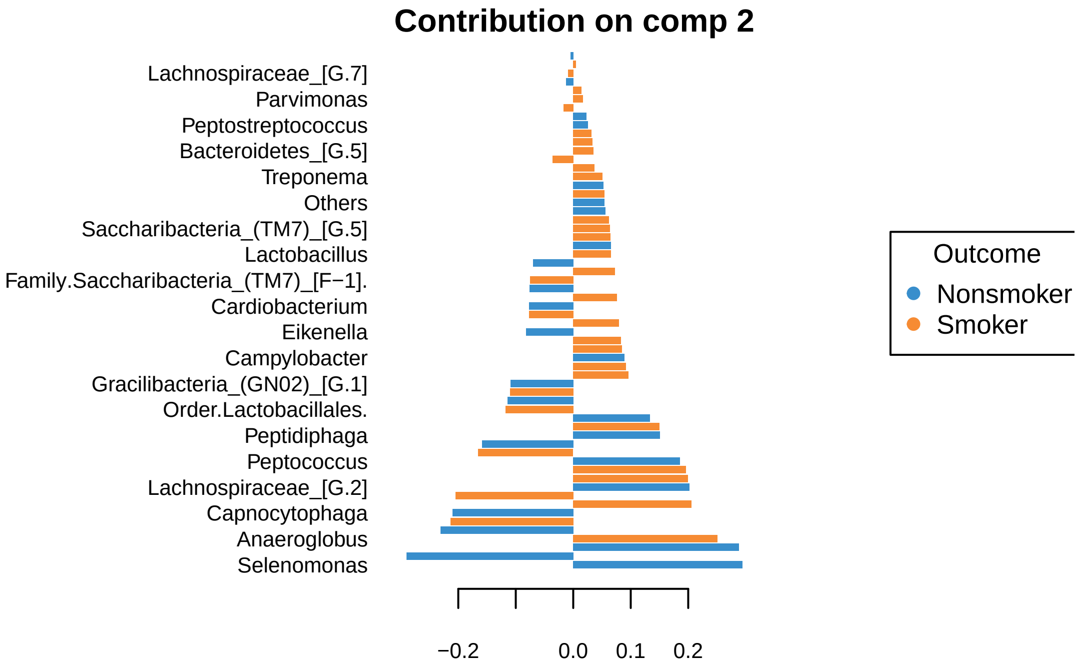


```
head(comp2, n=20)
```

```
##                                 Nonsmoker      Smoker Contrib.Nonsmoker
## Selenomonas                   0.085353051 -0.17269106              TRUE
## Bergeyella                    0.135645393 -0.27444533              TRUE
## Family.Actinomycetaceae.      0.242659447 -0.49096214              TRUE
## Anaeroglobus                 -0.077435667  0.15667216             FALSE
## Porphyromonas                 0.112876966 -0.22837898              TRUE
## Corynebacterium              -0.021886921  0.04428284             FALSE
## Capnocytophaga                0.128847773 -0.26069201              TRUE
## Scardovia                    -0.066370983  0.13428548             FALSE
## Granulicatella               -0.123541993  0.24995705             FALSE
## Lachnospiraceae_[G.2]         0.121910551 -0.24665623              TRUE
## Prevotella                   -0.063598908  0.12867686             FALSE
## Veillonellaceae_[G.1]        -0.050974159  0.10313376             FALSE
## Peptococcus                   0.142950170 -0.28922476              TRUE
## Rothia                       -0.170273785  0.34450742             FALSE
## Haemophilus                   0.074715836 -0.15116925              TRUE
## Peptidiphaga                  0.158759900 -0.32121189              TRUE
## Veillonella                  -0.132070487  0.26721238             FALSE
## Family.Selenomonadaceae.      0.115691099 -0.23407269              TRUE
## Order.Lactobacillales.       -0.007886563  0.01595653             FALSE
## Abiotrophia                   0.171229064 -0.34644020              TRUE
##                              Contrib.Smoker Contrib GroupContrib   color importance
## Selenomonas                           FALSE   FALSE    Nonsmoker #388ECC  0.2940747
```

```
## Bergeyella                         FALSE    FALSE    Nonsmoker #388ECC -0.2904094
## Family.Actinomycetaceae.           FALSE    FALSE    Nonsmoker #388ECC  0.2882483
## Anaeroglobus                        TRUE    FALSE       Smoker #F68B33  0.2509580
## Porphyromonas                      FALSE    FALSE    Nonsmoker #388ECC -0.2312762
## Corynebacterium                     TRUE    FALSE       Smoker #F68B33 -0.2138917
## Capnocytophaga                     FALSE    FALSE    Nonsmoker #388ECC -0.2096140
## Scardovia                           TRUE    FALSE       Smoker #F68B33  0.2057674
## Granulicatella                      TRUE    FALSE       Smoker #F68B33 -0.2045925
## Lachnospiraceae_[G.2]              FALSE    FALSE    Nonsmoker #388ECC  0.2024692
## Prevotella                          TRUE    FALSE       Smoker #F68B33  0.1998717
## Veillonellaceae_[G.1]               TRUE    FALSE       Smoker #F68B33  0.1959439
## Peptococcus                        FALSE    FALSE    Nonsmoker #388ECC  0.1854086
## Rothia                              TRUE    FALSE       Smoker #F68B33 -0.1652222
## Haemophilus                        FALSE    FALSE    Nonsmoker #388ECC -0.1584397
## Peptidiphaga                       FALSE    FALSE    Nonsmoker #388ECC  0.1504617
## Veillonella                         TRUE    FALSE       Smoker #F68B33  0.1504022
## Family.Selenomonadaceae.           FALSE    FALSE    Nonsmoker #388ECC  0.1332647
## Order.Lactobacillales.              TRUE    FALSE       Smoker #F68B33 -0.1179520
## Abiotrophia                        FALSE    FALSE    Nonsmoker #388ECC -0.1142662
```

```
write.table(comp2, file="Smoking_comp2.txt", sep="\t", dec=",", row.names=T)
comp3 <- plotLoadings(final.splsda, comp = 3, method = 'mean', contrib = 'max',
                      size.title = 1)
```

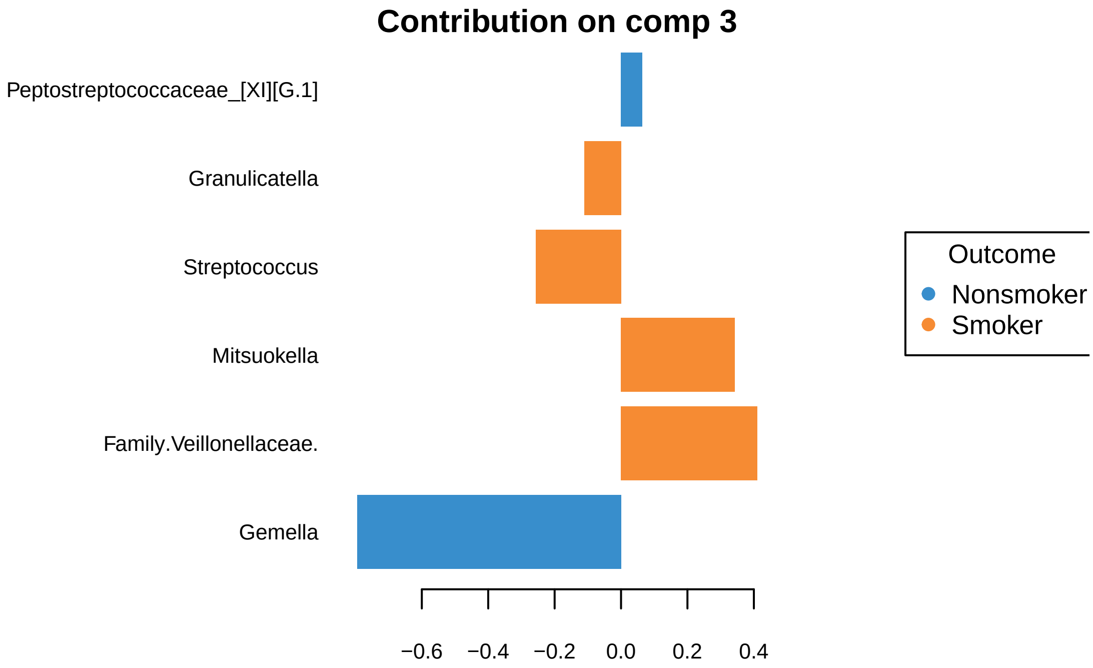

```r
head(comp3, n=20)
```

```
##                                     Nonsmoker     Smoker Contrib.Nonsmoker
## Gemella                            0.13424775 -0.2716176              TRUE
## Family.Veillonellaceae.           -0.17089467  0.3457636             FALSE
## Mitsuokella                       -0.09653153  0.1953080             FALSE
## Streptococcus                     -0.15285982  0.3092745             FALSE
## Granulicatella                    -0.12354199  0.2499571             FALSE
## Peptostreptococcaceae_[XI][G.1]    0.14126643 -0.2858181              TRUE
##                                   Contrib.Smoker Contrib GroupContrib   color
## Gemella                                    FALSE   FALSE    Nonsmoker #388ECC
## Family.Veillonellaceae.                     TRUE   FALSE       Smoker #F68B33
## Mitsuokella                                 TRUE   FALSE       Smoker #F68B33
## Streptococcus                               TRUE   FALSE       Smoker #F68B33
## Granulicatella                              TRUE   FALSE       Smoker #F68B33
## Peptostreptococcaceae_[XI][G.1]            FALSE   FALSE    Nonsmoker #388ECC
##                                    importance
## Gemella                           -0.79528314
## Family.Veillonellaceae.            0.40991677
## Mitsuokella                        0.34285534
## Streptococcus                     -0.25626638
## Granulicatella                    -0.11040561
## Peptostreptococcaceae_[XI][G.1]    0.06388527
```

```r
write.table(comp3, file="Smoking_comp3.txt", sep="\t", dec=",", row.names=T)
comp4 <- plotLoadings(final.splsda, comp = 4, method = 'mean', contrib = 'max',
                      size.title = 1)
```

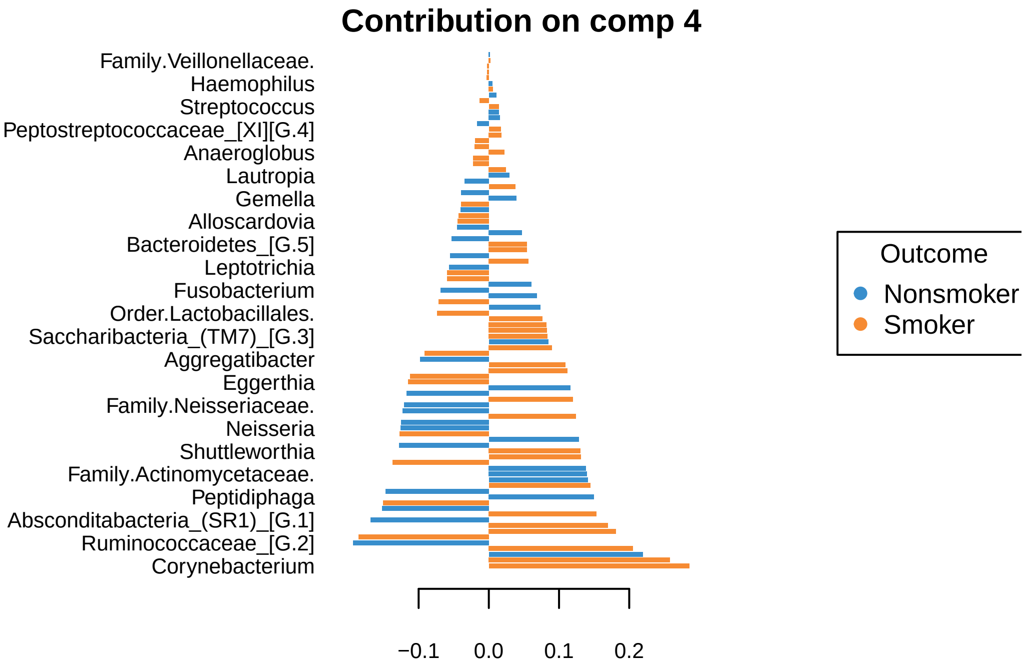


```r
head(comp4, n=20)
```

```
##                                  Nonsmoker      Smoker Contrib.Nonsmoker
## Corynebacterium               -0.021886921  0.04428284             FALSE
## Actinomyces                   -0.159091724  0.32188326             FALSE
## Stomatobaculum                 0.010086808 -0.02040819              TRUE
## Schaalia                      -0.143652171  0.29064509             FALSE
## Ruminococcaceae_[G.2]          0.057504411 -0.11634613              TRUE
## Parvimonas                    -0.041910234  0.08479512             FALSE
## Olsenella                     -0.068984083  0.13957245             FALSE
## Bacteroidales_[G.2]           -0.191627036  0.38771052             FALSE
## Absconditabacteria_(SR1)_[G.1]  0.122398690 -0.24764386              TRUE
## Family.Lachnospiraceae_[XIV].  -0.009669155  0.01956317             FALSE
## Bergeyella                     0.135645393 -0.27444533              TRUE
## Bulleidia                     -0.185831371  0.37598440             FALSE
## Peptidiphaga                   0.158759900 -0.32121189              TRUE
## Family.Pasteurellaceae.        0.049795199 -0.10074843              TRUE
## Mogibacterium                 -0.029231336  0.05914247             FALSE
## Catonella                      0.234605170 -0.47466627              TRUE
## Family.Actinomycetaceae.       0.242659447 -0.49096214              TRUE
## Butyrivibrio                   0.043823440 -0.08866603              TRUE
## Veillonella                   -0.132070487  0.26721238             FALSE
## Slackia                       -0.140143490  0.28354613             FALSE
##                               Contrib.Smoker Contrib GroupContrib   color
## Corynebacterium                         TRUE   FALSE       Smoker #F68B33
```

```
## Actinomyces                          TRUE   FALSE      Smoker #F68B33
## Stomatobaculum                      FALSE   FALSE   Nonsmoker #388ECC
## Schaalia                             TRUE   FALSE      Smoker #F68B33
## Ruminococcaceae_[G.2]               FALSE   FALSE   Nonsmoker #388ECC
## Parvimonas                           TRUE   FALSE      Smoker #F68B33
## Olsenella                            TRUE   FALSE      Smoker #F68B33
## Bacteroidales_[G.2]                  TRUE   FALSE      Smoker #F68B33
## Absconditabacteria_(SR1)_[G.1]      FALSE   FALSE   Nonsmoker #388ECC
## Family.Lachnospiraceae_[XIV].        TRUE   FALSE      Smoker #F68B33
## Bergeyella                          FALSE   FALSE   Nonsmoker #388ECC
## Bulleidia                            TRUE   FALSE      Smoker #F68B33
## Peptidiphaga                        FALSE   FALSE   Nonsmoker #388ECC
## Family.Pasteurellaceae.             FALSE   FALSE   Nonsmoker #388ECC
## Mogibacterium                        TRUE   FALSE      Smoker #F68B33
## Catonella                           FALSE   FALSE   Nonsmoker #388ECC
## Family.Actinomycetaceae.            FALSE   FALSE   Nonsmoker #388ECC
## Butyrivibrio                        FALSE   FALSE   Nonsmoker #388ECC
## Veillonella                          TRUE   FALSE      Smoker #F68B33
## Slackia                              TRUE   FALSE      Smoker #F68B33
##                                 importance
## Corynebacterium                  0.2856031
## Actinomyces                      0.2579829
## Stomatobaculum                   0.2193167
## Schaalia                         0.2052508
## Ruminococcaceae_[G.2]           -0.1930846
## Parvimonas                      -0.1858238
## Olsenella                        0.1812609
## Bacteroidales_[G.2]              0.1698564
## Absconditabacteria_(SR1)_[G.1] -0.1681472
## Family.Lachnospiraceae_[XIV].    0.1533623
## Bergeyella                      -0.1520305
## Bulleidia                       -0.1510275
## Peptidiphaga                     0.1498591
## Family.Pasteurellaceae.         -0.1469144
## Mogibacterium                    0.1444547
## Catonella                        0.1409776
## Family.Actinomycetaceae.         0.1399686
## Butyrivibrio                     0.1383540
## Veillonella                     -0.1370350
## Slackia                          0.1309909
```

```
write.table(comp4, file="Smoking_comp4.txt", sep="\t", dec=",", row.names=T)

##  Sample plots from sPLS-DA including 95% confidence ellipses. Samples are projected into the space s
plotIndiv(final.splsda, comp = c(1,2), # plot samples from final model
          group = Y, ind.names = FALSE, # colour by class label
          ellipse = TRUE, legend = TRUE, # include 95% confidence ellipse
          title = ' (a) sPLS-DA on addp, comp 1 & 2')
```

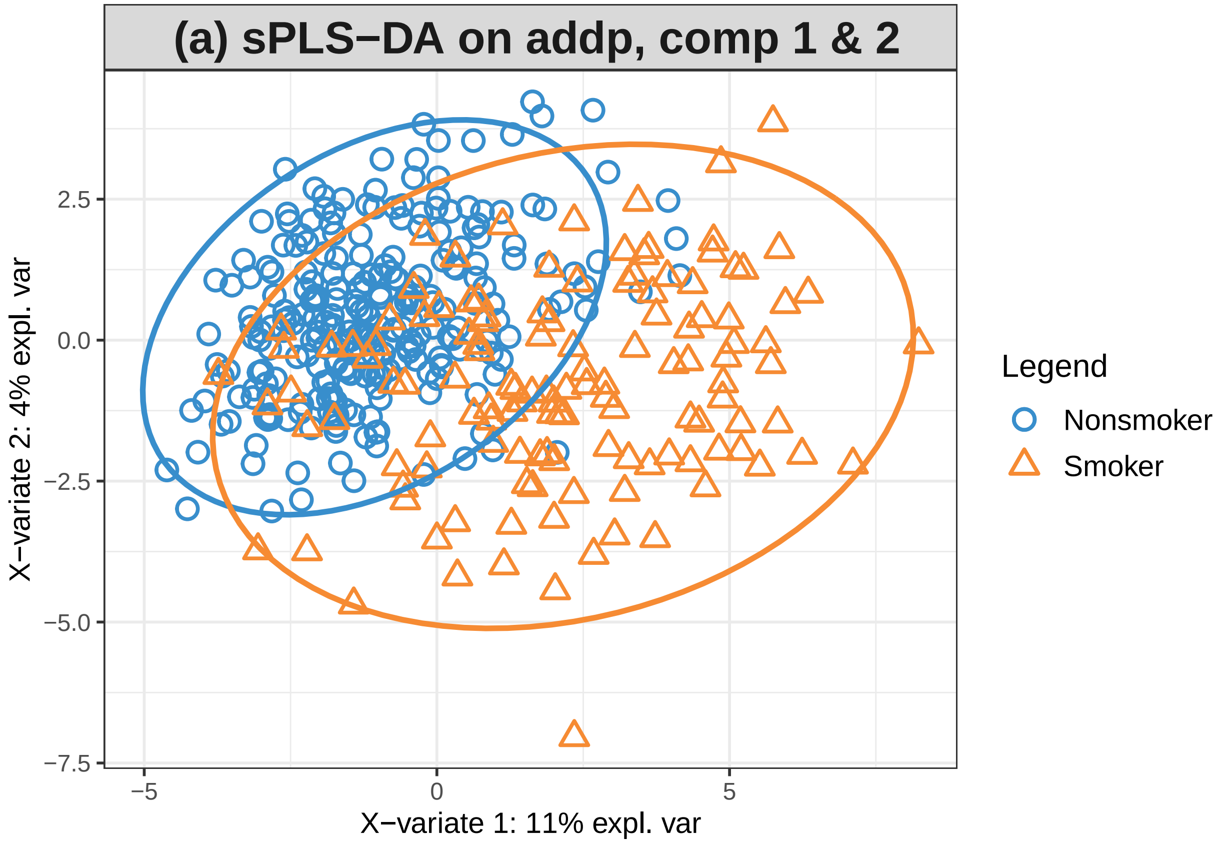


```r
# plotIndiv(final.splsda, comp = c(1,3), # plot samples from final model
#           group = Y, ind.names = FALSE,  # colour by class label
#           ellipse = TRUE, legend = TRUE, # include 95% confidence ellipse
#           title = '(b) sPLS-DA on addp, comp 1 & 3')

##Stability of variable selection from the sPLS-DA. The barplot represents the frequency of selection a
# form new perf() object which utilises the final model
perf.splsda.addp <- perf(final.splsda,
                          folds = 5, nrepeat = 10, # use repeated cross-validation
                          validation = "Mfold", dist = "max.dist",  # use max.dist measure
                          progressBar = FALSE)

# plot the stability of each feature for the first three components, 'h' type refers to histogram
par(mfrow=c(1,2))
plot(perf.splsda.addp$features$stable[[1]], type = 'h',
     ylab = 'Stability',
     xlab = 'Features',
     main = '(a) Comp 1', las =2)
plot(perf.splsda.addp$features$stable[[2]], type = 'h',
     ylab = 'Stability',
     xlab = 'Features',
     main = '(b) Comp 2', las =2)
```

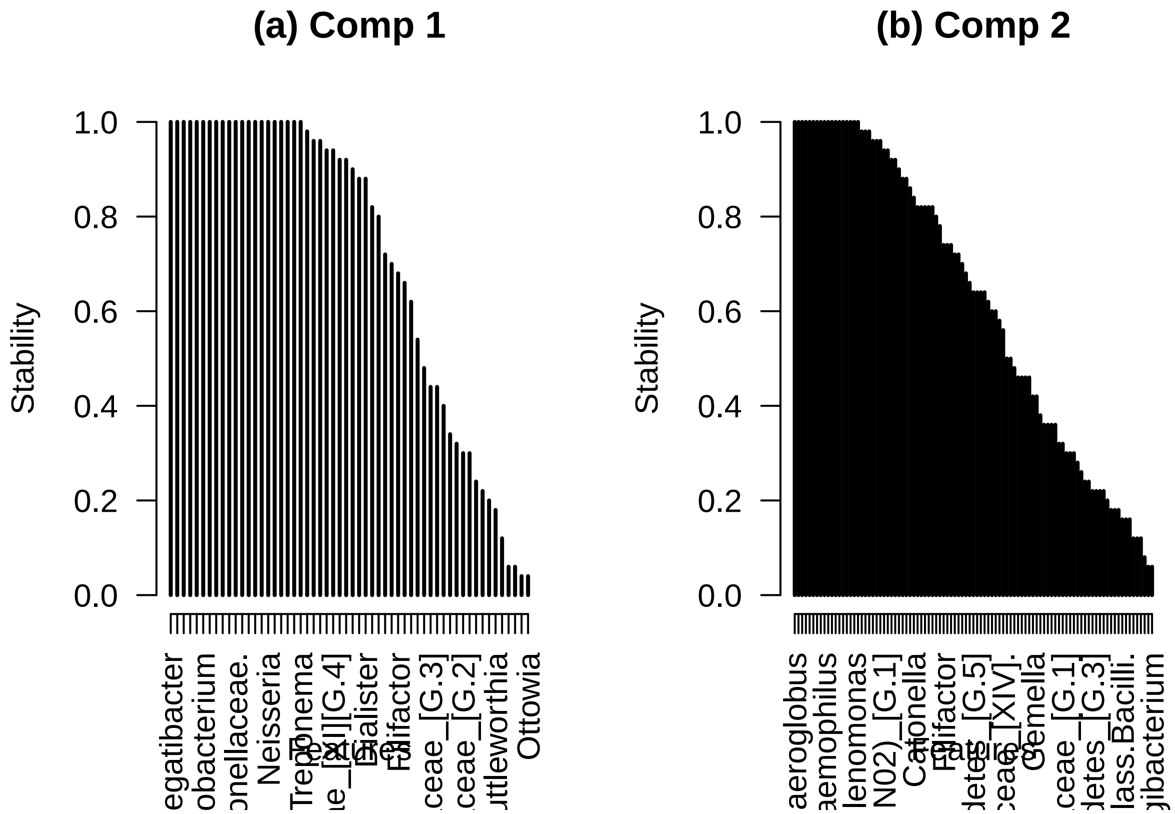


```r
# plot(perf.splsda.addp$features$stable[[3]], type = 'h',
#      ylab = 'Stability',
#      xlab = 'Features',
#      main = '(c) Comp 3', las =2)
par(mfrow=c(1,1))


train <- sample(1:nrow(X), 0.75*nrow(X)) # randomly select 75% of samples in training
test <- setdiff(1:nrow(X), train) # rest is part of the test set

# store matrices into training and test set:
X.train <- X[train, ]
X.test <- X[test,]
Y.train <- Y[train]
Y.test <- Y[test]

# train the model
train.splsda.addp <- splsda(X.train, Y.train, ncomp = optimal.ncomp, keepX = optimal.keepX)

# use the model on the Xtest set
predict.splsda.addp <- predict(train.splsda.addp, X.test, dist = "max.dist") #Changed from Mahalanobis

# evaluate the prediction accuracy for the first two components
predict.comp2 <- predict.splsda.addp$class$max.dist[,2]
table(factor(predict.comp2, levels = c("Nonsmoker", "Smoker")), Y.test)
```

```
##            Y.test
##             Nonsmoker Smoker
##   Nonsmoker        54     13
##   Smoker            7     24
```

```
#Correct classification rate
sum(diag(table(factor(predict.comp2, levels = c("Nonsmoker", "Smoker")), Y.test)))/
  sum(table(factor(predict.comp2, levels = c("Nonsmoker", "Smoker")), Y.test))
```

```
## [1] 0.7959184
```

```
## ROC curve and AUC from sPLS-DA on component 1 (a) and all (two) components (b) averaged across one-v
auc.splsda = auroc(final.splsda, roc.comp = 1, print = FALSE) # AUROC for the first component
```

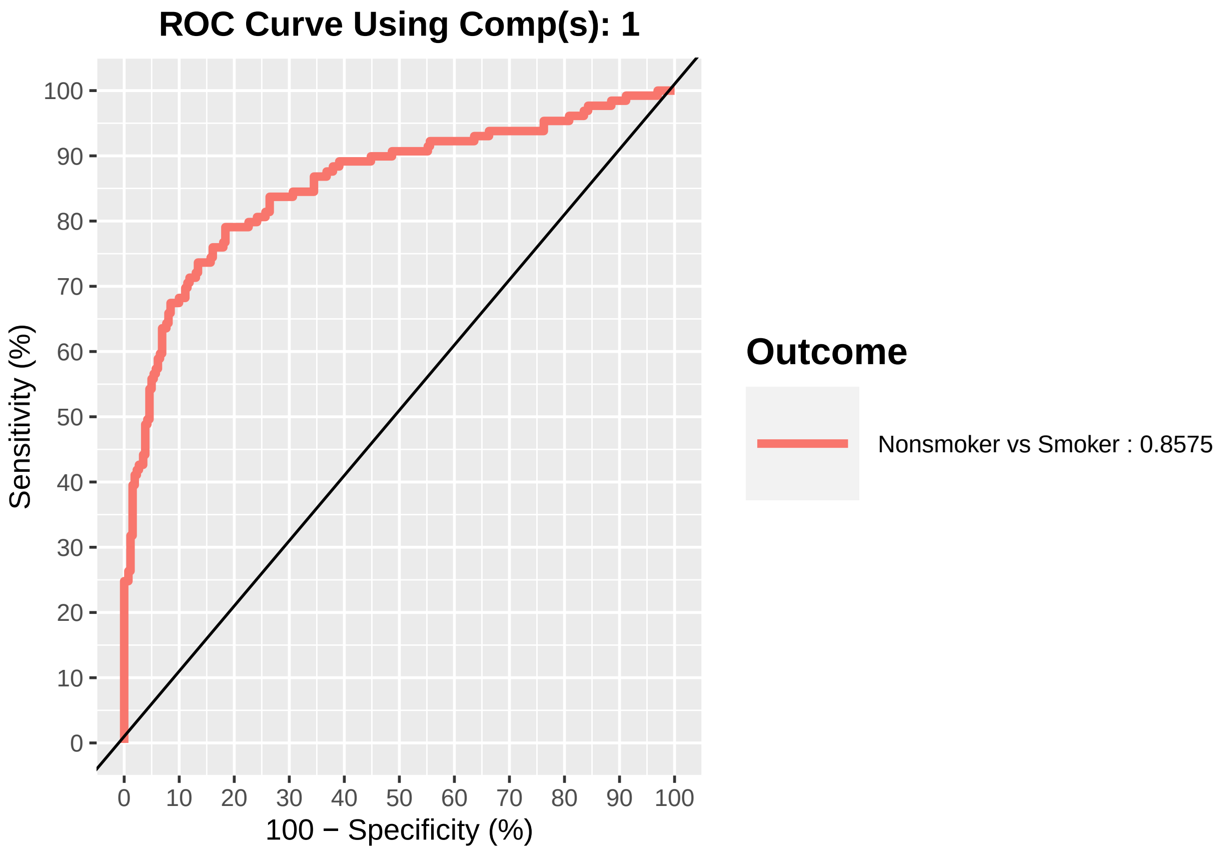


```
auc.splsda = auroc(final.splsda, roc.comp = 2, print = FALSE) # AUROC for the first and second componen
```

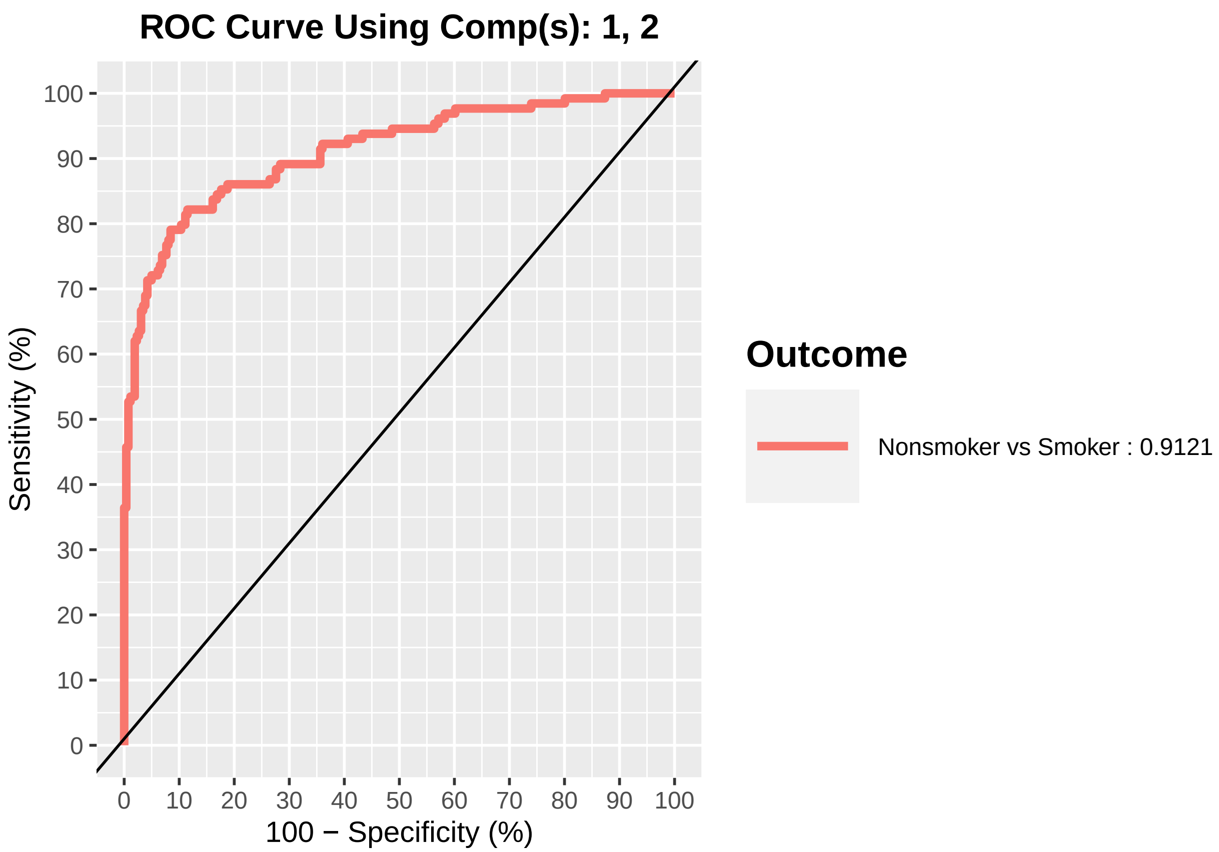


```
auc.splsda = auroc(final.splsda, roc.comp = 4, print = FALSE)
```

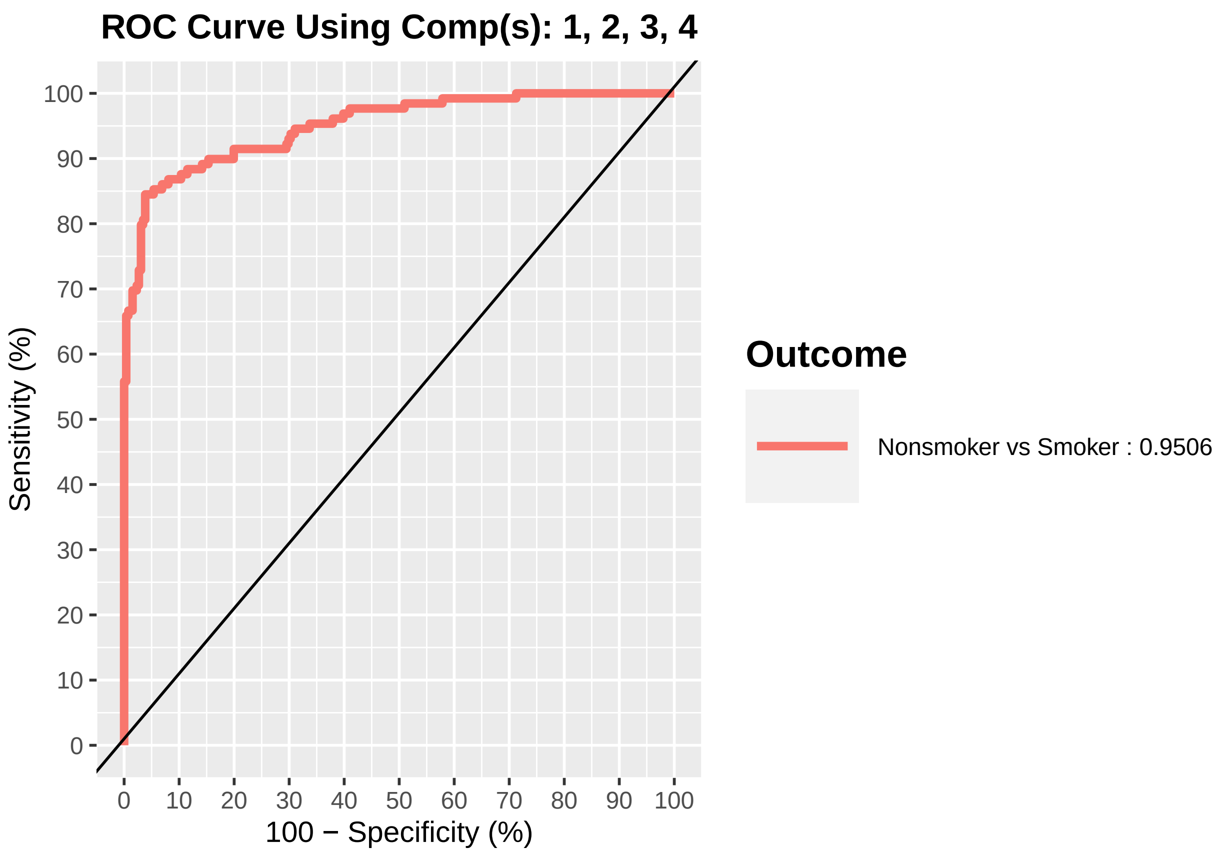


```
ROClist[["SmokingROC"]] <-auc.splsda$graph.Comp4

#AUC
print("AUC")
```

```
## [1] "AUC"
```

```
auc.splsda$Comp4
```

```
##                           AUC p-value
## Nonsmoker vs Smoker 0.9506       0
```

```
rm(list=setdiff(ls(), c("FeaturePic2", "Pic2", "Phe", "Microbio", "FeatureMic", "Metabo", "paretoscale"

set.seed(99) # for reproducibility, remove for normal use

Microbio2<-Microbio
#Subset select or just remove NA
table(Phe$Smoking, useNA="always")
```

Smoking clr

```
##
##  Exsmoker Nonsmoker    Smoker      <NA>
##       351       261       129         5
```

```
Phe2<-Phe[complete.cases(Phe$Smoking), ] #NA
Phe2<-subset(Phe2, Smoking %in% c("Smoker", "Nonsmoker")) #also removes NA, but above can be easier to
X<-dplyr::select(Microbio2, one_of(Phe2$IDX)) #Also in X
##Remove orgs that are not present after subsetting.
X <- X[rowSums(X)>0,]


# #Hellinger transformation
# X <- data.frame(t(decostand(t(X), method="hellinger")))
# #Maks TSS
# X<-sweep(X, 2, colSums(X), FUN="/")
# #rowSums(X)
# sum(colnames(X)!=Phe2$IDX)==0


X<-t(X)
Y<-Phe2$Smoking

sum(rownames(X)!=Phe2$IDX)==0
```

```
## [1] TRUE
```

```
dim(X) # check the dimensions of the X dataframe
```

```
## [1] 390  97
```

```
summary(Y) # check the distribution of class labels
```

```
##    Length     Class      Mode
##       390 character character
```

```
# Barplot of the variance each principal component explains
pca.addp = pca(X, ncomp = 10, center = TRUE, scale = TRUE) # run pca method on data
plot(pca.addp)  # barplot of the eigenvalues (explained variance per component)
```

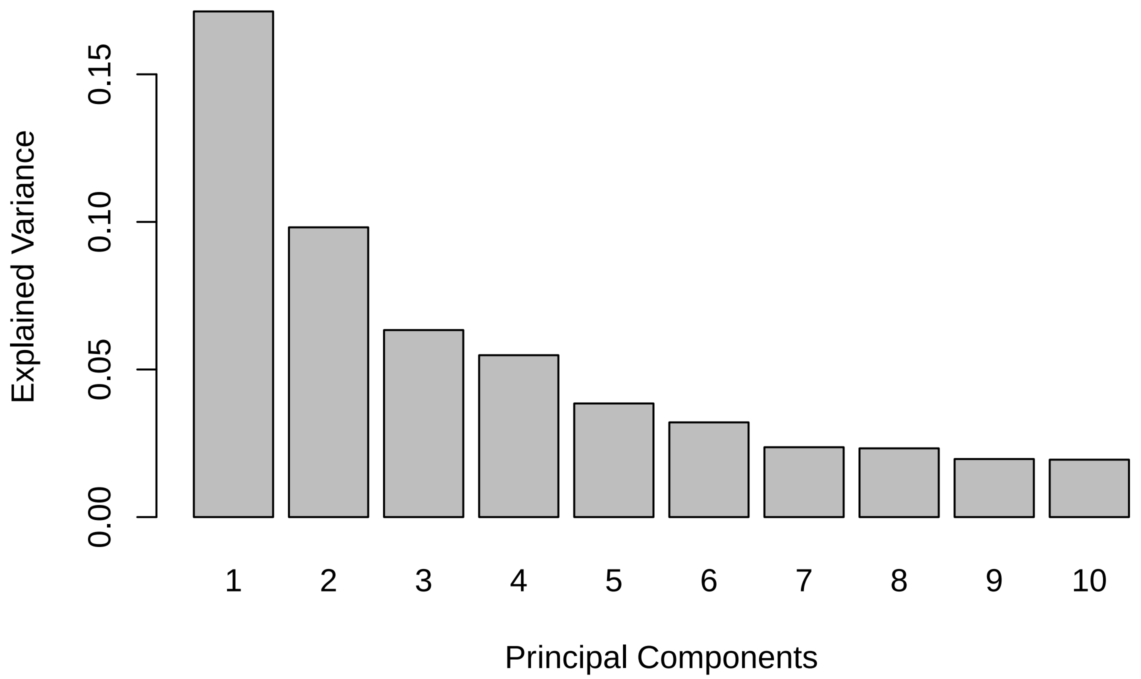


```
# Preliminary (unsupervised) analysis with PCA
plotIndiv(pca.addp, group = Y, ind.names = FALSE, # plot the samples projected
          legend = TRUE, title = 'PCA on addp, comp 1 - 2') # onto the PCA subspace
```

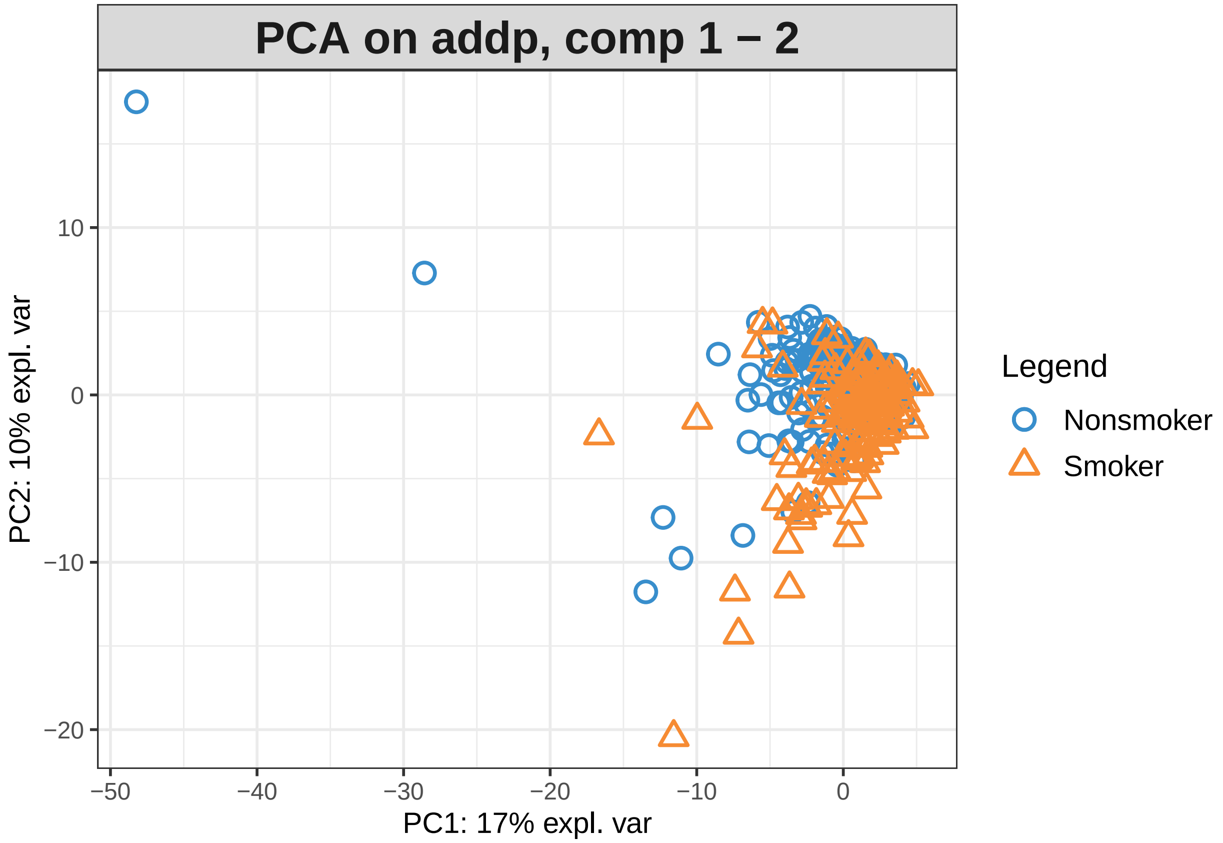


```
X<-X+1
addp.splsda <- splsda(X, Y, ncomp = 10, logratio = 'CLR')  # set ncomp to 10 for performance assessment

## Sample plots after a basic PLS-DA model was operated on this data.
# plot the samples projected onto the first two components of the PLS-DA subspace
plotIndiv(addp.splsda , comp = 1:2,
          group = Y, ind.names = FALSE,  # colour points by class
          ellipse = TRUE, # include 95% confidence ellipse for each class
          legend = TRUE, title = '(a) PLSDA with confidence ellipses')
```

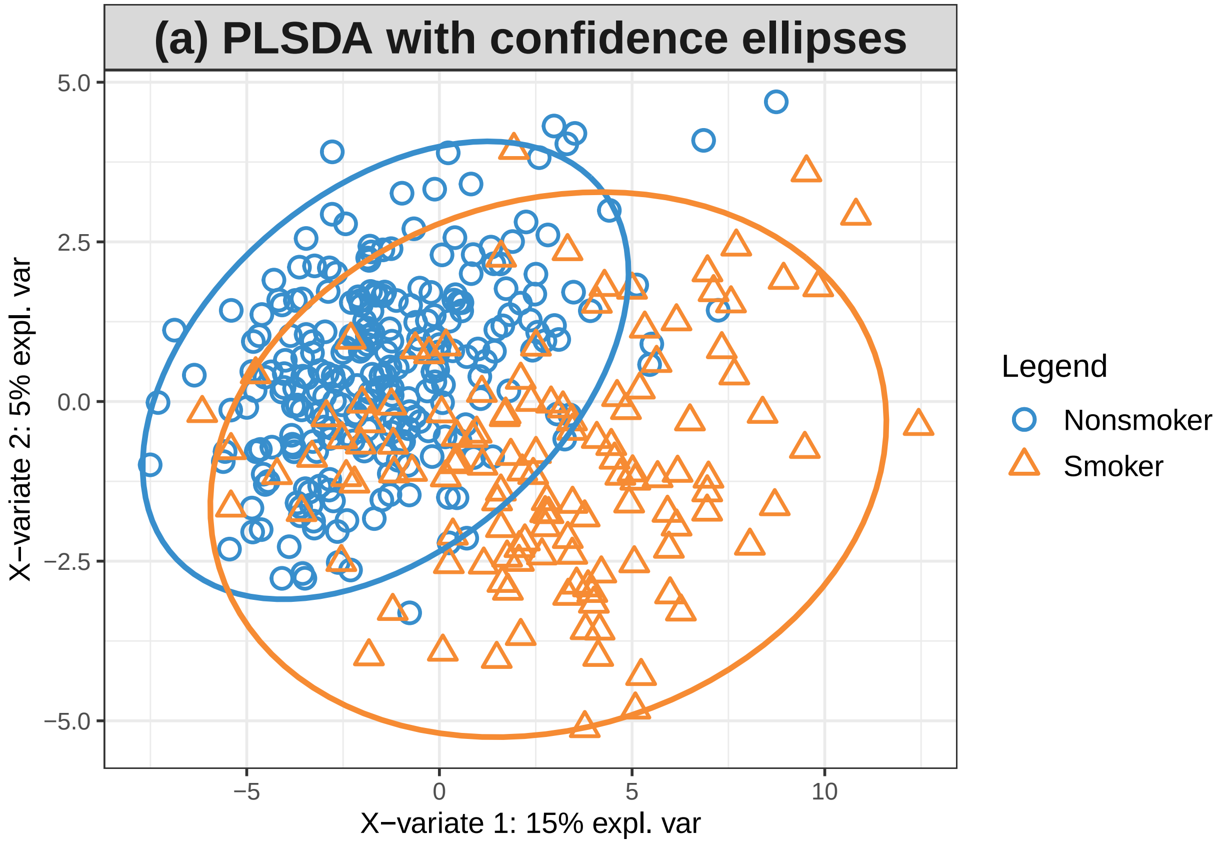


```
# use the max.dist measure to form decision boundaries between classes based on PLS-DA data
background = background.predict(addp.splsda, comp.predicted=2, dist = "max.dist")

# plot the samples projected onto the first two components of the PLS-DA subspace
plotIndiv(addp.splsda, comp = 1:2,
          group = Y, ind.names = FALSE, # colour points by class
          background = background, # include prediction background for each class
          legend = TRUE, title = " (b) PLSDA with prediction background")
```

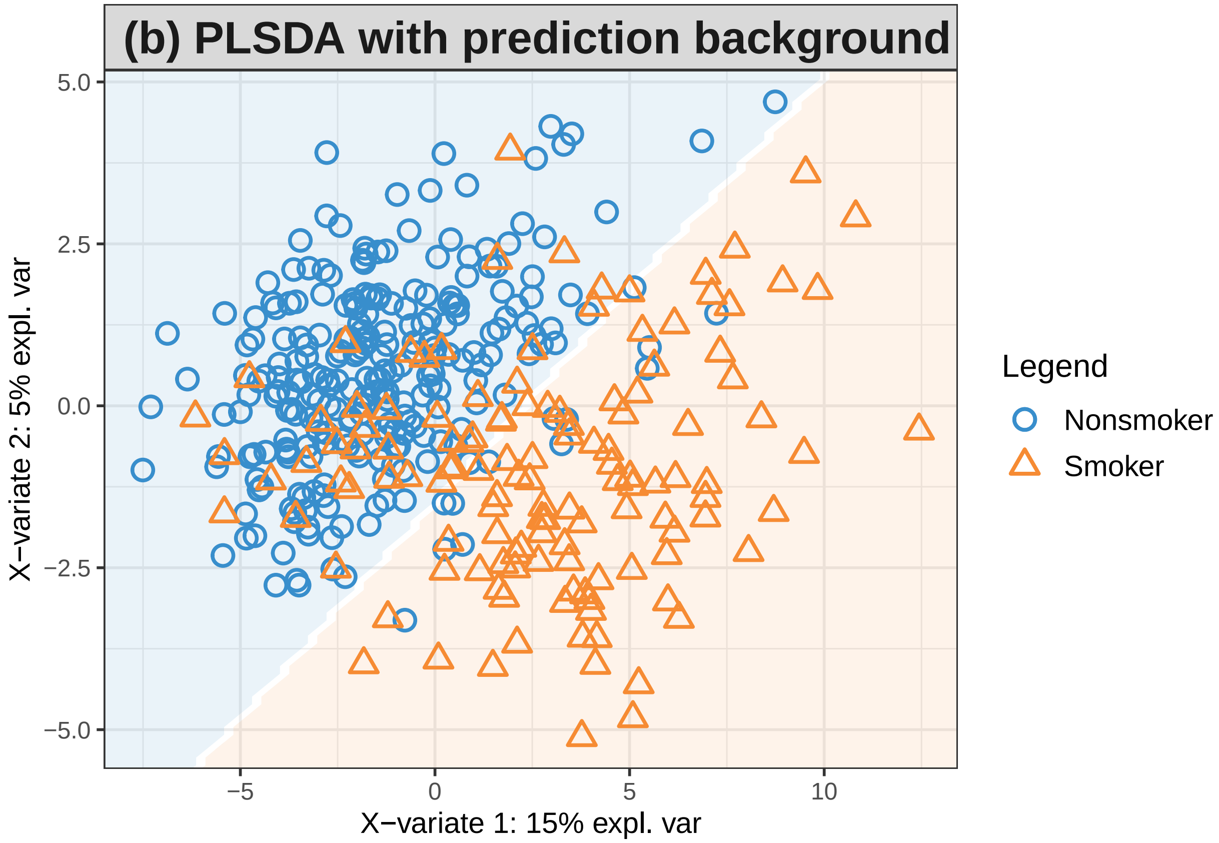


```
## Tuning the number of components in PLS-DA. For each component, repeated cross-validation (10 × 3-fol
# undergo performance evaluation in order to tune the number of components to use
perf.splsda.addp <- perf(addp.splsda, validation = "Mfold",
                         folds = 5, nrepeat = 10, # use repeated cross-validation
                         progressBar = FALSE, auc = TRUE) # include AUC values

# plot the outcome of performance evaluation across all ten components
plot(perf.splsda.addp, col = color.mixo(5:7), sd = TRUE,
     legend.position = "horizontal")
```

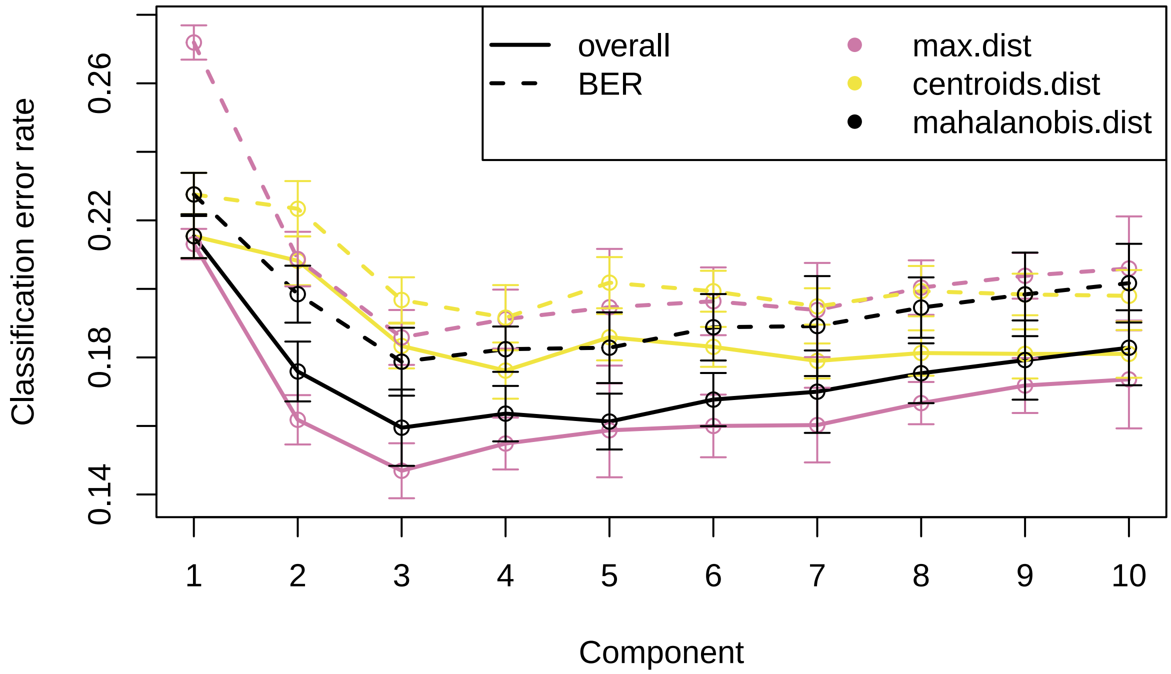


```
perf.splsda.addp$choice.ncomp # what is the optimal value of components according to perf()
```

```
##         max.dist centroids.dist mahalanobis.dist
## overall        3              3                3
## BER            3              3                3
```

```
## Tuning keepX for the sPLS-DA. Each coloured line represents the balanced error rate (y-axis) per comp
# grid of possible keepX values that will be tested for each component
list.keepX <- c(1:10,  seq(20, 300, 10))

# undergo the tuning process to determine the optimal number of variables
tune.splsda.addp <- tune.splsda(X, Y, ncomp = 4, # calculate for first 4 components
                                validation = 'Mfold',
                                folds = 5, nrepeat = 10, # use repeated cross-validation
                                dist = 'max.dist', # use max.dist measure
                                measure = "BER", # use balanced error rate of dist measure
                                test.keepX = list.keepX,
                                cpus = 2 # allow for parallelisation to decrease runtime
                                )

plot(tune.splsda.addp, col = color.jet(4))
```

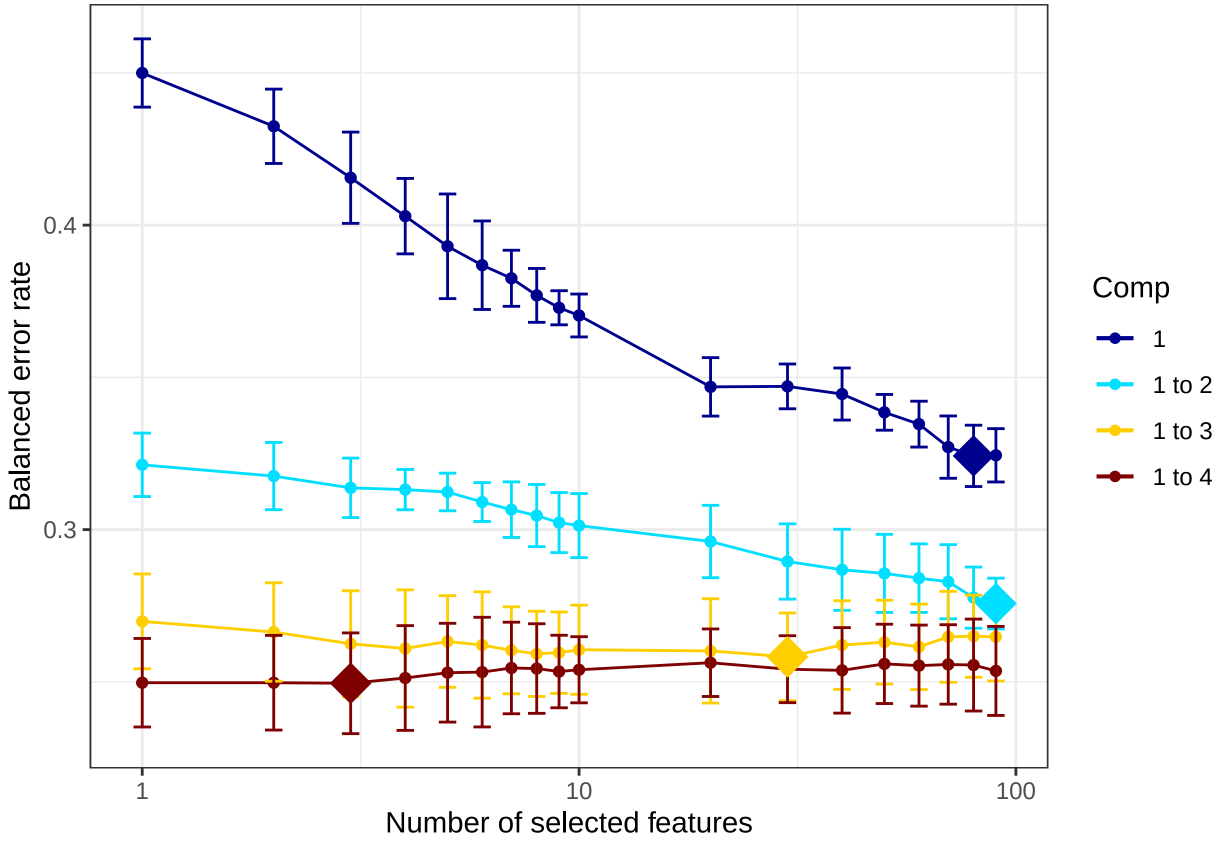


```r
tune.splsda.addp$choice.ncomp$ncomp # what is the optimal value of components according to tune.splsda(
```

```
## [1] 3
```

```r
tune.splsda.addp$choice.keepX # what are the optimal values of variables according to tune.splsda()
```

```
## comp1 comp2 comp3 comp4
##    80    90    30     3
```

```r
optimal.ncomp <- tune.splsda.addp$choice.ncomp$ncomp
optimal.keepX <- tune.splsda.addp$choice.keepX[1:optimal.ncomp]

# form final model with optimised values for component and variable count
final.splsda <- splsda(X, Y,
                       ncomp = optimal.ncomp,
                       keepX = optimal.keepX,
                       logratio = 'CLR')

#########Loadings
plotLoadings(final.splsda, comp=1, contrib = 'max', method = 'mean', size.title = 1)
```

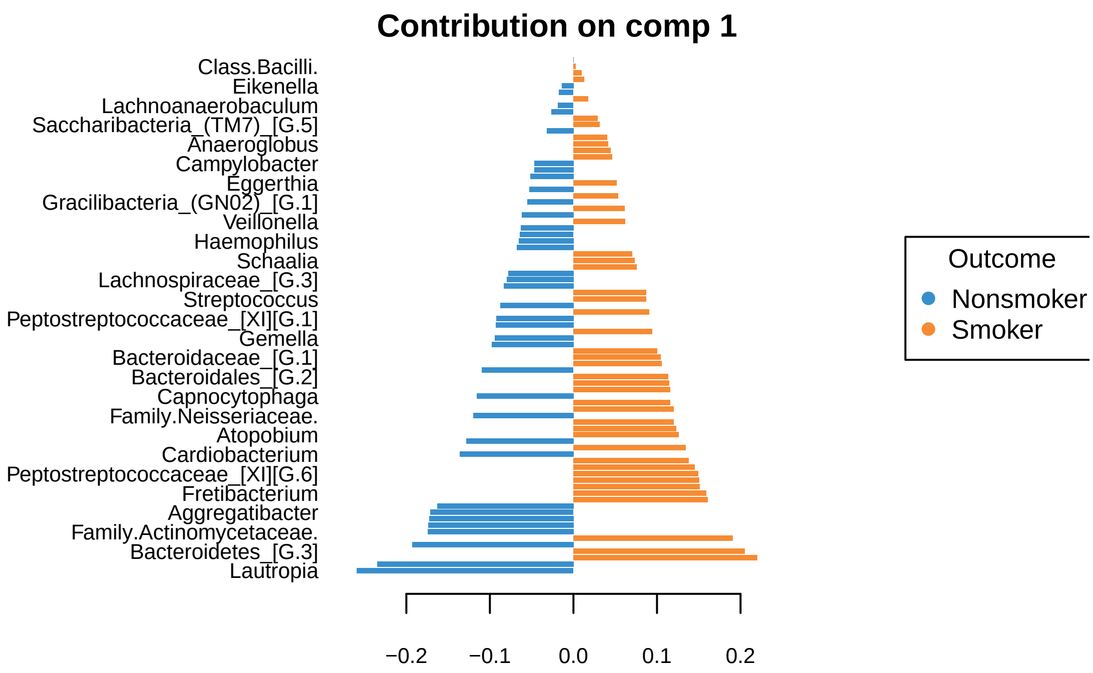


```
plotLoadings(final.splsda, comp=2, contrib = 'max', method = 'mean', size.title = 1)
```

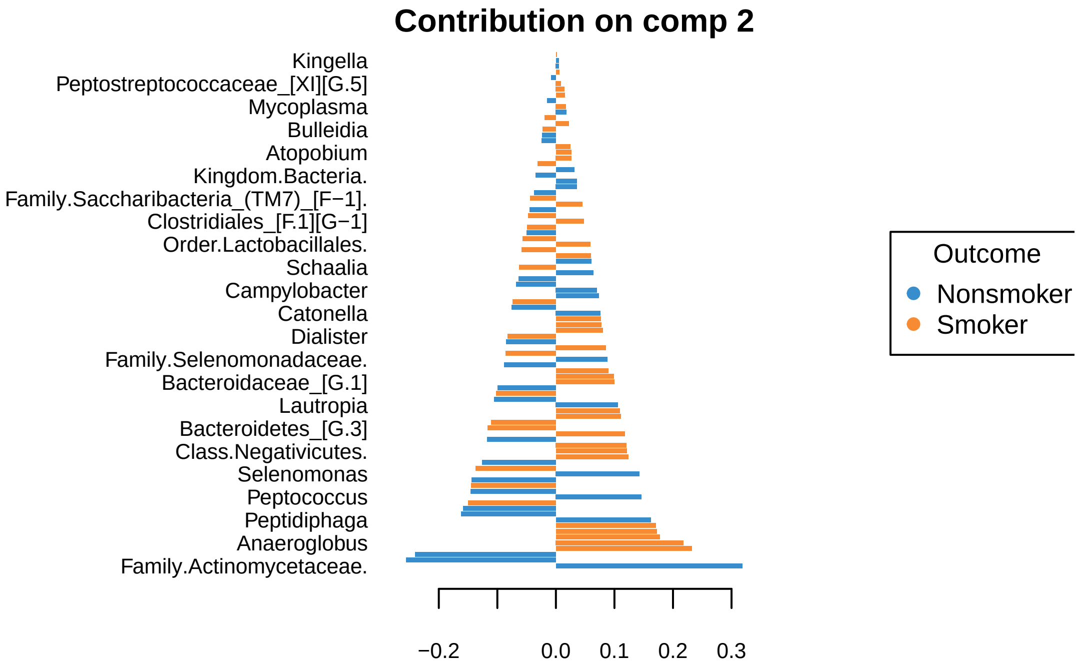


```
comp1 <- plotLoadings(final.splsda, comp = 1, method = 'mean', contrib = 'max',
                      size.title = 1)
```

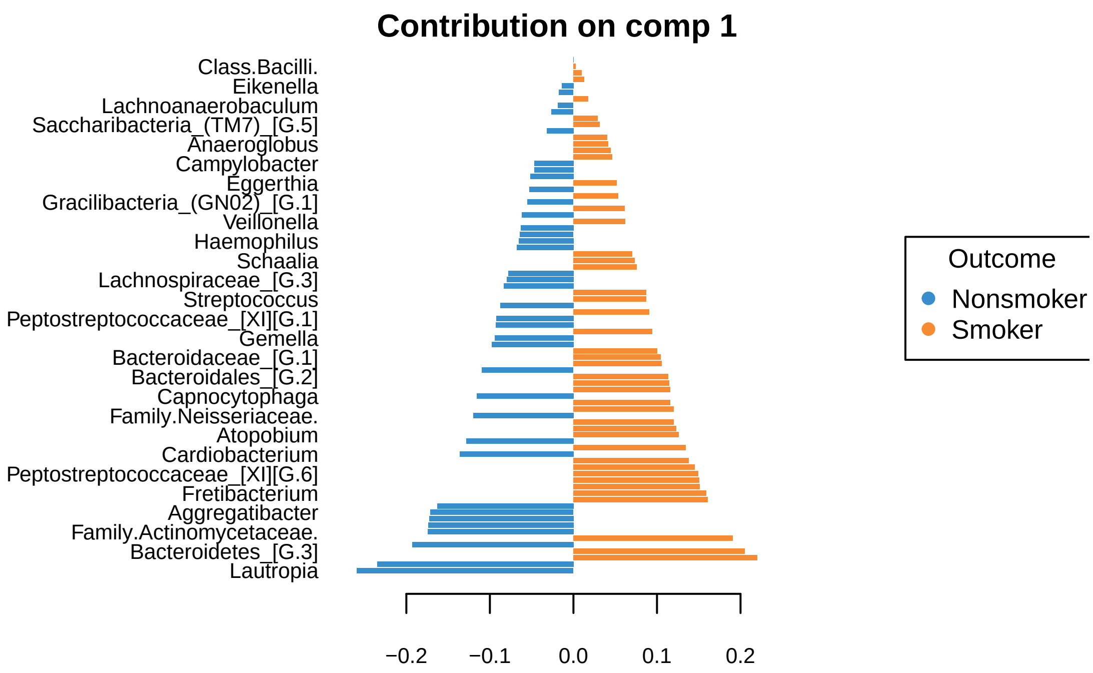


```
head(comp1, n=20)
```

```
##                                  Nonsmoker     Smoker Contrib.Nonsmoker
## Lautropia                        0.3242413 -0.6560231              TRUE
## Neisseria                        0.2973748 -0.6016653              TRUE
## Lachnospiraceae_[G.7]           -0.2804289  0.5673794             FALSE
## Bacteroidetes_[G.3]             -0.2638729  0.5338824             FALSE
## Catonella                        0.2504674 -0.5067597              TRUE
## Alloscardovia                   -0.2475349  0.5008264             FALSE
## Family.Actinomycetaceae.         0.2297187 -0.4647797              TRUE
## Kingella                         0.2287827 -0.4628860              TRUE
## Abiotrophia                      0.2277258 -0.4607476              TRUE
## Aggregatibacter                  0.2259961 -0.4572480              TRUE
## Peptostreptococcus               0.2170220 -0.4390911              TRUE
## Lactobacillus                   -0.2142549  0.4334925             FALSE
## Fretibacterium                  -0.2120441  0.4290194             FALSE
## Peptostreptococcaceae_[XI][G.9] -0.2039523  0.4126477             FALSE
## Bulleidia                       -0.2032859  0.4112993             FALSE
## Peptostreptococcaceae_[XI][G.6] -0.2017741  0.4082406             FALSE
## Bifidobacterium                 -0.1968839  0.3983464             FALSE
## Peptostreptococcaceae_[XI][G.4] -0.1886942  0.3817766             FALSE
## Cardiobacterium                  0.1868935 -0.3781334              TRUE
## Cryptobacterium                 -0.1851475  0.3746008             FALSE
##                                 Contrib.Smoker Contrib GroupContrib   color
## Lautropia                                FALSE   FALSE    Nonsmoker #388ECC
```

```
## Neisseria                                FALSE   FALSE    Nonsmoker #388ECC
## Lachnospiraceae_[G.7]                     TRUE   FALSE       Smoker #F68B33
## Bacteroidetes_[G.3]                       TRUE   FALSE       Smoker #F68B33
## Catonella                                FALSE   FALSE    Nonsmoker #388ECC
## Alloscardovia                             TRUE   FALSE       Smoker #F68B33
## Family.Actinomycetaceae.                 FALSE   FALSE    Nonsmoker #388ECC
## Kingella                                 FALSE   FALSE    Nonsmoker #388ECC
## Abiotrophia                              FALSE   FALSE    Nonsmoker #388ECC
## Aggregatibacter                          FALSE   FALSE    Nonsmoker #388ECC
## Peptostreptococcus                       FALSE   FALSE    Nonsmoker #388ECC
## Lactobacillus                             TRUE   FALSE       Smoker #F68B33
## Fretibacterium                            TRUE   FALSE       Smoker #F68B33
## Peptostreptococcaceae_[XI][G.9]           TRUE   FALSE       Smoker #F68B33
## Bulleidia                                 TRUE   FALSE       Smoker #F68B33
## Peptostreptococcaceae_[XI][G.6]           TRUE   FALSE       Smoker #F68B33
## Bifidobacterium                           TRUE   FALSE       Smoker #F68B33
## Peptostreptococcaceae_[XI][G.4]           TRUE   FALSE       Smoker #F68B33
## Cardiobacterium                          FALSE   FALSE    Nonsmoker #388ECC
## Cryptobacterium                           TRUE   FALSE       Smoker #F68B33
##                                  importance
## Lautropia                        -0.2591021
## Neisseria                        -0.2350558
## Lachnospiraceae_[G.7]             0.2198888
## Bacteroidetes_[G.3]               0.2050707
## Catonella                        -0.1930724
## Alloscardovia                     0.1904477
## Family.Actinomycetaceae.         -0.1745017
## Kingella                         -0.1736640
## Abiotrophia                      -0.1727180
## Aggregatibacter                  -0.1711699
## Peptostreptococcus               -0.1631379
## Lactobacillus                     0.1606612
## Fretibacterium                    0.1586824
## Peptostreptococcaceae_[XI][G.9]   0.1514401
## Bulleidia                         0.1508436
## Peptostreptococcaceae_[XI][G.6]   0.1494905
## Bifidobacterium                   0.1451136
## Peptostreptococcaceae_[XI][G.4]   0.1377836
## Cardiobacterium                  -0.1361720
## Cryptobacterium                   0.1346093
```

```
comp2 <- plotLoadings(final.splsda, comp = 2, method = 'mean', contrib = 'max',
                      size.title = 1)
```

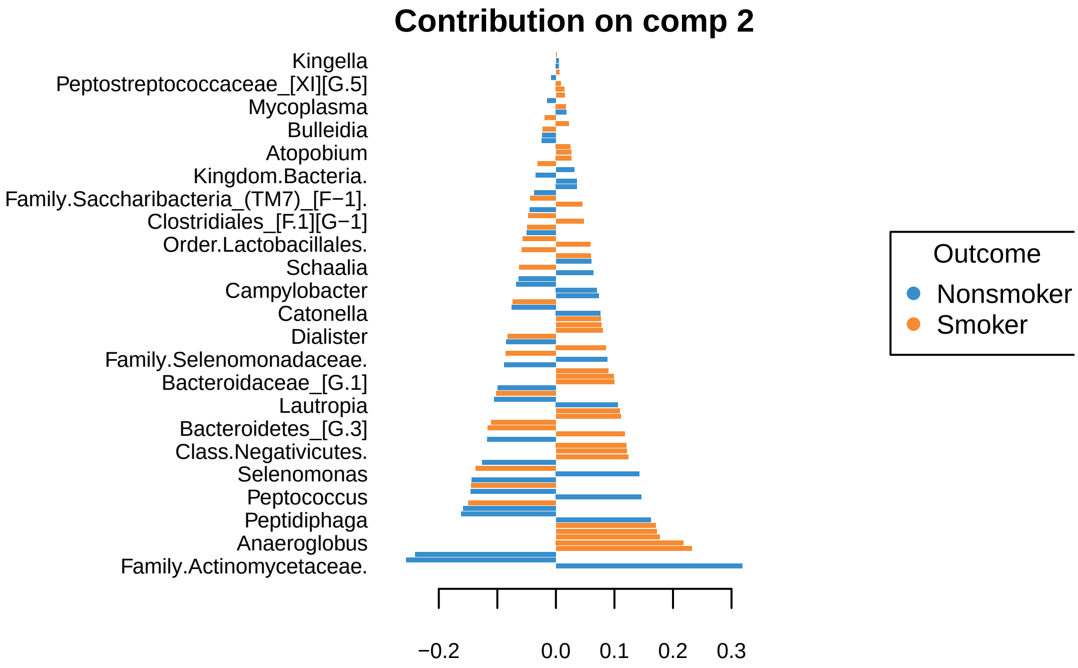


```r
head(comp2, n=20)
```

```
##                                    Nonsmoker       Smoker Contrib.Nonsmoker
## Family.Actinomycetaceae.         0.229718694 -0.464779683              TRUE
## Bergeyella                       0.138883235 -0.280996314              TRUE
## Porphyromonas                    0.106192576 -0.214854748              TRUE
## Scardovia                       -0.054310322  0.109883674             FALSE
## Anaeroglobus                    -0.081290267  0.164471006             FALSE
## Veillonella                     -0.104079074  0.210578591             FALSE
## Parascardovia                   -0.086866906  0.175753972             FALSE
## Veillonellaceae_[G.1]           -0.048963830  0.099066354             FALSE
## Peptidiphaga                     0.177970661 -0.360080175              TRUE
## Saccharibacteria_(TM7)_[G.1]     0.002409995 -0.004876037              TRUE
## Absconditabacteria_(SR1)_[G.1]   0.127846100 -0.258665366              TRUE
## Granulicatella                  -0.080146276  0.162156419             FALSE
## Peptococcus                      0.143878231 -0.291102467              TRUE
## Capnocytophaga                   0.164270533 -0.332361312              TRUE
## Corynebacterium                 -0.006102666  0.012347254             FALSE
## Ruminococcaceae_[G.1]            0.121750864 -0.246333143              TRUE
## Selenomonas                      0.092297830 -0.186742121              TRUE
## Saccharibacteria_(TM7)_[G.3]    -0.031676001  0.064088654             FALSE
## Gracilibacteria_(GN02)_[G.1]     0.096227124 -0.194692088              TRUE
## Prevotella                      -0.034283637  0.069364569             FALSE
##                                 Contrib.Smoker Contrib GroupContrib   color
## Family.Actinomycetaceae.                 FALSE   FALSE    Nonsmoker #388ECC
```

```
## Bergeyella                            FALSE   FALSE    Nonsmoker #388ECC
## Porphyromonas                         FALSE   FALSE    Nonsmoker #388ECC
## Scardovia                              TRUE   FALSE       Smoker #F68B33
## Anaeroglobus                           TRUE   FALSE       Smoker #F68B33
## Veillonella                            TRUE   FALSE       Smoker #F68B33
## Parascardovia                          TRUE   FALSE       Smoker #F68B33
## Veillonellaceae_[G.1]                  TRUE   FALSE       Smoker #F68B33
## Peptidiphaga                          FALSE   FALSE    Nonsmoker #388ECC
## Saccharibacteria_(TM7)_[G.1]          FALSE   FALSE    Nonsmoker #388ECC
## Absconditabacteria_(SR1)_[G.1]        FALSE   FALSE    Nonsmoker #388ECC
## Granulicatella                         TRUE   FALSE       Smoker #F68B33
## Peptococcus                           FALSE   FALSE    Nonsmoker #388ECC
## Capnocytophaga                        FALSE   FALSE    Nonsmoker #388ECC
## Corynebacterium                        TRUE   FALSE       Smoker #F68B33
## Ruminococcaceae_[G.1]                 FALSE   FALSE    Nonsmoker #388ECC
## Selenomonas                           FALSE   FALSE    Nonsmoker #388ECC
## Saccharibacteria_(TM7)_[G.3]           TRUE   FALSE       Smoker #F68B33
## Gracilibacteria_(GN02)_[G.1]          FALSE   FALSE    Nonsmoker #388ECC
## Prevotella                             TRUE   FALSE       Smoker #F68B33
##                                 importance
## Family.Actinomycetaceae.         0.3183872
## Bergeyella                      -0.2555021
## Porphyromonas                   -0.2402971
## Scardovia                        0.2317753
## Anaeroglobus                     0.2180269
## Veillonella                      0.1773716
## Parascardovia                    0.1724742
## Veillonellaceae_[G.1]            0.1703299
## Peptidiphaga                     0.1620820
## Saccharibacteria_(TM7)_[G.1]    -0.1616511
## Absconditabacteria_(SR1)_[G.1]  -0.1588063
## Granulicatella                  -0.1497155
## Peptococcus                      0.1461388
## Capnocytophaga                  -0.1455048
## Corynebacterium                 -0.1448767
## Ruminococcaceae_[G.1]           -0.1439532
## Selenomonas                      0.1426455
## Saccharibacteria_(TM7)_[G.3]    -0.1367037
## Gracilibacteria_(GN02)_[G.1]    -0.1258787
## Prevotella                       0.1234882
```

```
##  Sample plots from sPLS-DA including 95% confidence ellipses. Samples are projected into the space s
plotIndiv(final.splsda, comp = c(1,2), # plot samples from final model
          group = Y, ind.names = FALSE, # colour by class label
          ellipse = TRUE, legend = TRUE, # include 95% confidence ellipse
          title = ' (a) sPLS-DA on addp, comp 1 & 2')
```

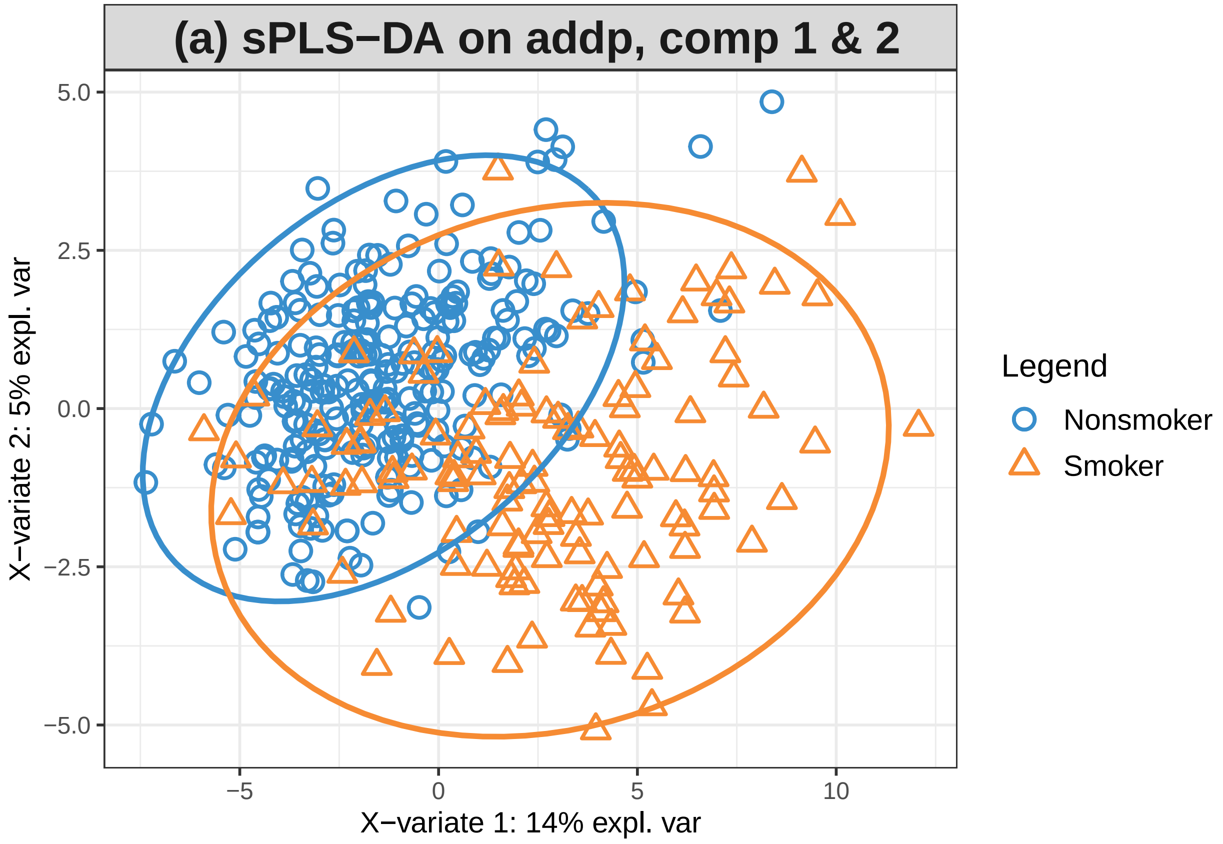


```
# plotIndiv(final.splsda, comp = c(1,3), # plot samples from final model
#           group = Y, ind.names = FALSE,  # colour by class label
#           ellipse = TRUE, legend = TRUE, # include 95% confidence ellipse
#           title = '(b) sPLS-DA on addp, comp 1 & 3')

##Stability of variable selection from the sPLS-DA. The barplot represents the frequency of selection a
# form new perf() object which utilises the final model
perf.splsda.addp <- perf(final.splsda,
                          folds = 5, nrepeat = 10, # use repeated cross-validation
                          validation = "Mfold", dist = "max.dist",  # use max.dist measure
                          progressBar = FALSE)

# plot the stability of each feature for the first three components, 'h' type refers to histogram
par(mfrow=c(1,2))
plot(perf.splsda.addp$features$stable[[1]], type = 'h',
     ylab = 'Stability',
     xlab = 'Features',
     main = '(a) Comp 1', las =2)
plot(perf.splsda.addp$features$stable[[2]], type = 'h',
     ylab = 'Stability',
     xlab = 'Features',
     main = '(b) Comp 2', las =2)
```

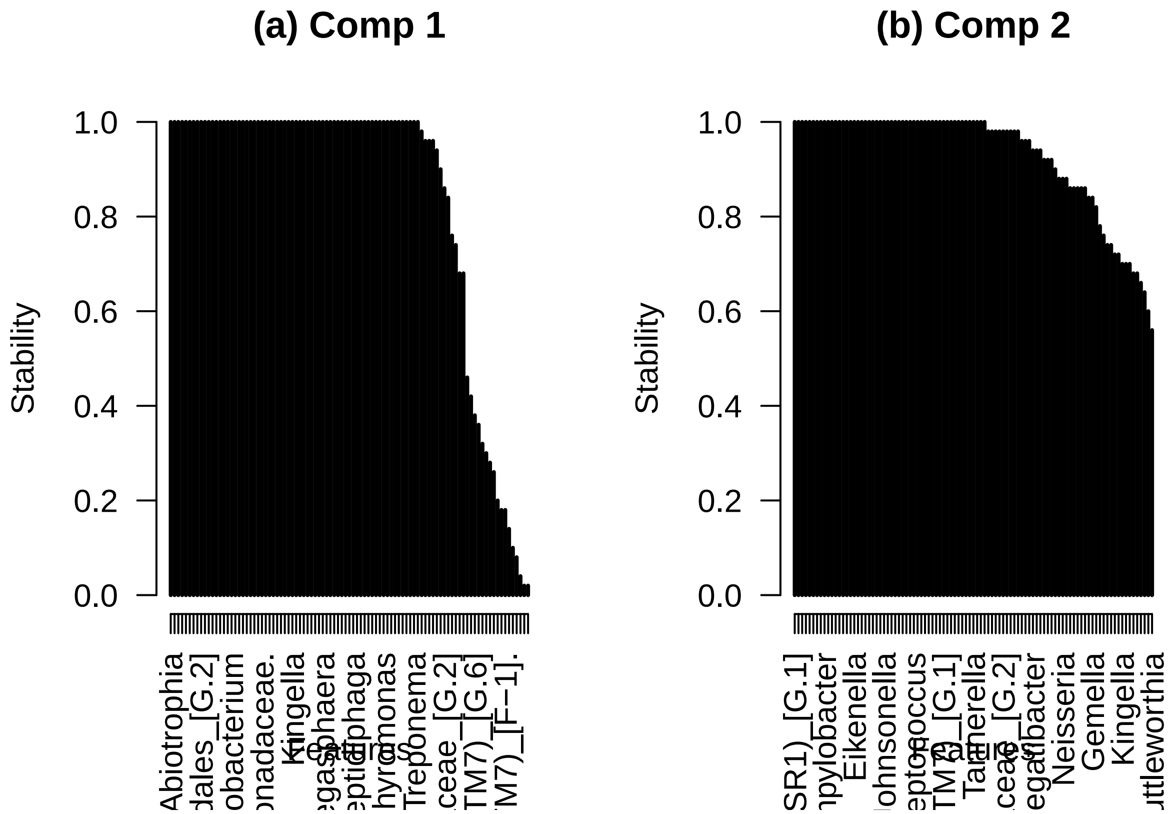


```
# plot(perf.splsda.addp$features$stable[[3]], type = 'h',
#      ylab = 'Stability',
#      xlab = 'Features',
#      main = '(c) Comp 3', las =2)
par(mfrow=c(1,1))


train <- sample(1:nrow(X), 0.75*nrow(X)) # randomly select 75% of samples in training
test <- setdiff(1:nrow(X), train) # rest is part of the test set

# store matrices into training and test set:
X.train <- X[train, ]
X.test <- X[test,]
Y.train <- Y[train]
Y.test <- Y[test]

# train the model
train.splsda.addp <- splsda(X.train, Y.train, ncomp = optimal.ncomp, keepX = optimal.keepX)

# use the model on the Xtest set
predict.splsda.addp <- predict(train.splsda.addp, X.test, dist = "max.dist")

# evaluate the prediction accuracy for the first two components
predict.comp2 <- predict.splsda.addp$class$max.dist[,2]
table(factor(predict.comp2, levels = c("Nonsmoker", "Smoker")), Y.test)
```

```
##            Y.test
##             Nonsmoker Smoker
##   Nonsmoker        64      9
##   Smoker            3     22
```

```
#Correct classification rate
sum(diag(table(factor(predict.comp2, levels = c("Nonsmoker", "Smoker")), Y.test)))/
  sum(table(factor(predict.comp2, levels = c("Nonsmoker", "Smoker")), Y.test))
```

```
## [1] 0.877551
```

```
## ROC curve and AUC from sPLS-DA on component 1 (a) and all (two) components (b) averaged across one-v
auc.splsda = auroc(final.splsda, roc.comp = 1, print = FALSE) # AUROC for the first component
```

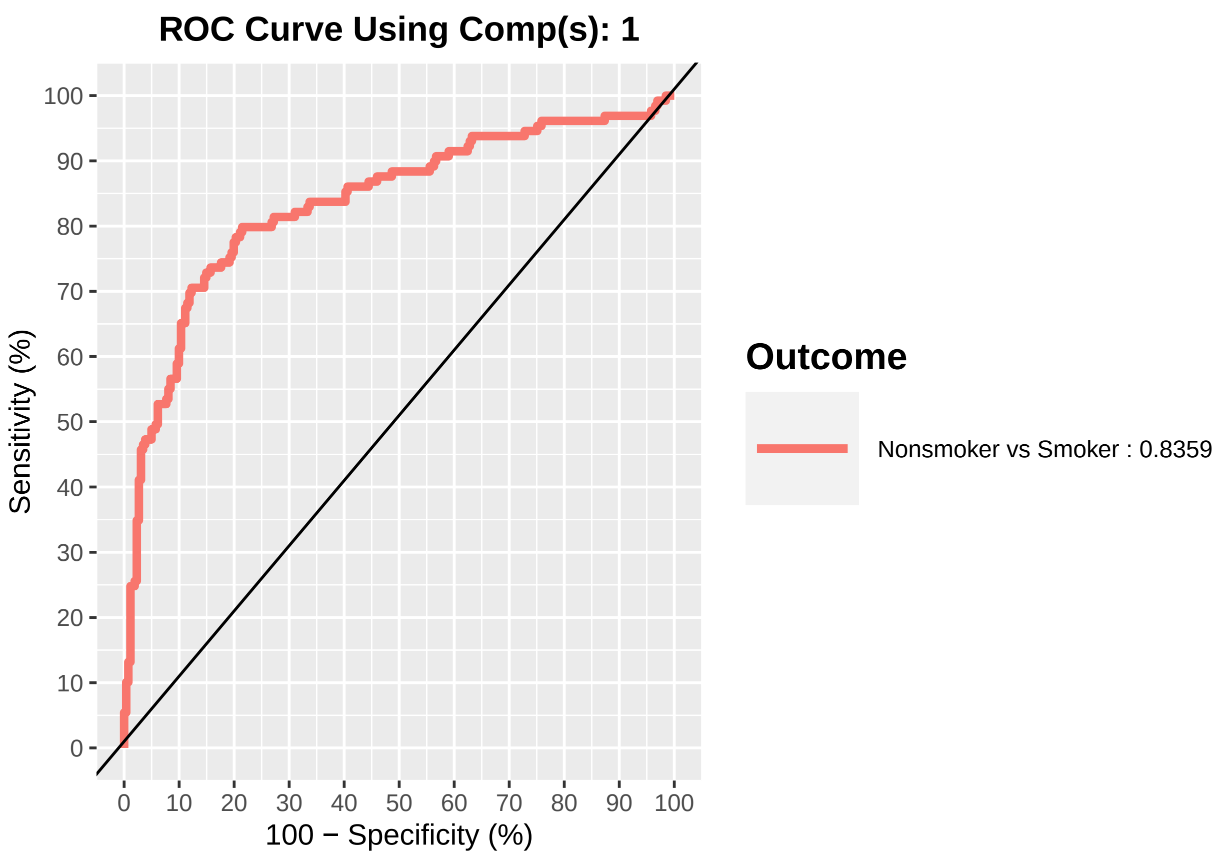


```
auc.splsda = auroc(final.splsda, roc.comp = 2, print = FALSE) # AUROC for the first and second componen
```

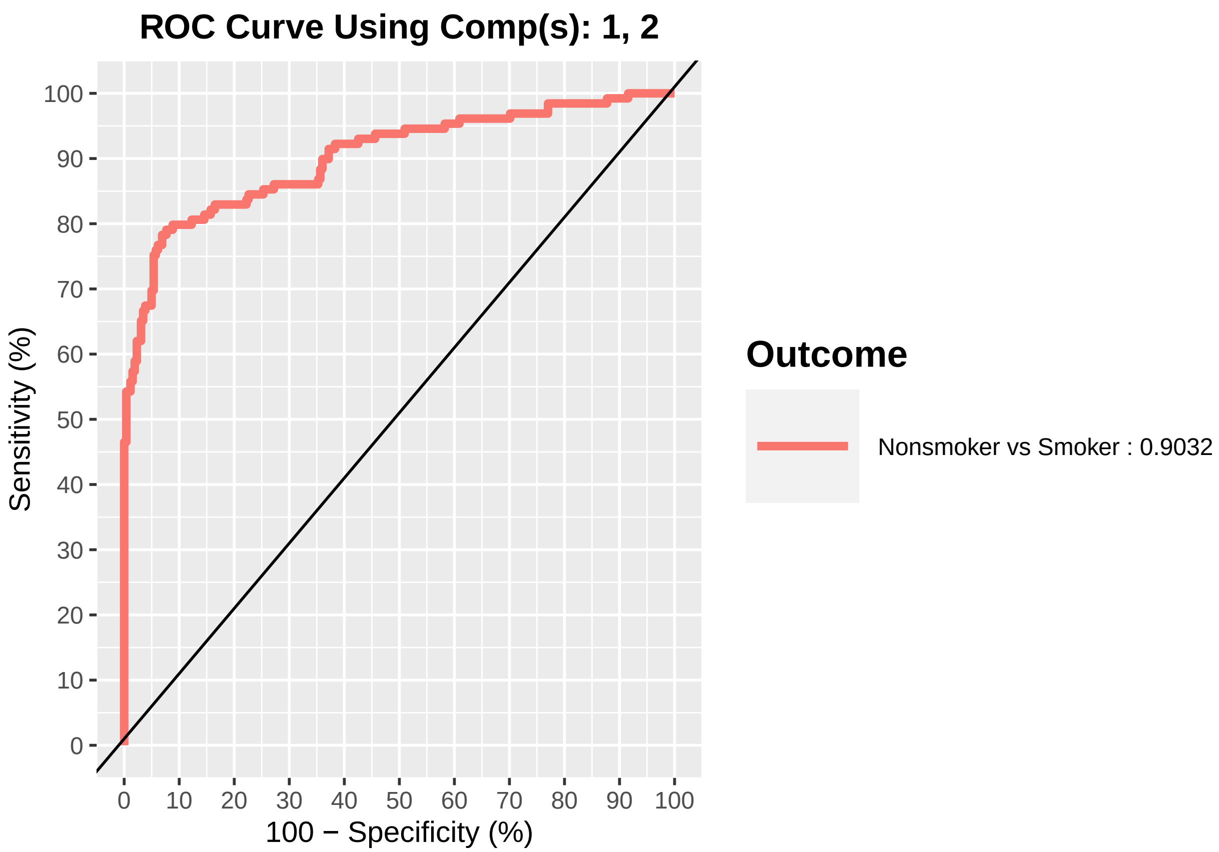


```
#AUC
print("AUC")
```

```
## [1] "AUC"
```

```
auc.splsda$Comp2
```

```
##                            AUC p-value
## Nonsmoker vs Smoker 0.9032       0
```

```
rm(list=setdiff(ls(), c("FeaturePic2", "Pic2", "Phe", "Microbio", "FeatureMic", "Metabo", "paretoscale"

set.seed(99) # for reproducibility, remove for normal use

Microbio2<-Microbio
#Subset select or just remove NA
table(Phe$sex_cat, useNA="always")
```

sex_cat

```
##
## Female   Male   <NA>
##    339    407      0
```

```r
Phe2<-Phe[complete.cases(Phe$sex_cat), ] #NA
#Phe2<-subset(Phe2, sex_cat %in% c("Female", "Male")) #also removes NA, but above can be easier to impl
X<-dplyr::select(Microbio2, one_of(Phe2$IDX)) #Also in X
##Remove orgs that are not present after subsetting.
X <- X[rowSums(X)>0,]


#Hellinger transformation
X <- data.frame(t(decostand(t(X), method="hellinger")))
#Maks TSS
X<-sweep(X, 2, colSums(X), FUN="/")
#rowSums(X)
sum(colnames(X)!=Phe2$IDX)==0
```

```
## [1] TRUE
```

```r
X<-t(X)
Y<-Phe2$sex_cat

sum(rownames(X)!=Phe2$IDX)==0
```

```
## [1] TRUE
```

```r
dim(X) # check the dimensions of the X dataframe
```

```
## [1] 746  97
```

```r
summary(Y) # check the distribution of class labels
```

```
## Female   Male
##    339    407
```

```r
# Barplot of the variance each principal component explains
pca.addp = pca(X, ncomp = 10, center = TRUE, scale = TRUE) # run pca method on data
plot(pca.addp)  # barplot of the eigenvalues (explained variance per component)
```

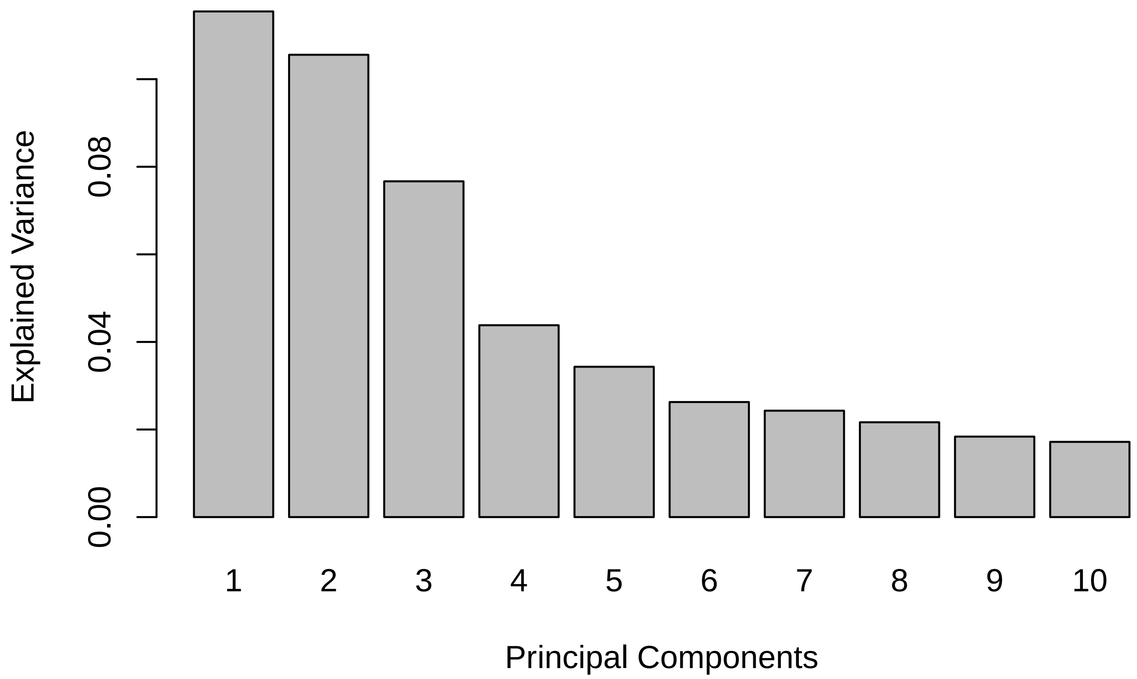


```r
# Preliminary (unsupervised) analysis with PCA
plotIndiv(pca.addp, group = Y, ind.names = FALSE, # plot the samples projected
          legend = TRUE, title = 'PCA on addp, comp 1 - 2') # onto the PCA subspace
```

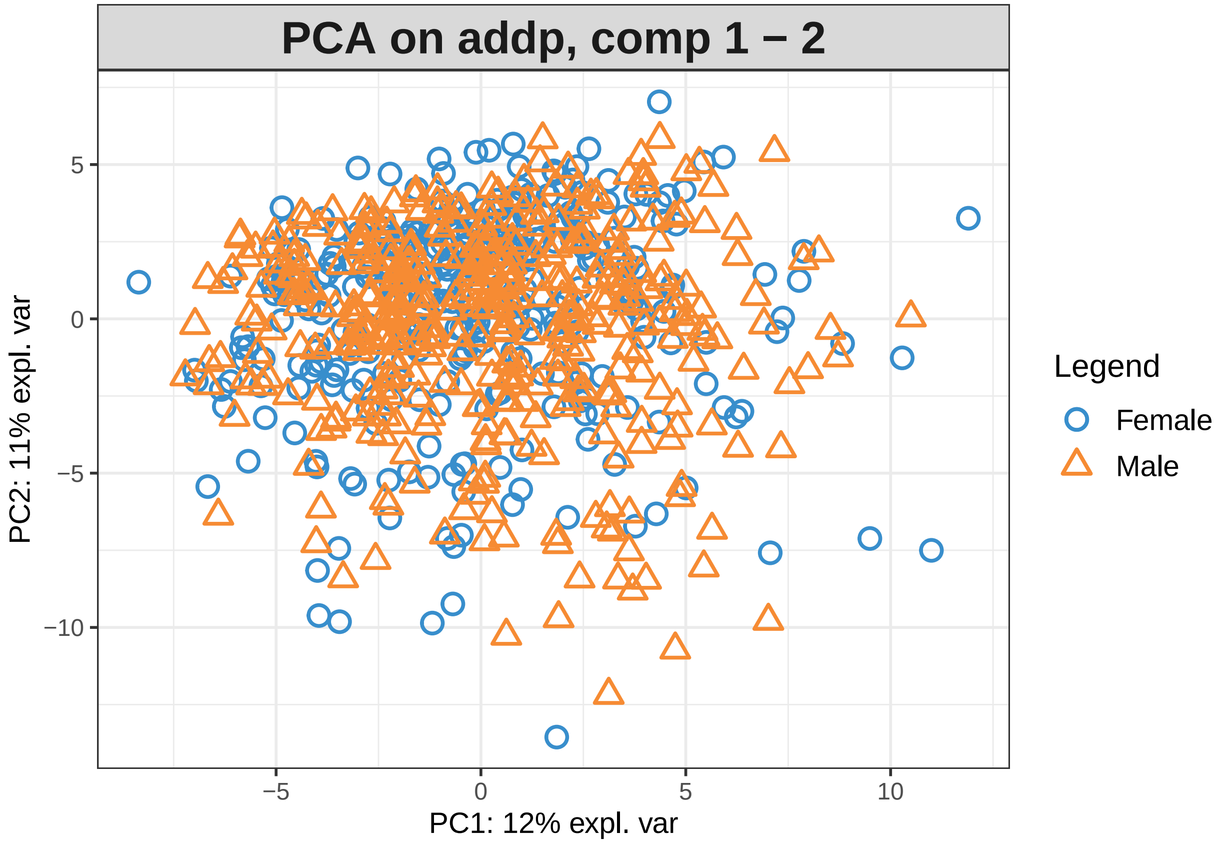


```
addp.splsda <- splsda(X, Y, ncomp = 10)  # set ncomp to 10 for performance assessment later

## Sample plots after a basic PLS-DA model was operated on this data.
# plot the samples projected onto the first two components of the PLS-DA subspace
plotIndiv(addp.splsda , comp = 1:2,
          group = Y, ind.names = FALSE,  # colour points by class
          ellipse = TRUE, # include 95% confidence ellipse for each class
          legend = TRUE, title = '(a) PLSDA with confidence ellipses')
```

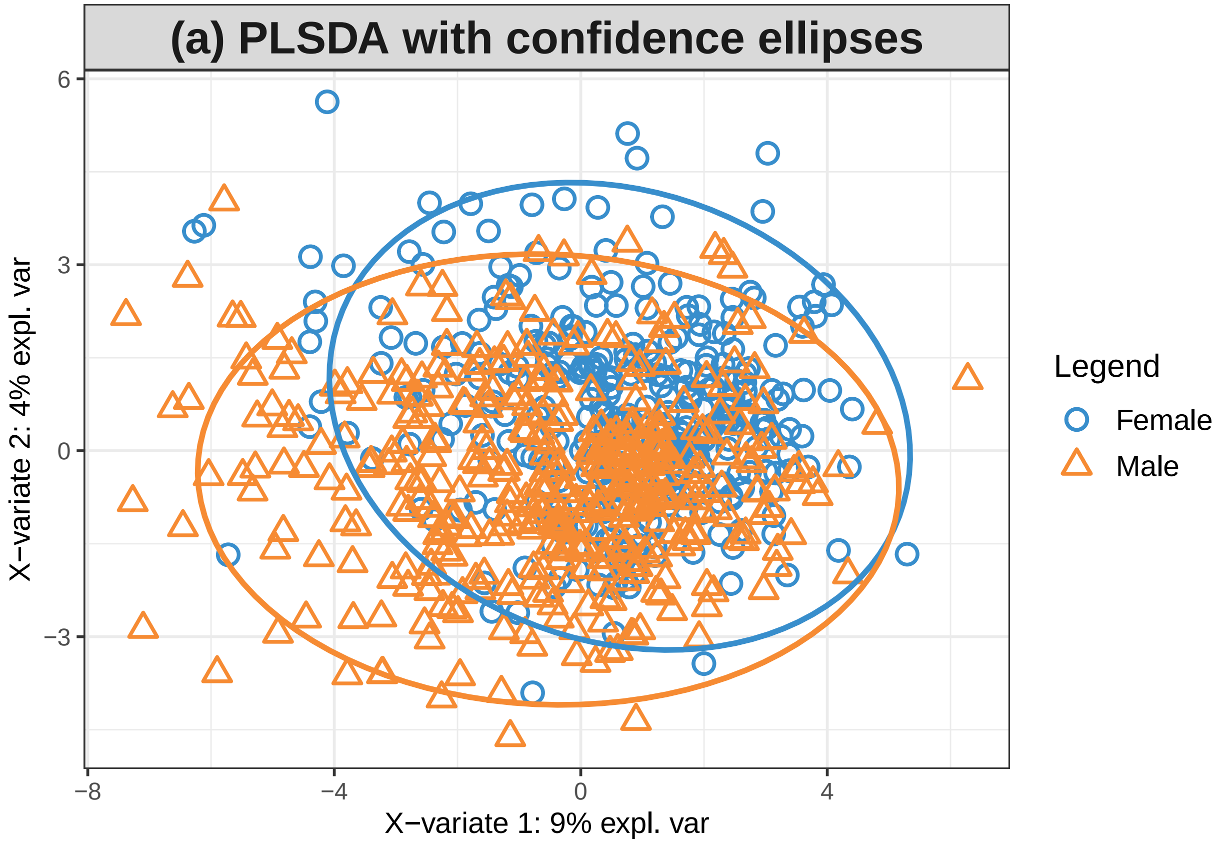


```
# use the max.dist measure to form decision boundaries between classes based on PLS-DA data
background = background.predict(addp.splsda, comp.predicted=2, dist = "max.dist")

# plot the samples projected onto the first two components of the PLS-DA subspace
plotIndiv(addp.splsda, comp = 1:2,
          group = Y, ind.names = FALSE, # colour points by class
          background = background, # include prediction background for each class
          legend = TRUE, title = " (b) PLSDA with prediction background")
```

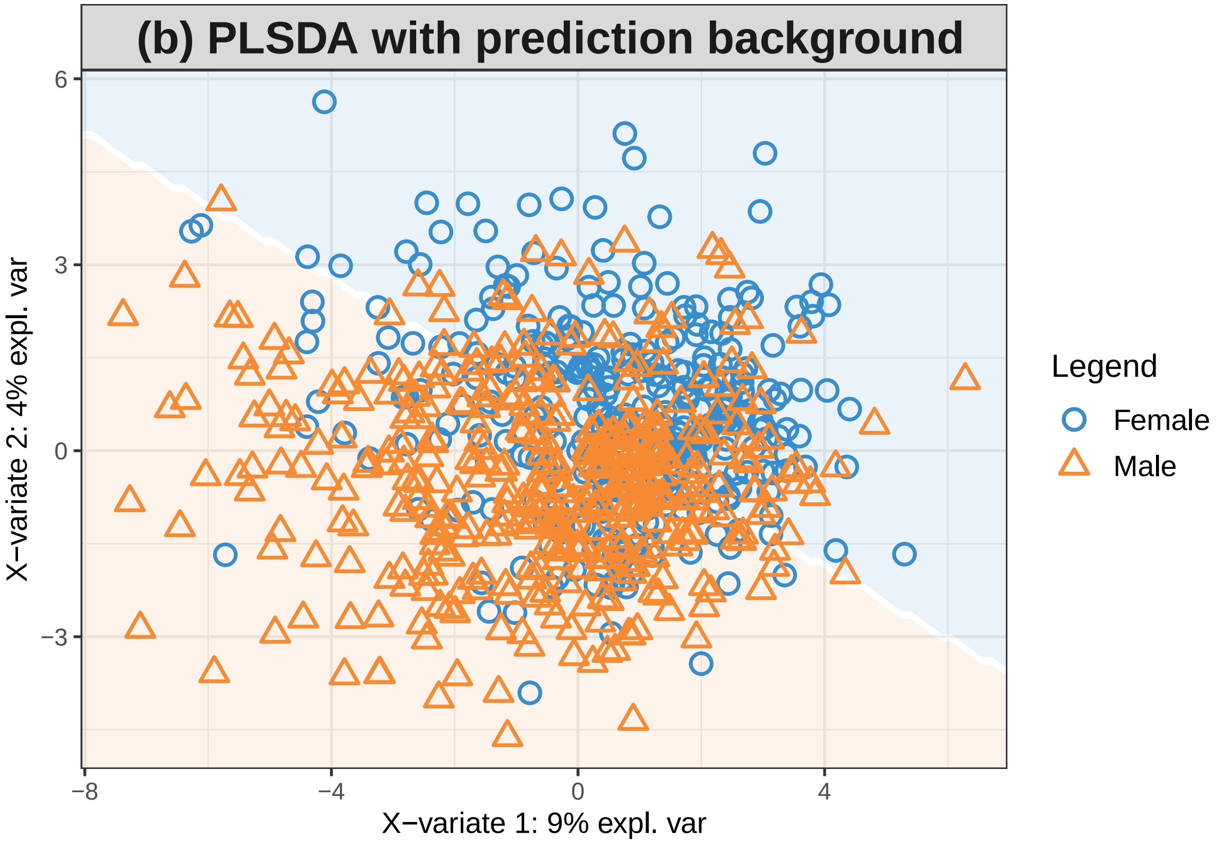


```
## Tuning the number of components in PLS-DA. For each component, repeated cross-validation (10 × 3-fol
# undergo performance evaluation in order to tune the number of components to use
perf.splsda.addp <- perf(addp.splsda, validation = "Mfold",
                         folds = 5, nrepeat = 10, # use repeated cross-validation
                         progressBar = FALSE, auc = TRUE) # include AUC values

# plot the outcome of performance evaluation across all ten components
plot(perf.splsda.addp, col = color.mixo(5:7), sd = TRUE,
     legend.position = "horizontal")
```

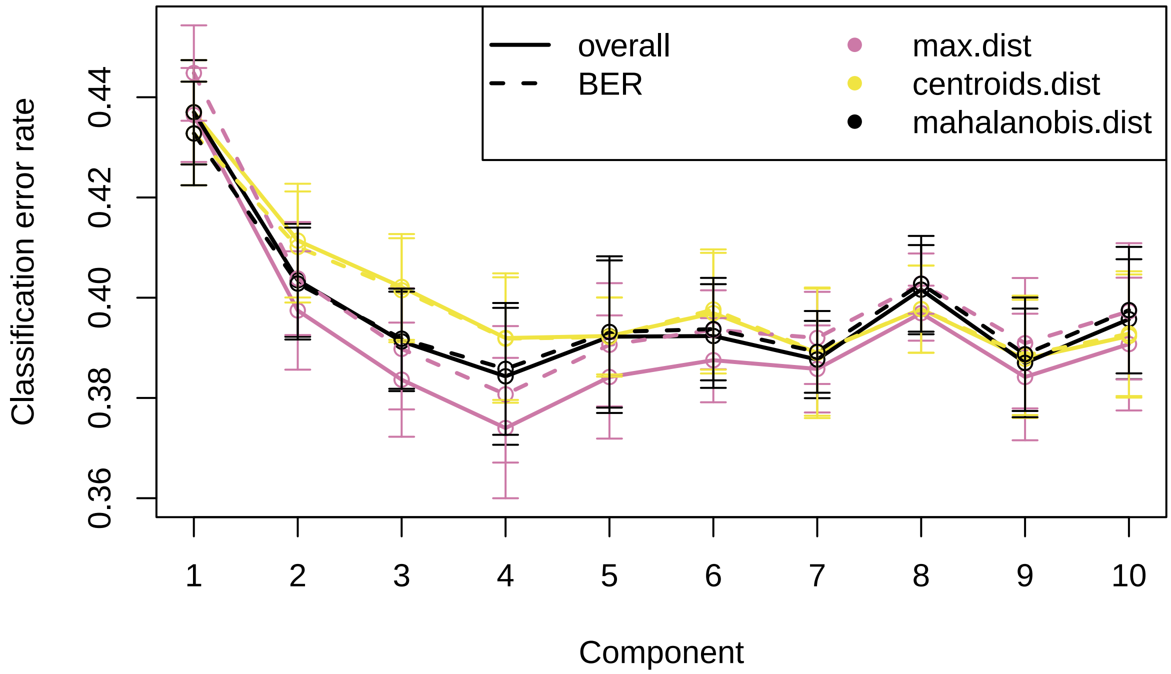


```
perf.splsda.addp$choice.ncomp # what is the optimal value of components according to perf()
```

```
##         max.dist centroids.dist mahalanobis.dist
## overall        3              4                4
## BER            3              4                4
```

```
## Tuning keepX for the sPLS-DA. Each coloured line represents the balanced error rate (y-axis) per comp
# grid of possible keepX values that will be tested for each component
list.keepX <- c(1:10,  seq(20, 300, 10))

# undergo the tuning process to determine the optimal number of variables
tune.splsda.addp <- tune.splsda(X, Y, ncomp = 2, # calculate for first 2 components
                                validation = 'Mfold',
                                folds = 5, nrepeat = 10, # use repeated cross-validation
                                dist = 'max.dist', # use max.dist measure
                                measure = "BER", # use balanced error rate of dist measure
                                test.keepX = list.keepX,
                                cpus = 2 # allow for parallelisation to decrease runtime
                                )

plot(tune.splsda.addp, col = color.jet(2))
```

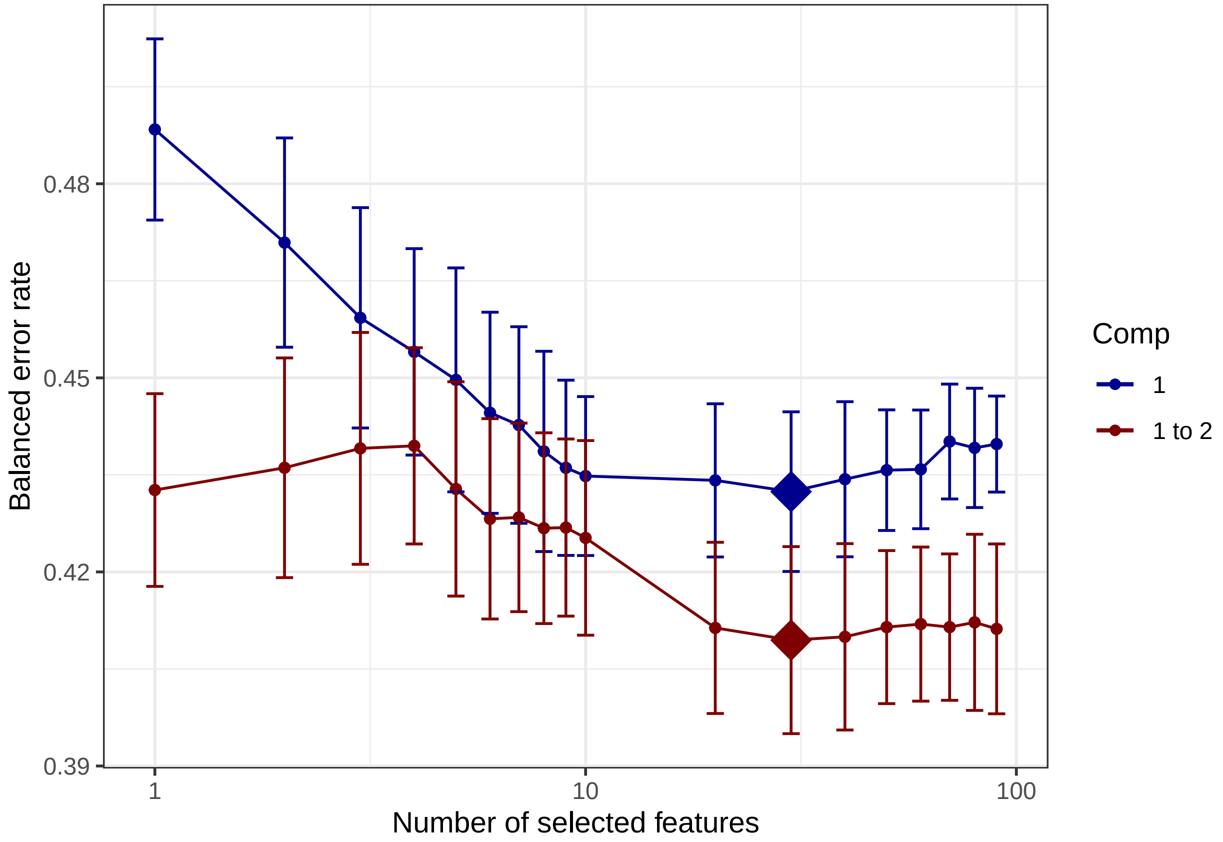


```r
tune.splsda.addp$choice.ncomp$ncomp # what is the optimal value of components according to tune.splsda(
```

```
## [1] 2
```

```r
tune.splsda.addp$choice.keepX # what are the optimal values of variables according to tune.splsda()
```

```
## comp1 comp2
##    30    30
```

```r
optimal.ncomp <- tune.splsda.addp$choice.ncomp$ncomp
optimal.keepX <- tune.splsda.addp$choice.keepX[1:optimal.ncomp]

# form final model with optimised values for component and variable count
final.splsda <- splsda(X, Y,
                       ncomp = optimal.ncomp,
                       keepX = optimal.keepX)

#########Loadings
#plotLoadings(final.splsda, comp=1, contrib = 'max', method = 'mean', size.title = 1)
#plotLoadings(final.splsda, comp=2, contrib = 'max', method = 'mean', size.title = 1)

comp1 <- plotLoadings(final.splsda, comp = 1, method = 'mean', contrib = 'max',
                      size.title = 1)
```

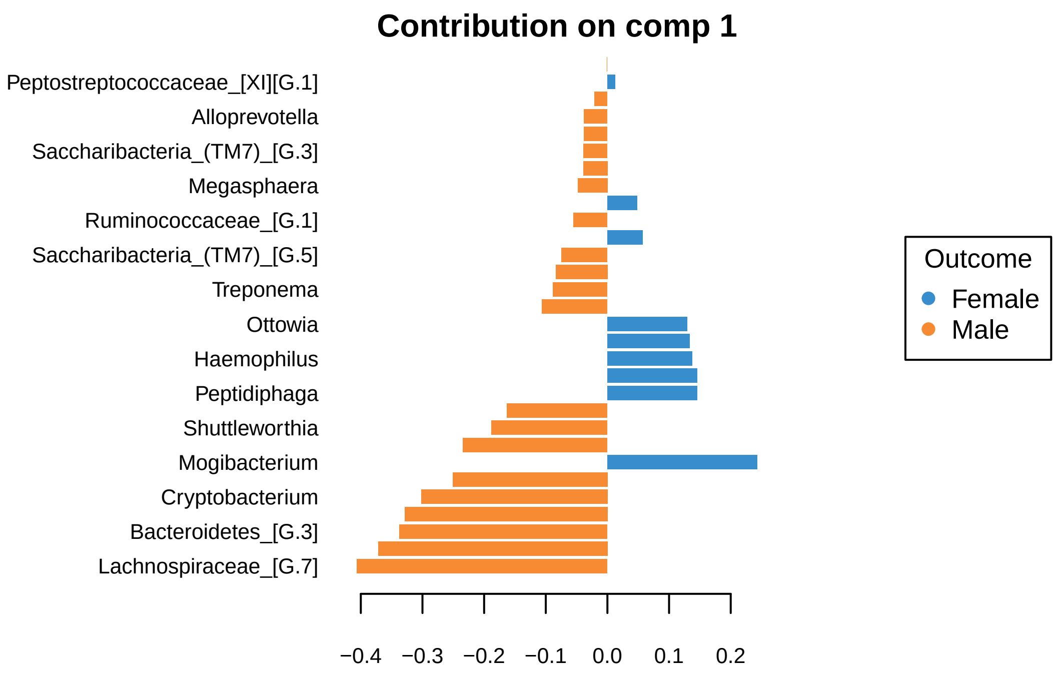


```r
head(comp1, n=20)
```

```
##                                    Female        Male Contrib.Female
## Lachnospiraceae_[G.7]         -0.15440191  0.12860503          FALSE
## Mitsuokella                   -0.14716532  0.12257750          FALSE
## Bacteroidetes_[G.3]           -0.13982976  0.11646754          FALSE
## Eggerthia                     -0.13806133  0.11499457          FALSE
## Cryptobacterium               -0.13236810  0.11025255          FALSE
## Bulleidia                     -0.12158475  0.10127084          FALSE
## Mogibacterium                  0.11990089 -0.09986831           TRUE
## Family.Selenomonadaceae.      -0.11805888  0.09833405          FALSE
## Shuttleworthia                -0.10834477  0.09024294          FALSE
## Alloscardovia                 -0.10304138  0.08582562          FALSE
## Peptidiphaga                   0.09940929 -0.08280037           TRUE
## Gemella                        0.09933270 -0.08273658           TRUE
## Haemophilus                    0.09768695 -0.08136579           TRUE
## Abiotrophia                    0.09673008 -0.08056879           TRUE
## Ottowia                        0.09594783 -0.07991723           TRUE
## Filifactor                    -0.09097981  0.07577926          FALSE
## Treponema                     -0.08723139  0.07265710          FALSE
## Clostridiales_[F.1][G-1]      -0.08628552  0.07186926          FALSE
## Saccharibacteria_(TM7)_[G.5] -0.08435140  0.07025829          FALSE
## Capnocytophaga                 0.08063138 -0.06715980           TRUE
##                               Contrib.Male Contrib GroupContrib   color
## Lachnospiraceae_[G.7]                 TRUE   FALSE         Male #F68B33
```

```
## Mitsuokella                          TRUE   FALSE         Male #F68B33
## Bacteroidetes_[G.3]                  TRUE   FALSE         Male #F68B33
## Eggerthia                            TRUE   FALSE         Male #F68B33
## Cryptobacterium                      TRUE   FALSE         Male #F68B33
## Bulleidia                            TRUE   FALSE         Male #F68B33
## Mogibacterium                       FALSE   FALSE       Female #388ECC
## Family.Selenomonadaceae.             TRUE   FALSE         Male #F68B33
## Shuttleworthia                       TRUE   FALSE         Male #F68B33
## Alloscardovia                        TRUE   FALSE         Male #F68B33
## Peptidiphaga                        FALSE   FALSE       Female #388ECC
## Gemella                             FALSE   FALSE       Female #388ECC
## Haemophilus                         FALSE   FALSE       Female #388ECC
## Abiotrophia                         FALSE   FALSE       Female #388ECC
## Ottowia                             FALSE   FALSE       Female #388ECC
## Filifactor                           TRUE   FALSE         Male #F68B33
## Treponema                            TRUE   FALSE         Male #F68B33
## Clostridiales_[F.1][G-1]             TRUE   FALSE         Male #F68B33
## Saccharibacteria_(TM7)_[G.5]         TRUE   FALSE         Male #F68B33
## Capnocytophaga                      FALSE   FALSE       Female #388ECC
##                               importance
## Lachnospiraceae_[G.7]        -0.40622399
## Mitsuokella                  -0.37196139
## Bacteroidetes_[G.3]          -0.33723024
## Eggerthia                    -0.32885737
## Cryptobacterium              -0.30190203
## Bulleidia                    -0.25084683
## Mogibacterium                 0.24287437
## Family.Selenomonadaceae.     -0.23415312
## Shuttleworthia               -0.18816039
## Alloscardovia                -0.16305079
## Peptidiphaga                  0.14585419
## Gemella                       0.14549158
## Haemophilus                   0.13769955
## Abiotrophia                   0.13316914
## Ottowia                       0.12946543
## Filifactor                   -0.10594373
## Treponema                    -0.08819630
## Clostridiales_[F.1][G-1]     -0.08371796
## Saccharibacteria_(TM7)_[G.5] -0.07456063
## Capnocytophaga                0.05694769
```

```r
write.table(comp1, file="sex_comp1.txt", sep="\t", dec=",", row.names=T)
comp2 <- plotLoadings(final.splsda, comp = 2, method = 'mean', contrib = 'max',
                      size.title = 1)
```

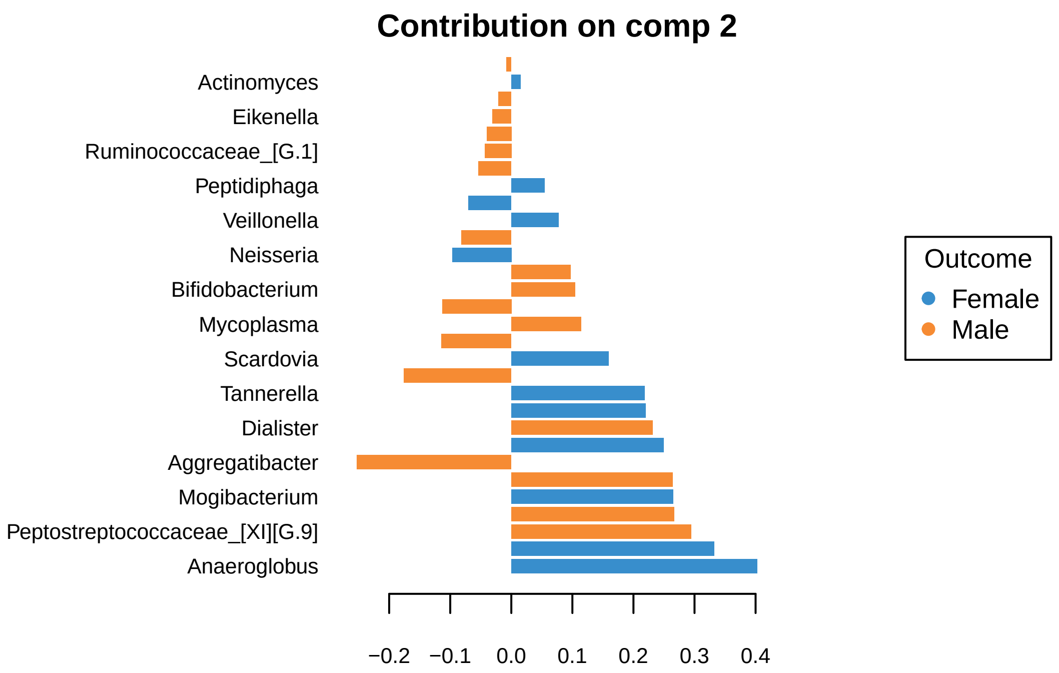


```
head(comp2, n=20)
```

```
##                                        Female          Male Contrib.Female
## Anaeroglobus                      0.012122752 -0.0100973292           TRUE
## Bacteroidaceae_[G.1]              0.037476574 -0.0312151315           TRUE
## Peptostreptococcaceae_[XI][G.9] -0.029509173  0.0245788934          FALSE
## Fretibacterium                  -0.050137347  0.0417605909          FALSE
## Mogibacterium                     0.119900891 -0.0998683099           TRUE
## Peptostreptococcaceae_[XI][G.4] -0.003927275  0.0032711212          FALSE
## Aggregatibacter                 -0.028940363  0.0241051177          FALSE
## Lachnospiraceae_[G.8]             0.014339715 -0.0119438907           TRUE
## Dialister                       -0.060809767  0.0506499040          FALSE
## Selenomonas                       0.015558658 -0.0129591774           TRUE
## Tannerella                        0.037172506 -0.0309618666           TRUE
## Oribacterium                    -0.063520536  0.0529077684          FALSE
## Scardovia                         0.009226443 -0.0076849240           TRUE
## Absconditabacteria_(SR1)_[G.1]  -0.042794658  0.0356446906          FALSE
## Mycoplasma                      -0.042770652  0.0356246951          FALSE
## Kingella                        -0.000808683  0.0006735713          FALSE
## Bifidobacterium                 -0.024058059  0.0200385305          FALSE
## Treponema                       -0.087231387  0.0726571009          FALSE
## Neisseria                         0.061529443 -0.0512493394           TRUE
## Alloprevotella                  -0.076569207  0.0637763175          FALSE
##                                  Contrib.Male Contrib GroupContrib   color
## Anaeroglobus                            FALSE   FALSE       Female #388ECC
```

```
## Bacteroidaceae_[G.1]                  FALSE   FALSE       Female #388ECC
## Peptostreptococcaceae_[XI][G.9]        TRUE   FALSE         Male #F68B33
## Fretibacterium                         TRUE   FALSE         Male #F68B33
## Mogibacterium                         FALSE   FALSE       Female #388ECC
## Peptostreptococcaceae_[XI][G.4]        TRUE   FALSE         Male #F68B33
## Aggregatibacter                        TRUE   FALSE         Male #F68B33
## Lachnospiraceae_[G.8]                 FALSE   FALSE       Female #388ECC
## Dialister                              TRUE   FALSE         Male #F68B33
## Selenomonas                           FALSE   FALSE       Female #388ECC
## Tannerella                            FALSE   FALSE       Female #388ECC
## Oribacterium                           TRUE   FALSE         Male #F68B33
## Scardovia                             FALSE   FALSE       Female #388ECC
## Absconditabacteria_(SR1)_[G.1]         TRUE   FALSE         Male #F68B33
## Mycoplasma                             TRUE   FALSE         Male #F68B33
## Kingella                               TRUE   FALSE         Male #F68B33
## Bifidobacterium                        TRUE   FALSE         Male #F68B33
## Treponema                              TRUE   FALSE         Male #F68B33
## Neisseria                             FALSE   FALSE       Female #388ECC
## Alloprevotella                         TRUE   FALSE         Male #F68B33
##                                  importance
## Anaeroglobus                     0.40259938
## Bacteroidaceae_[G.1]             0.33203149
## Peptostreptococcaceae_[XI][G.9]  0.29425866
## Fretibacterium                   0.26697158
## Mogibacterium                    0.26538019
## Peptostreptococcaceae_[XI][G.4]  0.26456866
## Aggregatibacter                 -0.25273034
## Lachnospiraceae_[G.8]            0.24968809
## Dialister                        0.23137238
## Selenomonas                      0.21997408
## Tannerella                       0.21824228
## Oribacterium                    -0.17575608
## Scardovia                        0.15916741
## Absconditabacteria_(SR1)_[G.1]  -0.11462020
## Mycoplasma                       0.11449680
## Kingella                        -0.11311181
## Bifidobacterium                  0.10414916
## Treponema                        0.09732315
## Neisseria                       -0.09673801
## Alloprevotella                  -0.08148411
```

```
write.table(comp2, file="sex_comp2.txt", sep="\t", dec=",", row.names=T)

##  Sample plots from sPLS-DA including 95% confidence ellipses. Samples are projected into the space s
plotIndiv(final.splsda, comp = c(1,2), # plot samples from final model
          group = Y, ind.names = FALSE, # colour by class label
          ellipse = TRUE, legend = TRUE, # include 95% confidence ellipse
          title = ' (a) sPLS-DA on addp, comp 1 & 2')
```

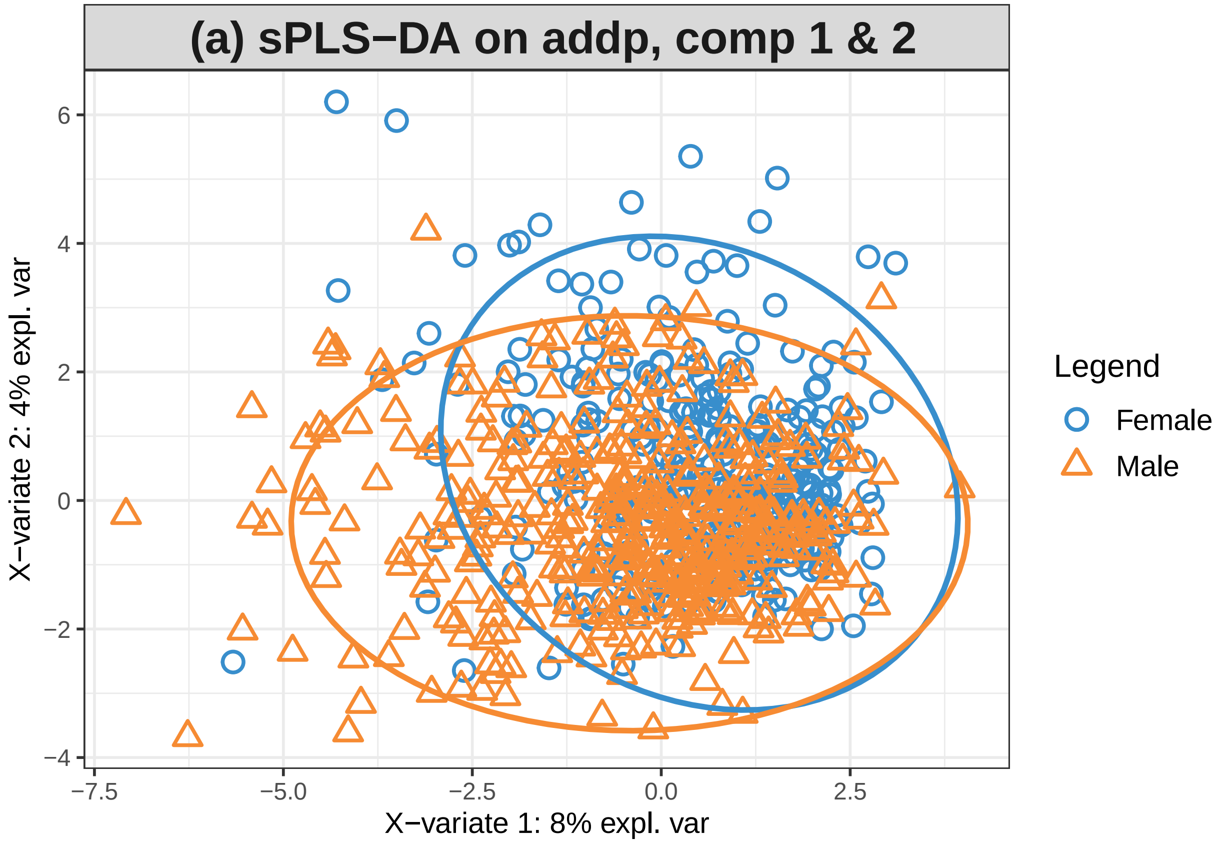


```
# plotIndiv(final.splsda, comp = c(1,3), # plot samples from final model
#           group = Y, ind.names = FALSE,  # colour by class label
#           ellipse = TRUE, legend = TRUE, # include 95% confidence ellipse
#           title = '(b) sPLS-DA on addp, comp 1 & 3')

##Stability of variable selection from the sPLS-DA. The barplot represents the frequency of selection a
# form new perf() object which utilises the final model
perf.splsda.addp <- perf(final.splsda,
                          folds = 5, nrepeat = 10, # use repeated cross-validation
                          validation = "Mfold", dist = "max.dist",  # use max.dist measure
                          progressBar = FALSE)

# plot the stability of each feature for the first three components, 'h' type refers to histogram
par(mfrow=c(1,2))
plot(perf.splsda.addp$features$stable[[1]], type = 'h',
     ylab = 'Stability',
     xlab = 'Features',
     main = '(a) Comp 1', las =2)
plot(perf.splsda.addp$features$stable[[2]], type = 'h',
     ylab = 'Stability',
     xlab = 'Features',
     main = '(b) Comp 2', las =2)
```

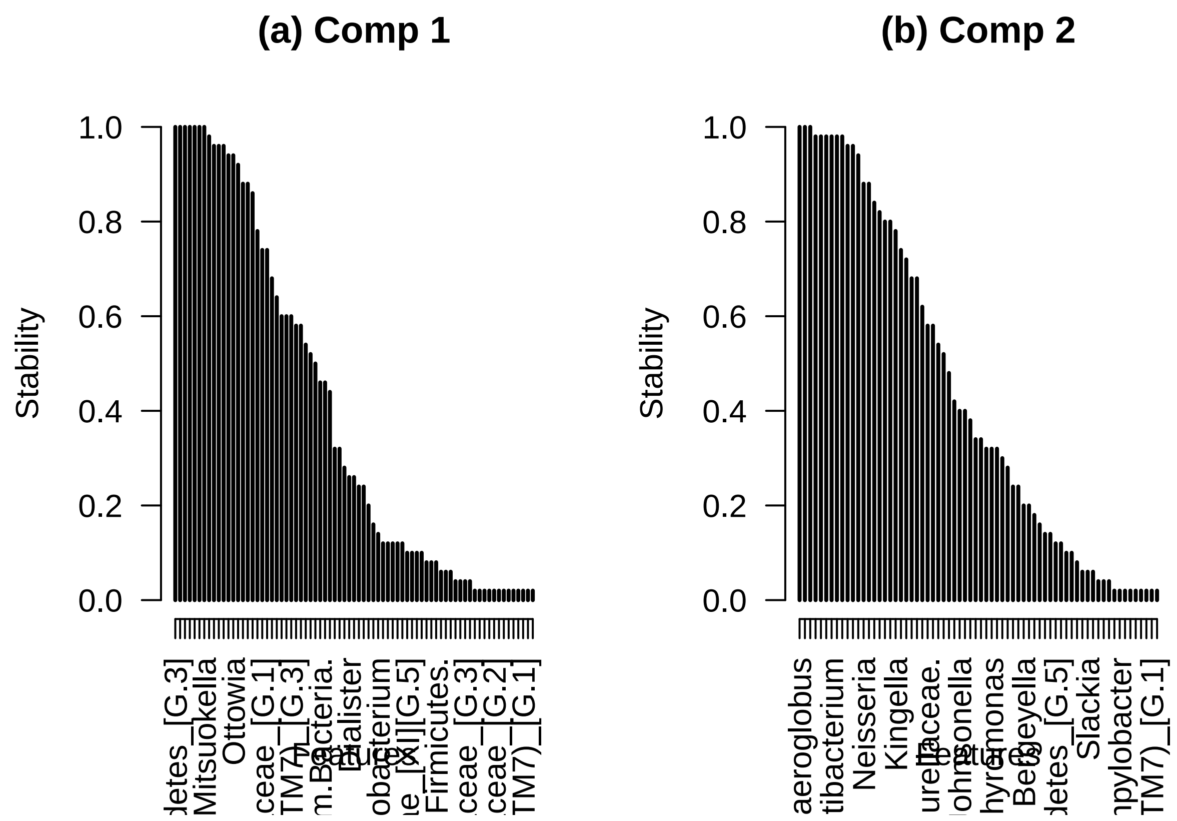


```
# plot(perf.splsda.addp$features$stable[[3]], type = 'h',
#      ylab = 'Stability',
#      xlab = 'Features',
#      main = '(c) Comp 3', las =2)
par(mfrow=c(1,1))


train <- sample(1:nrow(X), 0.75*nrow(X)) # randomly select 75% of samples in training
test <- setdiff(1:nrow(X), train) # rest is part of the test set

# store matrices into training and test set:
X.train <- X[train, ]
X.test <- X[test,]
Y.train <- Y[train]
Y.test <- Y[test]

# train the model
train.splsda.addp <- splsda(X.train, Y.train, ncomp = optimal.ncomp, keepX = optimal.keepX)

# use the model on the Xtest set
predict.splsda.addp <- predict(train.splsda.addp, X.test, dist = "max.dist")

# evaluate the prediction accuracy for the first two components
predict.comp2 <- predict.splsda.addp$class$max.dist[,2]
table(factor(predict.comp2, levels = c("Female", "Male")), Y.test)
```

```
##          Y.test
##           Female Male
##   Female      26   39
##   Male        52   70
```

```
#Correct classification rate
sum(diag(table(factor(predict.comp2, levels = c("Female", "Male")), Y.test)))/
  sum(table(factor(predict.comp2, levels = c("Female", "Male")), Y.test))
```

```
## [1] 0.513369
```

```
## ROC curve and AUC from sPLS-DA on component 1 (a) and all (two) components (b) averaged across one-v
auc.splsda = auroc(final.splsda, roc.comp = 1, print = FALSE) # AUROC for the first component
```

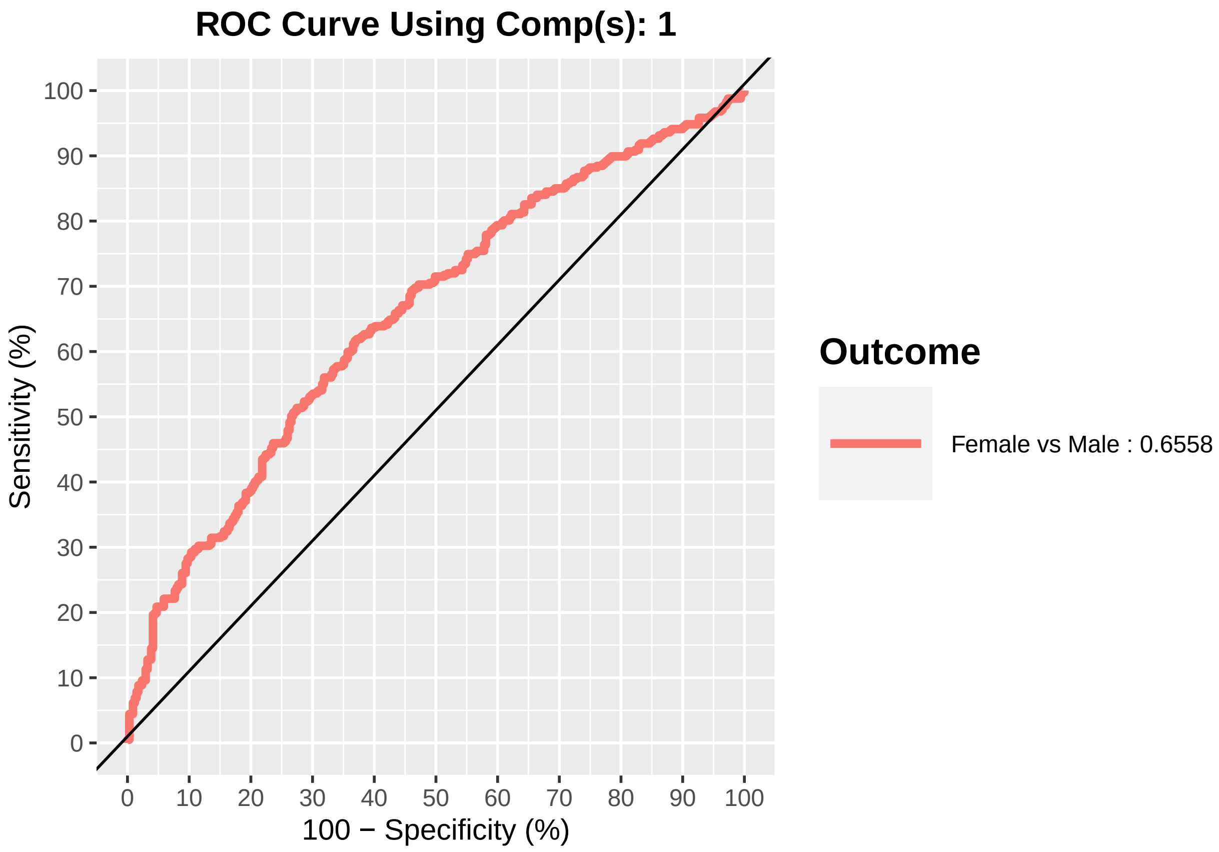


```
auc.splsda = auroc(final.splsda, roc.comp = 2, print = FALSE) # AUROC for the first and second componen
```

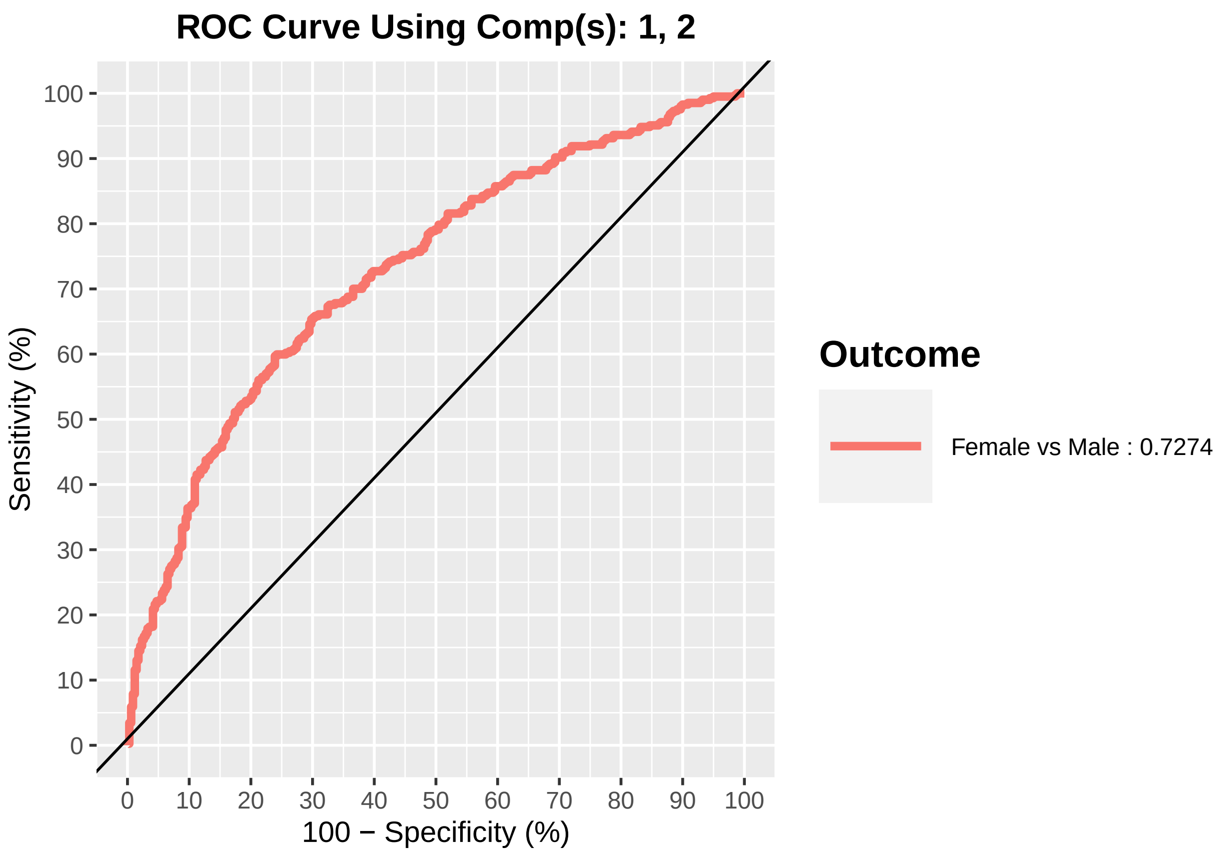


```
ROClist[["sexROC"]] <-auc.splsda$graph.Comp2


#AUC
print("AUC")
```

```
## [1] "AUC"
```

```
auc.splsda$Comp2
```

```
##                    AUC p-value
## Female vs Male 0.7274       0
```

```


rm(list=setdiff(ls(), c("FeaturePic2", "Pic2", "Phe", "Microbio", "FeatureMic", "Metabo", "paretoscale"

set.seed(99) # for reproducibility, remove for normal use

Microbio2<-Microbio
#Subset select or just remove NA
table(Phe$Risk, useNA="always")
```

Risk

```
##
##      Low     High      KDM unclass     <NA>
##      421      301       21        0        3
```

```
Phe2<-Phe[complete.cases(Phe$Risk), ] #NA
Phe2<-subset(Phe2, Risk %in% c("High", "Low")) #also removes NA, but above can be easier to implement i
Phe2$Risk<-droplevels(Phe2$Risk)
X<-dplyr::select(Microbio2, one_of(Phe2$IDX)) #Also in X
##Remove orgs that are not present after subsetting.
X <- X[rowSums(X)>0,]


#Hellinger transformation
X <- data.frame(t(decostand(t(X), method="hellinger")))
#Maks TSS
X<-sweep(X, 2, colSums(X), FUN="/")
#rowSums(X)
sum(colnames(X)!=Phe2$IDX)==0
```

```
## [1] TRUE
```

```
X<-t(X)
Y<-Phe2$Risk

sum(rownames(X)!=Phe2$IDX)==0
```

```
## [1] TRUE
```

```
dim(X) # check the dimensions of the X dataframe
```

```
## [1] 722  97
```

```
summary(Y) # check the distribution of class labels
```

```
##  Low High
##  421  301
```

```
# Barplot of the variance each principal component explains
pca.addp = pca(X, ncomp = 10, center = TRUE, scale = TRUE) # run pca method on data
plot(pca.addp)  # barplot of the eigenvalues (explained variance per component)
```

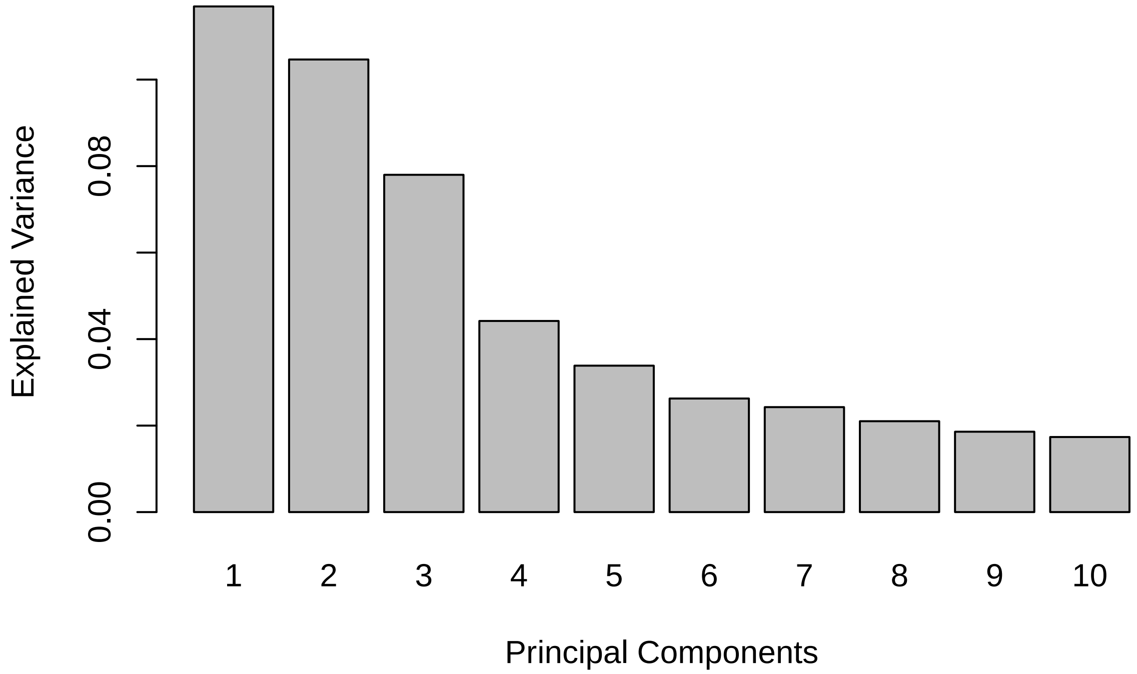


```r
# Preliminary (unsupervised) analysis with PCA
plotIndiv(pca.addp, group = Y, ind.names = FALSE, # plot the samples projected
          legend = TRUE, title = 'PCA on addp, comp 1 - 2') # onto the PCA subspace
```

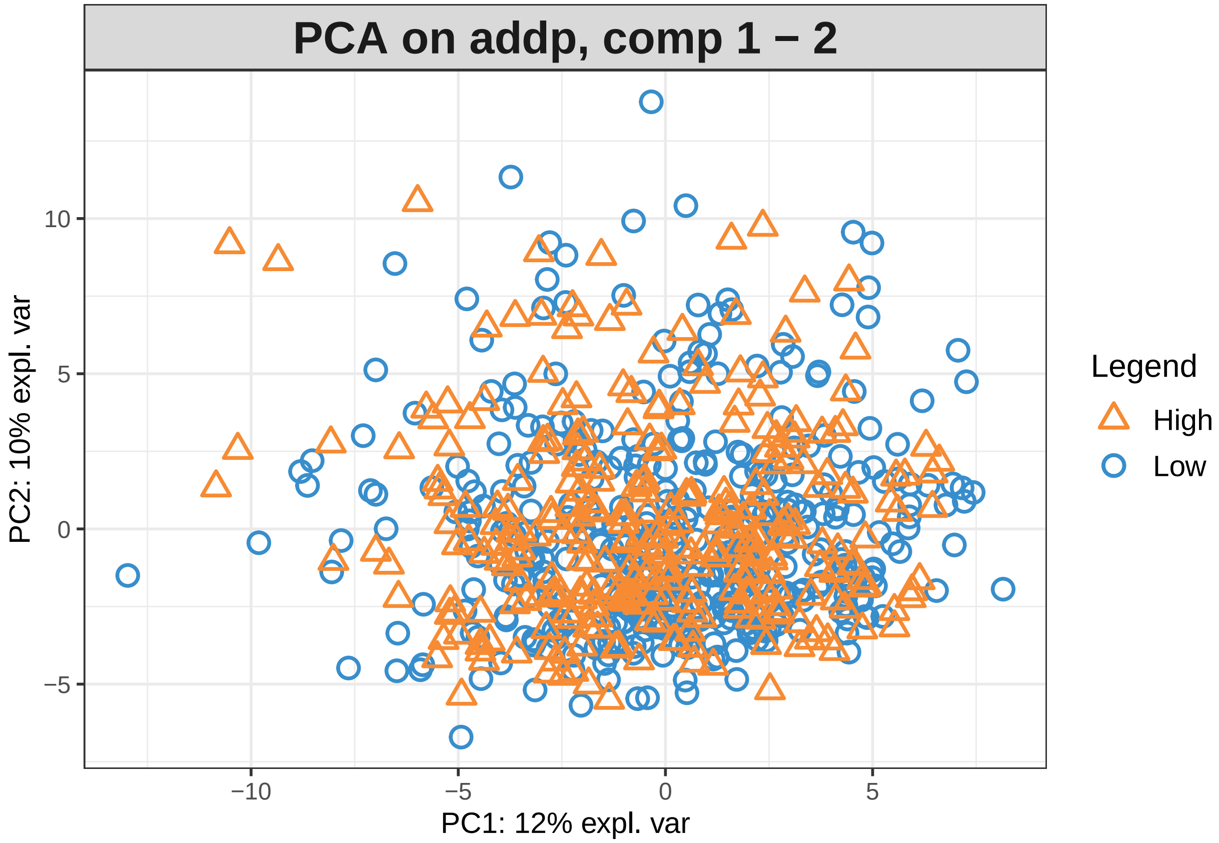


```
addp.splsda <- splsda(X, Y, ncomp = 10)  # set ncomp to 10 for performance assessment later

## Sample plots after a basic PLS-DA model was operated on this data.
# plot the samples projected onto the first two components of the PLS-DA subspace
plotIndiv(addp.splsda , comp = 1:2,
          group = Y, ind.names = FALSE,  # colour points by class
          ellipse = TRUE, # include 95% confidence ellipse for each class
          legend = TRUE, title = '(a) PLSDA with confidence ellipses')
```

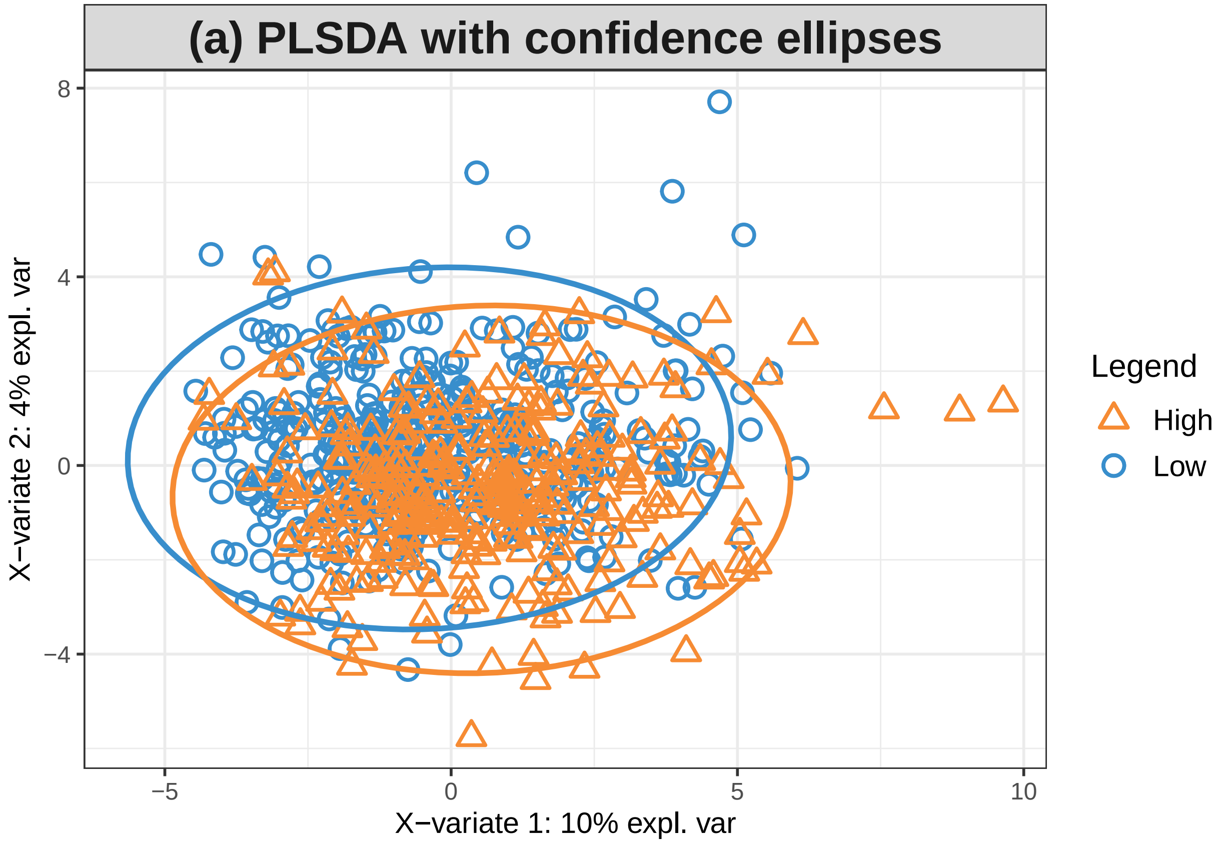


```
# use the max.dist measure to form decision boundaries between classes based on PLS-DA data
background = background.predict(addp.splsda, comp.predicted=2, dist = "max.dist")

# plot the samples projected onto the first two components of the PLS-DA subspace
plotIndiv(addp.splsda, comp = 1:2,
          group = Y, ind.names = FALSE, # colour points by class
          background = background, # include prediction background for each class
          legend = TRUE, title = " (b) PLSDA with prediction background")
```

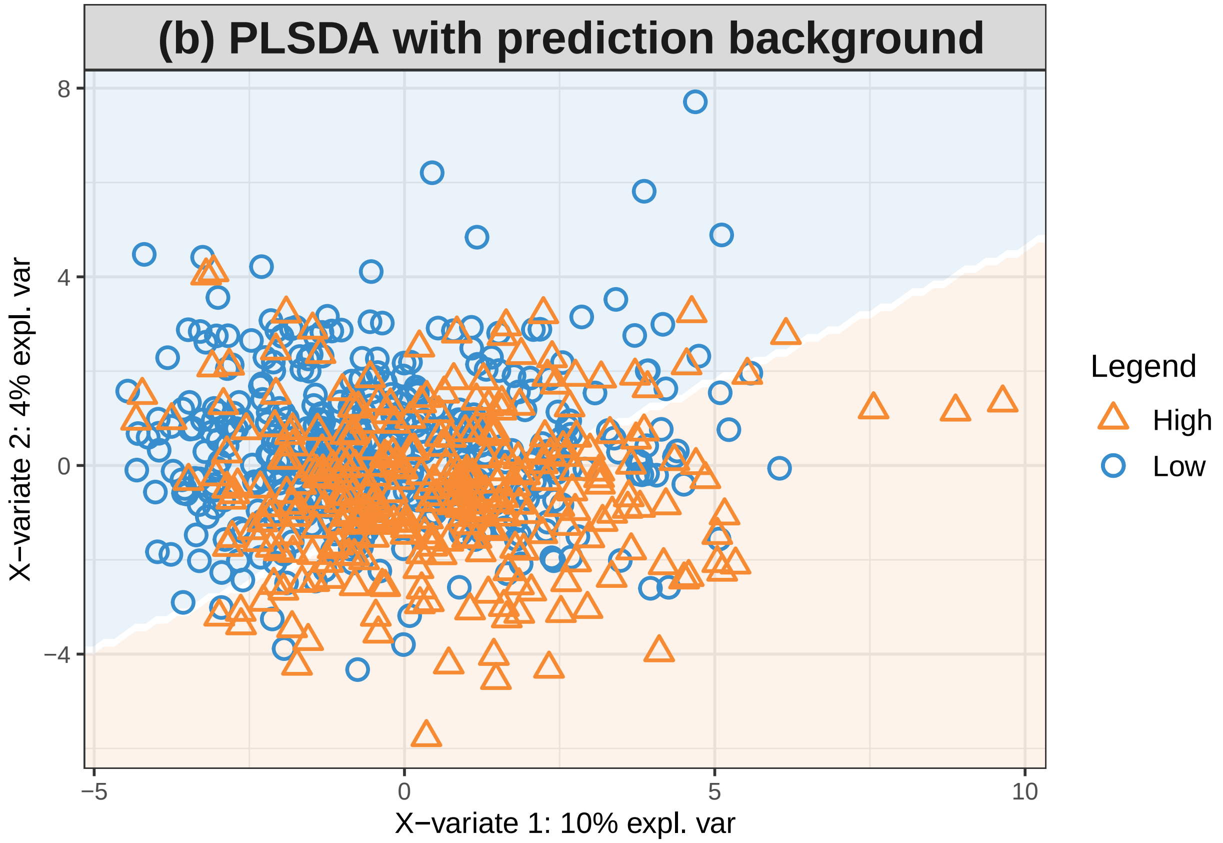


```
## Tuning the number of components in PLS-DA. For each component, repeated cross-validation (10 × 3-fol
# undergo performance evaluation in order to tune the number of components to use
perf.splsda.addp <- perf(addp.splsda, validation = "Mfold",
                         folds = 5, nrepeat = 10, # use repeated cross-validation
                         progressBar = FALSE, auc = TRUE) # include AUC values

# plot the outcome of performance evaluation across all ten components
plot(perf.splsda.addp, col = color.mixo(5:7), sd = TRUE,
     legend.position = "horizontal")
```

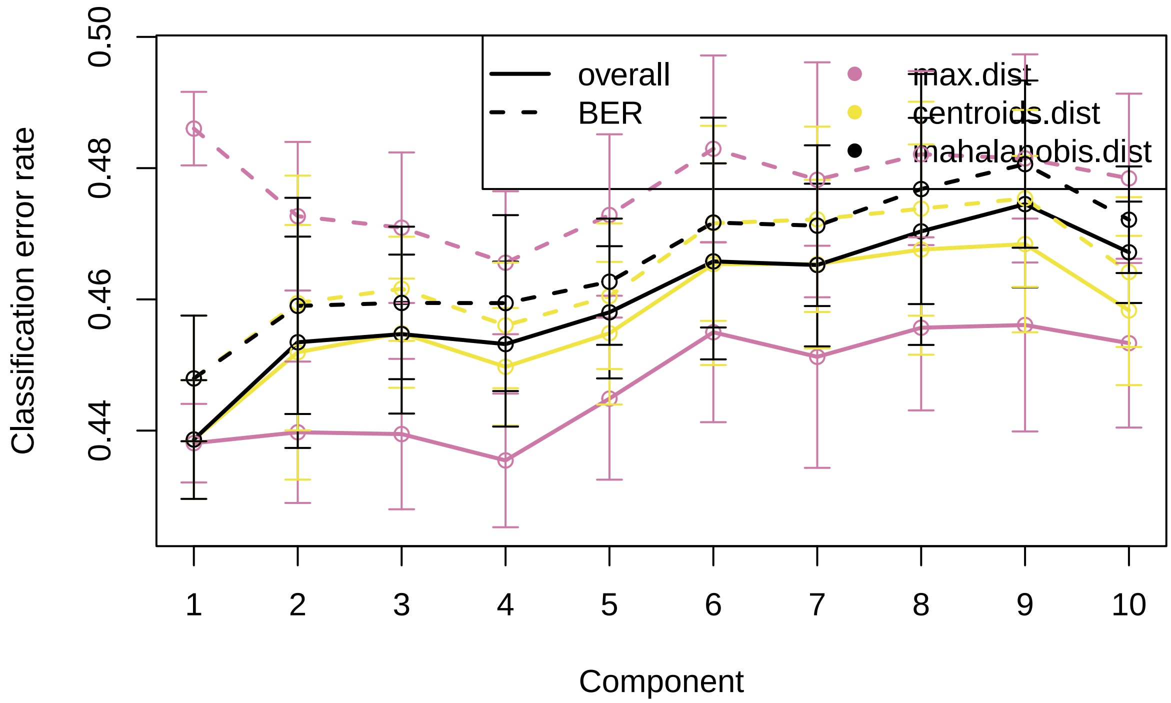


```
perf.splsda.addp$choice.ncomp # what is the optimal value of components according to perf()
```

```
##         max.dist centroids.dist mahalanobis.dist
## overall        2              1                1
## BER            2              1                1
```

```
## Tuning keepX for the sPLS-DA. Each coloured line represents the balanced error rate (y-axis) per com
# grid of possible keepX values that will be tested for each component
list.keepX <- c(1:10,  seq(20, 100, 10))

# undergo the tuning process to determine the optimal number of variables
tune.splsda.addp <- tune.splsda(X, Y, ncomp = 2, # calculate for first 2 components
                                validation = 'Mfold',
                                folds = 5, nrepeat = 10, # use repeated cross-validation
                                dist = 'max.dist', # use max.dist measure
                                measure = "BER", # use balanced error rate of dist measure
                                test.keepX = list.keepX,
                                cpus = 2 # allow for parallelisation to decrease runtime
                                )


plot(tune.splsda.addp, col = color.jet(2))
```

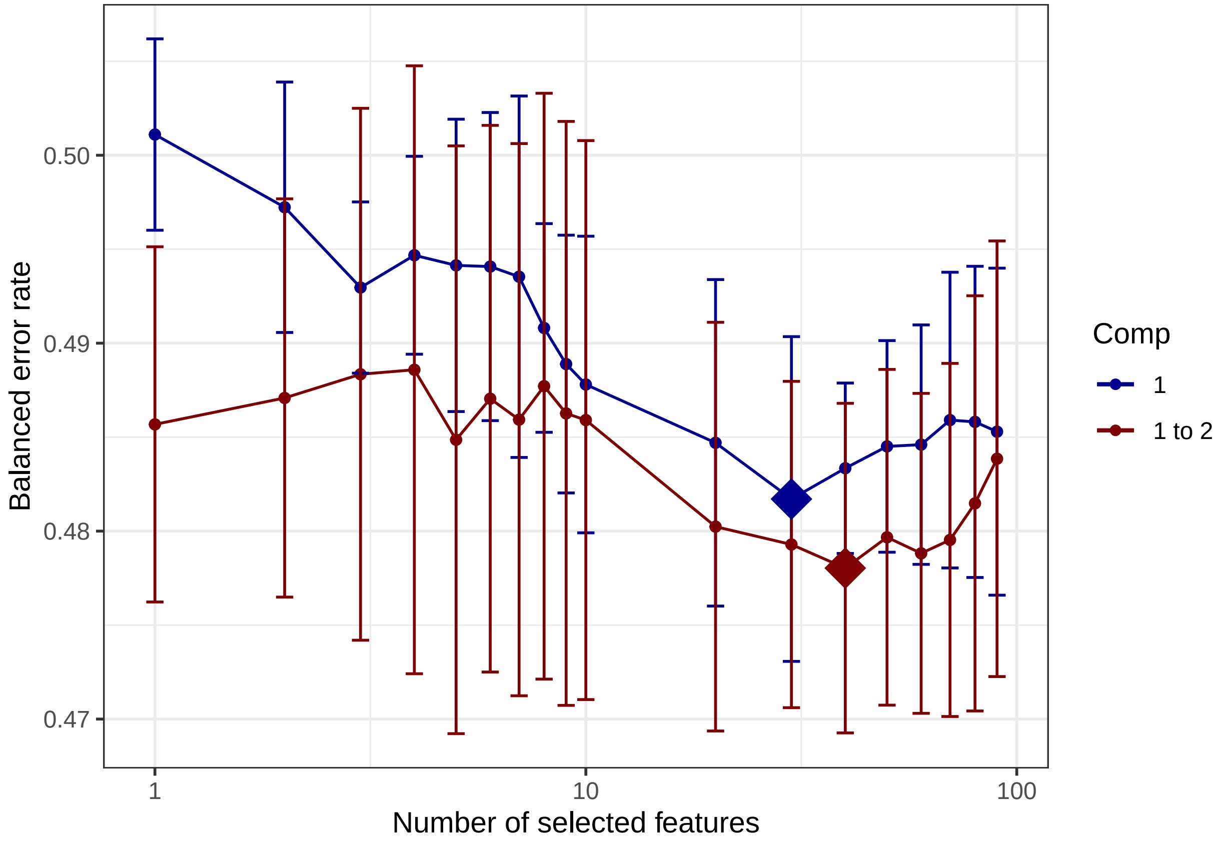


```
tune.splsda.addp$choice.ncomp$ncomp # what is the optimal value of components according to tune.splsda(
```

```
## [1] 1
```

```
tune.splsda.addp$choice.keepX # what are the optimal values of variables according to tune.splsda()
```

```
## comp1 comp2
##    30    40
```

```
#optimal.ncomp <- tune.splsda.addp$choice.ncomp$ncomp
optimal.ncomp <- 2 #manually set, see perf.splsda.addp$choice.ncomp above
optimal.keepX <- tune.splsda.addp$choice.keepX[1:optimal.ncomp]

# form final model with optimised values for component and variable count
final.splsda <- splsda(X, Y,
                       ncomp = optimal.ncomp,
                       keepX = optimal.keepX)

#########Loadings
#plotLoadings(final.splsda, comp=1, contrib = 'max', method = 'mean', size.title = 1)
#plotLoadings(final.splsda, comp=2, contrib = 'max', method = 'mean', size.title = 1)

comp1 <- plotLoadings(final.splsda, comp = 1, method = 'mean', contrib = 'max',
                      size.title = 1)
```

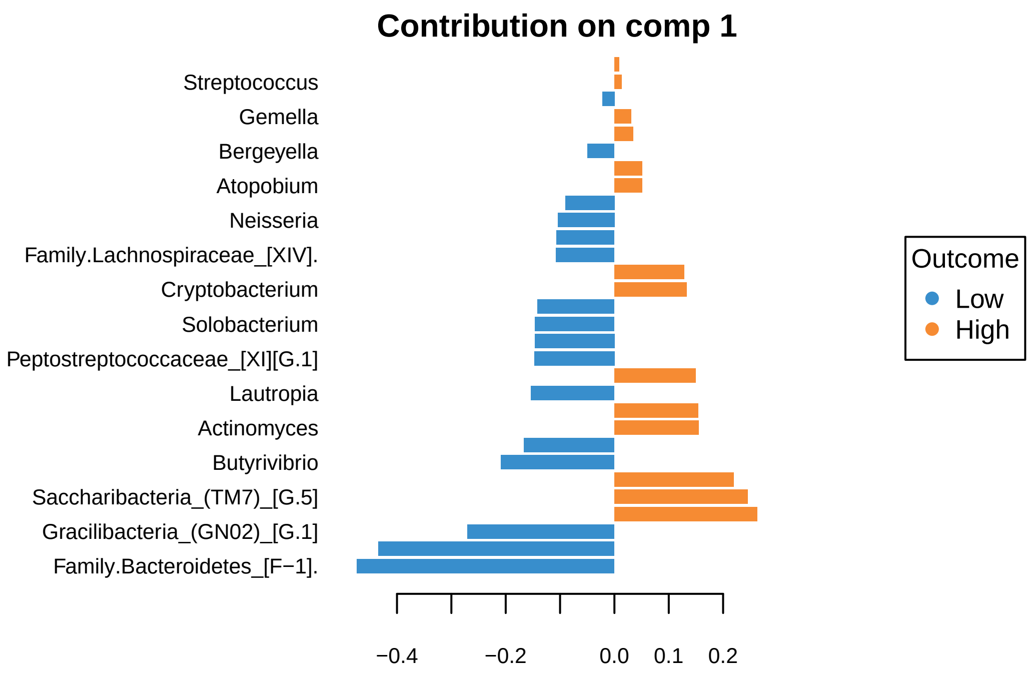


```r
head(comp1, n=20)
```

```
##                                          Low        High Contrib.Low
## Family.Bacteroidetes_[F-1].       0.10566735 -0.14779387        TRUE
## Lachnospiraceae_[G.2]             0.10011883 -0.14003331        TRUE
## Gracilibacteria_(GN02)_[G.1]      0.07696546 -0.10764936        TRUE
## Corynebacterium                  -0.07586840  0.10611494       FALSE
## Saccharibacteria_(TM7)_[G.5]     -0.07339662  0.10265773       FALSE
## Lachnospiraceae_[G.7]            -0.06973358  0.09753434       FALSE
## Butyrivibrio                      0.06825412 -0.09546507        TRUE
## Abiotrophia                       0.06228867 -0.08712136        TRUE
## Actinomyces                      -0.06067348  0.08486225       FALSE
## Bacteroidaceae_[G.1]             -0.06060311  0.08476382       FALSE
## Lautropia                         0.06046597 -0.08457200        TRUE
## Anaeroglobus                     -0.05993433  0.08382841       FALSE
## Peptostreptococcaceae_[XI][G.1]   0.05956834 -0.08331651        TRUE
## Leptotrichia                      0.05944067 -0.08313794        TRUE
## Solobacterium                     0.05937628 -0.08304788        TRUE
## Aggregatibacter                   0.05873027 -0.08214433        TRUE
## Cryptobacterium                  -0.05759185  0.08055206       FALSE
## Schaalia                         -0.05684180  0.07950298       FALSE
## Family.Lachnospiraceae_[XIV].     0.05393544 -0.07543794        TRUE
## Fusobacterium                     0.05381672 -0.07527190        TRUE
##                                  Contrib.High Contrib GroupContrib   color
## Family.Bacteroidetes_[F-1].             FALSE   FALSE          Low #388ECC
```

```
## Lachnospiraceae_[G.2]                   FALSE   FALSE          Low #388ECC
## Gracilibacteria_(GN02)_[G.1]            FALSE   FALSE          Low #388ECC
## Corynebacterium                          TRUE   FALSE         High #F68B33
## Saccharibacteria_(TM7)_[G.5]             TRUE   FALSE         High #F68B33
## Lachnospiraceae_[G.7]                    TRUE   FALSE         High #F68B33
## Butyrivibrio                            FALSE   FALSE          Low #388ECC
## Abiotrophia                             FALSE   FALSE          Low #388ECC
## Actinomyces                              TRUE   FALSE         High #F68B33
## Bacteroidaceae_[G.1]                     TRUE   FALSE         High #F68B33
## Lautropia                               FALSE   FALSE          Low #388ECC
## Anaeroglobus                             TRUE   FALSE         High #F68B33
## Peptostreptococcaceae_[XI][G.1]         FALSE   FALSE          Low #388ECC
## Leptotrichia                            FALSE   FALSE          Low #388ECC
## Solobacterium                           FALSE   FALSE          Low #388ECC
## Aggregatibacter                         FALSE   FALSE          Low #388ECC
## Cryptobacterium                          TRUE   FALSE         High #F68B33
## Schaalia                                 TRUE   FALSE         High #F68B33
## Family.Lachnospiraceae_[XIV].           FALSE   FALSE          Low #388ECC
## Fusobacterium                           FALSE   FALSE          Low #388ECC
##                                  importance
## Family.Bacteroidetes_[F-1].      -0.4736606
## Lachnospiraceae_[G.2]            -0.4343816
## Gracilibacteria_(GN02)_[G.1]     -0.2704746
## Corynebacterium                   0.2627084
## Saccharibacteria_(TM7)_[G.5]      0.2452102
## Lachnospiraceae_[G.7]             0.2192788
## Butyrivibrio                     -0.2088055
## Abiotrophia                      -0.1665749
## Actinomyces                       0.1551408
## Bacteroidaceae_[G.1]              0.1546426
## Lautropia                        -0.1536717
## Anaeroglobus                      0.1499081
## Peptostreptococcaceae_[XI][G.1] -0.1473172
## Leptotrichia                     -0.1464134
## Solobacterium                    -0.1459576
## Aggregatibacter                  -0.1413844
## Cryptobacterium                   0.1333253
## Schaalia                          0.1280155
## Family.Lachnospiraceae_[XIV].    -0.1074409
## Fusobacterium                    -0.1066005
```

```
write.table(comp1, file="Risk_comp1.txt", sep="\t", dec=",", row.names=T)
comp2 <- plotLoadings(final.splsda, comp = 2, method = 'mean', contrib = 'max',
                      size.title = 1)
```

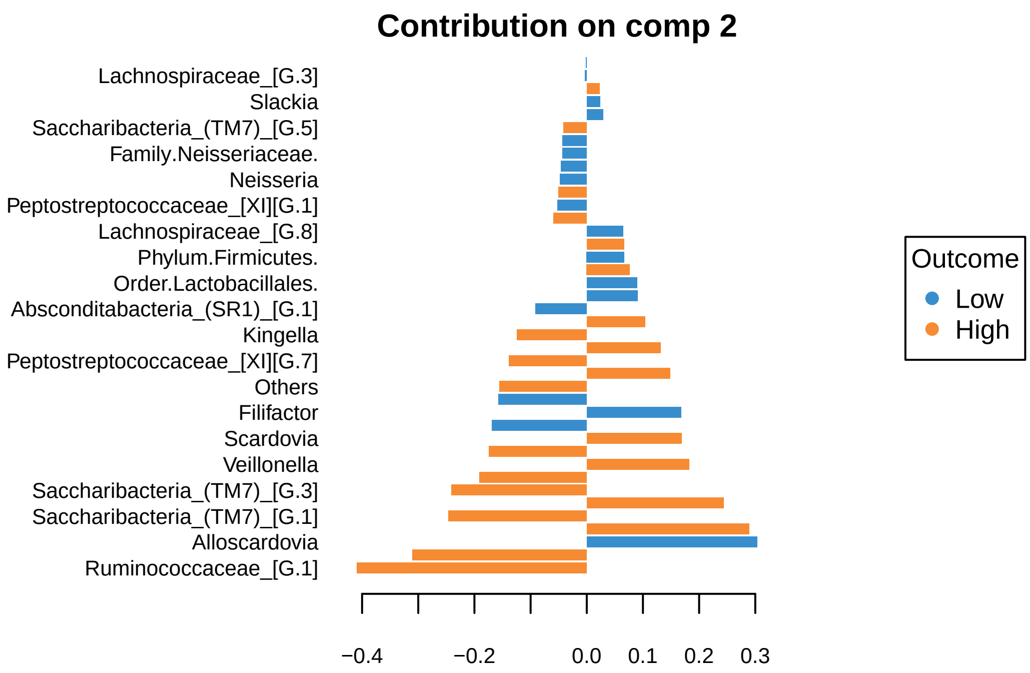


```r
head(comp2, n=20)
```

```
##                                           Low          High Contrib.Low
## Ruminococcaceae_[G.1]           -0.0076649992  0.0107208128       FALSE
## Alloprevotella                  -0.0130031058  0.0181870683       FALSE
## Alloscardovia                    0.0129147349 -0.0180634665        TRUE
## Lactobacillus                   -0.0041522923  0.0058076912       FALSE
## Saccharibacteria_(TM7)_[G.1]    -0.0245041296  0.0342732179       FALSE
## Bifidobacterium                 -0.0078018610  0.0109122375       FALSE
## Saccharibacteria_(TM7)_[G.3]    -0.0302767499  0.0423472150       FALSE
## Peptococcus                     -0.0006787447  0.0009493407       FALSE
## Veillonella                     -0.0219053433  0.0306383705       FALSE
## Ruminococcaceae_[G.2]           -0.0067336633  0.0094181802       FALSE
## Scardovia                       -0.0264955311  0.0370585335       FALSE
## Peptostreptococcus               0.0377151878 -0.0527511431        TRUE
## Filifactor                       0.0380455441 -0.0532132029        TRUE
## Catonella                        0.0298084170 -0.0416921713        TRUE
## Others                          -0.0459898476  0.0643246706       FALSE
## Fretibacterium                  -0.0032968109  0.0046111541       FALSE
## Peptostreptococcaceae_[XI][G.7] -0.0135896998  0.0190075203       FALSE
## Streptococcus                   -0.0406128153  0.0568039709       FALSE
## Kingella                        -0.0275765976  0.0385705900       FALSE
## Actinomyces                     -0.0606734848  0.0848622495       FALSE
##                                 Contrib.High Contrib GroupContrib   color
## Ruminococcaceae_[G.1]                   TRUE   FALSE         High #F68B33
```

```
## Alloprevotella                         TRUE   FALSE         High #F68B33
## Alloscardovia                         FALSE   FALSE          Low #388ECC
## Lactobacillus                          TRUE   FALSE         High #F68B33
## Saccharibacteria_(TM7)_[G.1]           TRUE   FALSE         High #F68B33
## Bifidobacterium                        TRUE   FALSE         High #F68B33
## Saccharibacteria_(TM7)_[G.3]           TRUE   FALSE         High #F68B33
## Peptococcus                            TRUE   FALSE         High #F68B33
## Veillonella                            TRUE   FALSE         High #F68B33
## Ruminococcaceae_[G.2]                  TRUE   FALSE         High #F68B33
## Scardovia                              TRUE   FALSE         High #F68B33
## Peptostreptococcus                    FALSE   FALSE          Low #388ECC
## Filifactor                            FALSE   FALSE          Low #388ECC
## Catonella                             FALSE   FALSE          Low #388ECC
## Others                                 TRUE   FALSE         High #F68B33
## Fretibacterium                         TRUE   FALSE         High #F68B33
## Peptostreptococcaceae_[XI][G.7]        TRUE   FALSE         High #F68B33
## Streptococcus                          TRUE   FALSE         High #F68B33
## Kingella                               TRUE   FALSE         High #F68B33
## Actinomyces                            TRUE   FALSE         High #F68B33
##                                  importance
## Ruminococcaceae_[G.1]            -0.4091144
## Alloprevotella                   -0.3106868
## Alloscardovia                     0.3033758
## Lactobacillus                     0.2888373
## Saccharibacteria_(TM7)_[G.1]     -0.2462087
## Bifidobacterium                   0.2436031
## Saccharibacteria_(TM7)_[G.3]     -0.2411665
## Peptococcus                      -0.1910014
## Veillonella                       0.1821756
## Ruminococcaceae_[G.2]            -0.1741172
## Scardovia                         0.1691825
## Peptostreptococcus               -0.1683938
## Filifactor                        0.1680894
## Catonella                        -0.1569383
## Others                           -0.1553134
## Fretibacterium                    0.1479710
## Peptostreptococcaceae_[XI][G.7] -0.1382492
## Streptococcus                     0.1316543
## Kingella                         -0.1243481
## Actinomyces                       0.1036173
```

```
write.table(comp2, file="Risk_comp2.txt", sep="\t", dec=",", row.names=T)

##  Sample plots from sPLS-DA including 95% confidence ellipses. Samples are projected into the space s
plotIndiv(final.splsda, comp = c(1,2), # plot samples from final model
          group = Y, ind.names = FALSE, # colour by class label
          ellipse = TRUE, legend = TRUE, # include 95% confidence ellipse
          title = ' (a) sPLS-DA on addp, comp 1 & 2')
```

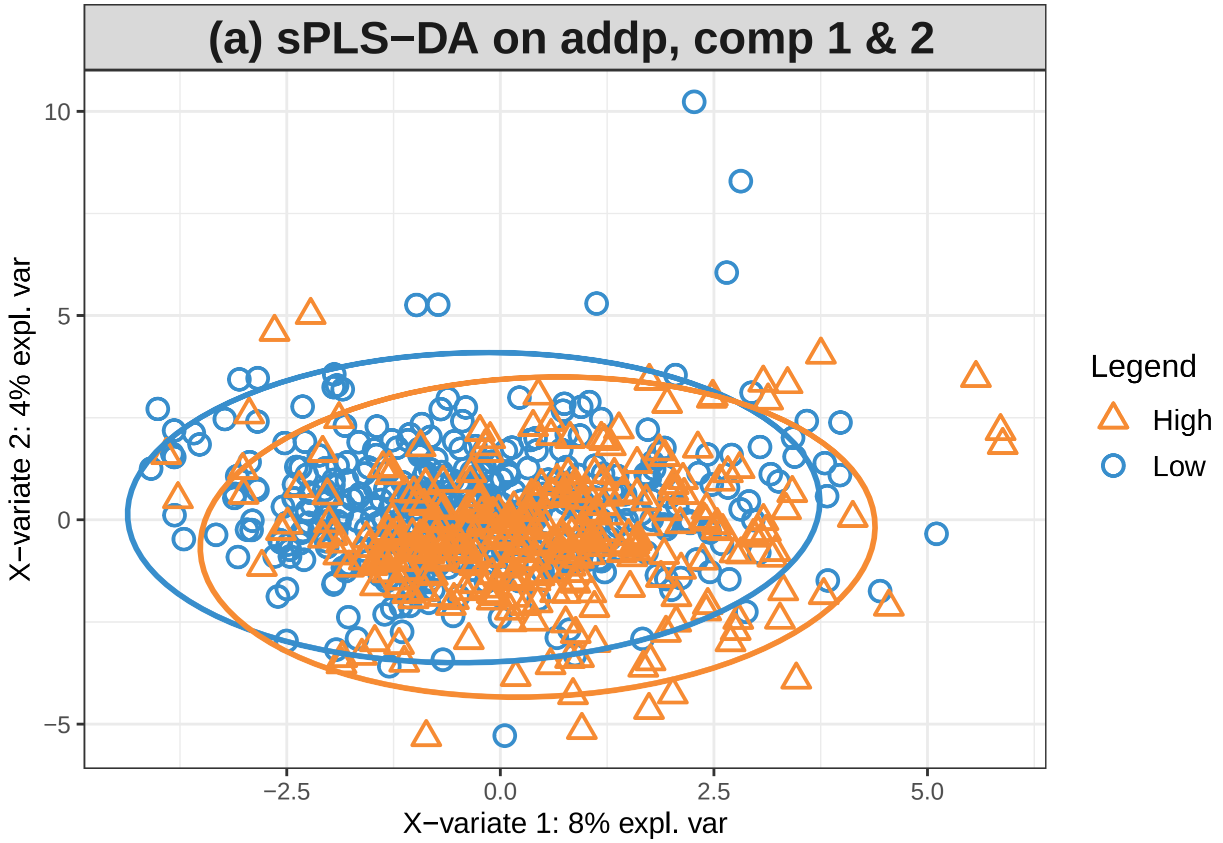


```
# plotIndiv(final.splsda, comp = c(1,3), # plot samples from final model
#           group = Y, ind.names = FALSE,  # colour by class label
#           ellipse = TRUE, legend = TRUE, # include 95% confidence ellipse
#           title = '(b) sPLS-DA on addp, comp 1 & 3')

##Stability of variable selection from the sPLS-DA. The barplot represents the frequency of selection a
# form new perf() object which utilises the final model
perf.splsda.addp <- perf(final.splsda,
                         folds = 5, nrepeat = 10, # use repeated cross-validation
                         validation = "Mfold", dist = "max.dist",  # use max.dist measure
                         progressBar = FALSE)

# plot the stability of each feature for the first three components, 'h' type refers to histogram
par(mfrow=c(1,2))
plot(perf.splsda.addp$features$stable[[1]], type = 'h',
     ylab = 'Stability',
     xlab = 'Features',
     main = '(a) Comp 1', las =2)
plot(perf.splsda.addp$features$stable[[2]], type = 'h',
     ylab = 'Stability',
     xlab = 'Features',
     main = '(b) Comp 2', las =2)
```

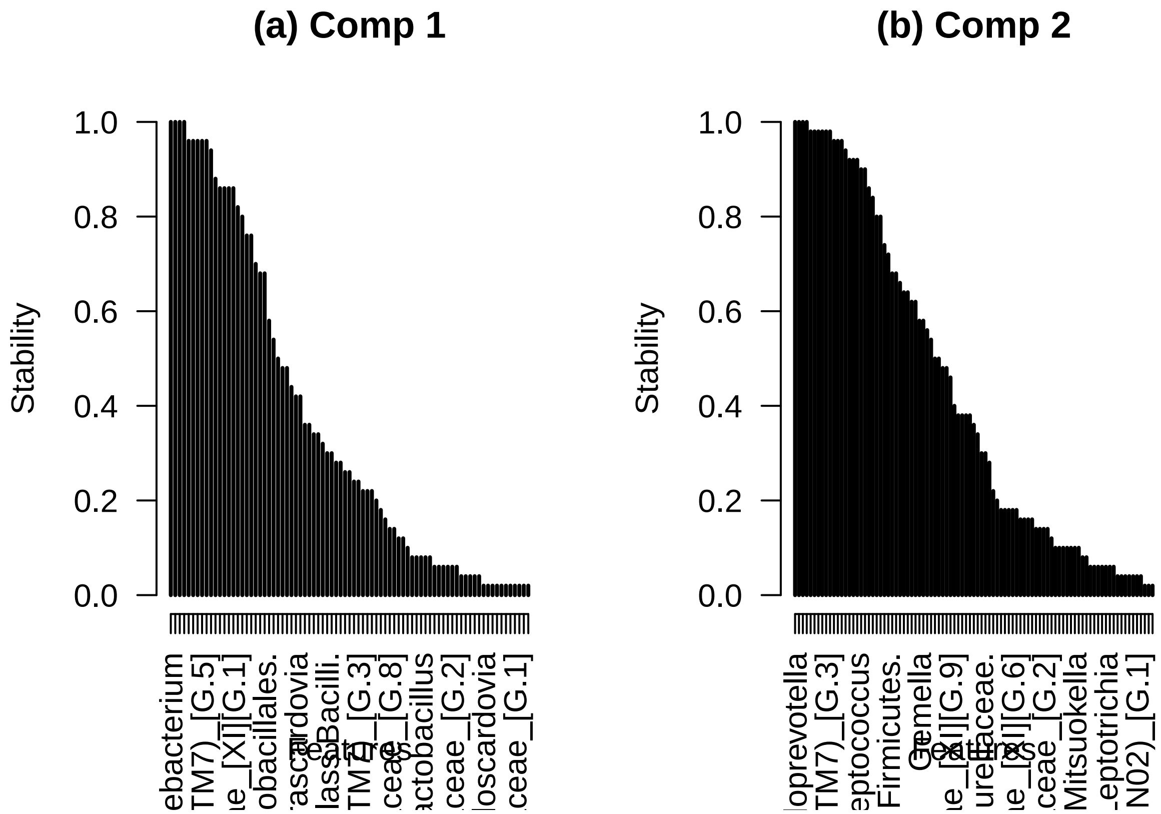


```r
# plot(perf.splsda.addp$features$stable[[3]], type = 'h',
#      ylab = 'Stability',
#      xlab = 'Features',
#      main = '(c) Comp 3', las =2)
par(mfrow=c(1,1))


train <- sample(1:nrow(X), 0.75*nrow(X)) # randomly select 75% of samples in training
test <- setdiff(1:nrow(X), train) # rest is part of the test set

# store matrices into training and test set:
X.train <- X[train, ]
X.test <- X[test,]
Y.train <- Y[train]
Y.test <- Y[test]

# train the model
train.splsda.addp <- splsda(X.train, Y.train, ncomp = optimal.ncomp, keepX = optimal.keepX)

# use the model on the Xtest set
predict.splsda.addp <- predict(train.splsda.addp, X.test, dist = "max.dist")

# evaluate the prediction accuracy for the first two components
predict.comp2 <- predict.splsda.addp$class$max.dist[,2]
table(factor(predict.comp2, levels = c("Low", "High")), Y.test)
```

```
##        Y.test
##         Low High
##    Low   79   54
##    High  26   22
```

```
#Correct classification rate
sum(diag(table(factor(predict.comp2, levels = c("Low", "High")), Y.test)))/
  sum(table(factor(predict.comp2, levels = c("High", "Low")), Y.test))
```

```
## [1] 0.558011
```

```
## ROC curve and AUC from sPLS-DA on component 1 (a) and all (two) components (b) averaged across one-v
auc.splsda = auroc(final.splsda, roc.comp = 1, print = FALSE) # AUROC for the first component
```

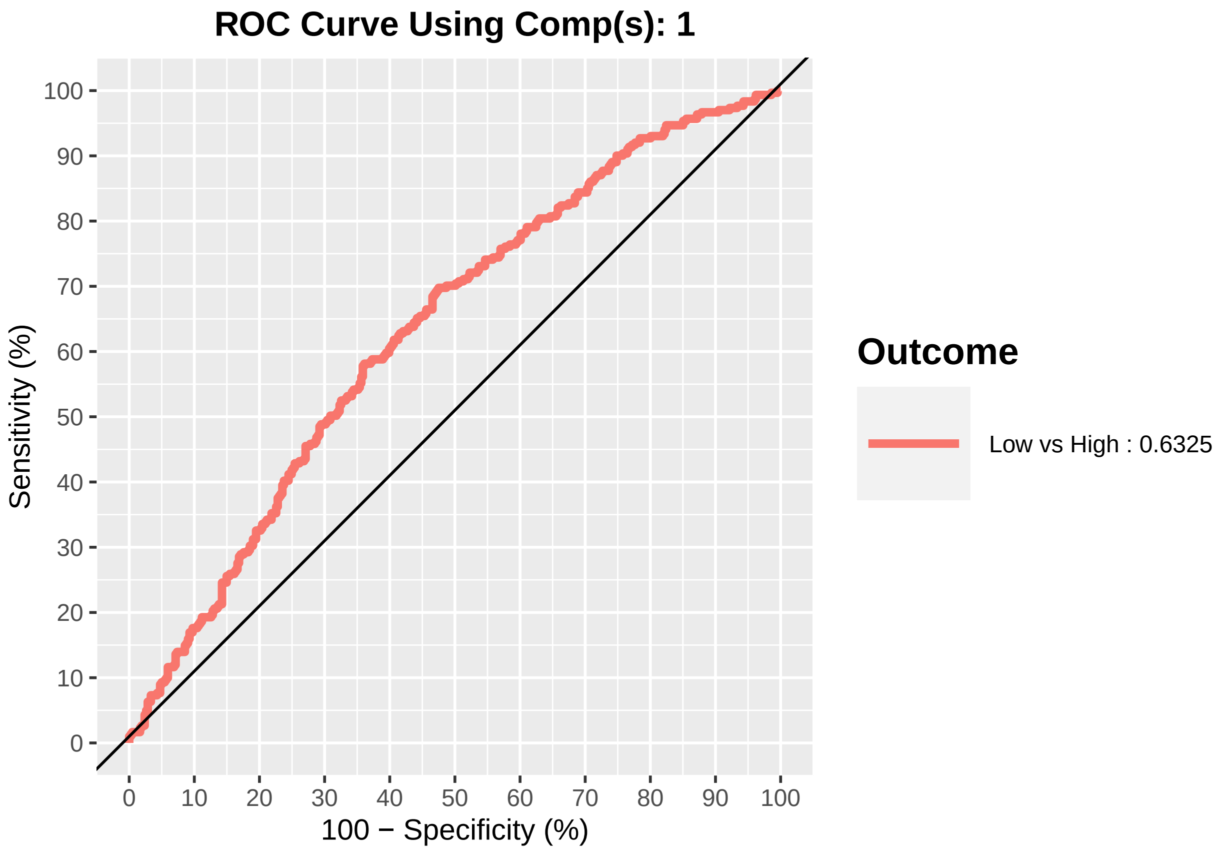


```
auc.splsda = auroc(final.splsda, roc.comp = 2, print = FALSE) # AUROC for the first and second componen
```

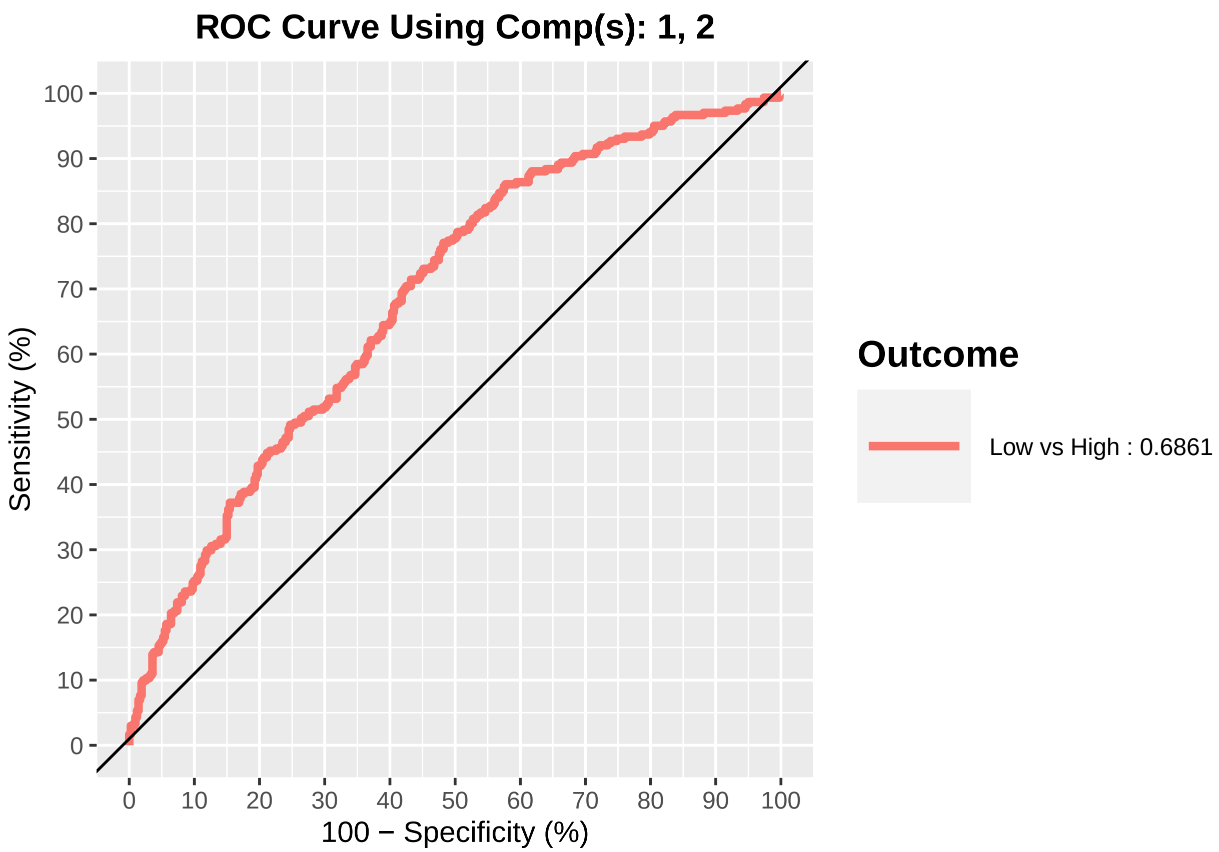


```
ROClist[["RiskROC"]] <-auc.splsda$graph.Comp2

#AUC
print("AUC")
```

```
## [1] "AUC"
```

```
auc.splsda$Comp2
```

```
##                  AUC p-value
## Low vs High 0.6861       0
```

```
rm(list=setdiff(ls(), c("FeaturePic2", "Pic2", "Phe", "Microbio", "FeatureMic", "Metabo", "paretoscale"

set.seed(99) # for reproducibility, remove for normal use

Microbio2<-Microbio
#Subset select or just remove NA
table(Phe$Alcprweek_cat, useNA="always")
```

Alcprweek_cat

```
##
## Abstinence   Moderate       High       <NA>
##         79        302        262        103
```

```
Phe2<-Phe[complete.cases(Phe$Alcprweek_cat), ] #NA
#Phe2<-subset(Phe2, Alcprweek_cat %in% c("High", "Abstinence")) #also removes NA, but above can be easi
X<-dplyr::select(Microbio2, one_of(Phe2$IDX)) #Also in X
##Remove orgs that are not present after subsetting.
X <- X[rowSums(X)>0,]


#Hellinger transformation
X <- data.frame(t(decostand(t(X), method="hellinger")))
#Maks TSS
X<-sweep(X, 2, colSums(X), FUN="/")
#rowSums(X)
sum(colnames(X)!=Phe2$IDX)==0
```

```
## [1] TRUE
```

```
X<-t(X)
Y<-Phe2$Alcprweek_cat

sum(rownames(X)!=Phe2$IDX)==0
```

```
## [1] TRUE
```

```
dim(X) # check the dimensions of the X dataframe
```

```
## [1] 643  97
```

```
summary(Y) # check the distribution of class labels
```

```
## Abstinence   Moderate       High
##         79        302        262
```

```
# Barplot of the variance each principal component explains
pca.addp = pca(X, ncomp = 10, center = TRUE, scale = TRUE) # run pca method on data
plot(pca.addp)  # barplot of the eigenvalues (explained variance per component)
```

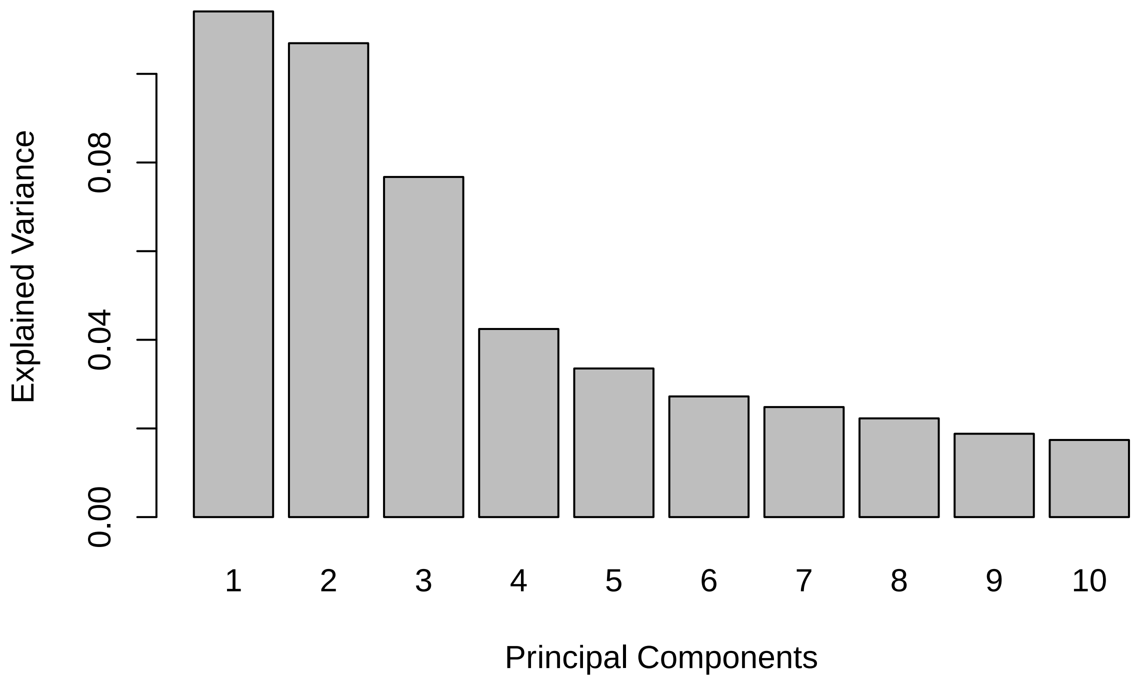


```
# Preliminary (unsupervised) analysis with PCA
plotIndiv(pca.addp, group = Y, ind.names = FALSE, # plot the samples projected
          legend = TRUE, title = 'PCA on addp, comp 1 - 2') # onto the PCA subspace
```

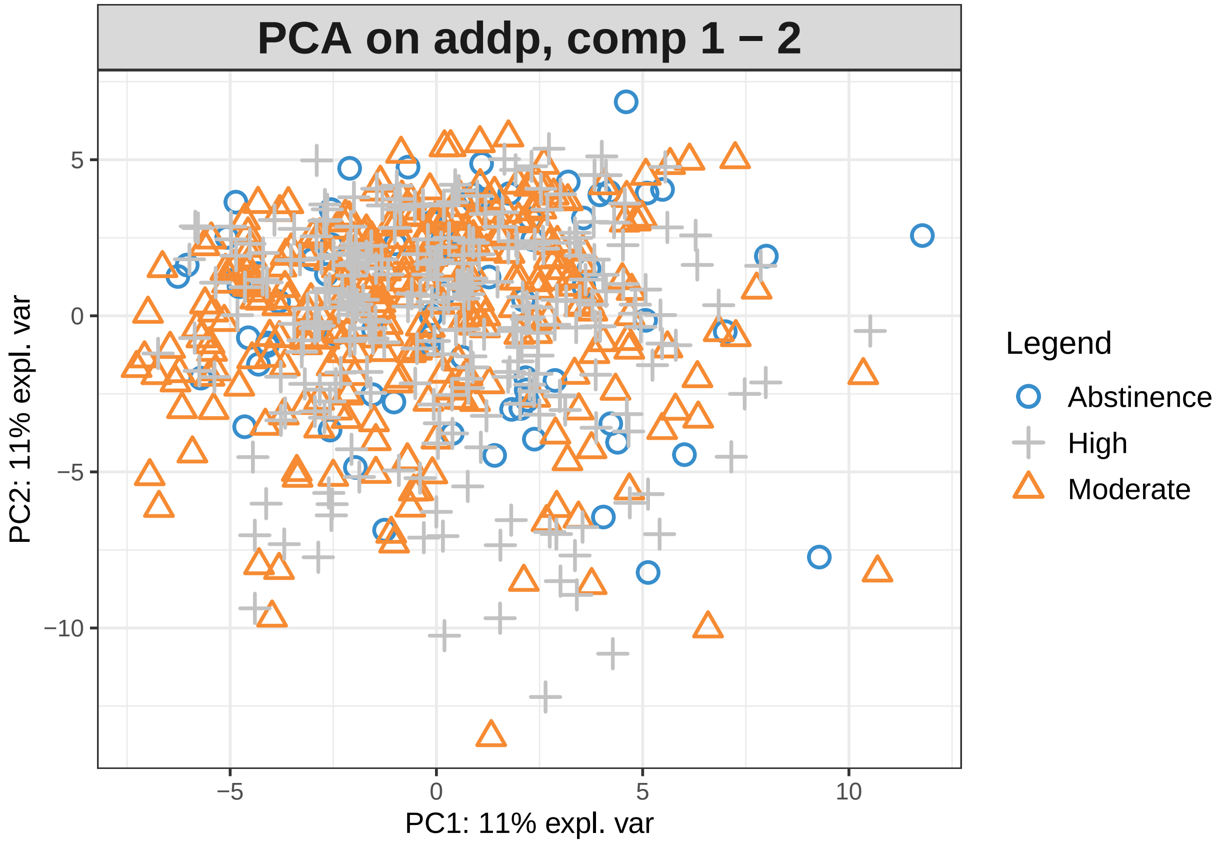


```r
addp.splsda <- splsda(X, Y, ncomp = 10)  # set ncomp to 10 for performance assessment later

## Sample plots after a basic PLS-DA model was operated on this data.
# plot the samples projected onto the first two components of the PLS-DA subspace
plotIndiv(addp.splsda , comp = 1:2,
          group = Y, ind.names = FALSE,  # colour points by class
          ellipse = TRUE, # include 95% confidence ellipse for each class
          legend = TRUE, title = '(a) PLSDA with confidence ellipses')
```

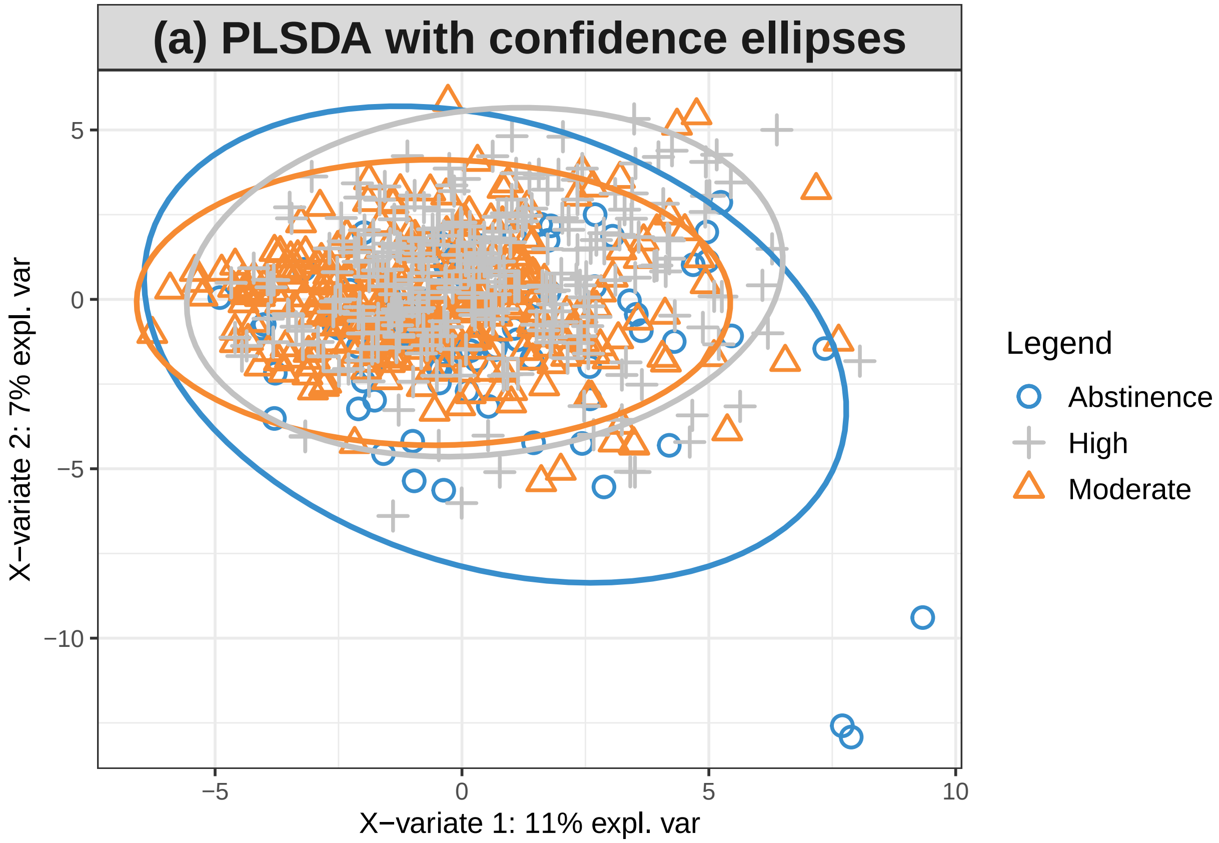


```r
# use the max.dist measure to form decision boundaries between classes based on PLS-DA data
background = background.predict(addp.splsda, comp.predicted=2, dist = "max.dist")

# plot the samples projected onto the first two components of the PLS-DA subspace
plotIndiv(addp.splsda, comp = 1:2,
          group = Y, ind.names = FALSE, # colour points by class
          background = background, # include prediction background for each class
          legend = TRUE, title = " (b) PLSDA with prediction background")
```

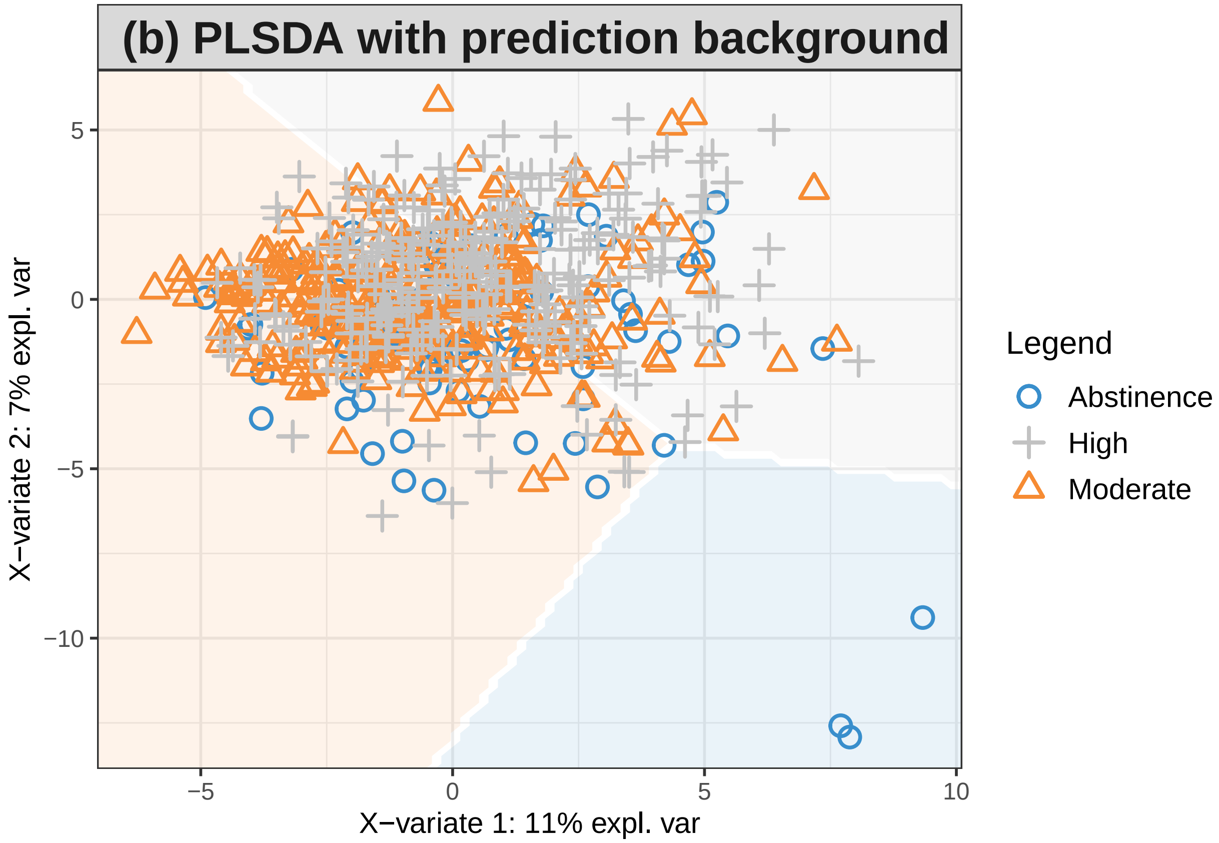


```
## Tuning the number of components in PLS-DA. For each component, repeated cross-validation (10 × 3-fol
# undergo performance evaluation in order to tune the number of components to use
perf.splsda.addp <- perf(addp.splsda, validation = "Mfold",
                          folds = 5, nrepeat = 10, # use repeated cross-validation
                          progressBar = FALSE, auc = TRUE) # include AUC values

# plot the outcome of performance evaluation across all ten components
plot(perf.splsda.addp, col = color.mixo(5:7), sd = TRUE,
     legend.position = "horizontal")
```

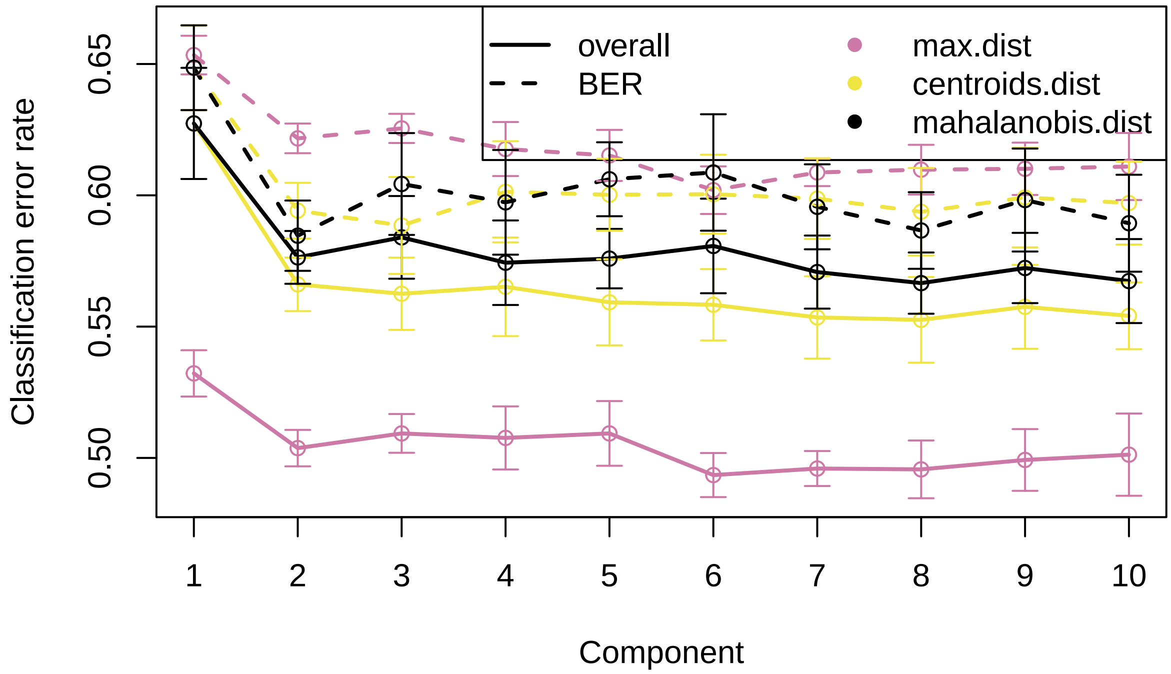


```
perf.splsda.addp$choice.ncomp # what is the optimal value of components according to perf()
```

```
##          max.dist centroids.dist mahalanobis.dist
## overall         6              2                2
## BER             6              2                2
```

```
## Tuning keepX for the sPLS-DA. Each coloured line represents the balanced error rate (y-axis) per comp
# grid of possible keepX values that will be tested for each component
list.keepX <- c(1:10,  seq(20, 180, 10))

# undergo the tuning process to determine the optimal number of variables
tune.splsda.addp <- tune.splsda(X, Y, ncomp = 2, # calculate for first 4 components
                                validation = 'Mfold',
                                folds = 5, nrepeat = 10, # use repeated cross-validation
                                dist = 'max.dist', # use max.dist measure
                                measure = "BER", # use balanced error rate of dist measure
                                test.keepX = list.keepX,
                                cpus = 2 # allow for parallelisation to decrease runtime
                                )

plot(tune.splsda.addp, col = color.jet(2))
```

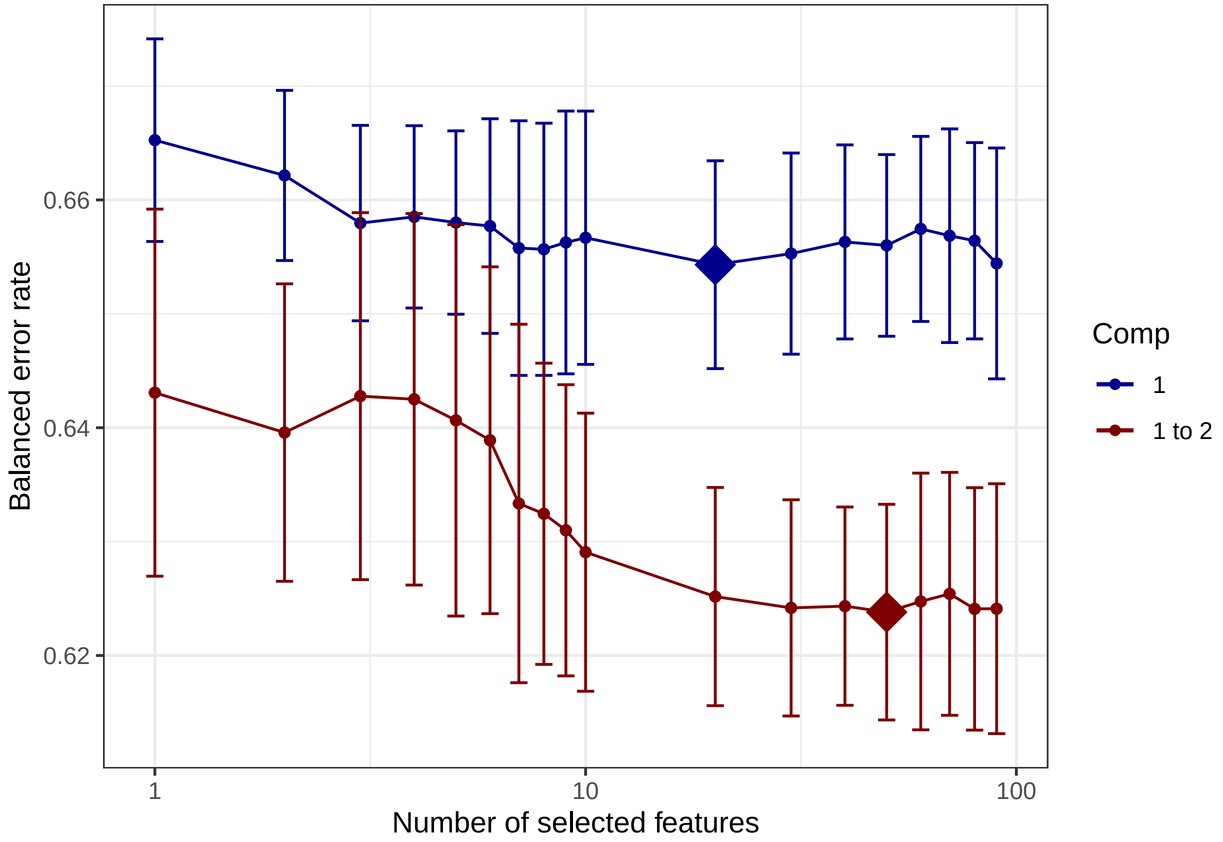


```
tune.splsda.addp$choice.ncomp$ncomp # what is the optimal value of components according to tune.splsda(
```

```
## [1] 2
```

```
tune.splsda.addp$choice.keepX # what are the optimal values of variables according to tune.splsda()
```

```
## comp1 comp2
##    20    50
```

```
optimal.ncomp <- tune.splsda.addp$choice.ncomp$ncomp
optimal.keepX <- tune.splsda.addp$choice.keepX[1:optimal.ncomp]

# form final model with optimised values for component and variable count
final.splsda <- splsda(X, Y,
                       ncomp = optimal.ncomp,
                       keepX = optimal.keepX)

#########Loadings
#plotLoadings(final.splsda, comp=1, contrib = 'max', method = 'mean', size.title = 1)
#plotLoadings(final.splsda, comp=2, contrib = 'max', method = 'mean', size.title = 1)

comp1 <- plotLoadings(final.splsda, comp = 1, method = 'mean', contrib = 'max',
                      size.title = 1)
```

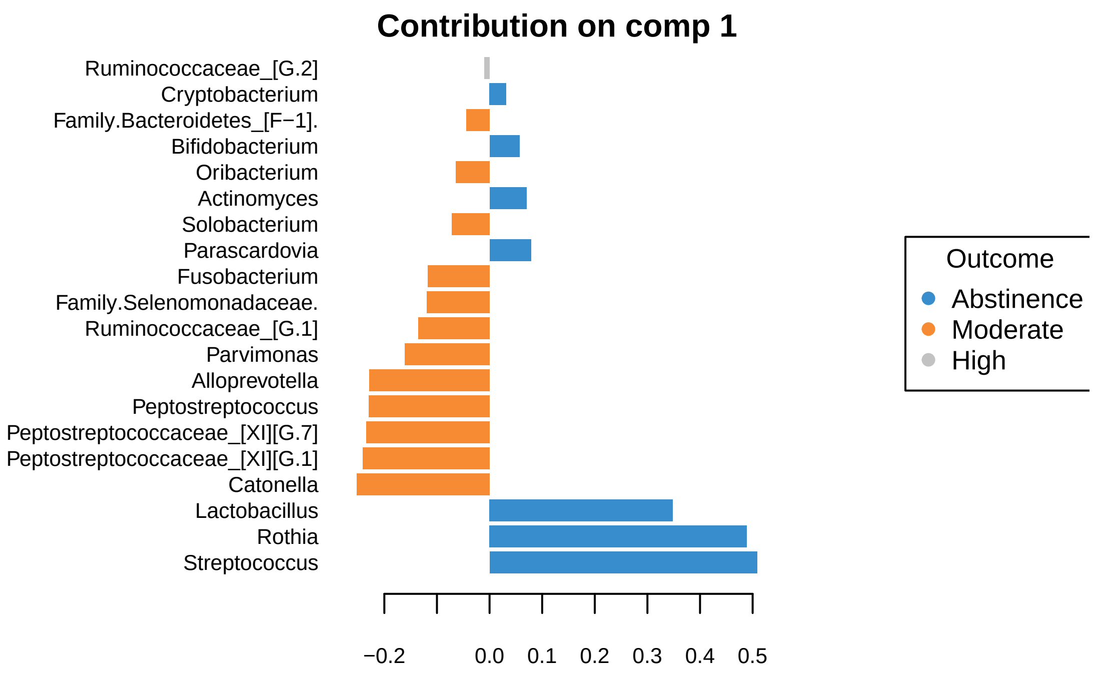


```
head(comp1, n=20)
```

```
##                                 Abstinence    Moderate         High
## Streptococcus                    0.5033871 -0.10769904 -0.027643034
## Rothia                           0.4699139 -0.11544358 -0.008623053
## Lactobacillus                    0.4432289 -0.06767069 -0.055643275
## Catonella                       -0.2829393  0.10408130 -0.034657814
## Peptostreptococcaceae_[XI][G.1] -0.2181555  0.13098683 -0.085205105
## Peptostreptococcaceae_[XI][G.7] -0.2555373  0.10990876 -0.049637397
## Peptostreptococcus              -0.2218589  0.12430614 -0.076387772
## Alloprevotella                  -0.3115241  0.07982677  0.001918784
## Parvimonas                      -0.2170680  0.09700321 -0.046361057
## Ruminococcaceae_[G.1]           -0.2712783  0.05921243  0.013545172
## Family.Selenomonadaceae.        -0.2900883  0.04313063  0.037753905
## Fusobacterium                   -0.2171306  0.07803351 -0.024476342
## Parascardovia                    0.1772480 -0.08059038  0.039449230
## Solobacterium                   -0.2455307  0.04446831  0.022776700
## Actinomyces                      0.1190647 -0.10527618  0.085447685
## Oribacterium                    -0.2335523  0.04700313  0.016243071
## Bifidobacterium                  0.2577992 -0.03180206 -0.041076012
## Family.Bacteroidetes_[F-1].     -0.1325385  0.08736605 -0.060740484
## Cryptobacterium                  0.1063557 -0.09468997  0.077077358
## Ruminococcaceae_[G.2]           -0.3281630 -0.02263166  0.125036805
##                                 Contrib.Abstinence Contrib.Moderate
## Streptococcus                                 TRUE            FALSE
```

```
## Rothia                                       TRUE           FALSE
## Lactobacillus                                TRUE           FALSE
## Catonella                                   FALSE            TRUE
## Peptostreptococcaceae_[XI][G.1]             FALSE            TRUE
## Peptostreptococcaceae_[XI][G.7]             FALSE            TRUE
## Peptostreptococcus                          FALSE            TRUE
## Alloprevotella                              FALSE            TRUE
## Parvimonas                                  FALSE            TRUE
## Ruminococcaceae_[G.1]                       FALSE            TRUE
## Family.Selenomonadaceae.                    FALSE            TRUE
## Fusobacterium                               FALSE            TRUE
## Parascardovia                                TRUE           FALSE
## Solobacterium                               FALSE            TRUE
## Actinomyces                                  TRUE           FALSE
## Oribacterium                                FALSE            TRUE
## Bifidobacterium                              TRUE           FALSE
## Family.Bacteroidetes_[F-1].                 FALSE            TRUE
## Cryptobacterium                              TRUE           FALSE
## Ruminococcaceae_[G.2]                       FALSE           FALSE
##                                 Contrib.High Contrib GroupContrib   color
## Streptococcus                          FALSE   FALSE   Abstinence #388ECC
## Rothia                                 FALSE   FALSE   Abstinence #388ECC
## Lactobacillus                          FALSE   FALSE   Abstinence #388ECC
## Catonella                              FALSE   FALSE     Moderate #F68B33
## Peptostreptococcaceae_[XI][G.1]        FALSE   FALSE     Moderate #F68B33
## Peptostreptococcaceae_[XI][G.7]        FALSE   FALSE     Moderate #F68B33
## Peptostreptococcus                     FALSE   FALSE     Moderate #F68B33
## Alloprevotella                         FALSE   FALSE     Moderate #F68B33
## Parvimonas                             FALSE   FALSE     Moderate #F68B33
## Ruminococcaceae_[G.1]                  FALSE   FALSE     Moderate #F68B33
## Family.Selenomonadaceae.               FALSE   FALSE     Moderate #F68B33
## Fusobacterium                          FALSE   FALSE     Moderate #F68B33
## Parascardovia                          FALSE   FALSE   Abstinence #388ECC
## Solobacterium                          FALSE   FALSE     Moderate #F68B33
## Actinomyces                            FALSE   FALSE   Abstinence #388ECC
## Oribacterium                           FALSE   FALSE     Moderate #F68B33
## Bifidobacterium                        FALSE   FALSE   Abstinence #388ECC
## Family.Bacteroidetes_[F-1].            FALSE   FALSE     Moderate #F68B33
## Cryptobacterium                        FALSE   FALSE   Abstinence #388ECC
## Ruminococcaceae_[G.2]                   TRUE   FALSE         High #C2C2C2
##                                  importance
## Streptococcus                    0.50858372
## Rothia                           0.48878592
## Lactobacillus                    0.34839870
## Catonella                       -0.25203635
## Peptostreptococcaceae_[XI][G.1] -0.24124484
## Peptostreptococcaceae_[XI][G.7] -0.23464587
## Peptostreptococcus              -0.22998358
## Alloprevotella                  -0.22819643
## Parvimonas                      -0.16152646
## Ruminococcaceae_[G.1]           -0.13527153
## Family.Selenomonadaceae.        -0.11930359
## Fusobacterium                   -0.11778150
## Parascardovia                    0.07878545
```

```
## Solobacterium                   -0.07222813
## Actinomyces                      0.07029804
## Oribacterium                    -0.06459709
## Bifidobacterium                  0.05678463
## Family.Bacteroidetes_[F-1].     -0.04409937
## Cryptobacterium                  0.03153799
## Ruminococcaceae_[G.2]           -0.01027490
```

```
write.table(comp1, file="Alcprweek_comp1.txt", sep="\t", dec=",", row.names=T)
comp2 <- plotLoadings(final.splsda, comp = 2, method = 'mean', contrib = 'max',
                      size.title = 1)
```

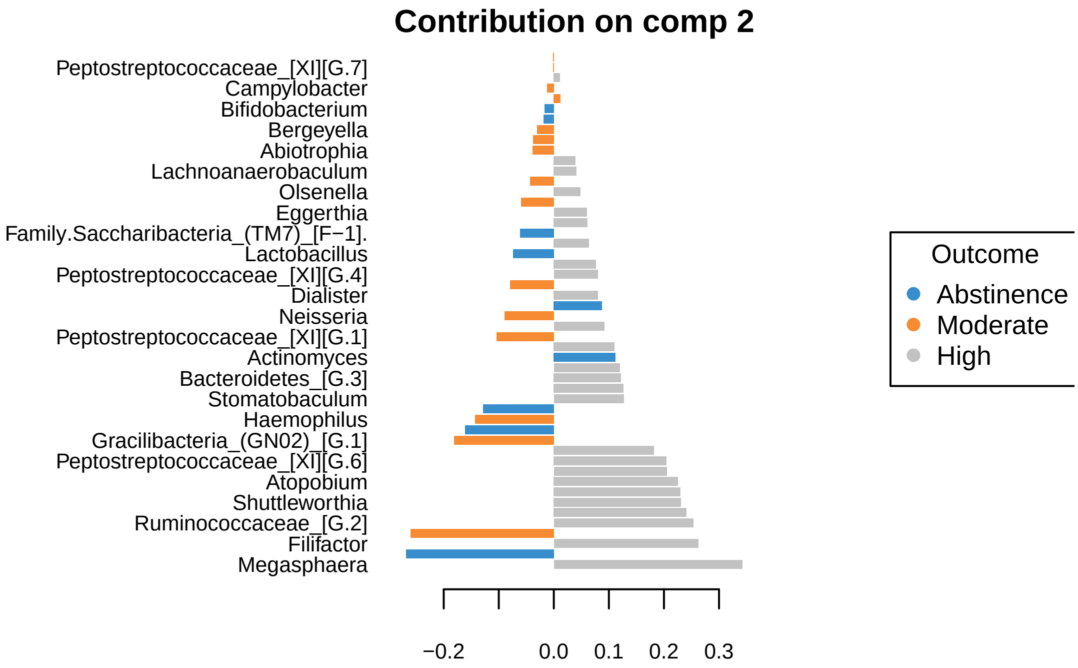


```
head(comp2, n=20)
```

```
##                                  Abstinence    Moderate        High
## Megasphaera                    -0.243770581 -0.07466678  0.15956963
## Saccharibacteria_(TM7)_[G.6]    0.149985876  0.07772196 -0.13481266
## Filifactor                     -0.179368432 -0.06831382  0.13282779
## Lautropia                      -0.004227792  0.11993801 -0.13697437
## Ruminococcaceae_[G.2]          -0.328163043 -0.02263166  0.12503680
## Mitsuokella                    -0.064615491 -0.09437168  0.12826286
## Shuttleworthia                 -0.064588933 -0.09142419  0.12485737
## Fretibacterium                 -0.026582390 -0.10218983  0.12580663
## Atopobium                      -0.054564249 -0.09201501  0.12251568
## Slackia                        -0.125886405 -0.06515177  0.11305672
```

```
## Peptostreptococcaceae_[XI][G.6] -0.156617275 -0.05610788  0.11189826
## Bacteroidales_[G.2]              -0.150507644 -0.05055329  0.10365343
## Gracilibacteria_(GN02)_[G.1]     -0.008059356  0.09580874 -0.10800591
## Lachnospiraceae_[G.3]             0.072638316  0.06543139 -0.09732330
## Haemophilus                       0.009188909  0.07956816 -0.09448667
## Capnocytophaga                    0.069740198  0.05642298 -0.08606571
## Stomatobaculum                   -0.096180252 -0.04926530  0.08578764
## Lachnospiraceae_[G.7]             0.013801104 -0.08040389  0.08851789
## Bacteroidetes_[G.3]               0.028053435 -0.08371309  0.08803486
## Schaalia                         -0.077179649 -0.05182292  0.08300654
##                                  Contrib.Abstinence Contrib.Moderate
## Megasphaera                                   FALSE            FALSE
## Saccharibacteria_(TM7)_[G.6]                   TRUE            FALSE
## Filifactor                                    FALSE            FALSE
## Lautropia                                     FALSE             TRUE
## Ruminococcaceae_[G.2]                         FALSE            FALSE
## Mitsuokella                                   FALSE            FALSE
## Shuttleworthia                                FALSE            FALSE
## Fretibacterium                                FALSE            FALSE
## Atopobium                                     FALSE            FALSE
## Slackia                                       FALSE            FALSE
## Peptostreptococcaceae_[XI][G.6]               FALSE            FALSE
## Bacteroidales_[G.2]                           FALSE            FALSE
## Gracilibacteria_(GN02)_[G.1]                  FALSE             TRUE
## Lachnospiraceae_[G.3]                          TRUE            FALSE
## Haemophilus                                   FALSE             TRUE
## Capnocytophaga                                 TRUE            FALSE
## Stomatobaculum                                FALSE            FALSE
## Lachnospiraceae_[G.7]                         FALSE            FALSE
## Bacteroidetes_[G.3]                           FALSE            FALSE
## Schaalia                                      FALSE            FALSE
##                                  Contrib.High Contrib GroupContrib   color
## Megasphaera                              TRUE   FALSE         High #C2C2C2
## Saccharibacteria_(TM7)_[G.6]            FALSE   FALSE   Abstinence #388ECC
## Filifactor                               TRUE   FALSE         High #C2C2C2
## Lautropia                               FALSE   FALSE     Moderate #F68B33
## Ruminococcaceae_[G.2]                    TRUE   FALSE         High #C2C2C2
## Mitsuokella                              TRUE   FALSE         High #C2C2C2
## Shuttleworthia                           TRUE   FALSE         High #C2C2C2
## Fretibacterium                           TRUE   FALSE         High #C2C2C2
## Atopobium                                TRUE   FALSE         High #C2C2C2
## Slackia                                  TRUE   FALSE         High #C2C2C2
## Peptostreptococcaceae_[XI][G.6]          TRUE   FALSE         High #C2C2C2
## Bacteroidales_[G.2]                      TRUE   FALSE         High #C2C2C2
## Gracilibacteria_(GN02)_[G.1]            FALSE   FALSE     Moderate #F68B33
## Lachnospiraceae_[G.3]                   FALSE   FALSE   Abstinence #388ECC
## Haemophilus                             FALSE   FALSE     Moderate #F68B33
## Capnocytophaga                          FALSE   FALSE   Abstinence #388ECC
## Stomatobaculum                           TRUE   FALSE         High #C2C2C2
## Lachnospiraceae_[G.7]                    TRUE   FALSE         High #C2C2C2
## Bacteroidetes_[G.3]                      TRUE   FALSE         High #C2C2C2
## Schaalia                                 TRUE   FALSE         High #C2C2C2
##                                  importance
## Megasphaera                       0.3422859
```

```
## Saccharibacteria_(TM7)_[G.6]    -0.2679430
## Filifactor                        0.2622728
## Lautropia                        -0.2598834
## Ruminococcaceae_[G.2]             0.2533270
## Mitsuokella                       0.2410384
## Shuttleworthia                    0.2312401
## Fretibacterium                    0.2302201
## Atopobium                         0.2256942
## Slackia                           0.2050179
## Peptostreptococcaceae_[XI][G.6]   0.2049092
## Bacteroidales_[G.2]               0.1819367
## Gracilibacteria_(GN02)_[G.1]    -0.1807358
## Lachnospiraceae_[G.3]            -0.1612221
## Haemophilus                      -0.1427766
## Capnocytophaga                   -0.1285615
## Stomatobaculum                    0.1270020
## Lachnospiraceae_[G.7]             0.1263149
## Bacteroidetes_[G.3]               0.1218346
## Schaalia                          0.1198586
```

```
write.table(comp2, file="Alcprweek_comp2.txt", sep="\t", dec=",", row.names=T)

##  Sample plots from sPLS-DA including 95% confidence ellipses. Samples are projected into the space s
plotIndiv(final.splsda, comp = c(1,2), # plot samples from final model
          group = Y, ind.names = FALSE, # colour by class label
          ellipse = TRUE, legend = TRUE, # include 95% confidence ellipse
          title = ' (a) sPLS-DA on addp, comp 1 & 2')
```

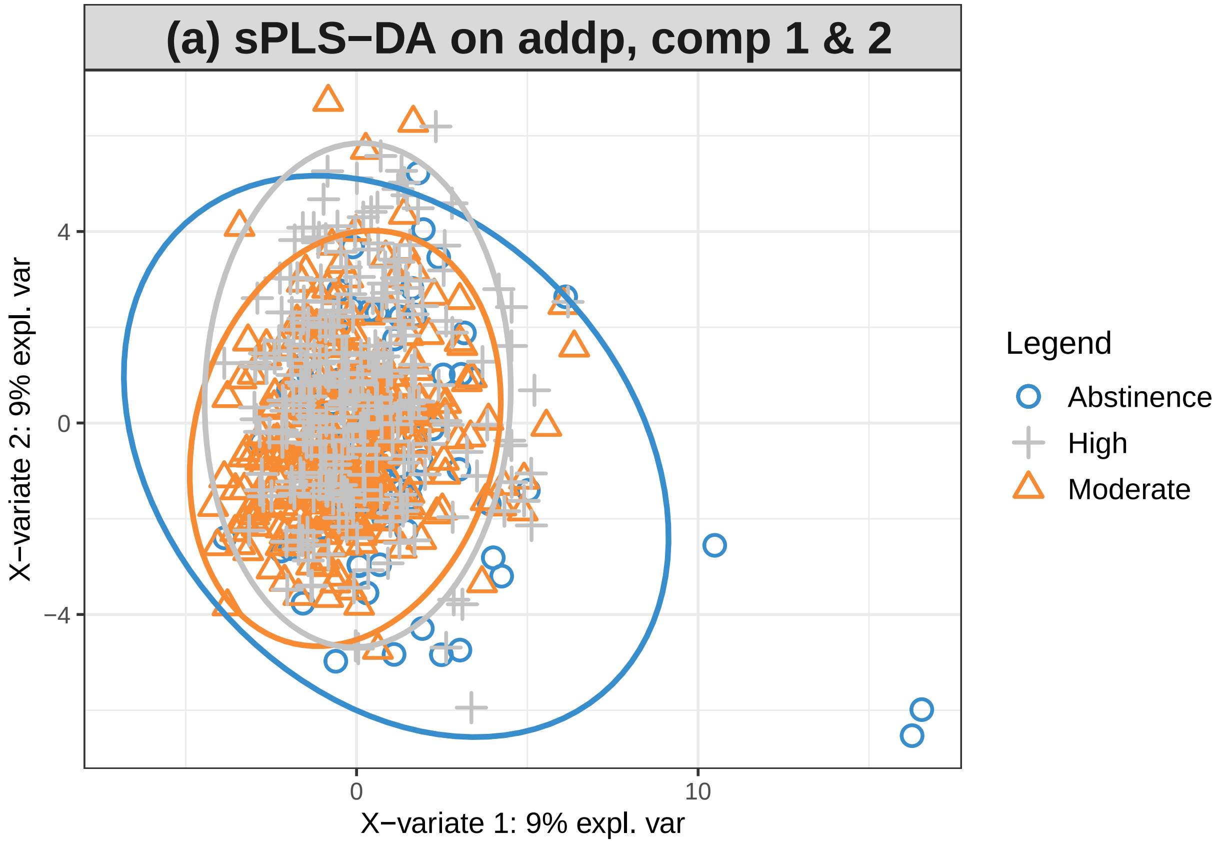


```
# plotIndiv(final.splsda, comp = c(1,3), # plot samples from final model
#           group = Y, ind.names = FALSE,  # colour by class label
#           ellipse = TRUE, legend = TRUE, # include 95% confidence ellipse
#           title = '(b) sPLS-DA on addp, comp 1 & 3')

##Stability of variable selection from the sPLS-DA. The barplot represents the frequency of selection a
# form new perf() object which utilises the final model
perf.splsda.addp <- perf(final.splsda,
                          folds = 5, nrepeat = 10, # use repeated cross-validation
                          validation = "Mfold", dist = "max.dist",  # use max.dist measure
                          progressBar = FALSE)

# plot the stability of each feature for the first three components, 'h' type refers to histogram
par(mfrow=c(1,2))
plot(perf.splsda.addp$features$stable[[1]], type = 'h',
     ylab = 'Stability',
     xlab = 'Features',
     main = '(a) Comp 1', las =2)
plot(perf.splsda.addp$features$stable[[2]], type = 'h',
     ylab = 'Stability',
     xlab = 'Features',
     main = '(b) Comp 2', las =2)
```

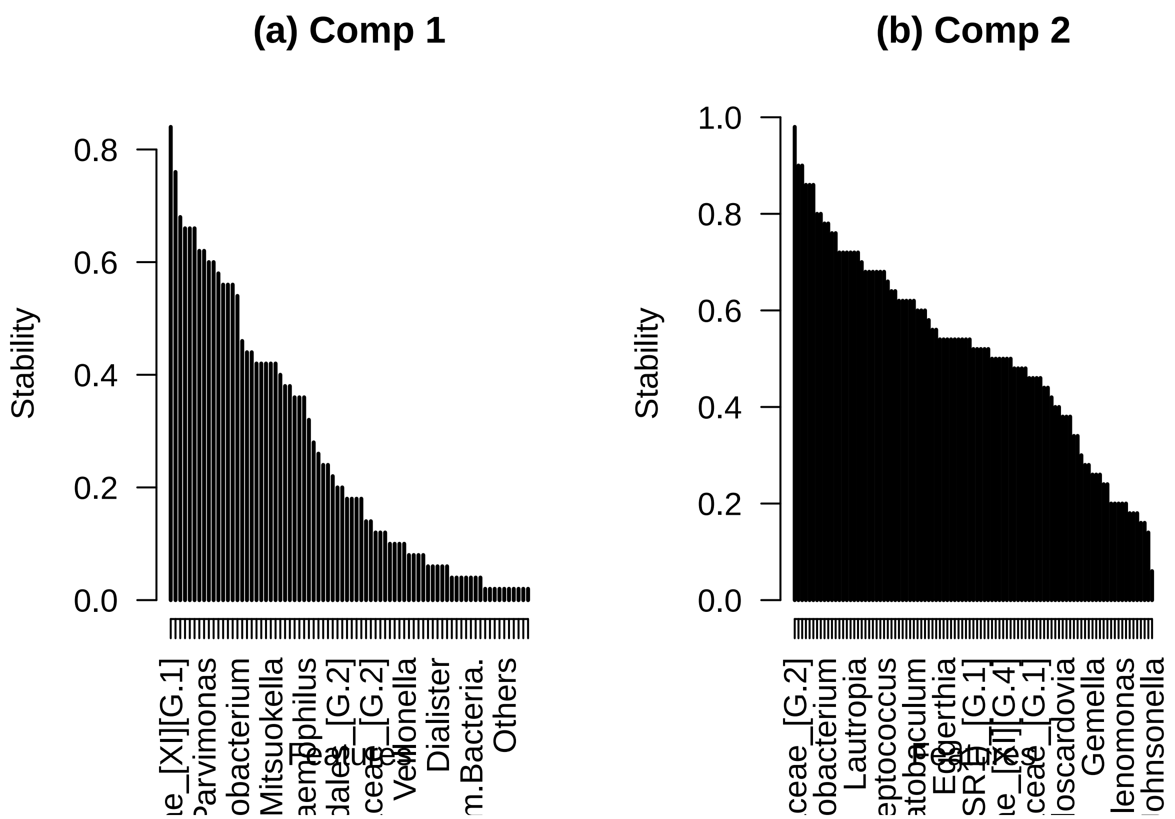


```
# plot(perf.splsda.addp$features$stable[[3]], type = 'h',
#      ylab = 'Stability',
#      xlab = 'Features',
#      main = '(c) Comp 3', las =2)
par(mfrow=c(1,1))


train <- sample(1:nrow(X), 0.75*nrow(X)) # randomly select 75% of samples in training
test <- setdiff(1:nrow(X), train) # rest is part of the test set

# store matrices into training and test set:
X.train <- X[train, ]
X.test <- X[test,]
Y.train <- Y[train]
Y.test <- Y[test]

# train the model
train.splsda.addp <- splsda(X.train, Y.train, ncomp = optimal.ncomp, keepX = optimal.keepX)

# use the model on the Xtest set
predict.splsda.addp <- predict(train.splsda.addp, X.test, dist = "max.dist") #Changed from Mahalanobis

# evaluate the prediction accuracy for the first two components
predict.comp2 <- predict.splsda.addp$class$max.dist[,2]
table(factor(predict.comp2, levels = c("Abstinence", "Moderate", "High")), Y.test)
```

```
##              Y.test
##               Abstinence Moderate High
##   Abstinence           0        0    1
##   Moderate            13       56   40
##   High                 5       19   27
```

```
#Correct classification rate
sum(diag(table(factor(predict.comp2, levels = c("Abstinence", "Moderate", "High")), Y.test)))/
  sum(table(factor(predict.comp2, levels = c("Abstinence", "Moderate", "High")), Y.test))
```

```
## [1] 0.515528
```

```
## ROC curve and AUC from sPLS-DA on component 1 (a) and all (two) components (b) averaged across one-v
auc.splsda = auroc(final.splsda, roc.comp = 1, print = FALSE) # AUROC for the first component
```

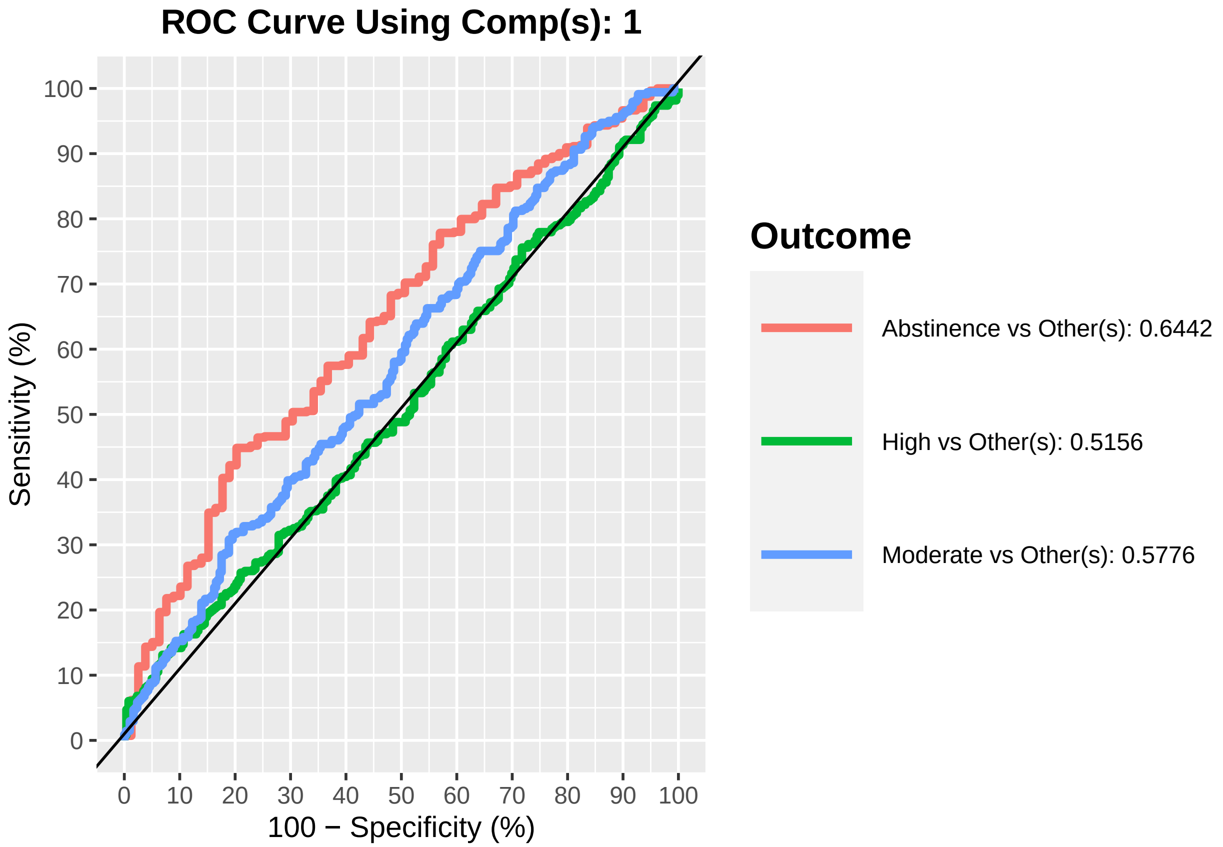


```
auc.splsda = auroc(final.splsda, roc.comp = 2, print = FALSE) # AUROC for the first and second componen
```

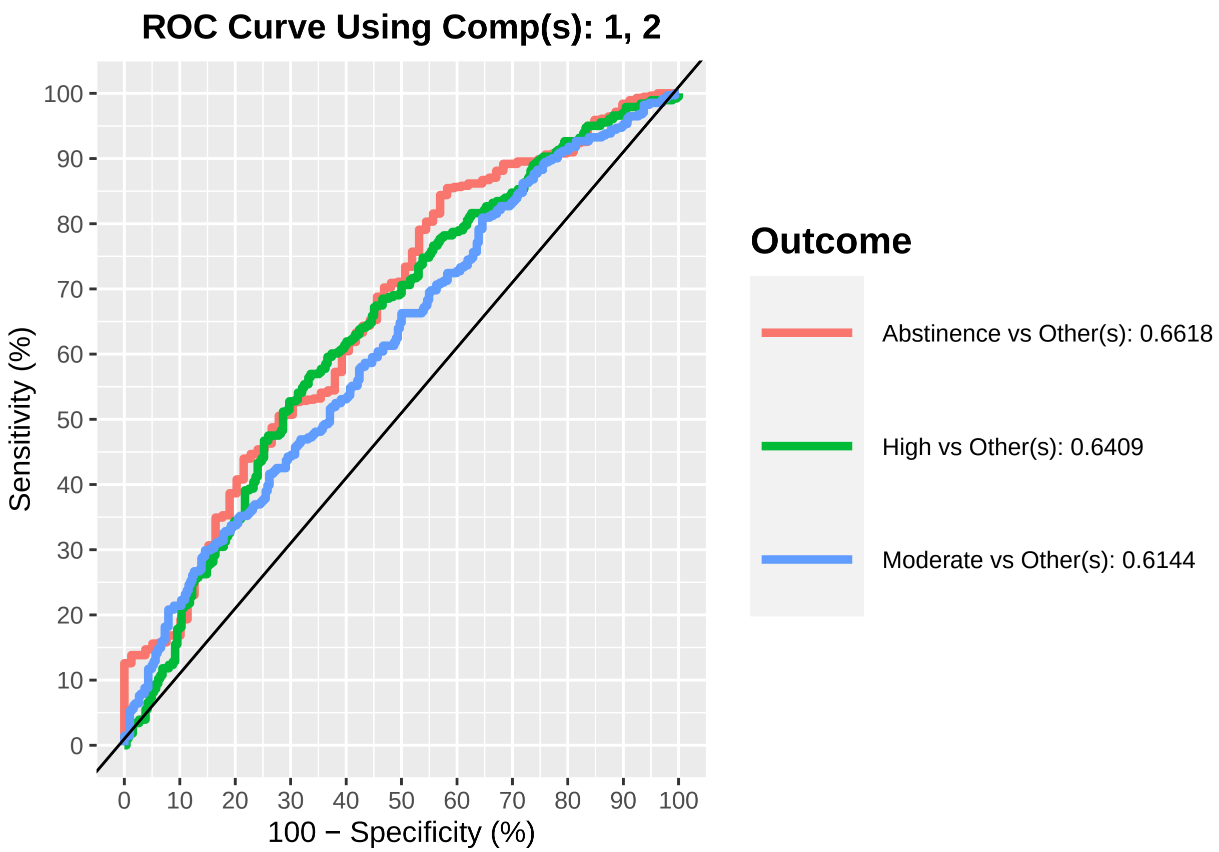


```
ROClist[["alcROC"]] <-auc.splsda$graph.Comp2

#AUC
print("AUC")
```

```
## [1] "AUC"
```

```
auc.splsda$Comp2
```

```
##                              AUC   p-value
## Abstinence vs Other(s) 0.6618 3.152e-06
## Moderate vs Other(s)   0.6144 5.406e-07
## High vs Other(s)       0.6409 1.216e-09
```

```
# rm(list=setdiff(ls(), c("FeaturePic2", "Pic2", "Phe", "Microbio", "FeatureMic", "Metabo", "paretoscal
#
# set.seed(99) # for reproducibility, remove for normal use
#
# Microbio2<-Microbio
# #Subset select or just remove NA
# table(Phe$BMI_cat, useNA="always")
```

```
# Phe2<-Phe[complete.cases(Phe$BMI_cat), ] #NA
# Phe2<-subset(Phe2, BMI_cat %in% c("Healthyweight", "Obese", "Overweight")) #also removes NA, but abov
# Phe2$BMI_cat<-droplevels(Phe2$BMI_cat)
# X<-dplyr::select(Microbio2, one_of(Phe2$IDX)) #Also in X
# ##Remove orgs that are not present after subsetting.
# X <- X[rowSums(X)>0,]
#
#
# #Hellinger transformation
# X <- data.frame(t(decostand(t(X), method="hellinger")))
# #Maks TSS
# X<-sweep(X, 2, colSums(X), FUN="/")
# #rowSums(X)
# sum(colnames(X)!=Phe2$IDX)==0
#
#
# X<-t(X)
# Y<-Phe2$BMI_cat
#
# sum(rownames(X)!=Phe2$IDX)==0
#
# dim(X) # check the dimensions of the X dataframe
# summary(Y) # check the distribution of class labels
#
# # Barplot of the variance each principal component explains
# pca.addp = pca(X, ncomp = 10, center = TRUE, scale = TRUE) # run pca method on data
# plot(pca.addp)  # barplot of the eigenvalues (explained variance per component)
#
#
# # Preliminary (unsupervised) analysis with PCA
# plotIndiv(pca.addp, group = Y, ind.names = FALSE, # plot the samples projected
#           legend = TRUE, title = 'PCA on addp, comp 1 - 2') # onto the PCA subspace
#
# addp.splsda <- splsda(X, Y, ncomp = 10)  # set ncomp to 10 for performance assessment later
#
# ## Sample plots after a basic PLS-DA model was operated on this data.
# # plot the samples projected onto the first two components of the PLS-DA subspace
# plotIndiv(addp.splsda , comp = 1:2,
#           group = Y, ind.names = FALSE,  # colour points by class
#           ellipse = TRUE, # include 95% confidence ellipse for each class
#           legend = TRUE, title = '(a) PLSDA with confidence ellipses')
#
# # use the max.dist measure to form decision boundaries between classes based on PLS-DA data
# background = background.predict(addp.splsda, comp.predicted=2, dist = "max.dist")
#
# # plot the samples projected onto the first two components of the PLS-DA subspace
# plotIndiv(addp.splsda, comp = 1:2,
#           group = Y, ind.names = FALSE, # colour points by class
#           background = background, # include prediction background for each class
#           legend = TRUE, title = " (b) PLSDA with prediction background")
#
# ## Tuning the number of components in PLS-DA. For each component, repeated cross-validation (10 × 3-f
# # undergo performance evaluation in order to tune the number of components to use
```

```
# perf.splsda.addp <- perf(addp.splsda, validation = "Mfold",
#                           folds = 5, nrepeat = 10, # use repeated cross-validation
#                           progressBar = FALSE, auc = TRUE) # include AUC values
#
# # plot the outcome of performance evaluation across all ten components
# plot(perf.splsda.addp, col = color.mixo(5:7), sd = TRUE,
#      legend.position = "horizontal")
#
#
# perf.splsda.addp$choice.ncomp # what is the optimal value of components according to perf()
#
# ## Tuning keepX for the sPLS-DA. Each coloured line represents the balanced error rate (y-axis) per c
# # grid of possible keepX values that will be tested for each component
# list.keepX <- c(1:10,  seq(20, 180, 10))
#
# # undergo the tuning process to determine the optimal number of variables
# tune.splsda.addp <- tune.splsda(X, Y, ncomp = 4, # calculate for first 4 components
#                                  validation = 'Mfold',
#                                  folds = 5, nrepeat = 10, # use repeated cross-validation
#                                  dist = 'max.dist', # use max.dist measure
#                                  measure = "BER", # use balanced error rate of dist measure
#                                  test.keepX = list.keepX,
#                                  cpus = 2 # allow for parallelisation to decrease runtime
#                                  )
#
# plot(tune.splsda.addp, col = color.jet(4))
#
# tune.splsda.addp$choice.ncomp$ncomp # what is the optimal value of components according to tune.splsd
#
# tune.splsda.addp$choice.keepX # what are the optimal values of variables according to tune.splsda()
#
# #optimal.ncomp <- tune.splsda.addp$choice.ncomp$ncomp
# optimal.ncomp <- 4 #See above
# optimal.keepX <- tune.splsda.addp$choice.keepX[1:optimal.ncomp]
#
# # form final model with optimised values for component and variable count
# final.splsda <- splsda(X, Y,
#                        ncomp = optimal.ncomp,
#                        keepX = optimal.keepX)
#
# #########Loadings
# #plotLoadings(final.splsda, comp=1, contrib = 'max', method = 'mean', size.title = 1)
# #plotLoadings(final.splsda, comp=2, contrib = 'max', method = 'mean', size.title = 1)
#
# comp1 <- plotLoadings(final.splsda, comp = 1, method = 'mean', contrib = 'max',
#                       size.title = 1)
# head(comp1, n=20)
# comp2 <- plotLoadings(final.splsda, comp = 2, method = 'mean', contrib = 'max',
#                       size.title = 1)
# head(comp2, n=20)
# comp3 <- plotLoadings(final.splsda, comp = 3, method = 'mean', contrib = 'max',
#                       size.title = 1)
# head(comp3, n=20)
```

```
# comp4 <- plotLoadings(final.splsda, comp = 4, method = 'mean', contrib = 'max',
#                       size.title = 1)
# head(comp4, n=20)
#
# ##  Sample plots from sPLS-DA including 95% confidence ellipses. Samples are projected into the space
# plotIndiv(final.splsda, comp = c(1,2), # plot samples from final model
#           group = Y, ind.names = FALSE, # colour by class label
#           ellipse = TRUE, legend = TRUE, # include 95% confidence ellipse
#           title = ' (a) sPLS-DA on addp, comp 1 & 2')
#
# # plotIndiv(final.splsda, comp = c(1,3), # plot samples from final model
# #           group = Y, ind.names = FALSE,  # colour by class label
# #           ellipse = TRUE, legend = TRUE, # include 95% confidence ellipse
# #           title = '(b) sPLS-DA on addp, comp 1 & 3')
#
# ##Stability of variable selection from the sPLS-DA. The barplot represents the frequency of selection
# # form new perf() object which utilises the final model
# perf.splsda.addp <- perf(final.splsda,
#                          folds = 5, nrepeat = 10, # use repeated cross-validation
#                          validation = "Mfold", dist = "max.dist",  # use max.dist measure
#                          progressBar = FALSE)
#
# # plot the stability of each feature for the first three components, 'h' type refers to histogram
# par(mfrow=c(1,2))
# plot(perf.splsda.addp$features$stable[[1]], type = 'h',
#      ylab = 'Stability',
#      xlab = 'Features',
#      main = '(a) Comp 1', las =2)
# plot(perf.splsda.addp$features$stable[[2]], type = 'h',
#      ylab = 'Stability',
#      xlab = 'Features',
#      main = '(b) Comp 2', las =2)
# # plot(perf.splsda.addp$features$stable[[3]], type = 'h',
# #      ylab = 'Stability',
# #      xlab = 'Features',
# #      main = '(c) Comp 3', las =2)
# par(mfrow=c(1,1))
#
#
# train <- sample(1:nrow(X), 0.75*nrow(X)) # randomly select 75% of samples in training
# test <- setdiff(1:nrow(X), train) # rest is part of the test set
#
# # store matrices into training and test set:
# X.train <- X[train, ]
# X.test <- X[test,]
# Y.train <- Y[train]
# Y.test <- Y[test]
#
# # train the model
# train.splsda.addp <- splsda(X.train, Y.train, ncomp = optimal.ncomp, keepX = optimal.keepX)
#
# # use the model on the Xtest set
# predict.splsda.addp <- predict(train.splsda.addp, X.test, dist = "max.dist") #Changed from Mahalanobi
```

```
#
# # evaluate the prediction accuracy for the first two components
# predict.comp2 <- predict.splsda.addp$class$max.dist[,2]
# table(factor(predict.comp2, levels = c("Healthyweight", "Overweight", "Obese")), Y.test)
# #Correct classification rate
# sum(diag(table(factor(predict.comp2, levels = c("Healthyweight", "Overweight", "Obese")), Y.test)))/
#   sum(table(factor(predict.comp2, levels = c("Healthyweight", "Overweight", "Obese")), Y.test))
#
#
# ## ROC curve and AUC from sPLS-DA on component 1 (a) and all (two) components (b) averaged across one
# auc.splsda = auroc(final.splsda, roc.comp = 1, print = FALSE) # AUROC for the first component
#
# auc.splsda = auroc(final.splsda, roc.comp = 4, print = FALSE) # AUROC for the first and fourth compon
#
# #AUC
# print("AUC")
# auc.splsda$Comp4
```

BMI_cat

```
rm(list=setdiff(ls(), c("FeaturePic2", "Pic2", "Phe", "Microbio", "FeatureMic", "Metabo", "paretoscale"

set.seed(99) # for reproducibility, remove for normal use

Microbio2<-Microbio
#Subset select or just remove NA
table(Phe$act_cat, useNA="always")
```

act_cat

```
##
##    Low Medium   High   <NA>
##    100    149    453     44
```

```
Phe2<-Phe[complete.cases(Phe$act_cat), ] #NA
#Phe2$act_cat<-droplevels(Phe2$act_cat)
X<-dplyr::select(Microbio2, one_of(Phe2$IDX)) #Also in X
##Remove orgs that are not present after subsetting.
X <- X[rowSums(X)>0,]


#Hellinger transformation
X <- data.frame(t(decostand(t(X), method="hellinger")))
#Maks TSS
X<-sweep(X, 2, colSums(X), FUN="/")
#rowSums(X)
sum(colnames(X)!=Phe2$IDX)==0
```

```
## [1] TRUE
```

```r
X<-t(X)
Y<-Phe2$act_cat

sum(rownames(X)!=Phe2$IDX)==0
```

```
## [1] TRUE
```

```r
dim(X) # check the dimensions of the X dataframe
```

```
## [1] 702  97
```

```r
summary(Y) # check the distribution of class labels
```

```
##    Low Medium   High
##    100    149    453
```

```r
# Barplot of the variance each principal component explains
pca.addp = pca(X, ncomp = 10, center = TRUE, scale = TRUE) # run pca method on data
plot(pca.addp)  # barplot of the eigenvalues (explained variance per component)
```

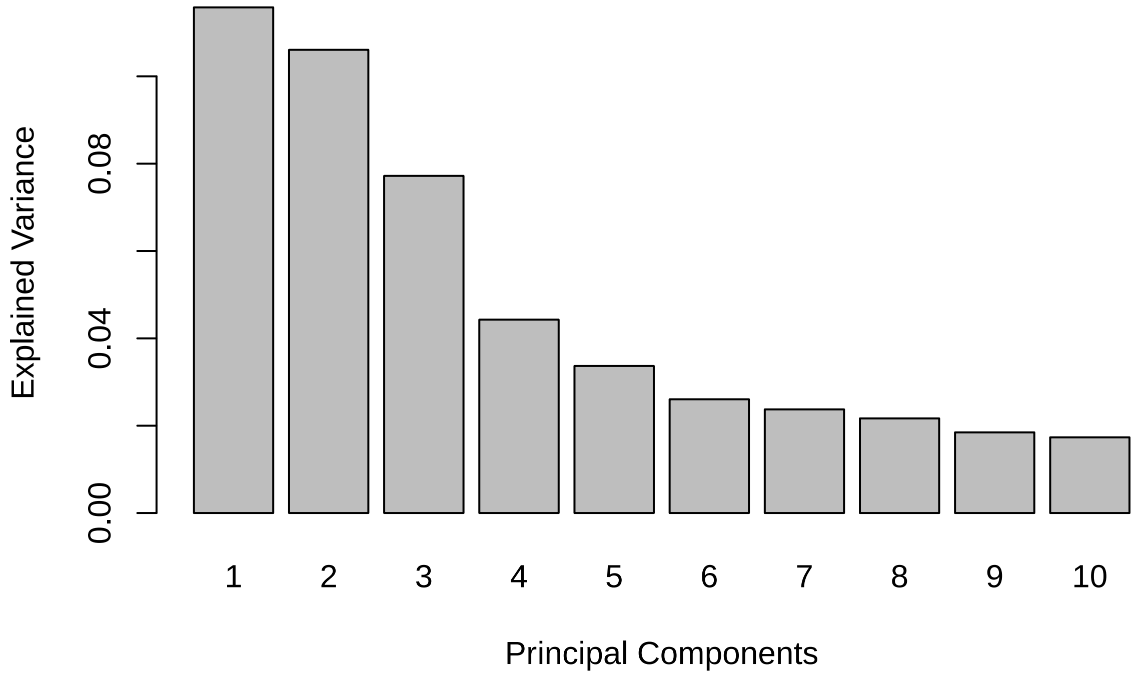


```r
# Preliminary (unsupervised) analysis with PCA
plotIndiv(pca.addp, group = Y, ind.names = FALSE, # plot the samples projected
          legend = TRUE, title = 'PCA on addp, comp 1 - 2') # onto the PCA subspace
```

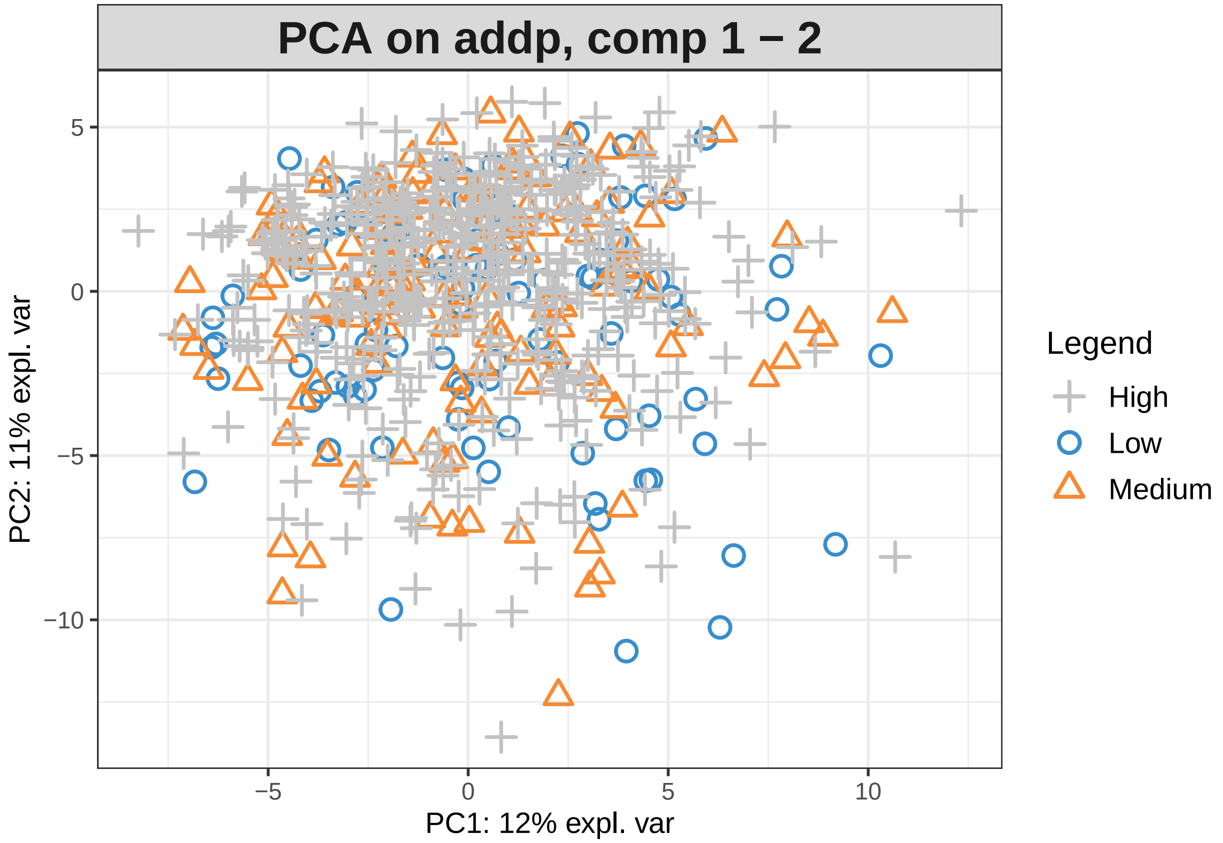


```
addp.splsda <- splsda(X, Y, ncomp = 10)  # set ncomp to 10 for performance assessment later

## Sample plots after a basic PLS-DA model was operated on this data.
# plot the samples projected onto the first two components of the PLS-DA subspace
plotIndiv(addp.splsda , comp = 1:2,
          group = Y, ind.names = FALSE,  # colour points by class
          ellipse = TRUE, # include 95% confidence ellipse for each class
          legend = TRUE, title = '(a) PLSDA with confidence ellipses')
```

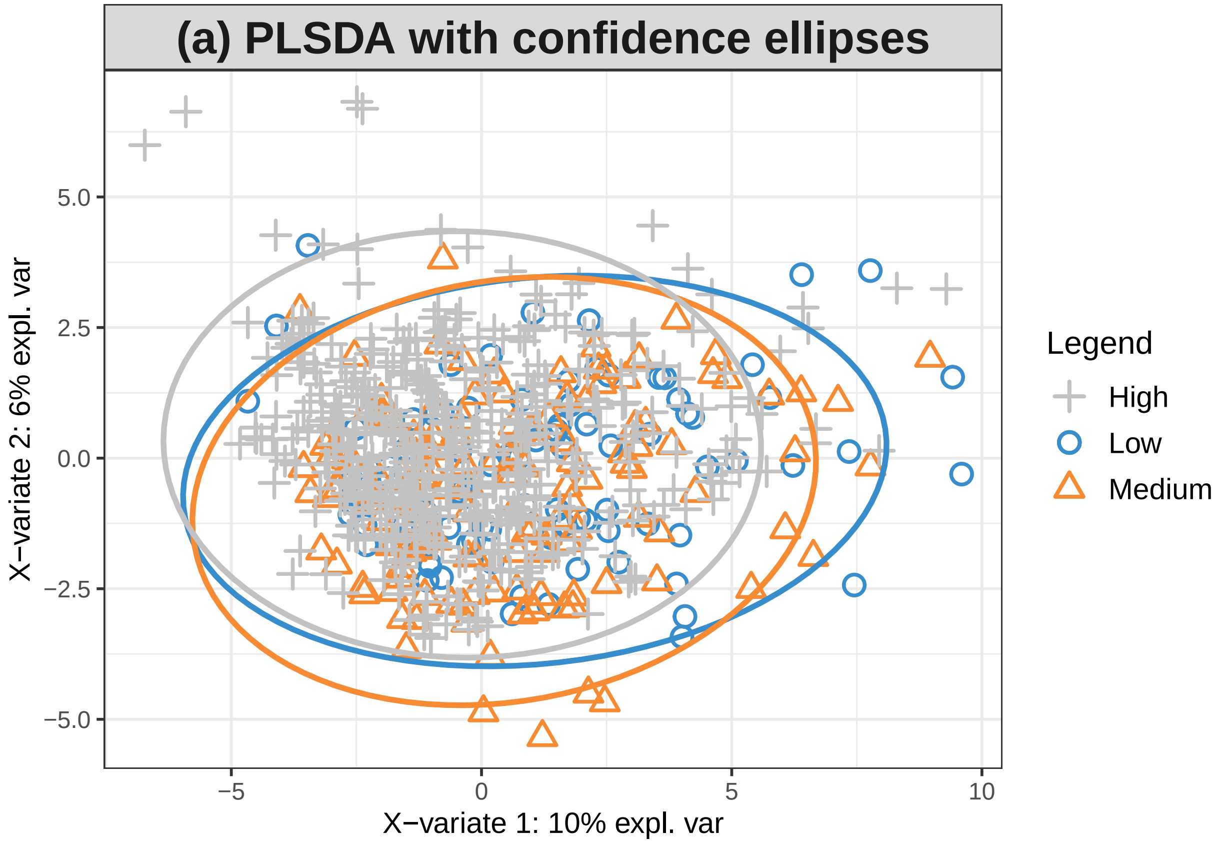


```
# use the max.dist measure to form decision boundaries between classes based on PLS-DA data
background = background.predict(addp.splsda, comp.predicted=2, dist = "max.dist")

# plot the samples projected onto the first two components of the PLS-DA subspace
plotIndiv(addp.splsda, comp = 1:2,
          group = Y, ind.names = FALSE, # colour points by class
          background = background, # include prediction background for each class
          legend = TRUE, title = " (b) PLSDA with prediction background")
```

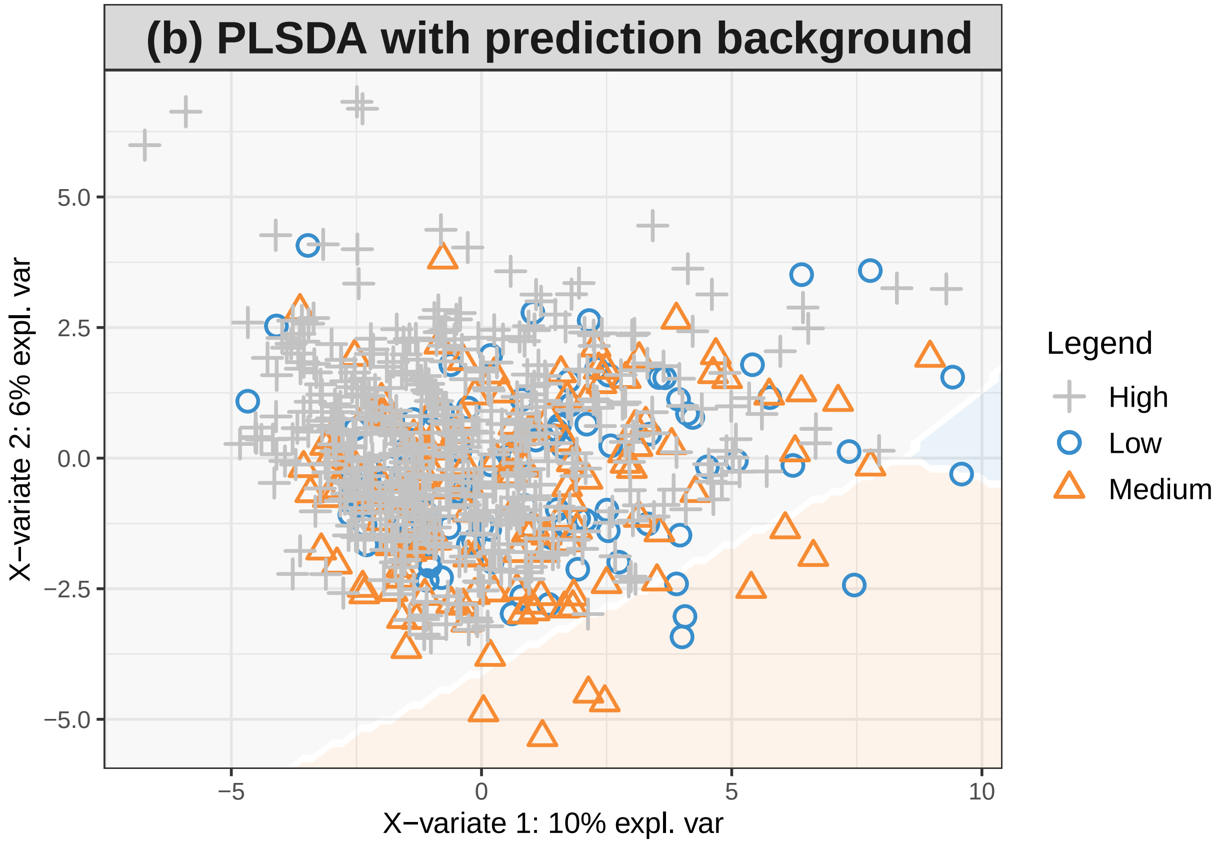


```
## Tuning the number of components in PLS-DA. For each component, repeated cross-validation (10 × 3-fol
# undergo performance evaluation in order to tune the number of components to use
perf.splsda.addp <- perf(addp.splsda, validation = "Mfold",
                          folds = 5, nrepeat = 10, # use repeated cross-validation
                          progressBar = FALSE, auc = TRUE) # include AUC values

# plot the outcome of performance evaluation across all ten components
plot(perf.splsda.addp, col = color.mixo(5:7), sd = TRUE,
     legend.position = "horizontal")
```

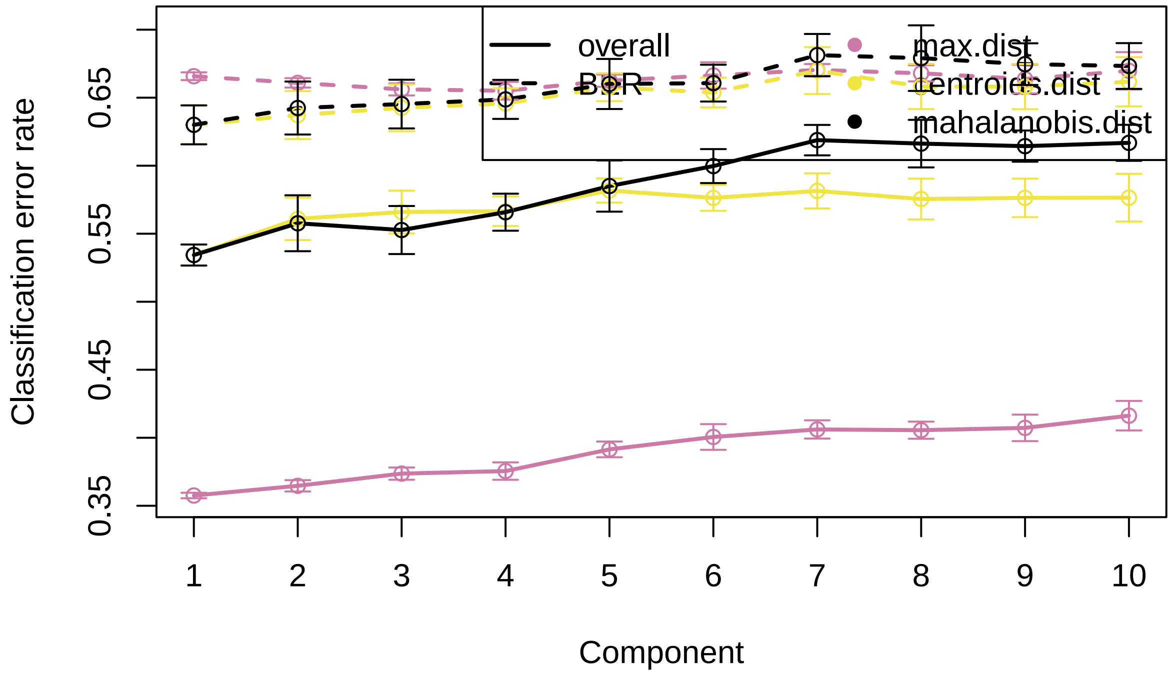


```
perf.splsda.addp$choice.ncomp # what is the optimal value of components according to perf()
```

```
##         max.dist centroids.dist mahalanobis.dist
## overall        3              1                1
## BER            3              1                1
```

```
## Tuning keepX for the sPLS-DA. Each coloured line represents the balanced error rate (y-axis) per comp
# grid of possible keepX values that will be tested for each component
list.keepX <- c(1:10,  seq(20, 180, 10))

# undergo the tuning process to determine the optimal number of variables
tune.splsda.addp <- tune.splsda(X, Y, ncomp = 4, # calculate for first 4 components
                                 validation = 'Mfold',
                                 folds = 5, nrepeat = 10, # use repeated cross-validation
                                 dist = 'max.dist', # use max.dist measure
                                 measure = "overall", # changed to overall because of unbalanced group s
                                 test.keepX = list.keepX,
                                 cpus = 2 # allow for parallelisation to decrease runtime
                                 )

plot(tune.splsda.addp, col = color.jet(4))
```

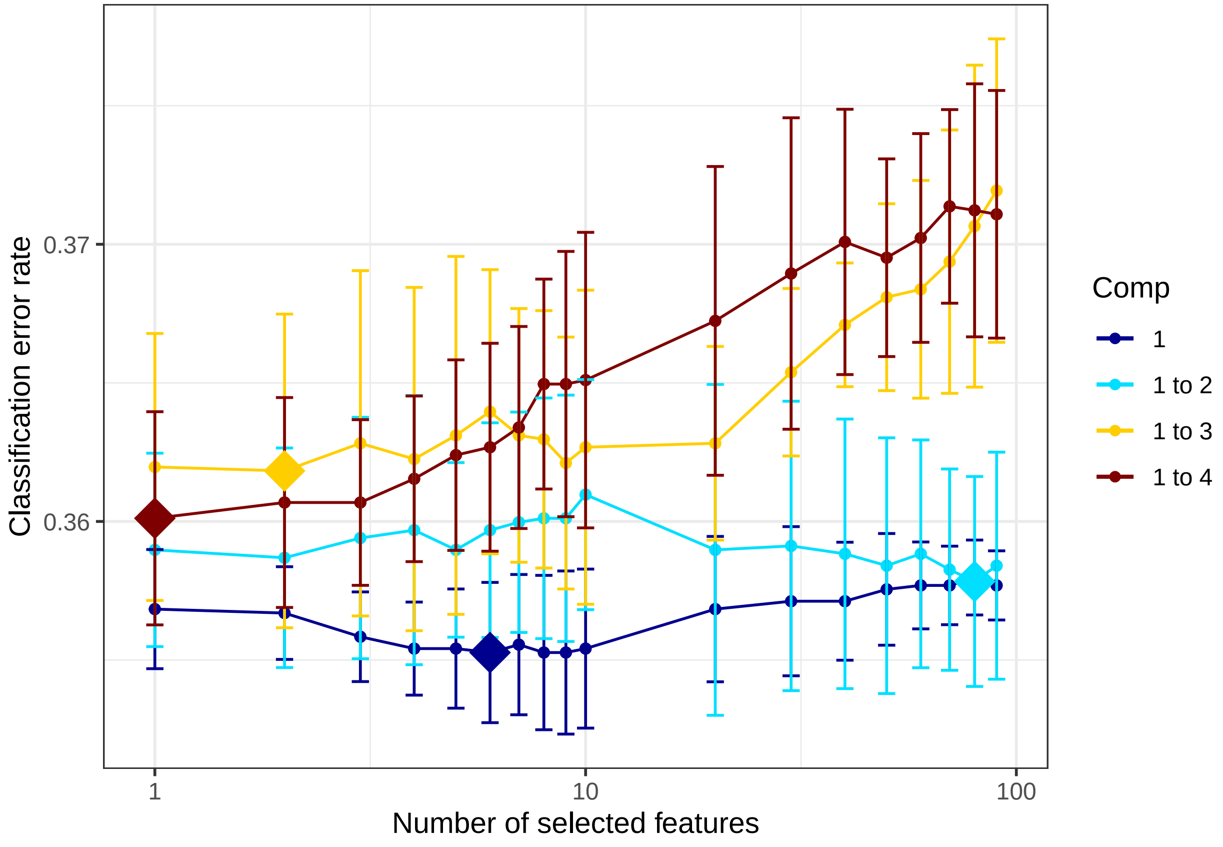


```
tune.splsda.addp$choice.ncomp$ncomp # what is the optimal value of components according to tune.splsda(
```

```
## [1] 1
```

```
tune.splsda.addp$choice.keepX # what are the optimal values of variables according to tune.splsda()
```

```
## comp1 comp2 comp3 comp4
##     6    80     2     1
```

```
optimal.ncomp <- 3 #See above max dist
optimal.keepX <- tune.splsda.addp$choice.keepX[1:optimal.ncomp]

# form final model with optimised values for component and variable count
final.splsda <- splsda(X, Y,
                       ncomp = optimal.ncomp,
                       keepX = optimal.keepX)

#########Loadings
#plotLoadings(final.splsda, comp=1, contrib = 'max', method = 'mean', size.title = 1)
#plotLoadings(final.splsda, comp=2, contrib = 'max', method = 'mean', size.title = 1)

comp1 <- plotLoadings(final.splsda, comp = 1, method = 'mean', contrib = 'max',
                      size.title = 1)
```

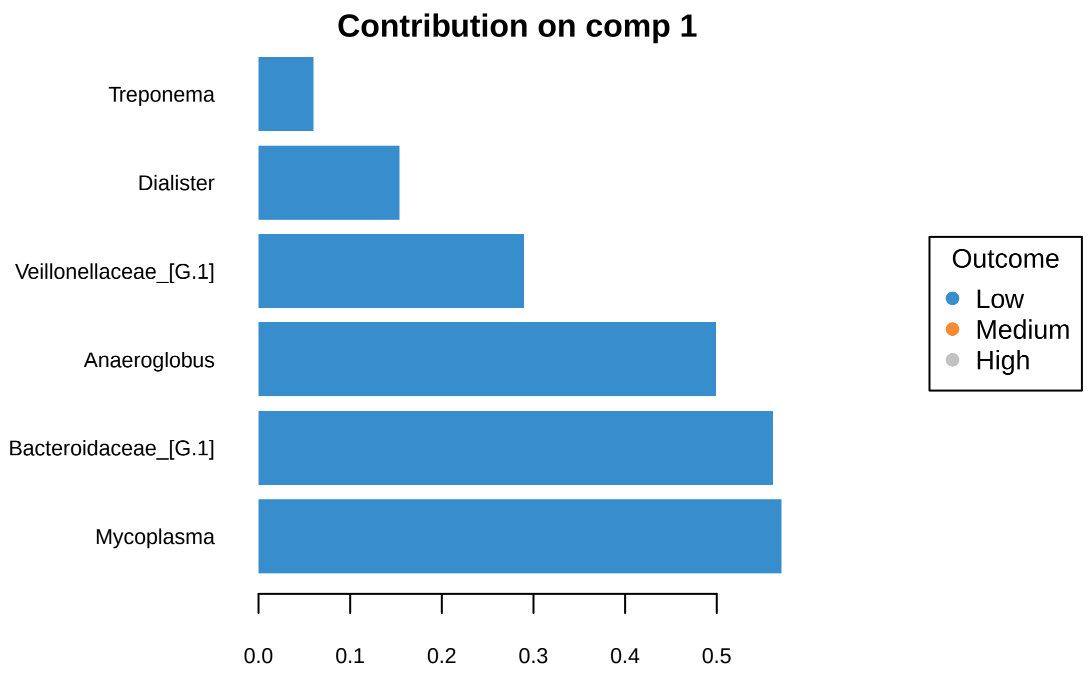


```
head(comp1, n=20)
```

```
##                              Low     Medium        High Contrib.Low
## Mycoplasma            0.3109698 0.05500425 -0.08673865        TRUE
## Bacteroidaceae_[G.1]  0.3181296 0.04405242 -0.08471694        TRUE
## Anaeroglobus          0.3126380 0.03902112 -0.08184978        TRUE
## Veillonellaceae_[G.1] 0.2572891 0.06882707 -0.07943519        TRUE
## Dialister             0.2016543 0.11342554 -0.08182304        TRUE
## Treponema             0.2166985 0.07599555 -0.07283265        TRUE
##                       Contrib.Medium Contrib.High Contrib GroupContrib   color
## Mycoplasma                     FALSE        FALSE   FALSE          Low #388ECC
## Bacteroidaceae_[G.1]           FALSE        FALSE   FALSE          Low #388ECC
## Anaeroglobus                   FALSE        FALSE   FALSE          Low #388ECC
## Veillonellaceae_[G.1]          FALSE        FALSE   FALSE          Low #388ECC
## Dialister                      FALSE        FALSE   FALSE          Low #388ECC
## Treponema                      FALSE        FALSE   FALSE          Low #388ECC
##                       importance
## Mycoplasma            0.57040721
## Bacteroidaceae_[G.1]  0.56105703
## Anaeroglobus          0.49912528
## Veillonellaceae_[G.1] 0.28917387
## Dialister             0.15345304
## Treponema             0.05962567
```

```r
write.table(comp1, file="act_comp1.txt", sep="\t", dec=",", row.names=T)
comp2 <- plotLoadings(final.splsda, comp = 2, method = 'mean', contrib = 'max',
                      size.title = 1)
```

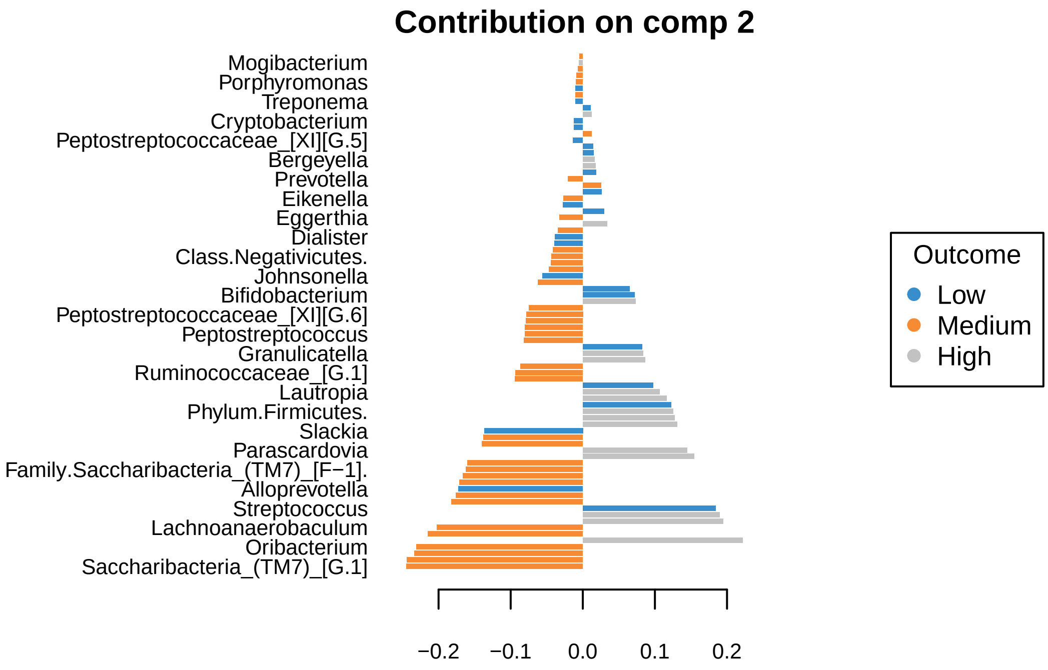


```r
head(comp2, n=20)
```

```
##                                             Low       Medium         High
## Saccharibacteria_(TM7)_[G.1]        -0.05368575  0.1547003191 -0.039032610
## Family.Selenomonadaceae.             0.06211215  0.1415665415 -0.060275121
## Kingdom.Bacteria.                    0.04161681  0.1363835795 -0.054045991
## Oribacterium                        -0.13067746  0.1522966107 -0.021246025
## Kingella                            -0.15149903 -0.0999116314  0.066306262
## Saccharibacteria_(TM7)_[G.6]        -0.12882661  0.1639737730 -0.025495434
## Lachnoanaerobaculum                 -0.12329646  0.1512333350 -0.022525653
## Cardiobacterium                     -0.11932049 -0.0933466833  0.057043499
## Haemophilus                         -0.06788003 -0.1701473989  0.070949151
## Streptococcus                        0.07597056 -0.1658607362  0.037784093
## Catonella                           -0.04443548  0.1101391045 -0.026417612
## Saccharibacteria_(TM7)_[G.3]        -0.11797470  0.1427923758 -0.020924048
## Alloprevotella                       0.07971638  0.0780571668 -0.043271866
## Peptostreptococcaceae_[XI][G.7]      0.02946357  0.1390070793 -0.052226074
## Fusobacterium                        0.06976020  0.1089785821 -0.051244656
## Family.Saccharibacteria_(TM7)_[F-1]. -0.16251992  0.1370310906 -0.009195675
## Solobacterium                       -0.18788167  0.1388360343 -0.004190733
```

```
## Lachnospiraceae_[G.2]                    -0.21571142 -0.0656656779  0.069217060
## Parascardovia                            -0.10647838 -0.0003072968  0.023606237
## Leptotrichia                             -0.11789614  0.1214372037 -0.013917282
##                                          Contrib.Low Contrib.Medium Contrib.High
## Saccharibacteria_(TM7)_[G.1]                   FALSE           TRUE        FALSE
## Family.Selenomonadaceae.                       FALSE           TRUE        FALSE
## Kingdom.Bacteria.                              FALSE           TRUE        FALSE
## Oribacterium                                   FALSE           TRUE        FALSE
## Kingella                                       FALSE          FALSE         TRUE
## Saccharibacteria_(TM7)_[G.6]                   FALSE           TRUE        FALSE
## Lachnoanaerobaculum                            FALSE           TRUE        FALSE
## Cardiobacterium                                FALSE          FALSE         TRUE
## Haemophilus                                    FALSE          FALSE         TRUE
## Streptococcus                                   TRUE          FALSE        FALSE
## Catonella                                      FALSE           TRUE        FALSE
## Saccharibacteria_(TM7)_[G.3]                   FALSE           TRUE        FALSE
## Alloprevotella                                  TRUE          FALSE        FALSE
## Peptostreptococcaceae_[XI][G.7]                FALSE           TRUE        FALSE
## Fusobacterium                                  FALSE           TRUE        FALSE
## Family.Saccharibacteria_(TM7)_[F-1].           FALSE           TRUE        FALSE
## Solobacterium                                  FALSE           TRUE        FALSE
## Lachnospiraceae_[G.2]                          FALSE          FALSE         TRUE
## Parascardovia                                  FALSE          FALSE         TRUE
## Leptotrichia                                   FALSE           TRUE        FALSE
##                                          Contrib GroupContrib   color importance
## Saccharibacteria_(TM7)_[G.1]               FALSE       Medium #F68B33 -0.2444898
## Family.Selenomonadaceae.                   FALSE       Medium #F68B33 -0.2441074
## Kingdom.Bacteria.                          FALSE       Medium #F68B33 -0.2335555
## Oribacterium                               FALSE       Medium #F68B33 -0.2306284
## Kingella                                   FALSE         High #C2C2C2  0.2216933
## Saccharibacteria_(TM7)_[G.6]               FALSE       Medium #F68B33 -0.2147329
## Lachnoanaerobaculum                        FALSE       Medium #F68B33 -0.2023359
## Cardiobacterium                            FALSE         High #C2C2C2  0.1944729
## Haemophilus                                FALSE         High #C2C2C2  0.1893801
## Streptococcus                              FALSE          Low #388ECC  0.1839472
## Catonella                                  FALSE       Medium #F68B33 -0.1821444
## Saccharibacteria_(TM7)_[G.3]               FALSE       Medium #F68B33 -0.1759744
## Alloprevotella                             FALSE          Low #388ECC -0.1724384
## Peptostreptococcaceae_[XI][G.7]            FALSE       Medium #F68B33 -0.1709193
## Fusobacterium                              FALSE       Medium #F68B33 -0.1663719
## Family.Saccharibacteria_(TM7)_[F-1].       FALSE       Medium #F68B33 -0.1619092
## Solobacterium                              FALSE       Medium #F68B33 -0.1598135
## Lachnospiraceae_[G.2]                      FALSE         High #C2C2C2  0.1542622
## Parascardovia                              FALSE         High #C2C2C2  0.1449705
## Leptotrichia                               FALSE       Medium #F68B33 -0.1399029
```

```
write.table(comp2, file="act_comp2.txt", sep="\t", dec=",", row.names=T)
comp3 <- plotLoadings(final.splsda, comp = 3, method = 'mean', contrib = 'max',
                      size.title = 1)
```

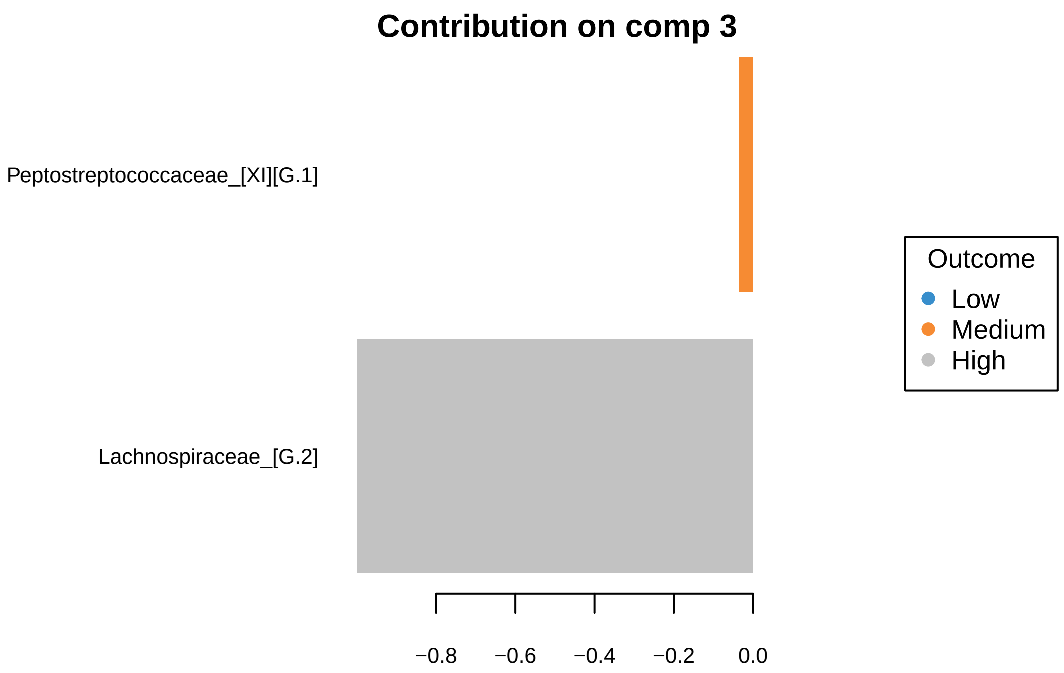


```
head(comp3, n=20)
```

```
##                                      Low      Medium       High Contrib.Low
## Lachnospiraceae_[G.2]           -0.2157114 -0.06566568 0.06921706       FALSE
## Peptostreptococcaceae_[XI][G.1] -0.1324912  0.04612510 0.01407612       FALSE
##                                 Contrib.Medium Contrib.High Contrib
## Lachnospiraceae_[G.2]                    FALSE         TRUE   FALSE
## Peptostreptococcaceae_[XI][G.1]           TRUE        FALSE   FALSE
##                                 GroupContrib   color  importance
## Lachnospiraceae_[G.2]                   High #C2C2C2 -0.99939091
## Peptostreptococcaceae_[XI][G.1]       Medium #F68B33 -0.03489715
```

```
write.table(comp3, file="act_comp3.txt", sep="\t", dec=",", row.names=T)

##  Sample plots from sPLS-DA including 95% confidence ellipses. Samples are projected into the space s
plotIndiv(final.splsda, comp = c(1,2), # plot samples from final model
          group = Y, ind.names = FALSE, # colour by class label
          ellipse = TRUE, legend = TRUE, # include 95% confidence ellipse
          title = ' (a) sPLS-DA on addp, comp 1 & 2')
```

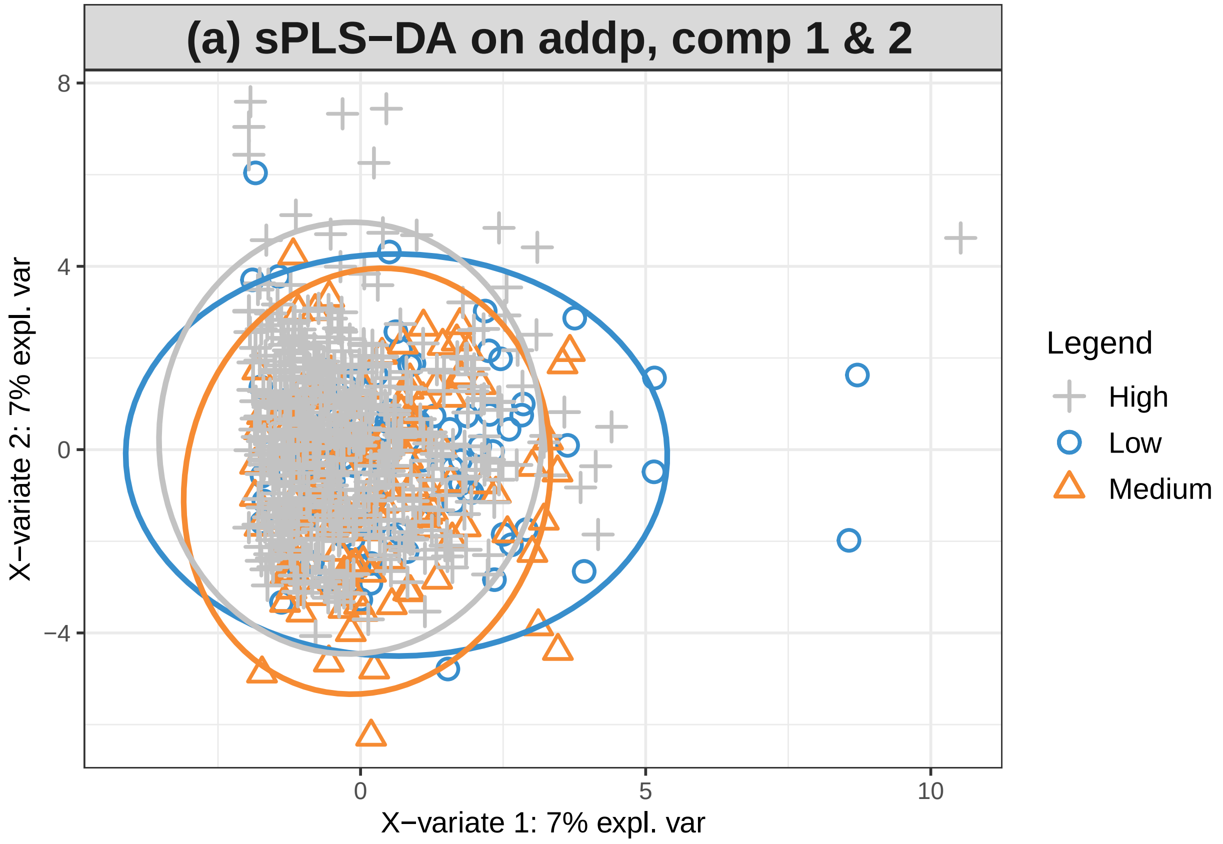


```
# plotIndiv(final.splsda, comp = c(1,3), # plot samples from final model
#           group = Y, ind.names = FALSE,  # colour by class label
#           ellipse = TRUE, legend = TRUE, # include 95% confidence ellipse
#           title = '(b) sPLS-DA on addp, comp 1 & 3')

##Stability of variable selection from the sPLS-DA. The barplot represents the frequency of selection a
# form new perf() object which utilises the final model
perf.splsda.addp <- perf(final.splsda,
                          folds = 5, nrepeat = 10, # use repeated cross-validation
                          validation = "Mfold", dist = "max.dist",  # use max.dist measure
                          progressBar = FALSE)

# plot the stability of each feature for the first three components, 'h' type refers to histogram
par(mfrow=c(1,2))
plot(perf.splsda.addp$features$stable[[1]], type = 'h',
     ylab = 'Stability',
     xlab = 'Features',
     main = '(a) Comp 1', las =2)
plot(perf.splsda.addp$features$stable[[2]], type = 'h',
     ylab = 'Stability',
     xlab = 'Features',
     main = '(b) Comp 2', las =2)
```

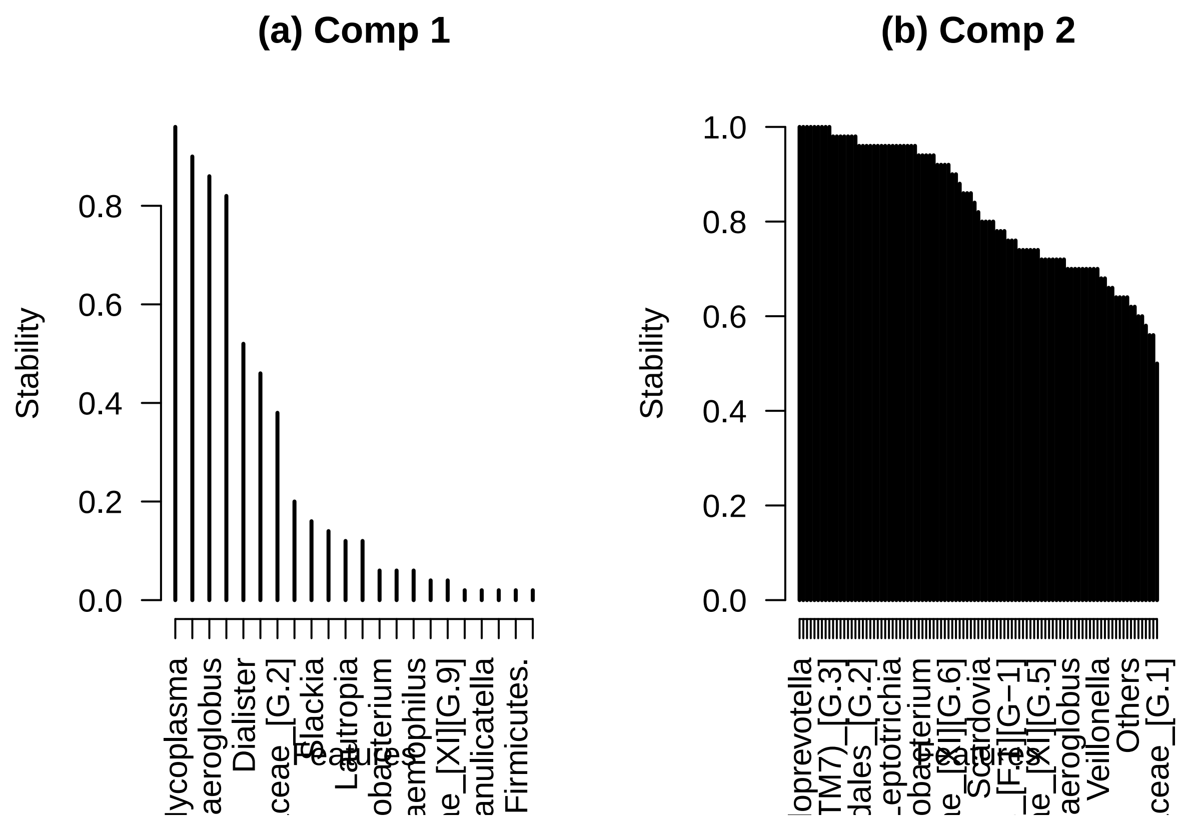


```
# plot(perf.splsda.addp$features$stable[[3]], type = 'h',
#      ylab = 'Stability',
#      xlab = 'Features',
#      main = '(c) Comp 3', las =2)
par(mfrow=c(1,1))


train <- sample(1:nrow(X), 0.75*nrow(X)) # randomly select 75% of samples in training
test <- setdiff(1:nrow(X), train) # rest is part of the test set

# store matrices into training and test set:
X.train <- X[train, ]
X.test <- X[test,]
Y.train <- Y[train]
Y.test <- Y[test]

# train the model
train.splsda.addp <- splsda(X.train, Y.train, ncomp = optimal.ncomp, keepX = optimal.keepX)

# use the model on the Xtest set
predict.splsda.addp <- predict(train.splsda.addp, X.test, dist = "max.dist") #Changed from Mahalanobis

# evaluate the prediction accuracy for the first two components
predict.comp2 <- predict.splsda.addp$class$max.dist[,3]
table(factor(predict.comp2, levels = c("Low", "Medium", "High")), Y.test)
```

```
##         Y.test
##          Low Medium High
##   Low      0      4    4
##   Medium   0      0    0
##   High    22     31  115
```

```
#Correct classification rate
sum(diag(table(factor(predict.comp2, levels = c("Low", "Medium", "High")), Y.test)))/
  sum(table(factor(predict.comp2, levels = c("Low", "Medium", "High")), Y.test))
```

```
## [1] 0.6534091
```

```
## ROC curve and AUC from sPLS-DA on component 1 (a) and all (two) components (b) averaged across one-v
auc.splsda = auroc(final.splsda, roc.comp = 1, print = FALSE) # AUROC for the first component
```

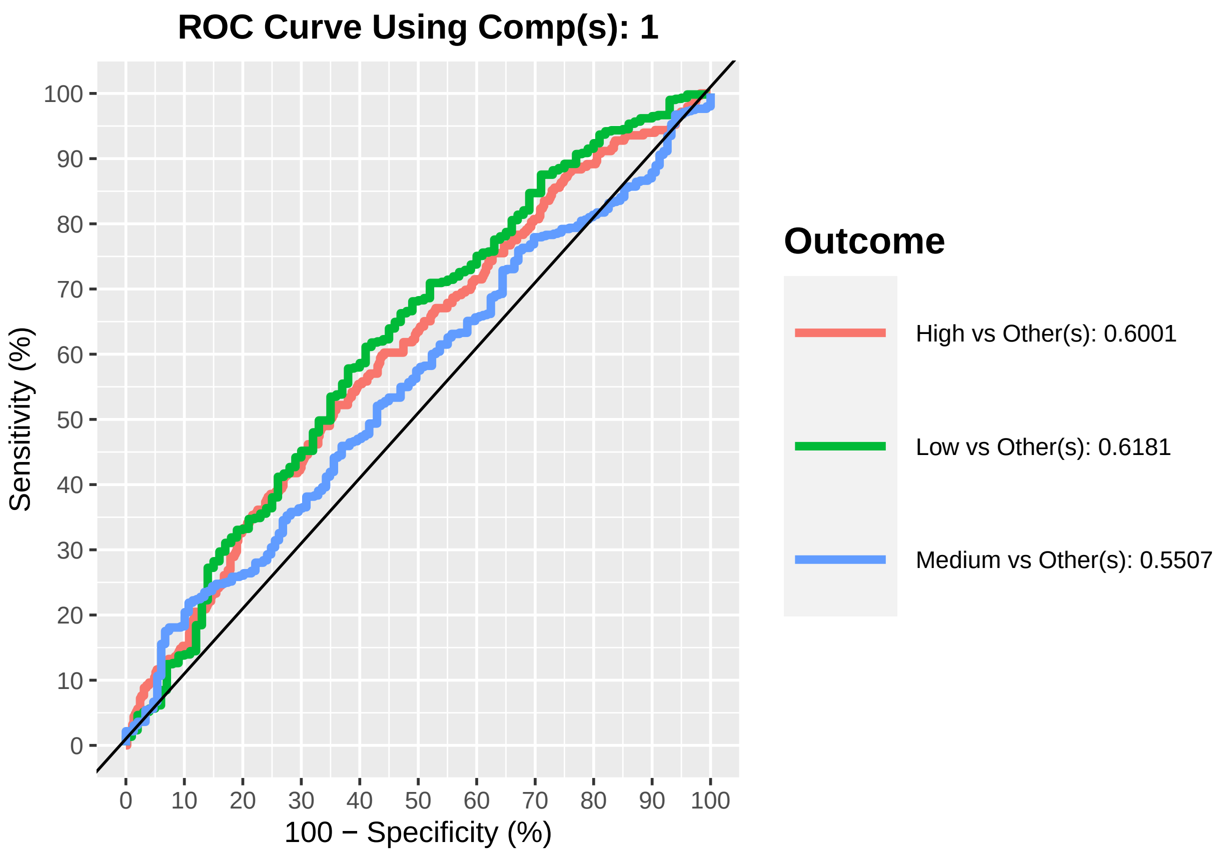


```
auc.splsda = auroc(final.splsda, roc.comp = 3, print = FALSE) # AUROC for the first and second componen
```

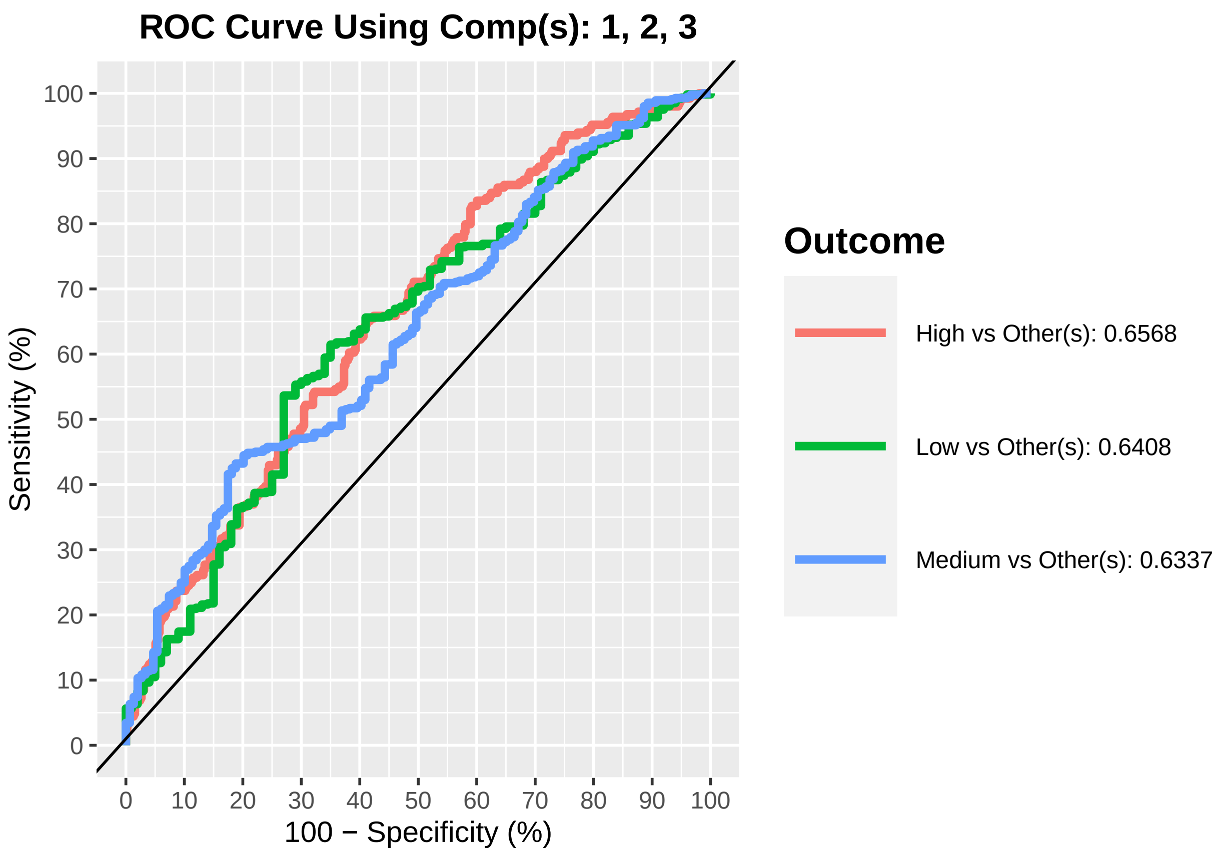


```
ROClist[["actROC"]] <-auc.splsda$graph.Comp3

#AUC
print("AUC")
```

```
## [1] "AUC"
```

```
auc.splsda$Comp3
```

```
##                          AUC   p-value
## Low vs Other(s)     0.6408 6.363e-06
## Medium vs Other(s) 0.6337 5.275e-07
## High vs Other(s)    0.6568 5.940e-12
```

```
rm(list=setdiff(ls(), c("FeaturePic2", "Pic2", "Phe", "Microbio", "FeatureMic", "Metabo", "paretoscale"

set.seed(99) # for reproducibility, remove for normal use

Microbio2<-Microbio
#Subset select or just remove NA
table(Phe$hba1c_cat, useNA="always")
```

hba1c_cat

```
##
##    Low Medium   High   <NA>
##    600    119     26      1
```

```
Phe2<-Phe[complete.cases(Phe$hba1c_cat), ] #NA
Phe2<-subset(Phe2, hba1c_cat %in% c("Low", "Medium")) #also removes NA, but above can be easier to impl
Phe2$hba1c_cat<-droplevels(Phe2$hba1c_cat)
X<-dplyr::select(Microbio2, one_of(Phe2$IDX)) #Also in X
##Remove orgs that are not present after subsetting.
X <- X[rowSums(X)>0,]


#Hellinger transformation
X <- data.frame(t(decostand(t(X), method="hellinger")))
#Maks TSS
X<-sweep(X, 2, colSums(X), FUN="/")
#rowSums(X)
sum(colnames(X)!=Phe2$IDX)==0
```

```
## [1] TRUE
```

```
X<-t(X)
Y<-Phe2$hba1c_cat

sum(rownames(X)!=Phe2$IDX)==0
```

```
## [1] TRUE
```

```
dim(X) # check the dimensions of the X dataframe
```

```
## [1] 719  97
```

```
summary(Y) # check the distribution of class labels
```

```
##    Low Medium
##    600    119
```

```
# Barplot of the variance each principal component explains
pca.addp = pca(X, ncomp = 10, center = TRUE, scale = TRUE) # run pca method on data
plot(pca.addp)  # barplot of the eigenvalues (explained variance per component)
```

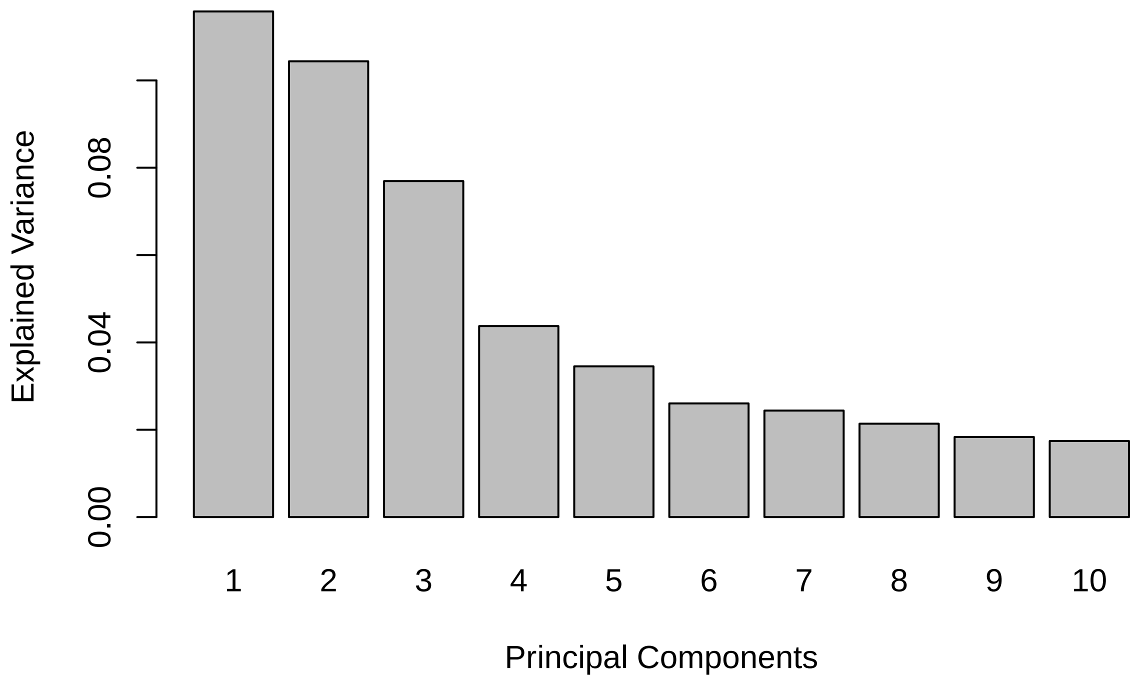


```
# Preliminary (unsupervised) analysis with PCA
plotIndiv(pca.addp, group = Y, ind.names = FALSE, # plot the samples projected
          legend = TRUE, title = 'PCA on addp, comp 1 - 2') # onto the PCA subspace
```

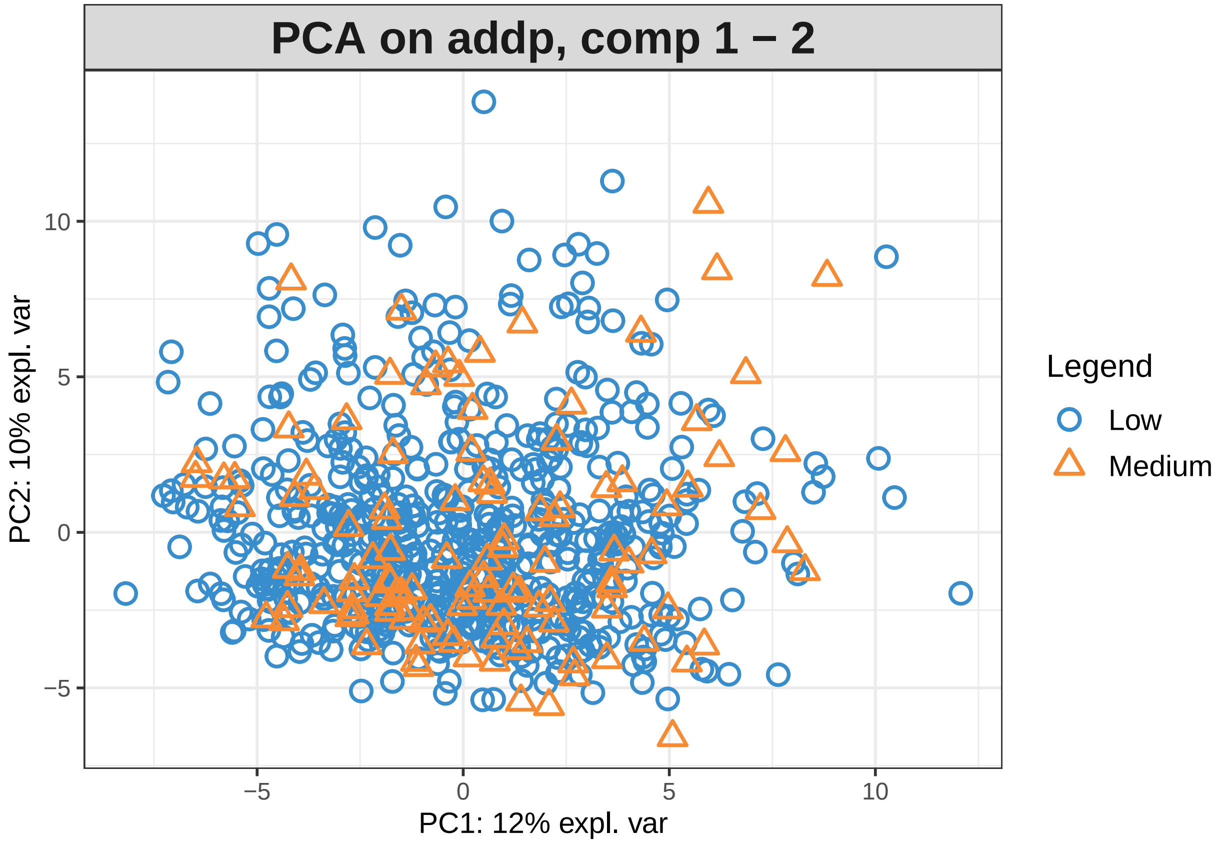


```
addp.splsda <- splsda(X, Y, ncomp = 10)  # set ncomp to 10 for performance assessment later

## Sample plots after a basic PLS-DA model was operated on this data.
# plot the samples projected onto the first two components of the PLS-DA subspace
plotIndiv(addp.splsda , comp = 1:2,
          group = Y, ind.names = FALSE,  # colour points by class
          ellipse = TRUE, # include 95% confidence ellipse for each class
          legend = TRUE, title = '(a) PLSDA with confidence ellipses')
```

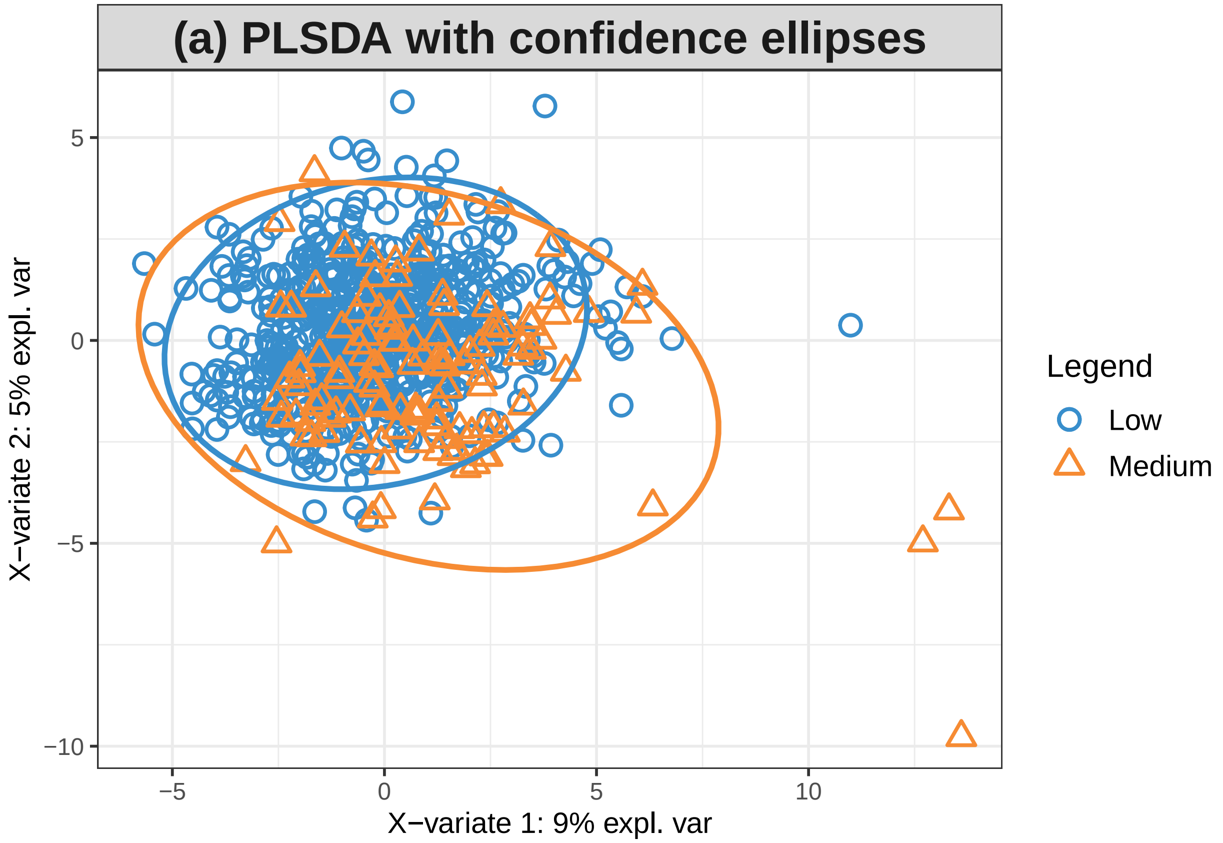


```
# use the max.dist measure to form decision boundaries between classes based on PLS-DA data
background = background.predict(addp.splsda, comp.predicted=2, dist = "max.dist")

# plot the samples projected onto the first two components of the PLS-DA subspace
plotIndiv(addp.splsda, comp = 1:2,
          group = Y, ind.names = FALSE, # colour points by class
          background = background, # include prediction background for each class
          legend = TRUE, title = " (b) PLSDA with prediction background")
```

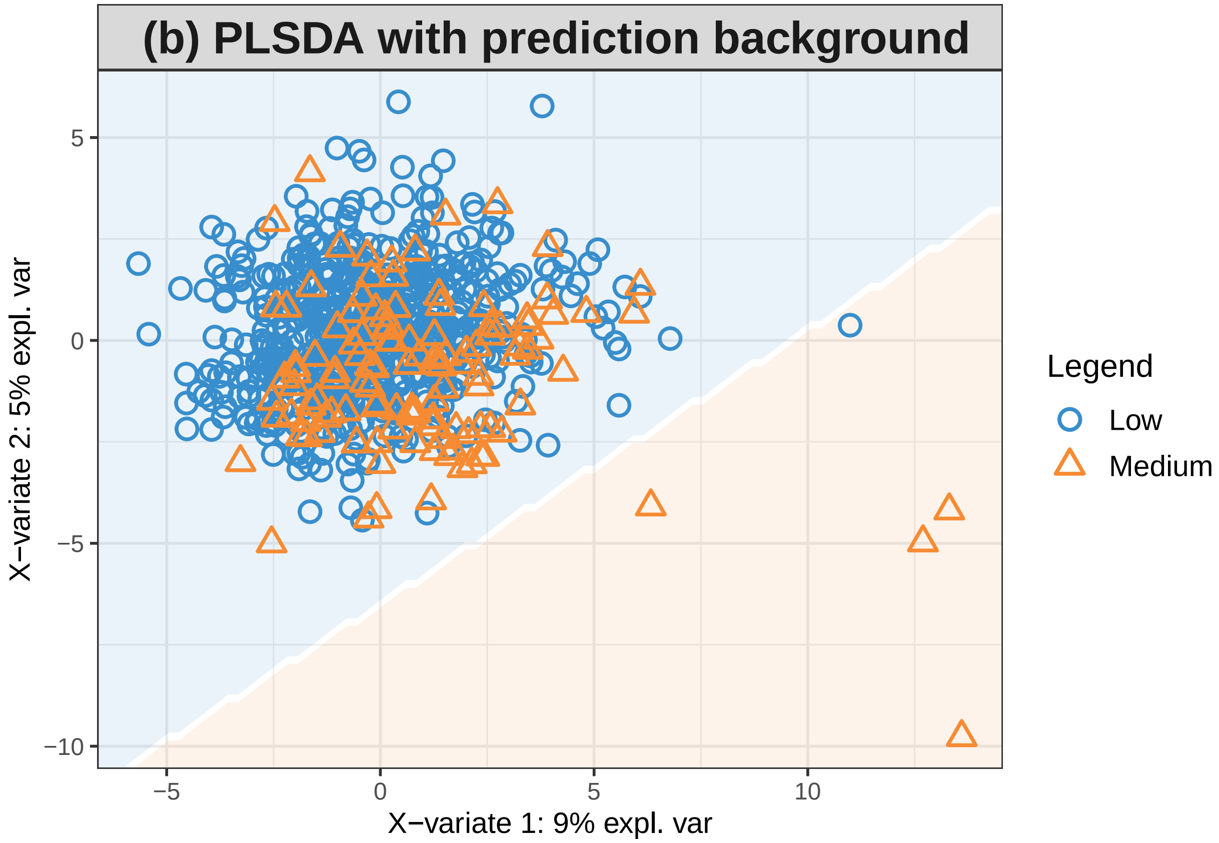


```
## Tuning the number of components in PLS-DA. For each component, repeated cross-validation (10 × 3-fol
# undergo performance evaluation in order to tune the number of components to use
perf.splsda.addp <- perf(addp.splsda, validation = "Mfold",
                          folds = 5, nrepeat = 10, # use repeated cross-validation
                          progressBar = FALSE, auc = TRUE) # include AUC values

# plot the outcome of performance evaluation across all ten components
plot(perf.splsda.addp, col = color.mixo(5:7), sd = TRUE,
     legend.position = "horizontal")
```

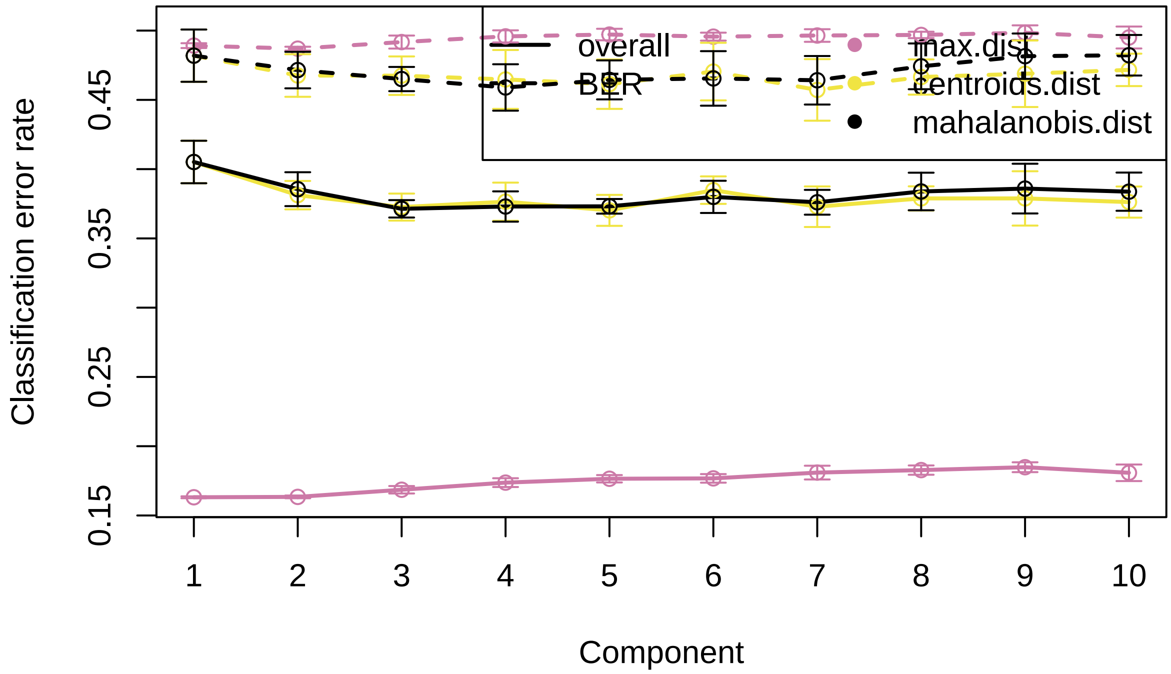


```
perf.splsda.addp$choice.ncomp # what is the optimal value of components according to perf()
```

```
##         max.dist centroids.dist mahalanobis.dist
## overall        2              7                4
## BER            2              7                4
```

```
## Tuning keepX for the sPLS-DA. Each coloured line represents the balanced error rate (y-axis) per com
# grid of possible keepX values that will be tested for each component
list.keepX <- c(1:10,  seq(20, 180, 10))

# undergo the tuning process to determine the optimal number of variables
tune.splsda.addp <- tune.splsda(X, Y, ncomp = 4, # calculate for first 4 components
                                validation = 'Mfold',
                                folds = 5, nrepeat = 10, # use repeated cross-validation
                                dist = 'max.dist', # use max.dist measure
                                measure = "BER", # use balanced error rate of dist measure
                                test.keepX = list.keepX,
                                cpus = 2 # allow for parallelisation to decrease runtime
                                )

plot(tune.splsda.addp, col = color.jet(4))
```

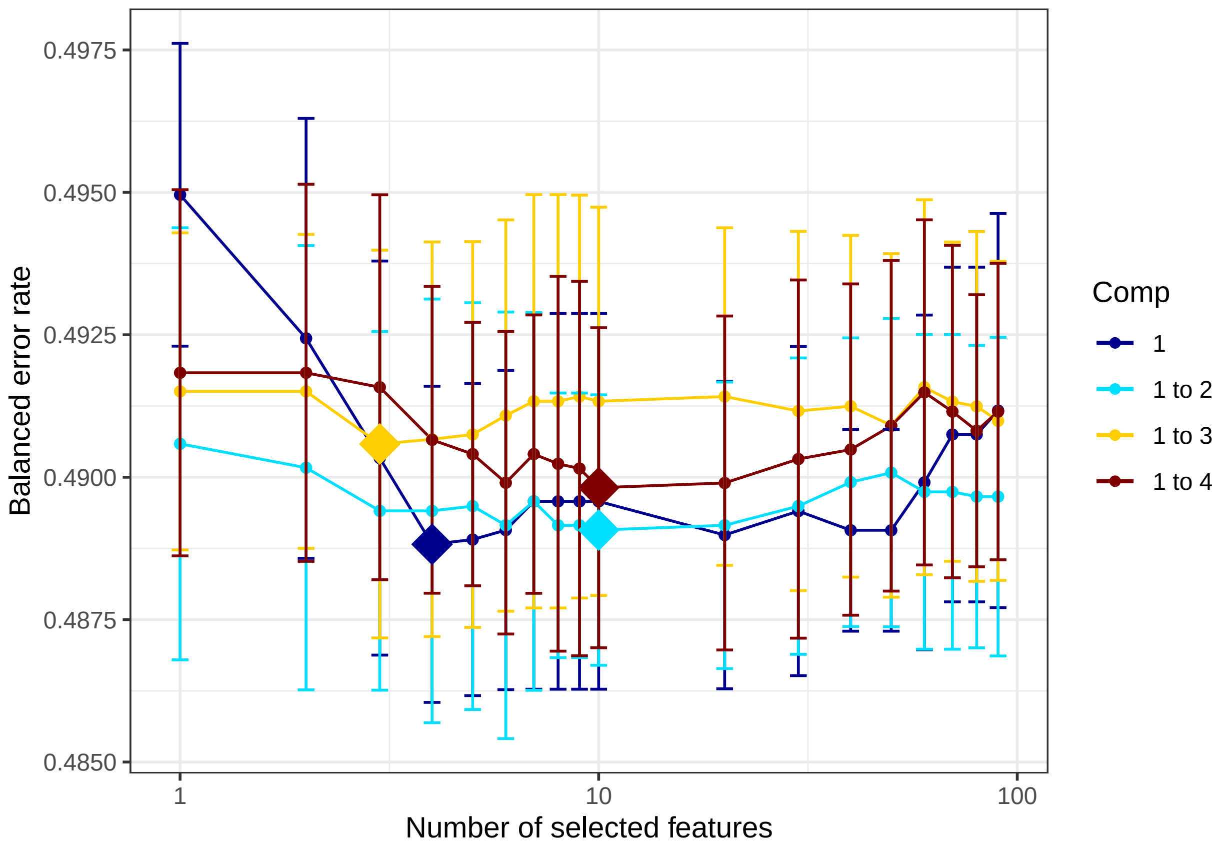


```
tune.splsda.addp$choice.ncomp$ncomp # what is the optimal value of components according to tune.splsda(
```

```
## [1] 1
```

```
tune.splsda.addp$choice.keepX # what are the optimal values of variables according to tune.splsda()
```

```
## comp1 comp2 comp3 comp4
##     4    10     3    10
```

```
optimal.ncomp <- 2 #See above max dist
optimal.keepX <- tune.splsda.addp$choice.keepX[1:optimal.ncomp]

# form final model with optimised values for component and variable count
final.splsda <- splsda(X, Y,
                       ncomp = optimal.ncomp,
                       keepX = optimal.keepX)

#########Loadings
#plotLoadings(final.splsda, comp=1, contrib = 'max', method = 'mean', size.title = 1)
#plotLoadings(final.splsda, comp=2, contrib = 'max', method = 'mean', size.title = 1)

comp1 <- plotLoadings(final.splsda, comp = 1, method = 'mean', contrib = 'max',
                      size.title = 1)
```

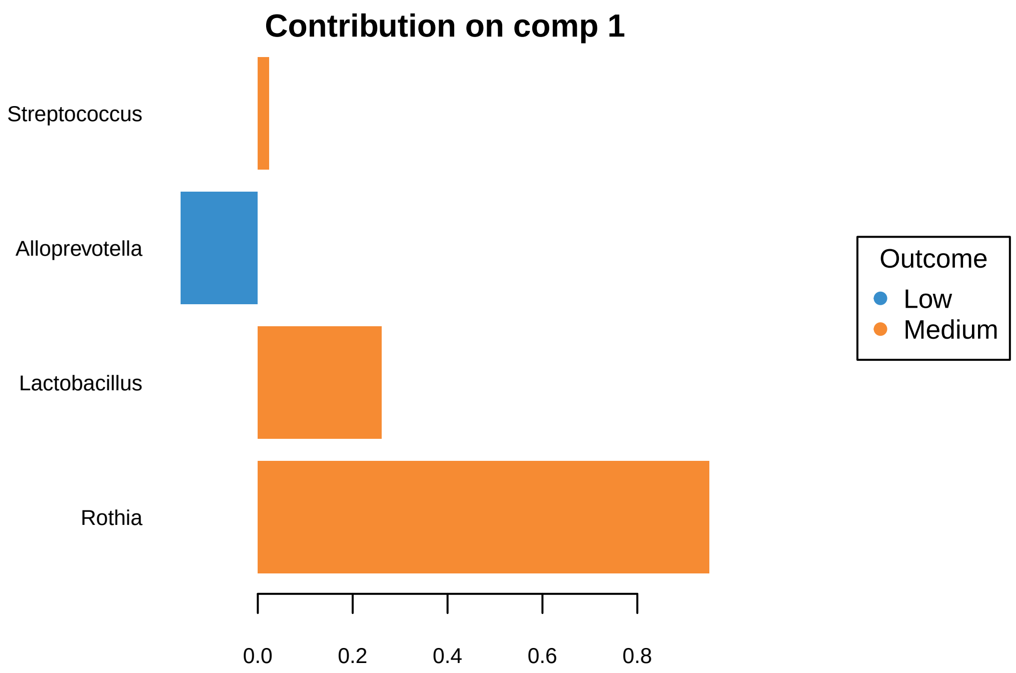


```
head(comp1, n=20)
```

```
##                       Low     Medium Contrib.Low Contrib.Medium Contrib
## Rothia         -0.07041553  0.3550363       FALSE           TRUE   FALSE
## Lactobacillus  -0.05425152  0.2735371       FALSE           TRUE   FALSE
## Alloprevotella  0.05194629 -0.2619141        TRUE          FALSE   FALSE
## Streptococcus  -0.04871844  0.2456392       FALSE           TRUE   FALSE
##                GroupContrib   color  importance
## Rothia               Medium #F68B33  0.95141334
## Lactobacillus        Medium #F68B33  0.26065046
## Alloprevotella          Low #388ECC -0.16213743
## Streptococcus        Medium #F68B33  0.02419629
```

```
write.table(comp1, file="hba1c_comp1.txt", sep="\t", dec=",", row.names=T)
comp2 <- plotLoadings(final.splsda, comp = 2, method = 'mean', contrib = 'max',
                      size.title = 1)
```

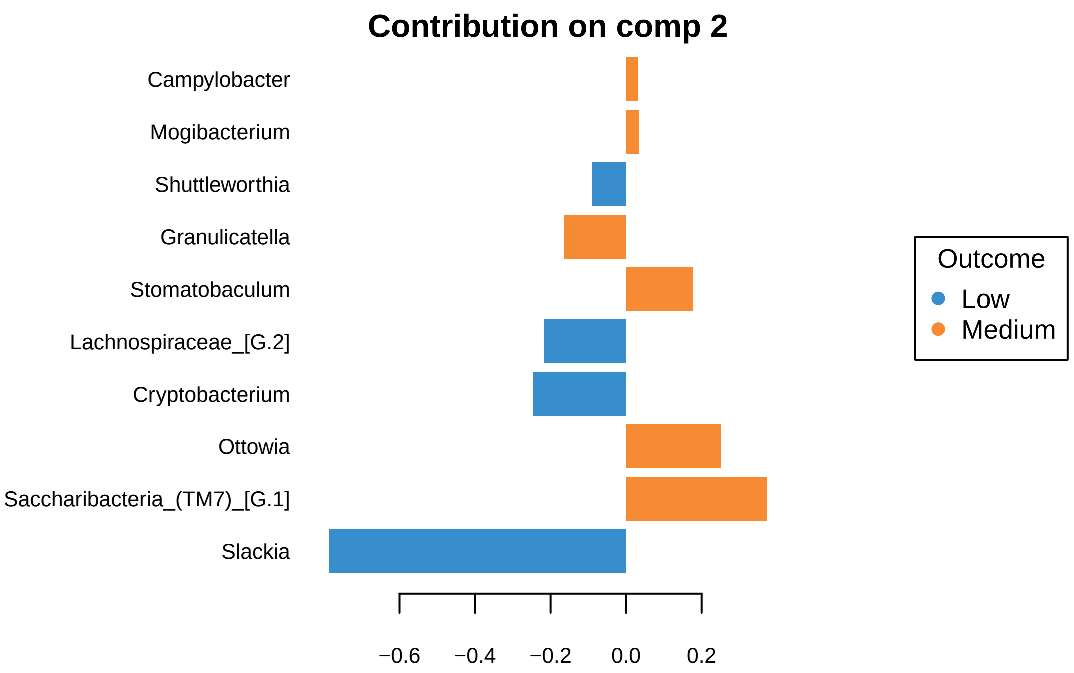


```r
head(comp2, n=20)
```

```
##                                      Low      Medium Contrib.Low
## Slackia                      0.048152237 -0.24278439        TRUE
## Saccharibacteria_(TM7)_[G.1] -0.011917257  0.06008701       FALSE
## Ottowia                      -0.021568668  0.10874959       FALSE
## Cryptobacterium              0.026168859 -0.13194383        TRUE
## Lachnospiraceae_[G.2]        0.044431790 -0.22402583        TRUE
## Stomatobaculum               -0.019805542  0.09985988       FALSE
## Granulicatella               -0.000309055  0.00155826       FALSE
## Shuttleworthia               0.029744795 -0.14997376        TRUE
## Mogibacterium                -0.011669931  0.05883999       FALSE
## Campylobacter                -0.018098327  0.09125207       FALSE
##                              Contrib.Medium Contrib GroupContrib   color
## Slackia                               FALSE   FALSE          Low #388ECC
## Saccharibacteria_(TM7)_[G.1]           TRUE   FALSE       Medium #F68B33
## Ottowia                                TRUE   FALSE       Medium #F68B33
## Cryptobacterium                       FALSE   FALSE          Low #388ECC
## Lachnospiraceae_[G.2]                 FALSE   FALSE          Low #388ECC
## Stomatobaculum                         TRUE   FALSE       Medium #F68B33
## Granulicatella                         TRUE   FALSE       Medium #F68B33
## Shuttleworthia                        FALSE   FALSE          Low #388ECC
## Mogibacterium                          TRUE   FALSE       Medium #F68B33
## Campylobacter                          TRUE   FALSE       Medium #F68B33
##                                importance
```

```
## Slackia                              -0.78764079
## Saccharibacteria_(TM7)_[G.1]          0.37328560
## Ottowia                               0.25177169
## Cryptobacterium                      -0.24761365
## Lachnospiraceae_[G.2]                -0.21711172
## Stomatobaculum                        0.17717488
## Granulicatella                       -0.16461118
## Shuttleworthia                       -0.08898655
## Mogibacterium                         0.03307739
## Campylobacter                         0.03066494
```

```r
write.table(comp2, file="hba1c_comp2.txt", sep="\t", dec=",", row.names=T)

##  Sample plots from sPLS-DA including 95% confidence ellipses. Samples are projected into the space sp
plotIndiv(final.splsda, comp = c(1,2), # plot samples from final model
          group = Y, ind.names = FALSE, # colour by class label
          ellipse = TRUE, legend = TRUE, # include 95% confidence ellipse
          title = ' (a) sPLS-DA on addp, comp 1 & 2')
```

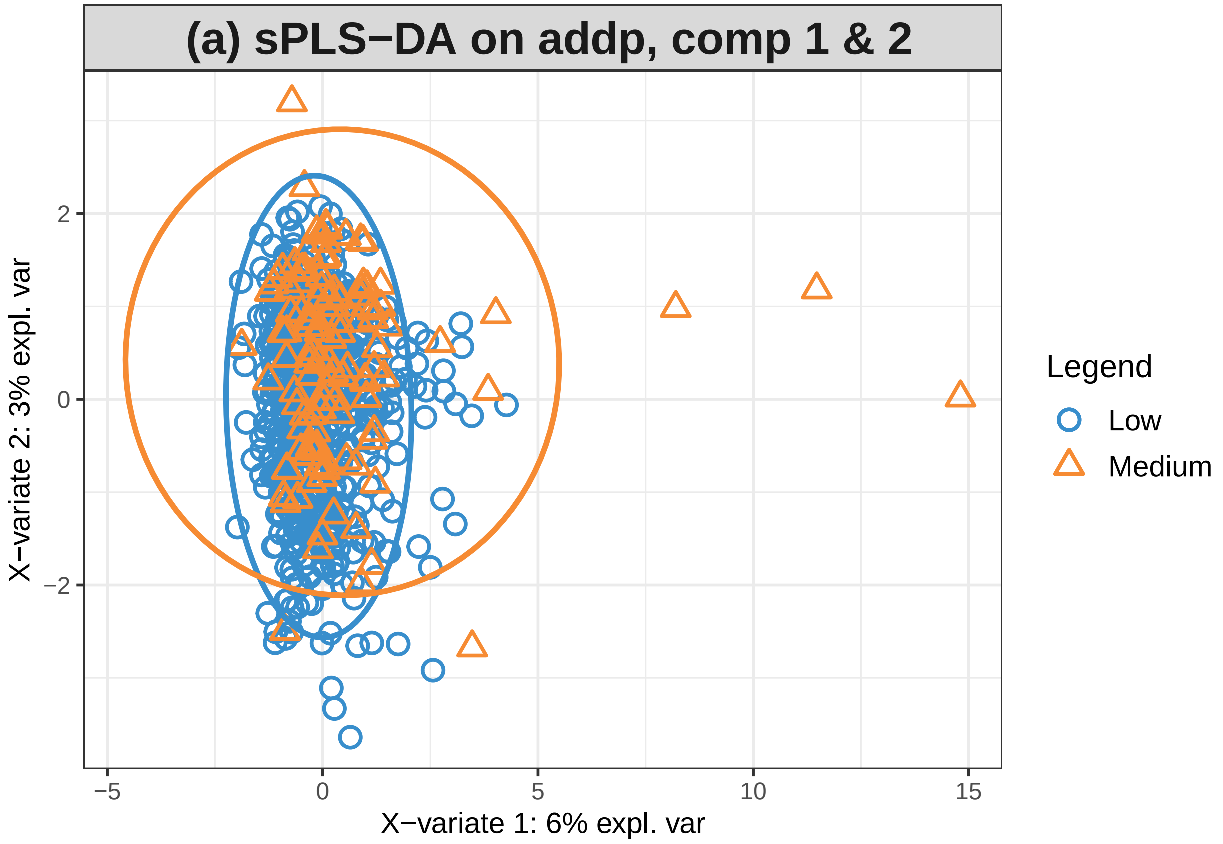


```r
# plotIndiv(final.splsda, comp = c(1,3), # plot samples from final model
#           group = Y, ind.names = FALSE,  # colour by class label
#           ellipse = TRUE, legend = TRUE, # include 95% confidence ellipse
#           title = '(b) sPLS-DA on addp, comp 1 & 3')

##Stability of variable selection from the sPLS-DA. The barplot represents the frequency of selection ac
```

```
# form new perf() object which utilises the final model
perf.splsda.addp <- perf(final.splsda,
                         folds = 5, nrepeat = 10, # use repeated cross-validation
                         validation = "Mfold", dist = "max.dist",  # use max.dist measure
                         progressBar = FALSE)

# plot the stability of each feature for the first three components, 'h' type refers to histogram
par(mfrow=c(1,2))
plot(perf.splsda.addp$features$stable[[1]], type = 'h',
     ylab = 'Stability',
     xlab = 'Features',
     main = '(a) Comp 1', las =2)
plot(perf.splsda.addp$features$stable[[2]], type = 'h',
     ylab = 'Stability',
     xlab = 'Features',
     main = '(b) Comp 2', las =2)
```

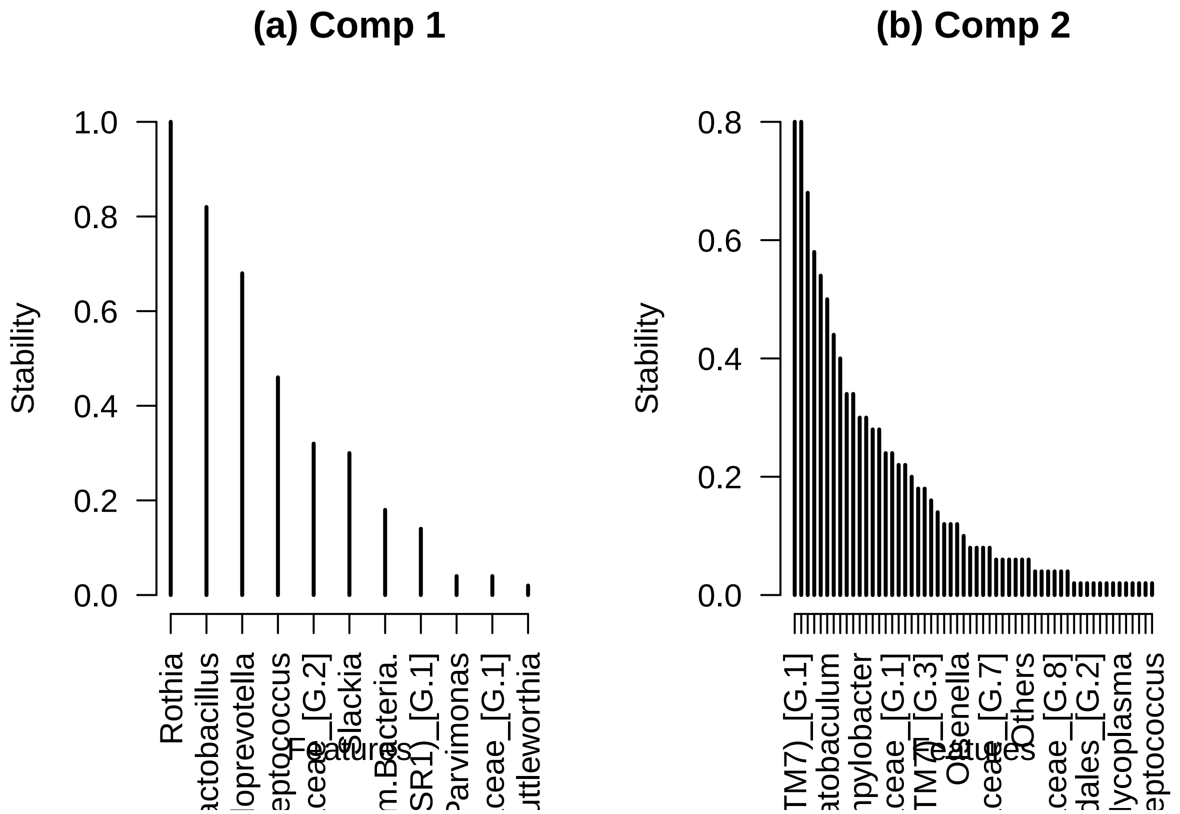


```
# plot(perf.splsda.addp$features$stable[[3]], type = 'h',
#      ylab = 'Stability',
#      xlab = 'Features',
#      main = '(c) Comp 3', las =2)
par(mfrow=c(1,1))


train <- sample(1:nrow(X), 0.75*nrow(X)) # randomly select 75% of samples in training
```

```r
test <- setdiff(1:nrow(X), train) # rest is part of the test set

# store matrices into training and test set:
X.train <- X[train, ]
X.test <- X[test,]
Y.train <- Y[train]
Y.test <- Y[test]

# train the model
train.splsda.addp <- splsda(X.train, Y.train, ncomp = optimal.ncomp, keepX = optimal.keepX)

# use the model on the Xtest set
predict.splsda.addp <- predict(train.splsda.addp, X.test, dist = "max.dist") #Changed from Mahalanobis

# evaluate the prediction accuracy for the first two components
predict.comp2 <- predict.splsda.addp$class$max.dist[,2]
table(factor(predict.comp2, levels = c("Low", "Medium")), Y.test)
```

```
##         Y.test
##          Low Medium
##   Low    151     29
##   Medium   0      0
```

```r
#Correct classification rate
sum(diag(table(factor(predict.comp2, levels = c("Low", "Medium")), Y.test)))/
  sum(table(factor(predict.comp2, levels = c("Low", "Medium")), Y.test))
```

```
## [1] 0.8388889
```

```r
## ROC curve and AUC from sPLS-DA on component 1 (a) and all (two) components (b) averaged across one-v
auc.splsda = auroc(final.splsda, roc.comp = 1, print = FALSE) # AUROC for the first component
```

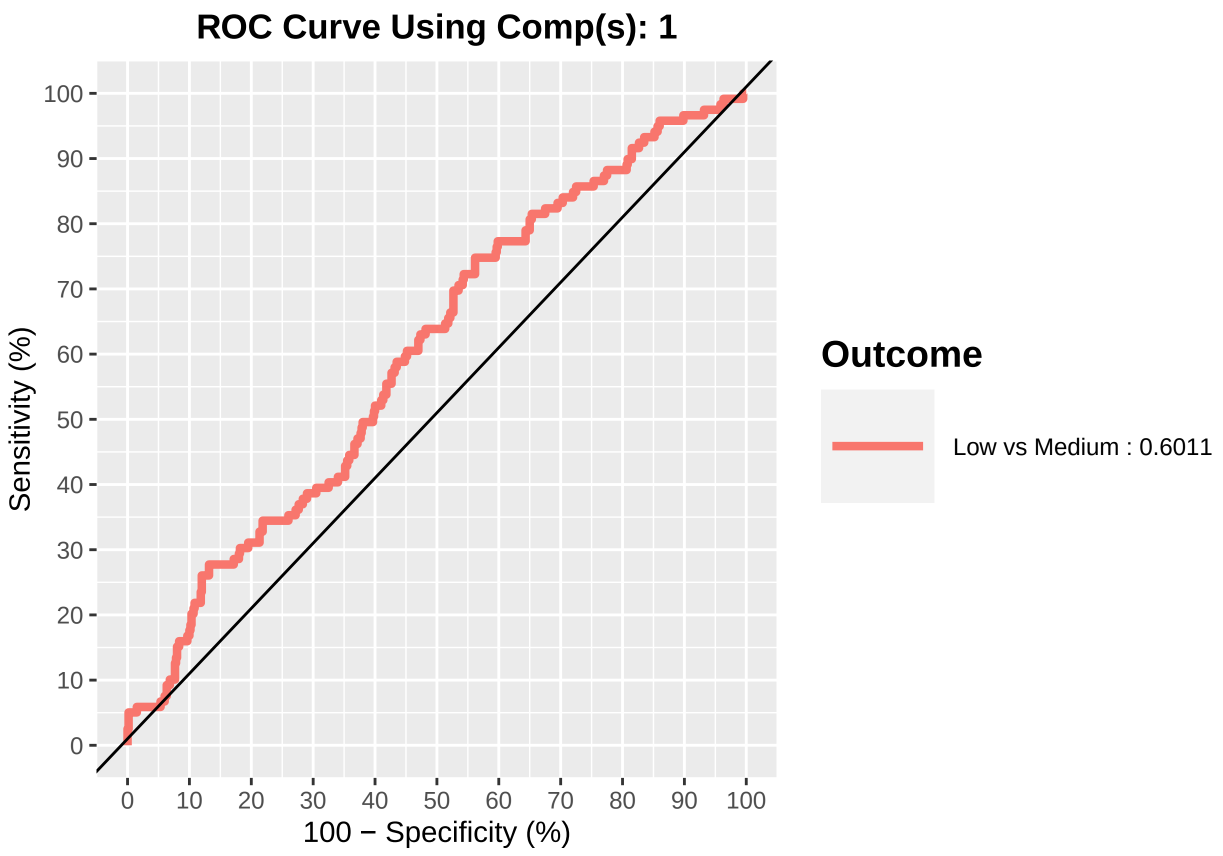


```
auc.splsda = auroc(final.splsda, roc.comp = 2, print = FALSE) # AUROC for the first and second componen
```

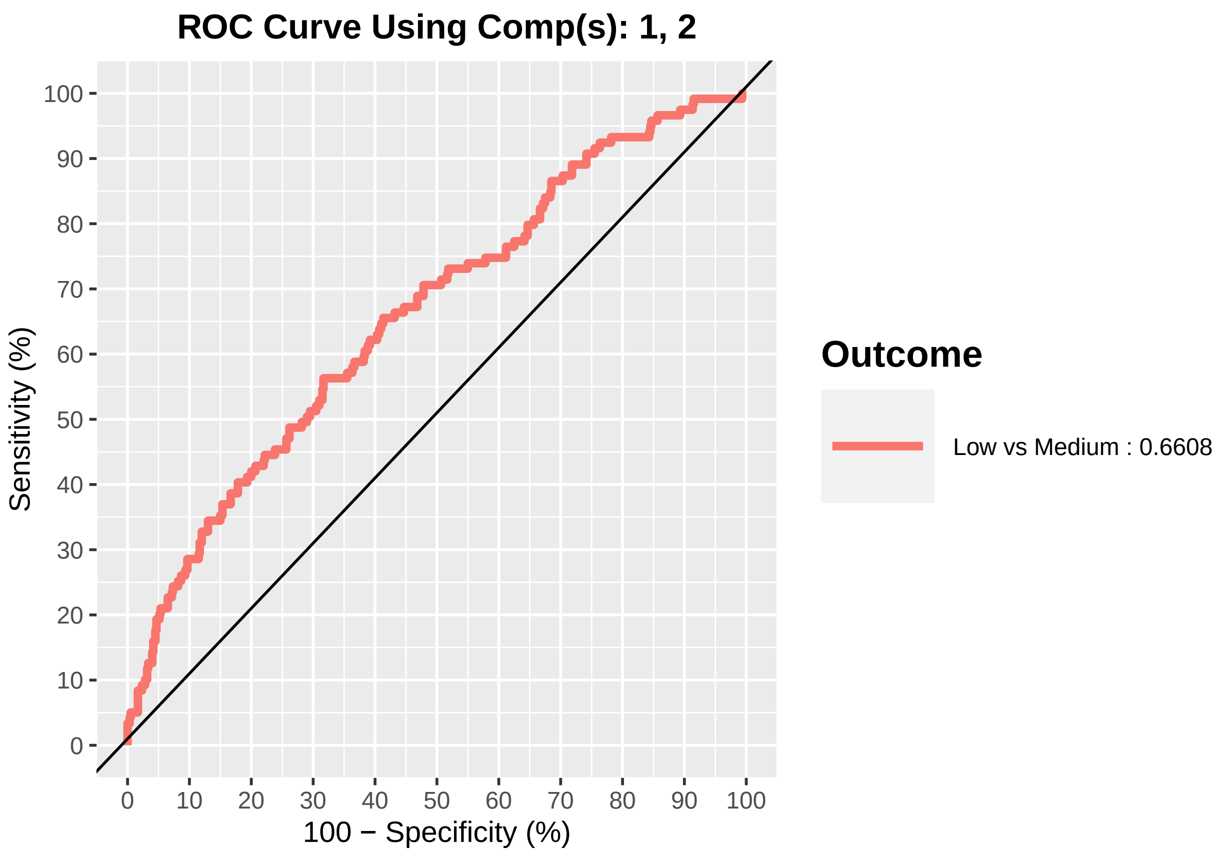


```
ROClist[["hba1cROC"]] <-auc.splsda$graph.Comp2

#AUC
print("AUC")
```

```
## [1] "AUC"
```

```
auc.splsda$Comp2
```

```
##                    AUC   p-value
## Low vs Medium 0.6608 2.931e-08
```

#####ROC figure

```
# #Have the plots stored in list
# lay <- rbind(c(1,2),
#              c(3,4),
#              c(5,6))
#
# pdf(paste("ADDPRO_ROC_main.pdf", sep=""), width=13, height=13)
# grid.arrange(ROClist$RiskROC,
#              ROClist$SmokingROC,
#              ROClist$sexROC,
#              ROClist$alcROC,
```

```r
#               ROClist$hba1cROC,
#               ROClist$actROC, layout_matrix = lay)
# dev.off()
```

Additional

Session information

```r
sessionInfo()
```

```
## R version 4.2.1 (2022-06-23 ucrt)
## Platform: x86_64-w64-mingw32/x64 (64-bit)
## Running under: Windows 10 x64 (build 19044)
##
## Matrix products: default
##
## locale:
## [1] LC_COLLATE=Danish_Denmark.utf8  LC_CTYPE=Danish_Denmark.utf8
## [3] LC_MONETARY=Danish_Denmark.utf8 LC_NUMERIC=C
## [5] LC_TIME=Danish_Denmark.utf8
##
## attached base packages:
##  [1] stats4    tcltk     grid      stats     graphics  grDevices utils
##  [8] datasets  methods   base
##
## other attached packages:
##  [1] eulerr_6.1.1                DAtest_2.8.0
##  [3] copiome_0.1.0               ggpubr_0.4.0
##  [5] mixOmics_6.20.0             MASS_7.3-57
##  [7] forcats_0.5.1               cowplot_1.1.1
##  [9] RColorBrewer_1.1-3          ggraph_2.0.6
## [11] rabuplot_0.0.1.04           phyloseq_1.40.0
## [13] Maaslin2_1.10.0             DESeq2_1.36.0
## [15] SummarizedExperiment_1.26.1 Biobase_2.56.0
## [17] MatrixGenerics_1.8.1        matrixStats_0.62.0
## [19] GenomicRanges_1.48.0        GenomeInfoDb_1.32.2
## [21] IRanges_2.30.0              S4Vectors_0.34.0
## [23] BiocGenerics_0.42.0         reshape2_1.4.4
## [25] BiodiversityR_2.14-3        knitr_1.39
## [27] igraph_1.3.4                plotly_4.10.0
## [29] vegan_2.6-2                 lattice_0.20-45
## [31] permute_0.9-7               ggplot2_3.3.6
## [33] stringr_1.4.0               gridExtra_2.3
## [35] VennDiagram_1.7.3           futile.logger_1.4.3
## [37] readxl_1.4.0                dplyr_1.0.9
## [39] plyr_1.8.7
##
## loaded via a namespace (and not attached):
##   [1] tidyr_1.2.0            bit64_4.0.5            DelayedArray_0.22.0
##   [4] data.table_1.14.2      rpart_4.1.16           KEGGREST_1.36.3
##   [7] RCurl_1.98-1.8         doParallel_1.0.17      generics_0.1.3
```

```
##  [10] snow_0.4-4             lambda.r_1.2.4         RSQLite_2.2.15
##  [13] proxy_0.4-27           bit_4.0.4              viridis_0.6.2
##  [16] relimp_1.0-5           xfun_0.31              hms_1.1.1
##  [19] evaluate_0.16          DEoptimR_1.0-11        fansi_1.0.3
##  [22] caTools_1.18.2         DBI_1.1.3              geneplotter_1.74.0
##  [25] htmlwidgets_1.5.4      rARPACK_0.11-0         purrr_0.3.4
##  [28] ellipsis_0.3.2         RSpectra_0.16-1        backports_1.4.1
##  [31] insight_0.18.2         survey_4.1-1           annotate_1.74.0
##  [34] deldir_1.0-6           vctrs_0.4.1            ggmosaic_0.3.3
##  [37] abind_1.4-5            cachem_1.0.6           withr_2.5.0
##  [40] ggforce_0.3.3          RcmdrMisc_2.7-2        robustbase_0.95-0
##  [43] checkmate_2.1.0        getopt_1.20.3          cluster_2.1.3
##  [46] ape_5.6-2              lazyeval_0.2.2         crayon_1.5.1
##  [49] ellipse_0.4.3          genefilter_1.78.0      glmnet_4.1-4
##  [52] pkgconfig_2.0.3        labeling_0.4.2         tweenr_1.0.2
##  [55] nlme_3.1-157           Rcmdr_2.8-0            nnet_7.3-17
##  [58] rlang_1.0.4            lifecycle_1.0.1        sandwich_3.0-2
##  [61] productplots_0.1.1     doSNOW_1.0.20          cellranger_1.1.0
##  [64] polyclip_1.10-0        Matrix_1.4-1           carData_3.0-5
##  [67] lpsymphony_1.24.0      Rhdf5lib_1.18.2        boot_1.3-28
##  [70] zoo_1.8-10             base64enc_0.1-3        pheatmap_1.0.12
##  [73] png_0.1-7              viridisLite_0.4.1      bitops_1.0-7
##  [76] pROC_1.18.0            KernSmooth_2.23-20     rhdf5filters_1.8.0
##  [79] Biostrings_2.64.0      blob_1.2.3             shape_1.4.6
##  [82] jpeg_0.1-9             rstatix_0.7.0          ggsignif_0.6.3
##  [85] scales_1.2.1           memoise_2.0.1          magrittr_2.0.3
##  [88] gplots_3.1.3           zlibbioc_1.42.0        compiler_4.2.1
##  [91] lme4_1.1-30            cli_3.3.0              ade4_1.7-19
##  [94] XVector_0.36.0         htmlTable_2.4.1        formatR_1.12
##  [97] Formula_1.2-4          mgcv_1.8-40            tidyselect_1.1.2
## [100] stringi_1.7.8          tcltk2_1.2-11          highr_0.9
## [103] mitools_2.4            yaml_2.3.5             locfit_1.5-9.6
## [106] latticeExtra_0.6-30    ggrepel_0.9.1          tools_4.2.1
## [109] parallel_4.2.1         rstudioapi_0.13        foreach_1.5.2
## [112] foreign_0.8-82         optparse_1.7.3         farver_2.1.1
## [115] digest_0.6.29          nortest_1.0-4          Rcpp_1.0.9
## [118] car_3.1-0              broom_1.0.1            httr_1.4.3
## [121] AnnotationDbi_1.58.0   effects_4.2-2          Wrench_1.14.0
## [124] colorspace_2.0-3       XML_3.99-0.10          splines_4.2.1
## [127] graphlayouts_0.8.0     multtest_2.52.0        xtable_1.8-4
## [130] jsonlite_1.8.0         nloptr_2.0.3           futile.options_1.0.1
## [133] tidygraph_1.2.1        corpcor_1.6.10         R6_2.5.1
## [136] Hmisc_4.7-0            pillar_1.8.1           htmltools_0.5.3
## [139] glue_1.6.2             fastmap_1.1.0          minqa_1.2.4
## [142] BiocParallel_1.30.3    class_7.3-20           codetools_0.2-18
## [145] pcaPP_2.0-2            mvtnorm_1.1-3          utf8_1.2.2
## [148] tibble_3.1.8           gtools_3.9.3           biglm_0.9-2.1
## [151] interp_1.1-3           metagenomeSeq_1.38.0   survival_3.3-1
## [154] limma_3.52.2           rmarkdown_2.14         biomformat_1.24.0
## [157] munsell_0.5.0          e1071_1.7-11           rhdf5_2.40.0
## [160] GenomeInfoDbData_1.2.8 iterators_1.0.14       haven_2.5.0
## [163] gtable_0.3.1
```

This document was processed on:

```
Sys.Date()
```

```
## [1] "2022-09-06"
```
