# Supplementary material for: Association of general health and lifestyle factors with the salivary microbiota – Lessons learned from the ADDITION-PRO cohort
Source: Front Cell Infect Microbiol. 2022 Nov 16;12:1055117. doi: 10.3389/fcimb.2022.1055117 (PMC9709502; doi:10.3389/fcimb.2022.1055117)
Supplement: Supplementary Figure 1 — Comparison of sequencing batches. Bray-Curtis dissimilarity calculated from Hellinger transformed total sum scaled data was used as beta-diversity measure and visualized with principal coordinate analysis (PCoA). [file DataSheet_1.zip › Supplementary_file_5_PERMANOVA_continuous_variables.docx]

|  | **All variables** | | **Select variables** | |
| --- | --- | --- | --- | --- |
| **Variable** | **R2** | **P (>F)** | **R2** | **P (>F)** |
| Sex | 0.003616 | 0.021 | 0.004018 | 0.012 |
| BMI | 0.002937 | 0.047 | 0.003153 | 0.03 |
| Waist | 0.002987 | 0.051 | 0.004555 | 0.003 |
| Age at follow-up | 0.005193 | 0.003 | 0.00658 | 0.001 |
| Smoking | 0.02922 | 0.001 | 0.030585 | 0.001 |
| Weekly alcohol intake | 0.00292 | 0.058 | 0.004008 | 0.009 |
| Diabetes risk | 0.003309 | 0.398 | - | - |
| Systolic blood pressure | 9.96e^-13^ | 0.519 | - | - |
| Diastolic blood pressure | 4.81e^-12^ | 0.501 | - | - |
| Heart rate | 0.001431 | 0.538 | - | - |
| Pulse pressure | -3.4e^-16^ |  | - | - |
| Triglycerides | 0.001098 | 0.761 | - | - |
| HDL cholesterol | 0.000996 | 0.832 | - | - |
| LDL cholesterol | 0.001068 | 0.769 | - | - |
| Total cholesterol | 0.001043 | 0.795 | - | - |
| Activity level | 0.001395 | 0.512 | - | - |
| HbA1c | 0.002438 | 0.12 | **-** | **-** |
| Waist-to-height ratio | 0.001517 | 0.469 | - | - |
| Residual | 0.923608 | - | 0,948475 | - |
| Total | 1 | - | 1 | - |

Supplementary file 7. PERMANOVA run of continuous variables.
