# Supplementary figures and images for: Association of general health and lifestyle factors with the salivary microbiota – Lessons learned from the ADDITION-PRO cohort
Source: Front Cell Infect Microbiol. 2022 Nov 16;12:1055117. doi: 10.3389/fcimb.2022.1055117 (PMC9709502; doi:10.3389/fcimb.2022.1055117)

PCoA sequencing batch – PERMANOVA pvalue = 0.451

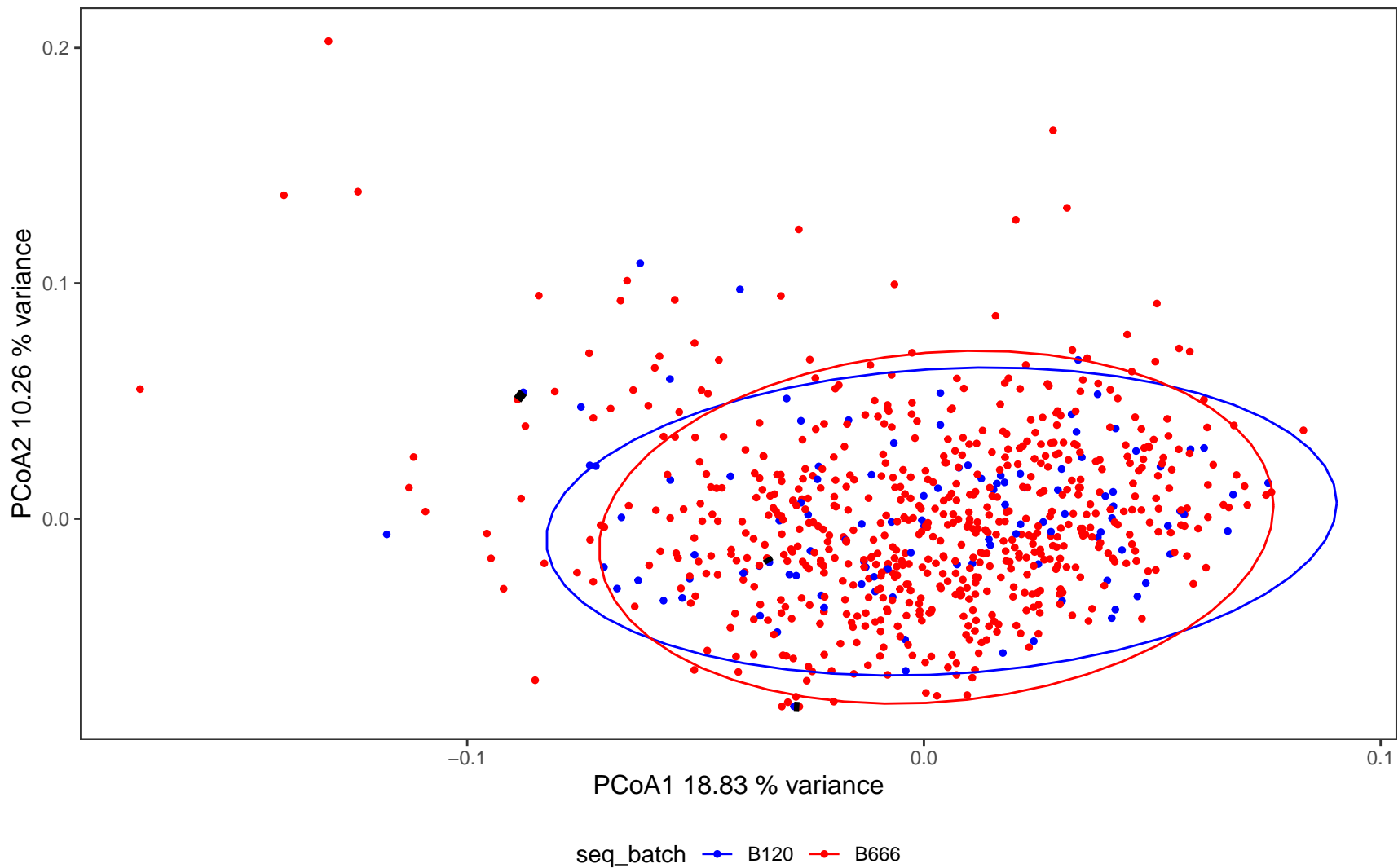

PCoA sequencing batch – PERMANOVA pvalue = 0.451

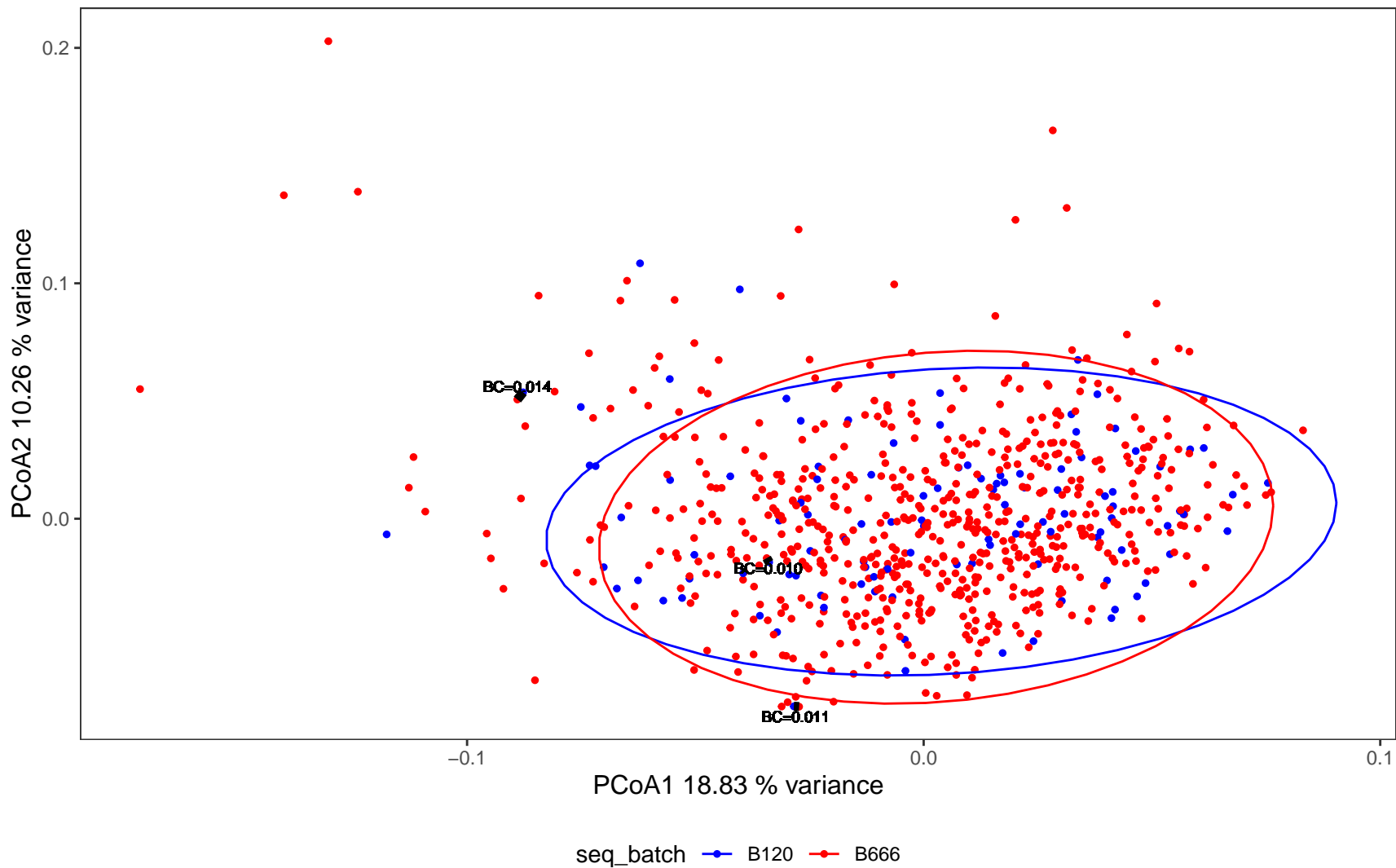

Supplement: Supplementary Figure 1 — Comparison of sequencing batches. Bray-Curtis dissimilarity calculated from Hellinger transformed total sum scaled data was used as beta-diversity measure and visualized with principal coordinate analysis (PCoA). [file DataSheet_1.zip › Supplementary_fig_1_ADDPRO_Microbio_seqbatch_PCoAAll.pdf]

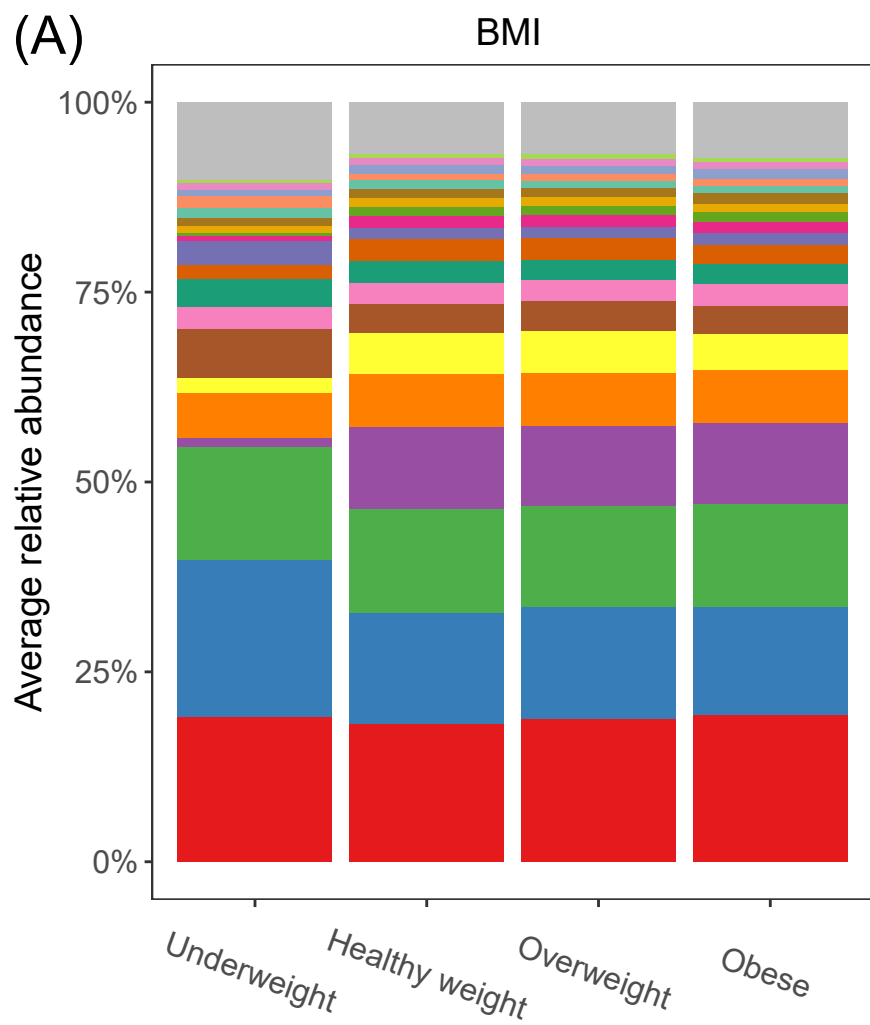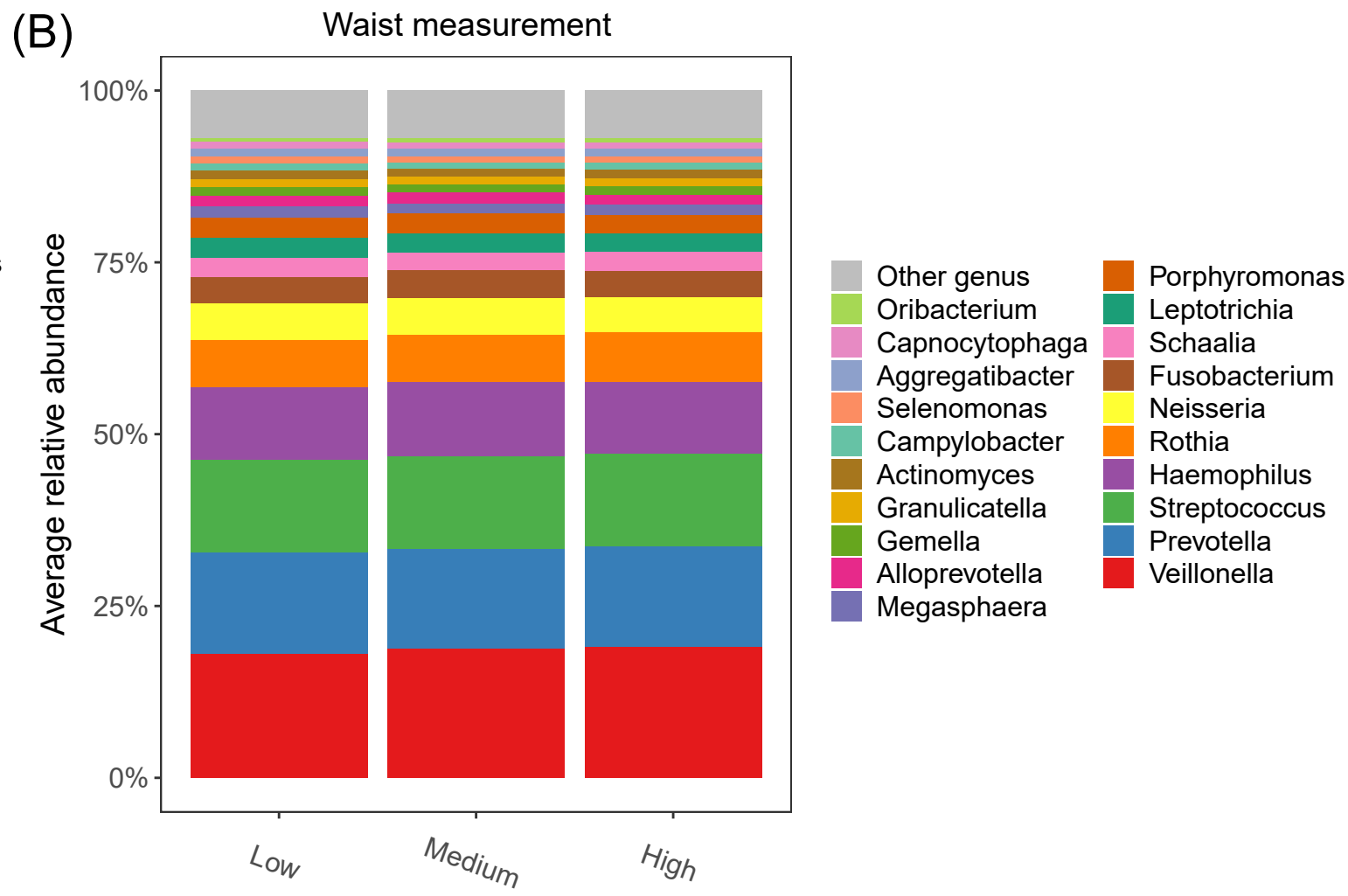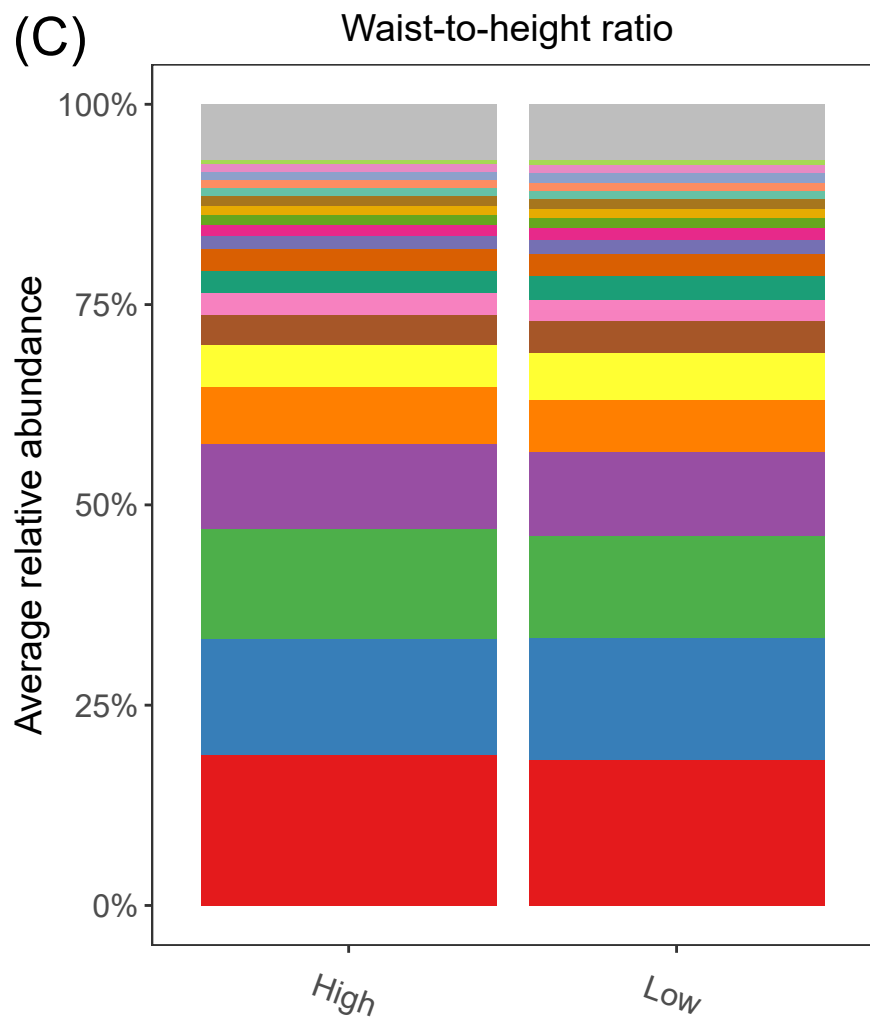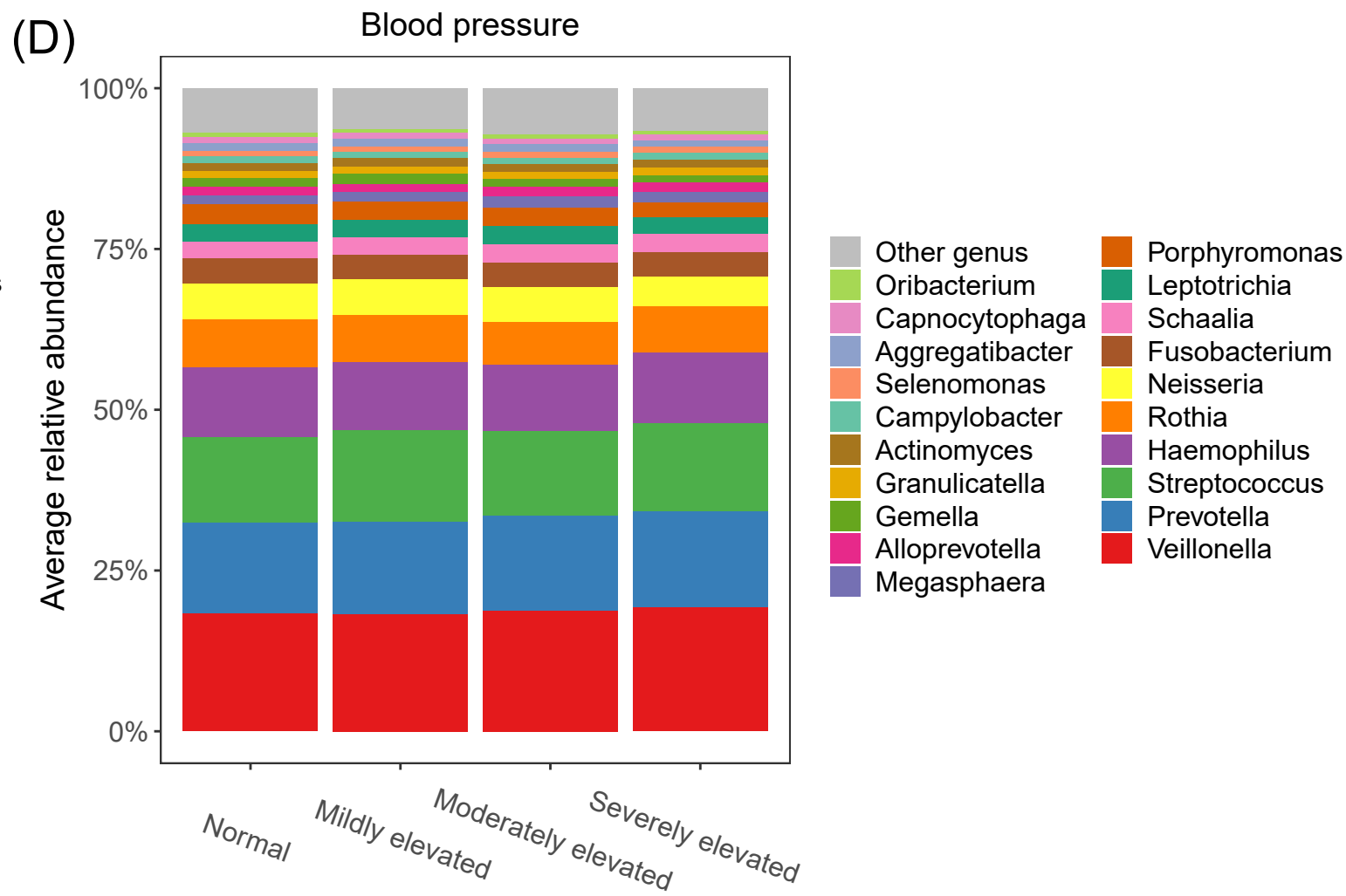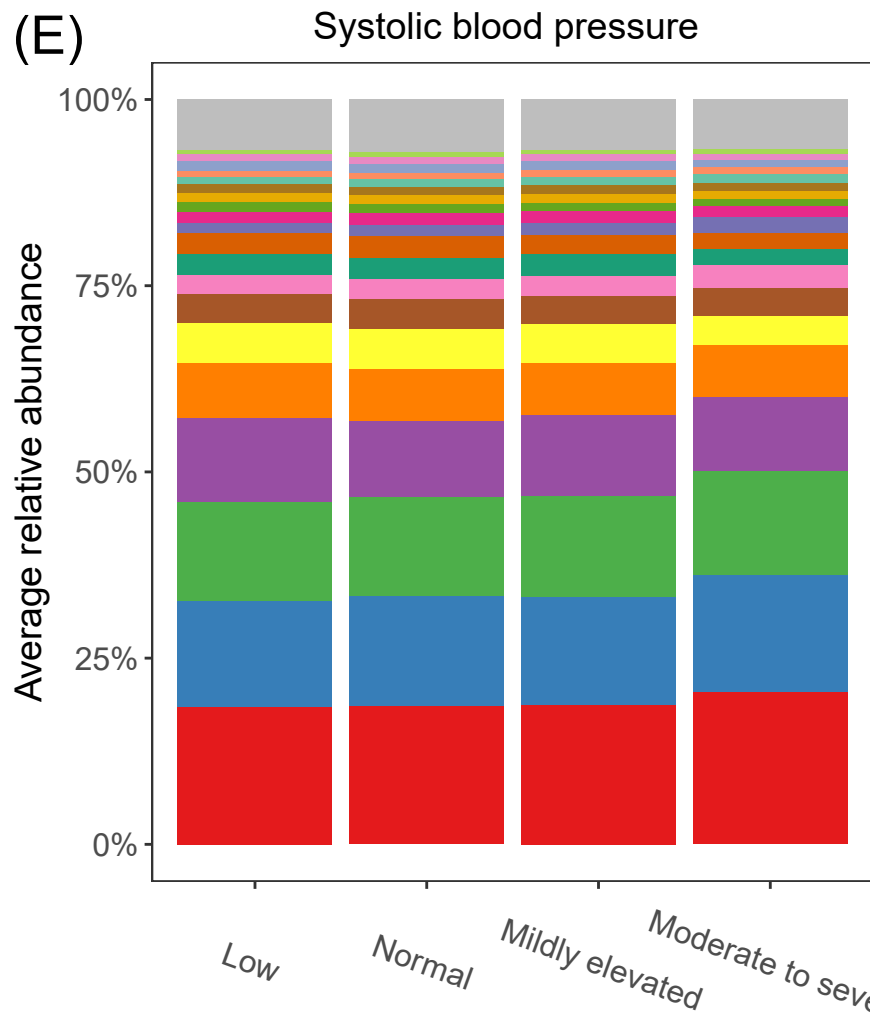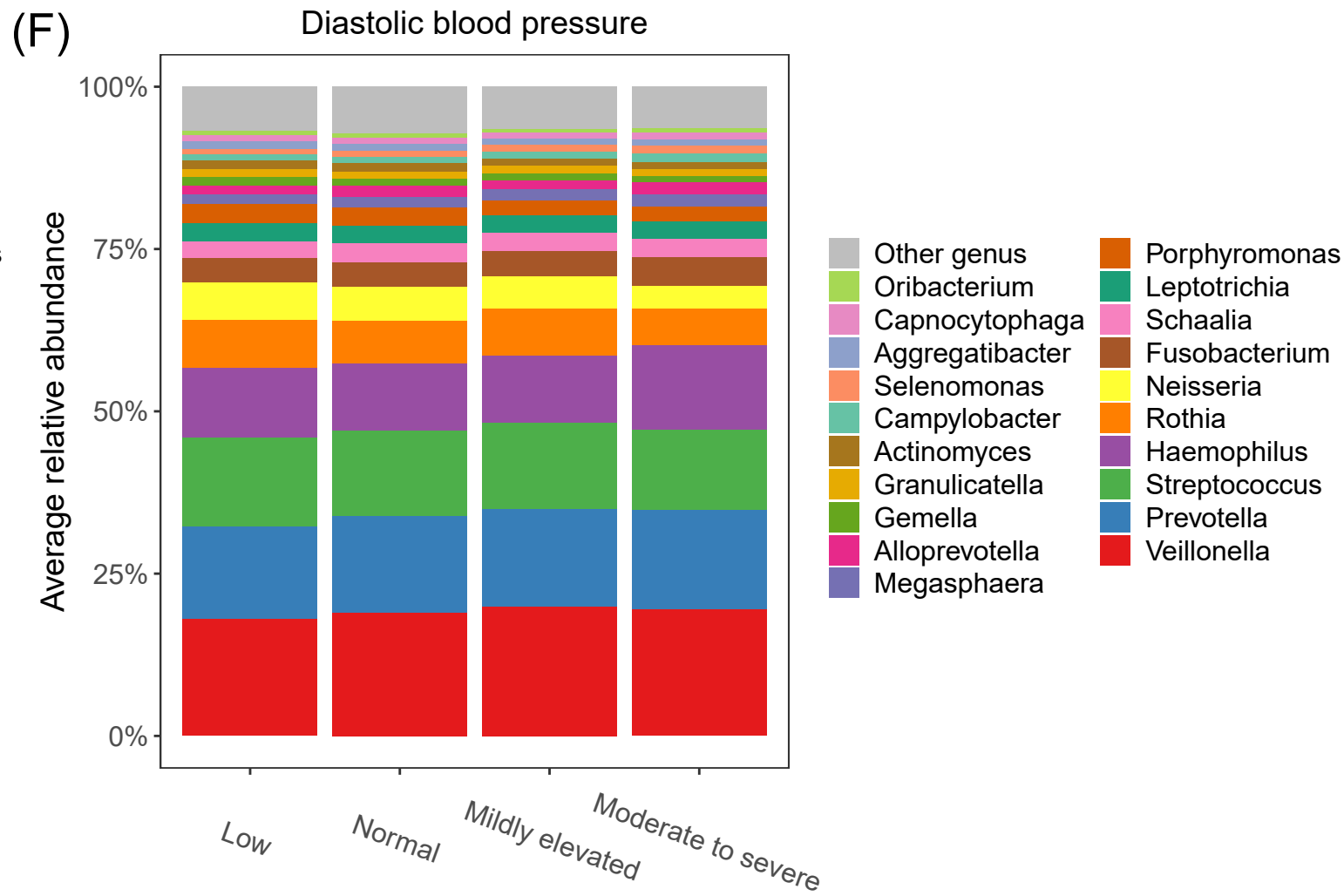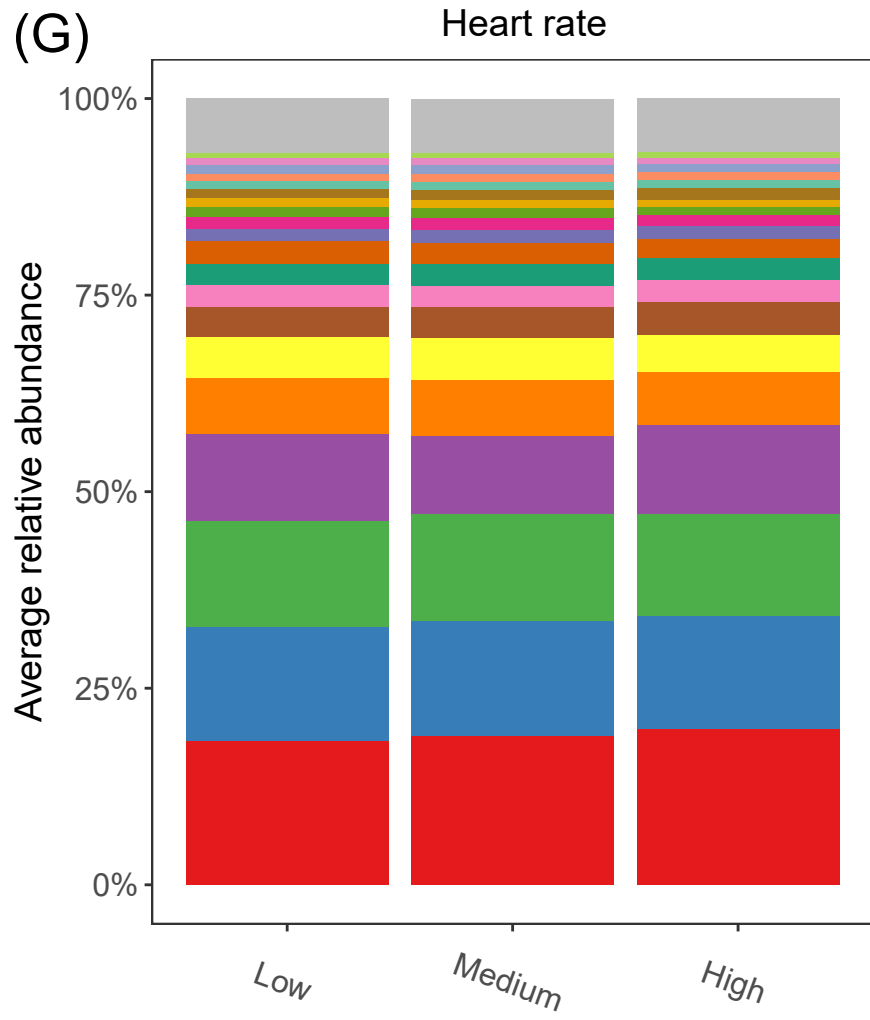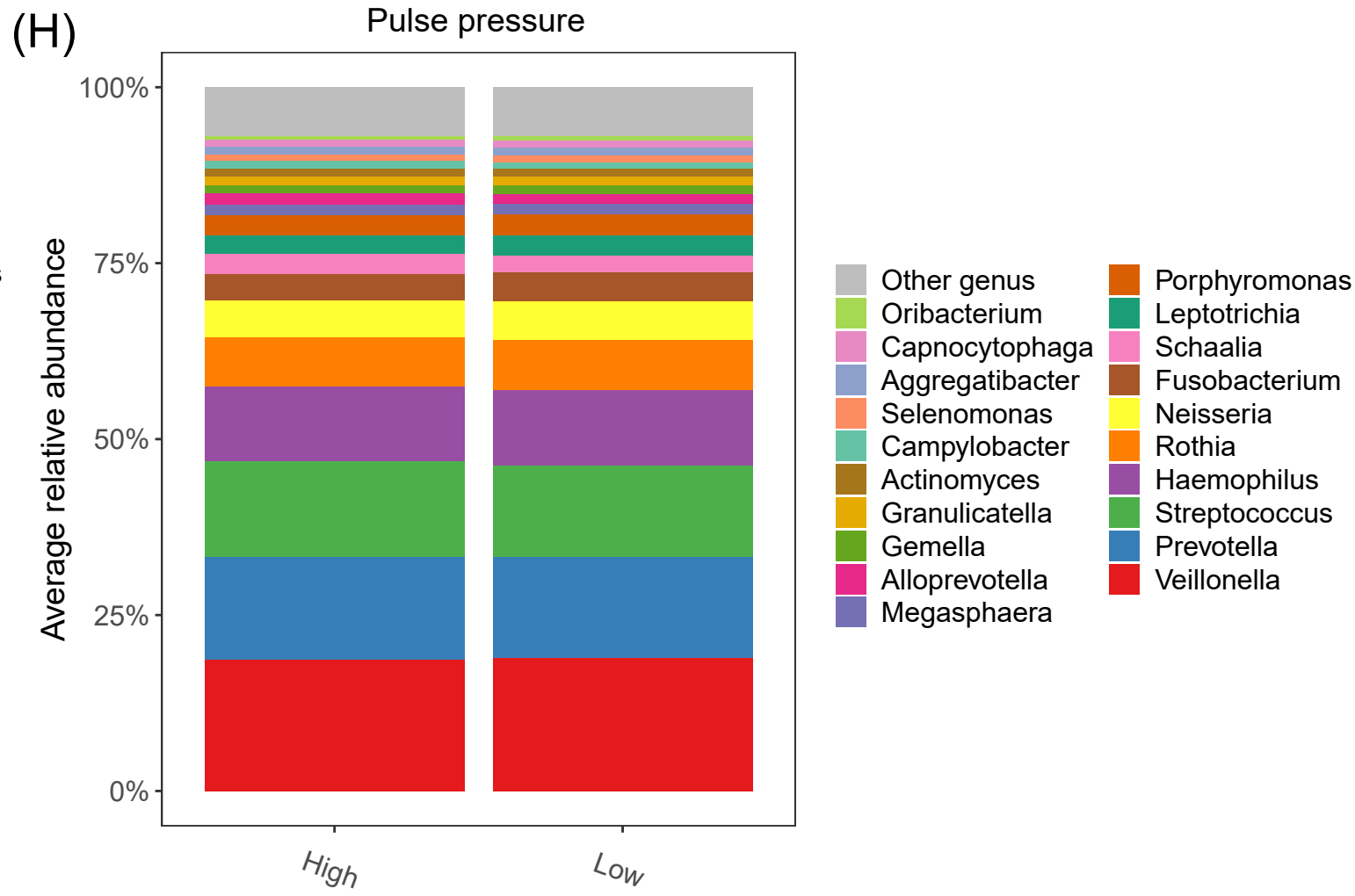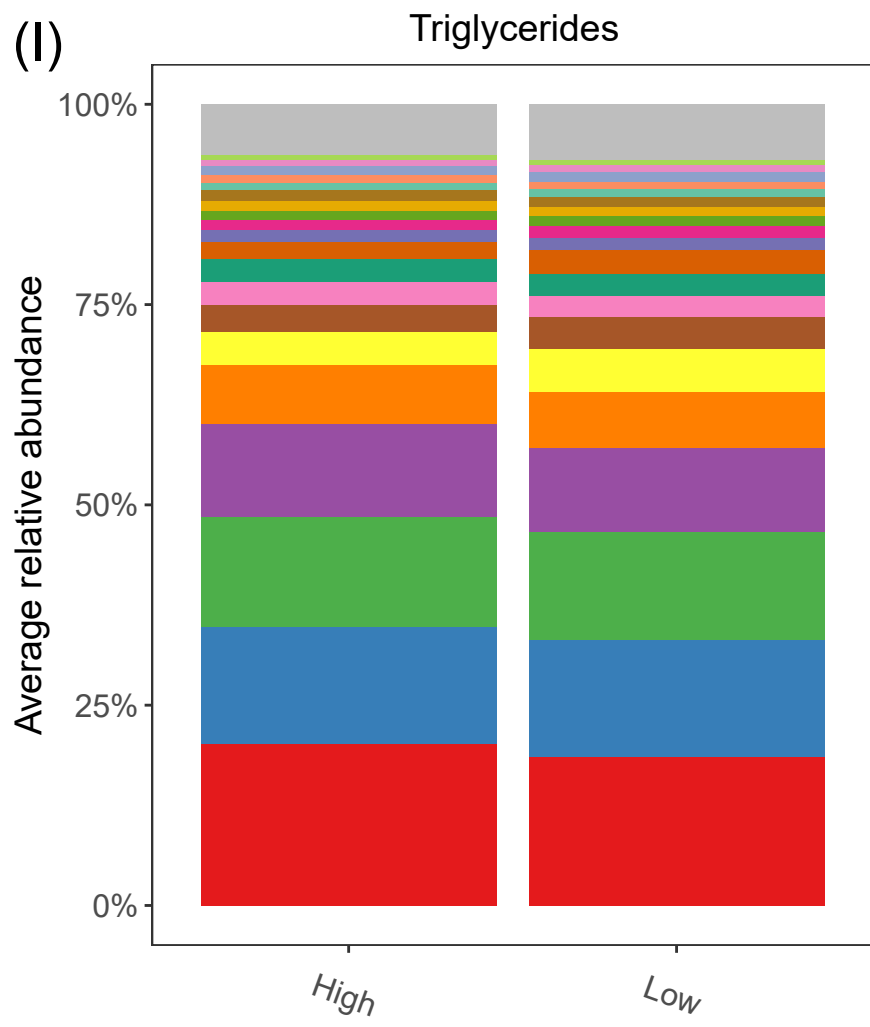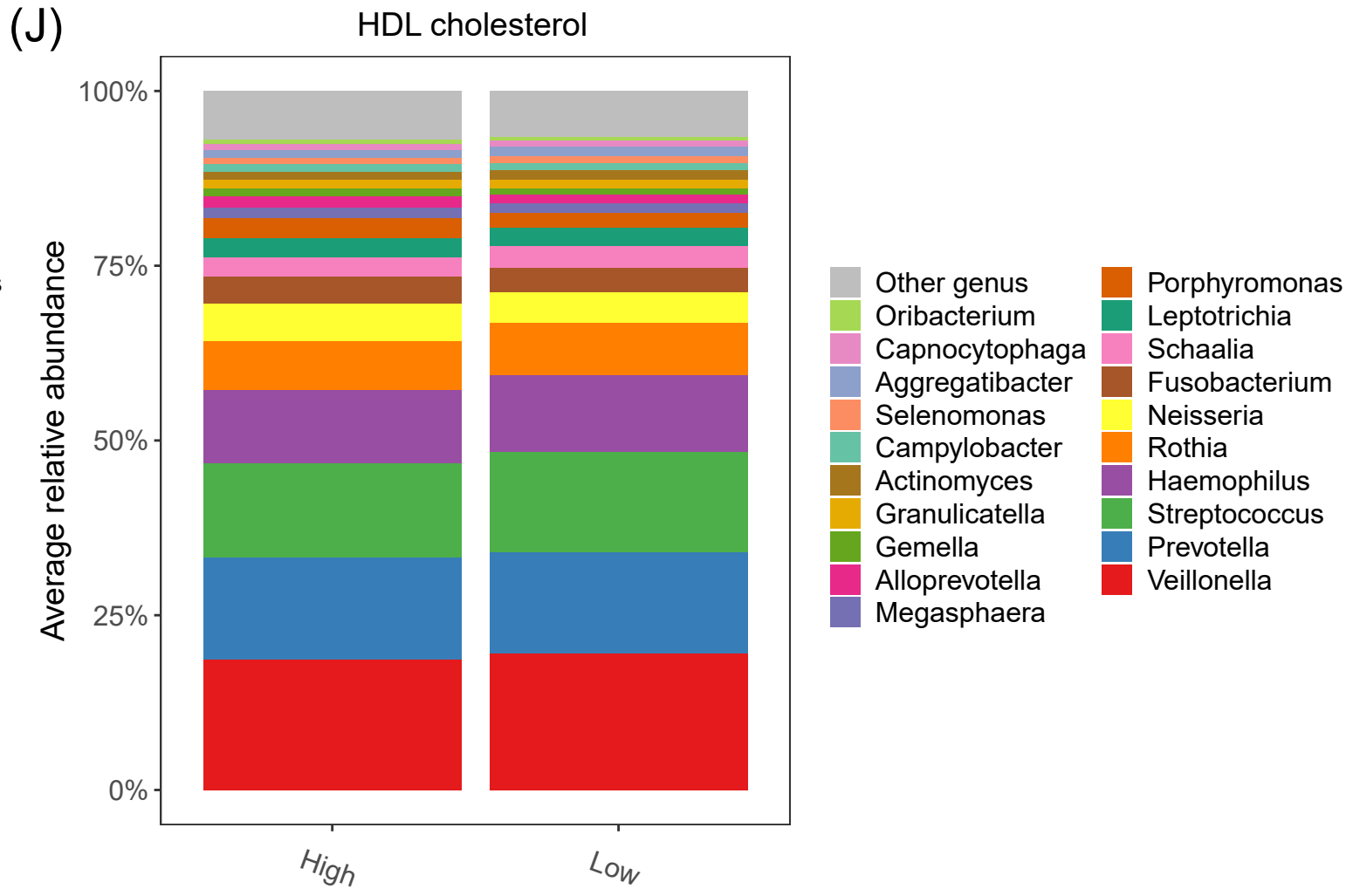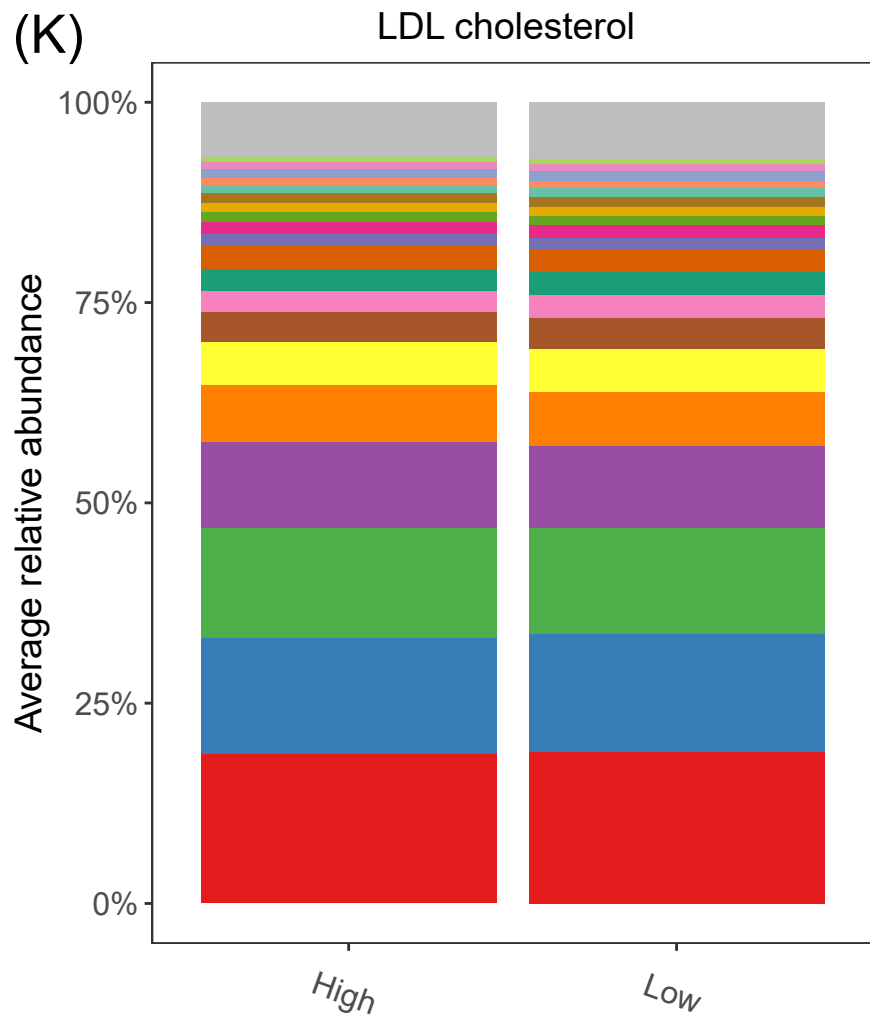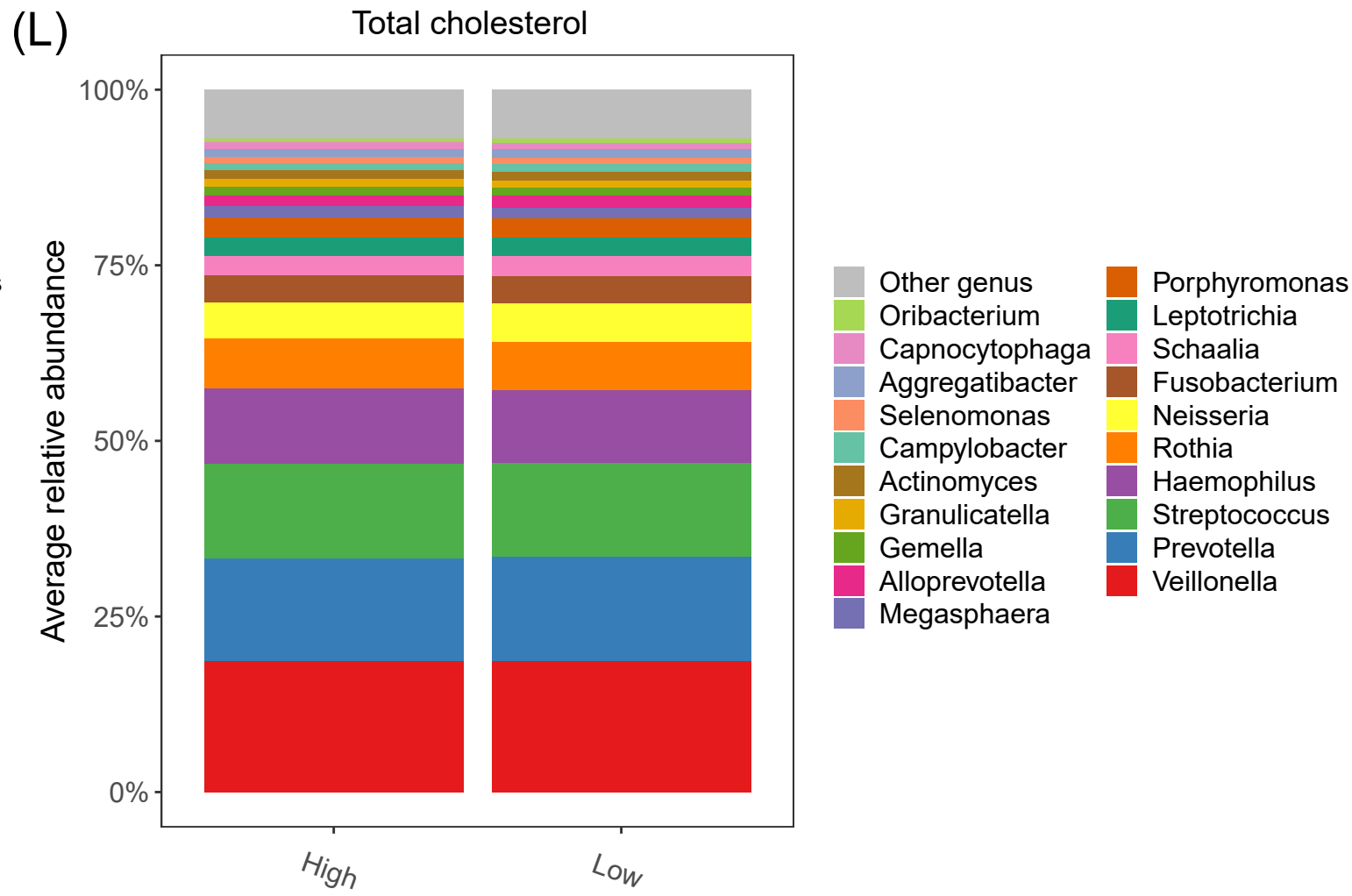

Supplement: Supplementary Figure 1 — Comparison of sequencing batches. Bray-Curtis dissimilarity calculated from Hellinger transformed total sum scaled data was used as beta-diversity measure and visualized with principal coordinate analysis (PCoA). [file DataSheet_1.zip › Supplementary_fig_2_ADDPRO_Stackbar_secondary.pdf]

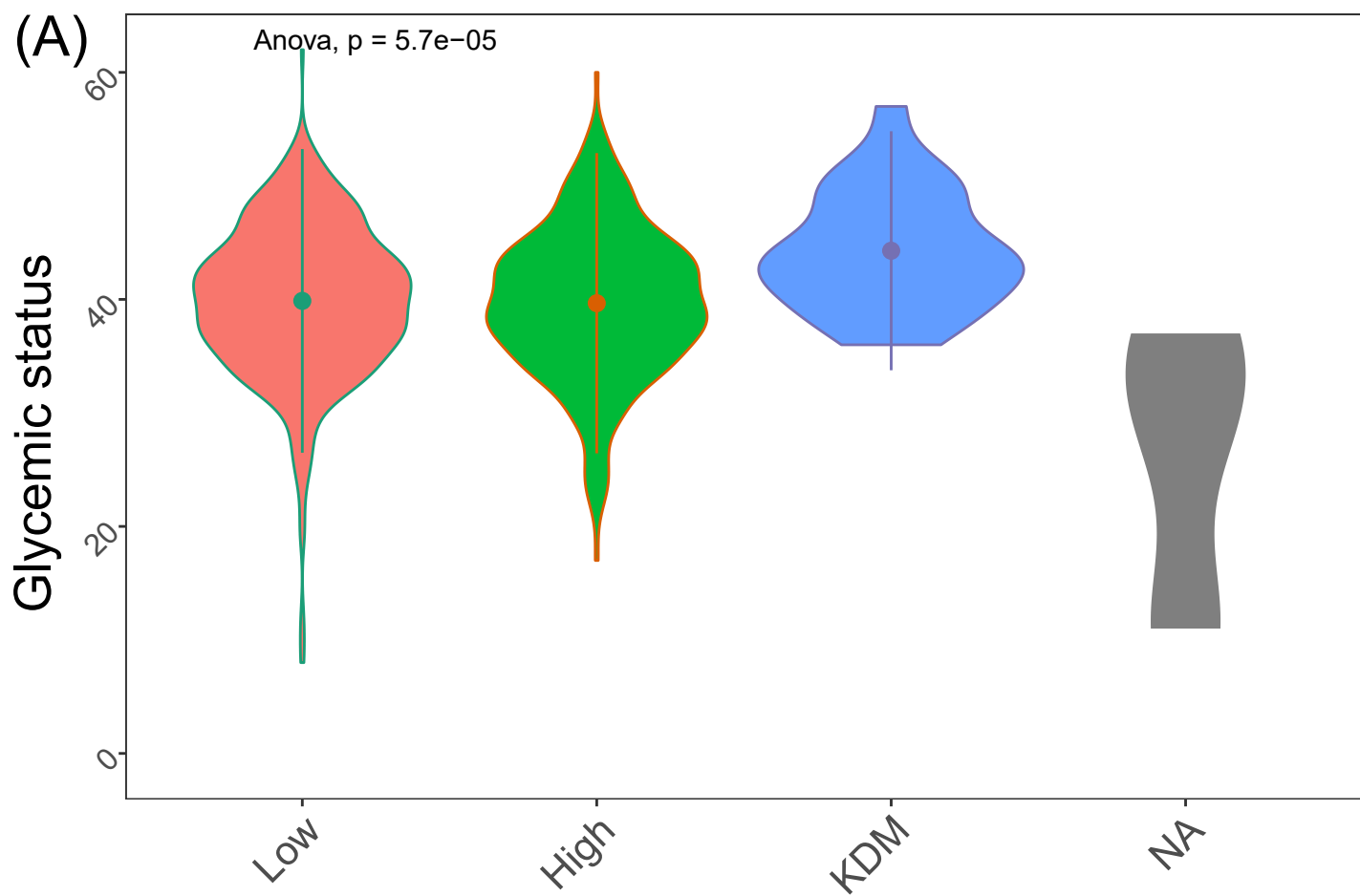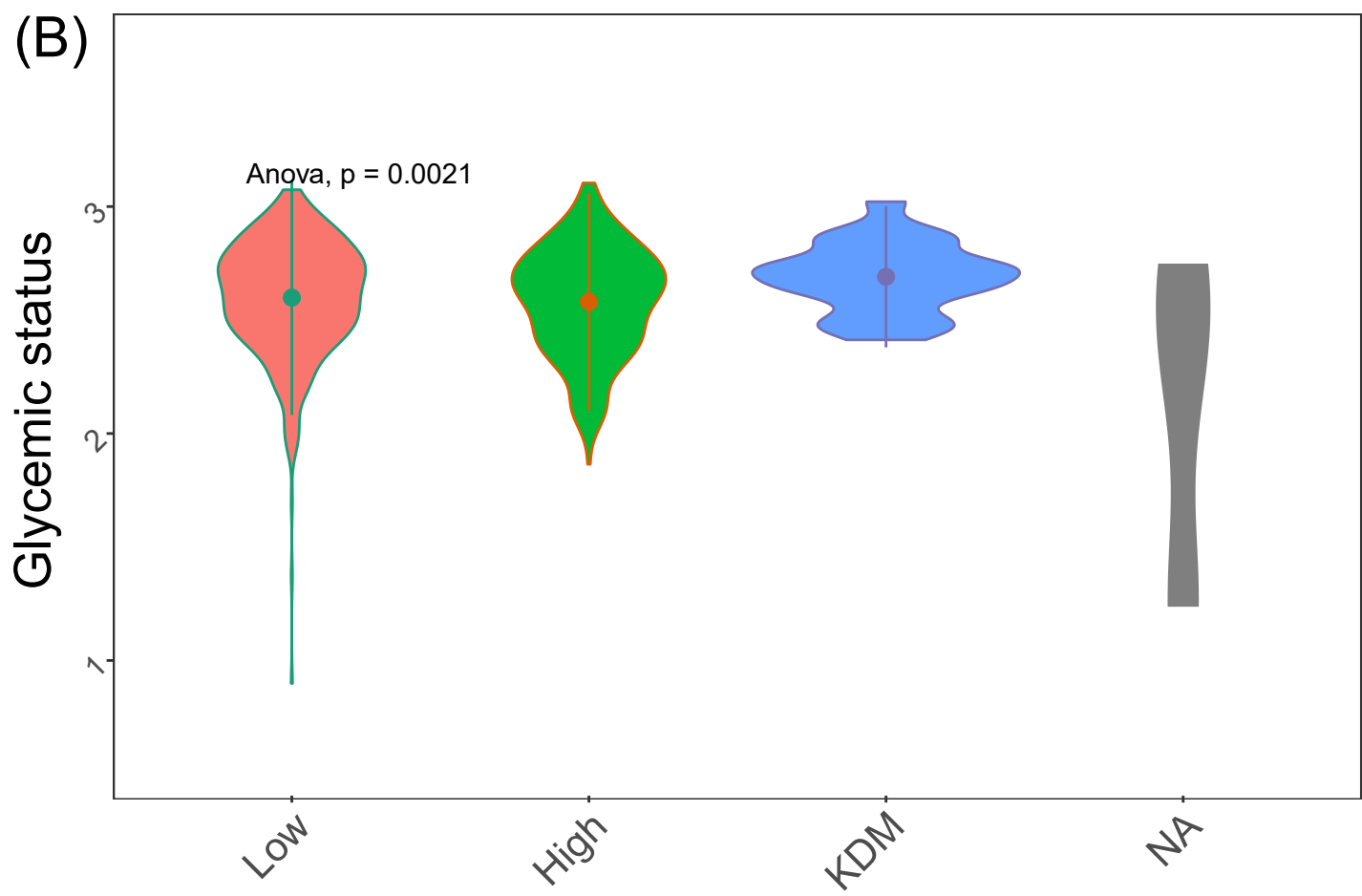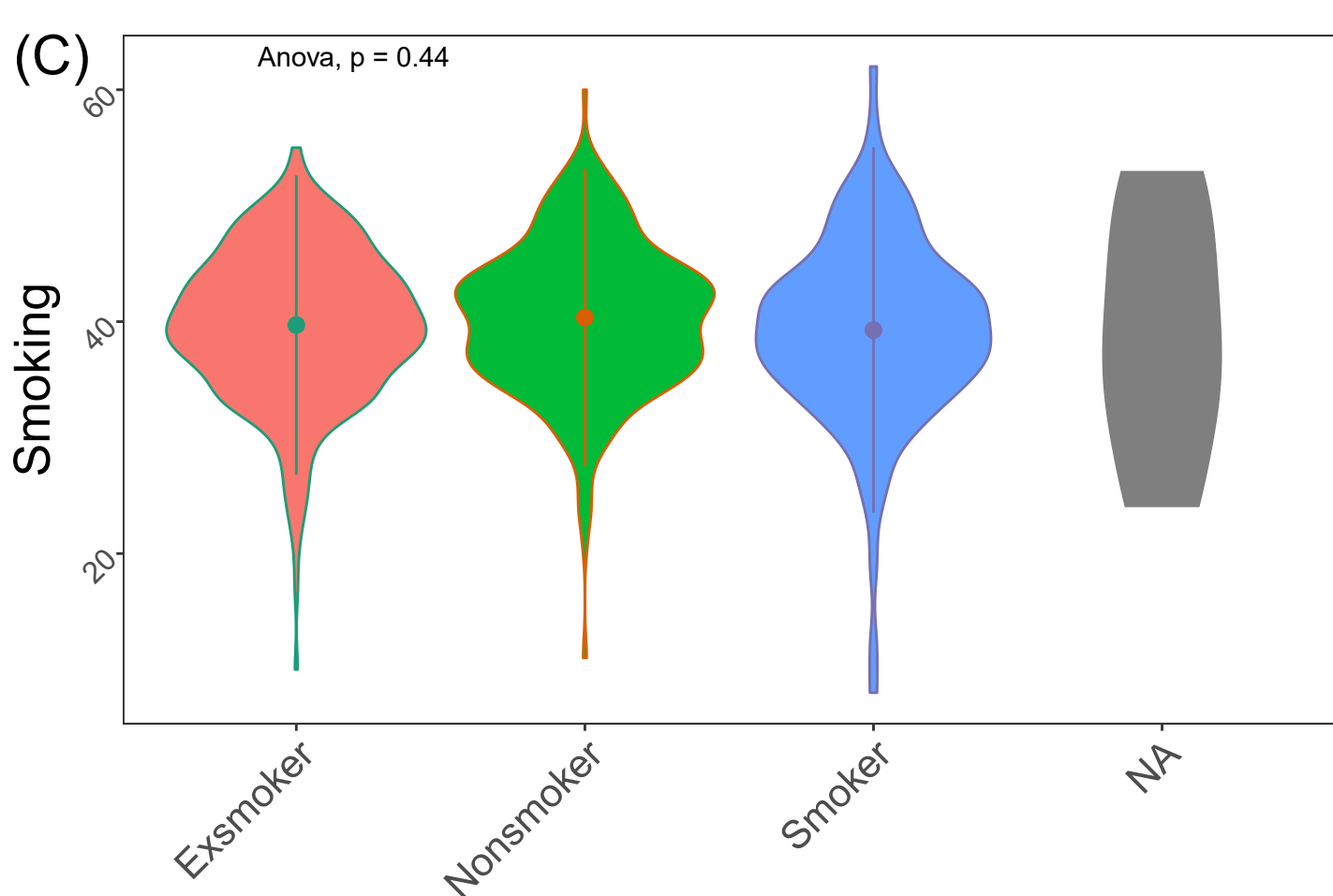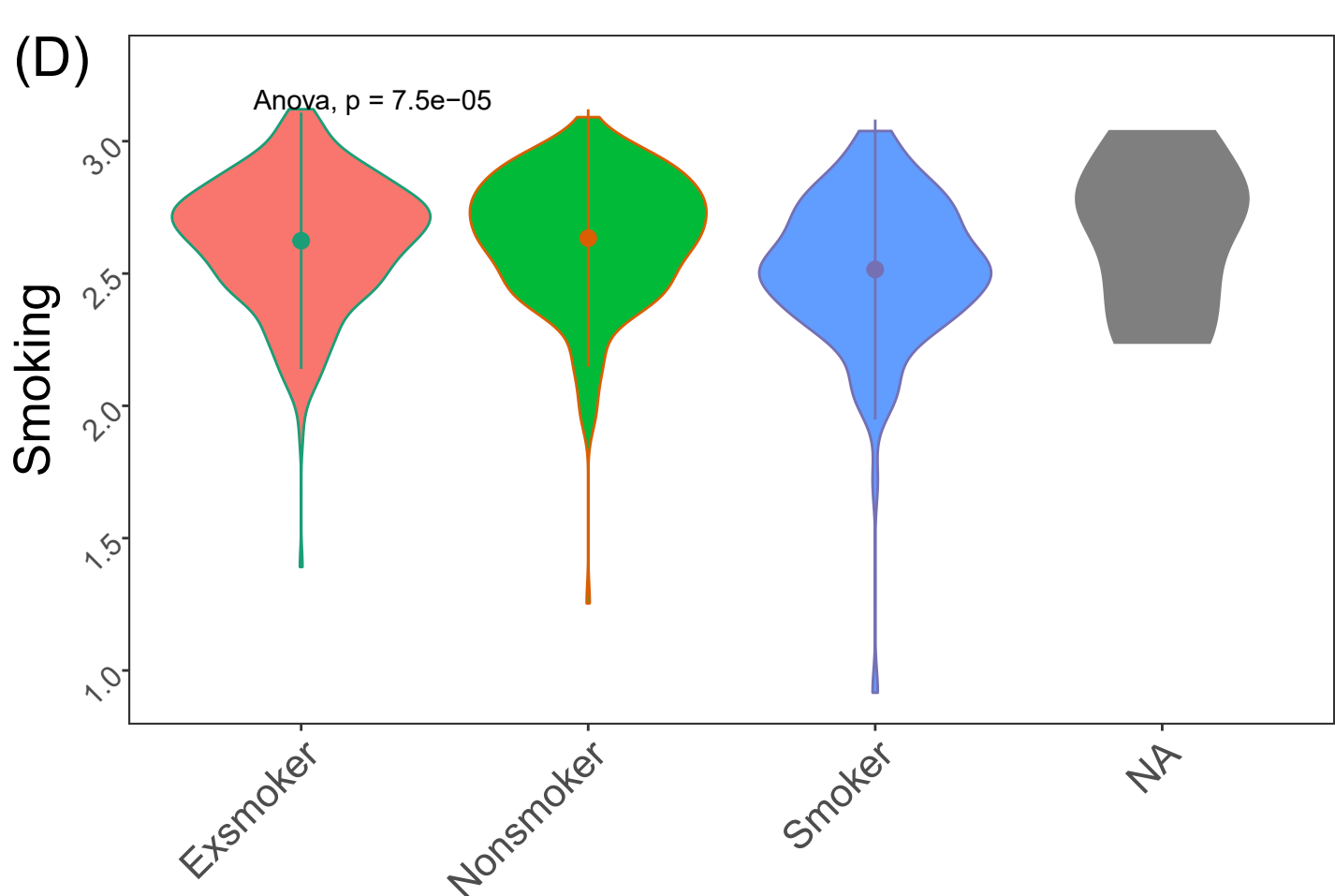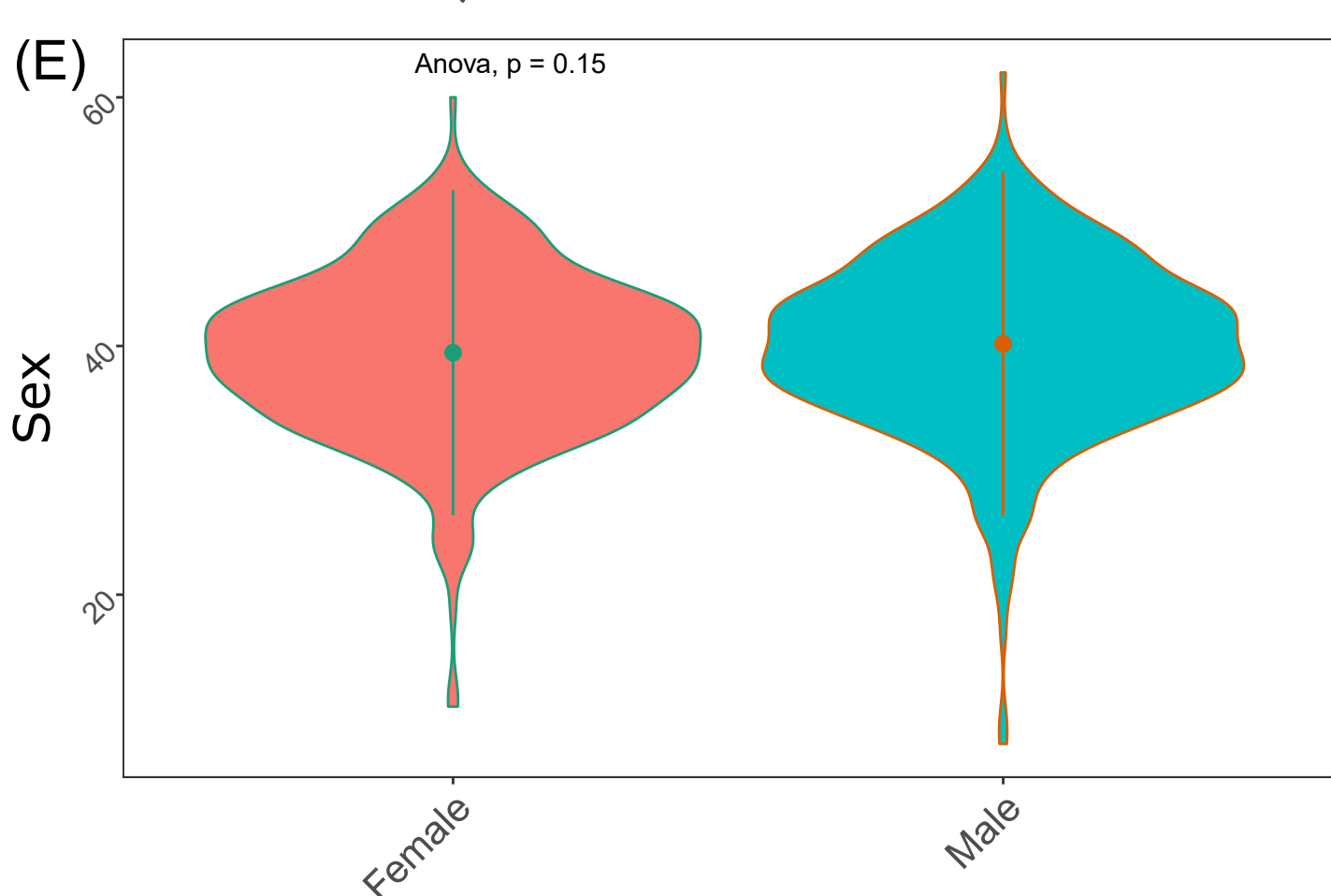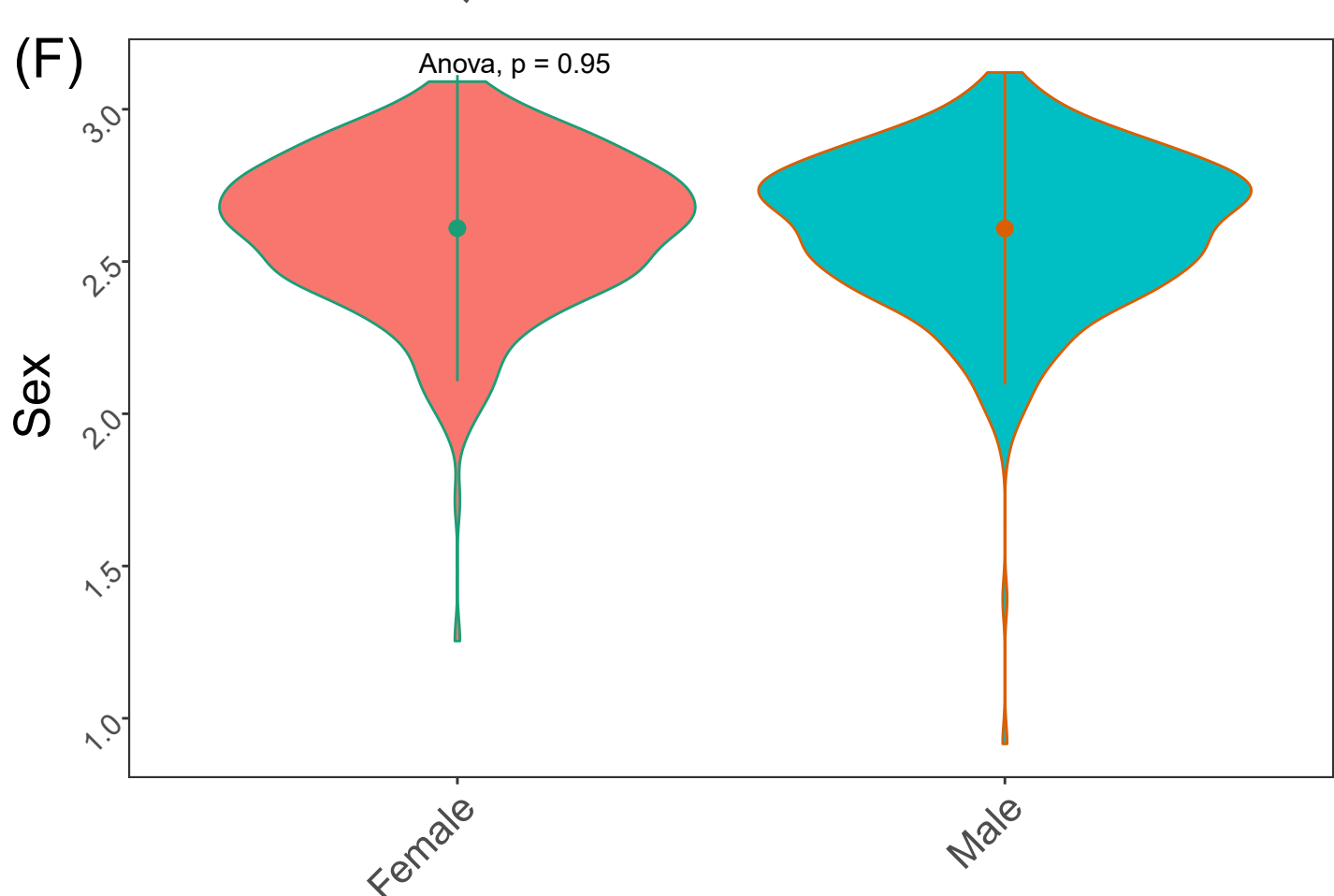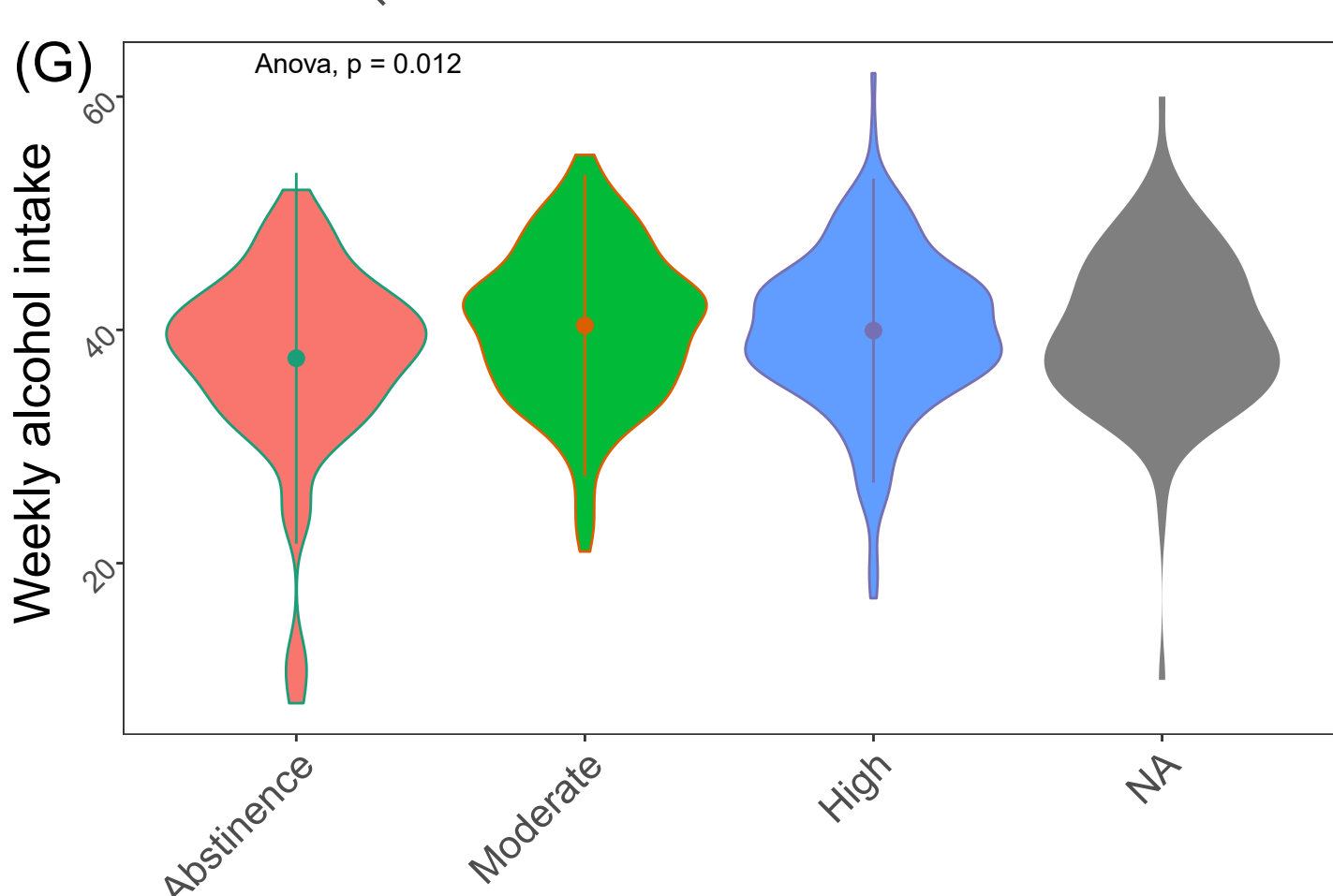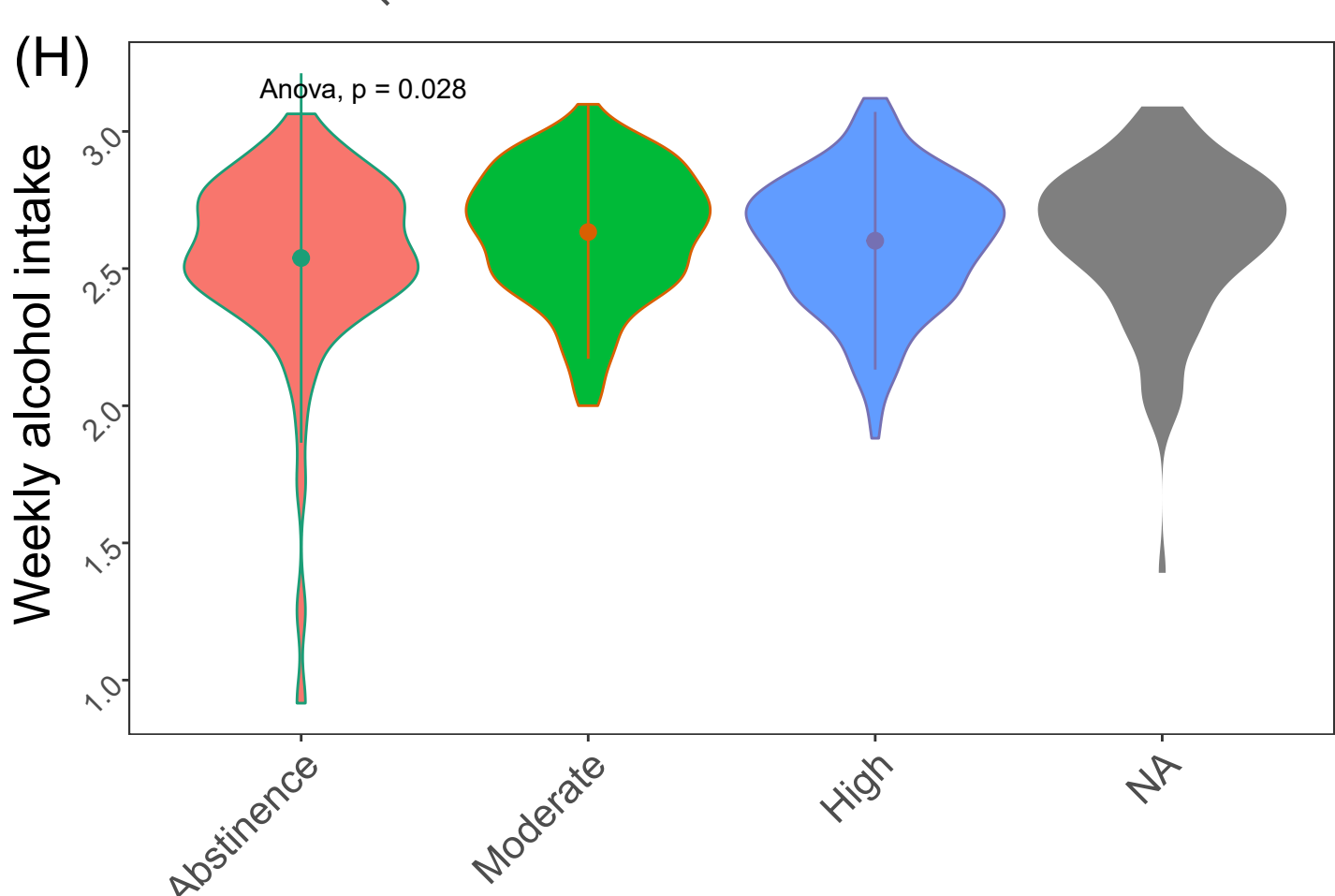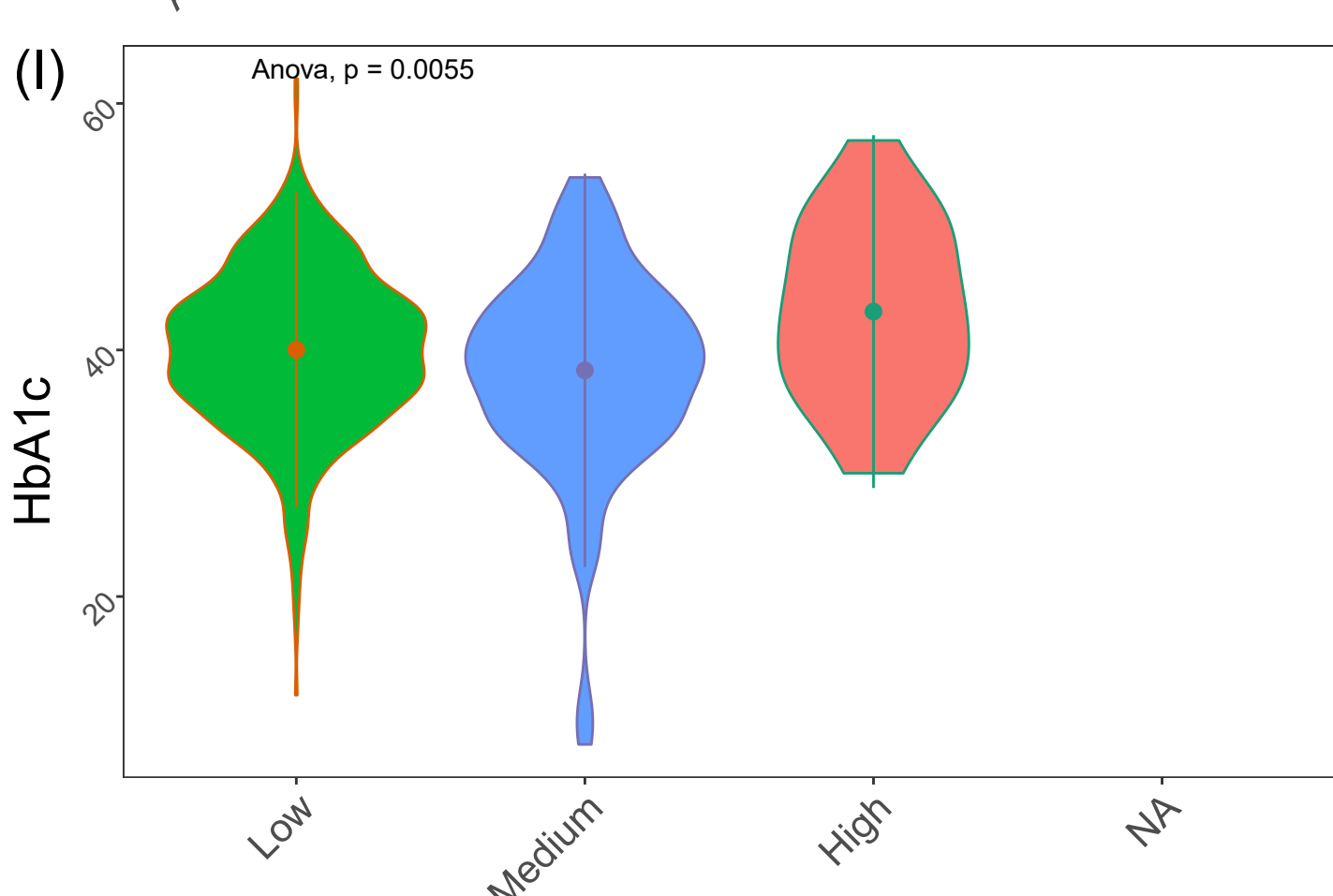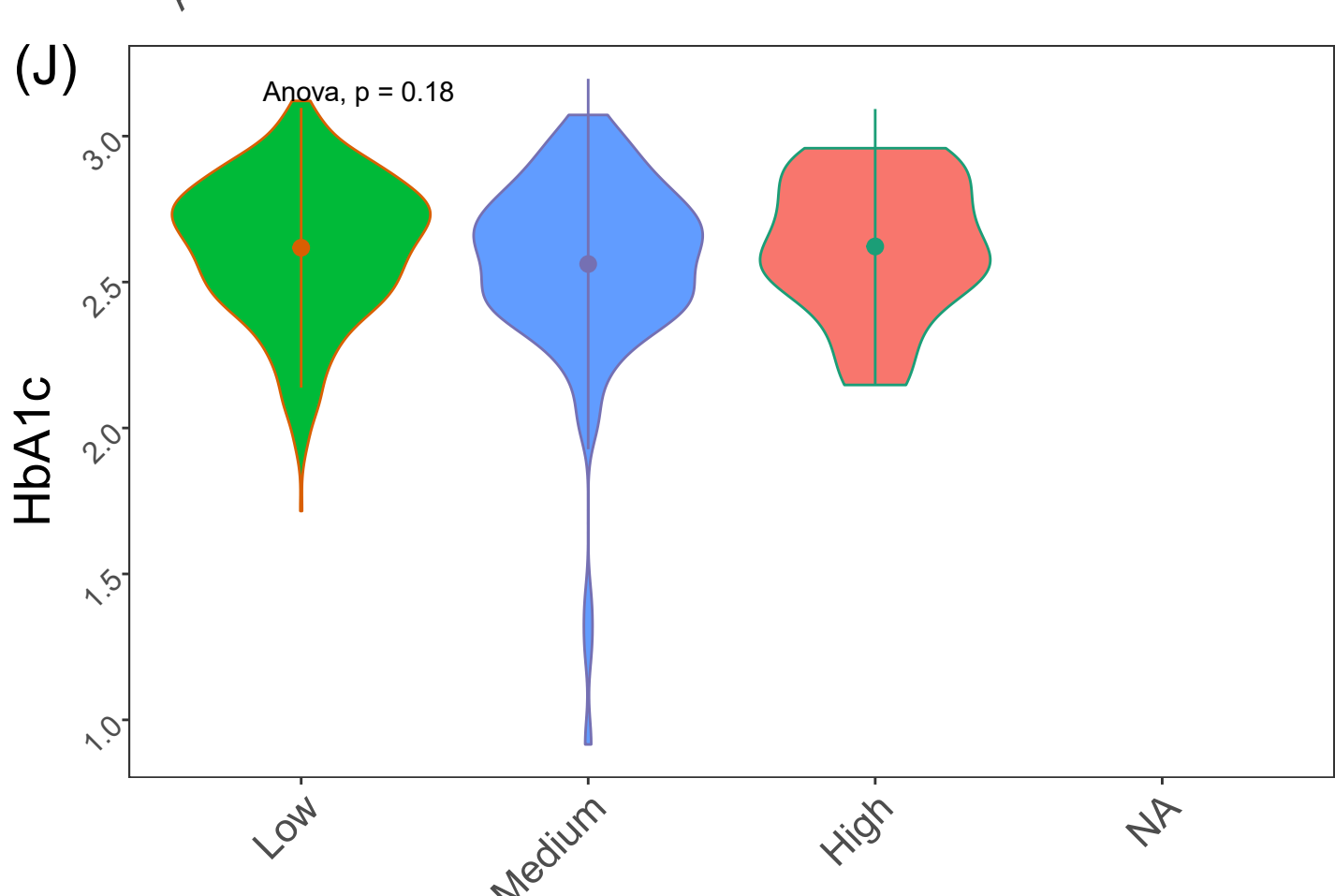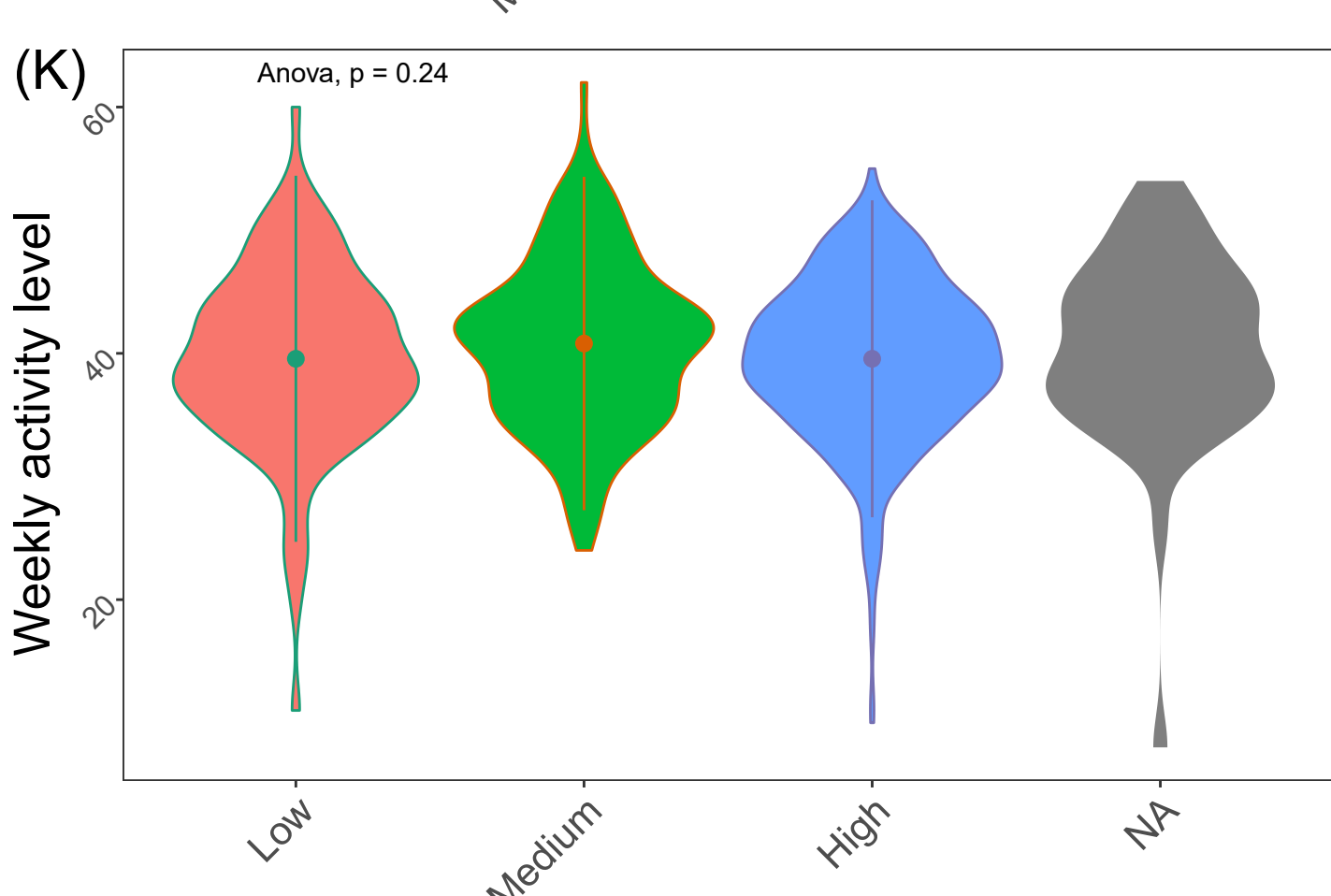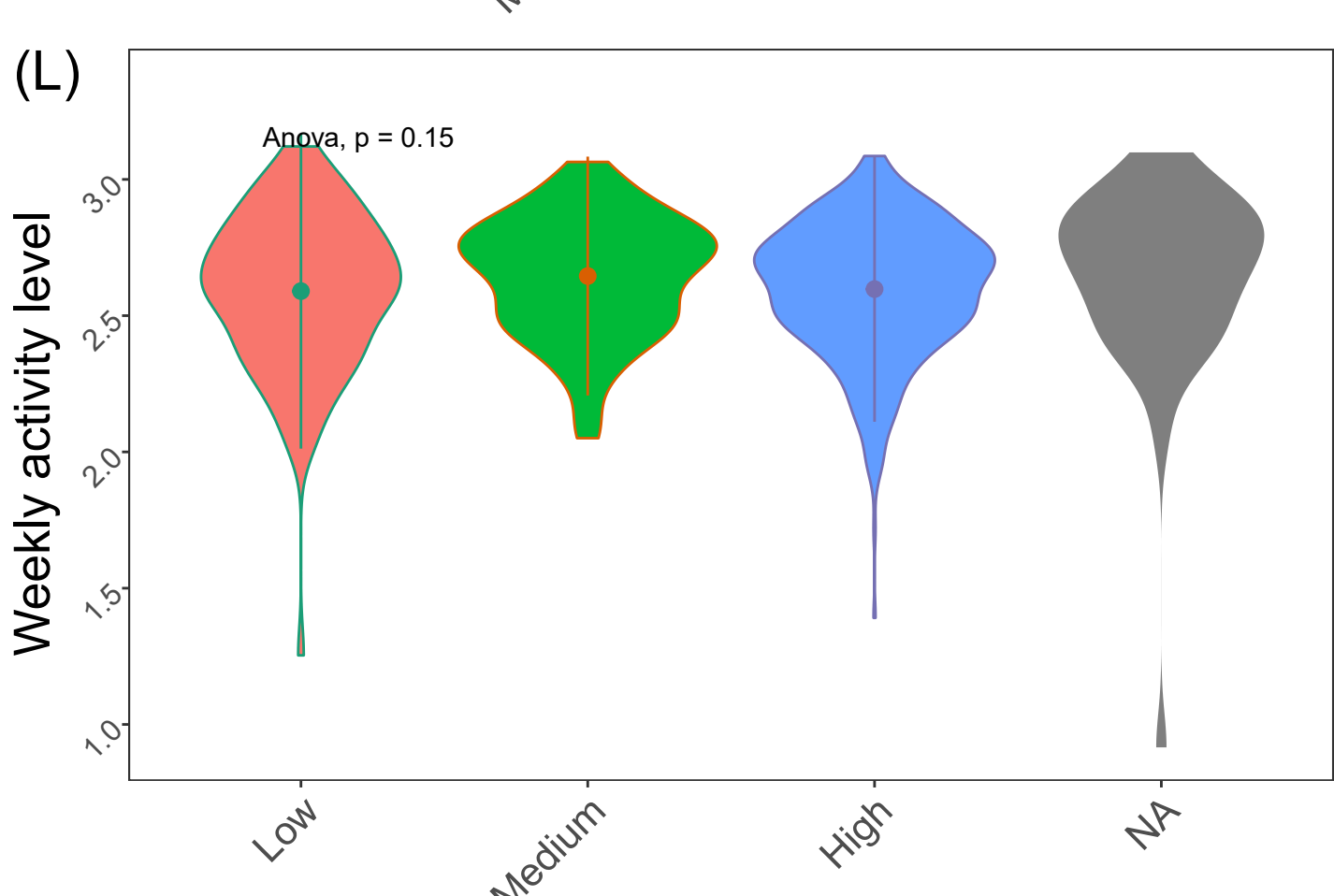

Supplement: Supplementary Figure 1 — Comparison of sequencing batches. Bray-Curtis dissimilarity calculated from Hellinger transformed total sum scaled data was used as beta-diversity measure and visualized with principal coordinate analysis (PCoA). [file DataSheet_1.zip › Supplementary_fig_3_1_ADDPRO_alpha_main.pdf]

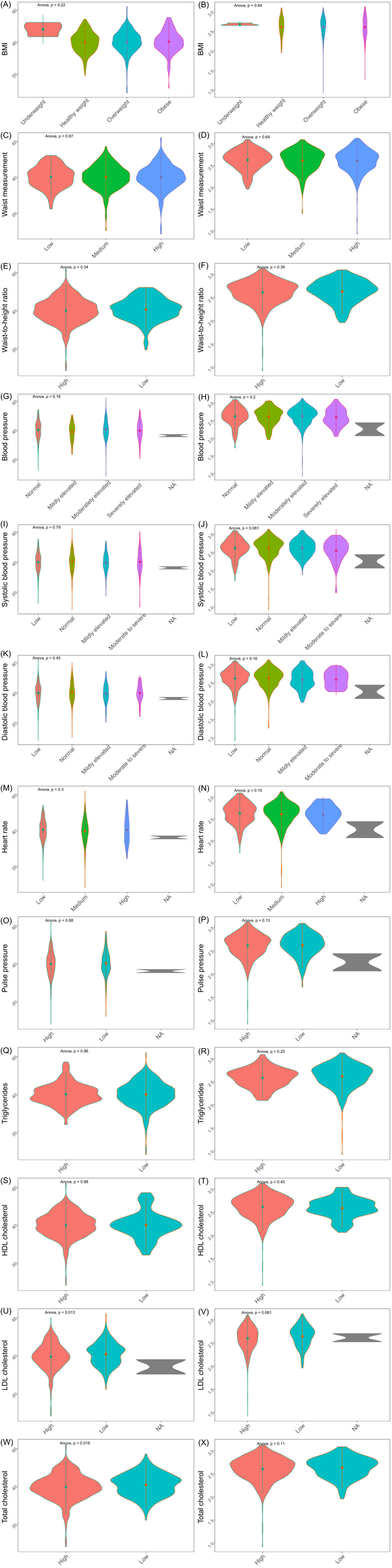

Supplement: Supplementary Figure 1 — Comparison of sequencing batches. Bray-Curtis dissimilarity calculated from Hellinger transformed total sum scaled data was used as beta-diversity measure and visualized with principal coordinate analysis (PCoA). [file DataSheet_1.zip › Supplementary_fig_3_2_ADDPRO_alpha_secondary.pdf]

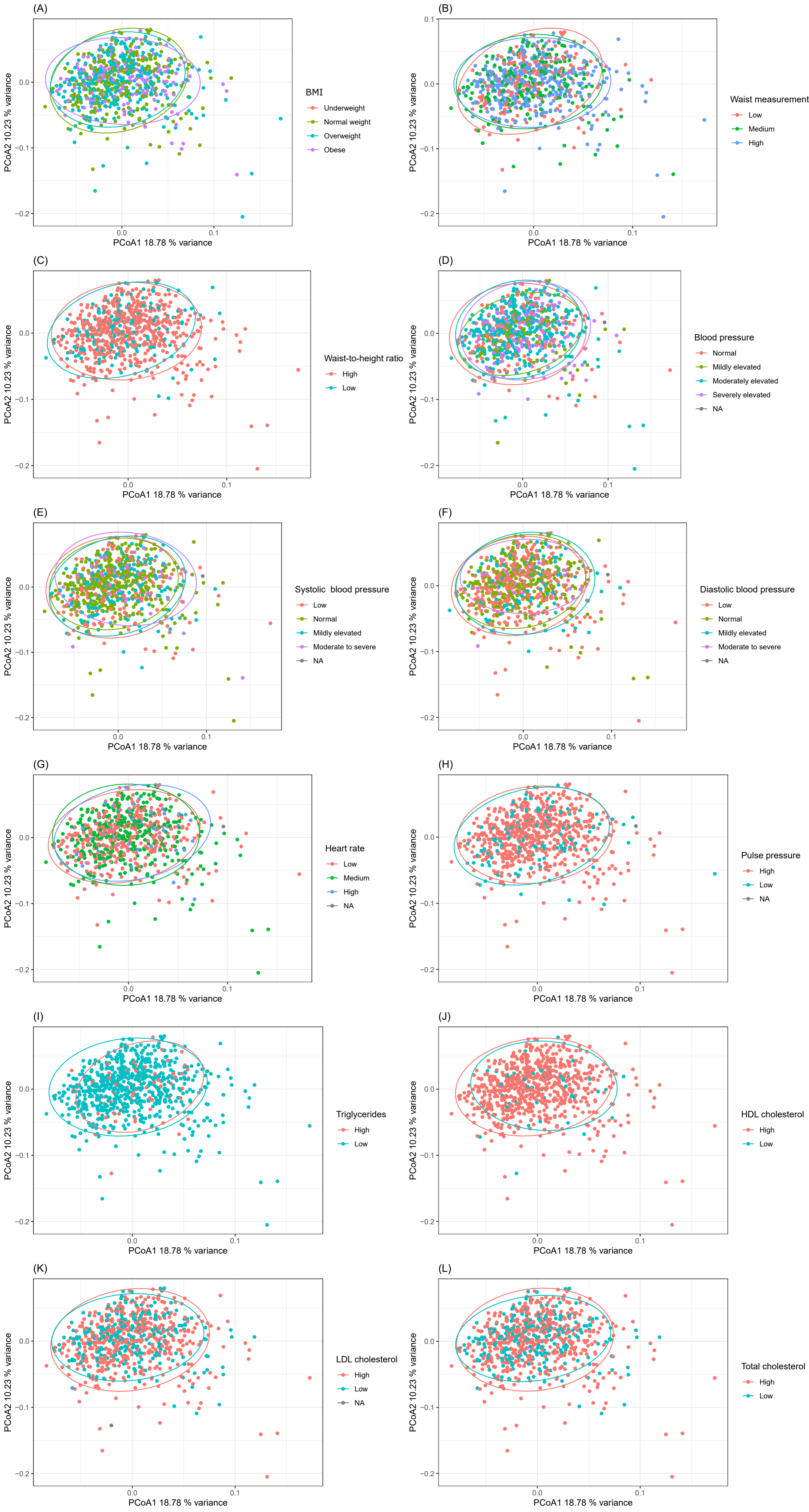

Supplement: Supplementary Figure 1 — Comparison of sequencing batches. Bray-Curtis dissimilarity calculated from Hellinger transformed total sum scaled data was used as beta-diversity measure and visualized with principal coordinate analysis (PCoA). [file DataSheet_1.zip › Supplementary_fig_4_ADDPRO_PCoA_secondary.pdf]

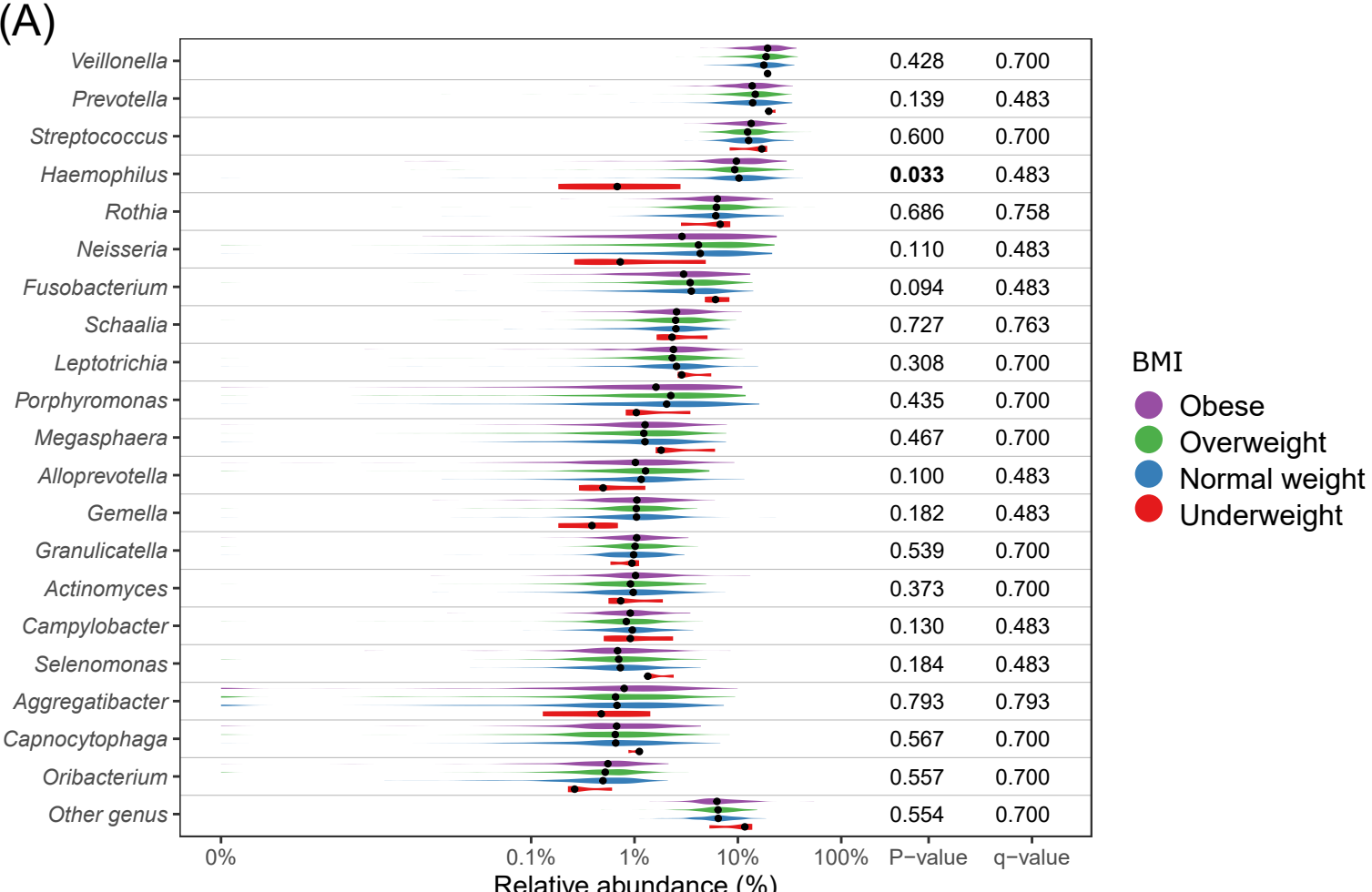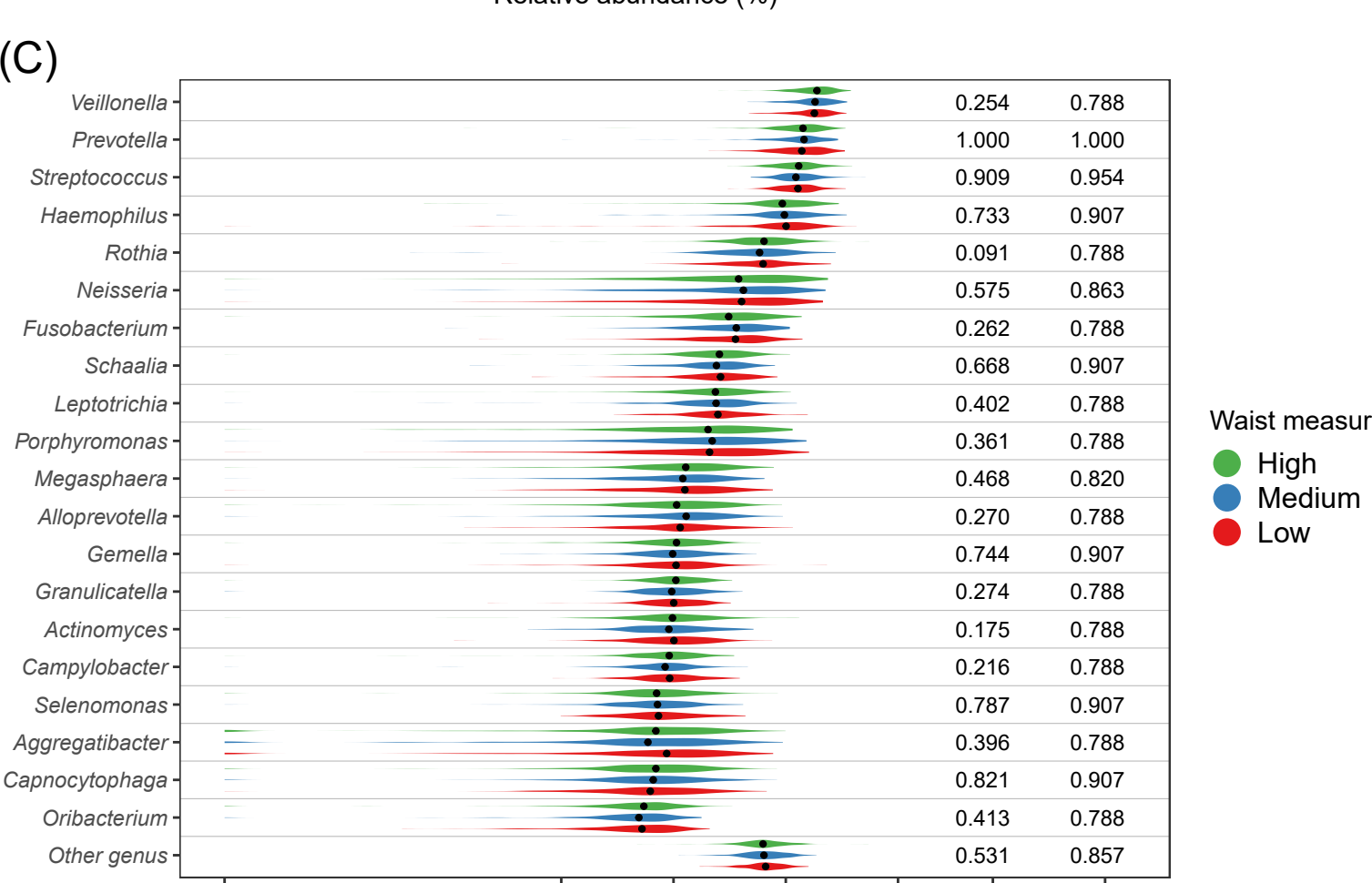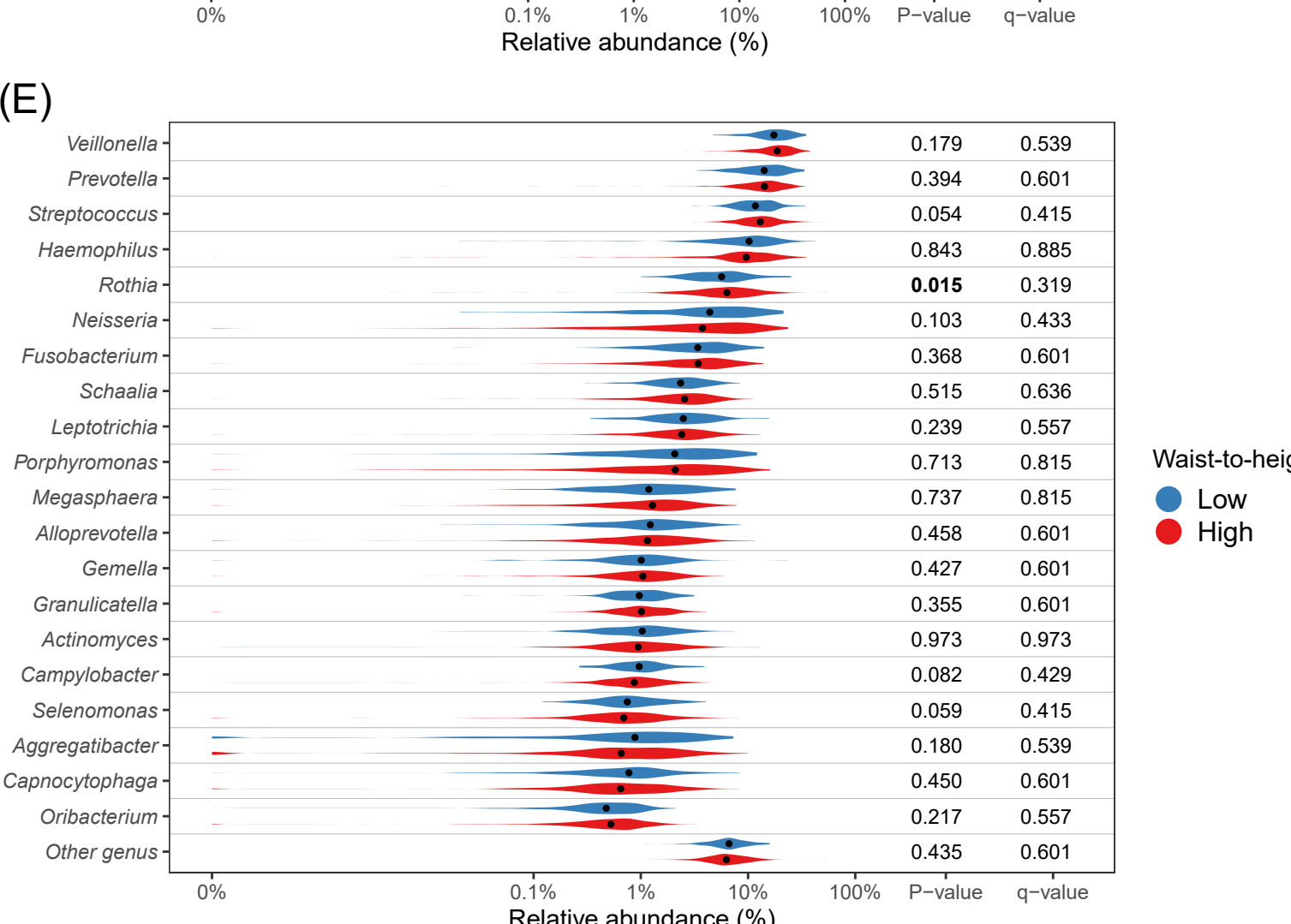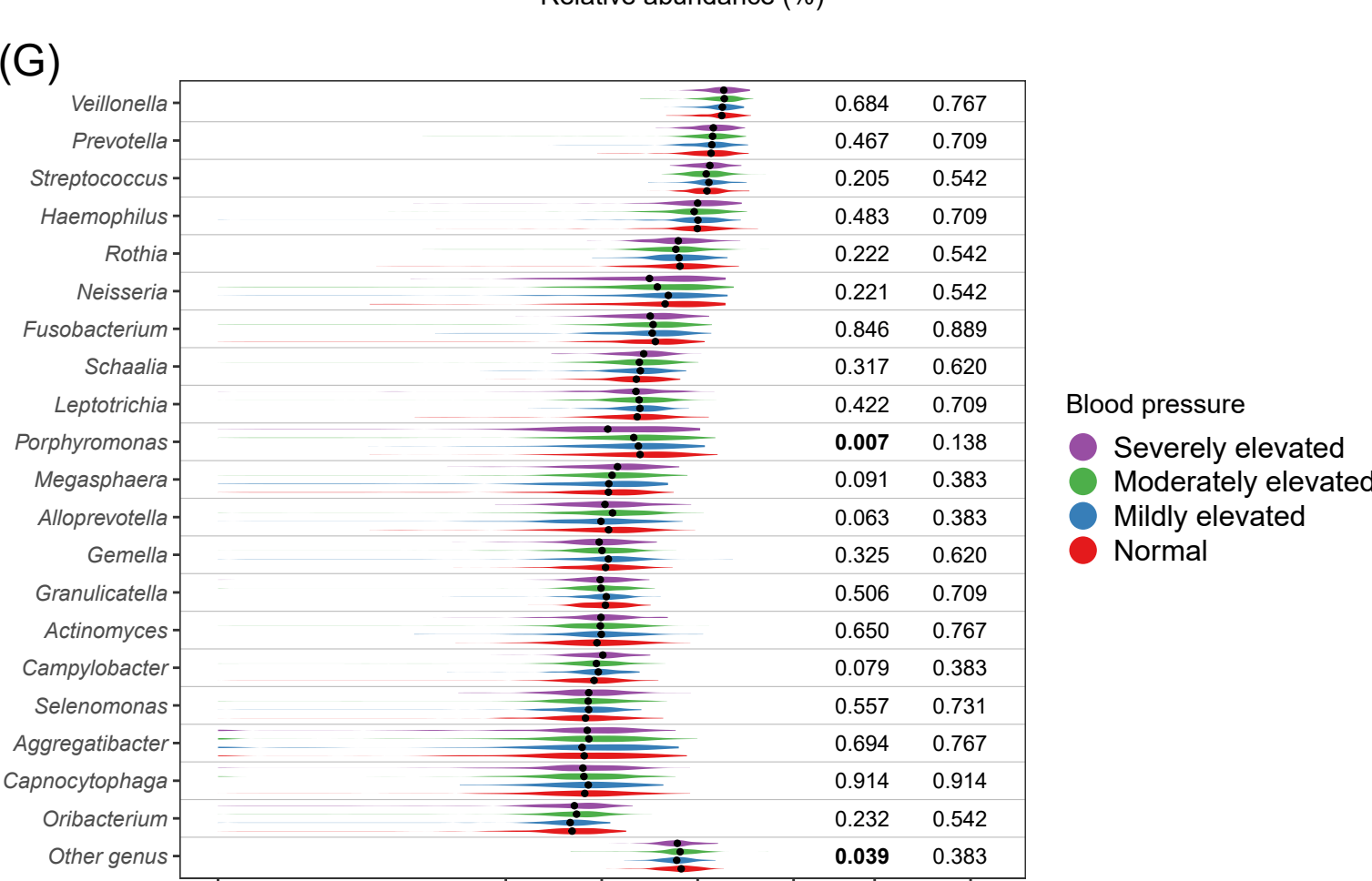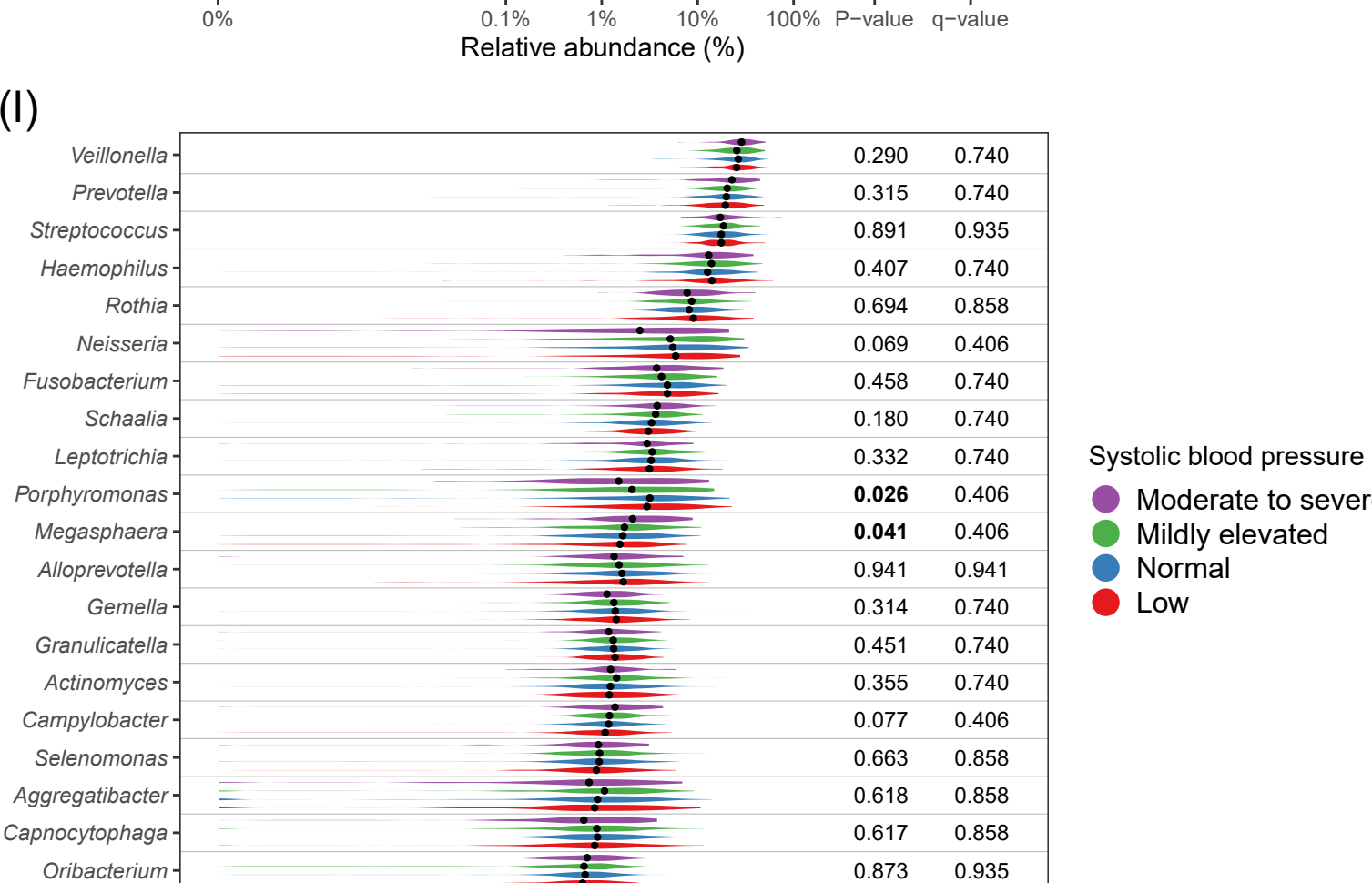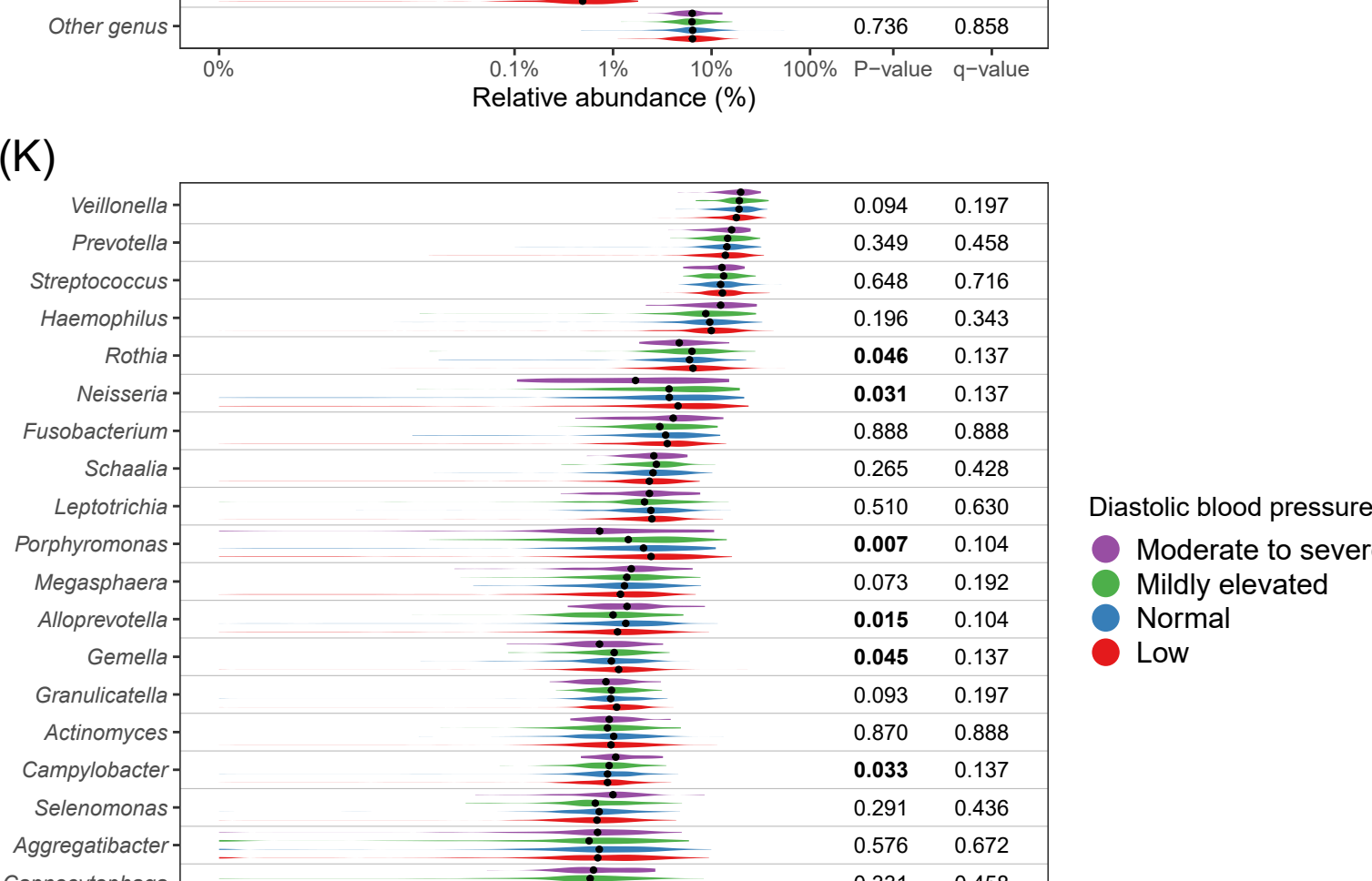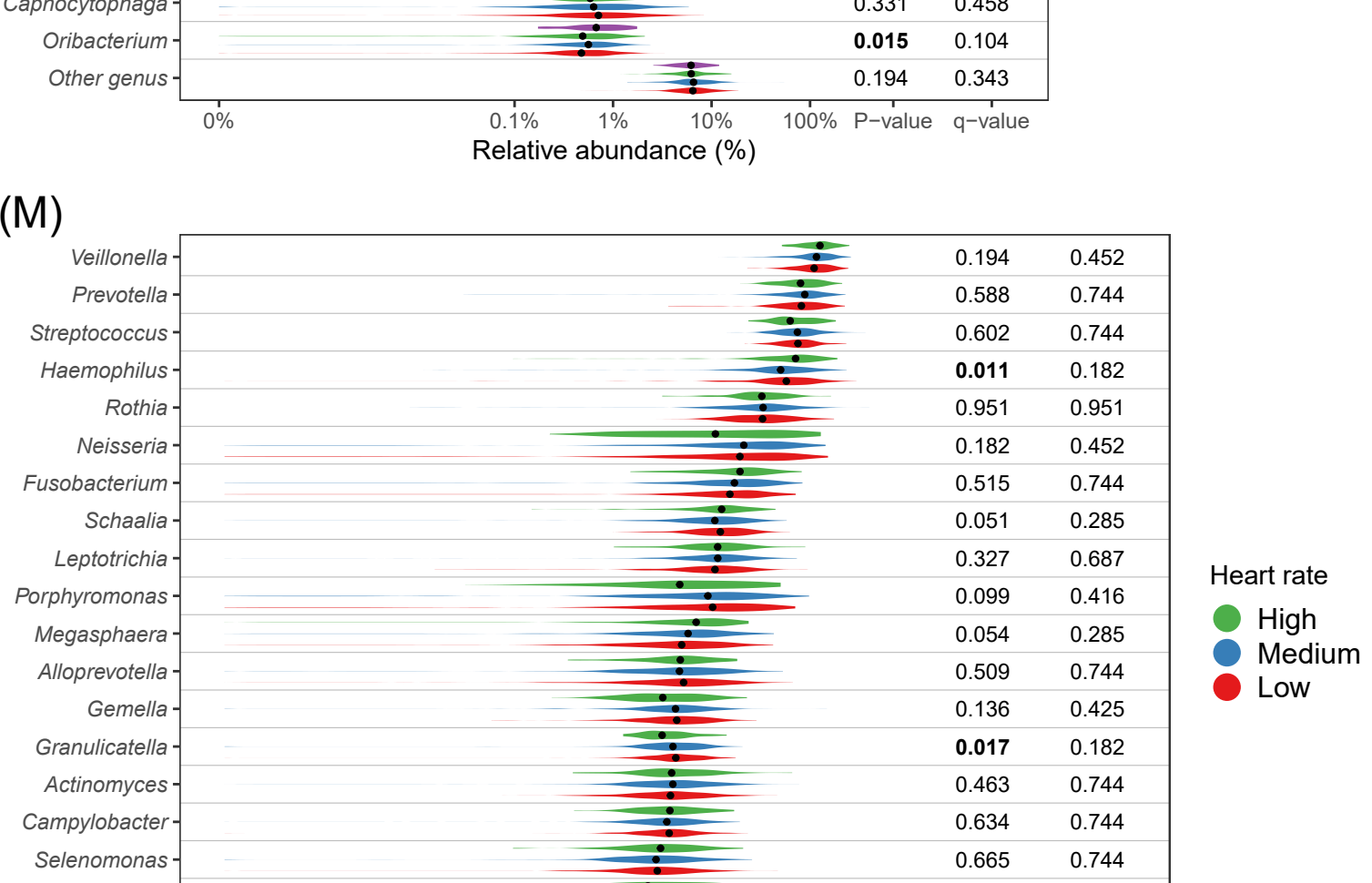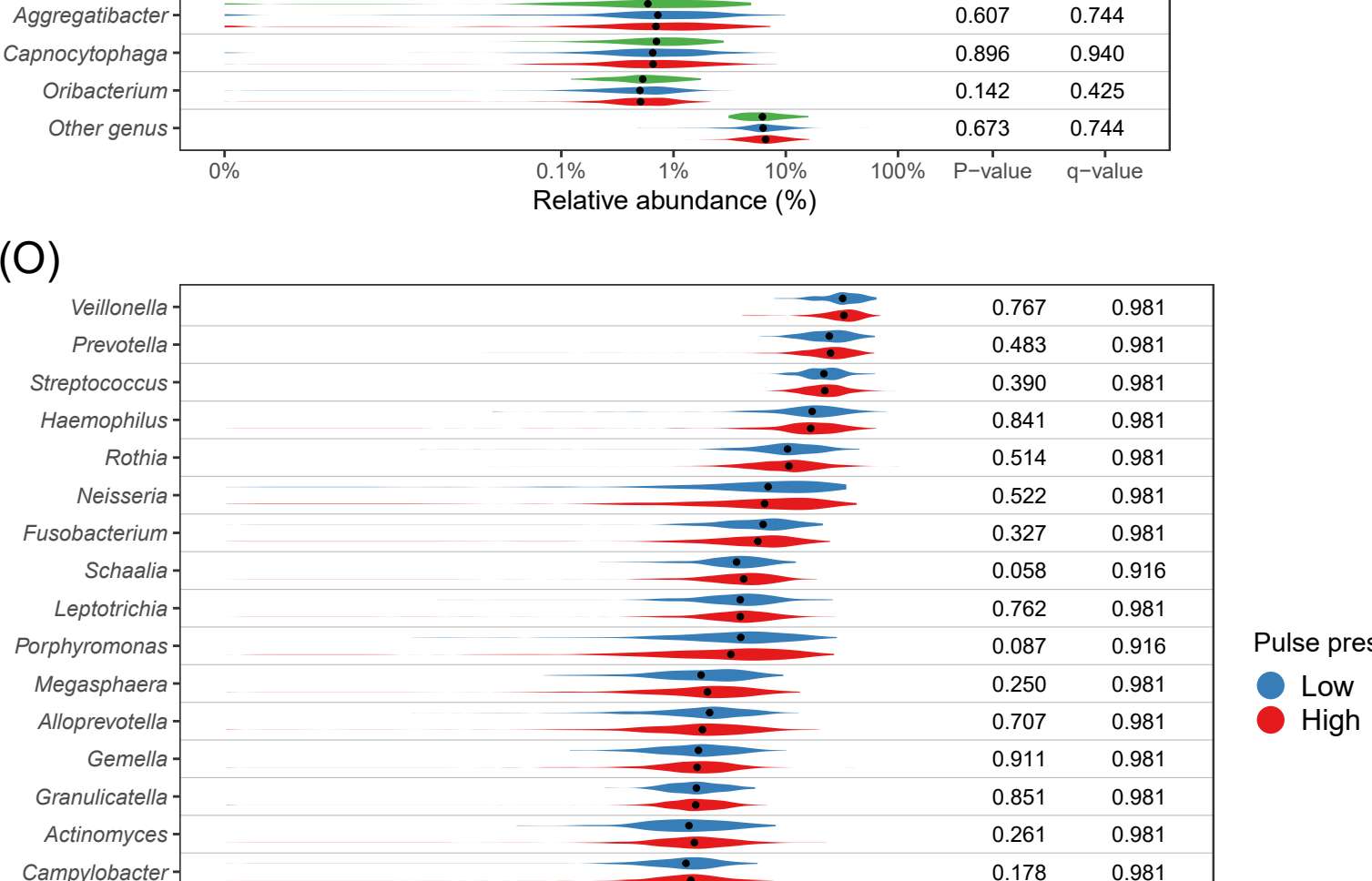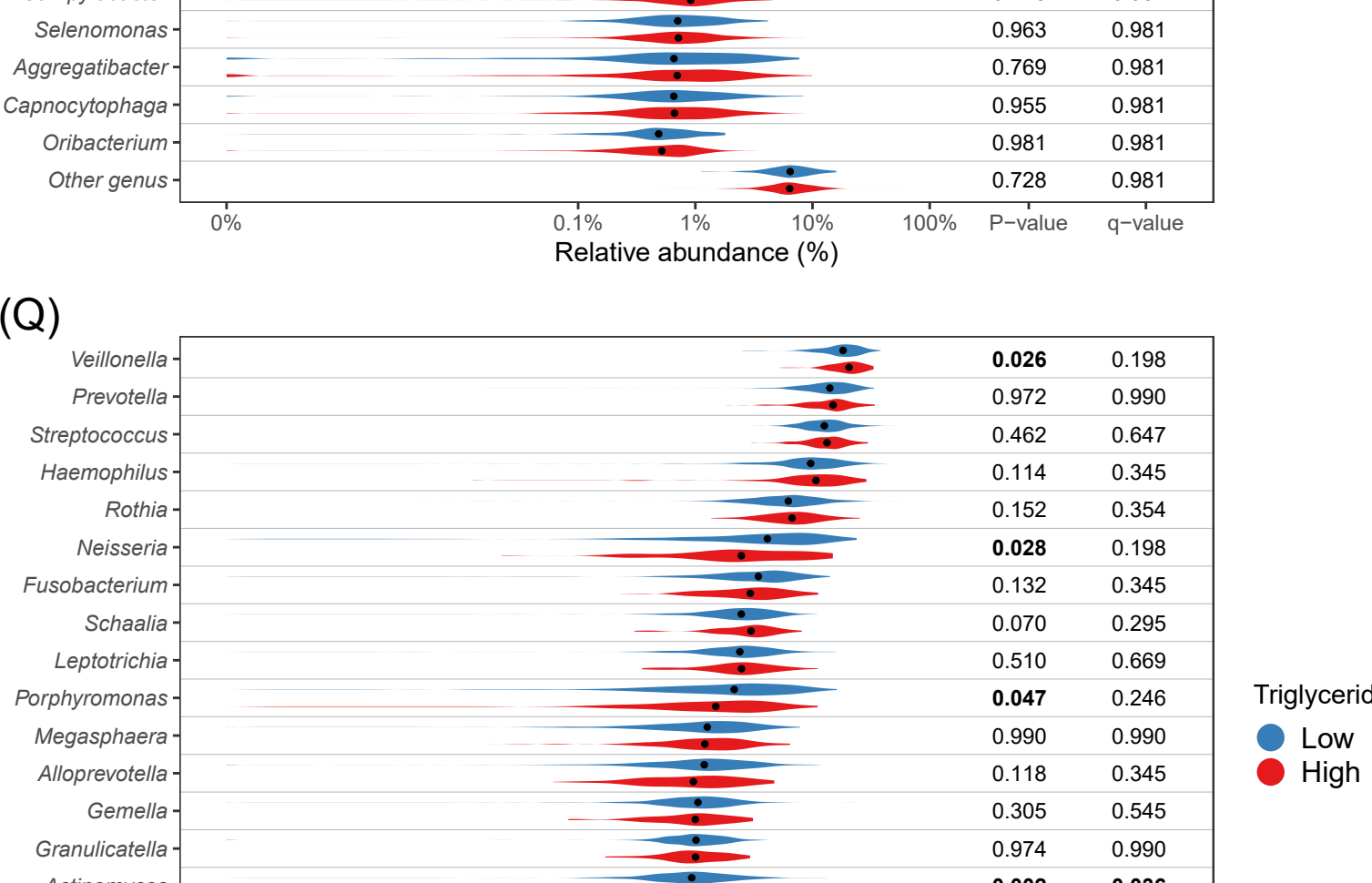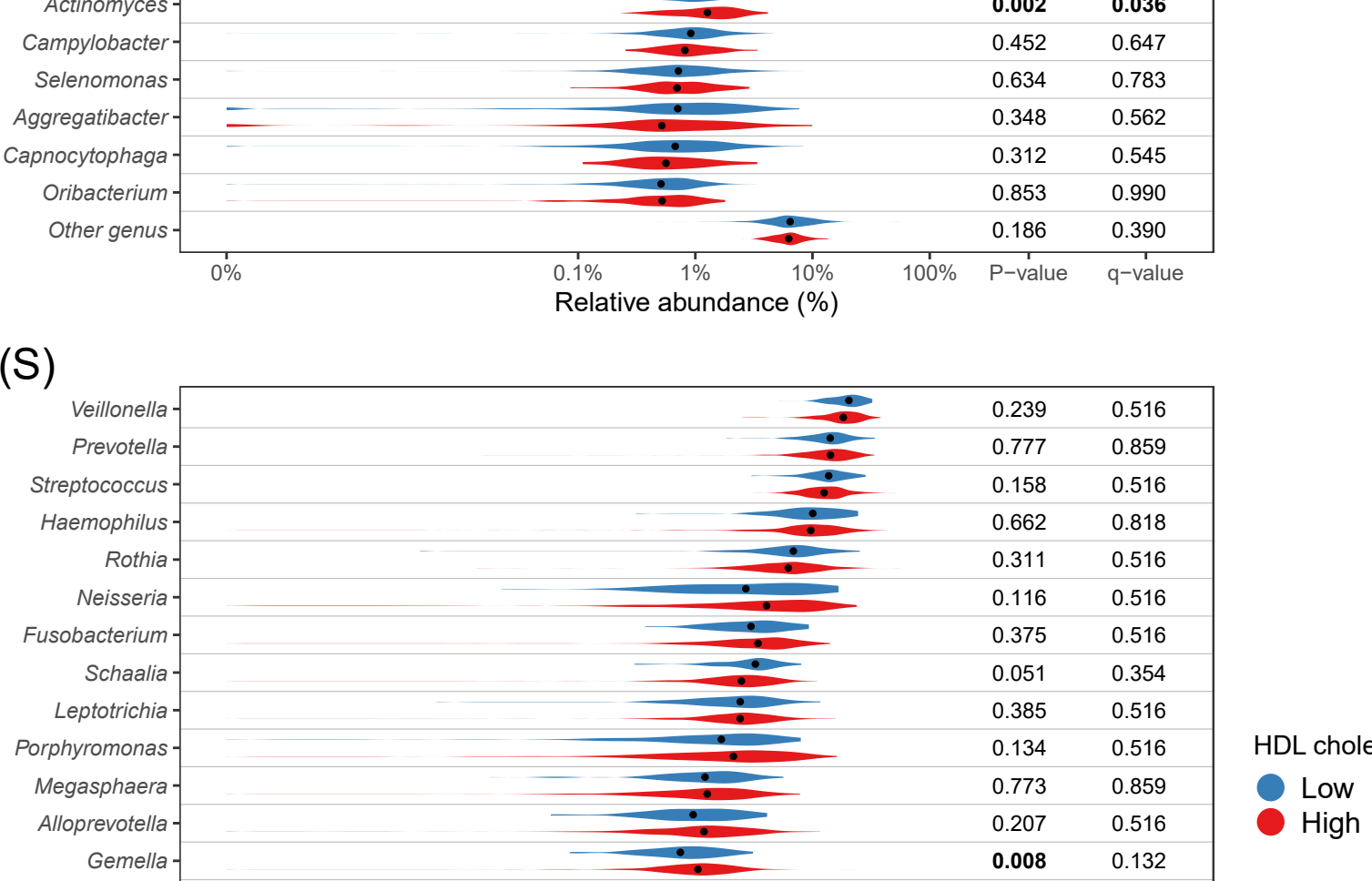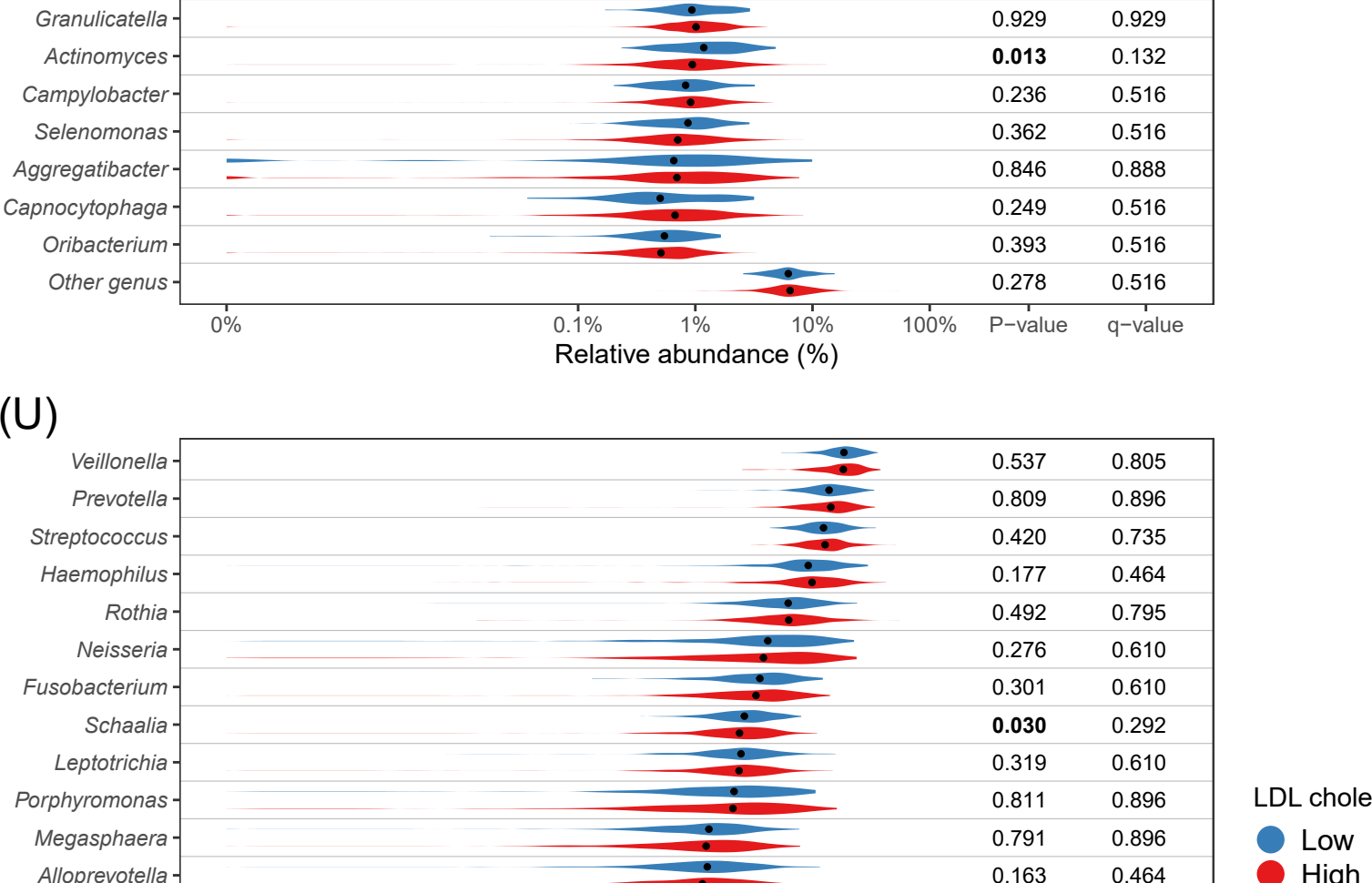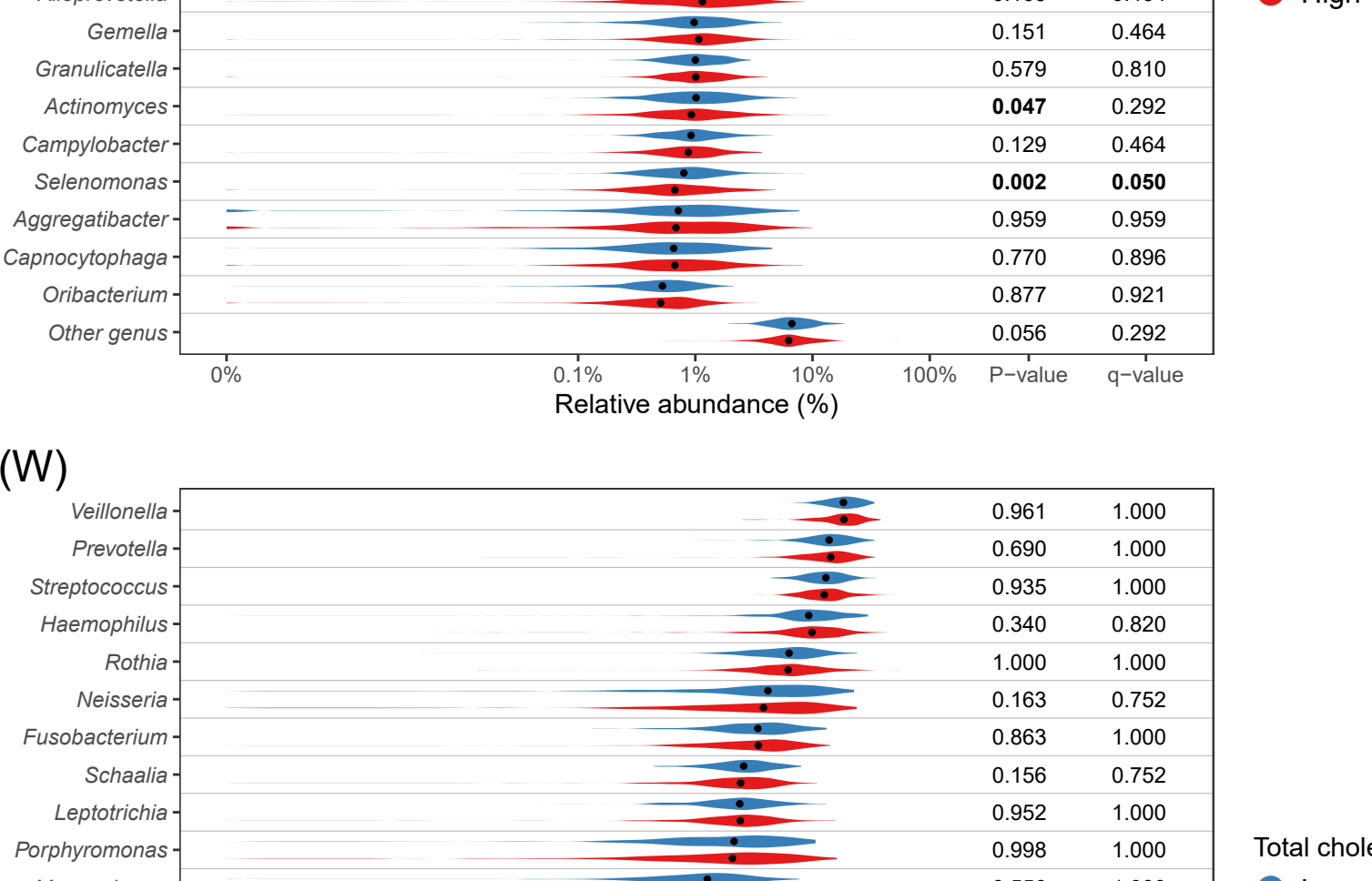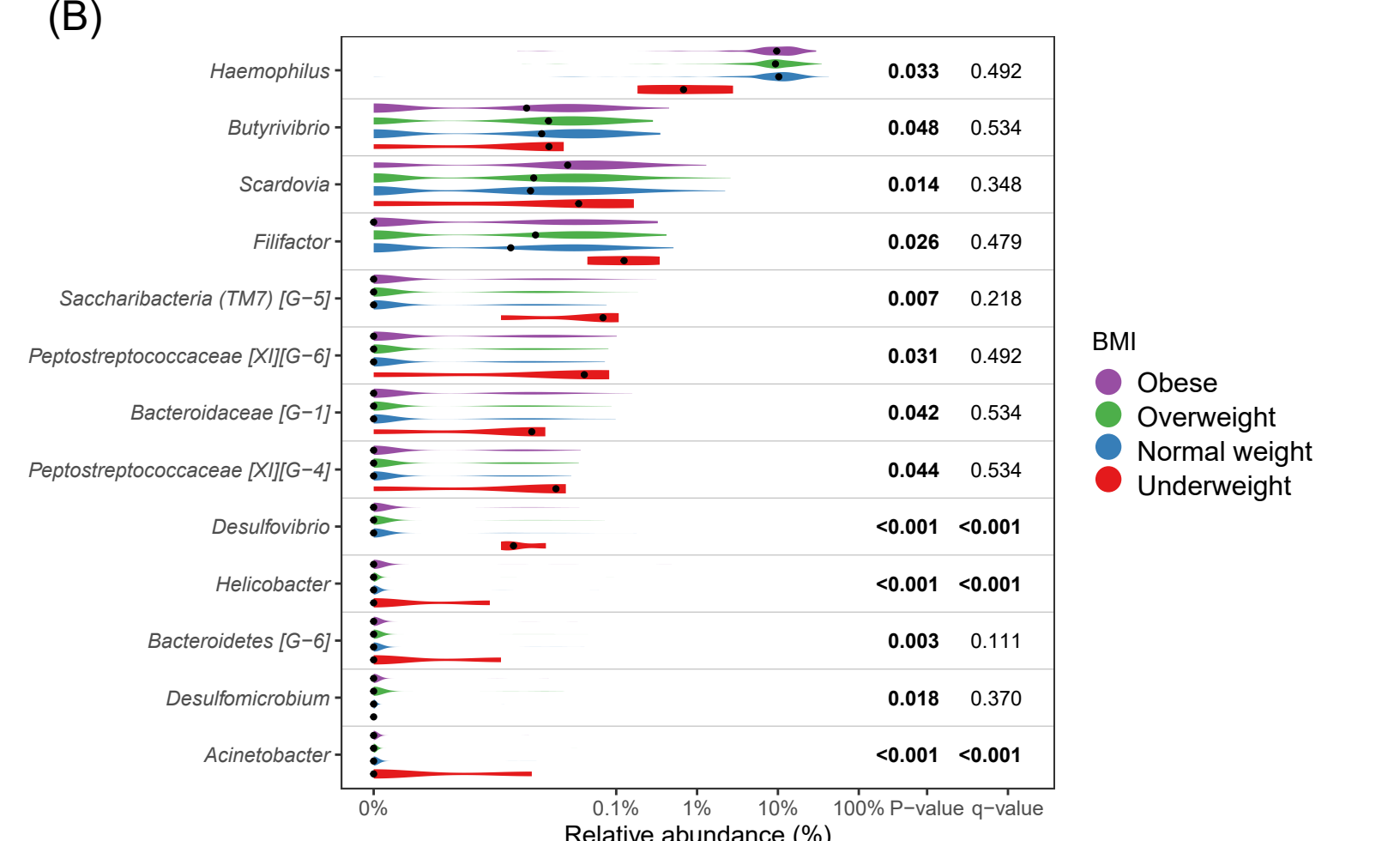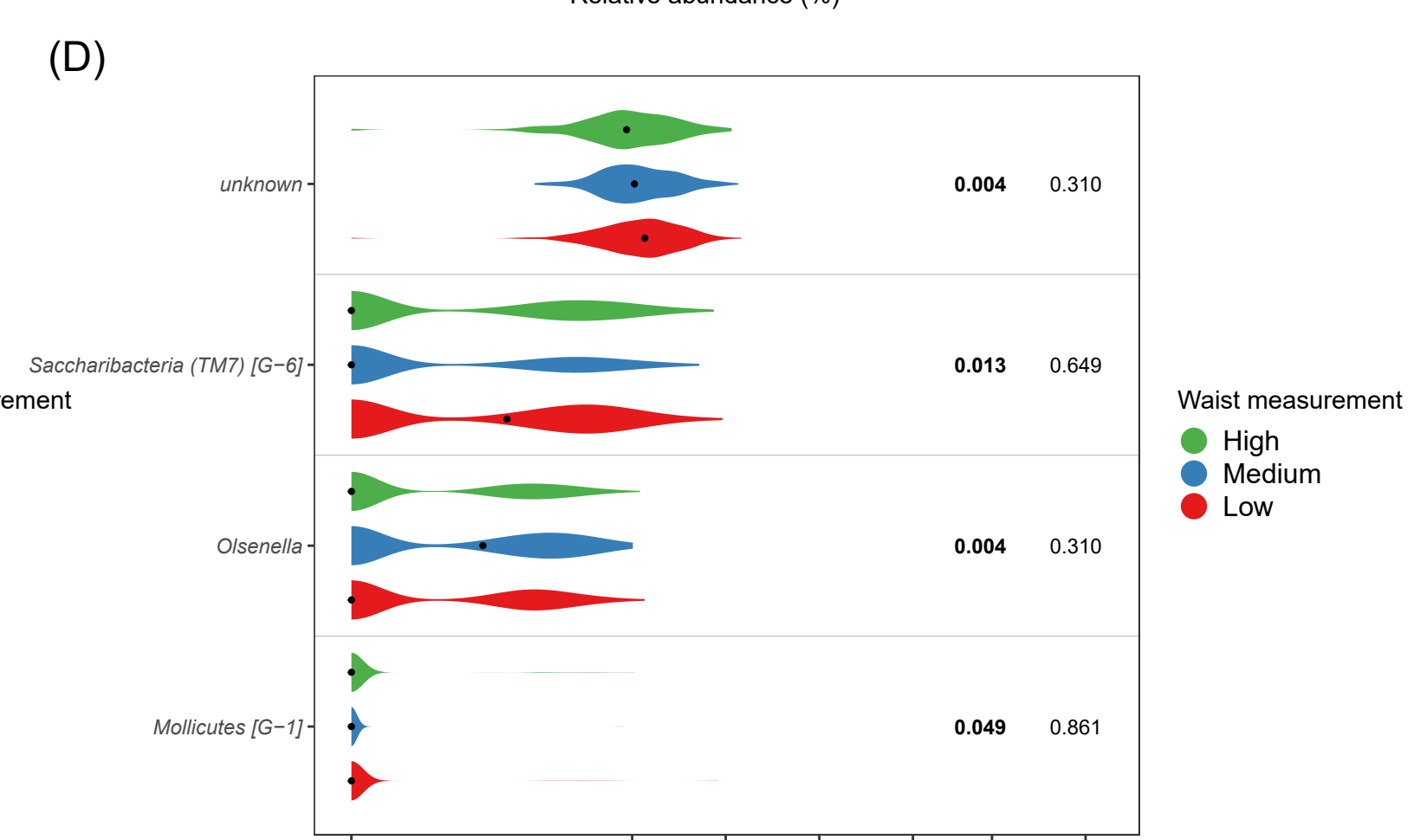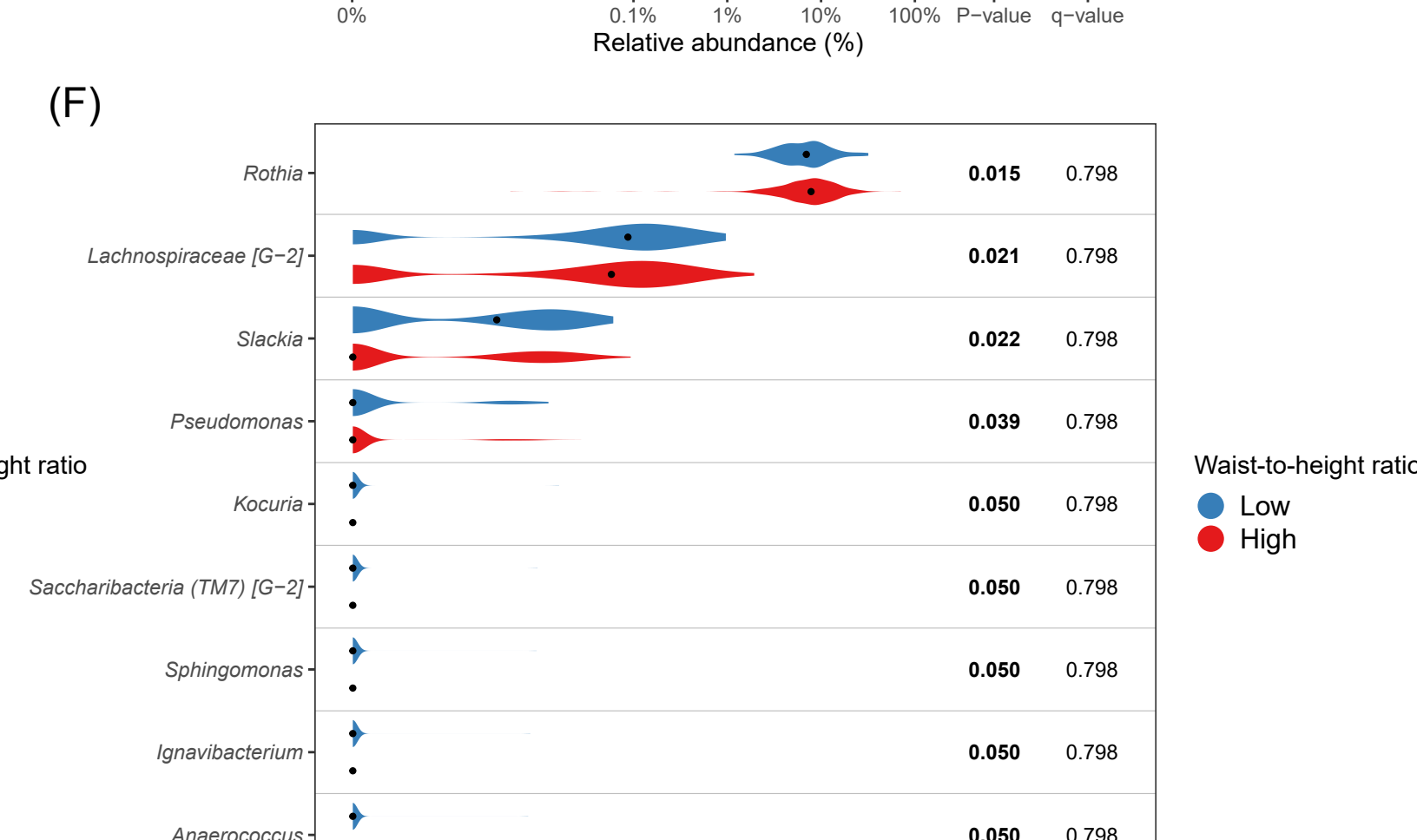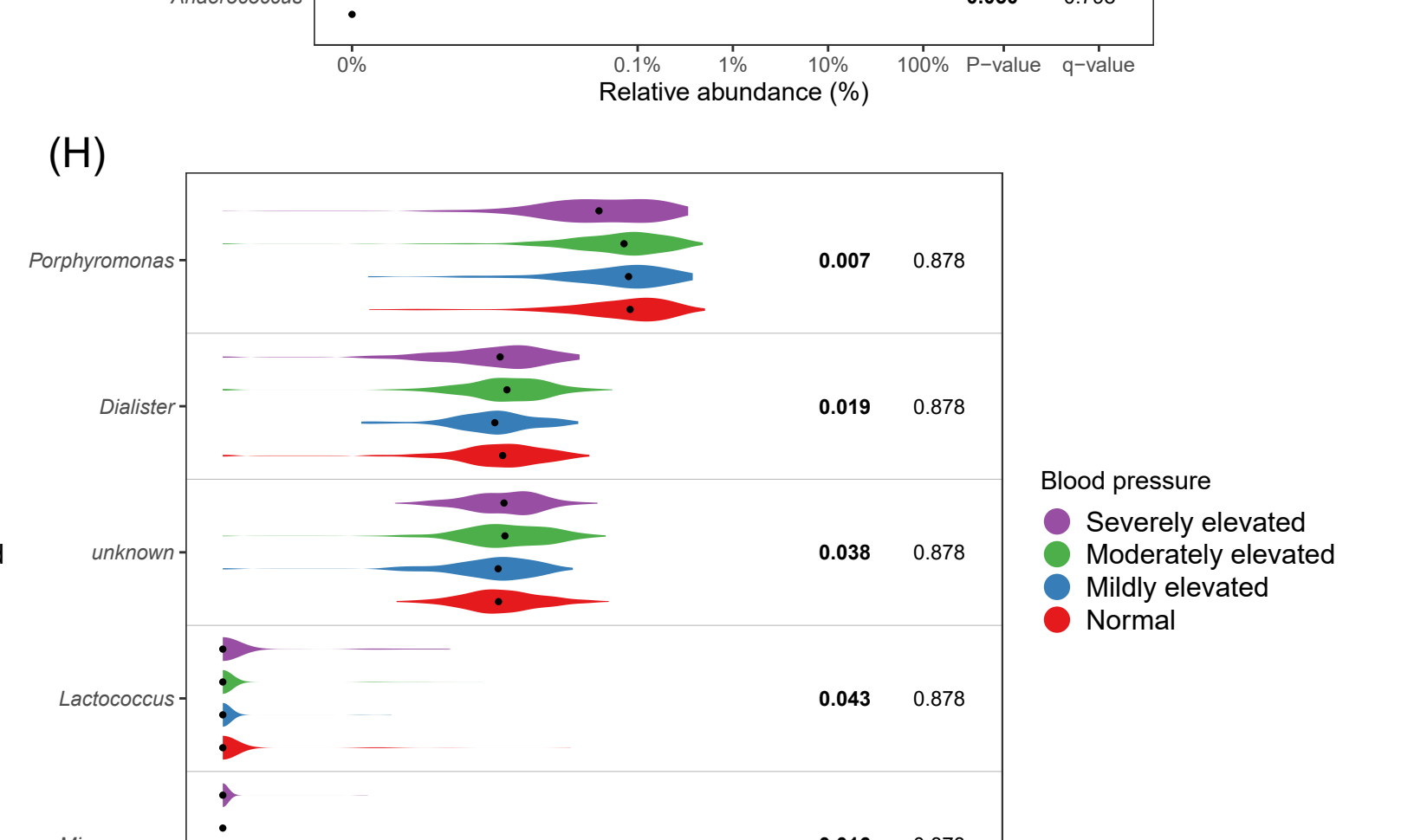

Supplement: Supplementary Figure 1 — Comparison of sequencing batches. Bray-Curtis dissimilarity calculated from Hellinger transformed total sum scaled data was used as beta-diversity measure and visualized with principal coordinate analysis (PCoA). [file DataSheet_1.zip › Supplementary_fig_5_ADDPRO_Rabu_secondary.pdf]
